# The regulatory roles of small nucleolar RNAs within their host locus

**DOI:** 10.1080/15476286.2024.2342685

**Published:** 2024-04-16

**Authors:** Étienne Fafard-Couture, Stéphane Labialle, Michelle S Scott

**Affiliations:** a Département de biochimie et de génomique fonctionnelle, Faculté de médecine et des sciences de la santé, Université de Sherbrooke, Sherbrooke, Québec, Canada; b Université de Lorraine, CNRS, Nancy, France

**Keywords:** snoRNA, *cis-*regulation, sno-lncRNAs, snoRTs, slb-snoRNAs, SPAs, host-derived extensions, snoRNA duplication, snoRNA pseudogenes, genomic imprinting

## Abstract

Small nucleolar RNAs (snoRNAs) are a class of conserved noncoding RNAs forming complexes with proteins to catalyse site-specific modifications on ribosomal RNA. Besides this canonical role, several snoRNAs are now known to regulate diverse levels of gene expression. While these functions are carried out in *trans* by mature snoRNAs, evidence has also been emerging of regulatory roles of snoRNAs in *cis*, either within their genomic locus or as longer transcription intermediates during their maturation. Herein, we review recent findings that snoRNAs can interact in *cis* with their intron to regulate the expression of their host gene. We also explore the ever-growing diversity of longer host-derived snoRNA extensions and their functional impact across the transcriptome. Finally, we discuss the role of snoRNA duplications into forging these new layers of snoRNA-mediated regulation, as well as their involvement in the genomic imprinting of their host locus.

## Introduction

Small nucleolar RNAs (snoRNAs) are a vast group of mid-size noncoding RNAs present in all eukaryotes and most extensively characterized for their role in ribosome biogenesis [1]. They are typically separated in two subclasses, the C/D and H/ACA box snoRNAs, which differ in the motifs they harbour, the secondary structure they adopt and the core binding proteins with which they interact [2]. The naming convention for genes of these two subclasses in human is *SNORD#* and *SNORA#* for C/D and H/ACA box snoRNAs, respectively, where *#* represents a number (sometimes followed by a letter) usually translatable to their order of discovery (e.g. *SNORD13*, *SNORA19*, *SNORD50A*, *SNORD50B*, etc.) [3]. C/D box snoRNAs are defined by the presence of boxes C (RUGAUGA, where R is a purine) and D (CUGA) located, respectively, at their 5’ and 3’ ends, which interact through non-canonical base pairing to form a characteristic kink-turn structure [4]. Although usually more degenerate than the C and D motifs, C/D box snoRNAs often display additional C’ and D’ boxes near the middle of the molecule [4]. In contrast, H/ACA box snoRNAs are characterized by the presence of two hairpins separated by a hinge or H box (ANANNA, where N is any nucleotide) and terminated by an ACA box located 3 nucleotides upstream of their 3’ end [5]. Through interactions with their respective core proteins and enzymes, snoRNAs form snoRNP (snoRNA ribonucleoprotein) complexes which catalyse specific modifications on target RNAs [6]. In particular, C/D box snoRNAs guide 2’-O-ribose methylation catalysed by the methyltransferase fibrillarin (FBL) and H/ACA box snoRNAs guide pseudouridylation catalysed by the pseudouridine synthase dyskerin (DKC1). The specificity by which a snoRNP interacts with a specific target is intrinsically linked to antisense elements (ASEs) located upstream of the D and D’ boxes for C/D box snoRNAs or in internal hairpin bulges for H/ACA box snoRNAs [7,8]. These ASEs are responsible for the base-pairing to a complementary target sequence and typically vary in length between 5 and 20 nucleotides. The best characterized snoRNA targets are ribosomal RNA (rRNA) and small nuclear RNAs (snRNAs), the snoRNA-guided modifications on these targets being crucial regulators of ribosome and spliceosome assembly [9,10]. Notably, most snRNA modifications are guided by a special class of snoRNAs located in the Cajal body (scaRNAs), which can be composed of either or both C/D and H/ACA motifs [9]. However, many snoRNAs still remain to this day with no known associated target, earning them the title of ‘orphan’ snoRNAs.

Combining computational prediction and experimental validation strategies, several reports in the last decades have led to the expansion of the spectrum of snoRNA interactors beyond their canonical targets and snoRNP core proteins (reviewed in [11–13]). Indeed, several transfer RNAs (tRNAs) were reported to interact with C/D box snoRNAs, oftentimes leading to their 2’-O-methylation which regulates their cellular fate [14,15]. In addition, some snoRNAs were found to interact in *trans* with pre-messenger RNAs or messenger RNAs (mRNA), most of these examples underlining the capacity of snoRNAs to regulate the splicing, stability and translation of their target by binding to regulatory elements and in some cases through a snoRNA-guided 2’-O-methylation of the target [16–20]. Interestingly, some C/D box snoRNAs were also observed to guide another type of modification, i.e. the acetylation of rRNA in the budding yeast as well as in animals such as the zebrafish and human [21,22].

**Figure 1.**
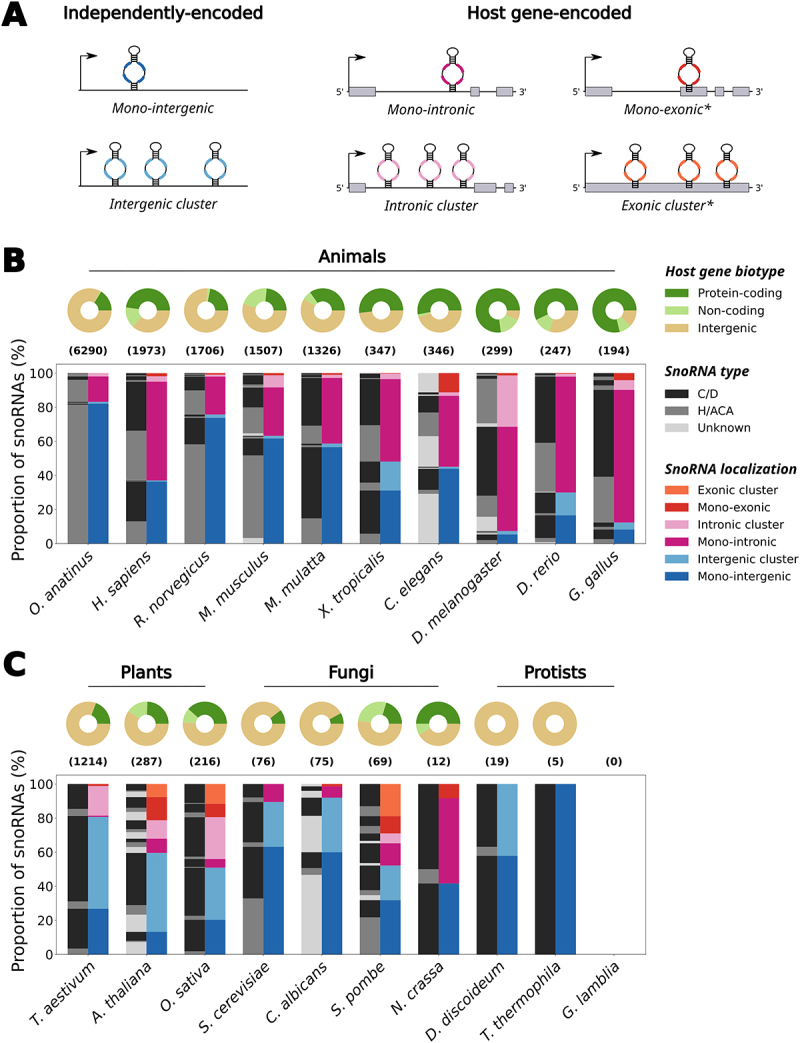
The genomic organization of snoRNAs across eukaryotic model organisms. (a) SnoRNAs can be encoded as independently transcribed units in intergenic regions, either alone as mono-intergenic snoRNAs or as co-transcribed intergenic snoRNA clusters. They can also be embedded within host genes, usually within their introns, relying on their host gene transcription to be expressed. Mono-intronic snoRNAs are encoded alone in their intron whereas some snoRNAs co-exist within the same intron, thereby forming intronic snoRNA clusters. Some snoRNA genes also overlap exon sequences in host genes, either alone as mono-exonic snoRNAs or with others thereby forming exonic snoRNA clusters. The exonic snoRNAs are marked with an asterisk, because they are quite rare and might be artifacts due to genomic annotation errors (e.g. an overlapping exon in the form of a retained intron might not actually exist, therefore making intronic snoRNAs appear as ‘exonic’). (b) The distribution of genomic localization of snoRNAs across animal species is represented as a grouped stacked bar chart. The species are sorted from left to right in decreasing order of the total number of snoRNAs annotated in that species, with the total number being represented in parentheses above the bar charts. The right bar for each species represents the stacked proportion of snoRNAs localized in one of the different organizations displayed in (a). The left bar represents the stacked proportions of snoRNAs of each type (C/D box, H/ACA box or unknown type) that compose each genomic organization represented in the right bar. The distribution of host gene biotype (i.e. protein-coding gene, non-coding gene or intergenic snoRNA) for all snoRNAs in a given species is represented as a donut chart above its respective bars. The represented animal species are the platypus (*Ornithorhynchus anatinus*), human (*Homo sapiens*), rat (*Rattus norvegicus*), mouse (*Mus musculus*), macaque (*Macaca mulatta*), frog (*Xenopus tropicalis*), worm (*Caenorhabditis elegans*), fruit fly (*Drosophila melanogaster*), zebrafish (*Danio rerio*) and chicken (*Gallus gallus*). (c) Same as in (b), except that the chosen species are from the plant, fungal and protist eukaryotic kingdoms. The species are still ordered by decreasing total number of annotated snoRNAs, but the sorting was applied within individual kingdoms. The represented plant species are the wheat (*Triticum aestivum*), thale cress (*Arabidopsis thaliana*) and rice (*Oryza sativa*); the represented fungal species are the budding yeast (*Saccharomyces cerevisiae*), *Candida albicans*, fission yeast (*Schizosaccharomyces pombe*) and *Neurospora crassa*; the represented protist species are *Dictyostelium discoideum*, *Tetrahymena thermophila* and *Giardia lamblia*. Of note, no snoRNA is annotated in *G. lamblia*, resulting in the empty bar and donut charts. The methodology relevant to the results presented in this figure is detailed in the Methods in the Supplementary Material.

Remarkably, the genomic location and expression strategies of snoRNAs vary significantly depending on the species and snoRNA type (Figure 1; Supplementary Table S1; see Methods in Supplementary Material) [23,24]. For instance, in human and in several animals, expressed snoRNAs are mainly embedded within the introns of protein-coding host genes whose functions are related to ribosome assembly, translation regulation and RNA processing, as well as in noncoding host genes (i.e. long noncoding RNAs (lncRNAs)) (Figure 1(b), donut charts) [24]. Following the transcription and splicing of the host gene, the intronic lariat containing the snoRNA is linearized by a debranching enzyme such as DBR1 [25]. The intron remnants that flank the snoRNA are then trimmed by exonucleases up to the mature snoRNA 5’ and 3’ ends, which are protected from degradation by bound core snoRNP proteins [26]. It is also worth mentioning that although most intronic snoRNAs are processed via the previous mechanism, some intronic snoRNAs were shown to be produced through a splicing-independent process in frog oocytes as well as in *in vitro* assays in human cell lines [27,28]. Albeit following predominantly a one snoRNA per intron rule (Figure 1(b), right bar chart for each species), host genes in animal genomes commonly harbour multiple snoRNAs distributed throughout different introns [23,24]. Interestingly, higher eukaryotes such as mammals show generally a greater proportion of intergenic snoRNAs than lower eukaryotic animals, which favour mostly intronic snoRNAs (Figure 1(b), right bar chart for each species; compare the five species furthest to the left with the five furthest to the right). In contrast to this genomic architecture, most fungi snoRNAs exist as mono-intergenic snoRNAs, i.e. as independent transcriptional units with their own promoter [29,30], whereas plant snoRNAs are usually organized in intergenic clusters for which all snoRNAs are transcribed at once from one independent promoter (compare the right bar charts in Figure 1(b) to those in the middle and left panels in Figure 1(c)) [31,32]. Notably, snoRNA annotations in protist species as well as in some fungi are still clearly lacking comprehensiveness compared to other eukaryotic kingdoms as shown by the low number or even the absence of annotated snoRNA genes in these species (Figure 1(c), right panel). In addition to species-specific organization, snoRNA location also varies according to the snoRNA type. For instance, in human, most H/ACA box snoRNAs are encoded alone in their host gene, whereas host genes of C/D box snoRNAs usually harbour overall more than one snoRNA across their introns [24]. Interestingly, a greater proportion of H/ACA box snoRNAs is observed in mammals compared to other types of animals and species from other eukaryotic kingdoms, which usually harbour more C/D box snoRNAs (Figure 1(b–c), compare the left bar chart for each species). These observations support the hypothesis that H/ACA and C/D box snoRNA genes propagate in genomes using different strategies (which are differentially used depending on the species), with the former favouring retrotransposition and the latter favouring *cis*-recombination [33–36].

Intriguingly, even though many snoRNAs are conserved across eukaryotes, the total number of snoRNA genes greatly differs across the Eukarya domain (Figure 1(b–c), compare the numbers above the bar charts). Simple unicellular organisms such as yeasts and protists usually harbour less than a hundred snoRNAs in their genome, which highly contrasts with multicellular organisms [37]. Indeed, plant and animal genomes typically display several hundred snoRNA genes, with mammalian genomes sometimes containing several thousand snoRNA genes [38,39]. These snoRNAs are often grouped into families based on sequence alignment and sequence covariance [36,40], which underlines that snoRNAs exist in multiple copies (sometimes with the exact same sequence) within a given genome. While having multiple exact copies creates a redundancy of snoRNAs targeting the same sites, most snoRNA family members, at least in human, do not display perfect sequence identity [36]. This raises the question as to the biological significance and even more so the functionality of these diverged copies.

To complicate matters, recent studies indicate that several annotated snoRNA genes are not expressed (or at a very low level) in a mature form, ranging from a quarter to more than two-third of all annotated snoRNAs depending on the species [24,41–43]. These non-expressed snoRNAs, also referred to as snoRNA pseudogenes, are often defined by the accumulation of mutations in their characteristic motifs which could impact their capacity to bind their protein interactors, form a stable snoRNP and/or interact with their given target [42]. Interestingly, snoRNA pseudogenes have been identified in many multicellular organisms including mammals, amphibians, plants and nematodes [33,38,44,45], which contrasts with observations in unicellular organisms like fungi that tend to turnover entire snoRNA families instead of maintaining these snoRNA remnants [46].

Based on these observations, one would expect snoRNA pseudogenes to be poorly conserved across species due to their lack of expression. Although this is the case for most snoRNA pseudogenes, almost 12% of these non-expressed snoRNAs in human show an intriguingly high level of sequence conservation throughout vertebrates (average phastCons score ≥ 0.5) [36], hinting to potential new regulatory functions of these snoRNAs outside of their typical *trans*-acting properties and that could be carried out from within their host locus. Supporting reports in recent years have shown that some snoRNAs can act as transient *cis*-regulators of their own host gene [47–49]. Others have observed that some snoRNAs can also exist in various kinds of host-derived longer hybrids, i.e. transient or sometimes highly stable composite RNAs for which the cellular function is starting to emerge. This review explores the different regulatory roles uncovered for snoRNAs, while they are not expressed as independent mature snoRNPs, from their host transcript maturation regulation to the myriad of transient or stable longer forms they can adopt from their host locus. We finish by highlighting the role of snoRNA copies in shaping these new layers of gene regulation across species’ genomes, as well as their implication in the induction of genomic imprinting of their host locus.

## Transcriptomic regulators in *cis* of their host gene

It is well understood that the expression of intron-encoded snoRNAs depends on the transcription and splicing of their host gene [23,26]. Yet, a growing body of literature demonstrates that, surprisingly enough, the abundance of most intronic snoRNAs does not correlate (and is sometimes anticorrelated) with that of their host gene [24,41,50,51]. Several hypotheses have been put forward to explain this uncoupling of expression, including the implication of alternative splicing, selective nonsense-mediated decay (NMD) of specific host transcripts and the use of alternative promoters, as well as the differing stability between the snoRNA and host transcript following their common transcription [24,52,53]. However, recent evidence has also started to demonstrate the active role snoRNAs can play in influencing the fate of their host gene.

**Figure 2.**
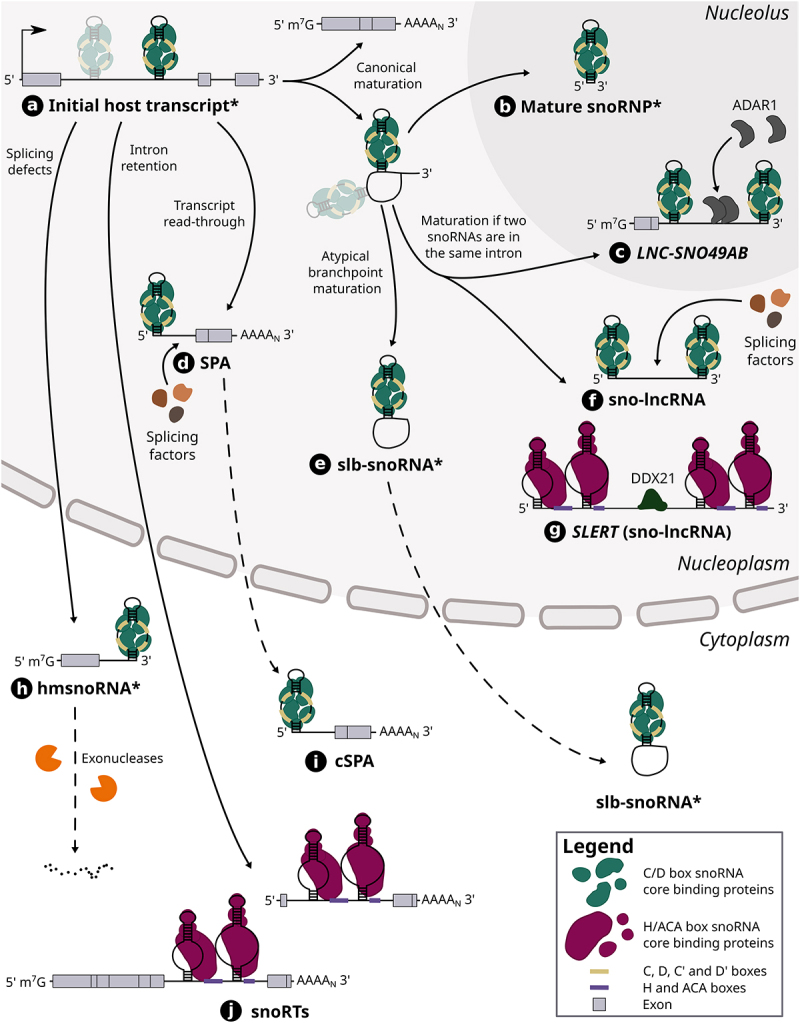
The maturation steps of different host-derived snoRNA hybrids. Host gene transcripts can harbour one or multiple (shaded) snoRNAs in their intron (see (a)). In the case of a snoRNA encoded alone in its intron, its canonical maturation requires the transcription and splicing of its host gene, which results in a snoRNA-containing lariat as well a mature host transcript. The lariat is typically debranched and its ends are then degraded by exonucleases up to the snoRNA ends which are bound by core proteins, thereby protecting the mature snoRNP from further cleavage (see (b)). When two snoRNAs are encoded in the same intron, the same maturation steps can lead to the formation of snoRNA-ended hybrids called sno-lncRNAs including *SLERT* (see (f) and (g)), a H/ACA sno-lncRNA, as well as the lncRNA *LNC-SNO49AB* (see (c)). *SLERT* was shown to interact with DDX21 and regulate cell proliferation, whereas *LNC-SNO49AB* was observed to bind with ADAR1 and promote its dimerization and activity. Other sno-lncRNAs have also been found to interact with splicing factors. On the other hand, atypical branch points (cytidines instead of adenosines) can hinder lariat debranching, thus leading to the formation of stable lariats bearing a snoRNA (slb-snoRNAs) which can be actively transported to the cytoplasm (see (e)). In addition, transcript readthrough of host genes can lead to the production of nuclear 5’ snoRNA-ended and 3’ polyadenylated lncRNAs (SPAs) that were shown to interact with splicing factors (see (d)). One example of SPA was also shown to be exported to the cytoplasm (*SNORD86* cSPA) (see (i)). The retention of introns harbouring a H/ACA box snoRNA leads to the generation of snoRNA retaining transcripts (snoRTs), which are exported to cytoplasm and vary in length with regards to their 5’ end (see (j)). Finally, knocking down splicing factors was shown to induce splicing defects including the formation of hybrid mRNA-snoRNA (hmsnoRNAs) transcripts, which were observed to be either degraded or stabilized in the cytoplasm (see (h)). Of note, dotted lines represent the possible path that a host-derived snoRNA extension can take, whereas full lines represent an obligatory step in its maturation pathway. The asterisk that marks the name of certain snoRNA-containing hybrids (i.e. initial host transcript, mature snoRNP, slb-snoRNA and hmsnoRNA) signifies that the snoRNA represented in the hybrid can either be a C/D box or H/ACA box snoRNA, although only C/D box snoRNAs are represented in those cases for clarity.

An emblematic example of this new *cis*-regulatory function is *SNORD86*, an orphan C/D box snoRNA which is embedded in the *NOP56* gene, i.e. a host gene which codes for the C/D snoRNP core protein of the same name. This snoRNA was shown to modulate the expression level of its host gene in response to the concentration of the NOP56 protein [47]. Indeed, within the intron in its host transcript, *SNORD86* was found to adopt two alternate conformations: 1) when NOP56 protein level is low, the snoRNA assumes a non-snoRNP structure which results in the complete splicing of its intron and thereby the formation of a functional transcript that can then be translated into the NOP56 protein to restore its adequate cellular levels; 2) when the NOP56 protein is abundant, it binds to the snoRNA in its intron, favouring a snoRNP conformation which, in turn, promotes the splicing and production of an alternative transcript that is exported to the cytoplasm and cleaved by the NMD machinery. This longer noncoding isoform, called cytosolic 5’-snoRNA-ended and 3’-polyadenylated lncRNA (SPA), contains the snoRNA that is bound by its core proteins and accumulates in the cytoplasm, possibly to sequester away an excess of core proteins from the nucleus (see (i) in Figure 2, and Supplementary Table S2). Intriguingly, the sequence of this cytosolic SPA is conserved across eutherians, suggesting that it might play a similar role in these species. In addition, this lncRNA constitutes the vast majority of the total *NOP56* transcripts (and thereby of the *SNORD86*-containing transcripts), which correlates with the observation that the shorter and mature form of *SNORD86* is only detected at very low levels in most human tissues and cell lines [39]. Overall, these results underline the fact that *SNORD86* does not play a major role as a mature snoRNP acting on targets in *trans* but acts rather in *cis* as a sensor of the output of its host gene, while still embedded in its host transcript, with important consequences for the regulation of snoRNP and ribosome assembly.

Interestingly, the advent of high-throughput RNA–RNA interaction datasets in recent years has led to the expansion of the snoRNA interactome, including the discovery of novel *cis*-interactions between snoRNAs and their host gene [19,48,49,54]. Similar to the *SNORD86* case, two studies have recently shown that another C/D box snoRNA, *SNORD2*, also regulates the splicing of its host gene, *EIF4A2* [48,49]. In most healthy human tissues, this snoRNA was shown to form a conventional snoRNP structure which is excised normally from its host intron [39,49]. Yet, *SNORD2* was also observed, in specific tissues and cell lines, to interact in *cis* with its own host intron, more specifically with the intronic region downstream of the snoRNA. This snoRNA-intron interaction gives rise to a structure that sequesters the branch point of the intron and favours the exclusion of the following exon of the host transcript [49]. This alternative splicing generates a premature stop codon, targeting this newly spliced isoform to the NMD machinery and its rapid degradation. Through the formation of an alternative *SNORD2*-intron structure, this splicing regulation mechanism was hypothesized to be influenced by the elongation rate of the RNA polymerase II, which varies across cell types and conditions. Furthermore, the sequence implicated in this *SNORD2*-intron interaction was shown to be highly conserved across vertebrates, suggesting that this regulation could occur in other species. Notably, the same study reported more than a hundred distinct snoRNA-host transcript interactions, hinting to a potential widespread *cis*-regulatory role played by snoRNAs which is yet to be explored.

Additional approaches such as classical structure–function studies present great potential to uncover *cis*-regulatory roles of snoRNA genes. Recently, CRISPR/Cas9 knockout of C/D box snoRNAs located in the introns of the lncRNA *GAS5* suggested that *SNORD74* harbours a regulatory region that could modulate the splicing and maturation of its host transcript, possibly through m6A modification of the snoRNA in its host transcript [55]. Thus, while intronic snoRNAs were long viewed as passive passengers of their host gene, at least three and perhaps many other snoRNAs share a complex bidirectional regulatory relationship with their host gene.

## Transcriptomic regulators as host-derived extensions

Although the previous examples have shown that snoRNAs can regulate their host gene fate by interacting directly in *cis* within the whole host transcript, several studies have also brought to light a myriad of diverse snoRNA hybrids that originate from their host locus, but that come in all shapes and sizes [56–62] (Figure 2). Found in a wide variety of eukaryotes, these composite RNAs sometimes exist only transiently but oftentimes persist in cells as stable products with putative functions. One such example are the snoRNA retaining transcripts (snoRTs) which were recently identified in human breast cancer cell lines upon screening for DKC1-bound transcripts [56] (see (j) in Figure 2, and Supplementary Table S2). These snoRTs are defined as host gene transcripts with a retained intron containing a H/ACA box snoRNA, with their length varying from the whole initial host transcript to truncated versions at their 5’ end (i.e. starting with a few exonic nucleotides upstream of the retained intron containing the snoRNA). Intriguingly, these snoRTs accumulate in the cytoplasm and are bound by the H/ACA core proteins GAR1 and NHP2 as well as DKC1, which is reminiscent of the *SNORD86* SPA mode of action in which snoRNP core protein levels are regulated through their sequestration in the cytoplasm [47]. Among the most significantly enriched transcripts upon DKC1 immunoprecipitation, more than 40 different snoRTs appear in the top candidates [56]. Prediction of the potential targets of these snoRTs combined with a *de novo* analysis of pseudouridine-seq datasets showed a significant overlap, suggesting that the role of at least some of these snoRTs would be to guide the pseudouridylation of other mRNAs.

While snoRTs are generally constituted of a large portion of the host transcript, smaller, yet highly stable snoRNA-containing RNAs were recently observed in various vertebrates including frog, chicken, mouse and human [57]. These stable lariats bearing a snoRNA (slb-snoRNA) are the natural product of the splicing of host genes (see (e) in Figure 2, and Supplementary Table S2). However, they are not linearized as efficiently by the debranching enzyme due to their atypical branchpoint (a cytidine instead of an adenosine), which confers these lariats a high stability in the cell [63]. Most of these lariats are usually species-specific, with some exceptions of conserved slb-snoRNAs between closely related frog species. While they are mainly found in the nucleus, slb-snoRNAs were also shown to be actively exported to the cytoplasm, thereby competing with the canonical snoRNA maturation process occurring in the nucleus (i.e. lariat debranching followed by exonucleolytic trimming up to the snoRNA ends that are protected by proteins). In addition to this regulatory role, it was also demonstrated that some cytoplasmic slb-snoRNAs can be bound by snoRNP proteins such as DKC1. Since yeast slb-snoRNAs (induced through the depletion of Dbr1) maintain their ability to guide modifications on their target RNA [25], it was proposed that this could also be the case with these vertebrate slb-snoRNAs. Yet, none of the tested slb-snoRNAs have shown the capacity to guide modifications on rRNA, hinting that their potential function could be instead to sequester RNA binding proteins (RBPs) in the cytoplasm, a common pattern seen with snoRNA-containing extensions and lariats exported to the cytoplasm [47,56,57,64].

Interestingly, not all snoRNA hybrids are stable: some also exist only transiently in cells and are rather often the product of snoRNA maturation defects. For instance, it was shown that when splicing factors are depleted in yeast cells, hybrid mRNA-snoRNA (hmsnoRNA) transcripts are generated from host genes of both C/D and H/ACA box snoRNAs [58] (see (h) in Figure 2, and Supplementary Table S2). These hmsnoRNAs consist of the host gene transcript up to the snoRNA 3’ end which is protected from the nuclear RNA exosome by core proteins bound to the snoRNA. After their export to the cytoplasm, most hmsnoRNAs are degraded through 5’-3’ decay pathways; yet some still accumulate to levels varying between 10% to almost 70% of their mature snoRNA counterparts [58]. Based on these observations, an interesting hypothesis was put forward that the splicing defects observed in many human diseases could not only impact protein-coding genes but could also generate similar stable hmsnoRNAs for which the pathogenic potential remains to be determined [58,65].

As emphasized previously, splicing is a key pillar to the effective biogenesis of intronic snoRNAs. Yet, when two snoRNAs are encoded within the same intron, the same splicing process can lead to the production of longer composite RNAs called sno-lncRNAs [59] (see (f) in Figure 2, and Supplementary Table S2). Indeed, after the intronic lariat is spliced out of the host gene and linearized, the 5’ and 3’ ends of the intron are trimmed by exonucleases. When two snoRNAs are embedded in that intron, the core proteins bound to the two snoRNAs act as shields against exonucleolytic cleavage, thereby creating a sno-lncRNA, i.e. an intron remnant flanked by two mature snoRNAs which are most often C/D box snoRNAs. First discovered originating from the 15q11-q13 locus in human [59], an important region hosting the *SNORD115* and *SNORD116* C/D box snoRNA families and that is deleted in the Prader-Willi Syndrome (PWS) [66], sno-lncRNAs have also been reported in other mammals including some arising from different genomic loci [67]. Interestingly, the known sno-lncRNAs are usually species-specific, except for the PWS sno-lncRNAs which were found to be conserved across several primates [67]. Unexpectedly, sno-lncRNAs from the PWS region were observed to localize to the nucleoplasm, but not in the nucleolus, suggesting an alternative function associated with these RNAs. Indeed, following immunoprecipitation and immunofluorescence assays, these sno-lncRNAs were found to bind to the splicing factor RBFOX2 and alter its availability in specific nuclear foci, thereby modulating splicing events in several neuronal-specific genes. This role of protein trapping is not unlike that of other lncRNAs which are known for their capacity to act as decoys for transcription factors and RBPs [68,69]. Interestingly, sno-lncRNAs can also be produced from host gene transcripts via the skipping of an exon that is flanked by two snoRNA-harbouring introns. This is the case of *SLERT*, a sno-lncRNA that enhances pre-rRNA transcription which was discovered and shown to be specific to human [60] (see (g) in Figure 2, and Supplementary Table S2). In contrast to most sno-lncRNAs, *SLERT* is constituted of H/ACA box snoRNAs at its ends (*SNORA5C* and *SNORA5A*), interacts with DKC1 and localizes to the nucleolus. Following its knockout, a significant reduction in pre-rRNA was observed, indicating its potential function in rRNA biogenesis. *SLERT* was also identified as an interactor of DDX21, an RNA helicase involved in ribosome synthesis, through its non-snoRNA internal sequence, whereas the flanking snoRNAs were found to be crucial for its nucleolar localization. Its interaction with DDX21 was shown to promote the transcription of ribosomal DNA (rDNA) genes, leading in turn to increased cell proliferation.

A recently discovered variation to sno-lncRNAs is the lncRNA *LNC-SNO49AB* which was characterized in blood samples of leukaemia patients [61] (see (c) in Figure 2, and Supplementary Table S2). This hybrid RNA resembles the structure of sno-lncRNAs, although its 5’ end is protected by an additional m^7^G cap. *LNC-SNO49AB* originates from the *SNHG29* host gene which harbours two C/D box snoRNAs in its second intron: *SNORD49B* followed by *SNORD49A*. The resulting 5’ capped lncRNA consists of the first and second exon of *SNHG29* as well as the second intron which is truncated immediately after *SNORD49A*. This lncRNA was observed to localize to the nucleolus and interact with the core C/D box snoRNP proteins; yet its knockdown did not show any effect on rRNA methylation level, suggesting it plays a different role. RNA pull-down assays coupled to mass spectrometry highlighted that the A-to-I editing enzyme ADAR1 could interact with several sites on *LNC-SNO49AB*. Further experiments demonstrated that *LNC-SNO49AB* acts as a scaffold to promote the dimerization and thereby the activity of ADAR1, which was found to be indicative of cancer progression [61,70]. Interestingly, the sequence of *LNC-SNO49AB* was shown to be conserved across primates, implying that it could exist and play a similar role in those species [61].

Finally, at the interface between sno-lncRNAs and mRNAs lie the SPAs, which, as a reminder, are polyadenylated lncRNAs that are capped by a snoRNA at their 5’ end. As opposed to the previously introduced cytosolic *SNORD86* SPA, the two other known SPAs to this date, *SPA1* and *SPA2*, are retained in the nucleus and are, respectively, capped by the C/D box snoRNAs *SNORD107* and *SNORD109A* [47,62] (see (d) in Figure 2, and Supplementary Table S2). Importantly, they are encoded in the PWS region downstream of the *SNURF-SNRPN* gene and of its weak poly(A) signal [62]. Following the transcription of *SNURF-SNRPN* and the cleavage of the resulting pre-mRNA at its poly(A) site, the fate of the downstream cleavage product remains under the control of RNA polymerase II (which continues to elongate it) and the exonuclease XRN2 (which trims it) according to the torpedo model [71]. Whereas in typical pre-mRNA maturation XRN2 would catch up to the polymerase and trigger transcription termination, it was shown that the presence of *SNORD107* and the core proteins bound to it in the downstream cleavage product block the exonuclease from further trimming. Therefore, this stabilizes the 5’ end of the nascent transcript which is elongated until the next poly(A) signal where it is cleaved and polyadenylated, resulting in the *SPA1* transcript. Moreover, RNA polymerase II continues to transcribe the region downstream of *SPA1*. This region harbours *SNORD109A* and a further downstream poly(A) signal, resulting in the formation of *SPA2* through the same mechanism described above. Notably, by substituting *SNORD107* with the H/ACA box snoRNA *SNORA5C*, *SPA1* was still produced to similar levels as its endogenous form, implying that both snoRNA types have the capacity to generate SPAs. To assess the functional role of SPAs, RNA fluorescence in situ hybridization (FISH) assays were undertaken and showed that both *SPA1* and *SPA2* co-localize with the PWS sno-lncRNAs in specific nuclear subdomains. Following individual-nucleotide resolution UV crosslinking and immunoprecipitation (iCLIP) experiments, all the PWS SPAs and sno-lncRNAs were shown to interact with various splicing factors including TDP43, RBFOX2 and hnRNP M. Through the sequestration of up to 1% of the total cellular level of these proteins, SPAs were found to regulate several splicing events in genes involved in synaptogenesis, hinting at a relationship between the aetiology of PWS and the dysregulation of splicing factor localization. Furthermore, a conserved *SPA1* (but not *SPA2*) was also identified in mouse (*mSPA1*) hippocampal tissue, suggesting that SPAs’ regulatory function could take place across various species.

## SnoRNA copies as a potential reservoir of new snoRNA forms and functions

The previous examples of transcriptomic regulation carried out by host-derived snoRNA extensions highlight the wide range of shapes and functions that snoRNAs can undertake in the cell. Indeed, most of the snoRNAs involved in longer hybrids also coexist with their mature and shorter snoRNP counterpart. However, the ratio of mature snoRNA to the extended snoRNA forms varies considerably depending on the snoRNA, ranging from a dominance of the mature snoRNA species to the opposite state [47,57,59–61]. As snoRNAs are crucial regulators of rRNA maturation and ribosome assembly [10], one can wonder how the cell can afford such fluctuations in mature snoRNA levels which depends oftentimes on the degree of production of its cognate longer form.

One plausible explanation resides in the fact that many snoRNAs exist in multiple copies in higher eukaryotes [23,36]. Due to uncountable rounds of recombination and retrotransposition events, the genome of current multicellular species harbours multiple snoRNA families that expanded in size and diversity throughout evolution [33–36,38]. Although members of the same snoRNA family can greatly vary in terms of abundance, it was recently shown that the overall total family abundance stays relatively constant across different cell types [36]. This observation supports the viability of having longer snoRNA extensions that compete with the formation of their corresponding mature snoRNA, since other ‘backup’ copies can be produced as mature snoRNPs to ensure the desired target modification level. This also coincides with the fact that all naturally occurring snoRNA extensions presented herein have only been identified in multicellular organisms, which are known to harbour multiple snoRNA copies in their genome [23], and not in simpler unicellular organisms.

Therefore, snoRNA gene duplication represents an interesting evolutionary force which can have an impact both at the family level, but also at the host gene level. Through these duplication events, snoRNAs can propagate in various genomic locations and sometimes to astonishing numbers (e.g. a H/ACA box snoRNA was copied almost 40,000 times in the platypus genome) [38,72]. It is thus highly probable that snoRNA duplication events in introns enabled the formation of many of the known snoRNA extensions including sno-lncRNAs, snoRTs and slb-snoRNAs. As reported previously, the presence of snoRNA in introns can dramatically alter the splicing patterns of the host gene [47–49]. Therefore, it is tempting to speculate that the insertion of snoRNAs in host genes could also impact several other steps of the host gene processing including its polyadenylation and translation by disrupting for instance poly(A) and RBP binding sites.

Since snoRNAs can be retrotransposed in antisense into host genes and intergenic regions [38,72], one could also hypothesize that the ASEs of these snoRNAs act as a binding site for their parental copy, thereby opening the possibility of a wide range of snoRNA-mediated regulation of the retrotransposon recipient gene including at the chromatin, transcriptional and post-transcriptional levels. While this type of regulation might seem unlikely at first glance, at least three lines of evidence support it. Firstly, various snoRNA interactors were reported by several groups [14,19,73], indicating that snoRNAs, at least in their canonical mature form, actively interact with a wide range of RNAs (e.g. with other snoRNAs). Secondly, several snoRNAs and sno-lncRNAs were observed to be associated with chromatin and oftentimes regulate its state in *Drosophila melanogaster*, mouse and human [74–78], indicating that some snoRNAs have at least the capacity to localize at different DNA regions. Lastly, a similar phenomenon is observed with microRNAs (miRNAs) in which miRNA-containing transposable elements can be inserted in antisense of the 3’ untranslated regions (UTR) of genes, thereby creating compatible target sites for the parental miRNA copy [79–81]. Thus, this suggests that, in principle, such regulation could also be functionally relevant for retrotransposed snoRNAs.

Ultimately, having multiple copies of the same snoRNA creates some flexibility for the cell in the same way protein-coding gene duplication allows for new functions to emerge [82]. For instance, different mutations can accumulate in the ASEs of snoRNAs without affecting their parental copy, leading to the loss of complementarity to the initial target and/or the acquisition of complementarity to new targets. Although this process usually takes place across a long evolutionary time, it was recently demonstrated to occur even between closely related amphibian species [44]. Furthermore, depending on the snoRNA, not all parts of their sequence are subject to the same selective pressure, as it was shown in the *SNORD116* snoRNA family that their two ASEs showed significant differences in conservation level while their C and D boxes remained largely unchanged between homologous snoRNAs [83]. Altogether, snoRNA gene duplications are likely to lead, on the one hand, to the degeneration of snoRNAs into pseudogenes if too many deleterious mutations accumulate, but on the other hand, to the creation of new regulatory roles including in *cis* and as extensions of their host gene.

## The involvement of large tandem repeats of snoRNAs in genomic imprinting

While snoRNA genes generally exist as part of families that are dispersed throughout the genome, some C/D box snoRNA families have been shown to occur locally in large tandem repeats. These unusual genomic organizations can contain hundreds of repeated snoRNA genes, consistent with the propensity of C/D box snoRNA genes to duplicate locally [33,34,36]. These families correspond to recent eutherian-specific innovations that produce orphan C/D box snoRNAs suspected to regulate unconventional targets [16,83,84]. These snoRNA genes are embedded within repeated introns of poorly characterized lncRNA genes and are found at two loci controlled by parental genomic imprinting (PGI): the *Dlk1-Dio3* (at human 14q32) and the PWS domains. PGI is an epigenetic mechanism that differentially marks the two parental genomes and causes the specific expression or repression of so-called imprinted genes from a given parent [85,86]. Each of the previously mentioned domains hosts two tandem repeats formed, respectively, by the *SNORD113*/*SNORD114* and *SNORD115*/*SNORD116* families. While these tandem repeats likely arose in a common ancestor of eutherian species, a fifth tandem repeat-containing lncRNA called *Bsr* is only present in the rat genome [87], suggesting that the acquisition of these genomic structures may be ongoing and that understudied genomes might harbour novel snoRNA tandem repeats.

Interestingly, the organization of C/D box snoRNA genes in tandem repeats presumably had a major influence on their evolution and function. Indeed, it likely favoured a complex genetic interplay between neighbouring gene copies, giving rise to a high rate of gene gains and losses likely due to non-allelic recombination events, e.g. resulting in four to ten times more *SNORD115* and *SNORD116* gene copies in the mouse than in the rat genome despite limited evolutionary distance [83,88]. In addition, it probably facilitated a high rate of non-allelic conversion events between highly similar gene copies that promoted gene homogenization and spreading of sequence polymorphisms. It is interesting to note, however, that both the *SNORD115* and *SNORD116* tandem repeats were able to form gene subfamilies in primates. *De novo* mutations favoured by selection can escape erasure and spread within copies if gene diversity is beneficial [89]. Therefore, the maintenance of snoRNA subfamilies argues in favour of the neofunctionalization of certain gene copies in primates [83,88]. Intriguingly, phylogenetic analyses have revealed a complex association between C/D box snoRNA tandem repeats and PGI, leading to two functional hypotheses. First, cross-species comparisons support the idea that it was the formation of snoRNA repeats that led to the installation of PGI at and around their site of amplification [90]. PGI is thought to derive from mechanisms dedicated to silencing transposable elements [91,92], so a consistent scenario would be that snoRNA tandem repeats are recognized as parasitic structures to be repressed. This possibility is also supported by the fact that a similar association applies to the few known large tandem repeats of microRNA genes [90,93–95]. Second, recent works have revealed that the adjacent *SNORD115* and *SNORD116* repeats evolved in a coordinated manner with respect to copy gains and losses, the emergence of gene subfamilies, and partial tandem duplication events [88,96]. It was also suggested that this coordination was orchestrated by PGI since differential chromatin compaction can favour concerted non-allelic homologous recombination events of closely spaced tandem repeats [88]. In short, it is thus possible that snoRNA tandem repeats favoured and then were affected by the installation of PGI, highlighting the complex role of snoRNA genes in regulating their host locus expression.

## Conclusion

Once perceived as mere guides of rRNA and snRNA modification, recent studies on snoRNAs have unequivocally painted a much more complex picture. From new protein interactors to novel types of targets, the modes of action of snoRNAs have kept expanding in recent years. In this review, we have focused on the extensive regulatory roles that snoRNAs exert not as mature snoRNPs, but rather from within their host locus. More specifically, we have explored the modulatory roles of snoRNAs with regard to the maturation of their host transcript in *cis*, as well as the profusion of different host-derived forms they can adopt. In addition, we have discussed their varied cellular roles and potential evolutionary origin through gene duplication, as well as the complex interplay between snoRNA repeats and their host locus imprinting. Yet, many open questions remain to be answered. Comprehensive studies are needed to better understand how both snoRNA and snoRNA extensions are co-regulated, and to what extent these occur in eukaryotes other than in the few animals in which they were first identified (e.g. in plant species). Since SPAs can theoretically be capped by H/ACA box snoRNAs [62], we also expect that future research will likely uncover such snoRNA hybrids through the use of snoRNA-adapted high-throughput detection methods (e.g. TGIRT-Seq [97], icSHAPE-MaP [98], RNA structure analysis using nanopore sequencing [99], etc.). With the myriad of forms they can adopt, it is highly plausible that even more eclectic host-derived snoRNA extensions will be characterized in the future, especially by expanding research to a wider range of species and conditions. Finally, while some functions have been determined for a few of the *cis*-interacting snoRNAs and of the host-derived hybrids, further studies implicating experimental validations will be needed to decipher the precise molecular role of these snoRNAs.

## Supplementary Material

Supplementary_tables_S1_S2_Methods.pdf

## Data Availability

The authors confirm that the data supporting the findings of this study are available within the article and its supplementary materials.
