# Supplementary material for: The regulatory roles of small nucleolar RNAs within their host locus
Source: RNA Biol. 2024 Apr 16;21(1):1–11. doi: 10.1080/15476286.2024.2342685 (PMC11028025; doi:10.1080/15476286.2024.2342685)
Supplement: Supplementary_tables_S1_S2_Methods.pdf [file KRNB_A_2342685_SM0684.pdf]

**Supplementary materials accompanying the review**

# The regulatory roles of small nucleolar RNAs within their host locus

**by**

Étienne Fafard-Couture, Stéphane Labialle & Michelle S Scott

# Methods

Retrieval of the genomic location of snoRNAs

The annotation (GTF file) and genome of all species were downloaded from Ensembl (v110 for animals and v58 for plants, protists and fungi species) [1]. The Homo sapiens annotation file was supplemented with all snoRNAs included in the specialized human snoRNA database snoDB v2.0 [2] with a box type being either “C/D”, “H/ACA”, “AluACA” or “unknown”. The resulting annotation file is accessible through Zenodo with the following link: https://zenodo.org/records/10728180/files/Homo_sapiens.GRCh38.110_snoRNAs_tRNAs.gtf.gz. The coordinates of snoRNA genes were retrieved from the annotation files by keeping entries with the gene biotype “snoRNA” (or “ncRNA” for *Dictyostelium discoideum*) and were filtered to remove duplicate entries. Potential host genes of snoRNAs were identified as any gene overlapping a snoRNA gene on the same strand, with the restriction that their biotype must not be “miRNA”, “Mt_tRNA”, “ribozyme”, “ncRNA”, “scaRNA”, “scRNA”, “snoRNA”, “snRNA”, “sRNA”, “tRNA”, “vault_RNA”, “TEC” or “artifact”. The host gene biotype was refined into two classes: protein-coding or non-coding (i.e., any biotype other than protein-coding). A representative host gene transcript was chosen (when existing) for each snoRNA in the following way: 1) protein-coding genes were favored over non-coding genes (in the case where two or more potential host genes overlapped); 2) if existing, a host transcript that has an intron overlapping the given snoRNA was favored over a transcript in which an exon would overlap the snoRNA; 3) in the case where multiple host transcripts pass the previous filter, those with a name ending with “-201” were favored over those ending with a name “-202” (and favored over “-203”, “-204”, etc.). SnoRNAs sharing the same host transcript intron were defined as an intronic cluster, whereas those encoded alone in their intron were defined as mono-intronic. When snoRNAs overlapped the same host transcript exon, they were defined as an exonic cluster, whereas those overlapping alone a host transcript exon were

defined as mono-exonic. All snoRNAs not overlapping with any of the potential host transcripts described above were defined as intergenic. Intergenic snoRNAs located at most at 500 nucleotides from the start or end of another intergenic snoRNA were defined as part of an intergenic cluster, whereas intergenic snoRNAs located alone in their genomic region were defined as mono-intergenic.

SnoRNA type information retrieval

The type of snoRNA (C/D or H/ACA box) was retrieved from snoDB v2.0 [2] for human snoRNAs and from the yeast snoRNA database [3] for *Saccharomyces cerevisiae*. For human snoRNAs, AluACA snoRNAs were considered as H/ACA box snoRNAs. For all other species, the snoRNA type was retrieved (if existing) for each snoRNA on RNAcentral v23.0 [4] using a custom Python script. When snoRNA type was still missing, an additional search was conducted using infernal v1.1.2 (cmscan with the parameters –cut_ga –rfam --nohmmonly) [5] and the Rfam covariance models v14.10 [6] to find the Rfam family ID (if existing) of the given snoRNA, with its sequence being used as input. The Rfam family ID of C/D and H/ACA box snoRNAs was manually obtained from the Rfam website [6] and used to define the box type of snoRNAs with a predicted Rfam family. Finally, snoRNAs with still a missing box type were supplemented with this information when available from a previous review on snoRNA genomic location for the following species: *Schizosaccharomyces pombe*, *Caenorhabditis elegans* and *Drosophila melanogaster* [7]. All snoRNAs for which no snoRNA type information was found using the previous approaches were assigned an "Unknown" snoRNA type.

All of the analyses mentioned in these Methods are grouped into a Snakemake workflow available at https://github.com/etiennefc/sno_genomic_organization.git. The resulting datasets of these analyses are presented in the Supplementary Table S1.

## References for the Methods

1. Martin FJ, Amode MR, Aneja A, et al. Ensembl 2023. Nucleic Acids Res. 2023; 51:D933–41.

2. Bergeron D, Paraqindes H, Fafard-Couture É, et al. snoDB 2.0: an enhanced interactive database, specializing in human snoRNAs. Nucleic Acids Res. 2023; 51:D291.

3. Piekna-Przybylska D, Decatur WA, Fournier MJ. New bioinformatic tools for analysis of nucleotide modifications in eukaryotic rRNA. RNA. 2007; 13:305.

4. Sweeney BA, Petrov AI, Ribas CE, et al. RNAcentral 2021: secondary structure integration, improved sequence search and new member databases. Nucleic Acids Res. 2021; 49:D212.

5. Nawrocki EP, Eddy SR. Infernal 1.1: 100-fold faster RNA homology searches. Bioinformatics. 2013; 29:2933.

6. Kalvari I, Nawrocki EP, Argasinska J, et al. Non-coding RNA analysis using the Rfam database. Curr Protoc Bioinforma. 2018; 62:e51.

7. Dieci G, Preti M, Montanini B. Eukaryotic snoRNAs: A paradigm for gene expression flexibility. Genomics. 2009; 94:83–8.

**Supplementary Table S1**: The localization, box type and host gene biotype of snoRNAs across eukaryotes

| gene_id_sno | gene_name_sno | species | kingdom | localization | type_sno | gene_id_host | gene_name_host | gene_biotype_host |
|---|---|---|---|---|---|---|---|---|
| WBGene00007539 | B0334.12 | C_elegans | Animals | mono_intergenic | C/D | | | intergenic |
| WBGene00014290 | C51F7.3 | C_elegans | Animals | mono_intronic | C/D | WBGene00001493 | frm-7 | protein_coding |
| WBGene00014397 | F55A11.10 | C_elegans | Animals | mono_intronic | C/D | WBGene00010079 | F55A11.6 | protein_coding |
| WBGene00014425 | K07C10.2 | C_elegans | Animals | mono_intronic | C/D | WBGene00194851 | K07C10.3 | non_coding |
| WBGene00014447 | M106.6 | C_elegans | Animals | mono_intronic | C/D | WBGene00003367 | mix-1 | protein_coding |
| WBGene00014506 | T06D8.11 | C_elegans | Animals | mono_intronic | C/D | WBGene00011524 | plpr-1 | protein_coding |
| WBGene00014541 | W04D2.7 | C_elegans | Animals | mono_intronic | C/D | WBGene00002133 | inx-11 | protein_coding |
| WBGene00022895 | B0213.18 | C_elegans | Animals | mono_intergenic | C/D | | | intergenic |
| WBGene00022932 | C16D9.10 | C_elegans | Animals | mono_intergenic | C/D | | | intergenic |
| WBGene00022968 | C55F2.3 | C_elegans | Animals | mono_intergenic | C/D | | | intergenic |
| WBGene00023020 | F30H5.4 | C_elegans | Animals | mono_intergenic | C/D | | | intergenic |
| WBGene00023027 | F35H10.12 | C_elegans | Animals | mono_intergenic | C/D | | | intergenic |
| WBGene00023071 | F56C9.12 | C_elegans | Animals | mono_intronic | C/D | WBGene00001748 | gsp-2 | protein_coding |
| WBGene00023083 | H09I01.2 | C_elegans | Animals | mono_intergenic | C/D | | | intergenic |
| WBGene00023183 | Y55F3BR.9 | C_elegans | Animals | mono_intergenic | C/D | | | intergenic |
| WBGene00023189 | Y71D11A.7 | C_elegans | Animals | mono_intronic | C/D | WBGene00022104 | acsd-1 | protein_coding |
| WBGene00023193 | Y74C9A.6 | C_elegans | Animals | mono_intergenic | C/D | | | intergenic |
| WBGene00023428 | K07C5.10 | C_elegans | Animals | mono_intronic | H/ACA | WBGene00010627 | nol-56 | protein_coding |
| WBGene00023429 | W08G11.6 | C_elegans | Animals | mono_intronic | H/ACA | WBGene00012347 | W08G11.3 | protein_coding |
| WBGene00023430 | D1054.17 | C_elegans | Animals | mono_intronic | C/D | WBGene00008371 | D1054.3 | protein_coding |
| WBGene00023461 | F53G12.12 | C_elegans | Animals | mono_intronic | H/ACA | WBGene00004418 | rpl-7 | protein_coding |
| WBGene00023466 | R151.11 | C_elegans | Animals | mono_intronic | H/ACA | WBGene00004417 | rpl-6 | protein_coding |
| WBGene00023467 | Y38F2AR.14 | C_elegans | Animals | mono_intronic | C/D | WBGene00021430 | Y38F2AR.12 | protein_coding |
| WBGene00023468 | B0412.6 | C_elegans | Animals | mono_intronic | H/ACA | WBGene00004498 | rps-29 | protein_coding |
| WBGene00023469 | C16A3.11 | C_elegans | Animals | mono_intronic | H/ACA | WBGene00004482 | rps-13 | protein_coding |
| WBGene00044704 | C18A3.12 | C_elegans | Animals | mono_intergenic | C/D | | | intergenic |
| WBGene00044925 | Y37E3.20 | C_elegans | Animals | mono_intronic | C/D | WBGene00021352 | pcyt-2.1 | protein_coding |
| WBGene00044926 | Y39G10AR.24 | C_elegans | Animals | mono_intergenic | C/D | | | intergenic |
| WBGene00044928 | Y71F9AM.7 | C_elegans | Animals | mono_intronic | H/ACA | WBGene00022121 | cogc-3 | protein_coding |
| WBGene00044929 | R12E2.17 | C_elegans | Animals | mono_intronic | C/D | WBGene00004464 | rpn-8 | protein_coding |
| WBGene00044930 | T19B4.8 | C_elegans | Animals | mono_intergenic | C/D | | | intergenic |
| WBGene00044931 | Y37E3.21 | C_elegans | Animals | mono_intronic | H/ACA | WBGene00021350 | Y37E3.8 | protein_coding |
| WBGene00044932 | Y37E3.22 | C_elegans | Animals | mono_intronic | H/ACA | WBGene00021350 | Y37E3.8 | protein_coding |
| WBGene00044933 | ZK484.9 | C_elegans | Animals | mono_intergenic | C/D | | | intergenic |
| WBGene00044934 | T27A3.9 | C_elegans | Animals | mono_intergenic | C/D | | | intergenic |
| WBGene00044935 | T08B2.13 | C_elegans | Animals | mono_intronic | H/ACA | WBGene00001497 | fars-1 | protein_coding |
| WBGene00044936 | T24H7.6 | C_elegans | Animals | mono_intronic | H/ACA | WBGene00020781 | T24H7.2 | protein_coding |
| WBGene00044937 | ZK546.18 | C_elegans | Animals | mono_intronic | H/ACA | WBGene00007012 | mdt-4 | protein_coding |
| WBGene00044938 | C27H5.9 | C_elegans | Animals | intergenic_cluster | C/D | | | intergenic |
| WBGene00044939 | B0412.7 | C_elegans | Animals | mono_intronic | H/ACA | WBGene00004498 | rps-29 | protein_coding |
| WBGene00044940 | H06I04.8 | C_elegans | Animals | mono_intronic | C/D | WBGene00006725 | ubl-1 | protein_coding |
| WBGene00044941 | F54E7.10 | C_elegans | Animals | mono_intronic | H/ACA | WBGene00004481 | rps-12 | protein_coding |

| | | | | | | | | |
|---|---|---|---|---|---|---|---|---|
| WBGene00044942 | F54E7.11 | C_elegans | Animals | mono_intronic | H/ACA | WBGene00004481 | rps-12 | protein_coding |
| WBGene00044943 | R151.12 | C_elegans | Animals | mono_intronic | H/ACA | WBGene00004417 | rpl-6 | protein_coding |
| WBGene00044944 | R151.13 | C_elegans | Animals | mono_intronic | H/ACA | WBGene00004417 | rpl-6 | protein_coding |
| WBGene00044945 | C07H6.10 | C_elegans | Animals | mono_intronic | C/D | WBGene00000479 | cgh-1 | protein_coding |
| WBGene00044946 | C03B8.5 | C_elegans | Animals | mono_intronic | H/ACA | WBGene00003002 | lin-13 | protein_coding |
| WBGene00044947 | F32E10.10 | C_elegans | Animals | mono_intergenic | C/D | | | intergenic |
| WBGene00044948 | K11H12.12 | C_elegans | Animals | mono_intronic | H/ACA | WBGene00004427 | rpl-15 | protein_coding |
| WBGene00044952 | Y24D9A.9 | C_elegans | Animals | mono_intronic | H/ACA | WBGene00004419 | rpl-7A | protein_coding |
| WBGene00044954 | D2096.14 | C_elegans | Animals | mono_intronic | C/D | WBGene00017075 | nap-1 | protein_coding |
| WBGene00044955 | K07C11.11 | C_elegans | Animals | mono_intronic | H/ACA | WBGene00000098 | air-1 | protein_coding |
| WBGene00044956 | K07C11.12 | C_elegans | Animals | mono_intronic | H/ACA | WBGene00000098 | air-1 | protein_coding |
| WBGene00044957 | C08D8.3 | C_elegans | Animals | mono_intronic | C/D | WBGene00015592 | C08D8.1 | protein_coding |
| WBGene00044958 | ZK994.7 | C_elegans | Animals | mono_intronic | C/D | WBGene00004256 | pxn-1 | protein_coding |
| WBGene00044962 | F28D1.13 | C_elegans | Animals | mono_intronic | H/ACA | WBGene00004492 | rps-23 | protein_coding |
| WBGene00044963 | K07C5.11 | C_elegans | Animals | mono_intronic | H/ACA | WBGene00010627 | nol-56 | protein_coding |
| WBGene00044964 | F22D6.16 | C_elegans | Animals | mono_intronic | H/ACA | WBGene00004186 | prpf-4 | protein_coding |
| WBGene00044970 | F38E11.14 | C_elegans | Animals | mono_intronic | C/D | WBGene00000563 | cng-3 | protein_coding |
| WBGene00044974 | C27H6.5 | C_elegans | Animals | mono_intronic | C/D | WBGene00006777 | unc-41 | protein_coding |
| WBGene00044975 | F47G4.9 | C_elegans | Animals | mono_intergenic | C/D | | | intergenic |
| WBGene00045051 | K11H12.13 | C_elegans | Animals | mono_intronic | H/ACA | WBGene00004427 | rpl-15 | protein_coding |
| WBGene00045055 | C16D9.11 | C_elegans | Animals | mono_intronic | C/D | WBGene00004395 | rol-3 | protein_coding |
| WBGene00045071 | T03F7.8 | C_elegans | Animals | mono_intergenic | C/D | | | intergenic |
| WBGene00045072 | Y43F4B.11 | C_elegans | Animals | intronic_cluster | C/D | WBGene00002229 | klp-19 | protein_coding |
| WBGene00045074 | R166.7 | C_elegans | Animals | mono_intronic | H/ACA | WBGene00011304 | mnk-1 | protein_coding |
| WBGene00045082 | F46F11.12 | C_elegans | Animals | mono_intronic | C/D | WBGene00018512 | trpp-8 | protein_coding |
| WBGene00045083 | C04G6.12 | C_elegans | Animals | mono_intergenic | C/D | | | intergenic |
| WBGene00045084 | K11C4.6 | C_elegans | Animals | mono_intronic | C/D | WBGene00006803 | unc-70 | protein_coding |
| WBGene00045085 | Y24D9A.10 | C_elegans | Animals | mono_intronic | C/D | WBGene00004419 | rpl-7A | protein_coding |
| WBGene00045086 | Y43B11AR.7 | C_elegans | Animals | mono_intronic | C/D | WBGene00004473 | rps-4 | protein_coding |
| WBGene00045087 | B0041.9 | C_elegans | Animals | mono_intronic | C/D | WBGene00004415 | rpl-4 | protein_coding |
| WBGene00045088 | F48A11.7 | C_elegans | Animals | mono_intronic | C/D | WBGene00018586 | ubxn-3 | protein_coding |
| WBGene00045089 | C05H8.3 | C_elegans | Animals | mono_intergenic | C/D | | | intergenic |
| WBGene00045090 | C27H5.10 | C_elegans | Animals | intergenic_cluster | C/D | | | intergenic |
| WBGene00045092 | H06I04.9 | C_elegans | Animals | mono_intronic | C/D | WBGene00006725 | ubl-1 | protein_coding |
| WBGene00045093 | Y23H5B.10 | C_elegans | Animals | mono_intergenic | C/D | | | intergenic |
| WBGene00045094 | D1007.20 | C_elegans | Animals | mono_intronic | H/ACA | WBGene00004436 | rpl-24.1 | protein_coding |
| WBGene00045109 | smy-2 | C_elegans | Animals | mono_intronic | Unknown | WBGene00007882 | C33A12.3 | protein_coding |
| WBGene00045110 | K12D12.6 | C_elegans | Animals | intronic_cluster | C/D | WBGene00010785 | top-2 | protein_coding |
| WBGene00045111 | K12D12.7 | C_elegans | Animals | intronic_cluster | C/D | WBGene00010785 | top-2 | protein_coding |
| WBGene00045112 | K12D12.8 | C_elegans | Animals | intronic_cluster | C/D | WBGene00010785 | top-2 | protein_coding |
| WBGene00045113 | Y43F4B.12 | C_elegans | Animals | intronic_cluster | H/ACA | WBGene00002229 | klp-19 | protein_coding |
| WBGene00045114 | W01C9.6 | C_elegans | Animals | mono_intergenic | H/ACA | | | intergenic |
| WBGene00045115 | K02C4.7 | C_elegans | Animals | mono_intergenic | C/D | | | intergenic |
| WBGene00045118 | T04A8.17 | C_elegans | Animals | mono_intergenic | H/ACA | | | intergenic |
| WBGene00045120 | Y43F8C.22 | C_elegans | Animals | mono_intronic | H/ACA | WBGene00012829 | Y43F8C.7 | protein_coding |

| | | | | | | | | |
|---|---|---|---|---|---|---|---|---|
| WBGene00045121 | C46C2.8 | C_elegans | Animals | mono_intergenic | C/D | | | intergenic |
| WBGene00045124 | C07A9.14 | C_elegans | Animals | mono_intergenic | C/D | | | intergenic |
| WBGene00045125 | F54C9.12 | C_elegans | Animals | mono_intronic | C/D | WBGene00002065 | iff-2 | protein_coding |
| WBGene00045126 | C14C10.8 | C_elegans | Animals | mono_intronic | H/ACA | WBGene00007586 | ril-2 | protein_coding |
| WBGene00045127 | W03C9.9 | C_elegans | Animals | mono_intergenic | C/D | | | intergenic |
| WBGene00045129 | Y75B12B.12 | C_elegans | Animals | mono_intergenic | C/D | | | intergenic |
| WBGene00045130 | F28C6.11 | C_elegans | Animals | mono_intronic | H/ACA | WBGene00004440 | rpl-26 | protein_coding |
| WBGene00045132 | ZK643.9 | C_elegans | Animals | mono_intergenic | H/ACA | | | intergenic |
| WBGene00045134 | K07C5.12 | C_elegans | Animals | mono_intronic | H/ACA | WBGene00010627 | nol-56 | protein_coding |
| WBGene00045136 | D1046.6 | C_elegans | Animals | mono_intronic | H/ACA | WBGene00008362 | cfim-2 | protein_coding |
| WBGene00045137 | F56A12.6 | C_elegans | Animals | mono_intergenic | C/D | | | intergenic |
| WBGene00045138 | F25H2.14 | C_elegans | Animals | mono_intronic | H/ACA | WBGene00009122 | tct-1 | protein_coding |
| WBGene00045139 | F01D5.12 | C_elegans | Animals | mono_intronic | C/D | WBGene00008500 | F01D5.10 | protein_coding |
| WBGene00045140 | T06C12.16 | C_elegans | Animals | mono_intergenic | C/D | | | intergenic |
| WBGene00045141 | Y53F4B.47 | C_elegans | Animals | mono_intergenic | C/D | | | intergenic |
| WBGene00045142 | Y53F4B.48 | C_elegans | Animals | mono_intergenic | H/ACA | | | intergenic |
| WBGene00045147 | T10B9.11 | C_elegans | Animals | mono_intergenic | C/D | | | intergenic |
| WBGene00045148 | C06A1.8 | C_elegans | Animals | mono_intronic | H/ACA | WBGene00007352 | cdc-48.1 | protein_coding |
| WBGene00045149 | W05B2.9 | C_elegans | Animals | mono_intergenic | C/D | | | intergenic |
| WBGene00045150 | ZK265.10 | C_elegans | Animals | mono_intergenic | C/D | | | intergenic |
| WBGene00045151 | F17C11.14 | C_elegans | Animals | mono_intronic | H/ACA | WBGene00008920 | eef-1G | protein_coding |
| WBGene00045152 | C06A1.9 | C_elegans | Animals | mono_intronic | H/ACA | WBGene00007352 | cdc-48.1 | protein_coding |
| WBGene00045153 | F11A10.9 | C_elegans | Animals | mono_intronic | H/ACA | WBGene00008688 | rbm-34 | protein_coding |
| WBGene00045154 | C10C6.8 | C_elegans | Animals | mono_intronic | H/ACA | WBGene00007514 | catp-8 | protein_coding |
| WBGene00045155 | Y116A8C.50 | C_elegans | Animals | mono_intronic | C/D | WBGene00006698 | uaf-2 | protein_coding |
| WBGene00045156 | T02E1.9 | C_elegans | Animals | mono_intergenic | C/D | | | intergenic |
| WBGene00045157 | K02C4.8 | C_elegans | Animals | mono_intergenic | H/ACA | | | intergenic |
| WBGene00045158 | M163.14 | C_elegans | Animals | mono_intergenic | C/D | | | intergenic |
| WBGene00045159 | smy-3 | C_elegans | Animals | mono_intergenic | Unknown | | | intergenic |
| WBGene00045160 | C41C4.11 | C_elegans | Animals | mono_intergenic | C/D | | | intergenic |
| WBGene00045162 | F25H2.15 | C_elegans | Animals | mono_intergenic | C/D | | | intergenic |
| WBGene00045163 | Y67A10A.14 | C_elegans | Animals | mono_intergenic | C/D | | | intergenic |
| WBGene00045164 | F59C6.15 | C_elegans | Animals | mono_intergenic | C/D | | | intergenic |
| WBGene00045167 | F43A11.8 | C_elegans | Animals | mono_intergenic | C/D | | | intergenic |
| WBGene00045170 | T19C4.12 | C_elegans | Animals | mono_intronic | C/D | WBGene00001663 | gpa-1 | protein_coding |
| WBGene00045171 | K09E9.5 | C_elegans | Animals | mono_intergenic | H/ACA | | | intergenic |
| WBGene00045172 | K08E3.11 | C_elegans | Animals | mono_intronic | C/D | WBGene00000875 | cyk-4 | protein_coding |
| WBGene00045176 | C53B7.8 | C_elegans | Animals | mono_intergenic | C/D | | | intergenic |
| WBGene00045367 | F17C11.15 | C_elegans | Animals | mono_intronic | H/ACA | WBGene00008920 | eef-1G | protein_coding |
| WBGene00045368 | T23G5.7 | C_elegans | Animals | mono_intronic | H/ACA | WBGene00004391 | rnr-1 | protein_coding |
| WBGene00045373 | Y46G5A.40 | C_elegans | Animals | mono_intronic | H/ACA | WBGene00012897 | pisy-1 | protein_coding |
| WBGene00045374 | B0393.10 | C_elegans | Animals | mono_intronic | H/ACA | WBGene00007168 | B0393.3 | protein_coding |
| WBGene00045375 | C17D12.8 | C_elegans | Animals | mono_intergenic | C/D | | | intergenic |
| WBGene00045376 | F25H5.9 | C_elegans | Animals | mono_intronic | H/ACA | WBGene00009126 | pyk-1 | protein_coding |
| WBGene00045377 | F19H8.6 | C_elegans | Animals | mono_intronic | C/D | WBGene00008964 | mltn-9 | protein_coding |

| | | | | | | | | |
|---|---|---|---|---|---|---|---|---|
| WBGene00045378 | H04D03.5 | C_elegans | Animals | mono_intergenic | H/ACA | | | intergenic |
| WBGene00077452 | Y62F5A.11 | C_elegans | Animals | mono_intronic | C/D | WBGene00007013 | mdt-8 | protein_coding |
| WBGene00077461 | F12F6.11 | C_elegans | Animals | mono_intergenic | C/D | | | intergenic |
| WBGene00077462 | JC8.15 | C_elegans | Animals | mono_intronic | H/ACA | WBGene00006466 | tag-115 | protein_coding |
| WBGene00077515 | E01A2.9 | C_elegans | Animals | mono_intronic | H/ACA | WBGene00017088 | akir-1 | protein_coding |
| WBGene00077516 | C50C3.13 | C_elegans | Animals | mono_intronic | H/ACA | WBGene00004187 | prp-8 | protein_coding |
| WBGene00077517 | C50C3.14 | C_elegans | Animals | mono_intronic | H/ACA | WBGene00004187 | prp-8 | protein_coding |
| WBGene00195245 | T26A8.6 | C_elegans | Animals | mono_intergenic | C/D | | | intergenic |
| WBGene00197666 | C05D2.15 | C_elegans | Animals | mono_intronic | Unknown | WBGene00015468 | madf-11 | protein_coding |
| WBGene00206375 | T08B6.64 | C_elegans | Animals | mono_intergenic | Unknown | | | intergenic |
| WBGene00219780 | B0228.10 | C_elegans | Animals | mono_intronic | Unknown | WBGene00015061 | cpna-2 | protein_coding |
| WBGene00219782 | B0250.19 | C_elegans | Animals | mono_intronic | Unknown | WBGene00004413 | rpl-2 | protein_coding |
| WBGene00219784 | B0348.8 | C_elegans | Animals | mono_intronic | Unknown | WBGene00001177 | egl-8 | protein_coding |
| WBGene00219787 | B0379.12 | C_elegans | Animals | mono_intronic | Unknown | WBGene00007054 | scpl-1 | protein_coding |
| WBGene00219788 | B0379.13 | C_elegans | Animals | mono_intergenic | Unknown | | | intergenic |
| WBGene00219790 | B0524.10 | C_elegans | Animals | mono_intronic | Unknown | WBGene00004128 | pqn-41 | protein_coding |
| WBGene00219791 | C01B10.48 | C_elegans | Animals | mono_intergenic | Unknown | | | intergenic |
| WBGene00219794 | C02C6.13 | C_elegans | Animals | mono_intronic | Unknown | WBGene00007261 | lron-3 | protein_coding |
| WBGene00219795 | C02F5.15 | C_elegans | Animals | mono_intronic | Unknown | WBGene00004342 | rfs-1 | protein_coding |
| WBGene00219797 | C04A2.16 | C_elegans | Animals | mono_intronic | Unknown | WBGene00001194 | egl-27 | protein_coding |
| WBGene00219798 | C04F12.17 | C_elegans | Animals | mono_intergenic | Unknown | | | intergenic |
| WBGene00219804 | C06E7.95 | C_elegans | Animals | mono_intergenic | Unknown | | | intergenic |
| WBGene00219808 | C09H10.19 | C_elegans | Animals | mono_exonic | Unknown | WBGene00007504 | nbs-1 | protein_coding |
| WBGene00219816 | C14E2.12 | C_elegans | Animals | mono_intergenic | Unknown | | | intergenic |
| WBGene00219818 | C15F1.15 | C_elegans | Animals | mono_intergenic | Unknown | | | intergenic |
| WBGene00219819 | C16D2.4 | C_elegans | Animals | mono_exonic | Unknown | WBGene00195143 | nlp-78 | protein_coding |
| WBGene00219821 | C17D12.18 | C_elegans | Animals | mono_intergenic | Unknown | | | intergenic |
| WBGene00219822 | C17H12.36 | C_elegans | Animals | mono_intergenic | Unknown | | | intergenic |
| WBGene00219823 | C18A3.24 | C_elegans | Animals | mono_intergenic | Unknown | | | intergenic |
| WBGene00219825 | C18D1.21 | C_elegans | Animals | mono_intergenic | Unknown | | | intergenic |
| WBGene00219827 | C23G10.12 | C_elegans | Animals | mono_intergenic | Unknown | | | intergenic |
| WBGene00219828 | C24A1.8 | C_elegans | Animals | mono_intronic | Unknown | WBGene00016029 | elf-1 | protein_coding |
| WBGene00219830 | C24G7.6 | C_elegans | Animals | mono_intronic | Unknown | WBGene00006820 | unc-89 | protein_coding |
| WBGene00219831 | C25F6.30 | C_elegans | Animals | mono_exonic | Unknown | WBGene00016104 | ddr-1 | protein_coding |
| WBGene00219832 | C26B2.14 | C_elegans | Animals | mono_intergenic | Unknown | | | intergenic |
| WBGene00219834 | C26C6.13 | C_elegans | Animals | mono_intergenic | Unknown | | | intergenic |
| WBGene00219836 | C26G2.11 | C_elegans | Animals | mono_intergenic | Unknown | | | intergenic |
| WBGene00219837 | C28D4.17 | C_elegans | Animals | mono_intronic | Unknown | WBGene00003728 | nhr-138 | protein_coding |
| WBGene00219839 | C29F5.11 | C_elegans | Animals | mono_intergenic | Unknown | | | intergenic |
| WBGene00219842 | C30C11.15 | C_elegans | Animals | mono_intronic | Unknown | WBGene00018369 | mig-39 | protein_coding |
| WBGene00219845 | C32F10.9 | C_elegans | Animals | mono_intergenic | Unknown | | | intergenic |
| WBGene00219846 | C33E10.11 | C_elegans | Animals | mono_intergenic | Unknown | | | intergenic |
| WBGene00219847 | C34E11.20 | C_elegans | Animals | mono_intergenic | Unknown | | | intergenic |
| WBGene00219850 | C36E8.11 | C_elegans | Animals | mono_intergenic | Unknown | | | intergenic |
| WBGene00219852 | C41C4.16 | C_elegans | Animals | mono_intronic | Unknown | WBGene00006739 | ulp-4 | protein_coding |

| | | | | | | | | |
|---|---|---|---|---|---|---|---|---|
| WBGene00219853 | C41C4.17 | C_elegans | Animals | mono_exonic | Unknown | WBGene00006739 | ulp-4 | protein_coding |
| WBGene00219860 | C51F7.4 | C_elegans | Animals | mono_exonic | Unknown | WBGene00001493 | frm-7 | protein_coding |
| WBGene00219862 | C52G5.13 | C_elegans | Animals | mono_intronic | Unknown | WBGene00001560 | gei-3 | protein_coding |
| WBGene00219863 | C54G4.16 | C_elegans | Animals | mono_intergenic | Unknown | | | intergenic |
| WBGene00219864 | C56G2.20 | C_elegans | Animals | mono_intronic | Unknown | WBGene00016977 | akap-1 | protein_coding |
| WBGene00219868 | D2021.6 | C_elegans | Animals | mono_intergenic | Unknown | | | intergenic |
| WBGene00219869 | D2023.18 | C_elegans | Animals | mono_intergenic | Unknown | | | intergenic |
| WBGene00219871 | E01A2.11 | C_elegans | Animals | mono_intronic | Unknown | WBGene00017088 | akir-1 | protein_coding |
| WBGene00219873 | EEED8.19 | C_elegans | Animals | mono_intronic | Unknown | WBGene00017143 | EEED8.15 | protein_coding |
| WBGene00219879 | F07A11.15 | C_elegans | Animals | mono_intergenic | Unknown | | | intergenic |
| WBGene00219888 | F09G2.14 | C_elegans | Animals | mono_intergenic | Unknown | | | intergenic |
| WBGene00219894 | F16A11.12 | C_elegans | Animals | mono_intronic | Unknown | WBGene00008878 | ppfr-1 | protein_coding |
| WBGene00219899 | F17C11.24 | C_elegans | Animals | mono_intergenic | Unknown | | | intergenic |
| WBGene00219900 | F17C11.25 | C_elegans | Animals | mono_exonic | Unknown | WBGene00077761 | zip-9 | protein_coding |
| WBGene00219901 | F18A1.11 | C_elegans | Animals | mono_intronic | Unknown | WBGene00003044 | lir-1 | protein_coding |
| WBGene00219902 | F18C5.15 | C_elegans | Animals | mono_intronic | Unknown | WBGene00007050 | utp-20 | protein_coding |
| WBGene00219906 | F19G12.14 | C_elegans | Animals | mono_intergenic | Unknown | | | intergenic |
| WBGene00219907 | F19H8.7 | C_elegans | Animals | mono_intronic | Unknown | WBGene00006603 | tps-2 | protein_coding |
| WBGene00219909 | F20D12.10 | C_elegans | Animals | mono_intergenic | Unknown | | | intergenic |
| WBGene00219911 | F21F12.3 | C_elegans | Animals | mono_intergenic | Unknown | | | intergenic |
| WBGene00219914 | F25G6.21 | C_elegans | Animals | mono_intronic | Unknown | WBGene00017798 | slc-17.8 | protein_coding |
| WBGene00219915 | F26A10.28 | C_elegans | Animals | mono_exonic | Unknown | WBGene00017814 | F26A10.2 | protein_coding |
| WBGene00219917 | F26D10.24 | C_elegans | Animals | mono_intronic | Unknown | WBGene00002005 | hsp-1 | protein_coding |
| WBGene00219923 | F28D9.6 | C_elegans | Animals | mono_intergenic | Unknown | | | intergenic |
| WBGene00219925 | F29G9.19 | C_elegans | Animals | mono_exonic | Unknown | WBGene00001345 | fos-1 | protein_coding |
| WBGene00219926 | F30A10.16 | C_elegans | Animals | mono_intergenic | Unknown | | | intergenic |
| WBGene00219929 | F32B4.12 | C_elegans | Animals | mono_intronic | Unknown | WBGene00009315 | F32B4.5 | protein_coding |
| WBGene00219930 | F32B5.17 | C_elegans | Animals | mono_intergenic | Unknown | | | intergenic |
| WBGene00219932 | F33E11.7 | C_elegans | Animals | mono_intronic | Unknown | WBGene00003614 | nhr-15 | protein_coding |
| WBGene00219936 | F37A8.7 | C_elegans | Animals | mono_intergenic | Unknown | | | intergenic |
| WBGene00219937 | F37F2.4 | C_elegans | Animals | mono_intergenic | Unknown | | | intergenic |
| WBGene00219940 | F39B2.14 | C_elegans | Animals | mono_exonic | Unknown | WBGene00043308 | tmem-107 | protein_coding |
| WBGene00219944 | F41E6.18 | C_elegans | Animals | mono_exonic | Unknown | WBGene00018285 | smk-1 | protein_coding |
| WBGene00219945 | F42A8.4 | C_elegans | Animals | mono_intergenic | Unknown | | | intergenic |
| WBGene00219946 | F42H10.17 | C_elegans | Animals | mono_exonic | Unknown | WBGene00018369 | mig-39 | protein_coding |
| WBGene00219954 | F47E1.19 | C_elegans | Animals | mono_exonic | Unknown | WBGene00219699 | linc-22 | non_coding |
| WBGene00219955 | F48A9.6 | C_elegans | Animals | mono_intronic | Unknown | WBGene00002783 | let-607 | protein_coding |
| WBGene00219957 | F48F5.7 | C_elegans | Animals | mono_intergenic | Unknown | | | intergenic |
| WBGene00219964 | F52G3.6 | C_elegans | Animals | mono_exonic | Unknown | WBGene00022951 | C30E1.9 | non_coding |
| WBGene00219967 | F54E4.17 | C_elegans | Animals | mono_intergenic | Unknown | | | intergenic |
| WBGene00219969 | F54F11.6 | C_elegans | Animals | mono_intronic | Unknown | WBGene00010071 | sre-45 | protein_coding |
| WBGene00219971 | F54G2.4 | C_elegans | Animals | mono_intronic | Unknown | WBGene00018837 | F54G2.1 | protein_coding |
| WBGene00219972 | F55A3.8 | C_elegans | Animals | mono_intergenic | Unknown | | | intergenic |
| WBGene00219974 | F55F1.11 | C_elegans | Animals | mono_intronic | Unknown | WBGene00006496 | cgef-1 | protein_coding |
| WBGene00219976 | F55H12.9 | C_elegans | Animals | mono_intergenic | Unknown | | | intergenic |

| | | | | | | | | |
|---|---|---|---|---|---|---|---|---|
| WBGene00219978 | F56A11.14 | C_elegans | Animals | mono_intronic | Unknown | WBGene00001165 | efn-4 | protein_coding |
| WBGene00219983 | F58D5.11 | C_elegans | Animals | mono_exonic | Unknown | WBGene00010244 | F58D5.5 | protein_coding |
| WBGene00219984 | F58D5.12 | C_elegans | Animals | mono_exonic | Unknown | WBGene00010244 | F58D5.5 | protein_coding |
| WBGene00219986 | F59A2.7 | C_elegans | Animals | mono_intronic | Unknown | WBGene00010306 | golg-4 | protein_coding |
| WBGene00219988 | F59G1.14 | C_elegans | Animals | mono_intronic | Unknown | WBGene00019127 | cgt-3 | protein_coding |
| WBGene00219998 | K02A11.12 | C_elegans | Animals | mono_intergenic | Unknown | | | intergenic |
| WBGene00219999 | K02B9.10 | C_elegans | Animals | mono_exonic | Unknown | WBGene00000105 | alg-1 | protein_coding |
| WBGene00220001 | K02F3.15 | C_elegans | Animals | mono_intronic | Unknown | WBGene00019327 | zip-2 | protein_coding |
| WBGene00220002 | K02F6.11 | C_elegans | Animals | mono_intergenic | Unknown | | | intergenic |
| WBGene00220005 | K04D7.17 | C_elegans | Animals | mono_intergenic | Unknown | | | intergenic |
| WBGene00220008 | K07C5.18 | C_elegans | Animals | mono_intronic | Unknown | WBGene00010627 | nol-56 | protein_coding |
| WBGene00220009 | K07E1.2 | C_elegans | Animals | mono_intergenic | Unknown | | | intergenic |
| WBGene00220012 | K08F11.8 | C_elegans | Animals | mono_intergenic | Unknown | | | intergenic |
| WBGene00220015 | K09H9.11 | C_elegans | Animals | mono_exonic | Unknown | WBGene00006888 | vbh-1 | protein_coding |
| WBGene00220017 | K10D2.10 | C_elegans | Animals | mono_intergenic | Unknown | | | intergenic |
| WBGene00220019 | K10D3.10 | C_elegans | Animals | mono_intronic | Unknown | WBGene00010738 | K10D3.4 | protein_coding |
| WBGene00220021 | K10G6.10 | C_elegans | Animals | mono_intronic | Unknown | WBGene00004751 | sea-2 | protein_coding |
| WBGene00220022 | K10G6.11 | C_elegans | Animals | mono_exonic | Unknown | WBGene00004751 | sea-2 | protein_coding |
| WBGene00220024 | K11D9.10 | C_elegans | Animals | mono_intergenic | Unknown | | | intergenic |
| WBGene00220026 | M01D1.11 | C_elegans | Animals | mono_intergenic | Unknown | | | intergenic |
| WBGene00220029 | M03D4.78 | C_elegans | Animals | mono_intergenic | Unknown | | | intergenic |
| WBGene00220032 | M7.15 | C_elegans | Animals | mono_intergenic | Unknown | | | intergenic |
| WBGene00220037 | R07G3.16 | C_elegans | Animals | mono_intergenic | Unknown | | | intergenic |
| WBGene00220038 | R08C7.19 | C_elegans | Animals | mono_exonic | Unknown | WBGene00197297 | R08C7.17 | non_coding |
| WBGene00220039 | R10A10.3 | C_elegans | Animals | mono_intergenic | Unknown | | | intergenic |
| WBGene00220041 | R11A5.8 | C_elegans | Animals | mono_intergenic | Unknown | | | intergenic |
| WBGene00220050 | R144.18 | C_elegans | Animals | mono_exonic | Unknown | WBGene00020097 | larp-1 | protein_coding |
| WBGene00220052 | R166.10 | C_elegans | Animals | mono_intergenic | Unknown | | | intergenic |
| WBGene00220056 | T01C2.6 | C_elegans | Animals | mono_intronic | Unknown | WBGene00000071 | acy-4 | protein_coding |
| WBGene00220058 | T05A6.20 | C_elegans | Animals | mono_intergenic | Unknown | | | intergenic |
| WBGene00220059 | T06E4.20 | C_elegans | Animals | mono_intergenic | Unknown | | | intergenic |
| WBGene00220061 | T07D4.7 | C_elegans | Animals | mono_intergenic | Unknown | | | intergenic |
| WBGene00220062 | T08D2.11 | C_elegans | Animals | mono_exonic | Unknown | WBGene00011613 | T08D2.8 | protein_coding |
| WBGene00220063 | T08H4.9 | C_elegans | Animals | mono_intronic | Unknown | WBGene00006476 | rhgf-2 | protein_coding |
| WBGene00220065 | T09B4.22 | C_elegans | Animals | mono_intergenic | Unknown | | | intergenic |
| WBGene00220066 | T09B9.10 | C_elegans | Animals | mono_intergenic | Unknown | | | intergenic |
| WBGene00220069 | T13E5.1 | C_elegans | Animals | mono_intergenic | Unknown | | | intergenic |
| WBGene00220071 | T15H9.8 | C_elegans | Animals | mono_intronic | Unknown | WBGene00001038 | dnj-20 | protein_coding |
| WBGene00220073 | T19E7.29 | C_elegans | Animals | mono_intergenic | Unknown | | | intergenic |
| WBGene00220075 | T21B4.20 | C_elegans | Animals | mono_intergenic | Unknown | | | intergenic |
| WBGene00220078 | T21F2.3 | C_elegans | Animals | mono_intergenic | Unknown | | | intergenic |
| WBGene00220080 | T21H8.11 | C_elegans | Animals | mono_intergenic | Unknown | | | intergenic |
| WBGene00220083 | T23B12.13 | C_elegans | Animals | mono_intronic | Unknown | WBGene00020718 | mrps-2 | protein_coding |
| WBGene00220085 | T23D8.11 | C_elegans | Animals | mono_exonic | Unknown | WBGene00011945 | alg-5 | protein_coding |
| WBGene00220087 | T23D8.13 | C_elegans | Animals | mono_exonic | Unknown | WBGene00000476 | cfi-1 | protein_coding |

| | | | | | | | | |
|---|---|---|---|---|---|---|---|---|
| WBGene00220089 | T23G11.12 | C_elegans | Animals | mono_intergenic | Unknown | | | intergenic |
| WBGene00220092 | T24H10.12 | C_elegans | Animals | mono_intergenic | Unknown | | | intergenic |
| WBGene00220093 | T25C12.11 | C_elegans | Animals | mono_exonic | Unknown | WBGene00003003 | lin-14 | protein_coding |
| WBGene00220094 | T25C12.12 | C_elegans | Animals | mono_exonic | Unknown | WBGene00003003 | lin-14 | protein_coding |
| WBGene00220095 | T25D3.8 | C_elegans | Animals | mono_intergenic | Unknown | | | intergenic |
| WBGene00220096 | T25D10.11 | C_elegans | Animals | mono_intergenic | Unknown | | | intergenic |
| WBGene00220099 | T26H10.10 | C_elegans | Animals | mono_intergenic | Unknown | | | intergenic |
| WBGene00220101 | T27A8.8 | C_elegans | Animals | mono_intergenic | Unknown | | | intergenic |
| WBGene00220102 | T27C4.7 | C_elegans | Animals | mono_intronic | Unknown | WBGene00003025 | lin-40 | protein_coding |
| WBGene00220106 | T28B8.11 | C_elegans | Animals | mono_intergenic | Unknown | | | intergenic |
| WBGene00220107 | W01A11.21 | C_elegans | Animals | mono_intronic | Unknown | WBGene00006815 | unc-83 | protein_coding |
| WBGene00220111 | W01H2.11 | C_elegans | Animals | mono_intergenic | Unknown | | | intergenic |
| WBGene00220112 | W02B9.3 | C_elegans | Animals | mono_intronic | Unknown | WBGene00001980 | hmr-1 | protein_coding |
| WBGene00220117 | W03B1.17 | C_elegans | Animals | mono_intergenic | Unknown | | | intergenic |
| WBGene00220120 | W05G11.7 | C_elegans | Animals | mono_intergenic | Unknown | | | intergenic |
| WBGene00220121 | W06A7.12 | C_elegans | Animals | mono_intronic | Unknown | WBGene00004336 | ret-1 | protein_coding |
| WBGene00220122 | W06D12.9 | C_elegans | Animals | mono_intergenic | Unknown | | | intergenic |
| WBGene00220124 | W06H8.17 | C_elegans | Animals | mono_exonic | Unknown | WBGene00199016 | W06H8.12 | non_coding |
| WBGene00220125 | W07G1.23 | C_elegans | Animals | mono_intergenic | Unknown | | | intergenic |
| WBGene00220126 | W10C8.17 | C_elegans | Animals | mono_intronic | Unknown | WBGene00004077 | pop-1 | protein_coding |
| WBGene00220128 | Y6B3B.15 | C_elegans | Animals | mono_intergenic | Unknown | | | intergenic |
| WBGene00220132 | Y11D7A.22 | C_elegans | Animals | mono_intronic | Unknown | WBGene00012437 | nmy-3 | protein_coding |
| WBGene00220134 | Y17G7B.25 | C_elegans | Animals | mono_intergenic | Unknown | | | intergenic |
| WBGene00220139 | Y32G9A.15 | C_elegans | Animals | mono_intronic | Unknown | WBGene00021305 | zig-11 | protein_coding |
| WBGene00220143 | Y38E10A.32 | C_elegans | Animals | mono_intergenic | Unknown | | | intergenic |
| WBGene00220144 | Y39A1A.30 | C_elegans | Animals | mono_intronic | Unknown | WBGene00000566 | cnt-2 | protein_coding |
| WBGene00220145 | Y39A3CR.11 | C_elegans | Animals | mono_intronic | Unknown | WBGene00021444 | ttc-7 | protein_coding |
| WBGene00220147 | Y40B1A.6 | C_elegans | Animals | mono_intergenic | Unknown | | | intergenic |
| WBGene00220155 | Y41D4B.36 | C_elegans | Animals | mono_exonic | Unknown | WBGene00021512 | Y41D4B.4 | protein_coding |
| WBGene00220157 | Y41E3.464 | C_elegans | Animals | mono_intergenic | Unknown | | | intergenic |
| WBGene00220159 | Y44A6C.3 | C_elegans | Animals | mono_intergenic | Unknown | | | intergenic |
| WBGene00220161 | Y46E12BL.11 | C_elegans | Animals | mono_exonic | Unknown | WBGene00021597 | spsb-1 | protein_coding |
| WBGene00220164 | Y47D3B.25 | C_elegans | Animals | mono_intergenic | Unknown | | | intergenic |
| WBGene00220167 | Y47G6A.38 | C_elegans | Animals | mono_intronic | Unknown | WBGene00021645 | Y47G6A.19 | protein_coding |
| WBGene00220168 | Y48A6C.10 | C_elegans | Animals | mono_intergenic | Unknown | | | intergenic |
| WBGene00220172 | Y49E10.36 | C_elegans | Animals | mono_intronic | Unknown | WBGene00001946 | his-72 | protein_coding |
| WBGene00220174 | Y51A2D.40 | C_elegans | Animals | mono_intronic | Unknown | WBGene00013082 | grdn-1 | protein_coding |
| WBGene00220176 | Y51H4A.940 | C_elegans | Animals | mono_intergenic | Unknown | | | intergenic |
| WBGene00220180 | Y54G2A.74 | C_elegans | Animals | mono_exonic | Unknown | WBGene00043702 | Y54G2A.7 | protein_coding |
| WBGene00220181 | Y55F3AM.23 | C_elegans | Animals | mono_exonic | Unknown | WBGene00043705 | egrh-2 | protein_coding |
| WBGene00220182 | Y55F3BL.5 | C_elegans | Animals | mono_exonic | Unknown | WBGene00001154 | ech-5 | protein_coding |
| WBGene00220185 | Y57G11C.1145 | C_elegans | Animals | mono_intergenic | Unknown | | | intergenic |
| WBGene00220188 | Y65B4A.10 | C_elegans | Animals | mono_exonic | Unknown | WBGene00022029 | Y65B4A.6 | protein_coding |
| WBGene00220189 | Y65B4A.11 | C_elegans | Animals | mono_exonic | Unknown | WBGene00022029 | Y65B4A.6 | protein_coding |
| WBGene00220191 | Y65B4BR.12 | C_elegans | Animals | mono_intronic | Unknown | WBGene00007009 | wwp-1 | protein_coding |

| | | | | | | | | |
|---|---|---|---|---|---|---|---|---|
| WBGene00220192 | Y66H1B.6 | C_elegans | Animals | mono_intronic | Unknown | WBGene00022048 | fln-1 | protein_coding |
| WBGene00220195 | Y71F9AL.24 | C_elegans | Animals | mono_intronic | Unknown | WBGene00000199 | arx-1 | protein_coding |
| WBGene00220196 | Y71G10AR.5 | C_elegans | Animals | mono_intronic | Unknown | WBGene00022134 | hrpu-2 | protein_coding |
| WBGene00220197 | Y71G12B.41 | C_elegans | Animals | mono_exonic | Unknown | WBGene00236797 | Y71G12B.44 | non_coding |
| WBGene00220201 | Y73B3A.30 | C_elegans | Animals | mono_intergenic | Unknown | | | intergenic |
| WBGene00220202 | Y73B3A.31 | C_elegans | Animals | mono_exonic | Unknown | WBGene00023191 | | non_coding |
| WBGene00220203 | Y73B6A.14 | C_elegans | Animals | mono_intronic | Unknown | WBGene00022228 | Y73B6A.1 | protein_coding |
| WBGene00220204 | Y73B6A.15 | C_elegans | Animals | mono_intergenic | Unknown | | | intergenic |
| WBGene00220205 | Y73F8A.1175 | C_elegans | Animals | mono_intergenic | Unknown | | | intergenic |
| WBGene00220206 | Y75B7AL.9 | C_elegans | Animals | mono_intergenic | Unknown | | | intergenic |
| WBGene00220211 | Y76B12C.11 | C_elegans | Animals | mono_exonic | Unknown | WBGene00022300 | Y76B12C.6 | protein_coding |
| WBGene00220212 | Y76G2B.1 | C_elegans | Animals | mono_intergenic | Unknown | | | intergenic |
| WBGene00220213 | Y77E11A.21 | C_elegans | Animals | mono_intergenic | Unknown | | | intergenic |
| WBGene00220215 | Y92C3B.4 | C_elegans | Animals | mono_intronic | Unknown | WBGene00006697 | uaf-1 | protein_coding |
| WBGene00220217 | Y95B8A.18 | C_elegans | Animals | mono_exonic | Unknown | WBGene00001678 | gpa-16 | protein_coding |
| WBGene00220219 | Y102A11A.16 | C_elegans | Animals | intergenic_cluster | Unknown | | | intergenic |
| WBGene00220220 | Y102A11A.17 | C_elegans | Animals | intergenic_cluster | Unknown | | | intergenic |
| WBGene00220222 | Y105C5A.1283 | C_elegans | Animals | mono_intergenic | Unknown | | | intergenic |
| WBGene00220224 | Y105C5B.1419 | C_elegans | Animals | mono_intronic | Unknown | WBGene00002175 | jac-1 | protein_coding |
| WBGene00220227 | Y110A2AL.20 | C_elegans | Animals | mono_exonic | Unknown | WBGene00022450 | ubc-26 | protein_coding |
| WBGene00220229 | Y111B2A.35 | C_elegans | Animals | intronic_cluster | Unknown | WBGene00007027 | ssl-1 | protein_coding |
| WBGene00220230 | Y111B2A.36 | C_elegans | Animals | intronic_cluster | Unknown | WBGene00007027 | ssl-1 | protein_coding |
| WBGene00220231 | Y119C1B.13 | C_elegans | Animals | mono_intergenic | Unknown | | | intergenic |
| WBGene00220233 | ZC395.12 | C_elegans | Animals | mono_intergenic | C/D | | | intergenic |
| WBGene00220234 | ZC410.9 | C_elegans | Animals | mono_intergenic | Unknown | | | intergenic |
| WBGene00220236 | ZK20.9 | C_elegans | Animals | mono_intergenic | Unknown | | | intergenic |
| WBGene00220238 | ZK185.9 | C_elegans | Animals | mono_intergenic | Unknown | | | intergenic |
| WBGene00220243 | ZK484.11 | C_elegans | Animals | mono_intergenic | Unknown | | | intergenic |
| WBGene00220244 | ZK632.18 | C_elegans | Animals | mono_intronic | Unknown | WBGene00000567 | cnx-1 | protein_coding |
| WBGene00220246 | ZK858.10 | C_elegans | Animals | mono_exonic | Unknown | WBGene00014115 | gld-4 | protein_coding |
| WBGene00220248 | ZK930.15 | C_elegans | Animals | mono_intergenic | Unknown | | | intergenic |
| WBGene00235198 | T09H2.2 | C_elegans | Animals | mono_intergenic | Unknown | | | intergenic |
| WBGene00235200 | T27A8.9 | C_elegans | Animals | mono_intergenic | Unknown | | | intergenic |
| WBGene00235235 | Y59E1B.14 | C_elegans | Animals | mono_intergenic | H/ACA | | | intergenic |
| ENSDARG00000080156 | SNORD30 | D_rerio | Animals | mono_intronic | C/D | ENSDARG00000103682 | si:dkey-23i12.5 | non_coding |
| ENSDARG00000080158 | SNORD18 | D_rerio | Animals | mono_intronic | C/D | ENSDARG00000041182 | rpl4 | protein_coding |
| ENSDARG00000080172 | ENSDARG00000080172 | D_rerio | Animals | mono_intronic | C/D | ENSDARG00000042094 | eef2a.1 | protein_coding |
| ENSDARG00000080188 | ENSDARG00000080188 | D_rerio | Animals | intergenic_cluster | C/D | | | intergenic |
| ENSDARG00000080205 | ENSDARG00000080205 | D_rerio | Animals | mono_intronic | C/D | ENSDARG00000092337 | | non_coding |
| ENSDARG00000080351 | ENSDARG00000080351 | D_rerio | Animals | mono_intronic | H/ACA | ENSDARG00000036298 | rps13 | protein_coding |
| ENSDARG00000080372 | ENSDARG00000080372 | D_rerio | Animals | mono_intronic | C/D | ENSDARG00000097452 | | non_coding |
| ENSDARG00000080422 | ENSDARG00000080422 | D_rerio | Animals | mono_intronic | C/D | ENSDARG00000053058 | rps11 | protein_coding |
| ENSDARG00000080424 | ENSDARG00000080424 | D_rerio | Animals | mono_intronic | C/D | ENSDARG00000097595 | | non_coding |
| ENSDARG00000080450 | ENSDARG00000080450 | D_rerio | Animals | mono_intronic | H/ACA | ENSDARG00000041182 | rpl4 | protein_coding |
| ENSDARG00000080462 | ENSDARG00000080462 | D_rerio | Animals | mono_intronic | Unknown | ENSDARG00000094097 | | non_coding |

| | | | | | | | | |
|---|---|---|---|---|---|---|---|---|
| ENSDARG00000080493 | ENSDARG00000080493 | D_rerio | Animals | mono_intronic | C/D | ENSDARG00000026842 | acin1b | protein_coding |
| ENSDARG00000080499 | ENSDARG00000080499 | D_rerio | Animals | mono_intronic | H/ACA | ENSDARG00000092780 | | protein_coding |
| ENSDARG00000080523 | ENSDARG00000080523 | D_rerio | Animals | mono_intronic | C/D | ENSDARG00000016477 | eif4a2 | protein_coding |
| ENSDARG00000080531 | SNORD31 | D_rerio | Animals | mono_intronic | C/D | ENSDARG00000103682 | si:dkey-23i12.5 | non_coding |
| ENSDARG00000080532 | SNORA71 | D_rerio | Animals | mono_intronic | H/ACA | ENSDARG00000046157 | RPS17 | protein_coding |
| ENSDARG00000080633 | U8 | D_rerio | Animals | mono_intergenic | C/D | | | intergenic |
| ENSDARG00000080636 | SNORD18 | D_rerio | Animals | mono_intronic | C/D | ENSDARG00000041182 | rpl4 | protein_coding |
| ENSDARG00000080647 | ENSDARG00000080647 | D_rerio | Animals | mono_intronic | H/ACA | ENSDARG00000010137 | ldb1a | protein_coding |
| ENSDARG00000080685 | ENSDARG00000080685 | D_rerio | Animals | mono_intergenic | H/ACA | | | intergenic |
| ENSDARG00000080698 | SNORD29 | D_rerio | Animals | mono_intronic | C/D | ENSDARG00000103682 | si:dkey-23i12.5 | non_coding |
| ENSDARG00000080744 | ENSDARG00000080744 | D_rerio | Animals | mono_intronic | C/D | ENSDARG00000103553 | sf3b3 | protein_coding |
| ENSDARG00000080775 | ENSDARG00000080775 | D_rerio | Animals | intergenic_cluster | C/D | | | intergenic |
| ENSDARG00000080777 | ENSDARG00000080777 | D_rerio | Animals | mono_intronic | H/ACA | ENSDARG00000040666 | nifk | protein_coding |
| ENSDARG00000080823 | SNORA73 | D_rerio | Animals | mono_intronic | H/ACA | ENSDARG00000042566 | rps7 | protein_coding |
| ENSDARG00000080840 | ENSDARG00000080840 | D_rerio | Animals | mono_intronic | H/ACA | ENSDARG00000092780 | | protein_coding |
| ENSDARG00000080855 | SNORA71 | D_rerio | Animals | mono_intronic | H/ACA | ENSDARG00000046157 | RPS17 | protein_coding |
| ENSDARG00000080895 | SNORD97 | D_rerio | Animals | mono_intronic | C/D | ENSDARG00000057167 | eif4g2b | protein_coding |
| ENSDARG00000080924 | SNORD79 | D_rerio | Animals | mono_intronic | C/D | ENSDARG00000092337 | | non_coding |
| ENSDARG00000080936 | SNORD36 | D_rerio | Animals | mono_intronic | C/D | ENSDARG00000019230 | rpl7a | protein_coding |
| ENSDARG00000080938 | SNORD70B | D_rerio | Animals | mono_intronic | C/D | ENSDARG00000104353 | nop58 | protein_coding |
| ENSDARG00000080942 | ENSDARG00000080942 | D_rerio | Animals | mono_intronic | C/D | ENSDARG00000006200 | eif4g1a | protein_coding |
| ENSDARG00000081030 | ENSDARG00000081030 | D_rerio | Animals | mono_intronic | C/D | ENSDARG00000005513 | naca | protein_coding |
| ENSDARG00000081082 | ENSDARG00000081082 | D_rerio | Animals | mono_intergenic | H/ACA | | | intergenic |
| ENSDARG00000081103 | ENSDARG00000081103 | D_rerio | Animals | mono_intronic | C/D | ENSDARG00000092115 | eif4a1a | protein_coding |
| ENSDARG00000081126 | ENSDARG00000081126 | D_rerio | Animals | mono_intronic | C/D | ENSDARG00000005058 | ncapd2 | protein_coding |
| ENSDARG00000081379 | U8 | D_rerio | Animals | mono_intergenic | C/D | | | intergenic |
| ENSDARG00000081440 | SNORA75 | D_rerio | Animals | mono_intronic | H/ACA | ENSDARG00000002710 | ncl | protein_coding |
| ENSDARG00000081450 | ENSDARG00000081450 | D_rerio | Animals | mono_intronic | C/D | ENSDARG00000007320 | rpl7 | protein_coding |
| ENSDARG00000081568 | SNORD33 | D_rerio | Animals | mono_intronic | C/D | ENSDARG00000044093 | rpl13a | protein_coding |
| ENSDARG00000081580 | ENSDARG00000081580 | D_rerio | Animals | mono_intergenic | C/D | | | intergenic |
| ENSDARG00000081621 | ENSDARG00000081621 | D_rerio | Animals | mono_intronic | C/D | ENSDARG00000034291 | rpl37 | protein_coding |
| ENSDARG00000081623 | ENSDARG00000081623 | D_rerio | Animals | mono_intronic | C/D | ENSDARG00000026842 | acin1b | protein_coding |
| ENSDARG00000081648 | ENSDARG00000081648 | D_rerio | Animals | mono_intronic | H/ACA | ENSDARG00000056318 | kansl2 | protein_coding |
| ENSDARG00000081654 | ENSDARG00000081654 | D_rerio | Animals | mono_intronic | C/D | ENSDARG00000036875 | rps12 | protein_coding |
| ENSDARG00000081702 | SNORA63 | D_rerio | Animals | mono_intronic | H/ACA | ENSDARG00000051783 | rplp0 | protein_coding |
| ENSDARG00000081799 | ENSDARG00000081799 | D_rerio | Animals | mono_intergenic | C/D | | | intergenic |
| ENSDARG00000081802 | ENSDARG00000081802 | D_rerio | Animals | mono_intronic | C/D | ENSDARG00000097452 | | non_coding |
| ENSDARG00000081849 | SNORD58 | D_rerio | Animals | mono_intronic | C/D | ENSDARG00000092693 | tpt1 | protein_coding |
| ENSDARG00000081850 | ENSDARG00000081850 | D_rerio | Animals | mono_intronic | H/ACA | ENSDARG00000094097 | | non_coding |
| ENSDARG00000081875 | ENSDARG00000081875 | D_rerio | Animals | mono_intronic | C/D | ENSDARG00000036875 | rps12 | protein_coding |
| ENSDARG00000081895 | ENSDARG00000081895 | D_rerio | Animals | mono_intronic | H/ACA | ENSDARG00000057010 | | protein_coding |
| ENSDARG00000081926 | SNORD33 | D_rerio | Animals | mono_intronic | C/D | ENSDARG00000044093 | rpl13a | protein_coding |
| ENSDARG00000081931 | ENSDARG00000081931 | D_rerio | Animals | mono_intergenic | H/ACA | | | intergenic |
| ENSDARG00000081937 | ENSDARG00000081937 | D_rerio | Animals | mono_intronic | H/ACA | ENSDARG00000094097 | | non_coding |
| ENSDARG00000081943 | SNORA62 | D_rerio | Animals | mono_intronic | H/ACA | ENSDARG00000019181 | rpsa | protein_coding |

| | | | | | | | | |
|---|---|---|---|---|---|---|---|---|
| ENSDARG00000081946 | ENSDARG00000081946 | D_rerio | Animals | mono_intronic | C/D | ENSDARG00000097452 | | non_coding |
| ENSDARG00000081961 | ENSDARG00000081961 | D_rerio | Animals | mono_intronic | C/D | ENSDARG00000078069 | rrm2 | protein_coding |
| ENSDARG00000081972 | ENSDARG00000081972 | D_rerio | Animals | mono_intronic | C/D | ENSDARG00000075380 | kri1 | protein_coding |
| ENSDARG00000082008 | SNORD14 | D_rerio | Animals | mono_intronic | C/D | ENSDARG00000068992 | hspa8 | protein_coding |
| ENSDARG00000082020 | ENSDARG00000082020 | D_rerio | Animals | mono_intronic | H/ACA | ENSDARG00000036316 | rpl39 | protein_coding |
| ENSDARG00000082087 | SNORD31 | D_rerio | Animals | mono_intronic | C/D | ENSDARG00000103682 | si:dkey-23i12.5 | non_coding |
| ENSDARG00000082213 | ENSDARG00000082213 | D_rerio | Animals | mono_intronic | C/D | ENSDARG00000053058 | rps11 | protein_coding |
| ENSDARG00000082242 | SNORA73 | D_rerio | Animals | mono_intronic | H/ACA | ENSDARG00000042566 | rps7 | protein_coding |
| ENSDARG00000082290 | ENSDARG00000082290 | D_rerio | Animals | mono_intronic | H/ACA | ENSDARG00000040245 | kpnb3 | protein_coding |
| ENSDARG00000082419 | ENSDARG00000082419 | D_rerio | Animals | mono_intronic | H/ACA | ENSDARG00000054818 | rpl32 | protein_coding |
| ENSDARG00000082440 | SNORD31 | D_rerio | Animals | mono_intronic | C/D | ENSDARG00000103682 | si:dkey-23i12.5 | non_coding |
| ENSDARG00000082493 | ENSDARG00000082493 | D_rerio | Animals | mono_intronic | H/ACA | ENSDARG00000032175 | puf60a | protein_coding |
| ENSDARG00000082511 | SNORD18 | D_rerio | Animals | mono_intronic | C/D | ENSDARG00000041182 | rpl4 | protein_coding |
| ENSDARG00000082607 | SNORD21 | D_rerio | Animals | mono_intronic | C/D | ENSDARG00000015862 | rpl5b | protein_coding |
| ENSDARG00000082611 | SNORA23 | D_rerio | Animals | mono_intronic | H/ACA | ENSDARG00000035751 | ipo7 | protein_coding |
| ENSDARG00000082639 | ENSDARG00000082639 | D_rerio | Animals | mono_intronic | C/D | ENSDARG00000016477 | eif4a2 | protein_coding |
| ENSDARG00000082652 | ENSDARG00000082652 | D_rerio | Animals | mono_intronic | C/D | ENSDARG00000014244 | rbmx | protein_coding |
| ENSDARG00000082739 | SNORD36 | D_rerio | Animals | mono_intronic | C/D | ENSDARG00000019230 | rpl7a | protein_coding |
| ENSDARG00000082811 | SNORD58 | D_rerio | Animals | mono_intronic | C/D | ENSDARG00000092693 | tpt1 | protein_coding |
| ENSDARG00000082833 | ENSDARG00000082833 | D_rerio | Animals | mono_intronic | C/D | ENSDARG00000005513 | naca | protein_coding |
| ENSDARG00000082850 | U8 | D_rerio | Animals | mono_intergenic | C/D | | | intergenic |
| ENSDARG00000082891 | SNORD42 | D_rerio | Animals | mono_intronic | C/D | ENSDARG00000006316 | rpl23a | protein_coding |
| ENSDARG00000082911 | SNORD18 | D_rerio | Animals | mono_intronic | C/D | ENSDARG00000041182 | rpl4 | protein_coding |
| ENSDARG00000082930 | SNORA74 | D_rerio | Animals | mono_intronic | H/ACA | ENSDARG00000018698 | carm1 | protein_coding |
| ENSDARG00000082960 | SNORD21 | D_rerio | Animals | mono_intronic | C/D | ENSDARG00000015862 | rpl5b | protein_coding |
| ENSDARG00000083056 | ENSDARG00000083056 | D_rerio | Animals | mono_intronic | C/D | ENSDARG00000016477 | eif4a2 | protein_coding |
| ENSDARG00000083089 | SNORA36B | D_rerio | Animals | mono_intronic | H/ACA | ENSDARG00000016484 | dkc1 | protein_coding |
| ENSDARG00000083108 | SNORA73 | D_rerio | Animals | mono_intronic | H/ACA | ENSDARG00000042566 | rps7 | protein_coding |
| ENSDARG00000083150 | SNORD83 | D_rerio | Animals | mono_intronic | C/D | ENSDARG00000016484 | dkc1 | protein_coding |
| ENSDARG00000083158 | SNORD54 | D_rerio | Animals | mono_intronic | C/D | ENSDARG00000036044 | rps20 | non_coding |
| ENSDARG00000083171 | ENSDARG00000083171 | D_rerio | Animals | mono_intronic | C/D | ENSDARG00000014244 | rbmx | protein_coding |
| ENSDARG00000083254 | SNORD79 | D_rerio | Animals | mono_intronic | C/D | ENSDARG00000092337 | | non_coding |
| ENSDARG00000083256 | ENSDARG00000083256 | D_rerio | Animals | mono_intronic | C/D | ENSDARG00000020711 | rrm2 | protein_coding |
| ENSDARG00000083266 | ENSDARG00000083266 | D_rerio | Animals | mono_intronic | C/D | ENSDARG00000097452 | | non_coding |
| ENSDARG00000083268 | ENSDARG00000083268 | D_rerio | Animals | mono_intronic | C/D | ENSDARG00000103553 | sf3b3 | protein_coding |
| ENSDARG00000083325 | ENSDARG00000083325 | D_rerio | Animals | mono_intronic | C/D | ENSDARG00000005058 | ncapd2 | protein_coding |
| ENSDARG00000083378 | SNORA63 | D_rerio | Animals | mono_intronic | H/ACA | ENSDARG00000051783 | rplp0 | protein_coding |
| ENSDARG00000083389 | ENSDARG00000083389 | D_rerio | Animals | mono_intronic | H/ACA | ENSDARG00000097815 | | non_coding |
| ENSDARG00000083400 | SNORA73 | D_rerio | Animals | mono_intronic | H/ACA | ENSDARG00000042566 | rps7 | protein_coding |
| ENSDARG00000083424 | ENSDARG00000083424 | D_rerio | Animals | mono_intergenic | H/ACA | | | intergenic |
| ENSDARG00000083434 | ENSDARG00000083434 | D_rerio | Animals | mono_intronic | C/D | ENSDARG00000026842 | acin1b | protein_coding |
| ENSDARG00000083457 | ENSDARG00000083457 | D_rerio | Animals | mono_intronic | C/D | ENSDARG00000092337 | | non_coding |
| ENSDARG00000083576 | ENSDARG00000083576 | D_rerio | Animals | mono_intronic | H/ACA | ENSDARG00000019299 | znf687b | protein_coding |
| ENSDARG00000083582 | SNORA75 | D_rerio | Animals | mono_intronic | H/ACA | ENSDARG00000002710 | ncl | protein_coding |
| ENSDARG00000083593 | ENSDARG00000083593 | D_rerio | Animals | mono_intronic | H/ACA | ENSDARG00000003599 | rpl3 | protein_coding |

| | | | | | | | | |
|---|---|---|---|---|---|---|---|---|
| ENSDARG00000083699 | ENSDARG00000083699 | D_rerio | Animals | mono_intronic | H/ACA | ENSDARG00000016477 | eif4a2 | protein_coding |
| ENSDARG00000083784 | SNORD22 | D_rerio | Animals | mono_intronic | C/D | ENSDARG00000103682 | si:dkey-23i12.5 | non_coding |
| ENSDARG00000083826 | ENSDARG00000083826 | D_rerio | Animals | mono_intronic | H/ACA | ENSDARG00000016484 | dkc1 | protein_coding |
| ENSDARG00000083842 | ENSDARG00000083842 | D_rerio | Animals | mono_intronic | H/ACA | ENSDARG00000043304 | nop2 | protein_coding |
| ENSDARG00000083885 | ENSDARG00000083885 | D_rerio | Animals | mono_intronic | H/ACA | ENSDARG00000040666 | nifk | protein_coding |
| ENSDARG00000083894 | ENSDARG00000083894 | D_rerio | Animals | mono_intronic | H/ACA | ENSDARG00000006691 | rpl12 | protein_coding |
| ENSDARG00000083937 | ENSDARG00000083937 | D_rerio | Animals | mono_intronic | H/ACA | ENSDARG00000006691 | rpl12 | protein_coding |
| ENSDARG00000083950 | ENSDARG00000083950 | D_rerio | Animals | mono_intronic | C/D | ENSDARG00000003599 | rpl3 | protein_coding |
| ENSDARG00000083986 | SNORA81 | D_rerio | Animals | mono_intronic | H/ACA | ENSDARG00000016477 | eif4a2 | protein_coding |
| ENSDARG00000084004 | ENSDARG00000084004 | D_rerio | Animals | mono_intronic | H/ACA | ENSDARG00000104609 | CREBBP | protein_coding |
| ENSDARG00000084034 | ENSDARG00000084034 | D_rerio | Animals | mono_intronic | H/ACA | ENSDARG00000055868 | rsl1d1 | protein_coding |
| ENSDARG00000084148 | SNORD63 | D_rerio | Animals | mono_intergenic | C/D | | | intergenic |
| ENSDARG00000084209 | ENSDARG00000084209 | D_rerio | Animals | mono_intronic | H/ACA | ENSDARG00000057010 | | protein_coding |
| ENSDARG00000084411 | ENSDARG00000084411 | D_rerio | Animals | mono_intronic | C/D | ENSDARG00000006200 | eif4g1a | protein_coding |
| ENSDARG00000084449 | ENSDARG00000084449 | D_rerio | Animals | mono_intronic | H/ACA | ENSDARG00000043304 | nop2 | protein_coding |
| ENSDARG00000084532 | ENSDARG00000084532 | D_rerio | Animals | mono_intronic | H/ACA | ENSDARG00000092693 | tpt1 | protein_coding |
| ENSDARG00000084543 | ENSDARG00000084543 | D_rerio | Animals | mono_intronic | H/ACA | ENSDARG00000036500 | pabir2 | protein_coding |
| ENSDARG00000084548 | ENSDARG00000084548 | D_rerio | Animals | mono_intronic | H/ACA | ENSDARG00000036500 | pabir2 | protein_coding |
| ENSDARG00000084628 | ENSDARG00000084628 | D_rerio | Animals | intergenic_cluster | H/ACA | | | intergenic |
| ENSDARG00000084670 | ENSDARG00000084670 | D_rerio | Animals | mono_intronic | H/ACA | ENSDARG00000055868 | rsl1d1 | protein_coding |
| ENSDARG00000084828 | ENSDARG00000084828 | D_rerio | Animals | mono_intronic | H/ACA | ENSDARG00000025073 | rpl18a | protein_coding |
| ENSDARG00000084991 | SNORA62 | D_rerio | Animals | mono_intergenic | H/ACA | | | intergenic |
| ENSDARG00000084995 | ENSDARG00000084995 | D_rerio | Animals | mono_intronic | H/ACA | ENSDARG00000094097 | | non_coding |
| ENSDARG00000085291 | ENSDARG00000085291 | D_rerio | Animals | mono_intronic | C/D | ENSDARG00000104353 | nop58 | protein_coding |
| ENSDARG00000085301 | ENSDARG00000085301 | D_rerio | Animals | mono_intronic | H/ACA | ENSDARG00000092693 | tpt1 | protein_coding |
| ENSDARG00000085358 | SNORD46 | D_rerio | Animals | mono_intronic | C/D | ENSDARG00000055996 | rps8a | protein_coding |
| ENSDARG00000085485 | ENSDARG00000085485 | D_rerio | Animals | intergenic_cluster | H/ACA | | | intergenic |
| ENSDARG00000085557 | ENSDARG00000085557 | D_rerio | Animals | mono_intronic | C/D | ENSDARG00000104353 | nop58 | protein_coding |
| ENSDARG00000085594 | ENSDARG00000085594 | D_rerio | Animals | mono_intronic | C/D | ENSDARG00000042065 | eef2a.2 | protein_coding |
| ENSDARG00000085696 | ENSDARG00000085696 | D_rerio | Animals | mono_intronic | C/D | ENSDARG00000014244 | rbmx | protein_coding |
| ENSDARG00000085713 | ENSDARG00000085713 | D_rerio | Animals | mono_intronic | H/ACA | ENSDARG00000006691 | rpl12 | protein_coding |
| ENSDARG00000085781 | ENSDARG00000085781 | D_rerio | Animals | mono_intergenic | C/D | | | intergenic |
| ENSDARG00000085829 | ENSDARG00000085829 | D_rerio | Animals | mono_intronic | H/ACA | ENSDARG00000032175 | puf60a | protein_coding |
| ENSDARG00000086007 | SNORA77B | D_rerio | Animals | mono_intronic | H/ACA | ENSDARG00000014817 | ranbp1 | protein_coding |
| ENSDARG00000086733 | ENSDARG00000086733 | D_rerio | Animals | mono_intronic | C/D | ENSDARG00000012820 | nop56 | protein_coding |
| ENSDARG00000087030 | ENSDARG00000087030 | D_rerio | Animals | mono_intronic | C/D | ENSDARG00000092337 | | non_coding |
| ENSDARG00000089164 | ENSDARG00000089164 | D_rerio | Animals | mono_intronic | C/D | ENSDARG00000043873 | arid4a | protein_coding |
| ENSDARG00000098419 | SNORD15 | D_rerio | Animals | mono_intronic | C/D | ENSDARG00000103007 | rps3 | protein_coding |
| ENSDARG00000098534 | SNORD15 | D_rerio | Animals | mono_intronic | C/D | ENSDARG00000103007 | rps3 | protein_coding |
| ENSDARG00000098998 | ENSDARG00000098998 | D_rerio | Animals | mono_intergenic | Unknown | | | intergenic |
| ENSDARG00000099125 | ENSDARG00000099125 | D_rerio | Animals | intergenic_cluster | C/D | | | intergenic |
| ENSDARG00000099827 | ENSDARG00000099827 | D_rerio | Animals | mono_intergenic | C/D | | | intergenic |
| ENSDARG00000100318 | ENSDARG00000100318 | D_rerio | Animals | mono_intronic | C/D | ENSDARG00000099380 | rpl13 | protein_coding |
| ENSDARG00000100696 | ENSDARG00000100696 | D_rerio | Animals | mono_intergenic | C/D | | | intergenic |
| ENSDARG00000100745 | ENSDARG00000100745 | D_rerio | Animals | mono_intronic | H/ACA | ENSDARG00000102624 | kif22 | protein_coding |

| | | | | | | | | |
|---|---|---|---|---|---|---|---|---|
| ENSDARG00000101659 | ENSDARG00000101659 | D_rerio | Animals | mono_intergenic | H/ACA | | | intergenic |
| ENSDARG00000102120 | SNORA74 | D_rerio | Animals | mono_intronic | H/ACA | ENSDARG00000100366 | si:dkey-102m7.3 | protein_coding |
| ENSDARG00000102909 | ENSDARG00000102909 | D_rerio | Animals | mono_intronic | C/D | ENSDARG00000099380 | rpl13 | protein_coding |
| ENSDARG00000103384 | ENSDARG00000103384 | D_rerio | Animals | intergenic_cluster | H/ACA | | | intergenic |
| ENSDARG00000103728 | U3 | D_rerio | Animals | intergenic_cluster | C/D | | | intergenic |
| ENSDARG00000104104 | SNORD68 | D_rerio | Animals | mono_intronic | C/D | ENSDARG00000099380 | rpl13 | protein_coding |
| ENSDARG00000104420 | ENSDARG00000104420 | D_rerio | Animals | mono_intronic | H/ACA | ENSDARG00000104889 | kpnb1 | protein_coding |
| ENSDARG00000104662 | ENSDARG00000104662 | D_rerio | Animals | mono_intronic | H/ACA | ENSDARG00000102624 | kif22 | protein_coding |
| ENSDARG00000104878 | ENSDARG00000104878 | D_rerio | Animals | mono_intergenic | C/D | | | intergenic |
| ENSDARG00000105016 | ENSDARG00000105016 | D_rerio | Animals | mono_intergenic | C/D | | | intergenic |
| ENSDARG00000105055 | ENSDARG00000105055 | D_rerio | Animals | mono_intergenic | C/D | | | intergenic |
| ENSDARG00000106293 | SNORD38A | D_rerio | Animals | mono_intronic | C/D | ENSDARG00000055996 | rps8a | protein_coding |
| ENSDARG00000107036 | SNORD82 | D_rerio | Animals | mono_intronic | C/D | ENSDARG00000002710 | ncl | protein_coding |
| ENSDARG00000109264 | SNORD24 | D_rerio | Animals | mono_intronic | C/D | ENSDARG00000019230 | rpl7a | protein_coding |
| ENSDARG00000109270 | U3 | D_rerio | Animals | mono_intergenic | C/D | | | intergenic |
| ENSDARG00000109375 | ENSDARG00000109375 | D_rerio | Animals | intergenic_cluster | C/D | | | intergenic |
| ENSDARG00000109437 | ENSDARG00000109437 | D_rerio | Animals | mono_intronic | H/ACA | ENSDARG00000025566 | slc25a3b | protein_coding |
| ENSDARG00000109514 | U8 | D_rerio | Animals | mono_intergenic | C/D | | | intergenic |
| ENSDARG00000109533 | U3 | D_rerio | Animals | mono_intergenic | C/D | | | intergenic |
| ENSDARG00000109557 | ENSDARG00000109557 | D_rerio | Animals | intronic_cluster | C/D | ENSDARG00000092337 | | non_coding |
| ENSDARG00000109652 | ENSDARG00000109652 | D_rerio | Animals | mono_intergenic | C/D | | | intergenic |
| ENSDARG00000109750 | U3 | D_rerio | Animals | intergenic_cluster | C/D | | | intergenic |
| ENSDARG00000109762 | ENSDARG00000109762 | D_rerio | Animals | intergenic_cluster | C/D | | | intergenic |
| ENSDARG00000109897 | ENSDARG00000109897 | D_rerio | Animals | mono_intronic | C/D | ENSDARG00000103553 | sf3b3 | protein_coding |
| ENSDARG00000109928 | U3 | D_rerio | Animals | mono_intergenic | C/D | | | intergenic |
| ENSDARG00000109938 | U3 | D_rerio | Animals | mono_intergenic | C/D | | | intergenic |
| ENSDARG00000109939 | U3 | D_rerio | Animals | mono_intergenic | C/D | | | intergenic |
| ENSDARG00000110189 | ENSDARG00000110189 | D_rerio | Animals | intronic_cluster | H/ACA | ENSDARG00000105273 | | non_coding |
| ENSDARG00000110201 | U3 | D_rerio | Animals | intergenic_cluster | C/D | | | intergenic |
| ENSDARG00000110554 | SNORD83 | D_rerio | Animals | mono_intronic | C/D | ENSDARG00000016484 | dkc1 | protein_coding |
| ENSDARG00000110604 | ENSDARG00000110604 | D_rerio | Animals | intergenic_cluster | C/D | | | intergenic |
| ENSDARG00000110617 | SNORD16 | D_rerio | Animals | mono_intronic | C/D | ENSDARG00000041182 | rpl4 | protein_coding |
| ENSDARG00000110758 | SNORD16 | D_rerio | Animals | mono_intronic | C/D | ENSDARG00000041182 | rpl4 | protein_coding |
| ENSDARG00000110897 | ENSDARG00000110897 | D_rerio | Animals | intergenic_cluster | C/D | | | intergenic |
| ENSDARG00000110937 | U3 | D_rerio | Animals | mono_intergenic | C/D | | | intergenic |
| ENSDARG00000111254 | U3 | D_rerio | Animals | intergenic_cluster | C/D | | | intergenic |
| ENSDARG00000111498 | U3 | D_rerio | Animals | mono_intergenic | C/D | | | intergenic |
| ENSDARG00000111557 | SNORD15 | D_rerio | Animals | mono_intronic | C/D | ENSDARG00000103007 | rps3 | protein_coding |
| ENSDARG00000111586 | ENSDARG00000111586 | D_rerio | Animals | mono_intronic | C/D | ENSDARG00000095427 | | non_coding |
| ENSDARG00000111622 | U3 | D_rerio | Animals | intergenic_cluster | C/D | | | intergenic |
| ENSDARG00000111793 | SNORD83 | D_rerio | Animals | mono_intronic | C/D | ENSDARG00000016484 | dkc1 | protein_coding |
| ENSDARG00000111813 | ENSDARG00000111813 | D_rerio | Animals | intergenic_cluster | C/D | | | intergenic |
| ENSDARG00000112042 | U3 | D_rerio | Animals | intergenic_cluster | C/D | | | intergenic |
| ENSDARG00000112208 | ENSDARG00000112208 | D_rerio | Animals | mono_intronic | H/ACA | ENSDARG00000105273 | | non_coding |
| ENSDARG00000112219 | ENSDARG00000112219 | D_rerio | Animals | intergenic_cluster | C/D | | | intergenic |

| | | | | | | | | |
|---|---|---|---|---|---|---|---|---|
| ENSDARG00000112530 | ENSDARG00000112530 | D_rerio | Animals | mono_intronic | H/ACA | ENSDARG00000099256 | pum1 | protein_coding |
| ENSDARG00000112553 | ENSDARG00000112553 | D_rerio | Animals | intergenic_cluster | C/D | | | intergenic |
| ENSDARG00000112580 | ENSDARG00000112580 | D_rerio | Animals | intronic_cluster | C/D | ENSDARG00000092337 | | non_coding |
| ENSDARG00000112804 | U3 | D_rerio | Animals | mono_intergenic | C/D | | | intergenic |
| ENSDARG00000112988 | ENSDARG00000112988 | D_rerio | Animals | mono_intronic | C/D | ENSDARG00000043873 | arid4a | protein_coding |
| ENSDARG00000113056 | SNORD14 | D_rerio | Animals | mono_intronic | C/D | ENSDARG00000068992 | hspa8 | protein_coding |
| ENSDARG00000113149 | U3 | D_rerio | Animals | mono_intergenic | C/D | | | intergenic |
| ENSDARG00000113221 | U3 | D_rerio | Animals | intergenic_cluster | C/D | | | intergenic |
| ENSDARG00000113231 | ENSDARG00000113231 | D_rerio | Animals | mono_intronic | H/ACA | ENSDARG00000102624 | kif22 | protein_coding |
| ENSDARG00000113271 | ENSDARG00000113271 | D_rerio | Animals | mono_intergenic | Unknown | | | intergenic |
| ENSDARG00000113344 | ENSDARG00000113344 | D_rerio | Animals | intergenic_cluster | C/D | | | intergenic |
| ENSDARG00000113464 | ENSDARG00000113464 | D_rerio | Animals | intronic_cluster | H/ACA | ENSDARG00000105273 | | non_coding |
| ENSDARG00000113571 | SNORA62 | D_rerio | Animals | mono_intronic | H/ACA | ENSDARG00000019181 | rpsa | protein_coding |
| ENSDARG00000113630 | ENSDARG00000113630 | D_rerio | Animals | mono_intronic | H/ACA | ENSDARG00000021140 | pabpc1b | protein_coding |
| ENSDARG00000113731 | ENSDARG00000113731 | D_rerio | Animals | intergenic_cluster | C/D | | | intergenic |
| ENSDARG00000113745 | U3 | D_rerio | Animals | mono_intergenic | C/D | | | intergenic |
| ENSDARG00000113840 | U3 | D_rerio | Animals | mono_intergenic | C/D | | | intergenic |
| ENSDARG00000113849 | U3 | D_rerio | Animals | intergenic_cluster | C/D | | | intergenic |
| ENSDARG00000113885 | SNORD15 | D_rerio | Animals | mono_intronic | C/D | ENSDARG00000103007 | rps3 | protein_coding |
| ENSDARG00000113902 | ENSDARG00000113902 | D_rerio | Animals | intergenic_cluster | C/D | | | intergenic |
| ENSDARG00000114361 | U3 | D_rerio | Animals | mono_intergenic | C/D | | | intergenic |
| ENSDARG00000114381 | ENSDARG00000114381 | D_rerio | Animals | mono_intronic | C/D | ENSDARG00000043873 | arid4a | protein_coding |
| ENSDARG00000114453 | ENSDARG00000114453 | D_rerio | Animals | mono_intronic | H/ACA | ENSDARG00000102624 | kif22 | protein_coding |
| ENSDARG00000114688 | ENSDARG00000114688 | D_rerio | Animals | intergenic_cluster | C/D | | | intergenic |
| ENSDARG00000114781 | ENSDARG00000114781 | D_rerio | Animals | mono_intronic | H/ACA | ENSDARG00000021140 | pabpc1b | protein_coding |
| ENSDARG00000114807 | U3 | D_rerio | Animals | intergenic_cluster | C/D | | | intergenic |
| ENSDARG00000115047 | SNORD14 | D_rerio | Animals | mono_intronic | C/D | ENSDARG00000068992 | hspa8 | protein_coding |
| ENSDARG00000115102 | ENSDARG00000115102 | D_rerio | Animals | mono_intergenic | C/D | | | intergenic |
| ENSDARG00000115154 | ENSDARG00000115154 | D_rerio | Animals | mono_exonic | C/D | ENSDARG00000095427 | | non_coding |
| ENSDARG00000115262 | SNORA63 | D_rerio | Animals | mono_intronic | H/ACA | ENSDARG00000051783 | rplp0 | protein_coding |
| ENSDARG00000115288 | ENSDARG00000115288 | D_rerio | Animals | mono_intergenic | C/D | | | intergenic |
| ENSDARG00000115429 | ENSDARG00000115429 | D_rerio | Animals | mono_intronic | H/ACA | ENSDARG00000099256 | pum1 | protein_coding |
| ENSDARG00000115494 | ENSDARG00000115494 | D_rerio | Animals | mono_intronic | C/D | ENSDARG00000053058 | rps11 | protein_coding |
| ENSDARG00000115495 | U3 | D_rerio | Animals | intergenic_cluster | C/D | | | intergenic |
| ENSDARG00000115569 | U3 | D_rerio | Animals | intergenic_cluster | C/D | | | intergenic |
| ENSDARG00000115663 | U8 | D_rerio | Animals | mono_intergenic | C/D | | | intergenic |
| ENSDARG00000115704 | U3 | D_rerio | Animals | intergenic_cluster | C/D | | | intergenic |
| ENSDARG00000115852 | ENSDARG00000115852 | D_rerio | Animals | mono_intronic | H/ACA | ENSDARG00000025566 | slc25a3b | protein_coding |
| ENSDARG00000115970 | SNORD83 | D_rerio | Animals | mono_intronic | C/D | ENSDARG00000016484 | dkc1 | protein_coding |
| ENSDARG00000116097 | ENSDARG00000116097 | D_rerio | Animals | mono_intronic | C/D | ENSDARG00000095427 | | non_coding |
| ENSDARG00000116147 | U3 | D_rerio | Animals | mono_intergenic | C/D | | | intergenic |
| ENSDARG00000116159 | ENSDARG00000116159 | D_rerio | Animals | intergenic_cluster | C/D | | | intergenic |
| ENSDARG00000116175 | ENSDARG00000116175 | D_rerio | Animals | mono_intronic | C/D | ENSDARG00000095427 | | non_coding |
| ENSDARG00000116248 | SNORD24 | D_rerio | Animals | mono_intronic | C/D | ENSDARG00000019230 | rpl7a | protein_coding |
| ENSDARG00000116406 | ENSDARG00000116406 | D_rerio | Animals | intergenic_cluster | C/D | | | intergenic |

| | | | | | | | | |
|---|---|---|---|---|---|---|---|---|
| ENSDARG00000116415 | U3 | D_rerio | Animals | intergenic_cluster | C/D | | | intergenic |
| ENSDARG00000116506 | U3 | D_rerio | Animals | intergenic_cluster | C/D | | | intergenic |
| ENSDARG00000116621 | ENSDARG00000116621 | D_rerio | Animals | mono_intronic | H/ACA | ENSDARG00000053457 | rpl23 | protein_coding |
| ENSDARG00000116745 | SNORA62 | D_rerio | Animals | mono_intronic | H/ACA | ENSDARG00000019181 | rpsa | protein_coding |
| ENSDARG00000116747 | U3 | D_rerio | Animals | mono_intergenic | C/D | | | intergenic |
| ENSDARG00000116829 | ENSDARG00000116829 | D_rerio | Animals | mono_intronic | C/D | ENSDARG00000092337 | | non_coding |
| ENSDARG00000116958 | U3 | D_rerio | Animals | mono_intergenic | C/D | | | intergenic |
| ENSDARG00000117016 | ENSDARG00000117016 | D_rerio | Animals | mono_intronic | C/D | ENSDARG00000053058 | rps11 | protein_coding |
| ENSDARG00000117034 | ENSDARG00000117034 | D_rerio | Animals | mono_intronic | H/ACA | ENSDARG00000103163 | tomm20a | protein_coding |
| ENSDARG00000117085 | SNORA70 | D_rerio | Animals | mono_intronic | H/ACA | ENSDARG00000025581 | rpl10 | protein_coding |
| ENSDARG00000117129 | ENSDARG00000117129 | D_rerio | Animals | mono_intergenic | C/D | | | intergenic |
| FBgn0015543 | snoRNA:Me28S-A1322 | D_melanogaster | Animals | mono_intronic | C/D | FBgn0284245 | eEF1alpha1 | protein_coding |
| FBgn0020518 | snoRNA:Me28S-G764 | D_melanogaster | Animals | mono_intronic | C/D | FBgn0010348 | Arf1 | protein_coding |
| FBgn0025881 | snoRNA:Me18S-A1576 | D_melanogaster | Animals | mono_intronic | C/D | FBgn0063449 | Uhg2 | non_coding |
| FBgn0025882 | snoRNA:MeU6-A47 | D_melanogaster | Animals | mono_intronic | C/D | FBgn0001565 | Hlc | protein_coding |
| FBgn0026169 | snoRNA:Psi18S-1820 | D_melanogaster | Animals | mono_intronic | H/ACA | FBgn0259937 | Nop60B | protein_coding |
| FBgn0043853 | scaRNA:MeU5-C46 | D_melanogaster | Animals | mono_intronic | C/D | FBgn0037504 | CG1142 | protein_coding |
| FBgn0044508 | snoRNA:M | D_melanogaster | Animals | intronic_cluster | H/ACA | FBgn0003517 | sta | protein_coding |
| FBgn0060291 | snoRNA:U49:66Db | D_melanogaster | Animals | mono_intronic | Unknown | FBgn0017579 | RpL14 | protein_coding |
| FBgn0060292 | snoRNA:U49:66Da | D_melanogaster | Animals | mono_intronic | Unknown | FBgn0017579 | RpL14 | protein_coding |
| FBgn0063373 | snoRNA:snR38:54Ec | D_melanogaster | Animals | mono_intronic | Unknown | FBgn0045800 | Uhg1 | non_coding |
| FBgn0063374 | snoRNA:snR38:54Eb | D_melanogaster | Animals | mono_intronic | Unknown | FBgn0045800 | Uhg1 | non_coding |
| FBgn0063375 | snoRNA:Me28S-G3277a | D_melanogaster | Animals | mono_intronic | C/D | FBgn0045800 | Uhg1 | non_coding |
| FBgn0063376 | snoRNA:U76:54Eb | D_melanogaster | Animals | mono_intronic | Unknown | FBgn0045800 | Uhg1 | non_coding |
| FBgn0063377 | snoRNA:Me28S-A1666a | D_melanogaster | Animals | mono_intronic | C/D | FBgn0045800 | Uhg1 | non_coding |
| FBgn0063378 | snoRNA:U31:54Ed | D_melanogaster | Animals | mono_intronic | C/D | FBgn0045800 | Uhg1 | non_coding |
| FBgn0063379 | snoRNA:U31:54Ec | D_melanogaster | Animals | mono_intronic | C/D | FBgn0045800 | Uhg1 | non_coding |
| FBgn0063380 | snoRNA:U31:54Eb | D_melanogaster | Animals | mono_intronic | C/D | FBgn0045800 | Uhg1 | non_coding |
| FBgn0063381 | snoRNA:Me28S-G3081a | D_melanogaster | Animals | mono_intronic | C/D | FBgn0045800 | Uhg1 | non_coding |
| FBgn0063382 | snoRNA:U29:54Ed | D_melanogaster | Animals | mono_intronic | Unknown | FBgn0045800 | Uhg1 | non_coding |
| FBgn0063383 | snoRNA:U29:54Ec | D_melanogaster | Animals | mono_intronic | Unknown | FBgn0045800 | Uhg1 | non_coding |
| FBgn0063384 | snoRNA:U29:54Eb | D_melanogaster | Animals | mono_intronic | Unknown | FBgn0045800 | Uhg1 | non_coding |
| FBgn0063385 | snoRNA:Me28S-A3407a | D_melanogaster | Animals | mono_intronic | C/D | FBgn0045800 | Uhg1 | non_coding |
| FBgn0063386 | snoRNA:Me18S-A28a | D_melanogaster | Animals | mono_intronic | C/D | FBgn0045800 | Uhg1 | non_coding |
| FBgn0063387 | snoRNA:U27:54Eb | D_melanogaster | Animals | mono_intronic | Unknown | FBgn0045800 | Uhg1 | non_coding |
| FBgn0063388 | snoRNA:U27:54Ea | D_melanogaster | Animals | mono_intronic | Unknown | FBgn0045800 | Uhg1 | non_coding |
| FBgn0063389 | snoRNA:Me18S-G1620 | D_melanogaster | Animals | mono_intronic | C/D | FBgn0063449 | Uhg2 | non_coding |
| FBgn0063391 | snoRNA:U14:30Eb | D_melanogaster | Animals | mono_intronic | Unknown | FBgn0063449 | Uhg2 | non_coding |
| FBgn0063392 | snoRNA:U14:30Ea | D_melanogaster | Animals | mono_intronic | Unknown | FBgn0063449 | Uhg2 | non_coding |
| FBgn0065046 | snoRNA:U3:9B | D_melanogaster | Animals | mono_intergenic | C/D | | | intergenic |
| FBgn0065047 | snoRNA:U3:54Ab | D_melanogaster | Animals | mono_intergenic | C/D | | | intergenic |
| FBgn0065048 | snoRNA:U3:54Aa | D_melanogaster | Animals | mono_intergenic | C/D | | | intergenic |
| FBgn0065051 | scaRNA:MeU5-U42 | D_melanogaster | Animals | mono_intronic | C/D | FBgn0036483 | CG12316 | protein_coding |
| FBgn0065053 | snoRNA:Me18S-C1831 | D_melanogaster | Animals | mono_intronic | C/D | FBgn0285948 | RpL27A | protein_coding |
| FBgn0065055 | snoRNA:Psi28S-2648 | D_melanogaster | Animals | mono_intronic | H/ACA | FBgn0031980 | RpL36A | protein_coding |

| | | | | | | | | |
|---|---|---|---|---|---|---|---|---|
| FBgn0065057 | scaRNA:MeU2-C28 | D_melanogaster | Animals | mono_intronic | C/D | FBgn0031149 | Stt3A | protein_coding |
| FBgn0065058 | snoRNA:684 | D_melanogaster | Animals | mono_intronic | C/D | FBgn0035630 | CG10576 | protein_coding |
| FBgn0065059 | snoRNA:660 | D_melanogaster | Animals | mono_exonic | H/ACA | FBgn0000044 | Act57B | protein_coding |
| FBgn0065064 | snoRNA:Psi28S-291 | D_melanogaster | Animals | mono_intronic | H/ACA | FBgn0020910 | RpL3 | protein_coding |
| FBgn0065066 | snoRNA:Me28S-A2564 | D_melanogaster | Animals | mono_intronic | C/D | FBgn0039713 | RpS8 | protein_coding |
| FBgn0065070 | snoRNA:291 | D_melanogaster | Animals | mono_intronic | C/D | FBgn0039600 | Prp39 | protein_coding |
| FBgn0065071 | snoRNA:Or-CD10 | D_melanogaster | Animals | mono_intronic | C/D | FBgn0014026 | RpL7A | protein_coding |
| FBgn0065073 | snoRNA:229 | D_melanogaster | Animals | mono_intronic | C/D | FBgn0003062 | Fib | protein_coding |
| FBgn0065076 | snoRNA:185 | D_melanogaster | Animals | mono_intergenic | C/D | | | intergenic |
| FBgn0065080 | snoRNA:Me18S-A1374 | D_melanogaster | Animals | mono_intronic | C/D | FBgn0260003 | Dys | protein_coding |
| FBgn0082919 | snoRNA:Or-CD4 | D_melanogaster | Animals | mono_intronic | C/D | FBgn0083123 | Uhg5 | non_coding |
| FBgn0082922 | snoRNA:Or-aca4 | D_melanogaster | Animals | mono_intergenic | H/ACA | | | intergenic |
| FBgn0082923 | snoRNA:Or-aca3 | D_melanogaster | Animals | mono_intronic | H/ACA | FBgn0003279 | RpL4 | protein_coding |
| FBgn0082924 | snoRNA:Or-aca2 | D_melanogaster | Animals | mono_intronic | H/ACA | FBgn0261592 | RpS6 | protein_coding |
| FBgn0082925 | snoRNA:Or-aca1 | D_melanogaster | Animals | intronic_cluster | H/ACA | FBgn0034743 | RpS16 | protein_coding |
| FBgn0082926 | snoRNA:Me28S-U1848 | D_melanogaster | Animals | mono_intronic | C/D | FBgn0083123 | Uhg5 | non_coding |
| FBgn0082927 | snoRNA:Me28S-G3255b | D_melanogaster | Animals | mono_intronic | C/D | FBgn0083124 | Uhg4 | non_coding |
| FBgn0082928 | snoRNA:Me28S-G3255a | D_melanogaster | Animals | mono_intronic | C/D | FBgn0083124 | Uhg4 | non_coding |
| FBgn0082929 | snoRNA:Me28S-G2703c | D_melanogaster | Animals | mono_intronic | C/D | FBgn0083123 | Uhg5 | non_coding |
| FBgn0082930 | snoRNA:Me28S-G2703b | D_melanogaster | Animals | mono_intronic | C/D | FBgn0083123 | Uhg5 | non_coding |
| FBgn0082931 | snoRNA:Me28S-G2703a | D_melanogaster | Animals | mono_intronic | C/D | FBgn0083123 | Uhg5 | non_coding |
| FBgn0082932 | snoRNA:Me28S-G1083d | D_melanogaster | Animals | mono_intronic | C/D | FBgn0259937 | Nop60B | protein_coding |
| FBgn0082933 | snoRNA:Me28S-G1083c | D_melanogaster | Animals | mono_intronic | C/D | FBgn0259937 | Nop60B | protein_coding |
| FBgn0082934 | snoRNA:Me28S-G1083b | D_melanogaster | Animals | mono_intronic | C/D | FBgn0259937 | Nop60B | protein_coding |
| FBgn0082935 | snoRNA:Me28S-G1083a | D_melanogaster | Animals | mono_intronic | C/D | FBgn0259937 | Nop60B | protein_coding |
| FBgn0082936 | snoRNA:Me28S-C2645c | D_melanogaster | Animals | mono_intronic | C/D | FBgn0259936 | Uhg3 | non_coding |
| FBgn0082937 | snoRNA:Me28S-C2645b | D_melanogaster | Animals | mono_intronic | C/D | FBgn0259936 | Uhg3 | non_coding |
| FBgn0082938 | snoRNA:Me28S-C2645a | D_melanogaster | Animals | mono_intronic | C/D | FBgn0259936 | Uhg3 | non_coding |
| FBgn0082939 | snoRNA:Me28S-C3227b | D_melanogaster | Animals | mono_intronic | C/D | FBgn0083123 | Uhg5 | non_coding |
| FBgn0082940 | snoRNA:Me28S-C3227a | D_melanogaster | Animals | mono_intronic | C/D | FBgn0083123 | Uhg5 | non_coding |
| FBgn0082941 | snoRNA:Me28S-A992 | D_melanogaster | Animals | mono_intronic | C/D | FBgn0083123 | Uhg5 | non_coding |
| FBgn0082942 | snoRNA:Me28S-A982b | D_melanogaster | Animals | mono_intronic | C/D | FBgn0083124 | Uhg4 | non_coding |
| FBgn0082943 | snoRNA:Me28S-A982a | D_melanogaster | Animals | mono_intronic | C/D | FBgn0083124 | Uhg4 | non_coding |
| FBgn0082944 | snoRNA:Me28S-A2634c | D_melanogaster | Animals | mono_intronic | C/D | FBgn0026372 | RpL23A | protein_coding |
| FBgn0082945 | snoRNA:Me28S-A2634b | D_melanogaster | Animals | mono_intronic | C/D | FBgn0026372 | RpL23A | protein_coding |
| FBgn0082946 | snoRNA:Me28S-A2634a | D_melanogaster | Animals | mono_intronic | C/D | FBgn0026372 | RpL23A | protein_coding |
| FBgn0082947 | snoRNA:Me18S-U1356c | D_melanogaster | Animals | mono_intronic | C/D | FBgn0083120 | Uhg8 | non_coding |
| FBgn0082948 | snoRNA:Me18S-U1356b | D_melanogaster | Animals | mono_intronic | C/D | FBgn0083120 | Uhg8 | non_coding |
| FBgn0082949 | snoRNA:Me18S-U1356a | D_melanogaster | Animals | mono_intronic | C/D | FBgn0083120 | Uhg8 | non_coding |
| FBgn0082950 | snoRNA:Me18S-G1952 | D_melanogaster | Animals | mono_intronic | C/D | FBgn0083123 | Uhg5 | non_coding |
| FBgn0082951 | snoRNA:Me18S-C1366 | D_melanogaster | Animals | mono_intronic | C/D | FBgn0083123 | Uhg5 | non_coding |
| FBgn0082952 | snoRNA:Me18S-A1061 | D_melanogaster | Animals | mono_intronic | C/D | FBgn0083123 | Uhg5 | non_coding |
| FBgn0082953 | snoRNA:Psi28S-612 | D_melanogaster | Animals | mono_intronic | H/ACA | FBgn0005533 | RpS17 | protein_coding |
| FBgn0082954 | snoRNA:Psi28S-3571 | D_melanogaster | Animals | mono_intronic | H/ACA | FBgn0250753 | kra | protein_coding |
| FBgn0082957 | snoRNA:Psi28S-3405d | D_melanogaster | Animals | intronic_cluster | H/ACA | FBgn0001215 | Hrb98DE | protein_coding |

| | | | | | | | | |
|---|---|---|---|---|---|---|---|---|
| FBgn0082958 | snoRNA:Psi28S-3405c | D_melanogaster | Animals | intronic_cluster | H/ACA | FBgn0001215 | Hrb98DE | protein_coding |
| FBgn0082959 | snoRNA:Psi28S-3405b | D_melanogaster | Animals | intronic_cluster | H/ACA | FBgn0001215 | Hrb98DE | protein_coding |
| FBgn0082960 | snoRNA:Psi28S-3405a | D_melanogaster | Animals | intronic_cluster | H/ACA | FBgn0001215 | Hrb98DE | protein_coding |
| FBgn0082961 | snoRNA:Psi28S-3385b | D_melanogaster | Animals | intronic_cluster | H/ACA | FBgn0250753 | kra | protein_coding |
| FBgn0082962 | snoRNA:Psi28S-3385a | D_melanogaster | Animals | intronic_cluster | H/ACA | FBgn0250753 | kra | protein_coding |
| FBgn0082963 | snoRNA:Psi28S-3378 | D_melanogaster | Animals | mono_intronic | H/ACA | FBgn0020306 | dom | protein_coding |
| FBgn0082966 | snoRNA:Psi28S-3327b | D_melanogaster | Animals | intronic_cluster | H/ACA | FBgn0011284 | RpS4 | protein_coding |
| FBgn0082967 | snoRNA:Psi28S-3327a | D_melanogaster | Animals | intronic_cluster | H/ACA | FBgn0011284 | RpS4 | protein_coding |
| FBgn0082968 | snoRNA:Psi28S-3316e | D_melanogaster | Animals | intronic_cluster | H/ACA | FBgn0020306 | dom | protein_coding |
| FBgn0082969 | snoRNA:Psi28S-3316d | D_melanogaster | Animals | intronic_cluster | H/ACA | FBgn0020306 | dom | protein_coding |
| FBgn0082970 | snoRNA:Psi28S-3316c | D_melanogaster | Animals | intronic_cluster | H/ACA | FBgn0020306 | dom | protein_coding |
| FBgn0082971 | snoRNA:Psi28S-3316b | D_melanogaster | Animals | intronic_cluster | H/ACA | FBgn0020306 | dom | protein_coding |
| FBgn0082972 | snoRNA:Psi28S-3316a | D_melanogaster | Animals | intronic_cluster | H/ACA | FBgn0020306 | dom | protein_coding |
| FBgn0082973 | snoRNA:Psi28S-3308 | D_melanogaster | Animals | mono_intronic | H/ACA | FBgn0035162 | Sf3b3 | protein_coding |
| FBgn0082974 | snoRNA:Psi28S-3305c | D_melanogaster | Animals | mono_intronic | H/ACA | FBgn0001215 | Hrb98DE | protein_coding |
| FBgn0082975 | snoRNA:Psi28S-3305b | D_melanogaster | Animals | intronic_cluster | H/ACA | FBgn0001215 | Hrb98DE | protein_coding |
| FBgn0082976 | snoRNA:Psi28S-3305a | D_melanogaster | Animals | intronic_cluster | H/ACA | FBgn0001215 | Hrb98DE | protein_coding |
| FBgn0082978 | snoRNA:Psi28S-3091b | D_melanogaster | Animals | intronic_cluster | H/ACA | FBgn0250753 | kra | protein_coding |
| FBgn0082979 | snoRNA:Psi28S-3091a | D_melanogaster | Animals | intronic_cluster | H/ACA | FBgn0250753 | kra | protein_coding |
| FBgn0082980 | snoRNA:Psi28S-2996 | D_melanogaster | Animals | mono_intronic | H/ACA | FBgn0064225 | RpL5 | protein_coding |
| FBgn0082981 | snoRNA:Psi28S-2949 | D_melanogaster | Animals | mono_intronic | H/ACA | FBgn0083124 | Uhg4 | non_coding |
| FBgn0082983 | snoRNA:Psi28S-2626 | D_melanogaster | Animals | intronic_cluster | H/ACA | FBgn0039757 | RpS7 | protein_coding |
| FBgn0082986 | snoRNA:Psi28S-2562 | D_melanogaster | Animals | mono_intronic | H/ACA | FBgn0019936 | RpS20 | protein_coding |
| FBgn0082987 | snoRNA:Psi28S-2444 | D_melanogaster | Animals | mono_intronic | H/ACA | FBgn0013325 | RpL11 | protein_coding |
| FBgn0082988 | snoRNA:Psi28S-2442b | D_melanogaster | Animals | intronic_cluster | H/ACA | FBgn0250753 | kra | protein_coding |
| FBgn0082989 | snoRNA:Psi28S-2442a | D_melanogaster | Animals | intronic_cluster | H/ACA | FBgn0250753 | kra | protein_coding |
| FBgn0082990 | snoRNA:Psi28S-2263 | D_melanogaster | Animals | mono_intronic | H/ACA | FBgn0261836 | Msp300 | protein_coding |
| FBgn0082993 | snoRNA:Psi28S-1936 | D_melanogaster | Animals | mono_intronic | H/ACA | FBgn0033636 | tou | protein_coding |
| FBgn0082994 | snoRNA:Psi28S-1837c | D_melanogaster | Animals | intronic_cluster | H/ACA | FBgn0035630 | CG10576 | protein_coding |
| FBgn0082995 | snoRNA:Psi28S-1837b | D_melanogaster | Animals | intronic_cluster | H/ACA | FBgn0035630 | CG10576 | protein_coding |
| FBgn0082997 | snoRNA:Psi28S-1232 | D_melanogaster | Animals | mono_intronic | H/ACA | FBgn0029897 | RpL17 | protein_coding |
| FBgn0082998 | snoRNA:Psi28S-1192d | D_melanogaster | Animals | intronic_cluster | H/ACA | FBgn0002590 | RpS5a | protein_coding |
| FBgn0082999 | snoRNA:Psi28S-1192c | D_melanogaster | Animals | intronic_cluster | H/ACA | FBgn0002590 | RpS5a | protein_coding |
| FBgn0083000 | snoRNA:Psi28S-1192b | D_melanogaster | Animals | intronic_cluster | H/ACA | FBgn0002590 | RpS5a | protein_coding |
| FBgn0083001 | snoRNA:Psi28S-1192a | D_melanogaster | Animals | intronic_cluster | H/ACA | FBgn0002590 | RpS5a | protein_coding |
| FBgn0083003 | snoRNA:Psi28S-1175c | D_melanogaster | Animals | intronic_cluster | H/ACA | FBgn0034743 | RpS16 | protein_coding |
| FBgn0083004 | snoRNA:Psi28S-1175b | D_melanogaster | Animals | intronic_cluster | H/ACA | FBgn0034743 | RpS16 | protein_coding |
| FBgn0083005 | snoRNA:Psi28S-1175a | D_melanogaster | Animals | intronic_cluster | H/ACA | FBgn0034743 | RpS16 | protein_coding |
| FBgn0083006 | snoRNA:Psi28S-1153 | D_melanogaster | Animals | intergenic_cluster | H/ACA | | | intergenic |
| FBgn0083007 | snoRNA:Psi28S-1135f | D_melanogaster | Animals | intronic_cluster | H/ACA | FBgn0002590 | RpS5a | protein_coding |
| FBgn0083008 | snoRNA:Psi28S-1135e | D_melanogaster | Animals | intronic_cluster | H/ACA | FBgn0002590 | RpS5a | protein_coding |
| FBgn0083009 | snoRNA:Psi28S-1135d | D_melanogaster | Animals | intronic_cluster | H/ACA | FBgn0002590 | RpS5a | protein_coding |
| FBgn0083010 | snoRNA:Psi28S-1135c | D_melanogaster | Animals | intronic_cluster | H/ACA | FBgn0002590 | RpS5a | protein_coding |
| FBgn0083011 | snoRNA:Psi28S-1135b | D_melanogaster | Animals | intronic_cluster | H/ACA | FBgn0002590 | RpS5a | protein_coding |
| FBgn0083012 | snoRNA:Psi28S-1135a | D_melanogaster | Animals | intronic_cluster | H/ACA | FBgn0002590 | RpS5a | protein_coding |

| | | | | | | | | |
|---|---|---|---|---|---|---|---|---|
| FBgn0083013 | snoRNA:Psi28S-1060 | D_melanogaster | Animals | intronic_cluster | H/ACA | FBgn0029897 | RpL17 | protein_coding |
| FBgn0083014 | snoRNA:Psi18S-996 | D_melanogaster | Animals | mono_intronic | H/ACA | FBgn0035121 | Tudor-SN | protein_coding |
| FBgn0083015 | snoRNA:Psi18S-920 | D_melanogaster | Animals | mono_intronic | H/ACA | FBgn0036213 | RpL10Ab | protein_coding |
| FBgn0083016 | snoRNA:Psi18S-841d | D_melanogaster | Animals | intronic_cluster | H/ACA | FBgn0020306 | dom | protein_coding |
| FBgn0083017 | snoRNA:Psi18S-841c | D_melanogaster | Animals | intronic_cluster | H/ACA | FBgn0020306 | dom | protein_coding |
| FBgn0083018 | snoRNA:Psi18S-841b | D_melanogaster | Animals | intronic_cluster | H/ACA | FBgn0020306 | dom | protein_coding |
| FBgn0083020 | snoRNA:Psi18S-640g | D_melanogaster | Animals | intronic_cluster | H/ACA | FBgn0032859 | Arpc2 | protein_coding |
| FBgn0083021 | snoRNA:Psi18S-640f | D_melanogaster | Animals | intronic_cluster | H/ACA | FBgn0032859 | Arpc2 | protein_coding |
| FBgn0083022 | snoRNA:Psi18S-640e | D_melanogaster | Animals | intronic_cluster | H/ACA | FBgn0032859 | Arpc2 | protein_coding |
| FBgn0083023 | snoRNA:Psi18S-640d | D_melanogaster | Animals | intronic_cluster | H/ACA | FBgn0032859 | Arpc2 | protein_coding |
| FBgn0083024 | snoRNA:Psi18S-640c | D_melanogaster | Animals | intronic_cluster | H/ACA | FBgn0032859 | Arpc2 | protein_coding |
| FBgn0083025 | snoRNA:Psi18S-640b | D_melanogaster | Animals | intronic_cluster | H/ACA | FBgn0032859 | Arpc2 | protein_coding |
| FBgn0083026 | snoRNA:Psi18S-640a | D_melanogaster | Animals | intronic_cluster | H/ACA | FBgn0032859 | Arpc2 | protein_coding |
| FBgn0083027 | snoRNA:Psi18S-531 | D_melanogaster | Animals | intronic_cluster | H/ACA | FBgn0015288 | RpL22 | protein_coding |
| FBgn0083028 | snoRNA:Psi18S-525k | D_melanogaster | Animals | mono_intronic | H/ACA | FBgn0261836 | Msp300 | protein_coding |
| FBgn0083029 | snoRNA:Psi18S-525j | D_melanogaster | Animals | intronic_cluster | H/ACA | FBgn0032859 | Arpc2 | protein_coding |
| FBgn0083030 | snoRNA:Psi18S-525i | D_melanogaster | Animals | intronic_cluster | H/ACA | FBgn0032859 | Arpc2 | protein_coding |
| FBgn0083031 | snoRNA:Psi18S-525h | D_melanogaster | Animals | intronic_cluster | H/ACA | FBgn0032859 | Arpc2 | protein_coding |
| FBgn0083032 | snoRNA:Psi18S-525g | D_melanogaster | Animals | intronic_cluster | H/ACA | FBgn0032859 | Arpc2 | protein_coding |
| FBgn0083033 | snoRNA:Psi18S-525f | D_melanogaster | Animals | intronic_cluster | H/ACA | FBgn0032859 | Arpc2 | protein_coding |
| FBgn0083034 | snoRNA:Psi18S-525e | D_melanogaster | Animals | intronic_cluster | H/ACA | FBgn0032859 | Arpc2 | protein_coding |
| FBgn0083035 | snoRNA:Psi18S-525d | D_melanogaster | Animals | intronic_cluster | H/ACA | FBgn0032859 | Arpc2 | protein_coding |
| FBgn0083036 | snoRNA:Psi18S-525c | D_melanogaster | Animals | intronic_cluster | H/ACA | FBgn0032859 | Arpc2 | protein_coding |
| FBgn0083037 | snoRNA:Psi18S-525b | D_melanogaster | Animals | intronic_cluster | H/ACA | FBgn0032859 | Arpc2 | protein_coding |
| FBgn0083038 | snoRNA:Psi18S-525a | D_melanogaster | Animals | intronic_cluster | H/ACA | FBgn0032859 | Arpc2 | protein_coding |
| FBgn0083039 | snoRNA:Psi18S-301 | D_melanogaster | Animals | mono_intergenic | H/ACA | | | intergenic |
| FBgn0083040 | snoRNA:Psi18S-1854c | D_melanogaster | Animals | intronic_cluster | H/ACA | FBgn0002590 | RpS5a | protein_coding |
| FBgn0083041 | snoRNA:Psi18S-1854b | D_melanogaster | Animals | intronic_cluster | H/ACA | FBgn0002590 | RpS5a | protein_coding |
| FBgn0083042 | snoRNA:Psi18S-1854a | D_melanogaster | Animals | intronic_cluster | H/ACA | FBgn0002590 | RpS5a | protein_coding |
| FBgn0083044 | snoRNA:Psi18S-1397 | D_melanogaster | Animals | mono_intronic | H/ACA | FBgn0035630 | CG10576 | protein_coding |
| FBgn0083045 | snoRNA:Psi18S-1389b | D_melanogaster | Animals | intronic_cluster | H/ACA | FBgn0020306 | dom | protein_coding |
| FBgn0083046 | snoRNA:Psi18S-1389a | D_melanogaster | Animals | intronic_cluster | H/ACA | FBgn0020306 | dom | protein_coding |
| FBgn0083047 | snoRNA:Psi18S-1377e | D_melanogaster | Animals | intronic_cluster | H/ACA | FBgn0039757 | RpS7 | protein_coding |
| FBgn0083048 | snoRNA:Psi18S-1377d | D_melanogaster | Animals | intronic_cluster | H/ACA | FBgn0039757 | RpS7 | protein_coding |
| FBgn0083049 | snoRNA:Psi18S-1377c | D_melanogaster | Animals | intronic_cluster | H/ACA | FBgn0039757 | RpS7 | protein_coding |
| FBgn0083050 | snoRNA:Psi18S-1377b | D_melanogaster | Animals | intronic_cluster | H/ACA | FBgn0039757 | RpS7 | protein_coding |
| FBgn0083053 | snoRNA:Psi18S-1347b | D_melanogaster | Animals | intronic_cluster | H/ACA | FBgn0265297 | pAbp | protein_coding |
| FBgn0083054 | snoRNA:Psi18S-1347a | D_melanogaster | Animals | intronic_cluster | H/ACA | FBgn0265297 | pAbp | protein_coding |
| FBgn0083055 | snoRNA:Psi18S-1295 | D_melanogaster | Animals | mono_intronic | H/ACA | FBgn0019936 | RpS20 | protein_coding |
| FBgn0083057 | snoRNA:Psi18S-110 | D_melanogaster | Animals | mono_intronic | H/ACA | FBgn0035162 | Sf3b3 | protein_coding |
| FBgn0083058 | snoRNA:Psi18S-1086 | D_melanogaster | Animals | mono_intronic | H/ACA | FBgn0035162 | Sf3b3 | protein_coding |
| FBgn0083988 | snoRNA:Me28S-C3420a | D_melanogaster | Animals | mono_intronic | C/D | FBgn0033699 | RpS11 | protein_coding |
| FBgn0083989 | snoRNA:Me28S-C3420b | D_melanogaster | Animals | mono_intronic | C/D | FBgn0033699 | RpS11 | protein_coding |
| FBgn0086021 | snoRNA:MeU6-C68 | D_melanogaster | Animals | mono_intronic | C/D | FBgn0021995 | Rs1 | protein_coding |
| FBgn0086022 | snoRNA:Me28S-U1130 | D_melanogaster | Animals | mono_intronic | C/D | FBgn0034408 | sano | protein_coding |

| | | | | | | | | |
|---|---|---|---|---|---|---|---|---|
| FBgn0086023 | snoRNA:Me28S-A2589a | D_melanogaster | Animals | mono_intronic | C/D | FBgn0020306 | dom | protein_coding |
| FBgn0086024 | snoRNA:Me28S-A2589b | D_melanogaster | Animals | mono_intronic | C/D | FBgn0020306 | dom | protein_coding |
| FBgn0086025 | snoRNA:Me28S-A2589c | D_melanogaster | Animals | mono_intronic | C/D | FBgn0020306 | dom | protein_coding |
| FBgn0086026 | snoRNA:Me28S-G2596 | D_melanogaster | Animals | mono_intronic | C/D | FBgn0020306 | dom | protein_coding |
| FBgn0086027 | snoRNA:Or-CD1 | D_melanogaster | Animals | mono_intronic | C/D | FBgn0034709 | Swim | protein_coding |
| FBgn0086028 | snoRNA:Me28S-G2173 | D_melanogaster | Animals | mono_intronic | C/D | FBgn0034743 | RpS16 | protein_coding |
| FBgn0086029 | snoRNA:Me18S-A1597 | D_melanogaster | Animals | mono_intronic | C/D | FBgn0050418 | nord | protein_coding |
| FBgn0086030 | snoRNA:Me18S-G393 | D_melanogaster | Animals | mono_intronic | C/D | FBgn0035041 | CG13594 | protein_coding |
| FBgn0086031 | snoRNA:Me28S-U1230 | D_melanogaster | Animals | mono_intronic | C/D | FBgn0029095 | aru | protein_coding |
| FBgn0086032 | snoRNA:Me28S-C437 | D_melanogaster | Animals | mono_intronic | C/D | FBgn0000547 | ed | protein_coding |
| FBgn0086033 | snoRNA:Me28S-A2486 | D_melanogaster | Animals | mono_intronic | C/D | FBgn0051647 | lncRNA:CR31647 | non_coding |
| FBgn0086034 | snoRNA:Me28S-G2743 | D_melanogaster | Animals | mono_intronic | C/D | FBgn0031855 | meng | protein_coding |
| FBgn0086035 | snoRNA:Me18S-G642 | D_melanogaster | Animals | mono_intronic | C/D | FBgn0264442 | ab | protein_coding |
| FBgn0086036 | snoRNA:Me18S-A934 | D_melanogaster | Animals | mono_intronic | C/D | FBgn0020309 | crol | protein_coding |
| FBgn0086037 | snoRNA:Me28S-A2958 | D_melanogaster | Animals | mono_intronic | C/D | FBgn0028523 | CG5888 | protein_coding |
| FBgn0086038 | snoRNA:Me28S-C1237 | D_melanogaster | Animals | mono_intronic | C/D | FBgn0032587 | CG5953 | protein_coding |
| FBgn0086039 | snoRNA:Me28S-A2113 | D_melanogaster | Animals | mono_intronic | C/D | FBgn0086710 | RpL30 | protein_coding |
| FBgn0086040 | snoRNA:Me28S-A3365 | D_melanogaster | Animals | mono_intronic | C/D | FBgn0015803 | RtGEF | protein_coding |
| FBgn0086041 | snoRNA:Me18S-A469 | D_melanogaster | Animals | mono_intronic | C/D | FBgn0032919 | pths | protein_coding |
| FBgn0086042 | snoRNA:Me28S-G980 | D_melanogaster | Animals | intronic_cluster | C/D | FBgn0003517 | sta | protein_coding |
| FBgn0086043 | snoRNA:Me18S-C1024 | D_melanogaster | Animals | mono_intronic | C/D | FBgn0000382 | csw | protein_coding |
| FBgn0086044 | snoRNA:Me28S-U1688 | D_melanogaster | Animals | mono_intronic | C/D | FBgn0026415 | Idgf4 | protein_coding |
| FBgn0086045 | snoRNA:Me18S-A627 | D_melanogaster | Animals | mono_intronic | C/D | FBgn0083940 | RhoU | protein_coding |
| FBgn0086046 | snoRNA:Me28S-A1705 | D_melanogaster | Animals | mono_intronic | C/D | FBgn0052683 | CG32683 | protein_coding |
| FBgn0086047 | snoRNA:Me18S-G894 | D_melanogaster | Animals | mono_intronic | C/D | FBgn0030396 | CG2556 | protein_coding |
| FBgn0086048 | snoRNA:Me28S-U3344b | D_melanogaster | Animals | mono_intronic | C/D | FBgn0031149 | Stt3A | protein_coding |
| FBgn0086049 | snoRNA:Me28S-U3344a | D_melanogaster | Animals | mono_intronic | C/D | FBgn0031149 | Stt3A | protein_coding |
| FBgn0086051 | snoRNA:Me28S-U2134a | D_melanogaster | Animals | intronic_cluster | C/D | FBgn0037351 | RpL13A | protein_coding |
| FBgn0086052 | snoRNA:Me18S-G423 | D_melanogaster | Animals | mono_intronic | C/D | FBgn0037546 | mAChR-B | protein_coding |
| FBgn0086053 | snoRNA:Me18S-G1358a | D_melanogaster | Animals | mono_intronic | C/D | FBgn0266410 | CG45050 | protein_coding |
| FBgn0086054 | snoRNA:Me18S-G1358b | D_melanogaster | Animals | mono_intronic | C/D | FBgn0266410 | CG45050 | protein_coding |
| FBgn0086055 | snoRNA:Me18S-C1096 | D_melanogaster | Animals | mono_intronic | C/D | FBgn0020910 | RpL3 | protein_coding |
| FBgn0086056 | snoRNA:Me18S-A425 | D_melanogaster | Animals | mono_intronic | C/D | FBgn0020910 | RpL3 | protein_coding |
| FBgn0086057 | snoRNA:Me28S-G2017 | D_melanogaster | Animals | mono_intronic | C/D | FBgn0020910 | RpL3 | protein_coding |
| FBgn0086058 | snoRNA:Me18S-G1189 | D_melanogaster | Animals | mono_intronic | C/D | FBgn0024321 | NK7.1 | protein_coding |
| FBgn0086059 | snoRNA:Me18S-C1280 | D_melanogaster | Animals | mono_intronic | C/D | FBgn0004885 | tok | protein_coding |
| FBgn0086060 | snoRNA:Me28S-U1554 | D_melanogaster | Animals | mono_intronic | C/D | FBgn0262473 | Tl | protein_coding |
| FBgn0086061 | snoRNA:Or-CD8 | D_melanogaster | Animals | mono_intronic | C/D | FBgn0039713 | RpS8 | protein_coding |
| FBgn0086062 | snoRNA:Me28S-C788b | D_melanogaster | Animals | mono_intronic | C/D | FBgn0035162 | Sf3b3 | protein_coding |
| FBgn0086063 | snoRNA:Me28S-C788a | D_melanogaster | Animals | mono_intronic | C/D | FBgn0035162 | Sf3b3 | protein_coding |
| FBgn0086064 | snoRNA:Me28S-G3113b | D_melanogaster | Animals | mono_intronic | C/D | FBgn0035162 | Sf3b3 | protein_coding |
| FBgn0086065 | snoRNA:Me28S-G3113a | D_melanogaster | Animals | mono_intronic | C/D | FBgn0035162 | Sf3b3 | protein_coding |
| FBgn0086066 | snoRNA:Me5.8S-G74 | D_melanogaster | Animals | mono_intronic | C/D | FBgn0035162 | Sf3b3 | protein_coding |
| FBgn0086067 | snoRNA:Me28S-A771 | D_melanogaster | Animals | mono_intronic | C/D | FBgn0035162 | Sf3b3 | protein_coding |
| FBgn0086068 | snoRNA:Or-CD12 | D_melanogaster | Animals | mono_intronic | C/D | FBgn0035422 | RpL28 | protein_coding |

| | | | | | | | | |
|---|---|---|---|---|---|---|---|---|
| FBgn0086069 | snoRNA:Me28S-C3351 | D_melanogaster | Animals | mono_intronic | C/D | FBgn0035422 | RpL28 | protein_coding |
| FBgn0086070 | snoRNA:Or-CD11c | D_melanogaster | Animals | intronic_cluster | C/D | FBgn0035422 | RpL28 | protein_coding |
| FBgn0086071 | snoRNA:Or-CD11b | D_melanogaster | Animals | intronic_cluster | C/D | FBgn0035422 | RpL28 | protein_coding |
| FBgn0086072 | snoRNA:Or-CD11a | D_melanogaster | Animals | intronic_cluster | C/D | FBgn0035422 | RpL28 | protein_coding |
| FBgn0086073 | snoRNA:Me18S-A1494 | D_melanogaster | Animals | mono_intronic | C/D | FBgn0284236 | CG46320 | protein_coding |
| FBgn0086074 | snoRNA:Me18S-A1806 | D_melanogaster | Animals | mono_intergenic | C/D | | | intergenic |
| FBgn0086075 | snoRNA:Me28S-G3253 | D_melanogaster | Animals | mono_intronic | C/D | FBgn0010408 | RpS9 | protein_coding |
| FBgn0086076 | snoRNA:MeU2-A31 | D_melanogaster | Animals | mono_intronic | C/D | FBgn0052057 | dpr10 | protein_coding |
| FBgn0086077 | snoRNA:Me18S-G962 | D_melanogaster | Animals | mono_intronic | C/D | FBgn0013469 | klu | protein_coding |
| FBgn0086078 | snoRNA:Me28S-A774b | D_melanogaster | Animals | mono_intronic | C/D | FBgn0286213 | RpS12 | protein_coding |
| FBgn0086079 | snoRNA:Me28S-A774a | D_melanogaster | Animals | mono_intronic | C/D | FBgn0286213 | RpS12 | protein_coding |
| FBgn0086080 | snoRNA:Me28S-A576 | D_melanogaster | Animals | mono_intronic | C/D | FBgn0000567 | Eip74EF | protein_coding |
| FBgn0086081 | snoRNA:Me28S-A30 | D_melanogaster | Animals | mono_intronic | C/D | FBgn0000568 | Eip75B | protein_coding |
| FBgn0086082 | snoRNA:Or-CD11 | D_melanogaster | Animals | mono_intronic | C/D | FBgn0083120 | Uhg8 | non_coding |
| FBgn0086533 | snoRNA:Me18S-C419 | D_melanogaster | Animals | mono_intronic | C/D | FBgn0028697 | RpL15 | protein_coding |
| FBgn0086600 | snoRNA:Psi18S-1347c | D_melanogaster | Animals | intronic_cluster | H/ACA | FBgn0265297 | pAbp | protein_coding |
| FBgn0086601 | snoRNA:Psi28S-3327c | D_melanogaster | Animals | mono_intronic | H/ACA | FBgn0011284 | RpS4 | protein_coding |
| FBgn0086602 | snoRNA:Psi28S-3436b | D_melanogaster | Animals | intronic_cluster | H/ACA | FBgn0029897 | RpL17 | protein_coding |
| FBgn0086603 | snoRNA:Or-CD2 | D_melanogaster | Animals | mono_intronic | C/D | FBgn0083124 | Uhg4 | non_coding |
| FBgn0086658 | snoRNA:Psi28S-1180 | D_melanogaster | Animals | mono_intronic | H/ACA | FBgn0033636 | tou | protein_coding |
| FBgn0086659 | snoRNA:Psi18S-176 | D_melanogaster | Animals | mono_intergenic | H/ACA | | | intergenic |
| FBgn0086660 | snoRNA:Psi28S-1837a | D_melanogaster | Animals | intronic_cluster | H/ACA | FBgn0035630 | CG10576 | protein_coding |
| FBgn0086661 | snoRNA:Psi28S-2566 | D_melanogaster | Animals | mono_intronic | H/ACA | FBgn0036824 | CG3902 | protein_coding |
| FBgn0086662 | snoRNA:Psi28S-3186 | D_melanogaster | Animals | mono_intronic | H/ACA | FBgn0250753 | kra | protein_coding |
| FBgn0086663 | snoRNA:Psi18S-1275 | D_melanogaster | Animals | mono_intronic | H/ACA | FBgn0261599 | RpS29 | protein_coding |
| FBgn0086664 | snoRNA:Psi28S-2149 | D_melanogaster | Animals | intronic_cluster | H/ACA | FBgn0039757 | RpS7 | protein_coding |
| FBgn0086665 | snoRNA:Psi18S-1377a | D_melanogaster | Animals | intronic_cluster | H/ACA | FBgn0039757 | RpS7 | protein_coding |
| FBgn0086666 | snoRNA:Psi28S-2179 | D_melanogaster | Animals | intronic_cluster | H/ACA | FBgn0015288 | RpL22 | protein_coding |
| FBgn0086667 | snoRNA:Psi28S-3342 | D_melanogaster | Animals | mono_intergenic | H/ACA | | | intergenic |
| FBgn0086668 | snoRNA:Psi28S-3436a | D_melanogaster | Animals | intronic_cluster | H/ACA | FBgn0029897 | RpL17 | protein_coding |
| FBgn0086669 | snoRNA:Psi18S-841a | D_melanogaster | Animals | intronic_cluster | H/ACA | FBgn0020306 | dom | protein_coding |
| FBgn0086670 | snoRNA:Psi28S-2622 | D_melanogaster | Animals | mono_intergenic | H/ACA | | | intergenic |
| FBgn0086671 | snoRNA:Psi28S-2876 | D_melanogaster | Animals | mono_intronic | H/ACA | FBgn0002590 | RpS5a | protein_coding |
| FBgn0086672 | snoRNA:Or-aca5 | D_melanogaster | Animals | mono_intronic | H/ACA | FBgn0083124 | Uhg4 | non_coding |
| FBgn0260002 | snoRNA:Me28S-U2134b | D_melanogaster | Animals | intronic_cluster | C/D | FBgn0037351 | RpL13A | protein_coding |
| FBgn0261006 | snoRNA:83E4-5 | D_melanogaster | Animals | mono_exonic | C/D | FBgn0260766 | CG42564 | protein_coding |
| FBgn0261452 | scaRNA:MeU2-G25 | D_melanogaster | Animals | mono_intronic | C/D | FBgn0043884 | mask | protein_coding |
| FBgn0261453 | scaRNA:MeU2-C41 | D_melanogaster | Animals | mono_intronic | C/D | FBgn0002542 | lds | protein_coding |
| FBgn0261454 | scaRNA:MeU4-A65 | D_melanogaster | Animals | mono_intronic | C/D | FBgn0036741 | anchor | protein_coding |
| FBgn0261455 | scaRNA:MeU5-G38 | D_melanogaster | Animals | mono_intronic | C/D | FBgn0266581 | pit | protein_coding |
| FBgn0261973 | snoRNA:Or-CD9a | D_melanogaster | Animals | mono_intronic | C/D | FBgn0259937 | Nop60B | protein_coding |
| FBgn0261974 | snoRNA:Or-CD9b | D_melanogaster | Animals | mono_intronic | C/D | FBgn0259937 | Nop60B | protein_coding |
| FBgn0263201 | scaRNA:MeU1b-A234 | D_melanogaster | Animals | intergenic_cluster | C/D | | | intergenic |
| FBgn0263458 | snoRNA:CG43051-a | D_melanogaster | Animals | mono_exonic | Unknown | FBgn0262353 | CG43051 | protein_coding |
| FBgn0263459 | snoRNA:2R:9445410 | D_melanogaster | Animals | intergenic_cluster | Unknown | | | intergenic |

| | | | | | | | | |
|---|---|---|---|---|---|---|---|---|
| FBgn0263460 | snoRNA:CG31191-a | D_melanogaster | Animals | mono_intronic | Unknown | FBgn0051191 | CG31191 | protein_coding |
| FBgn0263461 | snoRNA:CG32479-a | D_melanogaster | Animals | mono_intronic | Unknown | FBgn0052479 | Usp10 | protein_coding |
| FBgn0263462 | snoRNA:Dek-a | D_melanogaster | Animals | mono_intronic | Unknown | FBgn0026533 | Dek | protein_coding |
| FBgn0263463 | snoRNA:gcl-a | D_melanogaster | Animals | mono_intronic | Unknown | FBgn0005695 | gcl | protein_coding |
| FBgn0263464 | snoRNA:hts-a | D_melanogaster | Animals | mono_intronic | Unknown | FBgn0263391 | hts | protein_coding |
| FBgn0263465 | snoRNA:kis-a | D_melanogaster | Animals | mono_intronic | Unknown | FBgn0266557 | kis | protein_coding |
| FBgn0263466 | snoRNA:lola-a | D_melanogaster | Animals | intronic_cluster | Unknown | FBgn0283521 | lola | protein_coding |
| FBgn0263467 | snoRNA:nop5-x16-a | D_melanogaster | Animals | mono_intronic | C/D | FBgn0026196 | nop5 | protein_coding |
| FBgn0263468 | snoRNA:SC35-a | D_melanogaster | Animals | intronic_cluster | Unknown | FBgn0265298 | SC35 | protein_coding |
| FBgn0263469 | snoRNA:SC35-b | D_melanogaster | Animals | intronic_cluster | Unknown | FBgn0265298 | SC35 | protein_coding |
| FBgn0263470 | snoRNA:Tudor-SN-a | D_melanogaster | Animals | mono_intronic | Unknown | FBgn0035121 | Tudor-SN | protein_coding |
| FBgn0263471 | scaRNA:MeU2-48 | D_melanogaster | Animals | mono_intronic | C/D | FBgn0028577 | hfp | protein_coding |
| FBgn0263472 | snoRNA:2R:9445205 | D_melanogaster | Animals | intergenic_cluster | Unknown | | | intergenic |
| FBgn0263473 | snoRNA:abba-a | D_melanogaster | Animals | mono_intronic | Unknown | FBgn0265356 | tn | protein_coding |
| FBgn0263474 | scaRNA:PsiU2-38.40.42 | D_melanogaster | Animals | mono_intronic | H/ACA | FBgn0000239 | bur | protein_coding |
| FBgn0263475 | snoRNA:CG16892-a | D_melanogaster | Animals | mono_intronic | Unknown | FBgn0030122 | Aladin | protein_coding |
| FBgn0263476 | snoRNA:CG32479-b | D_melanogaster | Animals | mono_intronic | Unknown | FBgn0052479 | Usp10 | protein_coding |
| FBgn0263477 | scaRNA:PsiU1-6 | D_melanogaster | Animals | mono_intronic | H/ACA | FBgn0278604 | dmt | protein_coding |
| FBgn0263478 | snoRNA:Edc3-a | D_melanogaster | Animals | mono_intronic | Unknown | FBgn0036735 | Edc3 | protein_coding |
| FBgn0263479 | scaRNA:PsiU2-35.45 | D_melanogaster | Animals | mono_intronic | H/ACA | FBgn0266580 | Gp210 | protein_coding |
| FBgn0263480 | snoRNA:Irbp-a | D_melanogaster | Animals | mono_intronic | Unknown | FBgn0011774 | Irbp | protein_coding |
| FBgn0263481 | snoRNA:lola-b | D_melanogaster | Animals | intronic_cluster | Unknown | FBgn0283521 | lola | protein_coding |
| FBgn0263482 | snoRNA:lola-c | D_melanogaster | Animals | intronic_cluster | Unknown | FBgn0283521 | lola | protein_coding |
| FBgn0263483 | snoRNA:lola-d | D_melanogaster | Animals | intronic_cluster | Unknown | FBgn0283521 | lola | protein_coding |
| FBgn0263484 | snoRNA:Pi4KIIalpha-a | D_melanogaster | Animals | mono_intronic | Unknown | FBgn0037339 | Pi4KIIalpha | protein_coding |
| FBgn0263485 | scaRNA:PsiU2-55 | D_melanogaster | Animals | mono_intronic | H/ACA | FBgn0033688 | Prp8 | protein_coding |
| FBgn0263486 | scaRNA:PsiU6-40 | D_melanogaster | Animals | mono_intronic | H/ACA | FBgn0003415 | skd | protein_coding |
| FBgn0263487 | scaRNA:PsiU5-44 | D_melanogaster | Animals | mono_intronic | H/ACA | FBgn0003742 | tra2 | protein_coding |
| FBgn0263851 | snoRNA:CD-24 | D_melanogaster | Animals | mono_intronic | C/D | FBgn0017545 | RpS3A | protein_coding |
| FBgn0286726 | snoRNA:Or-ACA6 | D_melanogaster | Animals | intergenic_cluster | H/ACA | | | intergenic |
| FBgn0286727 | snoRNA:Or-ACA7 | D_melanogaster | Animals | intergenic_cluster | H/ACA | | | intergenic |
| FBgn0286728 | snoRNA:Or-CD13 | D_melanogaster | Animals | mono_intergenic | C/D | | | intergenic |
| FBgn0286729 | snoRNA:Or-CD15 | D_melanogaster | Animals | mono_exonic | C/D | FBgn0026372 | RpL23A | protein_coding |
| FBgn0286730 | snoRNA:Me28S-A2629 | D_melanogaster | Animals | mono_intronic | C/D | FBgn0052062 | Rbfox1 | protein_coding |
| FBgn0286731 | snoRNA:Or-ACA8 | D_melanogaster | Animals | mono_intergenic | H/ACA | | | intergenic |
| FBgn0286732 | snoRNA:Or-CD14 | D_melanogaster | Animals | mono_intergenic | C/D | | | intergenic |
| FBgn0286733 | snoRNA:Me28S-C2789 | D_melanogaster | Animals | mono_intergenic | C/D | | | intergenic |
| FBgn0286734 | snoRNA:Me18S-G1506 | D_melanogaster | Animals | mono_intergenic | C/D | | | intergenic |
| FBgn0286735 | scaRNA:MeU1:95C-A24 | D_melanogaster | Animals | mono_intergenic | C/D | | | intergenic |
| FBgn0286758 | snoRNA:Me18S-G1358c | D_melanogaster | Animals | mono_intronic | C/D | FBgn0028697 | RpL15 | protein_coding |
| ENSGALG00010000403 | ENSGALG00010000403 | G_gallus | Animals | mono_intronic | C/D | ENSGALG00010000350 | | protein_coding |
| ENSGALG00010000573 | SNORD125 | G_gallus | Animals | mono_intronic | C/D | ENSGALG00010000323 | AP1B1 | protein_coding |
| ENSGALG00010000782 | ENSGALG00010000782 | G_gallus | Animals | mono_intronic | H/ACA | ENSGALG00010000763 | | non_coding |
| ENSGALG00010001016 | SNORD123 | G_gallus | Animals | mono_intronic | C/D | ENSGALG00010000971 | | non_coding |
| ENSGALG00010002961 | ENSGALG00010002961 | G_gallus | Animals | mono_intronic | C/D | ENSGALG00010002785 | | non_coding |

| | | | | | | | | |
|---|---|---|---|---|---|---|---|---|
| ENSGALG00010003177 | SNORA19 | G_gallus | Animals | mono_intronic | H/ACA | ENSGALG00010002150 | EIF3A | protein_coding |
| ENSGALG00010003179 | ENSGALG00010003179 | G_gallus | Animals | mono_intronic | H/ACA | ENSGALG00010002150 | EIF3A | protein_coding |
| ENSGALG00010003798 | SNORD15 | G_gallus | Animals | mono_intronic | C/D | ENSGALG00010002912 | RPS3 | protein_coding |
| ENSGALG00010003799 | SNORD15 | G_gallus | Animals | mono_intronic | C/D | ENSGALG00010002912 | RPS3 | protein_coding |
| ENSGALG00010003800 | SNORD15 | G_gallus | Animals | mono_intronic | C/D | ENSGALG00010002912 | RPS3 | protein_coding |
| ENSGALG00010003801 | ENSGALG00010003801 | G_gallus | Animals | mono_intronic | C/D | ENSGALG00010001654 | RPS11 | protein_coding |
| ENSGALG00010003802 | ENSGALG00010003802 | G_gallus | Animals | mono_intronic | C/D | ENSGALG00010001654 | RPS11 | protein_coding |
| ENSGALG00010004672 | ENSGALG00010004672 | G_gallus | Animals | mono_exonic | H/ACA | ENSGALG00010004262 | | non_coding |
| ENSGALG00010004674 | ENSGALG00010004674 | G_gallus | Animals | mono_intronic | H/ACA | ENSGALG00010003558 | TPT1 | protein_coding |
| ENSGALG00010004675 | ENSGALG00010004675 | G_gallus | Animals | mono_intergenic | C/D | | | intergenic |
| ENSGALG00010004906 | ENSGALG00010004906 | G_gallus | Animals | mono_intergenic | C/D | | | intergenic |
| ENSGALG00010004908 | ENSGALG00010004908 | G_gallus | Animals | mono_intergenic | C/D | | | intergenic |
| ENSGALG00010005938 | ENSGALG00010005938 | G_gallus | Animals | mono_intergenic | H/ACA | | | intergenic |
| ENSGALG00010006048 | SNORD54 | G_gallus | Animals | mono_intronic | C/D | ENSGALG00010004081 | RPS20 | protein_coding |
| ENSGALG00010006224 | ENSGALG00010006224 | G_gallus | Animals | mono_intronic | H/ACA | ENSGALG00010006001 | NOP56 | protein_coding |
| ENSGALG00010006225 | ENSGALG00010006225 | G_gallus | Animals | mono_intronic | C/D | ENSGALG00010006001 | NOP56 | protein_coding |
| ENSGALG00010006226 | SNORD57 | G_gallus | Animals | mono_intronic | C/D | ENSGALG00010006001 | NOP56 | protein_coding |
| ENSGALG00010006773 | SNORD100 | G_gallus | Animals | mono_intronic | C/D | ENSGALG00010004255 | RPS12 | protein_coding |
| ENSGALG00010006777 | ENSGALG00010006777 | G_gallus | Animals | mono_intronic | H/ACA | ENSGALG00010004255 | RPS12 | protein_coding |
| ENSGALG00010006779 | SNORD101 | G_gallus | Animals | mono_intronic | C/D | ENSGALG00010004255 | RPS12 | protein_coding |
| ENSGALG00010006781 | ENSGALG00010006781 | G_gallus | Animals | mono_exonic | C/D | ENSGALG00010004255 | RPS12 | protein_coding |
| ENSGALG00010007174 | ENSGALG00010007174 | G_gallus | Animals | mono_intronic | H/ACA | ENSGALG00010004434 | TCP1 | protein_coding |
| ENSGALG00010007502 | ENSGALG00010007502 | G_gallus | Animals | mono_intronic | H/ACA | ENSGALG00010005785 | RPL21 | protein_coding |
| ENSGALG00010007505 | SNORD102 | G_gallus | Animals | mono_intronic | C/D | ENSGALG00010005785 | RPL21 | protein_coding |
| ENSGALG00010007723 | ENSGALG00010007723 | G_gallus | Animals | mono_intronic | C/D | ENSGALG00010006252 | COX7C | protein_coding |
| ENSGALG00010007727 | ENSGALG00010007727 | G_gallus | Animals | mono_intronic | C/D | ENSGALG00010006252 | COX7C | protein_coding |
| ENSGALG00010009060 | SNORD73 | G_gallus | Animals | mono_exonic | C/D | ENSGALG00010007539 | RPS3A | protein_coding |
| ENSGALG00010009413 | ENSGALG00010009413 | G_gallus | Animals | mono_exonic | C/D | ENSGALG00010009364 | | non_coding |
| ENSGALG00010009414 | ENSGALG00010009414 | G_gallus | Animals | mono_intronic | C/D | ENSGALG00010008837 | | protein_coding |
| ENSGALG00010009417 | ENSGALG00010009417 | G_gallus | Animals | mono_exonic | H/ACA | ENSGALG00010009364 | | non_coding |
| ENSGALG00010009418 | ENSGALG00010009418 | G_gallus | Animals | mono_intronic | C/D | ENSGALG00010009364 | | non_coding |
| ENSGALG00010009419 | ENSGALG00010009419 | G_gallus | Animals | mono_exonic | H/ACA | ENSGALG00010009364 | | non_coding |
| ENSGALG00010009420 | ENSGALG00010009420 | G_gallus | Animals | mono_intronic | H/ACA | ENSGALG00010009364 | | non_coding |
| ENSGALG00010009421 | ENSGALG00010009421 | G_gallus | Animals | mono_intronic | H/ACA | ENSGALG00010009364 | | non_coding |
| ENSGALG00010010344 | SNORD71 | G_gallus | Animals | mono_intronic | C/D | ENSGALG00010008426 | AP1G1 | protein_coding |
| ENSGALG00010010348 | ENSGALG00010010348 | G_gallus | Animals | mono_intronic | C/D | ENSGALG00010007998 | RPL13 | protein_coding |
| ENSGALG00010010808 | ENSGALG00010010808 | G_gallus | Animals | mono_intronic | H/ACA | ENSGALG00010010621 | | non_coding |
| ENSGALG00010010875 | ENSGALG00010010875 | G_gallus | Animals | mono_intronic | H/ACA | ENSGALG00010009491 | RBM34 | protein_coding |
| ENSGALG00010011391 | ENSGALG00010011391 | G_gallus | Animals | mono_intronic | H/ACA | ENSGALG00010008527 | CHUK | protein_coding |
| ENSGALG00010011396 | SNORA74 | G_gallus | Animals | mono_intronic | H/ACA | ENSGALG00010010768 | TIMM23B | protein_coding |
| ENSGALG00010012810 | SNORD58 | G_gallus | Animals | mono_intronic | C/D | ENSGALG00010011192 | RPL17 | protein_coding |
| ENSGALG00010012811 | SNORD58 | G_gallus | Animals | mono_intronic | C/D | ENSGALG00010011192 | RPL17 | protein_coding |
| ENSGALG00010012813 | SNORD58 | G_gallus | Animals | mono_intronic | C/D | ENSGALG00010011192 | RPL17 | protein_coding |
| ENSGALG00010012815 | SNORD58 | G_gallus | Animals | mono_intronic | C/D | ENSGALG00010011192 | RPL17 | protein_coding |
| ENSGALG00010012817 | ENSGALG00010012817 | G_gallus | Animals | mono_intronic | C/D | ENSGALG00010011429 | UBAP2 | protein_coding |

| | | | | | | | | |
|---|---|---|---|---|---|---|---|---|
| ENSGALG00010012820 | ENSGALG00010012820 | G_gallus | Animals | mono_intronic | C/D | ENSGALG00010011429 | UBAP2 | protein_coding |
| ENSGALG00010012936 | SNORD63 | G_gallus | Animals | mono_intronic | C/D | ENSGALG00010010262 | HSPA9 | protein_coding |
| ENSGALG00010013287 | ENSGALG00010013287 | G_gallus | Animals | mono_intergenic | H/ACA | | | intergenic |
| ENSGALG00010013832 | SNORD58 | G_gallus | Animals | mono_intronic | C/D | ENSGALG00010012282 | | protein_coding |
| ENSGALG00010013834 | SNORD58 | G_gallus | Animals | mono_intronic | C/D | ENSGALG00010012286 | | protein_coding |
| ENSGALG00010013839 | SNORD58 | G_gallus | Animals | mono_intronic | C/D | ENSGALG00010012286 | | protein_coding |
| ENSGALG00010013843 | SNORD58 | G_gallus | Animals | mono_intronic | C/D | ENSGALG00010012286 | | protein_coding |
| ENSGALG00010013844 | SNORD58 | G_gallus | Animals | mono_intronic | C/D | ENSGALG00010012282 | | protein_coding |
| ENSGALG00010013847 | SNORD58 | G_gallus | Animals | mono_intronic | C/D | ENSGALG00010012286 | | protein_coding |
| ENSGALG00010013849 | SNORD58 | G_gallus | Animals | mono_intronic | C/D | ENSGALG00010012282 | | protein_coding |
| ENSGALG00010013852 | ENSGALG00010013852 | G_gallus | Animals | mono_intronic | C/D | ENSGALG00010011436 | UBAP2L2 | protein_coding |
| ENSGALG00010014133 | SNORA72 | G_gallus | Animals | mono_intronic | H/ACA | ENSGALG00010011650 | RPL30 | protein_coding |
| ENSGALG00010014219 | SNORA53 | G_gallus | Animals | mono_intronic | H/ACA | ENSGALG00010010588 | SLC25A3 | protein_coding |
| ENSGALG00010014819 | SNORD24 | G_gallus | Animals | intergenic_cluster | C/D | | | intergenic |
| ENSGALG00010014820 | SNORD79 | G_gallus | Animals | mono_intronic | C/D | ENSGALG00010011434 | NPL | protein_coding |
| ENSGALG00010014823 | ENSGALG00010014823 | G_gallus | Animals | mono_intergenic | C/D | | | intergenic |
| ENSGALG00010014825 | ENSGALG00010014825 | G_gallus | Animals | intergenic_cluster | C/D | | | intergenic |
| ENSGALG00010014827 | ENSGALG00010014827 | G_gallus | Animals | intergenic_cluster | C/D | | | intergenic |
| ENSGALG00010014832 | SNORD81 | G_gallus | Animals | intronic_cluster | C/D | ENSGALG00010011434 | NPL | protein_coding |
| ENSGALG00010014834 | ENSGALG00010014834 | G_gallus | Animals | intergenic_cluster | C/D | | | intergenic |
| ENSGALG00010014836 | ENSGALG00010014836 | G_gallus | Animals | mono_intronic | C/D | ENSGALG00010011434 | NPL | protein_coding |
| ENSGALG00010014838 | ENSGALG00010014838 | G_gallus | Animals | intronic_cluster | C/D | ENSGALG00010011434 | NPL | protein_coding |
| ENSGALG00010014871 | ENSGALG00010014871 | G_gallus | Animals | mono_intronic | H/ACA | ENSGALG00010012988 | RPL18A | protein_coding |
| ENSGALG00010014901 | ENSGALG00010014901 | G_gallus | Animals | mono_intergenic | H/ACA | | | intergenic |
| ENSGALG00010015590 | SNORA66 | G_gallus | Animals | mono_intronic | H/ACA | ENSGALG00010014907 | | protein_coding |
| ENSGALG00010015594 | SNORD72 | G_gallus | Animals | mono_intronic | C/D | ENSGALG00010013438 | RPL37 | protein_coding |
| ENSGALG00010015878 | SNORD83A | G_gallus | Animals | mono_intronic | C/D | ENSGALG00010012610 | RPL3 | protein_coding |
| ENSGALG00010016351 | ENSGALG00010016351 | G_gallus | Animals | mono_intronic | H/ACA | ENSGALG00010012955 | RPL39L | protein_coding |
| ENSGALG00010016427 | SNORD89 | G_gallus | Animals | mono_exonic | C/D | ENSGALG00010016048 | | non_coding |
| ENSGALG00010017506 | ENSGALG00010017506 | G_gallus | Animals | mono_intronic | H/ACA | ENSGALG00010017231 | | non_coding |
| ENSGALG00010018194 | ENSGALG00010018194 | G_gallus | Animals | intronic_cluster | C/D | ENSGALG00010015937 | | protein_coding |
| ENSGALG00010018197 | ENSGALG00010018197 | G_gallus | Animals | intronic_cluster | H/ACA | ENSGALG00010015937 | | protein_coding |
| ENSGALG00010018200 | ENSGALG00010018200 | G_gallus | Animals | intronic_cluster | H/ACA | ENSGALG00010015937 | | protein_coding |
| ENSGALG00010018202 | ENSGALG00010018202 | G_gallus | Animals | intronic_cluster | H/ACA | ENSGALG00010015937 | | protein_coding |
| ENSGALG00010018203 | ENSGALG00010018203 | G_gallus | Animals | mono_intronic | H/ACA | ENSGALG00010017826 | PABPC4 | protein_coding |
| ENSGALG00010018205 | ENSGALG00010018205 | G_gallus | Animals | mono_intronic | H/ACA | ENSGALG00010017826 | PABPC4 | protein_coding |
| ENSGALG00010018208 | ENSGALG00010018208 | G_gallus | Animals | mono_intronic | H/ACA | ENSGALG00010017826 | PABPC4 | protein_coding |
| ENSGALG00010018212 | ENSGALG00010018212 | G_gallus | Animals | mono_intronic | C/D | ENSGALG00010013453 | PUM1 | protein_coding |
| ENSGALG00010018213 | ENSGALG00010018213 | G_gallus | Animals | mono_intronic | C/D | ENSGALG00010013453 | PUM1 | protein_coding |
| ENSGALG00010018224 | SNORA73 | G_gallus | Animals | mono_intronic | H/ACA | ENSGALG00010017943 | | non_coding |
| ENSGALG00010018475 | SNORA28 | G_gallus | Animals | mono_intronic | H/ACA | ENSGALG00010015759 | EIF5 | protein_coding |
| ENSGALG00010018964 | SNORA63 | G_gallus | Animals | mono_intronic | H/ACA | ENSGALG00010016240 | EIF4A2 | protein_coding |
| ENSGALG00010018967 | ENSGALG00010018967 | G_gallus | Animals | mono_intronic | H/ACA | ENSGALG00010016240 | EIF4A2 | protein_coding |
| ENSGALG00010018970 | ENSGALG00010018970 | G_gallus | Animals | mono_intronic | C/D | ENSGALG00010016240 | EIF4A2 | protein_coding |
| ENSGALG00010018976 | ENSGALG00010018976 | G_gallus | Animals | mono_intronic | C/D | ENSGALG00010017540 | EIF4G1 | protein_coding |

| | | | | | | | | |
|---|---|---|---|---|---|---|---|---|
| ENSGALG00010018978 | ENSGALG00010018978 | G_gallus | Animals | mono_intronic | C/D | ENSGALG00010017540 | EIF4G1 | protein_coding |
| ENSGALG00010018979 | SNORD20 | G_gallus | Animals | mono_intronic | C/D | ENSGALG00010015618 | NCL | protein_coding |
| ENSGALG00010018981 | SNORD82 | G_gallus | Animals | mono_intronic | C/D | ENSGALG00010015618 | NCL | protein_coding |
| ENSGALG00010018982 | SNORA75 | G_gallus | Animals | mono_intronic | H/ACA | ENSGALG00010015618 | NCL | protein_coding |
| ENSGALG00010019156 | ENSGALG00010019156 | G_gallus | Animals | mono_intronic | C/D | ENSGALG00010017770 | PTCD3 | protein_coding |
| ENSGALG00010019884 | SNORD61 | G_gallus | Animals | mono_intronic | C/D | ENSGALG00010015895 | RBMXL1 | protein_coding |
| ENSGALG00010019886 | ENSGALG00010019886 | G_gallus | Animals | mono_intronic | H/ACA | ENSGALG00010014691 | FAM122B | protein_coding |
| ENSGALG00010019888 | SNORD83 | G_gallus | Animals | mono_intronic | C/D | ENSGALG00010015521 | DKC1 | protein_coding |
| ENSGALG00010019890 | ENSGALG00010019890 | G_gallus | Animals | mono_intronic | C/D | ENSGALG00010015521 | DKC1 | protein_coding |
| ENSGALG00010019893 | ENSGALG00010019893 | G_gallus | Animals | mono_intronic | H/ACA | ENSGALG00010015521 | DKC1 | protein_coding |
| ENSGALG00010019894 | SNORD83 | G_gallus | Animals | mono_intronic | C/D | ENSGALG00010015521 | DKC1 | protein_coding |
| ENSGALG00010019895 | ENSGALG00010019895 | G_gallus | Animals | mono_intronic | C/D | ENSGALG00010015521 | DKC1 | protein_coding |
| ENSGALG00010019959 | ENSGALG00010019959 | G_gallus | Animals | mono_intronic | C/D | ENSGALG00010017677 | WDR43 | protein_coding |
| ENSGALG00010019963 | ENSGALG00010019963 | G_gallus | Animals | mono_intronic | C/D | ENSGALG00010017677 | WDR43 | protein_coding |
| ENSGALG00010019965 | ENSGALG00010019965 | G_gallus | Animals | mono_intronic | C/D | ENSGALG00010017677 | WDR43 | protein_coding |
| ENSGALG00010019969 | ENSGALG00010019969 | G_gallus | Animals | mono_intronic | C/D | ENSGALG00010017677 | WDR43 | protein_coding |
| ENSGALG00010020073 | SNORA62 | G_gallus | Animals | mono_intronic | H/ACA | ENSGALG00010016040 | RPSAP58 | protein_coding |
| ENSGALG00010020078 | SNORA62 | G_gallus | Animals | mono_intronic | H/ACA | ENSGALG00010016040 | RPSAP58 | protein_coding |
| ENSGALG00010020472 | SNORD127 | G_gallus | Animals | mono_intronic | C/D | ENSGALG00010018312 | PRPF39 | protein_coding |
| ENSGALG00010020850 | SNORD16 | G_gallus | Animals | mono_intronic | C/D | ENSGALG00010018036 | RPL4 | protein_coding |
| ENSGALG00010020852 | SNORD18 | G_gallus | Animals | mono_intronic | C/D | ENSGALG00010018036 | RPL4 | protein_coding |
| ENSGALG00010020860 | SNORD18 | G_gallus | Animals | mono_intronic | C/D | ENSGALG00010018036 | RPL4 | protein_coding |
| ENSGALG00010020863 | SNORD16 | G_gallus | Animals | mono_intronic | C/D | ENSGALG00010018036 | RPL4 | protein_coding |
| ENSGALG00010020867 | SNORD18 | G_gallus | Animals | mono_intronic | C/D | ENSGALG00010018036 | RPL4 | protein_coding |
| ENSGALG00010021006 | ENSGALG00010021006 | G_gallus | Animals | mono_intergenic | C/D | | | intergenic |
| ENSGALG00010023016 | ENSGALG00010023016 | G_gallus | Animals | mono_intergenic | H/ACA | | | intergenic |
| ENSGALG00010023768 | ENSGALG00010023768 | G_gallus | Animals | mono_intronic | C/D | ENSGALG00010022971 | CKAP5 | protein_coding |
| ENSGALG00010023795 | SNORD98 | G_gallus | Animals | mono_intronic | C/D | ENSGALG00010023140 | CCAR1 | protein_coding |
| ENSGALG00010024090 | SNORD97 | G_gallus | Animals | mono_intronic | C/D | ENSGALG00010021044 | EIF4G2 | protein_coding |
| ENSGALG00010024092 | ENSGALG00010024092 | G_gallus | Animals | mono_intronic | H/ACA | ENSGALG00010020612 | RPL27A | protein_coding |
| ENSGALG00010024093 | ENSGALG00010024093 | G_gallus | Animals | mono_intronic | H/ACA | ENSGALG00010020612 | RPL27A | protein_coding |
| ENSGALG00010024094 | SNORA23 | G_gallus | Animals | mono_intronic | H/ACA | ENSGALG00010021175 | IPO7 | protein_coding |
| ENSGALG00010024481 | SNORD21 | G_gallus | Animals | mono_intronic | C/D | ENSGALG00010021788 | RPL5 | protein_coding |
| ENSGALG00010025276 | SNORA71 | G_gallus | Animals | mono_intronic | H/ACA | ENSGALG00010023334 | RPS17 | protein_coding |
| ENSGALG00010025374 | ENSGALG00010025374 | G_gallus | Animals | mono_intergenic | C/D | | | intergenic |
| ENSGALG00010025376 | ENSGALG00010025376 | G_gallus | Animals | mono_intergenic | C/D | | | intergenic |
| ENSGALG00010025383 | ENSGALG00010025383 | G_gallus | Animals | mono_intergenic | C/D | | | intergenic |
| ENSGALG00010025385 | ENSGALG00010025385 | G_gallus | Animals | mono_intergenic | C/D | | | intergenic |
| ENSGALG00010025752 | ENSGALG00010025752 | G_gallus | Animals | mono_intronic | C/D | ENSGALG00010023190 | SF3B3 | protein_coding |
| ENSGALG00010025756 | ENSGALG00010025756 | G_gallus | Animals | mono_intronic | H/ACA | ENSGALG00010021665 | CNOT1 | protein_coding |
| ENSGALG00010025759 | SNORA50A | G_gallus | Animals | intronic_cluster | H/ACA | ENSGALG00010021665 | CNOT1 | protein_coding |
| ENSGALG00010025761 | SNORA50A | G_gallus | Animals | intronic_cluster | H/ACA | ENSGALG00010021665 | CNOT1 | protein_coding |
| ENSGALG00010025909 | ENSGALG00010025909 | G_gallus | Animals | mono_intronic | C/D | ENSGALG00010025098 | ATP5B | protein_coding |
| ENSGALG00010025911 | ENSGALG00010025911 | G_gallus | Animals | mono_intronic | C/D | ENSGALG00010025098 | ATP5B | protein_coding |
| ENSGALG00010026022 | SCARNA10 | G_gallus | Animals | mono_intronic | C/D | ENSGALG00010021989 | NCAPD2 | protein_coding |

| | | | | | | | | |
|---|---|---|---|---|---|---|---|---|
| ENSGALG00010026369 | SNORD14 | G_gallus | Animals | mono_intronic | C/D | ENSGALG00010025592 | HSPA8 | protein_coding |
| ENSGALG00010026371 | SNORD14 | G_gallus | Animals | mono_intronic | C/D | ENSGALG00010025592 | HSPA8 | protein_coding |
| ENSGALG00010026374 | SNORD14 | G_gallus | Animals | mono_intronic | C/D | ENSGALG00010025592 | HSPA8 | protein_coding |
| ENSGALG00010026639 | ENSGALG00010026639 | G_gallus | Animals | mono_intronic | C/D | ENSGALG00010025157 | NOP58 | protein_coding |
| ENSGALG00010026642 | ENSGALG00010026642 | G_gallus | Animals | mono_intronic | C/D | ENSGALG00010025157 | NOP58 | protein_coding |
| ENSGALG00010026644 | SNORD11B | G_gallus | Animals | mono_intronic | C/D | ENSGALG00010025157 | NOP58 | protein_coding |
| ENSGALG00010026646 | ENSGALG00010026646 | G_gallus | Animals | mono_intronic | C/D | ENSGALG00010025157 | NOP58 | protein_coding |
| ENSGALG00010026647 | ENSGALG00010026647 | G_gallus | Animals | mono_intronic | H/ACA | ENSGALG00010025974 | EEF1B2 | protein_coding |
| ENSGALG00010026648 | ENSGALG00010026648 | G_gallus | Animals | mono_intronic | C/D | ENSGALG00010025974 | EEF1B2 | protein_coding |
| ENSGALG00010026649 | ENSGALG00010026649 | G_gallus | Animals | mono_intronic | H/ACA | ENSGALG00010022173 | RPL23 | protein_coding |
| ENSGALG00010026875 | ENSGALG00010026875 | G_gallus | Animals | mono_intronic | H/ACA | ENSGALG00010024112 | NAP1L4 | protein_coding |
| ENSGALG00010026876 | ENSGALG00010026876 | G_gallus | Animals | mono_intronic | H/ACA | ENSGALG00010024112 | NAP1L4 | protein_coding |
| ENSGALG00010026878 | SNORA52 | G_gallus | Animals | mono_intronic | H/ACA | ENSGALG00010023115 | RPLP2 | protein_coding |
| ENSGALG00010026902 | ENSGALG00010026902 | G_gallus | Animals | mono_intronic | H/ACA | ENSGALG00010020833 | RANBP1 | protein_coding |
| ENSGALG00010027053 | SNORD39 | G_gallus | Animals | mono_intronic | C/D | ENSGALG00010025642 | RPS8 | protein_coding |
| ENSGALG00010027054 | ENSGALG00010027054 | G_gallus | Animals | mono_exonic | H/ACA | ENSGALG00010026946 | | non_coding |
| ENSGALG00010027060 | ENSGALG00010027060 | G_gallus | Animals | mono_intronic | C/D | ENSGALG00010025642 | RPS8 | protein_coding |
| ENSGALG00010027061 | ENSGALG00010027061 | G_gallus | Animals | mono_intronic | C/D | ENSGALG00010025642 | RPS8 | protein_coding |
| ENSGALG00010027062 | ENSGALG00010027062 | G_gallus | Animals | mono_intronic | C/D | ENSGALG00010024073 | RABGGTB | protein_coding |
| ENSGALG00010027064 | ENSGALG00010027064 | G_gallus | Animals | mono_intronic | C/D | ENSGALG00010024073 | RABGGTB | protein_coding |
| ENSGALG00010027673 | ENSGALG00010027673 | G_gallus | Animals | mono_intronic | H/ACA | ENSGALG00010025951 | TBRG4 | protein_coding |
| ENSGALG00010027675 | ENSGALG00010027675 | G_gallus | Animals | mono_intronic | H/ACA | ENSGALG00010025951 | TBRG4 | protein_coding |
| ENSGALG00010027676 | ENSGALG00010027676 | G_gallus | Animals | mono_intronic | H/ACA | ENSGALG00010025951 | TBRG4 | protein_coding |
| ENSGALG00010027677 | ENSGALG00010027677 | G_gallus | Animals | mono_intronic | H/ACA | ENSGALG00010025951 | TBRG4 | protein_coding |
| ENSGALG00010027678 | ENSGALG00010027678 | G_gallus | Animals | mono_intergenic | H/ACA | | | intergenic |
| ENSGALG00010028390 | ENSGALG00010028390 | G_gallus | Animals | mono_intronic | C/D | ENSGALG00010028051 | GNL3 | protein_coding |
| ENSGALG00010028393 | SNORD19 | G_gallus | Animals | mono_intronic | C/D | ENSGALG00010028051 | GNL3 | protein_coding |
| ENSGALG00010028395 | ENSGALG00010028395 | G_gallus | Animals | mono_intronic | C/D | ENSGALG00010028051 | GNL3 | protein_coding |
| ENSGALG00010028396 | SNORD69 | G_gallus | Animals | mono_intronic | C/D | ENSGALG00010028051 | GNL3 | protein_coding |
| ENSGALG00010028398 | SNORA84 | G_gallus | Animals | mono_intronic | H/ACA | ENSGALG00010027218 | IARS1 | protein_coding |
| ENSGALG00010028718 | ENSGALG00010028718 | G_gallus | Animals | mono_intronic | C/D | ENSGALG00010027732 | EEF2 | protein_coding |
| ENSGALG00010028843 | ENSGALG00010028843 | G_gallus | Animals | mono_intronic | H/ACA | ENSGALG00010027867 | UBAP2L | protein_coding |
| ENSGALG00010029119 | ENSGALG00010029119 | G_gallus | Animals | intergenic_cluster | H/ACA | | | intergenic |
| ENSGALG00010029120 | ENSGALG00010029120 | G_gallus | Animals | intergenic_cluster | H/ACA | | | intergenic |
| ENSGALG00010029122 | SNORD90 | G_gallus | Animals | mono_intronic | C/D | ENSGALG00010029145 | RC3H2 | protein_coding |
| ENSGALG00010029124 | SNORD36 | G_gallus | Animals | mono_intronic | C/D | ENSGALG00010028854 | RPL7A | protein_coding |
| ENSGALG00010029125 | SNORD24 | G_gallus | Animals | mono_intronic | C/D | ENSGALG00010028854 | RPL7A | protein_coding |
| ENSGALG00010029126 | SNORD36 | G_gallus | Animals | mono_intronic | C/D | ENSGALG00010028854 | RPL7A | protein_coding |
| ENSGALG00010029127 | SNORA65 | G_gallus | Animals | mono_intronic | H/ACA | ENSGALG00010027979 | RPL12 | protein_coding |
| ENSGALG00010029887 | SNORD49A | G_gallus | Animals | mono_intronic | C/D | ENSGALG00010029881 | | non_coding |
| ENSGALG00010029888 | ENSGALG00010029888 | G_gallus | Animals | mono_intronic | H/ACA | ENSGALG00010029653 | CCT6A | protein_coding |
| ENSGALG00010029889 | ENSGALG00010029889 | G_gallus | Animals | mono_intronic | H/ACA | ENSGALG00010029653 | CCT6A | protein_coding |
| ENSGALG00010029890 | ENSGALG00010029890 | G_gallus | Animals | mono_intronic | C/D | ENSGALG00010029881 | | non_coding |
| ENSGALG00010029891 | SNORD7 | G_gallus | Animals | mono_intergenic | C/D | | | intergenic |
| ENSGALG00010029892 | SNORD42 | G_gallus | Animals | mono_intronic | C/D | ENSGALG00010029218 | RPL23A | protein_coding |

| | | | | | | | | |
|---|---|---|---|---|---|---|---|---|
| ENSGALG00010029893 | SNORD4A | G_gallus | Animals | mono_intronic | C/D | ENSGALG00010029218 | RPL23A | protein_coding |
| ENSGALG00010029894 | ENSGALG00010029894 | G_gallus | Animals | mono_intronic | C/D | ENSGALG00010029402 | SMG6 | protein_coding |
| ENSGALG00010029896 | ENSGALG00010029896 | G_gallus | Animals | mono_intronic | C/D | ENSGALG00010029402 | SMG6 | protein_coding |
| ENSGALG00010029897 | U3 | G_gallus | Animals | intronic_cluster | C/D | ENSGALG00010029780 | TEX14 | protein_coding |
| ENSGALG00010029898 | U3 | G_gallus | Animals | intronic_cluster | C/D | ENSGALG00010029780 | TEX14 | protein_coding |
| ENSGALG00010029899 | U3 | G_gallus | Animals | intronic_cluster | C/D | ENSGALG00010029780 | TEX14 | protein_coding |
| ENSGALG00010030087 | ENSGALG00010030087 | G_gallus | Animals | mono_intergenic | C/D | | | intergenic |
| ENSGALG00010030088 | ENSGALG00010030088 | G_gallus | Animals | intergenic_cluster | H/ACA | | | intergenic |
| ENSGALG00010030089 | SNORD104 | G_gallus | Animals | intergenic_cluster | C/D | | | intergenic |
| snoDB0001 | SNORA77 | H_sapiens | Animals | mono_intronic | H/ACA | ENSG00000142599 | RERE | protein_coding |
| snoDB0002 | snoDB0002 | H_sapiens | Animals | mono_intergenic | H/ACA | | | intergenic |
| snoDB0003 | SNORA59A | H_sapiens | Animals | mono_intronic | H/ACA | ENSG00000048707 | VPS13D | protein_coding |
| snoDB0004 | SNORA70 | H_sapiens | Animals | mono_intergenic | H/ACA | | | intergenic |
| snoDB0006 | snoDB0006 | H_sapiens | Animals | intergenic_cluster | H/ACA | | | intergenic |
| snoDB0007 | snoDB0007 | H_sapiens | Animals | mono_intergenic | C/D | | | intergenic |
| snoDB0008 | SNORA63 | H_sapiens | Animals | mono_intergenic | H/ACA | | | intergenic |
| snoDB0009 | snoDB0009 | H_sapiens | Animals | intergenic_cluster | C/D | | | intergenic |
| snoDB0010 | SNORA26L6 | H_sapiens | Animals | mono_intergenic | H/ACA | | | intergenic |
| snoDB0011 | SNORD3G | H_sapiens | Animals | mono_intergenic | C/D | | | intergenic |
| snoDB0012 | snoDB0012 | H_sapiens | Animals | mono_intronic | H/ACA | ENSG00000203963 | C1orf141 | protein_coding |
| snoDB0013 | snoDB0013 | H_sapiens | Animals | mono_intergenic | H/ACA | | | intergenic |
| snoDB0014 | SNORA42L1 | H_sapiens | Animals | mono_intergenic | H/ACA | | | intergenic |
| snoDB0015 | AL137790.4 | H_sapiens | Animals | mono_intronic | H/ACA | ENSG00000116396 | KCNC4 | protein_coding |
| snoDB0016 | SNORA40L4 | H_sapiens | Animals | mono_intergenic | H/ACA | | | intergenic |
| snoDB0017 | snoDB0017 | H_sapiens | Animals | mono_intergenic | C/D | | | intergenic |
| snoDB0018 | SNORA58 | H_sapiens | Animals | mono_intronic | H/ACA | ENSG00000058804 | NDC1 | protein_coding |
| snoDB0019 | snoDB0019 | H_sapiens | Animals | mono_intergenic | H/ACA | | | intergenic |
| snoDB0020 | snoDB0020 | H_sapiens | Animals | mono_intergenic | H/ACA | | | intergenic |
| snoDB0021 | snoDB0021 | H_sapiens | Animals | mono_intergenic | H/ACA | | | intergenic |
| snoDB0022 | U3 | H_sapiens | Animals | mono_intergenic | C/D | | | intergenic |
| snoDB0023 | SNORD59BL1 | H_sapiens | Animals | mono_intergenic | C/D | | | intergenic |
| snoDB0024 | snoDB0024 | H_sapiens | Animals | mono_intergenic | H/ACA | | | intergenic |
| snoDB0025 | SNORA63 | H_sapiens | Animals | mono_intergenic | H/ACA | | | intergenic |
| snoDB0026 | SNORA67L4 | H_sapiens | Animals | mono_intergenic | H/ACA | | | intergenic |
| snoDB0027 | SNORA16B | H_sapiens | Animals | mono_intronic | H/ACA | ENSG00000066027 | PPP2R5A | protein_coding |
| snoDB0028 | SNORD116 | H_sapiens | Animals | mono_intronic | C/D | ENSG00000042781 | USH2A | protein_coding |
| snoDB0030 | U8 | H_sapiens | Animals | mono_intergenic | C/D | | | intergenic |
| snoDB0031 | SNORA14B | H_sapiens | Animals | mono_intronic | H/ACA | ENSG00000173726 | TOMM20 | protein_coding |
| snoDB0032 | U3 | H_sapiens | Animals | mono_intergenic | C/D | | | intergenic |
| snoDB0033 | SNORA26L8 | H_sapiens | Animals | mono_intergenic | H/ACA | | | intergenic |
| snoDB0034 | SNORA70H | H_sapiens | Animals | mono_intergenic | H/ACA | | | intergenic |
| snoDB0035 | U3 | H_sapiens | Animals | mono_intergenic | C/D | | | intergenic |
| snoDB0036 | SNORA72 | H_sapiens | Animals | mono_intronic | H/ACA | ENSG00000069275 | NUCKS1 | protein_coding |
| snoDB0037 | snoDB0037 | H_sapiens | Animals | mono_intronic | H/ACA | ENSG00000223519 | KIF28P | non_coding |
| snoDB0038 | SNORA70 | H_sapiens | Animals | mono_intergenic | H/ACA | | | intergenic |

| | | | | | | | | | |
|---|---|---|---|---|---|---|---|---|---|
| snoDB0039 | snoDB0039 | H_sapiens | Animals | mono_intergenic | H/ACA | | | | intergenic |
| snoDB0040 | SNORA36B | H_sapiens | Animals | mono_intronic | H/ACA | ENSG00000118873 | RAB3GAP2 | | protein_coding |
| snoDB0041 | SNORA58B | H_sapiens | Animals | mono_intronic | H/ACA | ENSG00000143569 | UBAP2L | | protein_coding |
| snoDB0043 | SNORD64L1 | H_sapiens | Animals | mono_intronic | C/D | ENSG00000256029 | | | protein_coding |
| snoDB0044 | U3 | H_sapiens | Animals | mono_intergenic | C/D | | | | intergenic |
| snoDB0045 | SNORA77 | H_sapiens | Animals | mono_intronic | H/ACA | ENSG00000058668 | ATP2B4 | | protein_coding |
| snoDB0047 | SNORA80E | H_sapiens | Animals | mono_intronic | H/ACA | ENSG00000132680 | KHDC4 | | protein_coding |
| snoDB0048 | snoDB0048 | H_sapiens | Animals | mono_intergenic | H/ACA | | | | intergenic |
| snoDB0049 | SNORD55 | H_sapiens | Animals | mono_intronic | C/D | ENSG00000142937 | RPS8 | | protein_coding |
| snoDB0050 | SNORD46 | H_sapiens | Animals | mono_intronic | C/D | ENSG00000142937 | RPS8 | | protein_coding |
| snoDB0051 | SNORD38A | H_sapiens | Animals | mono_intronic | C/D | ENSG00000142937 | RPS8 | | protein_coding |
| snoDB0053 | SNORD81 | H_sapiens | Animals | mono_intergenic | C/D | | | | intergenic |
| snoDB0054 | SNORA72 | H_sapiens | Animals | mono_intronic | H/ACA | ENSG00000143753 | DEGS1 | | protein_coding |
| snoDB0055 | SNORA55 | H_sapiens | Animals | mono_intronic | H/ACA | ENSG00000090621 | PABPC4 | | protein_coding |
| snoDB0056 | snoDB0056 | H_sapiens | Animals | mono_intergenic | H/ACA | | | | intergenic |
| snoDB0058 | snoDB0058 | H_sapiens | Animals | mono_intergenic | H/ACA | | | | intergenic |
| snoDB0059 | SNORD45C | H_sapiens | Animals | mono_intronic | C/D | ENSG00000137955 | RABGGTB | | protein_coding |
| snoDB0060 | SNORD45A | H_sapiens | Animals | mono_intronic | C/D | ENSG00000137955 | RABGGTB | | protein_coding |
| snoDB0061 | SNORD45B | H_sapiens | Animals | mono_intronic | C/D | ENSG00000137955 | RABGGTB | | protein_coding |
| snoDB0062 | SNORD21 | H_sapiens | Animals | mono_intronic | C/D | ENSG00000122406 | RPL5 | | protein_coding |
| snoDB0063 | SNORA66 | H_sapiens | Animals | mono_intronic | H/ACA | ENSG00000122406 | RPL5 | | protein_coding |
| snoDB0064 | SNORA51L8 | H_sapiens | Animals | mono_intergenic | H/ACA | | | | intergenic |
| snoDB0065 | snoDB0065 | H_sapiens | Animals | mono_intergenic | H/ACA | | | | intergenic |
| snoDB0066 | SNORA51L9 | H_sapiens | Animals | mono_intergenic | H/ACA | | | | intergenic |
| snoDB0067 | SNORA2BL1 | H_sapiens | Animals | mono_intergenic | H/ACA | | | | intergenic |
| snoDB0068 | snoDB0068 | H_sapiens | Animals | mono_intergenic | H/ACA | | | | intergenic |
| snoDB0069 | SNORA73A | H_sapiens | Animals | mono_intronic | H/ACA | ENSG00000180198 | RCC1 | | protein_coding |
| snoDB0070 | SNORA73B | H_sapiens | Animals | intronic_cluster | H/ACA | ENSG00000180198 | RCC1 | | protein_coding |
| snoDB0071 | SNORD99 | H_sapiens | Animals | mono_intronic | C/D | ENSG00000197989 | SNHG12 | | non_coding |
| snoDB0072 | SNORA61 | H_sapiens | Animals | mono_exonic | H/ACA | ENSG00000197989 | SNHG12 | | non_coding |
| snoDB0073 | SNORA44 | H_sapiens | Animals | mono_intronic | H/ACA | ENSG00000197989 | SNHG12 | | non_coding |
| snoDB0074 | SNORA62 | H_sapiens | Animals | mono_intronic | H/ACA | ENSG00000146463 | ZMYM4 | | protein_coding |
| snoDB0075 | snoDB0075 | H_sapiens | Animals | mono_intronic | C/D | ENSG00000263961 | RHEX | | protein_coding |
| snoDB0076 | SNORA63C | H_sapiens | Animals | mono_intronic | H/ACA | ENSG00000116885 | OSCP1 | | protein_coding |
| snoDB0077 | snoDB0077 | H_sapiens | Animals | mono_intergenic | H/ACA | | | | intergenic |
| snoDB0078 | snoDB0078 | H_sapiens | Animals | mono_intergenic | H/ACA | | | | intergenic |
| snoDB0079 | snoDB0079 | H_sapiens | Animals | mono_intergenic | C/D | | | | intergenic |
| snoDB0080 | SNORA40L7 | H_sapiens | Animals | mono_intergenic | H/ACA | | | | intergenic |
| snoDB0081 | SNORD18 | H_sapiens | Animals | mono_intergenic | C/D | | | | intergenic |
| snoDB0082 | snoDB0082 | H_sapiens | Animals | mono_intronic | H/ACA | ENSG00000198399 | ITSN2 | | protein_coding |
| snoDB0083 | SNORA36BL1 | H_sapiens | Animals | mono_intergenic | H/ACA | | | | intergenic |
| snoDB0084 | SNORA10B | H_sapiens | Animals | mono_intergenic | H/ACA | | | | intergenic |
| snoDB0085 | snoDB0085 | H_sapiens | Animals | mono_intronic | H/ACA | ENSG00000011566 | MAP4K3 | | protein_coding |
| snoDB0086 | SNORD75L2 | H_sapiens | Animals | mono_intergenic | C/D | | | | intergenic |
| snoDB0087 | SNORA12L2 | H_sapiens | Animals | mono_intergenic | H/ACA | | | | intergenic |

| | | | | | | | | |
|---|---|---|---|---|---|---|---|---|
| snoDB0088 | SNORD78L1 | H_sapiens | Animals | mono_intergenic | C/D | | | intergenic |
| snoDB0089 | SNORD92 | H_sapiens | Animals | mono_intronic | C/D | ENSG00000163811 | WDR43 | protein_coding |
| snoDB0090 | SNORD53 | H_sapiens | Animals | mono_intronic | C/D | ENSG00000163811 | WDR43 | protein_coding |
| snoDB0091 | SNORD53B | H_sapiens | Animals | mono_intronic | C/D | ENSG00000163811 | WDR43 | protein_coding |
| snoDB0092 | snoDB0092 | H_sapiens | Animals | mono_intergenic | H/ACA | | | intergenic |
| snoDB0093 | SNORA74 | H_sapiens | Animals | mono_intergenic | H/ACA | | | intergenic |
| snoDB0094 | U3 | H_sapiens | Animals | mono_intergenic | C/D | | | intergenic |
| snoDB0095 | snoDB0095 | H_sapiens | Animals | mono_intergenic | H/ACA | | | intergenic |
| snoDB0096 | SNORD78L2 | H_sapiens | Animals | mono_intergenic | C/D | | | intergenic |
| snoDB0097 | SNORA72 | H_sapiens | Animals | mono_intergenic | H/ACA | | | intergenic |
| snoDB0098 | SNORD89 | H_sapiens | Animals | mono_intronic | C/D | ENSG00000163162 | RNF149 | protein_coding |
| snoDB0099 | U3 | H_sapiens | Animals | mono_intergenic | C/D | | | intergenic |
| snoDB0100 | SNORA40B | H_sapiens | Animals | mono_intronic | H/ACA | ENSG00000115839 | RAB3GAP1 | protein_coding |
| snoDB0101 | SNORA72 | H_sapiens | Animals | mono_intergenic | H/ACA | | | intergenic |
| snoDB0102 | SNORD56L7 | H_sapiens | Animals | mono_intronic | C/D | ENSG00000227400 | | non_coding |
| snoDB0103 | snoDB0103 | H_sapiens | Animals | mono_intronic | C/D | ENSG00000144290 | SLC4A10 | protein_coding |
| snoDB0104 | SNORA77 | H_sapiens | Animals | mono_intergenic | H/ACA | | | intergenic |
| snoDB0105 | snoDB0105 | H_sapiens | Animals | mono_intergenic | C/D | | | intergenic |
| snoDB0106 | SNORD59BL2 | H_sapiens | Animals | mono_intergenic | C/D | | | intergenic |
| snoDB0107 | SNORD70 | H_sapiens | Animals | mono_intronic | C/D | ENSG00000055044 | NOP58 | protein_coding |
| snoDB0108 | SNORD11B | H_sapiens | Animals | mono_intronic | C/D | ENSG00000055044 | NOP58 | protein_coding |
| snoDB0109 | SNORD11 | H_sapiens | Animals | mono_intronic | C/D | ENSG00000055044 | NOP58 | protein_coding |
| snoDB0110 | SNORA41 | H_sapiens | Animals | mono_intronic | H/ACA | ENSG00000114942 | EEF1B2 | protein_coding |
| snoDB0111 | SNORD70B | H_sapiens | Animals | mono_intronic | C/D | ENSG00000055044 | NOP58 | protein_coding |
| snoDB0112 | U8 | H_sapiens | Animals | mono_intergenic | C/D | | | intergenic |
| snoDB0113 | SNORA70I | H_sapiens | Animals | mono_intronic | H/ACA | ENSG00000229267 | SNHG31 | non_coding |
| snoDB0116 | SNORD94 | H_sapiens | Animals | mono_intronic | C/D | ENSG00000132300 | PTCD3 | protein_coding |
| snoDB0117 | U8 | H_sapiens | Animals | mono_intergenic | C/D | | | intergenic |
| snoDB0118 | snoDB0118 | H_sapiens | Animals | mono_intergenic | H/ACA | | | intergenic |
| snoDB0119 | SNORA75 | H_sapiens | Animals | mono_intronic | H/ACA | ENSG00000115053 | NCL | protein_coding |
| snoDB0120 | SNORD20 | H_sapiens | Animals | mono_intronic | C/D | ENSG00000115053 | NCL | protein_coding |
| snoDB0121 | SNORD82 | H_sapiens | Animals | mono_intronic | C/D | ENSG00000115053 | NCL | protein_coding |
| snoDB0122 | SNORA75 | H_sapiens | Animals | mono_intergenic | H/ACA | | | intergenic |
| snoDB0123 | snoDB0123 | H_sapiens | Animals | mono_intronic | H/ACA | ENSG00000123977 | DAW1 | protein_coding |
| snoDB0124 | SNORA48L7 | H_sapiens | Animals | mono_intergenic | H/ACA | | | intergenic |
| snoDB0125 | snoDB0125 | H_sapiens | Animals | mono_intergenic | H/ACA | | | intergenic |
| snoDB0126 | snoDB0126 | H_sapiens | Animals | mono_intergenic | H/ACA | | | intergenic |
| snoDB0127 | SNORA4L2 | H_sapiens | Animals | mono_intergenic | H/ACA | | | intergenic |
| snoDB0128 | SNORA51 | H_sapiens | Animals | mono_intronic | H/ACA | ENSG00000213160 | KLHL23 | protein_coding |
| snoDB0129 | snoDB0129 | H_sapiens | Animals | mono_intronic | C/D | ENSG00000116044 | NFE2L2 | protein_coding |
| snoDB0130 | U3 | H_sapiens | Animals | mono_intergenic | C/D | | | intergenic |
| snoDB0131 | SNORA26L10 | H_sapiens | Animals | mono_intergenic | H/ACA | | | intergenic |
| snoDB0132 | SNORA2AL2 | H_sapiens | Animals | mono_intergenic | H/ACA | | | intergenic |
| snoDB0133 | SNORA51L10 | H_sapiens | Animals | mono_intronic | H/ACA | ENSG00000285872 | | non_coding |
| snoDB0134 | SNORA80B | H_sapiens | Animals | mono_intronic | H/ACA | ENSG00000115758 | ODC1 | protein_coding |

| | | | | | | | | |
|---|---|---|---|---|---|---|---|---|
| snoDB0135 | SNORA70B | H_sapiens | Animals | mono_intronic | H/ACA | ENSG00000115464 | USP34 | protein_coding |
| snoDB0136 | SNORA36C | H_sapiens | Animals | mono_intronic | H/ACA | ENSG00000115977 | AAK1 | protein_coding |
| snoDB0137 | SNORD39 | H_sapiens | Animals | mono_intronic | C/D | ENSG00000132329 | RAMP1 | protein_coding |
| snoDB0138 | SNORA70F | H_sapiens | Animals | mono_intronic | H/ACA | ENSG00000082438 | COBLL1 | protein_coding |
| snoDB0139 | SNORD14 | H_sapiens | Animals | mono_intronic | C/D | ENSG00000115137 | DNAJC27 | protein_coding |
| snoDB0140 | SNORA1L4 | H_sapiens | Animals | mono_intergenic | H/ACA | | | intergenic |
| snoDB0141 | SNORA48L8 | H_sapiens | Animals | mono_intergenic | H/ACA | | | intergenic |
| snoDB0143 | SNORA6 | H_sapiens | Animals | mono_intronic | H/ACA | ENSG00000168028 | RPSA | protein_coding |
| snoDB0144 | SNORA62 | H_sapiens | Animals | mono_intronic | H/ACA | ENSG00000168028 | RPSA | protein_coding |
| snoDB0145 | U3 | H_sapiens | Animals | mono_intergenic | C/D | | | intergenic |
| snoDB0146 | snoDB0146 | H_sapiens | Animals | mono_intronic | H/ACA | ENSG00000168038 | ULK4 | protein_coding |
| snoDB0147 | SNORA25L5 | H_sapiens | Animals | mono_intergenic | H/ACA | | | intergenic |
| snoDB0148 | U8 | H_sapiens | Animals | mono_intronic | C/D | ENSG00000168038 | ULK4 | protein_coding |
| snoDB0149 | SNORD19 | H_sapiens | Animals | mono_intronic | C/D | ENSG00000163938 | GNL3 | protein_coding |
| snoDB0150 | SNORD19C | H_sapiens | Animals | mono_intronic | C/D | ENSG00000163938 | GNL3 | protein_coding |
| snoDB0151 | SNORD69 | H_sapiens | Animals | mono_intronic | C/D | ENSG00000163938 | GNL3 | protein_coding |
| snoDB0152 | U3 | H_sapiens | Animals | mono_intergenic | C/D | | | intergenic |
| snoDB0153 | U3 | H_sapiens | Animals | mono_intronic | C/D | ENSG00000189283 | FHIT | protein_coding |
| snoDB0154 | SNORA64 | H_sapiens | Animals | mono_intronic | H/ACA | ENSG00000170011 | MYRIP | protein_coding |
| snoDB0155 | U3 | H_sapiens | Animals | mono_intergenic | C/D | | | intergenic |
| snoDB0156 | SNORA70 | H_sapiens | Animals | mono_intergenic | H/ACA | | | intergenic |
| snoDB0157 | U3 | H_sapiens | Animals | mono_intronic | C/D | ENSG00000114487 | MORC1 | protein_coding |
| snoDB0158 | SNORA58 | H_sapiens | Animals | mono_intronic | H/ACA | ENSG00000114686 | MRPL3 | protein_coding |
| snoDB0159 | snoDB0159 | H_sapiens | Animals | mono_intergenic | H/ACA | | | intergenic |
| snoDB0160 | SNORD19B | H_sapiens | Animals | mono_intronic | C/D | ENSG00000163938 | GNL3 | protein_coding |
| snoDB0161 | snoDB0161 | H_sapiens | Animals | mono_intergenic | C/D | | | intergenic |
| snoDB0162 | U8 | H_sapiens | Animals | mono_intergenic | C/D | | | intergenic |
| snoDB0163 | SNORA18L3 | H_sapiens | Animals | mono_intronic | H/ACA | ENSG00000236385 | | non_coding |
| snoDB0164 | snoDB0164 | H_sapiens | Animals | mono_intergenic | C/D | | | intergenic |
| snoDB0165 | SNORA63D | H_sapiens | Animals | mono_intronic | H/ACA | ENSG00000240024 | LINC00888 | non_coding |
| snoDB0166 | SNORA72 | H_sapiens | Animals | mono_intergenic | H/ACA | | | intergenic |
| snoDB0167 | snoDB0167 | H_sapiens | Animals | intronic_cluster | H/ACA | ENSG00000240024 | LINC00888 | non_coding |
| snoDB0168 | snoDB0168 | H_sapiens | Animals | mono_intronic | H/ACA | ENSG00000240024 | LINC00888 | non_coding |
| snoDB0169 | SNORA63E | H_sapiens | Animals | intronic_cluster | H/ACA | ENSG00000240024 | LINC00888 | non_coding |
| snoDB0170 | U8 | H_sapiens | Animals | mono_intergenic | C/D | | | intergenic |
| snoDB0171 | snoDB0171 | H_sapiens | Animals | mono_intronic | H/ACA | ENSG00000144820 | ADGRG7 | protein_coding |
| snoDB0172 | SNORA24L2 | H_sapiens | Animals | mono_intergenic | H/ACA | | | intergenic |
| snoDB0173 | SNORA5AL1 | H_sapiens | Animals | mono_intergenic | H/ACA | | | intergenic |
| snoDB0174 | snoDB0174 | H_sapiens | Animals | mono_intergenic | C/D | | | intergenic |
| snoDB0175 | SNORD66 | H_sapiens | Animals | mono_intronic | C/D | ENSG00000114867 | EIF4G1 | protein_coding |
| snoDB0176 | snoDB0176 | H_sapiens | Animals | mono_intergenic | C/D | | | intergenic |
| snoDB0177 | snoDB0177 | H_sapiens | Animals | mono_intergenic | C/D | | | intergenic |
| snoDB0178 | snoDB0178 | H_sapiens | Animals | mono_intergenic | H/ACA | | | intergenic |
| snoDB0179 | SNORD13P3 | H_sapiens | Animals | mono_intronic | C/D | ENSG00000088727 | KIF9 | protein_coding |
| snoDB0180 | SNORD2 | H_sapiens | Animals | mono_intronic | C/D | ENSG00000156976 | EIF4A2 | protein_coding |

| | | | | | | | | |
|---|---|---|---|---|---|---|---|---|
| snoDB0181 | SNORA63B | H_sapiens | Animals | mono_intronic | H/ACA | ENSG00000156976 | EIF4A2 | protein_coding |
| snoDB0182 | SNORA81 | H_sapiens | Animals | mono_intronic | H/ACA | ENSG00000156976 | EIF4A2 | protein_coding |
| snoDB0183 | SNORA63 | H_sapiens | Animals | mono_intronic | H/ACA | ENSG00000156976 | EIF4A2 | protein_coding |
| snoDB0184 | SNORA4 | H_sapiens | Animals | mono_intronic | H/ACA | ENSG00000156976 | EIF4A2 | protein_coding |
| snoDB0186 | SNORA72 | H_sapiens | Animals | mono_intergenic | H/ACA | | | intergenic |
| snoDB0187 | SNORD6L1 | H_sapiens | Animals | mono_intergenic | C/D | | | intergenic |
| snoDB0188 | SNORA7A | H_sapiens | Animals | mono_intronic | H/ACA | ENSG00000144713 | RPL32 | protein_coding |
| snoDB0189 | snoDB0189 | H_sapiens | Animals | mono_intergenic | H/ACA | | | intergenic |
| snoDB0190 | snoDB0190 | H_sapiens | Animals | mono_intronic | C/D | ENSG00000114279 | FGF12 | protein_coding |
| snoDB0191 | SNORD38C | H_sapiens | Animals | mono_intronic | C/D | ENSG00000272886 | DCP1A | protein_coding |
| snoDB0192 | SNORA26L4 | H_sapiens | Animals | mono_intergenic | H/ACA | | | intergenic |
| snoDB0193 | SNORD63 | H_sapiens | Animals | mono_intergenic | C/D | | | intergenic |
| snoDB0194 | SNORA7B | H_sapiens | Animals | mono_intronic | H/ACA | ENSG00000290993 | | non_coding |
| snoDB0195 | SNORA48L2 | H_sapiens | Animals | mono_intronic | H/ACA | ENSG00000251652 | | non_coding |
| snoDB0196 | SNORA63 | H_sapiens | Animals | mono_intergenic | H/ACA | | | intergenic |
| snoDB0197 | SNORA75B | H_sapiens | Animals | mono_intergenic | H/ACA | | | intergenic |
| snoDB0198 | SNORD74L7 | H_sapiens | Animals | mono_intronic | C/D | ENSG00000109680 | TBC1D19 | protein_coding |
| snoDB0199 | snoDB0199 | H_sapiens | Animals | mono_intergenic | C/D | | | intergenic |
| snoDB0200 | snoDB0200 | H_sapiens | Animals | mono_intronic | H/ACA | ENSG00000078177 | N4BP2 | protein_coding |
| snoDB0201 | SNORA62 | H_sapiens | Animals | mono_intronic | H/ACA | ENSG00000248049 | UBA6-DT | non_coding |
| snoDB0202 | SNORA45 | H_sapiens | Animals | mono_intronic | H/ACA | ENSG00000250220 | ANKRD17-DT | non_coding |
| snoDB0203 | snoDB0203 | H_sapiens | Animals | mono_intergenic | H/ACA | | | intergenic |
| snoDB0204 | SNORD42 | H_sapiens | Animals | mono_intergenic | C/D | | | intergenic |
| snoDB0205 | SNORD75L1 | H_sapiens | Animals | mono_intergenic | C/D | | | intergenic |
| snoDB0206 | SNORA24 | H_sapiens | Animals | mono_intronic | H/ACA | ENSG00000269893 | SNHG8 | non_coding |
| snoDB0207 | SNORA70 | H_sapiens | Animals | mono_intronic | H/ACA | ENSG00000251676 | SNHG27 | non_coding |
| snoDB0208 | SNORA11BL1 | H_sapiens | Animals | mono_intergenic | H/ACA | | | intergenic |
| snoDB0209 | SNORD73B | H_sapiens | Animals | mono_intronic | C/D | ENSG00000145425 | RPS3A | protein_coding |
| snoDB0210 | SNORD73A | H_sapiens | Animals | mono_intronic | C/D | ENSG00000145425 | RPS3A | protein_coding |
| snoDB0211 | SNORD39 | H_sapiens | Animals | mono_intronic | C/D | ENSG00000164123 | C4orf45 | protein_coding |
| snoDB0212 | SNORD79 | H_sapiens | Animals | mono_intergenic | C/D | | | intergenic |
| snoDB0213 | SNORA51 | H_sapiens | Animals | mono_intronic | H/ACA | ENSG00000150627 | WDR17 | protein_coding |
| snoDB0214 | snoDB0214 | H_sapiens | Animals | mono_intronic | H/ACA | ENSG00000109794 | FAM149A | protein_coding |
| snoDB0215 | SNORD65L1 | H_sapiens | Animals | mono_intergenic | C/D | | | intergenic |
| snoDB0216 | SNORA26 | H_sapiens | Animals | mono_intronic | H/ACA | ENSG00000226950 | DANCR | non_coding |
| snoDB0217 | SNORA26L2 | H_sapiens | Animals | mono_intronic | H/ACA | ENSG00000250302 | LINC01618 | non_coding |
| snoDB0218 | snoDB0218 | H_sapiens | Animals | mono_intronic | H/ACA | ENSG00000273247 | RAB33B-AS1 | non_coding |
| snoDB0219 | SNORA51L5 | H_sapiens | Animals | mono_intergenic | H/ACA | | | intergenic |
| snoDB0220 | snoDB0220 | H_sapiens | Animals | mono_intronic | H/ACA | ENSG00000248373 | | non_coding |
| snoDB0222 | snoDB0222 | H_sapiens | Animals | mono_intergenic | C/D | | | intergenic |
| snoDB0223 | SNORA75 | H_sapiens | Animals | mono_intronic | H/ACA | ENSG00000251321 | PCAT4 | non_coding |
| snoDB0224 | U3 | H_sapiens | Animals | mono_intronic | C/D | ENSG00000171503 | ETFDH | protein_coding |
| snoDB0225 | SNORD50AL1 | H_sapiens | Animals | mono_intergenic | C/D | | | intergenic |
| snoDB0226 | SNORD123 | H_sapiens | Animals | mono_intronic | C/D | ENSG00000250786 | SNHG18 | non_coding |
| snoDB0227 | U8 | H_sapiens | Animals | mono_intergenic | C/D | | | intergenic |

| | | | | | | | | |
|---|---|---|---|---|---|---|---|---|
| snoDB0228 | SNORD81 | H_sapiens | Animals | mono_intergenic | C/D | | | intergenic |
| snoDB0229 | snoDB0229 | H_sapiens | Animals | mono_intergenic | H/ACA | | | intergenic |
| snoDB0230 | SNORA18L1 | H_sapiens | Animals | mono_intronic | H/ACA | ENSG00000259786 | LINC02109 | non_coding |
| snoDB0231 | SNORD29 | H_sapiens | Animals | mono_intergenic | C/D | | | intergenic |
| snoDB0232 | U3 | H_sapiens | Animals | mono_intergenic | C/D | | | intergenic |
| snoDB0233 | SNORA57 | H_sapiens | Animals | mono_intronic | H/ACA | ENSG00000132356 | PRKAA1 | protein_coding |
| snoDB0234 | SNORA63 | H_sapiens | Animals | mono_intergenic | H/ACA | | | intergenic |
| snoDB0235 | SNORD72 | H_sapiens | Animals | mono_intronic | C/D | ENSG00000145592 | RPL37 | protein_coding |
| snoDB0236 | snoDB0236 | H_sapiens | Animals | mono_intergenic | C/D | | | intergenic |
| snoDB0237 | U8 | H_sapiens | Animals | mono_intergenic | C/D | | | intergenic |
| snoDB0238 | snoDB0238 | H_sapiens | Animals | mono_intergenic | H/ACA | | | intergenic |
| snoDB0239 | snoDB0239 | H_sapiens | Animals | mono_intergenic | H/ACA | | | intergenic |
| snoDB0240 | snoDB0240 | H_sapiens | Animals | mono_intergenic | H/ACA | | | intergenic |
| snoDB0242 | SNORA18L2 | H_sapiens | Animals | mono_intergenic | H/ACA | | | intergenic |
| snoDB0243 | SNORD138 | H_sapiens | Animals | mono_intronic | C/D | ENSG00000127184 | COX7C | protein_coding |
| snoDB0244 | SNORA70 | H_sapiens | Animals | mono_intronic | H/ACA | ENSG00000247828 | TMEM161B-DT | non_coding |
| snoDB0245 | SNORA47 | H_sapiens | Animals | mono_intronic | H/ACA | ENSG00000132846 | ZBED3 | protein_coding |
| snoDB0246 | SNORA13 | H_sapiens | Animals | mono_intronic | H/ACA | ENSG00000224032 | EPB41L4A-AS1 | non_coding |
| snoDB0247 | SNORA51 | H_sapiens | Animals | mono_intronic | H/ACA | ENSG00000186952 | TMEM232 | protein_coding |
| snoDB0248 | snoDB0248 | H_sapiens | Animals | mono_intergenic | H/ACA | | | intergenic |
| snoDB0249 | U3 | H_sapiens | Animals | mono_intergenic | C/D | | | intergenic |
| snoDB0250 | snoDB0250 | H_sapiens | Animals | mono_intronic | H/ACA | ENSG00000253256 | | non_coding |
| snoDB0251 | snoDB0251 | H_sapiens | Animals | mono_intergenic | H/ACA | | | intergenic |
| snoDB0252 | snoDB0252 | H_sapiens | Animals | mono_intronic | H/ACA | ENSG00000168246 | UBTD2 | protein_coding |
| snoDB0253 | SNORA74B | H_sapiens | Animals | mono_intronic | H/ACA | ENSG00000113732 | ATP6V0E1 | protein_coding |
| snoDB0254 | SNORD96A | H_sapiens | Animals | mono_intronic | C/D | ENSG00000204628 | RACK1 | protein_coding |
| snoDB0255 | SNORD95 | H_sapiens | Animals | mono_intronic | C/D | ENSG00000204628 | RACK1 | protein_coding |
| snoDB0256 | SNORA74D | H_sapiens | Animals | mono_intronic | H/ACA | ENSG00000280987 | MATR3 | protein_coding |
| snoDB0257 | SNORA74A | H_sapiens | Animals | mono_intronic | H/ACA | ENSG00000280987 | MATR3 | protein_coding |
| snoDB0258 | SNORD45CL1 | H_sapiens | Animals | mono_intergenic | C/D | | | intergenic |
| snoDB0259 | SNORA27L1 | H_sapiens | Animals | mono_intergenic | H/ACA | | | intergenic |
| snoDB0260 | SNORA70J | H_sapiens | Animals | mono_intronic | H/ACA | ENSG00000254256 | RPSAP71 | non_coding |
| snoDB0261 | SNORD63B | H_sapiens | Animals | mono_intronic | C/D | ENSG00000113013 | HSPA9 | protein_coding |
| snoDB0262 | SNORD63 | H_sapiens | Animals | mono_intronic | C/D | ENSG00000113013 | HSPA9 | protein_coding |
| snoDB0263 | snoDB0263 | H_sapiens | Animals | mono_intronic | C/D | ENSG00000044115 | CTNNA1 | protein_coding |
| snoDB0264 | snoDB0264 | H_sapiens | Animals | mono_intergenic | C/D | | | intergenic |
| snoDB0265 | SNORA67L6 | H_sapiens | Animals | mono_intergenic | H/ACA | | | intergenic |
| snoDB0266 | snoDB0266 | H_sapiens | Animals | mono_intergenic | C/D | | | intergenic |
| snoDB0267 | snoDB0267 | H_sapiens | Animals | mono_intergenic | H/ACA | | | intergenic |
| snoDB0268 | SNORD45BL3 | H_sapiens | Animals | mono_intergenic | C/D | | | intergenic |
| snoDB0269 | SNORD66L2 | H_sapiens | Animals | mono_intergenic | C/D | | | intergenic |
| snoDB0270 | SNORD65L4 | H_sapiens | Animals | mono_intergenic | C/D | | | intergenic |
| snoDB0271 | U3 | H_sapiens | Animals | mono_intergenic | C/D | | | intergenic |
| snoDB0272 | SNORA70 | H_sapiens | Animals | mono_intergenic | H/ACA | | | intergenic |
| snoDB0273 | SNORA18L7 | H_sapiens | Animals | mono_intergenic | H/ACA | | | intergenic |

| | | | | | | | | |
|---|---|---|---|---|---|---|---|---|
| snoDB0274 | SNORA33L2 | H_sapiens | Animals | mono_intergenic | H/ACA | | | intergenic |
| snoDB0275 | SNORA73 | H_sapiens | Animals | mono_intergenic | H/ACA | | | intergenic |
| snoDB0276 | U3 | H_sapiens | Animals | mono_intergenic | C/D | | | intergenic |
| snoDB0277 | U3 | H_sapiens | Animals | mono_intergenic | C/D | | | intergenic |
| snoDB0278 | SNORD101 | H_sapiens | Animals | mono_intronic | C/D | ENSG00000112306 | RPS12 | protein_coding |
| snoDB0279 | SNORD100 | H_sapiens | Animals | mono_intronic | C/D | ENSG00000112306 | RPS12 | protein_coding |
| snoDB0280 | SNORA33 | H_sapiens | Animals | mono_intronic | H/ACA | ENSG00000112306 | RPS12 | protein_coding |
| snoDB0281 | snoDB0281 | H_sapiens | Animals | mono_intronic | C/D | ENSG00000198729 | PPP1R14C | protein_coding |
| snoDB0282 | SNORD28B | H_sapiens | Animals | mono_intronic | C/D | ENSG00000285797 | | non_coding |
| snoDB0283 | SNORA2AL1 | H_sapiens | Animals | mono_intronic | H/ACA | ENSG00000220848 | RPS18P9 | non_coding |
| snoDB0284 | U3 | H_sapiens | Animals | mono_intergenic | C/D | | | intergenic |
| snoDB0285 | SNORD32B | H_sapiens | Animals | mono_intergenic | C/D | | | intergenic |
| snoDB0286 | SNORA20 | H_sapiens | Animals | mono_intronic | H/ACA | ENSG00000120438 | TCP1 | protein_coding |
| snoDB0287 | SNORA29 | H_sapiens | Animals | mono_intronic | H/ACA | ENSG00000120438 | TCP1 | protein_coding |
| snoDB0288 | SNORA40C | H_sapiens | Animals | mono_intergenic | H/ACA | | | intergenic |
| snoDB0289 | U8 | H_sapiens | Animals | mono_intergenic | C/D | | | intergenic |
| snoDB0290 | SNORD45BL1 | H_sapiens | Animals | mono_exonic | C/D | ENSG00000231297 | | non_coding |
| snoDB0291 | SNORD117 | H_sapiens | Animals | mono_intronic | C/D | ENSG00000198563 | DDX39B | protein_coding |
| snoDB0292 | SNORD84 | H_sapiens | Animals | mono_intronic | C/D | ENSG00000198563 | DDX39B | protein_coding |
| snoDB0293 | SNORA38 | H_sapiens | Animals | mono_intronic | H/ACA | ENSG00000204469 | PRRC2A | protein_coding |
| snoDB0294 | SNORA27L3 | H_sapiens | Animals | mono_intergenic | H/ACA | | | intergenic |
| snoDB0295 | snoDB0295 | H_sapiens | Animals | mono_intronic | H/ACA | ENSG00000171453 | POLR1C | protein_coding |
| snoDB0296 | SNORA8@ | H_sapiens | Animals | mono_intergenic | H/ACA | | | intergenic |
| snoDB0297 | SNORD48 | H_sapiens | Animals | mono_intronic | C/D | ENSG00000204387 | SNHG32 | non_coding |
| snoDB0298 | SNORD52 | H_sapiens | Animals | mono_intronic | C/D | ENSG00000204387 | SNHG32 | non_coding |
| snoDB0299 | snoDB0299 | H_sapiens | Animals | mono_intergenic | C/D | | | intergenic |
| snoDB0300 | SNORA73 | H_sapiens | Animals | mono_intronic | H/ACA | ENSG00000111880 | RNGTT | protein_coding |
| snoDB0301 | snoDB0301 | H_sapiens | Animals | mono_intergenic | H/ACA | | | intergenic |
| snoDB0302 | U3 | H_sapiens | Animals | mono_intronic | C/D | ENSG00000118407 | FILIP1 | protein_coding |
| snoDB0303 | SNORA40L9 | H_sapiens | Animals | mono_exonic | H/ACA | ENSG00000287458 | | non_coding |
| snoDB0304 | SNORD50B | H_sapiens | Animals | mono_intronic | C/D | ENSG00000271793 | | protein_coding |
| snoDB0305 | SNORA80D | H_sapiens | Animals | mono_intronic | H/ACA | ENSG00000106305 | AIMP2 | protein_coding |
| snoDB0306 | U3 | H_sapiens | Animals | mono_intergenic | C/D | | | intergenic |
| snoDB0307 | SNORA64L2 | H_sapiens | Animals | mono_intergenic | H/ACA | | | intergenic |
| snoDB0308 | SNORA63 | H_sapiens | Animals | mono_intergenic | H/ACA | | | intergenic |
| snoDB0309 | SNORD56L2 | H_sapiens | Animals | mono_intronic | C/D | ENSG00000105855 | ITGB8 | protein_coding |
| snoDB0310 | SNORD65C | H_sapiens | Animals | mono_intergenic | C/D | | | intergenic |
| snoDB0311 | U3 | H_sapiens | Animals | mono_intergenic | C/D | | | intergenic |
| snoDB0312 | snoDB0312 | H_sapiens | Animals | mono_intergenic | H/ACA | | | intergenic |
| snoDB0313 | SNORA20B | H_sapiens | Animals | mono_intergenic | H/ACA | | | intergenic |
| snoDB0314 | SNORA51L6 | H_sapiens | Animals | mono_intergenic | H/ACA | | | intergenic |
| snoDB0315 | snoDB0315 | H_sapiens | Animals | mono_intergenic | H/ACA | | | intergenic |
| snoDB0316 | SNORA5A | H_sapiens | Animals | mono_intronic | H/ACA | ENSG00000136270 | TBRG4 | protein_coding |
| snoDB0317 | SNORA5C | H_sapiens | Animals | mono_intronic | H/ACA | ENSG00000136270 | TBRG4 | protein_coding |
| snoDB0318 | SNORA5B | H_sapiens | Animals | mono_intronic | H/ACA | ENSG00000136270 | TBRG4 | protein_coding |

| | | | | | | | | |
|---|---|---|---|---|---|---|---|---|
| snoDB0319 | SNORA9 | H_sapiens | Animals | mono_intronic | H/ACA | ENSG00000232956 | SNHG15 | non_coding |
| snoDB0320 | SNORA4L1 | H_sapiens | Animals | mono_intergenic | H/ACA | | | intergenic |
| snoDB0321 | SNORA22B | H_sapiens | Animals | mono_intronic | H/ACA | ENSG00000146731 | CCT6A | protein_coding |
| snoDB0322 | SNORA15 | H_sapiens | Animals | mono_intronic | H/ACA | ENSG00000146731 | CCT6A | protein_coding |
| snoDB0323 | snoDB0323 | H_sapiens | Animals | mono_intergenic | H/ACA | | | intergenic |
| snoDB0324 | snoDB0324 | H_sapiens | Animals | mono_intergenic | C/D | | | intergenic |
| snoDB0325 | SNORA22 | H_sapiens | Animals | mono_intronic | H/ACA | ENSG00000228409 | CCT6P1 | non_coding |
| snoDB0326 | SNORA15B-2 | H_sapiens | Animals | mono_intronic | H/ACA | ENSG00000228409 | CCT6P1 | non_coding |
| snoDB0327 | SNORA22C | H_sapiens | Animals | mono_intronic | H/ACA | ENSG00000234585 | CCT6P3 | non_coding |
| snoDB0328 | SNORA15B-1 | H_sapiens | Animals | mono_intronic | H/ACA | ENSG00000234585 | CCT6P3 | non_coding |
| snoDB0329 | SNORA14A | H_sapiens | Animals | mono_intronic | H/ACA | ENSG00000127948 | POR | protein_coding |
| snoDB0330 | SNORA67L2 | H_sapiens | Animals | mono_intergenic | H/ACA | | | intergenic |
| snoDB0331 | snoDB0331 | H_sapiens | Animals | mono_intergenic | H/ACA | | | intergenic |
| snoDB0332 | SNORA25B | H_sapiens | Animals | mono_intergenic | H/ACA | | | intergenic |
| snoDB0333 | snoDB0333 | H_sapiens | Animals | mono_intronic | H/ACA | ENSG00000288558 | DUS4L-BCAP29 | protein_coding |
| snoDB0334 | snoDB0334 | H_sapiens | Animals | mono_intergenic | H/ACA | | | intergenic |
| snoDB0335 | SNORA51L7 | H_sapiens | Animals | mono_intergenic | H/ACA | | | intergenic |
| snoDB0336 | snoDB0336 | H_sapiens | Animals | mono_intronic | H/ACA | ENSG00000157703 | SVOPL | protein_coding |
| snoDB0337 | SNORA26L5 | H_sapiens | Animals | mono_intergenic | H/ACA | | | intergenic |
| snoDB0338 | U3 | H_sapiens | Animals | mono_intronic | C/D | ENSG00000174469 | CNTNAP2 | protein_coding |
| snoDB0339 | U3 | H_sapiens | Animals | mono_intronic | C/D | ENSG00000091136 | LAMB1 | protein_coding |
| snoDB0340 | snoDB0340 | H_sapiens | Animals | mono_intronic | C/D | ENSG00000122574 | WIPF3 | protein_coding |
| snoDB0341 | snoDB0341 | H_sapiens | Animals | mono_intergenic | C/D | | | intergenic |
| snoDB0342 | SNORD93 | H_sapiens | Animals | mono_intronic | C/D | ENSG00000228649 | SNHG26 | non_coding |
| snoDB0343 | SNORD81 | H_sapiens | Animals | mono_intergenic | C/D | | | intergenic |
| snoDB0344 | snoDB0344 | H_sapiens | Animals | mono_intronic | H/ACA | ENSG00000146938 | NLGN4X | protein_coding |
| snoDB0345 | SNORA48B | H_sapiens | Animals | mono_intergenic | H/ACA | | | intergenic |
| snoDB0346 | SNORA20L2 | H_sapiens | Animals | mono_intergenic | H/ACA | | | intergenic |
| snoDB0347 | SNORA16AL1 | H_sapiens | Animals | mono_intergenic | H/ACA | | | intergenic |
| snoDB0350 | snoDB0350 | H_sapiens | Animals | mono_intronic | C/D | ENSG00000169306 | IL1RAPL1 | protein_coding |
| snoDB0351 | SNORA68L3 | H_sapiens | Animals | mono_intergenic | H/ACA | | | intergenic |
| snoDB0352 | snoDB0352 | H_sapiens | Animals | mono_intronic | H/ACA | ENSG00000156313 | RPGR | protein_coding |
| snoDB0353 | SNORD77L1 | H_sapiens | Animals | mono_intergenic | C/D | | | intergenic |
| snoDB0354 | SNORA11 | H_sapiens | Animals | mono_intronic | H/ACA | ENSG00000102316 | MAGED2 | protein_coding |
| snoDB0355 | SNORA11G | H_sapiens | Animals | mono_intronic | H/ACA | ENSG00000067445 | TRO | protein_coding |
| snoDB0356 | U3 | H_sapiens | Animals | intronic_cluster | C/D | ENSG00000158813 | EDA | protein_coding |
| snoDB0357 | U3 | H_sapiens | Animals | mono_intergenic | C/D | | | intergenic |
| snoDB0358 | SNORA4L1 | H_sapiens | Animals | mono_intergenic | H/ACA | | | intergenic |
| snoDB0359 | SNORD45BL2 | H_sapiens | Animals | mono_intergenic | C/D | | | intergenic |
| snoDB0360 | snoDB0360 | H_sapiens | Animals | mono_intergenic | H/ACA | | | intergenic |
| snoDB0361 | snoDB0361 | H_sapiens | Animals | mono_intergenic | H/ACA | | | intergenic |
| snoDB0362 | snoDB0362 | H_sapiens | Animals | mono_intergenic | H/ACA | | | intergenic |
| snoDB0363 | SNORA35B | H_sapiens | Animals | mono_intronic | H/ACA | ENSG00000130224 | LRCH2 | protein_coding |
| snoDB0364 | SNORA35 | H_sapiens | Animals | mono_intronic | H/ACA | ENSG00000147246 | HTR2C | protein_coding |
| snoDB0365 | snoDB0365 | H_sapiens | Animals | mono_intergenic | H/ACA | | | intergenic |

| | | | | | | | | |
|---|---|---|---|---|---|---|---|---|
| snoDB0366 | snoDB0366 | H_sapiens | Animals | mono_intronic | H/ACA | ENSG00000131724 | IL13RA1 | protein_coding |
| snoDB0367 | U3 | H_sapiens | Animals | mono_intergenic | C/D | | | intergenic |
| snoDB0368 | snoDB0368 | H_sapiens | Animals | intronic_cluster | H/ACA | ENSG00000101972 | STAG2 | protein_coding |
| snoDB0369 | snoDB0369 | H_sapiens | Animals | mono_intergenic | C/D | | | intergenic |
| snoDB0370 | SNORA8 | H_sapiens | Animals | mono_intergenic | H/ACA | | | intergenic |
| snoDB0371 | SNORA11C | H_sapiens | Animals | mono_intronic | H/ACA | ENSG00000147117 | ZNF157 | protein_coding |
| snoDB0372 | SNORA70 | H_sapiens | Animals | mono_intronic | H/ACA | ENSG00000147403 | RPL10 | protein_coding |
| snoDB0373 | U3 | H_sapiens | Animals | mono_intronic | C/D | ENSG00000241743 | XACT | non_coding |
| snoDB0374 | SNORD30 | H_sapiens | Animals | mono_intergenic | C/D | | | intergenic |
| snoDB0375 | snoDB0375 | H_sapiens | Animals | mono_intergenic | C/D | | | intergenic |
| snoDB0376 | snoDB0376 | H_sapiens | Animals | mono_intergenic | C/D | | | intergenic |
| snoDB0377 | SNORD61 | H_sapiens | Animals | mono_intronic | C/D | ENSG00000147274 | RBMX | protein_coding |
| snoDB0378 | SNORA9 | H_sapiens | Animals | mono_intergenic | H/ACA | | | intergenic |
| snoDB0379 | SNORA7B | H_sapiens | Animals | intronic_cluster | H/ACA | ENSG00000169239 | CA5B | protein_coding |
| snoDB0381 | SNORD96B | H_sapiens | Animals | mono_intronic | C/D | ENSG00000101935 | AMMECR1 | protein_coding |
| snoDB0382 | SNORA69 | H_sapiens | Animals | mono_intronic | H/ACA | ENSG00000198918 | RPL39 | protein_coding |
| snoDB0383 | SNORA36A | H_sapiens | Animals | mono_intronic | H/ACA | ENSG00000130826 | DKC1 | protein_coding |
| snoDB0384 | SNORA56 | H_sapiens | Animals | mono_intronic | H/ACA | ENSG00000130826 | DKC1 | protein_coding |
| snoDB0385 | snoDB0385 | H_sapiens | Animals | mono_intronic | C/D | ENSG00000101977 | MCF2 | protein_coding |
| snoDB0386 | SNORA18L4 | H_sapiens | Animals | mono_intergenic | H/ACA | | | intergenic |
| snoDB0387 | SNORA11E | H_sapiens | Animals | mono_intronic | H/ACA | ENSG00000187243 | MAGED4B | protein_coding |
| snoDB0388 | SNORA11D | H_sapiens | Animals | mono_intronic | H/ACA | ENSG00000154545 | MAGED4 | protein_coding |
| snoDB0389 | SNORA70 | H_sapiens | Animals | mono_intergenic | H/ACA | | | intergenic |
| snoDB0390 | SNORA70 | H_sapiens | Animals | mono_intronic | H/ACA | ENSG00000233609 | RPL10P19 | non_coding |
| snoDB0391 | SNORA62 | H_sapiens | Animals | mono_intergenic | H/ACA | | | intergenic |
| snoDB0392 | SNORA67L5 | H_sapiens | Animals | mono_intergenic | H/ACA | | | intergenic |
| snoDB0393 | SNORA70 | H_sapiens | Animals | mono_intergenic | H/ACA | | | intergenic |
| snoDB0394 | SNORD65B | H_sapiens | Animals | mono_intergenic | C/D | | | intergenic |
| snoDB0395 | SNORD13 | H_sapiens | Animals | mono_intergenic | C/D | | | intergenic |
| snoDB0396 | SNORA7A | H_sapiens | Animals | mono_intergenic | H/ACA | | | intergenic |
| snoDB0397 | RNU105C | H_sapiens | Animals | mono_intergenic | H/ACA | | | intergenic |
| snoDB0398 | SNORA51L11 | H_sapiens | Animals | mono_intergenic | H/ACA | | | intergenic |
| snoDB0399 | SNORA20L3 | H_sapiens | Animals | mono_intergenic | H/ACA | | | intergenic |
| snoDB0400 | U8 | H_sapiens | Animals | mono_intergenic | C/D | | | intergenic |
| snoDB0401 | SNORA1B | H_sapiens | Animals | mono_intergenic | H/ACA | | | intergenic |
| snoDB0402 | SNORD3H | H_sapiens | Animals | mono_intergenic | C/D | | | intergenic |
| snoDB0403 | SNORA72 | H_sapiens | Animals | mono_intronic | H/ACA | ENSG00000156482 | RPL30 | protein_coding |
| snoDB0404 | SNORD77B | H_sapiens | Animals | mono_intergenic | C/D | | | intergenic |
| snoDB0405 | SNORA32L2 | H_sapiens | Animals | mono_intergenic | H/ACA | | | intergenic |
| snoDB0406 | U3 | H_sapiens | Animals | mono_intergenic | C/D | | | intergenic |
| snoDB0407 | SNORA72 | H_sapiens | Animals | mono_intergenic | H/ACA | | | intergenic |
| snoDB0408 | snoDB0408 | H_sapiens | Animals | mono_intronic | H/ACA | ENSG00000286308 | | non_coding |
| snoDB0409 | SNORA25 | H_sapiens | Animals | mono_intronic | H/ACA | ENSG00000153310 | CYRIB | protein_coding |
| snoDB0410 | snoDB0410 | H_sapiens | Animals | mono_intergenic | H/ACA | | | intergenic |
| snoDB0411 | snoDB0411 | H_sapiens | Animals | mono_intronic | C/D | ENSG00000104490 | NCALD | protein_coding |

| | | | | | | | | |
|---|---|---|---|---|---|---|---|---|
| snoDB0412 | SNORA40L8 | H_sapiens | Animals | mono_intergenic | H/ACA | | | intergenic |
| snoDB0413 | snoDB0413 | H_sapiens | Animals | mono_intergenic | C/D | | | intergenic |
| snoDB0414 | SNORA12L1 | H_sapiens | Animals | mono_intergenic | H/ACA | | | intergenic |
| snoDB0415 | snoDB0415 | H_sapiens | Animals | mono_intergenic | C/D | | | intergenic |
| snoDB0416 | SNORD54 | H_sapiens | Animals | mono_intronic | C/D | ENSG00000008988 | RPS20 | protein_coding |
| snoDB0417 | SNORA45L4 | H_sapiens | Animals | mono_intergenic | H/ACA | | | intergenic |
| snoDB0418 | U3 | H_sapiens | Animals | mono_intergenic | C/D | | | intergenic |
| snoDB0419 | SNORD87 | H_sapiens | Animals | mono_intronic | C/D | ENSG00000245910 | SNHG6 | non_coding |
| snoDB0420 | SNORD38D | H_sapiens | Animals | mono_intergenic | C/D | | | intergenic |
| snoDB0421 | snoDB0421 | H_sapiens | Animals | intronic_cluster | C/D | ENSG00000083168 | KAT6A | protein_coding |
| snoDB0423 | SNORD39 | H_sapiens | Animals | mono_intronic | C/D | ENSG00000171889 | MIR31HG | non_coding |
| snoDB0424 | snoDB0424 | H_sapiens | Animals | mono_intergenic | H/ACA | | | intergenic |
| snoDB0425 | SNORA70 | H_sapiens | Animals | mono_intergenic | H/ACA | | | intergenic |
| snoDB0426 | SNORA70 | H_sapiens | Animals | mono_intergenic | H/ACA | | | intergenic |
| snoDB0427 | SNORD95L1 | H_sapiens | Animals | mono_intergenic | C/D | | | intergenic |
| snoDB0428 | SNORA26L3 | H_sapiens | Animals | mono_intergenic | H/ACA | | | intergenic |
| snoDB0429 | SNORD116 | H_sapiens | Animals | mono_intronic | C/D | ENSG00000196814 | MVB12B | protein_coding |
| snoDB0430 | snoDB0430 | H_sapiens | Animals | mono_intergenic | H/ACA | | | intergenic |
| snoDB0431 | SNORA65 | H_sapiens | Animals | mono_intronic | H/ACA | ENSG00000197958 | RPL12 | protein_coding |
| snoDB0432 | SNORA84 | H_sapiens | Animals | mono_intronic | H/ACA | ENSG00000196305 | IARS1 | protein_coding |
| snoDB0433 | SNORA30B | H_sapiens | Animals | mono_intronic | H/ACA | ENSG00000188352 | FOCAD | protein_coding |
| snoDB0434 | snoDB0434 | H_sapiens | Animals | mono_intronic | H/ACA | ENSG00000148123 | PLPPR1 | protein_coding |
| snoDB0435 | SNORD62 | H_sapiens | Animals | mono_intronic | C/D | ENSG00000177239 | MAN1B1 | protein_coding |
| snoDB0436 | SNORA70 | H_sapiens | Animals | mono_intergenic | H/ACA | | | intergenic |
| snoDB0437 | SNORD90 | H_sapiens | Animals | mono_intronic | C/D | ENSG00000056586 | RC3H2 | protein_coding |
| snoDB0438 | U8 | H_sapiens | Animals | mono_intergenic | C/D | | | intergenic |
| snoDB0439 | snoDB0439 | H_sapiens | Animals | mono_intergenic | H/ACA | | | intergenic |
| snoDB0440 | SNORD62A | H_sapiens | Animals | mono_intronic | C/D | ENSG00000288701 | PRRC2B | protein_coding |
| snoDB0441 | SNORD62B | H_sapiens | Animals | mono_intronic | C/D | ENSG00000288701 | PRRC2B | protein_coding |
| snoDB0442 | SNORA70C | H_sapiens | Animals | mono_intronic | H/ACA | ENSG00000148219 | ASTN2 | protein_coding |
| snoDB0443 | SNORD121B | H_sapiens | Animals | mono_intronic | C/D | ENSG00000137073 | UBAP2 | protein_coding |
| snoDB0444 | SNORD121A | H_sapiens | Animals | mono_intronic | C/D | ENSG00000137073 | UBAP2 | protein_coding |
| snoDB0445 | SNORD24 | H_sapiens | Animals | mono_intronic | C/D | ENSG00000148303 | RPL7A | protein_coding |
| snoDB0446 | SNORD36B | H_sapiens | Animals | mono_intronic | C/D | ENSG00000148303 | RPL7A | protein_coding |
| snoDB0447 | SNORD36A | H_sapiens | Animals | mono_intronic | C/D | ENSG00000148303 | RPL7A | protein_coding |
| snoDB0448 | SNORD36C | H_sapiens | Animals | mono_intronic | C/D | ENSG00000148303 | RPL7A | protein_coding |
| snoDB0449 | SNORA70 | H_sapiens | Animals | mono_intergenic | H/ACA | | | intergenic |
| snoDB0450 | snoDB0450 | H_sapiens | Animals | mono_intergenic | H/ACA | | | intergenic |
| snoDB0451 | snoDB0451 | H_sapiens | Animals | mono_intergenic | C/D | | | intergenic |
| snoDB0452 | SNORA1L3 | H_sapiens | Animals | mono_intergenic | H/ACA | | | intergenic |
| snoDB0453 | SNORD14A | H_sapiens | Animals | mono_intronic | C/D | ENSG00000110700 | RPS13 | protein_coding |
| snoDB0454 | SNORD14B | H_sapiens | Animals | mono_intronic | C/D | ENSG00000110700 | RPS13 | protein_coding |
| snoDB0455 | U3 | H_sapiens | Animals | mono_intronic | C/D | ENSG00000285283 | | protein_coding |
| snoDB0456 | snoDB0456 | H_sapiens | Animals | mono_intergenic | H/ACA | | | intergenic |
| snoDB0457 | snoDB0457 | H_sapiens | Animals | mono_intronic | C/D | ENSG00000175216 | CKAP5 | protein_coding |

| | | | | | | | | |
|---|---|---|---|---|---|---|---|---|
| snoDB0458 | SNORD67 | H_sapiens | Animals | mono_intronic | C/D | ENSG00000175216 | CKAP5 | protein_coding |
| snoDB0459 | SNORD56L6 | H_sapiens | Animals | mono_intergenic | C/D | | | intergenic |
| snoDB0460 | SNORD6 | H_sapiens | Animals | mono_intronic | C/D | ENSG00000166012 | TAF1D | protein_coding |
| snoDB0461 | SNORA25 | H_sapiens | Animals | mono_intronic | H/ACA | ENSG00000166012 | TAF1D | protein_coding |
| snoDB0462 | SNORA32 | H_sapiens | Animals | mono_intronic | H/ACA | ENSG00000166012 | TAF1D | protein_coding |
| snoDB0463 | SNORA1 | H_sapiens | Animals | mono_intronic | H/ACA | ENSG00000166012 | TAF1D | protein_coding |
| snoDB0464 | SNORA8 | H_sapiens | Animals | mono_intronic | H/ACA | ENSG00000166012 | TAF1D | protein_coding |
| snoDB0465 | SNORD5 | H_sapiens | Animals | mono_intronic | C/D | ENSG00000166012 | TAF1D | protein_coding |
| snoDB0466 | SNORA18 | H_sapiens | Animals | mono_intronic | H/ACA | ENSG00000166012 | TAF1D | protein_coding |
| snoDB0467 | SNORA40 | H_sapiens | Animals | mono_intronic | H/ACA | ENSG00000166012 | TAF1D | protein_coding |
| snoDB0468 | SNORD39 | H_sapiens | Animals | mono_intergenic | C/D | | | intergenic |
| snoDB0469 | snoDB0469 | H_sapiens | Animals | mono_intergenic | C/D | | | intergenic |
| snoDB0470 | snosnR66 | H_sapiens | Animals | mono_intergenic | C/D | | | intergenic |
| snoDB0471 | U8 | H_sapiens | Animals | mono_intergenic | C/D | | | intergenic |
| snoDB0472 | snoDB0472 | H_sapiens | Animals | mono_intergenic | C/D | | | intergenic |
| snoDB0473 | snoDB0473 | H_sapiens | Animals | mono_intergenic | H/ACA | | | intergenic |
| snoDB0474 | SNORD14E | H_sapiens | Animals | mono_intronic | C/D | ENSG00000109971 | HSPA8 | protein_coding |
| snoDB0475 | SNORD14D | H_sapiens | Animals | mono_intronic | C/D | ENSG00000109971 | HSPA8 | protein_coding |
| snoDB0476 | SNORD14C | H_sapiens | Animals | mono_intronic | C/D | ENSG00000109971 | HSPA8 | protein_coding |
| snoDB0477 | SNORD15A | H_sapiens | Animals | mono_intronic | C/D | ENSG00000149273 | RPS3 | protein_coding |
| snoDB0478 | SNORD15B | H_sapiens | Animals | mono_intronic | C/D | ENSG00000149273 | RPS3 | protein_coding |
| snoDB0479 | SNORA62 | H_sapiens | Animals | mono_intronic | H/ACA | ENSG00000167346 | MMP26 | protein_coding |
| snoDB0480 | SNORA52 | H_sapiens | Animals | mono_intronic | H/ACA | ENSG00000177600 | RPLP2 | protein_coding |
| snoDB0481 | SNORA54 | H_sapiens | Animals | mono_intronic | H/ACA | ENSG00000205531 | NAP1L4 | protein_coding |
| snoDB0482 | snoDB0482 | H_sapiens | Animals | mono_intergenic | C/D | | | intergenic |
| snoDB0483 | snoDB0483 | H_sapiens | Animals | mono_intronic | H/ACA | ENSG00000167257 | RNF214 | protein_coding |
| snoDB0484 | SNORD22 | H_sapiens | Animals | mono_intronic | C/D | ENSG00000255717 | SNHG1 | non_coding |
| snoDB0485 | SNORD30 | H_sapiens | Animals | mono_intronic | C/D | ENSG00000255717 | SNHG1 | non_coding |
| snoDB0486 | SNORD22 | H_sapiens | Animals | mono_intronic | C/D | ENSG00000255717 | SNHG1 | non_coding |
| snoDB0487 | SNORD28 | H_sapiens | Animals | mono_intronic | C/D | ENSG00000255717 | SNHG1 | non_coding |
| snoDB0488 | SNORD27 | H_sapiens | Animals | mono_intronic | C/D | ENSG00000255717 | SNHG1 | non_coding |
| snoDB0489 | SNORD26 | H_sapiens | Animals | mono_intronic | C/D | ENSG00000255717 | SNHG1 | non_coding |
| snoDB0490 | SNORD25 | H_sapiens | Animals | mono_exonic | C/D | ENSG00000255717 | SNHG1 | non_coding |
| snoDB0491 | SNORA7A | H_sapiens | Animals | mono_intronic | H/ACA | ENSG00000149380 | P4HA3 | protein_coding |
| snoDB0492 | SNORD97 | H_sapiens | Animals | mono_intronic | C/D | ENSG00000110321 | EIF4G2 | protein_coding |
| snoDB0493 | snoDB0493 | H_sapiens | Animals | intergenic_cluster | H/ACA | | | intergenic |
| snoDB0494 | snoDB0494 | H_sapiens | Animals | mono_intronic | C/D | ENSG00000166483 | WEE1 | protein_coding |
| snoDB0495 | SNORA7B | H_sapiens | Animals | mono_intronic | H/ACA | ENSG00000167323 | STIM1 | protein_coding |
| snoDB0496 | snoDB0496 | H_sapiens | Animals | mono_intergenic | H/ACA | | | intergenic |
| snoDB0497 | SNORA70E | H_sapiens | Animals | mono_intronic | H/ACA | ENSG00000137502 | RAB30 | protein_coding |
| snoDB0498 | SNORA23 | H_sapiens | Animals | mono_intronic | H/ACA | ENSG00000205339 | IPO7 | protein_coding |
| snoDB0499 | SNORA57 | H_sapiens | Animals | mono_intronic | H/ACA | ENSG00000214756 | CSKMT | protein_coding |
| snoDB0500 | snoDB0500 | H_sapiens | Animals | mono_intergenic | H/ACA | | | intergenic |
| snoDB0501 | SNORA3A | H_sapiens | Animals | mono_intronic | H/ACA | ENSG00000166441 | RPL27A | protein_coding |
| snoDB0502 | SNORA3B | H_sapiens | Animals | mono_intronic | H/ACA | ENSG00000166441 | RPL27A | protein_coding |

| | | | | | | | | |
|---|---|---|---|---|---|---|---|---|
| snoDB0503 | U8 | H_sapiens | Animals | mono_intergenic | C/D | | | intergenic |
| snoDB0504 | U8 | H_sapiens | Animals | mono_intergenic | C/D | | | intergenic |
| snoDB0505 | U8 | H_sapiens | Animals | mono_intergenic | C/D | | | intergenic |
| snoDB0506 | snoDB0506 | H_sapiens | Animals | mono_intronic | H/ACA | ENSG00000134460 | IL2RA | protein_coding |
| snoDB0507 | snoDB0507 | H_sapiens | Animals | mono_intergenic | H/ACA | | | intergenic |
| snoDB0508 | U3 | H_sapiens | Animals | mono_intronic | C/D | ENSG00000204740 | MALRD1 | protein_coding |
| snoDB0509 | snoDB0509 | H_sapiens | Animals | mono_intronic | C/D | ENSG00000183049 | CAMK1D | protein_coding |
| snoDB0510 | snoDB0510 | H_sapiens | Animals | mono_intergenic | H/ACA | | | intergenic |
| snoDB0511 | SNORD115 | H_sapiens | Animals | mono_intergenic | C/D | | | intergenic |
| snoDB0512 | SNORA40L10 | H_sapiens | Animals | mono_exonic | H/ACA | ENSG00000237002 | | non_coding |
| snoDB0513 | SNORD3J | H_sapiens | Animals | mono_intergenic | C/D | | | intergenic |
| snoDB0514 | snoDB0514 | H_sapiens | Animals | mono_intergenic | C/D | | | intergenic |
| snoDB0515 | SNORD98 | H_sapiens | Animals | mono_intronic | C/D | ENSG00000060339 | CCAR1 | protein_coding |
| snoDB0516 | SNORA11F | H_sapiens | Animals | mono_intergenic | H/ACA | | | intergenic |
| snoDB0517 | snoDB0517 | H_sapiens | Animals | mono_intergenic | H/ACA | | | intergenic |
| snoDB0518 | SNORA71 | H_sapiens | Animals | mono_intronic | H/ACA | ENSG00000230417 | LINC00595 | non_coding |
| snoDB0519 | SNORA25L14 | H_sapiens | Animals | mono_intergenic | H/ACA | | | intergenic |
| snoDB0520 | SNORA12 | H_sapiens | Animals | mono_intronic | H/ACA | ENSG00000095485 | CWF19L1 | protein_coding |
| snoDB0521 | SNORA17L1 | H_sapiens | Animals | mono_intergenic | H/ACA | | | intergenic |
| snoDB0522 | SNORA19 | H_sapiens | Animals | mono_intronic | H/ACA | ENSG00000107581 | EIF3A | protein_coding |
| snoDB0523 | SNORD60L1 | H_sapiens | Animals | mono_intronic | C/D | ENSG00000154493 | C10orf90 | protein_coding |
| snoDB0524 | snoDB0524 | H_sapiens | Animals | mono_intronic | H/ACA | ENSG00000107581 | EIF3A | protein_coding |
| snoDB0525 | SNORA74C-2 | H_sapiens | Animals | mono_intronic | H/ACA | ENSG00000204152 | TIMM23B | protein_coding |
| snoDB0526 | snoDB0526 | H_sapiens | Animals | mono_intergenic | H/ACA | | | intergenic |
| snoDB0527 | SNORA74C-1 | H_sapiens | Animals | mono_intronic | H/ACA | ENSG00000265354 | TIMM23 | protein_coding |
| snoDB0528 | SNORA36CL2 | H_sapiens | Animals | mono_intergenic | H/ACA | | | intergenic |
| snoDB0529 | SNORD74L3 | H_sapiens | Animals | mono_intergenic | C/D | | | intergenic |
| snoDB0530 | U3 | H_sapiens | Animals | mono_intergenic | C/D | | | intergenic |
| snoDB0531 | SNORA75 | H_sapiens | Animals | mono_intergenic | H/ACA | | | intergenic |
| snoDB0532 | SNORA75 | H_sapiens | Animals | mono_intergenic | H/ACA | | | intergenic |
| snoDB0533 | snoDB0533 | H_sapiens | Animals | mono_intergenic | H/ACA | | | intergenic |
| snoDB0534 | SNORA75 | H_sapiens | Animals | mono_intergenic | H/ACA | | | intergenic |
| snoDB0535 | snoDB0535 | H_sapiens | Animals | mono_intergenic | H/ACA | | | intergenic |
| snoDB0536 | SNORA10L1 | H_sapiens | Animals | mono_intergenic | H/ACA | | | intergenic |
| snoDB0537 | SNORA2A | H_sapiens | Animals | mono_intronic | H/ACA | ENSG00000139620 | KANSL2 | protein_coding |
| snoDB0538 | SNORA2B | H_sapiens | Animals | mono_intronic | H/ACA | ENSG00000139620 | KANSL2 | protein_coding |
| snoDB0539 | SNORA2C | H_sapiens | Animals | mono_intronic | H/ACA | ENSG00000139620 | KANSL2 | protein_coding |
| snoDB0540 | SNORA22 | H_sapiens | Animals | mono_intronic | H/ACA | ENSG00000151229 | SLC2A13 | protein_coding |
| snoDB0541 | snoDB0541 | H_sapiens | Animals | mono_intergenic | H/ACA | | | intergenic |
| snoDB0542 | SNORA17L2 | H_sapiens | Animals | mono_intergenic | H/ACA | | | intergenic |
| snoDB0543 | SNORA70 | H_sapiens | Animals | mono_intronic | H/ACA | ENSG00000258077 | | non_coding |
| snoDB0544 | SNORA70G | H_sapiens | Animals | mono_intergenic | H/ACA | | | intergenic |
| snoDB0545 | U8 | H_sapiens | Animals | mono_intergenic | C/D | | | intergenic |
| snoDB0546 | SNORD74L4 | H_sapiens | Animals | intergenic_cluster | C/D | | | intergenic |
| snoDB0547 | SNORA45 | H_sapiens | Animals | mono_intronic | H/ACA | ENSG00000289309 | | non_coding |

| | | | | | | | | |
|---|---|---|---|---|---|---|---|---|
| snoDB0548 | snoDB0548 | H_sapiens | Animals | mono_intronic | C/D | ENSG00000151136 | ABTB3 | protein_coding |
| snoDB0549 | snoDB0549 | H_sapiens | Animals | mono_intronic | H/ACA | ENSG00000257817 | TBX3-AS1 | non_coding |
| snoDB0550 | SNORA38BL1 | H_sapiens | Animals | mono_intronic | H/ACA | ENSG00000256149 | | non_coding |
| snoDB0551 | SNORA40L5 | H_sapiens | Animals | mono_intergenic | H/ACA | | | intergenic |
| snoDB0552 | SNORD56L4 | H_sapiens | Animals | mono_intronic | C/D | ENSG00000257683 | LINC02463 | non_coding |
| snoDB0553 | snoDB0553 | H_sapiens | Animals | mono_intergenic | C/D | | | intergenic |
| snoDB0554 | SNORA49 | H_sapiens | Animals | mono_intronic | H/ACA | ENSG00000183495 | EP400 | protein_coding |
| snoDB0555 | SNORA53 | H_sapiens | Animals | mono_intronic | H/ACA | ENSG00000075415 | SLC25A3 | protein_coding |
| snoDB0557 | SNORA48L6 | H_sapiens | Animals | mono_intronic | H/ACA | ENSG00000258679 | | non_coding |
| snoDB0558 | SNORA9B | H_sapiens | Animals | mono_intronic | H/ACA | ENSG00000111364 | DDX55 | protein_coding |
| snoDB0559 | snoDB0559 | H_sapiens | Animals | mono_intronic | H/ACA | ENSG00000033030 | ZCCHC8 | protein_coding |
| snoDB0560 | U8 | H_sapiens | Animals | mono_intronic | C/D | ENSG00000136011 | STAB2 | protein_coding |
| snoDB0561 | SNORD59A | H_sapiens | Animals | mono_intronic | C/D | ENSG00000110955 | ATP5F1B | protein_coding |
| snoDB0564 | SNORA74 | H_sapiens | Animals | mono_intergenic | H/ACA | | | intergenic |
| snoDB0565 | snoDB0565 | H_sapiens | Animals | mono_intronic | C/D | ENSG00000185306 | C12orf56 | protein_coding |
| snoDB0566 | SNORD83 | H_sapiens | Animals | mono_intronic | C/D | ENSG00000153179 | RASSF3 | protein_coding |
| snoDB0567 | SNORA70 | H_sapiens | Animals | mono_intergenic | H/ACA | | | intergenic |
| snoDB0568 | SNORD50BL1 | H_sapiens | Animals | mono_intronic | C/D | ENSG00000111237 | VPS29 | protein_coding |
| snoDB0569 | snoDB0569 | H_sapiens | Animals | mono_intronic | C/D | ENSG00000247498 | GPRC5D-AS1 | non_coding |
| snoDB0570 | SNORD102 | H_sapiens | Animals | mono_intronic | C/D | ENSG00000122026 | RPL21 | protein_coding |
| snoDB0571 | SNORA27 | H_sapiens | Animals | mono_intronic | H/ACA | ENSG00000122026 | RPL21 | protein_coding |
| snoDB0572 | SNORD27 | H_sapiens | Animals | mono_intergenic | C/D | | | intergenic |
| snoDB0573 | snoDB0573 | H_sapiens | Animals | mono_intergenic | H/ACA | | | intergenic |
| snoDB0574 | SNORD116 | H_sapiens | Animals | mono_intergenic | C/D | | | intergenic |
| snoDB0575 | SNORD37L1 | H_sapiens | Animals | mono_intergenic | C/D | | | intergenic |
| snoDB0576 | SNORA9 | H_sapiens | Animals | mono_intergenic | H/ACA | | | intergenic |
| snoDB0577 | snoDB0577 | H_sapiens | Animals | mono_intergenic | H/ACA | | | intergenic |
| snoDB0578 | SNORD38B | H_sapiens | Animals | mono_intergenic | C/D | | | intergenic |
| snoDB0579 | SNORA25L4 | H_sapiens | Animals | mono_intergenic | H/ACA | | | intergenic |
| snoDB0580 | SNORD31B | H_sapiens | Animals | mono_intronic | C/D | ENSG00000204442 | NALF1 | protein_coding |
| snoDB0581 | SNORA25L3 | H_sapiens | Animals | intergenic_cluster | H/ACA | | | intergenic |
| snoDB0582 | SNORD44L1 | H_sapiens | Animals | mono_intergenic | C/D | | | intergenic |
| snoDB0583 | SNORD36 | H_sapiens | Animals | mono_intergenic | C/D | | | intergenic |
| snoDB0584 | snoDB0584 | H_sapiens | Animals | mono_intronic | H/ACA | ENSG00000139597 | N4BP2L1 | protein_coding |
| snoDB0585 | SNORA31B | H_sapiens | Animals | mono_exonic | H/ACA | ENSG00000133112 | TPT1 | protein_coding |
| snoDB0586 | SNORA31 | H_sapiens | Animals | mono_intronic | H/ACA | ENSG00000133112 | TPT1 | protein_coding |
| snoDB0587 | SNORD37L2 | H_sapiens | Animals | mono_intronic | C/D | ENSG00000257986 | LINC02306 | non_coding |
| snoDB0588 | snoDB0588 | H_sapiens | Animals | mono_intergenic | H/ACA | | | intergenic |
| snoDB0589 | SNORA42L2 | H_sapiens | Animals | mono_intronic | H/ACA | ENSG00000139865 | TTC6 | protein_coding |
| snoDB0590 | SNORA25L9 | H_sapiens | Animals | mono_intergenic | H/ACA | | | intergenic |
| snoDB0591 | SNORD58 | H_sapiens | Animals | mono_intronic | C/D | ENSG00000185246 | PRPF39 | protein_coding |
| snoDB0592 | SNORD127 | H_sapiens | Animals | mono_intronic | C/D | ENSG00000185246 | PRPF39 | protein_coding |
| snoDB0593 | SNORA70 | H_sapiens | Animals | mono_intronic | H/ACA | ENSG00000139921 | TMX1 | protein_coding |
| snoDB0594 | SNORA11BL2 | H_sapiens | Animals | mono_intergenic | H/ACA | | | intergenic |
| snoDB0595 | SNORD56B | H_sapiens | Animals | mono_intronic | C/D | ENSG00000197555 | SIPA1L1 | protein_coding |

| | | | | | | | | |
|---|---|---|---|---|---|---|---|---|
| snoDB0596 | snoDB0596 | H_sapiens | Animals | mono_intronic | H/ACA | ENSG00000100591 | AHSA1 | protein_coding |
| snoDB0597 | SNORA11B | H_sapiens | Animals | mono_intronic | H/ACA | ENSG00000133943 | DGLUCY | protein_coding |
| snoDB0599 | SNORD41L1 | H_sapiens | Animals | mono_intronic | C/D | ENSG00000258458 | OXA1L-DT | non_coding |
| snoDB0600 | snoDB0600 | H_sapiens | Animals | mono_intergenic | C/D | | | intergenic |
| snoDB0601 | SNORA79B | H_sapiens | Animals | mono_intronic | H/ACA | ENSG00000100814 | CCNB1IP1 | protein_coding |
| snoDB0602 | SNORD126 | H_sapiens | Animals | mono_intronic | C/D | ENSG00000100814 | CCNB1IP1 | protein_coding |
| snoDB0603 | SNORD9 | H_sapiens | Animals | mono_intronic | C/D | ENSG00000100888 | CHD8 | protein_coding |
| snoDB0604 | SNORD51L1 | H_sapiens | Animals | mono_intronic | C/D | ENSG00000088808 | PPP1R13B | protein_coding |
| snoDB0605 | SNORA28 | H_sapiens | Animals | mono_intronic | H/ACA | ENSG00000100664 | EIF5 | protein_coding |
| snoDB0606 | U3 | H_sapiens | Animals | mono_intergenic | C/D | | | intergenic |
| snoDB0607 | SNORD112 | H_sapiens | Animals | mono_intronic | C/D | ENSG00000225746 | MEG8 | non_coding |
| snoDB0608 | SNORD113-1 | H_sapiens | Animals | intronic_cluster | C/D | ENSG00000225746 | MEG8 | non_coding |
| snoDB0609 | SNORD113-2 | H_sapiens | Animals | intronic_cluster | C/D | ENSG00000225746 | MEG8 | non_coding |
| snoDB0610 | SNORD113-3 | H_sapiens | Animals | intronic_cluster | C/D | ENSG00000225746 | MEG8 | non_coding |
| snoDB0611 | SNORD113-4 | H_sapiens | Animals | mono_exonic | C/D | ENSG00000225746 | MEG8 | non_coding |
| snoDB0612 | SNORD113-5 | H_sapiens | Animals | mono_intronic | C/D | ENSG00000225746 | MEG8 | non_coding |
| snoDB0613 | SNORD113-6 | H_sapiens | Animals | mono_intronic | C/D | ENSG00000225746 | MEG8 | non_coding |
| snoDB0614 | SNORD113-7 | H_sapiens | Animals | mono_intronic | C/D | ENSG00000225746 | MEG8 | non_coding |
| snoDB0615 | SNORD113-8 | H_sapiens | Animals | mono_intronic | C/D | ENSG00000225746 | MEG8 | non_coding |
| snoDB0616 | SNORD113-9 | H_sapiens | Animals | mono_intronic | C/D | ENSG00000225746 | MEG8 | non_coding |
| snoDB0617 | SNORD114-1 | H_sapiens | Animals | mono_intronic | C/D | ENSG00000225746 | MEG8 | non_coding |
| snoDB0618 | SNORD114-2 | H_sapiens | Animals | intronic_cluster | C/D | ENSG00000225746 | MEG8 | non_coding |
| snoDB0619 | SNORD114-3 | H_sapiens | Animals | intronic_cluster | C/D | ENSG00000225746 | MEG8 | non_coding |
| snoDB0620 | SNORD114-4 | H_sapiens | Animals | mono_intronic | C/D | ENSG00000225746 | MEG8 | non_coding |
| snoDB0621 | SNORD114-5 | H_sapiens | Animals | mono_intronic | C/D | ENSG00000225746 | MEG8 | non_coding |
| snoDB0622 | SNORD114-15L1 | H_sapiens | Animals | intronic_cluster | C/D | ENSG00000225746 | MEG8 | non_coding |
| snoDB0623 | SNORD114-6 | H_sapiens | Animals | intronic_cluster | C/D | ENSG00000225746 | MEG8 | non_coding |
| snoDB0624 | SNORD114-7 | H_sapiens | Animals | mono_intronic | C/D | ENSG00000225746 | MEG8 | non_coding |
| snoDB0625 | SNORD114-9 | H_sapiens | Animals | intronic_cluster | C/D | ENSG00000225746 | MEG8 | non_coding |
| snoDB0626 | SNORD114-10 | H_sapiens | Animals | mono_intronic | C/D | ENSG00000225746 | MEG8 | non_coding |
| snoDB0627 | SNORD114-11 | H_sapiens | Animals | mono_intronic | C/D | ENSG00000225746 | MEG8 | non_coding |
| snoDB0628 | SNORD114-12 | H_sapiens | Animals | mono_intronic | C/D | ENSG00000225746 | MEG8 | non_coding |
| snoDB0629 | SNORD114-13 | H_sapiens | Animals | intronic_cluster | C/D | ENSG00000225746 | MEG8 | non_coding |
| snoDB0630 | SNORD114-14 | H_sapiens | Animals | intronic_cluster | C/D | ENSG00000225746 | MEG8 | non_coding |
| snoDB0631 | SNORD114-15 | H_sapiens | Animals | intronic_cluster | C/D | ENSG00000225746 | MEG8 | non_coding |
| snoDB0632 | SNORD114-16 | H_sapiens | Animals | intronic_cluster | C/D | ENSG00000225746 | MEG8 | non_coding |
| snoDB0633 | SNORD114-17 | H_sapiens | Animals | mono_intronic | C/D | ENSG00000225746 | MEG8 | non_coding |
| snoDB0634 | SNORD114-18 | H_sapiens | Animals | intronic_cluster | C/D | ENSG00000225746 | MEG8 | non_coding |
| snoDB0635 | SNORD114-19 | H_sapiens | Animals | intronic_cluster | C/D | ENSG00000225746 | MEG8 | non_coding |
| snoDB0636 | snoDB0636 | H_sapiens | Animals | intronic_cluster | C/D | ENSG00000225746 | MEG8 | non_coding |
| snoDB0637 | SNORD113-10 | H_sapiens | Animals | intronic_cluster | C/D | ENSG00000225746 | MEG8 | non_coding |
| snoDB0638 | snoDB0638 | H_sapiens | Animals | intronic_cluster | C/D | ENSG00000225746 | MEG8 | non_coding |
| snoDB0639 | SNORD114-20 | H_sapiens | Animals | intronic_cluster | C/D | ENSG00000225746 | MEG8 | non_coding |
| snoDB0640 | SNORD114-21 | H_sapiens | Animals | intronic_cluster | C/D | ENSG00000225746 | MEG8 | non_coding |
| snoDB0641 | SNORD114-22 | H_sapiens | Animals | mono_intronic | C/D | ENSG00000225746 | MEG8 | non_coding |

| | | | | | | | | |
|---|---|---|---|---|---|---|---|---|
| snoDB0642 | SNORD114-23 | H_sapiens | Animals | mono_intronic | C/D | ENSG00000225746 | MEG8 | non_coding |
| snoDB0643 | SNORD114-24 | H_sapiens | Animals | mono_intronic | C/D | ENSG00000225746 | MEG8 | non_coding |
| snoDB0644 | SNORD114-25 | H_sapiens | Animals | mono_intronic | C/D | ENSG00000225746 | MEG8 | non_coding |
| snoDB0645 | SNORD114-26 | H_sapiens | Animals | mono_intronic | C/D | ENSG00000225746 | MEG8 | non_coding |
| snoDB0646 | SNORD114-27 | H_sapiens | Animals | mono_intronic | C/D | ENSG00000225746 | MEG8 | non_coding |
| snoDB0647 | SNORD114-28 | H_sapiens | Animals | mono_intronic | C/D | ENSG00000225746 | MEG8 | non_coding |
| snoDB0648 | SNORD114-29 | H_sapiens | Animals | mono_intronic | C/D | ENSG00000225746 | MEG8 | non_coding |
| snoDB0649 | SNORD114-30 | H_sapiens | Animals | intronic_cluster | C/D | ENSG00000225746 | MEG8 | non_coding |
| snoDB0650 | SNORD114-31 | H_sapiens | Animals | intronic_cluster | C/D | ENSG00000225746 | MEG8 | non_coding |
| snoDB0651 | snoDB0651 | H_sapiens | Animals | mono_intronic | C/D | ENSG00000225746 | MEG8 | non_coding |
| snoDB0652 | snoDB0652 | H_sapiens | Animals | mono_intronic | C/D | ENSG00000225746 | MEG8 | non_coding |
| snoDB0653 | U3 | H_sapiens | Animals | mono_intronic | C/D | ENSG00000286551 | | non_coding |
| snoDB0654 | SNORD8 | H_sapiens | Animals | mono_intronic | C/D | ENSG00000100888 | CHD8 | protein_coding |
| snoDB0655 | SNORA79 | H_sapiens | Animals | mono_intronic | H/ACA | ENSG00000165417 | GTF2A1 | protein_coding |
| snoDB0656 | snoDB0656 | H_sapiens | Animals | mono_intergenic | H/ACA | | | intergenic |
| snoDB0657 | U3 | H_sapiens | Animals | mono_intronic | C/D | ENSG00000133962 | CATSPERB | protein_coding |
| snoDB0658 | snoDB0658 | H_sapiens | Animals | mono_intergenic | H/ACA | | | intergenic |
| snoDB0659 | snoDB0659 | H_sapiens | Animals | mono_intergenic | H/ACA | | | intergenic |
| snoDB0660 | SNORA32L1 | H_sapiens | Animals | mono_intergenic | H/ACA | | | intergenic |
| snoDB0661 | SNORA48L9 | H_sapiens | Animals | mono_intergenic | H/ACA | | | intergenic |
| snoDB0662 | U8 | H_sapiens | Animals | mono_intronic | C/D | ENSG00000178081 | ULK4P3 | non_coding |
| snoDB0663 | SNORD116-5 | H_sapiens | Animals | mono_intronic | C/D | ENSG00000224078 | SNHG14 | non_coding |
| snoDB0664 | SNORD116-10 | H_sapiens | Animals | mono_intronic | C/D | ENSG00000224078 | SNHG14 | non_coding |
| snoDB0665 | SNORD116-11 | H_sapiens | Animals | mono_intronic | C/D | ENSG00000224078 | SNHG14 | non_coding |
| snoDB0666 | SNORD116-17 | H_sapiens | Animals | mono_intronic | C/D | ENSG00000224078 | SNHG14 | non_coding |
| snoDB0667 | U8 | H_sapiens | Animals | mono_intronic | C/D | ENSG00000166664 | CHRFAM7A | protein_coding |
| snoDB0668 | SNORD107 | H_sapiens | Animals | mono_intronic | C/D | ENSG00000214265 | | protein_coding |
| snoDB0669 | SNORD64 | H_sapiens | Animals | intronic_cluster | C/D | ENSG00000214265 | | protein_coding |
| snoDB0670 | SNORD108 | H_sapiens | Animals | intronic_cluster | C/D | ENSG00000214265 | | protein_coding |
| snoDB0671 | SNORD109A | H_sapiens | Animals | mono_exonic | C/D | ENSG00000224078 | SNHG14 | non_coding |
| snoDB0672 | SNORD116-1 | H_sapiens | Animals | intronic_cluster | C/D | ENSG00000224078 | SNHG14 | non_coding |
| snoDB0673 | SNORD116-2 | H_sapiens | Animals | intronic_cluster | C/D | ENSG00000224078 | SNHG14 | non_coding |
| snoDB0674 | SNORD116-3 | H_sapiens | Animals | intronic_cluster | C/D | ENSG00000224078 | SNHG14 | non_coding |
| snoDB0675 | SNORD116-4 | H_sapiens | Animals | mono_exonic | C/D | ENSG00000224078 | SNHG14 | non_coding |
| snoDB0676 | SNORD116-6 | H_sapiens | Animals | intronic_cluster | C/D | ENSG00000224078 | SNHG14 | non_coding |
| snoDB0677 | SNORD116-7 | H_sapiens | Animals | intronic_cluster | C/D | ENSG00000224078 | SNHG14 | non_coding |
| snoDB0678 | SNORD116-8 | H_sapiens | Animals | mono_intronic | C/D | ENSG00000224078 | SNHG14 | non_coding |
| snoDB0679 | SNORD116-9 | H_sapiens | Animals | mono_intronic | C/D | ENSG00000224078 | SNHG14 | non_coding |
| snoDB0680 | SNORD116-12 | H_sapiens | Animals | mono_intronic | C/D | ENSG00000224078 | SNHG14 | non_coding |
| snoDB0681 | SNORD116-13 | H_sapiens | Animals | intronic_cluster | C/D | ENSG00000224078 | SNHG14 | non_coding |
| snoDB0682 | SNORD116-14 | H_sapiens | Animals | intronic_cluster | C/D | ENSG00000224078 | SNHG14 | non_coding |
| snoDB0683 | SNORD116-15 | H_sapiens | Animals | mono_intronic | C/D | ENSG00000224078 | SNHG14 | non_coding |
| snoDB0684 | SNORD116-16 | H_sapiens | Animals | mono_intronic | C/D | ENSG00000224078 | SNHG14 | non_coding |
| snoDB0685 | SNORD116-18 | H_sapiens | Animals | intronic_cluster | C/D | ENSG00000224078 | SNHG14 | non_coding |
| snoDB0686 | SNORD116-19 | H_sapiens | Animals | intronic_cluster | C/D | ENSG00000224078 | SNHG14 | non_coding |

| | | | | | | | | |
|---|---|---|---|---|---|---|---|---|
| snoDB0687 | SNORD116-20 | H_sapiens | Animals | exonic_cluster | C/D | ENSG00000261069 | | non_coding |
| snoDB0688 | SNORD116-21 | H_sapiens | Animals | exonic_cluster | C/D | ENSG00000261069 | | non_coding |
| snoDB0689 | SNORD116-22 | H_sapiens | Animals | mono_exonic | C/D | ENSG00000224078 | SNHG14 | non_coding |
| snoDB0690 | SNORD116-23 | H_sapiens | Animals | mono_intronic | C/D | ENSG00000224078 | SNHG14 | non_coding |
| snoDB0691 | SNORD116-24 | H_sapiens | Animals | mono_intronic | C/D | ENSG00000224078 | SNHG14 | non_coding |
| snoDB0692 | SNORD116-25 | H_sapiens | Animals | mono_intronic | C/D | ENSG00000224078 | SNHG14 | non_coding |
| snoDB0693 | SNORD116-26 | H_sapiens | Animals | intronic_cluster | C/D | ENSG00000224078 | SNHG14 | non_coding |
| snoDB0694 | SNORD116-27 | H_sapiens | Animals | intronic_cluster | C/D | ENSG00000224078 | SNHG14 | non_coding |
| snoDB0695 | SNORD116-28 | H_sapiens | Animals | mono_intronic | C/D | ENSG00000224078 | SNHG14 | non_coding |
| snoDB0696 | SNORD77L3 | H_sapiens | Animals | mono_intergenic | C/D | | | intergenic |
| snoDB0697 | snoDB0697 | H_sapiens | Animals | mono_intergenic | C/D | | | intergenic |
| snoDB0698 | SNORD3P1 | H_sapiens | Animals | mono_intergenic | C/D | | | intergenic |
| snoDB0699 | SNORD13D | H_sapiens | Animals | mono_intergenic | C/D | | | intergenic |
| snoDB0701 | SNORD18C | H_sapiens | Animals | mono_intronic | C/D | ENSG00000174444 | RPL4 | protein_coding |
| snoDB0702 | SNORD16 | H_sapiens | Animals | mono_intronic | C/D | ENSG00000174444 | RPL4 | protein_coding |
| snoDB0703 | SNORD18A | H_sapiens | Animals | mono_intronic | C/D | ENSG00000174444 | RPL4 | protein_coding |
| snoDB0704 | snoDB0704 | H_sapiens | Animals | mono_intergenic | H/ACA | | | intergenic |
| snoDB0705 | SNORD77L4 | H_sapiens | Animals | mono_intergenic | C/D | | | intergenic |
| snoDB0706 | snoDB0706 | H_sapiens | Animals | mono_intronic | H/ACA | ENSG00000140557 | ST8SIA2 | protein_coding |
| snoDB0707 | SNORA25L11 | H_sapiens | Animals | mono_intergenic | H/ACA | | | intergenic |
| snoDB0708 | SNORD116-29 | H_sapiens | Animals | mono_intronic | C/D | ENSG00000224078 | SNHG14 | non_coding |
| snoDB0709 | SNORD116-30 | H_sapiens | Animals | mono_intronic | C/D | ENSG00000224078 | SNHG14 | non_coding |
| snoDB0710 | SNORD115 | H_sapiens | Animals | mono_intronic | C/D | ENSG00000224078 | SNHG14 | non_coding |
| snoDB0711 | SNORD115-1 | H_sapiens | Animals | mono_intronic | C/D | ENSG00000224078 | SNHG14 | non_coding |
| snoDB0712 | SNORD115-2 | H_sapiens | Animals | mono_intronic | C/D | ENSG00000224078 | SNHG14 | non_coding |
| snoDB0713 | SNORD115-3 | H_sapiens | Animals | mono_intronic | C/D | ENSG00000224078 | SNHG14 | non_coding |
| snoDB0714 | SNORD115-4 | H_sapiens | Animals | mono_intronic | C/D | ENSG00000224078 | SNHG14 | non_coding |
| snoDB0715 | SNORD115-5 | H_sapiens | Animals | mono_intronic | C/D | ENSG00000224078 | SNHG14 | non_coding |
| snoDB0716 | SNORD115-6 | H_sapiens | Animals | mono_intronic | C/D | ENSG00000224078 | SNHG14 | non_coding |
| snoDB0717 | SNORD115-7 | H_sapiens | Animals | mono_intronic | C/D | ENSG00000224078 | SNHG14 | non_coding |
| snoDB0718 | SNORD115-8 | H_sapiens | Animals | mono_intronic | C/D | ENSG00000224078 | SNHG14 | non_coding |
| snoDB0719 | SNORD115-9 | H_sapiens | Animals | mono_intronic | C/D | ENSG00000224078 | SNHG14 | non_coding |
| snoDB0720 | SNORD115-10 | H_sapiens | Animals | mono_intronic | C/D | ENSG00000224078 | SNHG14 | non_coding |
| snoDB0721 | SNORD115-11 | H_sapiens | Animals | mono_intronic | C/D | ENSG00000224078 | SNHG14 | non_coding |
| snoDB0722 | SNORD115-12 | H_sapiens | Animals | mono_intronic | C/D | ENSG00000224078 | SNHG14 | non_coding |
| snoDB0723 | SNORD115-13 | H_sapiens | Animals | mono_exonic | C/D | ENSG00000224078 | SNHG14 | non_coding |
| snoDB0724 | SNORD115-14 | H_sapiens | Animals | mono_intronic | C/D | ENSG00000224078 | SNHG14 | non_coding |
| snoDB0725 | SNORD115-15 | H_sapiens | Animals | mono_intronic | C/D | ENSG00000224078 | SNHG14 | non_coding |
| snoDB0726 | SNORD115-16 | H_sapiens | Animals | mono_intronic | C/D | ENSG00000224078 | SNHG14 | non_coding |
| snoDB0727 | SNORD115-17 | H_sapiens | Animals | mono_intronic | C/D | ENSG00000224078 | SNHG14 | non_coding |
| snoDB0728 | SNORD115-18 | H_sapiens | Animals | mono_intronic | C/D | ENSG00000224078 | SNHG14 | non_coding |
| snoDB0729 | SNORD115-19 | H_sapiens | Animals | mono_intronic | C/D | ENSG00000224078 | SNHG14 | non_coding |
| snoDB0730 | SNORD115-20 | H_sapiens | Animals | intronic_cluster | C/D | ENSG00000224078 | SNHG14 | non_coding |
| snoDB0731 | SNORD115-21 | H_sapiens | Animals | intronic_cluster | C/D | ENSG00000224078 | SNHG14 | non_coding |
| snoDB0732 | SNORD115-22 | H_sapiens | Animals | intronic_cluster | C/D | ENSG00000224078 | SNHG14 | non_coding |

| | | | | | | | | |
|---|---|---|---|---|---|---|---|---|
| snoDB0733 | SNORD115-23 | H_sapiens | Animals | mono_intronic | C/D | ENSG00000224078 | SNHG14 | non_coding |
| snoDB0734 | SNORD115-24 | H_sapiens | Animals | mono_intronic | C/D | ENSG00000224078 | SNHG14 | non_coding |
| snoDB0735 | SNORD115-25 | H_sapiens | Animals | mono_intronic | C/D | ENSG00000224078 | SNHG14 | non_coding |
| snoDB0736 | SNORD115-26 | H_sapiens | Animals | mono_intronic | C/D | ENSG00000224078 | SNHG14 | non_coding |
| snoDB0737 | SNORD115-27 | H_sapiens | Animals | mono_intronic | C/D | ENSG00000224078 | SNHG14 | non_coding |
| snoDB0738 | SNORD115-28 | H_sapiens | Animals | mono_intronic | C/D | ENSG00000224078 | SNHG14 | non_coding |
| snoDB0739 | SNORD115-29 | H_sapiens | Animals | mono_intronic | C/D | ENSG00000224078 | SNHG14 | non_coding |
| snoDB0740 | SNORD115-30 | H_sapiens | Animals | mono_intronic | C/D | ENSG00000224078 | SNHG14 | non_coding |
| snoDB0741 | SNORD115-31 | H_sapiens | Animals | mono_intronic | C/D | ENSG00000224078 | SNHG14 | non_coding |
| snoDB0742 | SNORD115-32 | H_sapiens | Animals | mono_intronic | C/D | ENSG00000224078 | SNHG14 | non_coding |
| snoDB0743 | SNORD115-33 | H_sapiens | Animals | mono_intronic | C/D | ENSG00000224078 | SNHG14 | non_coding |
| snoDB0744 | SNORD115-34 | H_sapiens | Animals | mono_intronic | C/D | ENSG00000224078 | SNHG14 | non_coding |
| snoDB0745 | SNORD115-35 | H_sapiens | Animals | mono_intronic | C/D | ENSG00000224078 | SNHG14 | non_coding |
| snoDB0746 | SNORD115-36 | H_sapiens | Animals | mono_intronic | C/D | ENSG00000224078 | SNHG14 | non_coding |
| snoDB0747 | SNORD115-37 | H_sapiens | Animals | mono_intronic | C/D | ENSG00000224078 | SNHG14 | non_coding |
| snoDB0748 | SNORD115-38 | H_sapiens | Animals | mono_intronic | C/D | ENSG00000224078 | SNHG14 | non_coding |
| snoDB0749 | SNORD115-39 | H_sapiens | Animals | mono_intronic | C/D | ENSG00000224078 | SNHG14 | non_coding |
| snoDB0750 | SNORD115-40 | H_sapiens | Animals | mono_intronic | C/D | ENSG00000224078 | SNHG14 | non_coding |
| snoDB0751 | SNORD115-41 | H_sapiens | Animals | mono_intronic | C/D | ENSG00000224078 | SNHG14 | non_coding |
| snoDB0752 | SNORD115-42 | H_sapiens | Animals | mono_intronic | C/D | ENSG00000224078 | SNHG14 | non_coding |
| snoDB0753 | SNORD115-43 | H_sapiens | Animals | mono_intronic | C/D | ENSG00000224078 | SNHG14 | non_coding |
| snoDB0754 | SNORD115-44 | H_sapiens | Animals | mono_intronic | C/D | ENSG00000224078 | SNHG14 | non_coding |
| snoDB0755 | SNORD115-45 | H_sapiens | Animals | mono_intronic | C/D | ENSG00000224078 | SNHG14 | non_coding |
| snoDB0756 | SNORD115-46 | H_sapiens | Animals | mono_exonic | C/D | ENSG00000224078 | SNHG14 | non_coding |
| snoDB0757 | SNORD115-47 | H_sapiens | Animals | mono_intronic | C/D | ENSG00000224078 | SNHG14 | non_coding |
| snoDB0758 | SNORD115-48 | H_sapiens | Animals | mono_intronic | C/D | ENSG00000224078 | SNHG14 | non_coding |
| snoDB0759 | SNORD109B | H_sapiens | Animals | mono_intronic | C/D | ENSG00000224078 | SNHG14 | non_coding |
| snoDB0760 | SNORD13E | H_sapiens | Animals | mono_intronic | C/D | ENSG00000174485 | DENND4A | protein_coding |
| snoDB0761 | U8 | H_sapiens | Animals | mono_intronic | C/D | ENSG00000215304 | | non_coding |
| snoDB0762 | SNORA63 | H_sapiens | Animals | mono_intergenic | H/ACA | | | intergenic |
| snoDB0763 | SNORA18L5 | H_sapiens | Animals | mono_intronic | H/ACA | ENSG00000175344 | CHRNA7 | protein_coding |
| snoDB0764 | SNORA11EL1 | H_sapiens | Animals | mono_intergenic | H/ACA | | | intergenic |
| snoDB0765 | SNORA41B | H_sapiens | Animals | mono_intronic | H/ACA | ENSG00000179362 | HMGN2P46 | non_coding |
| snoDB0766 | snoDB0766 | H_sapiens | Animals | mono_intergenic | H/ACA | | | intergenic |
| snoDB0767 | U8 | H_sapiens | Animals | mono_intronic | C/D | ENSG00000260128 | ULK4P2 | non_coding |
| snoDB0768 | SNORA24B | H_sapiens | Animals | mono_intronic | H/ACA | ENSG00000138617 | PARP16 | protein_coding |
| snoDB0769 | U3 | H_sapiens | Animals | mono_intergenic | C/D | | | intergenic |
| snoDB0770 | SNORD18 | H_sapiens | Animals | mono_intergenic | C/D | | | intergenic |
| snoDB0772 | snoDB0772 | H_sapiens | Animals | mono_intergenic | C/D | | | intergenic |
| snoDB0773 | snoDB0773 | H_sapiens | Animals | mono_intergenic | C/D | | | intergenic |
| snoDB0774 | U3 | H_sapiens | Animals | mono_intergenic | C/D | | | intergenic |
| snoDB0775 | SNORA18L6 | H_sapiens | Animals | mono_intergenic | H/ACA | | | intergenic |
| snoDB0776 | SNORA10 | H_sapiens | Animals | mono_intronic | H/ACA | ENSG00000140988 | RPS2 | protein_coding |
| snoDB0777 | SNORA64 | H_sapiens | Animals | mono_intronic | H/ACA | ENSG00000140988 | RPS2 | protein_coding |
| snoDB0778 | SNORA78 | H_sapiens | Animals | mono_intronic | H/ACA | ENSG00000255198 | SNHG9 | non_coding |

| | | | | | | | | |
|---|---|---|---|---|---|---|---|---|
| snoDB0779 | SNORA27L2 | H_sapiens | Animals | mono_intergenic | H/ACA | | | intergenic |
| snoDB0780 | snoDB0780 | H_sapiens | Animals | mono_intergenic | H/ACA | | | intergenic |
| snoDB0781 | snoDB0781 | H_sapiens | Animals | mono_intergenic | C/D | | | intergenic |
| snoDB0782 | SNORD33 | H_sapiens | Animals | mono_intergenic | C/D | | | intergenic |
| snoDB0783 | SNORA62 | H_sapiens | Animals | mono_intergenic | H/ACA | | | intergenic |
| snoDB0784 | SNORA30 | H_sapiens | Animals | mono_intronic | H/ACA | ENSG00000282034 | | protein_coding |
| snoDB0785 | snoDB0785 | H_sapiens | Animals | mono_intronic | C/D | ENSG00000078328 | RBFOX1 | protein_coding |
| snoDB0786 | snoDB0786 | H_sapiens | Animals | mono_intergenic | H/ACA | | | intergenic |
| snoDB0787 | SNORA25L2 | H_sapiens | Animals | mono_intergenic | H/ACA | | | intergenic |
| snoDB0788 | snoDB0788 | H_sapiens | Animals | mono_intergenic | H/ACA | | | intergenic |
| snoDB0789 | snoDB0789 | H_sapiens | Animals | mono_intergenic | H/ACA | | | intergenic |
| snoDB0790 | SNORD111B | H_sapiens | Animals | mono_intronic | C/D | ENSG00000189091 | SF3B3 | protein_coding |
| snoDB0791 | SNORD111 | H_sapiens | Animals | mono_intronic | C/D | ENSG00000189091 | SF3B3 | protein_coding |
| snoDB0792 | SNORA76 | H_sapiens | Animals | mono_intronic | H/ACA | ENSG00000153774 | CFDP1 | protein_coding |
| snoDB0793 | SNORA48 | H_sapiens | Animals | mono_intronic | H/ACA | ENSG00000072736 | NFATC3 | protein_coding |
| snoDB0794 | SNORA75 | H_sapiens | Animals | mono_intergenic | H/ACA | | | intergenic |
| snoDB0795 | SNORD68 | H_sapiens | Animals | mono_intronic | C/D | ENSG00000167526 | RPL13 | protein_coding |
| snoDB0796 | SNORA70 | H_sapiens | Animals | mono_intergenic | H/ACA | | | intergenic |
| snoDB0797 | SNORA80C | H_sapiens | Animals | mono_intergenic | H/ACA | | | intergenic |
| snoDB0798 | SNORA3C | H_sapiens | Animals | mono_intergenic | H/ACA | | | intergenic |
| snoDB0799 | snoDB0799 | H_sapiens | Animals | intergenic_cluster | H/ACA | | | intergenic |
| snoDB0800 | snoDB0800 | H_sapiens | Animals | intergenic_cluster | H/ACA | | | intergenic |
| snoDB0801 | SNORA46 | H_sapiens | Animals | mono_intronic | H/ACA | ENSG00000125107 | CNOT1 | protein_coding |
| snoDB0802 | SNORA70D | H_sapiens | Animals | mono_intronic | H/ACA | ENSG00000040199 | PHLPP2 | protein_coding |
| snoDB0803 | SNORD71 | H_sapiens | Animals | mono_intronic | C/D | ENSG00000166747 | AP1G1 | protein_coding |
| snoDB0804 | snoDB0804 | H_sapiens | Animals | mono_intergenic | C/D | | | intergenic |
| snoDB0805 | SNORA1L1 | H_sapiens | Animals | mono_intergenic | H/ACA | | | intergenic |
| snoDB0806 | SNORA59B | H_sapiens | Animals | mono_intronic | H/ACA | ENSG00000142494 | SLC47A1 | protein_coding |
| snoDB0807 | SNORD3B-1 | H_sapiens | Animals | mono_intergenic | C/D | | | intergenic |
| snoDB0808 | SNORD3B-2 | H_sapiens | Animals | mono_intergenic | C/D | | | intergenic |
| snoDB0809 | SNORD49B | H_sapiens | Animals | mono_intronic | C/D | ENSG00000175061 | SNHG29 | non_coding |
| snoDB0810 | SNORD49A | H_sapiens | Animals | mono_intronic | C/D | ENSG00000175061 | SNHG29 | non_coding |
| snoDB0811 | SNORD65 | H_sapiens | Animals | mono_intronic | C/D | ENSG00000175061 | SNHG29 | non_coding |
| snoDB0812 | snoDB0812 | H_sapiens | Animals | mono_intergenic | H/ACA | | | intergenic |
| snoDB0813 | SNORA70 | H_sapiens | Animals | mono_intergenic | H/ACA | | | intergenic |
| snoDB0814 | SNORD68L1 | H_sapiens | Animals | mono_intergenic | C/D | | | intergenic |
| snoDB0815 | SNORA70 | H_sapiens | Animals | mono_intronic | H/ACA | ENSG00000263657 | | non_coding |
| snoDB0816 | snoDB0816 | H_sapiens | Animals | mono_intergenic | C/D | | | intergenic |
| snoDB0817 | SNORD63 | H_sapiens | Animals | mono_intergenic | C/D | | | intergenic |
| snoDB0818 | SNORD124 | H_sapiens | Animals | mono_intronic | C/D | ENSG00000008838 | MED24 | protein_coding |
| snoDB0819 | SNORA21B | H_sapiens | Animals | mono_intronic | H/ACA | ENSG00000125691 | RPL23 | protein_coding |
| snoDB0820 | SNORA21 | H_sapiens | Animals | mono_intronic | H/ACA | ENSG00000125691 | RPL23 | protein_coding |
| snoDB0821 | SNORA74 | H_sapiens | Animals | mono_intergenic | H/ACA | | | intergenic |
| snoDB0822 | SNORD7 | H_sapiens | Animals | mono_intronic | C/D | ENSG00000267321 | SNHG30 | non_coding |
| snoDB0823 | U3 | H_sapiens | Animals | mono_intronic | C/D | ENSG00000141293 | SKAP1 | protein_coding |

| | | | | | | | | |
|---|---|---|---|---|---|---|---|---|
| snoDB0824 | snoDB0824 | H_sapiens | Animals | mono_intergenic | C/D | | | intergenic |
| snoDB0825 | SNORA68L4 | H_sapiens | Animals | mono_intergenic | H/ACA | | | intergenic |
| snoDB0827 | U3 | H_sapiens | Animals | mono_intronic | C/D | ENSG00000121101 | TEX14 | protein_coding |
| snoDB0828 | snoDB0828 | H_sapiens | Animals | mono_intergenic | H/ACA | | | intergenic |
| snoDB0830 | SNORA30 | H_sapiens | Animals | mono_intronic | H/ACA | ENSG00000087157 | PGS1 | protein_coding |
| snoDB0831 | SNORA8 | H_sapiens | Animals | mono_intronic | H/ACA | ENSG00000285877 | | non_coding |
| snoDB0832 | SNORA40L6 | H_sapiens | Animals | mono_intergenic | H/ACA | | | intergenic |
| snoDB0833 | SNORA38B | H_sapiens | Animals | mono_intronic | H/ACA | ENSG00000130935 | NOL11 | protein_coding |
| snoDB0834 | snoDB0834 | H_sapiens | Animals | mono_intronic | H/ACA | ENSG00000072210 | ALDH3A2 | protein_coding |
| snoDB0835 | SNORA48 | H_sapiens | Animals | mono_intronic | H/ACA | ENSG00000161960 | EIF4A1 | protein_coding |
| snoDB0836 | SNORD10 | H_sapiens | Animals | mono_intronic | C/D | ENSG00000161960 | EIF4A1 | protein_coding |
| snoDB0837 | SNORA67 | H_sapiens | Animals | mono_intronic | H/ACA | ENSG00000161960 | EIF4A1 | protein_coding |
| snoDB0839 | SNORD118 | H_sapiens | Animals | mono_exonic | C/D | ENSG00000179029 | TMEM107 | protein_coding |
| snoDB0840 | SNORA69L1 | H_sapiens | Animals | mono_intergenic | H/ACA | | | intergenic |
| snoDB0841 | SNORA74 | H_sapiens | Animals | mono_intronic | H/ACA | ENSG00000006695 | COX10 | protein_coding |
| snoDB0842 | SNORD1C | H_sapiens | Animals | mono_intronic | C/D | ENSG00000163597 | SNHG16 | non_coding |
| snoDB0843 | SNORD1B | H_sapiens | Animals | mono_intronic | C/D | ENSG00000163597 | SNHG16 | non_coding |
| snoDB0844 | SNORD1A | H_sapiens | Animals | mono_intronic | C/D | ENSG00000163597 | SNHG16 | non_coding |
| snoDB0846 | SNORD3A | H_sapiens | Animals | mono_intergenic | C/D | | | intergenic |
| snoDB0847 | SNORD3C | H_sapiens | Animals | mono_intergenic | C/D | | | intergenic |
| snoDB0848 | snoDB0848 | H_sapiens | Animals | mono_intronic | C/D | ENSG00000227078 | LINC02094 | non_coding |
| snoDB0849 | SNORD42B | H_sapiens | Animals | mono_intronic | C/D | ENSG00000198242 | RPL23A | protein_coding |
| snoDB0850 | SNORD4A | H_sapiens | Animals | mono_intronic | C/D | ENSG00000198242 | RPL23A | protein_coding |
| snoDB0851 | SNORD42A | H_sapiens | Animals | mono_intronic | C/D | ENSG00000198242 | RPL23A | protein_coding |
| snoDB0852 | SNORD4B | H_sapiens | Animals | mono_intronic | C/D | ENSG00000198242 | RPL23A | protein_coding |
| snoDB0853 | SNORD91B | H_sapiens | Animals | mono_intronic | C/D | ENSG00000167721 | TSR1 | protein_coding |
| snoDB0854 | SNORD91A | H_sapiens | Animals | mono_intronic | C/D | ENSG00000167721 | TSR1 | protein_coding |
| snoDB0855 | SNORD104 | H_sapiens | Animals | mono_intronic | C/D | ENSG00000266402 | SNHG25 | non_coding |
| snoDB0856 | U3 | H_sapiens | Animals | mono_intergenic | C/D | | | intergenic |
| snoDB0857 | U3 | H_sapiens | Animals | mono_intergenic | C/D | | | intergenic |
| snoDB0858 | snoDB0858 | H_sapiens | Animals | mono_intergenic | H/ACA | | | intergenic |
| snoDB0859 | U3 | H_sapiens | Animals | mono_intronic | C/D | ENSG00000173482 | PTPRM | protein_coding |
| snoDB0860 | SNORA48L5 | H_sapiens | Animals | mono_intergenic | H/ACA | | | intergenic |
| snoDB0861 | SNORA73 | H_sapiens | Animals | mono_intronic | H/ACA | ENSG00000101752 | MIB1 | protein_coding |
| snoDB0862 | SNORA37 | H_sapiens | Animals | mono_intronic | H/ACA | ENSG00000134046 | MBD2 | protein_coding |
| snoDB0863 | U3 | H_sapiens | Animals | mono_intronic | C/D | ENSG00000091157 | WDR7 | protein_coding |
| snoDB0866 | SNORA73 | H_sapiens | Animals | mono_intronic | H/ACA | ENSG00000206129 | LINC03069 | non_coding |
| snoDB0867 | SNORA25L10 | H_sapiens | Animals | mono_intergenic | H/ACA | | | intergenic |
| snoDB0868 | SNORD58C | H_sapiens | Animals | mono_intronic | C/D | ENSG00000215472 | RPL17-C18orf32 | protein_coding |
| snoDB0869 | SNORD58A | H_sapiens | Animals | mono_intronic | C/D | ENSG00000215472 | RPL17-C18orf32 | protein_coding |
| snoDB0870 | SNORD58B | H_sapiens | Animals | mono_intronic | C/D | ENSG00000215472 | RPL17-C18orf32 | protein_coding |
| snoDB0871 | U3 | H_sapiens | Animals | mono_exonic | C/D | ENSG00000265369 | PCAT18 | non_coding |
| snoDB0872 | SNORA108 | H_sapiens | Animals | mono_intergenic | H/ACA | | | intergenic |
| snoDB0873 | U8 | H_sapiens | Animals | mono_intergenic | C/D | | | intergenic |
| snoDB0874 | SNORD23 | H_sapiens | Animals | mono_intronic | C/D | ENSG00000265751 | | non_coding |

| | | | | | | | | |
|---|---|---|---|---|---|---|---|---|
| snoDB0875 | snoDB0875 | H_sapiens | Animals | mono_intergenic | H/ACA | | | intergenic |
| snoDB0876 | SNORA70 | H_sapiens | Animals | mono_intergenic | H/ACA | | | intergenic |
| snoDB0877 | SNORA71B | H_sapiens | Animals | mono_intronic | H/ACA | ENSG00000196756 | SNHG17 | non_coding |
| snoDB0878 | SNORD110 | H_sapiens | Animals | mono_intronic | C/D | ENSG00000101361 | NOP56 | protein_coding |
| snoDB0879 | SNORA51 | H_sapiens | Animals | mono_intronic | H/ACA | ENSG00000101361 | NOP56 | protein_coding |
| snoDB0880 | SNORD86 | H_sapiens | Animals | mono_intronic | C/D | ENSG00000101361 | NOP56 | protein_coding |
| snoDB0881 | SNORD56 | H_sapiens | Animals | mono_intronic | C/D | ENSG00000101361 | NOP56 | protein_coding |
| snoDB0882 | SNORD57 | H_sapiens | Animals | mono_intronic | C/D | ENSG00000101361 | NOP56 | protein_coding |
| snoDB0883 | snoDB0883 | H_sapiens | Animals | mono_intergenic | H/ACA | | | intergenic |
| snoDB0884 | U3 | H_sapiens | Animals | mono_intergenic | C/D | | | intergenic |
| snoDB0885 | RNU105B | H_sapiens | Animals | mono_intronic | H/ACA | ENSG00000182621 | PLCB1 | protein_coding |
| snoDB0886 | SNORA40L3 | H_sapiens | Animals | mono_intergenic | H/ACA | | | intergenic |
| snoDB0887 | SNORD56L1 | H_sapiens | Animals | mono_intronic | C/D | ENSG00000088298 | EDEM2 | protein_coding |
| snoDB0888 | SNORA71 | H_sapiens | Animals | mono_intergenic | H/ACA | | | intergenic |
| snoDB0889 | SNORA71E | H_sapiens | Animals | mono_intronic | H/ACA | ENSG00000174365 | SNHG11 | non_coding |
| snoDB0890 | SNORA60 | H_sapiens | Animals | mono_intronic | H/ACA | ENSG00000174365 | SNHG11 | non_coding |
| snoDB0891 | SNORA71 | H_sapiens | Animals | mono_intronic | H/ACA | ENSG00000196756 | SNHG17 | non_coding |
| snoDB0892 | SNORA71A | H_sapiens | Animals | intronic_cluster | H/ACA | ENSG00000196756 | SNHG17 | non_coding |
| snoDB0893 | SNORA71C | H_sapiens | Animals | intronic_cluster | H/ACA | ENSG00000196756 | SNHG17 | non_coding |
| snoDB0894 | SNORA71D | H_sapiens | Animals | mono_intronic | H/ACA | ENSG00000196756 | SNHG17 | non_coding |
| snoDB0895 | U3 | H_sapiens | Animals | mono_intergenic | C/D | | | intergenic |
| snoDB0896 | SNORD17 | H_sapiens | Animals | mono_intronic | C/D | ENSG00000125850 | OVOL2 | protein_coding |
| snoDB0897 | snoDB0897 | H_sapiens | Animals | mono_intergenic | H/ACA | | | intergenic |
| snoDB0898 | SNORA26 | H_sapiens | Animals | mono_intronic | H/ACA | ENSG00000132646 | PCNA | protein_coding |
| snoDB0899 | SNORA26L9 | H_sapiens | Animals | mono_intronic | H/ACA | ENSG00000288843 | | non_coding |
| snoDB0900 | SNORD12C | H_sapiens | Animals | mono_intronic | C/D | ENSG00000177410 | ZFAS1 | non_coding |
| snoDB0901 | SNORD12B | H_sapiens | Animals | mono_intronic | C/D | ENSG00000177410 | ZFAS1 | non_coding |
| snoDB0902 | SNORD12 | H_sapiens | Animals | mono_intronic | C/D | ENSG00000177410 | ZFAS1 | non_coding |
| snoDB0903 | SNORD37 | H_sapiens | Animals | mono_intronic | C/D | ENSG00000167658 | EEF2 | protein_coding |
| snoDB0904 | SNORA68 | H_sapiens | Animals | mono_intronic | H/ACA | ENSG00000105640 | RPL18A | protein_coding |
| snoDB0905 | SNORA68B | H_sapiens | Animals | mono_intronic | H/ACA | ENSG00000105186 | ANKRD27 | protein_coding |
| snoDB0906 | snoDB0906 | H_sapiens | Animals | mono_intronic | C/D | ENSG00000205209 | SCGB2B2 | protein_coding |
| snoDB0907 | SNORA70 | H_sapiens | Animals | mono_intergenic | H/ACA | | | intergenic |
| snoDB0908 | snoDB0908 | H_sapiens | Animals | mono_intergenic | C/D | | | intergenic |
| snoDB0909 | snoDB0909 | H_sapiens | Animals | mono_intronic | C/D | ENSG00000267577 | DNAAF3-AS1 | non_coding |
| snoDB0910 | SNORA70 | H_sapiens | Animals | mono_intergenic | H/ACA | | | intergenic |
| snoDB0911 | SNORD23 | H_sapiens | Animals | mono_intronic | C/D | ENSG00000105373 | NOP53 | protein_coding |
| snoDB0912 | U3 | H_sapiens | Animals | mono_exonic | C/D | ENSG00000161640 | SIGLEC11 | protein_coding |
| snoDB0913 | SNORD88B | H_sapiens | Animals | mono_intronic | C/D | ENSG00000167747 | C19orf48P | non_coding |
| snoDB0914 | SNORD88A | H_sapiens | Animals | mono_intronic | C/D | ENSG00000167747 | C19orf48P | non_coding |
| snoDB0915 | SNORD88C | H_sapiens | Animals | mono_intronic | C/D | ENSG00000167747 | C19orf48P | non_coding |
| snoDB0916 | SNORD105 | H_sapiens | Animals | mono_intronic | C/D | ENSG00000243207 | PPAN-P2RY11 | protein_coding |
| snoDB0917 | SNORD105B | H_sapiens | Animals | mono_intronic | C/D | ENSG00000243207 | PPAN-P2RY11 | protein_coding |
| snoDB0918 | SNORD32A | H_sapiens | Animals | mono_intronic | C/D | ENSG00000142541 | RPL13A | protein_coding |
| snoDB0919 | SNORD33 | H_sapiens | Animals | mono_intronic | C/D | ENSG00000142541 | RPL13A | protein_coding |

| | | | | | | | | |
|---|---|---|---|---|---|---|---|---|
| snoDB0920 | SNORD34 | H_sapiens | Animals | mono_intronic | C/D | ENSG00000142541 | RPL13A | protein_coding |
| snoDB0921 | SNORD35A | H_sapiens | Animals | mono_intronic | C/D | ENSG00000142541 | RPL13A | protein_coding |
| snoDB0922 | SNORD35B | H_sapiens | Animals | mono_intronic | C/D | ENSG00000142534 | RPS11 | protein_coding |
| snoDB0923 | SNORD41 | H_sapiens | Animals | mono_intronic | C/D | ENSG00000105576 | TNPO2 | protein_coding |
| snoDB0924 | snoDB0924 | H_sapiens | Animals | mono_intergenic | C/D | | | intergenic |
| snoDB0925 | snoDB0925 | H_sapiens | Animals | mono_intergenic | H/ACA | | | intergenic |
| snoDB0926 | SNORA70 | H_sapiens | Animals | mono_intergenic | H/ACA | | | intergenic |
| snoDB0927 | SNORA70 | H_sapiens | Animals | mono_intergenic | H/ACA | | | intergenic |
| snoDB0928 | snoDB0928 | H_sapiens | Animals | mono_intergenic | H/ACA | | | intergenic |
| snoDB0929 | snoDB0929 | H_sapiens | Animals | mono_intergenic | C/D | | | intergenic |
| snoDB0930 | SNORD56L3 | H_sapiens | Animals | mono_intergenic | C/D | | | intergenic |
| snoDB0931 | SNORD42 | H_sapiens | Animals | mono_intronic | C/D | ENSG00000100154 | TTC28 | protein_coding |
| snoDB0932 | snoDB0932 | H_sapiens | Animals | mono_intergenic | C/D | | | intergenic |
| snoDB0933 | SNORD83A | H_sapiens | Animals | mono_intronic | C/D | ENSG00000100316 | RPL3 | protein_coding |
| snoDB0934 | SNORD83B | H_sapiens | Animals | mono_intronic | C/D | ENSG00000100316 | RPL3 | protein_coding |
| snoDB0935 | SNORD43 | H_sapiens | Animals | mono_intronic | C/D | ENSG00000100316 | RPL3 | protein_coding |
| snoDB0936 | snoDB0936 | H_sapiens | Animals | mono_intergenic | C/D | | | intergenic |
| snoDB0937 | SNORA77B | H_sapiens | Animals | mono_intronic | H/ACA | ENSG00000099901 | RANBP1 | protein_coding |
| snoDB0938 | SNORD13P1 | H_sapiens | Animals | mono_intergenic | C/D | | | intergenic |
| snoDB0939 | SNORD125 | H_sapiens | Animals | mono_intronic | C/D | ENSG00000100280 | AP1B1 | protein_coding |
| snoDB0940 | SNORA11E | H_sapiens | Animals | mono_intergenic | H/ACA | | | intergenic |
| snoDB0941 | SNORA70 | H_sapiens | Animals | mono_intergenic | H/ACA | | | intergenic |
| snoDB0942 | SNORD74B | H_sapiens | Animals | mono_intergenic | C/D | | | intergenic |
| snoDB0943 | U3 | H_sapiens | Animals | mono_intergenic | C/D | | | intergenic |
| snoDB0944 | SNORA62 | H_sapiens | Animals | mono_intronic | H/ACA | ENSG00000229925 | | non_coding |
| snoDB0945 | SNORA70 | H_sapiens | Animals | mono_intergenic | H/ACA | | | intergenic |
| snoDB0946 | snoDB0946 | H_sapiens | Animals | mono_intronic | H/ACA | ENSG00000142168 | SOD1 | protein_coding |
| snoDB0947 | SNORA80A | H_sapiens | Animals | mono_intronic | H/ACA | ENSG00000142207 | URB1 | protein_coding |
| snoDB0948 | snoDB0948 | H_sapiens | Animals | mono_intergenic | H/ACA | | | intergenic |
| snoDB0949 | snoDB0949 | H_sapiens | Animals | mono_intergenic | H/ACA | | | intergenic |
| snoDB0950 | SNORA32L3 | H_sapiens | Animals | mono_intergenic | H/ACA | | | intergenic |
| snoDB0951 | SNORA51L12 | H_sapiens | Animals | mono_intergenic | H/ACA | | | intergenic |
| snoDB0952 | snoDB0952 | H_sapiens | Animals | mono_exonic | C/D | ENSG00000142185 | TRPM2 | protein_coding |
| snoDB0953 | snoDB0953 | H_sapiens | Animals | mono_exonic | C/D | ENSG00000142185 | TRPM2 | protein_coding |
| snoDB0954 | SNORA11E | H_sapiens | Animals | mono_intronic | H/ACA | ENSG00000180509 | KCNE1 | protein_coding |
| snoDB0955 | SNORD136 | H_sapiens | Animals | mono_intronic | C/D | ENSG00000163938 | GNL3 | protein_coding |
| snoDB0956 | SNORA50D | H_sapiens | Animals | mono_intergenic | H/ACA | | | intergenic |
| snoDB0957 | SNORD51 | H_sapiens | Animals | mono_intronic | C/D | ENSG00000114942 | EEF1B2 | protein_coding |
| snoDB0958 | SNORA50C | H_sapiens | Animals | mono_exonic | H/ACA | ENSG00000266402 | SNHG25 | non_coding |
| snoDB0959 | SNORA43L2 | H_sapiens | Animals | mono_intergenic | H/ACA | | | intergenic |
| snoDB0960 | SNORA50B | H_sapiens | Animals | mono_intronic | H/ACA | ENSG00000133424 | LARGE1 | protein_coding |
| snoDB0961 | SNORD18B | H_sapiens | Animals | mono_intronic | C/D | ENSG00000174444 | RPL4 | protein_coding |
| snoDB0962 | SNORD60 | H_sapiens | Animals | mono_intronic | C/D | ENSG00000260260 | SNHG19 | non_coding |
| snoDB0963 | SNORD3D | H_sapiens | Animals | mono_exonic | C/D | ENSG00000262202 | | non_coding |
| snoDB0964 | SNORA43L4 | H_sapiens | Animals | mono_intergenic | H/ACA | | | intergenic |

| | | | | | | | | |
|---|---|---|---|---|---|---|---|---|
| snoDB0965 | SNORD3E | H_sapiens | Animals | mono_intronic | C/D | ENSG00000120498 | TEX11 | protein_coding |
| snoDB0966 | SNORA16A | H_sapiens | Animals | mono_intronic | H/ACA | ENSG00000197989 | SNHG12 | non_coding |
| snoDB0967 | SNORD38B | H_sapiens | Animals | mono_intronic | C/D | ENSG00000142937 | RPS8 | protein_coding |
| snoDB0968 | SNORA17A | H_sapiens | Animals | mono_intronic | H/ACA | ENSG00000233016 | SNHG7 | non_coding |
| snoDB0969 | U3 | H_sapiens | Animals | mono_intergenic | C/D | | | intergenic |
| snoDB0971 | SNORA17B | H_sapiens | Animals | mono_intronic | H/ACA | ENSG00000233016 | SNHG7 | non_coding |
| snoDB0973 | snoDB0973 | H_sapiens | Animals | mono_intergenic | H/ACA | | | intergenic |
| snoDB0974 | SNORA50A | H_sapiens | Animals | mono_intronic | H/ACA | ENSG00000125107 | CNOT1 | protein_coding |
| snoDB0975 | SNORD167 | H_sapiens | Animals | mono_intronic | C/D | ENSG00000131584 | ACAP3 | protein_coding |
| snoDB0976 | SNORD128 | H_sapiens | Animals | mono_intronic | C/D | ENSG00000142599 | RERE | protein_coding |
| snoDB0977 | SNORD103A | H_sapiens | Animals | mono_intronic | C/D | ENSG00000134644 | PUM1 | protein_coding |
| snoDB0978 | SNORD103B | H_sapiens | Animals | mono_intronic | C/D | ENSG00000134644 | PUM1 | protein_coding |
| snoDB0979 | SNORD103C | H_sapiens | Animals | mono_intronic | C/D | ENSG00000134644 | PUM1 | protein_coding |
| snoDB0980 | SNORA110 | H_sapiens | Animals | mono_intronic | H/ACA | ENSG00000117419 | ERI3 | protein_coding |
| snoDB0981 | SNORD145 | H_sapiens | Animals | mono_intronic | C/D | ENSG00000126106 | TMEM53 | protein_coding |
| snoDB0982 | SNORD160 | H_sapiens | Animals | mono_intronic | C/D | ENSG00000142945 | KIF2C | protein_coding |
| snoDB0983 | SNORD13C | H_sapiens | Animals | mono_intronic | C/D | ENSG00000118298 | CA14 | protein_coding |
| snoDB0986 | SNORD81 | H_sapiens | Animals | mono_intronic | C/D | ENSG00000234741 | GAS5 | non_coding |
| snoDB0987 | SNORD47 | H_sapiens | Animals | mono_intronic | C/D | ENSG00000234741 | GAS5 | non_coding |
| snoDB0988 | SNORD80 | H_sapiens | Animals | mono_intronic | C/D | ENSG00000234741 | GAS5 | non_coding |
| snoDB0989 | SNORD79 | H_sapiens | Animals | mono_intronic | C/D | ENSG00000234741 | GAS5 | non_coding |
| snoDB0990 | SNORD78 | H_sapiens | Animals | mono_intronic | C/D | ENSG00000234741 | GAS5 | non_coding |
| snoDB0991 | SNORD44 | H_sapiens | Animals | mono_intronic | C/D | ENSG00000234741 | GAS5 | non_coding |
| snoDB0992 | SNORA103 | H_sapiens | Animals | mono_exonic | H/ACA | ENSG00000234741 | GAS5 | non_coding |
| snoDB0993 | SNORD77 | H_sapiens | Animals | mono_intronic | C/D | ENSG00000234741 | GAS5 | non_coding |
| snoDB0994 | SNORD76 | H_sapiens | Animals | mono_intronic | C/D | ENSG00000234741 | GAS5 | non_coding |
| snoDB0995 | SNORD75 | H_sapiens | Animals | mono_intronic | C/D | ENSG00000234741 | GAS5 | non_coding |
| snoDB0996 | SNORD74 | H_sapiens | Animals | mono_intronic | C/D | ENSG00000234741 | GAS5 | non_coding |
| snoDB0997 | SNORA100 | H_sapiens | Animals | mono_exonic | H/ACA | ENSG00000153187 | HNRNPU | protein_coding |
| snoDB0998 | SNORA112 | H_sapiens | Animals | mono_intronic | H/ACA | ENSG00000204685 | STARD7-AS1 | non_coding |
| snoDB0999 | SNORD132 | H_sapiens | Animals | mono_intronic | C/D | ENSG00000169679 | BUB1 | protein_coding |
| snoDB1000 | SNORD3K | H_sapiens | Animals | mono_intergenic | C/D | | | intergenic |
| snoDB1001 | SNORA105B | H_sapiens | Animals | mono_exonic | H/ACA | ENSG00000144381 | HSPD1 | protein_coding |
| snoDB1002 | SNORA115 | H_sapiens | Animals | mono_intronic | H/ACA | ENSG00000079308 | TNS1 | protein_coding |
| snoDB1003 | SNORA93 | H_sapiens | Animals | mono_intronic | H/ACA | ENSG00000163520 | FBLN2 | protein_coding |
| snoDB1004 | SNORD146 | H_sapiens | Animals | mono_intronic | C/D | ENSG00000173473 | SMARCC1 | protein_coding |
| snoDB1005 | SNORA94 | H_sapiens | Animals | mono_intronic | H/ACA | ENSG00000010256 | UQCRC1 | protein_coding |
| snoDB1006 | SNORA95 | H_sapiens | Animals | mono_intergenic | H/ACA | | | intergenic |
| snoDB1007 | SNORD155 | H_sapiens | Animals | mono_intronic | C/D | ENSG00000185565 | LSAMP | protein_coding |
| snoDB1008 | SNORD3P4 | H_sapiens | Animals | mono_intergenic | C/D | | | intergenic |
| snoDB1009 | SNORD162 | H_sapiens | Animals | mono_intronic | C/D | ENSG00000247708 | STX18-AS1 | non_coding |
| snoDB1010 | SNORD161 | H_sapiens | Animals | mono_intronic | C/D | ENSG00000138759 | FRAS1 | protein_coding |
| snoDB1011 | SNORD143 | H_sapiens | Animals | intronic_cluster | C/D | ENSG00000138674 | SEC31A | protein_coding |
| snoDB1012 | SNORD144 | H_sapiens | Animals | intronic_cluster | C/D | ENSG00000138674 | SEC31A | protein_coding |
| snoDB1013 | SNORA101A | H_sapiens | Animals | mono_intergenic | H/ACA | | | intergenic |

| | | | | | | | | |
|---|---|---|---|---|---|---|---|---|
| snoDB1014 | SNORD170 | H_sapiens | Animals | mono_intronic | C/D | ENSG00000038382 | TRIO | protein_coding |
| snoDB1015 | SNORD141B | H_sapiens | Animals | mono_exonic | C/D | ENSG00000250182 | EEF1A1P13 | non_coding |
| snoDB1016 | SNORA105A | H_sapiens | Animals | mono_intergenic | H/ACA | | | intergenic |
| snoDB1017 | SNORD13B-1 | H_sapiens | Animals | mono_intergenic | C/D | | | intergenic |
| snoDB1018 | SNORD13B-2 | H_sapiens | Animals | mono_intergenic | C/D | | | intergenic |
| snoDB1020 | SNORD156 | H_sapiens | Animals | mono_intronic | C/D | ENSG00000111799 | COL12A1 | protein_coding |
| snoDB1021 | SNORD50A | H_sapiens | Animals | mono_intronic | C/D | ENSG00000271793 | | protein_coding |
| snoDB1022 | SNORD166 | H_sapiens | Animals | mono_intronic | C/D | ENSG00000112394 | SLC16A10 | protein_coding |
| snoDB1023 | SNORA98 | H_sapiens | Animals | mono_intronic | H/ACA | ENSG00000152818 | UTRN | protein_coding |
| snoDB1024 | SNORA116 | H_sapiens | Animals | mono_intronic | H/ACA | ENSG00000130338 | TULP4 | protein_coding |
| snoDB1025 | SNORA114 | H_sapiens | Animals | mono_intronic | H/ACA | ENSG00000002822 | MAD1L1 | protein_coding |
| snoDB1026 | SNORD13P2 | H_sapiens | Animals | mono_intronic | C/D | ENSG00000164916 | FOXK1 | protein_coding |
| snoDB1027 | SNORD165 | H_sapiens | Animals | mono_intronic | C/D | ENSG00000157927 | RADIL | protein_coding |
| snoDB1028 | SNORD151 | H_sapiens | Animals | mono_intronic | C/D | ENSG00000136205 | TNS3 | protein_coding |
| snoDB1030 | SNORD3I | H_sapiens | Animals | mono_intergenic | C/D | | | intergenic |
| snoDB1031 | SNORA99 | H_sapiens | Animals | mono_intronic | H/ACA | ENSG00000136574 | GATA4 | protein_coding |
| snoDB1032 | SNORD3F | H_sapiens | Animals | mono_intergenic | C/D | | | intergenic |
| snoDB1033 | SNORD173 | H_sapiens | Animals | mono_intronic | C/D | ENSG00000164930 | FZD6 | protein_coding |
| snoDB1034 | SNORD168 | H_sapiens | Animals | intronic_cluster | C/D | ENSG00000182197 | EXT1 | protein_coding |
| snoDB1035 | SNORD149 | H_sapiens | Animals | mono_intronic | C/D | ENSG00000014164 | ZC3H3 | protein_coding |
| snoDB1036 | SNORD137 | H_sapiens | Animals | mono_intronic | C/D | ENSG00000285637 | | non_coding |
| snoDB1037 | SNORD141A | H_sapiens | Animals | mono_exonic | C/D | ENSG00000196205 | EEF1A1P5 | non_coding |
| snoDB1038 | SNORD142 | H_sapiens | Animals | mono_intronic | C/D | ENSG00000067057 | PFKP | protein_coding |
| snoDB1039 | SNORD129 | H_sapiens | Animals | mono_intronic | C/D | ENSG00000198879 | SFMBT2 | protein_coding |
| snoDB1040 | SNORD130 | H_sapiens | Animals | mono_intronic | C/D | ENSG00000150054 | MPP7 | protein_coding |
| snoDB1041 | SNORA86 | H_sapiens | Animals | mono_exonic | H/ACA | ENSG00000150093 | ITGB1 | protein_coding |
| snoDB1042 | SNORD172 | H_sapiens | Animals | mono_intronic | C/D | ENSG00000156650 | KAT6B | protein_coding |
| snoDB1043 | SNORA87 | H_sapiens | Animals | mono_intronic | H/ACA | ENSG00000148737 | TCF7L2 | protein_coding |
| snoDB1044 | SNORD158 | H_sapiens | Animals | mono_intronic | C/D | ENSG00000151892 | GFRA1 | protein_coding |
| snoDB1045 | SNORD131 | H_sapiens | Animals | mono_intronic | C/D | ENSG00000214026 | MRPL23 | protein_coding |
| snoDB1046 | SNORD147 | H_sapiens | Animals | mono_intronic | C/D | ENSG00000166689 | PLEKHA7 | protein_coding |
| snoDB1047 | SNORA88 | H_sapiens | Animals | mono_intronic | H/ACA | ENSG00000227160 | THEM7P | non_coding |
| snoDB1048 | SNORD164 | H_sapiens | Animals | mono_intronic | C/D | ENSG00000026508 | CD44 | protein_coding |
| snoDB1049 | SNORD31 | H_sapiens | Animals | mono_intronic | C/D | ENSG00000255717 | SNHG1 | non_coding |
| snoDB1050 | SNORD29 | H_sapiens | Animals | mono_intronic | C/D | ENSG00000255717 | SNHG1 | non_coding |
| snoDB1051 | SNORD13F | H_sapiens | Animals | mono_intronic | C/D | ENSG00000087365 | SF3B2 | protein_coding |
| snoDB1052 | SNORD13I | H_sapiens | Animals | mono_intronic | C/D | ENSG00000165895 | ARHGAP42 | protein_coding |
| snoDB1053 | SNORD150 | H_sapiens | Animals | mono_intronic | C/D | ENSG00000248712 | CCDC153 | protein_coding |
| snoDB1054 | SNORD153 | H_sapiens | Animals | mono_intronic | C/D | ENSG00000151503 | NCAPD3 | protein_coding |
| snoDB1055 | SNORA120 | H_sapiens | Animals | mono_intronic | H/ACA | ENSG00000110799 | VWF | protein_coding |
| snoDB1056 | SNORD133 | H_sapiens | Animals | mono_intronic | C/D | ENSG00000161813 | LARP4 | protein_coding |
| snoDB1057 | SNORA105C | H_sapiens | Animals | mono_intergenic | H/ACA | | | intergenic |
| snoDB1058 | SNORD59B | H_sapiens | Animals | mono_intronic | C/D | ENSG00000110955 | ATP5F1B | protein_coding |
| snoDB1059 | SNORA113 | H_sapiens | Animals | mono_intronic | H/ACA | ENSG00000166225 | FRS2 | protein_coding |
| snoDB1060 | SNORA107 | H_sapiens | Animals | mono_intergenic | H/ACA | | | intergenic |

| | | | | | | | | |
|---|---|---|---|---|---|---|---|---|
| snoDB1061 | SNORD13G | H_sapiens | Animals | mono_intergenic | C/D | | | intergenic |
| snoDB1062 | SNORA89 | H_sapiens | Animals | mono_intronic | H/ACA | ENSG00000151322 | NPAS3 | protein_coding |
| snoDB1063 | SNORA101B | H_sapiens | Animals | mono_intronic | H/ACA | ENSG00000174373 | RALGAPA1 | protein_coding |
| snoDB1064 | SNORD169 | H_sapiens | Animals | mono_intronic | C/D | ENSG00000198732 | SMOC1 | protein_coding |
| snoDB1065 | SNORD3P3 | H_sapiens | Animals | mono_intergenic | C/D | | | intergenic |
| snoDB1066 | SNORD114-8 | H_sapiens | Animals | intronic_cluster | C/D | ENSG00000225746 | MEG8 | non_coding |
| snoDB1067 | SNORD148 | H_sapiens | Animals | mono_intronic | C/D | ENSG00000166123 | GPT2 | protein_coding |
| snoDB1068 | SNORD13H | H_sapiens | Animals | mono_intronic | C/D | ENSG00000181019 | NQO1 | protein_coding |
| snoDB1069 | SNORA119 | H_sapiens | Animals | mono_intronic | H/ACA | ENSG00000140995 | DEF8 | protein_coding |
| snoDB1070 | SNORD163 | H_sapiens | Animals | mono_intronic | C/D | ENSG00000141027 | NCOR1 | protein_coding |
| snoDB1071 | SNORA90 | H_sapiens | Animals | mono_intergenic | H/ACA | | | intergenic |
| snoDB1072 | SNORD134 | H_sapiens | Animals | mono_intronic | C/D | ENSG00000169710 | FASN | protein_coding |
| snoDB1073 | SNORA111 | H_sapiens | Animals | mono_intronic | H/ACA | ENSG00000101489 | CELF4 | protein_coding |
| snoDB1074 | SNORD135 | H_sapiens | Animals | mono_intronic | C/D | ENSG00000105576 | TNPO2 | protein_coding |
| snoDB1075 | SNORA104 | H_sapiens | Animals | mono_intergenic | H/ACA | | | intergenic |
| snoDB1076 | SNORA118 | H_sapiens | Animals | mono_intronic | H/ACA | ENSG00000099331 | MYO9B | protein_coding |
| snoDB1077 | SNORD152 | H_sapiens | Animals | mono_intronic | C/D | ENSG00000105738 | SIPA1L3 | protein_coding |
| snoDB1078 | SNORD175 | H_sapiens | Animals | mono_intronic | C/D | ENSG00000196235 | SUPT5H | protein_coding |
| snoDB1079 | SNORD157 | H_sapiens | Animals | mono_intronic | C/D | ENSG00000108106 | UBE2S | protein_coding |
| snoDB1080 | SNORD119 | H_sapiens | Animals | mono_intronic | C/D | ENSG00000125835 | SNRPB | protein_coding |
| snoDB1081 | SNORD154 | H_sapiens | Animals | mono_intergenic | C/D | | | intergenic |
| snoDB1082 | SNORA117 | H_sapiens | Animals | mono_intronic | H/ACA | ENSG00000101191 | DIDO1 | protein_coding |
| snoDB1083 | SNORA91 | H_sapiens | Animals | mono_intronic | H/ACA | ENSG00000157617 | C2CD2 | protein_coding |
| snoDB1084 | SNORD159 | H_sapiens | Animals | mono_intronic | C/D | ENSG00000160285 | LSS | protein_coding |
| snoDB1085 | SNORA92 | H_sapiens | Animals | mono_intronic | H/ACA | ENSG00000198792 | TMEM184B | protein_coding |
| snoDB1086 | SNORD139 | H_sapiens | Animals | mono_exonic | C/D | ENSG00000100316 | RPL3 | protein_coding |
| snoDB1087 | SNORD140 | H_sapiens | Animals | mono_intergenic | C/D | | | intergenic |
| snoDB1088 | SNORA109 | H_sapiens | Animals | mono_intergenic | H/ACA | | | intergenic |
| snoDB1089 | snoDB1089 | H_sapiens | Animals | mono_intronic | C/D | ENSG00000225830 | ERCC6 | protein_coding |
| snoDB1091 | snoDB1091 | H_sapiens | Animals | mono_intronic | C/D | ENSG00000149639 | SOGA1 | protein_coding |
| snoDB1092 | snoDB1092 | H_sapiens | Animals | mono_intronic | C/D | ENSG00000163882 | POLR2H | protein_coding |
| snoDB1093 | snoDB1093 | H_sapiens | Animals | mono_intronic | C/D | ENSG00000166949 | SMAD3 | protein_coding |
| snoDB1094 | snoDB1094 | H_sapiens | Animals | mono_intronic | C/D | ENSG00000198648 | STK39 | protein_coding |
| snoDB1097 | snoDB1097 | H_sapiens | Animals | mono_intronic | C/D | ENSG00000066654 | THUMPD1 | protein_coding |
| snoDB1098 | snoDB1098 | H_sapiens | Animals | mono_intronic | C/D | ENSG00000083307 | GRHL2 | protein_coding |
| snoDB1099 | snoDB1099 | H_sapiens | Animals | mono_intronic | C/D | ENSG00000174516 | PELI3 | protein_coding |
| snoDB1104 | snoDB1104 | H_sapiens | Animals | mono_intronic | C/D | ENSG00000107959 | PITRM1 | protein_coding |
| snoDB1106 | snoDB1106 | H_sapiens | Animals | mono_intronic | C/D | ENSG00000149177 | PTPRJ | protein_coding |
| snoDB1107 | snoDB1107 | H_sapiens | Animals | mono_intronic | C/D | ENSG00000071205 | ARHGAP10 | protein_coding |
| snoDB1109 | snoDB1109 | H_sapiens | Animals | mono_intronic | C/D | ENSG00000118058 | KMT2A | protein_coding |
| snoDB1110 | snoDB1110 | H_sapiens | Animals | mono_intronic | C/D | ENSG00000197694 | SPTAN1 | protein_coding |
| snoDB1112 | snoDB1112 | H_sapiens | Animals | mono_intronic | C/D | ENSG00000250241 | PCDH10-DT | non_coding |
| snoDB1113 | snoDB1113 | H_sapiens | Animals | mono_intronic | C/D | ENSG00000188487 | INSC | protein_coding |
| snoDB1114 | snoDB1114 | H_sapiens | Animals | mono_intronic | C/D | ENSG00000146477 | SLC22A3 | protein_coding |
| snoDB1115 | snoDB1115 | H_sapiens | Animals | mono_intronic | C/D | ENSG00000103091 | WDR59 | protein_coding |

| | | | | | | | | |
|---|---|---|---|---|---|---|---|---|
| snoDB1118 | snoDB1118 | H_sapiens | Animals | mono_intronic | C/D | ENSG00000131473 | ACLY | protein_coding |
| snoDB1119 | snoDB1119 | H_sapiens | Animals | mono_intronic | C/D | ENSG00000291072 | CROCCP2 | non_coding |
| snoDB1120 | snoDB1120 | H_sapiens | Animals | mono_intronic | C/D | ENSG00000138119 | MYOF | protein_coding |
| snoDB1122 | snoDB1122 | H_sapiens | Animals | mono_intronic | C/D | ENSG00000067057 | PFKP | protein_coding |
| snoDB1124 | snoDB1124 | H_sapiens | Animals | mono_intronic | C/D | ENSG00000288622 | PDCD6-AHRR | protein_coding |
| snoDB1129 | snoDB1129 | H_sapiens | Animals | mono_intronic | C/D | ENSG00000105053 | VRK3 | protein_coding |
| snoDB1130 | snoDB1130 | H_sapiens | Animals | mono_intronic | C/D | ENSG00000152127 | MGAT5 | protein_coding |
| snoDB1133 | snoDB1133 | H_sapiens | Animals | mono_intronic | C/D | ENSG00000163638 | ADAMTS9 | protein_coding |
| snoDB1134 | snoDB1134 | H_sapiens | Animals | mono_intergenic | C/D | | | intergenic |
| snoDB1135 | snoDB1135 | H_sapiens | Animals | mono_intronic | C/D | ENSG00000137693 | YAP1 | protein_coding |
| snoDB1214 | snoDB1214 | H_sapiens | Animals | mono_intronic | H/ACA | ENSG00000287017 | | non_coding |
| snoDB1215 | snoDB1215 | H_sapiens | Animals | mono_intronic | H/ACA | ENSG00000287017 | | non_coding |
| snoDB1216 | snoDB1216 | H_sapiens | Animals | mono_intergenic | H/ACA | | | intergenic |
| snoDB1217 | snoDB1217 | H_sapiens | Animals | mono_intronic | H/ACA | ENSG00000176658 | MYO1D | protein_coding |
| snoDB1218 | snoDB1218 | H_sapiens | Animals | mono_intronic | H/ACA | ENSG00000170322 | NFRKB | protein_coding |
| snoDB1219 | snoDB1219 | H_sapiens | Animals | mono_intronic | C/D | ENSG00000035681 | NSMAF | protein_coding |
| snoDB1222 | snoDB1222 | H_sapiens | Animals | mono_intronic | H/ACA | ENSG00000164305 | CASP3 | protein_coding |
| snoDB1223 | snoDB1223 | H_sapiens | Animals | mono_exonic | C/D | ENSG00000176986 | SEC24C | protein_coding |
| snoDB1224 | snoDB1224 | H_sapiens | Animals | mono_intronic | C/D | ENSG00000119772 | DNMT3A | protein_coding |
| snoDB1225 | snoDB1225 | H_sapiens | Animals | mono_intronic | C/D | ENSG00000165288 | BRWD3 | protein_coding |
| snoDB1226 | snoDB1226 | H_sapiens | Animals | mono_intronic | C/D | ENSG00000249115 | HAUS5 | protein_coding |
| snoDB1227 | snoDB1227 | H_sapiens | Animals | mono_intronic | C/D | ENSG00000141068 | KSR1 | protein_coding |
| snoDB1228 | snoDB1228 | H_sapiens | Animals | mono_intronic | C/D | ENSG00000204387 | SNHG32 | non_coding |
| snoDB1229 | snoDB1229 | H_sapiens | Animals | mono_intronic | C/D | ENSG00000148719 | DNAJB12 | protein_coding |
| snoDB1231 | snoDB1231 | H_sapiens | Animals | intronic_cluster | C/D | ENSG00000158813 | EDA | protein_coding |
| snoDB1233 | snoDB1233 | H_sapiens | Animals | mono_intronic | C/D | ENSG00000266967 | AARSD1 | protein_coding |
| snoDB1234 | snoDB1234 | H_sapiens | Animals | mono_intronic | C/D | ENSG00000140525 | FANCI | protein_coding |
| snoDB1237 | snoDB1237 | H_sapiens | Animals | mono_intronic | C/D | ENSG00000146592 | CREB5 | protein_coding |
| snoDB1240 | snoDB1240 | H_sapiens | Animals | mono_intronic | C/D | ENSG00000103274 | NUBP1 | protein_coding |
| snoDB1242 | snoDB1242 | H_sapiens | Animals | mono_intronic | C/D | ENSG00000112685 | EXOC2 | protein_coding |
| snoDB1243 | snoDB1243 | H_sapiens | Animals | mono_intronic | C/D | ENSG00000109501 | WFS1 | protein_coding |
| snoDB1244 | snoDB1244 | H_sapiens | Animals | mono_intronic | C/D | ENSG00000130783 | CCDC62 | protein_coding |
| snoDB1247 | snoDB1247 | H_sapiens | Animals | mono_intronic | C/D | ENSG00000275835 | TUBGCP5 | protein_coding |
| snoDB1248 | snoDB1248 | H_sapiens | Animals | mono_intronic | C/D | ENSG00000139687 | RB1 | protein_coding |
| snoDB1249 | snoDB1249 | H_sapiens | Animals | mono_intronic | C/D | ENSG00000120948 | TARDBP | protein_coding |
| snoDB1250 | snoDB1250 | H_sapiens | Animals | mono_intronic | C/D | ENSG00000177169 | ULK1 | protein_coding |
| snoDB1252 | snoDB1252 | H_sapiens | Animals | mono_intronic | C/D | ENSG00000130254 | SAFB2 | protein_coding |
| snoDB1253 | snoDB1253 | H_sapiens | Animals | mono_intronic | C/D | ENSG00000198563 | DDX39B | protein_coding |
| snoDB1255 | snoDB1255 | H_sapiens | Animals | mono_intronic | C/D | ENSG00000163811 | WDR43 | protein_coding |
| snoDB1257 | snoDB1257 | H_sapiens | Animals | mono_intronic | C/D | ENSG00000171843 | MLLT3 | protein_coding |
| snoDB1259 | snoDB1259 | H_sapiens | Animals | mono_intronic | C/D | ENSG00000130881 | LRP3 | protein_coding |
| snoDB1260 | snoDB1260 | H_sapiens | Animals | mono_intronic | C/D | ENSG00000083544 | TDRD3 | protein_coding |
| snoDB1261 | snoDB1261 | H_sapiens | Animals | mono_intronic | C/D | ENSG00000176124 | DLEU1 | non_coding |
| snoDB1263 | snoDB1263 | H_sapiens | Animals | mono_intronic | C/D | ENSG00000096063 | SRPK1 | protein_coding |
| snoDB1264 | snoDB1264 | H_sapiens | Animals | mono_intronic | C/D | ENSG00000204634 | TBC1D8 | protein_coding |

| | | | | | | | | |
|---|---|---|---|---|---|---|---|---|
| snoDB1265 | snoDB1265 | H_sapiens | Animals | mono_intronic | C/D | ENSG00000140470 | ADAMTS17 | protein_coding |
| snoDB1266 | snoDB1266 | H_sapiens | Animals | mono_intronic | C/D | ENSG00000088854 | DNAAF9 | protein_coding |
| snoDB1269 | snoDB1269 | H_sapiens | Animals | mono_intronic | C/D | ENSG00000130294 | KIF1A | protein_coding |
| snoDB1270 | snoDB1270 | H_sapiens | Animals | mono_intronic | H/ACA | ENSG00000196369 | SRGAP2B | protein_coding |
| snoDB1271 | snoDB1271 | H_sapiens | Animals | mono_intronic | H/ACA | ENSG00000101266 | CSNK2A1 | protein_coding |
| snoDB1272 | snoDB1272 | H_sapiens | Animals | mono_intronic | H/ACA | ENSG00000117000 | RLF | protein_coding |
| snoDB1273 | snoDB1273 | H_sapiens | Animals | mono_intronic | H/ACA | ENSG00000080298 | RFX3 | protein_coding |
| snoDB1274 | snoDB1274 | H_sapiens | Animals | mono_intronic | H/ACA | ENSG00000285756 | | non_coding |
| snoDB1275 | snoDB1275 | H_sapiens | Animals | mono_intronic | H/ACA | ENSG00000285756 | | non_coding |
| snoDB1276 | snoDB1276 | H_sapiens | Animals | mono_intronic | H/ACA | ENSG00000125826 | RBCK1 | protein_coding |
| snoDB1277 | snoDB1277 | H_sapiens | Animals | mono_intronic | H/ACA | ENSG00000065243 | PKN2 | protein_coding |
| snoDB1278 | snoDB1278 | H_sapiens | Animals | mono_intronic | H/ACA | ENSG00000125954 | CHURC1-FNTB | protein_coding |
| snoDB1279 | snoDB1279 | H_sapiens | Animals | mono_intronic | H/ACA | ENSG00000128881 | TTBK2 | protein_coding |
| snoDB1280 | snoDB1280 | H_sapiens | Animals | mono_intronic | H/ACA | ENSG00000122779 | TRIM24 | protein_coding |
| snoDB1281 | snoDB1281 | H_sapiens | Animals | mono_intronic | H/ACA | ENSG00000147912 | FBXO10 | protein_coding |
| snoDB1282 | snoDB1282 | H_sapiens | Animals | mono_intronic | H/ACA | ENSG00000197386 | HTT | protein_coding |
| snoDB1283 | snoDB1283 | H_sapiens | Animals | mono_intronic | H/ACA | ENSG00000170100 | ZNF778 | protein_coding |
| snoDB1284 | snoDB1284 | H_sapiens | Animals | mono_intronic | H/ACA | ENSG00000131941 | RHPN2 | protein_coding |
| snoDB1285 | snoDB1285 | H_sapiens | Animals | mono_intronic | H/ACA | ENSG00000066427 | ATXN3 | protein_coding |
| snoDB1286 | snoDB1286 | H_sapiens | Animals | mono_intronic | H/ACA | ENSG00000266028 | SRGAP2 | protein_coding |
| snoDB1287 | snoDB1287 | H_sapiens | Animals | mono_intronic | H/ACA | ENSG00000005339 | CREBBP | protein_coding |
| snoDB1288 | snoDB1288 | H_sapiens | Animals | mono_intronic | H/ACA | ENSG00000116106 | EPHA4 | protein_coding |
| snoDB1289 | snoDB1289 | H_sapiens | Animals | mono_intronic | H/ACA | ENSG00000178307 | TMEM11 | protein_coding |
| snoDB1290 | snoDB1290 | H_sapiens | Animals | mono_intronic | H/ACA | ENSG00000113615 | SEC24A | protein_coding |
| snoDB1291 | snoDB1291 | H_sapiens | Animals | mono_intronic | H/ACA | ENSG00000143799 | PARP1 | protein_coding |
| snoDB1292 | snoDB1292 | H_sapiens | Animals | mono_intronic | H/ACA | ENSG00000036054 | TBC1D23 | protein_coding |
| snoDB1293 | snoDB1293 | H_sapiens | Animals | mono_intronic | H/ACA | ENSG00000248333 | CDK11B | protein_coding |
| snoDB1294 | snoDB1294 | H_sapiens | Animals | mono_intronic | H/ACA | ENSG00000285756 | | non_coding |
| snoDB1295 | snoDB1295 | H_sapiens | Animals | intronic_cluster | H/ACA | ENSG00000187231 | SESTD1 | protein_coding |
| snoDB1296 | snoDB1296 | H_sapiens | Animals | mono_intronic | H/ACA | ENSG00000124831 | LRRFIP1 | protein_coding |
| snoDB1297 | snoDB1297 | H_sapiens | Animals | mono_intronic | H/ACA | ENSG00000068654 | POLR1A | protein_coding |
| snoDB1298 | snoDB1298 | H_sapiens | Animals | mono_intronic | H/ACA | ENSG00000123094 | RASSF8 | protein_coding |
| snoDB1299 | snoDB1299 | H_sapiens | Animals | mono_intronic | H/ACA | ENSG00000152894 | PTPRK | protein_coding |
| snoDB1300 | snoDB1300 | H_sapiens | Animals | mono_intronic | H/ACA | ENSG00000107625 | DDX50 | protein_coding |
| snoDB1301 | snoDB1301 | H_sapiens | Animals | mono_intronic | H/ACA | ENSG00000012983 | MAP4K5 | protein_coding |
| snoDB1302 | snoDB1302 | H_sapiens | Animals | mono_intronic | H/ACA | ENSG00000152104 | PTPN14 | protein_coding |
| snoDB1303 | snoDB1303 | H_sapiens | Animals | mono_intronic | H/ACA | ENSG00000011243 | AKAP8L | protein_coding |
| snoDB1304 | snoDB1304 | H_sapiens | Animals | mono_intronic | H/ACA | ENSG00000172530 | BANP | protein_coding |
| snoDB1305 | snoDB1305 | H_sapiens | Animals | mono_intronic | H/ACA | ENSG00000156795 | NTAQ1 | protein_coding |
| snoDB1306 | snoDB1306 | H_sapiens | Animals | mono_intronic | H/ACA | ENSG00000057608 | GDI2 | protein_coding |
| snoDB1307 | snoDB1307 | H_sapiens | Animals | mono_intronic | H/ACA | ENSG00000142002 | DPP9 | protein_coding |
| snoDB1308 | snoDB1308 | H_sapiens | Animals | mono_intronic | H/ACA | ENSG00000165219 | GAPVD1 | protein_coding |
| snoDB1309 | snoDB1309 | H_sapiens | Animals | mono_intronic | H/ACA | ENSG00000169371 | SNUPN | protein_coding |
| snoDB1310 | snoDB1310 | H_sapiens | Animals | mono_intronic | H/ACA | ENSG00000134717 | BTF3L4 | protein_coding |
| snoDB1311 | snoDB1311 | H_sapiens | Animals | mono_intronic | H/ACA | ENSG00000131591 | C1orf159 | protein_coding |

| | | | | | | | | |
|---|---|---|---|---|---|---|---|---|
| snoDB1312 | snoDB1312 | H_sapiens | Animals | mono_intronic | H/ACA | ENSG00000131473 | ACLY | protein_coding |
| snoDB1313 | snoDB1313 | H_sapiens | Animals | mono_intronic | H/ACA | ENSG00000164961 | WASHC5 | protein_coding |
| snoDB1314 | snoDB1314 | H_sapiens | Animals | mono_intronic | H/ACA | ENSG00000164053 | ATRIP | protein_coding |
| snoDB1315 | snoDB1315 | H_sapiens | Animals | mono_intronic | H/ACA | ENSG00000172197 | MBOAT1 | protein_coding |
| snoDB1316 | snoDB1316 | H_sapiens | Animals | mono_intronic | H/ACA | ENSG00000101052 | IFT52 | protein_coding |
| snoDB1317 | snoDB1317 | H_sapiens | Animals | mono_intronic | H/ACA | ENSG00000103978 | TMEM87A | protein_coding |
| snoDB1318 | snoDB1318 | H_sapiens | Animals | mono_intronic | H/ACA | ENSG00000113312 | TTC1 | protein_coding |
| snoDB1319 | snoDB1319 | H_sapiens | Animals | mono_intronic | H/ACA | ENSG00000143569 | UBAP2L | protein_coding |
| snoDB1320 | snoDB1320 | H_sapiens | Animals | mono_intronic | H/ACA | ENSG00000137574 | TGS1 | protein_coding |
| snoDB1321 | snoDB1321 | H_sapiens | Animals | mono_intronic | H/ACA | ENSG00000079805 | DNM2 | protein_coding |
| snoDB1322 | snoDB1322 | H_sapiens | Animals | mono_intronic | H/ACA | ENSG00000125482 | TTF1 | protein_coding |
| snoDB1323 | snoDB1323 | H_sapiens | Animals | mono_intronic | H/ACA | ENSG00000180104 | EXOC3 | protein_coding |
| snoDB1324 | snoDB1324 | H_sapiens | Animals | mono_intronic | H/ACA | ENSG00000120925 | RNF170 | protein_coding |
| snoDB1325 | snoDB1325 | H_sapiens | Animals | mono_intronic | H/ACA | ENSG00000100852 | ARHGAP5 | protein_coding |
| snoDB1326 | snoDB1326 | H_sapiens | Animals | mono_intronic | H/ACA | ENSG00000068354 | TBC1D25 | protein_coding |
| snoDB1327 | snoDB1327 | H_sapiens | Animals | mono_intronic | H/ACA | ENSG00000198742 | SMURF1 | protein_coding |
| snoDB1328 | snoDB1328 | H_sapiens | Animals | mono_intronic | H/ACA | ENSG00000179889 | PDXDC1 | protein_coding |
| snoDB1329 | snoDB1329 | H_sapiens | Animals | mono_intronic | H/ACA | ENSG00000157106 | SMG1 | protein_coding |
| snoDB1330 | snoDB1330 | H_sapiens | Animals | mono_intronic | H/ACA | ENSG00000107566 | ERLIN1 | protein_coding |
| snoDB1331 | snoDB1331 | H_sapiens | Animals | mono_intronic | H/ACA | ENSG00000274322 | | protein_coding |
| snoDB1332 | snoDB1332 | H_sapiens | Animals | mono_intronic | H/ACA | ENSG00000165733 | BMS1 | protein_coding |
| snoDB1333 | snoDB1333 | H_sapiens | Animals | mono_intronic | H/ACA | ENSG00000164808 | SPIDR | protein_coding |
| snoDB1334 | snoDB1334 | H_sapiens | Animals | mono_intronic | H/ACA | ENSG00000148411 | NACC2 | protein_coding |
| snoDB1335 | snoDB1335 | H_sapiens | Animals | mono_intronic | H/ACA | ENSG00000100592 | DAAM1 | protein_coding |
| snoDB1336 | snoDB1336 | H_sapiens | Animals | mono_intronic | H/ACA | ENSG00000171444 | MCC | protein_coding |
| snoDB1337 | snoDB1337 | H_sapiens | Animals | mono_intronic | H/ACA | ENSG00000198909 | MAP3K3 | protein_coding |
| snoDB1338 | snoDB1338 | H_sapiens | Animals | mono_intronic | H/ACA | ENSG00000153317 | ASAP1 | protein_coding |
| snoDB1339 | snoDB1339 | H_sapiens | Animals | mono_intronic | H/ACA | ENSG00000111962 | UST | protein_coding |
| snoDB1340 | snoDB1340 | H_sapiens | Animals | mono_intronic | H/ACA | ENSG00000168038 | ULK4 | protein_coding |
| snoDB1341 | snoDB1341 | H_sapiens | Animals | mono_intronic | H/ACA | ENSG00000117682 | DHDDS | protein_coding |
| snoDB1342 | snoDB1342 | H_sapiens | Animals | mono_intronic | H/ACA | ENSG00000029639 | TFB1M | protein_coding |
| snoDB1343 | snoDB1343 | H_sapiens | Animals | mono_intronic | H/ACA | ENSG00000119471 | HSDL2 | protein_coding |
| snoDB1344 | snoDB1344 | H_sapiens | Animals | mono_intronic | H/ACA | ENSG00000272886 | DCP1A | protein_coding |
| snoDB1345 | snoDB1345 | H_sapiens | Animals | mono_intronic | H/ACA | ENSG00000151208 | DLG5 | protein_coding |
| snoDB1346 | snoDB1346 | H_sapiens | Animals | mono_intronic | H/ACA | ENSG00000101333 | PLCB4 | protein_coding |
| snoDB1347 | snoDB1347 | H_sapiens | Animals | mono_intronic | H/ACA | ENSG00000129636 | ITFG1 | protein_coding |
| snoDB1348 | snoDB1348 | H_sapiens | Animals | mono_intronic | H/ACA | ENSG00000060237 | WNK1 | protein_coding |
| snoDB1349 | snoDB1349 | H_sapiens | Animals | mono_intronic | H/ACA | ENSG00000198707 | CEP290 | protein_coding |
| snoDB1350 | snoDB1350 | H_sapiens | Animals | mono_intronic | H/ACA | ENSG00000120306 | CYSTM1 | protein_coding |
| snoDB1351 | snoDB1351 | H_sapiens | Animals | mono_intronic | H/ACA | ENSG00000099991 | CABIN1 | protein_coding |
| snoDB1352 | snoDB1352 | H_sapiens | Animals | mono_intronic | H/ACA | ENSG00000086102 | NFX1 | protein_coding |
| snoDB1353 | snoDB1353 | H_sapiens | Animals | mono_intronic | H/ACA | ENSG00000278311 | GGNBP2 | protein_coding |
| snoDB1354 | snoDB1354 | H_sapiens | Animals | mono_intronic | H/ACA | ENSG00000075539 | FRYL | protein_coding |
| snoDB1355 | snoDB1355 | H_sapiens | Animals | mono_intronic | H/ACA | ENSG00000163939 | PBRM1 | protein_coding |
| snoDB1356 | snoDB1356 | H_sapiens | Animals | mono_intronic | H/ACA | ENSG00000005339 | CREBBP | protein_coding |

| | | | | | | | | |
|---|---|---|---|---|---|---|---|---|
| snoDB1357 | snoDB1357 | H_sapiens | Animals | mono_intronic | H/ACA | ENSG00000184349 | EFNA5 | protein_coding |
| snoDB1358 | snoDB1358 | H_sapiens | Animals | mono_intronic | H/ACA | ENSG00000147364 | FBXO25 | protein_coding |
| snoDB1359 | snoDB1359 | H_sapiens | Animals | mono_intronic | H/ACA | ENSG00000114573 | ATP6V1A | protein_coding |
| snoDB1360 | snoDB1360 | H_sapiens | Animals | mono_intronic | H/ACA | ENSG00000114423 | CBLB | protein_coding |
| snoDB1361 | snoDB1361 | H_sapiens | Animals | mono_intronic | H/ACA | ENSG00000156374 | PCGF6 | protein_coding |
| snoDB1362 | snoDB1362 | H_sapiens | Animals | mono_intronic | H/ACA | ENSG00000011114 | BTBD7 | protein_coding |
| snoDB1363 | snoDB1363 | H_sapiens | Animals | mono_intronic | H/ACA | ENSG00000197779 | ZNF81 | protein_coding |
| snoDB1364 | snoDB1364 | H_sapiens | Animals | mono_intronic | H/ACA | ENSG00000024048 | UBR2 | protein_coding |
| snoDB1365 | snoDB1365 | H_sapiens | Animals | mono_intronic | H/ACA | ENSG00000075223 | SEMA3C | protein_coding |
| snoDB1366 | snoDB1366 | H_sapiens | Animals | mono_intronic | H/ACA | ENSG00000124198 | ARFGEF2 | protein_coding |
| snoDB1367 | snoDB1367 | H_sapiens | Animals | mono_intronic | H/ACA | ENSG00000129515 | SNX6 | protein_coding |
| snoDB1368 | snoDB1368 | H_sapiens | Animals | mono_intronic | H/ACA | ENSG00000169567 | HINT1 | protein_coding |
| snoDB1369 | snoDB1369 | H_sapiens | Animals | mono_intronic | H/ACA | ENSG00000015475 | BID | protein_coding |
| snoDB1370 | snoDB1370 | H_sapiens | Animals | mono_intronic | H/ACA | ENSG00000134874 | DZIP1 | protein_coding |
| snoDB1371 | snoDB1371 | H_sapiens | Animals | mono_intronic | H/ACA | ENSG00000135090 | TAOK3 | protein_coding |
| snoDB1372 | snoDB1372 | H_sapiens | Animals | mono_intronic | H/ACA | ENSG00000070540 | WIPI1 | protein_coding |
| snoDB1373 | snoDB1373 | H_sapiens | Animals | mono_intronic | H/ACA | ENSG00000005100 | DHX33 | protein_coding |
| snoDB1374 | snoDB1374 | H_sapiens | Animals | mono_intronic | H/ACA | ENSG00000104695 | PPP2CB | protein_coding |
| snoDB1375 | snoDB1375 | H_sapiens | Animals | mono_intronic | H/ACA | ENSG00000196172 | ZNF681 | protein_coding |
| snoDB1376 | snoDB1376 | H_sapiens | Animals | mono_intronic | H/ACA | ENSG00000128731 | HERC2 | protein_coding |
| snoDB1377 | snoDB1377 | H_sapiens | Animals | mono_intronic | H/ACA | ENSG00000162961 | DPY30 | protein_coding |
| snoDB1378 | snoDB1378 | H_sapiens | Animals | mono_intronic | H/ACA | ENSG00000155903 | RASA2 | protein_coding |
| snoDB1379 | snoDB1379 | H_sapiens | Animals | mono_intronic | H/ACA | ENSG00000125676 | THOC2 | protein_coding |
| snoDB1380 | snoDB1380 | H_sapiens | Animals | mono_intronic | H/ACA | ENSG00000132676 | DAP3 | protein_coding |
| snoDB1381 | snoDB1381 | H_sapiens | Animals | mono_intergenic | H/ACA | | | intergenic |
| snoDB1382 | snoDB1382 | H_sapiens | Animals | mono_intronic | H/ACA | ENSG00000120254 | MTHFD1L | protein_coding |
| snoDB1383 | snoDB1383 | H_sapiens | Animals | mono_intronic | H/ACA | ENSG00000083642 | PDS5B | protein_coding |
| snoDB1384 | snoDB1384 | H_sapiens | Animals | mono_intronic | H/ACA | ENSG00000180098 | TRNAU1AP | protein_coding |
| snoDB1385 | snoDB1385 | H_sapiens | Animals | mono_intronic | H/ACA | ENSG00000130779 | CLIP1 | protein_coding |
| snoDB1386 | snoDB1386 | H_sapiens | Animals | mono_intronic | H/ACA | ENSG00000116044 | NFE2L2 | protein_coding |
| snoDB1387 | snoDB1387 | H_sapiens | Animals | mono_intronic | H/ACA | ENSG00000116151 | MORN1 | protein_coding |
| snoDB1388 | snoDB1388 | H_sapiens | Animals | mono_intronic | H/ACA | ENSG00000183624 | HMCES | protein_coding |
| snoDB1389 | snoDB1389 | H_sapiens | Animals | mono_intronic | H/ACA | ENSG00000115504 | EHBP1 | protein_coding |
| snoDB1390 | snoDB1390 | H_sapiens | Animals | mono_intronic | H/ACA | ENSG00000067955 | CBFB | protein_coding |
| snoDB1391 | snoDB1391 | H_sapiens | Animals | mono_intronic | H/ACA | ENSG00000143252 | SDHC | protein_coding |
| snoDB1392 | snoDB1392 | H_sapiens | Animals | mono_intronic | H/ACA | ENSG00000163468 | CCT3 | protein_coding |
| snoDB1393 | snoDB1393 | H_sapiens | Animals | mono_intronic | H/ACA | ENSG00000131018 | SYNE1 | protein_coding |
| snoDB1394 | snoDB1394 | H_sapiens | Animals | mono_intronic | H/ACA | ENSG00000102931 | ARL2BP | protein_coding |
| snoDB1395 | snoDB1395 | H_sapiens | Animals | mono_intronic | H/ACA | ENSG00000122299 | ZC3H7A | protein_coding |
| snoDB1396 | snoDB1396 | H_sapiens | Animals | mono_intronic | H/ACA | ENSG00000170802 | FOXN2 | protein_coding |
| snoDB1397 | snoDB1397 | H_sapiens | Animals | mono_intronic | H/ACA | ENSG00000117385 | P3H1 | protein_coding |
| snoDB1398 | snoDB1398 | H_sapiens | Animals | mono_intronic | H/ACA | ENSG00000078237 | TIGAR | protein_coding |
| snoDB1399 | snoDB1399 | H_sapiens | Animals | mono_intronic | H/ACA | ENSG00000155329 | ZCCHC10 | protein_coding |
| snoDB1400 | snoDB1400 | H_sapiens | Animals | intronic_cluster | H/ACA | ENSG00000182197 | EXT1 | protein_coding |
| snoDB1401 | snoDB1401 | H_sapiens | Animals | mono_intronic | H/ACA | ENSG00000104714 | ERICH1 | protein_coding |

| | | | | | | | | |
|---|---|---|---|---|---|---|---|---|
| snoDB1402 | snoDB1402 | H_sapiens | Animals | mono_intronic | H/ACA | ENSG00000151116 | UEVLD | protein_coding |
| snoDB1403 | snoDB1403 | H_sapiens | Animals | mono_intronic | H/ACA | ENSG00000133019 | CHRM3 | protein_coding |
| snoDB1404 | snoDB1404 | H_sapiens | Animals | mono_intronic | H/ACA | ENSG00000092201 | SUPT16H | protein_coding |
| snoDB1405 | snoDB1405 | H_sapiens | Animals | mono_intronic | H/ACA | ENSG00000120160 | EQTN | protein_coding |
| snoDB1406 | snoDB1406 | H_sapiens | Animals | mono_intronic | H/ACA | ENSG00000108799 | EZH1 | protein_coding |
| snoDB1407 | snoDB1407 | H_sapiens | Animals | mono_intronic | H/ACA | ENSG00000108256 | NUFIP2 | protein_coding |
| snoDB1408 | snoDB1408 | H_sapiens | Animals | mono_intronic | H/ACA | ENSG00000166747 | AP1G1 | protein_coding |
| snoDB1409 | snoDB1409 | H_sapiens | Animals | mono_intronic | H/ACA | ENSG00000108510 | MED13 | protein_coding |
| snoDB1410 | snoDB1410 | H_sapiens | Animals | mono_intronic | H/ACA | ENSG00000196712 | NF1 | protein_coding |
| snoDB1411 | snoDB1411 | H_sapiens | Animals | mono_intronic | H/ACA | ENSG00000204463 | BAG6 | protein_coding |
| snoDB1412 | snoDB1412 | H_sapiens | Animals | intronic_cluster | H/ACA | ENSG00000182463 | TSHZ2 | protein_coding |
| snoDB1413 | snoDB1413 | H_sapiens | Animals | intronic_cluster | H/ACA | ENSG00000284431 | | protein_coding |
| snoDB1414 | snoDB1414 | H_sapiens | Animals | mono_intronic | H/ACA | ENSG00000120910 | PPP3CC | protein_coding |
| snoDB1415 | snoDB1415 | H_sapiens | Animals | mono_intronic | H/ACA | ENSG00000160007 | ARHGAP35 | protein_coding |
| snoDB1416 | snoDB1416 | H_sapiens | Animals | mono_intronic | H/ACA | ENSG00000066279 | ASPM | protein_coding |
| snoDB1417 | snoDB1417 | H_sapiens | Animals | mono_intronic | H/ACA | ENSG00000197008 | ZNF138 | protein_coding |
| snoDB1418 | snoDB1418 | H_sapiens | Animals | mono_intronic | H/ACA | ENSG00000132383 | RPA1 | protein_coding |
| snoDB1419 | snoDB1419 | H_sapiens | Animals | mono_intronic | H/ACA | ENSG00000112237 | CCNC | protein_coding |
| snoDB1420 | snoDB1420 | H_sapiens | Animals | mono_intronic | H/ACA | ENSG00000138600 | SPPL2A | protein_coding |
| snoDB1421 | snoDB1421 | H_sapiens | Animals | mono_intronic | H/ACA | ENSG00000175764 | TTLL11 | protein_coding |
| snoDB1422 | snoDB1422 | H_sapiens | Animals | mono_intronic | H/ACA | ENSG00000083312 | TNPO1 | protein_coding |
| snoDB1423 | snoDB1423 | H_sapiens | Animals | mono_intronic | H/ACA | ENSG00000134317 | GRHL1 | protein_coding |
| snoDB1424 | snoDB1424 | H_sapiens | Animals | mono_intronic | H/ACA | ENSG00000116539 | ASH1L | protein_coding |
| snoDB1425 | snoDB1425 | H_sapiens | Animals | mono_intronic | H/ACA | ENSG00000171621 | SPSB1 | protein_coding |
| snoDB1426 | snoDB1426 | H_sapiens | Animals | mono_intronic | H/ACA | ENSG00000102898 | NUTF2 | protein_coding |
| snoDB1427 | snoDB1427 | H_sapiens | Animals | mono_intronic | H/ACA | ENSG00000198952 | SMG5 | protein_coding |
| snoDB1428 | snoDB1428 | H_sapiens | Animals | mono_intronic | H/ACA | ENSG00000153113 | CAST | protein_coding |
| snoDB1429 | snoDB1429 | H_sapiens | Animals | mono_intronic | H/ACA | ENSG00000125772 | GPCPD1 | protein_coding |
| snoDB1430 | snoDB1430 | H_sapiens | Animals | mono_intronic | H/ACA | ENSG00000174917 | MICOS13 | protein_coding |
| snoDB1431 | snoDB1431 | H_sapiens | Animals | mono_intronic | H/ACA | ENSG00000134769 | DTNA | protein_coding |
| snoDB1432 | snoDB1432 | H_sapiens | Animals | mono_intronic | H/ACA | ENSG00000100505 | TRIM9 | protein_coding |
| snoDB1433 | snoDB1433 | H_sapiens | Animals | mono_intronic | H/ACA | ENSG00000121774 | KHDRBS1 | protein_coding |
| snoDB1434 | snoDB1434 | H_sapiens | Animals | mono_intronic | H/ACA | ENSG00000284770 | TBCE | protein_coding |
| snoDB1435 | snoDB1435 | H_sapiens | Animals | mono_intronic | H/ACA | ENSG00000113163 | CERT1 | protein_coding |
| snoDB1436 | snoDB1436 | H_sapiens | Animals | mono_intronic | H/ACA | ENSG00000119231 | SENP5 | protein_coding |
| snoDB1437 | snoDB1437 | H_sapiens | Animals | mono_intronic | H/ACA | ENSG00000161813 | LARP4 | protein_coding |
| snoDB1438 | snoDB1438 | H_sapiens | Animals | mono_intronic | H/ACA | ENSG00000082068 | WDR70 | protein_coding |
| snoDB1439 | snoDB1439 | H_sapiens | Animals | mono_intronic | H/ACA | ENSG00000123136 | DDX39A | protein_coding |
| snoDB1440 | snoDB1440 | H_sapiens | Animals | mono_intronic | H/ACA | ENSG00000102910 | LONP2 | protein_coding |
| snoDB1441 | snoDB1441 | H_sapiens | Animals | intronic_cluster | H/ACA | ENSG00000187231 | SESTD1 | protein_coding |
| snoDB1442 | snoDB1442 | H_sapiens | Animals | mono_intronic | H/ACA | ENSG00000179151 | EDC3 | protein_coding |
| snoDB1443 | snoDB1443 | H_sapiens | Animals | mono_intronic | H/ACA | ENSG00000120798 | NR2C1 | protein_coding |
| snoDB1444 | snoDB1444 | H_sapiens | Animals | mono_intronic | H/ACA | ENSG00000175581 | MRPL48 | protein_coding |
| snoDB1445 | snoDB1445 | H_sapiens | Animals | mono_intronic | H/ACA | ENSG00000157654 | PALM2AKAP2 | protein_coding |
| snoDB1446 | snoDB1446 | H_sapiens | Animals | mono_intronic | H/ACA | ENSG00000135525 | MAP7 | protein_coding |

| | | | | | | | | |
|---|---|---|---|---|---|---|---|---|
| snoDB1447 | snoDB1447 | H_sapiens | Animals | mono_intronic | H/ACA | ENSG00000197912 | SPG7 | protein_coding |
| snoDB1448 | snoDB1448 | H_sapiens | Animals | mono_intronic | H/ACA | ENSG00000007541 | PIGQ | protein_coding |
| snoDB1449 | snoDB1449 | H_sapiens | Animals | mono_intronic | H/ACA | ENSG00000070018 | LRP6 | protein_coding |
| snoDB1450 | snoDB1450 | H_sapiens | Animals | mono_intronic | H/ACA | ENSG00000111231 | GPN3 | protein_coding |
| snoDB1451 | snoDB1451 | H_sapiens | Animals | mono_intronic | H/ACA | ENSG00000065485 | PDIA5 | protein_coding |
| snoDB1452 | snoDB1452 | H_sapiens | Animals | mono_intronic | H/ACA | ENSG00000081923 | ATP8B1 | protein_coding |
| snoDB1453 | snoDB1453 | H_sapiens | Animals | mono_intronic | H/ACA | ENSG00000141568 | FOXK2 | protein_coding |
| snoDB1454 | snoDB1454 | H_sapiens | Animals | mono_intronic | H/ACA | ENSG00000092931 | MFSD11 | protein_coding |
| snoDB1455 | snoDB1455 | H_sapiens | Animals | mono_intronic | H/ACA | ENSG00000005812 | FBXL3 | protein_coding |
| snoDB1456 | snoDB1456 | H_sapiens | Animals | mono_intronic | H/ACA | ENSG00000172731 | LRRC20 | protein_coding |
| snoDB1457 | snoDB1457 | H_sapiens | Animals | mono_intronic | H/ACA | ENSG00000099250 | NRP1 | protein_coding |
| snoDB1458 | snoDB1458 | H_sapiens | Animals | mono_intronic | H/ACA | ENSG00000106459 | NRF1 | protein_coding |
| snoDB1459 | snoDB1459 | H_sapiens | Animals | mono_intergenic | H/ACA | | | intergenic |
| snoDB1460 | snoDB1460 | H_sapiens | Animals | mono_intronic | H/ACA | ENSG00000185049 | NELFA | protein_coding |
| snoDB1461 | snoDB1461 | H_sapiens | Animals | mono_intronic | H/ACA | ENSG00000131043 | AAR2 | protein_coding |
| snoDB1462 | snoDB1462 | H_sapiens | Animals | mono_intronic | H/ACA | ENSG00000092148 | HECTD1 | protein_coding |
| snoDB1463 | snoDB1463 | H_sapiens | Animals | mono_intronic | H/ACA | ENSG00000166532 | RIMKLB | protein_coding |
| snoDB1464 | snoDB1464 | H_sapiens | Animals | mono_intronic | H/ACA | ENSG00000150054 | MPP7 | protein_coding |
| snoDB1465 | snoDB1465 | H_sapiens | Animals | mono_intronic | H/ACA | ENSG00000136240 | KDELR2 | protein_coding |
| snoDB1466 | snoDB1466 | H_sapiens | Animals | mono_intronic | H/ACA | ENSG00000174652 | ZNF266 | protein_coding |
| snoDB1467 | snoDB1467 | H_sapiens | Animals | mono_intronic | H/ACA | ENSG00000033627 | ATP6V0A1 | protein_coding |
| snoDB1468 | snoDB1468 | H_sapiens | Animals | mono_intronic | H/ACA | ENSG00000072736 | NFATC3 | protein_coding |
| snoDB1469 | snoDB1469 | H_sapiens | Animals | mono_intronic | H/ACA | ENSG00000185480 | PARPBP | protein_coding |
| snoDB1470 | snoDB1470 | H_sapiens | Animals | mono_intronic | H/ACA | ENSG00000131828 | PDHA1 | protein_coding |
| snoDB1471 | snoDB1471 | H_sapiens | Animals | mono_intronic | H/ACA | ENSG00000100354 | TNRC6B | protein_coding |
| snoDB1472 | snoDB1472 | H_sapiens | Animals | mono_intronic | H/ACA | ENSG00000105372 | RPS19 | protein_coding |
| snoDB1473 | snoDB1473 | H_sapiens | Animals | mono_intronic | H/ACA | ENSG00000138777 | PPA2 | protein_coding |
| snoDB1474 | snoDB1474 | H_sapiens | Animals | mono_intronic | H/ACA | ENSG00000278259 | MYO19 | protein_coding |
| snoDB1475 | snoDB1475 | H_sapiens | Animals | mono_intronic | H/ACA | ENSG00000160551 | TAOK1 | protein_coding |
| snoDB1476 | snoDB1476 | H_sapiens | Animals | mono_intronic | H/ACA | ENSG00000135108 | FBXO21 | protein_coding |
| snoDB1477 | snoDB1477 | H_sapiens | Animals | mono_intronic | H/ACA | ENSG00000137574 | TGS1 | protein_coding |
| snoDB1478 | snoDB1478 | H_sapiens | Animals | mono_intronic | H/ACA | ENSG00000196715 | VKORC1L1 | protein_coding |
| snoDB1479 | snoDB1479 | H_sapiens | Animals | mono_intronic | H/ACA | ENSG00000106100 | NOD1 | protein_coding |
| snoDB1480 | snoDB1480 | H_sapiens | Animals | mono_intronic | H/ACA | ENSG00000055609 | KMT2C | protein_coding |
| snoDB1481 | snoDB1481 | H_sapiens | Animals | mono_intronic | H/ACA | ENSG00000049618 | ARID1B | protein_coding |
| snoDB1482 | snoDB1482 | H_sapiens | Animals | mono_intronic | H/ACA | ENSG00000144724 | PTPRG | protein_coding |
| snoDB1483 | snoDB1483 | H_sapiens | Animals | mono_intronic | H/ACA | ENSG00000239305 | RNF103 | protein_coding |
| snoDB1484 | snoDB1484 | H_sapiens | Animals | mono_intronic | H/ACA | ENSG00000100344 | PNPLA3 | protein_coding |
| snoDB1485 | snoDB1485 | H_sapiens | Animals | mono_intronic | H/ACA | ENSG00000100209 | HSCB | protein_coding |
| snoDB1486 | snoDB1486 | H_sapiens | Animals | mono_intronic | H/ACA | ENSG00000061676 | NCKAP1 | protein_coding |
| snoDB1487 | snoDB1487 | H_sapiens | Animals | mono_intronic | H/ACA | ENSG00000166226 | CCT2 | protein_coding |
| snoDB1488 | snoDB1488 | H_sapiens | Animals | mono_intronic | H/ACA | ENSG00000082805 | ERC1 | protein_coding |
| snoDB1489 | snoDB1489 | H_sapiens | Animals | mono_intronic | H/ACA | ENSG00000121671 | CRY2 | protein_coding |
| snoDB1490 | snoDB1490 | H_sapiens | Animals | mono_intronic | H/ACA | ENSG00000173218 | VANGL1 | protein_coding |
| snoDB1491 | snoDB1491 | H_sapiens | Animals | mono_intronic | H/ACA | ENSG00000258539 | | protein_coding |

| | | | | | | | | |
|---|---|---|---|---|---|---|---|---|
| snoDB1492 | snoDB1492 | H_sapiens | Animals | mono_intronic | H/ACA | ENSG00000137073 | UBAP2 | protein_coding |
| snoDB1493 | snoDB1493 | H_sapiens | Animals | mono_intronic | H/ACA | ENSG00000171843 | MLLT3 | protein_coding |
| snoDB1494 | snoDB1494 | H_sapiens | Animals | mono_intronic | H/ACA | ENSG00000145685 | LHFPL2 | protein_coding |
| snoDB1495 | snoDB1495 | H_sapiens | Animals | mono_intronic | H/ACA | ENSG00000197603 | CPLANE1 | protein_coding |
| snoDB1496 | snoDB1496 | H_sapiens | Animals | mono_intronic | H/ACA | ENSG00000113761 | ZNF346 | protein_coding |
| snoDB1497 | snoDB1497 | H_sapiens | Animals | mono_intronic | H/ACA | ENSG00000143951 | WDPCP | protein_coding |
| snoDB1498 | snoDB1498 | H_sapiens | Animals | mono_intronic | H/ACA | ENSG00000115464 | USP34 | protein_coding |
| snoDB1499 | snoDB1499 | H_sapiens | Animals | mono_intergenic | H/ACA | | | intergenic |
| snoDB1500 | snoDB1500 | H_sapiens | Animals | mono_intronic | H/ACA | ENSG00000172985 | SH3RF3 | protein_coding |
| snoDB1501 | snoDB1501 | H_sapiens | Animals | mono_intronic | H/ACA | ENSG00000141664 | ZCCHC2 | protein_coding |
| snoDB1502 | snoDB1502 | H_sapiens | Animals | mono_intronic | H/ACA | ENSG00000140443 | IGF1R | protein_coding |
| snoDB1503 | snoDB1503 | H_sapiens | Animals | mono_intronic | H/ACA | ENSG00000078900 | TP73 | protein_coding |
| snoDB1504 | snoDB1504 | H_sapiens | Animals | mono_intronic | H/ACA | ENSG00000124486 | USP9X | protein_coding |
| snoDB1505 | snoDB1505 | H_sapiens | Animals | mono_intronic | H/ACA | ENSG00000062194 | GPBP1 | protein_coding |
| snoDB1506 | snoDB1506 | H_sapiens | Animals | mono_intronic | H/ACA | ENSG00000143889 | HNRNPLL | protein_coding |
| snoDB1507 | snoDB1507 | H_sapiens | Animals | intronic_cluster | H/ACA | ENSG00000182463 | TSHZ2 | protein_coding |
| snoDB1508 | snoDB1508 | H_sapiens | Animals | mono_intronic | H/ACA | ENSG00000118454 | ANKRD13C | protein_coding |
| snoDB1509 | snoDB1509 | H_sapiens | Animals | mono_intronic | H/ACA | ENSG00000169032 | MAP2K1 | protein_coding |
| snoDB1510 | snoDB1510 | H_sapiens | Animals | mono_intronic | H/ACA | ENSG00000054654 | SYNE2 | protein_coding |
| snoDB1511 | snoDB1511 | H_sapiens | Animals | mono_intronic | H/ACA | ENSG00000131023 | LATS1 | protein_coding |
| snoDB1512 | snoDB1512 | H_sapiens | Animals | mono_intronic | H/ACA | ENSG00000164190 | NIPBL | protein_coding |
| snoDB1513 | snoDB1513 | H_sapiens | Animals | mono_intronic | H/ACA | ENSG00000121210 | TMEM131L | protein_coding |
| snoDB1514 | snoDB1514 | H_sapiens | Animals | mono_intronic | H/ACA | ENSG00000109686 | SH3D19 | protein_coding |
| snoDB1515 | snoDB1515 | H_sapiens | Animals | intronic_cluster | H/ACA | ENSG00000284431 | | protein_coding |
| snoDB1516 | snoDB1516 | H_sapiens | Animals | mono_intronic | H/ACA | ENSG00000142507 | PSMB6 | protein_coding |
| snoDB1517 | snoDB1517 | H_sapiens | Animals | mono_intronic | H/ACA | ENSG00000122971 | ACADS | protein_coding |
| snoDB1518 | snoDB1518 | H_sapiens | Animals | mono_intronic | H/ACA | ENSG00000111249 | CUX2 | protein_coding |
| snoDB1519 | snoDB1519 | H_sapiens | Animals | mono_intergenic | H/ACA | | | intergenic |
| snoDB1520 | snoDB1520 | H_sapiens | Animals | mono_intronic | H/ACA | ENSG00000065833 | ME1 | protein_coding |
| snoDB1521 | snoDB1521 | H_sapiens | Animals | mono_intronic | H/ACA | ENSG00000124782 | RREB1 | protein_coding |
| snoDB1522 | snoDB1522 | H_sapiens | Animals | mono_intronic | H/ACA | ENSG00000185261 | KIAA0825 | protein_coding |
| snoDB1523 | snoDB1523 | H_sapiens | Animals | mono_intronic | H/ACA | ENSG00000155508 | CNOT8 | protein_coding |
| snoDB1524 | snoDB1524 | H_sapiens | Animals | mono_intronic | H/ACA | ENSG00000145833 | DDX46 | protein_coding |
| snoDB1525 | snoDB1525 | H_sapiens | Animals | mono_intronic | H/ACA | ENSG00000155749 | FLACC1 | protein_coding |
| snoDB1526 | snoDB1526 | H_sapiens | Animals | mono_intronic | H/ACA | ENSG00000198554 | WDHD1 | protein_coding |
| snoDB1527 | snoDB1527 | H_sapiens | Animals | mono_intronic | H/ACA | ENSG00000139832 | RAB20 | protein_coding |
| snoDB1528 | snoDB1528 | H_sapiens | Animals | mono_intronic | H/ACA | ENSG00000136044 | APPL2 | protein_coding |
| snoDB1529 | snoDB1529 | H_sapiens | Animals | mono_intronic | H/ACA | ENSG00000224901 | | non_coding |
| snoDB1530 | snoDB1530 | H_sapiens | Animals | mono_intergenic | H/ACA | | | intergenic |
| snoDB1531 | snoDB1531 | H_sapiens | Animals | mono_intronic | H/ACA | ENSG00000163125 | RPRD2 | protein_coding |
| snoDB1532 | snoDB1532 | H_sapiens | Animals | mono_intronic | H/ACA | ENSG00000135049 | AGTPBP1 | protein_coding |
| snoDB1533 | snoDB1533 | H_sapiens | Animals | mono_intronic | H/ACA | ENSG00000160445 | ZER1 | protein_coding |
| snoDB1534 | snoDB1534 | H_sapiens | Animals | mono_intronic | H/ACA | ENSG00000272325 | NUDT3 | protein_coding |
| snoDB1535 | snoDB1535 | H_sapiens | Animals | mono_intergenic | H/ACA | | | intergenic |
| snoDB1536 | snoDB1536 | H_sapiens | Animals | mono_intronic | H/ACA | ENSG00000164070 | HSPA4L | protein_coding |

| | | | | | | | | |
|---|---|---|---|---|---|---|---|---|
| snoDB1537 | snoDB1537 | H_sapiens | Animals | mono_intronic | H/ACA | ENSG00000154822 | PLCL2 | protein_coding |
| snoDB1538 | snoDB1538 | H_sapiens | Animals | mono_intronic | H/ACA | ENSG00000163001 | CFAP36 | protein_coding |
| snoDB1539 | snoDB1539 | H_sapiens | Animals | mono_intronic | H/ACA | ENSG00000047578 | KATNIP | protein_coding |
| snoDB1540 | snoDB1540 | H_sapiens | Animals | mono_intronic | H/ACA | ENSG00000129493 | HEATR5A | protein_coding |
| snoDB1541 | snoDB1541 | H_sapiens | Animals | mono_intronic | H/ACA | ENSG00000125249 | RAP2A | protein_coding |
| snoDB1542 | snoDB1542 | H_sapiens | Animals | mono_intergenic | H/ACA | | | intergenic |
| snoDB1543 | snoDB1543 | H_sapiens | Animals | intronic_cluster | H/ACA | ENSG00000083168 | KAT6A | protein_coding |
| snoDB1544 | snoDB1544 | H_sapiens | Animals | mono_intergenic | H/ACA | | | intergenic |
| snoDB1545 | snoDB1545 | H_sapiens | Animals | mono_intronic | H/ACA | ENSG00000113269 | RNF130 | protein_coding |
| snoDB1546 | snoDB1546 | H_sapiens | Animals | mono_intronic | H/ACA | ENSG00000113615 | SEC24A | protein_coding |
| snoDB1547 | snoDB1547 | H_sapiens | Animals | mono_intronic | H/ACA | ENSG00000188517 | COL25A1 | protein_coding |
| snoDB1548 | snoDB1548 | H_sapiens | Animals | mono_intronic | H/ACA | ENSG00000186854 | TRABD2A | protein_coding |
| snoDB1549 | snoDB1549 | H_sapiens | Animals | mono_intronic | H/ACA | ENSG00000128191 | DGCR8 | protein_coding |
| snoDB1550 | snoDB1550 | H_sapiens | Animals | mono_intronic | H/ACA | ENSG00000185359 | HGS | protein_coding |
| snoDB1551 | snoDB1551 | H_sapiens | Animals | mono_intronic | H/ACA | ENSG00000213658 | LAT | protein_coding |
| snoDB1552 | snoDB1552 | H_sapiens | Animals | mono_intronic | H/ACA | ENSG00000117461 | PIK3R3 | protein_coding |
| snoDB1553 | snoDB1553 | H_sapiens | Animals | mono_intronic | H/ACA | ENSG00000126067 | PSMB2 | protein_coding |
| snoDB1554 | snoDB1554 | H_sapiens | Animals | mono_intronic | H/ACA | ENSG00000025800 | KPNA6 | protein_coding |
| snoDB1555 | snoDB1555 | H_sapiens | Animals | mono_intronic | H/ACA | ENSG00000060982 | BCAT1 | protein_coding |
| snoDB1556 | snoDB1556 | H_sapiens | Animals | mono_intronic | H/ACA | ENSG00000117625 | RCOR3 | protein_coding |
| snoDB1557 | snoDB1557 | H_sapiens | Animals | mono_intronic | H/ACA | ENSG00000198837 | DENND4B | protein_coding |
| snoDB1558 | snoDB1558 | H_sapiens | Animals | mono_intronic | H/ACA | ENSG00000130939 | UBE4B | protein_coding |
| snoDB1559 | snoDB1559 | H_sapiens | Animals | mono_intronic | H/ACA | ENSG00000127947 | PTPN12 | protein_coding |
| snoDB1560 | snoDB1560 | H_sapiens | Animals | mono_intronic | H/ACA | ENSG00000135315 | CEP162 | protein_coding |
| snoDB1561 | snoDB1561 | H_sapiens | Animals | mono_intronic | H/ACA | ENSG00000124614 | RPS10 | protein_coding |
| snoDB1562 | snoDB1562 | H_sapiens | Animals | mono_exonic | H/ACA | ENSG00000206562 | METTL6 | protein_coding |
| snoDB1563 | snoDB1563 | H_sapiens | Animals | mono_intronic | H/ACA | ENSG00000175054 | ATR | protein_coding |
| snoDB1564 | snoDB1564 | H_sapiens | Animals | mono_intronic | H/ACA | ENSG00000241973 | PI4KA | protein_coding |
| snoDB1565 | snoDB1565 | H_sapiens | Animals | mono_intergenic | H/ACA | | | intergenic |
| snoDB1566 | snoDB1566 | H_sapiens | Animals | mono_intronic | H/ACA | ENSG00000108984 | MAP2K6 | protein_coding |
| snoDB1567 | snoDB1567 | H_sapiens | Animals | mono_intronic | H/ACA | ENSG00000162039 | MEIOB | protein_coding |
| snoDB1568 | snoDB1568 | H_sapiens | Animals | mono_intronic | H/ACA | ENSG00000131697 | NPHP4 | protein_coding |
| snoDB1569 | snoDB1569 | H_sapiens | Animals | mono_intronic | H/ACA | ENSG00000137814 | HAUS2 | protein_coding |
| snoDB1570 | snoDB1570 | H_sapiens | Animals | mono_intergenic | H/ACA | | | intergenic |
| snoDB1571 | snoDB1571 | H_sapiens | Animals | mono_intronic | H/ACA | ENSG00000154001 | PPP2R5E | protein_coding |
| snoDB1572 | snoDB1572 | H_sapiens | Animals | mono_intronic | H/ACA | ENSG00000227533 | SLC2A1-DT | non_coding |
| snoDB1573 | snoDB1573 | H_sapiens | Animals | mono_intronic | H/ACA | ENSG00000173120 | KDM2A | protein_coding |
| snoDB1574 | snoDB1574 | H_sapiens | Animals | mono_intronic | H/ACA | ENSG00000147050 | KDM6A | protein_coding |
| snoDB1575 | snoDB1575 | H_sapiens | Animals | mono_intronic | H/ACA | ENSG00000183337 | BCOR | protein_coding |
| snoDB1576 | snoDB1576 | H_sapiens | Animals | intronic_cluster | H/ACA | ENSG00000169239 | CA5B | protein_coding |
| snoDB1577 | snoDB1577 | H_sapiens | Animals | mono_intronic | H/ACA | ENSG00000158683 | PKD1L1 | protein_coding |
| snoDB1578 | snoDB1578 | H_sapiens | Animals | mono_intronic | H/ACA | ENSG00000146535 | GNA12 | protein_coding |
| snoDB1579 | snoDB1579 | H_sapiens | Animals | mono_intronic | H/ACA | ENSG00000081803 | CADPS2 | protein_coding |
| snoDB1580 | snoDB1580 | H_sapiens | Animals | mono_intronic | H/ACA | ENSG00000024048 | UBR2 | protein_coding |
| snoDB1581 | snoDB1581 | H_sapiens | Animals | mono_intergenic | H/ACA | | | intergenic |

| | | | | | | | | |
|---|---|---|---|---|---|---|---|---|
| snoDB1582 | snoDB1582 | H_sapiens | Animals | mono_intronic | H/ACA | ENSG00000144744 | UBA3 | protein_coding |
| snoDB1583 | snoDB1583 | H_sapiens | Animals | mono_intronic | H/ACA | ENSG00000163788 | SNRK | protein_coding |
| snoDB1584 | snoDB1584 | H_sapiens | Animals | mono_intronic | H/ACA | ENSG00000138100 | TRIM54 | protein_coding |
| snoDB1585 | snoDB1585 | H_sapiens | Animals | mono_intronic | H/ACA | ENSG00000130702 | LAMA5 | protein_coding |
| snoDB1586 | snoDB1586 | H_sapiens | Animals | mono_intronic | H/ACA | ENSG00000053747 | LAMA3 | protein_coding |
| snoDB1587 | snoDB1587 | H_sapiens | Animals | mono_intronic | H/ACA | ENSG00000083093 | PALB2 | protein_coding |
| snoDB1588 | snoDB1588 | H_sapiens | Animals | mono_intronic | H/ACA | ENSG00000058600 | POLR3E | protein_coding |
| snoDB1589 | snoDB1589 | H_sapiens | Animals | intergenic_cluster | H/ACA | | | intergenic |
| snoDB1590 | snoDB1590 | H_sapiens | Animals | mono_intronic | H/ACA | ENSG00000116138 | DNAJC16 | protein_coding |
| snoDB1591 | snoDB1591 | H_sapiens | Animals | mono_intronic | H/ACA | ENSG00000198162 | MAN1A2 | protein_coding |
| snoDB1592 | snoDB1592 | H_sapiens | Animals | mono_intergenic | H/ACA | | | intergenic |
| snoDB1593 | snoDB1593 | H_sapiens | Animals | mono_intronic | H/ACA | ENSG00000148019 | CEP78 | protein_coding |
| snoDB1594 | snoDB1594 | H_sapiens | Animals | mono_intronic | H/ACA | ENSG00000188372 | ZP3 | protein_coding |
| snoDB1595 | snoDB1595 | H_sapiens | Animals | mono_intergenic | H/ACA | | | intergenic |
| snoDB1596 | snoDB1596 | H_sapiens | Animals | mono_intronic | H/ACA | ENSG00000111886 | GABRR2 | protein_coding |
| snoDB1597 | snoDB1597 | H_sapiens | Animals | mono_intergenic | H/ACA | | | intergenic |
| snoDB1598 | snoDB1598 | H_sapiens | Animals | mono_intronic | H/ACA | ENSG00000132842 | AP3B1 | protein_coding |
| snoDB1599 | snoDB1599 | H_sapiens | Animals | mono_intergenic | H/ACA | | | intergenic |
| snoDB1600 | snoDB1600 | H_sapiens | Animals | mono_intronic | H/ACA | ENSG00000129595 | EPB41L4A | protein_coding |
| snoDB1601 | snoDB1601 | H_sapiens | Animals | mono_intronic | H/ACA | ENSG00000164164 | OTUD4 | protein_coding |
| snoDB1602 | snoDB1602 | H_sapiens | Animals | mono_intronic | H/ACA | ENSG00000213123 | DYNLT2B | protein_coding |
| snoDB1603 | snoDB1603 | H_sapiens | Animals | mono_intronic | H/ACA | ENSG00000175697 | GPR156 | protein_coding |
| snoDB1604 | snoDB1604 | H_sapiens | Animals | mono_intergenic | H/ACA | | | intergenic |
| snoDB1605 | snoDB1605 | H_sapiens | Animals | mono_intronic | H/ACA | ENSG00000144034 | TPRKB | protein_coding |
| snoDB1606 | snoDB1606 | H_sapiens | Animals | mono_intronic | H/ACA | ENSG00000231918 | | non_coding |
| snoDB1607 | snoDB1607 | H_sapiens | Animals | mono_intronic | H/ACA | ENSG00000100403 | ZC3H7B | protein_coding |
| snoDB1608 | snoDB1608 | H_sapiens | Animals | mono_intronic | H/ACA | ENSG00000100154 | TTC28 | protein_coding |
| snoDB1609 | snoDB1609 | H_sapiens | Animals | mono_intronic | H/ACA | ENSG00000184470 | TXNRD2 | protein_coding |
| snoDB1610 | snoDB1610 | H_sapiens | Animals | mono_intronic | H/ACA | ENSG00000018510 | AGPS | protein_coding |
| snoDB1611 | snoDB1611 | H_sapiens | Animals | mono_intronic | H/ACA | ENSG00000142197 | DOP1B | protein_coding |
| snoDB1612 | snoDB1612 | H_sapiens | Animals | mono_intronic | H/ACA | ENSG00000166979 | EVA1C | protein_coding |
| snoDB1613 | snoDB1613 | H_sapiens | Animals | mono_intronic | H/ACA | ENSG00000196961 | AP2A1 | protein_coding |
| snoDB1614 | snoDB1614 | H_sapiens | Animals | mono_intergenic | H/ACA | | | intergenic |
| snoDB1615 | snoDB1615 | H_sapiens | Animals | mono_intronic | H/ACA | ENSG00000198690 | FAN1 | protein_coding |
| snoDB1616 | snoDB1616 | H_sapiens | Animals | mono_intronic | H/ACA | ENSG00000020426 | MNAT1 | protein_coding |
| snoDB1617 | snoDB1617 | H_sapiens | Animals | mono_intronic | H/ACA | ENSG00000139344 | AMDHD1 | protein_coding |
| snoDB1618 | snoDB1618 | H_sapiens | Animals | mono_intronic | H/ACA | ENSG00000170456 | DENND5B | protein_coding |
| snoDB1619 | snoDB1619 | H_sapiens | Animals | mono_intronic | H/ACA | ENSG00000159063 | ALG8 | protein_coding |
| snoDB1620 | snoDB1620 | H_sapiens | Animals | mono_intronic | H/ACA | ENSG00000143183 | TMCO1 | protein_coding |
| snoDB1621 | snoDB1621 | H_sapiens | Animals | mono_intergenic | H/ACA | | | intergenic |
| snoDB1622 | snoU13 | H_sapiens | Animals | mono_intergenic | C/D | | | intergenic |
| snoDB1623 | snoU13 | H_sapiens | Animals | mono_intronic | C/D | ENSG00000100865 | CINP | protein_coding |
| snoDB1624 | snoU13 | H_sapiens | Animals | mono_intergenic | C/D | | | intergenic |
| snoDB1625 | snoU13 | H_sapiens | Animals | mono_intergenic | C/D | | | intergenic |
| snoDB1626 | snoU13 | H_sapiens | Animals | mono_intronic | C/D | ENSG00000196975 | ANXA4 | protein_coding |

| | | | | | | | | |
|---|---|---|---|---|---|---|---|---|
| snoDB1627 | snoU13 | H_sapiens | Animals | mono_intergenic | C/D | | | intergenic |
| snoDB1628 | snoU13 | H_sapiens | Animals | mono_intergenic | C/D | | | intergenic |
| snoDB1629 | snoU13 | H_sapiens | Animals | mono_intronic | C/D | ENSG00000175115 | PACS1 | protein_coding |
| snoDB1630 | snoU13 | H_sapiens | Animals | mono_intergenic | C/D | | | intergenic |
| snoDB1631 | snoU13 | H_sapiens | Animals | mono_intergenic | C/D | | | intergenic |
| snoDB1632 | snoU13 | H_sapiens | Animals | mono_intergenic | C/D | | | intergenic |
| snoDB1633 | snoU13 | H_sapiens | Animals | mono_intronic | C/D | ENSG00000157470 | FAM81A | protein_coding |
| snoDB1634 | snoU13 | H_sapiens | Animals | mono_intergenic | C/D | | | intergenic |
| snoDB1635 | snoU13 | H_sapiens | Animals | mono_intronic | C/D | ENSG00000116678 | LEPR | protein_coding |
| snoDB1636 | snoU13 | H_sapiens | Animals | mono_intergenic | C/D | | | intergenic |
| snoDB1637 | snoU13 | H_sapiens | Animals | mono_intronic | C/D | ENSG00000066739 | ATG2B | protein_coding |
| snoDB1638 | snoU13 | H_sapiens | Animals | mono_intronic | C/D | ENSG00000163697 | APBB2 | protein_coding |
| snoDB1639 | snoU13 | H_sapiens | Animals | mono_intergenic | C/D | | | intergenic |
| snoDB1640 | snoU13 | H_sapiens | Animals | mono_intergenic | C/D | | | intergenic |
| snoDB1641 | snoU13 | H_sapiens | Animals | mono_intergenic | C/D | | | intergenic |
| snoDB1642 | snoU13 | H_sapiens | Animals | mono_intergenic | C/D | | | intergenic |
| snoDB1643 | snoU13 | H_sapiens | Animals | mono_intergenic | C/D | | | intergenic |
| snoDB1644 | snoU13 | H_sapiens | Animals | mono_intergenic | C/D | | | intergenic |
| snoDB1645 | snoU13 | H_sapiens | Animals | mono_intronic | C/D | ENSG00000069869 | NEDD4 | protein_coding |
| snoDB1646 | snoU13 | H_sapiens | Animals | mono_intergenic | C/D | | | intergenic |
| snoDB1647 | snoU13 | H_sapiens | Animals | mono_intergenic | C/D | | | intergenic |
| snoDB1648 | snoU13 | H_sapiens | Animals | mono_intronic | C/D | ENSG00000151151 | IPMK | protein_coding |
| snoDB1649 | snoU13 | H_sapiens | Animals | mono_intronic | C/D | ENSG00000204611 | ZNF616 | protein_coding |
| snoDB1650 | SNORD43L1 | H_sapiens | Animals | mono_intronic | C/D | ENSG00000054938 | CHRDL2 | protein_coding |
| snoDB1651 | snoU13 | H_sapiens | Animals | mono_intergenic | C/D | | | intergenic |
| snoDB1652 | SNORA70 | H_sapiens | Animals | mono_intronic | H/ACA | ENSG00000225189 | REREP1Y | non_coding |
| snoDB1653 | SNORA70 | H_sapiens | Animals | mono_intergenic | H/ACA | | | intergenic |
| snoDB1654 | snoU13 | H_sapiens | Animals | mono_intergenic | C/D | | | intergenic |
| snoDB1655 | snoU13 | H_sapiens | Animals | mono_intergenic | C/D | | | intergenic |
| snoDB1656 | snoU13 | H_sapiens | Animals | mono_intronic | C/D | ENSG00000116489 | CAPZA1 | protein_coding |
| snoDB1657 | snoU13 | H_sapiens | Animals | mono_intergenic | C/D | | | intergenic |
| snoDB1658 | U3 | H_sapiens | Animals | mono_intergenic | C/D | | | intergenic |
| snoDB1659 | snoU13 | H_sapiens | Animals | mono_intronic | C/D | ENSG00000179520 | SLC17A8 | protein_coding |
| snoDB1660 | snoU13 | H_sapiens | Animals | mono_intronic | C/D | ENSG00000106009 | BRAT1 | protein_coding |
| snoDB1661 | snoU13 | H_sapiens | Animals | mono_intronic | C/D | ENSG00000228857 | ACTR3-AS1 | non_coding |
| snoDB1662 | snoU13 | H_sapiens | Animals | mono_intergenic | C/D | | | intergenic |
| snoDB1663 | snoU13 | H_sapiens | Animals | mono_intronic | C/D | ENSG00000183722 | LHFPL6 | protein_coding |
| snoDB1664 | snoU13 | H_sapiens | Animals | mono_exonic | C/D | ENSG00000063587 | ZNF275 | protein_coding |
| snoDB1665 | snoU13 | H_sapiens | Animals | mono_intergenic | C/D | | | intergenic |
| snoDB1666 | snoU13 | H_sapiens | Animals | mono_intergenic | C/D | | | intergenic |
| snoDB1667 | U3 | H_sapiens | Animals | mono_intergenic | C/D | | | intergenic |
| snoDB1668 | snoU13 | H_sapiens | Animals | mono_intronic | C/D | ENSG00000236107 | SCN1A-AS1 | non_coding |
| snoDB1669 | snoU13 | H_sapiens | Animals | mono_intronic | C/D | ENSG00000155849 | ELMO1 | protein_coding |
| snoDB1670 | snoU13 | H_sapiens | Animals | mono_intergenic | C/D | | | intergenic |
| snoDB1671 | snoU13 | H_sapiens | Animals | mono_intergenic | C/D | | | intergenic |

| | | | | | | | | |
|---|---|---|---|---|---|---|---|---|
| snoDB1672 | snoU13 | H_sapiens | Animals | mono_intergenic | C/D | | | intergenic |
| snoDB1673 | snoU13 | H_sapiens | Animals | mono_intronic | C/D | ENSG00000134748 | PRPF38A | protein_coding |
| snoDB1674 | snoU13 | H_sapiens | Animals | mono_intronic | C/D | ENSG00000163453 | IGFBP7 | protein_coding |
| snoDB1675 | snoU13 | H_sapiens | Animals | mono_intergenic | C/D | | | intergenic |
| snoDB1676 | snoU13 | H_sapiens | Animals | mono_intronic | C/D | ENSG00000134871 | COL4A2 | protein_coding |
| snoDB1677 | U3 | H_sapiens | Animals | intergenic_cluster | C/D | | | intergenic |
| snoDB1678 | snoU13 | H_sapiens | Animals | mono_intergenic | C/D | | | intergenic |
| snoDB1679 | snoU13 | H_sapiens | Animals | mono_intergenic | C/D | | | intergenic |
| snoDB1680 | snoU13 | H_sapiens | Animals | mono_intergenic | C/D | | | intergenic |
| snoDB1681 | U3 | H_sapiens | Animals | mono_intergenic | C/D | | | intergenic |
| snoDB1682 | snoU13 | H_sapiens | Animals | mono_intergenic | C/D | | | intergenic |
| snoDB1683 | snoU13 | H_sapiens | Animals | mono_intergenic | C/D | | | intergenic |
| snoDB1684 | snoU13 | H_sapiens | Animals | mono_intergenic | C/D | | | intergenic |
| snoDB1685 | snoU13 | H_sapiens | Animals | mono_intergenic | C/D | | | intergenic |
| snoDB1686 | snoU13 | H_sapiens | Animals | mono_intergenic | C/D | | | intergenic |
| snoDB1687 | snoU13 | H_sapiens | Animals | mono_intronic | C/D | ENSG00000157800 | SLC37A3 | protein_coding |
| snoDB1688 | snoU13 | H_sapiens | Animals | mono_intergenic | C/D | | | intergenic |
| snoDB1689 | SNORD45AL1 | H_sapiens | Animals | mono_intergenic | C/D | | | intergenic |
| snoDB1690 | snoU13 | H_sapiens | Animals | mono_intergenic | C/D | | | intergenic |
| snoDB1691 | snoU13 | H_sapiens | Animals | mono_intronic | C/D | ENSG00000109332 | UBE2D3 | protein_coding |
| snoDB1692 | snoU13 | H_sapiens | Animals | mono_intergenic | C/D | | | intergenic |
| snoDB1693 | snoU13 | H_sapiens | Animals | mono_intergenic | C/D | | | intergenic |
| snoDB1694 | snoU13 | H_sapiens | Animals | mono_intergenic | C/D | | | intergenic |
| snoDB1695 | snoU13 | H_sapiens | Animals | mono_intergenic | C/D | | | intergenic |
| snoDB1696 | snoU13 | H_sapiens | Animals | mono_intergenic | C/D | | | intergenic |
| snoDB1697 | snoU13 | H_sapiens | Animals | mono_intronic | C/D | ENSG00000183230 | CTNNA3 | protein_coding |
| snoDB1698 | snoU13 | H_sapiens | Animals | mono_intronic | C/D | ENSG00000065526 | SPEN | protein_coding |
| snoDB1699 | snoU13 | H_sapiens | Animals | mono_intergenic | C/D | | | intergenic |
| snoDB1700 | snoU13 | H_sapiens | Animals | mono_intergenic | C/D | | | intergenic |
| snoDB1701 | snoU13 | H_sapiens | Animals | mono_intronic | C/D | ENSG00000198947 | DMD | protein_coding |
| snoDB1702 | U3 | H_sapiens | Animals | mono_intergenic | C/D | | | intergenic |
| snoDB1703 | snoU13 | H_sapiens | Animals | mono_intergenic | C/D | | | intergenic |
| snoDB1704 | snoU13 | H_sapiens | Animals | mono_intergenic | C/D | | | intergenic |
| snoDB1705 | U3 | H_sapiens | Animals | mono_intronic | C/D | ENSG00000006453 | BAIAP2L1 | protein_coding |
| snoDB1706 | snoU13 | H_sapiens | Animals | mono_intergenic | C/D | | | intergenic |
| snoDB1707 | snoU13 | H_sapiens | Animals | mono_intergenic | C/D | | | intergenic |
| snoDB1708 | snoU13 | H_sapiens | Animals | mono_intergenic | C/D | | | intergenic |
| snoDB1709 | snoU13 | H_sapiens | Animals | mono_intergenic | C/D | | | intergenic |
| snoDB1710 | U3 | H_sapiens | Animals | mono_intergenic | C/D | | | intergenic |
| snoDB1711 | U3 | H_sapiens | Animals | mono_intronic | C/D | ENSG00000115556 | PLCD4 | protein_coding |
| snoDB1712 | SNORA70FL2 | H_sapiens | Animals | mono_intergenic | H/ACA | | | intergenic |
| snoDB1713 | snoU13 | H_sapiens | Animals | mono_intergenic | C/D | | | intergenic |
| snoDB1714 | snoU13 | H_sapiens | Animals | mono_exonic | C/D | ENSG00000230314 | ELOVL2-AS1 | non_coding |
| snoDB1715 | snoU13 | H_sapiens | Animals | mono_intergenic | C/D | | | intergenic |
| snoDB1716 | snoU13 | H_sapiens | Animals | mono_intronic | C/D | ENSG00000101745 | ANKRD12 | protein_coding |

| | | | | | | | | |
|---|---|---|---|---|---|---|---|---|
| snoDB1717 | snoU13 | H_sapiens | Animals | mono_intergenic | C/D | | | intergenic |
| snoDB1718 | snoU13 | H_sapiens | Animals | mono_intronic | C/D | ENSG00000158169 | FANCC | protein_coding |
| snoDB1719 | snoU13 | H_sapiens | Animals | mono_intergenic | C/D | | | intergenic |
| snoDB1720 | U3 | H_sapiens | Animals | mono_intergenic | C/D | | | intergenic |
| snoDB1721 | snoU13 | H_sapiens | Animals | mono_intronic | C/D | ENSG00000158806 | NPM2 | protein_coding |
| snoDB1722 | U3 | H_sapiens | Animals | mono_intergenic | C/D | | | intergenic |
| snoDB1723 | snoU13 | H_sapiens | Animals | mono_intergenic | C/D | | | intergenic |
| snoDB1724 | snoU13 | H_sapiens | Animals | mono_intergenic | C/D | | | intergenic |
| snoDB1725 | snoU13 | H_sapiens | Animals | mono_intergenic | C/D | | | intergenic |
| snoDB1726 | snoU13 | H_sapiens | Animals | mono_intergenic | C/D | | | intergenic |
| snoDB1727 | snoU13 | H_sapiens | Animals | mono_intergenic | C/D | | | intergenic |
| snoDB1728 | snoU13 | H_sapiens | Animals | mono_intergenic | C/D | | | intergenic |
| snoDB1729 | snoU13 | H_sapiens | Animals | mono_intronic | C/D | ENSG00000166164 | BRD7 | protein_coding |
| snoDB1730 | snoU13 | H_sapiens | Animals | mono_intergenic | C/D | | | intergenic |
| snoDB1731 | snoU13 | H_sapiens | Animals | mono_intronic | C/D | ENSG00000137691 | CFAP300 | protein_coding |
| snoDB1732 | snoU13 | H_sapiens | Animals | mono_intronic | C/D | ENSG00000104154 | SLC30A4 | protein_coding |
| snoDB1733 | U3 | H_sapiens | Animals | mono_intergenic | C/D | | | intergenic |
| snoDB1734 | snoU13 | H_sapiens | Animals | mono_intergenic | C/D | | | intergenic |
| snoDB1735 | snoU13 | H_sapiens | Animals | mono_intergenic | C/D | | | intergenic |
| snoDB1736 | snoU13 | H_sapiens | Animals | mono_intronic | C/D | ENSG00000092871 | RFFL | protein_coding |
| snoDB1737 | snoU13 | H_sapiens | Animals | mono_intergenic | C/D | | | intergenic |
| snoDB1738 | snoU13 | H_sapiens | Animals | mono_intergenic | C/D | | | intergenic |
| snoDB1739 | snoU13 | H_sapiens | Animals | mono_intergenic | C/D | | | intergenic |
| snoDB1740 | snoU13 | H_sapiens | Animals | mono_exonic | C/D | ENSG00000215712 | TMEM242 | protein_coding |
| snoDB1741 | snoU13 | H_sapiens | Animals | mono_intergenic | C/D | | | intergenic |
| snoDB1742 | snoU13 | H_sapiens | Animals | mono_intergenic | C/D | | | intergenic |
| snoDB1743 | U3 | H_sapiens | Animals | mono_intergenic | C/D | | | intergenic |
| snoDB1744 | snoU13 | H_sapiens | Animals | mono_intergenic | C/D | | | intergenic |
| snoDB1745 | snoU13 | H_sapiens | Animals | mono_intronic | C/D | ENSG00000137845 | ADAM10 | protein_coding |
| snoDB1746 | snoU13 | H_sapiens | Animals | mono_intronic | C/D | ENSG00000128245 | YWHAH | protein_coding |
| snoDB1747 | snoU13 | H_sapiens | Animals | mono_intergenic | C/D | | | intergenic |
| snoDB1748 | snoU13 | H_sapiens | Animals | mono_intergenic | C/D | | | intergenic |
| snoDB1749 | snoU13 | H_sapiens | Animals | mono_intergenic | C/D | | | intergenic |
| snoDB1750 | snoU13 | H_sapiens | Animals | mono_intergenic | C/D | | | intergenic |
| snoDB1751 | snoU13 | H_sapiens | Animals | mono_intergenic | C/D | | | intergenic |
| snoDB1752 | U3 | H_sapiens | Animals | mono_intergenic | C/D | | | intergenic |
| snoDB1753 | SNORD66 | H_sapiens | Animals | mono_intergenic | C/D | | | intergenic |
| snoDB1754 | snoU13 | H_sapiens | Animals | mono_intronic | C/D | ENSG00000234352 | | non_coding |
| snoDB1755 | snoU13 | H_sapiens | Animals | mono_intergenic | C/D | | | intergenic |
| snoDB1756 | U3 | H_sapiens | Animals | mono_intergenic | C/D | | | intergenic |
| snoDB1757 | snoU13 | H_sapiens | Animals | mono_intergenic | C/D | | | intergenic |
| snoDB1758 | snoU13 | H_sapiens | Animals | mono_intergenic | C/D | | | intergenic |
| snoDB1759 | snoU13 | H_sapiens | Animals | mono_intergenic | C/D | | | intergenic |
| snoDB1760 | snoU13 | H_sapiens | Animals | mono_intergenic | C/D | | | intergenic |
| snoDB1761 | snoU13 | H_sapiens | Animals | mono_intergenic | C/D | | | intergenic |

| | | | | | | | | |
|---|---|---|---|---|---|---|---|---|
| snoDB1762 | snoU13 | H_sapiens | Animals | mono_intergenic | C/D | | | intergenic |
| snoDB1763 | snoU13 | H_sapiens | Animals | mono_intergenic | C/D | | | intergenic |
| snoDB1764 | SNORA20 | H_sapiens | Animals | mono_intronic | H/ACA | ENSG00000183878 | UTY | protein_coding |
| snoDB1765 | snoU13 | H_sapiens | Animals | mono_intergenic | C/D | | | intergenic |
| snoDB1766 | snoU13 | H_sapiens | Animals | mono_intronic | C/D | ENSG00000196236 | XPNPEP3 | protein_coding |
| snoDB1767 | snoU13 | H_sapiens | Animals | mono_intergenic | C/D | | | intergenic |
| snoDB1768 | snoU13 | H_sapiens | Animals | mono_intergenic | C/D | | | intergenic |
| snoDB1769 | snoU13 | H_sapiens | Animals | mono_intergenic | C/D | | | intergenic |
| snoDB1770 | snoU13 | H_sapiens | Animals | mono_intronic | C/D | ENSG00000138411 | HECW2 | protein_coding |
| snoDB1771 | snoU13 | H_sapiens | Animals | mono_intronic | C/D | ENSG00000133401 | PDZD2 | protein_coding |
| snoDB1772 | snoU13 | H_sapiens | Animals | mono_intergenic | C/D | | | intergenic |
| snoDB1773 | snoU13 | H_sapiens | Animals | mono_intronic | C/D | ENSG00000261556 | SMG1P7 | non_coding |
| snoDB1774 | snoU13 | H_sapiens | Animals | mono_intronic | C/D | ENSG00000065665 | SEC61A2 | protein_coding |
| snoDB1775 | snoU13 | H_sapiens | Animals | mono_intergenic | C/D | | | intergenic |
| snoDB1776 | snoU13 | H_sapiens | Animals | mono_intergenic | C/D | | | intergenic |
| snoDB1777 | snoU13 | H_sapiens | Animals | mono_intergenic | C/D | | | intergenic |
| snoDB1778 | snoU13 | H_sapiens | Animals | mono_intergenic | C/D | | | intergenic |
| snoDB1779 | snoU13 | H_sapiens | Animals | mono_intergenic | C/D | | | intergenic |
| snoDB1780 | snoU13 | H_sapiens | Animals | mono_intergenic | C/D | | | intergenic |
| snoDB1781 | snoU13 | H_sapiens | Animals | mono_intergenic | C/D | | | intergenic |
| snoDB1782 | U3 | H_sapiens | Animals | mono_intergenic | C/D | | | intergenic |
| snoDB1783 | SNORA48 | H_sapiens | Animals | mono_intergenic | H/ACA | | | intergenic |
| snoDB1784 | snoU13 | H_sapiens | Animals | mono_exonic | C/D | ENSG00000112981 | NME5 | protein_coding |
| snoDB1785 | snoU13 | H_sapiens | Animals | mono_intronic | C/D | ENSG00000251442 | LINC01094 | non_coding |
| snoDB1786 | snoU13 | H_sapiens | Animals | mono_intergenic | C/D | | | intergenic |
| snoDB1787 | snoU13 | H_sapiens | Animals | mono_intergenic | C/D | | | intergenic |
| snoDB1788 | snoU13 | H_sapiens | Animals | mono_intronic | C/D | ENSG00000249494 | DMXL1-DT | non_coding |
| snoDB1789 | snoU13 | H_sapiens | Animals | mono_intergenic | C/D | | | intergenic |
| snoDB1790 | snoU13 | H_sapiens | Animals | mono_exonic | C/D | ENSG00000243321 | LINC01998 | non_coding |
| snoDB1791 | snoU13 | H_sapiens | Animals | mono_intronic | C/D | ENSG00000128739 | SNRPN | protein_coding |
| snoDB1792 | snoU13 | H_sapiens | Animals | mono_intergenic | C/D | | | intergenic |
| snoDB1793 | snoU13 | H_sapiens | Animals | mono_intronic | C/D | ENSG00000205581 | HMGN1 | protein_coding |
| snoDB1794 | snoU13 | H_sapiens | Animals | mono_intronic | C/D | ENSG00000164796 | CSMD3 | protein_coding |
| snoDB1795 | U3 | H_sapiens | Animals | mono_intronic | C/D | ENSG00000196323 | ZBTB44 | protein_coding |
| snoDB1796 | snoU13 | H_sapiens | Animals | mono_intergenic | C/D | | | intergenic |
| snoDB1797 | snoU13 | H_sapiens | Animals | mono_intergenic | C/D | | | intergenic |
| snoDB1798 | snoU13 | H_sapiens | Animals | mono_intergenic | C/D | | | intergenic |
| snoDB1799 | snoU13 | H_sapiens | Animals | mono_intronic | C/D | ENSG00000259305 | ZHX1-C8orf76 | protein_coding |
| snoDB1800 | snoU13 | H_sapiens | Animals | mono_intronic | C/D | ENSG00000149136 | SSRP1 | protein_coding |
| snoDB1801 | snoU13 | H_sapiens | Animals | mono_intergenic | C/D | | | intergenic |
| snoDB1802 | snoU13 | H_sapiens | Animals | mono_intergenic | C/D | | | intergenic |
| snoDB1803 | SNORD71 | H_sapiens | Animals | mono_intronic | C/D | ENSG00000259200 | | non_coding |
| snoDB1804 | snoU13 | H_sapiens | Animals | mono_intergenic | C/D | | | intergenic |
| snoDB1805 | snoU13 | H_sapiens | Animals | mono_intronic | C/D | ENSG00000183715 | OPCML | protein_coding |
| snoDB1806 | snoU13 | H_sapiens | Animals | mono_intergenic | C/D | | | intergenic |

| | | | | | | | | |
|---|---|---|---|---|---|---|---|---|
| snoDB1807 | snoU13 | H_sapiens | Animals | mono_intergenic | C/D | | | intergenic |
| snoDB1808 | snoU13 | H_sapiens | Animals | mono_intronic | C/D | ENSG00000008294 | SPAG9 | protein_coding |
| snoDB1809 | snoU13 | H_sapiens | Animals | mono_intronic | C/D | ENSG00000109099 | PMP22 | protein_coding |
| snoDB1810 | snoU13 | H_sapiens | Animals | intergenic_cluster | C/D | | | intergenic |
| snoDB1811 | snoU13 | H_sapiens | Animals | mono_intergenic | C/D | | | intergenic |
| snoDB1812 | snoU13 | H_sapiens | Animals | mono_intergenic | C/D | | | intergenic |
| snoDB1813 | snoU13 | H_sapiens | Animals | mono_intergenic | C/D | | | intergenic |
| snoDB1814 | snoU13 | H_sapiens | Animals | mono_intronic | C/D | ENSG00000183831 | ANKRD45 | protein_coding |
| snoDB1815 | snoU13 | H_sapiens | Animals | mono_intergenic | C/D | | | intergenic |
| snoDB1816 | snoU13 | H_sapiens | Animals | mono_intergenic | C/D | | | intergenic |
| snoDB1817 | snoU13 | H_sapiens | Animals | mono_intronic | C/D | ENSG00000171791 | BCL2 | protein_coding |
| snoDB1818 | snoU13 | H_sapiens | Animals | mono_intronic | C/D | ENSG00000079691 | CARMIL1 | protein_coding |
| snoDB1819 | snoU13 | H_sapiens | Animals | mono_intronic | C/D | ENSG00000227930 | | non_coding |
| snoDB1820 | snoU13 | H_sapiens | Animals | mono_intergenic | C/D | | | intergenic |
| snoDB1821 | snoU13 | H_sapiens | Animals | mono_intergenic | C/D | | | intergenic |
| snoDB1822 | snoU13 | H_sapiens | Animals | mono_intergenic | C/D | | | intergenic |
| snoDB1823 | snoU13 | H_sapiens | Animals | mono_intronic | C/D | ENSG00000231171 | LINC01098 | non_coding |
| snoDB1824 | snoU13 | H_sapiens | Animals | mono_intergenic | C/D | | | intergenic |
| snoDB1825 | snoU13 | H_sapiens | Animals | mono_intronic | C/D | ENSG00000112530 | PACRG | protein_coding |
| snoDB1826 | snoU13 | H_sapiens | Animals | mono_intergenic | C/D | | | intergenic |
| snoDB1827 | snoU13 | H_sapiens | Animals | mono_intronic | C/D | ENSG00000203907 | OOEP | protein_coding |
| snoDB1828 | snoU13 | H_sapiens | Animals | mono_intronic | C/D | ENSG00000229846 | | non_coding |
| snoDB1829 | snoU13 | H_sapiens | Animals | mono_intergenic | C/D | | | intergenic |
| snoDB1830 | U3 | H_sapiens | Animals | mono_intronic | C/D | ENSG00000057019 | DCBLD2 | protein_coding |
| snoDB1831 | snoU13 | H_sapiens | Animals | mono_intergenic | C/D | | | intergenic |
| snoDB1832 | snoU13 | H_sapiens | Animals | mono_intronic | C/D | ENSG00000196586 | MYO6 | protein_coding |
| snoDB1833 | snoU13 | H_sapiens | Animals | mono_intergenic | C/D | | | intergenic |
| snoDB1834 | snoU13 | H_sapiens | Animals | mono_intergenic | C/D | | | intergenic |
| snoDB1835 | snoU13 | H_sapiens | Animals | mono_intergenic | C/D | | | intergenic |
| snoDB1836 | snoU13 | H_sapiens | Animals | mono_intergenic | C/D | | | intergenic |
| snoDB1837 | snoU13 | H_sapiens | Animals | mono_intergenic | C/D | | | intergenic |
| snoDB1838 | snoU13 | H_sapiens | Animals | mono_intronic | C/D | ENSG00000170579 | DLGAP1 | protein_coding |
| snoDB1839 | snoU13 | H_sapiens | Animals | mono_intergenic | C/D | | | intergenic |
| snoDB1840 | snoU13 | H_sapiens | Animals | mono_intergenic | C/D | | | intergenic |
| snoDB1841 | snoU13 | H_sapiens | Animals | mono_intronic | C/D | ENSG00000204764 | RANBP17 | protein_coding |
| snoDB1842 | snoU13 | H_sapiens | Animals | mono_intergenic | C/D | | | intergenic |
| snoDB1843 | snoU13 | H_sapiens | Animals | mono_intergenic | C/D | | | intergenic |
| snoDB1844 | snoU13 | H_sapiens | Animals | mono_intronic | C/D | ENSG00000067704 | IARS2 | protein_coding |
| snoDB1845 | snoU13 | H_sapiens | Animals | mono_intergenic | C/D | | | intergenic |
| snoDB1846 | snoU13 | H_sapiens | Animals | mono_intergenic | C/D | | | intergenic |
| snoDB1847 | snoU13 | H_sapiens | Animals | mono_intergenic | C/D | | | intergenic |
| snoDB1848 | SNORD43 | H_sapiens | Animals | mono_intergenic | C/D | | | intergenic |
| snoDB1849 | snoU13 | H_sapiens | Animals | mono_intronic | C/D | ENSG00000290046 | | non_coding |
| snoDB1850 | snoU13 | H_sapiens | Animals | mono_intronic | C/D | ENSG00000116539 | ASH1L | protein_coding |
| snoDB1851 | snoU13 | H_sapiens | Animals | mono_intergenic | C/D | | | intergenic |

| | | | | | | | | |
|---|---|---|---|---|---|---|---|---|
| snoDB1852 | snoU13 | H_sapiens | Animals | mono_intronic | C/D | ENSG00000155115 | GTF3C6 | protein_coding |
| snoDB1853 | snoU13 | H_sapiens | Animals | mono_intergenic | C/D | | | intergenic |
| snoDB1854 | snoU13 | H_sapiens | Animals | mono_intergenic | C/D | | | intergenic |
| snoDB1855 | snoU13 | H_sapiens | Animals | mono_intronic | C/D | ENSG00000284922 | LRTOMT | protein_coding |
| snoDB1856 | snoU13 | H_sapiens | Animals | mono_intergenic | C/D | | | intergenic |
| snoDB1857 | snoU13 | H_sapiens | Animals | mono_intergenic | C/D | | | intergenic |
| snoDB1858 | snoU13 | H_sapiens | Animals | mono_intergenic | C/D | | | intergenic |
| snoDB1859 | snoU13 | H_sapiens | Animals | mono_intergenic | C/D | | | intergenic |
| snoDB1860 | snoU13 | H_sapiens | Animals | mono_intergenic | C/D | | | intergenic |
| snoDB1861 | snoU13 | H_sapiens | Animals | mono_intergenic | C/D | | | intergenic |
| snoDB1862 | snoU13 | H_sapiens | Animals | mono_intergenic | C/D | | | intergenic |
| snoDB1863 | snoU13 | H_sapiens | Animals | mono_intronic | C/D | ENSG00000158528 | PPP1R9A | protein_coding |
| snoDB1864 | snoU13 | H_sapiens | Animals | mono_intergenic | C/D | | | intergenic |
| snoDB1865 | snoU13 | H_sapiens | Animals | mono_intronic | C/D | ENSG00000141568 | FOXK2 | protein_coding |
| snoDB1866 | snoU13 | H_sapiens | Animals | mono_intergenic | C/D | | | intergenic |
| snoDB1867 | snoU13 | H_sapiens | Animals | mono_intergenic | C/D | | | intergenic |
| snoDB1868 | U3 | H_sapiens | Animals | mono_intronic | C/D | ENSG00000137270 | GCM1 | protein_coding |
| snoDB1869 | snoU13 | H_sapiens | Animals | mono_intergenic | C/D | | | intergenic |
| snoDB1870 | snoU13 | H_sapiens | Animals | mono_intergenic | C/D | | | intergenic |
| snoDB1871 | snoU13 | H_sapiens | Animals | mono_intergenic | C/D | | | intergenic |
| snoDB1872 | snoU13 | H_sapiens | Animals | mono_intergenic | C/D | | | intergenic |
| snoDB1873 | snoU13 | H_sapiens | Animals | mono_intergenic | C/D | | | intergenic |
| snoDB1874 | snoU13 | H_sapiens | Animals | mono_intergenic | C/D | | | intergenic |
| snoDB1875 | U3 | H_sapiens | Animals | mono_intronic | C/D | ENSG00000132388 | UBE2G1 | protein_coding |
| snoDB1876 | U3 | H_sapiens | Animals | mono_intergenic | C/D | | | intergenic |
| snoDB1877 | snoU13 | H_sapiens | Animals | mono_intergenic | C/D | | | intergenic |
| snoDB1878 | snoU13 | H_sapiens | Animals | mono_intergenic | C/D | | | intergenic |
| snoDB1879 | snoU13 | H_sapiens | Animals | mono_intronic | C/D | ENSG00000134278 | SPIRE1 | protein_coding |
| snoDB1880 | snoU13 | H_sapiens | Animals | mono_intergenic | C/D | | | intergenic |
| snoDB1881 | snoU13 | H_sapiens | Animals | mono_intronic | C/D | ENSG00000144962 | SPATA16 | protein_coding |
| snoDB1882 | snoU13 | H_sapiens | Animals | mono_intergenic | C/D | | | intergenic |
| snoDB1883 | snoU13 | H_sapiens | Animals | mono_intronic | C/D | ENSG00000180198 | RCC1 | protein_coding |
| snoDB1884 | snoU13 | H_sapiens | Animals | mono_intergenic | C/D | | | intergenic |
| snoDB1885 | snoU13 | H_sapiens | Animals | mono_intergenic | C/D | | | intergenic |
| snoDB1886 | snoU13 | H_sapiens | Animals | mono_intergenic | C/D | | | intergenic |
| snoDB1887 | snoU13 | H_sapiens | Animals | mono_intronic | C/D | ENSG00000120051 | CFAP58 | protein_coding |
| snoDB1888 | snoU13 | H_sapiens | Animals | intronic_cluster | C/D | ENSG00000101972 | STAG2 | protein_coding |
| snoDB1889 | snoU13 | H_sapiens | Animals | mono_intronic | C/D | ENSG00000165091 | TMC1 | protein_coding |
| snoDB1890 | snoU13 | H_sapiens | Animals | mono_intergenic | C/D | | | intergenic |
| snoDB1891 | snoU13 | H_sapiens | Animals | mono_intronic | C/D | ENSG00000147419 | CCDC25 | protein_coding |
| snoDB1892 | snoU13 | H_sapiens | Animals | mono_intronic | C/D | ENSG00000135127 | BICDL1 | protein_coding |
| snoDB1893 | snoU13 | H_sapiens | Animals | mono_intergenic | C/D | | | intergenic |
| snoDB1894 | snoU13 | H_sapiens | Animals | mono_intronic | C/D | ENSG00000104517 | UBR5 | protein_coding |
| snoDB1895 | snoU13 | H_sapiens | Animals | mono_intergenic | C/D | | | intergenic |
| snoDB1896 | U3 | H_sapiens | Animals | mono_intronic | C/D | ENSG00000099942 | CRKL | protein_coding |

| | | | | | | | | |
|---|---|---|---|---|---|---|---|---|
| snoDB1897 | snoU13 | H_sapiens | Animals | mono_intergenic | C/D | | | intergenic |
| snoDB1898 | snoU13 | H_sapiens | Animals | mono_intronic | C/D | ENSG00000172738 | TMEM217 | protein_coding |
| snoDB1899 | snoU13 | H_sapiens | Animals | mono_intergenic | C/D | | | intergenic |
| snoDB1900 | snoU13 | H_sapiens | Animals | mono_intergenic | C/D | | | intergenic |
| snoDB1901 | snoU13 | H_sapiens | Animals | mono_intergenic | C/D | | | intergenic |
| snoDB1902 | snoU13 | H_sapiens | Animals | mono_intronic | C/D | ENSG00000198246 | SLC29A3 | protein_coding |
| snoDB1903 | snoU13 | H_sapiens | Animals | mono_intergenic | C/D | | | intergenic |
| snoDB1904 | snoU13 | H_sapiens | Animals | mono_intergenic | C/D | | | intergenic |
| snoDB1905 | snoU13 | H_sapiens | Animals | mono_intergenic | C/D | | | intergenic |
| snoDB1906 | snoU13 | H_sapiens | Animals | mono_intergenic | C/D | | | intergenic |
| snoDB1907 | snoU13 | H_sapiens | Animals | mono_intronic | C/D | ENSG00000260908 | PLA2G10CP | non_coding |
| snoDB1908 | snoU13 | H_sapiens | Animals | mono_intergenic | C/D | | | intergenic |
| snoDB1909 | snoU13 | H_sapiens | Animals | mono_intronic | C/D | ENSG00000115468 | EFHD1 | protein_coding |
| snoDB1910 | snoU13 | H_sapiens | Animals | intergenic_cluster | C/D | | | intergenic |
| snoDB1911 | snoU13 | H_sapiens | Animals | mono_intronic | C/D | ENSG00000266850 | | non_coding |
| snoDB1912 | U3 | H_sapiens | Animals | mono_intronic | C/D | ENSG00000274015 | | non_coding |
| snoDB1913 | snoU13 | H_sapiens | Animals | mono_intergenic | C/D | | | intergenic |
| snoDB1914 | snoU13 | H_sapiens | Animals | mono_intronic | C/D | ENSG00000047315 | POLR2B | protein_coding |
| snoDB1915 | snoU13 | H_sapiens | Animals | mono_intergenic | C/D | | | intergenic |
| snoDB1916 | snoU13 | H_sapiens | Animals | mono_intergenic | C/D | | | intergenic |
| snoDB1917 | snoU13 | H_sapiens | Animals | mono_intronic | C/D | ENSG00000151694 | ADAM17 | protein_coding |
| snoDB1918 | snoU13 | H_sapiens | Animals | mono_intergenic | C/D | | | intergenic |
| snoDB1919 | snoU13 | H_sapiens | Animals | mono_intronic | C/D | ENSG00000227115 | LINC01630 | non_coding |
| snoDB1920 | snoU13 | H_sapiens | Animals | mono_intronic | C/D | ENSG00000134758 | RNF138 | protein_coding |
| snoDB1921 | snoU13 | H_sapiens | Animals | mono_intronic | C/D | ENSG00000173120 | KDM2A | protein_coding |
| snoDB1922 | snoU13 | H_sapiens | Animals | mono_intergenic | C/D | | | intergenic |
| snoDB1923 | snoU13 | H_sapiens | Animals | mono_intronic | C/D | ENSG00000288208 | | protein_coding |
| snoDB1924 | snoU13 | H_sapiens | Animals | mono_intergenic | C/D | | | intergenic |
| snoDB1925 | SNORA51 | H_sapiens | Animals | mono_intergenic | H/ACA | | | intergenic |
| snoDB1926 | snoU13 | H_sapiens | Animals | mono_intergenic | C/D | | | intergenic |
| snoDB1927 | snoU13 | H_sapiens | Animals | mono_intronic | C/D | ENSG00000088367 | EPB41L1 | protein_coding |
| snoDB1928 | snoU13 | H_sapiens | Animals | mono_intronic | C/D | ENSG00000128829 | EIF2AK4 | protein_coding |
| snoDB1929 | snoU13 | H_sapiens | Animals | mono_intronic | C/D | ENSG00000168754 | FAM178B | protein_coding |
| snoDB1930 | snoU13 | H_sapiens | Animals | mono_intronic | C/D | ENSG00000091536 | MYO15A | protein_coding |
| snoDB1931 | snoU13 | H_sapiens | Animals | mono_intergenic | C/D | | | intergenic |
| snoDB1932 | snoU13 | H_sapiens | Animals | mono_intergenic | C/D | | | intergenic |
| snoDB1933 | snoU13 | H_sapiens | Animals | mono_intergenic | C/D | | | intergenic |
| snoDB1934 | snoU13 | H_sapiens | Animals | mono_intronic | C/D | ENSG00000127838 | PNKD | protein_coding |
| snoDB1935 | snoU13 | H_sapiens | Animals | mono_intronic | C/D | ENSG00000205302 | SNX2 | protein_coding |
| snoDB1936 | snoU13 | H_sapiens | Animals | mono_intergenic | C/D | | | intergenic |
| snoDB1937 | snoU13 | H_sapiens | Animals | mono_intergenic | C/D | | | intergenic |
| snoDB1938 | snoU13 | H_sapiens | Animals | mono_intergenic | C/D | | | intergenic |
| snoDB1939 | SNORA36C | H_sapiens | Animals | mono_intronic | H/ACA | ENSG00000113578 | FGF1 | protein_coding |
| snoDB1940 | snoU13 | H_sapiens | Animals | mono_intronic | C/D | ENSG00000181234 | TMEM132C | protein_coding |
| snoDB1941 | snoU13 | H_sapiens | Animals | mono_intergenic | C/D | | | intergenic |

| | | | | | | | | |
|---|---|---|---|---|---|---|---|---|
| snoDB1942 | snoU13 | H_sapiens | Animals | mono_intronic | C/D | ENSG00000198793 | MTOR | protein_coding |
| snoDB1943 | snoU13 | H_sapiens | Animals | mono_intergenic | C/D | | | intergenic |
| snoDB1944 | snoU13 | H_sapiens | Animals | mono_intronic | C/D | ENSG00000073803 | MAP3K13 | protein_coding |
| snoDB1945 | snoU13 | H_sapiens | Animals | mono_intergenic | C/D | | | intergenic |
| snoDB1946 | snoU13 | H_sapiens | Animals | mono_intronic | C/D | ENSG00000231365 | WARS2-AS1 | non_coding |
| snoDB1947 | snoU13 | H_sapiens | Animals | mono_intergenic | C/D | | | intergenic |
| snoDB1948 | snoU13 | H_sapiens | Animals | mono_intergenic | C/D | | | intergenic |
| snoDB1949 | snoU13 | H_sapiens | Animals | mono_intergenic | C/D | | | intergenic |
| snoDB1950 | snoU13 | H_sapiens | Animals | mono_intergenic | C/D | | | intergenic |
| snoDB1951 | snoU13 | H_sapiens | Animals | mono_intergenic | C/D | | | intergenic |
| snoDB1952 | snoU13 | H_sapiens | Animals | mono_intergenic | C/D | | | intergenic |
| snoDB1953 | snoU13 | H_sapiens | Animals | mono_intronic | C/D | ENSG00000164300 | SERINC5 | protein_coding |
| snoDB1954 | snoU13 | H_sapiens | Animals | mono_intergenic | C/D | | | intergenic |
| snoDB1955 | snoU13 | H_sapiens | Animals | mono_intergenic | C/D | | | intergenic |
| snoDB1956 | snoU13 | H_sapiens | Animals | mono_intronic | C/D | ENSG00000231427 | LINC01445 | non_coding |
| snoDB1957 | snoU13 | H_sapiens | Animals | mono_intergenic | C/D | | | intergenic |
| snoDB1958 | snoU13 | H_sapiens | Animals | mono_intronic | C/D | ENSG00000174373 | RALGAPA1 | protein_coding |
| snoDB1959 | snoU13 | H_sapiens | Animals | mono_intergenic | C/D | | | intergenic |
| snoDB1960 | snoU13 | H_sapiens | Animals | mono_intronic | C/D | ENSG00000184903 | IMMP2L | protein_coding |
| snoDB1961 | snoU13 | H_sapiens | Animals | mono_intergenic | C/D | | | intergenic |
| snoDB1962 | snoU13 | H_sapiens | Animals | mono_intergenic | C/D | | | intergenic |
| snoDB1963 | snoU13 | H_sapiens | Animals | mono_intronic | C/D | ENSG00000203356 | LINC01562 | non_coding |
| snoDB1964 | snoU13 | H_sapiens | Animals | mono_intronic | C/D | ENSG00000007168 | PAFAH1B1 | protein_coding |
| snoDB1965 | snoU13 | H_sapiens | Animals | mono_intergenic | C/D | | | intergenic |
| snoDB1966 | snoU13 | H_sapiens | Animals | mono_intergenic | C/D | | | intergenic |
| snoDB1967 | snoU13 | H_sapiens | Animals | mono_intergenic | C/D | | | intergenic |
| snoDB1968 | snoU13 | H_sapiens | Animals | mono_intergenic | C/D | | | intergenic |
| snoDB1969 | snoU13 | H_sapiens | Animals | mono_intronic | C/D | ENSG00000284057 | | protein_coding |
| snoDB1970 | snoU13 | H_sapiens | Animals | mono_intronic | C/D | ENSG00000137145 | DENND4C | protein_coding |
| snoDB1971 | snoU13 | H_sapiens | Animals | mono_intronic | C/D | ENSG00000130707 | ASS1 | protein_coding |
| snoDB1972 | snoU13 | H_sapiens | Animals | mono_intronic | C/D | ENSG00000059728 | MXD1 | protein_coding |
| snoDB1973 | snoU13 | H_sapiens | Animals | mono_intergenic | C/D | | | intergenic |
| snoDB1974 | snoU13 | H_sapiens | Animals | mono_intronic | C/D | ENSG00000095015 | MAP3K1 | protein_coding |
| snoDB1975 | snoU13 | H_sapiens | Animals | mono_intronic | C/D | ENSG00000241369 | LINC01192 | non_coding |
| snoDB1976 | SNORA77 | H_sapiens | Animals | mono_intergenic | H/ACA | | | intergenic |
| snoDB1977 | snoU13 | H_sapiens | Animals | mono_intergenic | C/D | | | intergenic |
| snoDB1978 | snoU13 | H_sapiens | Animals | mono_intronic | C/D | ENSG00000187231 | SESTD1 | protein_coding |
| snoDB1979 | snoU13 | H_sapiens | Animals | mono_intronic | C/D | ENSG00000108055 | SMC3 | protein_coding |
| snoDB1980 | snoU13 | H_sapiens | Animals | mono_intergenic | C/D | | | intergenic |
| snoDB1981 | snoU13 | H_sapiens | Animals | mono_intergenic | C/D | | | intergenic |
| snoDB1982 | U3 | H_sapiens | Animals | intergenic_cluster | C/D | | | intergenic |
| snoDB1983 | snoU13 | H_sapiens | Animals | mono_intergenic | C/D | | | intergenic |
| snoDB1984 | snoU13 | H_sapiens | Animals | mono_intronic | C/D | ENSG00000118515 | SGK1 | protein_coding |
| snoDB1985 | snoU13 | H_sapiens | Animals | mono_intergenic | C/D | | | intergenic |
| snoDB1986 | snoU13 | H_sapiens | Animals | mono_intronic | C/D | ENSG00000165487 | MICU2 | protein_coding |

| | | | | | | | | |
|---|---|---|---|---|---|---|---|---|
| snoDB1987 | snoU13 | H_sapiens | Animals | mono_intergenic | C/D | | | intergenic |
| snoDB1988 | snoU13 | H_sapiens | Animals | mono_intronic | C/D | ENSG00000171208 | NETO2 | protein_coding |
| snoDB1989 | snoU13 | H_sapiens | Animals | mono_intergenic | C/D | | | intergenic |
| snoDB1990 | snoU13 | H_sapiens | Animals | mono_intronic | C/D | ENSG00000237638 | LINC02245 | non_coding |
| snoDB1991 | snoU13 | H_sapiens | Animals | mono_intergenic | C/D | | | intergenic |
| snoDB1992 | snoU13 | H_sapiens | Animals | mono_intronic | C/D | ENSG00000136628 | EPRS1 | protein_coding |
| snoDB1993 | snoU13 | H_sapiens | Animals | mono_intronic | C/D | ENSG00000174844 | DNAH12 | protein_coding |
| snoDB1994 | snoU13 | H_sapiens | Animals | mono_intergenic | C/D | | | intergenic |
| snoDB1995 | U3 | H_sapiens | Animals | mono_intronic | C/D | ENSG00000143552 | NUP210L | protein_coding |
| snoDB1996 | snoU13 | H_sapiens | Animals | mono_intronic | C/D | ENSG00000157106 | SMG1 | protein_coding |
| snoDB1997 | snoU13 | H_sapiens | Animals | mono_intergenic | C/D | | | intergenic |
| snoDB1998 | snoU13 | H_sapiens | Animals | mono_intronic | C/D | ENSG00000175643 | RMI2 | protein_coding |
| snoDB1999 | snoU13 | H_sapiens | Animals | mono_intergenic | C/D | | | intergenic |
| snoDB2000 | snoU13 | H_sapiens | Animals | mono_intergenic | C/D | | | intergenic |
| snoDB2001 | snoU13 | H_sapiens | Animals | mono_intergenic | C/D | | | intergenic |
| snoDB2002 | snoU13 | H_sapiens | Animals | mono_intronic | C/D | ENSG00000101596 | SMCHD1 | protein_coding |
| snoDB2003 | SNORD81L4 | H_sapiens | Animals | mono_intergenic | C/D | | | intergenic |
| snoDB2004 | Z73421.1 | H_sapiens | Animals | mono_intronic | Unknown | ENSG00000133424 | LARGE1 | protein_coding |
| snoDB2005 | AC006023.2 | H_sapiens | Animals | mono_intergenic | C/D | | | intergenic |
| snoDB2006 | SNORD80L1 | H_sapiens | Animals | mono_intergenic | C/D | | | intergenic |
| snoDB2007 | AC138972.8 | H_sapiens | Animals | mono_intergenic | H/ACA | | | intergenic |
| snoDB2008 | AP000818.4 | H_sapiens | Animals | mono_intronic | Unknown | ENSG00000110002 | VWA5A | protein_coding |
| snoDB2009 | SNORD66L1 | H_sapiens | Animals | mono_intergenic | C/D | | | intergenic |
| snoDB2010 | SNORA63L3 | H_sapiens | Animals | mono_intergenic | H/ACA | | | intergenic |
| snoDB2011 | U3 | H_sapiens | Animals | mono_intergenic | C/D | | | intergenic |
| snoDB2012 | snoU13 | H_sapiens | Animals | mono_intergenic | C/D | | | intergenic |
| snoDB2013 | snoU13 | H_sapiens | Animals | mono_intergenic | C/D | | | intergenic |
| snoDB2014 | snoU13 | H_sapiens | Animals | mono_intergenic | C/D | | | intergenic |
| snoDB2015 | U3 | H_sapiens | Animals | mono_intergenic | C/D | | | intergenic |
| snoDB2016 | snoU13 | H_sapiens | Animals | mono_intergenic | C/D | | | intergenic |
| snoDB2017 | snoU13 | H_sapiens | Animals | mono_intronic | C/D | ENSG00000262999 | | non_coding |
| snoDB2018 | snoU13 | H_sapiens | Animals | mono_intergenic | C/D | | | intergenic |
| snoDB2019 | snoU13 | H_sapiens | Animals | mono_intergenic | C/D | | | intergenic |
| snoDB2020 | snoU13 | H_sapiens | Animals | mono_intronic | C/D | ENSG00000234423 | LINC01250 | non_coding |
| snoDB2021 | U3 | H_sapiens | Animals | mono_intergenic | C/D | | | intergenic |
| snoDB2022 | U3 | H_sapiens | Animals | mono_intronic | C/D | ENSG00000130695 | CEP85 | protein_coding |
| snoDB2023 | snoU13 | H_sapiens | Animals | mono_intergenic | C/D | | | intergenic |
| snoDB2024 | snoU13 | H_sapiens | Animals | mono_intergenic | C/D | | | intergenic |
| snoDB2025 | snoU13 | H_sapiens | Animals | mono_intronic | C/D | ENSG00000236393 | | non_coding |
| snoDB2026 | snoU13 | H_sapiens | Animals | mono_intergenic | C/D | | | intergenic |
| snoDB2027 | snoU13 | H_sapiens | Animals | mono_intergenic | C/D | | | intergenic |
| snoDB2028 | snoU13 | H_sapiens | Animals | mono_intronic | C/D | ENSG00000259065 | MIDEAS-AS1 | non_coding |
| snoDB2029 | snoU13 | H_sapiens | Animals | mono_intergenic | C/D | | | intergenic |
| snoDB2030 | snoU13 | H_sapiens | Animals | mono_intergenic | C/D | | | intergenic |
| snoDB2031 | snoU13 | H_sapiens | Animals | mono_intergenic | C/D | | | intergenic |

| | | | | | | | | |
|---|---|---|---|---|---|---|---|---|
| snoDB2032 | U3 | H_sapiens | Animals | mono_intronic | C/D | ENSG00000236491 | | non_coding |
| snoDB2033 | snoU13 | H_sapiens | Animals | mono_intergenic | C/D | | | intergenic |
| snoDB2034 | snoU13 | H_sapiens | Animals | mono_intergenic | C/D | | | intergenic |
| snoDB2035 | snoU13 | H_sapiens | Animals | mono_intergenic | C/D | | | intergenic |
| snoDB2036 | snoU13 | H_sapiens | Animals | mono_intergenic | C/D | | | intergenic |
| snoDB2037 | snoU13 | H_sapiens | Animals | mono_intergenic | C/D | | | intergenic |
| snoDB2038 | snoU13 | H_sapiens | Animals | mono_intergenic | C/D | | | intergenic |
| snoDB2039 | U3 | H_sapiens | Animals | mono_intergenic | C/D | | | intergenic |
| snoDB2040 | U3 | H_sapiens | Animals | mono_intergenic | C/D | | | intergenic |
| snoDB2041 | U3 | H_sapiens | Animals | mono_intronic | C/D | ENSG00000286329 | | non_coding |
| snoDB2042 | U3 | H_sapiens | Animals | mono_intergenic | C/D | | | intergenic |
| snoDB2043 | snoU13 | H_sapiens | Animals | mono_intronic | C/D | ENSG00000115239 | ASB3 | protein_coding |
| snoDB2044 | U3 | H_sapiens | Animals | mono_intergenic | C/D | | | intergenic |
| snoDB2045 | snoU13 | H_sapiens | Animals | mono_intronic | C/D | ENSG00000156042 | CFAP70 | protein_coding |
| snoDB2046 | SNORD39 | H_sapiens | Animals | mono_intergenic | C/D | | | intergenic |
| snoDB2047 | snoU13 | H_sapiens | Animals | mono_intergenic | C/D | | | intergenic |
| snoDB2048 | snoU13 | H_sapiens | Animals | mono_intronic | C/D | ENSG00000130175 | PRKCSH | protein_coding |
| snoDB2049 | snoU13 | H_sapiens | Animals | mono_intergenic | C/D | | | intergenic |
| snoDB2050 | snoU13 | H_sapiens | Animals | mono_intergenic | C/D | | | intergenic |
| snoDB2051 | snoU13 | H_sapiens | Animals | mono_intergenic | C/D | | | intergenic |
| snoDB2052 | snoU13 | H_sapiens | Animals | mono_intergenic | C/D | | | intergenic |
| snoDB2053 | U3 | H_sapiens | Animals | mono_intronic | C/D | ENSG00000175779 | LINC02694 | non_coding |
| snoDB2054 | snoU13 | H_sapiens | Animals | mono_intergenic | C/D | | | intergenic |
| snoDB2055 | snoU13 | H_sapiens | Animals | mono_intronic | C/D | ENSG00000267339 | LINC00906 | non_coding |
| snoDB2056 | snoU13 | H_sapiens | Animals | mono_intergenic | C/D | | | intergenic |
| snoDB2057 | U3 | H_sapiens | Animals | mono_intergenic | C/D | | | intergenic |
| snoDB2058 | snoU13 | H_sapiens | Animals | mono_intergenic | C/D | | | intergenic |
| snoDB2059 | snoU13 | H_sapiens | Animals | mono_intronic | C/D | ENSG00000235978 | | non_coding |
| snoDB2060 | U3 | H_sapiens | Animals | mono_intergenic | C/D | | | intergenic |
| snoDB2061 | snoU13 | H_sapiens | Animals | mono_intronic | C/D | ENSG00000167703 | SLC43A2 | protein_coding |
| snoDB2062 | snoU13 | H_sapiens | Animals | mono_intergenic | C/D | | | intergenic |
| snoDB2063 | snoU13 | H_sapiens | Animals | mono_intronic | C/D | ENSG00000177694 | NAALADL2 | protein_coding |
| snoDB2064 | snoU13 | H_sapiens | Animals | mono_intronic | C/D | ENSG00000128510 | CPA4 | protein_coding |
| snoDB2065 | snoU13 | H_sapiens | Animals | mono_intronic | C/D | ENSG00000088179 | PTPN4 | protein_coding |
| snoDB2066 | snoU13 | H_sapiens | Animals | mono_intergenic | C/D | | | intergenic |
| snoDB2067 | U3 | H_sapiens | Animals | mono_intergenic | C/D | | | intergenic |
| snoDB2068 | U3 | H_sapiens | Animals | mono_intergenic | C/D | | | intergenic |
| snoDB2069 | U3 | H_sapiens | Animals | mono_intergenic | C/D | | | intergenic |
| snoDB2070 | snoU13 | H_sapiens | Animals | mono_intronic | C/D | ENSG00000022277 | RTF2 | protein_coding |
| snoDB2071 | snoU13 | H_sapiens | Animals | mono_intergenic | C/D | | | intergenic |
| snoDB2072 | snoU13 | H_sapiens | Animals | mono_intergenic | C/D | | | intergenic |
| snoDB2073 | snoU13 | H_sapiens | Animals | mono_intergenic | C/D | | | intergenic |
| snoDB2074 | U3 | H_sapiens | Animals | mono_intergenic | C/D | | | intergenic |
| snoDB2075 | U3 | H_sapiens | Animals | mono_intronic | C/D | ENSG00000132549 | VPS13B | protein_coding |
| snoDB2076 | snoU13 | H_sapiens | Animals | mono_intronic | C/D | ENSG00000182329 | KIAA2012 | protein_coding |

| | | | | | | | | |
|---|---|---|---|---|---|---|---|---|
| snoDB2077 | snoU13 | H_sapiens | Animals | mono_intergenic | C/D | | | intergenic |
| snoDB2078 | snoU13 | H_sapiens | Animals | mono_intergenic | C/D | | | intergenic |
| snoDB2079 | snoU13 | H_sapiens | Animals | mono_intronic | C/D | ENSG00000133067 | LGR6 | protein_coding |
| snoDB2080 | U3 | H_sapiens | Animals | mono_intergenic | C/D | | | intergenic |
| snoDB2081 | U3 | H_sapiens | Animals | mono_intronic | C/D | ENSG00000228922 | | non_coding |
| snoDB2082 | U3 | H_sapiens | Animals | mono_intronic | C/D | ENSG00000282278 | | protein_coding |
| snoDB2083 | U3 | H_sapiens | Animals | mono_intergenic | C/D | | | intergenic |
| snoDB2084 | U3 | H_sapiens | Animals | mono_intergenic | C/D | | | intergenic |
| snoDB2085 | U3 | H_sapiens | Animals | mono_intergenic | C/D | | | intergenic |
| snoDB2086 | snoU13 | H_sapiens | Animals | mono_intergenic | C/D | | | intergenic |
| snoDB2087 | U3 | H_sapiens | Animals | mono_intergenic | C/D | | | intergenic |
| snoDB2088 | U3 | H_sapiens | Animals | mono_intergenic | C/D | | | intergenic |
| snoDB2089 | snoU13 | H_sapiens | Animals | mono_intergenic | C/D | | | intergenic |
| snoDB2090 | snoU13 | H_sapiens | Animals | mono_intergenic | C/D | | | intergenic |
| snoDB2091 | snoU13 | H_sapiens | Animals | mono_intergenic | C/D | | | intergenic |
| snoDB2092 | snoU13 | H_sapiens | Animals | mono_intronic | C/D | ENSG00000176406 | RIMS2 | protein_coding |
| snoDB2093 | U3 | H_sapiens | Animals | mono_intergenic | C/D | | | intergenic |
| snoDB2094 | U3 | H_sapiens | Animals | mono_intergenic | C/D | | | intergenic |
| snoDB2095 | SNODB2065 | H_sapiens | Animals | mono_intronic | H/ACA | ENSG00000146416 | AIG1 | protein_coding |
| snoDB2096 | SNODB2071 | H_sapiens | Animals | mono_intronic | H/ACA | ENSG00000136720 | HS6ST1 | protein_coding |
| snoDB2097 | SNODB2072 | H_sapiens | Animals | mono_intronic | H/ACA | ENSG00000114446 | IFT57 | protein_coding |
| snoDB2098 | SNODB2073 | H_sapiens | Animals | mono_intronic | C/D | ENSG00000164136 | IL15 | protein_coding |
| snoDB2099 | SNODB2074 | H_sapiens | Animals | mono_intronic | H/ACA | ENSG00000278811 | LINC00624 | non_coding |
| snoDB2100 | SNODB2075 | H_sapiens | Animals | mono_intronic | H/ACA | ENSG00000136141 | LRCH1 | protein_coding |
| snoDB2101 | SNODB2076 | H_sapiens | Animals | mono_intronic | H/ACA | ENSG00000141905 | NFIC | protein_coding |
| snoDB2102 | SNODB2077 | H_sapiens | Animals | mono_intronic | H/ACA | ENSG00000132849 | PATJ | protein_coding |
| snoDB2103 | SNODB2083 | H_sapiens | Animals | mono_intronic | C/D | ENSG00000167881 | SRP68 | protein_coding |
| snoDB2104 | SNODB2084 | H_sapiens | Animals | mono_intronic | C/D | ENSG00000104980 | TIMM44 | protein_coding |
| snoDB2105 | SNODB2087 | H_sapiens | Animals | mono_intronic | H/ACA | ENSG00000095787 | WAC | protein_coding |
| snoDB2106 | SNORD3@L5 | H_sapiens | Animals | mono_intronic | C/D | ENSG00000152661 | GJA1 | protein_coding |
| snoDB2107 | U3 | H_sapiens | Animals | mono_intergenic | C/D | | | intergenic |
| snoDB2108 | SNORD3@L38 | H_sapiens | Animals | mono_intergenic | C/D | | | intergenic |
| snoDB2109 | SNORD3@L11 | H_sapiens | Animals | mono_intergenic | C/D | | | intergenic |
| snoDB2110 | SNORD3@L36 | H_sapiens | Animals | mono_intergenic | C/D | | | intergenic |
| snoDB2111 | SNORD3@L47 | H_sapiens | Animals | mono_intronic | C/D | ENSG00000124496 | TRERF1 | protein_coding |
| snoDB2112 | snoDB2112 | H_sapiens | Animals | intronic_cluster | Unknown | ENSG00000180198 | RCC1 | protein_coding |
| snoDB2113 | snoDB2113 | H_sapiens | Animals | mono_intronic | C/D | ENSG00000120549 | KIAA1217 | protein_coding |
| snoDB2114 | snoDB2114 | H_sapiens | Animals | mono_intronic | C/D | ENSG00000111727 | HCFC2 | protein_coding |
| snoDB2115 | snoDB2115 | H_sapiens | Animals | mono_exonic | H/ACA | ENSG00000137801 | THBS1 | protein_coding |
| snoDB2116 | snoDB2116 | H_sapiens | Animals | mono_intronic | C/D | ENSG00000038382 | TRIO | protein_coding |
| snoDB2117 | snoDB2117 | H_sapiens | Animals | mono_intronic | H/ACA | ENSG00000228716 | DHFR | protein_coding |
| snoDB2118 | snoDB2118 | H_sapiens | Animals | mono_intronic | H/ACA | ENSG00000132570 | PCBD2 | protein_coding |
| snoDB2119 | snoDB2119 | H_sapiens | Animals | mono_intergenic | H/ACA | | | intergenic |
| snoDB2120 | snoDB2120 | H_sapiens | Animals | mono_intergenic | C/D | | | intergenic |
| snoDB2121 | snoDB2121 | H_sapiens | Animals | mono_intergenic | Unknown | | | intergenic |

| | | | | | | | | |
|---|---|---|---|---|---|---|---|---|
| snoDB2122 | snoDB2122 | H_sapiens | Animals | mono_intergenic | H/ACA | | | intergenic |
| snoDB2123 | snoDB2123 | H_sapiens | Animals | mono_intergenic | C/D | | | intergenic |
| ENSMMUG00000023820 | SNORA72 | M_mulatta | Animals | mono_intergenic | H/ACA | | | intergenic |
| ENSMMUG00000023821 | ENSMMUG00000023821 | M_mulatta | Animals | intergenic_cluster | C/D | | | intergenic |
| ENSMMUG00000023828 | SNORD82 | M_mulatta | Animals | mono_intronic | C/D | ENSMMUG00000018094 | NCL | protein_coding |
| ENSMMUG00000023833 | SNORD43 | M_mulatta | Animals | mono_intronic | C/D | ENSMMUG00000010029 | RPL3 | protein_coding |
| ENSMMUG00000023834 | U3 | M_mulatta | Animals | mono_intronic | C/D | ENSMMUG00000000768 | PTPRM | protein_coding |
| ENSMMUG00000023839 | ENSMMUG00000023839 | M_mulatta | Animals | mono_intergenic | H/ACA | | | intergenic |
| ENSMMUG00000023878 | SNORA37 | M_mulatta | Animals | mono_intronic | H/ACA | ENSMMUG00000003136 | MBD2 | protein_coding |
| ENSMMUG00000023887 | SNORA71 | M_mulatta | Animals | mono_intergenic | H/ACA | | | intergenic |
| ENSMMUG00000023891 | ENSMMUG00000023891 | M_mulatta | Animals | mono_intergenic | H/ACA | | | intergenic |
| ENSMMUG00000023904 | ENSMMUG00000023904 | M_mulatta | Animals | mono_intergenic | H/ACA | | | intergenic |
| ENSMMUG00000023907 | SNORD87 | M_mulatta | Animals | mono_intronic | C/D | ENSMMUG00000059017 | | non_coding |
| ENSMMUG00000023909 | SNORA70 | M_mulatta | Animals | mono_intergenic | H/ACA | | | intergenic |
| ENSMMUG00000023917 | SNORA67 | M_mulatta | Animals | mono_intronic | H/ACA | ENSMMUG00000013499 | EIF4A1 | protein_coding |
| ENSMMUG00000023922 | SNORA54 | M_mulatta | Animals | mono_intronic | H/ACA | ENSMMUG00000007753 | NAP1L4 | protein_coding |
| ENSMMUG00000023926 | SNORD114-15 | M_mulatta | Animals | intronic_cluster | C/D | ENSMMUG00000063833 | | non_coding |
| ENSMMUG00000023932 | SNORD35A | M_mulatta | Animals | mono_intronic | C/D | ENSMMUG00000007551 | RPL13A | protein_coding |
| ENSMMUG00000023939 | SNORA17B | M_mulatta | Animals | mono_intergenic | H/ACA | | | intergenic |
| ENSMMUG00000023942 | SNORD14 | M_mulatta | Animals | mono_intronic | C/D | ENSMMUG00000004696 | HSPA8 | protein_coding |
| ENSMMUG00000023953 | SNORA70 | M_mulatta | Animals | mono_intronic | H/ACA | ENSMMUG00000005494 | RPL10 | protein_coding |
| ENSMMUG00000023961 | ENSMMUG00000023961 | M_mulatta | Animals | mono_intergenic | H/ACA | | | intergenic |
| ENSMMUG00000023963 | U3 | M_mulatta | Animals | mono_intergenic | C/D | | | intergenic |
| ENSMMUG00000023988 | U8 | M_mulatta | Animals | mono_intergenic | C/D | | | intergenic |
| ENSMMUG00000023996 | SNORD20 | M_mulatta | Animals | mono_intronic | C/D | ENSMMUG00000018094 | NCL | protein_coding |
| ENSMMUG00000024003 | SNORD33 | M_mulatta | Animals | mono_intronic | C/D | ENSMMUG00000007551 | RPL13A | protein_coding |
| ENSMMUG00000024018 | ENSMMUG00000024018 | M_mulatta | Animals | mono_intergenic | H/ACA | | | intergenic |
| ENSMMUG00000024022 | ENSMMUG00000024022 | M_mulatta | Animals | mono_intergenic | H/ACA | | | intergenic |
| ENSMMUG00000024041 | ENSMMUG00000024041 | M_mulatta | Animals | mono_intergenic | H/ACA | | | intergenic |
| ENSMMUG00000024056 | SNORD52 | M_mulatta | Animals | intergenic_cluster | C/D | | | intergenic |
| ENSMMUG00000024064 | U3 | M_mulatta | Animals | mono_intergenic | C/D | | | intergenic |
| ENSMMUG00000024072 | ENSMMUG00000024072 | M_mulatta | Animals | intergenic_cluster | H/ACA | | | intergenic |
| ENSMMUG00000024091 | ENSMMUG00000024091 | M_mulatta | Animals | mono_intergenic | H/ACA | | | intergenic |
| ENSMMUG00000024096 | SNORA64 | M_mulatta | Animals | mono_intronic | H/ACA | ENSMMUG00000029267 | RPS2 | protein_coding |
| ENSMMUG00000024102 | SNORA16A | M_mulatta | Animals | intergenic_cluster | H/ACA | | | intergenic |
| ENSMMUG00000024125 | SNORA58 | M_mulatta | Animals | mono_intronic | H/ACA | ENSMMUG00000000220 | MRPL3 | protein_coding |
| ENSMMUG00000024127 | U3 | M_mulatta | Animals | mono_intergenic | C/D | | | intergenic |
| ENSMMUG00000024139 | SNORA63 | M_mulatta | Animals | mono_intronic | H/ACA | ENSMMUG00000020432 | OSCP1 | protein_coding |
| ENSMMUG00000024169 | SNORA56 | M_mulatta | Animals | mono_intronic | H/ACA | ENSMMUG00000005768 | DKC1 | protein_coding |
| ENSMMUG00000024201 | SNORD115 | M_mulatta | Animals | mono_intergenic | C/D | | | intergenic |
| ENSMMUG00000024242 | SNORD79 | M_mulatta | Animals | mono_intronic | C/D | ENSMMUG00000060229 | | non_coding |
| ENSMMUG00000024245 | SNORA1 | M_mulatta | Animals | intergenic_cluster | H/ACA | | | intergenic |
| ENSMMUG00000024262 | ENSMMUG00000024262 | M_mulatta | Animals | mono_intronic | H/ACA | ENSMMUG00000028844 | | protein_coding |
| ENSMMUG00000024280 | ENSMMUG00000024280 | M_mulatta | Animals | mono_exonic | C/D | ENSMMUG00000053357 | | non_coding |
| ENSMMUG00000024301 | SNORA63 | M_mulatta | Animals | mono_intronic | H/ACA | ENSMMUG00000003702 | EIF4A2 | protein_coding |

| | | | | | | | | |
|---|---|---|---|---|---|---|---|---|
| ENSMMUG00000024302 | SNORD114-7 | M_mulatta | Animals | mono_intergenic | C/D | | | intergenic |
| ENSMMUG00000024314 | SNORA73 | M_mulatta | Animals | mono_intronic | H/ACA | ENSMMUG00000018338 | RCC1 | protein_coding |
| ENSMMUG00000024323 | SNORD33 | M_mulatta | Animals | mono_intronic | C/D | ENSMMUG00000007551 | RPL13A | protein_coding |
| ENSMMUG00000024346 | ENSMMUG00000024346 | M_mulatta | Animals | mono_intergenic | H/ACA | | | intergenic |
| ENSMMUG00000024350 | U3 | M_mulatta | Animals | mono_intergenic | C/D | | | intergenic |
| ENSMMUG00000024353 | SNORD18 | M_mulatta | Animals | mono_intronic | C/D | ENSMMUG00000016844 | RPL4 | protein_coding |
| ENSMMUG00000024365 | SNORD56 | M_mulatta | Animals | mono_intronic | C/D | ENSMMUG00000013888 | NOP56 | protein_coding |
| ENSMMUG00000024369 | SNORA30B | M_mulatta | Animals | mono_intronic | H/ACA | ENSMMUG00000019070 | FOCAD | protein_coding |
| ENSMMUG00000024387 | ENSMMUG00000024387 | M_mulatta | Animals | mono_intergenic | C/D | | | intergenic |
| ENSMMUG00000024400 | ENSMMUG00000024400 | M_mulatta | Animals | mono_intronic | H/ACA | ENSMMUG00000060933 | | protein_coding |
| ENSMMUG00000024421 | SNORA61 | M_mulatta | Animals | intergenic_cluster | H/ACA | | | intergenic |
| ENSMMUG00000024424 | SNORD63 | M_mulatta | Animals | mono_intronic | C/D | ENSMMUG00000005875 | HSPA9 | protein_coding |
| ENSMMUG00000024433 | SNORA70 | M_mulatta | Animals | mono_intergenic | H/ACA | | | intergenic |
| ENSMMUG00000024448 | ENSMMUG00000024448 | M_mulatta | Animals | mono_intergenic | H/ACA | | | intergenic |
| ENSMMUG00000024450 | SNORA74 | M_mulatta | Animals | mono_intergenic | H/ACA | | | intergenic |
| ENSMMUG00000024460 | SNORA68B | M_mulatta | Animals | mono_intronic | H/ACA | ENSMMUG00000008041 | ANKRD27 | protein_coding |
| ENSMMUG00000024461 | ENSMMUG00000024461 | M_mulatta | Animals | mono_intergenic | H/ACA | | | intergenic |
| ENSMMUG00000024470 | ENSMMUG00000024470 | M_mulatta | Animals | intergenic_cluster | H/ACA | | | intergenic |
| ENSMMUG00000024487 | SNORD22 | M_mulatta | Animals | mono_intronic | C/D | ENSMMUG00000064232 | | protein_coding |
| ENSMMUG00000024499 | SNORD31 | M_mulatta | Animals | mono_intronic | C/D | ENSMMUG00000064232 | | protein_coding |
| ENSMMUG00000024515 | U8 | M_mulatta | Animals | mono_intergenic | C/D | | | intergenic |
| ENSMMUG00000024535 | ENSMMUG00000024535 | M_mulatta | Animals | mono_intronic | C/D | ENSMMUG00000013520 | PUM1 | protein_coding |
| ENSMMUG00000024536 | SNORD83 | M_mulatta | Animals | mono_intronic | C/D | ENSMMUG00000008839 | DDX39B | protein_coding |
| ENSMMUG00000024537 | ENSMMUG00000024537 | M_mulatta | Animals | mono_intergenic | H/ACA | | | intergenic |
| ENSMMUG00000024552 | SNORA65 | M_mulatta | Animals | mono_intronic | H/ACA | ENSMMUG00000000422 | RPL12 | protein_coding |
| ENSMMUG00000024558 | SNORD114-21 | M_mulatta | Animals | mono_intronic | C/D | ENSMMUG00000063833 | | non_coding |
| ENSMMUG00000024570 | SNORA70 | M_mulatta | Animals | mono_intergenic | H/ACA | | | intergenic |
| ENSMMUG00000024632 | U3 | M_mulatta | Animals | mono_intergenic | C/D | | | intergenic |
| ENSMMUG00000024641 | ENSMMUG00000024641 | M_mulatta | Animals | mono_intergenic | C/D | | | intergenic |
| ENSMMUG00000024646 | ENSMMUG00000024646 | M_mulatta | Animals | intergenic_cluster | H/ACA | | | intergenic |
| ENSMMUG00000024647 | SNORD1C | M_mulatta | Animals | mono_intergenic | C/D | | | intergenic |
| ENSMMUG00000024665 | ENSMMUG00000024665 | M_mulatta | Animals | intergenic_cluster | H/ACA | | | intergenic |
| ENSMMUG00000024671 | U8 | M_mulatta | Animals | mono_intergenic | C/D | | | intergenic |
| ENSMMUG00000024688 | ENSMMUG00000024688 | M_mulatta | Animals | mono_intronic | C/D | ENSMMUG00000001027 | ATP5F1B | protein_coding |
| ENSMMUG00000024701 | ENSMMUG00000024701 | M_mulatta | Animals | mono_intergenic | H/ACA | | | intergenic |
| ENSMMUG00000024706 | SNORA62 | M_mulatta | Animals | mono_intronic | H/ACA | ENSMMUG00000022965 | RPSA | protein_coding |
| ENSMMUG00000024707 | SNORA72 | M_mulatta | Animals | mono_intergenic | H/ACA | | | intergenic |
| ENSMMUG00000024720 | U8 | M_mulatta | Animals | mono_intergenic | C/D | | | intergenic |
| ENSMMUG00000024733 | SNORD115 | M_mulatta | Animals | mono_intergenic | C/D | | | intergenic |
| ENSMMUG00000024743 | SNORD24 | M_mulatta | Animals | mono_intronic | C/D | ENSMMUG00000022341 | RPL7A | protein_coding |
| ENSMMUG00000024747 | SNORD36 | M_mulatta | Animals | mono_intronic | C/D | ENSMMUG00000022341 | RPL7A | protein_coding |
| ENSMMUG00000024750 | U3 | M_mulatta | Animals | mono_intergenic | C/D | | | intergenic |
| ENSMMUG00000024754 | ENSMMUG00000024754 | M_mulatta | Animals | mono_intergenic | H/ACA | | | intergenic |
| ENSMMUG00000024764 | SNORA72 | M_mulatta | Animals | mono_intronic | H/ACA | ENSMMUG00000020073 | RPL30 | protein_coding |
| ENSMMUG00000024783 | SNORA30 | M_mulatta | Animals | mono_intronic | H/ACA | ENSMMUG00000010556 | SRCAP | protein_coding |

| | | | | | | | | |
|---|---|---|---|---|---|---|---|---|
| ENSMMUG00000024785 | SNORA73 | M_mulatta | Animals | mono_intronic | H/ACA | ENSMMUG00000055822 | MIB1 | protein_coding |
| ENSMMUG00000024803 | ENSMMUG00000024803 | M_mulatta | Animals | mono_intergenic | H/ACA | | | intergenic |
| ENSMMUG00000024805 | ENSMMUG00000024805 | M_mulatta | Animals | mono_intergenic | H/ACA | | | intergenic |
| ENSMMUG00000024815 | SNORA5A | M_mulatta | Animals | mono_intronic | H/ACA | ENSMMUG00000010823 | TBRG4 | protein_coding |
| ENSMMUG00000024826 | SNORD9 | M_mulatta | Animals | mono_intronic | C/D | ENSMMUG00000015002 | CHD8 | protein_coding |
| ENSMMUG00000024834 | SNORA70 | M_mulatta | Animals | mono_intergenic | H/ACA | | | intergenic |
| ENSMMUG00000024837 | SNORA19 | M_mulatta | Animals | mono_intronic | H/ACA | ENSMMUG00000008994 | EIF3A | protein_coding |
| ENSMMUG00000024846 | SNORD81 | M_mulatta | Animals | mono_intronic | C/D | ENSMMUG00000060229 | | non_coding |
| ENSMMUG00000024847 | SNORA33 | M_mulatta | Animals | mono_intronic | H/ACA | ENSMMUG00000013237 | RPS12 | protein_coding |
| ENSMMUG00000024851 | SNORD58 | M_mulatta | Animals | mono_intronic | C/D | ENSMMUG00000018837 | RPL17 | protein_coding |
| ENSMMUG00000024869 | SNORD68 | M_mulatta | Animals | mono_intronic | C/D | ENSMMUG00000017928 | RPL13 | protein_coding |
| ENSMMUG00000024873 | SNORD8 | M_mulatta | Animals | mono_intronic | C/D | ENSMMUG00000015002 | CHD8 | protein_coding |
| ENSMMUG00000024882 | SNORD42 | M_mulatta | Animals | mono_intronic | C/D | ENSMMUG00000015777 | HNRNPDL | protein_coding |
| ENSMMUG00000024896 | SNORA69 | M_mulatta | Animals | mono_intronic | H/ACA | ENSMMUG00000018391 | RPL39 | protein_coding |
| ENSMMUG00000024902 | SNORD101 | M_mulatta | Animals | mono_intronic | C/D | ENSMMUG00000013237 | RPS12 | protein_coding |
| ENSMMUG00000024923 | SNORD48 | M_mulatta | Animals | mono_intergenic | C/D | | | intergenic |
| ENSMMUG00000024944 | SNORA41B | M_mulatta | Animals | mono_intronic | H/ACA | ENSMMUG00000062254 | | non_coding |
| ENSMMUG00000024955 | ENSMMUG00000024955 | M_mulatta | Animals | mono_intergenic | H/ACA | | | intergenic |
| ENSMMUG00000024975 | SNORA2B | M_mulatta | Animals | mono_intronic | H/ACA | ENSMMUG00000003877 | KANSL2 | protein_coding |
| ENSMMUG00000024976 | ENSMMUG00000024976 | M_mulatta | Animals | mono_intronic | H/ACA | ENSMMUG00000046328 | CSKMT | protein_coding |
| ENSMMUG00000024995 | SNORA20B | M_mulatta | Animals | mono_intergenic | H/ACA | | | intergenic |
| ENSMMUG00000024996 | ENSMMUG00000024996 | M_mulatta | Animals | mono_intergenic | H/ACA | | | intergenic |
| ENSMMUG00000025017 | U3 | M_mulatta | Animals | mono_intergenic | C/D | | | intergenic |
| ENSMMUG00000025038 | ENSMMUG00000025038 | M_mulatta | Animals | mono_intergenic | H/ACA | | | intergenic |
| ENSMMUG00000025039 | SNORA73 | M_mulatta | Animals | mono_intergenic | H/ACA | | | intergenic |
| ENSMMUG00000025057 | SNORD14 | M_mulatta | Animals | mono_intronic | C/D | ENSMMUG00000022547 | RPS13 | protein_coding |
| ENSMMUG00000025058 | ENSMMUG00000025058 | M_mulatta | Animals | mono_intergenic | H/ACA | | | intergenic |
| ENSMMUG00000025102 | SNORD104 | M_mulatta | Animals | intergenic_cluster | C/D | | | intergenic |
| ENSMMUG00000025110 | ENSMMUG00000025110 | M_mulatta | Animals | mono_intronic | H/ACA | ENSMMUG00000008237 | PGS1 | protein_coding |
| ENSMMUG00000025111 | SNORD36 | M_mulatta | Animals | mono_intronic | C/D | ENSMMUG00000022341 | RPL7A | protein_coding |
| ENSMMUG00000025116 | ENSMMUG00000025116 | M_mulatta | Animals | mono_intergenic | H/ACA | | | intergenic |
| ENSMMUG00000025132 | SNORA14B | M_mulatta | Animals | mono_intronic | H/ACA | ENSMMUG00000058824 | TOMM20 | protein_coding |
| ENSMMUG00000025136 | SNORA36C | M_mulatta | Animals | mono_intronic | H/ACA | ENSMMUG00000007868 | AAK1 | protein_coding |
| ENSMMUG00000025148 | SNORA55 | M_mulatta | Animals | mono_intronic | H/ACA | ENSMMUG00000007497 | PABPC4 | protein_coding |
| ENSMMUG00000025180 | ENSMMUG00000025180 | M_mulatta | Animals | mono_intergenic | H/ACA | | | intergenic |
| ENSMMUG00000025184 | SNORA22B | M_mulatta | Animals | mono_intronic | H/ACA | ENSMMUG00000015272 | CCT6A | protein_coding |
| ENSMMUG00000025201 | SNORD58 | M_mulatta | Animals | mono_intronic | C/D | ENSMMUG00000018837 | RPL17 | protein_coding |
| ENSMMUG00000025207 | SNORA63 | M_mulatta | Animals | mono_intergenic | H/ACA | | | intergenic |
| ENSMMUG00000025228 | SNORD45A | M_mulatta | Animals | mono_intronic | C/D | ENSMMUG00000000873 | RABGGTB | protein_coding |
| ENSMMUG00000025233 | SNORA23 | M_mulatta | Animals | mono_intronic | H/ACA | ENSMMUG00000023636 | IPO7 | protein_coding |
| ENSMMUG00000025257 | SNORA46 | M_mulatta | Animals | mono_intronic | H/ACA | ENSMMUG00000014625 | CNOT1 | protein_coding |
| ENSMMUG00000025267 | ENSMMUG00000025267 | M_mulatta | Animals | mono_intergenic | H/ACA | | | intergenic |
| ENSMMUG00000025279 | SNORA14A | M_mulatta | Animals | mono_intronic | H/ACA | ENSMMUG00000020086 | POR | protein_coding |
| ENSMMUG00000025294 | SNORA72 | M_mulatta | Animals | mono_intergenic | H/ACA | | | intergenic |
| ENSMMUG00000025304 | ENSMMUG00000025304 | M_mulatta | Animals | mono_intergenic | H/ACA | | | intergenic |

| | | | | | | | | |
|---|---|---|---|---|---|---|---|---|
| ENSMMUG00000025305 | ENSMMUG00000025305 | M_mulatta | Animals | mono_intergenic | H/ACA | | | intergenic |
| ENSMMUG00000025308 | ENSMMUG00000025308 | M_mulatta | Animals | mono_intronic | H/ACA | ENSMMUG00000057828 | FGF1 | protein_coding |
| ENSMMUG00000025340 | SNORD116 | M_mulatta | Animals | mono_intronic | C/D | ENSMMUG00000064025 | | non_coding |
| ENSMMUG00000025344 | SNORA29 | M_mulatta | Animals | mono_intronic | H/ACA | ENSMMUG00000012846 | TCP1 | protein_coding |
| ENSMMUG00000025346 | ENSMMUG00000025346 | M_mulatta | Animals | mono_intronic | C/D | ENSMMUG00000015540 | BTBD11 | protein_coding |
| ENSMMUG00000025348 | SNORA71 | M_mulatta | Animals | mono_intronic | H/ACA | ENSMMUG00000017350 | RALGAPB | protein_coding |
| ENSMMUG00000025353 | ENSMMUG00000025353 | M_mulatta | Animals | mono_intergenic | H/ACA | | | intergenic |
| ENSMMUG00000025358 | ENSMMUG00000025358 | M_mulatta | Animals | mono_intergenic | H/ACA | | | intergenic |
| ENSMMUG00000025366 | ENSMMUG00000025366 | M_mulatta | Animals | mono_intergenic | H/ACA | | | intergenic |
| ENSMMUG00000025382 | U8 | M_mulatta | Animals | mono_intergenic | C/D | | | intergenic |
| ENSMMUG00000025398 | SNORD28 | M_mulatta | Animals | mono_intronic | C/D | ENSMMUG00000064232 | | protein_coding |
| ENSMMUG00000025407 | ENSMMUG00000025407 | M_mulatta | Animals | mono_intronic | C/D | ENSMMUG00000019239 | TBC1D19 | protein_coding |
| ENSMMUG00000025415 | U3 | M_mulatta | Animals | mono_intergenic | C/D | | | intergenic |
| ENSMMUG00000025417 | SNORA36A | M_mulatta | Animals | mono_intronic | H/ACA | ENSMMUG00000005768 | DKC1 | protein_coding |
| ENSMMUG00000025419 | ENSMMUG00000025419 | M_mulatta | Animals | mono_intronic | C/D | ENSMMUG00000040409 | COX7C | protein_coding |
| ENSMMUG00000025423 | SNORA62 | M_mulatta | Animals | mono_intergenic | H/ACA | | | intergenic |
| ENSMMUG00000025424 | ENSMMUG00000025424 | M_mulatta | Animals | intronic_cluster | C/D | ENSMMUG00000063833 | | non_coding |
| ENSMMUG00000025469 | ENSMMUG00000025469 | M_mulatta | Animals | mono_intergenic | H/ACA | | | intergenic |
| ENSMMUG00000025470 | U3 | M_mulatta | Animals | mono_intronic | C/D | ENSMMUG00000048865 | MALRD1 | protein_coding |
| ENSMMUG00000025472 | SNORA41 | M_mulatta | Animals | mono_intronic | H/ACA | ENSMMUG00000001054 | EEF1B2 | protein_coding |
| ENSMMUG00000025481 | ENSMMUG00000025481 | M_mulatta | Animals | mono_intronic | C/D | ENSMMUG00000053357 | | non_coding |
| ENSMMUG00000025503 | ENSMMUG00000025503 | M_mulatta | Animals | mono_intronic | C/D | ENSMMUG00000007549 | RPS11 | protein_coding |
| ENSMMUG00000025526 | SNORD114-27 | M_mulatta | Animals | mono_intronic | C/D | ENSMMUG00000063833 | | non_coding |
| ENSMMUG00000025533 | ENSMMUG00000025533 | M_mulatta | Animals | mono_intronic | C/D | ENSMMUG00000013520 | PUM1 | protein_coding |
| ENSMMUG00000025554 | ENSMMUG00000025554 | M_mulatta | Animals | mono_intronic | C/D | ENSMMUG00000013520 | PUM1 | protein_coding |
| ENSMMUG00000025556 | ENSMMUG00000025556 | M_mulatta | Animals | mono_intergenic | H/ACA | | | intergenic |
| ENSMMUG00000025560 | SNORD37 | M_mulatta | Animals | mono_intronic | C/D | ENSMMUG00000022767 | EEF2 | protein_coding |
| ENSMMUG00000025577 | SNORA50A | M_mulatta | Animals | intronic_cluster | H/ACA | ENSMMUG00000014625 | CNOT1 | protein_coding |
| ENSMMUG00000025585 | ENSMMUG00000025585 | M_mulatta | Animals | mono_intergenic | C/D | | | intergenic |
| ENSMMUG00000025590 | SNORA18 | M_mulatta | Animals | intronic_cluster | H/ACA | ENSMMUG00000011646 | TAF1D | protein_coding |
| ENSMMUG00000025599 | ENSMMUG00000025599 | M_mulatta | Animals | mono_intergenic | H/ACA | | | intergenic |
| ENSMMUG00000025608 | SNORD114-16 | M_mulatta | Animals | intronic_cluster | C/D | ENSMMUG00000063833 | | non_coding |
| ENSMMUG00000025615 | ENSMMUG00000025615 | M_mulatta | Animals | mono_intergenic | H/ACA | | | intergenic |
| ENSMMUG00000025616 | ENSMMUG00000025616 | M_mulatta | Animals | mono_intergenic | H/ACA | | | intergenic |
| ENSMMUG00000025617 | SNORA71 | M_mulatta | Animals | mono_intergenic | H/ACA | | | intergenic |
| ENSMMUG00000025627 | SNORA25B | M_mulatta | Animals | mono_intergenic | H/ACA | | | intergenic |
| ENSMMUG00000025635 | ENSMMUG00000025635 | M_mulatta | Animals | mono_intergenic | C/D | | | intergenic |
| ENSMMUG00000025650 | ENSMMUG00000025650 | M_mulatta | Animals | mono_intergenic | H/ACA | | | intergenic |
| ENSMMUG00000025672 | SNORD49A | M_mulatta | Animals | mono_intronic | C/D | ENSMMUG00000057308 | | protein_coding |
| ENSMMUG00000025693 | SNORD113-8 | M_mulatta | Animals | mono_intronic | C/D | ENSMMUG00000053357 | | non_coding |
| ENSMMUG00000025705 | ENSMMUG00000025705 | M_mulatta | Animals | mono_intergenic | H/ACA | | | intergenic |
| ENSMMUG00000025711 | ENSMMUG00000025711 | M_mulatta | Animals | mono_intergenic | H/ACA | | | intergenic |
| ENSMMUG00000025719 | SNORD29 | M_mulatta | Animals | mono_intronic | C/D | ENSMMUG00000064232 | | protein_coding |
| ENSMMUG00000025722 | ENSMMUG00000025722 | M_mulatta | Animals | mono_intergenic | H/ACA | | | intergenic |
| ENSMMUG00000025749 | SNORA25 | M_mulatta | Animals | intergenic_cluster | H/ACA | | | intergenic |

| | | | | | | | | |
|---|---|---|---|---|---|---|---|---|
| ENSMMUG00000025750 | SNORA73 | M_mulatta | Animals | mono_intronic | H/ACA | ENSMMUG00000018338 | RCC1 | protein_coding |
| ENSMMUG00000025758 | ENSMMUG00000025758 | M_mulatta | Animals | mono_intergenic | C/D | | | intergenic |
| ENSMMUG00000025788 | ENSMMUG00000025788 | M_mulatta | Animals | mono_intergenic | H/ACA | | | intergenic |
| ENSMMUG00000025793 | SNORD114-5 | M_mulatta | Animals | mono_intronic | C/D | ENSMMUG00000053357 | | non_coding |
| ENSMMUG00000025802 | SNORD45C | M_mulatta | Animals | mono_intronic | C/D | ENSMMUG00000000873 | RABGGTB | protein_coding |
| ENSMMUG00000025809 | SNORA3A | M_mulatta | Animals | mono_intronic | H/ACA | ENSMMUG00000021828 | RPL27A | protein_coding |
| ENSMMUG00000025822 | U8 | M_mulatta | Animals | mono_intergenic | C/D | | | intergenic |
| ENSMMUG00000025827 | ENSMMUG00000025827 | M_mulatta | Animals | mono_intergenic | H/ACA | | | intergenic |
| ENSMMUG00000025830 | ENSMMUG00000025830 | M_mulatta | Animals | mono_intergenic | H/ACA | | | intergenic |
| ENSMMUG00000025832 | ENSMMUG00000025832 | M_mulatta | Animals | mono_intronic | C/D | ENSMMUG00000060229 | | non_coding |
| ENSMMUG00000025856 | ENSMMUG00000025856 | M_mulatta | Animals | mono_intronic | H/ACA | ENSMMUG00000013730 | WDR17 | protein_coding |
| ENSMMUG00000025857 | SNORA13 | M_mulatta | Animals | mono_intergenic | H/ACA | | | intergenic |
| ENSMMUG00000025863 | ENSMMUG00000025863 | M_mulatta | Animals | mono_intronic | H/ACA | ENSMMUG00000002667 | N4BP2 | protein_coding |
| ENSMMUG00000025888 | ENSMMUG00000025888 | M_mulatta | Animals | mono_intergenic | H/ACA | | | intergenic |
| ENSMMUG00000025901 | ENSMMUG00000025901 | M_mulatta | Animals | mono_intergenic | H/ACA | | | intergenic |
| ENSMMUG00000025903 | ENSMMUG00000025903 | M_mulatta | Animals | mono_intergenic | C/D | | | intergenic |
| ENSMMUG00000025913 | ENSMMUG00000025913 | M_mulatta | Animals | mono_intergenic | H/ACA | | | intergenic |
| ENSMMUG00000025918 | SNORA38 | M_mulatta | Animals | mono_intronic | H/ACA | ENSMMUG00000005297 | PRRC2A | protein_coding |
| ENSMMUG00000025942 | U3 | M_mulatta | Animals | mono_intergenic | C/D | | | intergenic |
| ENSMMUG00000025943 | SNORA20 | M_mulatta | Animals | mono_intronic | H/ACA | ENSMMUG00000012846 | TCP1 | protein_coding |
| ENSMMUG00000025979 | U8 | M_mulatta | Animals | mono_intronic | C/D | ENSMMUG00000001074 | ULK4 | protein_coding |
| ENSMMUG00000025983 | U8 | M_mulatta | Animals | mono_intronic | C/D | ENSMMUG00000057647 | | non_coding |
| ENSMMUG00000025990 | ENSMMUG00000025990 | M_mulatta | Animals | mono_intergenic | H/ACA | | | intergenic |
| ENSMMUG00000025991 | ENSMMUG00000025991 | M_mulatta | Animals | mono_intergenic | H/ACA | | | intergenic |
| ENSMMUG00000025998 | SNORD113-7 | M_mulatta | Animals | mono_intronic | C/D | ENSMMUG00000053357 | | non_coding |
| ENSMMUG00000026025 | SNORA21 | M_mulatta | Animals | mono_intronic | H/ACA | ENSMMUG00000062400 | RPL23 | protein_coding |
| ENSMMUG00000026037 | SNORD16 | M_mulatta | Animals | mono_intronic | C/D | ENSMMUG00000016844 | RPL4 | protein_coding |
| ENSMMUG00000026039 | SNORD34 | M_mulatta | Animals | mono_intronic | C/D | ENSMMUG00000007551 | RPL13A | protein_coding |
| ENSMMUG00000026044 | U8 | M_mulatta | Animals | mono_intergenic | C/D | | | intergenic |
| ENSMMUG00000026047 | ENSMMUG00000026047 | M_mulatta | Animals | mono_intronic | H/ACA | ENSMMUG00000010078 | SLC2A13 | protein_coding |
| ENSMMUG00000026050 | ENSMMUG00000026050 | M_mulatta | Animals | mono_intergenic | H/ACA | | | intergenic |
| ENSMMUG00000026051 | ENSMMUG00000026051 | M_mulatta | Animals | mono_intergenic | H/ACA | | | intergenic |
| ENSMMUG00000026059 | SNORD4B | M_mulatta | Animals | mono_intronic | C/D | ENSMMUG00000018010 | RPL23A | protein_coding |
| ENSMMUG00000026062 | SNORD46 | M_mulatta | Animals | mono_intronic | C/D | ENSMMUG00000015270 | RPS8 | protein_coding |
| ENSMMUG00000026065 | ENSMMUG00000026065 | M_mulatta | Animals | intronic_cluster | C/D | ENSMMUG00000063833 | | non_coding |
| ENSMMUG00000026081 | SNORD114-25 | M_mulatta | Animals | mono_intronic | C/D | ENSMMUG00000063833 | | non_coding |
| ENSMMUG00000026085 | SNORD58 | M_mulatta | Animals | mono_intronic | C/D | ENSMMUG00000018837 | RPL17 | protein_coding |
| ENSMMUG00000026093 | SNORD18 | M_mulatta | Animals | mono_intronic | C/D | ENSMMUG00000016844 | RPL4 | protein_coding |
| ENSMMUG00000026100 | ENSMMUG00000026100 | M_mulatta | Animals | mono_intergenic | H/ACA | | | intergenic |
| ENSMMUG00000026102 | SNORA5C | M_mulatta | Animals | mono_intronic | H/ACA | ENSMMUG00000010823 | TBRG4 | protein_coding |
| ENSMMUG00000026121 | SNORA8 | M_mulatta | Animals | intergenic_cluster | H/ACA | | | intergenic |
| ENSMMUG00000026127 | SNORD38A | M_mulatta | Animals | mono_intronic | C/D | ENSMMUG00000015270 | RPS8 | protein_coding |
| ENSMMUG00000026130 | SNORD15 | M_mulatta | Animals | mono_intronic | C/D | ENSMMUG00000009019 | RPS3 | protein_coding |
| ENSMMUG00000026131 | ENSMMUG00000026131 | M_mulatta | Animals | mono_intergenic | H/ACA | | | intergenic |
| ENSMMUG00000026139 | ENSMMUG00000026139 | M_mulatta | Animals | mono_intronic | H/ACA | ENSMMUG00000015161 | GINM1 | protein_coding |

| | | | | | | | | |
|---|---|---|---|---|---|---|---|---|
| ENSMMUG00000026141 | ENSMMUG00000026141 | M_mulatta | Animals | mono_intergenic | H/ACA | | | intergenic |
| ENSMMUG00000026143 | SNORA72 | M_mulatta | Animals | mono_intronic | H/ACA | ENSMMUG00000055835 | NUCKS1 | protein_coding |
| ENSMMUG00000026144 | ENSMMUG00000026144 | M_mulatta | Animals | mono_intergenic | H/ACA | | | intergenic |
| ENSMMUG00000026160 | SNORD30 | M_mulatta | Animals | mono_intronic | C/D | ENSMMUG00000064232 | | protein_coding |
| ENSMMUG00000026177 | SNORA63 | M_mulatta | Animals | mono_intronic | H/ACA | ENSMMUG00000003702 | EIF4A2 | protein_coding |
| ENSMMUG00000026185 | SNORA62 | M_mulatta | Animals | mono_intronic | H/ACA | ENSMMUG00000022965 | RPSA | protein_coding |
| ENSMMUG00000026192 | ENSMMUG00000026192 | M_mulatta | Animals | mono_intronic | C/D | ENSMMUG00000001663 | IL1RAPL1 | protein_coding |
| ENSMMUG00000026204 | SNORD42 | M_mulatta | Animals | mono_intronic | C/D | ENSMMUG00000018010 | RPL23A | protein_coding |
| ENSMMUG00000026229 | ENSMMUG00000026229 | M_mulatta | Animals | mono_intronic | C/D | ENSMMUG00000060229 | | non_coding |
| ENSMMUG00000026265 | SNORD27 | M_mulatta | Animals | mono_intronic | C/D | ENSMMUG00000064232 | | protein_coding |
| ENSMMUG00000026295 | SNORD49B | M_mulatta | Animals | mono_intronic | C/D | ENSMMUG00000057308 | | protein_coding |
| ENSMMUG00000026317 | SNORA70 | M_mulatta | Animals | mono_intergenic | H/ACA | | | intergenic |
| ENSMMUG00000026331 | SNORD18 | M_mulatta | Animals | mono_intergenic | C/D | | | intergenic |
| ENSMMUG00000026335 | ENSMMUG00000026335 | M_mulatta | Animals | mono_intergenic | C/D | | | intergenic |
| ENSMMUG00000026405 | SNORD6 | M_mulatta | Animals | intergenic_cluster | C/D | | | intergenic |
| ENSMMUG00000026409 | ENSMMUG00000026409 | M_mulatta | Animals | mono_intergenic | H/ACA | | | intergenic |
| ENSMMUG00000026410 | ENSMMUG00000026410 | M_mulatta | Animals | mono_intronic | H/ACA | ENSMMUG00000000031 | TMEM132C | protein_coding |
| ENSMMUG00000026414 | SNORD113-5 | M_mulatta | Animals | mono_intergenic | C/D | | | intergenic |
| ENSMMUG00000026424 | SNORA16B | M_mulatta | Animals | mono_intronic | H/ACA | ENSMMUG00000019771 | PPP2R5A | protein_coding |
| ENSMMUG00000026432 | SNORD7 | M_mulatta | Animals | mono_intronic | C/D | ENSMMUG00000031736 | | protein_coding |
| ENSMMUG00000026447 | U3 | M_mulatta | Animals | mono_intergenic | C/D | | | intergenic |
| ENSMMUG00000026454 | SNORA51 | M_mulatta | Animals | mono_intronic | H/ACA | ENSMMUG00000013888 | NOP56 | protein_coding |
| ENSMMUG00000026455 | SNORD60 | M_mulatta | Animals | mono_intergenic | C/D | | | intergenic |
| ENSMMUG00000026456 | SNORD95 | M_mulatta | Animals | mono_intronic | C/D | ENSMMUG00000003604 | RACK1 | protein_coding |
| ENSMMUG00000026461 | ENSMMUG00000026461 | M_mulatta | Animals | mono_intergenic | H/ACA | | | intergenic |
| ENSMMUG00000026474 | SNORD83 | M_mulatta | Animals | mono_intronic | C/D | ENSMMUG00000008839 | DDX39B | protein_coding |
| ENSMMUG00000026475 | ENSMMUG00000026475 | M_mulatta | Animals | mono_intergenic | C/D | | | intergenic |
| ENSMMUG00000026493 | SNORD36 | M_mulatta | Animals | mono_intronic | C/D | ENSMMUG00000022341 | RPL7A | protein_coding |
| ENSMMUG00000026502 | ENSMMUG00000026502 | M_mulatta | Animals | mono_intergenic | H/ACA | | | intergenic |
| ENSMMUG00000026507 | ENSMMUG00000026507 | M_mulatta | Animals | mono_intronic | C/D | ENSMMUG00000006898 | ITGB8 | protein_coding |
| ENSMMUG00000026512 | SNORA80B | M_mulatta | Animals | mono_intronic | H/ACA | ENSMMUG00000020441 | ODC1 | protein_coding |
| ENSMMUG00000026515 | ENSMMUG00000026515 | M_mulatta | Animals | mono_intergenic | C/D | | | intergenic |
| ENSMMUG00000026536 | SNORD15 | M_mulatta | Animals | mono_intronic | C/D | ENSMMUG00000009019 | RPS3 | protein_coding |
| ENSMMUG00000026541 | SNORA73 | M_mulatta | Animals | mono_intergenic | H/ACA | | | intergenic |
| ENSMMUG00000026549 | SNORD114-13 | M_mulatta | Animals | mono_intronic | C/D | ENSMMUG00000063833 | | non_coding |
| ENSMMUG00000026551 | ENSMMUG00000026551 | M_mulatta | Animals | mono_intronic | C/D | ENSMMUG00000010032 | EDEM2 | protein_coding |
| ENSMMUG00000026563 | SNORA62 | M_mulatta | Animals | mono_intergenic | H/ACA | | | intergenic |
| ENSMMUG00000026566 | ENSMMUG00000026566 | M_mulatta | Animals | mono_intergenic | C/D | | | intergenic |
| ENSMMUG00000026577 | SNORA5B | M_mulatta | Animals | mono_intronic | H/ACA | ENSMMUG00000010823 | TBRG4 | protein_coding |
| ENSMMUG00000026582 | SNORD116 | M_mulatta | Animals | mono_intronic | C/D | ENSMMUG00000064025 | | non_coding |
| ENSMMUG00000026595 | SNORD31 | M_mulatta | Animals | mono_intronic | C/D | ENSMMUG00000030627 | NALF1 | protein_coding |
| ENSMMUG00000026596 | SNORD14 | M_mulatta | Animals | mono_intronic | C/D | ENSMMUG00000004696 | HSPA8 | protein_coding |
| ENSMMUG00000026606 | ENSMMUG00000026606 | M_mulatta | Animals | mono_intergenic | H/ACA | | | intergenic |
| ENSMMUG00000026614 | SNORD1A | M_mulatta | Animals | mono_intergenic | C/D | | | intergenic |
| ENSMMUG00000026618 | SNORA72 | M_mulatta | Animals | mono_intergenic | H/ACA | | | intergenic |

| | | | | | | | | |
|---|---|---|---|---|---|---|---|---|
| ENSMMUG00000026639 | SNORA27 | M_mulatta | Animals | mono_intronic | H/ACA | ENSMMUG00000058755 | | protein_coding |
| ENSMMUG00000026641 | ENSMMUG00000026641 | M_mulatta | Animals | mono_intergenic | C/D | | | intergenic |
| ENSMMUG00000026648 | SNORA66 | M_mulatta | Animals | mono_intronic | H/ACA | ENSMMUG00000005072 | RPL5 | protein_coding |
| ENSMMUG00000026671 | SNORD18 | M_mulatta | Animals | mono_intergenic | C/D | | | intergenic |
| ENSMMUG00000026673 | SNORD45B | M_mulatta | Animals | mono_intronic | C/D | ENSMMUG00000000873 | RABGGTB | protein_coding |
| ENSMMUG00000026681 | U3 | M_mulatta | Animals | mono_intergenic | C/D | | | intergenic |
| ENSMMUG00000026686 | SNORA31 | M_mulatta | Animals | mono_intronic | H/ACA | ENSMMUG00000022979 | TPT1 | protein_coding |
| ENSMMUG00000026727 | SNORA9B | M_mulatta | Animals | mono_intronic | H/ACA | ENSMMUG00000013178 | DDX55 | protein_coding |
| ENSMMUG00000026730 | ENSMMUG00000026730 | M_mulatta | Animals | mono_intergenic | C/D | | | intergenic |
| ENSMMUG00000026741 | SNORA4 | M_mulatta | Animals | mono_intronic | H/ACA | ENSMMUG00000003702 | EIF4A2 | protein_coding |
| ENSMMUG00000026756 | ENSMMUG00000026756 | M_mulatta | Animals | mono_intergenic | H/ACA | | | intergenic |
| ENSMMUG00000026761 | SNORD115 | M_mulatta | Animals | mono_intergenic | C/D | | | intergenic |
| ENSMMUG00000026768 | ENSMMUG00000026768 | M_mulatta | Animals | mono_intergenic | H/ACA | | | intergenic |
| ENSMMUG00000026786 | ENSMMUG00000026786 | M_mulatta | Animals | mono_intergenic | C/D | | | intergenic |
| ENSMMUG00000026792 | SNORD18 | M_mulatta | Animals | mono_intronic | C/D | ENSMMUG00000016844 | RPL4 | protein_coding |
| ENSMMUG00000026811 | SNORA28 | M_mulatta | Animals | mono_intronic | H/ACA | ENSMMUG00000005630 | EIF5 | protein_coding |
| ENSMMUG00000026829 | SNORA62 | M_mulatta | Animals | mono_intergenic | H/ACA | | | intergenic |
| ENSMMUG00000027352 | SNORA70 | M_mulatta | Animals | mono_intronic | H/ACA | ENSMMUG00000012007 | PHLPP2 | protein_coding |
| ENSMMUG00000027366 | SNORD4A | M_mulatta | Animals | mono_intronic | C/D | ENSMMUG00000018010 | RPL23A | protein_coding |
| ENSMMUG00000027387 | ENSMMUG00000027387 | M_mulatta | Animals | mono_intronic | H/ACA | ENSMMUG00000011639 | BMP2K | protein_coding |
| ENSMMUG00000027410 | U3 | M_mulatta | Animals | intronic_cluster | C/D | ENSMMUG00000046572 | TEX14 | protein_coding |
| ENSMMUG00000027418 | ENSMMUG00000027418 | M_mulatta | Animals | mono_intergenic | H/ACA | | | intergenic |
| ENSMMUG00000027434 | ENSMMUG00000027434 | M_mulatta | Animals | intronic_cluster | C/D | ENSMMUG00000008025 | CEP295 | protein_coding |
| ENSMMUG00000027438 | ENSMMUG00000027438 | M_mulatta | Animals | mono_intronic | C/D | ENSMMUG00000009255 | VPS29 | protein_coding |
| ENSMMUG00000027476 | SNORD115 | M_mulatta | Animals | mono_intergenic | C/D | | | intergenic |
| ENSMMUG00000027541 | SNORA70 | M_mulatta | Animals | mono_intergenic | H/ACA | | | intergenic |
| ENSMMUG00000027582 | SNORA38B | M_mulatta | Animals | mono_intronic | H/ACA | ENSMMUG00000002414 | NOL11 | protein_coding |
| ENSMMUG00000027614 | SNORD56B | M_mulatta | Animals | mono_intronic | C/D | ENSMMUG00000020067 | SIPA1L1 | protein_coding |
| ENSMMUG00000027673 | ENSMMUG00000027673 | M_mulatta | Animals | mono_intergenic | C/D | | | intergenic |
| ENSMMUG00000027695 | U3 | M_mulatta | Animals | mono_intronic | C/D | ENSMMUG00000015772 | CNTNAP2 | protein_coding |
| ENSMMUG00000027740 | SNORD115 | M_mulatta | Animals | mono_intergenic | C/D | | | intergenic |
| ENSMMUG00000027748 | U8 | M_mulatta | Animals | mono_intergenic | C/D | | | intergenic |
| ENSMMUG00000027767 | ENSMMUG00000027767 | M_mulatta | Animals | mono_intergenic | H/ACA | | | intergenic |
| ENSMMUG00000027786 | SNORD50B | M_mulatta | Animals | mono_intronic | C/D | ENSMMUG00000061913 | | non_coding |
| ENSMMUG00000027793 | SNORA80E | M_mulatta | Animals | mono_intronic | H/ACA | ENSMMUG00000004648 | KHDC4 | protein_coding |
| ENSMMUG00000027796 | SNORA70 | M_mulatta | Animals | mono_intergenic | H/ACA | | | intergenic |
| ENSMMUG00000027845 | SNORA70 | M_mulatta | Animals | mono_intronic | H/ACA | ENSMMUG00000009400 | COBLL1 | protein_coding |
| ENSMMUG00000027866 | ENSMMUG00000027866 | M_mulatta | Animals | mono_intergenic | H/ACA | | | intergenic |
| ENSMMUG00000027925 | SNORA70 | M_mulatta | Animals | mono_intergenic | H/ACA | | | intergenic |
| ENSMMUG00000027928 | ENSMMUG00000027928 | M_mulatta | Animals | mono_intergenic | H/ACA | | | intergenic |
| ENSMMUG00000027955 | ENSMMUG00000027955 | M_mulatta | Animals | mono_intergenic | C/D | | | intergenic |
| ENSMMUG00000028064 | ENSMMUG00000028064 | M_mulatta | Animals | intronic_cluster | C/D | ENSMMUG00000008025 | CEP295 | protein_coding |
| ENSMMUG00000028083 | ENSMMUG00000028083 | M_mulatta | Animals | intronic_cluster | C/D | ENSMMUG00000051148 | EIF1AX | protein_coding |
| ENSMMUG00000028098 | SNORA70 | M_mulatta | Animals | mono_intronic | H/ACA | ENSMMUG00000007842 | RAB30 | protein_coding |
| ENSMMUG00000028121 | U3 | M_mulatta | Animals | mono_intergenic | C/D | | | intergenic |

| | | | | | | | | |
|---|---|---|---|---|---|---|---|---|
| ENSMMUG00000028137 | SNORA1B | M_mulatta | Animals | mono_intergenic | H/ACA | | | intergenic |
| ENSMMUG00000028178 | SNORA75 | M_mulatta | Animals | mono_intergenic | H/ACA | | | intergenic |
| ENSMMUG00000028237 | SNORA24B | M_mulatta | Animals | mono_intronic | H/ACA | ENSMMUG00000005749 | PARP16 | protein_coding |
| ENSMMUG00000028279 | ENSMMUG00000028279 | M_mulatta | Animals | mono_intergenic | H/ACA | | | intergenic |
| ENSMMUG00000028346 | ENSMMUG00000028346 | M_mulatta | Animals | mono_intergenic | H/ACA | | | intergenic |
| ENSMMUG00000028350 | SNORA2A | M_mulatta | Animals | mono_intronic | H/ACA | ENSMMUG00000003877 | KANSL2 | protein_coding |
| ENSMMUG00000028360 | ENSMMUG00000028360 | M_mulatta | Animals | intronic_cluster | C/D | ENSMMUG00000051148 | EIF1AX | protein_coding |
| ENSMMUG00000028384 | SNORA75 | M_mulatta | Animals | mono_intronic | H/ACA | ENSMMUG00000018094 | NCL | protein_coding |
| ENSMMUG00000028422 | SNORA70 | M_mulatta | Animals | mono_intergenic | H/ACA | | | intergenic |
| ENSMMUG00000028447 | ENSMMUG00000028447 | M_mulatta | Animals | mono_intergenic | C/D | | | intergenic |
| ENSMMUG00000028449 | SNORD21 | M_mulatta | Animals | mono_intronic | C/D | ENSMMUG00000005072 | RPL5 | protein_coding |
| ENSMMUG00000028468 | SNORA70 | M_mulatta | Animals | mono_intergenic | H/ACA | | | intergenic |
| ENSMMUG00000028485 | U3 | M_mulatta | Animals | mono_intergenic | C/D | | | intergenic |
| ENSMMUG00000028538 | SNORA70 | M_mulatta | Animals | mono_intergenic | H/ACA | | | intergenic |
| ENSMMUG00000032689 | ENSMMUG00000032689 | M_mulatta | Animals | mono_intergenic | C/D | | | intergenic |
| ENSMMUG00000032696 | ENSMMUG00000032696 | M_mulatta | Animals | mono_intergenic | H/ACA | | | intergenic |
| ENSMMUG00000032702 | SNORD73 | M_mulatta | Animals | mono_intronic | C/D | ENSMMUG00000002676 | RPS3A | protein_coding |
| ENSMMUG00000032720 | SNORD64 | M_mulatta | Animals | mono_intergenic | C/D | | | intergenic |
| ENSMMUG00000032726 | SNORD73 | M_mulatta | Animals | mono_intronic | C/D | ENSMMUG00000002676 | RPS3A | protein_coding |
| ENSMMUG00000032743 | SNORD30 | M_mulatta | Animals | mono_intergenic | C/D | | | intergenic |
| ENSMMUG00000032744 | ENSMMUG00000032744 | M_mulatta | Animals | mono_intergenic | H/ACA | | | intergenic |
| ENSMMUG00000032749 | ENSMMUG00000032749 | M_mulatta | Animals | mono_intergenic | C/D | | | intergenic |
| ENSMMUG00000032750 | SNORD89 | M_mulatta | Animals | mono_intergenic | C/D | | | intergenic |
| ENSMMUG00000032751 | ENSMMUG00000032751 | M_mulatta | Animals | mono_intronic | C/D | ENSMMUG00000017519 | TSR1 | protein_coding |
| ENSMMUG00000032760 | SNORA3B | M_mulatta | Animals | mono_intronic | H/ACA | ENSMMUG00000021828 | RPL27A | protein_coding |
| ENSMMUG00000032769 | ENSMMUG00000032769 | M_mulatta | Animals | mono_intronic | H/ACA | ENSMMUG00000063307 | DEFB118 | protein_coding |
| ENSMMUG00000032774 | SNORD105 | M_mulatta | Animals | mono_intronic | C/D | ENSMMUG00000017211 | PPAN | protein_coding |
| ENSMMUG00000032777 | ENSMMUG00000032777 | M_mulatta | Animals | mono_intergenic | C/D | | | intergenic |
| ENSMMUG00000032785 | ENSMMUG00000032785 | M_mulatta | Animals | mono_intergenic | H/ACA | | | intergenic |
| ENSMMUG00000032787 | SNORD65B | M_mulatta | Animals | mono_intergenic | C/D | | | intergenic |
| ENSMMUG00000032788 | SNORD115 | M_mulatta | Animals | mono_intergenic | C/D | | | intergenic |
| ENSMMUG00000032794 | ENSMMUG00000032794 | M_mulatta | Animals | mono_intergenic | H/ACA | | | intergenic |
| ENSMMUG00000032797 | ENSMMUG00000032797 | M_mulatta | Animals | mono_intronic | H/ACA | ENSMMUG00000014630 | RAB3GAP1 | protein_coding |
| ENSMMUG00000032802 | ENSMMUG00000032802 | M_mulatta | Animals | mono_intergenic | H/ACA | | | intergenic |
| ENSMMUG00000032805 | SNORD115 | M_mulatta | Animals | mono_intergenic | C/D | | | intergenic |
| ENSMMUG00000032814 | SNORD17 | M_mulatta | Animals | mono_intronic | C/D | ENSMMUG00000011476 | SNX5 | protein_coding |
| ENSMMUG00000032815 | SNORD65 | M_mulatta | Animals | mono_intronic | C/D | ENSMMUG00000057308 | | protein_coding |
| ENSMMUG00000032816 | ENSMMUG00000032816 | M_mulatta | Animals | mono_intronic | H/ACA | ENSMMUG00000013259 | PCNA | protein_coding |
| ENSMMUG00000032817 | ENSMMUG00000032817 | M_mulatta | Animals | mono_intergenic | H/ACA | | | intergenic |
| ENSMMUG00000032818 | ENSMMUG00000032818 | M_mulatta | Animals | mono_intergenic | H/ACA | | | intergenic |
| ENSMMUG00000032819 | SNORD86 | M_mulatta | Animals | mono_intronic | C/D | ENSMMUG00000013888 | NOP56 | protein_coding |
| ENSMMUG00000032827 | ENSMMUG00000032827 | M_mulatta | Animals | mono_intronic | H/ACA | ENSMMUG00000012178 | CALCOCO2 | protein_coding |
| ENSMMUG00000032834 | SNORD72 | M_mulatta | Animals | mono_intronic | C/D | ENSMMUG00000062350 | | protein_coding |
| ENSMMUG00000032848 | ENSMMUG00000032848 | M_mulatta | Animals | mono_intronic | H/ACA | ENSMMUG00000008994 | EIF3A | protein_coding |
| ENSMMUG00000032849 | ENSMMUG00000032849 | M_mulatta | Animals | mono_intergenic | H/ACA | | | intergenic |

| | | | | | | | | |
|---|---|---|---|---|---|---|---|---|
| ENSMMUG00000032856 | SNORD42 | M_mulatta | Animals | mono_intronic | C/D | ENSMMUG00000018010 | RPL23A | protein_coding |
| ENSMMUG00000032860 | ENSMMUG00000032860 | M_mulatta | Animals | mono_intronic | C/D | ENSMMUG00000018123 | PPP1R13B | protein_coding |
| ENSMMUG00000032864 | ENSMMUG00000032864 | M_mulatta | Animals | mono_intergenic | H/ACA | | | intergenic |
| ENSMMUG00000032866 | SNORD74B | M_mulatta | Animals | mono_intergenic | C/D | | | intergenic |
| ENSMMUG00000032870 | ENSMMUG00000032870 | M_mulatta | Animals | mono_intergenic | H/ACA | | | intergenic |
| ENSMMUG00000032872 | SNORD19 | M_mulatta | Animals | mono_intronic | C/D | ENSMMUG00000012511 | GNL3 | protein_coding |
| ENSMMUG00000032878 | SNORA74 | M_mulatta | Animals | mono_intronic | H/ACA | ENSMMUG00000059290 | ATP6V0E1 | protein_coding |
| ENSMMUG00000032883 | U3 | M_mulatta | Animals | mono_intergenic | C/D | | | intergenic |
| ENSMMUG00000032884 | SNORA49 | M_mulatta | Animals | mono_intronic | H/ACA | ENSMMUG00000017729 | EP400 | protein_coding |
| ENSMMUG00000032890 | ENSMMUG00000032890 | M_mulatta | Animals | mono_intergenic | C/D | | | intergenic |
| ENSMMUG00000032891 | U3 | M_mulatta | Animals | mono_intronic | C/D | ENSMMUG00000012916 | WDR7 | protein_coding |
| ENSMMUG00000032897 | ENSMMUG00000032897 | M_mulatta | Animals | mono_intergenic | C/D | | | intergenic |
| ENSMMUG00000032902 | U3 | M_mulatta | Animals | mono_intergenic | C/D | | | intergenic |
| ENSMMUG00000032912 | ENSMMUG00000032912 | M_mulatta | Animals | mono_intronic | C/D | ENSMMUG00000017519 | TSR1 | protein_coding |
| ENSMMUG00000032915 | ENSMMUG00000032915 | M_mulatta | Animals | mono_intergenic | H/ACA | | | intergenic |
| ENSMMUG00000032924 | SNORD28 | M_mulatta | Animals | mono_intergenic | C/D | | | intergenic |
| ENSMMUG00000032928 | SNORA26 | M_mulatta | Animals | mono_exonic | H/ACA | ENSMMUG00000055376 | | non_coding |
| ENSMMUG00000032940 | U8 | M_mulatta | Animals | mono_intergenic | C/D | | | intergenic |
| ENSMMUG00000032941 | SNORA74 | M_mulatta | Animals | mono_intergenic | H/ACA | | | intergenic |
| ENSMMUG00000032942 | ENSMMUG00000032942 | M_mulatta | Animals | mono_intronic | H/ACA | ENSMMUG00000017701 | MYRIP | protein_coding |
| ENSMMUG00000032945 | ENSMMUG00000032945 | M_mulatta | Animals | mono_intergenic | H/ACA | | | intergenic |
| ENSMMUG00000032946 | ENSMMUG00000032946 | M_mulatta | Animals | mono_intronic | C/D | ENSMMUG00000005684 | TNPO2 | protein_coding |
| ENSMMUG00000032958 | U8 | M_mulatta | Animals | mono_intergenic | C/D | | | intergenic |
| ENSMMUG00000032960 | SNORD96 | M_mulatta | Animals | mono_intronic | C/D | ENSMMUG00000003604 | RACK1 | protein_coding |
| ENSMMUG00000032965 | SNORD81 | M_mulatta | Animals | mono_intergenic | C/D | | | intergenic |
| ENSMMUG00000032978 | ENSMMUG00000032978 | M_mulatta | Animals | mono_intronic | C/D | ENSMMUG00000053984 | | non_coding |
| ENSMMUG00000032988 | ENSMMUG00000032988 | M_mulatta | Animals | mono_intergenic | H/ACA | | | intergenic |
| ENSMMUG00000032995 | ENSMMUG00000032995 | M_mulatta | Animals | mono_intergenic | H/ACA | | | intergenic |
| ENSMMUG00000033004 | ENSMMUG00000033004 | M_mulatta | Animals | mono_intergenic | H/ACA | | | intergenic |
| ENSMMUG00000033007 | ENSMMUG00000033007 | M_mulatta | Animals | mono_intronic | H/ACA | ENSMMUG00000007198 | N4BP2L1 | protein_coding |
| ENSMMUG00000033015 | SNORD65C | M_mulatta | Animals | mono_intergenic | C/D | | | intergenic |
| ENSMMUG00000033018 | SNORD116 | M_mulatta | Animals | mono_intronic | C/D | ENSMMUG00000022640 | USH2A | protein_coding |
| ENSMMUG00000033025 | ENSMMUG00000033025 | M_mulatta | Animals | mono_intronic | H/ACA | ENSMMUG00000021843 | NFATC3 | protein_coding |
| ENSMMUG00000033028 | ENSMMUG00000033028 | M_mulatta | Animals | mono_intergenic | C/D | | | intergenic |
| ENSMMUG00000033033 | SNORD83A | M_mulatta | Animals | mono_intronic | C/D | ENSMMUG00000010029 | RPL3 | protein_coding |
| ENSMMUG00000033035 | SNORD12 | M_mulatta | Animals | mono_intronic | C/D | ENSMMUG00000058919 | | protein_coding |
| ENSMMUG00000033050 | SNORD69 | M_mulatta | Animals | mono_intronic | C/D | ENSMMUG00000012511 | GNL3 | protein_coding |
| ENSMMUG00000033051 | ENSMMUG00000033051 | M_mulatta | Animals | mono_intergenic | C/D | | | intergenic |
| ENSMMUG00000033056 | SNORD83B | M_mulatta | Animals | mono_intronic | C/D | ENSMMUG00000010029 | RPL3 | protein_coding |
| ENSMMUG00000033058 | ENSMMUG00000033058 | M_mulatta | Animals | mono_intergenic | H/ACA | | | intergenic |
| ENSMMUG00000033069 | SNORD70 | M_mulatta | Animals | mono_intronic | C/D | ENSMMUG00000000582 | NOP58 | protein_coding |
| ENSMMUG00000033071 | ENSMMUG00000033071 | M_mulatta | Animals | mono_intergenic | H/ACA | | | intergenic |
| ENSMMUG00000033074 | SNORA35 | M_mulatta | Animals | mono_intronic | H/ACA | ENSMMUG00000009607 | HTR2C | protein_coding |
| ENSMMUG00000033077 | SNORD77B | M_mulatta | Animals | mono_intergenic | C/D | | | intergenic |
| ENSMMUG00000033085 | ENSMMUG00000033085 | M_mulatta | Animals | mono_intronic | H/ACA | ENSMMUG00000008306 | PRKX | protein_coding |

| | | | | | | | | |
|---|---|---|---|---|---|---|---|---|
| ENSMMUG00000033091 | SNORA74 | M_mulatta | Animals | mono_intronic | H/ACA | ENSMMUG00000009847 | TIMM23 | protein_coding |
| ENSMMUG00000033096 | U3 | M_mulatta | Animals | mono_intronic | C/D | ENSMMUG00000017753 | EDA | protein_coding |
| ENSMMUG00000033103 | U3 | M_mulatta | Animals | mono_intergenic | C/D | | | intergenic |
| ENSMMUG00000033108 | SNORD66 | M_mulatta | Animals | mono_intronic | C/D | ENSMMUG00000010934 | EIF4G1 | protein_coding |
| ENSMMUG00000033113 | SNORD115 | M_mulatta | Animals | mono_intergenic | C/D | | | intergenic |
| ENSMMUG00000033121 | U3 | M_mulatta | Animals | mono_intergenic | C/D | | | intergenic |
| ENSMMUG00000033122 | ENSMMUG00000033122 | M_mulatta | Animals | mono_intergenic | H/ACA | | | intergenic |
| ENSMMUG00000033131 | U3 | M_mulatta | Animals | mono_intergenic | C/D | | | intergenic |
| ENSMMUG00000033134 | ENSMMUG00000033134 | M_mulatta | Animals | mono_intergenic | C/D | | | intergenic |
| ENSMMUG00000033140 | ENSMMUG00000033140 | M_mulatta | Animals | mono_intronic | H/ACA | ENSMMUG00000023806 | PRKAA1 | protein_coding |
| ENSMMUG00000033145 | ENSMMUG00000033145 | M_mulatta | Animals | mono_intergenic | C/D | | | intergenic |
| ENSMMUG00000033147 | ENSMMUG00000033147 | M_mulatta | Animals | mono_intronic | C/D | ENSMMUG00000053697 | | non_coding |
| ENSMMUG00000033148 | U3 | M_mulatta | Animals | mono_intronic | C/D | ENSMMUG00000004644 | ETFDH | protein_coding |
| ENSMMUG00000033155 | SNORA48 | M_mulatta | Animals | mono_intronic | H/ACA | ENSMMUG00000013499 | EIF4A1 | protein_coding |
| ENSMMUG00000033160 | ENSMMUG00000033160 | M_mulatta | Animals | mono_intergenic | H/ACA | | | intergenic |
| ENSMMUG00000033166 | ENSMMUG00000033166 | M_mulatta | Animals | mono_intronic | C/D | ENSMMUG00000021826 | UBL7 | protein_coding |
| ENSMMUG00000033169 | ENSMMUG00000033169 | M_mulatta | Animals | mono_intergenic | H/ACA | | | intergenic |
| ENSMMUG00000033170 | ENSMMUG00000033170 | M_mulatta | Animals | mono_intergenic | C/D | | | intergenic |
| ENSMMUG00000033176 | SNORD12C | M_mulatta | Animals | mono_intronic | C/D | ENSMMUG00000058919 | | protein_coding |
| ENSMMUG00000033178 | ENSMMUG00000033178 | M_mulatta | Animals | mono_intergenic | H/ACA | | | intergenic |
| ENSMMUG00000033180 | ENSMMUG00000033180 | M_mulatta | Animals | mono_intergenic | H/ACA | | | intergenic |
| ENSMMUG00000033186 | U3 | M_mulatta | Animals | mono_intergenic | C/D | | | intergenic |
| ENSMMUG00000033187 | ENSMMUG00000033187 | M_mulatta | Animals | mono_intergenic | H/ACA | | | intergenic |
| ENSMMUG00000033194 | ENSMMUG00000033194 | M_mulatta | Animals | mono_intergenic | C/D | | | intergenic |
| ENSMMUG00000033195 | SNORA12 | M_mulatta | Animals | mono_intronic | H/ACA | ENSMMUG00000018472 | CWF19L1 | protein_coding |
| ENSMMUG00000033200 | U3 | M_mulatta | Animals | mono_intergenic | C/D | | | intergenic |
| ENSMMUG00000033201 | SNORA40 | M_mulatta | Animals | intronic_cluster | H/ACA | ENSMMUG00000011646 | TAF1D | protein_coding |
| ENSMMUG00000033202 | SNORD81 | M_mulatta | Animals | mono_intergenic | C/D | | | intergenic |
| ENSMMUG00000033208 | SNORD26 | M_mulatta | Animals | mono_intronic | C/D | ENSMMUG00000064232 | | protein_coding |
| ENSMMUG00000033216 | U3 | M_mulatta | Animals | mono_intergenic | C/D | | | intergenic |
| ENSMMUG00000033220 | U8 | M_mulatta | Animals | mono_intergenic | C/D | | | intergenic |
| ENSMMUG00000033222 | SNORA53 | M_mulatta | Animals | mono_intronic | H/ACA | ENSMMUG00000023036 | SLC25A3 | protein_coding |
| ENSMMUG00000033223 | SNORD102 | M_mulatta | Animals | mono_intronic | C/D | ENSMMUG00000058755 | | protein_coding |
| ENSMMUG00000033225 | ENSMMUG00000033225 | M_mulatta | Animals | mono_intergenic | C/D | | | intergenic |
| ENSMMUG00000033230 | SNORD70B | M_mulatta | Animals | mono_intronic | C/D | ENSMMUG00000000582 | NOP58 | protein_coding |
| ENSMMUG00000033244 | SNORD81 | M_mulatta | Animals | mono_intergenic | C/D | | | intergenic |
| ENSMMUG00000033248 | SNORD90 | M_mulatta | Animals | mono_intronic | C/D | ENSMMUG00000021134 | RC3H2 | protein_coding |
| ENSMMUG00000033249 | U3 | M_mulatta | Animals | mono_intergenic | C/D | | | intergenic |
| ENSMMUG00000033250 | U3 | M_mulatta | Animals | mono_intergenic | C/D | | | intergenic |
| ENSMMUG00000033264 | ENSMMUG00000033264 | M_mulatta | Animals | mono_intergenic | H/ACA | | | intergenic |
| ENSMMUG00000033277 | U3 | M_mulatta | Animals | mono_intronic | C/D | ENSMMUG00000018360 | RCN1 | protein_coding |
| ENSMMUG00000033282 | ENSMMUG00000033282 | M_mulatta | Animals | mono_intergenic | H/ACA | | | intergenic |
| ENSMMUG00000033295 | ENSMMUG00000033295 | M_mulatta | Animals | mono_intergenic | H/ACA | | | intergenic |
| ENSMMUG00000033297 | ENSMMUG00000033297 | M_mulatta | Animals | mono_intergenic | H/ACA | | | intergenic |
| ENSMMUG00000033307 | ENSMMUG00000033307 | M_mulatta | Animals | mono_intergenic | H/ACA | | | intergenic |

| | | | | | | | | |
|---|---|---|---|---|---|---|---|---|
| ENSMMUG00000033312 | ENSMMUG00000033312 | M_mulatta | Animals | mono_intergenic | H/ACA | | | intergenic |
| ENSMMUG00000033319 | ENSMMUG00000033319 | M_mulatta | Animals | mono_intronic | H/ACA | ENSMMUG00000052139 | UBTD2 | protein_coding |
| ENSMMUG00000033322 | SNORD33 | M_mulatta | Animals | mono_intergenic | C/D | | | intergenic |
| ENSMMUG00000033527 | ENSMMUG00000033527 | M_mulatta | Animals | mono_intergenic | H/ACA | | | intergenic |
| ENSMMUG00000033530 | SNORA77B | M_mulatta | Animals | mono_intronic | H/ACA | ENSMMUG00000020100 | RANBP1 | protein_coding |
| ENSMMUG00000033539 | U3 | M_mulatta | Animals | mono_intergenic | C/D | | | intergenic |
| ENSMMUG00000033542 | SNORA77 | M_mulatta | Animals | mono_intronic | H/ACA | ENSMMUG00000021116 | ATP2B4 | protein_coding |
| ENSMMUG00000033551 | U3 | M_mulatta | Animals | mono_intergenic | C/D | | | intergenic |
| ENSMMUG00000033552 | SNORA11B | M_mulatta | Animals | mono_intronic | H/ACA | ENSMMUG00000008964 | DGLUCY | protein_coding |
| ENSMMUG00000033563 | U3 | M_mulatta | Animals | mono_intergenic | C/D | | | intergenic |
| ENSMMUG00000033572 | SNORA11 | M_mulatta | Animals | mono_intronic | H/ACA | ENSMMUG00000007345 | MAGED2 | protein_coding |
| ENSMMUG00000033584 | U3 | M_mulatta | Animals | mono_exonic | C/D | ENSMMUG00000054052 | | protein_coding |
| ENSMMUG00000033587 | SNORD111B | M_mulatta | Animals | mono_intronic | C/D | ENSMMUG00000017812 | SF3B3 | protein_coding |
| ENSMMUG00000033610 | SNORD100 | M_mulatta | Animals | mono_intronic | C/D | ENSMMUG00000013237 | RPS12 | protein_coding |
| ENSMMUG00000033622 | U3 | M_mulatta | Animals | mono_intergenic | C/D | | | intergenic |
| ENSMMUG00000033632 | ENSMMUG00000033632 | M_mulatta | Animals | mono_intergenic | H/ACA | | | intergenic |
| ENSMMUG00000033660 | ENSMMUG00000033660 | M_mulatta | Animals | mono_intergenic | C/D | | | intergenic |
| ENSMMUG00000033676 | U3 | M_mulatta | Animals | mono_intergenic | C/D | | | intergenic |
| ENSMMUG00000033704 | U3 | M_mulatta | Animals | mono_intronic | C/D | ENSMMUG00000019275 | FILIP1 | protein_coding |
| ENSMMUG00000033713 | SNORA11G | M_mulatta | Animals | mono_intronic | H/ACA | ENSMMUG00000008422 | TRO | protein_coding |
| ENSMMUG00000033737 | ENSMMUG00000033737 | M_mulatta | Animals | mono_intergenic | H/ACA | | | intergenic |
| ENSMMUG00000033740 | SNORA11F | M_mulatta | Animals | mono_intronic | H/ACA | ENSMMUG00000011280 | ECD | protein_coding |
| ENSMMUG00000033750 | ENSMMUG00000033750 | M_mulatta | Animals | mono_intergenic | H/ACA | | | intergenic |
| ENSMMUG00000033798 | SNORA79 | M_mulatta | Animals | mono_intronic | H/ACA | ENSMMUG00000022122 | GTF2A1 | protein_coding |
| ENSMMUG00000033806 | ENSMMUG00000033806 | M_mulatta | Animals | intergenic_cluster | H/ACA | | | intergenic |
| ENSMMUG00000033816 | ENSMMUG00000033816 | M_mulatta | Animals | mono_intergenic | H/ACA | | | intergenic |
| ENSMMUG00000033819 | ENSMMUG00000033819 | M_mulatta | Animals | mono_intergenic | C/D | | | intergenic |
| ENSMMUG00000033822 | U3 | M_mulatta | Animals | mono_intronic | C/D | ENSMMUG00000006567 | LAMB1 | protein_coding |
| ENSMMUG00000033830 | U3 | M_mulatta | Animals | mono_intergenic | C/D | | | intergenic |
| ENSMMUG00000033837 | SNORD93 | M_mulatta | Animals | mono_intergenic | C/D | | | intergenic |
| ENSMMUG00000033863 | SNORD98 | M_mulatta | Animals | mono_intronic | C/D | ENSMMUG00000016652 | CCAR1 | protein_coding |
| ENSMMUG00000033896 | SNORD88A | M_mulatta | Animals | mono_intronic | C/D | ENSMMUG00000051878 | | protein_coding |
| ENSMMUG00000033904 | U3 | M_mulatta | Animals | mono_intergenic | C/D | | | intergenic |
| ENSMMUG00000033913 | ENSMMUG00000033913 | M_mulatta | Animals | mono_intergenic | H/ACA | | | intergenic |
| ENSMMUG00000033934 | ENSMMUG00000033934 | M_mulatta | Animals | mono_intergenic | H/ACA | | | intergenic |
| ENSMMUG00000033951 | SNORD94 | M_mulatta | Animals | mono_intronic | C/D | ENSMMUG00000015820 | PTCD3 | protein_coding |
| ENSMMUG00000033987 | SNORD111 | M_mulatta | Animals | mono_intronic | C/D | ENSMMUG00000017812 | SF3B3 | protein_coding |
| ENSMMUG00000034006 | ENSMMUG00000034006 | M_mulatta | Animals | mono_intergenic | H/ACA | | | intergenic |
| ENSMMUG00000034027 | ENSMMUG00000034027 | M_mulatta | Animals | mono_intronic | H/ACA | ENSMMUG00000062608 | SMIM34 | protein_coding |
| ENSMMUG00000034048 | ENSMMUG00000034048 | M_mulatta | Animals | mono_intronic | H/ACA | ENSMMUG00000021399 | RERE | protein_coding |
| ENSMMUG00000034053 | SNORA11C | M_mulatta | Animals | mono_intronic | H/ACA | ENSMMUG00000008566 | ZNF157 | protein_coding |
| ENSMMUG00000034081 | SNORD99 | M_mulatta | Animals | mono_intergenic | C/D | | | intergenic |
| ENSMMUG00000034189 | ENSMMUG00000034189 | M_mulatta | Animals | mono_intergenic | H/ACA | | | intergenic |
| ENSMMUG00000034323 | SNORD12B | M_mulatta | Animals | mono_intronic | C/D | ENSMMUG00000058919 | | protein_coding |
| ENSMMUG00000034465 | U3 | M_mulatta | Animals | mono_intergenic | C/D | | | intergenic |

| | | | | | | | | |
|---|---|---|---|---|---|---|---|---|
| ENSMMUG00000034509 | U3 | M_mulatta | Animals | intronic_cluster | C/D | ENSMMUG00000046572 | TEX14 | protein_coding |
| ENSMMUG00000034542 | ENSMMUG00000034542 | M_mulatta | Animals | mono_intronic | C/D | ENSMMUG00000063833 | | non_coding |
| ENSMMUG00000034550 | SNORA35B | M_mulatta | Animals | mono_intronic | H/ACA | ENSMMUG00000017323 | LRCH2 | protein_coding |
| ENSMMUG00000034621 | SNORA36B | M_mulatta | Animals | mono_intronic | H/ACA | ENSMMUG00000007784 | RAB3GAP2 | protein_coding |
| ENSMMUG00000034773 | SNORD71 | M_mulatta | Animals | mono_intronic | C/D | ENSMMUG00000010878 | AP1G1 | protein_coding |
| ENSMMUG00000034790 | SNORD79 | M_mulatta | Animals | mono_intergenic | C/D | | | intergenic |
| ENSMMUG00000034870 | SNORA2C | M_mulatta | Animals | mono_intronic | H/ACA | ENSMMUG00000003877 | KANSL2 | protein_coding |
| ENSMMUG00000034909 | SNORA79B | M_mulatta | Animals | mono_intronic | H/ACA | ENSMMUG00000016251 | CCNB1IP1 | protein_coding |
| ENSMMUG00000035047 | SNORD63 | M_mulatta | Animals | mono_intronic | C/D | ENSMMUG00000005875 | HSPA9 | protein_coding |
| ENSMMUG00000035204 | U3 | M_mulatta | Animals | mono_intronic | C/D | ENSMMUG00000016487 | RALGAPA1 | protein_coding |
| ENSMMUG00000035223 | ENSMMUG00000035223 | M_mulatta | Animals | mono_exonic | C/D | ENSMMUG00000063833 | | non_coding |
| ENSMMUG00000035398 | SNORA73 | M_mulatta | Animals | mono_intronic | H/ACA | ENSMMUG00000020097 | RNGTT | protein_coding |
| ENSMMUG00000035418 | U3 | M_mulatta | Animals | mono_intergenic | C/D | | | intergenic |
| ENSMMUG00000035434 | ENSMMUG00000035434 | M_mulatta | Animals | mono_intergenic | C/D | | | intergenic |
| ENSMMUG00000035440 | ENSMMUG00000035440 | M_mulatta | Animals | mono_intronic | H/ACA | ENSMMUG00000014130 | ALDH3A2 | protein_coding |
| ENSMMUG00000035508 | SNORD116 | M_mulatta | Animals | mono_intergenic | C/D | | | intergenic |
| ENSMMUG00000035551 | SNORD11B | M_mulatta | Animals | mono_intronic | C/D | ENSMMUG00000000582 | NOP58 | protein_coding |
| ENSMMUG00000035560 | ENSMMUG00000035560 | M_mulatta | Animals | mono_intergenic | H/ACA | | | intergenic |
| ENSMMUG00000035561 | ENSMMUG00000035561 | M_mulatta | Animals | mono_intergenic | H/ACA | | | intergenic |
| ENSMMUG00000035580 | SNORA50D | M_mulatta | Animals | mono_intergenic | H/ACA | | | intergenic |
| ENSMMUG00000035606 | SCARNA12 | M_mulatta | Animals | mono_intronic | C/D | ENSMMUG00000010205 | PHB2 | protein_coding |
| ENSMMUG00000035634 | SNORA70 | M_mulatta | Animals | intronic_cluster | H/ACA | ENSMMUG00000008098 | USP34 | protein_coding |
| ENSMMUG00000035653 | SCARNA18 | M_mulatta | Animals | mono_intronic | H/ACA | ENSMMUG00000046560 | TMEM167A | protein_coding |
| ENSMMUG00000035674 | ENSMMUG00000035674 | M_mulatta | Animals | mono_intergenic | C/D | | | intergenic |
| ENSMMUG00000035691 | SNORD116 | M_mulatta | Animals | mono_intronic | C/D | ENSMMUG00000051317 | | non_coding |
| ENSMMUG00000035708 | ENSMMUG00000035708 | M_mulatta | Animals | intronic_cluster | C/D | ENSMMUG00000010891 | KAT6A | protein_coding |
| ENSMMUG00000035716 | ENSMMUG00000035716 | M_mulatta | Animals | mono_intergenic | H/ACA | | | intergenic |
| ENSMMUG00000035728 | ENSMMUG00000035728 | M_mulatta | Animals | mono_intronic | C/D | ENSMMUG00000013004 | MASP1 | protein_coding |
| ENSMMUG00000035737 | ENSMMUG00000035737 | M_mulatta | Animals | mono_intergenic | H/ACA | | | intergenic |
| ENSMMUG00000035738 | SNORD5 | M_mulatta | Animals | intronic_cluster | C/D | ENSMMUG00000011646 | TAF1D | protein_coding |
| ENSMMUG00000035742 | ENSMMUG00000035742 | M_mulatta | Animals | mono_intergenic | H/ACA | | | intergenic |
| ENSMMUG00000035747 | SNORA72 | M_mulatta | Animals | mono_intergenic | H/ACA | | | intergenic |
| ENSMMUG00000035750 | SNORD127 | M_mulatta | Animals | mono_intronic | C/D | ENSMMUG00000010862 | PRPF39 | protein_coding |
| ENSMMUG00000035752 | ENSMMUG00000035752 | M_mulatta | Animals | mono_intergenic | C/D | | | intergenic |
| ENSMMUG00000035755 | ENSMMUG00000035755 | M_mulatta | Animals | mono_intergenic | C/D | | | intergenic |
| ENSMMUG00000035758 | SNORD97 | M_mulatta | Animals | mono_intronic | C/D | ENSMMUG00000011436 | EIF4G2 | protein_coding |
| ENSMMUG00000035788 | SNORD22 | M_mulatta | Animals | mono_intronic | C/D | ENSMMUG00000064232 | | protein_coding |
| ENSMMUG00000035846 | SNORA70 | M_mulatta | Animals | mono_intergenic | H/ACA | | | intergenic |
| ENSMMUG00000035865 | SNORD123 | M_mulatta | Animals | mono_intronic | C/D | ENSMMUG00000059940 | | non_coding |
| ENSMMUG00000035888 | SNORD121A | M_mulatta | Animals | mono_intronic | C/D | ENSMMUG00000012597 | UBAP2 | protein_coding |
| ENSMMUG00000035912 | ENSMMUG00000035912 | M_mulatta | Animals | mono_intergenic | H/ACA | | | intergenic |
| ENSMMUG00000035947 | ENSMMUG00000035947 | M_mulatta | Animals | mono_intergenic | H/ACA | | | intergenic |
| ENSMMUG00000035968 | SNORA70 | M_mulatta | Animals | intronic_cluster | H/ACA | ENSMMUG00000008098 | USP34 | protein_coding |
| ENSMMUG00000036105 | ENSMMUG00000036105 | M_mulatta | Animals | mono_intergenic | C/D | | | intergenic |
| ENSMMUG00000036123 | ENSMMUG00000036123 | M_mulatta | Animals | mono_intergenic | H/ACA | | | intergenic |

| | | | | | | | | |
|---|---|---|---|---|---|---|---|---|
| ENSMMUG00000036133 | ENSMMUG00000036133 | M_mulatta | Animals | mono_intergenic | C/D | | | intergenic |
| ENSMMUG00000036136 | ENSMMUG00000036136 | M_mulatta | Animals | mono_intergenic | C/D | | | intergenic |
| ENSMMUG00000036166 | ENSMMUG00000036166 | M_mulatta | Animals | mono_intronic | H/ACA | ENSMMUG00000057928 | | protein_coding |
| ENSMMUG00000036194 | SNORA70 | M_mulatta | Animals | mono_intergenic | H/ACA | | | intergenic |
| ENSMMUG00000036223 | ENSMMUG00000036223 | M_mulatta | Animals | mono_intergenic | H/ACA | | | intergenic |
| ENSMMUG00000036243 | ENSMMUG00000036243 | M_mulatta | Animals | mono_intergenic | H/ACA | | | intergenic |
| ENSMMUG00000036250 | ENSMMUG00000036250 | M_mulatta | Animals | mono_intronic | H/ACA | ENSMMUG00000030218 | BCAP29 | protein_coding |
| ENSMMUG00000036267 | SNORD112 | M_mulatta | Animals | mono_intronic | C/D | ENSMMUG00000056002 | | non_coding |
| ENSMMUG00000036268 | ENSMMUG00000036268 | M_mulatta | Animals | mono_intergenic | H/ACA | | | intergenic |
| ENSMMUG00000036270 | SNORD22 | M_mulatta | Animals | mono_intergenic | C/D | | | intergenic |
| ENSMMUG00000036276 | ENSMMUG00000036276 | M_mulatta | Animals | mono_intronic | C/D | ENSMMUG00000022528 | SPAG9 | protein_coding |
| ENSMMUG00000036280 | ENSMMUG00000036280 | M_mulatta | Animals | mono_intergenic | C/D | | | intergenic |
| ENSMMUG00000036302 | ENSMMUG00000036302 | M_mulatta | Animals | mono_intergenic | H/ACA | | | intergenic |
| ENSMMUG00000036338 | ENSMMUG00000036338 | M_mulatta | Animals | mono_intergenic | H/ACA | | | intergenic |
| ENSMMUG00000036360 | ENSMMUG00000036360 | M_mulatta | Animals | mono_intergenic | H/ACA | | | intergenic |
| ENSMMUG00000036396 | ENSMMUG00000036396 | M_mulatta | Animals | mono_intergenic | H/ACA | | | intergenic |
| ENSMMUG00000036407 | ENSMMUG00000036407 | M_mulatta | Animals | mono_intronic | C/D | ENSMMUG00000045852 | WIPF3 | protein_coding |
| ENSMMUG00000036439 | ENSMMUG00000036439 | M_mulatta | Animals | mono_intergenic | H/ACA | | | intergenic |
| ENSMMUG00000036441 | ENSMMUG00000036441 | M_mulatta | Animals | mono_intergenic | H/ACA | | | intergenic |
| ENSMMUG00000036450 | SNORA70 | M_mulatta | Animals | mono_intergenic | H/ACA | | | intergenic |
| ENSMMUG00000036457 | SNORA70 | M_mulatta | Animals | mono_intergenic | H/ACA | | | intergenic |
| ENSMMUG00000036464 | ENSMMUG00000036464 | M_mulatta | Animals | mono_intergenic | H/ACA | | | intergenic |
| ENSMMUG00000036486 | SCARNA18B | M_mulatta | Animals | mono_intronic | H/ACA | ENSMMUG00000022641 | UCHL5 | protein_coding |
| ENSMMUG00000036502 | ENSMMUG00000036502 | M_mulatta | Animals | mono_intergenic | H/ACA | | | intergenic |
| ENSMMUG00000036516 | SNORD13 | M_mulatta | Animals | mono_intergenic | C/D | | | intergenic |
| ENSMMUG00000036546 | SNORD108 | M_mulatta | Animals | mono_intergenic | C/D | | | intergenic |
| ENSMMUG00000036552 | SNORD27 | M_mulatta | Animals | mono_intergenic | C/D | | | intergenic |
| ENSMMUG00000036554 | U8 | M_mulatta | Animals | mono_intronic | C/D | ENSMMUG00000000958 | AKR1C2 | protein_coding |
| ENSMMUG00000036561 | SNORA70 | M_mulatta | Animals | mono_intergenic | H/ACA | | | intergenic |
| ENSMMUG00000036563 | ENSMMUG00000036563 | M_mulatta | Animals | mono_intergenic | H/ACA | | | intergenic |
| ENSMMUG00000036570 | SNORA70 | M_mulatta | Animals | mono_intergenic | H/ACA | | | intergenic |
| ENSMMUG00000036585 | SNORA70 | M_mulatta | Animals | mono_intronic | H/ACA | ENSMMUG00000001901 | TMX1 | protein_coding |
| ENSMMUG00000036590 | SNORD107 | M_mulatta | Animals | mono_exonic | C/D | ENSMMUG00000023139 | SNRPN | protein_coding |
| ENSMMUG00000036603 | SNORA47 | M_mulatta | Animals | mono_intronic | H/ACA | ENSMMUG00000021357 | ZBED3 | protein_coding |
| ENSMMUG00000036683 | SNORA81 | M_mulatta | Animals | mono_intronic | H/ACA | ENSMMUG00000003702 | EIF4A2 | protein_coding |
| ENSMMUG00000036696 | ENSMMUG00000036696 | M_mulatta | Animals | mono_intergenic | H/ACA | | | intergenic |
| ENSMMUG00000036705 | ENSMMUG00000036705 | M_mulatta | Animals | mono_intergenic | C/D | | | intergenic |
| ENSMMUG00000036707 | SNORD125 | M_mulatta | Animals | mono_intronic | C/D | ENSMMUG00000021380 | AP1B1 | protein_coding |
| ENSMMUG00000036715 | ENSMMUG00000036715 | M_mulatta | Animals | mono_intergenic | H/ACA | | | intergenic |
| ENSMMUG00000036733 | ENSMMUG00000036733 | M_mulatta | Animals | mono_intronic | H/ACA | ENSMMUG00000051237 | | non_coding |
| ENSMMUG00000036746 | ENSMMUG00000036746 | M_mulatta | Animals | mono_intergenic | H/ACA | | | intergenic |
| ENSMMUG00000036758 | SNORD105B | M_mulatta | Animals | mono_intronic | C/D | ENSMMUG00000017211 | PPAN | protein_coding |
| ENSMMUG00000036773 | ENSMMUG00000036773 | M_mulatta | Animals | mono_intergenic | C/D | | | intergenic |
| ENSMMUG00000036785 | SNORD121B | M_mulatta | Animals | mono_intronic | C/D | ENSMMUG00000012597 | UBAP2 | protein_coding |
| ENSMMUG00000036830 | SCARNA10 | M_mulatta | Animals | mono_intronic | C/D | ENSMMUG00000018677 | NCAPD2 | protein_coding |

| | | | | | | | | |
|---|---|---|---|---|---|---|---|---|
| ENSMMUG00000036833 | U8 | M_mulatta | Animals | mono_intronic | C/D | ENSMMUG00000006347 | STAB2 | protein_coding |
| ENSMMUG00000036842 | ENSMMUG00000036842 | M_mulatta | Animals | mono_intergenic | H/ACA | | | intergenic |
| ENSMMUG00000036886 | ENSMMUG00000036886 | M_mulatta | Animals | mono_intergenic | H/ACA | | | intergenic |
| ENSMMUG00000036929 | ENSMMUG00000036929 | M_mulatta | Animals | mono_intergenic | H/ACA | | | intergenic |
| ENSMMUG00000036948 | ENSMMUG00000036948 | M_mulatta | Animals | mono_intergenic | H/ACA | | | intergenic |
| ENSMMUG00000036994 | SNORA78 | M_mulatta | Animals | mono_intergenic | H/ACA | | | intergenic |
| ENSMMUG00000037053 | SNORD124 | M_mulatta | Animals | mono_intronic | C/D | ENSMMUG00000002926 | | protein_coding |
| ENSMMUG00000037056 | SNORD11 | M_mulatta | Animals | mono_intronic | C/D | ENSMMUG00000000582 | NOP58 | protein_coding |
| ENSMMUG00000037061 | ENSMMUG00000037061 | M_mulatta | Animals | mono_intergenic | H/ACA | | | intergenic |
| ENSMMUG00000037064 | SNORD126 | M_mulatta | Animals | mono_intronic | C/D | ENSMMUG00000016251 | CCNB1IP1 | protein_coding |
| ENSMMUG00000037073 | SNORD19B | M_mulatta | Animals | mono_intronic | C/D | ENSMMUG00000012511 | GNL3 | protein_coding |
| ENSMMUG00000037082 | ENSMMUG00000037082 | M_mulatta | Animals | mono_intergenic | H/ACA | | | intergenic |
| ENSMMUG00000037097 | SNORA70 | M_mulatta | Animals | mono_intergenic | H/ACA | | | intergenic |
| ENSMMUG00000037143 | SNORD116 | M_mulatta | Animals | mono_intergenic | C/D | | | intergenic |
| ENSMMUG00000037163 | ENSMMUG00000037163 | M_mulatta | Animals | mono_intergenic | H/ACA | | | intergenic |
| ENSMMUG00000037181 | SNORA84 | M_mulatta | Animals | mono_intronic | H/ACA | ENSMMUG00000004628 | IARS1 | protein_coding |
| ENSMMUG00000037192 | SNORD2 | M_mulatta | Animals | mono_intronic | C/D | ENSMMUG00000003702 | EIF4A2 | protein_coding |
| ENSMMUG00000037202 | ENSMMUG00000037202 | M_mulatta | Animals | mono_intronic | H/ACA | ENSMMUG00000000647 | C1H1orf141 | protein_coding |
| ENSMMUG00000037214 | SNORD54 | M_mulatta | Animals | mono_intronic | C/D | ENSMMUG00000053276 | RPS20 | protein_coding |
| ENSMMUG00000037227 | ENSMMUG00000037227 | M_mulatta | Animals | mono_intronic | H/ACA | ENSMMUG00000001711 | SOD1 | protein_coding |
| ENSMMUG00000037244 | ENSMMUG00000037244 | M_mulatta | Animals | mono_intergenic | H/ACA | | | intergenic |
| ENSMMUG00000037284 | SNORA59A | M_mulatta | Animals | mono_intronic | H/ACA | ENSMMUG00000021449 | VPS13D | protein_coding |
| ENSMMUG00000037295 | SNORD116 | M_mulatta | Animals | mono_intronic | C/D | ENSMMUG00000000871 | MVB12B | protein_coding |
| ENSMMUG00000037310 | SNORD10 | M_mulatta | Animals | mono_intronic | C/D | ENSMMUG00000013499 | EIF4A1 | protein_coding |
| ENSMMUG00000037339 | ENSMMUG00000037339 | M_mulatta | Animals | mono_intergenic | H/ACA | | | intergenic |
| ENSMMUG00000037358 | ENSMMUG00000037358 | M_mulatta | Animals | mono_intergenic | C/D | | | intergenic |
| ENSMMUG00000037402 | ENSMMUG00000037402 | M_mulatta | Animals | mono_intergenic | H/ACA | | | intergenic |
| ENSMMUG00000037428 | ENSMMUG00000037428 | M_mulatta | Animals | mono_intergenic | H/ACA | | | intergenic |
| ENSMMUG00000037445 | ENSMMUG00000037445 | M_mulatta | Animals | mono_intergenic | H/ACA | | | intergenic |
| ENSMMUG00000037615 | U3 | M_mulatta | Animals | intronic_cluster | C/D | ENSMMUG00000011776 | | protein_coding |
| ENSMMUG00000038597 | SNORD23 | M_mulatta | Animals | mono_intronic | C/D | ENSMMUG00000013348 | NOP53 | protein_coding |
| ENSMMUG00000039114 | SNORA70 | M_mulatta | Animals | mono_intergenic | H/ACA | | | intergenic |
| ENSMMUG00000039286 | ENSMMUG00000039286 | M_mulatta | Animals | mono_intronic | C/D | ENSMMUG00000061109 | | non_coding |
| ENSMMUG00000039914 | SNORD51 | M_mulatta | Animals | mono_intronic | C/D | ENSMMUG00000001054 | EEF1B2 | protein_coding |
| ENSMMUG00000039920 | SNORD39 | M_mulatta | Animals | mono_intronic | C/D | ENSMMUG00000062207 | | non_coding |
| ENSMMUG00000040148 | SNORD92 | M_mulatta | Animals | mono_intronic | C/D | ENSMMUG00000022268 | WDR43 | protein_coding |
| ENSMMUG00000040201 | ENSMMUG00000040201 | M_mulatta | Animals | mono_exonic | C/D | ENSMMUG00000004175 | TRPM2 | protein_coding |
| ENSMMUG00000040821 | U3 | M_mulatta | Animals | mono_intergenic | C/D | | | intergenic |
| ENSMMUG00000040893 | SNORA50A | M_mulatta | Animals | intronic_cluster | H/ACA | ENSMMUG00000014625 | CNOT1 | protein_coding |
| ENSMMUG00000041436 | SNORA10 | M_mulatta | Animals | mono_intronic | H/ACA | ENSMMUG00000029267 | RPS2 | protein_coding |
| ENSMMUG00000041514 | ENSMMUG00000041514 | M_mulatta | Animals | intergenic_cluster | H/ACA | | | intergenic |
| ENSMMUG00000041843 | SNORD116 | M_mulatta | Animals | mono_intronic | C/D | ENSMMUG00000064025 | | non_coding |
| ENSMMUG00000041899 | ENSMMUG00000041899 | M_mulatta | Animals | mono_intergenic | H/ACA | | | intergenic |
| ENSMMUG00000042209 | U3 | M_mulatta | Animals | mono_intergenic | C/D | | | intergenic |
| ENSMMUG00000042811 | SNORD38C | M_mulatta | Animals | mono_intronic | C/D | ENSMMUG00000011208 | DCP1A | protein_coding |

| | | | | | | | | |
|---|---|---|---|---|---|---|---|---|
| ENSMMUG00000043406 | SNORA50B | M_mulatta | Animals | mono_intronic | H/ACA | ENSMMUG00000012113 | LARGE1 | protein_coding |
| ENSMMUG00000043460 | ENSMMUG00000043460 | M_mulatta | Animals | mono_exonic | C/D | ENSMMUG00000004175 | TRPM2 | protein_coding |
| ENSMMUG00000043954 | SNORA63 | M_mulatta | Animals | mono_intergenic | H/ACA | | | intergenic |
| ENSMMUG00000044156 | SNORD36 | M_mulatta | Animals | mono_intergenic | C/D | | | intergenic |
| ENSMMUG00000044625 | SNORD39 | M_mulatta | Animals | mono_intronic | C/D | ENSMMUG00000015270 | RPS8 | protein_coding |
| ENSMMUG00000044993 | SNORD53 | M_mulatta | Animals | mono_intronic | C/D | ENSMMUG00000022268 | WDR43 | protein_coding |
| ENSMMUG00000045100 | SNORD110 | M_mulatta | Animals | mono_intronic | C/D | ENSMMUG00000013888 | NOP56 | protein_coding |
| ENSMMUG00000045373 | SNORD61 | M_mulatta | Animals | mono_intronic | C/D | ENSMMUG00000040370 | | protein_coding |
| ENSMMUG00000045443 | ENSMMUG00000045443 | M_mulatta | Animals | mono_intergenic | H/ACA | | | intergenic |
| ENSMMUG00000045756 | U3 | M_mulatta | Animals | mono_intronic | C/D | ENSMMUG00000014950 | CATSPERB | protein_coding |
| ENSMMUG00000045792 | ENSMMUG00000045792 | M_mulatta | Animals | intergenic_cluster | H/ACA | | | intergenic |
| ENSMMUG00000046471 | ENSMMUG00000046471 | M_mulatta | Animals | intergenic_cluster | H/ACA | | | intergenic |
| ENSMMUG00000047032 | ENSMMUG00000047032 | M_mulatta | Animals | intronic_cluster | C/D | ENSMMUG00000011776 | | protein_coding |
| ENSMMUG00000047727 | ENSMMUG00000047727 | M_mulatta | Animals | intergenic_cluster | H/ACA | | | intergenic |
| ENSMMUG00000047794 | ENSMMUG00000047794 | M_mulatta | Animals | mono_intronic | H/ACA | ENSMMUG00000003546 | NDC1 | protein_coding |
| ENSMMUG00000047993 | SNORD53B | M_mulatta | Animals | mono_intronic | C/D | ENSMMUG00000022268 | WDR43 | protein_coding |
| ENSMMUG00000048011 | SNORD39 | M_mulatta | Animals | mono_intergenic | C/D | | | intergenic |
| ENSMMUG00000048052 | ENSMMUG00000048052 | M_mulatta | Animals | intergenic_cluster | H/ACA | | | intergenic |
| ENSMMUG00000048598 | ENSMMUG00000048598 | M_mulatta | Animals | mono_intergenic | H/ACA | | | intergenic |
| ENSMMUG00000048996 | SNORD39 | M_mulatta | Animals | mono_intronic | C/D | ENSMMUG00000003488 | C5H4orf45 | protein_coding |
| ENSMMUG00000049141 | SNORD38B | M_mulatta | Animals | mono_intronic | C/D | ENSMMUG00000015270 | RPS8 | protein_coding |
| ENSMMUG00000049269 | SNORD115 | M_mulatta | Animals | mono_intergenic | C/D | | | intergenic |
| ENSMMUG00000049300 | U3 | M_mulatta | Animals | mono_intergenic | C/D | | | intergenic |
| ENSMMUG00000049322 | ENSMMUG00000049322 | M_mulatta | Animals | mono_intergenic | C/D | | | intergenic |
| ENSMMUG00000049362 | ENSMMUG00000049362 | M_mulatta | Animals | mono_intronic | C/D | ENSMMUG00000004485 | LHFPL6 | protein_coding |
| ENSMMUG00000049402 | SNORD62 | M_mulatta | Animals | mono_intronic | C/D | ENSMMUG00000008036 | MAN1B1 | protein_coding |
| ENSMMUG00000049409 | SNORD115 | M_mulatta | Animals | mono_intergenic | C/D | | | intergenic |
| ENSMMUG00000049421 | SNORD115 | M_mulatta | Animals | mono_intergenic | C/D | | | intergenic |
| ENSMMUG00000049430 | ENSMMUG00000049430 | M_mulatta | Animals | mono_intergenic | C/D | | | intergenic |
| ENSMMUG00000049475 | ENSMMUG00000049475 | M_mulatta | Animals | mono_intronic | C/D | ENSMMUG00000004087 | BRAT1 | protein_coding |
| ENSMMUG00000049481 | ENSMMUG00000049481 | M_mulatta | Animals | mono_intergenic | C/D | | | intergenic |
| ENSMMUG00000049513 | ENSMMUG00000049513 | M_mulatta | Animals | mono_intergenic | C/D | | | intergenic |
| ENSMMUG00000049546 | ENSMMUG00000049546 | M_mulatta | Animals | mono_intronic | C/D | ENSMMUG00000018600 | MYL4 | protein_coding |
| ENSMMUG00000049571 | SNORD116 | M_mulatta | Animals | mono_intronic | C/D | ENSMMUG00000064025 | | non_coding |
| ENSMMUG00000049579 | SNORD115 | M_mulatta | Animals | mono_intergenic | C/D | | | intergenic |
| ENSMMUG00000049681 | ENSMMUG00000049681 | M_mulatta | Animals | mono_intergenic | C/D | | | intergenic |
| ENSMMUG00000049700 | ENSMMUG00000049700 | M_mulatta | Animals | mono_intergenic | H/ACA | | | intergenic |
| ENSMMUG00000049707 | SNORD115 | M_mulatta | Animals | mono_intergenic | C/D | | | intergenic |
| ENSMMUG00000049711 | ENSMMUG00000049711 | M_mulatta | Animals | mono_intergenic | C/D | | | intergenic |
| ENSMMUG00000049732 | ENSMMUG00000049732 | M_mulatta | Animals | mono_intergenic | C/D | | | intergenic |
| ENSMMUG00000049791 | ENSMMUG00000049791 | M_mulatta | Animals | mono_intergenic | C/D | | | intergenic |
| ENSMMUG00000049914 | SNORD115 | M_mulatta | Animals | mono_intergenic | C/D | | | intergenic |
| ENSMMUG00000049923 | ENSMMUG00000049923 | M_mulatta | Animals | mono_intronic | C/D | ENSMMUG00000007870 | | protein_coding |
| ENSMMUG00000049933 | ENSMMUG00000049933 | M_mulatta | Animals | mono_intronic | C/D | ENSMMUG00000061905 | | non_coding |
| ENSMMUG00000049938 | SNORD115 | M_mulatta | Animals | mono_intergenic | C/D | | | intergenic |

| | | | | | | | | |
|---|---|---|---|---|---|---|---|---|
| ENSMMUG00000050012 | ENSMMUG00000050012 | M_mulatta | Animals | mono_intergenic | C/D | | | intergenic |
| ENSMMUG00000050055 | ENSMMUG00000050055 | M_mulatta | Animals | mono_intergenic | C/D | | | intergenic |
| ENSMMUG00000050103 | SNORD115 | M_mulatta | Animals | mono_intergenic | C/D | | | intergenic |
| ENSMMUG00000050118 | ENSMMUG00000050118 | M_mulatta | Animals | mono_intronic | C/D | ENSMMUG00000015339 | CFAP58 | protein_coding |
| ENSMMUG00000050129 | ENSMMUG00000050129 | M_mulatta | Animals | mono_intronic | C/D | ENSMMUG00000020644 | CHRDL2 | protein_coding |
| ENSMMUG00000050138 | SNORD114-4 | M_mulatta | Animals | mono_intronic | C/D | ENSMMUG00000053357 | | non_coding |
| ENSMMUG00000050153 | U3 | M_mulatta | Animals | mono_intergenic | C/D | | | intergenic |
| ENSMMUG00000050171 | SNORD116 | M_mulatta | Animals | mono_intronic | C/D | ENSMMUG00000064025 | | non_coding |
| ENSMMUG00000050222 | SNORD114-3 | M_mulatta | Animals | mono_intronic | C/D | ENSMMUG00000053357 | | non_coding |
| ENSMMUG00000050243 | ENSMMUG00000050243 | M_mulatta | Animals | mono_intronic | C/D | ENSMMUG00000019372 | SUPT5H | protein_coding |
| ENSMMUG00000050248 | SNORD115 | M_mulatta | Animals | mono_intergenic | C/D | | | intergenic |
| ENSMMUG00000050262 | ENSMMUG00000050262 | M_mulatta | Animals | mono_intergenic | C/D | | | intergenic |
| ENSMMUG00000050292 | SNORD116 | M_mulatta | Animals | mono_intergenic | C/D | | | intergenic |
| ENSMMUG00000050309 | SNORD62 | M_mulatta | Animals | mono_intronic | C/D | ENSMMUG00000009973 | PRRC2B | protein_coding |
| ENSMMUG00000050310 | ENSMMUG00000050310 | M_mulatta | Animals | mono_intergenic | C/D | | | intergenic |
| ENSMMUG00000050330 | ENSMMUG00000050330 | M_mulatta | Animals | mono_intergenic | C/D | | | intergenic |
| ENSMMUG00000050335 | ENSMMUG00000050335 | M_mulatta | Animals | mono_intronic | C/D | ENSMMUG00000023104 | ELMO1 | protein_coding |
| ENSMMUG00000050351 | ENSMMUG00000050351 | M_mulatta | Animals | mono_intergenic | C/D | | | intergenic |
| ENSMMUG00000050362 | ENSMMUG00000050362 | M_mulatta | Animals | mono_intronic | C/D | ENSMMUG00000013601 | MED17 | protein_coding |
| ENSMMUG00000050391 | SNORD115 | M_mulatta | Animals | mono_intergenic | C/D | | | intergenic |
| ENSMMUG00000050464 | ENSMMUG00000050464 | M_mulatta | Animals | mono_intergenic | C/D | | | intergenic |
| ENSMMUG00000050476 | SNORD115 | M_mulatta | Animals | mono_intergenic | C/D | | | intergenic |
| ENSMMUG00000050520 | ENSMMUG00000050520 | M_mulatta | Animals | mono_intergenic | C/D | | | intergenic |
| ENSMMUG00000050521 | ENSMMUG00000050521 | M_mulatta | Animals | mono_intronic | C/D | ENSMMUG00000020083 | TESK2 | protein_coding |
| ENSMMUG00000050522 | ENSMMUG00000050522 | M_mulatta | Animals | mono_intergenic | C/D | | | intergenic |
| ENSMMUG00000050590 | U3 | M_mulatta | Animals | mono_intronic | C/D | ENSMMUG00000009626 | PLCD4 | protein_coding |
| ENSMMUG00000050607 | ENSMMUG00000050607 | M_mulatta | Animals | mono_intronic | C/D | ENSMMUG00000059889 | | non_coding |
| ENSMMUG00000050681 | SNORD116 | M_mulatta | Animals | mono_intronic | C/D | ENSMMUG00000064025 | | non_coding |
| ENSMMUG00000050691 | SNORD116 | M_mulatta | Animals | mono_intergenic | C/D | | | intergenic |
| ENSMMUG00000050729 | ENSMMUG00000050729 | M_mulatta | Animals | mono_intergenic | C/D | | | intergenic |
| ENSMMUG00000050738 | SNORD115 | M_mulatta | Animals | mono_intergenic | C/D | | | intergenic |
| ENSMMUG00000050848 | SNORD115 | M_mulatta | Animals | mono_intergenic | C/D | | | intergenic |
| ENSMMUG00000050901 | U3 | M_mulatta | Animals | mono_intergenic | C/D | | | intergenic |
| ENSMMUG00000050914 | ENSMMUG00000050914 | M_mulatta | Animals | mono_intergenic | C/D | | | intergenic |
| ENSMMUG00000050936 | ENSMMUG00000050936 | M_mulatta | Animals | mono_intergenic | C/D | | | intergenic |
| ENSMMUG00000050938 | ENSMMUG00000050938 | M_mulatta | Animals | mono_intergenic | C/D | | | intergenic |
| ENSMMUG00000050949 | ENSMMUG00000050949 | M_mulatta | Animals | mono_intergenic | C/D | | | intergenic |
| ENSMMUG00000050959 | ENSMMUG00000050959 | M_mulatta | Animals | mono_intronic | C/D | ENSMMUG00000007870 | | protein_coding |
| ENSMMUG00000050963 | SNORD24 | M_mulatta | Animals | mono_exonic | C/D | ENSMMUG00000060229 | | non_coding |
| ENSMMUG00000050977 | ENSMMUG00000050977 | M_mulatta | Animals | mono_intergenic | C/D | | | intergenic |
| ENSMMUG00000050992 | ENSMMUG00000050992 | M_mulatta | Animals | mono_intronic | C/D | ENSMMUG00000011743 | SF3B2 | protein_coding |
| ENSMMUG00000051013 | ENSMMUG00000051013 | M_mulatta | Animals | mono_intronic | C/D | ENSMMUG00000007783 | IARS2 | protein_coding |
| ENSMMUG00000051018 | ENSMMUG00000051018 | M_mulatta | Animals | mono_intergenic | C/D | | | intergenic |
| ENSMMUG00000051049 | SNORD116 | M_mulatta | Animals | mono_intergenic | C/D | | | intergenic |
| ENSMMUG00000051056 | SNORD116 | M_mulatta | Animals | mono_intronic | C/D | ENSMMUG00000064025 | | non_coding |

| | | | | | | | | |
|---|---|---|---|---|---|---|---|---|
| ENSMMUG00000051092 | ENSMMUG00000051092 | M_mulatta | Animals | mono_intronic | C/D | ENSMMUG00000028921 | NETO2 | protein_coding |
| ENSMMUG00000051098 | U3 | M_mulatta | Animals | mono_intronic | C/D | ENSMMUG00000013885 | MORC1 | non_coding |
| ENSMMUG00000051103 | U3 | M_mulatta | Animals | mono_intergenic | C/D | | | intergenic |
| ENSMMUG00000051104 | ENSMMUG00000051104 | M_mulatta | Animals | mono_intergenic | C/D | | | intergenic |
| ENSMMUG00000051135 | ENSMMUG00000051135 | M_mulatta | Animals | mono_intergenic | C/D | | | intergenic |
| ENSMMUG00000051156 | ENSMMUG00000051156 | M_mulatta | Animals | mono_intronic | C/D | ENSMMUG00000041221 | SNURF | protein_coding |
| ENSMMUG00000051164 | ENSMMUG00000051164 | M_mulatta | Animals | mono_intergenic | C/D | | | intergenic |
| ENSMMUG00000051170 | ENSMMUG00000051170 | M_mulatta | Animals | mono_intronic | H/ACA | ENSMMUG00000061165 | | non_coding |
| ENSMMUG00000051214 | ENSMMUG00000051214 | M_mulatta | Animals | mono_intergenic | C/D | | | intergenic |
| ENSMMUG00000051281 | ENSMMUG00000051281 | M_mulatta | Animals | mono_intronic | C/D | ENSMMUG00000003658 | ATG2B | protein_coding |
| ENSMMUG00000051292 | SNORD115 | M_mulatta | Animals | mono_intergenic | C/D | | | intergenic |
| ENSMMUG00000051293 | ENSMMUG00000051293 | M_mulatta | Animals | mono_intergenic | C/D | | | intergenic |
| ENSMMUG00000051316 | ENSMMUG00000051316 | M_mulatta | Animals | mono_intergenic | C/D | | | intergenic |
| ENSMMUG00000051339 | ENSMMUG00000051339 | M_mulatta | Animals | mono_intergenic | C/D | | | intergenic |
| ENSMMUG00000051356 | SNORD115 | M_mulatta | Animals | mono_intergenic | C/D | | | intergenic |
| ENSMMUG00000051359 | ENSMMUG00000051359 | M_mulatta | Animals | mono_intergenic | C/D | | | intergenic |
| ENSMMUG00000051394 | ENSMMUG00000051394 | M_mulatta | Animals | mono_intergenic | C/D | | | intergenic |
| ENSMMUG00000051432 | SNORD114-30 | M_mulatta | Animals | mono_intronic | C/D | ENSMMUG00000063833 | | non_coding |
| ENSMMUG00000051433 | ENSMMUG00000051433 | M_mulatta | Animals | mono_intronic | C/D | ENSMMUG00000060229 | | non_coding |
| ENSMMUG00000051473 | ENSMMUG00000051473 | M_mulatta | Animals | mono_intronic | H/ACA | ENSMMUG00000062026 | | protein_coding |
| ENSMMUG00000051496 | ENSMMUG00000051496 | M_mulatta | Animals | mono_intronic | C/D | ENSMMUG00000018344 | MYO15A | protein_coding |
| ENSMMUG00000051539 | ENSMMUG00000051539 | M_mulatta | Animals | mono_intronic | C/D | ENSMMUG00000050185 | | protein_coding |
| ENSMMUG00000051542 | SNORD115 | M_mulatta | Animals | mono_intergenic | C/D | | | intergenic |
| ENSMMUG00000051564 | ENSMMUG00000051564 | M_mulatta | Animals | mono_intronic | C/D | ENSMMUG00000019399 | FZD6 | protein_coding |
| ENSMMUG00000051629 | ENSMMUG00000051629 | M_mulatta | Animals | mono_intergenic | C/D | | | intergenic |
| ENSMMUG00000051666 | ENSMMUG00000051666 | M_mulatta | Animals | mono_intronic | C/D | ENSMMUG00000020524 | PAFAH1B1 | protein_coding |
| ENSMMUG00000051764 | ENSMMUG00000051764 | M_mulatta | Animals | mono_intergenic | C/D | | | intergenic |
| ENSMMUG00000051796 | ENSMMUG00000051796 | M_mulatta | Animals | mono_intergenic | C/D | | | intergenic |
| ENSMMUG00000051798 | U3 | M_mulatta | Animals | mono_intergenic | C/D | | | intergenic |
| ENSMMUG00000051802 | U3 | M_mulatta | Animals | mono_intergenic | C/D | | | intergenic |
| ENSMMUG00000051830 | ENSMMUG00000051830 | M_mulatta | Animals | mono_intergenic | C/D | | | intergenic |
| ENSMMUG00000051838 | SNORD116 | M_mulatta | Animals | mono_intergenic | C/D | | | intergenic |
| ENSMMUG00000051841 | ENSMMUG00000051841 | M_mulatta | Animals | mono_intergenic | C/D | | | intergenic |
| ENSMMUG00000051847 | ENSMMUG00000051847 | M_mulatta | Animals | mono_intergenic | C/D | | | intergenic |
| ENSMMUG00000051851 | ENSMMUG00000051851 | M_mulatta | Animals | mono_intergenic | C/D | | | intergenic |
| ENSMMUG00000051868 | ENSMMUG00000051868 | M_mulatta | Animals | mono_intergenic | C/D | | | intergenic |
| ENSMMUG00000051881 | ENSMMUG00000051881 | M_mulatta | Animals | mono_intergenic | C/D | | | intergenic |
| ENSMMUG00000051897 | U3 | M_mulatta | Animals | mono_intergenic | C/D | | | intergenic |
| ENSMMUG00000051899 | ENSMMUG00000051899 | M_mulatta | Animals | mono_intronic | C/D | ENSMMUG00000009129 | FOXK2 | protein_coding |
| ENSMMUG00000051908 | ENSMMUG00000051908 | M_mulatta | Animals | mono_intergenic | C/D | | | intergenic |
| ENSMMUG00000051935 | SNORD115 | M_mulatta | Animals | mono_intergenic | C/D | | | intergenic |
| ENSMMUG00000051986 | ENSMMUG00000051986 | M_mulatta | Animals | mono_intergenic | C/D | | | intergenic |
| ENSMMUG00000052022 | ENSMMUG00000052022 | M_mulatta | Animals | mono_intergenic | C/D | | | intergenic |
| ENSMMUG00000052057 | SNORD114-9 | M_mulatta | Animals | mono_intergenic | C/D | | | intergenic |
| ENSMMUG00000052119 | ENSMMUG00000052119 | M_mulatta | Animals | mono_intergenic | C/D | | | intergenic |

| | | | | | | | | |
|---|---|---|---|---|---|---|---|---|
| ENSMMUG00000052123 | SNORA52 | M_mulatta | Animals | mono_intronic | H/ACA | ENSMMUG00000020627 | RPLP2 | protein_coding |
| ENSMMUG00000052126 | ENSMMUG00000052126 | M_mulatta | Animals | mono_intronic | C/D | ENSMMUG00000011510 | SLC30A4 | protein_coding |
| ENSMMUG00000052145 | U3 | M_mulatta | Animals | mono_intronic | C/D | ENSMMUG00000022944 | ZBTB44 | protein_coding |
| ENSMMUG00000052152 | ENSMMUG00000052152 | M_mulatta | Animals | mono_intergenic | C/D | | | intergenic |
| ENSMMUG00000052165 | ENSMMUG00000052165 | M_mulatta | Animals | mono_intergenic | C/D | | | intergenic |
| ENSMMUG00000052170 | ENSMMUG00000052170 | M_mulatta | Animals | mono_intergenic | C/D | | | intergenic |
| ENSMMUG00000052175 | SNORD39 | M_mulatta | Animals | mono_intronic | C/D | ENSMMUG00000052071 | RAMP1 | protein_coding |
| ENSMMUG00000052189 | ENSMMUG00000052189 | M_mulatta | Animals | mono_intronic | C/D | ENSMMUG00000020996 | NCALD | protein_coding |
| ENSMMUG00000052197 | ENSMMUG00000052197 | M_mulatta | Animals | mono_intergenic | C/D | | | intergenic |
| ENSMMUG00000052200 | ENSMMUG00000052200 | M_mulatta | Animals | mono_intergenic | C/D | | | intergenic |
| ENSMMUG00000052217 | ENSMMUG00000052217 | M_mulatta | Animals | mono_intergenic | C/D | | | intergenic |
| ENSMMUG00000052244 | ENSMMUG00000052244 | M_mulatta | Animals | mono_intergenic | C/D | | | intergenic |
| ENSMMUG00000052415 | ENSMMUG00000052415 | M_mulatta | Animals | mono_intronic | C/D | ENSMMUG00000050546 | EFHD1 | protein_coding |
| ENSMMUG00000052419 | ENSMMUG00000052419 | M_mulatta | Animals | mono_intergenic | C/D | | | intergenic |
| ENSMMUG00000052458 | ENSMMUG00000052458 | M_mulatta | Animals | mono_intronic | H/ACA | ENSMMUG00000021676 | SVOPL | protein_coding |
| ENSMMUG00000052498 | ENSMMUG00000052498 | M_mulatta | Animals | mono_intronic | C/D | ENSMMUG00000052792 | | protein_coding |
| ENSMMUG00000052528 | ENSMMUG00000052528 | M_mulatta | Animals | mono_intronic | C/D | ENSMMUG00000059670 | GTF3C6 | protein_coding |
| ENSMMUG00000052553 | ENSMMUG00000052553 | M_mulatta | Animals | mono_intronic | C/D | ENSMMUG00000045714 | FAM178B | protein_coding |
| ENSMMUG00000052583 | ENSMMUG00000052583 | M_mulatta | Animals | mono_intergenic | C/D | | | intergenic |
| ENSMMUG00000052617 | ENSMMUG00000052617 | M_mulatta | Animals | mono_intergenic | C/D | | | intergenic |
| ENSMMUG00000052622 | ENSMMUG00000052622 | M_mulatta | Animals | intergenic_cluster | C/D | | | intergenic |
| ENSMMUG00000052639 | SNORD116 | M_mulatta | Animals | mono_intronic | C/D | ENSMMUG00000054792 | | non_coding |
| ENSMMUG00000052640 | ENSMMUG00000052640 | M_mulatta | Animals | mono_intergenic | C/D | | | intergenic |
| ENSMMUG00000052657 | SNORD57 | M_mulatta | Animals | mono_intronic | C/D | ENSMMUG00000013888 | NOP56 | protein_coding |
| ENSMMUG00000052707 | SNORD116 | M_mulatta | Animals | mono_intronic | C/D | ENSMMUG00000064025 | | non_coding |
| ENSMMUG00000052746 | SNORD115 | M_mulatta | Animals | mono_intergenic | C/D | | | intergenic |
| ENSMMUG00000052748 | ENSMMUG00000052748 | M_mulatta | Animals | mono_exonic | C/D | ENSMMUG00000058662 | | protein_coding |
| ENSMMUG00000052756 | ENSMMUG00000052756 | M_mulatta | Animals | mono_intergenic | C/D | | | intergenic |
| ENSMMUG00000052767 | ENSMMUG00000052767 | M_mulatta | Animals | mono_intronic | C/D | ENSMMUG00000014011 | IGFBP7 | protein_coding |
| ENSMMUG00000052778 | ENSMMUG00000052778 | M_mulatta | Animals | mono_intronic | C/D | ENSMMUG00000023533 | SERINC5 | protein_coding |
| ENSMMUG00000052808 | ENSMMUG00000052808 | M_mulatta | Animals | mono_intronic | C/D | ENSMMUG00000060229 | | non_coding |
| ENSMMUG00000052813 | SNORD115 | M_mulatta | Animals | mono_intergenic | C/D | | | intergenic |
| ENSMMUG00000052815 | ENSMMUG00000052815 | M_mulatta | Animals | mono_intergenic | C/D | | | intergenic |
| ENSMMUG00000052824 | ENSMMUG00000052824 | M_mulatta | Animals | mono_intergenic | C/D | | | intergenic |
| ENSMMUG00000052834 | ENSMMUG00000052834 | M_mulatta | Animals | mono_intronic | C/D | ENSMMUG00000011201 | UBE2D3 | protein_coding |
| ENSMMUG00000052855 | SNORD113-9 | M_mulatta | Animals | mono_intronic | C/D | ENSMMUG00000053357 | | non_coding |
| ENSMMUG00000052861 | ENSMMUG00000052861 | M_mulatta | Animals | mono_intergenic | C/D | | | intergenic |
| ENSMMUG00000052862 | ENSMMUG00000052862 | M_mulatta | Animals | mono_intergenic | C/D | | | intergenic |
| ENSMMUG00000052863 | ENSMMUG00000052863 | M_mulatta | Animals | mono_intronic | C/D | ENSMMUG00000012849 | RNF138 | protein_coding |
| ENSMMUG00000052883 | ENSMMUG00000052883 | M_mulatta | Animals | mono_intergenic | C/D | | | intergenic |
| ENSMMUG00000052898 | ENSMMUG00000052898 | M_mulatta | Animals | mono_intronic | C/D | ENSMMUG00000001027 | ATP5F1B | protein_coding |
| ENSMMUG00000052903 | ENSMMUG00000052903 | M_mulatta | Animals | mono_intergenic | C/D | | | intergenic |
| ENSMMUG00000052916 | SNORD116 | M_mulatta | Animals | mono_intronic | C/D | ENSMMUG00000054792 | | non_coding |
| ENSMMUG00000052947 | ENSMMUG00000052947 | M_mulatta | Animals | mono_intergenic | C/D | | | intergenic |
| ENSMMUG00000052951 | ENSMMUG00000052951 | M_mulatta | Animals | mono_intronic | C/D | ENSMMUG00000010634 | ASB3 | protein_coding |

| | | | | | | | | |
|---|---|---|---|---|---|---|---|---|
| ENSMMUG00000052968 | ENSMMUG00000052968 | M_mulatta | Animals | mono_intergenic | C/D | | | intergenic |
| ENSMMUG00000053019 | U3 | M_mulatta | Animals | mono_intergenic | C/D | | | intergenic |
| ENSMMUG00000053034 | ENSMMUG00000053034 | M_mulatta | Animals | mono_intergenic | C/D | | | intergenic |
| ENSMMUG00000053040 | ENSMMUG00000053040 | M_mulatta | Animals | mono_intronic | C/D | ENSMMUG00000016016 | CCDC25 | protein_coding |
| ENSMMUG00000053066 | ENSMMUG00000053066 | M_mulatta | Animals | mono_intronic | C/D | ENSMMUG00000011209 | BICDL1 | protein_coding |
| ENSMMUG00000053082 | SNORD115 | M_mulatta | Animals | mono_intergenic | C/D | | | intergenic |
| ENSMMUG00000053086 | SNORD114-24 | M_mulatta | Animals | mono_intergenic | C/D | | | intergenic |
| ENSMMUG00000053095 | SNORA71 | M_mulatta | Animals | mono_intergenic | H/ACA | | | intergenic |
| ENSMMUG00000053168 | ENSMMUG00000053168 | M_mulatta | Animals | mono_intergenic | C/D | | | intergenic |
| ENSMMUG00000053197 | SNORA80A | M_mulatta | Animals | mono_intronic | H/ACA | ENSMMUG00000010295 | URB1 | protein_coding |
| ENSMMUG00000053210 | ENSMMUG00000053210 | M_mulatta | Animals | mono_intronic | C/D | ENSMMUG00000020533 | C11H12orf56 | protein_coding |
| ENSMMUG00000053240 | ENSMMUG00000053240 | M_mulatta | Animals | mono_intergenic | C/D | | | intergenic |
| ENSMMUG00000053243 | SNORD114-11 | M_mulatta | Animals | mono_intronic | C/D | ENSMMUG00000063833 | | non_coding |
| ENSMMUG00000053279 | SNORA70 | M_mulatta | Animals | mono_intronic | H/ACA | ENSMMUG00000001924 | TIMM17A | protein_coding |
| ENSMMUG00000053286 | SNORD115 | M_mulatta | Animals | mono_intergenic | C/D | | | intergenic |
| ENSMMUG00000053342 | ENSMMUG00000053342 | M_mulatta | Animals | mono_intronic | C/D | ENSMMUG00000016902 | CTNNA3 | protein_coding |
| ENSMMUG00000053349 | ENSMMUG00000053349 | M_mulatta | Animals | mono_intergenic | C/D | | | intergenic |
| ENSMMUG00000053406 | ENSMMUG00000053406 | M_mulatta | Animals | mono_intronic | C/D | ENSMMUG00000019690 | SESTD1 | protein_coding |
| ENSMMUG00000053417 | SNORD116 | M_mulatta | Animals | mono_intronic | C/D | ENSMMUG00000064025 | | non_coding |
| ENSMMUG00000053446 | SNORD116 | M_mulatta | Animals | mono_intronic | C/D | ENSMMUG00000064025 | | non_coding |
| ENSMMUG00000053461 | ENSMMUG00000053461 | M_mulatta | Animals | mono_intergenic | C/D | | | intergenic |
| ENSMMUG00000053464 | ENSMMUG00000053464 | M_mulatta | Animals | mono_intergenic | C/D | | | intergenic |
| ENSMMUG00000053468 | ENSMMUG00000053468 | M_mulatta | Animals | mono_intergenic | C/D | | | intergenic |
| ENSMMUG00000053469 | ENSMMUG00000053469 | M_mulatta | Animals | mono_intronic | C/D | ENSMMUG00000000416 | OOEP | protein_coding |
| ENSMMUG00000053473 | ENSMMUG00000053473 | M_mulatta | Animals | mono_intronic | C/D | ENSMMUG00000007982 | SMC3 | protein_coding |
| ENSMMUG00000053519 | ENSMMUG00000053519 | M_mulatta | Animals | mono_intergenic | C/D | | | intergenic |
| ENSMMUG00000053566 | SNORD115 | M_mulatta | Animals | mono_intergenic | C/D | | | intergenic |
| ENSMMUG00000053583 | SNORD116 | M_mulatta | Animals | mono_intronic | C/D | ENSMMUG00000064025 | | non_coding |
| ENSMMUG00000053655 | ENSMMUG00000053655 | M_mulatta | Animals | mono_intergenic | C/D | | | intergenic |
| ENSMMUG00000053669 | ENSMMUG00000053669 | M_mulatta | Animals | mono_intronic | C/D | ENSMMUG00000008026 | SPEN | protein_coding |
| ENSMMUG00000053679 | U3 | M_mulatta | Animals | mono_intergenic | C/D | | | intergenic |
| ENSMMUG00000053681 | ENSMMUG00000053681 | M_mulatta | Animals | mono_intergenic | C/D | | | intergenic |
| ENSMMUG00000053695 | U3 | M_mulatta | Animals | mono_intergenic | C/D | | | intergenic |
| ENSMMUG00000053703 | SNORA70 | M_mulatta | Animals | mono_intergenic | H/ACA | | | intergenic |
| ENSMMUG00000053706 | U3 | M_mulatta | Animals | mono_intergenic | C/D | | | intergenic |
| ENSMMUG00000053740 | ENSMMUG00000053740 | M_mulatta | Animals | mono_intergenic | C/D | | | intergenic |
| ENSMMUG00000053775 | ENSMMUG00000053775 | M_mulatta | Animals | mono_intergenic | C/D | | | intergenic |
| ENSMMUG00000053777 | ENSMMUG00000053777 | M_mulatta | Animals | mono_exonic | C/D | ENSMMUG00000019684 | NQO1 | protein_coding |
| ENSMMUG00000053815 | U3 | M_mulatta | Animals | mono_intergenic | C/D | | | intergenic |
| ENSMMUG00000053820 | ENSMMUG00000053820 | M_mulatta | Animals | mono_intergenic | C/D | | | intergenic |
| ENSMMUG00000053821 | ENSMMUG00000053821 | M_mulatta | Animals | mono_intergenic | C/D | | | intergenic |
| ENSMMUG00000053822 | ENSMMUG00000053822 | M_mulatta | Animals | mono_intronic | C/D | ENSMMUG00000023598 | SNX2 | protein_coding |
| ENSMMUG00000053833 | SNORD115 | M_mulatta | Animals | mono_intergenic | C/D | | | intergenic |
| ENSMMUG00000053839 | U3 | M_mulatta | Animals | mono_intergenic | C/D | | | intergenic |
| ENSMMUG00000053848 | ENSMMUG00000053848 | M_mulatta | Animals | mono_intergenic | C/D | | | intergenic |

| | | | | | | | | |
|---|---|---|---|---|---|---|---|---|
| ENSMMUG00000053854 | ENSMMUG00000053854 | M_mulatta | Animals | mono_intronic | C/D | ENSMMUG00000063429 | IPMK | protein_coding |
| ENSMMUG00000053887 | U3 | M_mulatta | Animals | mono_intergenic | C/D | | | intergenic |
| ENSMMUG00000053894 | ENSMMUG00000053894 | M_mulatta | Animals | mono_intergenic | C/D | | | intergenic |
| ENSMMUG00000053902 | SNORD114-22 | M_mulatta | Animals | mono_intronic | C/D | ENSMMUG00000063833 | | non_coding |
| ENSMMUG00000053922 | ENSMMUG00000053922 | M_mulatta | Animals | intronic_cluster | C/D | ENSMMUG00000053357 | | non_coding |
| ENSMMUG00000053954 | ENSMMUG00000053954 | M_mulatta | Animals | mono_intronic | C/D | ENSMMUG00000038299 | PNKD | protein_coding |
| ENSMMUG00000053968 | ENSMMUG00000053968 | M_mulatta | Animals | mono_intronic | C/D | ENSMMUG00000021385 | CA14 | protein_coding |
| ENSMMUG00000053982 | ENSMMUG00000053982 | M_mulatta | Animals | mono_intergenic | C/D | | | intergenic |
| ENSMMUG00000054022 | SNORD13D | M_mulatta | Animals | mono_intergenic | C/D | | | intergenic |
| ENSMMUG00000054117 | SNORD115 | M_mulatta | Animals | mono_intergenic | C/D | | | intergenic |
| ENSMMUG00000054134 | ENSMMUG00000054134 | M_mulatta | Animals | mono_intronic | C/D | ENSMMUG00000015668 | CAPZA1 | protein_coding |
| ENSMMUG00000054141 | ENSMMUG00000054141 | M_mulatta | Animals | mono_intergenic | C/D | | | intergenic |
| ENSMMUG00000054148 | U3 | M_mulatta | Animals | mono_intergenic | C/D | | | intergenic |
| ENSMMUG00000054215 | SNORD115 | M_mulatta | Animals | mono_intergenic | C/D | | | intergenic |
| ENSMMUG00000054224 | ENSMMUG00000054224 | M_mulatta | Animals | mono_intronic | C/D | ENSMMUG00000022282 | COL4A2 | protein_coding |
| ENSMMUG00000054227 | ENSMMUG00000054227 | M_mulatta | Animals | mono_intronic | C/D | ENSMMUG00000022006 | TMEM217 | protein_coding |
| ENSMMUG00000054229 | ENSMMUG00000054229 | M_mulatta | Animals | mono_intronic | C/D | ENSMMUG00000059194 | BCL2 | protein_coding |
| ENSMMUG00000054232 | U3 | M_mulatta | Animals | mono_intergenic | C/D | | | intergenic |
| ENSMMUG00000054240 | ENSMMUG00000054240 | M_mulatta | Animals | mono_intergenic | C/D | | | intergenic |
| ENSMMUG00000054242 | ENSMMUG00000054242 | M_mulatta | Animals | mono_intergenic | C/D | | | intergenic |
| ENSMMUG00000054250 | SNORD116 | M_mulatta | Animals | mono_intergenic | C/D | | | intergenic |
| ENSMMUG00000054285 | ENSMMUG00000054285 | M_mulatta | Animals | mono_intronic | C/D | ENSMMUG00000017507 | ADAM10 | protein_coding |
| ENSMMUG00000054305 | ENSMMUG00000054305 | M_mulatta | Animals | mono_intergenic | C/D | | | intergenic |
| ENSMMUG00000054311 | ENSMMUG00000054311 | M_mulatta | Animals | mono_intergenic | C/D | | | intergenic |
| ENSMMUG00000054318 | ENSMMUG00000054318 | M_mulatta | Animals | mono_intergenic | C/D | | | intergenic |
| ENSMMUG00000054320 | SNORD115 | M_mulatta | Animals | mono_intergenic | C/D | | | intergenic |
| ENSMMUG00000054333 | ENSMMUG00000054333 | M_mulatta | Animals | mono_intergenic | C/D | | | intergenic |
| ENSMMUG00000054346 | ENSMMUG00000054346 | M_mulatta | Animals | mono_intergenic | C/D | | | intergenic |
| ENSMMUG00000054347 | ENSMMUG00000054347 | M_mulatta | Animals | mono_intronic | C/D | ENSMMUG00000007342 | CSMD3 | protein_coding |
| ENSMMUG00000054354 | ENSMMUG00000054354 | M_mulatta | Animals | mono_intronic | H/ACA | ENSMMUG00000021120 | MAGED1 | protein_coding |
| ENSMMUG00000054364 | U3 | M_mulatta | Animals | mono_intronic | C/D | ENSMMUG00000017626 | C14H11orf58 | protein_coding |
| ENSMMUG00000054375 | ENSMMUG00000054375 | M_mulatta | Animals | mono_intergenic | C/D | | | intergenic |
| ENSMMUG00000054378 | ENSMMUG00000054378 | M_mulatta | Animals | mono_intronic | C/D | ENSMMUG00000002206 | PMP22 | protein_coding |
| ENSMMUG00000054388 | ENSMMUG00000054388 | M_mulatta | Animals | mono_intergenic | C/D | | | intergenic |
| ENSMMUG00000054415 | ENSMMUG00000054415 | M_mulatta | Animals | mono_intronic | C/D | ENSMMUG00000019377 | CPA4 | protein_coding |
| ENSMMUG00000054456 | SNORD115 | M_mulatta | Animals | mono_intergenic | C/D | | | intergenic |
| ENSMMUG00000054470 | ENSMMUG00000054470 | M_mulatta | Animals | mono_intronic | C/D | ENSMMUG00000012921 | ADAM17 | protein_coding |
| ENSMMUG00000054482 | ENSMMUG00000054482 | M_mulatta | Animals | mono_intergenic | C/D | | | intergenic |
| ENSMMUG00000054553 | ENSMMUG00000054553 | M_mulatta | Animals | mono_intronic | C/D | ENSMMUG00000003265 | LEPR | protein_coding |
| ENSMMUG00000054555 | ENSMMUG00000054555 | M_mulatta | Animals | mono_intronic | C/D | ENSMMUG00000002132 | PSMD12 | protein_coding |
| ENSMMUG00000054557 | ENSMMUG00000054557 | M_mulatta | Animals | mono_intronic | C/D | ENSMMUG00000014008 | POLR2B | protein_coding |
| ENSMMUG00000054582 | SNORD114-29 | M_mulatta | Animals | mono_intronic | C/D | ENSMMUG00000063833 | | non_coding |
| ENSMMUG00000054642 | ENSMMUG00000054642 | M_mulatta | Animals | mono_intergenic | C/D | | | intergenic |
| ENSMMUG00000054644 | SNORD116 | M_mulatta | Animals | mono_intronic | C/D | ENSMMUG00000064025 | | non_coding |
| ENSMMUG00000054648 | ENSMMUG00000054648 | M_mulatta | Animals | mono_intergenic | C/D | | | intergenic |

| | | | | | | | | |
|---|---|---|---|---|---|---|---|---|
| ENSMMUG00000054651 | ENSMMUG00000054651 | M_mulatta | Animals | mono_intronic | C/D | ENSMMUG00000017810 | SSRP1 | protein_coding |
| ENSMMUG00000054654 | ENSMMUG00000054654 | M_mulatta | Animals | intergenic_cluster | C/D | | | intergenic |
| ENSMMUG00000054685 | ENSMMUG00000054685 | M_mulatta | Animals | mono_intergenic | C/D | | | intergenic |
| ENSMMUG00000054698 | ENSMMUG00000054698 | M_mulatta | Animals | mono_intergenic | C/D | | | intergenic |
| ENSMMUG00000054717 | SNORD116 | M_mulatta | Animals | mono_intronic | C/D | ENSMMUG00000054792 | | non_coding |
| ENSMMUG00000054738 | ENSMMUG00000054738 | M_mulatta | Animals | mono_intronic | C/D | ENSMMUG00000012387 | MAP3K1 | protein_coding |
| ENSMMUG00000054746 | ENSMMUG00000054746 | M_mulatta | Animals | mono_intergenic | C/D | | | intergenic |
| ENSMMUG00000054753 | U3 | M_mulatta | Animals | mono_intronic | C/D | ENSMMUG00000000644 | CEP85 | protein_coding |
| ENSMMUG00000054775 | ENSMMUG00000054775 | M_mulatta | Animals | mono_intronic | C/D | ENSMMUG00000017605 | MRPL39 | protein_coding |
| ENSMMUG00000054783 | ENSMMUG00000054783 | M_mulatta | Animals | mono_intergenic | C/D | | | intergenic |
| ENSMMUG00000054837 | ENSMMUG00000054837 | M_mulatta | Animals | mono_intergenic | C/D | | | intergenic |
| ENSMMUG00000054842 | U3 | M_mulatta | Animals | mono_intergenic | C/D | | | intergenic |
| ENSMMUG00000054883 | ENSMMUG00000054883 | M_mulatta | Animals | mono_intergenic | C/D | | | intergenic |
| ENSMMUG00000054905 | SNORD115 | M_mulatta | Animals | mono_intergenic | C/D | | | intergenic |
| ENSMMUG00000054995 | ENSMMUG00000054995 | M_mulatta | Animals | mono_intergenic | C/D | | | intergenic |
| ENSMMUG00000055001 | ENSMMUG00000055001 | M_mulatta | Animals | mono_intergenic | C/D | | | intergenic |
| ENSMMUG00000055014 | ENSMMUG00000055014 | M_mulatta | Animals | mono_intergenic | C/D | | | intergenic |
| ENSMMUG00000055043 | ENSMMUG00000055043 | M_mulatta | Animals | mono_intergenic | C/D | | | intergenic |
| ENSMMUG00000055050 | ENSMMUG00000055050 | M_mulatta | Animals | mono_intronic | C/D | ENSMMUG00000015870 | SLC37A3 | protein_coding |
| ENSMMUG00000055078 | U3 | M_mulatta | Animals | mono_intergenic | C/D | | | intergenic |
| ENSMMUG00000055086 | ENSMMUG00000055086 | M_mulatta | Animals | mono_intergenic | C/D | | | intergenic |
| ENSMMUG00000055127 | U3 | M_mulatta | Animals | mono_intergenic | C/D | | | intergenic |
| ENSMMUG00000055152 | ENSMMUG00000055152 | M_mulatta | Animals | mono_intergenic | C/D | | | intergenic |
| ENSMMUG00000055153 | U3 | M_mulatta | Animals | mono_intergenic | C/D | | | intergenic |
| ENSMMUG00000055172 | ENSMMUG00000055172 | M_mulatta | Animals | mono_intergenic | C/D | | | intergenic |
| ENSMMUG00000055177 | ENSMMUG00000055177 | M_mulatta | Animals | mono_intergenic | C/D | | | intergenic |
| ENSMMUG00000055183 | ENSMMUG00000055183 | M_mulatta | Animals | mono_intergenic | C/D | | | intergenic |
| ENSMMUG00000055196 | ENSMMUG00000055196 | M_mulatta | Animals | mono_intergenic | C/D | | | intergenic |
| ENSMMUG00000055200 | ENSMMUG00000055200 | M_mulatta | Animals | mono_intergenic | C/D | | | intergenic |
| ENSMMUG00000055202 | U3 | M_mulatta | Animals | mono_intergenic | C/D | | | intergenic |
| ENSMMUG00000055205 | U3 | M_mulatta | Animals | intergenic_cluster | C/D | | | intergenic |
| ENSMMUG00000055222 | SNORD114-19 | M_mulatta | Animals | intronic_cluster | C/D | ENSMMUG00000063833 | | non_coding |
| ENSMMUG00000055253 | ENSMMUG00000055253 | M_mulatta | Animals | mono_intergenic | C/D | | | intergenic |
| ENSMMUG00000055267 | ENSMMUG00000055267 | M_mulatta | Animals | mono_intronic | C/D | ENSMMUG00000022110 | PPP1R14C | protein_coding |
| ENSMMUG00000055288 | ENSMMUG00000055288 | M_mulatta | Animals | mono_intergenic | C/D | | | intergenic |
| ENSMMUG00000055325 | ENSMMUG00000055325 | M_mulatta | Animals | mono_intergenic | C/D | | | intergenic |
| ENSMMUG00000055374 | ENSMMUG00000055374 | M_mulatta | Animals | mono_intergenic | C/D | | | intergenic |
| ENSMMUG00000055429 | SNORD115 | M_mulatta | Animals | mono_intergenic | C/D | | | intergenic |
| ENSMMUG00000055431 | ENSMMUG00000055431 | M_mulatta | Animals | mono_intergenic | C/D | | | intergenic |
| ENSMMUG00000055480 | ENSMMUG00000055480 | M_mulatta | Animals | mono_intergenic | C/D | | | intergenic |
| ENSMMUG00000055516 | ENSMMUG00000055516 | M_mulatta | Animals | mono_intronic | C/D | ENSMMUG00000005610 | PTPN4 | protein_coding |
| ENSMMUG00000055521 | ENSMMUG00000055521 | M_mulatta | Animals | mono_intergenic | H/ACA | | | intergenic |
| ENSMMUG00000055528 | ENSMMUG00000055528 | M_mulatta | Animals | mono_intronic | C/D | ENSMMUG00000017561 | SLC43A2 | protein_coding |
| ENSMMUG00000055545 | ENSMMUG00000055545 | M_mulatta | Animals | mono_intronic | C/D | ENSMMUG00000031755 | GLDC | protein_coding |
| ENSMMUG00000055558 | SNORD116 | M_mulatta | Animals | mono_intronic | C/D | ENSMMUG00000064025 | | non_coding |

| | | | | | | | | |
|---|---|---|---|---|---|---|---|---|
| ENSMMUG00000055577 | ENSMMUG00000055577 | M_mulatta | Animals | mono_intronic | C/D | ENSMMUG00000012563 | APBB2 | protein_coding |
| ENSMMUG00000055648 | ENSMMUG00000055648 | M_mulatta | Animals | mono_intergenic | C/D | | | intergenic |
| ENSMMUG00000055661 | ENSMMUG00000055661 | M_mulatta | Animals | mono_intergenic | C/D | | | intergenic |
| ENSMMUG00000055685 | ENSMMUG00000055685 | M_mulatta | Animals | mono_intergenic | C/D | | | intergenic |
| ENSMMUG00000055695 | ENSMMUG00000055695 | M_mulatta | Animals | mono_intergenic | H/ACA | | | intergenic |
| ENSMMUG00000055706 | ENSMMUG00000055706 | M_mulatta | Animals | mono_intergenic | C/D | | | intergenic |
| ENSMMUG00000055730 | SNORD115 | M_mulatta | Animals | mono_intergenic | C/D | | | intergenic |
| ENSMMUG00000055758 | ENSMMUG00000055758 | M_mulatta | Animals | mono_intronic | C/D | ENSMMUG00000002328 | PRKCSH | protein_coding |
| ENSMMUG00000055770 | ENSMMUG00000055770 | M_mulatta | Animals | mono_intronic | C/D | ENSMMUG00000001883 | KIAA2012 | protein_coding |
| ENSMMUG00000055777 | SNORD115 | M_mulatta | Animals | mono_intergenic | C/D | | | intergenic |
| ENSMMUG00000055847 | ENSMMUG00000055847 | M_mulatta | Animals | mono_intergenic | C/D | | | intergenic |
| ENSMMUG00000055879 | U3 | M_mulatta | Animals | mono_intergenic | C/D | | | intergenic |
| ENSMMUG00000055888 | ENSMMUG00000055888 | M_mulatta | Animals | mono_intergenic | C/D | | | intergenic |
| ENSMMUG00000055911 | ENSMMUG00000055911 | M_mulatta | Animals | mono_intergenic | C/D | | | intergenic |
| ENSMMUG00000055931 | ENSMMUG00000055931 | M_mulatta | Animals | mono_intergenic | C/D | | | intergenic |
| ENSMMUG00000055951 | SNORD115 | M_mulatta | Animals | mono_intergenic | C/D | | | intergenic |
| ENSMMUG00000055967 | ENSMMUG00000055967 | M_mulatta | Animals | mono_intronic | C/D | ENSMMUG00000005684 | TNPO2 | protein_coding |
| ENSMMUG00000055995 | ENSMMUG00000055995 | M_mulatta | Animals | mono_intergenic | C/D | | | intergenic |
| ENSMMUG00000056034 | ENSMMUG00000056034 | M_mulatta | Animals | mono_intergenic | C/D | | | intergenic |
| ENSMMUG00000056055 | ENSMMUG00000056055 | M_mulatta | Animals | mono_intergenic | C/D | | | intergenic |
| ENSMMUG00000056108 | ENSMMUG00000056108 | M_mulatta | Animals | mono_intronic | C/D | ENSMMUG00000000750 | RFFL | protein_coding |
| ENSMMUG00000056111 | SNORD115 | M_mulatta | Animals | mono_intergenic | C/D | | | intergenic |
| ENSMMUG00000056133 | ENSMMUG00000056133 | M_mulatta | Animals | mono_intergenic | C/D | | | intergenic |
| ENSMMUG00000056153 | ENSMMUG00000056153 | M_mulatta | Animals | mono_intergenic | C/D | | | intergenic |
| ENSMMUG00000056195 | U3 | M_mulatta | Animals | mono_intergenic | C/D | | | intergenic |
| ENSMMUG00000056201 | ENSMMUG00000056201 | M_mulatta | Animals | mono_intronic | C/D | ENSMMUG00000000031 | TMEM132C | protein_coding |
| ENSMMUG00000056350 | ENSMMUG00000056350 | M_mulatta | Animals | mono_intergenic | C/D | | | intergenic |
| ENSMMUG00000056377 | ENSMMUG00000056377 | M_mulatta | Animals | mono_intronic | C/D | ENSMMUG00000008140 | SGK1 | protein_coding |
| ENSMMUG00000056389 | ENSMMUG00000056389 | M_mulatta | Animals | mono_intergenic | C/D | | | intergenic |
| ENSMMUG00000056412 | ENSMMUG00000056412 | M_mulatta | Animals | mono_intronic | C/D | ENSMMUG00000020766 | UBR5 | protein_coding |
| ENSMMUG00000056424 | ENSMMUG00000056424 | M_mulatta | Animals | mono_intergenic | C/D | | | intergenic |
| ENSMMUG00000056442 | ENSMMUG00000056442 | M_mulatta | Animals | mono_intergenic | C/D | | | intergenic |
| ENSMMUG00000056483 | ENSMMUG00000056483 | M_mulatta | Animals | mono_intergenic | C/D | | | intergenic |
| ENSMMUG00000056524 | U3 | M_mulatta | Animals | mono_intergenic | C/D | | | intergenic |
| ENSMMUG00000056552 | ENSMMUG00000056552 | M_mulatta | Animals | mono_intronic | C/D | ENSMMUG00000021727 | YWHAH | protein_coding |
| ENSMMUG00000056583 | ENSMMUG00000056583 | M_mulatta | Animals | mono_intergenic | C/D | | | intergenic |
| ENSMMUG00000056584 | ENSMMUG00000056584 | M_mulatta | Animals | mono_intergenic | C/D | | | intergenic |
| ENSMMUG00000056593 | ENSMMUG00000056593 | M_mulatta | Animals | mono_intergenic | C/D | | | intergenic |
| ENSMMUG00000056643 | ENSMMUG00000056643 | M_mulatta | Animals | mono_intronic | C/D | ENSMMUG00000020412 | PACRG | protein_coding |
| ENSMMUG00000056667 | ENSMMUG00000056667 | M_mulatta | Animals | mono_intergenic | C/D | | | intergenic |
| ENSMMUG00000056721 | ENSMMUG00000056721 | M_mulatta | Animals | mono_intergenic | C/D | | | intergenic |
| ENSMMUG00000056724 | ENSMMUG00000056724 | M_mulatta | Animals | mono_intergenic | C/D | | | intergenic |
| ENSMMUG00000056726 | ENSMMUG00000056726 | M_mulatta | Animals | mono_intergenic | C/D | | | intergenic |
| ENSMMUG00000056762 | ENSMMUG00000056762 | M_mulatta | Animals | mono_intronic | C/D | ENSMMUG00000049523 | ANKRD12 | protein_coding |
| ENSMMUG00000056763 | ENSMMUG00000056763 | M_mulatta | Animals | mono_intronic | C/D | ENSMMUG00000001768 | ZNF616 | protein_coding |

| | | | | | | | | |
|---|---|---|---|---|---|---|---|---|
| ENSMMUG00000056819 | SNORD115 | M_mulatta | Animals | mono_intergenic | C/D | | | intergenic |
| ENSMMUG00000056830 | ENSMMUG00000056830 | M_mulatta | Animals | mono_intergenic | C/D | | | intergenic |
| ENSMMUG00000056857 | ENSMMUG00000056857 | M_mulatta | Animals | mono_intergenic | C/D | | | intergenic |
| ENSMMUG00000056932 | ENSMMUG00000056932 | M_mulatta | Animals | mono_intergenic | C/D | | | intergenic |
| ENSMMUG00000056943 | SNORD114-12 | M_mulatta | Animals | mono_intronic | C/D | ENSMMUG00000063833 | | non_coding |
| ENSMMUG00000057026 | SNORA80C | M_mulatta | Animals | mono_intergenic | H/ACA | | | intergenic |
| ENSMMUG00000057047 | SNORD115 | M_mulatta | Animals | mono_intergenic | C/D | | | intergenic |
| ENSMMUG00000057068 | ENSMMUG00000057068 | M_mulatta | Animals | mono_intergenic | C/D | | | intergenic |
| ENSMMUG00000057069 | SNORA15 | M_mulatta | Animals | mono_intronic | H/ACA | ENSMMUG00000015272 | CCT6A | protein_coding |
| ENSMMUG00000057075 | ENSMMUG00000057075 | M_mulatta | Animals | mono_intronic | C/D | ENSMMUG00000060229 | | non_coding |
| ENSMMUG00000057078 | ENSMMUG00000057078 | M_mulatta | Animals | mono_intronic | C/D | ENSMMUG00000063791 | | non_coding |
| ENSMMUG00000057133 | ENSMMUG00000057133 | M_mulatta | Animals | mono_intergenic | C/D | | | intergenic |
| ENSMMUG00000057175 | ENSMMUG00000057175 | M_mulatta | Animals | mono_intergenic | C/D | | | intergenic |
| ENSMMUG00000057218 | SNORD114-1 | M_mulatta | Animals | intronic_cluster | C/D | ENSMMUG00000053357 | | non_coding |
| ENSMMUG00000057232 | ENSMMUG00000057232 | M_mulatta | Animals | mono_intergenic | C/D | | | intergenic |
| ENSMMUG00000057238 | ENSMMUG00000057238 | M_mulatta | Animals | mono_intergenic | C/D | | | intergenic |
| ENSMMUG00000057241 | ENSMMUG00000057241 | M_mulatta | Animals | mono_intergenic | C/D | | | intergenic |
| ENSMMUG00000057260 | ENSMMUG00000057260 | M_mulatta | Animals | mono_intergenic | C/D | | | intergenic |
| ENSMMUG00000057300 | ENSMMUG00000057300 | M_mulatta | Animals | mono_intergenic | C/D | | | intergenic |
| ENSMMUG00000057334 | ENSMMUG00000057334 | M_mulatta | Animals | mono_intergenic | C/D | | | intergenic |
| ENSMMUG00000057352 | ENSMMUG00000057352 | M_mulatta | Animals | mono_intergenic | C/D | | | intergenic |
| ENSMMUG00000057362 | ENSMMUG00000057362 | M_mulatta | Animals | mono_intergenic | H/ACA | | | intergenic |
| ENSMMUG00000057405 | ENSMMUG00000057405 | M_mulatta | Animals | mono_intronic | C/D | ENSMMUG00000062269 | | protein_coding |
| ENSMMUG00000057410 | ENSMMUG00000057410 | M_mulatta | Animals | mono_intronic | C/D | ENSMMUG00000018338 | RCC1 | protein_coding |
| ENSMMUG00000057440 | ENSMMUG00000057440 | M_mulatta | Animals | mono_intronic | C/D | ENSMMUG00000001861 | NFE2L2 | protein_coding |
| ENSMMUG00000057460 | ENSMMUG00000057460 | M_mulatta | Animals | mono_intronic | C/D | ENSMMUG00000038966 | EIF2AK4 | protein_coding |
| ENSMMUG00000057490 | ENSMMUG00000057490 | M_mulatta | Animals | mono_intergenic | C/D | | | intergenic |
| ENSMMUG00000057504 | ENSMMUG00000057504 | M_mulatta | Animals | mono_intergenic | C/D | | | intergenic |
| ENSMMUG00000057518 | ENSMMUG00000057518 | M_mulatta | Animals | mono_intronic | C/D | ENSMMUG00000008112 | NEDD4 | protein_coding |
| ENSMMUG00000057537 | ENSMMUG00000057537 | M_mulatta | Animals | mono_intergenic | C/D | | | intergenic |
| ENSMMUG00000057628 | SNORD88C | M_mulatta | Animals | mono_intronic | C/D | ENSMMUG00000051878 | | protein_coding |
| ENSMMUG00000057667 | ENSMMUG00000057667 | M_mulatta | Animals | mono_intronic | C/D | ENSMMUG00000014333 | PRPF38A | protein_coding |
| ENSMMUG00000057685 | SNORD39 | M_mulatta | Animals | mono_intergenic | C/D | | | intergenic |
| ENSMMUG00000057695 | SNORD114-20 | M_mulatta | Animals | mono_intronic | C/D | ENSMMUG00000063833 | | non_coding |
| ENSMMUG00000057721 | ENSMMUG00000057721 | M_mulatta | Animals | mono_intergenic | C/D | | | intergenic |
| ENSMMUG00000057722 | U3 | M_mulatta | Animals | mono_intergenic | C/D | | | intergenic |
| ENSMMUG00000057750 | ENSMMUG00000057750 | M_mulatta | Animals | mono_intergenic | C/D | | | intergenic |
| ENSMMUG00000057755 | ENSMMUG00000057755 | M_mulatta | Animals | mono_intergenic | C/D | | | intergenic |
| ENSMMUG00000057783 | SNORD115 | M_mulatta | Animals | mono_intergenic | C/D | | | intergenic |
| ENSMMUG00000057797 | ENSMMUG00000057797 | M_mulatta | Animals | mono_intergenic | C/D | | | intergenic |
| ENSMMUG00000057824 | U3 | M_mulatta | Animals | mono_intergenic | C/D | | | intergenic |
| ENSMMUG00000057848 | ENSMMUG00000057848 | M_mulatta | Animals | mono_intergenic | C/D | | | intergenic |
| ENSMMUG00000057862 | ENSMMUG00000057862 | M_mulatta | Animals | mono_intronic | C/D | ENSMMUG00000060229 | | non_coding |
| ENSMMUG00000057877 | ENSMMUG00000057877 | M_mulatta | Animals | mono_intergenic | C/D | | | intergenic |
| ENSMMUG00000057891 | ENSMMUG00000057891 | M_mulatta | Animals | intergenic_cluster | C/D | | | intergenic |

| | | | | | | | | |
|---|---|---|---|---|---|---|---|---|
| ENSMMUG00000057902 | ENSMMUG00000057902 | M_mulatta | Animals | mono_intronic | C/D | ENSMMUG00000049347 | | non_coding |
| ENSMMUG00000057920 | ENSMMUG00000057920 | M_mulatta | Animals | mono_intergenic | C/D | | | intergenic |
| ENSMMUG00000057955 | U3 | M_mulatta | Animals | mono_intergenic | C/D | | | intergenic |
| ENSMMUG00000057967 | ENSMMUG00000057967 | M_mulatta | Animals | mono_intronic | C/D | ENSMMUG00000019345 | DMD | protein_coding |
| ENSMMUG00000057974 | ENSMMUG00000057974 | M_mulatta | Animals | mono_intergenic | C/D | | | intergenic |
| ENSMMUG00000057975 | ENSMMUG00000057975 | M_mulatta | Animals | mono_intergenic | C/D | | | intergenic |
| ENSMMUG00000057992 | ENSMMUG00000057992 | M_mulatta | Animals | mono_intergenic | C/D | | | intergenic |
| ENSMMUG00000057998 | ENSMMUG00000057998 | M_mulatta | Animals | mono_intergenic | C/D | | | intergenic |
| ENSMMUG00000058012 | SNORD114-17 | M_mulatta | Animals | mono_intronic | C/D | ENSMMUG00000063833 | | non_coding |
| ENSMMUG00000058045 | ENSMMUG00000058045 | M_mulatta | Animals | mono_intergenic | C/D | | | intergenic |
| ENSMMUG00000058048 | ENSMMUG00000058048 | M_mulatta | Animals | mono_intergenic | C/D | | | intergenic |
| ENSMMUG00000058087 | U3 | M_mulatta | Animals | mono_intergenic | C/D | | | intergenic |
| ENSMMUG00000058104 | ENSMMUG00000058104 | M_mulatta | Animals | mono_intergenic | C/D | | | intergenic |
| ENSMMUG00000058196 | ENSMMUG00000058196 | M_mulatta | Animals | mono_intergenic | C/D | | | intergenic |
| ENSMMUG00000058199 | ENSMMUG00000058199 | M_mulatta | Animals | mono_intergenic | H/ACA | | | intergenic |
| ENSMMUG00000058203 | ENSMMUG00000058203 | M_mulatta | Animals | mono_intergenic | C/D | | | intergenic |
| ENSMMUG00000058207 | ENSMMUG00000058207 | M_mulatta | Animals | mono_intergenic | C/D | | | intergenic |
| ENSMMUG00000058272 | SNORD115 | M_mulatta | Animals | mono_intergenic | C/D | | | intergenic |
| ENSMMUG00000058284 | SNORD114-14 | M_mulatta | Animals | mono_intronic | C/D | ENSMMUG00000063833 | | non_coding |
| ENSMMUG00000058293 | ENSMMUG00000058293 | M_mulatta | Animals | mono_intergenic | C/D | | | intergenic |
| ENSMMUG00000058298 | ENSMMUG00000058298 | M_mulatta | Animals | mono_intergenic | C/D | | | intergenic |
| ENSMMUG00000058301 | SNORD115 | M_mulatta | Animals | mono_intergenic | C/D | | | intergenic |
| ENSMMUG00000058309 | ENSMMUG00000058309 | M_mulatta | Animals | mono_intronic | C/D | ENSMMUG00000011227 | FOXK1 | protein_coding |
| ENSMMUG00000058347 | ENSMMUG00000058347 | M_mulatta | Animals | mono_intergenic | C/D | | | intergenic |
| ENSMMUG00000058355 | ENSMMUG00000058355 | M_mulatta | Animals | mono_intronic | C/D | ENSMMUG00000005627 | HECW2 | protein_coding |
| ENSMMUG00000058366 | U3 | M_mulatta | Animals | mono_intronic | C/D | ENSMMUG00000019967 | KLHL1 | protein_coding |
| ENSMMUG00000058371 | ENSMMUG00000058371 | M_mulatta | Animals | mono_intronic | C/D | ENSMMUG00000002573 | CARMIL1 | protein_coding |
| ENSMMUG00000058390 | SNORD114-26 | M_mulatta | Animals | mono_intronic | C/D | ENSMMUG00000063833 | | non_coding |
| ENSMMUG00000058451 | ENSMMUG00000058451 | M_mulatta | Animals | mono_intronic | C/D | ENSMMUG00000011252 | MICU2 | protein_coding |
| ENSMMUG00000058492 | ENSMMUG00000058492 | M_mulatta | Animals | mono_intergenic | C/D | | | intergenic |
| ENSMMUG00000058526 | SNORD115 | M_mulatta | Animals | mono_intergenic | C/D | | | intergenic |
| ENSMMUG00000058528 | ENSMMUG00000058528 | M_mulatta | Animals | mono_intronic | C/D | ENSMMUG00000017139 | SEC61A2 | protein_coding |
| ENSMMUG00000058570 | SNORD114-10 | M_mulatta | Animals | mono_intergenic | C/D | | | intergenic |
| ENSMMUG00000058576 | ENSMMUG00000058576 | M_mulatta | Animals | mono_intronic | H/ACA | ENSMMUG00000015151 | IL2RA | protein_coding |
| ENSMMUG00000058591 | ENSMMUG00000058591 | M_mulatta | Animals | mono_intergenic | C/D | | | intergenic |
| ENSMMUG00000058601 | ENSMMUG00000058601 | M_mulatta | Animals | mono_intergenic | H/ACA | | | intergenic |
| ENSMMUG00000058624 | ENSMMUG00000058624 | M_mulatta | Animals | mono_intronic | C/D | ENSMMUG00000040869 | SPATA16 | protein_coding |
| ENSMMUG00000058636 | U3 | M_mulatta | Animals | mono_intergenic | C/D | | | intergenic |
| ENSMMUG00000058666 | ENSMMUG00000058666 | M_mulatta | Animals | mono_intergenic | C/D | | | intergenic |
| ENSMMUG00000058667 | ENSMMUG00000058667 | M_mulatta | Animals | mono_intergenic | C/D | | | intergenic |
| ENSMMUG00000058674 | ENSMMUG00000058674 | M_mulatta | Animals | mono_intergenic | C/D | | | intergenic |
| ENSMMUG00000058686 | ENSMMUG00000058686 | M_mulatta | Animals | mono_intronic | C/D | ENSMMUG00000008299 | FANCC | protein_coding |
| ENSMMUG00000058744 | SNORD115 | M_mulatta | Animals | mono_intergenic | C/D | | | intergenic |
| ENSMMUG00000058756 | SNORD115 | M_mulatta | Animals | mono_intergenic | C/D | | | intergenic |
| ENSMMUG00000058778 | SNORD115 | M_mulatta | Animals | mono_intergenic | C/D | | | intergenic |

| | | | | | | | | |
|---|---|---|---|---|---|---|---|---|
| ENSMMUG00000058780 | ENSMMUG00000058780 | M_mulatta | Animals | mono_intergenic | C/D | | | intergenic |
| ENSMMUG00000058797 | ENSMMUG00000058797 | M_mulatta | Animals | mono_intergenic | C/D | | | intergenic |
| ENSMMUG00000058826 | ENSMMUG00000058826 | M_mulatta | Animals | mono_intergenic | C/D | | | intergenic |
| ENSMMUG00000058870 | ENSMMUG00000058870 | M_mulatta | Animals | mono_intergenic | C/D | | | intergenic |
| ENSMMUG00000058888 | ENSMMUG00000058888 | M_mulatta | Animals | mono_intronic | C/D | ENSMMUG00000008771 | KDM2A | protein_coding |
| ENSMMUG00000058916 | ENSMMUG00000058916 | M_mulatta | Animals | mono_intergenic | C/D | | | intergenic |
| ENSMMUG00000058982 | ENSMMUG00000058982 | M_mulatta | Animals | mono_intergenic | C/D | | | intergenic |
| ENSMMUG00000058991 | ENSMMUG00000058991 | M_mulatta | Animals | mono_intergenic | C/D | | | intergenic |
| ENSMMUG00000059042 | SNORD88B | M_mulatta | Animals | mono_intronic | C/D | ENSMMUG00000051878 | | protein_coding |
| ENSMMUG00000059047 | ENSMMUG00000059047 | M_mulatta | Animals | mono_intergenic | C/D | | | intergenic |
| ENSMMUG00000059079 | ENSMMUG00000059079 | M_mulatta | Animals | mono_intronic | C/D | ENSMMUG00000005032 | MYO6 | protein_coding |
| ENSMMUG00000059098 | SNORD62 | M_mulatta | Animals | mono_intronic | C/D | ENSMMUG00000009973 | PRRC2B | protein_coding |
| ENSMMUG00000059109 | ENSMMUG00000059109 | M_mulatta | Animals | mono_exonic | C/D | ENSMMUG00000060229 | | non_coding |
| ENSMMUG00000059149 | ENSMMUG00000059149 | M_mulatta | Animals | mono_intergenic | C/D | | | intergenic |
| ENSMMUG00000059163 | U3 | M_mulatta | Animals | mono_intronic | C/D | ENSMMUG00000005575 | TRERF1 | protein_coding |
| ENSMMUG00000059212 | SNORD115 | M_mulatta | Animals | mono_intergenic | C/D | | | intergenic |
| ENSMMUG00000059230 | SNORD115 | M_mulatta | Animals | mono_intergenic | C/D | | | intergenic |
| ENSMMUG00000059252 | SNORD115 | M_mulatta | Animals | mono_intergenic | C/D | | | intergenic |
| ENSMMUG00000059264 | ENSMMUG00000059264 | M_mulatta | Animals | mono_intergenic | C/D | | | intergenic |
| ENSMMUG00000059291 | ENSMMUG00000059291 | M_mulatta | Animals | mono_intronic | C/D | ENSMMUG00000013790 | LGR6 | protein_coding |
| ENSMMUG00000059294 | SNORD116 | M_mulatta | Animals | mono_intronic | C/D | ENSMMUG00000064025 | | non_coding |
| ENSMMUG00000059314 | ENSMMUG00000059314 | M_mulatta | Animals | mono_intergenic | C/D | | | intergenic |
| ENSMMUG00000059416 | U3 | M_mulatta | Animals | mono_intergenic | C/D | | | intergenic |
| ENSMMUG00000059427 | ENSMMUG00000059427 | M_mulatta | Animals | mono_intronic | C/D | ENSMMUG00000022518 | PACS1 | protein_coding |
| ENSMMUG00000059434 | ENSMMUG00000059434 | M_mulatta | Animals | mono_intergenic | C/D | | | intergenic |
| ENSMMUG00000059453 | ENSMMUG00000059453 | M_mulatta | Animals | mono_intronic | C/D | ENSMMUG00000003119 | BRD7 | protein_coding |
| ENSMMUG00000059456 | SNORD114-28 | M_mulatta | Animals | mono_intronic | C/D | ENSMMUG00000063833 | | non_coding |
| ENSMMUG00000059514 | ENSMMUG00000059514 | M_mulatta | Animals | mono_intergenic | C/D | | | intergenic |
| ENSMMUG00000059594 | SNORD115 | M_mulatta | Animals | mono_intergenic | C/D | | | intergenic |
| ENSMMUG00000059611 | ENSMMUG00000059611 | M_mulatta | Animals | mono_intergenic | C/D | | | intergenic |
| ENSMMUG00000059635 | ENSMMUG00000059635 | M_mulatta | Animals | mono_intergenic | C/D | | | intergenic |
| ENSMMUG00000059685 | ENSMMUG00000059685 | M_mulatta | Animals | mono_intergenic | C/D | | | intergenic |
| ENSMMUG00000059689 | ENSMMUG00000059689 | M_mulatta | Animals | mono_intronic | C/D | ENSMMUG00000000074 | RTF2 | protein_coding |
| ENSMMUG00000059694 | ENSMMUG00000059694 | M_mulatta | Animals | mono_intergenic | H/ACA | | | intergenic |
| ENSMMUG00000059700 | ENSMMUG00000059700 | M_mulatta | Animals | mono_intergenic | C/D | | | intergenic |
| ENSMMUG00000059718 | ENSMMUG00000059718 | M_mulatta | Animals | mono_intronic | C/D | ENSMMUG00000021238 | EDARADD | protein_coding |
| ENSMMUG00000059760 | ENSMMUG00000059760 | M_mulatta | Animals | mono_intergenic | C/D | | | intergenic |
| ENSMMUG00000059819 | ENSMMUG00000059819 | M_mulatta | Animals | mono_intronic | C/D | ENSMMUG00000061913 | | non_coding |
| ENSMMUG00000059849 | ENSMMUG00000059849 | M_mulatta | Animals | mono_intronic | C/D | ENSMMUG00000003483 | CINP | protein_coding |
| ENSMMUG00000059858 | ENSMMUG00000059858 | M_mulatta | Animals | mono_intergenic | C/D | | | intergenic |
| ENSMMUG00000059866 | ENSMMUG00000059866 | M_mulatta | Animals | mono_intergenic | C/D | | | intergenic |
| ENSMMUG00000059869 | SNORD116 | M_mulatta | Animals | mono_intergenic | C/D | | | intergenic |
| ENSMMUG00000059884 | ENSMMUG00000059884 | M_mulatta | Animals | mono_intergenic | C/D | | | intergenic |
| ENSMMUG00000059904 | ENSMMUG00000059904 | M_mulatta | Animals | mono_intergenic | C/D | | | intergenic |
| ENSMMUG00000059916 | ENSMMUG00000059916 | M_mulatta | Animals | mono_intronic | C/D | ENSMMUG00000006433 | SLC29A3 | protein_coding |

| | | | | | | | | |
|---|---|---|---|---|---|---|---|---|
| ENSMMUG00000059929 | U3 | M_mulatta | Animals | mono_intergenic | C/D | | | intergenic |
| ENSMMUG00000059931 | ENSMMUG00000059931 | M_mulatta | Animals | mono_intergenic | C/D | | | intergenic |
| ENSMMUG00000059941 | ENSMMUG00000059941 | M_mulatta | Animals | mono_intronic | C/D | ENSMMUG00000008722 | EPRS1 | protein_coding |
| ENSMMUG00000059952 | SNORD116 | M_mulatta | Animals | mono_intronic | C/D | ENSMMUG00000064025 | | non_coding |
| ENSMMUG00000059953 | ENSMMUG00000059953 | M_mulatta | Animals | mono_intergenic | C/D | | | intergenic |
| ENSMMUG00000059962 | ENSMMUG00000059962 | M_mulatta | Animals | intergenic_cluster | C/D | | | intergenic |
| ENSMMUG00000059967 | U3 | M_mulatta | Animals | mono_intergenic | C/D | | | intergenic |
| ENSMMUG00000059968 | SNORA71 | M_mulatta | Animals | mono_intergenic | H/ACA | | | intergenic |
| ENSMMUG00000060006 | ENSMMUG00000060006 | M_mulatta | Animals | mono_intronic | C/D | ENSMMUG00000023146 | ASH1L | protein_coding |
| ENSMMUG00000060037 | ENSMMUG00000060037 | M_mulatta | Animals | mono_intergenic | C/D | | | intergenic |
| ENSMMUG00000060122 | ENSMMUG00000060122 | M_mulatta | Animals | mono_intergenic | C/D | | | intergenic |
| ENSMMUG00000060124 | ENSMMUG00000060124 | M_mulatta | Animals | mono_intronic | C/D | ENSMMUG00000020690 | EPB41L1 | protein_coding |
| ENSMMUG00000060178 | SNORD116 | M_mulatta | Animals | mono_intronic | C/D | ENSMMUG00000064025 | | non_coding |
| ENSMMUG00000060241 | SNORD114-18 | M_mulatta | Animals | mono_intronic | C/D | ENSMMUG00000063833 | | non_coding |
| ENSMMUG00000060285 | ENSMMUG00000060285 | M_mulatta | Animals | mono_exonic | C/D | ENSMMUG00000040135 | | protein_coding |
| ENSMMUG00000060371 | SNORD115 | M_mulatta | Animals | mono_intergenic | C/D | | | intergenic |
| ENSMMUG00000060375 | ENSMMUG00000060375 | M_mulatta | Animals | mono_intergenic | C/D | | | intergenic |
| ENSMMUG00000060383 | ENSMMUG00000060383 | M_mulatta | Animals | mono_intergenic | C/D | | | intergenic |
| ENSMMUG00000060416 | ENSMMUG00000060416 | M_mulatta | Animals | mono_intergenic | C/D | | | intergenic |
| ENSMMUG00000060438 | ENSMMUG00000060438 | M_mulatta | Animals | mono_intronic | C/D | ENSMMUG00000017646 | SLC17A8 | protein_coding |
| ENSMMUG00000060442 | ENSMMUG00000060442 | M_mulatta | Animals | mono_intergenic | C/D | | | intergenic |
| ENSMMUG00000060454 | ENSMMUG00000060454 | M_mulatta | Animals | mono_intergenic | C/D | | | intergenic |
| ENSMMUG00000060488 | ENSMMUG00000060488 | M_mulatta | Animals | mono_intergenic | C/D | | | intergenic |
| ENSMMUG00000060518 | SNORD13P1 | M_mulatta | Animals | mono_intergenic | C/D | | | intergenic |
| ENSMMUG00000060548 | U3 | M_mulatta | Animals | mono_intergenic | C/D | | | intergenic |
| ENSMMUG00000060560 | ENSMMUG00000060560 | M_mulatta | Animals | mono_intergenic | C/D | | | intergenic |
| ENSMMUG00000060563 | ENSMMUG00000060563 | M_mulatta | Animals | mono_intergenic | C/D | | | intergenic |
| ENSMMUG00000060569 | ENSMMUG00000060569 | M_mulatta | Animals | mono_intergenic | C/D | | | intergenic |
| ENSMMUG00000060617 | ENSMMUG00000060617 | M_mulatta | Animals | mono_intergenic | C/D | | | intergenic |
| ENSMMUG00000060640 | SNORD115 | M_mulatta | Animals | mono_intergenic | C/D | | | intergenic |
| ENSMMUG00000060709 | ENSMMUG00000060709 | M_mulatta | Animals | mono_intergenic | C/D | | | intergenic |
| ENSMMUG00000060718 | SNORD115 | M_mulatta | Animals | mono_intergenic | C/D | | | intergenic |
| ENSMMUG00000060773 | SNORA58B | M_mulatta | Animals | mono_intronic | H/ACA | ENSMMUG00000010810 | UBAP2L | protein_coding |
| ENSMMUG00000060796 | ENSMMUG00000060796 | M_mulatta | Animals | mono_intronic | C/D | ENSMMUG00000053733 | | protein_coding |
| ENSMMUG00000060829 | ENSMMUG00000060829 | M_mulatta | Animals | mono_intronic | H/ACA | ENSMMUG00000001340 | RPL32 | protein_coding |
| ENSMMUG00000060839 | ENSMMUG00000060839 | M_mulatta | Animals | mono_intergenic | C/D | | | intergenic |
| ENSMMUG00000060841 | ENSMMUG00000060841 | M_mulatta | Animals | mono_intergenic | C/D | | | intergenic |
| ENSMMUG00000060882 | ENSMMUG00000060882 | M_mulatta | Animals | mono_intergenic | C/D | | | intergenic |
| ENSMMUG00000060884 | SNORD113-4 | M_mulatta | Animals | mono_intergenic | C/D | | | intergenic |
| ENSMMUG00000060919 | U3 | M_mulatta | Animals | intergenic_cluster | C/D | | | intergenic |
| ENSMMUG00000061066 | ENSMMUG00000061066 | M_mulatta | Animals | mono_intergenic | C/D | | | intergenic |
| ENSMMUG00000061084 | ENSMMUG00000061084 | M_mulatta | Animals | mono_intronic | C/D | ENSMMUG00000052641 | TMEM242 | protein_coding |
| ENSMMUG00000061091 | SNORD115 | M_mulatta | Animals | mono_intergenic | C/D | | | intergenic |
| ENSMMUG00000061160 | ENSMMUG00000061160 | M_mulatta | Animals | mono_intergenic | C/D | | | intergenic |
| ENSMMUG00000061179 | U3 | M_mulatta | Animals | mono_intergenic | C/D | | | intergenic |

| | | | | | | | | |
|---|---|---|---|---|---|---|---|---|
| ENSMMUG00000061183 | ENSMMUG00000061183 | M_mulatta | Animals | mono_intronic | C/D | ENSMMUG00000016487 | RALGAPA1 | protein_coding |
| ENSMMUG00000061188 | ENSMMUG00000061188 | M_mulatta | Animals | mono_intergenic | C/D | | | intergenic |
| ENSMMUG00000061197 | ENSMMUG00000061197 | M_mulatta | Animals | mono_intergenic | C/D | | | intergenic |
| ENSMMUG00000061214 | U3 | M_mulatta | Animals | mono_intronic | C/D | ENSMMUG00000013276 | BAIAP2L1 | protein_coding |
| ENSMMUG00000061313 | ENSMMUG00000061313 | M_mulatta | Animals | mono_intronic | C/D | ENSMMUG00000022441 | SMG1 | protein_coding |
| ENSMMUG00000061317 | U3 | M_mulatta | Animals | intergenic_cluster | C/D | | | intergenic |
| ENSMMUG00000061352 | SNORD13E | M_mulatta | Animals | mono_intronic | C/D | ENSMMUG00000022056 | DENND4A | protein_coding |
| ENSMMUG00000061427 | ENSMMUG00000061427 | M_mulatta | Animals | mono_intergenic | C/D | | | intergenic |
| ENSMMUG00000061438 | ENSMMUG00000061438 | M_mulatta | Animals | mono_intergenic | C/D | | | intergenic |
| ENSMMUG00000061448 | ENSMMUG00000061448 | M_mulatta | Animals | mono_intergenic | C/D | | | intergenic |
| ENSMMUG00000061482 | ENSMMUG00000061482 | M_mulatta | Animals | mono_intronic | C/D | ENSMMUG00000021280 | LRTOMT | protein_coding |
| ENSMMUG00000061485 | ENSMMUG00000061485 | M_mulatta | Animals | mono_intergenic | C/D | | | intergenic |
| ENSMMUG00000061489 | ENSMMUG00000061489 | M_mulatta | Animals | mono_intergenic | C/D | | | intergenic |
| ENSMMUG00000061497 | U3 | M_mulatta | Animals | mono_intergenic | C/D | | | intergenic |
| ENSMMUG00000061512 | ENSMMUG00000061512 | M_mulatta | Animals | mono_intergenic | C/D | | | intergenic |
| ENSMMUG00000061559 | ENSMMUG00000061559 | M_mulatta | Animals | mono_intergenic | C/D | | | intergenic |
| ENSMMUG00000061608 | SNORD114-31 | M_mulatta | Animals | mono_intronic | C/D | ENSMMUG00000063833 | | non_coding |
| ENSMMUG00000061627 | ENSMMUG00000061627 | M_mulatta | Animals | mono_intergenic | C/D | | | intergenic |
| ENSMMUG00000061657 | ENSMMUG00000061657 | M_mulatta | Animals | mono_intergenic | C/D | | | intergenic |
| ENSMMUG00000061662 | ENSMMUG00000061662 | M_mulatta | Animals | mono_intergenic | C/D | | | intergenic |
| ENSMMUG00000061674 | U3 | M_mulatta | Animals | mono_intronic | C/D | ENSMMUG00000019407 | RIMS2 | protein_coding |
| ENSMMUG00000061677 | SNORD116 | M_mulatta | Animals | mono_intronic | C/D | ENSMMUG00000064025 | | non_coding |
| ENSMMUG00000061740 | ENSMMUG00000061740 | M_mulatta | Animals | mono_intergenic | C/D | | | intergenic |
| ENSMMUG00000061746 | ENSMMUG00000061746 | M_mulatta | Animals | mono_intergenic | C/D | | | intergenic |
| ENSMMUG00000061770 | ENSMMUG00000061770 | M_mulatta | Animals | mono_intergenic | C/D | | | intergenic |
| ENSMMUG00000061842 | ENSMMUG00000061842 | M_mulatta | Animals | mono_intergenic | H/ACA | | | intergenic |
| ENSMMUG00000061947 | ENSMMUG00000061947 | M_mulatta | Animals | mono_intergenic | C/D | | | intergenic |
| ENSMMUG00000061980 | ENSMMUG00000061980 | M_mulatta | Animals | mono_intronic | C/D | ENSMMUG00000009341 | ACTA2 | protein_coding |
| ENSMMUG00000062027 | ENSMMUG00000062027 | M_mulatta | Animals | mono_intergenic | C/D | | | intergenic |
| ENSMMUG00000062032 | ENSMMUG00000062032 | M_mulatta | Animals | mono_intergenic | C/D | | | intergenic |
| ENSMMUG00000062038 | U3 | M_mulatta | Animals | intergenic_cluster | C/D | | | intergenic |
| ENSMMUG00000062052 | SNORD116 | M_mulatta | Animals | mono_intronic | C/D | ENSMMUG00000064025 | | non_coding |
| ENSMMUG00000062092 | ENSMMUG00000062092 | M_mulatta | Animals | mono_intronic | C/D | ENSMMUG00000015535 | IMMP2L | protein_coding |
| ENSMMUG00000062114 | ENSMMUG00000062114 | M_mulatta | Animals | mono_intergenic | C/D | | | intergenic |
| ENSMMUG00000062118 | U3 | M_mulatta | Animals | mono_intronic | C/D | ENSMMUG00000018715 | | protein_coding |
| ENSMMUG00000062123 | ENSMMUG00000062123 | M_mulatta | Animals | mono_intergenic | C/D | | | intergenic |
| ENSMMUG00000062184 | ENSMMUG00000062184 | M_mulatta | Animals | mono_intronic | C/D | ENSMMUG00000005461 | SMCHD1 | protein_coding |
| ENSMMUG00000062214 | ENSMMUG00000062214 | M_mulatta | Animals | mono_intergenic | C/D | | | intergenic |
| ENSMMUG00000062221 | ENSMMUG00000062221 | M_mulatta | Animals | mono_intergenic | C/D | | | intergenic |
| ENSMMUG00000062222 | ENSMMUG00000062222 | M_mulatta | Animals | mono_intronic | C/D | ENSMMUG00000061977 | SPIRE1 | protein_coding |
| ENSMMUG00000062234 | SNORD115 | M_mulatta | Animals | mono_intergenic | C/D | | | intergenic |
| ENSMMUG00000062235 | U3 | M_mulatta | Animals | mono_intergenic | C/D | | | intergenic |
| ENSMMUG00000062245 | ENSMMUG00000062245 | M_mulatta | Animals | mono_intergenic | C/D | | | intergenic |
| ENSMMUG00000062291 | ENSMMUG00000062291 | M_mulatta | Animals | mono_intergenic | C/D | | | intergenic |
| ENSMMUG00000062319 | ENSMMUG00000062319 | M_mulatta | Animals | mono_intronic | H/ACA | ENSMMUG00000020202 | | protein_coding |

| | | | | | | | | |
|---|---|---|---|---|---|---|---|---|
| ENSMMUG00000062349 | ENSMMUG00000062349 | M_mulatta | Animals | mono_intergenic | C/D | | | intergenic |
| ENSMMUG00000062366 | ENSMMUG00000062366 | M_mulatta | Animals | mono_intronic | C/D | ENSMMUG00000023318 | TRRAP | protein_coding |
| ENSMMUG00000062383 | U3 | M_mulatta | Animals | mono_intergenic | C/D | | | intergenic |
| ENSMMUG00000062389 | ENSMMUG00000062389 | M_mulatta | Animals | mono_intergenic | C/D | | | intergenic |
| ENSMMUG00000062409 | ENSMMUG00000062409 | M_mulatta | Animals | mono_intergenic | C/D | | | intergenic |
| ENSMMUG00000062432 | ENSMMUG00000062432 | M_mulatta | Animals | mono_intergenic | C/D | | | intergenic |
| ENSMMUG00000062460 | SNORA73 | M_mulatta | Animals | mono_intronic | H/ACA | ENSMMUG00000004313 | PLCB1 | protein_coding |
| ENSMMUG00000062472 | ENSMMUG00000062472 | M_mulatta | Animals | mono_intergenic | C/D | | | intergenic |
| ENSMMUG00000062476 | ENSMMUG00000062476 | M_mulatta | Animals | mono_intergenic | C/D | | | intergenic |
| ENSMMUG00000062505 | SNORD115 | M_mulatta | Animals | mono_intergenic | C/D | | | intergenic |
| ENSMMUG00000062516 | SNORD115 | M_mulatta | Animals | mono_intergenic | C/D | | | intergenic |
| ENSMMUG00000062562 | SNORD113-3 | M_mulatta | Animals | mono_intergenic | C/D | | | intergenic |
| ENSMMUG00000062601 | ENSMMUG00000062601 | M_mulatta | Animals | mono_intronic | C/D | ENSMMUG00000001522 | | protein_coding |
| ENSMMUG00000062626 | ENSMMUG00000062626 | M_mulatta | Animals | mono_intergenic | C/D | | | intergenic |
| ENSMMUG00000062631 | ENSMMUG00000062631 | M_mulatta | Animals | mono_intergenic | C/D | | | intergenic |
| ENSMMUG00000062671 | SNORD115 | M_mulatta | Animals | mono_intergenic | C/D | | | intergenic |
| ENSMMUG00000062687 | ENSMMUG00000062687 | M_mulatta | Animals | mono_intronic | C/D | ENSMMUG00000010934 | EIF4G1 | protein_coding |
| ENSMMUG00000062692 | ENSMMUG00000062692 | M_mulatta | Animals | mono_intergenic | C/D | | | intergenic |
| ENSMMUG00000062710 | ENSMMUG00000062710 | M_mulatta | Animals | mono_intergenic | C/D | | | intergenic |
| ENSMMUG00000062711 | ENSMMUG00000062711 | M_mulatta | Animals | mono_intergenic | C/D | | | intergenic |
| ENSMMUG00000062715 | ENSMMUG00000062715 | M_mulatta | Animals | mono_intergenic | C/D | | | intergenic |
| ENSMMUG00000062728 | ENSMMUG00000062728 | M_mulatta | Animals | mono_intergenic | C/D | | | intergenic |
| ENSMMUG00000062762 | ENSMMUG00000062762 | M_mulatta | Animals | mono_intergenic | C/D | | | intergenic |
| ENSMMUG00000062805 | ENSMMUG00000062805 | M_mulatta | Animals | mono_intergenic | C/D | | | intergenic |
| ENSMMUG00000062806 | ENSMMUG00000062806 | M_mulatta | Animals | mono_intergenic | C/D | | | intergenic |
| ENSMMUG00000062839 | ENSMMUG00000062839 | M_mulatta | Animals | mono_intronic | C/D | ENSMMUG00000005888 | TTC13 | protein_coding |
| ENSMMUG00000062855 | ENSMMUG00000062855 | M_mulatta | Animals | mono_intergenic | C/D | | | intergenic |
| ENSMMUG00000062856 | ENSMMUG00000062856 | M_mulatta | Animals | mono_intergenic | C/D | | | intergenic |
| ENSMMUG00000062984 | SNORD115 | M_mulatta | Animals | mono_intergenic | C/D | | | intergenic |
| ENSMMUG00000063006 | ENSMMUG00000063006 | M_mulatta | Animals | mono_intergenic | C/D | | | intergenic |
| ENSMMUG00000063035 | ENSMMUG00000063035 | M_mulatta | Animals | mono_intergenic | C/D | | | intergenic |
| ENSMMUG00000063036 | SNORD115 | M_mulatta | Animals | mono_intergenic | C/D | | | intergenic |
| ENSMMUG00000063045 | SNORD67 | M_mulatta | Animals | mono_intronic | C/D | ENSMMUG00000003631 | CKAP5 | protein_coding |
| ENSMMUG00000063095 | ENSMMUG00000063095 | M_mulatta | Animals | mono_intronic | C/D | ENSMMUG00000002032 | XPO7 | protein_coding |
| ENSMMUG00000063114 | SNORA71 | M_mulatta | Animals | mono_intergenic | H/ACA | | | intergenic |
| ENSMMUG00000063137 | ENSMMUG00000063137 | M_mulatta | Animals | mono_intergenic | C/D | | | intergenic |
| ENSMMUG00000063146 | SNORA71 | M_mulatta | Animals | mono_intronic | H/ACA | ENSMMUG00000017350 | RALGAPB | protein_coding |
| ENSMMUG00000063209 | ENSMMUG00000063209 | M_mulatta | Animals | mono_intergenic | C/D | | | intergenic |
| ENSMMUG00000063211 | ENSMMUG00000063211 | M_mulatta | Animals | mono_intergenic | C/D | | | intergenic |
| ENSMMUG00000063313 | U3 | M_mulatta | Animals | mono_intergenic | C/D | | | intergenic |
| ENSMMUG00000063331 | ENSMMUG00000063331 | M_mulatta | Animals | mono_intergenic | C/D | | | intergenic |
| ENSMMUG00000063352 | SNORD115 | M_mulatta | Animals | mono_intergenic | C/D | | | intergenic |
| ENSMMUG00000063381 | ENSMMUG00000063381 | M_mulatta | Animals | mono_intergenic | C/D | | | intergenic |
| ENSMMUG00000063392 | ENSMMUG00000063392 | M_mulatta | Animals | mono_intergenic | C/D | | | intergenic |
| ENSMMUG00000063398 | U3 | M_mulatta | Animals | mono_intergenic | C/D | | | intergenic |

| | | | | | | | | |
|---|---|---|---|---|---|---|---|---|
| ENSMMUG00000063400 | U3 | M_mulatta | Animals | mono_intronic | C/D | ENSMMUG00000014952 | NUP210L | protein_coding |
| ENSMMUG00000063438 | ENSMMUG00000063438 | M_mulatta | Animals | mono_intergenic | C/D | | | intergenic |
| ENSMMUG00000063487 | ENSMMUG00000063487 | M_mulatta | Animals | mono_intronic | C/D | ENSMMUG00000010029 | RPL3 | protein_coding |
| ENSMMUG00000063491 | U3 | M_mulatta | Animals | mono_intronic | C/D | ENSMMUG00000017438 | DCBLD2 | protein_coding |
| ENSMMUG00000063533 | U3 | M_mulatta | Animals | mono_intergenic | C/D | | | intergenic |
| ENSMMUG00000063567 | SNORD115 | M_mulatta | Animals | mono_intergenic | C/D | | | intergenic |
| ENSMMUG00000063582 | ENSMMUG00000063582 | M_mulatta | Animals | intergenic_cluster | C/D | | | intergenic |
| ENSMMUG00000063595 | ENSMMUG00000063595 | M_mulatta | Animals | mono_intronic | H/ACA | ENSMMUG00000063541 | | protein_coding |
| ENSMMUG00000063636 | ENSMMUG00000063636 | M_mulatta | Animals | mono_intronic | C/D | ENSMMUG00000022603 | ARHGAP42 | protein_coding |
| ENSMMUG00000063667 | ENSMMUG00000063667 | M_mulatta | Animals | mono_intergenic | C/D | | | intergenic |
| ENSMMUG00000063691 | ENSMMUG00000063691 | M_mulatta | Animals | mono_intergenic | C/D | | | intergenic |
| ENSMMUG00000063704 | SNORD115 | M_mulatta | Animals | mono_intergenic | C/D | | | intergenic |
| ENSMMUG00000063709 | ENSMMUG00000063709 | M_mulatta | Animals | mono_intergenic | C/D | | | intergenic |
| ENSMMUG00000063730 | ENSMMUG00000063730 | M_mulatta | Animals | mono_intronic | C/D | ENSMMUG00000008008 | PPP1R9A | protein_coding |
| ENSMMUG00000063767 | ENSMMUG00000063767 | M_mulatta | Animals | mono_intronic | C/D | ENSMMUG00000039612 | NME5 | protein_coding |
| ENSMMUG00000063775 | ENSMMUG00000063775 | M_mulatta | Animals | mono_intergenic | C/D | | | intergenic |
| ENSMMUG00000063802 | SNORD116 | M_mulatta | Animals | mono_intergenic | C/D | | | intergenic |
| ENSMMUG00000063827 | ENSMMUG00000063827 | M_mulatta | Animals | mono_intronic | C/D | ENSMMUG00000045509 | NAALADL2 | protein_coding |
| ENSMMUG00000063875 | ENSMMUG00000063875 | M_mulatta | Animals | mono_intergenic | H/ACA | | | intergenic |
| ENSMMUG00000063890 | ENSMMUG00000063890 | M_mulatta | Animals | mono_intergenic | C/D | | | intergenic |
| ENSMMUG00000063908 | ENSMMUG00000063908 | M_mulatta | Animals | mono_intergenic | C/D | | | intergenic |
| ENSMMUG00000063927 | SNORD116 | M_mulatta | Animals | mono_intronic | C/D | ENSMMUG00000054792 | | non_coding |
| ENSMMUG00000063975 | ENSMMUG00000063975 | M_mulatta | Animals | mono_intergenic | C/D | | | intergenic |
| ENSMMUG00000063993 | ENSMMUG00000063993 | M_mulatta | Animals | mono_intronic | C/D | ENSMMUG00000008759 | WEE1 | protein_coding |
| ENSMMUG00000063994 | U3 | M_mulatta | Animals | mono_intergenic | C/D | | | intergenic |
| ENSMMUG00000064001 | ENSMMUG00000064001 | M_mulatta | Animals | mono_intronic | C/D | ENSMMUG00000013954 | PDZD2 | protein_coding |
| ENSMMUG00000064024 | ENSMMUG00000064024 | M_mulatta | Animals | mono_intergenic | C/D | | | intergenic |
| ENSMMUG00000064032 | ENSMMUG00000064032 | M_mulatta | Animals | mono_intergenic | C/D | | | intergenic |
| ENSMMUG00000064165 | ENSMMUG00000064165 | M_mulatta | Animals | mono_intronic | C/D | ENSMMUG00000006050 | ZHX1 | protein_coding |
| ENSMMUG00000064175 | SNORD115 | M_mulatta | Animals | mono_intergenic | C/D | | | intergenic |
| ENSMMUG00000064211 | ENSMMUG00000064211 | M_mulatta | Animals | mono_intergenic | C/D | | | intergenic |
| ENSMMUG00000064217 | ENSMMUG00000064217 | M_mulatta | Animals | mono_intergenic | C/D | | | intergenic |
| ENSMMUG00000064276 | ENSMMUG00000064276 | M_mulatta | Animals | mono_intronic | C/D | ENSMMUG00000019325 | MTOR | protein_coding |
| ENSMMUG00000064283 | ENSMMUG00000064283 | M_mulatta | Animals | mono_intergenic | C/D | | | intergenic |
| ENSMMUG00000064345 | ENSMMUG00000064345 | M_mulatta | Animals | mono_intronic | H/ACA | ENSMMUG00000049577 | | non_coding |
| ENSMMUG00000064362 | U3 | M_mulatta | Animals | mono_intronic | C/D | ENSMMUG00000050742 | SKAP1 | protein_coding |
| ENSMMUG00000064484 | ENSMMUG00000064484 | M_mulatta | Animals | mono_intergenic | C/D | | | intergenic |
| ENSMMUG00000064498 | U3 | M_mulatta | Animals | mono_intronic | C/D | ENSMMUG00000007102 | NCAPD3 | protein_coding |
| ENSMMUG00000064536 | ENSMMUG00000064536 | M_mulatta | Animals | mono_intergenic | C/D | | | intergenic |
| ENSMMUG00000064655 | U3 | M_mulatta | Animals | mono_intergenic | C/D | | | intergenic |
| ENSMMUG00000064660 | ENSMMUG00000064660 | M_mulatta | Animals | mono_intergenic | C/D | | | intergenic |
| ENSMMUG00000064676 | ENSMMUG00000064676 | M_mulatta | Animals | mono_intergenic | C/D | | | intergenic |
| ENSMMUG00000064693 | ENSMMUG00000064693 | M_mulatta | Animals | mono_intergenic | C/D | | | intergenic |
| ENSMMUG00000064725 | SNORD116 | M_mulatta | Animals | mono_intergenic | C/D | | | intergenic |
| ENSMMUG00000064759 | ENSMMUG00000064759 | M_mulatta | Animals | mono_intergenic | C/D | | | intergenic |

| | | | | | | | | | |
|---|---|---|---|---|---|---|---|---|---|
| ENSMMUG00000064815 | ENSMMUG00000064815 | M_mulatta | Animals | mono_intergenic | C/D | | | intergenic |
| ENSMMUG00000064818 | ENSMMUG00000064818 | M_mulatta | Animals | mono_intronic | C/D | ENSMMUG00000019407 | RIMS2 | protein_coding |
| ENSMMUG00000064831 | U3 | M_mulatta | Animals | mono_intronic | C/D | ENSMMUG00000022083 | GCM1 | protein_coding |
| ENSMMUG00000064881 | ENSMMUG00000064881 | M_mulatta | Animals | mono_intergenic | C/D | | | intergenic |
| ENSMMUG00000064943 | ENSMMUG00000064943 | M_mulatta | Animals | mono_intergenic | C/D | | | intergenic |
| ENSMMUG00000064969 | ENSMMUG00000064969 | M_mulatta | Animals | mono_intronic | C/D | ENSMMUG00000011285 | CFAP70 | protein_coding |
| ENSMMUG00000064999 | ENSMMUG00000064999 | M_mulatta | Animals | mono_intergenic | C/D | | | intergenic |
| ENSMMUG00000065011 | ENSMMUG00000065011 | M_mulatta | Animals | intronic_cluster | C/D | ENSMMUG00000010891 | KAT6A | protein_coding |
| ENSMMUG00000065014 | ENSMMUG00000065014 | M_mulatta | Animals | mono_intergenic | C/D | | | intergenic |
| ENSMMUG00000065027 | SNORA80D | M_mulatta | Animals | mono_intronic | H/ACA | ENSMMUG00000030420 | AIMP2 | protein_coding |
| ENSMMUG00000065030 | SNORD115 | M_mulatta | Animals | mono_intergenic | C/D | | | intergenic |
| ENSMMUG00000065085 | ENSMMUG00000065085 | M_mulatta | Animals | mono_intergenic | C/D | | | intergenic |
| ENSMMUG00000065124 | ENSMMUG00000065124 | M_mulatta | Animals | mono_intergenic | C/D | | | intergenic |
| ENSMMUG00000065129 | ENSMMUG00000065129 | M_mulatta | Animals | mono_intergenic | C/D | | | intergenic |
| ENSMMUG00000065164 | SNORD115 | M_mulatta | Animals | mono_intergenic | C/D | | | intergenic |
| ENSMMUG00000065171 | ENSMMUG00000065171 | M_mulatta | Animals | mono_intronic | C/D | ENSMMUG00000022750 | DPP7 | protein_coding |
| ENSMMUG00000065184 | SNORA70 | M_mulatta | Animals | mono_exonic | H/ACA | ENSMMUG00000058063 | | protein_coding |
| ENSMMUG00000065191 | SNORD113-6 | M_mulatta | Animals | mono_intronic | C/D | ENSMMUG00000053357 | | non_coding |
| ENSMMUG00000065200 | SNORD115 | M_mulatta | Animals | mono_intergenic | C/D | | | intergenic |
| ENSMMUG00000065223 | ENSMMUG00000065223 | M_mulatta | Animals | mono_intergenic | C/D | | | intergenic |
| ENSMMUG00000065279 | SNORD115 | M_mulatta | Animals | mono_intergenic | C/D | | | intergenic |
| ENSMMUG00000065291 | SNORD36 | M_mulatta | Animals | mono_intergenic | C/D | | | intergenic |
| ENSMMUG00000065320 | ENSMMUG00000065320 | M_mulatta | Animals | mono_intergenic | C/D | | | intergenic |
| ENSMUSG00000064377 | Gm24966 | M_musculus | Animals | mono_intergenic | C/D | ENSMUSG00000100826 | Snhg14 | non_coding |
| ENSMUSG00000064380 | Gm26448 | M_musculus | Animals | mono_intronic | H/ACA | ENSMUSG00000032518 | Rpsa | protein_coding |
| ENSMUSG00000064382 | Gm26447 | M_musculus | Animals | mono_intronic | H/ACA | ENSMUSG00000022884 | Eif4a2 | protein_coding |
| ENSMUSG00000064387 | Snora73a | M_musculus | Animals | mono_intronic | H/ACA | ENSMUSG00000028896 | Rcc1 | protein_coding |
| ENSMUSG00000064390 | Snord73b | M_musculus | Animals | mono_intronic | C/D | ENSMUSG00000028081 | Rps3a1 | protein_coding |
| ENSMUSG00000064392 | Gm23626 | M_musculus | Animals | mono_intergenic | H/ACA | | | intergenic |
| ENSMUSG00000064400 | Snord3b-ps1 | M_musculus | Animals | intergenic_cluster | C/D | | | intergenic |
| ENSMUSG00000064405 | Gm23925 | M_musculus | Animals | mono_intronic | H/ACA | ENSMUSG00000085385 | Snhg17 | non_coding |
| ENSMUSG00000064410 | Gm22247 | M_musculus | Animals | mono_intronic | H/ACA | ENSMUSG00000028334 | Nans | protein_coding |
| ENSMUSG00000064417 | DQ267102 | M_musculus | Animals | mono_intronic | C/D | ENSMUSG00000097451 | Rian | non_coding |
| ENSMUSG00000064427 | Gm22748 | M_musculus | Animals | mono_intronic | H/ACA | ENSMUSG00000011179 | Odc1 | protein_coding |
| ENSMUSG00000064435 | Gm25588 | M_musculus | Animals | mono_intronic | H/ACA | ENSMUSG00000042708 | Shcbp1l | protein_coding |
| ENSMUSG00000064437 | Snord49b | M_musculus | Animals | mono_intronic | C/D | ENSMUSG00000086841 | 2410006H16Rik | non_coding |
| ENSMUSG00000064440 | Gm26224 | M_musculus | Animals | mono_intronic | C/D | ENSMUSG00000053332 | Gas5 | non_coding |
| ENSMUSG00000064441 | Snord37 | M_musculus | Animals | mono_intronic | C/D | ENSMUSG00000034994 | Eef2 | protein_coding |
| ENSMUSG00000064442 | Gm26225 | M_musculus | Animals | mono_intronic | H/ACA | ENSMUSG00000060012 | Kif13b | protein_coding |
| ENSMUSG00000064450 | Snord68 | M_musculus | Animals | mono_intronic | C/D | ENSMUSG00000000740 | Rpl13 | protein_coding |
| ENSMUSG00000064451 | Snora23 | M_musculus | Animals | mono_intronic | H/ACA | ENSMUSG00000066232 | Ipo7 | protein_coding |
| ENSMUSG00000064452 | Gm24564 | M_musculus | Animals | intronic_cluster | C/D | ENSMUSG00000097451 | Rian | non_coding |
| ENSMUSG00000064453 | Snord21 | M_musculus | Animals | mono_intronic | C/D | ENSMUSG00000058558 | Rpl5 | protein_coding |
| ENSMUSG00000064463 | Gm25079 | M_musculus | Animals | mono_intergenic | H/ACA | | | intergenic |
| ENSMUSG00000064471 | Gm23406 | M_musculus | Animals | mono_exonic | H/ACA | ENSMUSG00000116830 | Gm49714 | non_coding |

| | | | | | | | | |
|---|---|---|---|---|---|---|---|---|
| ENSMUSG00000064477 | Gm23404 | M_musculus | Animals | mono_intergenic | H/ACA | | | intergenic |
| ENSMUSG00000064487 | Gm23736 | M_musculus | Animals | mono_intronic | C/D | ENSMUSG00000098202 | B830012L14Rik | non_coding |
| ENSMUSG00000064493 | Snora28 | M_musculus | Animals | mono_intronic | H/ACA | ENSMUSG00000021282 | Eif5 | protein_coding |
| ENSMUSG00000064495 | Gm24313 | M_musculus | Animals | mono_intronic | H/ACA | ENSMUSG00000000384 | Tbrg4 | protein_coding |
| ENSMUSG00000064496 | DQ267101 | M_musculus | Animals | mono_intronic | C/D | ENSMUSG00000097451 | Rian | non_coding |
| ENSMUSG00000064500 | Gm25296 | M_musculus | Animals | mono_intronic | C/D | ENSMUSG00000020372 | Rack1 | protein_coding |
| ENSMUSG00000064511 | Gm22459 | M_musculus | Animals | mono_intergenic | H/ACA | | | intergenic |
| ENSMUSG00000064513 | Snora9 | M_musculus | Animals | mono_intronic | H/ACA | ENSMUSG00000085156 | Snhg15 | non_coding |
| ENSMUSG00000064514 | Gm22455 | M_musculus | Animals | mono_intronic | C/D | ENSMUSG00000032399 | Rpl4 | protein_coding |
| ENSMUSG00000064525 | Gm26440 | M_musculus | Animals | mono_intergenic | H/ACA | | | intergenic |
| ENSMUSG00000064530 | Gm23613 | M_musculus | Animals | mono_intronic | H/ACA | ENSMUSG00000037636 | Slc25a43 | protein_coding |
| ENSMUSG00000064536 | Gm23615 | M_musculus | Animals | mono_intergenic | C/D | | | intergenic |
| ENSMUSG00000064540 | Snord42a | M_musculus | Animals | mono_intronic | C/D | ENSMUSG00000058546 | Rpl23a | protein_coding |
| ENSMUSG00000064542 | Gm22980 | M_musculus | Animals | mono_intronic | C/D | ENSMUSG00000047675 | Rps8 | protein_coding |
| ENSMUSG00000064545 | Gm22981 | M_musculus | Animals | mono_intronic | C/D | ENSMUSG00000098202 | B830012L14Rik | non_coding |
| ENSMUSG00000064556 | Gm24625 | M_musculus | Animals | mono_intergenic | H/ACA | | | intergenic |
| ENSMUSG00000064558 | Gm24620 | M_musculus | Animals | mono_intronic | H/ACA | ENSMUSG00000021375 | Kif13a | protein_coding |
| ENSMUSG00000064563 | Snora7a | M_musculus | Animals | mono_intronic | H/ACA | ENSMUSG00000057841 | Rpl32 | protein_coding |
| ENSMUSG00000064567 | Gm24143 | M_musculus | Animals | mono_intergenic | H/ACA | | | intergenic |
| ENSMUSG00000064569 | Gm25592 | M_musculus | Animals | mono_intergenic | H/ACA | | | intergenic |
| ENSMUSG00000064570 | Gm25800 | M_musculus | Animals | mono_intergenic | H/ACA | | | intergenic |
| ENSMUSG00000064573 | Gm25802 | M_musculus | Animals | mono_intergenic | C/D | | | intergenic |
| ENSMUSG00000064581 | Gm25133 | M_musculus | Animals | mono_intergenic | H/ACA | | | intergenic |
| ENSMUSG00000064585 | Gm25129 | M_musculus | Animals | intronic_cluster | H/ACA | ENSMUSG00000044349 | Snhg11 | protein_coding |
| ENSMUSG00000064595 | Gm22300 | M_musculus | Animals | mono_intergenic | H/ACA | | | intergenic |
| ENSMUSG00000064596 | Gm22298 | M_musculus | Animals | mono_intergenic | H/ACA | | | intergenic |
| ENSMUSG00000064597 | Gm22299 | M_musculus | Animals | mono_intronic | H/ACA | ENSMUSG00000022362 | Gm29394 | protein_coding |
| ENSMUSG00000064600 | Gm25636 | M_musculus | Animals | mono_intronic | H/ACA | ENSMUSG00000066232 | Ipo7 | protein_coding |
| ENSMUSG00000064602 | Snora41 | M_musculus | Animals | mono_intronic | H/ACA | ENSMUSG00000025967 | Eef1b2 | protein_coding |
| ENSMUSG00000064604 | Snora44 | M_musculus | Animals | mono_intronic | H/ACA | ENSMUSG00000086290 | Snhg12 | non_coding |
| ENSMUSG00000064609 | Gm25635 | M_musculus | Animals | mono_intronic | H/ACA | ENSMUSG00000019790 | Stxbp5 | protein_coding |
| ENSMUSG00000064612 | Gm26457 | M_musculus | Animals | intronic_cluster | C/D | ENSMUSG00000025967 | Eef1b2 | protein_coding |
| ENSMUSG00000064620 | Gm22303 | M_musculus | Animals | mono_intronic | H/ACA | ENSMUSG00000020580 | Rock2 | protein_coding |
| ENSMUSG00000064621 | Gm26922 | M_musculus | Animals | mono_intronic | C/D | ENSMUSG00000097451 | Rian | non_coding |
| ENSMUSG00000064622 | Gm22304 | M_musculus | Animals | mono_intergenic | H/ACA | | | intergenic |
| ENSMUSG00000064634 | Gm22620 | M_musculus | Animals | mono_intronic | H/ACA | ENSMUSG00000031939 | Taf1d | protein_coding |
| ENSMUSG00000064637 | Snora20 | M_musculus | Animals | mono_intronic | H/ACA | ENSMUSG00000068039 | Tcp1 | protein_coding |
| ENSMUSG00000064646 | Gm23300 | M_musculus | Animals | mono_intronic | H/ACA | ENSMUSG00000024989 | Cep55 | protein_coding |
| ENSMUSG00000064647 | Gm23301 | M_musculus | Animals | mono_intronic | C/D | ENSMUSG00000062328 | Rpl17 | protein_coding |
| ENSMUSG00000064652 | Gm24165 | M_musculus | Animals | mono_intergenic | H/ACA | | | intergenic |
| ENSMUSG00000064655 | Gm25788 | M_musculus | Animals | mono_intronic | H/ACA | ENSMUSG00000011257 | Pabpc4 | protein_coding |
| ENSMUSG00000064658 | Gm24166 | M_musculus | Animals | mono_intergenic | H/ACA | | | intergenic |
| ENSMUSG00000064665 | Gm24460 | M_musculus | Animals | mono_intergenic | H/ACA | | | intergenic |
| ENSMUSG00000064666 | Snora52 | M_musculus | Animals | mono_intronic | H/ACA | ENSMUSG00000025508 | Rplp2 | protein_coding |
| ENSMUSG00000064671 | Gm22807 | M_musculus | Animals | mono_intergenic | H/ACA | | | intergenic |

| | | | | | | | | |
|---|---|---|---|---|---|---|---|---|
| ENSMUSG00000064672 | Gm22806 | M_musculus | Animals | mono_intronic | H/ACA | ENSMUSG00000062248 | Cks2 | protein_coding |
| ENSMUSG00000064679 | Gm25357 | M_musculus | Animals | mono_intronic | C/D | ENSMUSG00000097451 | Rian | non_coding |
| ENSMUSG00000064692 | Gm24151 | M_musculus | Animals | mono_intergenic | H/ACA | | | intergenic |
| ENSMUSG00000064697 | Gm24147 | M_musculus | Animals | mono_intronic | H/ACA | ENSMUSG00000071769 | Rhox3h | protein_coding |
| ENSMUSG00000064717 | Gm24208 | M_musculus | Animals | mono_intergenic | C/D | | | intergenic |
| ENSMUSG00000064720 | Gm25856 | M_musculus | Animals | mono_intronic | C/D | ENSMUSG00000098202 | B830012L14Rik | non_coding |
| ENSMUSG00000064721 | Gm25855 | M_musculus | Animals | mono_exonic | C/D | ENSMUSG00000108414 | Snhg1 | non_coding |
| ENSMUSG00000064722 | Gm25857 | M_musculus | Animals | mono_intergenic | H/ACA | | | intergenic |
| ENSMUSG00000064724 | Gm25852 | M_musculus | Animals | mono_intronic | H/ACA | ENSMUSG00000030064 | Frmd4b | protein_coding |
| ENSMUSG00000064725 | Snord96a | M_musculus | Animals | mono_intronic | C/D | ENSMUSG00000020372 | Rack1 | protein_coding |
| ENSMUSG00000064726 | Gm25854 | M_musculus | Animals | mono_intronic | C/D | ENSMUSG00000098202 | B830012L14Rik | non_coding |
| ENSMUSG00000064727 | Gm25853 | M_musculus | Animals | mono_intergenic | H/ACA | | | intergenic |
| ENSMUSG00000064731 | Snord45b | M_musculus | Animals | mono_intronic | C/D | ENSMUSG00000038975 | Rabggtb | protein_coding |
| ENSMUSG00000064741 | Snord14a | M_musculus | Animals | mono_intronic | C/D | ENSMUSG00000090862 | Rps13 | protein_coding |
| ENSMUSG00000064751 | Gm26330 | M_musculus | Animals | mono_intronic | C/D | ENSMUSG00000047675 | Rps8 | protein_coding |
| ENSMUSG00000064761 | Gm23514 | M_musculus | Animals | mono_intergenic | H/ACA | | | intergenic |
| ENSMUSG00000064766 | Gm26458 | M_musculus | Animals | mono_intronic | H/ACA | ENSMUSG00000020193 | Zpbp | protein_coding |
| ENSMUSG00000064767 | Snord35b | M_musculus | Animals | mono_intronic | C/D | ENSMUSG00000003429 | Rps11 | protein_coding |
| ENSMUSG00000064768 | Snord60 | M_musculus | Animals | mono_exonic | C/D | ENSMUSG00000093565 | Rab26os | non_coding |
| ENSMUSG00000064776 | Gm25203 | M_musculus | Animals | intergenic_cluster | C/D | | | intergenic |
| ENSMUSG00000064778 | Snord59a | M_musculus | Animals | mono_intronic | C/D | ENSMUSG00000025393 | Atp5b | protein_coding |
| ENSMUSG00000064780 | Snord95 | M_musculus | Animals | mono_intronic | C/D | ENSMUSG00000020372 | Rack1 | protein_coding |
| ENSMUSG00000064783 | Gm22684 | M_musculus | Animals | mono_intronic | H/ACA | ENSMUSG00000118150 | Gm3728 | non_coding |
| ENSMUSG00000064788 | Gm22683 | M_musculus | Animals | mono_intergenic | H/ACA | | | intergenic |
| ENSMUSG00000064792 | Gm24355 | M_musculus | Animals | mono_intronic | H/ACA | ENSMUSG00000029776 | Hibadh | protein_coding |
| ENSMUSG00000064797 | Gm24357 | M_musculus | Animals | mono_exonic | C/D | ENSMUSG00000031939 | Taf1d | protein_coding |
| ENSMUSG00000064800 | Gm24948 | M_musculus | Animals | mono_intergenic | H/ACA | | | intergenic |
| ENSMUSG00000064811 | Gm22358 | M_musculus | Animals | mono_intergenic | H/ACA | | | intergenic |
| ENSMUSG00000064814 | Gm23939 | M_musculus | Animals | mono_intergenic | H/ACA | | | intergenic |
| ENSMUSG00000064816 | Gm22357 | M_musculus | Animals | mono_intronic | C/D | ENSMUSG00000053332 | Gas5 | non_coding |
| ENSMUSG00000064823 | Snord82 | M_musculus | Animals | mono_intronic | C/D | ENSMUSG00000026234 | Ncl | protein_coding |
| ENSMUSG00000064829 | Gm22879 | M_musculus | Animals | mono_intronic | C/D | ENSMUSG00000062647 | Rpl7a | protein_coding |
| ENSMUSG00000064836 | Gm25697 | M_musculus | Animals | mono_intergenic | H/ACA | | | intergenic |
| ENSMUSG00000064837 | Snora75 | M_musculus | Animals | mono_intronic | H/ACA | ENSMUSG00000026234 | Ncl | protein_coding |
| ENSMUSG00000064841 | Gm26205 | M_musculus | Animals | mono_intronic | H/ACA | ENSMUSG00000029614 | Rpl6 | protein_coding |
| ENSMUSG00000064844 | Gm26202 | M_musculus | Animals | mono_intronic | C/D | ENSMUSG00000062328 | Rpl17 | protein_coding |
| ENSMUSG00000064850 | Gm23443 | M_musculus | Animals | mono_intronic | H/ACA | ENSMUSG00000084833 | Gm12649 | non_coding |
| ENSMUSG00000064853 | Gm23442 | M_musculus | Animals | mono_intronic | H/ACA | ENSMUSG00000024393 | Prrc2a | protein_coding |
| ENSMUSG00000064858 | Snora43 | M_musculus | Animals | intronic_cluster | H/ACA | ENSMUSG00000120369 | Snhg7 | non_coding |
| ENSMUSG00000064862 | Gm25038 | M_musculus | Animals | mono_intergenic | H/ACA | | | intergenic |
| ENSMUSG00000064871 | Snord58b | M_musculus | Animals | mono_intronic | C/D | ENSMUSG00000062328 | Rpl17 | protein_coding |
| ENSMUSG00000064877 | Gm23377 | M_musculus | Animals | intergenic_cluster | H/ACA | | | intergenic |
| ENSMUSG00000064880 | Gm24201 | M_musculus | Animals | mono_intronic | H/ACA | ENSMUSG00000022884 | Eif4a2 | protein_coding |
| ENSMUSG00000064897 | Gm22504 | M_musculus | Animals | mono_intergenic | H/ACA | | | intergenic |
| ENSMUSG00000064899 | Snord118 | M_musculus | Animals | mono_intergenic | C/D | | | intergenic |

| | | | | | | | | |
|---|---|---|---|---|---|---|---|---|
| ENSMUSG00000064900 | Gm25402 | M_musculus | Animals | mono_intergenic | H/ACA | | | intergenic |
| ENSMUSG00000064901 | Snora21 | M_musculus | Animals | mono_intronic | H/ACA | ENSMUSG00000071415 | Rpl23 | protein_coding |
| ENSMUSG00000064918 | Gm22571 | M_musculus | Animals | mono_intronic | C/D | ENSMUSG00000032399 | Rpl4 | protein_coding |
| ENSMUSG00000064925 | Snora62 | M_musculus | Animals | mono_intronic | H/ACA | ENSMUSG00000032518 | Rpsa | protein_coding |
| ENSMUSG00000064930 | Gm23723 | M_musculus | Animals | mono_intronic | H/ACA | ENSMUSG00000027939 | Nup210l | protein_coding |
| ENSMUSG00000064936 | Gm23722 | M_musculus | Animals | mono_intronic | H/ACA | ENSMUSG00000048234 | Rnf149 | protein_coding |
| ENSMUSG00000064949 | Snora61 | M_musculus | Animals | mono_intronic | H/ACA | ENSMUSG00000086290 | Snhg12 | non_coding |
| ENSMUSG00000064952 | Gm24920 | M_musculus | Animals | mono_intronic | C/D | ENSMUSG00000028126 | Pip5k1a | protein_coding |
| ENSMUSG00000064956 | Gm24918 | M_musculus | Animals | mono_intronic | H/ACA | ENSMUSG00000025899 | Alkbh8 | protein_coding |
| ENSMUSG00000064961 | Gm22704 | M_musculus | Animals | mono_intergenic | H/ACA | | | intergenic |
| ENSMUSG00000064966 | Snord15b | M_musculus | Animals | mono_intronic | C/D | ENSMUSG00000030744 | Rps3 | protein_coding |
| ENSMUSG00000064968 | Snord47 | M_musculus | Animals | mono_intronic | C/D | ENSMUSG00000053332 | Gas5 | non_coding |
| ENSMUSG00000064984 | Snord73a | M_musculus | Animals | mono_intronic | C/D | ENSMUSG00000028081 | Rps3a1 | protein_coding |
| ENSMUSG00000064994 | Gm22422 | M_musculus | Animals | mono_intronic | H/ACA | ENSMUSG00000044763 | Trmt10c | protein_coding |
| ENSMUSG00000064995 | Gm26003 | M_musculus | Animals | mono_intronic | H/ACA | ENSMUSG00000085385 | Snhg17 | non_coding |
| ENSMUSG00000065004 | Gm26326 | M_musculus | Animals | mono_intergenic | H/ACA | | | intergenic |
| ENSMUSG00000065013 | Gm23508 | M_musculus | Animals | mono_intronic | C/D | ENSMUSG00000097451 | Rian | non_coding |
| ENSMUSG00000065016 | Snora3 | M_musculus | Animals | mono_intronic | H/ACA | ENSMUSG00000046364 | Rpl27a | protein_coding |
| ENSMUSG00000065022 | AF357341 | M_musculus | Animals | mono_intronic | C/D | ENSMUSG00000098202 | B830012L14Rik | non_coding |
| ENSMUSG00000065028 | Gm25200 | M_musculus | Animals | mono_intergenic | H/ACA | | | intergenic |
| ENSMUSG00000065036 | Gm22362 | M_musculus | Animals | mono_intronic | H/ACA | ENSMUSG00000030189 | Ybx3 | protein_coding |
| ENSMUSG00000065041 | Gm24044 | M_musculus | Animals | mono_intronic | H/ACA | ENSMUSG00000032518 | Rpsa | protein_coding |
| ENSMUSG00000065061 | Gm22884 | M_musculus | Animals | mono_intronic | H/ACA | ENSMUSG00000018583 | G3bp1 | protein_coding |
| ENSMUSG00000065072 | Gm24533 | M_musculus | Animals | mono_intronic | H/ACA | ENSMUSG00000052861 | Dnah6 | protein_coding |
| ENSMUSG00000065086 | Gm26495 | M_musculus | Animals | mono_intronic | H/ACA | ENSMUSG00000020154 | Ptprb | protein_coding |
| ENSMUSG00000065087 | Snord22 | M_musculus | Animals | mono_exonic | C/D | ENSMUSG00000108414 | Snhg1 | non_coding |
| ENSMUSG00000065089 | Gm26493 | M_musculus | Animals | mono_intronic | H/ACA | ENSMUSG00000036550 | Cnot1 | protein_coding |
| ENSMUSG00000065094 | Snord1a | M_musculus | Animals | mono_intronic | C/D | ENSMUSG00000020812 | Snhg16 | non_coding |
| ENSMUSG00000065097 | Snora16a | M_musculus | Animals | intronic_cluster | H/ACA | ENSMUSG00000086290 | Snhg12 | non_coding |
| ENSMUSG00000065100 | Gm26132 | M_musculus | Animals | mono_intronic | H/ACA | ENSMUSG00000031749 | St3gal2 | protein_coding |
| ENSMUSG00000065105 | Gm26130 | M_musculus | Animals | mono_intronic | H/ACA | ENSMUSG00000068039 | Tcp1 | protein_coding |
| ENSMUSG00000065110 | Snord61 | M_musculus | Animals | mono_intronic | C/D | ENSMUSG00000031134 | Rbmx | protein_coding |
| ENSMUSG00000065118 | Gm23297 | M_musculus | Animals | mono_intronic | H/ACA | ENSMUSG00000059119 | Nap1l4 | protein_coding |
| ENSMUSG00000065124 | Snora65 | M_musculus | Animals | mono_intronic | H/ACA | ENSMUSG00000038900 | Rpl12 | protein_coding |
| ENSMUSG00000065126 | Snord104 | M_musculus | Animals | mono_intronic | C/D | ENSMUSG00000120012 | Gm36220 | non_coding |
| ENSMUSG00000065141 | Gm22652 | M_musculus | Animals | mono_intergenic | H/ACA | | | intergenic |
| ENSMUSG00000065147 | Snora31 | M_musculus | Animals | mono_intronic | H/ACA | ENSMUSG00000060126 | Tpt1 | protein_coding |
| ENSMUSG00000065151 | Gm25480 | M_musculus | Animals | mono_intergenic | H/ACA | | | intergenic |
| ENSMUSG00000065160 | Gm50452 | M_musculus | Animals | mono_intronic | C/D | ENSMUSG00000053332 | Gas5 | non_coding |
| ENSMUSG00000065181 | Gm25139 | M_musculus | Animals | mono_intergenic | H/ACA | | | intergenic |
| ENSMUSG00000065182 | Gm25138 | M_musculus | Animals | mono_intergenic | H/ACA | | | intergenic |

| | | | | | | | | | |
|---|---|---|---|---|---|---|---|---|---|
| ENSMUSG00000065187 | Gm25136 | M_musculus | Animals | mono_intergenic | H/ACA | | | intergenic |
| ENSMUSG00000065196 | Snord85 | M_musculus | Animals | mono_intronic | C/D | ENSMUSG00000028580 | Pum1 | protein_coding |
| ENSMUSG00000065200 | Gm24613 | M_musculus | Animals | mono_intergenic | H/ACA | | | intergenic |
| ENSMUSG00000065202 | Gm24615 | M_musculus | Animals | mono_intergenic | H/ACA | | | intergenic |
| ENSMUSG00000065204 | Gm24610 | M_musculus | Animals | mono_intronic | H/ACA | ENSMUSG00000025227 | Mfsd13a | protein_coding |
| ENSMUSG00000065208 | Gm24616 | M_musculus | Animals | mono_intronic | H/ACA | ENSMUSG00000022884 | Eif4a2 | protein_coding |
| ENSMUSG00000065211 | Gm26265 | M_musculus | Animals | intronic_cluster | H/ACA | ENSMUSG00000036550 | Cnot1 | protein_coding |
| ENSMUSG00000065219 | Snord32a | M_musculus | Animals | mono_intronic | C/D | ENSMUSG00000074129 | Rpl13a | protein_coding |
| ENSMUSG00000065226 | Gm25791 | M_musculus | Animals | mono_intronic | H/ACA | ENSMUSG00000031939 | Taf1d | protein_coding |
| ENSMUSG00000065228 | Gm25789 | M_musculus | Animals | mono_intronic | C/D | ENSMUSG00000053332 | Gas5 | non_coding |
| ENSMUSG00000065231 | Gm22972 | M_musculus | Animals | mono_intronic | H/ACA | ENSMUSG00000053687 | Dpep2 | protein_coding |
| ENSMUSG00000065248 | Gm22293 | M_musculus | Animals | mono_intergenic | H/ACA | | | intergenic |
| ENSMUSG00000065255 | Gm23974 | M_musculus | Animals | mono_intergenic | H/ACA | | | intergenic |
| ENSMUSG00000065258 | Gm23969 | M_musculus | Animals | mono_intronic | C/D | ENSMUSG00000062647 | Rpl7a | protein_coding |
| ENSMUSG00000065259 | Snora30 | M_musculus | Animals | mono_intronic | H/ACA | ENSMUSG00000053877 | Srcap | protein_coding |
| ENSMUSG00000065262 | Gm23451 | M_musculus | Animals | mono_intronic | H/ACA | ENSMUSG00000020914 | Top2a | protein_coding |
| ENSMUSG00000065265 | Gm23455 | M_musculus | Animals | mono_exonic | H/ACA | ENSMUSG00000031939 | Taf1d | protein_coding |
| ENSMUSG00000065266 | Gm23454 | M_musculus | Animals | mono_intergenic | H/ACA | | | intergenic |
| ENSMUSG00000065272 | Snord57 | M_musculus | Animals | mono_intronic | C/D | ENSMUSG00000027405 | Nop56 | protein_coding |
| ENSMUSG00000065273 | Gm25128 | M_musculus | Animals | mono_intronic | C/D | ENSMUSG00000019432 | Ddx39b | protein_coding |
| ENSMUSG00000065274 | Gm25125 | M_musculus | Animals | mono_intergenic | H/ACA | | | intergenic |
| ENSMUSG00000065280 | Gm24453 | M_musculus | Animals | mono_intronic | C/D | ENSMUSG00000108414 | Snhg1 | non_coding |
| ENSMUSG00000065281 | Gm24452 | M_musculus | Animals | mono_exonic | C/D | ENSMUSG00000108414 | Snhg1 | non_coding |
| ENSMUSG00000065282 | Gm24455 | M_musculus | Animals | mono_exonic | H/ACA | ENSMUSG00000031939 | Taf1d | protein_coding |
| ENSMUSG00000065283 | Gm24454 | M_musculus | Animals | mono_intergenic | H/ACA | | | intergenic |
| ENSMUSG00000065287 | Gm24451 | M_musculus | Animals | mono_intronic | H/ACA | ENSMUSG00000027405 | Nop56 | protein_coding |
| ENSMUSG00000065289 | Gm23650 | M_musculus | Animals | mono_intronic | C/D | ENSMUSG00000027405 | Nop56 | protein_coding |
| ENSMUSG00000065295 | Gm24134 | M_musculus | Animals | mono_intronic | C/D | ENSMUSG00000062647 | Rpl7a | protein_coding |
| ENSMUSG00000065297 | Gm26123 | M_musculus | Animals | mono_intronic | H/ACA | ENSMUSG00000036469 | Marchf1 | protein_coding |
| ENSMUSG00000065304 | Gm23245 | M_musculus | Animals | mono_intronic | H/ACA | ENSMUSG00000029447 | Cct6a | protein_coding |
| ENSMUSG00000065305 | Gm23246 | M_musculus | Animals | mono_intronic | C/D | ENSMUSG00000108414 | Snhg1 | non_coding |
| ENSMUSG00000065328 | Gm22053 | M_musculus | Animals | mono_intergenic | H/ACA | | | intergenic |
| ENSMUSG00000065346 | Gm25582 | M_musculus | Animals | mono_intergenic | H/ACA | | | intergenic |
| ENSMUSG00000065353 | Snora73b | M_musculus | Animals | mono_intronic | H/ACA | ENSMUSG00000028896 | Rcc1 | protein_coding |
| ENSMUSG00000065360 | Gm24412 | M_musculus | Animals | mono_intronic | H/ACA | ENSMUSG00000025133 | Ints4 | protein_coding |
| ENSMUSG00000065362 | Gm24411 | M_musculus | Animals | mono_intronic | H/ACA | ENSMUSG00000085385 | Snhg17 | non_coding |
| ENSMUSG00000065378 | Gm22744 | M_musculus | Animals | mono_intronic | C/D | ENSMUSG00000108414 | Snhg1 | non_coding |
| ENSMUSG00000065379 | Gm22743 | M_musculus | Animals | mono_intronic | H/ACA | ENSMUSG00000085134 | Gm11659 | non_coding |
| ENSMUSG00000065392 | Gm25894 | M_musculus | Animals | mono_intronic | C/D | ENSMUSG00000108414 | Snhg1 | non_coding |
| ENSMUSG00000065463 | Snord45c | M_musculus | Animals | mono_intronic | C/D | ENSMUSG00000038975 | Rabggtb | protein_coding |
| ENSMUSG00000065629 | Gm24826 | M_musculus | Animals | intergenic_cluster | C/D | | | intergenic |

| | | | | | | | | |
|---|---|---|---|---|---|---|---|---|
| ENSMUSG00000065634 | Gm24252 | M_musculus | Animals | mono_intergenic | H/ACA | | | intergenic |
| ENSMUSG00000065635 | Gm24253 | M_musculus | Animals | mono_intergenic | C/D | | | intergenic |
| ENSMUSG00000065637 | Gm26397 | M_musculus | Animals | mono_intronic | H/ACA | ENSMUSG00000093904 | Tomm20 | protein_coding |
| ENSMUSG00000065640 | Snord1c | M_musculus | Animals | mono_intronic | C/D | ENSMUSG00000020812 | Snhg16 | non_coding |
| ENSMUSG00000065642 | Snora69 | M_musculus | Animals | mono_intronic | H/ACA | ENSMUSG00000079641 | Rpl39 | protein_coding |
| ENSMUSG00000065647 | Gm25896 | M_musculus | Animals | mono_intronic | H/ACA | ENSMUSG00000052299 | Ltn1 | protein_coding |
| ENSMUSG00000065649 | Snora74a | M_musculus | Animals | mono_intronic | H/ACA | ENSMUSG00000117694 | Snhg4 | protein_coding |
| ENSMUSG00000065651 | Gm25973 | M_musculus | Animals | mono_intergenic | H/ACA | | | intergenic |
| ENSMUSG00000065653 | Gm23105 | M_musculus | Animals | mono_intronic | H/ACA | ENSMUSG00000035873 | Pawr | protein_coding |
| ENSMUSG00000065663 | Gm22579 | M_musculus | Animals | mono_exonic | H/ACA | ENSMUSG00000031939 | Taf1d | protein_coding |
| ENSMUSG00000065669 | Gm22581 | M_musculus | Animals | mono_intronic | H/ACA | ENSMUSG00000028099 | Polr3c | protein_coding |
| ENSMUSG00000065673 | Gm24258 | M_musculus | Animals | mono_intergenic | H/ACA | | | intergenic |
| ENSMUSG00000065676 | Snord42b | M_musculus | Animals | mono_intronic | C/D | ENSMUSG00000058546 | Rpl23a | protein_coding |
| ENSMUSG00000065680 | Snord38a | M_musculus | Animals | mono_intronic | C/D | ENSMUSG00000047675 | Rps8 | protein_coding |
| ENSMUSG00000065686 | Snora5c | M_musculus | Animals | mono_intronic | H/ACA | ENSMUSG00000000384 | Tbrg4 | protein_coding |
| ENSMUSG00000065687 | Gm23734 | M_musculus | Animals | mono_intronic | C/D | ENSMUSG00000036155 | Mgat5 | protein_coding |
| ENSMUSG00000065692 | Gm25410 | M_musculus | Animals | mono_intronic | H/ACA | ENSMUSG00000015305 | Sash1 | protein_coding |
| ENSMUSG00000065698 | Gm25409 | M_musculus | Animals | mono_intergenic | H/ACA | | | intergenic |
| ENSMUSG00000065706 | Gm22830 | M_musculus | Animals | mono_intergenic | H/ACA | | | intergenic |
| ENSMUSG00000065709 | Gm22341 | M_musculus | Animals | mono_intergenic | H/ACA | | | intergenic |
| ENSMUSG00000065715 | Snord7 | M_musculus | Animals | mono_intronic | C/D | ENSMUSG00000093483 | AA465934 | non_coding |
| ENSMUSG00000065716 | Gm25651 | M_musculus | Animals | mono_intergenic | H/ACA | | | intergenic |
| ENSMUSG00000065725 | Gm26165 | M_musculus | Animals | mono_intronic | H/ACA | ENSMUSG00000027423 | Snx5 | protein_coding |
| ENSMUSG00000065726 | Gm26163 | M_musculus | Animals | mono_intergenic | H/ACA | | | intergenic |
| ENSMUSG00000065728 | Gm26175 | M_musculus | Animals | mono_intronic | H/ACA | ENSMUSG00000030059 | Tmf1 | protein_coding |
| ENSMUSG00000065734 | Snord49a | M_musculus | Animals | mono_intronic | C/D | ENSMUSG00000086841 | 2410006H16Rik | non_coding |
| ENSMUSG00000065735 | Snord80 | M_musculus | Animals | intronic_cluster | C/D | ENSMUSG00000053332 | Gas5 | non_coding |
| ENSMUSG00000065738 | Gm24494 | M_musculus | Animals | mono_intronic | C/D | ENSMUSG00000038975 | Rabggtb | protein_coding |
| ENSMUSG00000065749 | AF357428 | M_musculus | Animals | mono_intronic | C/D | ENSMUSG00000098202 | B830012L14Rik | non_coding |
| ENSMUSG00000065750 | Gm23346 | M_musculus | Animals | mono_intronic | H/ACA | ENSMUSG00000031668 | Eif2ak3 | protein_coding |
| ENSMUSG00000065752 | Gm23344 | M_musculus | Animals | mono_intronic | C/D | ENSMUSG00000032399 | Rpl4 | protein_coding |
| ENSMUSG00000065757 | Gm23347 | M_musculus | Animals | mono_intergenic | C/D | | | intergenic |
| ENSMUSG00000065778 | Gm22154 | M_musculus | Animals | mono_intronic | H/ACA | ENSMUSG00000011257 | Pabpc4 | protein_coding |
| ENSMUSG00000065782 | Gm22680 | M_musculus | Animals | mono_intronic | C/D | ENSMUSG00000108414 | Snhg1 | non_coding |
| ENSMUSG00000065799 | Gm25517 | M_musculus | Animals | mono_intronic | H/ACA | ENSMUSG00000067995 | Gtf2f2 | protein_coding |
| ENSMUSG00000065812 | Gm24696 | M_musculus | Animals | mono_intergenic | H/ACA | | | intergenic |
| ENSMUSG00000065817 | Gm24698 | M_musculus | Animals | mono_intronic | H/ACA | ENSMUSG00000047710 | Champ1 | protein_coding |
| ENSMUSG00000065818 | Snord35a | M_musculus | Animals | mono_intronic | C/D | ENSMUSG00000074129 | Rpl13a | protein_coding |
| ENSMUSG00000065822 | Snord15a | M_musculus | Animals | mono_intronic | C/D | ENSMUSG00000030744 | Rps3 | protein_coding |
| ENSMUSG00000065840 | Gm25187 | M_musculus | Animals | intronic_cluster | H/ACA | ENSMUSG00000044349 | Snhg11 | protein_coding |
| ENSMUSG00000065847 | Gm25188 | M_musculus | Animals | mono_intronic | H/ACA | ENSMUSG00000028082 | Sh3d19 | protein_coding |

| | | | | | | | | |
|---|---|---|---|---|---|---|---|---|
| ENSMUSG00000065852 | Gm22353 | M_musculus | Animals | mono_intergenic | H/ACA | | | intergenic |
| ENSMUSG00000065853 | Gm22354 | M_musculus | Animals | mono_intergenic | C/D | | | intergenic |
| ENSMUSG00000065862 | Gm24029 | M_musculus | Animals | mono_intronic | H/ACA | ENSMUSG00000059796 | Eif4a1 | protein_coding |
| ENSMUSG00000065864 | Gm24031 | M_musculus | Animals | mono_intergenic | H/ACA | | | intergenic |
| ENSMUSG00000065874 | Gm25683 | M_musculus | Animals | mono_intergenic | H/ACA | | | intergenic |
| ENSMUSG00000065878 | Snord34 | M_musculus | Animals | mono_intronic | C/D | ENSMUSG00000074129 | Rpl13a | protein_coding |
| ENSMUSG00000065883 | Snord3b-ps2 | M_musculus | Animals | mono_intronic | C/D | ENSMUSG00000054484 | Tmem62 | protein_coding |
| ENSMUSG00000065899 | Gm24523 | M_musculus | Animals | mono_intronic | H/ACA | ENSMUSG00000058600 | Rpl30 | protein_coding |
| ENSMUSG00000065904 | Gm26109 | M_musculus | Animals | mono_intronic | C/D | ENSMUSG00000024359 | Hspa9 | protein_coding |
| ENSMUSG00000065924 | Gm22797 | M_musculus | Animals | mono_intergenic | H/ACA | | | intergenic |
| ENSMUSG00000065939 | Snora2b | M_musculus | Animals | mono_intronic | H/ACA | ENSMUSG00000022992 | Kansl2 | protein_coding |
| ENSMUSG00000065940 | Gm23795 | M_musculus | Animals | mono_intergenic | H/ACA | | | intergenic |
| ENSMUSG00000070063 | Snora33 | M_musculus | Animals | mono_intronic | H/ACA | ENSMUSG00000061983 | Rps12 | protein_coding |
| ENSMUSG00000070166 | Gm25821 | M_musculus | Animals | mono_intronic | H/ACA | ENSMUSG00000054309 | Cpsf3 | protein_coding |
| ENSMUSG00000070167 | Snora57 | M_musculus | Animals | mono_intergenic | H/ACA | | | intergenic |
| ENSMUSG00000070170 | Gm24163 | M_musculus | Animals | mono_intergenic | H/ACA | | | intergenic |
| ENSMUSG00000075726 | Gm23254 | M_musculus | Animals | mono_intergenic | C/D | ENSMUSG00000100826 | Snhg14 | non_coding |
| ENSMUSG00000075770 | Gm22589 | M_musculus | Animals | mono_intronic | C/D | ENSMUSG00000019432 | Ddx39b | protein_coding |
| ENSMUSG00000075968 | Gm25089 | M_musculus | Animals | mono_intergenic | C/D | ENSMUSG00000100826 | Snhg14 | non_coding |
| ENSMUSG00000077142 | Gm22937 | M_musculus | Animals | mono_intergenic | H/ACA | | | intergenic |
| ENSMUSG00000077143 | Gm22936 | M_musculus | Animals | mono_intergenic | H/ACA | | | intergenic |
| ENSMUSG00000077144 | Gm22938 | M_musculus | Animals | mono_intergenic | H/ACA | | | intergenic |
| ENSMUSG00000077146 | Gm22939 | M_musculus | Animals | mono_intergenic | H/ACA | | | intergenic |
| ENSMUSG00000077149 | Gm22934 | M_musculus | Animals | mono_intergenic | H/ACA | | | intergenic |
| ENSMUSG00000077150 | Gm24581 | M_musculus | Animals | mono_intergenic | H/ACA | | | intergenic |
| ENSMUSG00000077152 | Gm24579 | M_musculus | Animals | mono_intergenic | H/ACA | | | intergenic |
| ENSMUSG00000077153 | Gm24580 | M_musculus | Animals | mono_intergenic | H/ACA | | | intergenic |
| ENSMUSG00000077158 | Gm24578 | M_musculus | Animals | mono_intergenic | H/ACA | | | intergenic |
| ENSMUSG00000077160 | Gm24118 | M_musculus | Animals | mono_intergenic | H/ACA | | | intergenic |
| ENSMUSG00000077161 | Gm24117 | M_musculus | Animals | mono_intergenic | H/ACA | | | intergenic |
| ENSMUSG00000077166 | Gm24120 | M_musculus | Animals | mono_intergenic | H/ACA | | | intergenic |
| ENSMUSG00000077167 | Gm24119 | M_musculus | Animals | mono_intronic | H/ACA | ENSMUSG00000061904 | Slc25a3 | protein_coding |
| ENSMUSG00000077169 | Gm25396 | M_musculus | Animals | mono_intergenic | H/ACA | | | intergenic |
| ENSMUSG00000077171 | Gm25761 | M_musculus | Animals | mono_intergenic | H/ACA | | | intergenic |
| ENSMUSG00000077172 | Gm25762 | M_musculus | Animals | mono_intergenic | H/ACA | | | intergenic |
| ENSMUSG00000077174 | Gm25757 | M_musculus | Animals | mono_intergenic | H/ACA | | | intergenic |
| ENSMUSG00000077175 | Gm25758 | M_musculus | Animals | mono_intergenic | H/ACA | | | intergenic |
| ENSMUSG00000077176 | Gm25759 | M_musculus | Animals | mono_intronic | H/ACA | ENSMUSG00000078591 | Hs3st4 | protein_coding |
| ENSMUSG00000077177 | Gm25760 | M_musculus | Animals | mono_intronic | H/ACA | ENSMUSG00000098172 | Gm26973 | non_coding |
| ENSMUSG00000077178 | Gm25756 | M_musculus | Animals | mono_intergenic | H/ACA | | | intergenic |
| ENSMUSG00000077180 | Gm25094 | M_musculus | Animals | mono_intergenic | H/ACA | | | intergenic |

| | | | | | | | | |
|---|---|---|---|---|---|---|---|---|
| ENSMUSG00000077181 | Gm25093 | M_musculus | Animals | mono_intergenic | H/ACA | | | intergenic |
| ENSMUSG00000077184 | Gm25092 | M_musculus | Animals | mono_intergenic | H/ACA | | | intergenic |
| ENSMUSG00000077185 | Gm25091 | M_musculus | Animals | mono_intronic | C/D | ENSMUSG00000041453 | Rpl21 | protein_coding |
| ENSMUSG00000077191 | Snord64 | M_musculus | Animals | mono_intronic | C/D | ENSMUSG00000000948 | Gm38393 | protein_coding |
| ENSMUSG00000077192 | Snora17 | M_musculus | Animals | mono_intronic | H/ACA | ENSMUSG00000120369 | Snhg7 | non_coding |
| ENSMUSG00000077196 | Gm22267 | M_musculus | Animals | mono_intergenic | H/ACA | | | intergenic |
| ENSMUSG00000077197 | Gm22268 | M_musculus | Animals | mono_intergenic | H/ACA | | | intergenic |
| ENSMUSG00000077199 | Gm22266 | M_musculus | Animals | mono_intergenic | H/ACA | | | intergenic |
| ENSMUSG00000077200 | Gm25613 | M_musculus | Animals | mono_intergenic | H/ACA | | | intergenic |
| ENSMUSG00000077201 | Gm25614 | M_musculus | Animals | mono_intergenic | H/ACA | | | intergenic |
| ENSMUSG00000077202 | Gm25612 | M_musculus | Animals | mono_intronic | H/ACA | ENSMUSG00000042229 | Rabif | protein_coding |
| ENSMUSG00000077204 | Gm25610 | M_musculus | Animals | mono_intergenic | H/ACA | | | intergenic |
| ENSMUSG00000077205 | Gm25611 | M_musculus | Animals | mono_intronic | H/ACA | ENSMUSG00000118212 | Gm41836 | non_coding |
| ENSMUSG00000077208 | Gm25607 | M_musculus | Animals | mono_intergenic | H/ACA | | | intergenic |
| ENSMUSG00000077209 | Gm25608 | M_musculus | Animals | mono_intergenic | H/ACA | | | intergenic |
| ENSMUSG00000077212 | Snord69 | M_musculus | Animals | mono_intronic | C/D | ENSMUSG00000042354 | Gnl3 | protein_coding |
| ENSMUSG00000077214 | Gm23950 | M_musculus | Animals | mono_intergenic | H/ACA | | | intergenic |
| ENSMUSG00000077215 | Gm23949 | M_musculus | Animals | mono_intergenic | H/ACA | | | intergenic |
| ENSMUSG00000077217 | Gm23951 | M_musculus | Animals | mono_intergenic | H/ACA | | | intergenic |
| ENSMUSG00000077220 | Snord78 | M_musculus | Animals | mono_exonic | C/D | ENSMUSG00000053332 | Gas5 | non_coding |
| ENSMUSG00000077221 | Snord67 | M_musculus | Animals | mono_intronic | C/D | ENSMUSG00000040549 | Ckap5 | protein_coding |
| ENSMUSG00000077222 | Gm22270 | M_musculus | Animals | mono_intronic | H/ACA | ENSMUSG00000058558 | Rpl5 | protein_coding |
| ENSMUSG00000077223 | Gm22271 | M_musculus | Animals | mono_intergenic | H/ACA | | | intergenic |
| ENSMUSG00000077224 | Gm23437 | M_musculus | Animals | mono_intergenic | H/ACA | | | intergenic |
| ENSMUSG00000077227 | Gm22272 | M_musculus | Animals | mono_intergenic | H/ACA | | | intergenic |
| ENSMUSG00000077228 | Gm22273 | M_musculus | Animals | mono_intergenic | H/ACA | | | intergenic |
| ENSMUSG00000077231 | Gm25096 | M_musculus | Animals | mono_intergenic | H/ACA | | | intergenic |
| ENSMUSG00000077235 | Gm25095 | M_musculus | Animals | mono_intergenic | H/ACA | | | intergenic |
| ENSMUSG00000077237 | Gm22438 | M_musculus | Animals | mono_intergenic | H/ACA | | | intergenic |
| ENSMUSG00000077239 | Snord66 | M_musculus | Animals | mono_intronic | C/D | ENSMUSG00000045983 | Eif4g1 | protein_coding |
| ENSMUSG00000077240 | Gm23271 | M_musculus | Animals | mono_intergenic | H/ACA | | | intergenic |
| ENSMUSG00000077241 | Gm23272 | M_musculus | Animals | mono_intergenic | H/ACA | | | intergenic |
| ENSMUSG00000077243 | Gm23270 | M_musculus | Animals | mono_intergenic | H/ACA | | | intergenic |
| ENSMUSG00000077244 | Gm23274 | M_musculus | Animals | mono_intergenic | H/ACA | | | intergenic |
| ENSMUSG00000077246 | Gm23273 | M_musculus | Animals | mono_intergenic | H/ACA | | | intergenic |
| ENSMUSG00000077250 | Gm26075 | M_musculus | Animals | mono_intronic | H/ACA | ENSMUSG00000087305 | A430035B10Rik | non_coding |
| ENSMUSG00000077251 | Gm26074 | M_musculus | Animals | mono_intergenic | H/ACA | | | intergenic |
| ENSMUSG00000077252 | Gm26077 | M_musculus | Animals | mono_intergenic | H/ACA | | | intergenic |
| ENSMUSG00000077254 | Gm26079 | M_musculus | Animals | mono_intronic | H/ACA | ENSMUSG00000029505 | Ep400 | protein_coding |
| ENSMUSG00000077255 | Gm26078 | M_musculus | Animals | mono_intergenic | H/ACA | | | intergenic |
| ENSMUSG00000077256 | Gm26081 | M_musculus | Animals | mono_intergenic | H/ACA | | | intergenic |

| | | | | | | | | |
|---|---|---|---|---|---|---|---|---|
| ENSMUSG00000077257 | Gm26080 | M_musculus | Animals | mono_intergenic | H/ACA | | | intergenic |
| ENSMUSG00000077258 | Gm26082 | M_musculus | Animals | mono_intergenic | H/ACA | | | intergenic |
| ENSMUSG00000077259 | Gm23979 | M_musculus | Animals | mono_intergenic | H/ACA | | | intergenic |
| ENSMUSG00000077260 | Gm24869 | M_musculus | Animals | mono_intergenic | H/ACA | | | intergenic |
| ENSMUSG00000077263 | Gm24432 | M_musculus | Animals | mono_intergenic | H/ACA | | | intergenic |
| ENSMUSG00000077264 | Gm24429 | M_musculus | Animals | mono_intergenic | H/ACA | | | intergenic |
| ENSMUSG00000077268 | Gm24433 | M_musculus | Animals | mono_intergenic | H/ACA | | | intergenic |
| ENSMUSG00000077269 | Gm24434 | M_musculus | Animals | mono_intergenic | H/ACA | | | intergenic |
| ENSMUSG00000077271 | Gm22781 | M_musculus | Animals | mono_intergenic | H/ACA | | | intergenic |
| ENSMUSG00000077274 | Gm22786 | M_musculus | Animals | mono_intronic | H/ACA | ENSMUSG00000026229 | Psmd1 | protein_coding |
| ENSMUSG00000077275 | Gm22785 | M_musculus | Animals | mono_intergenic | H/ACA | | | intergenic |
| ENSMUSG00000077276 | Gm22784 | M_musculus | Animals | mono_intronic | H/ACA | ENSMUSG00000027799 | Nbea | protein_coding |
| ENSMUSG00000077278 | Gm22778 | M_musculus | Animals | mono_intergenic | H/ACA | | | intergenic |
| ENSMUSG00000077279 | Gm22777 | M_musculus | Animals | mono_intergenic | H/ACA | | | intergenic |
| ENSMUSG00000077280 | Gm25764 | M_musculus | Animals | mono_intergenic | H/ACA | | | intergenic |
| ENSMUSG00000077282 | Gm26395 | M_musculus | Animals | mono_intronic | H/ACA | ENSMUSG00000021420 | Fars2 | protein_coding |
| ENSMUSG00000077287 | Gm25763 | M_musculus | Animals | mono_intergenic | H/ACA | | | intergenic |
| ENSMUSG00000077288 | Gm25765 | M_musculus | Animals | mono_intergenic | H/ACA | | | intergenic |
| ENSMUSG00000077289 | Gm25766 | M_musculus | Animals | mono_intergenic | H/ACA | | | intergenic |
| ENSMUSG00000077290 | Gm25407 | M_musculus | Animals | mono_intergenic | H/ACA | | | intergenic |
| ENSMUSG00000077291 | Gm24124 | M_musculus | Animals | mono_intergenic | H/ACA | | | intergenic |
| ENSMUSG00000077292 | Gm25408 | M_musculus | Animals | mono_intergenic | H/ACA | | | intergenic |
| ENSMUSG00000077293 | Gm24125 | M_musculus | Animals | mono_intergenic | H/ACA | | | intergenic |
| ENSMUSG00000077294 | Gm24123 | M_musculus | Animals | mono_intergenic | C/D | | | intergenic |
| ENSMUSG00000077295 | Gm24122 | M_musculus | Animals | mono_intergenic | H/ACA | | | intergenic |
| ENSMUSG00000077298 | Gm24127 | M_musculus | Animals | mono_intergenic | H/ACA | | | intergenic |
| ENSMUSG00000077302 | Gm22473 | M_musculus | Animals | mono_intergenic | H/ACA | | | intergenic |
| ENSMUSG00000077304 | Gm22471 | M_musculus | Animals | mono_intergenic | H/ACA | | | intergenic |
| ENSMUSG00000077305 | Gm22470 | M_musculus | Animals | mono_intronic | H/ACA | ENSMUSG00000087170 | 1700001G01Rik | non_coding |
| ENSMUSG00000077306 | Gm22469 | M_musculus | Animals | mono_intergenic | H/ACA | | | intergenic |
| ENSMUSG00000077312 | Gm24170 | M_musculus | Animals | mono_intergenic | H/ACA | | | intergenic |
| ENSMUSG00000077314 | Gm24171 | M_musculus | Animals | mono_intergenic | H/ACA | | | intergenic |
| ENSMUSG00000077315 | Gm24172 | M_musculus | Animals | mono_intergenic | H/ACA | | | intergenic |
| ENSMUSG00000077318 | Gm24173 | M_musculus | Animals | mono_intergenic | H/ACA | | | intergenic |
| ENSMUSG00000077320 | Gm22284 | M_musculus | Animals | mono_intergenic | H/ACA | | | intergenic |
| ENSMUSG00000077325 | Gm25833 | M_musculus | Animals | mono_intergenic | H/ACA | | | intergenic |
| ENSMUSG00000077328 | Gm25832 | M_musculus | Animals | mono_intergenic | H/ACA | | | intergenic |
| ENSMUSG00000077332 | Gm23014 | M_musculus | Animals | mono_intergenic | H/ACA | | | intergenic |
| ENSMUSG00000077333 | Gm23015 | M_musculus | Animals | mono_intergenic | H/ACA | | | intergenic |
| ENSMUSG00000077336 | Gm23011 | M_musculus | Animals | mono_intergenic | H/ACA | | | intergenic |
| ENSMUSG00000077337 | Gm23012 | M_musculus | Animals | mono_intergenic | H/ACA | | | intergenic |

| | | | | | | | | |
|---|---|---|---|---|---|---|---|---|
| ENSMUSG00000077339 | Gm23010 | M_musculus | Animals | mono_intronic | H/ACA | ENSMUSG00000029925 | Tbxas1 | protein_coding |
| ENSMUSG00000077342 | Gm24670 | M_musculus | Animals | mono_intergenic | H/ACA | | | intergenic |
| ENSMUSG00000077344 | Gm24326 | M_musculus | Animals | mono_intergenic | H/ACA | | | intergenic |
| ENSMUSG00000077345 | Snord70 | M_musculus | Animals | mono_intronic | C/D | ENSMUSG00000026020 | Nop58 | protein_coding |
| ENSMUSG00000077348 | Gm24665 | M_musculus | Animals | mono_intergenic | H/ACA | | | intergenic |
| ENSMUSG00000077354 | Gm26286 | M_musculus | Animals | mono_intergenic | H/ACA | | | intergenic |
| ENSMUSG00000077357 | Gm26289 | M_musculus | Animals | mono_intergenic | H/ACA | | | intergenic |
| ENSMUSG00000077358 | Gm26293 | M_musculus | Animals | mono_intronic | C/D | ENSMUSG00000026020 | Nop58 | protein_coding |
| ENSMUSG00000077360 | Gm23482 | M_musculus | Animals | mono_intronic | H/ACA | ENSMUSG00000037851 | Iars | protein_coding |
| ENSMUSG00000077361 | Gm23481 | M_musculus | Animals | mono_intergenic | H/ACA | | | intergenic |
| ENSMUSG00000077366 | Gm23484 | M_musculus | Animals | mono_intergenic | H/ACA | | | intergenic |
| ENSMUSG00000077368 | Gm26273 | M_musculus | Animals | mono_intergenic | H/ACA | | | intergenic |
| ENSMUSG00000077376 | Gm25167 | M_musculus | Animals | mono_intergenic | H/ACA | | | intergenic |
| ENSMUSG00000077377 | Gm25168 | M_musculus | Animals | mono_intergenic | H/ACA | | | intergenic |
| ENSMUSG00000077378 | Gm25169 | M_musculus | Animals | mono_intergenic | H/ACA | | | intergenic |
| ENSMUSG00000077379 | Gm25170 | M_musculus | Animals | mono_intergenic | H/ACA | | | intergenic |
| ENSMUSG00000077380 | Gm22661 | M_musculus | Animals | intronic_cluster | H/ACA | ENSMUSG00000041995 | Zbed3 | protein_coding |
| ENSMUSG00000077381 | Gm22660 | M_musculus | Animals | mono_intergenic | H/ACA | | | intergenic |
| ENSMUSG00000077383 | Gm22659 | M_musculus | Animals | mono_intergenic | H/ACA | | | intergenic |
| ENSMUSG00000077385 | Gm22657 | M_musculus | Animals | mono_intergenic | H/ACA | | | intergenic |
| ENSMUSG00000077391 | Gm24336 | M_musculus | Animals | mono_intronic | H/ACA | ENSMUSG00000025200 | Cwf19l1 | protein_coding |
| ENSMUSG00000077392 | Gm24337 | M_musculus | Animals | mono_intergenic | H/ACA | | | intergenic |
| ENSMUSG00000077393 | Gm24338 | M_musculus | Animals | mono_intergenic | H/ACA | | | intergenic |
| ENSMUSG00000077394 | Gm24339 | M_musculus | Animals | intronic_cluster | C/D | ENSMUSG00000046111 | Cep295 | protein_coding |
| ENSMUSG00000077397 | Gm24341 | M_musculus | Animals | mono_intergenic | H/ACA | | | intergenic |
| ENSMUSG00000077398 | Gm24334 | M_musculus | Animals | mono_intergenic | H/ACA | | | intergenic |
| ENSMUSG00000077399 | Gm24335 | M_musculus | Animals | mono_intergenic | H/ACA | | | intergenic |
| ENSMUSG00000077400 | Gm25417 | M_musculus | Animals | mono_intergenic | H/ACA | | | intergenic |
| ENSMUSG00000077402 | Gm25418 | M_musculus | Animals | mono_intergenic | H/ACA | | | intergenic |
| ENSMUSG00000077403 | Gm25419 | M_musculus | Animals | mono_intergenic | C/D | | | intergenic |
| ENSMUSG00000077404 | Gm25420 | M_musculus | Animals | mono_intergenic | H/ACA | | | intergenic |
| ENSMUSG00000077407 | Gm25422 | M_musculus | Animals | mono_intergenic | H/ACA | | | intergenic |
| ENSMUSG00000077408 | Gm25415 | M_musculus | Animals | mono_intergenic | H/ACA | | | intergenic |
| ENSMUSG00000077410 | Gm23750 | M_musculus | Animals | mono_intergenic | H/ACA | | | intergenic |
| ENSMUSG00000077411 | Gm23749 | M_musculus | Animals | mono_intergenic | H/ACA | | | intergenic |
| ENSMUSG00000077413 | Gm23389 | M_musculus | Animals | mono_intergenic | C/D | | | intergenic |
| ENSMUSG00000077415 | Gm23748 | M_musculus | Animals | mono_intergenic | H/ACA | | | intergenic |
| ENSMUSG00000077416 | Gm23747 | M_musculus | Animals | mono_intergenic | H/ACA | | | intergenic |
| ENSMUSG00000077417 | Gm23746 | M_musculus | Animals | mono_intergenic | H/ACA | | | intergenic |
| ENSMUSG00000077418 | Gm23745 | M_musculus | Animals | mono_intergenic | H/ACA | | | intergenic |
| ENSMUSG00000077422 | Gm24281 | M_musculus | Animals | mono_intergenic | H/ACA | | | intergenic |

| | | | | | | | | |
|---|---|---|---|---|---|---|---|---|
| ENSMUSG00000077424 | Gm24279 | M_musculus | Animals | mono_intronic | H/ACA | ENSMUSG00000057816 | Cfap299 | protein_coding |
| ENSMUSG00000077425 | Gm24280 | M_musculus | Animals | mono_intergenic | H/ACA | | | intergenic |
| ENSMUSG00000077426 | Gm26387 | M_musculus | Animals | mono_intronic | H/ACA | ENSMUSG00000058558 | Rpl5 | protein_coding |
| ENSMUSG00000077427 | Gm24278 | M_musculus | Animals | mono_intergenic | H/ACA | | | intergenic |
| ENSMUSG00000077430 | Gm22592 | M_musculus | Animals | mono_intergenic | H/ACA | | | intergenic |
| ENSMUSG00000077435 | Gm22590 | M_musculus | Animals | mono_intergenic | H/ACA | | | intergenic |
| ENSMUSG00000077438 | Gm22595 | M_musculus | Animals | mono_intergenic | H/ACA | | | intergenic |
| ENSMUSG00000077439 | Gm22594 | M_musculus | Animals | mono_intergenic | H/ACA | | | intergenic |
| ENSMUSG00000077440 | Gm23130 | M_musculus | Animals | mono_intronic | C/D | ENSMUSG00000061983 | Rps12 | protein_coding |
| ENSMUSG00000077441 | Gm23131 | M_musculus | Animals | mono_intergenic | H/ACA | | | intergenic |
| ENSMUSG00000077447 | Gm23129 | M_musculus | Animals | mono_intergenic | H/ACA | | | intergenic |
| ENSMUSG00000077448 | Gm23126 | M_musculus | Animals | mono_intergenic | H/ACA | | | intergenic |
| ENSMUSG00000077449 | Gm23127 | M_musculus | Animals | mono_intronic | H/ACA | ENSMUSG00000001542 | Ell2 | protein_coding |
| ENSMUSG00000077451 | Gm25916 | M_musculus | Animals | mono_intergenic | H/ACA | | | intergenic |
| ENSMUSG00000077453 | Gm25915 | M_musculus | Animals | mono_intergenic | H/ACA | | | intergenic |
| ENSMUSG00000077455 | Gm25917 | M_musculus | Animals | mono_intronic | H/ACA | ENSMUSG00000112211 | Gm47682 | non_coding |
| ENSMUSG00000077456 | Gm22927 | M_musculus | Animals | mono_intergenic | H/ACA | | | intergenic |
| ENSMUSG00000077457 | Snord65 | M_musculus | Animals | mono_intronic | C/D | ENSMUSG00000086841 | 2410006H16Rik | non_coding |
| ENSMUSG00000077459 | Gm25919 | M_musculus | Animals | mono_intergenic | H/ACA | | | intergenic |
| ENSMUSG00000077460 | Gm26419 | M_musculus | Animals | mono_intergenic | H/ACA | | | intergenic |
| ENSMUSG00000077462 | Gm26417 | M_musculus | Animals | mono_intergenic | H/ACA | | | intergenic |
| ENSMUSG00000077463 | Gm26418 | M_musculus | Animals | mono_intronic | H/ACA | ENSMUSG00000026141 | Col19a1 | protein_coding |
| ENSMUSG00000077465 | Gm26423 | M_musculus | Animals | mono_intergenic | H/ACA | | | intergenic |
| ENSMUSG00000077468 | Gm26416 | M_musculus | Animals | mono_intergenic | H/ACA | | | intergenic |
| ENSMUSG00000077469 | Gm25922 | M_musculus | Animals | mono_intergenic | H/ACA | | | intergenic |
| ENSMUSG00000077470 | Gm24789 | M_musculus | Animals | mono_exonic | H/ACA | ENSMUSG00000115508 | Gm49786 | non_coding |
| ENSMUSG00000077472 | Gm24791 | M_musculus | Animals | mono_intronic | H/ACA | ENSMUSG00000036815 | Dpp10 | protein_coding |
| ENSMUSG00000077473 | Gm24790 | M_musculus | Animals | mono_intergenic | H/ACA | | | intergenic |
| ENSMUSG00000077474 | Gm24792 | M_musculus | Animals | mono_intergenic | H/ACA | | | intergenic |
| ENSMUSG00000077476 | Gm24794 | M_musculus | Animals | mono_intergenic | H/ACA | | | intergenic |
| ENSMUSG00000077477 | Gm24793 | M_musculus | Animals | mono_intergenic | H/ACA | | | intergenic |
| ENSMUSG00000077481 | Gm25272 | M_musculus | Animals | mono_intronic | H/ACA | ENSMUSG00000032925 | Itgbl1 | protein_coding |
| ENSMUSG00000077484 | Gm25274 | M_musculus | Animals | mono_intergenic | H/ACA | | | intergenic |
| ENSMUSG00000077485 | Gm25275 | M_musculus | Animals | mono_intergenic | H/ACA | | | intergenic |
| ENSMUSG00000077488 | Gm25276 | M_musculus | Animals | mono_intergenic | H/ACA | | | intergenic |
| ENSMUSG00000077492 | Gm23596 | M_musculus | Animals | mono_intergenic | H/ACA | | | intergenic |
| ENSMUSG00000077493 | Snord91a | M_musculus | Animals | mono_intronic | C/D | ENSMUSG00000038335 | Tsr1 | protein_coding |
| ENSMUSG00000077494 | Gm23595 | M_musculus | Animals | mono_intergenic | H/ACA | | | intergenic |
| ENSMUSG00000077495 | Gm23594 | M_musculus | Animals | mono_intergenic | H/ACA | | | intergenic |
| ENSMUSG00000077497 | Gm23593 | M_musculus | Animals | mono_intronic | H/ACA | ENSMUSG00000021947 | Cryl1 | protein_coding |
| ENSMUSG00000077501 | Gm24230 | M_musculus | Animals | mono_intergenic | H/ACA | | | intergenic |

| | | | | | | | | |
|---|---|---|---|---|---|---|---|---|
| ENSMUSG00000077502 | Gm24232 | M_musculus | Animals | mono_intergenic | H/ACA | | | intergenic |
| ENSMUSG00000077503 | Gm24231 | M_musculus | Animals | mono_intergenic | H/ACA | | | intergenic |
| ENSMUSG00000077505 | Gm24233 | M_musculus | Animals | mono_intronic | H/ACA | ENSMUSG00000021000 | Mia2 | protein_coding |
| ENSMUSG00000077506 | Scarna9 | M_musculus | Animals | intronic_cluster | C/D | ENSMUSG00000046111 | Cep295 | protein_coding |
| ENSMUSG00000077507 | Gm24235 | M_musculus | Animals | mono_intergenic | H/ACA | | | intergenic |
| ENSMUSG00000077514 | Gm25877 | M_musculus | Animals | mono_intergenic | H/ACA | | | intergenic |
| ENSMUSG00000077515 | Gm25878 | M_musculus | Animals | mono_intronic | C/D | ENSMUSG00000074578 | Zfas1 | non_coding |
| ENSMUSG00000077519 | Gm25880 | M_musculus | Animals | mono_intergenic | H/ACA | | | intergenic |
| ENSMUSG00000077521 | Gm25378 | M_musculus | Animals | mono_intergenic | H/ACA | | | intergenic |
| ENSMUSG00000077522 | Gm25377 | M_musculus | Animals | mono_intronic | H/ACA | ENSMUSG00000106741 | 4930513D17Rik | non_coding |
| ENSMUSG00000077526 | Gm25379 | M_musculus | Animals | mono_intergenic | H/ACA | | | intergenic |
| ENSMUSG00000077527 | Gm25506 | M_musculus | Animals | mono_intronic | C/D | ENSMUSG00000031691 | Tnpo2 | protein_coding |
| ENSMUSG00000077528 | Gm25376 | M_musculus | Animals | mono_intergenic | H/ACA | | | intergenic |
| ENSMUSG00000077537 | Gm22544 | M_musculus | Animals | mono_intronic | H/ACA | ENSMUSG00000110485 | Gm45740 | non_coding |
| ENSMUSG00000077538 | Gm22545 | M_musculus | Animals | mono_intergenic | H/ACA | | | intergenic |
| ENSMUSG00000077539 | Gm22546 | M_musculus | Animals | mono_intergenic | H/ACA | | | intergenic |
| ENSMUSG00000077542 | Gm22023 | M_musculus | Animals | mono_intronic | H/ACA | ENSMUSG00000031379 | Pir | protein_coding |
| ENSMUSG00000077543 | Gm22022 | M_musculus | Animals | mono_intergenic | H/ACA | | | intergenic |
| ENSMUSG00000077545 | Gm22018 | M_musculus | Animals | mono_intergenic | H/ACA | | | intergenic |
| ENSMUSG00000077546 | Gm22020 | M_musculus | Animals | mono_intronic | H/ACA | ENSMUSG00000059742 | Kcnh7 | protein_coding |
| ENSMUSG00000077549 | Snord71 | M_musculus | Animals | mono_intronic | C/D | ENSMUSG00000031731 | Ap1g1 | protein_coding |
| ENSMUSG00000077552 | Gm23709 | M_musculus | Animals | mono_intergenic | H/ACA | | | intergenic |
| ENSMUSG00000077553 | Gm23176 | M_musculus | Animals | mono_intergenic | H/ACA | | | intergenic |
| ENSMUSG00000077554 | Gm23707 | M_musculus | Animals | mono_intergenic | H/ACA | | | intergenic |
| ENSMUSG00000077555 | Gm23708 | M_musculus | Animals | mono_intergenic | H/ACA | | | intergenic |
| ENSMUSG00000077557 | Gm23706 | M_musculus | Animals | mono_intergenic | C/D | | | intergenic |
| ENSMUSG00000077558 | Gm23705 | M_musculus | Animals | mono_intronic | H/ACA | ENSMUSG00000016150 | Tenm1 | protein_coding |
| ENSMUSG00000077561 | Gm24607 | M_musculus | Animals | mono_intronic | H/ACA | ENSMUSG00000040410 | Fbxl4 | protein_coding |
| ENSMUSG00000077563 | Snora68 | M_musculus | Animals | mono_intronic | H/ACA | ENSMUSG00000045128 | Rpl18a | protein_coding |
| ENSMUSG00000077564 | Gm23202 | M_musculus | Animals | mono_intronic | H/ACA | ENSMUSG00000041453 | Rpl21 | protein_coding |
| ENSMUSG00000077565 | Gm23201 | M_musculus | Animals | mono_intronic | C/D | ENSMUSG00000074578 | Zfas1 | non_coding |
| ENSMUSG00000077567 | Gm23200 | M_musculus | Animals | mono_intronic | H/ACA | ENSMUSG00000058656 | Samd12 | protein_coding |
| ENSMUSG00000077569 | Gm24608 | M_musculus | Animals | mono_intronic | H/ACA | ENSMUSG00000042520 | Ubap2l | protein_coding |
| ENSMUSG00000077572 | Gm24886 | M_musculus | Animals | mono_intergenic | H/ACA | | | intergenic |
| ENSMUSG00000077575 | Gm24888 | M_musculus | Animals | mono_intronic | H/ACA | ENSMUSG00000046364 | Rpl27a | protein_coding |
| ENSMUSG00000077576 | Gm24889 | M_musculus | Animals | mono_intronic | H/ACA | ENSMUSG00000111234 | 4930591E09Rik | non_coding |
| ENSMUSG00000077578 | Gm25631 | M_musculus | Animals | mono_intronic | H/ACA | ENSMUSG00000021720 | Rnf180 | protein_coding |
| ENSMUSG00000077579 | Gm24891 | M_musculus | Animals | mono_intergenic | H/ACA | | | intergenic |
| ENSMUSG00000077581 | Gm24373 | M_musculus | Animals | mono_intronic | H/ACA | ENSMUSG00000040037 | Negr1 | protein_coding |
| ENSMUSG00000077585 | Gm24375 | M_musculus | Animals | mono_intergenic | H/ACA | | | intergenic |
| ENSMUSG00000077586 | Gm24377 | M_musculus | Animals | mono_intergenic | H/ACA | | | intergenic |

| | | | | | | | | |
|---|---|---|---|---|---|---|---|---|
| ENSMUSG00000077587 | Gm24376 | M_musculus | Animals | mono_intergenic | H/ACA | | | intergenic |
| ENSMUSG00000077590 | Gm26015 | M_musculus | Animals | mono_intergenic | H/ACA | | | intergenic |
| ENSMUSG00000077591 | Gm26016 | M_musculus | Animals | mono_intergenic | H/ACA | | | intergenic |
| ENSMUSG00000077592 | Gm26013 | M_musculus | Animals | mono_intergenic | H/ACA | | | intergenic |
| ENSMUSG00000077593 | Gm26014 | M_musculus | Animals | mono_intergenic | H/ACA | | | intergenic |
| ENSMUSG00000077594 | Gm26018 | M_musculus | Animals | mono_intronic | H/ACA | ENSMUSG00000113573 | AU017674 | non_coding |
| ENSMUSG00000077595 | Gm26019 | M_musculus | Animals | mono_intergenic | H/ACA | | | intergenic |
| ENSMUSG00000077599 | Gm26012 | M_musculus | Animals | mono_intergenic | H/ACA | | | intergenic |
| ENSMUSG00000077601 | Gm22674 | M_musculus | Animals | mono_intergenic | H/ACA | | | intergenic |
| ENSMUSG00000077602 | Gm22672 | M_musculus | Animals | mono_intronic | H/ACA | ENSMUSG00000033306 | Lpp | protein_coding |
| ENSMUSG00000077603 | Gm22673 | M_musculus | Animals | mono_intergenic | H/ACA | | | intergenic |
| ENSMUSG00000077604 | Snord1b | M_musculus | Animals | mono_intronic | C/D | ENSMUSG00000020812 | Snhg16 | non_coding |
| ENSMUSG00000077607 | Gm25803 | M_musculus | Animals | mono_intronic | H/ACA | ENSMUSG00000028402 | Mpdz | protein_coding |
| ENSMUSG00000077608 | Gm22671 | M_musculus | Animals | mono_intronic | H/ACA | ENSMUSG00000071890 | Mroh9 | protein_coding |
| ENSMUSG00000077609 | Gm25795 | M_musculus | Animals | mono_intronic | H/ACA | ENSMUSG00000057457 | Phex | protein_coding |
| ENSMUSG00000077611 | Gm23946 | M_musculus | Animals | mono_intronic | H/ACA | ENSMUSG00000013701 | Timm23 | protein_coding |
| ENSMUSG00000077612 | Gm25498 | M_musculus | Animals | mono_intergenic | H/ACA | | | intergenic |
| ENSMUSG00000077615 | Gm25499 | M_musculus | Animals | mono_intergenic | C/D | ENSMUSG00000100826 | Snhg14 | non_coding |
| ENSMUSG00000077616 | Gm25502 | M_musculus | Animals | mono_intergenic | H/ACA | | | intergenic |
| ENSMUSG00000077617 | Gm25501 | M_musculus | Animals | mono_intergenic | H/ACA | | | intergenic |
| ENSMUSG00000077618 | Gm25496 | M_musculus | Animals | mono_intergenic | H/ACA | | | intergenic |
| ENSMUSG00000077619 | Gm25495 | M_musculus | Animals | mono_intergenic | H/ACA | | | intergenic |
| ENSMUSG00000077624 | Gm23834 | M_musculus | Animals | mono_intergenic | H/ACA | | | intergenic |
| ENSMUSG00000077625 | Snord4a | M_musculus | Animals | mono_intronic | C/D | ENSMUSG00000058546 | Rpl23a | protein_coding |
| ENSMUSG00000077627 | Gm23835 | M_musculus | Animals | mono_intergenic | H/ACA | | | intergenic |
| ENSMUSG00000077628 | Gm23768 | M_musculus | Animals | mono_intergenic | H/ACA | | | intergenic |
| ENSMUSG00000077629 | Gm23769 | M_musculus | Animals | mono_intergenic | H/ACA | | | intergenic |
| ENSMUSG00000077630 | Gm22149 | M_musculus | Animals | intergenic_cluster | H/ACA | | | intergenic |
| ENSMUSG00000077635 | Gm22150 | M_musculus | Animals | mono_intergenic | H/ACA | | | intergenic |
| ENSMUSG00000077637 | Gm22771 | M_musculus | Animals | mono_intronic | C/D | ENSMUSG00000038335 | Tsr1 | protein_coding |
| ENSMUSG00000077639 | Gm22772 | M_musculus | Animals | mono_intergenic | H/ACA | | | intergenic |
| ENSMUSG00000077641 | Gm24987 | M_musculus | Animals | mono_intergenic | H/ACA | | | intergenic |
| ENSMUSG00000077642 | Gm24984 | M_musculus | Animals | mono_intergenic | H/ACA | | | intergenic |
| ENSMUSG00000077643 | Gm24985 | M_musculus | Animals | mono_intergenic | H/ACA | | | intergenic |
| ENSMUSG00000077645 | Gm24983 | M_musculus | Animals | mono_intergenic | C/D | ENSMUSG00000100826 | Snhg14 | non_coding |
| ENSMUSG00000077647 | Gm24981 | M_musculus | Animals | mono_intergenic | H/ACA | | | intergenic |
| ENSMUSG00000077648 | Gm24988 | M_musculus | Animals | mono_intergenic | H/ACA | | | intergenic |
| ENSMUSG00000077649 | Gm24989 | M_musculus | Animals | mono_intergenic | C/D | | | intergenic |
| ENSMUSG00000077650 | Gm23320 | M_musculus | Animals | mono_intergenic | H/ACA | | | intergenic |
| ENSMUSG00000077653 | Gm23321 | M_musculus | Animals | mono_intergenic | H/ACA | | | intergenic |
| ENSMUSG00000077655 | Gm23319 | M_musculus | Animals | mono_intergenic | H/ACA | | | intergenic |

| | | | | | | | | |
|---|---|---|---|---|---|---|---|---|
| ENSMUSG00000077659 | Gm23323 | M_musculus | Animals | mono_intergenic | H/ACA | | | intergenic |
| ENSMUSG00000077660 | Gm24257 | M_musculus | Animals | mono_intergenic | H/ACA | | | intergenic |
| ENSMUSG00000077663 | Gm26144 | M_musculus | Animals | mono_intergenic | H/ACA | | | intergenic |
| ENSMUSG00000077664 | Gm26145 | M_musculus | Animals | mono_intergenic | H/ACA | | | intergenic |
| ENSMUSG00000077667 | Gm26147 | M_musculus | Animals | mono_intronic | H/ACA | ENSMUSG00000030982 | Vps35l | protein_coding |
| ENSMUSG00000077669 | Gm24255 | M_musculus | Animals | mono_intergenic | H/ACA | | | intergenic |
| ENSMUSG00000077671 | Gm24473 | M_musculus | Animals | mono_intergenic | H/ACA | | | intergenic |
| ENSMUSG00000077672 | Gm24472 | M_musculus | Animals | mono_intergenic | H/ACA | | | intergenic |
| ENSMUSG00000077673 | Gm24471 | M_musculus | Animals | mono_intergenic | H/ACA | | | intergenic |
| ENSMUSG00000077674 | Gm24470 | M_musculus | Animals | mono_intergenic | H/ACA | | | intergenic |
| ENSMUSG00000077675 | Gm24469 | M_musculus | Animals | mono_intergenic | H/ACA | | | intergenic |
| ENSMUSG00000077678 | Gm24318 | M_musculus | Animals | mono_intergenic | H/ACA | | | intergenic |
| ENSMUSG00000077680 | Gm22495 | M_musculus | Animals | mono_intergenic | H/ACA | | | intergenic |
| ENSMUSG00000077681 | Gm22496 | M_musculus | Animals | mono_intergenic | C/D | ENSMUSG00000100826 | Snhg14 | non_coding |
| ENSMUSG00000077682 | Gm22493 | M_musculus | Animals | intronic_cluster | H/ACA | ENSMUSG00000004347 | Pde1c | protein_coding |
| ENSMUSG00000077683 | Gm22494 | M_musculus | Animals | mono_intergenic | H/ACA | | | intergenic |
| ENSMUSG00000077685 | Gm22498 | M_musculus | Animals | mono_intergenic | C/D | | | intergenic |
| ENSMUSG00000077687 | Gm22497 | M_musculus | Animals | mono_intergenic | H/ACA | | | intergenic |
| ENSMUSG00000077689 | Gm22499 | M_musculus | Animals | mono_intergenic | H/ACA | | | intergenic |
| ENSMUSG00000077691 | Gm25344 | M_musculus | Animals | mono_intergenic | H/ACA | | | intergenic |
| ENSMUSG00000077693 | Gm25345 | M_musculus | Animals | mono_intergenic | H/ACA | | | intergenic |
| ENSMUSG00000077697 | Gm25346 | M_musculus | Animals | mono_intergenic | H/ACA | | | intergenic |
| ENSMUSG00000077698 | Snord12 | M_musculus | Animals | mono_exonic | C/D | ENSMUSG00000074578 | Zfas1 | non_coding |
| ENSMUSG00000077700 | Gm24069 | M_musculus | Animals | mono_intergenic | H/ACA | | | intergenic |
| ENSMUSG00000077701 | Gm24068 | M_musculus | Animals | mono_intergenic | H/ACA | | | intergenic |
| ENSMUSG00000077702 | Gm24067 | M_musculus | Animals | mono_intronic | C/D | ENSMUSG00000004100 | Ppan | protein_coding |
| ENSMUSG00000077703 | Gm24066 | M_musculus | Animals | mono_intergenic | H/ACA | | | intergenic |
| ENSMUSG00000077704 | Snord89 | M_musculus | Animals | mono_intronic | C/D | ENSMUSG00000085894 | Gm15832 | non_coding |
| ENSMUSG00000077707 | Gm24070 | M_musculus | Animals | mono_intergenic | H/ACA | | | intergenic |
| ENSMUSG00000077708 | Gm24071 | M_musculus | Animals | mono_intergenic | H/ACA | | | intergenic |
| ENSMUSG00000077709 | Snora64 | M_musculus | Animals | mono_intronic | H/ACA | ENSMUSG00000044533 | Rps2 | protein_coding |
| ENSMUSG00000077711 | AF357399 | M_musculus | Animals | mono_intronic | H/ACA | ENSMUSG00000037563 | Rps16 | protein_coding |
| ENSMUSG00000077714 | Snord17 | M_musculus | Animals | mono_intronic | C/D | ENSMUSG00000027423 | Snx5 | protein_coding |
| ENSMUSG00000077716 | Gm25725 | M_musculus | Animals | mono_intergenic | H/ACA | | | intergenic |
| ENSMUSG00000077717 | Gm25726 | M_musculus | Animals | mono_intergenic | H/ACA | | | intergenic |
| ENSMUSG00000077718 | Gm25723 | M_musculus | Animals | mono_intergenic | H/ACA | | | intergenic |
| ENSMUSG00000077719 | Gm25724 | M_musculus | Animals | mono_intergenic | H/ACA | | | intergenic |
| ENSMUSG00000077730 | Gm23609 | M_musculus | Animals | mono_intergenic | H/ACA | | | intergenic |
| ENSMUSG00000077733 | Gm23608 | M_musculus | Animals | mono_intronic | H/ACA | ENSMUSG00000025781 | Atp5c1 | protein_coding |
| ENSMUSG00000077734 | Snord83b | M_musculus | Animals | mono_intronic | C/D | ENSMUSG00000060036 | Rpl3 | protein_coding |
| ENSMUSG00000077735 | Gm24556 | M_musculus | Animals | mono_intergenic | H/ACA | | | intergenic |

| | | | | | | | | |
|---|---|---|---|---|---|---|---|---|
| ENSMUSG00000077736 | Gm24555 | M_musculus | Animals | mono_intergenic | H/ACA | | | intergenic |
| ENSMUSG00000077737 | Snord72 | M_musculus | Animals | mono_intronic | C/D | ENSMUSG00000041841 | Rpl37 | protein_coding |
| ENSMUSG00000077738 | Gm23607 | M_musculus | Animals | mono_intergenic | H/ACA | | | intergenic |
| ENSMUSG00000077744 | Gm26343 | M_musculus | Animals | mono_intergenic | H/ACA | | | intergenic |
| ENSMUSG00000077745 | Gm26342 | M_musculus | Animals | mono_intronic | H/ACA | ENSMUSG00000086204 | Gm15668 | non_coding |
| ENSMUSG00000077749 | Gm26341 | M_musculus | Animals | mono_intergenic | H/ACA | | | intergenic |
| ENSMUSG00000077752 | Gm23530 | M_musculus | Animals | mono_intergenic | H/ACA | | | intergenic |
| ENSMUSG00000077756 | Snord90 | M_musculus | Animals | mono_intronic | C/D | ENSMUSG00000075376 | Rc3h2 | protein_coding |
| ENSMUSG00000077757 | Gm23532 | M_musculus | Animals | mono_intergenic | H/ACA | | | intergenic |
| ENSMUSG00000077758 | Gm23527 | M_musculus | Animals | mono_intergenic | H/ACA | | | intergenic |
| ENSMUSG00000077759 | Gm23528 | M_musculus | Animals | mono_intergenic | H/ACA | | | intergenic |
| ENSMUSG00000077761 | Gm25214 | M_musculus | Animals | mono_intergenic | H/ACA | | | intergenic |
| ENSMUSG00000077767 | Snora35 | M_musculus | Animals | mono_intronic | H/ACA | ENSMUSG00000041380 | Htr2c | protein_coding |
| ENSMUSG00000077768 | Gm25216 | M_musculus | Animals | mono_intergenic | H/ACA | | | intergenic |
| ENSMUSG00000077769 | Gm25215 | M_musculus | Animals | mono_intergenic | H/ACA | | | intergenic |
| ENSMUSG00000077772 | Gm22373 | M_musculus | Animals | mono_intergenic | C/D | ENSMUSG00000100826 | Snhg14 | non_coding |
| ENSMUSG00000077776 | Gm22371 | M_musculus | Animals | mono_intergenic | H/ACA | | | intergenic |
| ENSMUSG00000077777 | Gm22372 | M_musculus | Animals | mono_intergenic | H/ACA | | | intergenic |
| ENSMUSG00000077780 | Gm24161 | M_musculus | Animals | mono_intergenic | H/ACA | | | intergenic |
| ENSMUSG00000077782 | Gm24160 | M_musculus | Animals | mono_intergenic | H/ACA | | | intergenic |
| ENSMUSG00000077784 | Gm23911 | M_musculus | Animals | mono_intergenic | H/ACA | | | intergenic |
| ENSMUSG00000077786 | Gm23909 | M_musculus | Animals | mono_intergenic | H/ACA | | | intergenic |
| ENSMUSG00000077787 | Gm23908 | M_musculus | Animals | mono_intronic | H/ACA | ENSMUSG00000042042 | Csgalnact2 | protein_coding |
| ENSMUSG00000077795 | Gm25561 | M_musculus | Animals | mono_intergenic | H/ACA | | | intergenic |
| ENSMUSG00000077796 | Gm25562 | M_musculus | Animals | mono_intergenic | H/ACA | | | intergenic |
| ENSMUSG00000077797 | Snord19 | M_musculus | Animals | mono_intronic | C/D | ENSMUSG00000042354 | Gnl3 | protein_coding |
| ENSMUSG00000077799 | Gm25148 | M_musculus | Animals | mono_intergenic | H/ACA | | | intergenic |
| ENSMUSG00000077804 | Gm24403 | M_musculus | Animals | mono_intergenic | H/ACA | | | intergenic |
| ENSMUSG00000080352 | Gm26247 | M_musculus | Animals | mono_intronic | C/D | ENSMUSG00000045411 | 2410002F23Rik | protein_coding |
| ENSMUSG00000080364 | Gm25777 | M_musculus | Animals | mono_intronic | H/ACA | ENSMUSG00000005732 | Ranbp1 | protein_coding |
| ENSMUSG00000080365 | Gm25776 | M_musculus | Animals | mono_intronic | H/ACA | ENSMUSG00000020962 | Gtf2a1 | protein_coding |
| ENSMUSG00000080396 | Snord111 | M_musculus | Animals | mono_intronic | C/D | ENSMUSG00000033732 | Sf3b3 | protein_coding |
| ENSMUSG00000080440 | Gm25848 | M_musculus | Animals | mono_intronic | H/ACA | ENSMUSG00000035764 | Fbxo45 | protein_coding |
| ENSMUSG00000080461 | Gm22488 | M_musculus | Animals | mono_intronic | H/ACA | ENSMUSG00000022359 | Wdyhv1 | protein_coding |
| ENSMUSG00000080463 | Gm22489 | M_musculus | Animals | mono_intronic | C/D | ENSMUSG00000053332 | Gas5 | non_coding |
| ENSMUSG00000080465 | Snord94 | M_musculus | Animals | mono_intronic | C/D | ENSMUSG00000063884 | Ptcd3 | protein_coding |
| ENSMUSG00000080469 | Snord98 | M_musculus | Animals | mono_intronic | C/D | ENSMUSG00000020074 | Ccar1 | protein_coding |
| ENSMUSG00000080478 | Snord23 | M_musculus | Animals | mono_intronic | C/D | ENSMUSG00000041560 | Nop53 | protein_coding |
| ENSMUSG00000080518 | Gm22193 | M_musculus | Animals | mono_intronic | C/D | ENSMUSG00000033732 | Sf3b3 | protein_coding |
| ENSMUSG00000080536 | Gm25539 | M_musculus | Animals | mono_intergenic | H/ACA | | | intergenic |
| ENSMUSG00000080540 | Gm22711 | M_musculus | Animals | mono_exonic | H/ACA | ENSMUSG00000120012 | Gm36220 | non_coding |

| | | | | | | | | |
|---|---|---|---|---|---|---|---|---|
| ENSMUSG00000080582 | Gm24907 | M_musculus | Animals | mono_intronic | H/ACA | ENSMUSG00000025268 | Maged2 | protein_coding |
| ENSMUSG00000080607 | Gm23839 | M_musculus | Animals | mono_intergenic | C/D | | | intergenic |
| ENSMUSG00000080610 | Snord110 | M_musculus | Animals | mono_intronic | C/D | ENSMUSG00000027405 | Nop56 | protein_coding |
| ENSMUSG00000080615 | Snord99 | M_musculus | Animals | mono_intronic | C/D | ENSMUSG00000086290 | Snhg12 | non_coding |
| ENSMUSG00000080616 | Gm22152 | M_musculus | Animals | mono_intronic | C/D | ENSMUSG00000086835 | Gm11775 | non_coding |
| ENSMUSG00000080622 | Gm22676 | M_musculus | Animals | mono_intronic | H/ACA | ENSMUSG00000003604 | Aven | protein_coding |
| ENSMUSG00000080683 | Gm23991 | M_musculus | Animals | mono_intronic | C/D | ENSMUSG00000045411 | 2410002F23Rik | protein_coding |
| ENSMUSG00000080689 | Gm23987 | M_musculus | Animals | mono_intergenic | H/ACA | | | intergenic |
| ENSMUSG00000084421 | Gm25107 | M_musculus | Animals | mono_intronic | H/ACA | ENSMUSG00000031290 | Lrch2 | protein_coding |
| ENSMUSG00000084447 | Gm26251 | M_musculus | Animals | mono_intergenic | H/ACA | | | intergenic |
| ENSMUSG00000084450 | Gm24595 | M_musculus | Animals | mono_intronic | H/ACA | ENSMUSG00000118620 | Gm53011 | non_coding |
| ENSMUSG00000084459 | Gm24598 | M_musculus | Animals | mono_intergenic | C/D | | | intergenic |
| ENSMUSG00000084487 | Gm23787 | M_musculus | Animals | mono_intronic | C/D | ENSMUSG00000098202 | B830012L14Rik | non_coding |
| ENSMUSG00000084517 | Gm22480 | M_musculus | Animals | mono_intergenic | H/ACA | | | intergenic |
| ENSMUSG00000084521 | Gm24183 | M_musculus | Animals | mono_intergenic | H/ACA | | | intergenic |
| ENSMUSG00000084549 | DQ267100 | M_musculus | Animals | mono_intronic | C/D | ENSMUSG00000097451 | Rian | non_coding |
| ENSMUSG00000084604 | Gm22205 | M_musculus | Animals | mono_intronic | C/D | ENSMUSG00000098202 | B830012L14Rik | non_coding |
| ENSMUSG00000084667 | Gm23212 | M_musculus | Animals | mono_exonic | C/D | ENSMUSG00000053332 | Gas5 | non_coding |
| ENSMUSG00000084686 | Gm22027 | M_musculus | Animals | mono_intronic | H/ACA | ENSMUSG00000068856 | Sf3b4 | protein_coding |
| ENSMUSG00000084690 | Gm24896 | M_musculus | Animals | mono_intergenic | H/ACA | | | intergenic |
| ENSMUSG00000084691 | Gm24895 | M_musculus | Animals | mono_intronic | C/D | ENSMUSG00000097451 | Rian | non_coding |
| ENSMUSG00000084745 | Gm25290 | M_musculus | Animals | mono_intronic | H/ACA | ENSMUSG00000029407 | Uso1 | protein_coding |
| ENSMUSG00000087707 | Gm22999 | M_musculus | Animals | mono_intronic | H/ACA | ENSMUSG00000031841 | Cdh13 | protein_coding |
| ENSMUSG00000087715 | Gm25826 | M_musculus | Animals | mono_intergenic | H/ACA | | | intergenic |
| ENSMUSG00000087717 | Gm25824 | M_musculus | Animals | mono_intergenic | H/ACA | | | intergenic |
| ENSMUSG00000087722 | Gm26277 | M_musculus | Animals | mono_intronic | H/ACA | ENSMUSG00000007080 | Pole | protein_coding |
| ENSMUSG00000087723 | Gm26278 | M_musculus | Animals | mono_intergenic | H/ACA | | | intergenic |
| ENSMUSG00000087724 | Gm26276 | M_musculus | Animals | mono_intergenic | H/ACA | | | intergenic |
| ENSMUSG00000087736 | Gm24648 | M_musculus | Animals | mono_intergenic | H/ACA | | | intergenic |
| ENSMUSG00000087742 | Gm25152 | M_musculus | Animals | mono_intergenic | H/ACA | | | intergenic |
| ENSMUSG00000087747 | Gm25151 | M_musculus | Animals | mono_intergenic | H/ACA | | | intergenic |
| ENSMUSG00000087765 | Gm23985 | M_musculus | Animals | mono_intergenic | H/ACA | | | intergenic |
| ENSMUSG00000087772 | Gm22319 | M_musculus | Animals | mono_intergenic | H/ACA | | | intergenic |
| ENSMUSG00000087776 | Gm22322 | M_musculus | Animals | mono_intergenic | H/ACA | | | intergenic |
| ENSMUSG00000087781 | Gm23168 | M_musculus | Animals | mono_intergenic | H/ACA | | | intergenic |
| ENSMUSG00000087790 | Gm25970 | M_musculus | Animals | mono_intronic | C/D | ENSMUSG00000035597 | Prpf39 | protein_coding |
| ENSMUSG00000087793 | Gm25967 | M_musculus | Animals | mono_intronic | H/ACA | ENSMUSG00000022848 | Slc49a4 | protein_coding |
| ENSMUSG00000087795 | Gm25965 | M_musculus | Animals | mono_intergenic | H/ACA | | | intergenic |
| ENSMUSG00000087800 | Gm25569 | M_musculus | Animals | mono_intergenic | H/ACA | | | intergenic |
| ENSMUSG00000087802 | Gm25979 | M_musculus | Animals | mono_intergenic | H/ACA | | | intergenic |
| ENSMUSG00000087806 | Gm23432 | M_musculus | Animals | mono_intergenic | H/ACA | | | intergenic |

| | | | | | | | | |
|---|---|---|---|---|---|---|---|---|
| ENSMUSG00000087810 | Gm25113 | M_musculus | Animals | mono_intergenic | H/ACA | | | intergenic |
| ENSMUSG00000087813 | Gm22490 | M_musculus | Animals | mono_intergenic | H/ACA | | | intergenic |
| ENSMUSG00000087816 | Gm25116 | M_musculus | Animals | mono_intronic | H/ACA | ENSMUSG00000024294 | Mib1 | protein_coding |
| ENSMUSG00000087819 | Gm25117 | M_musculus | Animals | mono_intronic | H/ACA | ENSMUSG00000058799 | Nap1l1 | protein_coding |
| ENSMUSG00000087831 | Gm23960 | M_musculus | Animals | mono_intergenic | H/ACA | | | intergenic |
| ENSMUSG00000087850 | Gm22792 | M_musculus | Animals | mono_intergenic | H/ACA | | | intergenic |
| ENSMUSG00000087857 | Gm22791 | M_musculus | Animals | mono_intergenic | H/ACA | | | intergenic |
| ENSMUSG00000087858 | Gm22795 | M_musculus | Animals | mono_intergenic | H/ACA | | | intergenic |
| ENSMUSG00000087862 | Gm23048 | M_musculus | Animals | mono_intergenic | H/ACA | | | intergenic |
| ENSMUSG00000087867 | Gm24443 | M_musculus | Animals | mono_intergenic | H/ACA | | | intergenic |
| ENSMUSG00000087870 | Gm26100 | M_musculus | Animals | mono_intergenic | H/ACA | | | intergenic |
| ENSMUSG00000087880 | Gm23605 | M_musculus | Animals | mono_intergenic | H/ACA | | | intergenic |
| ENSMUSG00000087883 | Gm23602 | M_musculus | Animals | mono_intergenic | H/ACA | | | intergenic |
| ENSMUSG00000087884 | Gm23601 | M_musculus | Animals | mono_intergenic | H/ACA | | | intergenic |
| ENSMUSG00000087887 | Gm23598 | M_musculus | Animals | mono_intergenic | H/ACA | | | intergenic |
| ENSMUSG00000087892 | Gm25278 | M_musculus | Animals | mono_intergenic | H/ACA | | | intergenic |
| ENSMUSG00000087903 | Gm22039 | M_musculus | Animals | mono_intergenic | H/ACA | | | intergenic |
| ENSMUSG00000087908 | Gm22034 | M_musculus | Animals | mono_intergenic | H/ACA | | | intergenic |
| ENSMUSG00000087912 | Gm24916 | M_musculus | Animals | mono_intronic | C/D | ENSMUSG00000042354 | Gnl3 | protein_coding |
| ENSMUSG00000087916 | Gm24912 | M_musculus | Animals | mono_intergenic | H/ACA | | | intergenic |
| ENSMUSG00000087917 | Gm24911 | M_musculus | Animals | mono_intergenic | H/ACA | | | intergenic |
| ENSMUSG00000087919 | Gm24908 | M_musculus | Animals | mono_intergenic | H/ACA | | | intergenic |
| ENSMUSG00000087926 | Gm23233 | M_musculus | Animals | mono_intronic | H/ACA | ENSMUSG00000030556 | Lrrc28 | protein_coding |
| ENSMUSG00000087929 | Gm23234 | M_musculus | Animals | mono_intergenic | H/ACA | | | intergenic |
| ENSMUSG00000087930 | Gm26048 | M_musculus | Animals | mono_intergenic | H/ACA | | | intergenic |
| ENSMUSG00000087933 | Gm26047 | M_musculus | Animals | intergenic_cluster | H/ACA | | | intergenic |
| ENSMUSG00000087935 | Snora81 | M_musculus | Animals | mono_intronic | H/ACA | ENSMUSG00000022884 | Eif4a2 | protein_coding |
| ENSMUSG00000087936 | Gm23675 | M_musculus | Animals | mono_intergenic | H/ACA | | | intergenic |
| ENSMUSG00000087940 | Gm24246 | M_musculus | Animals | mono_intergenic | H/ACA | | | intergenic |
| ENSMUSG00000087942 | Gm24244 | M_musculus | Animals | mono_intronic | H/ACA | ENSMUSG00000117433 | Gm41654 | non_coding |
| ENSMUSG00000087954 | Gm22559 | M_musculus | Animals | mono_intergenic | H/ACA | | | intergenic |
| ENSMUSG00000087963 | Gm25394 | M_musculus | Animals | mono_intronic | H/ACA | ENSMUSG00000037851 | Iars | protein_coding |
| ENSMUSG00000087964 | Gm25388 | M_musculus | Animals | mono_intergenic | H/ACA | | | intergenic |
| ENSMUSG00000087967 | Gm25391 | M_musculus | Animals | mono_intergenic | H/ACA | | | intergenic |
| ENSMUSG00000087973 | Gm23716 | M_musculus | Animals | mono_intergenic | H/ACA | | | intergenic |
| ENSMUSG00000087985 | Gm22223 | M_musculus | Animals | mono_intergenic | H/ACA | | | intergenic |
| ENSMUSG00000087992 | Gm25059 | M_musculus | Animals | mono_intergenic | H/ACA | | | intergenic |
| ENSMUSG00000088005 | Gm24787 | M_musculus | Animals | mono_intergenic | H/ACA | | | intergenic |
| ENSMUSG00000088009 | Gm24774 | M_musculus | Animals | mono_intergenic | H/ACA | | | intergenic |
| ENSMUSG00000088019 | Gm23826 | M_musculus | Animals | mono_intronic | H/ACA | ENSMUSG00000034912 | Mdga2 | protein_coding |
| ENSMUSG00000088020 | Gm22762 | M_musculus | Animals | mono_intergenic | H/ACA | | | intergenic |

| | | | | | | | | |
|---|---|---|---|---|---|---|---|---|
| ENSMUSG00000088027 | Gm22144 | M_musculus | Animals | mono_intergenic | H/ACA | | | intergenic |
| ENSMUSG00000088037 | Gm24992 | M_musculus | Animals | mono_intronic | H/ACA | ENSMUSG00000054823 | Nsd3 | protein_coding |
| ENSMUSG00000088039 | Gm24994 | M_musculus | Animals | mono_intergenic | H/ACA | | | intergenic |
| ENSMUSG00000088040 | Gm23329 | M_musculus | Animals | mono_intergenic | H/ACA | | | intergenic |
| ENSMUSG00000088041 | Gm25261 | M_musculus | Animals | mono_intergenic | H/ACA | | | intergenic |
| ENSMUSG00000088043 | Gm23328 | M_musculus | Animals | mono_intronic | H/ACA | ENSMUSG00000027022 | Xirp2 | protein_coding |
| ENSMUSG00000088045 | Gm23327 | M_musculus | Animals | intergenic_cluster | H/ACA | | | intergenic |
| ENSMUSG00000088046 | Gm23324 | M_musculus | Animals | mono_intergenic | H/ACA | | | intergenic |
| ENSMUSG00000088047 | Gm23325 | M_musculus | Animals | mono_intergenic | H/ACA | | | intergenic |
| ENSMUSG00000088055 | Gm26148 | M_musculus | Animals | mono_intergenic | H/ACA | | | intergenic |
| ENSMUSG00000088058 | Gm26153 | M_musculus | Animals | mono_intergenic | H/ACA | | | intergenic |
| ENSMUSG00000088060 | Gm24475 | M_musculus | Animals | mono_intergenic | H/ACA | | | intergenic |
| ENSMUSG00000088084 | Gm25342 | M_musculus | Animals | mono_intergenic | H/ACA | | | intergenic |
| ENSMUSG00000088094 | Gm23670 | M_musculus | Animals | mono_intergenic | H/ACA | | | intergenic |
| ENSMUSG00000088095 | Gm23669 | M_musculus | Animals | mono_intergenic | H/ACA | | | intergenic |
| ENSMUSG00000088098 | Gm23674 | M_musculus | Animals | mono_intergenic | H/ACA | | | intergenic |
| ENSMUSG00000088103 | Gm23993 | M_musculus | Animals | mono_intergenic | H/ACA | | | intergenic |
| ENSMUSG00000088104 | Gm22380 | M_musculus | Animals | mono_intronic | H/ACA | ENSMUSG00000074465 | Gm10701 | non_coding |
| ENSMUSG00000088105 | Gm22379 | M_musculus | Animals | mono_intergenic | H/ACA | | | intergenic |
| ENSMUSG00000088108 | Snora47 | M_musculus | Animals | intronic_cluster | H/ACA | ENSMUSG00000041995 | Zbed3 | protein_coding |
| ENSMUSG00000088111 | Gm24064 | M_musculus | Animals | mono_intergenic | H/ACA | | | intergenic |
| ENSMUSG00000088114 | Gm24061 | M_musculus | Animals | mono_intronic | H/ACA | ENSMUSG00000046032 | Snx12 | protein_coding |
| ENSMUSG00000088125 | Gm26070 | M_musculus | Animals | mono_intronic | H/ACA | ENSMUSG00000056427 | Slit3 | protein_coding |
| ENSMUSG00000088126 | Gm22870 | M_musculus | Animals | mono_intergenic | H/ACA | | | intergenic |
| ENSMUSG00000088134 | Gm22310 | M_musculus | Animals | mono_intergenic | H/ACA | | | intergenic |
| ENSMUSG00000088135 | Gm22588 | M_musculus | Animals | mono_intergenic | H/ACA | | | intergenic |
| ENSMUSG00000088145 | Gm24719 | M_musculus | Animals | mono_intergenic | H/ACA | | | intergenic |
| ENSMUSG00000088147 | Gm24717 | M_musculus | Animals | mono_intergenic | H/ACA | | | intergenic |
| ENSMUSG00000088155 | Gm26349 | M_musculus | Animals | mono_intergenic | H/ACA | | | intergenic |
| ENSMUSG00000088159 | Gm26345 | M_musculus | Animals | mono_intergenic | H/ACA | | | intergenic |
| ENSMUSG00000088164 | Gm23534 | M_musculus | Animals | mono_intergenic | H/ACA | | | intergenic |
| ENSMUSG00000088169 | Gm22059 | M_musculus | Animals | mono_intronic | C/D | ENSMUSG00000017210 | Med24 | protein_coding |
| ENSMUSG00000088191 | Gm23903 | M_musculus | Animals | mono_intergenic | H/ACA | | | intergenic |
| ENSMUSG00000088192 | Gm23904 | M_musculus | Animals | mono_intergenic | H/ACA | | | intergenic |
| ENSMUSG00000088196 | Gm23902 | M_musculus | Animals | mono_intergenic | H/ACA | | | intergenic |
| ENSMUSG00000088197 | Gm24138 | M_musculus | Animals | mono_intergenic | H/ACA | | | intergenic |
| ENSMUSG00000088198 | Gm23906 | M_musculus | Animals | mono_intergenic | H/ACA | | | intergenic |
| ENSMUSG00000088224 | Gm25406 | M_musculus | Animals | mono_intronic | H/ACA | ENSMUSG00000054469 | Lclat1 | protein_coding |
| ENSMUSG00000088225 | Gm22598 | M_musculus | Animals | mono_intergenic | H/ACA | | | intergenic |
| ENSMUSG00000088228 | Gm22603 | M_musculus | Animals | mono_intergenic | H/ACA | | | intergenic |
| ENSMUSG00000088232 | Gm25429 | M_musculus | Animals | mono_intergenic | H/ACA | | | intergenic |

| | | | | | | | | |
|---|---|---|---|---|---|---|---|---|
| ENSMUSG00000088240 | Gm22915 | M_musculus | Animals | mono_intergenic | H/ACA | | | intergenic |
| ENSMUSG00000088241 | Gm22916 | M_musculus | Animals | mono_intergenic | H/ACA | | | intergenic |
| ENSMUSG00000088244 | Gm25909 | M_musculus | Animals | mono_intergenic | H/ACA | | | intergenic |
| ENSMUSG00000088245 | Gm25910 | M_musculus | Animals | mono_intergenic | H/ACA | | | intergenic |
| ENSMUSG00000088252 | Snord13 | M_musculus | Animals | mono_intergenic | C/D | | | intergenic |
| ENSMUSG00000088255 | Gm24288 | M_musculus | Animals | mono_intergenic | H/ACA | | | intergenic |
| ENSMUSG00000088257 | Gm24286 | M_musculus | Animals | mono_intergenic | H/ACA | | | intergenic |
| ENSMUSG00000088260 | Gm24780 | M_musculus | Animals | mono_intergenic | H/ACA | | | intergenic |
| ENSMUSG00000088261 | Gm24781 | M_musculus | Animals | mono_intergenic | H/ACA | | | intergenic |
| ENSMUSG00000088266 | Gm24782 | M_musculus | Animals | mono_intergenic | H/ACA | | | intergenic |
| ENSMUSG00000088273 | Gm23123 | M_musculus | Animals | mono_intergenic | H/ACA | | | intergenic |
| ENSMUSG00000088282 | Gm23586 | M_musculus | Animals | mono_intergenic | H/ACA | | | intergenic |
| ENSMUSG00000088297 | Gm26408 | M_musculus | Animals | mono_intergenic | H/ACA | | | intergenic |
| ENSMUSG00000088303 | Gm23382 | M_musculus | Animals | mono_intergenic | H/ACA | | | intergenic |
| ENSMUSG00000088307 | Gm24505 | M_musculus | Animals | mono_intergenic | H/ACA | | | intergenic |
| ENSMUSG00000088308 | Gm24507 | M_musculus | Animals | mono_intronic | H/ACA | ENSMUSG00000012422 | Tmem167 | protein_coding |
| ENSMUSG00000088311 | Gm26184 | M_musculus | Animals | mono_intergenic | H/ACA | | | intergenic |
| ENSMUSG00000088313 | Gm26182 | M_musculus | Animals | mono_intergenic | H/ACA | | | intergenic |
| ENSMUSG00000088320 | Gm25665 | M_musculus | Animals | mono_intergenic | H/ACA | | | intergenic |
| ENSMUSG00000088323 | Gm25663 | M_musculus | Animals | mono_intronic | H/ACA | ENSMUSG00000018189 | Uchl5 | protein_coding |
| ENSMUSG00000088325 | Gm25668 | M_musculus | Animals | mono_intergenic | H/ACA | | | intergenic |
| ENSMUSG00000088346 | Gm22337 | M_musculus | Animals | mono_intergenic | H/ACA | | | intergenic |
| ENSMUSG00000088351 | Gm24016 | M_musculus | Animals | mono_intronic | C/D | ENSMUSG00000028234 | Rps20 | protein_coding |
| ENSMUSG00000088352 | Gm24013 | M_musculus | Animals | mono_intergenic | H/ACA | | | intergenic |
| ENSMUSG00000088358 | Gm24008 | M_musculus | Animals | mono_intergenic | H/ACA | | | intergenic |
| ENSMUSG00000088362 | Gm23498 | M_musculus | Animals | mono_intergenic | H/ACA | | | intergenic |
| ENSMUSG00000088372 | Gm22881 | M_musculus | Animals | mono_intergenic | H/ACA | | | intergenic |
| ENSMUSG00000088374 | Gm22882 | M_musculus | Animals | mono_intergenic | H/ACA | | | intergenic |
| ENSMUSG00000088377 | Gm25183 | M_musculus | Animals | mono_intergenic | H/ACA | | | intergenic |
| ENSMUSG00000088379 | Gm25185 | M_musculus | Animals | mono_intergenic | H/ACA | | | intergenic |
| ENSMUSG00000088391 | Gm26310 | M_musculus | Animals | mono_intergenic | H/ACA | | | intergenic |
| ENSMUSG00000088392 | Gm26307 | M_musculus | Animals | mono_intergenic | H/ACA | | | intergenic |
| ENSMUSG00000088398 | Gm26306 | M_musculus | Animals | mono_intergenic | H/ACA | | | intergenic |
| ENSMUSG00000088410 | Gm24240 | M_musculus | Animals | mono_intergenic | H/ACA | | | intergenic |
| ENSMUSG00000088412 | Gm24241 | M_musculus | Animals | mono_intronic | H/ACA | ENSMUSG00000019988 | Nedd1 | protein_coding |
| ENSMUSG00000088418 | Gm26162 | M_musculus | Animals | mono_intergenic | H/ACA | | | intergenic |
| ENSMUSG00000088419 | Gm24236 | M_musculus | Animals | mono_intergenic | H/ACA | | | intergenic |
| ENSMUSG00000088420 | Gm22551 | M_musculus | Animals | mono_intergenic | H/ACA | | | intergenic |
| ENSMUSG00000088423 | Gm22554 | M_musculus | Animals | mono_intergenic | H/ACA | | | intergenic |
| ENSMUSG00000088425 | Gm24422 | M_musculus | Animals | mono_intergenic | H/ACA | | | intergenic |
| ENSMUSG00000088435 | Gm25383 | M_musculus | Animals | mono_intergenic | H/ACA | | | intergenic |

| | | | | | | | | |
|---|---|---|---|---|---|---|---|---|
| ENSMUSG00000088440 | Gm23555 | M_musculus | Animals | mono_intergenic | H/ACA | | | intergenic |
| ENSMUSG00000088443 | Gm23554 | M_musculus | Animals | mono_intergenic | H/ACA | | | intergenic |
| ENSMUSG00000088444 | Gm22187 | M_musculus | Animals | mono_intergenic | H/ACA | | | intergenic |
| ENSMUSG00000088447 | Gm22186 | M_musculus | Animals | mono_intronic | H/ACA | ENSMUSG00000101801 | 1700020G17Rik | non_coding |
| ENSMUSG00000088451 | Gm26368 | M_musculus | Animals | mono_intronic | H/ACA | ENSMUSG00000025742 | Prps2 | protein_coding |
| ENSMUSG00000088465 | Gm24745 | M_musculus | Animals | mono_intergenic | H/ACA | | | intergenic |
| ENSMUSG00000088469 | Gm24751 | M_musculus | Animals | mono_intergenic | H/ACA | | | intergenic |
| ENSMUSG00000088472 | Gm23079 | M_musculus | Animals | mono_intergenic | H/ACA | | | intergenic |
| ENSMUSG00000088476 | Gm23083 | M_musculus | Animals | mono_intergenic | H/ACA | | | intergenic |
| ENSMUSG00000088477 | Gm23082 | M_musculus | Animals | mono_intergenic | H/ACA | | | intergenic |
| ENSMUSG00000088479 | Gm23077 | M_musculus | Animals | mono_intergenic | H/ACA | | | intergenic |
| ENSMUSG00000088481 | Gm23644 | M_musculus | Animals | mono_intergenic | H/ACA | | | intergenic |
| ENSMUSG00000088484 | Gm23592 | M_musculus | Animals | mono_intergenic | H/ACA | | | intergenic |
| ENSMUSG00000088493 | Gm24401 | M_musculus | Animals | mono_intronic | H/ACA | ENSMUSG00000103144 | Pcdhga1 | protein_coding |
| ENSMUSG00000088495 | Gm24398 | M_musculus | Animals | mono_intergenic | H/ACA | | | intergenic |
| ENSMUSG00000088504 | Gm26490 | M_musculus | Animals | mono_intergenic | H/ACA | | | intergenic |
| ENSMUSG00000088509 | Gm22789 | M_musculus | Animals | mono_intergenic | H/ACA | | | intergenic |
| ENSMUSG00000088510 | Gm23036 | M_musculus | Animals | mono_intergenic | H/ACA | | | intergenic |
| ENSMUSG00000088512 | Gm24467 | M_musculus | Animals | mono_intergenic | H/ACA | | | intergenic |
| ENSMUSG00000088515 | Gm23043 | M_musculus | Animals | mono_intergenic | H/ACA | | | intergenic |
| ENSMUSG00000088521 | Gm26088 | M_musculus | Animals | mono_intergenic | H/ACA | | | intergenic |
| ENSMUSG00000088524 | Snord2 | M_musculus | Animals | mono_intronic | C/D | ENSMUSG00000022884 | Eif4a2 | protein_coding |
| ENSMUSG00000088525 | Gm26085 | M_musculus | Animals | mono_intergenic | H/ACA | | | intergenic |
| ENSMUSG00000088530 | Gm23280 | M_musculus | Animals | mono_intergenic | H/ACA | | | intergenic |
| ENSMUSG00000088533 | Gm24998 | M_musculus | Animals | mono_intergenic | H/ACA | | | intergenic |
| ENSMUSG00000088536 | Gm23278 | M_musculus | Animals | mono_intergenic | H/ACA | | | intergenic |
| ENSMUSG00000088538 | Gm23276 | M_musculus | Animals | mono_intergenic | H/ACA | | | intergenic |
| ENSMUSG00000088539 | Gm23277 | M_musculus | Animals | mono_intergenic | H/ACA | | | intergenic |
| ENSMUSG00000088541 | Gm24963 | M_musculus | Animals | mono_intergenic | H/ACA | | | intergenic |
| ENSMUSG00000088550 | Gm22099 | M_musculus | Animals | mono_intergenic | H/ACA | | | intergenic |
| ENSMUSG00000088558 | Gm22506 | M_musculus | Animals | mono_intergenic | H/ACA | | | intergenic |
| ENSMUSG00000088577 | Gm25456 | M_musculus | Animals | mono_intergenic | H/ACA | | | intergenic |
| ENSMUSG00000088578 | Gm25459 | M_musculus | Animals | mono_intergenic | H/ACA | | | intergenic |
| ENSMUSG00000088579 | Gm25460 | M_musculus | Animals | mono_intergenic | H/ACA | | | intergenic |
| ENSMUSG00000088582 | Gm22966 | M_musculus | Animals | mono_intergenic | H/ACA | | | intergenic |
| ENSMUSG00000088594 | Gm23965 | M_musculus | Animals | mono_intergenic | H/ACA | | | intergenic |
| ENSMUSG00000088621 | Gm23033 | M_musculus | Animals | mono_intergenic | H/ACA | | | intergenic |
| ENSMUSG00000088626 | Gm23034 | M_musculus | Animals | mono_intergenic | H/ACA | | | intergenic |
| ENSMUSG00000088628 | Gm23315 | M_musculus | Animals | mono_intergenic | H/ACA | | | intergenic |
| ENSMUSG00000088630 | Gm22312 | M_musculus | Animals | mono_intergenic | H/ACA | | | intergenic |
| ENSMUSG00000088633 | Gm22313 | M_musculus | Animals | intronic_cluster | H/ACA | ENSMUSG00000050587 | Lrrc4c | protein_coding |

| | | | | | | | | |
|---|---|---|---|---|---|---|---|---|
| ENSMUSG00000088634 | Gm24958 | M_musculus | Animals | mono_intronic | C/D | ENSMUSG00000009090 | Ap1b1 | protein_coding |
| ENSMUSG00000088635 | Gm22315 | M_musculus | Animals | mono_intergenic | H/ACA | | | intergenic |
| ENSMUSG00000088638 | Gm25846 | M_musculus | Animals | mono_intergenic | H/ACA | | | intergenic |
| ENSMUSG00000088649 | Gm26262 | M_musculus | Animals | mono_intergenic | H/ACA | | | intergenic |
| ENSMUSG00000088651 | Gm24853 | M_musculus | Animals | mono_intergenic | H/ACA | | | intergenic |
| ENSMUSG00000088654 | Gm24850 | M_musculus | Animals | mono_intergenic | H/ACA | | | intergenic |
| ENSMUSG00000088669 | Gm25336 | M_musculus | Animals | mono_intronic | H/ACA | ENSMUSG00000100627 | A830008E24Rik | non_coding |
| ENSMUSG00000088678 | Gm23666 | M_musculus | Animals | mono_intergenic | H/ACA | | | intergenic |
| ENSMUSG00000088684 | Gm23994 | M_musculus | Animals | mono_intergenic | H/ACA | | | intergenic |
| ENSMUSG00000088685 | Gm23995 | M_musculus | Animals | mono_intronic | H/ACA | ENSMUSG00000045482 | Trrap | protein_coding |
| ENSMUSG00000088687 | Gm23996 | M_musculus | Animals | mono_intergenic | H/ACA | | | intergenic |
| ENSMUSG00000088688 | Gm24000 | M_musculus | Animals | mono_intergenic | H/ACA | | | intergenic |
| ENSMUSG00000088691 | Gm22331 | M_musculus | Animals | mono_intergenic | H/ACA | | | intergenic |
| ENSMUSG00000088693 | Gm22330 | M_musculus | Animals | mono_intergenic | H/ACA | | | intergenic |
| ENSMUSG00000088695 | Gm22334 | M_musculus | Animals | mono_intronic | H/ACA | ENSMUSG00000038324 | Trpc4ap | protein_coding |
| ENSMUSG00000088697 | Gm22332 | M_musculus | Animals | mono_intergenic | H/ACA | | | intergenic |
| ENSMUSG00000088704 | Gm25550 | M_musculus | Animals | mono_intronic | H/ACA | ENSMUSG00000115266 | 4930557F08Rik | non_coding |
| ENSMUSG00000088715 | Gm22727 | M_musculus | Animals | mono_intergenic | H/ACA | | | intergenic |
| ENSMUSG00000088716 | Gm22724 | M_musculus | Animals | mono_intergenic | H/ACA | | | intergenic |
| ENSMUSG00000088717 | Gm22725 | M_musculus | Animals | mono_intronic | H/ACA | ENSMUSG00000033644 | Piwil2 | protein_coding |
| ENSMUSG00000088718 | Gm22732 | M_musculus | Animals | mono_intronic | H/ACA | ENSMUSG00000086026 | Gm12374 | non_coding |
| ENSMUSG00000088726 | Gm22210 | M_musculus | Animals | mono_intergenic | H/ACA | | | intergenic |
| ENSMUSG00000088730 | Gm23892 | M_musculus | Animals | mono_intergenic | H/ACA | | | intergenic |
| ENSMUSG00000088734 | Gm23896 | M_musculus | Animals | mono_intergenic | H/ACA | | | intergenic |
| ENSMUSG00000088735 | Gm23897 | M_musculus | Animals | mono_intergenic | H/ACA | | | intergenic |
| ENSMUSG00000088739 | Gm23891 | M_musculus | Animals | mono_intergenic | H/ACA | | | intergenic |
| ENSMUSG00000088740 | Gm23220 | M_musculus | Animals | mono_intronic | H/ACA | ENSMUSG00000050439 | Enthd1 | protein_coding |
| ENSMUSG00000088744 | Gm23223 | M_musculus | Animals | mono_intergenic | H/ACA | | | intergenic |
| ENSMUSG00000088746 | Gm23225 | M_musculus | Animals | mono_intergenic | H/ACA | | | intergenic |
| ENSMUSG00000088751 | Gm24902 | M_musculus | Animals | mono_intergenic | H/ACA | | | intergenic |
| ENSMUSG00000088756 | Gm24903 | M_musculus | Animals | mono_intergenic | H/ACA | | | intergenic |
| ENSMUSG00000088764 | Gm24395 | M_musculus | Animals | mono_intergenic | H/ACA | | | intergenic |
| ENSMUSG00000088766 | Gm24394 | M_musculus | Animals | mono_intergenic | H/ACA | | | intergenic |
| ENSMUSG00000088772 | Gm26040 | M_musculus | Animals | mono_intergenic | H/ACA | | | intergenic |
| ENSMUSG00000088774 | Gm26036 | M_musculus | Animals | mono_intergenic | H/ACA | | | intergenic |
| ENSMUSG00000088781 | Gm25373 | M_musculus | Animals | mono_intergenic | H/ACA | | | intergenic |
| ENSMUSG00000088782 | Gm25375 | M_musculus | Animals | mono_intergenic | H/ACA | | | intergenic |
| ENSMUSG00000088784 | Gm25370 | M_musculus | Animals | mono_intergenic | H/ACA | | | intergenic |
| ENSMUSG00000088792 | Gm22539 | M_musculus | Animals | mono_intergenic | H/ACA | | | intergenic |
| ENSMUSG00000088796 | Gm22536 | M_musculus | Animals | mono_intergenic | H/ACA | | | intergenic |
| ENSMUSG00000088800 | Gm24075 | M_musculus | Animals | mono_intergenic | H/ACA | | | intergenic |

| | | | | | | | | |
|---|---|---|---|---|---|---|---|---|
| ENSMUSG00000088810 | Gm22382 | M_musculus | Animals | mono_intergenic | H/ACA | | | intergenic |
| ENSMUSG00000088817 | Gm22386 | M_musculus | Animals | mono_intergenic | H/ACA | | | intergenic |
| ENSMUSG00000088820 | Gm25224 | M_musculus | Animals | mono_intronic | C/D | ENSMUSG00000098202 | B830012L14Rik | non_coding |
| ENSMUSG00000088823 | Gm25226 | M_musculus | Animals | mono_intergenic | H/ACA | | | intergenic |
| ENSMUSG00000088824 | Gm25222 | M_musculus | Animals | mono_intergenic | H/ACA | | | intergenic |
| ENSMUSG00000088829 | Gm25227 | M_musculus | Animals | mono_intergenic | H/ACA | | | intergenic |
| ENSMUSG00000088835 | Gm23547 | M_musculus | Animals | mono_intronic | C/D | ENSMUSG00000004264 | Phb2 | protein_coding |
| ENSMUSG00000088836 | Gm23546 | M_musculus | Animals | mono_intronic | H/ACA | ENSMUSG00000038831 | Ralgps1 | protein_coding |
| ENSMUSG00000088840 | Gm26358 | M_musculus | Animals | mono_intergenic | H/ACA | | | intergenic |
| ENSMUSG00000088846 | Gm26354 | M_musculus | Animals | mono_intergenic | H/ACA | | | intergenic |
| ENSMUSG00000088848 | Gm26352 | M_musculus | Animals | mono_intergenic | H/ACA | | | intergenic |
| ENSMUSG00000088850 | Gm24729 | M_musculus | Animals | mono_intronic | H/ACA | ENSMUSG00000015222 | Map2 | protein_coding |
| ENSMUSG00000088851 | Gm24728 | M_musculus | Animals | mono_intergenic | H/ACA | | | intergenic |
| ENSMUSG00000088852 | Gm24731 | M_musculus | Animals | mono_intronic | H/ACA | ENSMUSG00000112112 | Gm48508 | non_coding |
| ENSMUSG00000088853 | Gm24730 | M_musculus | Animals | mono_intergenic | H/ACA | | | intergenic |
| ENSMUSG00000088857 | Gm24726 | M_musculus | Animals | mono_intergenic | H/ACA | | | intergenic |
| ENSMUSG00000088858 | Gm24723 | M_musculus | Animals | mono_intergenic | H/ACA | | | intergenic |
| ENSMUSG00000088868 | Gm23072 | M_musculus | Animals | mono_intergenic | H/ACA | | | intergenic |
| ENSMUSG00000088870 | Gm25866 | M_musculus | Animals | mono_intergenic | H/ACA | | | intergenic |
| ENSMUSG00000088875 | Gm25862 | M_musculus | Animals | mono_intergenic | H/ACA | | | intergenic |
| ENSMUSG00000088876 | Gm25861 | M_musculus | Animals | mono_intergenic | H/ACA | | | intergenic |
| ENSMUSG00000088877 | Gm25860 | M_musculus | Animals | mono_intergenic | H/ACA | | | intergenic |
| ENSMUSG00000088883 | Gm24212 | M_musculus | Animals | mono_intergenic | H/ACA | | | intergenic |
| ENSMUSG00000088884 | Gm24216 | M_musculus | Animals | mono_intergenic | H/ACA | | | intergenic |
| ENSMUSG00000088888 | Gm24210 | M_musculus | Animals | mono_intergenic | H/ACA | | | intergenic |
| ENSMUSG00000088891 | Gm22516 | M_musculus | Animals | mono_intronic | H/ACA | ENSMUSG00000000194 | Gpr107 | protein_coding |
| ENSMUSG00000088894 | Gm22520 | M_musculus | Animals | mono_intergenic | H/ACA | | | intergenic |
| ENSMUSG00000088895 | Gm22519 | M_musculus | Animals | mono_intergenic | H/ACA | | | intergenic |
| ENSMUSG00000088896 | Gm22522 | M_musculus | Animals | mono_intergenic | H/ACA | | | intergenic |
| ENSMUSG00000088897 | Gm22521 | M_musculus | Animals | mono_intergenic | H/ACA | | | intergenic |
| ENSMUSG00000088900 | Gm25436 | M_musculus | Animals | mono_intergenic | H/ACA | | | intergenic |
| ENSMUSG00000088905 | Gm25439 | M_musculus | Animals | mono_intronic | H/ACA | ENSMUSG00000110771 | Gm47785 | non_coding |
| ENSMUSG00000088906 | Gm25438 | M_musculus | Animals | mono_intergenic | H/ACA | | | intergenic |
| ENSMUSG00000088907 | Gm25437 | M_musculus | Animals | mono_intergenic | H/ACA | | | intergenic |
| ENSMUSG00000088911 | Gm22609 | M_musculus | Animals | mono_intergenic | H/ACA | | | intergenic |
| ENSMUSG00000088923 | Gm24295 | M_musculus | Animals | mono_intergenic | H/ACA | | | intergenic |
| ENSMUSG00000088924 | Gm24298 | M_musculus | Animals | mono_intergenic | H/ACA | | | intergenic |
| ENSMUSG00000088929 | Gm24299 | M_musculus | Animals | mono_intronic | C/D | ENSMUSG00000031939 | Taf1d | protein_coding |
| ENSMUSG00000088930 | Gm25921 | M_musculus | Animals | mono_intergenic | H/ACA | | | intergenic |
| ENSMUSG00000088948 | Gm23262 | M_musculus | Animals | mono_intronic | C/D | ENSMUSG00000005610 | Eif4g2 | protein_coding |
| ENSMUSG00000088953 | Gm24942 | M_musculus | Animals | mono_intergenic | H/ACA | | | intergenic |

| | | | | | | | | |
|---|---|---|---|---|---|---|---|---|
| ENSMUSG00000088975 | Gm23760 | M_musculus | Animals | mono_intergenic | H/ACA | | | intergenic |
| ENSMUSG00000088979 | Gm23758 | M_musculus | Animals | mono_intergenic | H/ACA | | | intergenic |
| ENSMUSG00000088984 | Gm25604 | M_musculus | Animals | mono_intergenic | H/ACA | | | intergenic |
| ENSMUSG00000088999 | Gm22763 | M_musculus | Animals | mono_intergenic | H/ACA | | | intergenic |
| ENSMUSG00000089001 | Gm23852 | M_musculus | Animals | mono_intergenic | C/D | | | intergenic |
| ENSMUSG00000089002 | Gm23855 | M_musculus | Animals | mono_intergenic | H/ACA | | | intergenic |
| ENSMUSG00000089007 | Gm23877 | M_musculus | Animals | mono_intergenic | H/ACA | | | intergenic |
| ENSMUSG00000089011 | Gm24879 | M_musculus | Animals | mono_intergenic | H/ACA | | | intergenic |
| ENSMUSG00000089014 | Gm25520 | M_musculus | Animals | mono_intronic | H/ACA | ENSMUSG00000031403 | Dkc1 | protein_coding |
| ENSMUSG00000089017 | Gm25519 | M_musculus | Animals | mono_intergenic | H/ACA | | | intergenic |
| ENSMUSG00000089024 | Gm25016 | M_musculus | Animals | mono_intronic | H/ACA | ENSMUSG00000039166 | Akap7 | protein_coding |
| ENSMUSG00000089025 | Gm25015 | M_musculus | Animals | mono_intergenic | H/ACA | | | intergenic |
| ENSMUSG00000089027 | Gm25013 | M_musculus | Animals | mono_intronic | H/ACA | ENSMUSG00000098097 | 6530403H02Rik | non_coding |
| ENSMUSG00000089029 | Gm25009 | M_musculus | Animals | mono_intergenic | H/ACA | | | intergenic |
| ENSMUSG00000089033 | Gm22163 | M_musculus | Animals | mono_intronic | H/ACA | ENSMUSG00000052516 | Robo2 | protein_coding |
| ENSMUSG00000089034 | Gm22156 | M_musculus | Animals | mono_intergenic | H/ACA | | | intergenic |
| ENSMUSG00000089036 | Gm22158 | M_musculus | Animals | mono_intergenic | H/ACA | | | intergenic |
| ENSMUSG00000089037 | Gm22159 | M_musculus | Animals | mono_intergenic | H/ACA | | | intergenic |
| ENSMUSG00000089045 | Gm26169 | M_musculus | Animals | mono_intergenic | H/ACA | | | intergenic |
| ENSMUSG00000089046 | Gm26172 | M_musculus | Animals | mono_intergenic | H/ACA | | | intergenic |
| ENSMUSG00000089052 | Gm23351 | M_musculus | Animals | mono_intergenic | H/ACA | | | intergenic |
| ENSMUSG00000089069 | Gm22840 | M_musculus | Animals | mono_intergenic | H/ACA | | | intergenic |
| ENSMUSG00000089079 | Gm24503 | M_musculus | Animals | mono_intergenic | H/ACA | | | intergenic |
| ENSMUSG00000089087 | Gm24006 | M_musculus | Animals | mono_intergenic | H/ACA | | | intergenic |
| ENSMUSG00000089093 | Snord11 | M_musculus | Animals | mono_intronic | C/D | ENSMUSG00000026020 | Nop58 | protein_coding |
| ENSMUSG00000089097 | Gm25658 | M_musculus | Animals | mono_intronic | H/ACA | ENSMUSG00000074776 | Gm10754 | non_coding |
| ENSMUSG00000089098 | Gm25653 | M_musculus | Animals | mono_intergenic | H/ACA | | | intergenic |
| ENSMUSG00000089114 | Gm25906 | M_musculus | Animals | mono_intergenic | H/ACA | | | intergenic |
| ENSMUSG00000089116 | Gm25904 | M_musculus | Animals | mono_intergenic | H/ACA | | | intergenic |
| ENSMUSG00000089119 | Gm25901 | M_musculus | Animals | mono_intergenic | H/ACA | | | intergenic |
| ENSMUSG00000089123 | Gm26404 | M_musculus | Animals | mono_intergenic | H/ACA | | | intergenic |
| ENSMUSG00000089127 | Gm26401 | M_musculus | Animals | mono_intergenic | H/ACA | | | intergenic |
| ENSMUSG00000089128 | Gm26406 | M_musculus | Animals | mono_intergenic | H/ACA | | | intergenic |
| ENSMUSG00000089130 | Gm24770 | M_musculus | Animals | mono_intergenic | H/ACA | | | intergenic |
| ENSMUSG00000089133 | Gm24771 | M_musculus | Animals | mono_intronic | H/ACA | ENSMUSG00000037646 | Vps13b | protein_coding |
| ENSMUSG00000089144 | Gm25264 | M_musculus | Animals | mono_intergenic | H/ACA | | | intergenic |
| ENSMUSG00000089152 | Gm22424 | M_musculus | Animals | mono_intergenic | H/ACA | | | intergenic |
| ENSMUSG00000089155 | Gm23582 | M_musculus | Animals | mono_intergenic | H/ACA | | | intergenic |
| ENSMUSG00000089157 | Gm22425 | M_musculus | Animals | mono_intergenic | H/ACA | | | intergenic |
| ENSMUSG00000089161 | Gm24130 | M_musculus | Animals | mono_intergenic | H/ACA | | | intergenic |
| ENSMUSG00000089171 | Gm22430 | M_musculus | Animals | mono_intergenic | H/ACA | | | intergenic |

| | | | | | | | | |
|---|---|---|---|---|---|---|---|---|
| ENSMUSG00000089174 | Gm22434 | M_musculus | Animals | mono_intergenic | H/ACA | | | intergenic |
| ENSMUSG00000089178 | Gm22429 | M_musculus | Animals | mono_intergenic | H/ACA | | | intergenic |
| ENSMUSG00000089179 | Gm22428 | M_musculus | Animals | mono_intergenic | H/ACA | | | intergenic |
| ENSMUSG00000089184 | Gm22947 | M_musculus | Animals | mono_intergenic | H/ACA | | | intergenic |
| ENSMUSG00000089188 | Gm22942 | M_musculus | Animals | mono_intergenic | H/ACA | | | intergenic |
| ENSMUSG00000089190 | Gm25771 | M_musculus | Animals | mono_intergenic | H/ACA | | | intergenic |
| ENSMUSG00000089191 | Gm25770 | M_musculus | Animals | mono_intergenic | H/ACA | | | intergenic |
| ENSMUSG00000089194 | Gm25769 | M_musculus | Animals | mono_intergenic | H/ACA | | | intergenic |
| ENSMUSG00000089197 | Gm25767 | M_musculus | Animals | mono_intergenic | H/ACA | | | intergenic |
| ENSMUSG00000089203 | Gm26211 | M_musculus | Animals | mono_intergenic | H/ACA | | | intergenic |
| ENSMUSG00000089220 | Gm22074 | M_musculus | Animals | mono_intergenic | H/ACA | | | intergenic |
| ENSMUSG00000089231 | Gm23112 | M_musculus | Animals | mono_intergenic | H/ACA | | | intergenic |
| ENSMUSG00000089237 | Gm22206 | M_musculus | Animals | mono_intergenic | H/ACA | | | intergenic |
| ENSMUSG00000089239 | Gm22200 | M_musculus | Animals | mono_intronic | C/D | ENSMUSG00000024359 | Hspa9 | protein_coding |
| ENSMUSG00000089252 | Gm25715 | M_musculus | Animals | mono_intronic | H/ACA | ENSMUSG00000116810 | Gm32624 | non_coding |
| ENSMUSG00000089255 | Snora78 | M_musculus | Animals | mono_intronic | H/ACA | ENSMUSG00000090101 | Snhg9 | non_coding |
| ENSMUSG00000089258 | Gm25711 | M_musculus | Animals | mono_intronic | H/ACA | ENSMUSG00000040452 | Cdh12 | protein_coding |
| ENSMUSG00000089262 | Gm22893 | M_musculus | Animals | mono_intergenic | H/ACA | | | intergenic |
| ENSMUSG00000089264 | Gm22889 | M_musculus | Animals | mono_intergenic | Unknown | | | intergenic |
| ENSMUSG00000089265 | Gm22888 | M_musculus | Animals | mono_intronic | C/D | ENSMUSG00000028433 | Ubap2 | protein_coding |
| ENSMUSG00000089267 | Gm22890 | M_musculus | Animals | mono_intergenic | H/ACA | | | intergenic |
| ENSMUSG00000089272 | Gm24549 | M_musculus | Animals | mono_intergenic | H/ACA | | | intergenic |
| ENSMUSG00000089275 | Gm24548 | M_musculus | Animals | mono_intergenic | H/ACA | | | intergenic |
| ENSMUSG00000089278 | Gm24553 | M_musculus | Animals | mono_intergenic | H/ACA | | | intergenic |
| ENSMUSG00000089279 | Gm24554 | M_musculus | Animals | intergenic_cluster | H/ACA | | | intergenic |
| ENSMUSG00000089284 | Gm26029 | M_musculus | Animals | mono_intergenic | H/ACA | | | intergenic |
| ENSMUSG00000089287 | Gm22944 | M_musculus | Animals | mono_intergenic | H/ACA | | | intergenic |
| ENSMUSG00000089289 | Gm26021 | M_musculus | Animals | mono_intergenic | H/ACA | | | intergenic |
| ENSMUSG00000089299 | Gm24637 | M_musculus | Animals | mono_intergenic | H/ACA | | | intergenic |
| ENSMUSG00000089302 | Gm26208 | M_musculus | Animals | mono_intergenic | H/ACA | | | intergenic |
| ENSMUSG00000089304 | Gm24844 | M_musculus | Animals | mono_intergenic | H/ACA | | | intergenic |
| ENSMUSG00000089307 | Gm24843 | M_musculus | Animals | mono_intergenic | H/ACA | | | intergenic |
| ENSMUSG00000089308 | Gm24837 | M_musculus | Animals | mono_intronic | C/D | ENSMUSG00000028433 | Ubap2 | protein_coding |
| ENSMUSG00000089309 | Gm24838 | M_musculus | Animals | mono_intergenic | H/ACA | | | intergenic |
| ENSMUSG00000089310 | Gm23181 | M_musculus | Animals | mono_intergenic | H/ACA | | | intergenic |
| ENSMUSG00000089311 | Gm23180 | M_musculus | Animals | mono_intergenic | H/ACA | | | intergenic |
| ENSMUSG00000089313 | Gm23182 | M_musculus | Animals | mono_intronic | C/D | ENSMUSG00000005683 | Cs | protein_coding |
| ENSMUSG00000089317 | Snord123 | M_musculus | Animals | mono_intronic | C/D | ENSMUSG00000096956 | Snhg18 | non_coding |
| ENSMUSG00000089320 | Gm25976 | M_musculus | Animals | mono_intergenic | H/ACA | | | intergenic |
| ENSMUSG00000089322 | Gm25977 | M_musculus | Animals | mono_intergenic | H/ACA | | | intergenic |
| ENSMUSG00000089327 | Gm25975 | M_musculus | Animals | mono_intergenic | H/ACA | | | intergenic |

| | | | | | | | | |
|---|---|---|---|---|---|---|---|---|
| ENSMUSG00000089331 | Gm24345 | M_musculus | Animals | mono_intergenic | H/ACA | | | intergenic |
| ENSMUSG00000089333 | Gm24343 | M_musculus | Animals | mono_intronic | H/ACA | ENSMUSG00000112284 | Gm48845 | non_coding |
| ENSMUSG00000089334 | Gm24349 | M_musculus | Animals | mono_intergenic | H/ACA | | | intergenic |
| ENSMUSG00000089335 | Gm24348 | M_musculus | Animals | mono_intergenic | H/ACA | | | intergenic |
| ENSMUSG00000089336 | Gm24347 | M_musculus | Animals | mono_intergenic | H/ACA | | | intergenic |
| ENSMUSG00000089349 | Gm22670 | M_musculus | Animals | mono_intergenic | H/ACA | | | intergenic |
| ENSMUSG00000089350 | Gm25487 | M_musculus | Animals | mono_intergenic | C/D | | | intergenic |
| ENSMUSG00000089359 | Gm25490 | M_musculus | Animals | mono_intergenic | H/ACA | | | intergenic |
| ENSMUSG00000089364 | Gm23822 | M_musculus | Animals | mono_intergenic | H/ACA | | | intergenic |
| ENSMUSG00000089373 | Gm22138 | M_musculus | Animals | mono_intergenic | H/ACA | | | intergenic |
| ENSMUSG00000089375 | Snord107 | M_musculus | Animals | mono_intronic | C/D | ENSMUSG00000000948 | Gm38393 | protein_coding |
| ENSMUSG00000089380 | Gm24677 | M_musculus | Animals | mono_intergenic | H/ACA | | | intergenic |
| ENSMUSG00000089384 | Gm24680 | M_musculus | Animals | mono_intergenic | H/ACA | | | intergenic |
| ENSMUSG00000089385 | Gm24681 | M_musculus | Animals | mono_intergenic | H/ACA | | | intergenic |
| ENSMUSG00000089387 | Gm24679 | M_musculus | Animals | mono_intergenic | H/ACA | | | intergenic |
| ENSMUSG00000089409 | Gm24884 | M_musculus | Animals | mono_intergenic | H/ACA | | | intergenic |
| ENSMUSG00000089415 | Gm22011 | M_musculus | Animals | mono_intergenic | H/ACA | | | intergenic |
| ENSMUSG00000089425 | Gm26006 | M_musculus | Animals | mono_intergenic | H/ACA | | | intergenic |
| ENSMUSG00000089427 | Gm26005 | M_musculus | Animals | mono_intergenic | H/ACA | | | intergenic |
| ENSMUSG00000089439 | Gm23195 | M_musculus | Animals | mono_intronic | H/ACA | ENSMUSG00000097157 | Gm26512 | non_coding |
| ENSMUSG00000089442 | Gm22529 | M_musculus | Animals | mono_intergenic | H/ACA | | | intergenic |
| ENSMUSG00000089444 | Gm22531 | M_musculus | Animals | mono_intergenic | H/ACA | | | intergenic |
| ENSMUSG00000089445 | Gm22530 | M_musculus | Animals | mono_intergenic | H/ACA | | | intergenic |
| ENSMUSG00000089454 | Gm24228 | M_musculus | Animals | mono_intergenic | H/ACA | | | intergenic |
| ENSMUSG00000089455 | Gm24229 | M_musculus | Animals | mono_intergenic | H/ACA | | | intergenic |
| ENSMUSG00000089458 | Gm24221 | M_musculus | Animals | mono_intergenic | C/D | | | intergenic |
| ENSMUSG00000089460 | Gm23699 | M_musculus | Animals | mono_intergenic | H/ACA | | | intergenic |
| ENSMUSG00000089461 | Gm23698 | M_musculus | Animals | mono_intergenic | H/ACA | | | intergenic |
| ENSMUSG00000089464 | Gm23703 | M_musculus | Animals | mono_intergenic | H/ACA | | | intergenic |
| ENSMUSG00000089473 | Gm25369 | M_musculus | Animals | mono_intergenic | C/D | | | intergenic |
| ENSMUSG00000089501 | Gm23479 | M_musculus | Animals | mono_intergenic | H/ACA | | | intergenic |
| ENSMUSG00000089507 | Gm23477 | M_musculus | Animals | mono_intergenic | H/ACA | | | intergenic |
| ENSMUSG00000089512 | Gm26287 | M_musculus | Animals | mono_intronic | C/D | ENSMUSG00000026020 | Nop58 | protein_coding |
| ENSMUSG00000089514 | Gm26291 | M_musculus | Animals | mono_intergenic | H/ACA | | | intergenic |
| ENSMUSG00000089524 | Gm22327 | M_musculus | Animals | mono_intergenic | H/ACA | | | intergenic |
| ENSMUSG00000089528 | Gm22324 | M_musculus | Animals | mono_intergenic | H/ACA | | | intergenic |
| ENSMUSG00000089531 | Gm25162 | M_musculus | Animals | mono_intronic | H/ACA | ENSMUSG00000091155 | Serpine3 | protein_coding |
| ENSMUSG00000089534 | Gm25166 | M_musculus | Animals | mono_intergenic | H/ACA | | | intergenic |
| ENSMUSG00000089538 | Gm25161 | M_musculus | Animals | mono_intergenic | H/ACA | | | intergenic |
| ENSMUSG00000089542 | Gm25835 | M_musculus | Animals | mono_intronic | C/D | ENSMUSG00000059796 | Eif4a1 | protein_coding |
| ENSMUSG00000089543 | Gm25836 | M_musculus | Animals | mono_intergenic | H/ACA | | | intergenic |

| | | | | | | | | |
|---|---|---|---|---|---|---|---|---|
| ENSMUSG00000089548 | Gm25830 | M_musculus | Animals | mono_intergenic | H/ACA | | | intergenic |
| ENSMUSG00000089550 | Gm24179 | M_musculus | Animals | mono_intergenic | H/ACA | | | intergenic |
| ENSMUSG00000089552 | Gm24177 | M_musculus | Animals | mono_intergenic | H/ACA | | | intergenic |
| ENSMUSG00000089553 | Gm24176 | M_musculus | Animals | mono_intergenic | H/ACA | | | intergenic |
| ENSMUSG00000089556 | Gm24175 | M_musculus | Animals | mono_intergenic | H/ACA | | | intergenic |
| ENSMUSG00000089571 | Gm23008 | M_musculus | Animals | mono_intronic | C/D | ENSMUSG00000004100 | Ppan | protein_coding |
| ENSMUSG00000089575 | Gm23005 | M_musculus | Animals | mono_intergenic | H/ACA | | | intergenic |
| ENSMUSG00000089580 | Gm23340 | M_musculus | Animals | mono_intergenic | H/ACA | | | intergenic |
| ENSMUSG00000089585 | Gm23338 | M_musculus | Animals | mono_intergenic | H/ACA | | | intergenic |
| ENSMUSG00000089587 | Gm23339 | M_musculus | Animals | mono_intergenic | H/ACA | | | intergenic |
| ENSMUSG00000089589 | Gm23343 | M_musculus | Animals | mono_intergenic | H/ACA | | | intergenic |
| ENSMUSG00000089591 | Gm24352 | M_musculus | Animals | mono_intronic | H/ACA | ENSMUSG00000085895 | Gm12861 | non_coding |
| ENSMUSG00000089606 | Gm22501 | M_musculus | Animals | mono_intronic | H/ACA | ENSMUSG00000068373 | D430041D05Rik | protein_coding |
| ENSMUSG00000089615 | Gm23774 | M_musculus | Animals | mono_intergenic | H/ACA | | | intergenic |
| ENSMUSG00000089617 | Scarna10 | M_musculus | Animals | mono_intronic | C/D | ENSMUSG00000038252 | Ncapd2 | protein_coding |
| ENSMUSG00000092680 | Snord55 | M_musculus | Animals | mono_intronic | C/D | ENSMUSG00000047675 | Rps8 | protein_coding |
| ENSMUSG00000092687 | Gm25744 | M_musculus | Animals | mono_intronic | C/D | ENSMUSG00000092203 | 1110038B12Rik | non_coding |
| ENSMUSG00000092710 | Gm22857 | M_musculus | Animals | mono_intergenic | H/ACA | | | intergenic |
| ENSMUSG00000092713 | Gm22858 | M_musculus | Animals | mono_intronic | C/D | ENSMUSG00000041057 | Wdr43 | protein_coding |
| ENSMUSG00000092730 | Snora24 | M_musculus | Animals | mono_intronic | H/ACA | ENSMUSG00000104960 | Snhg8 | non_coding |
| ENSMUSG00000092819 | Gm23639 | M_musculus | Animals | mono_intronic | H/ACA | ENSMUSG00000087590 | Epb41l4aos | non_coding |
| ENSMUSG00000092887 | Snord53 | M_musculus | Animals | mono_intronic | C/D | ENSMUSG00000041057 | Wdr43 | protein_coding |
| ENSMUSG00000093002 | Gm24154 | M_musculus | Animals | mono_intronic | H/ACA | ENSMUSG00000085945 | 2310014F06Rik | non_coding |
| ENSMUSG00000093009 | Snord52 | M_musculus | Animals | mono_intronic | C/D | ENSMUSG00000092203 | 1110038B12Rik | non_coding |
| ENSMUSG00000093044 | Snord8 | M_musculus | Animals | mono_intronic | C/D | ENSMUSG00000053754 | Chd8 | protein_coding |
| ENSMUSG00000093139 | Gm25999 | M_musculus | Animals | mono_intergenic | H/ACA | | | intergenic |
| ENSMUSG00000093178 | Snord87 | M_musculus | Animals | mono_intronic | C/D | ENSMUSG00000098234 | Snhg6 | non_coding |
| ENSMUSG00000093277 | Gm26236 | M_musculus | Animals | mono_intronic | H/ACA | ENSMUSG00000026814 | Eng | protein_coding |
| ENSMUSG00000093289 | Snord92 | M_musculus | Animals | mono_intronic | C/D | ENSMUSG00000041057 | Wdr43 | protein_coding |
| ENSMUSG00000093355 | Snora26 | M_musculus | Animals | mono_exonic | H/ACA | ENSMUSG00000106943 | Dancr | non_coding |
| ENSMUSG00000093413 | Snora15 | M_musculus | Animals | mono_intronic | H/ACA | ENSMUSG00000029447 | Cct6a | protein_coding |
| ENSMUSG00000093443 | Gm22871 | M_musculus | Animals | mono_exonic | C/D | ENSMUSG00000009292 | Trpm2 | protein_coding |
| ENSMUSG00000093562 | Gm23090 | M_musculus | Animals | mono_intronic | C/D | ENSMUSG00000028433 | Ubap2 | protein_coding |
| ENSMUSG00000093645 | Gm23187 | M_musculus | Animals | mono_intronic | C/D | ENSMUSG00000027405 | Nop56 | protein_coding |
| ENSMUSG00000093784 | Gm22394 | M_musculus | Animals | mono_exonic | C/D | ENSMUSG00000009292 | Trpm2 | protein_coding |
| ENSMUSG00000094771 | Gm23860 | M_musculus | Animals | mono_intergenic | H/ACA | | | intergenic |
| ENSMUSG00000095349 | Gm26097 | M_musculus | Animals | intronic_cluster | C/D | ENSMUSG00000100826 | Snhg14 | non_coding |
| ENSMUSG00000095394 | Gm25485 | M_musculus | Animals | mono_intergenic | H/ACA | | | intergenic |
| ENSMUSG00000095971 | Gm26436 | M_musculus | Animals | mono_intergenic | H/ACA | | | intergenic |
| ENSMUSG00000096231 | Gm22584 | M_musculus | Animals | intronic_cluster | C/D | ENSMUSG00000100826 | Snhg14 | non_coding |
| ENSMUSG00000096896 | Gm25244 | M_musculus | Animals | mono_intergenic | H/ACA | | | intergenic |

| | | | | | | | | |
|---|---|---|---|---|---|---|---|---|
| ENSMUSG00000097052 | Snora43 | M_musculus | Animals | intronic_cluster | H/ACA | ENSMUSG00000120369 | Snhg7 | non_coding |
| ENSMUSG00000099280 | Gm27991 | M_musculus | Animals | mono_intergenic | H/ACA | | | intergenic |
| ENSMUSG00000104536 | Gm23786 | M_musculus | Animals | intergenic_cluster | H/ACA | | | intergenic |
| ENSMUSG00000104700 | Gm26379 | M_musculus | Animals | intergenic_cluster | H/ACA | | | intergenic |
| ENSMUSG00000104921 | Snord3b-ps1 | M_musculus | Animals | intergenic_cluster | C/D | | | intergenic |
| ENSMUSG00000105037 | Gm25203 | M_musculus | Animals | intergenic_cluster | C/D | | | intergenic |
| ENSMUSG00000105187 | Gm23377 | M_musculus | Animals | intergenic_cluster | H/ACA | | | intergenic |
| ENSMUSG00000105690 | Gm26265 | M_musculus | Animals | intronic_cluster | H/ACA | ENSMUSG00000036550 | Cnot1 | protein_coding |
| ENSMUSG00000106147 | Snord3a | M_musculus | Animals | intergenic_cluster | C/D | | | intergenic |
| ENSMUSG00000106585 | Gm25053 | M_musculus | Animals | intronic_cluster | H/ACA | ENSMUSG00000055296 | Tmem245 | protein_coding |
| ENSMUSG00000118674 | Gm26467 | M_musculus | Animals | mono_intergenic | C/D | ENSMUSG00000100826 | Snhg14 | non_coding |
| ENSMUSG00000118676 | Gm24585 | M_musculus | Animals | mono_intergenic | C/D | ENSMUSG00000100826 | Snhg14 | non_coding |
| ENSMUSG00000118680 | Gm26502 | M_musculus | Animals | intronic_cluster | C/D | ENSMUSG00000100826 | Snhg14 | non_coding |
| ENSMUSG00000118688 | Gm23076 | M_musculus | Animals | mono_intronic | C/D | ENSMUSG00000100826 | Snhg14 | non_coding |
| ENSMUSG00000118696 | Snord116l13 | M_musculus | Animals | intronic_cluster | C/D | ENSMUSG00000100826 | Snhg14 | non_coding |
| ENSMUSG00000118697 | Gm23803 | M_musculus | Animals | mono_intergenic | H/ACA | | | intergenic |
| ENSMUSG00000118699 | Gm24866 | M_musculus | Animals | mono_intronic | C/D | ENSMUSG00000100826 | Snhg14 | non_coding |
| ENSMUSG00000118700 | Gm23267 | M_musculus | Animals | mono_intergenic | H/ACA | | | intergenic |
| ENSMUSG00000118703 | Gm22367 | M_musculus | Animals | mono_intergenic | H/ACA | | | intergenic |
| ENSMUSG00000118706 | Snord14d | M_musculus | Animals | mono_intronic | C/D | ENSMUSG00000015656 | Hspa8 | protein_coding |
| ENSMUSG00000118711 | Gm24306 | M_musculus | Animals | mono_intergenic | C/D | ENSMUSG00000100826 | Snhg14 | non_coding |
| ENSMUSG00000118714 | Gm23439 | M_musculus | Animals | mono_intergenic | H/ACA | | | intergenic |
| ENSMUSG00000118716 | Gm24137 | M_musculus | Animals | mono_intergenic | C/D | ENSMUSG00000100826 | Snhg14 | non_coding |
| ENSMUSG00000118719 | Gm22780 | M_musculus | Animals | mono_intergenic | H/ACA | | | intergenic |
| ENSMUSG00000118720 | Gm26434 | M_musculus | Animals | mono_intergenic | C/D | ENSMUSG00000100826 | Snhg14 | non_coding |
| ENSMUSG00000118728 | Gm24463 | M_musculus | Animals | mono_intergenic | C/D | | | intergenic |
| ENSMUSG00000118729 | Snord88c | M_musculus | Animals | mono_intronic | C/D | ENSMUSG00000045411 | 2410002F23Rik | protein_coding |
| ENSMUSG00000118730 | Gm25157 | M_musculus | Animals | intronic_cluster | C/D | ENSMUSG00000100826 | Snhg14 | non_coding |
| ENSMUSG00000118732 | Gm22274 | M_musculus | Animals | mono_intergenic | C/D | ENSMUSG00000100826 | Snhg14 | non_coding |
| ENSMUSG00000118737 | Gm26188 | M_musculus | Animals | intronic_cluster | C/D | ENSMUSG00000100826 | Snhg14 | non_coding |
| ENSMUSG00000118744 | Gm50451 | M_musculus | Animals | mono_intronic | C/D | ENSMUSG00000105987 | AI506816 | non_coding |
| ENSMUSG00000118748 | Gm23132 | M_musculus | Animals | mono_intergenic | H/ACA | | | intergenic |
| ENSMUSG00000118753 | Gm22439 | M_musculus | Animals | mono_intergenic | H/ACA | | | intergenic |
| ENSMUSG00000118755 | Gm24021 | M_musculus | Animals | mono_intergenic | C/D | ENSMUSG00000100826 | Snhg14 | non_coding |
| ENSMUSG00000118760 | Snord116l10 | M_musculus | Animals | intronic_cluster | C/D | ENSMUSG00000100826 | Snhg14 | non_coding |
| ENSMUSG00000118761 | Gm25597 | M_musculus | Animals | intronic_cluster | C/D | ENSMUSG00000100826 | Snhg14 | non_coding |
| ENSMUSG00000118777 | Gm22899 | M_musculus | Animals | mono_intergenic | H/ACA | | | intergenic |
| ENSMUSG00000118786 | Gm26179 | M_musculus | Animals | mono_intergenic | H/ACA | | | intergenic |
| ENSMUSG00000118787 | Gm26392 | M_musculus | Animals | mono_intergenic | C/D | ENSMUSG00000100826 | Snhg14 | non_coding |
| ENSMUSG00000118788 | Gm25194 | M_musculus | Animals | mono_intergenic | C/D | ENSMUSG00000100826 | Snhg14 | non_coding |
| ENSMUSG00000118792 | Gm25229 | M_musculus | Animals | mono_intergenic | C/D | ENSMUSG00000100826 | Snhg14 | non_coding |

| | | | | | | | | |
|---|---|---|---|---|---|---|---|---|
| ENSMUSG00000118810 | Gm24952 | M_musculus | Animals | mono_intergenic | C/D | ENSMUSG00000100826 | Snhg14 | non_coding |
| ENSMUSG00000118820 | Gm25065 | M_musculus | Animals | mono_intergenic | H/ACA | | | intergenic |
| ENSMUSG00000118828 | Gm26488 | M_musculus | Animals | mono_intergenic | C/D | ENSMUSG00000100826 | Snhg14 | non_coding |
| ENSMUSG00000118832 | Gm26374 | M_musculus | Animals | mono_intergenic | C/D | ENSMUSG00000100826 | Snhg14 | non_coding |
| ENSMUSG00000118834 | Gm24969 | M_musculus | Animals | mono_intergenic | C/D | ENSMUSG00000100826 | Snhg14 | non_coding |
| ENSMUSG00000118836 | Gm23471 | M_musculus | Animals | mono_intergenic | C/D | ENSMUSG00000100826 | Snhg14 | non_coding |
| ENSMUSG00000118846 | Gm25934 | M_musculus | Animals | mono_intergenic | C/D | ENSMUSG00000100826 | Snhg14 | non_coding |
| ENSMUSG00000118852 | Gm23604 | M_musculus | Animals | intronic_cluster | H/ACA | ENSMUSG00000084954 | Gm16351 | non_coding |
| ENSMUSG00000118856 | Gm23265 | M_musculus | Animals | intronic_cluster | C/D | ENSMUSG00000100826 | Snhg14 | non_coding |
| ENSMUSG00000118861 | Gm25840 | M_musculus | Animals | mono_intergenic | C/D | ENSMUSG00000100826 | Snhg14 | non_coding |
| ENSMUSG00000118873 | Gm22627 | M_musculus | Animals | mono_intergenic | C/D | ENSMUSG00000100826 | Snhg14 | non_coding |
| ENSMUSG00000118877 | Gm23449 | M_musculus | Animals | mono_intergenic | C/D | ENSMUSG00000100826 | Snhg14 | non_coding |
| ENSMUSG00000118878 | Gm22348 | M_musculus | Animals | mono_intronic | C/D | ENSMUSG00000100826 | Snhg14 | non_coding |
| ENSMUSG00000118879 | Gm26246 | M_musculus | Animals | intronic_cluster | C/D | ENSMUSG00000100826 | Snhg14 | non_coding |
| ENSMUSG00000118886 | Gm22834 | M_musculus | Animals | mono_intergenic | C/D | ENSMUSG00000100826 | Snhg14 | non_coding |
| ENSMUSG00000118888 | Gm25209 | M_musculus | Animals | mono_intergenic | C/D | ENSMUSG00000100826 | Snhg14 | non_coding |
| ENSMUSG00000118899 | Gm26504 | M_musculus | Animals | mono_intronic | C/D | ENSMUSG00000100826 | Snhg14 | non_coding |
| ENSMUSG00000118901 | Gm25564 | M_musculus | Animals | mono_intergenic | H/ACA | | | intergenic |
| ENSMUSG00000118902 | Gm24310 | M_musculus | Animals | mono_intergenic | C/D | ENSMUSG00000100826 | Snhg14 | non_coding |
| ENSMUSG00000118903 | Gm23139 | M_musculus | Animals | mono_intergenic | H/ACA | | | intergenic |
| ENSMUSG00000118905 | Snord93 | M_musculus | Animals | mono_intronic | C/D | ENSMUSG00000097180 | 2700038G22Rik | non_coding |
| ENSMUSG00000118911 | Gm22629 | M_musculus | Animals | mono_intergenic | C/D | ENSMUSG00000100826 | Snhg14 | non_coding |
| ENSMUSG00000118916 | Gm25474 | M_musculus | Animals | intronic_cluster | C/D | ENSMUSG00000100826 | Snhg14 | non_coding |
| ENSMUSG00000118924 | Gm23660 | M_musculus | Animals | mono_intronic | C/D | ENSMUSG00000100826 | Snhg14 | non_coding |
| ENSMUSG00000118925 | Gm22115 | M_musculus | Animals | mono_intergenic | H/ACA | | | intergenic |
| ENSMUSG00000118926 | Gm22285 | M_musculus | Animals | intronic_cluster | C/D | ENSMUSG00000100826 | Snhg14 | non_coding |
| ENSMUSG00000118933 | Gm25314 | M_musculus | Animals | mono_intergenic | C/D | ENSMUSG00000100826 | Snhg14 | non_coding |
| ENSMUSG00000118934 | Gm23145 | M_musculus | Animals | mono_intergenic | C/D | ENSMUSG00000100826 | Snhg14 | non_coding |
| ENSMUSG00000118941 | Snord116l12 | M_musculus | Animals | mono_intergenic | C/D | ENSMUSG00000100826 | Snhg14 | non_coding |
| ENSMUSG00000118943 | Gm25528 | M_musculus | Animals | mono_intergenic | H/ACA | | | intergenic |
| ENSMUSG00000118946 | Gm24982 | M_musculus | Animals | mono_intergenic | H/ACA | | | intergenic |
| ENSMUSG00000118955 | Gm22912 | M_musculus | Animals | mono_intergenic | C/D | ENSMUSG00000100826 | Snhg14 | non_coding |
| ENSMUSG00000118957 | Gm22006 | M_musculus | Animals | mono_intergenic | H/ACA | | | intergenic |
| ENSMUSG00000118960 | Gm22269 | M_musculus | Animals | mono_intergenic | H/ACA | | | intergenic |
| ENSMUSG00000118963 | Gm23862 | M_musculus | Animals | mono_intronic | C/D | ENSMUSG00000100826 | Snhg14 | non_coding |
| ENSMUSG00000118965 | Gm24872 | M_musculus | Animals | mono_intergenic | C/D | ENSMUSG00000100826 | Snhg14 | non_coding |
| ENSMUSG00000118969 | Gm22722 | M_musculus | Animals | mono_intergenic | H/ACA | | | intergenic |
| ENSMUSG00000118975 | Gm23141 | M_musculus | Animals | intronic_cluster | C/D | ENSMUSG00000100826 | Snhg14 | non_coding |
| ENSMUSG00000118976 | Gm23286 | M_musculus | Animals | mono_intergenic | C/D | ENSMUSG00000100826 | Snhg14 | non_coding |
| ENSMUSG00000118978 | Gm23089 | M_musculus | Animals | intronic_cluster | C/D | ENSMUSG00000100826 | Snhg14 | non_coding |
| ENSMUSG00000118981 | Gm22128 | M_musculus | Animals | intronic_cluster | C/D | ENSMUSG00000100826 | Snhg14 | non_coding |

| | | | | | | | | |
|---|---|---|---|---|---|---|---|---|
| ENSMUSG00000118983 | Gm24760 | M_musculus | Animals | intronic_cluster | C/D | ENSMUSG00000100826 | Snhg14 | non_coding |
| ENSMUSG00000118984 | Snord116l17 | M_musculus | Animals | intronic_cluster | C/D | ENSMUSG00000100826 | Snhg14 | non_coding |
| ENSMUSG00000118996 | Gm26332 | M_musculus | Animals | intronic_cluster | C/D | ENSMUSG00000100826 | Snhg14 | non_coding |
| ENSMUSG00000118997 | Gm23767 | M_musculus | Animals | intronic_cluster | C/D | ENSMUSG00000100826 | Snhg14 | non_coding |
| ENSMUSG00000118999 | Gm24986 | M_musculus | Animals | mono_intergenic | H/ACA | | | intergenic |
| ENSMUSG00000119001 | Gm25546 | M_musculus | Animals | mono_intergenic | H/ACA | | | intergenic |
| ENSMUSG00000119005 | Gm26367 | M_musculus | Animals | mono_intergenic | H/ACA | | | intergenic |
| ENSMUSG00000119010 | Gm22347 | M_musculus | Animals | mono_intergenic | H/ACA | | | intergenic |
| ENSMUSG00000119011 | Gm25291 | M_musculus | Animals | mono_intronic | H/ACA | ENSMUSG00000036391 | Sec24a | protein_coding |
| ENSMUSG00000119020 | Gm24570 | M_musculus | Animals | mono_intergenic | C/D | ENSMUSG00000100826 | Snhg14 | non_coding |
| ENSMUSG00000119029 | Gm25617 | M_musculus | Animals | intronic_cluster | H/ACA | ENSMUSG00000022812 | Gsk3b | protein_coding |
| ENSMUSG00000119047 | Gm22776 | M_musculus | Animals | intronic_cluster | C/D | ENSMUSG00000100826 | Snhg14 | non_coding |
| ENSMUSG00000119048 | Gm24653 | M_musculus | Animals | mono_intergenic | C/D | ENSMUSG00000100826 | Snhg14 | non_coding |
| ENSMUSG00000119052 | Gm23513 | M_musculus | Animals | mono_intergenic | H/ACA | | | intergenic |
| ENSMUSG00000119055 | Gm22851 | M_musculus | Animals | intronic_cluster | C/D | ENSMUSG00000100826 | Snhg14 | non_coding |
| ENSMUSG00000119057 | Gm24206 | M_musculus | Animals | mono_intergenic | C/D | ENSMUSG00000100826 | Snhg14 | non_coding |
| ENSMUSG00000119065 | Gm25387 | M_musculus | Animals | mono_intergenic | H/ACA | | | intergenic |
| ENSMUSG00000119068 | Gm24039 | M_musculus | Animals | mono_intergenic | H/ACA | | | intergenic |
| ENSMUSG00000119072 | Gm25230 | M_musculus | Animals | mono_intergenic | C/D | ENSMUSG00000100826 | Snhg14 | non_coding |
| ENSMUSG00000119073 | Gm25175 | M_musculus | Animals | mono_intergenic | C/D | ENSMUSG00000100826 | Snhg14 | non_coding |
| ENSMUSG00000119079 | Gm24949 | M_musculus | Animals | mono_intergenic | C/D | | | intergenic |
| ENSMUSG00000119080 | Gm26389 | M_musculus | Animals | mono_intergenic | C/D | ENSMUSG00000100826 | Snhg14 | non_coding |
| ENSMUSG00000119087 | Gm25311 | M_musculus | Animals | mono_intergenic | H/ACA | | | intergenic |
| ENSMUSG00000119088 | Gm24652 | M_musculus | Animals | mono_intergenic | C/D | ENSMUSG00000100826 | Snhg14 | non_coding |
| ENSMUSG00000119090 | Gm26272 | M_musculus | Animals | mono_intergenic | H/ACA | | | intergenic |
| ENSMUSG00000119091 | Gm22258 | M_musculus | Animals | intronic_cluster | C/D | ENSMUSG00000100826 | Snhg14 | non_coding |
| ENSMUSG00000119093 | Gm25090 | M_musculus | Animals | mono_intergenic | H/ACA | | | intergenic |
| ENSMUSG00000119095 | Gm22047 | M_musculus | Animals | intronic_cluster | C/D | ENSMUSG00000100826 | Snhg14 | non_coding |
| ENSMUSG00000119097 | Gm23687 | M_musculus | Animals | mono_intergenic | C/D | ENSMUSG00000100826 | Snhg14 | non_coding |
| ENSMUSG00000119100 | Gm22833 | M_musculus | Animals | mono_intergenic | H/ACA | | | intergenic |
| ENSMUSG00000119101 | Gm25452 | M_musculus | Animals | mono_intergenic | C/D | ENSMUSG00000100826 | Snhg14 | non_coding |
| ENSMUSG00000119104 | Gm25632 | M_musculus | Animals | mono_intronic | C/D | ENSMUSG00000039262 | Prrc2b | protein_coding |
| ENSMUSG00000119105 | Gm26366 | M_musculus | Animals | mono_intergenic | C/D | ENSMUSG00000100826 | Snhg14 | non_coding |
| ENSMUSG00000119112 | Gm24350 | M_musculus | Animals | intergenic_cluster | H/ACA | | | intergenic |
| ENSMUSG00000119119 | Snord116l11 | M_musculus | Animals | intronic_cluster | C/D | ENSMUSG00000100826 | Snhg14 | non_coding |
| ENSMUSG00000119121 | Gm25120 | M_musculus | Animals | mono_intergenic | C/D | ENSMUSG00000100826 | Snhg14 | non_coding |
| ENSMUSG00000119126 | Gm26466 | M_musculus | Animals | mono_intronic | C/D | ENSMUSG00000100826 | Snhg14 | non_coding |
| ENSMUSG00000119128 | Gm23831 | M_musculus | Animals | mono_intergenic | H/ACA | | | intergenic |
| ENSMUSG00000119130 | Gm23313 | M_musculus | Animals | intronic_cluster | C/D | ENSMUSG00000100826 | Snhg14 | non_coding |
| ENSMUSG00000119133 | Gm23266 | M_musculus | Animals | mono_intergenic | H/ACA | | | intergenic |
| ENSMUSG00000119143 | Snord116l7 | M_musculus | Animals | intronic_cluster | C/D | ENSMUSG00000100826 | Snhg14 | non_coding |

| | | | | | | | | |
|---|---|---|---|---|---|---|---|---|
| ENSMUSG00000119144 | Gm23711 | M_musculus | Animals | mono_intronic | C/D | ENSMUSG00000028580 | Pum1 | protein_coding |
| ENSMUSG00000119148 | Gm25870 | M_musculus | Animals | mono_intergenic | C/D | ENSMUSG00000100826 | Snhg14 | non_coding |
| ENSMUSG00000119150 | Gm22599 | M_musculus | Animals | mono_intronic | H/ACA | ENSMUSG00000063549 | Or2w3 | protein_coding |
| ENSMUSG00000119157 | Gm22812 | M_musculus | Animals | intronic_cluster | C/D | ENSMUSG00000100826 | Snhg14 | non_coding |
| ENSMUSG00000119160 | Gm24639 | M_musculus | Animals | mono_intergenic | C/D | ENSMUSG00000100826 | Snhg14 | non_coding |
| ENSMUSG00000119162 | Gm25989 | M_musculus | Animals | mono_intronic | H/ACA | ENSMUSG00000022704 | Qtrt2 | protein_coding |
| ENSMUSG00000119163 | Gm24539 | M_musculus | Animals | intronic_cluster | H/ACA | ENSMUSG00000074867 | Zfp808 | protein_coding |
| ENSMUSG00000119166 | Gm23568 | M_musculus | Animals | mono_intronic | H/ACA | ENSMUSG00000079114 | Defa42 | protein_coding |
| ENSMUSG00000119170 | Snord116l16 | M_musculus | Animals | intronic_cluster | C/D | ENSMUSG00000100826 | Snhg14 | non_coding |
| ENSMUSG00000119172 | Gm22723 | M_musculus | Animals | mono_intergenic | H/ACA | | | intergenic |
| ENSMUSG00000119173 | Gm26201 | M_musculus | Animals | intronic_cluster | C/D | ENSMUSG00000100826 | Snhg14 | non_coding |
| ENSMUSG00000119179 | Gm22910 | M_musculus | Animals | mono_intergenic | H/ACA | | | intergenic |
| ENSMUSG00000119186 | Snord116l6 | M_musculus | Animals | intronic_cluster | C/D | ENSMUSG00000100826 | Snhg14 | non_coding |
| ENSMUSG00000119191 | Gm25526 | M_musculus | Animals | mono_intergenic | H/ACA | | | intergenic |
| ENSMUSG00000119192 | Gm22900 | M_musculus | Animals | mono_intergenic | H/ACA | | | intergenic |
| ENSMUSG00000119196 | Gm23816 | M_musculus | Animals | mono_intergenic | H/ACA | | | intergenic |
| ENSMUSG00000119197 | Gm26433 | M_musculus | Animals | intronic_cluster | C/D | ENSMUSG00000100826 | Snhg14 | non_coding |
| ENSMUSG00000119201 | Gm24153 | M_musculus | Animals | intronic_cluster | C/D | ENSMUSG00000100826 | Snhg14 | non_coding |
| ENSMUSG00000119215 | Gm22517 | M_musculus | Animals | mono_intergenic | H/ACA | | | intergenic |
| ENSMUSG00000119219 | Gm25615 | M_musculus | Animals | intronic_cluster | C/D | ENSMUSG00000100826 | Snhg14 | non_coding |
| ENSMUSG00000119221 | Gm25936 | M_musculus | Animals | mono_intergenic | H/ACA | | | intergenic |
| ENSMUSG00000119224 | Gm22996 | M_musculus | Animals | mono_intergenic | C/D | ENSMUSG00000100826 | Snhg14 | non_coding |
| ENSMUSG00000119225 | Gm26284 | M_musculus | Animals | mono_intergenic | C/D | ENSMUSG00000100826 | Snhg14 | non_coding |
| ENSMUSG00000119226 | Gm26421 | M_musculus | Animals | mono_intergenic | H/ACA | | | intergenic |
| ENSMUSG00000119234 | Gm22941 | M_musculus | Animals | intronic_cluster | C/D | ENSMUSG00000100826 | Snhg14 | non_coding |
| ENSMUSG00000119238 | Gm26230 | M_musculus | Animals | mono_intergenic | C/D | ENSMUSG00000100826 | Snhg14 | non_coding |
| ENSMUSG00000119239 | Gm22417 | M_musculus | Animals | intronic_cluster | C/D | ENSMUSG00000100826 | Snhg14 | non_coding |
| ENSMUSG00000119244 | Gm26334 | M_musculus | Animals | mono_intergenic | C/D | ENSMUSG00000100826 | Snhg14 | non_coding |
| ENSMUSG00000119250 | Gm24759 | M_musculus | Animals | mono_intergenic | C/D | ENSMUSG00000100826 | Snhg14 | non_coding |
| ENSMUSG00000119254 | Snord116l15 | M_musculus | Animals | intronic_cluster | C/D | ENSMUSG00000100826 | Snhg14 | non_coding |
| ENSMUSG00000119256 | Gm26344 | M_musculus | Animals | mono_intergenic | H/ACA | | | intergenic |
| ENSMUSG00000119258 | Gm26096 | M_musculus | Animals | mono_intergenic | C/D | ENSMUSG00000100826 | Snhg14 | non_coding |
| ENSMUSG00000119262 | Snord3b1 | M_musculus | Animals | intronic_cluster | C/D | ENSMUSG00000010342 | Tex14 | protein_coding |
| ENSMUSG00000119268 | Gm25232 | M_musculus | Animals | mono_intergenic | H/ACA | | | intergenic |
| ENSMUSG00000119269 | Gm25816 | M_musculus | Animals | intronic_cluster | C/D | ENSMUSG00000100826 | Snhg14 | non_coding |
| ENSMUSG00000119270 | Gm24314 | M_musculus | Animals | mono_intergenic | H/ACA | | | intergenic |
| ENSMUSG00000119271 | Gm25742 | M_musculus | Animals | mono_intergenic | C/D | ENSMUSG00000100826 | Snhg14 | non_coding |
| ENSMUSG00000119274 | Gm26297 | M_musculus | Animals | mono_intergenic | H/ACA | | | intergenic |
| ENSMUSG00000119279 | Gm23575 | M_musculus | Animals | mono_intergenic | C/D | ENSMUSG00000100826 | Snhg14 | non_coding |
| ENSMUSG00000119282 | Snord116l3 | M_musculus | Animals | intronic_cluster | C/D | ENSMUSG00000100826 | Snhg14 | non_coding |
| ENSMUSG00000119285 | Gm22111 | M_musculus | Animals | mono_intronic | C/D | ENSMUSG00000100826 | Snhg14 | non_coding |

| | | | | | | | | |
|---|---|---|---|---|---|---|---|---|
| ENSMUSG00000119291 | Gm24957 | M_musculus | Animals | mono_intergenic | H/ACA | | | intergenic |
| ENSMUSG00000119294 | Gm22393 | M_musculus | Animals | mono_intergenic | C/D | ENSMUSG00000100826 | Snhg14 | non_coding |
| ENSMUSG00000119295 | Gm26059 | M_musculus | Animals | mono_intergenic | C/D | ENSMUSG00000100826 | Snhg14 | non_coding |
| ENSMUSG00000119296 | Gm22252 | M_musculus | Animals | mono_intergenic | C/D | ENSMUSG00000100826 | Snhg14 | non_coding |
| ENSMUSG00000119298 | Gm22524 | M_musculus | Animals | mono_intergenic | C/D | ENSMUSG00000100826 | Snhg14 | non_coding |
| ENSMUSG00000119311 | Gm25988 | M_musculus | Animals | mono_intergenic | C/D | ENSMUSG00000100826 | Snhg14 | non_coding |
| ENSMUSG00000119316 | Gm26336 | M_musculus | Animals | mono_intergenic | C/D | ENSMUSG00000100826 | Snhg14 | non_coding |
| ENSMUSG00000119320 | Snora70 | M_musculus | Animals | mono_intronic | H/ACA | ENSMUSG00000008682 | Rpl10 | protein_coding |
| ENSMUSG00000119322 | Gm24654 | M_musculus | Animals | mono_intergenic | C/D | ENSMUSG00000100826 | Snhg14 | non_coding |
| ENSMUSG00000119326 | Snord116l9 | M_musculus | Animals | intronic_cluster | C/D | ENSMUSG00000100826 | Snhg14 | non_coding |
| ENSMUSG00000119327 | Gm25155 | M_musculus | Animals | intronic_cluster | C/D | ENSMUSG00000100826 | Snhg14 | non_coding |
| ENSMUSG00000119329 | Gm26494 | M_musculus | Animals | mono_intergenic | H/ACA | | | intergenic |
| ENSMUSG00000119331 | Gm24219 | M_musculus | Animals | mono_intergenic | C/D | ENSMUSG00000100826 | Snhg14 | non_coding |
| ENSMUSG00000119336 | Gm22255 | M_musculus | Animals | mono_intergenic | C/D | ENSMUSG00000100826 | Snhg14 | non_coding |
| ENSMUSG00000119347 | Gm25210 | M_musculus | Animals | intronic_cluster | C/D | ENSMUSG00000100826 | Snhg14 | non_coding |
| ENSMUSG00000119350 | Gm23618 | M_musculus | Animals | mono_intergenic | C/D | ENSMUSG00000100826 | Snhg14 | non_coding |
| ENSMUSG00000119351 | Gm24799 | M_musculus | Animals | mono_intronic | C/D | ENSMUSG00000100826 | Snhg14 | non_coding |
| ENSMUSG00000119352 | Snord116l2 | M_musculus | Animals | intronic_cluster | C/D | ENSMUSG00000100826 | Snhg14 | non_coding |
| ENSMUSG00000119355 | Gm23311 | M_musculus | Animals | mono_intergenic | H/ACA | | | intergenic |
| ENSMUSG00000119357 | Gm26390 | M_musculus | Animals | mono_intergenic | C/D | ENSMUSG00000100826 | Snhg14 | non_coding |
| ENSMUSG00000119358 | Gm24868 | M_musculus | Animals | mono_intergenic | H/ACA | | | intergenic |
| ENSMUSG00000119359 | Gm22640 | M_musculus | Animals | mono_intergenic | C/D | ENSMUSG00000100826 | Snhg14 | non_coding |
| ENSMUSG00000119367 | Snord116l14 | M_musculus | Animals | intronic_cluster | C/D | ENSMUSG00000100826 | Snhg14 | non_coding |
| ENSMUSG00000119371 | Gm24618 | M_musculus | Animals | intronic_cluster | C/D | ENSMUSG00000100826 | Snhg14 | non_coding |
| ENSMUSG00000119373 | Gm23953 | M_musculus | Animals | intronic_cluster | C/D | ENSMUSG00000100826 | Snhg14 | non_coding |
| ENSMUSG00000119374 | Gm24264 | M_musculus | Animals | intronic_cluster | C/D | ENSMUSG00000100826 | Snhg14 | non_coding |
| ENSMUSG00000119375 | Gm23944 | M_musculus | Animals | mono_intergenic | C/D | ENSMUSG00000100826 | Snhg14 | non_coding |
| ENSMUSG00000119386 | Gm22511 | M_musculus | Animals | mono_intergenic | C/D | ENSMUSG00000100826 | Snhg14 | non_coding |
| ENSMUSG00000119387 | Gm24566 | M_musculus | Animals | mono_intergenic | C/D | ENSMUSG00000100826 | Snhg14 | non_coding |
| ENSMUSG00000119388 | Snord16a | M_musculus | Animals | mono_intronic | C/D | ENSMUSG00000032399 | Rpl4 | protein_coding |
| ENSMUSG00000119391 | Gm25727 | M_musculus | Animals | mono_intergenic | H/ACA | | | intergenic |
| ENSMUSG00000119394 | Gm25741 | M_musculus | Animals | mono_intergenic | C/D | ENSMUSG00000100826 | Snhg14 | non_coding |
| ENSMUSG00000119397 | Gm23310 | M_musculus | Animals | mono_intergenic | C/D | ENSMUSG00000100826 | Snhg14 | non_coding |
| ENSMUSG00000119405 | Gm25647 | M_musculus | Animals | mono_intergenic | C/D | ENSMUSG00000100826 | Snhg14 | non_coding |
| ENSMUSG00000119410 | Gm25461 | M_musculus | Animals | mono_intergenic | C/D | ENSMUSG00000100826 | Snhg14 | non_coding |
| ENSMUSG00000119412 | Gm25648 | M_musculus | Animals | mono_intergenic | C/D | ENSMUSG00000100826 | Snhg14 | non_coding |
| ENSMUSG00000119415 | Snord116l1 | M_musculus | Animals | intronic_cluster | C/D | ENSMUSG00000100826 | Snhg14 | non_coding |
| ENSMUSG00000119417 | Gm24599 | M_musculus | Animals | mono_intergenic | H/ACA | | | intergenic |
| ENSMUSG00000119428 | Gm22253 | M_musculus | Animals | mono_intergenic | C/D | ENSMUSG00000100826 | Snhg14 | non_coding |
| ENSMUSG00000119431 | Gm57326 | M_musculus | Animals | intergenic_cluster | H/ACA | | | intergenic |
| ENSMUSG00000119432 | Snord50b | M_musculus | Animals | mono_intronic | C/D | ENSMUSG00000097195 | Snhg5 | non_coding |

| | | | | | | | | |
|---|---|---|---|---|---|---|---|---|
| ENSMUSG00000119444 | Gm26337 | M_musculus | Animals | mono_intergenic | C/D | ENSMUSG00000100826 | Snhg14 | non_coding |
| ENSMUSG00000119445 | Gm23440 | M_musculus | Animals | mono_intergenic | H/ACA | | | intergenic |
| ENSMUSG00000119446 | Gm25121 | M_musculus | Animals | mono_intergenic | C/D | ENSMUSG00000100826 | Snhg14 | non_coding |
| ENSMUSG00000119463 | Gm25017 | M_musculus | Animals | mono_intergenic | C/D | ENSMUSG00000100826 | Snhg14 | non_coding |
| ENSMUSG00000119464 | Gm22447 | M_musculus | Animals | mono_intronic | H/ACA | ENSMUSG00000058618 | Defa39 | protein_coding |
| ENSMUSG00000119465 | Gm25449 | M_musculus | Animals | mono_intergenic | C/D | ENSMUSG00000100826 | Snhg14 | non_coding |
| ENSMUSG00000119468 | Gm24519 | M_musculus | Animals | mono_intergenic | H/ACA | | | intergenic |
| ENSMUSG00000119475 | Gm25823 | M_musculus | Animals | mono_intergenic | C/D | ENSMUSG00000100826 | Snhg14 | non_coding |
| ENSMUSG00000119477 | Gm23682 | M_musculus | Animals | mono_intergenic | C/D | ENSMUSG00000100826 | Snhg14 | non_coding |
| ENSMUSG00000119485 | Gm23017 | M_musculus | Animals | mono_intronic | C/D | ENSMUSG00000097073 | 9430037G07Rik | non_coding |
| ENSMUSG00000119486 | Gm26422 | M_musculus | Animals | mono_intergenic | H/ACA | | | intergenic |
| ENSMUSG00000119487 | Gm24709 | M_musculus | Animals | intronic_cluster | H/ACA | ENSMUSG00000004347 | Pde1c | protein_coding |
| ENSMUSG00000119490 | Gm22046 | M_musculus | Animals | intronic_cluster | C/D | ENSMUSG00000100826 | Snhg14 | non_coding |
| ENSMUSG00000119491 | Gm26135 | M_musculus | Animals | mono_intronic | C/D | ENSMUSG00000097177 | 9330159M07Rik | non_coding |
| ENSMUSG00000119496 | Gm24088 | M_musculus | Animals | mono_intronic | C/D | ENSMUSG00000100826 | Snhg14 | non_coding |
| ENSMUSG00000119497 | Gm26032 | M_musculus | Animals | intronic_cluster | C/D | ENSMUSG00000100826 | Snhg14 | non_coding |
| ENSMUSG00000119504 | Gm25074 | M_musculus | Animals | intronic_cluster | C/D | ENSMUSG00000100826 | Snhg14 | non_coding |
| ENSMUSG00000119505 | Gm22391 | M_musculus | Animals | mono_intergenic | C/D | ENSMUSG00000100826 | Snhg14 | non_coding |
| ENSMUSG00000119506 | Gm25874 | M_musculus | Animals | mono_intronic | C/D | ENSMUSG00000100826 | Snhg14 | non_coding |
| ENSMUSG00000119513 | Gm23561 | M_musculus | Animals | mono_intronic | H/ACA | ENSMUSG00000074437 | Defa29 | protein_coding |
| ENSMUSG00000119525 | Gm23261 | M_musculus | Animals | mono_intergenic | H/ACA | | | intergenic |
| ENSMUSG00000119528 | Gm24495 | M_musculus | Animals | mono_intergenic | C/D | ENSMUSG00000100826 | Snhg14 | non_coding |
| ENSMUSG00000119530 | Gm25085 | M_musculus | Animals | mono_intergenic | C/D | ENSMUSG00000100826 | Snhg14 | non_coding |
| ENSMUSG00000119532 | Gm24493 | M_musculus | Animals | mono_intergenic | H/ACA | | | intergenic |
| ENSMUSG00000119539 | Gm22050 | M_musculus | Animals | mono_intergenic | C/D | ENSMUSG00000100826 | Snhg14 | non_coding |
| ENSMUSG00000119542 | Gm25804 | M_musculus | Animals | mono_intronic | H/ACA | ENSMUSG00000110123 | Gm18562 | non_coding |
| ENSMUSG00000119548 | Gm26288 | M_musculus | Animals | mono_intergenic | H/ACA | | | intergenic |
| ENSMUSG00000119550 | Gm25156 | M_musculus | Animals | mono_intergenic | C/D | ENSMUSG00000100826 | Snhg14 | non_coding |
| ENSMUSG00000119553 | Gm22631 | M_musculus | Animals | intronic_cluster | C/D | ENSMUSG00000100826 | Snhg14 | non_coding |
| ENSMUSG00000119560 | Gm24418 | M_musculus | Animals | mono_intergenic | C/D | ENSMUSG00000100826 | Snhg14 | non_coding |
| ENSMUSG00000119568 | Gm26365 | M_musculus | Animals | intronic_cluster | C/D | ENSMUSG00000100826 | Snhg14 | non_coding |
| ENSMUSG00000119569 | Gm22233 | M_musculus | Animals | mono_intergenic | H/ACA | | | intergenic |
| ENSMUSG00000119570 | Gm25944 | M_musculus | Animals | intronic_cluster | C/D | ENSMUSG00000100826 | Snhg14 | non_coding |
| ENSMUSG00000119573 | Gm23529 | M_musculus | Animals | mono_intergenic | H/ACA | | | intergenic |
| ENSMUSG00000119576 | Gm24702 | M_musculus | Animals | mono_intergenic | C/D | ENSMUSG00000100826 | Snhg14 | non_coding |
| ENSMUSG00000119577 | Gm24727 | M_musculus | Animals | mono_intronic | H/ACA | ENSMUSG00000031584 | Gsr | protein_coding |
| ENSMUSG00000119578 | Gm26283 | M_musculus | Animals | mono_intergenic | C/D | ENSMUSG00000100826 | Snhg14 | non_coding |
| ENSMUSG00000119588 | Gm25891 | M_musculus | Animals | mono_intergenic | H/ACA | | | intergenic |
| ENSMUSG00000119589 | Gm24095 | M_musculus | Animals | intronic_cluster | H/ACA | ENSMUSG00000055228 | Gm49359 | protein_coding |
| ENSMUSG00000119603 | Gm26270 | M_musculus | Animals | intronic_cluster | C/D | ENSMUSG00000100826 | Snhg14 | non_coding |
| ENSMUSG00000119606 | Gm25221 | M_musculus | Animals | mono_intergenic | C/D | ENSMUSG00000100826 | Snhg14 | non_coding |

| | | | | | | | | |
|---|---|---|---|---|---|---|---|---|
| ENSMUSG00000119610 | Gm24656 | M_musculus | Animals | mono_intronic | H/ACA | ENSMUSG00000040420 | Cdh18 | protein_coding |
| ENSMUSG00000119611 | Gm24706 | M_musculus | Animals | mono_intergenic | H/ACA | | | intergenic |
| ENSMUSG00000119612 | Gm23948 | M_musculus | Animals | mono_intergenic | H/ACA | | | intergenic |
| ENSMUSG00000119617 | Gm25984 | M_musculus | Animals | mono_intergenic | C/D | ENSMUSG00000100826 | Snhg14 | non_coding |
| ENSMUSG00000119619 | Gm24199 | M_musculus | Animals | mono_intergenic | H/ACA | | | intergenic |
| ENSMUSG00000119625 | Gm22130 | M_musculus | Animals | mono_intergenic | C/D | ENSMUSG00000100826 | Snhg14 | non_coding |
| ENSMUSG00000119635 | Gm25497 | M_musculus | Animals | mono_intergenic | H/ACA | | | intergenic |
| ENSMUSG00000119637 | Snord116l8 | M_musculus | Animals | intronic_cluster | C/D | ENSMUSG00000100826 | Snhg14 | non_coding |
| ENSMUSG00000119643 | Snord88a | M_musculus | Animals | mono_intronic | C/D | ENSMUSG00000045411 | 2410002F23Rik | protein_coding |
| ENSMUSG00000119647 | Gm25087 | M_musculus | Animals | mono_intergenic | C/D | ENSMUSG00000100826 | Snhg14 | non_coding |
| ENSMUSG00000119648 | Snord3b3 | M_musculus | Animals | intronic_cluster | C/D | ENSMUSG00000010342 | Tex14 | protein_coding |
| ENSMUSG00000119649 | Gm24091 | M_musculus | Animals | mono_intronic | C/D | ENSMUSG00000028580 | Pum1 | protein_coding |
| ENSMUSG00000119662 | Gm22988 | M_musculus | Animals | mono_intronic | H/ACA | ENSMUSG00000059796 | Eif4a1 | protein_coding |
| ENSMUSG00000119665 | Gm22632 | M_musculus | Animals | mono_intergenic | C/D | ENSMUSG00000100826 | Snhg14 | non_coding |
| ENSMUSG00000119666 | Snord50a | M_musculus | Animals | mono_intronic | C/D | ENSMUSG00000097195 | Snhg5 | non_coding |
| ENSMUSG00000119682 | Gm22909 | M_musculus | Animals | mono_intergenic | C/D | ENSMUSG00000100826 | Snhg14 | non_coding |
| ENSMUSG00000119687 | Gm24657 | M_musculus | Animals | mono_intergenic | C/D | ENSMUSG00000100826 | Snhg14 | non_coding |
| ENSMUSG00000119688 | Gm22192 | M_musculus | Animals | mono_intronic | C/D | ENSMUSG00000039262 | Prrc2b | protein_coding |
| ENSMUSG00000119689 | Gm23446 | M_musculus | Animals | intronic_cluster | C/D | ENSMUSG00000100826 | Snhg14 | non_coding |
| ENSMUSG00000119694 | Gm23560 | M_musculus | Animals | mono_intergenic | C/D | ENSMUSG00000100826 | Snhg14 | non_coding |
| ENSMUSG00000119696 | Gm24609 | M_musculus | Animals | intronic_cluster | C/D | ENSMUSG00000100826 | Snhg14 | non_coding |
| ENSMUSG00000119700 | Snord116l4 | M_musculus | Animals | intronic_cluster | C/D | ENSMUSG00000100826 | Snhg14 | non_coding |
| ENSMUSG00000119705 | Gm23933 | M_musculus | Animals | mono_intronic | C/D | ENSMUSG00000100826 | Snhg14 | non_coding |
| ENSMUSG00000119710 | Gm25710 | M_musculus | Animals | mono_intergenic | C/D | ENSMUSG00000100826 | Snhg14 | non_coding |
| ENSMUSG00000119713 | Gm24638 | M_musculus | Animals | mono_intergenic | H/ACA | | | intergenic |
| ENSMUSG00000119722 | Snord116l5 | M_musculus | Animals | intronic_cluster | C/D | ENSMUSG00000100826 | Snhg14 | non_coding |
| ENSMUSG00000119724 | Gm23688 | M_musculus | Animals | mono_intergenic | C/D | ENSMUSG00000100826 | Snhg14 | non_coding |
| ENSMUSG00000119726 | Gm26499 | M_musculus | Animals | mono_intergenic | C/D | ENSMUSG00000100826 | Snhg14 | non_coding |
| ENSMUSG00000119733 | Gm25076 | M_musculus | Animals | mono_intergenic | H/ACA | | | intergenic |
| ENSMUSG00000119737 | Gm23923 | M_musculus | Animals | mono_intergenic | H/ACA | | | intergenic |
| ENSMUSG00000119739 | Gm23922 | M_musculus | Animals | mono_intergenic | C/D | ENSMUSG00000100826 | Snhg14 | non_coding |
| ENSMUSG00000119740 | Gm24100 | M_musculus | Animals | mono_intergenic | C/D | ENSMUSG00000100826 | Snhg14 | non_coding |
| ENSMUSG00000119746 | Gm24417 | M_musculus | Animals | mono_intergenic | C/D | ENSMUSG00000100826 | Snhg14 | non_coding |
| ENSMUSG00000119750 | Gm26498 | M_musculus | Animals | mono_intronic | C/D | ENSMUSG00000100826 | Snhg14 | non_coding |
| ENSMUSG00000119751 | Gm22510 | M_musculus | Animals | mono_intergenic | C/D | ENSMUSG00000100826 | Snhg14 | non_coding |
| ENSMUSG00000119753 | Gm25077 | M_musculus | Animals | mono_intergenic | C/D | ENSMUSG00000100826 | Snhg14 | non_coding |
| ENSMUSG00000119761 | Snord3b2 | M_musculus | Animals | intronic_cluster | C/D | ENSMUSG00000010342 | Tex14 | protein_coding |
| ENSMUSG00000119763 | Gm24416 | M_musculus | Animals | mono_intergenic | H/ACA | | | intergenic |
| ENSMUSG00000119764 | Gm24518 | M_musculus | Animals | intronic_cluster | C/D | ENSMUSG00000100826 | Snhg14 | non_coding |
| ENSMUSG00000119765 | Gm23136 | M_musculus | Animals | mono_intronic | C/D | ENSMUSG00000032399 | Rpl4 | protein_coding |
| ENSMUSG00000119769 | Gm22863 | M_musculus | Animals | intronic_cluster | C/D | ENSMUSG00000100826 | Snhg14 | non_coding |

| | | | | | | | | |
|---|---|---|---|---|---|---|---|---|
| ENSMUSG00000119770 | Gm25483 | M_musculus | Animals | mono_intronic | H/ACA | ENSMUSG00000057069 | Ero1b | protein_coding |
| ENSMUSG00000119773 | Gm24220 | M_musculus | Animals | mono_intergenic | C/D | ENSMUSG00000100826 | Snhg14 | non_coding |
| ENSMUSG00000119777 | Gm25098 | M_musculus | Animals | mono_intergenic | C/D | ENSMUSG00000100826 | Snhg14 | non_coding |
| ENSMUSG00000119778 | Gm26432 | M_musculus | Animals | mono_intergenic | C/D | ENSMUSG00000100826 | Snhg14 | non_coding |
| ENSMUSG00000119779 | Gm23128 | M_musculus | Animals | intergenic_cluster | H/ACA | | | intergenic |
| ENSMUSG00000119783 | Gm24262 | M_musculus | Animals | mono_intergenic | H/ACA | | | intergenic |
| ENSMUSG00000119786 | Gm22630 | M_musculus | Animals | mono_intergenic | C/D | ENSMUSG00000100826 | Snhg14 | non_coding |
| ENSMUSG00000119800 | Gm24926 | M_musculus | Animals | mono_intergenic | C/D | ENSMUSG00000100826 | Snhg14 | non_coding |
| ENSMUSG00000119805 | Snord14c | M_musculus | Animals | mono_intronic | C/D | ENSMUSG00000015656 | Hspa8 | protein_coding |
| ENSMUSG00000119806 | Gm24077 | M_musculus | Animals | mono_intergenic | H/ACA | | | intergenic |
| ENSMUSG00000119816 | Gm25088 | M_musculus | Animals | mono_intronic | C/D | ENSMUSG00000100826 | Snhg14 | non_coding |
| ENSMUSG00000119817 | Gm24862 | M_musculus | Animals | mono_intronic | C/D | ENSMUSG00000100826 | Snhg14 | non_coding |
| ENSMUSG00000119819 | Gm24711 | M_musculus | Animals | intronic_cluster | C/D | ENSMUSG00000100826 | Snhg14 | non_coding |
| ENSMUSG00000119823 | Gm25471 | M_musculus | Animals | intronic_cluster | C/D | ENSMUSG00000100826 | Snhg14 | non_coding |
| ENSMUSG00000119827 | Gm23356 | M_musculus | Animals | mono_intergenic | C/D | ENSMUSG00000100826 | Snhg14 | non_coding |
| ENSMUSG00000119831 | Gm26200 | M_musculus | Animals | mono_intergenic | C/D | ENSMUSG00000100826 | Snhg14 | non_coding |
| ENSMUSG00000119841 | Gm23619 | M_musculus | Animals | intronic_cluster | C/D | ENSMUSG00000100826 | Snhg14 | non_coding |
| ENSMUSG00000119843 | Gm25646 | M_musculus | Animals | mono_intergenic | C/D | ENSMUSG00000100826 | Snhg14 | non_coding |
| ENSMUSG00000119846 | Gm26396 | M_musculus | Animals | mono_intergenic | H/ACA | | | intergenic |
| ENSMUSG00000119847 | Gm25523 | M_musculus | Animals | mono_intergenic | C/D | ENSMUSG00000100826 | Snhg14 | non_coding |
| ENSMUSG00000119851 | Snord3b4 | M_musculus | Animals | intronic_cluster | C/D | ENSMUSG00000010342 | Tex14 | protein_coding |
| ENSMUSG00000119855 | Gm24568 | M_musculus | Animals | mono_intronic | H/ACA | ENSMUSG00000061958 | Defa38 | protein_coding |
| ENSMUSG00000119857 | Gm25466 | M_musculus | Animals | mono_intergenic | H/ACA | | | intergenic |
| ENSMUSG00000119858 | Gm24079 | M_musculus | Animals | mono_intergenic | H/ACA | | | intergenic |
| ENSMUSG00000119860 | Gm23305 | M_musculus | Animals | mono_intergenic | C/D | ENSMUSG00000100826 | Snhg14 | non_coding |
| ENSMUSG00000119863 | Gm24528 | M_musculus | Animals | mono_intergenic | C/D | ENSMUSG00000100826 | Snhg14 | non_coding |
| ENSMUSG00000119864 | Gm25462 | M_musculus | Animals | mono_intergenic | C/D | ENSMUSG00000100826 | Snhg14 | non_coding |
| ENSMUSG00000119872 | Gm23175 | M_musculus | Animals | mono_intergenic | H/ACA | | | intergenic |
| ENSMUSG00000119878 | Gm24027 | M_musculus | Animals | mono_intergenic | C/D | ENSMUSG00000100826 | Snhg14 | non_coding |
| ENSMUSG00000119884 | Gm22374 | M_musculus | Animals | mono_intergenic | H/ACA | | | intergenic |
| ENSMUSG00000119885 | Gm23047 | M_musculus | Animals | intronic_cluster | C/D | ENSMUSG00000100826 | Snhg14 | non_coding |
| ENSMUSG00000119897 | Gm25585 | M_musculus | Animals | mono_intergenic | C/D | ENSMUSG00000100826 | Snhg14 | non_coding |
| ENSMUSG00000119901 | Gm22782 | M_musculus | Animals | mono_intergenic | H/ACA | | | intergenic |
| ENSMUSG00000119905 | Gm22449 | M_musculus | Animals | mono_intergenic | C/D | ENSMUSG00000100826 | Snhg14 | non_coding |
| ENSMUSG00000119913 | Gm25350 | M_musculus | Animals | intronic_cluster | C/D | ENSMUSG00000100826 | Snhg14 | non_coding |
| ENSMUSG00000119922 | Gm22131 | M_musculus | Animals | intronic_cluster | C/D | ENSMUSG00000100826 | Snhg14 | non_coding |
| ENSMUSG00000119927 | Gm24040 | M_musculus | Animals | mono_intergenic | C/D | ENSMUSG00000100826 | Snhg14 | non_coding |
| ENSMUSG00000119928 | Gm23910 | M_musculus | Animals | mono_intergenic | H/ACA | | | intergenic |
| ENSMUSG00000119929 | Gm25146 | M_musculus | Animals | mono_intergenic | C/D | ENSMUSG00000100826 | Snhg14 | non_coding |
| ENSMUSG00000119930 | Gm25066 | M_musculus | Animals | mono_intergenic | H/ACA | | | intergenic |
| ENSMUSG00000119932 | Gm25868 | M_musculus | Animals | mono_intergenic | H/ACA | | | intergenic |

| | | | | | | | | |
|---|---|---|---|---|---|---|---|---|
| ENSMUSG00000119937 | Gm25463 | M_musculus | Animals | mono_intergenic | C/D | ENSMUSG00000100826 | Snhg14 | non_coding |
| ENSMUSG00001118660 | Gm22622 | M_musculus | Animals | mono_intergenic | H/ACA | | | intergenic |
| ENSMUSG00001118671 | Gm24829 | M_musculus | Animals | mono_intronic | H/ACA | ENSMUSG00000079113 | Defa43 | protein_coding |
| ENSMUSG00002074855 | Gm26457 | M_musculus | Animals | intronic_cluster | Unknown | ENSMUSG00000025967 | Eef1b2 | protein_coding |
| ENSMUSG00002074875 | Snord3a | M_musculus | Animals | intergenic_cluster | C/D | | | intergenic |
| ENSMUSG00002074888 | Gm55112 | M_musculus | Animals | mono_intronic | Unknown | ENSMUSG00000097451 | Rian | non_coding |
| ENSMUSG00002074900 | Gm56414 | M_musculus | Animals | mono_intergenic | H/ACA | | | intergenic |
| ENSMUSG00002074943 | Gm56345 | M_musculus | Animals | mono_intronic | H/ACA | ENSMUSG00000014361 | Mertk | protein_coding |
| ENSMUSG00002074951 | Gm56076 | M_musculus | Animals | mono_intergenic | Unknown | | | intergenic |
| ENSMUSG00002074955 | Snord3b4 | M_musculus | Animals | intronic_cluster | C/D | ENSMUSG00000010342 | Tex14 | protein_coding |
| ENSMUSG00002074983 | Gm55799 | M_musculus | Animals | mono_intergenic | Unknown | | | intergenic |
| ENSMUSG00002074987 | Gm54625 | M_musculus | Animals | mono_intergenic | Unknown | | | intergenic |
| ENSMUSG00002075008 | Gm55190 | M_musculus | Animals | mono_intergenic | Unknown | | | intergenic |
| ENSMUSG00002075018 | Gm54544 | M_musculus | Animals | mono_intergenic | Unknown | | | intergenic |
| ENSMUSG00002075059 | Gm55104 | M_musculus | Animals | mono_intronic | Unknown | ENSMUSG00000106095 | 2010110G14Rik | non_coding |
| ENSMUSG00002075119 | Gm54688 | M_musculus | Animals | mono_intergenic | Unknown | | | intergenic |
| ENSMUSG00002075125 | Gm54606 | M_musculus | Animals | mono_intronic | Unknown | ENSMUSG00000024991 | Eif3a | protein_coding |
| ENSMUSG00002075132 | Gm55812 | M_musculus | Animals | mono_intronic | Unknown | ENSMUSG00000004383 | Large1 | protein_coding |
| ENSMUSG00002075146 | Snora36b | M_musculus | Animals | mono_intronic | H/ACA | ENSMUSG00000039318 | Rab3gap2 | protein_coding |
| ENSMUSG00002075155 | Gm54781 | M_musculus | Animals | mono_intergenic | H/ACA | | | intergenic |
| ENSMUSG00002075197 | Gm56153 | M_musculus | Animals | mono_exonic | H/ACA | ENSMUSG00000031939 | Taf1d | protein_coding |
| ENSMUSG00002075203 | Gm55498 | M_musculus | Animals | mono_intronic | Unknown | ENSMUSG00000028060 | Khdc4 | protein_coding |
| ENSMUSG00002075238 | Gm56083 | M_musculus | Animals | mono_intergenic | Unknown | | | intergenic |
| ENSMUSG00002075243 | Gm55993 | M_musculus | Animals | mono_intergenic | Unknown | | | intergenic |
| ENSMUSG00002075255 | Gm55357 | M_musculus | Animals | mono_intronic | Unknown | ENSMUSG00000060036 | Rpl3 | protein_coding |
| ENSMUSG00002075266 | Gm55109 | M_musculus | Animals | intronic_cluster | Unknown | ENSMUSG00000097451 | Rian | non_coding |
| ENSMUSG00002075329 | Gm54692 | M_musculus | Animals | mono_intergenic | Unknown | | | intergenic |
| ENSMUSG00002075355 | Gm54983 | M_musculus | Animals | mono_intergenic | H/ACA | | | intergenic |
| ENSMUSG00002075374 | Gm55458 | M_musculus | Animals | mono_intronic | C/D | ENSMUSG00000025393 | Atp5b | protein_coding |
| ENSMUSG00002075381 | Gm54426 | M_musculus | Animals | mono_intronic | H/ACA | ENSMUSG00000024878 | Cbwd1 | protein_coding |
| ENSMUSG00002075396 | Gm55296 | M_musculus | Animals | mono_intergenic | H/ACA | | | intergenic |
| ENSMUSG00002075399 | Gm54495 | M_musculus | Animals | mono_intergenic | H/ACA | | | intergenic |
| ENSMUSG00002075421 | Gm55268 | M_musculus | Animals | mono_intronic | H/ACA | ENSMUSG00000079323 | Gm20661 | protein_coding |
| ENSMUSG00002075425 | Gm23604 | M_musculus | Animals | intronic_cluster | H/ACA | ENSMUSG00000084954 | Gm16351 | non_coding |
| ENSMUSG00002075453 | Snord3b3 | M_musculus | Animals | intronic_cluster | C/D | ENSMUSG00000010342 | Tex14 | protein_coding |
| ENSMUSG00002075506 | Gm56060 | M_musculus | Animals | mono_intergenic | Unknown | | | intergenic |
| ENSMUSG00002075508 | Gm25053 | M_musculus | Animals | intronic_cluster | H/ACA | ENSMUSG00000055296 | Tmem245 | protein_coding |
| ENSMUSG00002075514 | Gm55577 | M_musculus | Animals | mono_intronic | Unknown | ENSMUSG00000037443 | Cep85 | protein_coding |
| ENSMUSG00002075525 | Snora16a | M_musculus | Animals | intronic_cluster | H/ACA | ENSMUSG00000086290 | Snhg12 | non_coding |
| ENSMUSG00002075554 | Gm55990 | M_musculus | Animals | mono_intergenic | Unknown | | | intergenic |
| ENSMUSG00002075584 | Gm54643 | M_musculus | Animals | mono_intergenic | Unknown | | | intergenic |

| | | | | | | | | |
|---|---|---|---|---|---|---|---|---|
| ENSMUSG00002075586 | Gm54825 | M_musculus | Animals | mono_intronic | Unknown | ENSMUSG00000071415 | Rpl23 | protein_coding |
| ENSMUSG00002075605 | Gm54594 | M_musculus | Animals | mono_intronic | Unknown | ENSMUSG00000051606 | 2010001K21Rik | non_coding |
| ENSMUSG00002075618 | Gm54904 | M_musculus | Animals | mono_intronic | Unknown | ENSMUSG00000021693 | Kif2a | protein_coding |
| ENSMUSG00002075638 | Gm55138 | M_musculus | Animals | mono_intergenic | Unknown | | | intergenic |
| ENSMUSG00002075645 | Gm55920 | M_musculus | Animals | mono_intergenic | H/ACA | | | intergenic |
| ENSMUSG00002075669 | Gm55131 | M_musculus | Animals | mono_intergenic | Unknown | | | intergenic |
| ENSMUSG00002075696 | Gm56132 | M_musculus | Animals | mono_intronic | H/ACA | ENSMUSG00000028431 | Elp1 | protein_coding |
| ENSMUSG00002075731 | Gm55493 | M_musculus | Animals | mono_intergenic | Unknown | | | intergenic |
| ENSMUSG00002075734 | Gm56179 | M_musculus | Animals | mono_intergenic | Unknown | | | intergenic |
| ENSMUSG00002075766 | Gm55482 | M_musculus | Animals | mono_intronic | H/ACA | ENSMUSG00000107388 | Gm42788 | non_coding |
| ENSMUSG00002075771 | Gm54636 | M_musculus | Animals | mono_intergenic | C/D | | | intergenic |
| ENSMUSG00002075779 | Gm56029 | M_musculus | Animals | mono_intronic | Unknown | ENSMUSG00000070280 | Slc22a14 | protein_coding |
| ENSMUSG00002075811 | Gm54830 | M_musculus | Animals | mono_intergenic | H/ACA | | | intergenic |
| ENSMUSG00002075818 | Gm55085 | M_musculus | Animals | mono_intronic | Unknown | ENSMUSG00000047996 | Prrg1 | protein_coding |
| ENSMUSG00002075822 | Gm54541 | M_musculus | Animals | mono_intronic | Unknown | ENSMUSG00000029863 | Casp2 | protein_coding |
| ENSMUSG00002075829 | Gm56239 | M_musculus | Animals | mono_intergenic | Unknown | | | intergenic |
| ENSMUSG00002075833 | Gm26379 | M_musculus | Animals | intergenic_cluster | H/ACA | | | intergenic |
| ENSMUSG00002075875 | Gm54861 | M_musculus | Animals | mono_intergenic | Unknown | | | intergenic |
| ENSMUSG00002075900 | Gm54889 | M_musculus | Animals | mono_intergenic | Unknown | | | intergenic |
| ENSMUSG00002075905 | Gm22149 | M_musculus | Animals | intergenic_cluster | H/ACA | | | intergenic |
| ENSMUSG00002075931 | Snord3b2 | M_musculus | Animals | intronic_cluster | C/D | ENSMUSG00000010342 | Tex14 | protein_coding |
| ENSMUSG00002075950 | Snord80 | M_musculus | Animals | intronic_cluster | C/D | ENSMUSG00000053332 | Gas5 | non_coding |
| ENSMUSG00002075991 | Snora19 | M_musculus | Animals | mono_intronic | H/ACA | ENSMUSG00000024991 | Eif3a | protein_coding |
| ENSMUSG00002076009 | Gm54387 | M_musculus | Animals | mono_intergenic | Unknown | | | intergenic |
| ENSMUSG00002076018 | Gm54926 | M_musculus | Animals | mono_intergenic | Unknown | | | intergenic |
| ENSMUSG00002076040 | Gm56119 | M_musculus | Animals | mono_intergenic | Unknown | | | intergenic |
| ENSMUSG00002076049 | Gm23128 | M_musculus | Animals | intergenic_cluster | H/ACA | | | intergenic |
| ENSMUSG00002076055 | Gm56113 | M_musculus | Animals | mono_intergenic | Unknown | | | intergenic |
| ENSMUSG00002076078 | Gm54444 | M_musculus | Animals | mono_intergenic | Unknown | | | intergenic |
| ENSMUSG00002076123 | Gm55673 | M_musculus | Animals | mono_intergenic | H/ACA | | | intergenic |
| ENSMUSG00002076126 | Gm55563 | M_musculus | Animals | intronic_cluster | Unknown | ENSMUSG00000074867 | Zfp808 | protein_coding |
| ENSMUSG00002076148 | Gm55448 | M_musculus | Animals | mono_intergenic | Unknown | | | intergenic |
| ENSMUSG00002076155 | AF357425 | M_musculus | Animals | mono_intronic | Unknown | ENSMUSG00000097451 | Rian | non_coding |
| ENSMUSG00002076195 | Gm55110 | M_musculus | Animals | mono_intronic | Unknown | ENSMUSG00000097451 | Rian | non_coding |
| ENSMUSG00002076218 | Gm24826 | M_musculus | Animals | intergenic_cluster | C/D | | | intergenic |
| ENSMUSG00002076288 | Snora34 | M_musculus | Animals | mono_intronic | H/ACA | ENSMUSG00000022992 | Kansl2 | protein_coding |
| ENSMUSG00002076292 | Gm54654 | M_musculus | Animals | mono_intergenic | Unknown | | | intergenic |
| ENSMUSG00002076326 | Gm55134 | M_musculus | Animals | mono_intergenic | H/ACA | | | intergenic |
| ENSMUSG00002076359 | Gm55184 | M_musculus | Animals | mono_intergenic | Unknown | | | intergenic |
| ENSMUSG00002076369 | Gm55358 | M_musculus | Animals | mono_intergenic | H/ACA | | | intergenic |
| ENSMUSG00002076372 | Gm54694 | M_musculus | Animals | mono_intergenic | Unknown | | | intergenic |

| | | | | | | | | |
|---|---|---|---|---|---|---|---|---|
| ENSMUSG00002076387 | Gm55013 | M_musculus | Animals | mono_intergenic | Unknown | | | intergenic |
| ENSMUSG00002076402 | Gm55797 | M_musculus | Animals | mono_intergenic | H/ACA | | | intergenic |
| ENSMUSG00002076404 | Gm56318 | M_musculus | Animals | intronic_cluster | Unknown | ENSMUSG00000050587 | Lrrc4c | protein_coding |
| ENSMUSG00002076413 | Gm55141 | M_musculus | Animals | mono_intergenic | Unknown | | | intergenic |
| ENSMUSG00002076417 | Gm54463 | M_musculus | Animals | mono_intergenic | Unknown | | | intergenic |
| ENSMUSG00002076423 | Gm23786 | M_musculus | Animals | intergenic_cluster | H/ACA | | | intergenic |
| ENSMUSG00002076470 | Gm25617 | M_musculus | Animals | intronic_cluster | H/ACA | ENSMUSG00000022812 | Gsk3b | protein_coding |
| ENSMUSG00002076471 | Gm56111 | M_musculus | Animals | mono_intronic | Unknown | ENSMUSG00000062151 | Unc13c | protein_coding |
| ENSMUSG00002076481 | Gm55474 | M_musculus | Animals | mono_intergenic | Unknown | | | intergenic |
| ENSMUSG00002076490 | Gm54634 | M_musculus | Animals | mono_intergenic | Unknown | | | intergenic |
| ENSMUSG00002076499 | Gm54912 | M_musculus | Animals | mono_intergenic | Unknown | | | intergenic |
| ENSMUSG00002076517 | Gm26047 | M_musculus | Animals | intergenic_cluster | H/ACA | | | intergenic |
| ENSMUSG00002076568 | Gm56130 | M_musculus | Animals | mono_intronic | Unknown | ENSMUSG00000036617 | Etl4 | protein_coding |
| ENSMUSG00002076580 | Gm56470 | M_musculus | Animals | mono_intergenic | Unknown | | | intergenic |
| ENSMUSG00002076587 | Gm56121 | M_musculus | Animals | mono_intergenic | Unknown | | | intergenic |
| ENSMUSG00002076594 | Gm56482 | M_musculus | Animals | mono_intergenic | Unknown | | | intergenic |
| ENSMUSG00002076596 | Gm54509 | M_musculus | Animals | mono_intronic | H/ACA | ENSMUSG00000079036 | Alkbh1 | protein_coding |
| ENSMUSG00002076604 | Gm56128 | M_musculus | Animals | mono_intronic | Unknown | ENSMUSG00000037795 | N4bp2 | protein_coding |
| ENSMUSG00002076606 | Gm55862 | M_musculus | Animals | mono_intergenic | Unknown | | | intergenic |
| ENSMUSG00002076646 | Gm56367 | M_musculus | Animals | mono_intergenic | Unknown | | | intergenic |
| ENSMUSG00002076650 | Snord3b1 | M_musculus | Animals | intronic_cluster | C/D | ENSMUSG00000010342 | Tex14 | protein_coding |
| ENSMUSG00002076690 | Gm55111 | M_musculus | Animals | mono_intronic | Unknown | ENSMUSG00000097451 | Rian | non_coding |
| ENSMUSG00002076696 | Gm54507 | M_musculus | Animals | mono_intronic | Unknown | ENSMUSG00000117694 | Snhg4 | protein_coding |
| ENSMUSG00002076715 | Gm55183 | M_musculus | Animals | mono_intergenic | Unknown | | | intergenic |
| ENSMUSG00002076724 | Gm56180 | M_musculus | Animals | mono_intronic | Unknown | ENSMUSG00000026784 | Pdss1 | protein_coding |
| ENSMUSG00002076744 | Gm54405 | M_musculus | Animals | mono_intergenic | Unknown | | | intergenic |
| ENSMUSG00002076766 | Snord33 | M_musculus | Animals | mono_intronic | C/D | ENSMUSG00000074129 | Rpl13a | protein_coding |
| ENSMUSG00002076778 | Gm56319 | M_musculus | Animals | mono_intergenic | Unknown | | | intergenic |
| ENSMUSG00002076802 | Gm55776 | M_musculus | Animals | mono_intronic | Unknown | ENSMUSG00000025583 | Rptor | protein_coding |
| ENSMUSG00002076805 | Gm56170 | M_musculus | Animals | mono_intergenic | Unknown | | | intergenic |
| ENSMUSG00002076811 | Gm55974 | M_musculus | Animals | mono_intronic | H/ACA | ENSMUSG00000043207 | Zmpste24 | protein_coding |
| ENSMUSG00002076818 | Snord14e | M_musculus | Animals | mono_intronic | C/D | ENSMUSG00000015656 | Hspa8 | protein_coding |
| ENSMUSG00002076827 | Gm55462 | M_musculus | Animals | mono_intronic | C/D | ENSMUSG00000026234 | Ncl | protein_coding |
| ENSMUSG00002076883 | Gm55451 | M_musculus | Animals | mono_intergenic | Unknown | | | intergenic |
| ENSMUSG00002076905 | Gm56136 | M_musculus | Animals | mono_intergenic | Unknown | | | intergenic |
| ENSMUSG00002076924 | Gm56109 | M_musculus | Animals | mono_intergenic | Unknown | | | intergenic |
| ENSMUSG00002076935 | Gm55566 | M_musculus | Animals | intronic_cluster | Unknown | ENSMUSG00000055228 | Gm49359 | protein_coding |
| ENSMUSG00002076938 | Gm54937 | M_musculus | Animals | mono_intergenic | Unknown | | | intergenic |
| ENSMUSG00002076946 | Gm54847 | M_musculus | Animals | mono_intergenic | Unknown | | | intergenic |
| ENSMUSG00002076971 | Snord100 | M_musculus | Animals | mono_intronic | C/D | ENSMUSG00000061983 | Rps12 | protein_coding |
| ENSMUSG00002076975 | Gm23823 | M_musculus | Animals | mono_intergenic | H/ACA | | | intergenic |

| | | | | | | | | |
|---|---|---|---|---|---|---|---|---|
| ENSMUSG00002076990 | Gm22711 | M_musculus | Animals | mono_exonic | H/ACA | ENSMUSG00000120012 | Gm36220 | non_coding |
| ENSOANG00000016314 | SNORD29 | O_anatinus | Animals | intergenic_cluster | C/D | | | intergenic |
| ENSOANG00000016482 | SNORA41 | O_anatinus | Animals | mono_intronic | H/ACA | ENSOANG00000012964 | EEF1B2 | protein_coding |
| ENSOANG00000016536 | SNORD58 | O_anatinus | Animals | mono_intronic | C/D | ENSOANG00000002910 | | protein_coding |
| ENSOANG00000016633 | ENSOANG00000016633 | O_anatinus | Animals | mono_intronic | C/D | ENSOANG00000008205 | | protein_coding |
| ENSOANG00000016852 | ENSOANG00000016852 | O_anatinus | Animals | mono_intronic | C/D | ENSOANG00000008205 | | protein_coding |
| ENSOANG00000016886 | ENSOANG00000016886 | O_anatinus | Animals | intergenic_cluster | H/ACA | | | intergenic |
| ENSOANG00000016931 | SNORD36 | O_anatinus | Animals | mono_intronic | C/D | ENSOANG00000007060 | | protein_coding |
| ENSOANG00000016941 | ENSOANG00000016941 | O_anatinus | Animals | mono_intronic | C/D | ENSOANG00000008205 | | protein_coding |
| ENSOANG00000016942 | SNORD42 | O_anatinus | Animals | mono_intronic | C/D | ENSOANG00000009558 | RPL23A | protein_coding |
| ENSOANG00000017111 | SNORD73 | O_anatinus | Animals | mono_intronic | C/D | ENSOANG00000000209 | | protein_coding |
| ENSOANG00000017183 | ENSOANG00000017183 | O_anatinus | Animals | mono_intronic | H/ACA | ENSOANG00000009054 | | protein_coding |
| ENSOANG00000017185 | ENSOANG00000017185 | O_anatinus | Animals | mono_intronic | C/D | ENSOANG00000002420 | NOP58 | protein_coding |
| ENSOANG00000017212 | ENSOANG00000017212 | O_anatinus | Animals | mono_intronic | H/ACA | ENSOANG00000007359 | EIF3A | protein_coding |
| ENSOANG00000017220 | ENSOANG00000017220 | O_anatinus | Animals | mono_intronic | C/D | ENSOANG00000008637 | TSR1 | protein_coding |
| ENSOANG00000017225 | SNORA62 | O_anatinus | Animals | mono_intronic | H/ACA | ENSOANG00000007053 | | protein_coding |
| ENSOANG00000017239 | SNORD83 | O_anatinus | Animals | mono_intergenic | C/D | | | intergenic |
| ENSOANG00000017253 | ENSOANG00000017253 | O_anatinus | Animals | mono_intronic | H/ACA | ENSOANG00000036977 | SSR4 | protein_coding |
| ENSOANG00000017257 | ENSOANG00000017257 | O_anatinus | Animals | mono_intronic | C/D | ENSOANG00000002600 | RACK1 | protein_coding |
| ENSOANG00000017290 | ENSOANG00000017290 | O_anatinus | Animals | mono_intronic | C/D | ENSOANG00000003235 | ATP5F1B | protein_coding |
| ENSOANG00000017326 | SNORD62 | O_anatinus | Animals | intronic_cluster | C/D | ENSOANG00000043186 | STIMATE | protein_coding |
| ENSOANG00000017327 | ENSOANG00000017327 | O_anatinus | Animals | mono_intergenic | H/ACA | | | intergenic |
| ENSOANG00000017340 | SNORD7 | O_anatinus | Animals | mono_intergenic | C/D | | | intergenic |
| ENSOANG00000017345 | ENSOANG00000017345 | O_anatinus | Animals | intergenic_cluster | H/ACA | | | intergenic |
| ENSOANG00000017419 | SNORD104 | O_anatinus | Animals | intergenic_cluster | C/D | | | intergenic |
| ENSOANG00000017420 | ENSOANG00000017420 | O_anatinus | Animals | intergenic_cluster | C/D | | | intergenic |
| ENSOANG00000017424 | ENSOANG00000017424 | O_anatinus | Animals | mono_intronic | C/D | ENSOANG00000008637 | TSR1 | protein_coding |
| ENSOANG00000017486 | ENSOANG00000017486 | O_anatinus | Animals | intergenic_cluster | H/ACA | | | intergenic |
| ENSOANG00000017488 | SNORA21 | O_anatinus | Animals | mono_intronic | H/ACA | ENSOANG00000039366 | RPL23 | protein_coding |
| ENSOANG00000017507 | SNORD42 | O_anatinus | Animals | mono_intronic | C/D | ENSOANG00000009558 | RPL23A | protein_coding |
| ENSOANG00000017519 | SNORD90 | O_anatinus | Animals | mono_intronic | C/D | ENSOANG00000008194 | RC3H2 | protein_coding |
| ENSOANG00000017546 | ENSOANG00000017546 | O_anatinus | Animals | mono_intronic | H/ACA | ENSOANG00000005844 | KANSL2 | protein_coding |
| ENSOANG00000017559 | ENSOANG00000017559 | O_anatinus | Animals | mono_intronic | C/D | ENSOANG00000041245 | CHD8 | protein_coding |
| ENSOANG00000017566 | SNORA66 | O_anatinus | Animals | mono_intronic | H/ACA | ENSOANG00000004669 | RPL5 | protein_coding |
| ENSOANG00000017580 | ENSOANG00000017580 | O_anatinus | Animals | mono_intergenic | H/ACA | | | intergenic |
| ENSOANG00000017597 | SNORD68 | O_anatinus | Animals | mono_intronic | C/D | ENSOANG00000006729 | RPL13 | protein_coding |
| ENSOANG00000017614 | SNORD36 | O_anatinus | Animals | mono_intronic | C/D | ENSOANG00000007060 | | protein_coding |
| ENSOANG00000017615 | SNORA23 | O_anatinus | Animals | mono_intronic | H/ACA | ENSOANG00000005852 | IPO7 | protein_coding |
| ENSOANG00000017637 | SNORD70B | O_anatinus | Animals | mono_intronic | C/D | ENSOANG00000002420 | NOP58 | protein_coding |
| ENSOANG00000017653 | ENSOANG00000017653 | O_anatinus | Animals | intergenic_cluster | C/D | | | intergenic |
| ENSOANG00000017671 | SNORD57 | O_anatinus | Animals | mono_intronic | C/D | ENSOANG00000000505 | NOP56 | protein_coding |

| | | | | | | | | |
|---|---|---|---|---|---|---|---|---|
| ENSOANG00000017736 | SNORA53 | O_anatinus | Animals | mono_intronic | H/ACA | ENSOANG00000005594 | SLC25A3 | protein_coding |
| ENSOANG00000017750 | ENSOANG00000017750 | O_anatinus | Animals | mono_intergenic | C/D | | | intergenic |
| ENSOANG00000017759 | SNORD73 | O_anatinus | Animals | mono_intronic | C/D | ENSOANG00000039121 | | protein_coding |
| ENSOANG00000017769 | SNORD58 | O_anatinus | Animals | mono_intronic | C/D | ENSOANG00000002910 | | protein_coding |
| ENSOANG00000017805 | SNORA2C | O_anatinus | Animals | mono_intronic | H/ACA | ENSOANG00000005844 | KANSL2 | protein_coding |
| ENSOANG00000017835 | SNORD71 | O_anatinus | Animals | mono_intronic | C/D | ENSOANG00000008186 | AP1G1 | protein_coding |
| ENSOANG00000017857 | ENSOANG00000017857 | O_anatinus | Animals | mono_intronic | H/ACA | ENSOANG00000011713 | | protein_coding |
| ENSOANG00000017859 | ENSOANG00000017859 | O_anatinus | Animals | mono_intronic | H/ACA | ENSOANG00000012109 | CNOT1 | protein_coding |
| ENSOANG00000017868 | ENSOANG00000017868 | O_anatinus | Animals | mono_intronic | H/ACA | ENSOANG00000000505 | NOP56 | protein_coding |
| ENSOANG00000017951 | ENSOANG00000017951 | O_anatinus | Animals | mono_intronic | H/ACA | ENSOANG00000036977 | SSR4 | protein_coding |
| ENSOANG00000017978 | ENSOANG00000017978 | O_anatinus | Animals | mono_intronic | H/ACA | ENSOANG00000047981 | TPT1 | protein_coding |
| ENSOANG00000017997 | SNORD83 | O_anatinus | Animals | mono_intronic | C/D | ENSOANG00000008713 | DKC1 | protein_coding |
| ENSOANG00000018008 | SNORD14 | O_anatinus | Animals | mono_intronic | C/D | ENSOANG00000014580 | HSPA8 | protein_coding |
| ENSOANG00000018037 | SNORA75 | O_anatinus | Animals | mono_intronic | H/ACA | ENSOANG00000008732 | | protein_coding |
| ENSOANG00000018059 | SNORD18 | O_anatinus | Animals | mono_intronic | C/D | ENSOANG00000048650 | RPL4 | protein_coding |
| ENSOANG00000018074 | ENSOANG00000018074 | O_anatinus | Animals | mono_intronic | H/ACA | ENSOANG00000010001 | PABPC4 | protein_coding |
| ENSOANG00000018079 | ENSOANG00000018079 | O_anatinus | Animals | intergenic_cluster | H/ACA | | | intergenic |
| ENSOANG00000018092 | SNORD33 | O_anatinus | Animals | mono_intronic | C/D | ENSOANG00000000828 | RPL13A | protein_coding |
| ENSOANG00000018133 | SNORD18 | O_anatinus | Animals | mono_intronic | C/D | ENSOANG00000048650 | RPL4 | protein_coding |
| ENSOANG00000018147 | ENSOANG00000018147 | O_anatinus | Animals | mono_intronic | C/D | ENSOANG00000000505 | NOP56 | protein_coding |
| ENSOANG00000018200 | ENSOANG00000018200 | O_anatinus | Animals | mono_intronic | H/ACA | ENSOANG00000036977 | SSR4 | protein_coding |
| ENSOANG00000018225 | SNORA72 | O_anatinus | Animals | mono_exonic | H/ACA | ENSOANG00000047660 | | protein_coding |
| ENSOANG00000018226 | ENSOANG00000018226 | O_anatinus | Animals | intergenic_cluster | C/D | | | intergenic |
| ENSOANG00000018253 | SNORD19 | O_anatinus | Animals | mono_intronic | C/D | ENSOANG00000013437 | GNL3 | protein_coding |
| ENSOANG00000018255 | ENSOANG00000018255 | O_anatinus | Animals | intergenic_cluster | C/D | | | intergenic |
| ENSOANG00000018268 | SNORD18 | O_anatinus | Animals | mono_intronic | C/D | ENSOANG00000048650 | RPL4 | protein_coding |
| ENSOANG00000018280 | ENSOANG00000018280 | O_anatinus | Animals | intergenic_cluster | C/D | | | intergenic |
| ENSOANG00000018304 | ENSOANG00000018304 | O_anatinus | Animals | mono_intronic | H/ACA | ENSOANG00000015366 | TBRG4 | protein_coding |
| ENSOANG00000018325 | SNORD18 | O_anatinus | Animals | mono_intronic | C/D | ENSOANG00000048650 | RPL4 | protein_coding |
| ENSOANG00000018344 | SNORD12C | O_anatinus | Animals | mono_intergenic | C/D | | | intergenic |
| ENSOANG00000018359 | SNORD21 | O_anatinus | Animals | mono_intronic | C/D | ENSOANG00000004669 | RPL5 | protein_coding |
| ENSOANG00000018391 | SNORD14 | O_anatinus | Animals | mono_intronic | C/D | ENSOANG00000014580 | HSPA8 | protein_coding |
| ENSOANG00000018400 | SNORD20 | O_anatinus | Animals | mono_intronic | C/D | ENSOANG00000008732 | | protein_coding |
| ENSOANG00000018440 | ENSOANG00000018440 | O_anatinus | Animals | intergenic_cluster | H/ACA | | | intergenic |
| ENSOANG00000018474 | ENSOANG00000018474 | O_anatinus | Animals | mono_intergenic | C/D | | | intergenic |
| ENSOANG00000018485 | ENSOANG00000018485 | O_anatinus | Animals | mono_intronic | C/D | ENSOANG00000002765 | CKAP5 | protein_coding |
| ENSOANG00000018496 | SNORA1 | O_anatinus | Animals | intergenic_cluster | H/ACA | | | intergenic |
| ENSOANG00000018500 | ENSOANG00000018500 | O_anatinus | Animals | mono_intergenic | C/D | | | intergenic |
| ENSOANG00000018504 | ENSOANG00000018504 | O_anatinus | Animals | mono_intronic | H/ACA | ENSOANG00000010001 | PABPC4 | protein_coding |
| ENSOANG00000018538 | ENSOANG00000018538 | O_anatinus | Animals | mono_intronic | C/D | ENSOANG00000014032 | | protein_coding |
| ENSOANG00000018540 | ENSOANG00000018540 | O_anatinus | Animals | mono_intronic | H/ACA | ENSOANG00000006440 | | protein_coding |

| | | | | | | | | |
|---|---|---|---|---|---|---|---|---|
| ENSOANG00000018544 | ENSOANG00000018544 | O_anatinus | Animals | mono_intronic | H/ACA | ENSOANG00000010444 | CCT6B | protein_coding |
| ENSOANG00000018545 | ENSOANG00000018545 | O_anatinus | Animals | mono_intronic | C/D | ENSOANG00000008205 | | protein_coding |
| ENSOANG00000018574 | ENSOANG00000018574 | O_anatinus | Animals | intergenic_cluster | C/D | | | intergenic |
| ENSOANG00000018620 | U3 | O_anatinus | Animals | intronic_cluster | C/D | ENSOANG00000008488 | | protein_coding |
| ENSOANG00000018639 | SNORD58 | O_anatinus | Animals | mono_intronic | C/D | ENSOANG00000002910 | | protein_coding |
| ENSOANG00000018682 | SNORD69 | O_anatinus | Animals | mono_intronic | C/D | ENSOANG00000013437 | GNL3 | protein_coding |
| ENSOANG00000018692 | SNORA49 | O_anatinus | Animals | mono_intronic | H/ACA | ENSOANG00000006596 | EP400 | protein_coding |
| ENSOANG00000018728 | ENSOANG00000018728 | O_anatinus | Animals | mono_intronic | C/D | ENSOANG00000013665 | RPL3 | protein_coding |
| ENSOANG00000018741 | ENSOANG00000018741 | O_anatinus | Animals | mono_intronic | C/D | ENSOANG00000005781 | EIF4G1 | protein_coding |
| ENSOANG00000018758 | ENSOANG00000018758 | O_anatinus | Animals | mono_intronic | H/ACA | ENSOANG00000009633 | EIF4E | protein_coding |
| ENSOANG00000018802 | ENSOANG00000018802 | O_anatinus | Animals | intergenic_cluster | C/D | | | intergenic |
| ENSOANG00000018848 | SNORD22 | O_anatinus | Animals | intergenic_cluster | C/D | | | intergenic |
| ENSOANG00000018853 | ENSOANG00000018853 | O_anatinus | Animals | mono_intronic | C/D | ENSOANG00000008637 | TSR1 | protein_coding |
| ENSOANG00000018859 | SNORD16 | O_anatinus | Animals | mono_intronic | C/D | ENSOANG00000048650 | RPL4 | protein_coding |
| ENSOANG00000018870 | ENSOANG00000018870 | O_anatinus | Animals | mono_intergenic | C/D | | | intergenic |
| ENSOANG00000018875 | ENSOANG00000018875 | O_anatinus | Animals | intergenic_cluster | H/ACA | | | intergenic |
| ENSOANG00000018919 | SNORD83 | O_anatinus | Animals | mono_intronic | C/D | ENSOANG00000008361 | Bat1 | protein_coding |
| ENSOANG00000018963 | ENSOANG00000018963 | O_anatinus | Animals | intergenic_cluster | C/D | | | intergenic |
| ENSOANG00000018966 | ENSOANG00000018966 | O_anatinus | Animals | mono_intergenic | C/D | | | intergenic |
| ENSOANG00000018969 | SNORA71 | O_anatinus | Animals | mono_intronic | H/ACA | ENSOANG00000013360 | RPS17 | protein_coding |
| ENSOANG00000018997 | SNORA70 | O_anatinus | Animals | mono_intronic | H/ACA | ENSOANG00000011015 | | protein_coding |
| ENSOANG00000019011 | ENSOANG00000019011 | O_anatinus | Animals | mono_intronic | C/D | ENSOANG00000014755 | COX7C | protein_coding |
| ENSOANG00000019014 | ENSOANG00000019014 | O_anatinus | Animals | intergenic_cluster | C/D | | | intergenic |
| ENSOANG00000019015 | ENSOANG00000019015 | O_anatinus | Animals | mono_intergenic | C/D | | | intergenic |
| ENSOANG00000019017 | ENSOANG00000019017 | O_anatinus | Animals | intergenic_cluster | C/D | | | intergenic |
| ENSOANG00000019033 | ENSOANG00000019033 | O_anatinus | Animals | mono_intronic | C/D | ENSOANG00000012964 | EEF1B2 | protein_coding |
| ENSOANG00000019037 | SNORD61 | O_anatinus | Animals | mono_intronic | C/D | ENSOANG00000011670 | | protein_coding |
| ENSOANG00000019058 | ENSOANG00000019058 | O_anatinus | Animals | mono_intronic | H/ACA | ENSOANG00000046605 | RPL39 | protein_coding |
| ENSOANG00000019083 | SNORD14 | O_anatinus | Animals | mono_intronic | C/D | ENSOANG00000007687 | RPS13 | protein_coding |
| ENSOANG00000019091 | ENSOANG00000019091 | O_anatinus | Animals | mono_intergenic | C/D | | | intergenic |
| ENSOANG00000019092 | SNORD62 | O_anatinus | Animals | mono_intronic | C/D | ENSOANG00000014780 | PRRC2B | protein_coding |
| ENSOANG00000019122 | ENSOANG00000019122 | O_anatinus | Animals | mono_intronic | H/ACA | ENSOANG00000000767 | EIF5 | protein_coding |
| ENSOANG00000019125 | SNORD28 | O_anatinus | Animals | intergenic_cluster | C/D | | | intergenic |
| ENSOANG00000019127 | SNORD16 | O_anatinus | Animals | mono_intronic | C/D | ENSOANG00000048650 | RPL4 | protein_coding |
| ENSOANG00000019193 | ENSOANG00000019193 | O_anatinus | Animals | mono_intronic | C/D | ENSOANG00000005781 | EIF4G1 | protein_coding |
| ENSOANG00000019198 | SNORA70 | O_anatinus | Animals | mono_intronic | H/ACA | ENSOANG00000011015 | | protein_coding |
| ENSOANG00000019204 | ENSOANG00000019204 | O_anatinus | Animals | mono_intronic | H/ACA | ENSOANG00000041148 | | protein_coding |
| ENSOANG00000019207 | SNORD24 | O_anatinus | Animals | mono_intronic | C/D | ENSOANG00000007060 | | protein_coding |
| ENSOANG00000019269 | ENSOANG00000019269 | O_anatinus | Animals | mono_intronic | C/D | ENSOANG00000002600 | RACK1 | protein_coding |
| ENSOANG00000019283 | SNORD14 | O_anatinus | Animals | mono_intronic | C/D | ENSOANG00000007687 | RPS13 | protein_coding |
| ENSOANG00000019300 | ENSOANG00000019300 | O_anatinus | Animals | mono_intronic | H/ACA | ENSOANG00000006440 | | protein_coding |

| | | | | | | | | |
|---|---|---|---|---|---|---|---|---|
| ENSOANG00000019307 | ENSOANG00000019307 | O_anatinus | Animals | mono_intronic | H/ACA | ENSOANG00000014453 | CLPTM1L | protein_coding |
| ENSOANG00000019328 | ENSOANG00000019328 | O_anatinus | Animals | mono_intergenic | C/D | | | intergenic |
| ENSOANG00000019329 | ENSOANG00000019329 | O_anatinus | Animals | mono_intergenic | C/D | | | intergenic |
| ENSOANG00000022867 | SNORD99 | O_anatinus | Animals | intergenic_cluster | C/D | | | intergenic |
| ENSOANG00000023059 | ENSOANG00000023059 | O_anatinus | Animals | mono_intronic | H/ACA | ENSOANG00000014576 | RANBP1 | protein_coding |
| ENSOANG00000023578 | ENSOANG00000023578 | O_anatinus | Animals | intergenic_cluster | C/D | | | intergenic |
| ENSOANG00000023622 | SNORD89 | O_anatinus | Animals | mono_intronic | C/D | ENSOANG00000003086 | RNF149 | protein_coding |
| ENSOANG00000023630 | ENSOANG00000023630 | O_anatinus | Animals | intergenic_cluster | C/D | | | intergenic |
| ENSOANG00000023888 | ENSOANG00000023888 | O_anatinus | Animals | mono_intronic | C/D | ENSOANG00000001161 | SF3B3 | protein_coding |
| ENSOANG00000024060 | SNORD111B | O_anatinus | Animals | mono_intronic | C/D | ENSOANG00000001161 | SF3B3 | protein_coding |
| ENSOANG00000024268 | ENSOANG00000024268 | O_anatinus | Animals | mono_intergenic | C/D | | | intergenic |
| ENSOANG00000024339 | ENSOANG00000024339 | O_anatinus | Animals | mono_intronic | C/D | ENSOANG00000001161 | SF3B3 | protein_coding |
| ENSOANG00000024420 | ENSOANG00000024420 | O_anatinus | Animals | mono_intergenic | C/D | | | intergenic |
| ENSOANG00000024540 | ENSOANG00000024540 | O_anatinus | Animals | mono_intergenic | H/ACA | | | intergenic |
| ENSOANG00000024575 | ENSOANG00000024575 | O_anatinus | Animals | mono_intronic | C/D | ENSOANG00000004902 | PUM1 | protein_coding |
| ENSOANG00000024666 | ENSOANG00000024666 | O_anatinus | Animals | mono_intronic | C/D | ENSOANG00000004902 | PUM1 | protein_coding |
| ENSOANG00000024771 | ENSOANG00000024771 | O_anatinus | Animals | intergenic_cluster | C/D | | | intergenic |
| ENSOANG00000024781 | SNORA79B | O_anatinus | Animals | mono_intergenic | H/ACA | | | intergenic |
| ENSOANG00000024863 | ENSOANG00000024863 | O_anatinus | Animals | mono_intronic | C/D | ENSOANG00000008713 | DKC1 | protein_coding |
| ENSOANG00000024913 | ENSOANG00000024913 | O_anatinus | Animals | mono_intergenic | H/ACA | | | intergenic |
| ENSOANG00000024964 | ENSOANG00000024964 | O_anatinus | Animals | mono_intergenic | C/D | | | intergenic |
| ENSOANG00000025006 | ENSOANG00000025006 | O_anatinus | Animals | mono_intergenic | H/ACA | | | intergenic |
| ENSOANG00000025060 | ENSOANG00000025060 | O_anatinus | Animals | mono_intergenic | H/ACA | | | intergenic |
| ENSOANG00000025075 | ENSOANG00000025075 | O_anatinus | Animals | intergenic_cluster | C/D | | | intergenic |
| ENSOANG00000025131 | ENSOANG00000025131 | O_anatinus | Animals | mono_intronic | H/ACA | ENSOANG00000007359 | EIF3A | protein_coding |
| ENSOANG00000025161 | ENSOANG00000025161 | O_anatinus | Animals | mono_intergenic | H/ACA | | | intergenic |
| ENSOANG00000025176 | ENSOANG00000025176 | O_anatinus | Animals | mono_intergenic | H/ACA | | | intergenic |
| ENSOANG00000025199 | ENSOANG00000025199 | O_anatinus | Animals | mono_intronic | H/ACA | ENSOANG00000008713 | DKC1 | protein_coding |
| ENSOANG00000025246 | SNORD63 | O_anatinus | Animals | mono_intronic | C/D | ENSOANG00000002817 | HSPA9 | protein_coding |
| ENSOANG00000025284 | SNORD82 | O_anatinus | Animals | mono_intronic | C/D | ENSOANG00000008732 | | protein_coding |
| ENSOANG00000025337 | ENSOANG00000025337 | O_anatinus | Animals | mono_intergenic | H/ACA | | | intergenic |
| ENSOANG00000025369 | ENSOANG00000025369 | O_anatinus | Animals | mono_intergenic | H/ACA | | | intergenic |
| ENSOANG00000025441 | ENSOANG00000025441 | O_anatinus | Animals | mono_intergenic | H/ACA | | | intergenic |
| ENSOANG00000025452 | U3 | O_anatinus | Animals | mono_intergenic | C/D | | | intergenic |
| ENSOANG00000025484 | ENSOANG00000025484 | O_anatinus | Animals | mono_intergenic | H/ACA | | | intergenic |
| ENSOANG00000025490 | ENSOANG00000025490 | O_anatinus | Animals | intergenic_cluster | H/ACA | | | intergenic |
| ENSOANG00000025493 | ENSOANG00000025493 | O_anatinus | Animals | mono_intergenic | H/ACA | | | intergenic |
| ENSOANG00000025507 | ENSOANG00000025507 | O_anatinus | Animals | mono_intronic | C/D | ENSOANG00000004902 | PUM1 | protein_coding |
| ENSOANG00000025513 | ENSOANG00000025513 | O_anatinus | Animals | mono_intergenic | H/ACA | | | intergenic |
| ENSOANG00000025517 | ENSOANG00000025517 | O_anatinus | Animals | mono_intronic | H/ACA | ENSOANG00000012198 | TCP1 | protein_coding |
| ENSOANG00000025548 | ENSOANG00000025548 | O_anatinus | Animals | mono_intronic | C/D | ENSOANG00000004863 | RPL37 | protein_coding |

| | | | | | | | | |
|---|---|---|---|---|---|---|---|---|
| ENSOANG00000025593 | ENSOANG00000025593 | O_anatinus | Animals | mono_intronic | H/ACA | ENSOANG00000045478 | LRMDA | protein_coding |
| ENSOANG00000025615 | SNORA74 | O_anatinus | Animals | mono_intronic | H/ACA | ENSOANG00000050738 | | protein_coding |
| ENSOANG00000025639 | ENSOANG00000025639 | O_anatinus | Animals | mono_intronic | H/ACA | ENSOANG00000003214 | | protein_coding |
| ENSOANG00000025715 | ENSOANG00000025715 | O_anatinus | Animals | mono_intronic | H/ACA | ENSOANG00000012198 | TCP1 | protein_coding |
| ENSOANG00000025740 | ENSOANG00000025740 | O_anatinus | Animals | mono_intronic | H/ACA | ENSOANG00000009129 | RPL18A | protein_coding |
| ENSOANG00000025754 | ENSOANG00000025754 | O_anatinus | Animals | mono_intergenic | H/ACA | | | intergenic |
| ENSOANG00000025774 | ENSOANG00000025774 | O_anatinus | Animals | mono_intronic | H/ACA | ENSOANG00000004644 | | protein_coding |
| ENSOANG00000025793 | ENSOANG00000025793 | O_anatinus | Animals | mono_intronic | H/ACA | ENSOANG00000050686 | EIF4A1 | protein_coding |
| ENSOANG00000025939 | ENSOANG00000025939 | O_anatinus | Animals | mono_intergenic | H/ACA | | | intergenic |
| ENSOANG00000025977 | ENSOANG00000025977 | O_anatinus | Animals | mono_intergenic | H/ACA | | | intergenic |
| ENSOANG00000026047 | U3 | O_anatinus | Animals | mono_intergenic | C/D | | | intergenic |
| ENSOANG00000026111 | SNORD4A | O_anatinus | Animals | mono_intronic | C/D | ENSOANG00000009558 | RPL23A | protein_coding |
| ENSOANG00000026224 | ENSOANG00000026224 | O_anatinus | Animals | mono_intergenic | H/ACA | | | intergenic |
| ENSOANG00000026231 | ENSOANG00000026231 | O_anatinus | Animals | intergenic_cluster | C/D | | | intergenic |
| ENSOANG00000026260 | ENSOANG00000026260 | O_anatinus | Animals | mono_intergenic | H/ACA | | | intergenic |
| ENSOANG00000026348 | ENSOANG00000026348 | O_anatinus | Animals | mono_intronic | H/ACA | ENSOANG00000042440 | | non_coding |
| ENSOANG00000026354 | SNORA19 | O_anatinus | Animals | mono_intronic | H/ACA | ENSOANG00000007359 | EIF3A | protein_coding |
| ENSOANG00000026369 | ENSOANG00000026369 | O_anatinus | Animals | mono_intergenic | H/ACA | | | intergenic |
| ENSOANG00000026416 | ENSOANG00000026416 | O_anatinus | Animals | mono_intergenic | C/D | | | intergenic |
| ENSOANG00000026445 | ENSOANG00000026445 | O_anatinus | Animals | intergenic_cluster | C/D | | | intergenic |
| ENSOANG00000026483 | SNORA74 | O_anatinus | Animals | mono_intergenic | H/ACA | | | intergenic |
| ENSOANG00000026547 | ENSOANG00000026547 | O_anatinus | Animals | mono_intergenic | H/ACA | | | intergenic |
| ENSOANG00000026550 | ENSOANG00000026550 | O_anatinus | Animals | mono_intergenic | H/ACA | | | intergenic |
| ENSOANG00000026552 | ENSOANG00000026552 | O_anatinus | Animals | mono_intergenic | H/ACA | | | intergenic |
| ENSOANG00000026554 | ENSOANG00000026554 | O_anatinus | Animals | mono_intronic | H/ACA | ENSOANG00000000767 | EIF5 | protein_coding |
| ENSOANG00000026577 | ENSOANG00000026577 | O_anatinus | Animals | mono_intergenic | H/ACA | | | intergenic |
| ENSOANG00000026686 | ENSOANG00000026686 | O_anatinus | Animals | mono_intronic | H/ACA | ENSOANG00000004150 | EIF3L | protein_coding |
| ENSOANG00000026783 | ENSOANG00000026783 | O_anatinus | Animals | mono_intergenic | H/ACA | | | intergenic |
| ENSOANG00000026792 | ENSOANG00000026792 | O_anatinus | Animals | mono_intergenic | H/ACA | | | intergenic |
| ENSOANG00000026800 | ENSOANG00000026800 | O_anatinus | Animals | mono_intergenic | C/D | | | intergenic |
| ENSOANG00000026815 | ENSOANG00000026815 | O_anatinus | Animals | mono_intronic | C/D | ENSOANG00000008713 | DKC1 | protein_coding |
| ENSOANG00000026837 | ENSOANG00000026837 | O_anatinus | Animals | mono_intergenic | H/ACA | | | intergenic |
| ENSOANG00000026845 | ENSOANG00000026845 | O_anatinus | Animals | mono_intergenic | H/ACA | | | intergenic |
| ENSOANG00000026861 | U3 | O_anatinus | Animals | mono_intergenic | C/D | | | intergenic |
| ENSOANG00000026873 | ENSOANG00000026873 | O_anatinus | Animals | mono_intergenic | H/ACA | | | intergenic |
| ENSOANG00000026944 | ENSOANG00000026944 | O_anatinus | Animals | mono_intergenic | H/ACA | | | intergenic |
| ENSOANG00000026973 | ENSOANG00000026973 | O_anatinus | Animals | mono_intergenic | H/ACA | | | intergenic |
| ENSOANG00000027041 | ENSOANG00000027041 | O_anatinus | Animals | mono_intergenic | H/ACA | | | intergenic |
| ENSOANG00000027108 | ENSOANG00000027108 | O_anatinus | Animals | mono_intergenic | H/ACA | | | intergenic |
| ENSOANG00000027132 | ENSOANG00000027132 | O_anatinus | Animals | intergenic_cluster | C/D | | | intergenic |
| ENSOANG00000027145 | ENSOANG00000027145 | O_anatinus | Animals | mono_intergenic | H/ACA | | | intergenic |

| | | | | | | | | |
|---|---|---|---|---|---|---|---|---|
| ENSOANG00000027149 | ENSOANG00000027149 | O_anatinus | Animals | mono_intergenic | H/ACA | | | intergenic |
| ENSOANG00000027164 | ENSOANG00000027164 | O_anatinus | Animals | mono_intergenic | H/ACA | | | intergenic |
| ENSOANG00000027172 | ENSOANG00000027172 | O_anatinus | Animals | mono_intronic | C/D | ENSOANG00000013665 | RPL3 | protein_coding |
| ENSOANG00000027173 | ENSOANG00000027173 | O_anatinus | Animals | mono_intergenic | C/D | | | intergenic |
| ENSOANG00000027184 | ENSOANG00000027184 | O_anatinus | Animals | mono_intronic | H/ACA | ENSOANG00000051221 | SNRNP200 | protein_coding |
| ENSOANG00000027229 | U3 | O_anatinus | Animals | mono_intergenic | C/D | | | intergenic |
| ENSOANG00000027266 | ENSOANG00000027266 | O_anatinus | Animals | mono_intronic | C/D | ENSOANG00000010219 | UBAP2 | protein_coding |
| ENSOANG00000027320 | ENSOANG00000027320 | O_anatinus | Animals | mono_intronic | H/ACA | ENSOANG00000011192 | EML6 | protein_coding |
| ENSOANG00000027333 | ENSOANG00000027333 | O_anatinus | Animals | mono_intronic | H/ACA | ENSOANG00000004527 | CORO2A | protein_coding |
| ENSOANG00000027337 | ENSOANG00000027337 | O_anatinus | Animals | mono_intergenic | H/ACA | | | intergenic |
| ENSOANG00000027364 | ENSOANG00000027364 | O_anatinus | Animals | mono_intronic | H/ACA | ENSOANG00000011688 | PABIR2 | protein_coding |
| ENSOANG00000027367 | ENSOANG00000027367 | O_anatinus | Animals | mono_intergenic | H/ACA | | | intergenic |
| ENSOANG00000027382 | ENSOANG00000027382 | O_anatinus | Animals | mono_intronic | C/D | ENSOANG00000042142 | EIF4A2 | protein_coding |
| ENSOANG00000027403 | ENSOANG00000027403 | O_anatinus | Animals | mono_intergenic | H/ACA | | | intergenic |
| ENSOANG00000027417 | ENSOANG00000027417 | O_anatinus | Animals | mono_intergenic | H/ACA | | | intergenic |
| ENSOANG00000027422 | ENSOANG00000027422 | O_anatinus | Animals | mono_intronic | H/ACA | ENSOANG00000042142 | EIF4A2 | protein_coding |
| ENSOANG00000027424 | SNORD126 | O_anatinus | Animals | intergenic_cluster | C/D | | | intergenic |
| ENSOANG00000027448 | ENSOANG00000027448 | O_anatinus | Animals | mono_intergenic | H/ACA | | | intergenic |
| ENSOANG00000027466 | ENSOANG00000027466 | O_anatinus | Animals | mono_intergenic | H/ACA | | | intergenic |
| ENSOANG00000027475 | SNORD27 | O_anatinus | Animals | intergenic_cluster | C/D | | | intergenic |
| ENSOANG00000027477 | ENSOANG00000027477 | O_anatinus | Animals | mono_intronic | C/D | ENSOANG00000010219 | UBAP2 | protein_coding |
| ENSOANG00000027485 | ENSOANG00000027485 | O_anatinus | Animals | mono_intronic | H/ACA | ENSOANG00000011688 | PABIR2 | protein_coding |
| ENSOANG00000027486 | ENSOANG00000027486 | O_anatinus | Animals | mono_intronic | C/D | ENSOANG00000010219 | UBAP2 | protein_coding |
| ENSOANG00000027498 | ENSOANG00000027498 | O_anatinus | Animals | mono_intergenic | C/D | | | intergenic |
| ENSOANG00000027537 | ENSOANG00000027537 | O_anatinus | Animals | mono_intronic | H/ACA | ENSOANG00000002486 | CWF19L1 | protein_coding |
| ENSOANG00000027577 | ENSOANG00000027577 | O_anatinus | Animals | intergenic_cluster | H/ACA | | | intergenic |
| ENSOANG00000027598 | ENSOANG00000027598 | O_anatinus | Animals | mono_intronic | H/ACA | ENSOANG00000001726 | DLG5 | protein_coding |
| ENSOANG00000027609 | ENSOANG00000027609 | O_anatinus | Animals | mono_intergenic | H/ACA | | | intergenic |
| ENSOANG00000027611 | ENSOANG00000027611 | O_anatinus | Animals | mono_intronic | H/ACA | ENSOANG00000050686 | EIF4A1 | protein_coding |
| ENSOANG00000027614 | ENSOANG00000027614 | O_anatinus | Animals | mono_intergenic | H/ACA | | | intergenic |
| ENSOANG00000027686 | ENSOANG00000027686 | O_anatinus | Animals | mono_intergenic | H/ACA | | | intergenic |
| ENSOANG00000027730 | ENSOANG00000027730 | O_anatinus | Animals | mono_intronic | H/ACA | ENSOANG00000007451 | NAV3 | protein_coding |
| ENSOANG00000027751 | SNORD102 | O_anatinus | Animals | mono_intronic | C/D | ENSOANG00000009054 | | protein_coding |
| ENSOANG00000027765 | ENSOANG00000027765 | O_anatinus | Animals | mono_intergenic | H/ACA | | | intergenic |
| ENSOANG00000027827 | SNORD4B | O_anatinus | Animals | mono_intronic | C/D | ENSOANG00000009558 | RPL23A | protein_coding |
| ENSOANG00000027861 | ENSOANG00000027861 | O_anatinus | Animals | mono_intronic | H/ACA | ENSOANG00000013671 | ECT2 | protein_coding |
| ENSOANG00000027868 | ENSOANG00000027868 | O_anatinus | Animals | mono_intronic | H/ACA | ENSOANG00000008726 | | protein_coding |
| ENSOANG00000027906 | SNORD19B | O_anatinus | Animals | mono_intronic | C/D | ENSOANG00000013437 | GNL3 | protein_coding |
| ENSOANG00000027909 | ENSOANG00000027909 | O_anatinus | Animals | mono_intergenic | C/D | | | intergenic |
| ENSOANG00000027944 | ENSOANG00000027944 | O_anatinus | Animals | mono_intronic | C/D | ENSOANG00000002420 | NOP58 | protein_coding |
| ENSOANG00000027952 | ENSOANG00000027952 | O_anatinus | Animals | intergenic_cluster | C/D | | | intergenic |

| | | | | | | | | |
|---|---|---|---|---|---|---|---|---|
| ENSOANG00000027983 | SNORA47 | O_anatinus | Animals | mono_intergenic | H/ACA | | | intergenic |
| ENSOANG00000027984 | ENSOANG00000027984 | O_anatinus | Animals | mono_intergenic | H/ACA | | | intergenic |
| ENSOANG00000028044 | ENSOANG00000028044 | O_anatinus | Animals | mono_intronic | C/D | ENSOANG00000014755 | COX7C | protein_coding |
| ENSOANG00000028069 | ENSOANG00000028069 | O_anatinus | Animals | mono_intergenic | H/ACA | | | intergenic |
| ENSOANG00000028071 | SNORD123 | O_anatinus | Animals | mono_intergenic | C/D | | | intergenic |
| ENSOANG00000028074 | ENSOANG00000028074 | O_anatinus | Animals | mono_intergenic | H/ACA | | | intergenic |
| ENSOANG00000028079 | SNORD121A | O_anatinus | Animals | mono_intronic | C/D | ENSOANG00000010219 | UBAP2 | protein_coding |
| ENSOANG00000028106 | ENSOANG00000028106 | O_anatinus | Animals | mono_intergenic | H/ACA | | | intergenic |
| ENSOANG00000028108 | SNORD11B | O_anatinus | Animals | mono_intronic | C/D | ENSOANG00000002420 | NOP58 | protein_coding |
| ENSOANG00000028109 | ENSOANG00000028109 | O_anatinus | Animals | intergenic_cluster | C/D | | | intergenic |
| ENSOANG00000028150 | ENSOANG00000028150 | O_anatinus | Animals | mono_intergenic | C/D | | | intergenic |
| ENSOANG00000028153 | ENSOANG00000028153 | O_anatinus | Animals | mono_intronic | H/ACA | ENSOANG00000020508 | CDC16 | protein_coding |
| ENSOANG00000028167 | ENSOANG00000028167 | O_anatinus | Animals | mono_intronic | H/ACA | ENSOANG00000020955 | | protein_coding |
| ENSOANG00000028222 | ENSOANG00000028222 | O_anatinus | Animals | mono_intergenic | H/ACA | | | intergenic |
| ENSOANG00000028226 | SNORA84 | O_anatinus | Animals | mono_intronic | H/ACA | ENSOANG00000000112 | IARS1 | protein_coding |
| ENSOANG00000035979 | ENSOANG00000035979 | O_anatinus | Animals | mono_intergenic | H/ACA | | | intergenic |
| ENSOANG00000035980 | ENSOANG00000035980 | O_anatinus | Animals | mono_intergenic | H/ACA | | | intergenic |
| ENSOANG00000035983 | ENSOANG00000035983 | O_anatinus | Animals | mono_intergenic | H/ACA | | | intergenic |
| ENSOANG00000035986 | ENSOANG00000035986 | O_anatinus | Animals | mono_intergenic | H/ACA | | | intergenic |
| ENSOANG00000035987 | ENSOANG00000035987 | O_anatinus | Animals | mono_intergenic | H/ACA | | | intergenic |
| ENSOANG00000035988 | ENSOANG00000035988 | O_anatinus | Animals | mono_intergenic | H/ACA | | | intergenic |
| ENSOANG00000035993 | ENSOANG00000035993 | O_anatinus | Animals | mono_intronic | H/ACA | ENSOANG00000022364 | | protein_coding |
| ENSOANG00000035995 | ENSOANG00000035995 | O_anatinus | Animals | mono_intergenic | H/ACA | | | intergenic |
| ENSOANG00000035997 | ENSOANG00000035997 | O_anatinus | Animals | mono_intergenic | H/ACA | | | intergenic |
| ENSOANG00000035999 | ENSOANG00000035999 | O_anatinus | Animals | mono_intergenic | H/ACA | | | intergenic |
| ENSOANG00000036002 | ENSOANG00000036002 | O_anatinus | Animals | mono_intronic | H/ACA | ENSOANG00000008142 | PEPD | protein_coding |
| ENSOANG00000036009 | ENSOANG00000036009 | O_anatinus | Animals | mono_intergenic | H/ACA | | | intergenic |
| ENSOANG00000036010 | ENSOANG00000036010 | O_anatinus | Animals | mono_intergenic | H/ACA | | | intergenic |
| ENSOANG00000036011 | ENSOANG00000036011 | O_anatinus | Animals | mono_intergenic | H/ACA | | | intergenic |
| ENSOANG00000036020 | ENSOANG00000036020 | O_anatinus | Animals | mono_intergenic | H/ACA | | | intergenic |
| ENSOANG00000036024 | ENSOANG00000036024 | O_anatinus | Animals | mono_intergenic | H/ACA | | | intergenic |
| ENSOANG00000036026 | ENSOANG00000036026 | O_anatinus | Animals | mono_intergenic | H/ACA | | | intergenic |
| ENSOANG00000036027 | ENSOANG00000036027 | O_anatinus | Animals | mono_intergenic | H/ACA | | | intergenic |
| ENSOANG00000036028 | ENSOANG00000036028 | O_anatinus | Animals | mono_intergenic | H/ACA | | | intergenic |
| ENSOANG00000036029 | ENSOANG00000036029 | O_anatinus | Animals | mono_intergenic | H/ACA | | | intergenic |
| ENSOANG00000036030 | ENSOANG00000036030 | O_anatinus | Animals | mono_intergenic | H/ACA | | | intergenic |
| ENSOANG00000036035 | ENSOANG00000036035 | O_anatinus | Animals | mono_intergenic | H/ACA | | | intergenic |
| ENSOANG00000036036 | ENSOANG00000036036 | O_anatinus | Animals | mono_intergenic | H/ACA | | | intergenic |
| ENSOANG00000036038 | ENSOANG00000036038 | O_anatinus | Animals | mono_intergenic | H/ACA | | | intergenic |
| ENSOANG00000036040 | ENSOANG00000036040 | O_anatinus | Animals | mono_intergenic | H/ACA | | | intergenic |
| ENSOANG00000036042 | ENSOANG00000036042 | O_anatinus | Animals | mono_intergenic | H/ACA | | | intergenic |

| | | | | | | | | |
|---|---|---|---|---|---|---|---|---|
| ENSOANG00000036046 | ENSOANG00000036046 | O_anatinus | Animals | mono_intronic | H/ACA | ENSOANG00000008030 | SLC7A14 | protein_coding |
| ENSOANG00000036049 | ENSOANG00000036049 | O_anatinus | Animals | mono_intergenic | H/ACA | | | intergenic |
| ENSOANG00000036050 | ENSOANG00000036050 | O_anatinus | Animals | mono_intergenic | H/ACA | | | intergenic |
| ENSOANG00000036053 | ENSOANG00000036053 | O_anatinus | Animals | mono_intergenic | H/ACA | | | intergenic |
| ENSOANG00000036056 | ENSOANG00000036056 | O_anatinus | Animals | mono_intergenic | H/ACA | | | intergenic |
| ENSOANG00000036058 | ENSOANG00000036058 | O_anatinus | Animals | mono_intergenic | H/ACA | | | intergenic |
| ENSOANG00000036061 | ENSOANG00000036061 | O_anatinus | Animals | mono_intergenic | H/ACA | | | intergenic |
| ENSOANG00000036067 | ENSOANG00000036067 | O_anatinus | Animals | mono_intergenic | H/ACA | | | intergenic |
| ENSOANG00000036070 | ENSOANG00000036070 | O_anatinus | Animals | mono_intergenic | H/ACA | | | intergenic |
| ENSOANG00000036071 | ENSOANG00000036071 | O_anatinus | Animals | mono_intergenic | H/ACA | | | intergenic |
| ENSOANG00000036078 | ENSOANG00000036078 | O_anatinus | Animals | mono_intronic | H/ACA | ENSOANG00000006221 | CSRNP3 | protein_coding |
| ENSOANG00000036082 | ENSOANG00000036082 | O_anatinus | Animals | mono_intergenic | H/ACA | | | intergenic |
| ENSOANG00000036085 | ENSOANG00000036085 | O_anatinus | Animals | mono_intergenic | H/ACA | | | intergenic |
| ENSOANG00000036086 | ENSOANG00000036086 | O_anatinus | Animals | mono_intergenic | H/ACA | | | intergenic |
| ENSOANG00000036087 | ENSOANG00000036087 | O_anatinus | Animals | mono_intergenic | H/ACA | | | intergenic |
| ENSOANG00000036097 | ENSOANG00000036097 | O_anatinus | Animals | mono_intergenic | H/ACA | | | intergenic |
| ENSOANG00000036099 | ENSOANG00000036099 | O_anatinus | Animals | mono_intergenic | H/ACA | | | intergenic |
| ENSOANG00000036101 | ENSOANG00000036101 | O_anatinus | Animals | mono_intergenic | H/ACA | | | intergenic |
| ENSOANG00000036103 | ENSOANG00000036103 | O_anatinus | Animals | intronic_cluster | H/ACA | ENSOANG00000041495 | | protein_coding |
| ENSOANG00000036105 | ENSOANG00000036105 | O_anatinus | Animals | mono_intergenic | H/ACA | | | intergenic |
| ENSOANG00000036106 | ENSOANG00000036106 | O_anatinus | Animals | mono_intergenic | H/ACA | | | intergenic |
| ENSOANG00000036107 | ENSOANG00000036107 | O_anatinus | Animals | mono_intergenic | H/ACA | | | intergenic |
| ENSOANG00000036109 | ENSOANG00000036109 | O_anatinus | Animals | mono_intergenic | H/ACA | | | intergenic |
| ENSOANG00000036111 | ENSOANG00000036111 | O_anatinus | Animals | mono_intronic | H/ACA | ENSOANG00000022345 | | protein_coding |
| ENSOANG00000036112 | ENSOANG00000036112 | O_anatinus | Animals | mono_intergenic | H/ACA | | | intergenic |
| ENSOANG00000036117 | ENSOANG00000036117 | O_anatinus | Animals | mono_intergenic | H/ACA | | | intergenic |
| ENSOANG00000036123 | ENSOANG00000036123 | O_anatinus | Animals | mono_intergenic | H/ACA | | | intergenic |
| ENSOANG00000036125 | ENSOANG00000036125 | O_anatinus | Animals | mono_intergenic | H/ACA | | | intergenic |
| ENSOANG00000036129 | ENSOANG00000036129 | O_anatinus | Animals | mono_intergenic | H/ACA | | | intergenic |
| ENSOANG00000036130 | ENSOANG00000036130 | O_anatinus | Animals | mono_intergenic | H/ACA | | | intergenic |
| ENSOANG00000036143 | ENSOANG00000036143 | O_anatinus | Animals | mono_intergenic | H/ACA | | | intergenic |
| ENSOANG00000036146 | ENSOANG00000036146 | O_anatinus | Animals | mono_intergenic | H/ACA | | | intergenic |
| ENSOANG00000036147 | ENSOANG00000036147 | O_anatinus | Animals | mono_intergenic | H/ACA | | | intergenic |
| ENSOANG00000036152 | ENSOANG00000036152 | O_anatinus | Animals | mono_intergenic | H/ACA | | | intergenic |
| ENSOANG00000036158 | ENSOANG00000036158 | O_anatinus | Animals | mono_intergenic | H/ACA | | | intergenic |
| ENSOANG00000036160 | ENSOANG00000036160 | O_anatinus | Animals | mono_intergenic | H/ACA | | | intergenic |
| ENSOANG00000036163 | ENSOANG00000036163 | O_anatinus | Animals | mono_intergenic | H/ACA | | | intergenic |
| ENSOANG00000036164 | ENSOANG00000036164 | O_anatinus | Animals | mono_intergenic | H/ACA | | | intergenic |
| ENSOANG00000036165 | ENSOANG00000036165 | O_anatinus | Animals | mono_intergenic | H/ACA | | | intergenic |
| ENSOANG00000036169 | ENSOANG00000036169 | O_anatinus | Animals | mono_intergenic | H/ACA | | | intergenic |
| ENSOANG00000036172 | ENSOANG00000036172 | O_anatinus | Animals | mono_intronic | H/ACA | ENSOANG00000008672 | KCNG1 | protein_coding |

| | | | | | | | | |
|---|---|---|---|---|---|---|---|---|
| ENSOANG00000036174 | ENSOANG00000036174 | O_anatinus | Animals | mono_intergenic | H/ACA | | | intergenic |
| ENSOANG00000036175 | ENSOANG00000036175 | O_anatinus | Animals | mono_intergenic | H/ACA | | | intergenic |
| ENSOANG00000036176 | ENSOANG00000036176 | O_anatinus | Animals | mono_intergenic | H/ACA | | | intergenic |
| ENSOANG00000036178 | ENSOANG00000036178 | O_anatinus | Animals | mono_intergenic | H/ACA | | | intergenic |
| ENSOANG00000036179 | ENSOANG00000036179 | O_anatinus | Animals | mono_intergenic | H/ACA | | | intergenic |
| ENSOANG00000036182 | ENSOANG00000036182 | O_anatinus | Animals | mono_intergenic | H/ACA | | | intergenic |
| ENSOANG00000036185 | ENSOANG00000036185 | O_anatinus | Animals | mono_intergenic | H/ACA | | | intergenic |
| ENSOANG00000036186 | ENSOANG00000036186 | O_anatinus | Animals | mono_intergenic | H/ACA | | | intergenic |
| ENSOANG00000036190 | ENSOANG00000036190 | O_anatinus | Animals | mono_intergenic | H/ACA | | | intergenic |
| ENSOANG00000036192 | ENSOANG00000036192 | O_anatinus | Animals | mono_intronic | H/ACA | ENSOANG00000006419 | | protein_coding |
| ENSOANG00000036196 | ENSOANG00000036196 | O_anatinus | Animals | mono_intergenic | H/ACA | | | intergenic |
| ENSOANG00000036198 | ENSOANG00000036198 | O_anatinus | Animals | mono_intergenic | H/ACA | | | intergenic |
| ENSOANG00000036202 | ENSOANG00000036202 | O_anatinus | Animals | mono_intergenic | H/ACA | | | intergenic |
| ENSOANG00000036203 | ENSOANG00000036203 | O_anatinus | Animals | mono_intronic | H/ACA | ENSOANG00000015083 | | protein_coding |
| ENSOANG00000036205 | ENSOANG00000036205 | O_anatinus | Animals | mono_intergenic | H/ACA | | | intergenic |
| ENSOANG00000036206 | ENSOANG00000036206 | O_anatinus | Animals | mono_intronic | H/ACA | ENSOANG00000047662 | | protein_coding |
| ENSOANG00000036207 | ENSOANG00000036207 | O_anatinus | Animals | mono_intergenic | H/ACA | | | intergenic |
| ENSOANG00000036208 | ENSOANG00000036208 | O_anatinus | Animals | mono_intronic | H/ACA | ENSOANG00000041887 | PNPLA8 | protein_coding |
| ENSOANG00000036212 | ENSOANG00000036212 | O_anatinus | Animals | mono_intergenic | H/ACA | | | intergenic |
| ENSOANG00000036221 | ENSOANG00000036221 | O_anatinus | Animals | mono_intergenic | H/ACA | | | intergenic |
| ENSOANG00000036226 | ENSOANG00000036226 | O_anatinus | Animals | mono_intergenic | H/ACA | | | intergenic |
| ENSOANG00000036227 | ENSOANG00000036227 | O_anatinus | Animals | mono_intronic | H/ACA | ENSOANG00000050304 | | protein_coding |
| ENSOANG00000036229 | ENSOANG00000036229 | O_anatinus | Animals | mono_intergenic | H/ACA | | | intergenic |
| ENSOANG00000036230 | ENSOANG00000036230 | O_anatinus | Animals | mono_intergenic | H/ACA | | | intergenic |
| ENSOANG00000036234 | ENSOANG00000036234 | O_anatinus | Animals | mono_intergenic | H/ACA | | | intergenic |
| ENSOANG00000036235 | ENSOANG00000036235 | O_anatinus | Animals | mono_intergenic | H/ACA | | | intergenic |
| ENSOANG00000036236 | ENSOANG00000036236 | O_anatinus | Animals | mono_intergenic | H/ACA | | | intergenic |
| ENSOANG00000036244 | ENSOANG00000036244 | O_anatinus | Animals | mono_intergenic | H/ACA | | | intergenic |
| ENSOANG00000036246 | ENSOANG00000036246 | O_anatinus | Animals | mono_intergenic | H/ACA | | | intergenic |
| ENSOANG00000036247 | ENSOANG00000036247 | O_anatinus | Animals | mono_intergenic | H/ACA | | | intergenic |
| ENSOANG00000036249 | ENSOANG00000036249 | O_anatinus | Animals | mono_intergenic | H/ACA | | | intergenic |
| ENSOANG00000036250 | ENSOANG00000036250 | O_anatinus | Animals | mono_intergenic | H/ACA | | | intergenic |
| ENSOANG00000036252 | ENSOANG00000036252 | O_anatinus | Animals | mono_intergenic | H/ACA | | | intergenic |
| ENSOANG00000036253 | ENSOANG00000036253 | O_anatinus | Animals | mono_intergenic | H/ACA | | | intergenic |
| ENSOANG00000036254 | ENSOANG00000036254 | O_anatinus | Animals | mono_intergenic | H/ACA | | | intergenic |
| ENSOANG00000036256 | ENSOANG00000036256 | O_anatinus | Animals | mono_intergenic | H/ACA | | | intergenic |
| ENSOANG00000036258 | ENSOANG00000036258 | O_anatinus | Animals | mono_intronic | H/ACA | ENSOANG00000011500 | SYPL2 | protein_coding |
| ENSOANG00000036259 | ENSOANG00000036259 | O_anatinus | Animals | mono_intergenic | H/ACA | | | intergenic |
| ENSOANG00000036262 | ENSOANG00000036262 | O_anatinus | Animals | mono_intergenic | H/ACA | | | intergenic |
| ENSOANG00000036263 | ENSOANG00000036263 | O_anatinus | Animals | mono_intronic | H/ACA | ENSOANG00000009183 | | protein_coding |
| ENSOANG00000036264 | ENSOANG00000036264 | O_anatinus | Animals | mono_intergenic | H/ACA | | | intergenic |

| | | | | | | | | |
|---|---|---|---|---|---|---|---|---|
| ENSOANG00000036265 | ENSOANG00000036265 | O_anatinus | Animals | mono_intergenic | H/ACA | | | intergenic |
| ENSOANG00000036267 | ENSOANG00000036267 | O_anatinus | Animals | mono_intronic | H/ACA | ENSOANG00000005387 | RXFP1 | protein_coding |
| ENSOANG00000036268 | ENSOANG00000036268 | O_anatinus | Animals | mono_intergenic | H/ACA | | | intergenic |
| ENSOANG00000036269 | ENSOANG00000036269 | O_anatinus | Animals | mono_intronic | C/D | ENSOANG00000000829 | RPS11 | protein_coding |
| ENSOANG00000036270 | ENSOANG00000036270 | O_anatinus | Animals | mono_intergenic | H/ACA | | | intergenic |
| ENSOANG00000036272 | ENSOANG00000036272 | O_anatinus | Animals | mono_intergenic | H/ACA | | | intergenic |
| ENSOANG00000036273 | ENSOANG00000036273 | O_anatinus | Animals | mono_intergenic | H/ACA | | | intergenic |
| ENSOANG00000036275 | ENSOANG00000036275 | O_anatinus | Animals | mono_intergenic | H/ACA | | | intergenic |
| ENSOANG00000036279 | ENSOANG00000036279 | O_anatinus | Animals | mono_intergenic | H/ACA | | | intergenic |
| ENSOANG00000036280 | ENSOANG00000036280 | O_anatinus | Animals | mono_intergenic | H/ACA | | | intergenic |
| ENSOANG00000036282 | ENSOANG00000036282 | O_anatinus | Animals | mono_intergenic | H/ACA | | | intergenic |
| ENSOANG00000036283 | ENSOANG00000036283 | O_anatinus | Animals | mono_intergenic | H/ACA | | | intergenic |
| ENSOANG00000036284 | ENSOANG00000036284 | O_anatinus | Animals | mono_intergenic | H/ACA | | | intergenic |
| ENSOANG00000036285 | ENSOANG00000036285 | O_anatinus | Animals | mono_intergenic | H/ACA | | | intergenic |
| ENSOANG00000036288 | ENSOANG00000036288 | O_anatinus | Animals | mono_intergenic | H/ACA | | | intergenic |
| ENSOANG00000036289 | ENSOANG00000036289 | O_anatinus | Animals | mono_intergenic | H/ACA | | | intergenic |
| ENSOANG00000036291 | ENSOANG00000036291 | O_anatinus | Animals | mono_intergenic | H/ACA | | | intergenic |
| ENSOANG00000036292 | ENSOANG00000036292 | O_anatinus | Animals | mono_intergenic | H/ACA | | | intergenic |
| ENSOANG00000036303 | ENSOANG00000036303 | O_anatinus | Animals | mono_intergenic | H/ACA | | | intergenic |
| ENSOANG00000036304 | ENSOANG00000036304 | O_anatinus | Animals | mono_intronic | H/ACA | ENSOANG00000006998 | PRKAR1B | protein_coding |
| ENSOANG00000036309 | ENSOANG00000036309 | O_anatinus | Animals | mono_intergenic | H/ACA | | | intergenic |
| ENSOANG00000036310 | ENSOANG00000036310 | O_anatinus | Animals | mono_intergenic | H/ACA | | | intergenic |
| ENSOANG00000036312 | ENSOANG00000036312 | O_anatinus | Animals | mono_intergenic | H/ACA | | | intergenic |
| ENSOANG00000036316 | ENSOANG00000036316 | O_anatinus | Animals | mono_intergenic | H/ACA | | | intergenic |
| ENSOANG00000036318 | ENSOANG00000036318 | O_anatinus | Animals | mono_intergenic | H/ACA | | | intergenic |
| ENSOANG00000036322 | ENSOANG00000036322 | O_anatinus | Animals | mono_intergenic | H/ACA | | | intergenic |
| ENSOANG00000036329 | ENSOANG00000036329 | O_anatinus | Animals | mono_intergenic | H/ACA | | | intergenic |
| ENSOANG00000036330 | ENSOANG00000036330 | O_anatinus | Animals | mono_intergenic | H/ACA | | | intergenic |
| ENSOANG00000036332 | ENSOANG00000036332 | O_anatinus | Animals | mono_intergenic | H/ACA | | | intergenic |
| ENSOANG00000036334 | ENSOANG00000036334 | O_anatinus | Animals | mono_intergenic | H/ACA | | | intergenic |
| ENSOANG00000036335 | ENSOANG00000036335 | O_anatinus | Animals | mono_intergenic | H/ACA | | | intergenic |
| ENSOANG00000036336 | ENSOANG00000036336 | O_anatinus | Animals | mono_intergenic | H/ACA | | | intergenic |
| ENSOANG00000036337 | ENSOANG00000036337 | O_anatinus | Animals | mono_intergenic | H/ACA | | | intergenic |
| ENSOANG00000036344 | ENSOANG00000036344 | O_anatinus | Animals | mono_intergenic | C/D | | | intergenic |
| ENSOANG00000036350 | ENSOANG00000036350 | O_anatinus | Animals | mono_intergenic | H/ACA | | | intergenic |
| ENSOANG00000036354 | ENSOANG00000036354 | O_anatinus | Animals | mono_intergenic | H/ACA | | | intergenic |
| ENSOANG00000036358 | ENSOANG00000036358 | O_anatinus | Animals | mono_intronic | H/ACA | ENSOANG00000040886 | | non_coding |
| ENSOANG00000036359 | ENSOANG00000036359 | O_anatinus | Animals | mono_intronic | H/ACA | ENSOANG00000011076 | MEF2C | protein_coding |
| ENSOANG00000036360 | ENSOANG00000036360 | O_anatinus | Animals | mono_intergenic | H/ACA | | | intergenic |
| ENSOANG00000036362 | ENSOANG00000036362 | O_anatinus | Animals | mono_intergenic | H/ACA | | | intergenic |
| ENSOANG00000036363 | ENSOANG00000036363 | O_anatinus | Animals | mono_intergenic | H/ACA | | | intergenic |

| | | | | | | | | |
|---|---|---|---|---|---|---|---|---|
| ENSOANG00000036364 | ENSOANG00000036364 | O_anatinus | Animals | mono_intergenic | H/ACA | | | intergenic |
| ENSOANG00000036365 | ENSOANG00000036365 | O_anatinus | Animals | mono_intergenic | H/ACA | | | intergenic |
| ENSOANG00000036366 | ENSOANG00000036366 | O_anatinus | Animals | mono_intergenic | H/ACA | | | intergenic |
| ENSOANG00000036369 | ENSOANG00000036369 | O_anatinus | Animals | mono_intergenic | H/ACA | | | intergenic |
| ENSOANG00000036371 | ENSOANG00000036371 | O_anatinus | Animals | mono_intergenic | H/ACA | | | intergenic |
| ENSOANG00000036372 | ENSOANG00000036372 | O_anatinus | Animals | mono_intronic | H/ACA | ENSOANG00000045613 | F13A1 | protein_coding |
| ENSOANG00000036373 | ENSOANG00000036373 | O_anatinus | Animals | mono_intergenic | H/ACA | | | intergenic |
| ENSOANG00000036375 | ENSOANG00000036375 | O_anatinus | Animals | mono_intergenic | H/ACA | | | intergenic |
| ENSOANG00000036386 | ENSOANG00000036386 | O_anatinus | Animals | mono_intergenic | H/ACA | | | intergenic |
| ENSOANG00000036391 | ENSOANG00000036391 | O_anatinus | Animals | mono_intergenic | H/ACA | | | intergenic |
| ENSOANG00000036393 | ENSOANG00000036393 | O_anatinus | Animals | mono_intergenic | H/ACA | | | intergenic |
| ENSOANG00000036399 | ENSOANG00000036399 | O_anatinus | Animals | mono_intergenic | H/ACA | | | intergenic |
| ENSOANG00000036400 | ENSOANG00000036400 | O_anatinus | Animals | mono_intronic | H/ACA | ENSOANG00000033365 | | non_coding |
| ENSOANG00000036401 | ENSOANG00000036401 | O_anatinus | Animals | mono_intergenic | H/ACA | | | intergenic |
| ENSOANG00000036405 | ENSOANG00000036405 | O_anatinus | Animals | intronic_cluster | H/ACA | ENSOANG00000040015 | KCNMB2 | protein_coding |
| ENSOANG00000036406 | ENSOANG00000036406 | O_anatinus | Animals | mono_intergenic | H/ACA | | | intergenic |
| ENSOANG00000036412 | ENSOANG00000036412 | O_anatinus | Animals | mono_intergenic | H/ACA | | | intergenic |
| ENSOANG00000036413 | ENSOANG00000036413 | O_anatinus | Animals | mono_intergenic | H/ACA | | | intergenic |
| ENSOANG00000036414 | ENSOANG00000036414 | O_anatinus | Animals | mono_intergenic | H/ACA | | | intergenic |
| ENSOANG00000036415 | ENSOANG00000036415 | O_anatinus | Animals | mono_intergenic | H/ACA | | | intergenic |
| ENSOANG00000036417 | ENSOANG00000036417 | O_anatinus | Animals | mono_intronic | H/ACA | ENSOANG00000008493 | CERS6 | protein_coding |
| ENSOANG00000036418 | ENSOANG00000036418 | O_anatinus | Animals | mono_intronic | H/ACA | ENSOANG00000009322 | | protein_coding |
| ENSOANG00000036419 | ENSOANG00000036419 | O_anatinus | Animals | mono_intergenic | H/ACA | | | intergenic |
| ENSOANG00000036423 | ENSOANG00000036423 | O_anatinus | Animals | mono_intergenic | H/ACA | | | intergenic |
| ENSOANG00000036425 | ENSOANG00000036425 | O_anatinus | Animals | intronic_cluster | H/ACA | ENSOANG00000005417 | | protein_coding |
| ENSOANG00000036428 | ENSOANG00000036428 | O_anatinus | Animals | mono_intronic | H/ACA | ENSOANG00000008728 | GABRA3 | protein_coding |
| ENSOANG00000036432 | ENSOANG00000036432 | O_anatinus | Animals | mono_intergenic | H/ACA | | | intergenic |
| ENSOANG00000036437 | ENSOANG00000036437 | O_anatinus | Animals | mono_intergenic | H/ACA | | | intergenic |
| ENSOANG00000036438 | ENSOANG00000036438 | O_anatinus | Animals | mono_intergenic | H/ACA | | | intergenic |
| ENSOANG00000036439 | ENSOANG00000036439 | O_anatinus | Animals | mono_intergenic | H/ACA | | | intergenic |
| ENSOANG00000036442 | ENSOANG00000036442 | O_anatinus | Animals | mono_intergenic | H/ACA | | | intergenic |
| ENSOANG00000036445 | ENSOANG00000036445 | O_anatinus | Animals | mono_intergenic | H/ACA | | | intergenic |
| ENSOANG00000036452 | ENSOANG00000036452 | O_anatinus | Animals | mono_intergenic | H/ACA | | | intergenic |
| ENSOANG00000036453 | ENSOANG00000036453 | O_anatinus | Animals | intronic_cluster | H/ACA | ENSOANG00000013547 | PIAS1 | protein_coding |
| ENSOANG00000036454 | ENSOANG00000036454 | O_anatinus | Animals | mono_intergenic | H/ACA | | | intergenic |
| ENSOANG00000036458 | ENSOANG00000036458 | O_anatinus | Animals | mono_intergenic | H/ACA | | | intergenic |
| ENSOANG00000036459 | ENSOANG00000036459 | O_anatinus | Animals | mono_intergenic | H/ACA | | | intergenic |
| ENSOANG00000036460 | ENSOANG00000036460 | O_anatinus | Animals | mono_intergenic | H/ACA | | | intergenic |
| ENSOANG00000036462 | ENSOANG00000036462 | O_anatinus | Animals | mono_intergenic | H/ACA | | | intergenic |
| ENSOANG00000036466 | ENSOANG00000036466 | O_anatinus | Animals | mono_intergenic | H/ACA | | | intergenic |
| ENSOANG00000036468 | ENSOANG00000036468 | O_anatinus | Animals | mono_intergenic | H/ACA | | | intergenic |

| | | | | | | | | |
|---|---|---|---|---|---|---|---|---|
| ENSOANG00000036469 | ENSOANG00000036469 | O_anatinus | Animals | mono_intergenic | H/ACA | | | intergenic |
| ENSOANG00000036470 | ENSOANG00000036470 | O_anatinus | Animals | mono_intergenic | H/ACA | | | intergenic |
| ENSOANG00000036471 | ENSOANG00000036471 | O_anatinus | Animals | mono_intergenic | H/ACA | | | intergenic |
| ENSOANG00000036472 | ENSOANG00000036472 | O_anatinus | Animals | mono_intergenic | H/ACA | | | intergenic |
| ENSOANG00000036473 | ENSOANG00000036473 | O_anatinus | Animals | mono_intergenic | H/ACA | | | intergenic |
| ENSOANG00000036474 | ENSOANG00000036474 | O_anatinus | Animals | mono_intergenic | H/ACA | | | intergenic |
| ENSOANG00000036475 | ENSOANG00000036475 | O_anatinus | Animals | mono_intergenic | C/D | | | intergenic |
| ENSOANG00000036477 | ENSOANG00000036477 | O_anatinus | Animals | mono_intergenic | H/ACA | | | intergenic |
| ENSOANG00000036478 | ENSOANG00000036478 | O_anatinus | Animals | mono_intergenic | H/ACA | | | intergenic |
| ENSOANG00000036479 | ENSOANG00000036479 | O_anatinus | Animals | mono_intergenic | H/ACA | | | intergenic |
| ENSOANG00000036483 | ENSOANG00000036483 | O_anatinus | Animals | mono_intergenic | H/ACA | | | intergenic |
| ENSOANG00000036486 | ENSOANG00000036486 | O_anatinus | Animals | mono_intergenic | H/ACA | | | intergenic |
| ENSOANG00000036488 | ENSOANG00000036488 | O_anatinus | Animals | mono_intergenic | H/ACA | | | intergenic |
| ENSOANG00000036491 | ENSOANG00000036491 | O_anatinus | Animals | mono_intergenic | H/ACA | | | intergenic |
| ENSOANG00000036494 | ENSOANG00000036494 | O_anatinus | Animals | mono_intergenic | H/ACA | | | intergenic |
| ENSOANG00000036495 | ENSOANG00000036495 | O_anatinus | Animals | mono_intergenic | H/ACA | | | intergenic |
| ENSOANG00000036496 | ENSOANG00000036496 | O_anatinus | Animals | mono_intergenic | H/ACA | | | intergenic |
| ENSOANG00000036498 | ENSOANG00000036498 | O_anatinus | Animals | mono_intergenic | H/ACA | | | intergenic |
| ENSOANG00000036501 | ENSOANG00000036501 | O_anatinus | Animals | mono_intergenic | H/ACA | | | intergenic |
| ENSOANG00000036504 | ENSOANG00000036504 | O_anatinus | Animals | mono_intergenic | H/ACA | | | intergenic |
| ENSOANG00000036506 | ENSOANG00000036506 | O_anatinus | Animals | mono_intergenic | H/ACA | | | intergenic |
| ENSOANG00000036509 | ENSOANG00000036509 | O_anatinus | Animals | mono_intergenic | H/ACA | | | intergenic |
| ENSOANG00000036510 | ENSOANG00000036510 | O_anatinus | Animals | mono_intronic | H/ACA | ENSOANG00000004045 | EML1 | protein_coding |
| ENSOANG00000036513 | ENSOANG00000036513 | O_anatinus | Animals | mono_intronic | H/ACA | ENSOANG00000015522 | LRRC7 | protein_coding |
| ENSOANG00000036514 | ENSOANG00000036514 | O_anatinus | Animals | mono_intergenic | H/ACA | | | intergenic |
| ENSOANG00000036518 | ENSOANG00000036518 | O_anatinus | Animals | mono_intronic | H/ACA | ENSOANG00000049229 | PDGFD | protein_coding |
| ENSOANG00000036520 | ENSOANG00000036520 | O_anatinus | Animals | mono_intergenic | H/ACA | | | intergenic |
| ENSOANG00000036525 | ENSOANG00000036525 | O_anatinus | Animals | mono_intergenic | H/ACA | | | intergenic |
| ENSOANG00000036527 | ENSOANG00000036527 | O_anatinus | Animals | mono_intergenic | H/ACA | | | intergenic |
| ENSOANG00000036529 | ENSOANG00000036529 | O_anatinus | Animals | mono_intronic | H/ACA | ENSOANG00000029705 | CPQ | protein_coding |
| ENSOANG00000036532 | ENSOANG00000036532 | O_anatinus | Animals | mono_intergenic | H/ACA | | | intergenic |
| ENSOANG00000036534 | ENSOANG00000036534 | O_anatinus | Animals | mono_intergenic | H/ACA | | | intergenic |
| ENSOANG00000036540 | ENSOANG00000036540 | O_anatinus | Animals | mono_intergenic | H/ACA | | | intergenic |
| ENSOANG00000036544 | ENSOANG00000036544 | O_anatinus | Animals | mono_intergenic | H/ACA | | | intergenic |
| ENSOANG00000036546 | ENSOANG00000036546 | O_anatinus | Animals | mono_intergenic | H/ACA | | | intergenic |
| ENSOANG00000036547 | ENSOANG00000036547 | O_anatinus | Animals | mono_intergenic | H/ACA | | | intergenic |
| ENSOANG00000036549 | ENSOANG00000036549 | O_anatinus | Animals | mono_intergenic | H/ACA | | | intergenic |
| ENSOANG00000036551 | ENSOANG00000036551 | O_anatinus | Animals | mono_intergenic | H/ACA | | | intergenic |
| ENSOANG00000036556 | ENSOANG00000036556 | O_anatinus | Animals | mono_intronic | H/ACA | ENSOANG00000013198 | MED12L | protein_coding |
| ENSOANG00000036563 | ENSOANG00000036563 | O_anatinus | Animals | mono_intergenic | H/ACA | | | intergenic |
| ENSOANG00000036566 | ENSOANG00000036566 | O_anatinus | Animals | intronic_cluster | H/ACA | ENSOANG00000032096 | | protein_coding |

| | | | | | | | | |
|---|---|---|---|---|---|---|---|---|
| ENSOANG00000036570 | ENSOANG00000036570 | O_anatinus | Animals | mono_intergenic | H/ACA | | | intergenic |
| ENSOANG00000036571 | ENSOANG00000036571 | O_anatinus | Animals | mono_intronic | H/ACA | ENSOANG00000014859 | IGF2BP1 | protein_coding |
| ENSOANG00000036572 | ENSOANG00000036572 | O_anatinus | Animals | mono_intergenic | H/ACA | | | intergenic |
| ENSOANG00000036575 | ENSOANG00000036575 | O_anatinus | Animals | mono_intergenic | H/ACA | | | intergenic |
| ENSOANG00000036576 | ENSOANG00000036576 | O_anatinus | Animals | mono_intergenic | H/ACA | | | intergenic |
| ENSOANG00000036578 | ENSOANG00000036578 | O_anatinus | Animals | mono_intergenic | H/ACA | | | intergenic |
| ENSOANG00000036579 | ENSOANG00000036579 | O_anatinus | Animals | mono_intergenic | H/ACA | | | intergenic |
| ENSOANG00000036581 | ENSOANG00000036581 | O_anatinus | Animals | mono_intergenic | H/ACA | | | intergenic |
| ENSOANG00000036582 | ENSOANG00000036582 | O_anatinus | Animals | mono_intergenic | H/ACA | | | intergenic |
| ENSOANG00000036583 | ENSOANG00000036583 | O_anatinus | Animals | mono_intergenic | H/ACA | | | intergenic |
| ENSOANG00000036584 | ENSOANG00000036584 | O_anatinus | Animals | mono_intronic | H/ACA | ENSOANG00000038907 | BMP6 | protein_coding |
| ENSOANG00000036585 | ENSOANG00000036585 | O_anatinus | Animals | mono_intergenic | H/ACA | | | intergenic |
| ENSOANG00000036586 | ENSOANG00000036586 | O_anatinus | Animals | mono_intergenic | H/ACA | | | intergenic |
| ENSOANG00000036587 | ENSOANG00000036587 | O_anatinus | Animals | mono_intergenic | H/ACA | | | intergenic |
| ENSOANG00000036589 | ENSOANG00000036589 | O_anatinus | Animals | mono_intergenic | H/ACA | | | intergenic |
| ENSOANG00000036590 | ENSOANG00000036590 | O_anatinus | Animals | mono_intergenic | H/ACA | | | intergenic |
| ENSOANG00000036591 | ENSOANG00000036591 | O_anatinus | Animals | mono_intergenic | H/ACA | | | intergenic |
| ENSOANG00000036593 | ENSOANG00000036593 | O_anatinus | Animals | mono_intergenic | H/ACA | | | intergenic |
| ENSOANG00000036597 | ENSOANG00000036597 | O_anatinus | Animals | mono_intergenic | H/ACA | | | intergenic |
| ENSOANG00000036600 | ENSOANG00000036600 | O_anatinus | Animals | mono_intronic | H/ACA | ENSOANG00000015645 | GAS6 | protein_coding |
| ENSOANG00000036601 | ENSOANG00000036601 | O_anatinus | Animals | mono_intergenic | H/ACA | | | intergenic |
| ENSOANG00000036602 | ENSOANG00000036602 | O_anatinus | Animals | mono_intergenic | H/ACA | | | intergenic |
| ENSOANG00000036603 | ENSOANG00000036603 | O_anatinus | Animals | mono_intergenic | H/ACA | | | intergenic |
| ENSOANG00000036606 | ENSOANG00000036606 | O_anatinus | Animals | mono_intergenic | H/ACA | | | intergenic |
| ENSOANG00000036608 | ENSOANG00000036608 | O_anatinus | Animals | mono_intergenic | H/ACA | | | intergenic |
| ENSOANG00000036609 | ENSOANG00000036609 | O_anatinus | Animals | mono_intergenic | H/ACA | | | intergenic |
| ENSOANG00000036610 | ENSOANG00000036610 | O_anatinus | Animals | mono_intergenic | H/ACA | | | intergenic |
| ENSOANG00000036611 | ENSOANG00000036611 | O_anatinus | Animals | mono_intergenic | H/ACA | | | intergenic |
| ENSOANG00000036616 | ENSOANG00000036616 | O_anatinus | Animals | mono_intronic | H/ACA | ENSOANG00000039591 | TMED5 | protein_coding |
| ENSOANG00000036618 | ENSOANG00000036618 | O_anatinus | Animals | mono_intronic | H/ACA | ENSOANG00000046365 | | non_coding |
| ENSOANG00000036620 | ENSOANG00000036620 | O_anatinus | Animals | mono_intergenic | H/ACA | | | intergenic |
| ENSOANG00000036624 | ENSOANG00000036624 | O_anatinus | Animals | mono_intergenic | H/ACA | | | intergenic |
| ENSOANG00000036625 | ENSOANG00000036625 | O_anatinus | Animals | intronic_cluster | H/ACA | ENSOANG00000045478 | LRMDA | protein_coding |
| ENSOANG00000036626 | ENSOANG00000036626 | O_anatinus | Animals | mono_intergenic | H/ACA | | | intergenic |
| ENSOANG00000036628 | ENSOANG00000036628 | O_anatinus | Animals | mono_intergenic | H/ACA | | | intergenic |
| ENSOANG00000036632 | ENSOANG00000036632 | O_anatinus | Animals | mono_intergenic | H/ACA | | | intergenic |
| ENSOANG00000036635 | ENSOANG00000036635 | O_anatinus | Animals | mono_intergenic | H/ACA | | | intergenic |
| ENSOANG00000036640 | ENSOANG00000036640 | O_anatinus | Animals | mono_intergenic | H/ACA | | | intergenic |
| ENSOANG00000036644 | ENSOANG00000036644 | O_anatinus | Animals | mono_intergenic | H/ACA | | | intergenic |
| ENSOANG00000036645 | ENSOANG00000036645 | O_anatinus | Animals | mono_intronic | H/ACA | ENSOANG00000007817 | TENM2 | protein_coding |
| ENSOANG00000036649 | ENSOANG00000036649 | O_anatinus | Animals | mono_intergenic | H/ACA | | | intergenic |

| | | | | | | | | |
|---|---|---|---|---|---|---|---|---|
| ENSOANG00000036650 | ENSOANG00000036650 | O_anatinus | Animals | intronic_cluster | H/ACA | ENSOANG00000012559 | | protein_coding |
| ENSOANG00000036651 | ENSOANG00000036651 | O_anatinus | Animals | mono_intergenic | H/ACA | | | intergenic |
| ENSOANG00000036659 | ENSOANG00000036659 | O_anatinus | Animals | mono_intergenic | H/ACA | | | intergenic |
| ENSOANG00000036662 | ENSOANG00000036662 | O_anatinus | Animals | mono_intergenic | H/ACA | | | intergenic |
| ENSOANG00000036666 | SNORD30 | O_anatinus | Animals | intergenic_cluster | C/D | | | intergenic |
| ENSOANG00000036671 | ENSOANG00000036671 | O_anatinus | Animals | mono_intergenic | H/ACA | | | intergenic |
| ENSOANG00000036672 | ENSOANG00000036672 | O_anatinus | Animals | mono_intergenic | H/ACA | | | intergenic |
| ENSOANG00000036673 | ENSOANG00000036673 | O_anatinus | Animals | mono_intergenic | H/ACA | | | intergenic |
| ENSOANG00000036674 | ENSOANG00000036674 | O_anatinus | Animals | mono_intergenic | H/ACA | | | intergenic |
| ENSOANG00000036675 | ENSOANG00000036675 | O_anatinus | Animals | mono_intergenic | H/ACA | | | intergenic |
| ENSOANG00000036680 | ENSOANG00000036680 | O_anatinus | Animals | mono_intergenic | H/ACA | | | intergenic |
| ENSOANG00000036683 | ENSOANG00000036683 | O_anatinus | Animals | mono_intergenic | H/ACA | | | intergenic |
| ENSOANG00000036686 | ENSOANG00000036686 | O_anatinus | Animals | mono_intergenic | H/ACA | | | intergenic |
| ENSOANG00000036689 | ENSOANG00000036689 | O_anatinus | Animals | intronic_cluster | H/ACA | ENSOANG00000049189 | UGCG | protein_coding |
| ENSOANG00000036692 | ENSOANG00000036692 | O_anatinus | Animals | mono_intergenic | H/ACA | | | intergenic |
| ENSOANG00000036698 | ENSOANG00000036698 | O_anatinus | Animals | mono_intergenic | H/ACA | | | intergenic |
| ENSOANG00000036699 | ENSOANG00000036699 | O_anatinus | Animals | mono_intergenic | H/ACA | | | intergenic |
| ENSOANG00000036704 | ENSOANG00000036704 | O_anatinus | Animals | mono_intergenic | H/ACA | | | intergenic |
| ENSOANG00000036712 | ENSOANG00000036712 | O_anatinus | Animals | mono_intergenic | H/ACA | | | intergenic |
| ENSOANG00000036717 | SNORA62 | O_anatinus | Animals | mono_intronic | H/ACA | ENSOANG00000007053 | | protein_coding |
| ENSOANG00000036719 | ENSOANG00000036719 | O_anatinus | Animals | mono_intergenic | H/ACA | | | intergenic |
| ENSOANG00000036722 | ENSOANG00000036722 | O_anatinus | Animals | mono_intronic | H/ACA | ENSOANG00000010028 | PIBF1 | protein_coding |
| ENSOANG00000036723 | ENSOANG00000036723 | O_anatinus | Animals | mono_intergenic | H/ACA | | | intergenic |
| ENSOANG00000036725 | ENSOANG00000036725 | O_anatinus | Animals | mono_intergenic | H/ACA | | | intergenic |
| ENSOANG00000036727 | ENSOANG00000036727 | O_anatinus | Animals | mono_intergenic | H/ACA | | | intergenic |
| ENSOANG00000036732 | ENSOANG00000036732 | O_anatinus | Animals | mono_intronic | H/ACA | ENSOANG00000036747 | VPS8 | protein_coding |
| ENSOANG00000036734 | ENSOANG00000036734 | O_anatinus | Animals | mono_intronic | H/ACA | ENSOANG00000013271 | VPS13B | protein_coding |
| ENSOANG00000036735 | ENSOANG00000036735 | O_anatinus | Animals | mono_intergenic | H/ACA | | | intergenic |
| ENSOANG00000036737 | ENSOANG00000036737 | O_anatinus | Animals | mono_intergenic | H/ACA | | | intergenic |
| ENSOANG00000036740 | ENSOANG00000036740 | O_anatinus | Animals | mono_intergenic | H/ACA | | | intergenic |
| ENSOANG00000036741 | ENSOANG00000036741 | O_anatinus | Animals | mono_intergenic | H/ACA | | | intergenic |
| ENSOANG00000036742 | SNORA73 | O_anatinus | Animals | intergenic_cluster | H/ACA | | | intergenic |
| ENSOANG00000036744 | ENSOANG00000036744 | O_anatinus | Animals | mono_intergenic | H/ACA | | | intergenic |
| ENSOANG00000036748 | ENSOANG00000036748 | O_anatinus | Animals | mono_intergenic | H/ACA | | | intergenic |
| ENSOANG00000036761 | ENSOANG00000036761 | O_anatinus | Animals | mono_intergenic | H/ACA | | | intergenic |
| ENSOANG00000036762 | ENSOANG00000036762 | O_anatinus | Animals | mono_intergenic | H/ACA | | | intergenic |
| ENSOANG00000036764 | ENSOANG00000036764 | O_anatinus | Animals | mono_intergenic | H/ACA | | | intergenic |
| ENSOANG00000036769 | ENSOANG00000036769 | O_anatinus | Animals | mono_intergenic | H/ACA | | | intergenic |
| ENSOANG00000036770 | ENSOANG00000036770 | O_anatinus | Animals | mono_intergenic | H/ACA | | | intergenic |
| ENSOANG00000036771 | ENSOANG00000036771 | O_anatinus | Animals | mono_intergenic | H/ACA | | | intergenic |
| ENSOANG00000036773 | ENSOANG00000036773 | O_anatinus | Animals | mono_intergenic | H/ACA | | | intergenic |

| | | | | | | | | |
|---|---|---|---|---|---|---|---|---|
| ENSOANG00000036775 | ENSOANG00000036775 | O_anatinus | Animals | mono_intergenic | H/ACA | | | intergenic |
| ENSOANG00000036776 | ENSOANG00000036776 | O_anatinus | Animals | mono_intergenic | H/ACA | | | intergenic |
| ENSOANG00000036779 | ENSOANG00000036779 | O_anatinus | Animals | mono_intergenic | H/ACA | | | intergenic |
| ENSOANG00000036781 | ENSOANG00000036781 | O_anatinus | Animals | mono_intergenic | H/ACA | | | intergenic |
| ENSOANG00000036783 | ENSOANG00000036783 | O_anatinus | Animals | mono_intergenic | H/ACA | | | intergenic |
| ENSOANG00000036786 | ENSOANG00000036786 | O_anatinus | Animals | mono_intergenic | H/ACA | | | intergenic |
| ENSOANG00000036787 | ENSOANG00000036787 | O_anatinus | Animals | mono_intronic | C/D | ENSOANG00000012383 | PTCD3 | protein_coding |
| ENSOANG00000036792 | ENSOANG00000036792 | O_anatinus | Animals | mono_intergenic | H/ACA | | | intergenic |
| ENSOANG00000036794 | ENSOANG00000036794 | O_anatinus | Animals | mono_intergenic | H/ACA | | | intergenic |
| ENSOANG00000036795 | ENSOANG00000036795 | O_anatinus | Animals | mono_intergenic | H/ACA | | | intergenic |
| ENSOANG00000036800 | ENSOANG00000036800 | O_anatinus | Animals | mono_intergenic | H/ACA | | | intergenic |
| ENSOANG00000036805 | ENSOANG00000036805 | O_anatinus | Animals | mono_intergenic | H/ACA | | | intergenic |
| ENSOANG00000036806 | ENSOANG00000036806 | O_anatinus | Animals | mono_intergenic | H/ACA | | | intergenic |
| ENSOANG00000036807 | ENSOANG00000036807 | O_anatinus | Animals | mono_intergenic | H/ACA | | | intergenic |
| ENSOANG00000036810 | ENSOANG00000036810 | O_anatinus | Animals | mono_intergenic | H/ACA | | | intergenic |
| ENSOANG00000036811 | ENSOANG00000036811 | O_anatinus | Animals | mono_intergenic | H/ACA | | | intergenic |
| ENSOANG00000036813 | ENSOANG00000036813 | O_anatinus | Animals | mono_intergenic | H/ACA | | | intergenic |
| ENSOANG00000036815 | ENSOANG00000036815 | O_anatinus | Animals | mono_intronic | H/ACA | ENSOANG00000046982 | DOK6 | protein_coding |
| ENSOANG00000036816 | ENSOANG00000036816 | O_anatinus | Animals | mono_intronic | H/ACA | ENSOANG00000049143 | | non_coding |
| ENSOANG00000036817 | ENSOANG00000036817 | O_anatinus | Animals | intronic_cluster | H/ACA | ENSOANG00000044098 | | protein_coding |
| ENSOANG00000036822 | ENSOANG00000036822 | O_anatinus | Animals | mono_intergenic | H/ACA | | | intergenic |
| ENSOANG00000036823 | ENSOANG00000036823 | O_anatinus | Animals | mono_intergenic | H/ACA | | | intergenic |
| ENSOANG00000036825 | ENSOANG00000036825 | O_anatinus | Animals | mono_intergenic | H/ACA | | | intergenic |
| ENSOANG00000036829 | ENSOANG00000036829 | O_anatinus | Animals | mono_intergenic | H/ACA | | | intergenic |
| ENSOANG00000036837 | ENSOANG00000036837 | O_anatinus | Animals | mono_intergenic | H/ACA | | | intergenic |
| ENSOANG00000036838 | ENSOANG00000036838 | O_anatinus | Animals | mono_intergenic | H/ACA | | | intergenic |
| ENSOANG00000036839 | ENSOANG00000036839 | O_anatinus | Animals | mono_intergenic | H/ACA | | | intergenic |
| ENSOANG00000036844 | ENSOANG00000036844 | O_anatinus | Animals | mono_intergenic | H/ACA | | | intergenic |
| ENSOANG00000036845 | ENSOANG00000036845 | O_anatinus | Animals | mono_intergenic | H/ACA | | | intergenic |
| ENSOANG00000036847 | ENSOANG00000036847 | O_anatinus | Animals | mono_intergenic | H/ACA | | | intergenic |
| ENSOANG00000036848 | ENSOANG00000036848 | O_anatinus | Animals | mono_intergenic | H/ACA | | | intergenic |
| ENSOANG00000036850 | ENSOANG00000036850 | O_anatinus | Animals | mono_intergenic | H/ACA | | | intergenic |
| ENSOANG00000036853 | ENSOANG00000036853 | O_anatinus | Animals | mono_intergenic | H/ACA | | | intergenic |
| ENSOANG00000036854 | ENSOANG00000036854 | O_anatinus | Animals | mono_intergenic | H/ACA | | | intergenic |
| ENSOANG00000036857 | ENSOANG00000036857 | O_anatinus | Animals | mono_intergenic | H/ACA | | | intergenic |
| ENSOANG00000036860 | ENSOANG00000036860 | O_anatinus | Animals | mono_intergenic | H/ACA | | | intergenic |
| ENSOANG00000036862 | ENSOANG00000036862 | O_anatinus | Animals | mono_intergenic | H/ACA | | | intergenic |
| ENSOANG00000036863 | ENSOANG00000036863 | O_anatinus | Animals | mono_intergenic | H/ACA | | | intergenic |
| ENSOANG00000036869 | ENSOANG00000036869 | O_anatinus | Animals | mono_intergenic | H/ACA | | | intergenic |
| ENSOANG00000036872 | ENSOANG00000036872 | O_anatinus | Animals | mono_intergenic | H/ACA | | | intergenic |
| ENSOANG00000036873 | ENSOANG00000036873 | O_anatinus | Animals | mono_intergenic | H/ACA | | | intergenic |

| | | | | | | | | |
|---|---|---|---|---|---|---|---|---|
| ENSOANG00000036876 | ENSOANG00000036876 | O_anatinus | Animals | mono_intergenic | H/ACA | | | intergenic |
| ENSOANG00000036877 | ENSOANG00000036877 | O_anatinus | Animals | mono_intergenic | H/ACA | | | intergenic |
| ENSOANG00000036878 | ENSOANG00000036878 | O_anatinus | Animals | mono_intronic | H/ACA | ENSOANG00000005539 | ZNF618 | protein_coding |
| ENSOANG00000036879 | ENSOANG00000036879 | O_anatinus | Animals | mono_intergenic | H/ACA | | | intergenic |
| ENSOANG00000036883 | ENSOANG00000036883 | O_anatinus | Animals | mono_intergenic | H/ACA | | | intergenic |
| ENSOANG00000036884 | ENSOANG00000036884 | O_anatinus | Animals | intergenic_cluster | H/ACA | | | intergenic |
| ENSOANG00000036885 | ENSOANG00000036885 | O_anatinus | Animals | mono_intergenic | H/ACA | | | intergenic |
| ENSOANG00000036886 | ENSOANG00000036886 | O_anatinus | Animals | mono_intergenic | H/ACA | | | intergenic |
| ENSOANG00000036887 | ENSOANG00000036887 | O_anatinus | Animals | mono_intergenic | H/ACA | | | intergenic |
| ENSOANG00000036889 | ENSOANG00000036889 | O_anatinus | Animals | mono_intergenic | H/ACA | | | intergenic |
| ENSOANG00000036891 | ENSOANG00000036891 | O_anatinus | Animals | mono_intergenic | H/ACA | | | intergenic |
| ENSOANG00000036892 | ENSOANG00000036892 | O_anatinus | Animals | mono_intergenic | H/ACA | | | intergenic |
| ENSOANG00000036893 | ENSOANG00000036893 | O_anatinus | Animals | mono_intergenic | H/ACA | | | intergenic |
| ENSOANG00000036895 | ENSOANG00000036895 | O_anatinus | Animals | mono_intergenic | H/ACA | | | intergenic |
| ENSOANG00000036899 | ENSOANG00000036899 | O_anatinus | Animals | mono_intergenic | H/ACA | | | intergenic |
| ENSOANG00000036900 | ENSOANG00000036900 | O_anatinus | Animals | mono_intergenic | H/ACA | | | intergenic |
| ENSOANG00000036904 | ENSOANG00000036904 | O_anatinus | Animals | mono_intergenic | H/ACA | | | intergenic |
| ENSOANG00000036906 | ENSOANG00000036906 | O_anatinus | Animals | mono_intergenic | H/ACA | | | intergenic |
| ENSOANG00000036907 | ENSOANG00000036907 | O_anatinus | Animals | mono_intergenic | H/ACA | | | intergenic |
| ENSOANG00000036908 | ENSOANG00000036908 | O_anatinus | Animals | mono_intergenic | H/ACA | | | intergenic |
| ENSOANG00000036909 | ENSOANG00000036909 | O_anatinus | Animals | mono_intergenic | H/ACA | | | intergenic |
| ENSOANG00000036912 | ENSOANG00000036912 | O_anatinus | Animals | mono_intergenic | H/ACA | | | intergenic |
| ENSOANG00000036922 | ENSOANG00000036922 | O_anatinus | Animals | mono_intergenic | H/ACA | | | intergenic |
| ENSOANG00000036926 | ENSOANG00000036926 | O_anatinus | Animals | mono_intergenic | C/D | | | intergenic |
| ENSOANG00000036927 | ENSOANG00000036927 | O_anatinus | Animals | mono_intergenic | H/ACA | | | intergenic |
| ENSOANG00000036928 | ENSOANG00000036928 | O_anatinus | Animals | mono_intergenic | H/ACA | | | intergenic |
| ENSOANG00000036931 | ENSOANG00000036931 | O_anatinus | Animals | mono_intronic | H/ACA | ENSOANG00000002405 | PTPRM | protein_coding |
| ENSOANG00000036933 | ENSOANG00000036933 | O_anatinus | Animals | mono_intergenic | H/ACA | | | intergenic |
| ENSOANG00000036934 | ENSOANG00000036934 | O_anatinus | Animals | mono_intergenic | H/ACA | | | intergenic |
| ENSOANG00000036942 | ENSOANG00000036942 | O_anatinus | Animals | mono_intergenic | H/ACA | | | intergenic |
| ENSOANG00000036943 | ENSOANG00000036943 | O_anatinus | Animals | mono_intergenic | H/ACA | | | intergenic |
| ENSOANG00000036948 | ENSOANG00000036948 | O_anatinus | Animals | mono_intergenic | H/ACA | | | intergenic |
| ENSOANG00000036949 | ENSOANG00000036949 | O_anatinus | Animals | mono_intergenic | H/ACA | | | intergenic |
| ENSOANG00000036951 | ENSOANG00000036951 | O_anatinus | Animals | mono_intergenic | H/ACA | | | intergenic |
| ENSOANG00000036953 | ENSOANG00000036953 | O_anatinus | Animals | mono_intergenic | H/ACA | | | intergenic |
| ENSOANG00000036954 | ENSOANG00000036954 | O_anatinus | Animals | mono_intergenic | H/ACA | | | intergenic |
| ENSOANG00000036955 | ENSOANG00000036955 | O_anatinus | Animals | mono_intergenic | H/ACA | | | intergenic |
| ENSOANG00000036962 | ENSOANG00000036962 | O_anatinus | Animals | mono_intergenic | H/ACA | | | intergenic |
| ENSOANG00000036964 | ENSOANG00000036964 | O_anatinus | Animals | mono_intergenic | H/ACA | | | intergenic |
| ENSOANG00000036965 | ENSOANG00000036965 | O_anatinus | Animals | mono_intergenic | H/ACA | | | intergenic |
| ENSOANG00000036966 | ENSOANG00000036966 | O_anatinus | Animals | mono_intergenic | H/ACA | | | intergenic |

| | | | | | | | | |
|---|---|---|---|---|---|---|---|---|
| ENSOANG00000036968 | ENSOANG00000036968 | O_anatinus | Animals | mono_intergenic | H/ACA | | | intergenic |
| ENSOANG00000036969 | ENSOANG00000036969 | O_anatinus | Animals | mono_intergenic | H/ACA | | | intergenic |
| ENSOANG00000036972 | ENSOANG00000036972 | O_anatinus | Animals | mono_intergenic | H/ACA | | | intergenic |
| ENSOANG00000036973 | ENSOANG00000036973 | O_anatinus | Animals | mono_intergenic | H/ACA | | | intergenic |
| ENSOANG00000036974 | ENSOANG00000036974 | O_anatinus | Animals | mono_intergenic | H/ACA | | | intergenic |
| ENSOANG00000036981 | ENSOANG00000036981 | O_anatinus | Animals | mono_intergenic | H/ACA | | | intergenic |
| ENSOANG00000036982 | ENSOANG00000036982 | O_anatinus | Animals | mono_intergenic | H/ACA | | | intergenic |
| ENSOANG00000036984 | ENSOANG00000036984 | O_anatinus | Animals | mono_intergenic | H/ACA | | | intergenic |
| ENSOANG00000036985 | ENSOANG00000036985 | O_anatinus | Animals | mono_intergenic | H/ACA | | | intergenic |
| ENSOANG00000036988 | ENSOANG00000036988 | O_anatinus | Animals | mono_intergenic | H/ACA | | | intergenic |
| ENSOANG00000036990 | ENSOANG00000036990 | O_anatinus | Animals | mono_intergenic | H/ACA | | | intergenic |
| ENSOANG00000036991 | ENSOANG00000036991 | O_anatinus | Animals | mono_intergenic | H/ACA | | | intergenic |
| ENSOANG00000036993 | ENSOANG00000036993 | O_anatinus | Animals | mono_intergenic | H/ACA | | | intergenic |
| ENSOANG00000036994 | ENSOANG00000036994 | O_anatinus | Animals | mono_intronic | C/D | ENSOANG00000050134 | | non_coding |
| ENSOANG00000036996 | ENSOANG00000036996 | O_anatinus | Animals | mono_intergenic | H/ACA | | | intergenic |
| ENSOANG00000036997 | ENSOANG00000036997 | O_anatinus | Animals | mono_intergenic | H/ACA | | | intergenic |
| ENSOANG00000037000 | ENSOANG00000037000 | O_anatinus | Animals | mono_intergenic | H/ACA | | | intergenic |
| ENSOANG00000037002 | ENSOANG00000037002 | O_anatinus | Animals | mono_intergenic | H/ACA | | | intergenic |
| ENSOANG00000037009 | ENSOANG00000037009 | O_anatinus | Animals | mono_intergenic | H/ACA | | | intergenic |
| ENSOANG00000037014 | ENSOANG00000037014 | O_anatinus | Animals | mono_intergenic | H/ACA | | | intergenic |
| ENSOANG00000037016 | ENSOANG00000037016 | O_anatinus | Animals | mono_intergenic | H/ACA | | | intergenic |
| ENSOANG00000037017 | ENSOANG00000037017 | O_anatinus | Animals | mono_intergenic | H/ACA | | | intergenic |
| ENSOANG00000037020 | ENSOANG00000037020 | O_anatinus | Animals | mono_intergenic | H/ACA | | | intergenic |
| ENSOANG00000037023 | ENSOANG00000037023 | O_anatinus | Animals | mono_intergenic | H/ACA | | | intergenic |
| ENSOANG00000037027 | SNORD14 | O_anatinus | Animals | mono_intronic | C/D | ENSOANG00000014580 | HSPA8 | protein_coding |
| ENSOANG00000037031 | ENSOANG00000037031 | O_anatinus | Animals | mono_intergenic | H/ACA | | | intergenic |
| ENSOANG00000037032 | ENSOANG00000037032 | O_anatinus | Animals | mono_intergenic | H/ACA | | | intergenic |
| ENSOANG00000037035 | ENSOANG00000037035 | O_anatinus | Animals | mono_intergenic | H/ACA | | | intergenic |
| ENSOANG00000037037 | ENSOANG00000037037 | O_anatinus | Animals | mono_intergenic | H/ACA | | | intergenic |
| ENSOANG00000037041 | ENSOANG00000037041 | O_anatinus | Animals | mono_intergenic | H/ACA | | | intergenic |
| ENSOANG00000037042 | ENSOANG00000037042 | O_anatinus | Animals | mono_intergenic | H/ACA | | | intergenic |
| ENSOANG00000037043 | ENSOANG00000037043 | O_anatinus | Animals | mono_intergenic | H/ACA | | | intergenic |
| ENSOANG00000037046 | ENSOANG00000037046 | O_anatinus | Animals | mono_intergenic | H/ACA | | | intergenic |
| ENSOANG00000037047 | ENSOANG00000037047 | O_anatinus | Animals | mono_intergenic | H/ACA | | | intergenic |
| ENSOANG00000037051 | ENSOANG00000037051 | O_anatinus | Animals | mono_intronic | H/ACA | ENSOANG00000031345 | LDLRAD3 | protein_coding |
| ENSOANG00000037053 | ENSOANG00000037053 | O_anatinus | Animals | mono_intergenic | H/ACA | | | intergenic |
| ENSOANG00000037054 | ENSOANG00000037054 | O_anatinus | Animals | mono_intergenic | H/ACA | | | intergenic |
| ENSOANG00000037055 | ENSOANG00000037055 | O_anatinus | Animals | mono_intergenic | H/ACA | | | intergenic |
| ENSOANG00000037057 | ENSOANG00000037057 | O_anatinus | Animals | mono_intronic | H/ACA | ENSOANG00000044098 | | protein_coding |
| ENSOANG00000037059 | ENSOANG00000037059 | O_anatinus | Animals | mono_intergenic | H/ACA | | | intergenic |
| ENSOANG00000037062 | ENSOANG00000037062 | O_anatinus | Animals | mono_intergenic | H/ACA | | | intergenic |

| | | | | | | | | |
|---|---|---|---|---|---|---|---|---|
| ENSOANG00000037063 | ENSOANG00000037063 | O_anatinus | Animals | mono_intergenic | H/ACA | | | intergenic |
| ENSOANG00000037064 | ENSOANG00000037064 | O_anatinus | Animals | mono_intergenic | H/ACA | | | intergenic |
| ENSOANG00000037066 | ENSOANG00000037066 | O_anatinus | Animals | mono_intergenic | H/ACA | | | intergenic |
| ENSOANG00000037067 | ENSOANG00000037067 | O_anatinus | Animals | mono_intergenic | H/ACA | | | intergenic |
| ENSOANG00000037068 | ENSOANG00000037068 | O_anatinus | Animals | mono_intergenic | H/ACA | | | intergenic |
| ENSOANG00000037070 | ENSOANG00000037070 | O_anatinus | Animals | mono_intergenic | H/ACA | | | intergenic |
| ENSOANG00000037071 | ENSOANG00000037071 | O_anatinus | Animals | mono_intergenic | H/ACA | | | intergenic |
| ENSOANG00000037074 | ENSOANG00000037074 | O_anatinus | Animals | mono_intergenic | H/ACA | | | intergenic |
| ENSOANG00000037082 | ENSOANG00000037082 | O_anatinus | Animals | mono_intergenic | H/ACA | | | intergenic |
| ENSOANG00000037083 | ENSOANG00000037083 | O_anatinus | Animals | mono_intergenic | H/ACA | | | intergenic |
| ENSOANG00000037084 | ENSOANG00000037084 | O_anatinus | Animals | mono_intergenic | H/ACA | | | intergenic |
| ENSOANG00000037086 | ENSOANG00000037086 | O_anatinus | Animals | mono_intergenic | H/ACA | | | intergenic |
| ENSOANG00000037090 | ENSOANG00000037090 | O_anatinus | Animals | mono_intronic | H/ACA | ENSOANG00000041489 | SLIT1 | protein_coding |
| ENSOANG00000037091 | ENSOANG00000037091 | O_anatinus | Animals | intronic_cluster | H/ACA | ENSOANG00000041495 | | protein_coding |
| ENSOANG00000037093 | ENSOANG00000037093 | O_anatinus | Animals | mono_intronic | H/ACA | ENSOANG00000012119 | | protein_coding |
| ENSOANG00000037095 | ENSOANG00000037095 | O_anatinus | Animals | mono_intergenic | H/ACA | | | intergenic |
| ENSOANG00000037096 | ENSOANG00000037096 | O_anatinus | Animals | mono_intergenic | H/ACA | | | intergenic |
| ENSOANG00000037101 | ENSOANG00000037101 | O_anatinus | Animals | mono_intergenic | H/ACA | | | intergenic |
| ENSOANG00000037106 | ENSOANG00000037106 | O_anatinus | Animals | mono_intergenic | H/ACA | | | intergenic |
| ENSOANG00000037107 | ENSOANG00000037107 | O_anatinus | Animals | mono_intergenic | H/ACA | | | intergenic |
| ENSOANG00000037108 | ENSOANG00000037108 | O_anatinus | Animals | mono_intergenic | H/ACA | | | intergenic |
| ENSOANG00000037111 | ENSOANG00000037111 | O_anatinus | Animals | mono_intergenic | H/ACA | | | intergenic |
| ENSOANG00000037113 | ENSOANG00000037113 | O_anatinus | Animals | mono_intergenic | H/ACA | | | intergenic |
| ENSOANG00000037116 | SNORD10 | O_anatinus | Animals | mono_intronic | C/D | ENSOANG00000050686 | EIF4A1 | protein_coding |
| ENSOANG00000037117 | ENSOANG00000037117 | O_anatinus | Animals | mono_intergenic | H/ACA | | | intergenic |
| ENSOANG00000037118 | ENSOANG00000037118 | O_anatinus | Animals | mono_intergenic | H/ACA | | | intergenic |
| ENSOANG00000037119 | ENSOANG00000037119 | O_anatinus | Animals | mono_intergenic | H/ACA | | | intergenic |
| ENSOANG00000037122 | ENSOANG00000037122 | O_anatinus | Animals | mono_intergenic | H/ACA | | | intergenic |
| ENSOANG00000037124 | ENSOANG00000037124 | O_anatinus | Animals | mono_intergenic | H/ACA | | | intergenic |
| ENSOANG00000037125 | ENSOANG00000037125 | O_anatinus | Animals | mono_intergenic | H/ACA | | | intergenic |
| ENSOANG00000037127 | ENSOANG00000037127 | O_anatinus | Animals | mono_intergenic | H/ACA | | | intergenic |
| ENSOANG00000037129 | ENSOANG00000037129 | O_anatinus | Animals | mono_intronic | H/ACA | ENSOANG00000041330 | COQ4 | protein_coding |
| ENSOANG00000037132 | ENSOANG00000037132 | O_anatinus | Animals | mono_intergenic | H/ACA | | | intergenic |
| ENSOANG00000037134 | ENSOANG00000037134 | O_anatinus | Animals | mono_intergenic | H/ACA | | | intergenic |
| ENSOANG00000037136 | ENSOANG00000037136 | O_anatinus | Animals | mono_intergenic | H/ACA | | | intergenic |
| ENSOANG00000037139 | ENSOANG00000037139 | O_anatinus | Animals | mono_intronic | H/ACA | ENSOANG00000001905 | IRAK4 | protein_coding |
| ENSOANG00000037140 | ENSOANG00000037140 | O_anatinus | Animals | intronic_cluster | H/ACA | ENSOANG00000007531 | SYT1 | protein_coding |
| ENSOANG00000037143 | ENSOANG00000037143 | O_anatinus | Animals | mono_intergenic | H/ACA | | | intergenic |
| ENSOANG00000037144 | ENSOANG00000037144 | O_anatinus | Animals | mono_intergenic | H/ACA | | | intergenic |
| ENSOANG00000037147 | ENSOANG00000037147 | O_anatinus | Animals | mono_intergenic | H/ACA | | | intergenic |
| ENSOANG00000037152 | ENSOANG00000037152 | O_anatinus | Animals | mono_intergenic | H/ACA | | | intergenic |

| | | | | | | | | |
|---|---|---|---|---|---|---|---|---|
| ENSOANG00000037154 | ENSOANG00000037154 | O_anatinus | Animals | mono_intergenic | H/ACA | | | intergenic |
| ENSOANG00000037157 | ENSOANG00000037157 | O_anatinus | Animals | mono_intergenic | H/ACA | | | intergenic |
| ENSOANG00000037159 | ENSOANG00000037159 | O_anatinus | Animals | mono_intergenic | H/ACA | | | intergenic |
| ENSOANG00000037161 | ENSOANG00000037161 | O_anatinus | Animals | mono_intergenic | H/ACA | | | intergenic |
| ENSOANG00000037163 | ENSOANG00000037163 | O_anatinus | Animals | mono_intergenic | H/ACA | | | intergenic |
| ENSOANG00000037166 | ENSOANG00000037166 | O_anatinus | Animals | mono_intronic | H/ACA | ENSOANG00000003850 | DPYD | protein_coding |
| ENSOANG00000037167 | ENSOANG00000037167 | O_anatinus | Animals | mono_intergenic | H/ACA | | | intergenic |
| ENSOANG00000037168 | ENSOANG00000037168 | O_anatinus | Animals | mono_intergenic | H/ACA | | | intergenic |
| ENSOANG00000037172 | ENSOANG00000037172 | O_anatinus | Animals | mono_intronic | H/ACA | ENSOANG00000011618 | ZFPM2 | protein_coding |
| ENSOANG00000037174 | ENSOANG00000037174 | O_anatinus | Animals | mono_intergenic | H/ACA | | | intergenic |
| ENSOANG00000037178 | ENSOANG00000037178 | O_anatinus | Animals | mono_intergenic | H/ACA | | | intergenic |
| ENSOANG00000037180 | ENSOANG00000037180 | O_anatinus | Animals | mono_intergenic | H/ACA | | | intergenic |
| ENSOANG00000037183 | ENSOANG00000037183 | O_anatinus | Animals | mono_intronic | H/ACA | ENSOANG00000006134 | ACVR1 | protein_coding |
| ENSOANG00000037188 | ENSOANG00000037188 | O_anatinus | Animals | mono_intergenic | H/ACA | | | intergenic |
| ENSOANG00000037191 | ENSOANG00000037191 | O_anatinus | Animals | mono_intergenic | H/ACA | | | intergenic |
| ENSOANG00000037194 | ENSOANG00000037194 | O_anatinus | Animals | mono_intergenic | H/ACA | | | intergenic |
| ENSOANG00000037195 | ENSOANG00000037195 | O_anatinus | Animals | mono_intergenic | H/ACA | | | intergenic |
| ENSOANG00000037196 | ENSOANG00000037196 | O_anatinus | Animals | mono_intergenic | H/ACA | | | intergenic |
| ENSOANG00000037197 | ENSOANG00000037197 | O_anatinus | Animals | mono_intergenic | H/ACA | | | intergenic |
| ENSOANG00000037201 | ENSOANG00000037201 | O_anatinus | Animals | mono_intronic | H/ACA | ENSOANG00000003940 | PRKD3 | protein_coding |
| ENSOANG00000037203 | ENSOANG00000037203 | O_anatinus | Animals | mono_intergenic | H/ACA | | | intergenic |
| ENSOANG00000037206 | ENSOANG00000037206 | O_anatinus | Animals | mono_intergenic | H/ACA | | | intergenic |
| ENSOANG00000037209 | ENSOANG00000037209 | O_anatinus | Animals | mono_intergenic | H/ACA | | | intergenic |
| ENSOANG00000037210 | ENSOANG00000037210 | O_anatinus | Animals | mono_intergenic | H/ACA | | | intergenic |
| ENSOANG00000037214 | ENSOANG00000037214 | O_anatinus | Animals | mono_intronic | H/ACA | ENSOANG00000049977 | | protein_coding |
| ENSOANG00000037215 | ENSOANG00000037215 | O_anatinus | Animals | mono_intergenic | H/ACA | | | intergenic |
| ENSOANG00000037217 | ENSOANG00000037217 | O_anatinus | Animals | mono_intronic | H/ACA | ENSOANG00000006227 | MAPK10 | protein_coding |
| ENSOANG00000037222 | ENSOANG00000037222 | O_anatinus | Animals | mono_intergenic | H/ACA | | | intergenic |
| ENSOANG00000037229 | ENSOANG00000037229 | O_anatinus | Animals | mono_intergenic | H/ACA | | | intergenic |
| ENSOANG00000037231 | ENSOANG00000037231 | O_anatinus | Animals | mono_intergenic | H/ACA | | | intergenic |
| ENSOANG00000037232 | ENSOANG00000037232 | O_anatinus | Animals | mono_intergenic | H/ACA | | | intergenic |
| ENSOANG00000037234 | ENSOANG00000037234 | O_anatinus | Animals | mono_intergenic | H/ACA | | | intergenic |
| ENSOANG00000037237 | ENSOANG00000037237 | O_anatinus | Animals | mono_intergenic | H/ACA | | | intergenic |
| ENSOANG00000037238 | ENSOANG00000037238 | O_anatinus | Animals | mono_intergenic | H/ACA | | | intergenic |
| ENSOANG00000037240 | ENSOANG00000037240 | O_anatinus | Animals | mono_intergenic | H/ACA | | | intergenic |
| ENSOANG00000037243 | ENSOANG00000037243 | O_anatinus | Animals | mono_intergenic | H/ACA | | | intergenic |
| ENSOANG00000037244 | ENSOANG00000037244 | O_anatinus | Animals | mono_intergenic | H/ACA | | | intergenic |
| ENSOANG00000037245 | ENSOANG00000037245 | O_anatinus | Animals | mono_intergenic | H/ACA | | | intergenic |
| ENSOANG00000037247 | ENSOANG00000037247 | O_anatinus | Animals | intergenic_cluster | H/ACA | | | intergenic |
| ENSOANG00000037248 | ENSOANG00000037248 | O_anatinus | Animals | mono_intronic | H/ACA | ENSOANG00000037105 | PLD1 | protein_coding |
| ENSOANG00000037249 | ENSOANG00000037249 | O_anatinus | Animals | mono_intronic | H/ACA | ENSOANG00000040515 | YES1 | protein_coding |

| | | | | | | | | |
|---|---|---|---|---|---|---|---|---|
| ENSOANG00000037251 | ENSOANG00000037251 | O_anatinus | Animals | mono_intergenic | H/ACA | | | intergenic |
| ENSOANG00000037253 | ENSOANG00000037253 | O_anatinus | Animals | mono_intergenic | H/ACA | | | intergenic |
| ENSOANG00000037254 | ENSOANG00000037254 | O_anatinus | Animals | mono_intergenic | H/ACA | | | intergenic |
| ENSOANG00000037255 | ENSOANG00000037255 | O_anatinus | Animals | mono_intergenic | H/ACA | | | intergenic |
| ENSOANG00000037257 | ENSOANG00000037257 | O_anatinus | Animals | mono_intergenic | H/ACA | | | intergenic |
| ENSOANG00000037261 | ENSOANG00000037261 | O_anatinus | Animals | mono_intergenic | H/ACA | | | intergenic |
| ENSOANG00000037270 | ENSOANG00000037270 | O_anatinus | Animals | mono_intergenic | H/ACA | | | intergenic |
| ENSOANG00000037273 | ENSOANG00000037273 | O_anatinus | Animals | mono_intergenic | H/ACA | | | intergenic |
| ENSOANG00000037275 | ENSOANG00000037275 | O_anatinus | Animals | mono_intergenic | H/ACA | | | intergenic |
| ENSOANG00000037279 | ENSOANG00000037279 | O_anatinus | Animals | mono_intergenic | H/ACA | | | intergenic |
| ENSOANG00000037284 | ENSOANG00000037284 | O_anatinus | Animals | mono_intergenic | H/ACA | | | intergenic |
| ENSOANG00000037286 | ENSOANG00000037286 | O_anatinus | Animals | mono_intergenic | H/ACA | | | intergenic |
| ENSOANG00000037288 | ENSOANG00000037288 | O_anatinus | Animals | mono_intergenic | H/ACA | | | intergenic |
| ENSOANG00000037297 | ENSOANG00000037297 | O_anatinus | Animals | mono_intergenic | H/ACA | | | intergenic |
| ENSOANG00000037298 | ENSOANG00000037298 | O_anatinus | Animals | mono_intergenic | H/ACA | | | intergenic |
| ENSOANG00000037299 | ENSOANG00000037299 | O_anatinus | Animals | mono_intergenic | H/ACA | | | intergenic |
| ENSOANG00000037300 | ENSOANG00000037300 | O_anatinus | Animals | mono_intergenic | H/ACA | | | intergenic |
| ENSOANG00000037301 | ENSOANG00000037301 | O_anatinus | Animals | mono_intronic | H/ACA | ENSOANG00000007612 | DYM | protein_coding |
| ENSOANG00000037304 | ENSOANG00000037304 | O_anatinus | Animals | mono_intronic | H/ACA | ENSOANG00000009682 | AGBL1 | protein_coding |
| ENSOANG00000037305 | ENSOANG00000037305 | O_anatinus | Animals | mono_intergenic | H/ACA | | | intergenic |
| ENSOANG00000037312 | ENSOANG00000037312 | O_anatinus | Animals | mono_intergenic | H/ACA | | | intergenic |
| ENSOANG00000037314 | ENSOANG00000037314 | O_anatinus | Animals | mono_intergenic | H/ACA | | | intergenic |
| ENSOANG00000037315 | ENSOANG00000037315 | O_anatinus | Animals | mono_intergenic | H/ACA | | | intergenic |
| ENSOANG00000037318 | ENSOANG00000037318 | O_anatinus | Animals | mono_intergenic | H/ACA | | | intergenic |
| ENSOANG00000037323 | ENSOANG00000037323 | O_anatinus | Animals | mono_intergenic | H/ACA | | | intergenic |
| ENSOANG00000037324 | SNORD63 | O_anatinus | Animals | mono_intronic | C/D | ENSOANG00000002817 | HSPA9 | protein_coding |
| ENSOANG00000037325 | ENSOANG00000037325 | O_anatinus | Animals | mono_intronic | H/ACA | ENSOANG00000028802 | | protein_coding |
| ENSOANG00000037334 | ENSOANG00000037334 | O_anatinus | Animals | mono_intronic | H/ACA | ENSOANG00000047971 | | protein_coding |
| ENSOANG00000037336 | ENSOANG00000037336 | O_anatinus | Animals | mono_intergenic | H/ACA | | | intergenic |
| ENSOANG00000037337 | ENSOANG00000037337 | O_anatinus | Animals | mono_intergenic | H/ACA | | | intergenic |
| ENSOANG00000037341 | ENSOANG00000037341 | O_anatinus | Animals | mono_intronic | H/ACA | ENSOANG00000042142 | EIF4A2 | protein_coding |
| ENSOANG00000037343 | ENSOANG00000037343 | O_anatinus | Animals | mono_intergenic | H/ACA | | | intergenic |
| ENSOANG00000037354 | ENSOANG00000037354 | O_anatinus | Animals | mono_intergenic | H/ACA | | | intergenic |
| ENSOANG00000037356 | ENSOANG00000037356 | O_anatinus | Animals | mono_intergenic | C/D | | | intergenic |
| ENSOANG00000037358 | ENSOANG00000037358 | O_anatinus | Animals | mono_intergenic | H/ACA | | | intergenic |
| ENSOANG00000037366 | ENSOANG00000037366 | O_anatinus | Animals | mono_intergenic | H/ACA | | | intergenic |
| ENSOANG00000037367 | ENSOANG00000037367 | O_anatinus | Animals | mono_intergenic | H/ACA | | | intergenic |
| ENSOANG00000037368 | ENSOANG00000037368 | O_anatinus | Animals | mono_intergenic | H/ACA | | | intergenic |
| ENSOANG00000037374 | ENSOANG00000037374 | O_anatinus | Animals | mono_intergenic | H/ACA | | | intergenic |
| ENSOANG00000037377 | ENSOANG00000037377 | O_anatinus | Animals | mono_intergenic | H/ACA | | | intergenic |
| ENSOANG00000037379 | ENSOANG00000037379 | O_anatinus | Animals | mono_intergenic | H/ACA | | | intergenic |

| | | | | | | | | |
|---|---|---|---|---|---|---|---|---|
| ENSOANG00000037383 | ENSOANG00000037383 | O_anatinus | Animals | mono_intronic | H/ACA | ENSOANG00000000112 | IARS1 | protein_coding |
| ENSOANG00000037385 | ENSOANG00000037385 | O_anatinus | Animals | mono_intergenic | H/ACA | | | intergenic |
| ENSOANG00000037387 | ENSOANG00000037387 | O_anatinus | Animals | mono_intergenic | H/ACA | | | intergenic |
| ENSOANG00000037393 | ENSOANG00000037393 | O_anatinus | Animals | mono_intergenic | H/ACA | | | intergenic |
| ENSOANG00000037394 | ENSOANG00000037394 | O_anatinus | Animals | mono_intergenic | H/ACA | | | intergenic |
| ENSOANG00000037398 | ENSOANG00000037398 | O_anatinus | Animals | mono_intergenic | H/ACA | | | intergenic |
| ENSOANG00000037401 | ENSOANG00000037401 | O_anatinus | Animals | mono_intronic | H/ACA | ENSOANG00000002822 | SMOC2 | protein_coding |
| ENSOANG00000037402 | ENSOANG00000037402 | O_anatinus | Animals | intronic_cluster | H/ACA | ENSOANG00000008229 | | protein_coding |
| ENSOANG00000037403 | ENSOANG00000037403 | O_anatinus | Animals | mono_intergenic | H/ACA | | | intergenic |
| ENSOANG00000037404 | ENSOANG00000037404 | O_anatinus | Animals | mono_intergenic | H/ACA | | | intergenic |
| ENSOANG00000037405 | ENSOANG00000037405 | O_anatinus | Animals | mono_intergenic | H/ACA | | | intergenic |
| ENSOANG00000037406 | ENSOANG00000037406 | O_anatinus | Animals | mono_intergenic | H/ACA | | | intergenic |
| ENSOANG00000037407 | ENSOANG00000037407 | O_anatinus | Animals | mono_intergenic | H/ACA | | | intergenic |
| ENSOANG00000037408 | ENSOANG00000037408 | O_anatinus | Animals | mono_intergenic | H/ACA | | | intergenic |
| ENSOANG00000037409 | ENSOANG00000037409 | O_anatinus | Animals | mono_intergenic | H/ACA | | | intergenic |
| ENSOANG00000037411 | ENSOANG00000037411 | O_anatinus | Animals | mono_intergenic | H/ACA | | | intergenic |
| ENSOANG00000037412 | ENSOANG00000037412 | O_anatinus | Animals | mono_intergenic | H/ACA | | | intergenic |
| ENSOANG00000037413 | ENSOANG00000037413 | O_anatinus | Animals | mono_intergenic | H/ACA | | | intergenic |
| ENSOANG00000037420 | ENSOANG00000037420 | O_anatinus | Animals | mono_intergenic | H/ACA | | | intergenic |
| ENSOANG00000037422 | ENSOANG00000037422 | O_anatinus | Animals | mono_intergenic | H/ACA | | | intergenic |
| ENSOANG00000037425 | ENSOANG00000037425 | O_anatinus | Animals | mono_intergenic | H/ACA | | | intergenic |
| ENSOANG00000037428 | ENSOANG00000037428 | O_anatinus | Animals | mono_intergenic | H/ACA | | | intergenic |
| ENSOANG00000037432 | ENSOANG00000037432 | O_anatinus | Animals | mono_intronic | H/ACA | ENSOANG00000008715 | MPP1 | protein_coding |
| ENSOANG00000037434 | ENSOANG00000037434 | O_anatinus | Animals | mono_intronic | H/ACA | ENSOANG00000013096 | SLC2A12 | protein_coding |
| ENSOANG00000037444 | ENSOANG00000037444 | O_anatinus | Animals | mono_intergenic | H/ACA | | | intergenic |
| ENSOANG00000037446 | ENSOANG00000037446 | O_anatinus | Animals | mono_intergenic | H/ACA | | | intergenic |
| ENSOANG00000037450 | ENSOANG00000037450 | O_anatinus | Animals | mono_intergenic | H/ACA | | | intergenic |
| ENSOANG00000037451 | ENSOANG00000037451 | O_anatinus | Animals | mono_intergenic | H/ACA | | | intergenic |
| ENSOANG00000037454 | ENSOANG00000037454 | O_anatinus | Animals | mono_intergenic | H/ACA | | | intergenic |
| ENSOANG00000037455 | ENSOANG00000037455 | O_anatinus | Animals | mono_intronic | H/ACA | ENSOANG00000020097 | TM6SF1 | protein_coding |
| ENSOANG00000037457 | ENSOANG00000037457 | O_anatinus | Animals | mono_intergenic | H/ACA | | | intergenic |
| ENSOANG00000037458 | ENSOANG00000037458 | O_anatinus | Animals | mono_intergenic | H/ACA | | | intergenic |
| ENSOANG00000037459 | ENSOANG00000037459 | O_anatinus | Animals | mono_intergenic | H/ACA | | | intergenic |
| ENSOANG00000037464 | SNORD26 | O_anatinus | Animals | intergenic_cluster | C/D | | | intergenic |
| ENSOANG00000037467 | ENSOANG00000037467 | O_anatinus | Animals | mono_intergenic | H/ACA | | | intergenic |
| ENSOANG00000037469 | ENSOANG00000037469 | O_anatinus | Animals | mono_intergenic | H/ACA | | | intergenic |
| ENSOANG00000037474 | ENSOANG00000037474 | O_anatinus | Animals | mono_intergenic | H/ACA | | | intergenic |
| ENSOANG00000037475 | ENSOANG00000037475 | O_anatinus | Animals | mono_intergenic | H/ACA | | | intergenic |
| ENSOANG00000037481 | ENSOANG00000037481 | O_anatinus | Animals | mono_intergenic | H/ACA | | | intergenic |
| ENSOANG00000037483 | ENSOANG00000037483 | O_anatinus | Animals | intergenic_cluster | H/ACA | | | intergenic |
| ENSOANG00000037484 | ENSOANG00000037484 | O_anatinus | Animals | mono_intergenic | H/ACA | | | intergenic |

| | | | | | | | | |
|---|---|---|---|---|---|---|---|---|
| ENSOANG00000037489 | ENSOANG00000037489 | O_anatinus | Animals | mono_intergenic | H/ACA | | | intergenic |
| ENSOANG00000037490 | ENSOANG00000037490 | O_anatinus | Animals | mono_intergenic | H/ACA | | | intergenic |
| ENSOANG00000037494 | ENSOANG00000037494 | O_anatinus | Animals | mono_intergenic | H/ACA | | | intergenic |
| ENSOANG00000037496 | ENSOANG00000037496 | O_anatinus | Animals | mono_intergenic | H/ACA | | | intergenic |
| ENSOANG00000037497 | ENSOANG00000037497 | O_anatinus | Animals | mono_intronic | H/ACA | ENSOANG00000009896 | NPHP4 | protein_coding |
| ENSOANG00000037499 | ENSOANG00000037499 | O_anatinus | Animals | mono_intergenic | H/ACA | | | intergenic |
| ENSOANG00000037502 | SNORD33 | O_anatinus | Animals | intronic_cluster | C/D | ENSOANG00000000828 | RPL13A | protein_coding |
| ENSOANG00000037503 | ENSOANG00000037503 | O_anatinus | Animals | mono_intergenic | H/ACA | | | intergenic |
| ENSOANG00000037504 | ENSOANG00000037504 | O_anatinus | Animals | mono_intergenic | H/ACA | | | intergenic |
| ENSOANG00000037508 | ENSOANG00000037508 | O_anatinus | Animals | mono_intergenic | H/ACA | | | intergenic |
| ENSOANG00000037509 | ENSOANG00000037509 | O_anatinus | Animals | mono_intergenic | H/ACA | | | intergenic |
| ENSOANG00000037513 | ENSOANG00000037513 | O_anatinus | Animals | mono_intergenic | H/ACA | | | intergenic |
| ENSOANG00000037521 | ENSOANG00000037521 | O_anatinus | Animals | mono_intergenic | H/ACA | | | intergenic |
| ENSOANG00000037522 | ENSOANG00000037522 | O_anatinus | Animals | mono_intergenic | H/ACA | | | intergenic |
| ENSOANG00000037523 | ENSOANG00000037523 | O_anatinus | Animals | mono_intergenic | H/ACA | | | intergenic |
| ENSOANG00000037526 | ENSOANG00000037526 | O_anatinus | Animals | mono_intergenic | H/ACA | | | intergenic |
| ENSOANG00000037532 | ENSOANG00000037532 | O_anatinus | Animals | mono_intronic | H/ACA | ENSOANG00000004670 | | protein_coding |
| ENSOANG00000037535 | ENSOANG00000037535 | O_anatinus | Animals | mono_intergenic | H/ACA | | | intergenic |
| ENSOANG00000037538 | ENSOANG00000037538 | O_anatinus | Animals | mono_intergenic | H/ACA | | | intergenic |
| ENSOANG00000037541 | ENSOANG00000037541 | O_anatinus | Animals | mono_intergenic | H/ACA | | | intergenic |
| ENSOANG00000037544 | ENSOANG00000037544 | O_anatinus | Animals | mono_intergenic | H/ACA | | | intergenic |
| ENSOANG00000037546 | ENSOANG00000037546 | O_anatinus | Animals | mono_intergenic | H/ACA | | | intergenic |
| ENSOANG00000037548 | ENSOANG00000037548 | O_anatinus | Animals | mono_intergenic | H/ACA | | | intergenic |
| ENSOANG00000037552 | ENSOANG00000037552 | O_anatinus | Animals | mono_intergenic | H/ACA | | | intergenic |
| ENSOANG00000037558 | ENSOANG00000037558 | O_anatinus | Animals | mono_intergenic | H/ACA | | | intergenic |
| ENSOANG00000037562 | ENSOANG00000037562 | O_anatinus | Animals | mono_intergenic | H/ACA | | | intergenic |
| ENSOANG00000037565 | ENSOANG00000037565 | O_anatinus | Animals | mono_intronic | H/ACA | ENSOANG00000015608 | | protein_coding |
| ENSOANG00000037568 | ENSOANG00000037568 | O_anatinus | Animals | mono_intergenic | H/ACA | | | intergenic |
| ENSOANG00000037577 | ENSOANG00000037577 | O_anatinus | Animals | mono_intronic | H/ACA | ENSOANG00000014531 | MAP3K13 | protein_coding |
| ENSOANG00000037578 | ENSOANG00000037578 | O_anatinus | Animals | mono_intergenic | H/ACA | | | intergenic |
| ENSOANG00000037584 | ENSOANG00000037584 | O_anatinus | Animals | mono_intergenic | H/ACA | | | intergenic |
| ENSOANG00000037591 | ENSOANG00000037591 | O_anatinus | Animals | mono_intergenic | H/ACA | | | intergenic |
| ENSOANG00000037595 | ENSOANG00000037595 | O_anatinus | Animals | mono_intergenic | H/ACA | | | intergenic |
| ENSOANG00000037601 | ENSOANG00000037601 | O_anatinus | Animals | mono_intergenic | H/ACA | | | intergenic |
| ENSOANG00000037603 | ENSOANG00000037603 | O_anatinus | Animals | mono_intergenic | H/ACA | | | intergenic |
| ENSOANG00000037608 | ENSOANG00000037608 | O_anatinus | Animals | mono_intergenic | H/ACA | | | intergenic |
| ENSOANG00000037609 | SNORD19C | O_anatinus | Animals | mono_intronic | C/D | ENSOANG00000013437 | GNL3 | protein_coding |
| ENSOANG00000037611 | ENSOANG00000037611 | O_anatinus | Animals | mono_intergenic | H/ACA | | | intergenic |
| ENSOANG00000037613 | ENSOANG00000037613 | O_anatinus | Animals | mono_intronic | H/ACA | ENSOANG00000009618 | MACC1 | protein_coding |
| ENSOANG00000037616 | ENSOANG00000037616 | O_anatinus | Animals | mono_intergenic | H/ACA | | | intergenic |
| ENSOANG00000037619 | ENSOANG00000037619 | O_anatinus | Animals | mono_intergenic | H/ACA | | | intergenic |

| | | | | | | | | |
|---|---|---|---|---|---|---|---|---|
| ENSOANG00000037620 | ENSOANG00000037620 | O_anatinus | Animals | mono_intergenic | H/ACA | | | intergenic |
| ENSOANG00000037623 | ENSOANG00000037623 | O_anatinus | Animals | mono_intergenic | H/ACA | | | intergenic |
| ENSOANG00000037630 | ENSOANG00000037630 | O_anatinus | Animals | mono_intergenic | H/ACA | | | intergenic |
| ENSOANG00000037631 | ENSOANG00000037631 | O_anatinus | Animals | mono_intergenic | H/ACA | | | intergenic |
| ENSOANG00000037634 | ENSOANG00000037634 | O_anatinus | Animals | mono_intergenic | H/ACA | | | intergenic |
| ENSOANG00000037638 | ENSOANG00000037638 | O_anatinus | Animals | mono_intergenic | H/ACA | | | intergenic |
| ENSOANG00000037639 | ENSOANG00000037639 | O_anatinus | Animals | mono_intergenic | H/ACA | | | intergenic |
| ENSOANG00000037646 | ENSOANG00000037646 | O_anatinus | Animals | mono_intergenic | H/ACA | | | intergenic |
| ENSOANG00000037652 | ENSOANG00000037652 | O_anatinus | Animals | mono_intergenic | H/ACA | | | intergenic |
| ENSOANG00000037653 | ENSOANG00000037653 | O_anatinus | Animals | mono_intergenic | H/ACA | | | intergenic |
| ENSOANG00000037654 | ENSOANG00000037654 | O_anatinus | Animals | mono_intergenic | H/ACA | | | intergenic |
| ENSOANG00000037655 | ENSOANG00000037655 | O_anatinus | Animals | mono_intergenic | H/ACA | | | intergenic |
| ENSOANG00000037656 | ENSOANG00000037656 | O_anatinus | Animals | mono_intergenic | H/ACA | | | intergenic |
| ENSOANG00000037663 | ENSOANG00000037663 | O_anatinus | Animals | mono_intergenic | H/ACA | | | intergenic |
| ENSOANG00000037666 | ENSOANG00000037666 | O_anatinus | Animals | mono_intergenic | H/ACA | | | intergenic |
| ENSOANG00000037669 | ENSOANG00000037669 | O_anatinus | Animals | mono_intergenic | H/ACA | | | intergenic |
| ENSOANG00000037673 | ENSOANG00000037673 | O_anatinus | Animals | mono_intergenic | H/ACA | | | intergenic |
| ENSOANG00000037674 | ENSOANG00000037674 | O_anatinus | Animals | mono_intergenic | H/ACA | | | intergenic |
| ENSOANG00000037676 | ENSOANG00000037676 | O_anatinus | Animals | mono_intergenic | H/ACA | | | intergenic |
| ENSOANG00000037677 | ENSOANG00000037677 | O_anatinus | Animals | mono_intergenic | H/ACA | | | intergenic |
| ENSOANG00000037678 | ENSOANG00000037678 | O_anatinus | Animals | mono_intergenic | H/ACA | | | intergenic |
| ENSOANG00000037679 | ENSOANG00000037679 | O_anatinus | Animals | mono_intergenic | H/ACA | | | intergenic |
| ENSOANG00000037681 | ENSOANG00000037681 | O_anatinus | Animals | mono_intergenic | H/ACA | | | intergenic |
| ENSOANG00000037682 | ENSOANG00000037682 | O_anatinus | Animals | mono_intergenic | H/ACA | | | intergenic |
| ENSOANG00000037685 | ENSOANG00000037685 | O_anatinus | Animals | mono_intergenic | H/ACA | | | intergenic |
| ENSOANG00000037689 | ENSOANG00000037689 | O_anatinus | Animals | mono_intergenic | H/ACA | | | intergenic |
| ENSOANG00000037690 | ENSOANG00000037690 | O_anatinus | Animals | mono_intergenic | H/ACA | | | intergenic |
| ENSOANG00000037691 | ENSOANG00000037691 | O_anatinus | Animals | mono_intronic | H/ACA | ENSOANG00000012398 | SLC24A2 | protein_coding |
| ENSOANG00000037693 | ENSOANG00000037693 | O_anatinus | Animals | mono_intergenic | H/ACA | | | intergenic |
| ENSOANG00000037697 | ENSOANG00000037697 | O_anatinus | Animals | mono_intergenic | H/ACA | | | intergenic |
| ENSOANG00000037702 | ENSOANG00000037702 | O_anatinus | Animals | mono_intergenic | H/ACA | | | intergenic |
| ENSOANG00000037706 | ENSOANG00000037706 | O_anatinus | Animals | mono_intergenic | H/ACA | | | intergenic |
| ENSOANG00000037709 | ENSOANG00000037709 | O_anatinus | Animals | mono_intronic | H/ACA | ENSOANG00000005607 | DTNA | protein_coding |
| ENSOANG00000037714 | ENSOANG00000037714 | O_anatinus | Animals | mono_intergenic | H/ACA | | | intergenic |
| ENSOANG00000037719 | ENSOANG00000037719 | O_anatinus | Animals | mono_intergenic | H/ACA | | | intergenic |
| ENSOANG00000037720 | ENSOANG00000037720 | O_anatinus | Animals | mono_intronic | H/ACA | ENSOANG00000038402 | | protein_coding |
| ENSOANG00000037721 | ENSOANG00000037721 | O_anatinus | Animals | mono_intergenic | H/ACA | | | intergenic |
| ENSOANG00000037722 | ENSOANG00000037722 | O_anatinus | Animals | mono_intergenic | H/ACA | | | intergenic |
| ENSOANG00000037724 | ENSOANG00000037724 | O_anatinus | Animals | mono_intergenic | H/ACA | | | intergenic |
| ENSOANG00000037728 | ENSOANG00000037728 | O_anatinus | Animals | mono_intergenic | H/ACA | | | intergenic |
| ENSOANG00000037731 | ENSOANG00000037731 | O_anatinus | Animals | mono_intergenic | H/ACA | | | intergenic |

| | | | | | | | | | |
|---|---|---|---|---|---|---|---|---|---|
| ENSOANG00000037733 | ENSOANG00000037733 | O_anatinus | Animals | mono_intergenic | H/ACA | | | | intergenic |
| ENSOANG00000037734 | ENSOANG00000037734 | O_anatinus | Animals | mono_intergenic | H/ACA | | | | intergenic |
| ENSOANG00000037736 | ENSOANG00000037736 | O_anatinus | Animals | mono_intergenic | H/ACA | | | | intergenic |
| ENSOANG00000037739 | ENSOANG00000037739 | O_anatinus | Animals | mono_intergenic | H/ACA | | | | intergenic |
| ENSOANG00000037740 | ENSOANG00000037740 | O_anatinus | Animals | mono_intergenic | H/ACA | | | | intergenic |
| ENSOANG00000037743 | ENSOANG00000037743 | O_anatinus | Animals | mono_intergenic | H/ACA | | | | intergenic |
| ENSOANG00000037744 | ENSOANG00000037744 | O_anatinus | Animals | mono_intergenic | H/ACA | | | | intergenic |
| ENSOANG00000037750 | ENSOANG00000037750 | O_anatinus | Animals | mono_intergenic | H/ACA | | | | intergenic |
| ENSOANG00000037752 | ENSOANG00000037752 | O_anatinus | Animals | mono_intergenic | H/ACA | | | | intergenic |
| ENSOANG00000037753 | ENSOANG00000037753 | O_anatinus | Animals | mono_intronic | H/ACA | ENSOANG00000021690 | | LVRN | protein_coding |
| ENSOANG00000037761 | ENSOANG00000037761 | O_anatinus | Animals | mono_intergenic | H/ACA | | | | intergenic |
| ENSOANG00000037766 | ENSOANG00000037766 | O_anatinus | Animals | mono_intergenic | H/ACA | | | | intergenic |
| ENSOANG00000037770 | ENSOANG00000037770 | O_anatinus | Animals | mono_intergenic | H/ACA | | | | intergenic |
| ENSOANG00000037779 | ENSOANG00000037779 | O_anatinus | Animals | intronic_cluster | H/ACA | ENSOANG00000004035 | | CAMTA1 | protein_coding |
| ENSOANG00000037780 | ENSOANG00000037780 | O_anatinus | Animals | mono_intergenic | H/ACA | | | | intergenic |
| ENSOANG00000037781 | ENSOANG00000037781 | O_anatinus | Animals | mono_intergenic | H/ACA | | | | intergenic |
| ENSOANG00000037784 | ENSOANG00000037784 | O_anatinus | Animals | mono_intergenic | H/ACA | | | | intergenic |
| ENSOANG00000037785 | ENSOANG00000037785 | O_anatinus | Animals | mono_intergenic | H/ACA | | | | intergenic |
| ENSOANG00000037794 | ENSOANG00000037794 | O_anatinus | Animals | mono_intergenic | H/ACA | | | | intergenic |
| ENSOANG00000037796 | ENSOANG00000037796 | O_anatinus | Animals | mono_intergenic | H/ACA | | | | intergenic |
| ENSOANG00000037798 | ENSOANG00000037798 | O_anatinus | Animals | mono_intergenic | H/ACA | | | | intergenic |
| ENSOANG00000037799 | ENSOANG00000037799 | O_anatinus | Animals | mono_intergenic | H/ACA | | | | intergenic |
| ENSOANG00000037800 | ENSOANG00000037800 | O_anatinus | Animals | mono_intergenic | H/ACA | | | | intergenic |
| ENSOANG00000037802 | ENSOANG00000037802 | O_anatinus | Animals | mono_intergenic | H/ACA | | | | intergenic |
| ENSOANG00000037808 | ENSOANG00000037808 | O_anatinus | Animals | mono_intergenic | H/ACA | | | | intergenic |
| ENSOANG00000037810 | ENSOANG00000037810 | O_anatinus | Animals | mono_intergenic | H/ACA | | | | intergenic |
| ENSOANG00000037811 | ENSOANG00000037811 | O_anatinus | Animals | mono_intergenic | H/ACA | | | | intergenic |
| ENSOANG00000037812 | ENSOANG00000037812 | O_anatinus | Animals | mono_intergenic | H/ACA | | | | intergenic |
| ENSOANG00000037813 | ENSOANG00000037813 | O_anatinus | Animals | mono_intergenic | H/ACA | | | | intergenic |
| ENSOANG00000037814 | ENSOANG00000037814 | O_anatinus | Animals | mono_intergenic | H/ACA | | | | intergenic |
| ENSOANG00000037817 | ENSOANG00000037817 | O_anatinus | Animals | mono_intergenic | H/ACA | | | | intergenic |
| ENSOANG00000037819 | ENSOANG00000037819 | O_anatinus | Animals | mono_intergenic | H/ACA | | | | intergenic |
| ENSOANG00000037824 | ENSOANG00000037824 | O_anatinus | Animals | mono_intergenic | H/ACA | | | | intergenic |
| ENSOANG00000037828 | ENSOANG00000037828 | O_anatinus | Animals | mono_intergenic | H/ACA | | | | intergenic |
| ENSOANG00000037831 | ENSOANG00000037831 | O_anatinus | Animals | mono_intergenic | H/ACA | | | | intergenic |
| ENSOANG00000037833 | ENSOANG00000037833 | O_anatinus | Animals | mono_intergenic | H/ACA | | | | intergenic |
| ENSOANG00000037836 | ENSOANG00000037836 | O_anatinus | Animals | mono_intergenic | H/ACA | | | | intergenic |
| ENSOANG00000037841 | ENSOANG00000037841 | O_anatinus | Animals | mono_intergenic | H/ACA | | | | intergenic |
| ENSOANG00000037842 | ENSOANG00000037842 | O_anatinus | Animals | mono_intergenic | H/ACA | | | | intergenic |
| ENSOANG00000037844 | ENSOANG00000037844 | O_anatinus | Animals | mono_intronic | H/ACA | ENSOANG00000003682 | | MATN2 | protein_coding |
| ENSOANG00000037846 | ENSOANG00000037846 | O_anatinus | Animals | mono_intergenic | H/ACA | | | | intergenic |

| | | | | | | | | |
|---|---|---|---|---|---|---|---|---|
| ENSOANG00000037847 | ENSOANG00000037847 | O_anatinus | Animals | mono_intronic | H/ACA | ENSOANG00000009055 | ZNF521 | protein_coding |
| ENSOANG00000037848 | ENSOANG00000037848 | O_anatinus | Animals | mono_intergenic | H/ACA | | | intergenic |
| ENSOANG00000037850 | ENSOANG00000037850 | O_anatinus | Animals | mono_intergenic | H/ACA | | | intergenic |
| ENSOANG00000037854 | ENSOANG00000037854 | O_anatinus | Animals | mono_intergenic | H/ACA | | | intergenic |
| ENSOANG00000037856 | ENSOANG00000037856 | O_anatinus | Animals | mono_intergenic | H/ACA | | | intergenic |
| ENSOANG00000037857 | ENSOANG00000037857 | O_anatinus | Animals | intronic_cluster | C/D | ENSOANG00000008488 | | protein_coding |
| ENSOANG00000037858 | ENSOANG00000037858 | O_anatinus | Animals | mono_intergenic | H/ACA | | | intergenic |
| ENSOANG00000037861 | ENSOANG00000037861 | O_anatinus | Animals | mono_intergenic | H/ACA | | | intergenic |
| ENSOANG00000037862 | ENSOANG00000037862 | O_anatinus | Animals | mono_intergenic | H/ACA | | | intergenic |
| ENSOANG00000037863 | ENSOANG00000037863 | O_anatinus | Animals | mono_intergenic | H/ACA | | | intergenic |
| ENSOANG00000037865 | ENSOANG00000037865 | O_anatinus | Animals | mono_intergenic | H/ACA | | | intergenic |
| ENSOANG00000037866 | ENSOANG00000037866 | O_anatinus | Animals | mono_intergenic | H/ACA | | | intergenic |
| ENSOANG00000037870 | ENSOANG00000037870 | O_anatinus | Animals | mono_intergenic | H/ACA | | | intergenic |
| ENSOANG00000037871 | ENSOANG00000037871 | O_anatinus | Animals | mono_intergenic | H/ACA | | | intergenic |
| ENSOANG00000037874 | ENSOANG00000037874 | O_anatinus | Animals | mono_intergenic | H/ACA | | | intergenic |
| ENSOANG00000037875 | ENSOANG00000037875 | O_anatinus | Animals | mono_intergenic | H/ACA | | | intergenic |
| ENSOANG00000037876 | ENSOANG00000037876 | O_anatinus | Animals | mono_intergenic | H/ACA | | | intergenic |
| ENSOANG00000037878 | ENSOANG00000037878 | O_anatinus | Animals | mono_intergenic | H/ACA | | | intergenic |
| ENSOANG00000037880 | ENSOANG00000037880 | O_anatinus | Animals | mono_intergenic | H/ACA | | | intergenic |
| ENSOANG00000037882 | ENSOANG00000037882 | O_anatinus | Animals | mono_intergenic | H/ACA | | | intergenic |
| ENSOANG00000037888 | ENSOANG00000037888 | O_anatinus | Animals | mono_intergenic | H/ACA | | | intergenic |
| ENSOANG00000037889 | ENSOANG00000037889 | O_anatinus | Animals | mono_intergenic | H/ACA | | | intergenic |
| ENSOANG00000037890 | ENSOANG00000037890 | O_anatinus | Animals | mono_intergenic | H/ACA | | | intergenic |
| ENSOANG00000037892 | ENSOANG00000037892 | O_anatinus | Animals | mono_intergenic | H/ACA | | | intergenic |
| ENSOANG00000037894 | ENSOANG00000037894 | O_anatinus | Animals | mono_intergenic | H/ACA | | | intergenic |
| ENSOANG00000037897 | ENSOANG00000037897 | O_anatinus | Animals | mono_intergenic | H/ACA | | | intergenic |
| ENSOANG00000037904 | ENSOANG00000037904 | O_anatinus | Animals | mono_intergenic | H/ACA | | | intergenic |
| ENSOANG00000037905 | ENSOANG00000037905 | O_anatinus | Animals | mono_intergenic | H/ACA | | | intergenic |
| ENSOANG00000037906 | ENSOANG00000037906 | O_anatinus | Animals | mono_intergenic | H/ACA | | | intergenic |
| ENSOANG00000037908 | ENSOANG00000037908 | O_anatinus | Animals | mono_intergenic | H/ACA | | | intergenic |
| ENSOANG00000037910 | ENSOANG00000037910 | O_anatinus | Animals | mono_intergenic | H/ACA | | | intergenic |
| ENSOANG00000037912 | ENSOANG00000037912 | O_anatinus | Animals | mono_intergenic | H/ACA | | | intergenic |
| ENSOANG00000037914 | ENSOANG00000037914 | O_anatinus | Animals | mono_intergenic | H/ACA | | | intergenic |
| ENSOANG00000037916 | ENSOANG00000037916 | O_anatinus | Animals | mono_intergenic | H/ACA | | | intergenic |
| ENSOANG00000037918 | ENSOANG00000037918 | O_anatinus | Animals | mono_intergenic | H/ACA | | | intergenic |
| ENSOANG00000037919 | ENSOANG00000037919 | O_anatinus | Animals | mono_intronic | H/ACA | ENSOANG00000041984 | DIS3L2 | protein_coding |
| ENSOANG00000037921 | ENSOANG00000037921 | O_anatinus | Animals | mono_intergenic | H/ACA | | | intergenic |
| ENSOANG00000037922 | ENSOANG00000037922 | O_anatinus | Animals | mono_intronic | H/ACA | ENSOANG00000050805 | MN1 | protein_coding |
| ENSOANG00000037924 | ENSOANG00000037924 | O_anatinus | Animals | mono_intronic | H/ACA | ENSOANG00000007817 | TENM2 | protein_coding |
| ENSOANG00000037925 | ENSOANG00000037925 | O_anatinus | Animals | intergenic_cluster | H/ACA | | | intergenic |
| ENSOANG00000037930 | ENSOANG00000037930 | O_anatinus | Animals | mono_intergenic | H/ACA | | | intergenic |

| | | | | | | | | |
|---|---|---|---|---|---|---|---|---|
| ENSOANG00000037933 | ENSOANG00000037933 | O_anatinus | Animals | mono_intergenic | H/ACA | | | intergenic |
| ENSOANG00000037935 | ENSOANG00000037935 | O_anatinus | Animals | mono_intergenic | H/ACA | | | intergenic |
| ENSOANG00000037939 | ENSOANG00000037939 | O_anatinus | Animals | mono_intronic | H/ACA | ENSOANG00000010047 | SNCAIP | protein_coding |
| ENSOANG00000037943 | ENSOANG00000037943 | O_anatinus | Animals | mono_intergenic | H/ACA | | | intergenic |
| ENSOANG00000037947 | ENSOANG00000037947 | O_anatinus | Animals | mono_intergenic | H/ACA | | | intergenic |
| ENSOANG00000037950 | ENSOANG00000037950 | O_anatinus | Animals | mono_intergenic | H/ACA | | | intergenic |
| ENSOANG00000037951 | ENSOANG00000037951 | O_anatinus | Animals | mono_intergenic | H/ACA | | | intergenic |
| ENSOANG00000037955 | ENSOANG00000037955 | O_anatinus | Animals | mono_intergenic | H/ACA | | | intergenic |
| ENSOANG00000037957 | ENSOANG00000037957 | O_anatinus | Animals | mono_intergenic | H/ACA | | | intergenic |
| ENSOANG00000037963 | ENSOANG00000037963 | O_anatinus | Animals | mono_intergenic | H/ACA | | | intergenic |
| ENSOANG00000037972 | ENSOANG00000037972 | O_anatinus | Animals | mono_intergenic | H/ACA | | | intergenic |
| ENSOANG00000037975 | ENSOANG00000037975 | O_anatinus | Animals | mono_intergenic | H/ACA | | | intergenic |
| ENSOANG00000037976 | ENSOANG00000037976 | O_anatinus | Animals | mono_intergenic | H/ACA | | | intergenic |
| ENSOANG00000037979 | ENSOANG00000037979 | O_anatinus | Animals | mono_intergenic | H/ACA | | | intergenic |
| ENSOANG00000037980 | ENSOANG00000037980 | O_anatinus | Animals | mono_intronic | H/ACA | ENSOANG00000002500 | CDH12 | protein_coding |
| ENSOANG00000037982 | ENSOANG00000037982 | O_anatinus | Animals | mono_intergenic | H/ACA | | | intergenic |
| ENSOANG00000037984 | ENSOANG00000037984 | O_anatinus | Animals | mono_intergenic | H/ACA | | | intergenic |
| ENSOANG00000037986 | ENSOANG00000037986 | O_anatinus | Animals | mono_intergenic | H/ACA | | | intergenic |
| ENSOANG00000037987 | ENSOANG00000037987 | O_anatinus | Animals | mono_intergenic | H/ACA | | | intergenic |
| ENSOANG00000037988 | ENSOANG00000037988 | O_anatinus | Animals | mono_intergenic | H/ACA | | | intergenic |
| ENSOANG00000037989 | ENSOANG00000037989 | O_anatinus | Animals | mono_intergenic | H/ACA | | | intergenic |
| ENSOANG00000037992 | ENSOANG00000037992 | O_anatinus | Animals | mono_intronic | H/ACA | ENSOANG00000005068 | | protein_coding |
| ENSOANG00000037996 | ENSOANG00000037996 | O_anatinus | Animals | mono_intergenic | H/ACA | | | intergenic |
| ENSOANG00000037998 | ENSOANG00000037998 | O_anatinus | Animals | mono_intergenic | H/ACA | | | intergenic |
| ENSOANG00000038003 | ENSOANG00000038003 | O_anatinus | Animals | mono_intergenic | H/ACA | | | intergenic |
| ENSOANG00000038004 | ENSOANG00000038004 | O_anatinus | Animals | mono_intergenic | H/ACA | | | intergenic |
| ENSOANG00000038006 | ENSOANG00000038006 | O_anatinus | Animals | mono_intergenic | H/ACA | | | intergenic |
| ENSOANG00000038008 | ENSOANG00000038008 | O_anatinus | Animals | mono_intronic | H/ACA | ENSOANG00000013772 | CSMD1 | protein_coding |
| ENSOANG00000038009 | ENSOANG00000038009 | O_anatinus | Animals | mono_intergenic | H/ACA | | | intergenic |
| ENSOANG00000038010 | ENSOANG00000038010 | O_anatinus | Animals | mono_intergenic | H/ACA | | | intergenic |
| ENSOANG00000038011 | ENSOANG00000038011 | O_anatinus | Animals | mono_intergenic | H/ACA | | | intergenic |
| ENSOANG00000038014 | ENSOANG00000038014 | O_anatinus | Animals | mono_intergenic | H/ACA | | | intergenic |
| ENSOANG00000038019 | ENSOANG00000038019 | O_anatinus | Animals | mono_intergenic | H/ACA | | | intergenic |
| ENSOANG00000038025 | ENSOANG00000038025 | O_anatinus | Animals | mono_intergenic | H/ACA | | | intergenic |
| ENSOANG00000038028 | ENSOANG00000038028 | O_anatinus | Animals | mono_intergenic | H/ACA | | | intergenic |
| ENSOANG00000038029 | ENSOANG00000038029 | O_anatinus | Animals | mono_intergenic | H/ACA | | | intergenic |
| ENSOANG00000038034 | ENSOANG00000038034 | O_anatinus | Animals | mono_intergenic | H/ACA | | | intergenic |
| ENSOANG00000038036 | ENSOANG00000038036 | O_anatinus | Animals | mono_intergenic | H/ACA | | | intergenic |
| ENSOANG00000038044 | ENSOANG00000038044 | O_anatinus | Animals | mono_intergenic | H/ACA | | | intergenic |
| ENSOANG00000038046 | ENSOANG00000038046 | O_anatinus | Animals | mono_intergenic | H/ACA | | | intergenic |
| ENSOANG00000038047 | ENSOANG00000038047 | O_anatinus | Animals | mono_intronic | H/ACA | ENSOANG00000014997 | GLIS3 | protein_coding |

| | | | | | | | | |
|---|---|---|---|---|---|---|---|---|
| ENSOANG00000038048 | ENSOANG00000038048 | O_anatinus | Animals | mono_intergenic | H/ACA | | | intergenic |
| ENSOANG00000038052 | ENSOANG00000038052 | O_anatinus | Animals | mono_intergenic | H/ACA | | | intergenic |
| ENSOANG00000038053 | ENSOANG00000038053 | O_anatinus | Animals | mono_intergenic | H/ACA | | | intergenic |
| ENSOANG00000038055 | ENSOANG00000038055 | O_anatinus | Animals | mono_intronic | H/ACA | ENSOANG00000010041 | PRKCZ | protein_coding |
| ENSOANG00000038058 | ENSOANG00000038058 | O_anatinus | Animals | mono_intergenic | H/ACA | | | intergenic |
| ENSOANG00000038059 | ENSOANG00000038059 | O_anatinus | Animals | mono_intergenic | H/ACA | | | intergenic |
| ENSOANG00000038062 | ENSOANG00000038062 | O_anatinus | Animals | mono_intronic | H/ACA | ENSOANG00000006643 | C12orf50 | protein_coding |
| ENSOANG00000038068 | ENSOANG00000038068 | O_anatinus | Animals | mono_intergenic | H/ACA | | | intergenic |
| ENSOANG00000038070 | ENSOANG00000038070 | O_anatinus | Animals | mono_intergenic | H/ACA | | | intergenic |
| ENSOANG00000038074 | ENSOANG00000038074 | O_anatinus | Animals | intergenic_cluster | H/ACA | | | intergenic |
| ENSOANG00000038077 | ENSOANG00000038077 | O_anatinus | Animals | mono_intergenic | H/ACA | | | intergenic |
| ENSOANG00000038082 | ENSOANG00000038082 | O_anatinus | Animals | mono_intronic | H/ACA | ENSOANG00000004648 | IGF2BP2 | protein_coding |
| ENSOANG00000038087 | ENSOANG00000038087 | O_anatinus | Animals | mono_intergenic | H/ACA | | | intergenic |
| ENSOANG00000038091 | ENSOANG00000038091 | O_anatinus | Animals | mono_intergenic | H/ACA | | | intergenic |
| ENSOANG00000038093 | ENSOANG00000038093 | O_anatinus | Animals | mono_intergenic | H/ACA | | | intergenic |
| ENSOANG00000038095 | ENSOANG00000038095 | O_anatinus | Animals | mono_intergenic | H/ACA | | | intergenic |
| ENSOANG00000038098 | ENSOANG00000038098 | O_anatinus | Animals | mono_intergenic | H/ACA | | | intergenic |
| ENSOANG00000038105 | ENSOANG00000038105 | O_anatinus | Animals | mono_intronic | H/ACA | ENSOANG00000037777 | | protein_coding |
| ENSOANG00000038107 | ENSOANG00000038107 | O_anatinus | Animals | mono_intronic | H/ACA | ENSOANG00000010870 | SYNDIG1 | protein_coding |
| ENSOANG00000038108 | ENSOANG00000038108 | O_anatinus | Animals | mono_intergenic | H/ACA | | | intergenic |
| ENSOANG00000038115 | ENSOANG00000038115 | O_anatinus | Animals | mono_intergenic | H/ACA | | | intergenic |
| ENSOANG00000038117 | ENSOANG00000038117 | O_anatinus | Animals | mono_intergenic | H/ACA | | | intergenic |
| ENSOANG00000038118 | ENSOANG00000038118 | O_anatinus | Animals | mono_intergenic | H/ACA | | | intergenic |
| ENSOANG00000038119 | ENSOANG00000038119 | O_anatinus | Animals | mono_intergenic | H/ACA | | | intergenic |
| ENSOANG00000038120 | ENSOANG00000038120 | O_anatinus | Animals | mono_intergenic | H/ACA | | | intergenic |
| ENSOANG00000038124 | ENSOANG00000038124 | O_anatinus | Animals | mono_intergenic | H/ACA | | | intergenic |
| ENSOANG00000038126 | ENSOANG00000038126 | O_anatinus | Animals | mono_intronic | H/ACA | ENSOANG00000021408 | | protein_coding |
| ENSOANG00000038128 | ENSOANG00000038128 | O_anatinus | Animals | mono_intergenic | H/ACA | | | intergenic |
| ENSOANG00000038129 | ENSOANG00000038129 | O_anatinus | Animals | mono_intergenic | H/ACA | | | intergenic |
| ENSOANG00000038131 | ENSOANG00000038131 | O_anatinus | Animals | mono_intergenic | H/ACA | | | intergenic |
| ENSOANG00000038133 | ENSOANG00000038133 | O_anatinus | Animals | mono_intergenic | H/ACA | | | intergenic |
| ENSOANG00000038135 | ENSOANG00000038135 | O_anatinus | Animals | mono_intergenic | H/ACA | | | intergenic |
| ENSOANG00000038137 | ENSOANG00000038137 | O_anatinus | Animals | mono_intronic | H/ACA | ENSOANG00000004983 | CHST9 | protein_coding |
| ENSOANG00000038138 | ENSOANG00000038138 | O_anatinus | Animals | mono_intergenic | H/ACA | | | intergenic |
| ENSOANG00000038141 | ENSOANG00000038141 | O_anatinus | Animals | mono_intronic | H/ACA | ENSOANG00000002772 | TBCK | protein_coding |
| ENSOANG00000038142 | ENSOANG00000038142 | O_anatinus | Animals | mono_intergenic | H/ACA | | | intergenic |
| ENSOANG00000038146 | ENSOANG00000038146 | O_anatinus | Animals | mono_intergenic | H/ACA | | | intergenic |
| ENSOANG00000038150 | ENSOANG00000038150 | O_anatinus | Animals | mono_intergenic | H/ACA | | | intergenic |
| ENSOANG00000038154 | ENSOANG00000038154 | O_anatinus | Animals | mono_intergenic | H/ACA | | | intergenic |
| ENSOANG00000038159 | ENSOANG00000038159 | O_anatinus | Animals | mono_intergenic | H/ACA | | | intergenic |
| ENSOANG00000038163 | ENSOANG00000038163 | O_anatinus | Animals | mono_intergenic | H/ACA | | | intergenic |

| | | | | | | | | |
|---|---|---|---|---|---|---|---|---|
| ENSOANG00000038166 | ENSOANG00000038166 | O_anatinus | Animals | mono_intergenic | H/ACA | | | intergenic |
| ENSOANG00000038168 | ENSOANG00000038168 | O_anatinus | Animals | mono_intergenic | H/ACA | | | intergenic |
| ENSOANG00000038169 | ENSOANG00000038169 | O_anatinus | Animals | mono_intergenic | H/ACA | | | intergenic |
| ENSOANG00000038170 | ENSOANG00000038170 | O_anatinus | Animals | mono_intronic | H/ACA | ENSOANG00000020618 | | protein_coding |
| ENSOANG00000038171 | ENSOANG00000038171 | O_anatinus | Animals | mono_intronic | H/ACA | ENSOANG00000015335 | CHRDL2 | protein_coding |
| ENSOANG00000038174 | ENSOANG00000038174 | O_anatinus | Animals | mono_intergenic | H/ACA | | | intergenic |
| ENSOANG00000038177 | ENSOANG00000038177 | O_anatinus | Animals | mono_intergenic | H/ACA | | | intergenic |
| ENSOANG00000038179 | ENSOANG00000038179 | O_anatinus | Animals | mono_intronic | H/ACA | ENSOANG00000041538 | ARL4C | protein_coding |
| ENSOANG00000038181 | ENSOANG00000038181 | O_anatinus | Animals | mono_intergenic | H/ACA | | | intergenic |
| ENSOANG00000038183 | ENSOANG00000038183 | O_anatinus | Animals | mono_intergenic | H/ACA | | | intergenic |
| ENSOANG00000038186 | ENSOANG00000038186 | O_anatinus | Animals | mono_intergenic | H/ACA | | | intergenic |
| ENSOANG00000038187 | ENSOANG00000038187 | O_anatinus | Animals | mono_intronic | H/ACA | ENSOANG00000037760 | | protein_coding |
| ENSOANG00000038188 | ENSOANG00000038188 | O_anatinus | Animals | mono_intergenic | H/ACA | | | intergenic |
| ENSOANG00000038191 | ENSOANG00000038191 | O_anatinus | Animals | mono_intergenic | H/ACA | | | intergenic |
| ENSOANG00000038193 | ENSOANG00000038193 | O_anatinus | Animals | mono_intergenic | H/ACA | | | intergenic |
| ENSOANG00000038194 | ENSOANG00000038194 | O_anatinus | Animals | mono_intergenic | H/ACA | | | intergenic |
| ENSOANG00000038200 | ENSOANG00000038200 | O_anatinus | Animals | mono_intergenic | H/ACA | | | intergenic |
| ENSOANG00000038202 | ENSOANG00000038202 | O_anatinus | Animals | mono_intergenic | H/ACA | | | intergenic |
| ENSOANG00000038207 | ENSOANG00000038207 | O_anatinus | Animals | mono_intergenic | H/ACA | | | intergenic |
| ENSOANG00000038208 | ENSOANG00000038208 | O_anatinus | Animals | mono_intergenic | H/ACA | | | intergenic |
| ENSOANG00000038209 | ENSOANG00000038209 | O_anatinus | Animals | mono_intergenic | H/ACA | | | intergenic |
| ENSOANG00000038212 | ENSOANG00000038212 | O_anatinus | Animals | mono_intergenic | H/ACA | | | intergenic |
| ENSOANG00000038215 | ENSOANG00000038215 | O_anatinus | Animals | mono_intergenic | H/ACA | | | intergenic |
| ENSOANG00000038216 | ENSOANG00000038216 | O_anatinus | Animals | mono_intergenic | H/ACA | | | intergenic |
| ENSOANG00000038218 | ENSOANG00000038218 | O_anatinus | Animals | mono_intergenic | H/ACA | | | intergenic |
| ENSOANG00000038219 | ENSOANG00000038219 | O_anatinus | Animals | mono_intergenic | H/ACA | | | intergenic |
| ENSOANG00000038221 | ENSOANG00000038221 | O_anatinus | Animals | mono_intergenic | H/ACA | | | intergenic |
| ENSOANG00000038222 | ENSOANG00000038222 | O_anatinus | Animals | mono_intergenic | H/ACA | | | intergenic |
| ENSOANG00000038223 | ENSOANG00000038223 | O_anatinus | Animals | mono_intergenic | H/ACA | | | intergenic |
| ENSOANG00000038225 | ENSOANG00000038225 | O_anatinus | Animals | mono_intergenic | H/ACA | | | intergenic |
| ENSOANG00000038231 | ENSOANG00000038231 | O_anatinus | Animals | mono_intergenic | H/ACA | | | intergenic |
| ENSOANG00000038232 | ENSOANG00000038232 | O_anatinus | Animals | mono_intergenic | H/ACA | | | intergenic |
| ENSOANG00000038235 | ENSOANG00000038235 | O_anatinus | Animals | mono_intergenic | H/ACA | | | intergenic |
| ENSOANG00000038238 | ENSOANG00000038238 | O_anatinus | Animals | mono_intergenic | H/ACA | | | intergenic |
| ENSOANG00000038241 | ENSOANG00000038241 | O_anatinus | Animals | mono_intergenic | H/ACA | | | intergenic |
| ENSOANG00000038248 | ENSOANG00000038248 | O_anatinus | Animals | mono_intergenic | H/ACA | | | intergenic |
| ENSOANG00000038250 | ENSOANG00000038250 | O_anatinus | Animals | mono_intergenic | H/ACA | | | intergenic |
| ENSOANG00000038254 | ENSOANG00000038254 | O_anatinus | Animals | mono_intergenic | H/ACA | | | intergenic |
| ENSOANG00000038258 | ENSOANG00000038258 | O_anatinus | Animals | mono_intronic | H/ACA | ENSOANG00000043553 | WDR18 | protein_coding |
| ENSOANG00000038260 | ENSOANG00000038260 | O_anatinus | Animals | mono_intergenic | H/ACA | | | intergenic |
| ENSOANG00000038264 | ENSOANG00000038264 | O_anatinus | Animals | mono_intergenic | H/ACA | | | intergenic |

| | | | | | | | | |
|---|---|---|---|---|---|---|---|---|
| ENSOANG00000038266 | ENSOANG00000038266 | O_anatinus | Animals | intronic_cluster | H/ACA | ENSOANG00000037357 | LRRC1 | protein_coding |
| ENSOANG00000038267 | ENSOANG00000038267 | O_anatinus | Animals | mono_intergenic | H/ACA | | | intergenic |
| ENSOANG00000038268 | ENSOANG00000038268 | O_anatinus | Animals | mono_intergenic | H/ACA | | | intergenic |
| ENSOANG00000038275 | ENSOANG00000038275 | O_anatinus | Animals | mono_intergenic | H/ACA | | | intergenic |
| ENSOANG00000038280 | ENSOANG00000038280 | O_anatinus | Animals | mono_intergenic | H/ACA | | | intergenic |
| ENSOANG00000038284 | ENSOANG00000038284 | O_anatinus | Animals | mono_intergenic | H/ACA | | | intergenic |
| ENSOANG00000038286 | ENSOANG00000038286 | O_anatinus | Animals | mono_intergenic | H/ACA | | | intergenic |
| ENSOANG00000038290 | ENSOANG00000038290 | O_anatinus | Animals | mono_intergenic | H/ACA | | | intergenic |
| ENSOANG00000038292 | ENSOANG00000038292 | O_anatinus | Animals | mono_intergenic | H/ACA | | | intergenic |
| ENSOANG00000038293 | ENSOANG00000038293 | O_anatinus | Animals | mono_intergenic | H/ACA | | | intergenic |
| ENSOANG00000038294 | ENSOANG00000038294 | O_anatinus | Animals | mono_intergenic | H/ACA | | | intergenic |
| ENSOANG00000038296 | ENSOANG00000038296 | O_anatinus | Animals | mono_intergenic | H/ACA | | | intergenic |
| ENSOANG00000038298 | ENSOANG00000038298 | O_anatinus | Animals | mono_intergenic | H/ACA | | | intergenic |
| ENSOANG00000038299 | ENSOANG00000038299 | O_anatinus | Animals | mono_intronic | H/ACA | ENSOANG00000005388 | | protein_coding |
| ENSOANG00000038304 | ENSOANG00000038304 | O_anatinus | Animals | mono_intergenic | H/ACA | | | intergenic |
| ENSOANG00000038305 | ENSOANG00000038305 | O_anatinus | Animals | mono_intergenic | H/ACA | | | intergenic |
| ENSOANG00000038306 | ENSOANG00000038306 | O_anatinus | Animals | mono_intergenic | H/ACA | | | intergenic |
| ENSOANG00000038307 | ENSOANG00000038307 | O_anatinus | Animals | mono_intergenic | H/ACA | | | intergenic |
| ENSOANG00000038311 | ENSOANG00000038311 | O_anatinus | Animals | mono_intergenic | H/ACA | | | intergenic |
| ENSOANG00000038314 | ENSOANG00000038314 | O_anatinus | Animals | mono_intergenic | H/ACA | | | intergenic |
| ENSOANG00000038320 | ENSOANG00000038320 | O_anatinus | Animals | mono_intergenic | H/ACA | | | intergenic |
| ENSOANG00000038323 | ENSOANG00000038323 | O_anatinus | Animals | mono_intergenic | H/ACA | | | intergenic |
| ENSOANG00000038324 | ENSOANG00000038324 | O_anatinus | Animals | mono_intergenic | H/ACA | | | intergenic |
| ENSOANG00000038327 | ENSOANG00000038327 | O_anatinus | Animals | mono_intergenic | H/ACA | | | intergenic |
| ENSOANG00000038331 | ENSOANG00000038331 | O_anatinus | Animals | mono_intergenic | H/ACA | | | intergenic |
| ENSOANG00000038332 | ENSOANG00000038332 | O_anatinus | Animals | mono_intergenic | H/ACA | | | intergenic |
| ENSOANG00000038334 | ENSOANG00000038334 | O_anatinus | Animals | mono_intronic | H/ACA | ENSOANG00000002670 | LPIN1 | protein_coding |
| ENSOANG00000038337 | ENSOANG00000038337 | O_anatinus | Animals | mono_intergenic | H/ACA | | | intergenic |
| ENSOANG00000038339 | ENSOANG00000038339 | O_anatinus | Animals | intronic_cluster | H/ACA | ENSOANG00000029079 | | protein_coding |
| ENSOANG00000038340 | ENSOANG00000038340 | O_anatinus | Animals | mono_intergenic | H/ACA | | | intergenic |
| ENSOANG00000038343 | ENSOANG00000038343 | O_anatinus | Animals | mono_intergenic | H/ACA | | | intergenic |
| ENSOANG00000038345 | ENSOANG00000038345 | O_anatinus | Animals | mono_intergenic | H/ACA | | | intergenic |
| ENSOANG00000038346 | ENSOANG00000038346 | O_anatinus | Animals | mono_intergenic | H/ACA | | | intergenic |
| ENSOANG00000038347 | ENSOANG00000038347 | O_anatinus | Animals | mono_intergenic | H/ACA | | | intergenic |
| ENSOANG00000038349 | ENSOANG00000038349 | O_anatinus | Animals | mono_intergenic | H/ACA | | | intergenic |
| ENSOANG00000038352 | ENSOANG00000038352 | O_anatinus | Animals | mono_intronic | H/ACA | ENSOANG00000002822 | SMOC2 | protein_coding |
| ENSOANG00000038354 | ENSOANG00000038354 | O_anatinus | Animals | mono_intergenic | H/ACA | | | intergenic |
| ENSOANG00000038355 | ENSOANG00000038355 | O_anatinus | Animals | mono_intergenic | H/ACA | | | intergenic |
| ENSOANG00000038358 | ENSOANG00000038358 | O_anatinus | Animals | mono_intergenic | H/ACA | | | intergenic |
| ENSOANG00000038361 | ENSOANG00000038361 | O_anatinus | Animals | mono_intergenic | H/ACA | | | intergenic |
| ENSOANG00000038364 | ENSOANG00000038364 | O_anatinus | Animals | mono_intergenic | H/ACA | | | intergenic |

| | | | | | | | | |
|---|---|---|---|---|---|---|---|---|
| ENSOANG00000038367 | ENSOANG00000038367 | O_anatinus | Animals | mono_intergenic | H/ACA | | | intergenic |
| ENSOANG00000038369 | ENSOANG00000038369 | O_anatinus | Animals | mono_intergenic | H/ACA | | | intergenic |
| ENSOANG00000038370 | ENSOANG00000038370 | O_anatinus | Animals | mono_intergenic | H/ACA | | | intergenic |
| ENSOANG00000038375 | ENSOANG00000038375 | O_anatinus | Animals | mono_intergenic | H/ACA | | | intergenic |
| ENSOANG00000038376 | ENSOANG00000038376 | O_anatinus | Animals | mono_intronic | H/ACA | ENSOANG00000010149 | CYP2W1 | protein_coding |
| ENSOANG00000038377 | ENSOANG00000038377 | O_anatinus | Animals | mono_intergenic | H/ACA | | | intergenic |
| ENSOANG00000038380 | ENSOANG00000038380 | O_anatinus | Animals | mono_intergenic | H/ACA | | | intergenic |
| ENSOANG00000038383 | ENSOANG00000038383 | O_anatinus | Animals | mono_intergenic | H/ACA | | | intergenic |
| ENSOANG00000038389 | ENSOANG00000038389 | O_anatinus | Animals | mono_intergenic | H/ACA | | | intergenic |
| ENSOANG00000038397 | ENSOANG00000038397 | O_anatinus | Animals | mono_intronic | H/ACA | ENSOANG00000047996 | MGLL | protein_coding |
| ENSOANG00000038400 | ENSOANG00000038400 | O_anatinus | Animals | mono_intergenic | H/ACA | | | intergenic |
| ENSOANG00000038403 | ENSOANG00000038403 | O_anatinus | Animals | mono_intergenic | H/ACA | | | intergenic |
| ENSOANG00000038406 | ENSOANG00000038406 | O_anatinus | Animals | mono_intergenic | H/ACA | | | intergenic |
| ENSOANG00000038407 | ENSOANG00000038407 | O_anatinus | Animals | mono_intergenic | H/ACA | | | intergenic |
| ENSOANG00000038408 | ENSOANG00000038408 | O_anatinus | Animals | mono_intergenic | H/ACA | | | intergenic |
| ENSOANG00000038409 | ENSOANG00000038409 | O_anatinus | Animals | mono_intronic | H/ACA | ENSOANG00000000848 | | protein_coding |
| ENSOANG00000038410 | ENSOANG00000038410 | O_anatinus | Animals | mono_intronic | C/D | ENSOANG00000050134 | | non_coding |
| ENSOANG00000038411 | ENSOANG00000038411 | O_anatinus | Animals | mono_intergenic | H/ACA | | | intergenic |
| ENSOANG00000038413 | ENSOANG00000038413 | O_anatinus | Animals | mono_intergenic | H/ACA | | | intergenic |
| ENSOANG00000038416 | ENSOANG00000038416 | O_anatinus | Animals | mono_intergenic | H/ACA | | | intergenic |
| ENSOANG00000038417 | ENSOANG00000038417 | O_anatinus | Animals | mono_intergenic | H/ACA | | | intergenic |
| ENSOANG00000038419 | ENSOANG00000038419 | O_anatinus | Animals | mono_intergenic | H/ACA | | | intergenic |
| ENSOANG00000038420 | ENSOANG00000038420 | O_anatinus | Animals | mono_intergenic | H/ACA | | | intergenic |
| ENSOANG00000038422 | ENSOANG00000038422 | O_anatinus | Animals | mono_intergenic | H/ACA | | | intergenic |
| ENSOANG00000038423 | ENSOANG00000038423 | O_anatinus | Animals | mono_intronic | H/ACA | ENSOANG00000013582 | | protein_coding |
| ENSOANG00000038428 | ENSOANG00000038428 | O_anatinus | Animals | mono_intergenic | H/ACA | | | intergenic |
| ENSOANG00000038431 | ENSOANG00000038431 | O_anatinus | Animals | mono_intergenic | H/ACA | | | intergenic |
| ENSOANG00000038438 | ENSOANG00000038438 | O_anatinus | Animals | mono_intergenic | H/ACA | | | intergenic |
| ENSOANG00000038439 | ENSOANG00000038439 | O_anatinus | Animals | mono_intronic | H/ACA | ENSOANG00000028968 | SLC1A3 | protein_coding |
| ENSOANG00000038449 | ENSOANG00000038449 | O_anatinus | Animals | mono_intergenic | H/ACA | | | intergenic |
| ENSOANG00000038453 | ENSOANG00000038453 | O_anatinus | Animals | mono_intronic | H/ACA | ENSOANG00000010079 | TCAIM | protein_coding |
| ENSOANG00000038454 | ENSOANG00000038454 | O_anatinus | Animals | mono_intergenic | H/ACA | | | intergenic |
| ENSOANG00000038460 | ENSOANG00000038460 | O_anatinus | Animals | mono_intergenic | H/ACA | | | intergenic |
| ENSOANG00000038463 | ENSOANG00000038463 | O_anatinus | Animals | mono_intergenic | H/ACA | | | intergenic |
| ENSOANG00000038467 | ENSOANG00000038467 | O_anatinus | Animals | mono_intergenic | H/ACA | | | intergenic |
| ENSOANG00000038469 | ENSOANG00000038469 | O_anatinus | Animals | mono_intergenic | H/ACA | | | intergenic |
| ENSOANG00000038470 | ENSOANG00000038470 | O_anatinus | Animals | mono_intergenic | H/ACA | | | intergenic |
| ENSOANG00000038473 | ENSOANG00000038473 | O_anatinus | Animals | mono_intergenic | H/ACA | | | intergenic |
| ENSOANG00000038474 | ENSOANG00000038474 | O_anatinus | Animals | mono_intergenic | H/ACA | | | intergenic |
| ENSOANG00000038475 | ENSOANG00000038475 | O_anatinus | Animals | mono_intergenic | H/ACA | | | intergenic |
| ENSOANG00000038476 | ENSOANG00000038476 | O_anatinus | Animals | mono_intergenic | H/ACA | | | intergenic |

| | | | | | | | | |
|---|---|---|---|---|---|---|---|---|
| ENSOANG00000038485 | ENSOANG00000038485 | O_anatinus | Animals | mono_intergenic | H/ACA | | | intergenic |
| ENSOANG00000038490 | ENSOANG00000038490 | O_anatinus | Animals | mono_intergenic | H/ACA | | | intergenic |
| ENSOANG00000038493 | ENSOANG00000038493 | O_anatinus | Animals | mono_intergenic | H/ACA | | | intergenic |
| ENSOANG00000038494 | ENSOANG00000038494 | O_anatinus | Animals | mono_intergenic | H/ACA | | | intergenic |
| ENSOANG00000038496 | ENSOANG00000038496 | O_anatinus | Animals | mono_intergenic | H/ACA | | | intergenic |
| ENSOANG00000038506 | ENSOANG00000038506 | O_anatinus | Animals | mono_intergenic | H/ACA | | | intergenic |
| ENSOANG00000038509 | ENSOANG00000038509 | O_anatinus | Animals | mono_intergenic | H/ACA | | | intergenic |
| ENSOANG00000038510 | ENSOANG00000038510 | O_anatinus | Animals | mono_intergenic | H/ACA | | | intergenic |
| ENSOANG00000038511 | ENSOANG00000038511 | O_anatinus | Animals | mono_intergenic | H/ACA | | | intergenic |
| ENSOANG00000038514 | ENSOANG00000038514 | O_anatinus | Animals | mono_intergenic | H/ACA | | | intergenic |
| ENSOANG00000038515 | ENSOANG00000038515 | O_anatinus | Animals | mono_intergenic | H/ACA | | | intergenic |
| ENSOANG00000038516 | ENSOANG00000038516 | O_anatinus | Animals | mono_intergenic | H/ACA | | | intergenic |
| ENSOANG00000038518 | ENSOANG00000038518 | O_anatinus | Animals | mono_intergenic | H/ACA | | | intergenic |
| ENSOANG00000038520 | ENSOANG00000038520 | O_anatinus | Animals | mono_intronic | H/ACA | ENSOANG00000043047 | | protein_coding |
| ENSOANG00000038529 | ENSOANG00000038529 | O_anatinus | Animals | mono_intergenic | H/ACA | | | intergenic |
| ENSOANG00000038532 | ENSOANG00000038532 | O_anatinus | Animals | mono_intronic | H/ACA | ENSOANG00000005418 | VPS41 | protein_coding |
| ENSOANG00000038537 | ENSOANG00000038537 | O_anatinus | Animals | mono_intergenic | H/ACA | | | intergenic |
| ENSOANG00000038538 | ENSOANG00000038538 | O_anatinus | Animals | mono_intergenic | H/ACA | | | intergenic |
| ENSOANG00000038551 | ENSOANG00000038551 | O_anatinus | Animals | mono_intergenic | H/ACA | | | intergenic |
| ENSOANG00000038553 | ENSOANG00000038553 | O_anatinus | Animals | mono_intergenic | H/ACA | | | intergenic |
| ENSOANG00000038554 | ENSOANG00000038554 | O_anatinus | Animals | mono_intergenic | H/ACA | | | intergenic |
| ENSOANG00000038555 | ENSOANG00000038555 | O_anatinus | Animals | mono_intergenic | H/ACA | | | intergenic |
| ENSOANG00000038556 | ENSOANG00000038556 | O_anatinus | Animals | mono_intergenic | H/ACA | | | intergenic |
| ENSOANG00000038557 | ENSOANG00000038557 | O_anatinus | Animals | mono_intergenic | H/ACA | | | intergenic |
| ENSOANG00000038558 | ENSOANG00000038558 | O_anatinus | Animals | mono_intergenic | H/ACA | | | intergenic |
| ENSOANG00000038567 | ENSOANG00000038567 | O_anatinus | Animals | mono_intergenic | H/ACA | | | intergenic |
| ENSOANG00000038568 | ENSOANG00000038568 | O_anatinus | Animals | mono_intronic | H/ACA | ENSOANG00000051013 | MTHFD1 | protein_coding |
| ENSOANG00000038569 | ENSOANG00000038569 | O_anatinus | Animals | mono_intergenic | H/ACA | | | intergenic |
| ENSOANG00000038571 | ENSOANG00000038571 | O_anatinus | Animals | mono_intergenic | H/ACA | | | intergenic |
| ENSOANG00000038574 | ENSOANG00000038574 | O_anatinus | Animals | mono_intronic | H/ACA | ENSOANG00000011656 | LONRF3 | protein_coding |
| ENSOANG00000038581 | ENSOANG00000038581 | O_anatinus | Animals | mono_intronic | H/ACA | ENSOANG00000001738 | TMEM117 | protein_coding |
| ENSOANG00000038582 | ENSOANG00000038582 | O_anatinus | Animals | mono_intergenic | H/ACA | | | intergenic |
| ENSOANG00000038585 | ENSOANG00000038585 | O_anatinus | Animals | mono_intergenic | H/ACA | | | intergenic |
| ENSOANG00000038589 | ENSOANG00000038589 | O_anatinus | Animals | mono_intergenic | H/ACA | | | intergenic |
| ENSOANG00000038590 | ENSOANG00000038590 | O_anatinus | Animals | mono_intronic | H/ACA | ENSOANG00000001356 | SCAF11 | protein_coding |
| ENSOANG00000038591 | ENSOANG00000038591 | O_anatinus | Animals | mono_intergenic | H/ACA | | | intergenic |
| ENSOANG00000038593 | ENSOANG00000038593 | O_anatinus | Animals | mono_intergenic | H/ACA | | | intergenic |
| ENSOANG00000038595 | ENSOANG00000038595 | O_anatinus | Animals | mono_intergenic | H/ACA | | | intergenic |
| ENSOANG00000038601 | ENSOANG00000038601 | O_anatinus | Animals | mono_intergenic | H/ACA | | | intergenic |
| ENSOANG00000038602 | ENSOANG00000038602 | O_anatinus | Animals | mono_intergenic | H/ACA | | | intergenic |
| ENSOANG00000038605 | ENSOANG00000038605 | O_anatinus | Animals | mono_intergenic | H/ACA | | | intergenic |

| | | | | | | | | |
|---|---|---|---|---|---|---|---|---|
| ENSOANG00000038606 | ENSOANG00000038606 | O_anatinus | Animals | mono_intergenic | H/ACA | | | intergenic |
| ENSOANG00000038609 | ENSOANG00000038609 | O_anatinus | Animals | mono_intergenic | H/ACA | | | intergenic |
| ENSOANG00000038611 | ENSOANG00000038611 | O_anatinus | Animals | mono_intergenic | H/ACA | | | intergenic |
| ENSOANG00000038613 | ENSOANG00000038613 | O_anatinus | Animals | mono_intergenic | H/ACA | | | intergenic |
| ENSOANG00000038614 | ENSOANG00000038614 | O_anatinus | Animals | mono_intergenic | H/ACA | | | intergenic |
| ENSOANG00000038618 | ENSOANG00000038618 | O_anatinus | Animals | mono_intronic | H/ACA | ENSOANG00000013014 | WDR72 | protein_coding |
| ENSOANG00000038621 | ENSOANG00000038621 | O_anatinus | Animals | mono_intergenic | H/ACA | | | intergenic |
| ENSOANG00000038624 | ENSOANG00000038624 | O_anatinus | Animals | mono_intergenic | H/ACA | | | intergenic |
| ENSOANG00000038627 | ENSOANG00000038627 | O_anatinus | Animals | mono_intergenic | H/ACA | | | intergenic |
| ENSOANG00000038629 | ENSOANG00000038629 | O_anatinus | Animals | mono_intergenic | H/ACA | | | intergenic |
| ENSOANG00000038632 | ENSOANG00000038632 | O_anatinus | Animals | mono_intergenic | H/ACA | | | intergenic |
| ENSOANG00000038633 | ENSOANG00000038633 | O_anatinus | Animals | mono_intergenic | H/ACA | | | intergenic |
| ENSOANG00000038634 | ENSOANG00000038634 | O_anatinus | Animals | mono_intergenic | H/ACA | | | intergenic |
| ENSOANG00000038635 | ENSOANG00000038635 | O_anatinus | Animals | mono_intergenic | H/ACA | | | intergenic |
| ENSOANG00000038639 | ENSOANG00000038639 | O_anatinus | Animals | mono_intronic | H/ACA | ENSOANG00000013317 | CDK5RAP2 | protein_coding |
| ENSOANG00000038641 | ENSOANG00000038641 | O_anatinus | Animals | mono_intergenic | H/ACA | | | intergenic |
| ENSOANG00000038642 | ENSOANG00000038642 | O_anatinus | Animals | mono_intergenic | H/ACA | | | intergenic |
| ENSOANG00000038646 | ENSOANG00000038646 | O_anatinus | Animals | mono_intronic | H/ACA | ENSOANG00000003826 | EPB41L3 | protein_coding |
| ENSOANG00000038649 | ENSOANG00000038649 | O_anatinus | Animals | mono_intergenic | H/ACA | | | intergenic |
| ENSOANG00000038650 | ENSOANG00000038650 | O_anatinus | Animals | mono_intergenic | H/ACA | | | intergenic |
| ENSOANG00000038651 | ENSOANG00000038651 | O_anatinus | Animals | mono_intergenic | H/ACA | | | intergenic |
| ENSOANG00000038652 | SNORD33 | O_anatinus | Animals | intronic_cluster | C/D | ENSOANG00000000828 | RPL13A | protein_coding |
| ENSOANG00000038653 | ENSOANG00000038653 | O_anatinus | Animals | mono_intergenic | H/ACA | | | intergenic |
| ENSOANG00000038654 | ENSOANG00000038654 | O_anatinus | Animals | mono_intronic | H/ACA | ENSOANG00000002095 | ASTN2 | protein_coding |
| ENSOANG00000038656 | ENSOANG00000038656 | O_anatinus | Animals | mono_intergenic | H/ACA | | | intergenic |
| ENSOANG00000038657 | ENSOANG00000038657 | O_anatinus | Animals | mono_intergenic | H/ACA | | | intergenic |
| ENSOANG00000038662 | ENSOANG00000038662 | O_anatinus | Animals | mono_intergenic | H/ACA | | | intergenic |
| ENSOANG00000038664 | ENSOANG00000038664 | O_anatinus | Animals | mono_intergenic | H/ACA | | | intergenic |
| ENSOANG00000038665 | ENSOANG00000038665 | O_anatinus | Animals | mono_intergenic | H/ACA | | | intergenic |
| ENSOANG00000038667 | ENSOANG00000038667 | O_anatinus | Animals | mono_intergenic | H/ACA | | | intergenic |
| ENSOANG00000038673 | ENSOANG00000038673 | O_anatinus | Animals | mono_intronic | H/ACA | ENSOANG00000011166 | PLCL2 | protein_coding |
| ENSOANG00000038675 | ENSOANG00000038675 | O_anatinus | Animals | mono_intergenic | H/ACA | | | intergenic |
| ENSOANG00000038677 | ENSOANG00000038677 | O_anatinus | Animals | mono_intergenic | H/ACA | | | intergenic |
| ENSOANG00000038678 | ENSOANG00000038678 | O_anatinus | Animals | mono_intergenic | H/ACA | | | intergenic |
| ENSOANG00000038686 | ENSOANG00000038686 | O_anatinus | Animals | mono_intergenic | H/ACA | | | intergenic |
| ENSOANG00000038687 | ENSOANG00000038687 | O_anatinus | Animals | mono_intergenic | H/ACA | | | intergenic |
| ENSOANG00000038690 | ENSOANG00000038690 | O_anatinus | Animals | mono_intergenic | H/ACA | | | intergenic |
| ENSOANG00000038695 | ENSOANG00000038695 | O_anatinus | Animals | mono_intergenic | H/ACA | | | intergenic |
| ENSOANG00000038697 | ENSOANG00000038697 | O_anatinus | Animals | mono_intergenic | H/ACA | | | intergenic |
| ENSOANG00000038699 | ENSOANG00000038699 | O_anatinus | Animals | mono_intergenic | H/ACA | | | intergenic |
| ENSOANG00000038700 | ENSOANG00000038700 | O_anatinus | Animals | mono_intergenic | H/ACA | | | intergenic |

| | | | | | | | | |
|---|---|---|---|---|---|---|---|---|
| ENSOANG00000038701 | ENSOANG00000038701 | O_anatinus | Animals | mono_intronic | H/ACA | ENSOANG00000002138 | MMP16 | protein_coding |
| ENSOANG00000038702 | ENSOANG00000038702 | O_anatinus | Animals | mono_intergenic | H/ACA | | | intergenic |
| ENSOANG00000038703 | ENSOANG00000038703 | O_anatinus | Animals | mono_intergenic | H/ACA | | | intergenic |
| ENSOANG00000038704 | ENSOANG00000038704 | O_anatinus | Animals | mono_intergenic | H/ACA | | | intergenic |
| ENSOANG00000038710 | ENSOANG00000038710 | O_anatinus | Animals | mono_intronic | H/ACA | ENSOANG00000011995 | | protein_coding |
| ENSOANG00000038711 | ENSOANG00000038711 | O_anatinus | Animals | mono_intergenic | H/ACA | | | intergenic |
| ENSOANG00000038713 | ENSOANG00000038713 | O_anatinus | Animals | mono_intergenic | H/ACA | | | intergenic |
| ENSOANG00000038714 | ENSOANG00000038714 | O_anatinus | Animals | mono_intergenic | H/ACA | | | intergenic |
| ENSOANG00000038715 | ENSOANG00000038715 | O_anatinus | Animals | mono_intergenic | H/ACA | | | intergenic |
| ENSOANG00000038717 | ENSOANG00000038717 | O_anatinus | Animals | mono_intergenic | H/ACA | | | intergenic |
| ENSOANG00000038724 | ENSOANG00000038724 | O_anatinus | Animals | mono_intergenic | H/ACA | | | intergenic |
| ENSOANG00000038726 | ENSOANG00000038726 | O_anatinus | Animals | mono_intronic | H/ACA | ENSOANG00000015656 | GLRB | protein_coding |
| ENSOANG00000038728 | ENSOANG00000038728 | O_anatinus | Animals | mono_intergenic | H/ACA | | | intergenic |
| ENSOANG00000038730 | ENSOANG00000038730 | O_anatinus | Animals | mono_intronic | H/ACA | ENSOANG00000013795 | TP63 | protein_coding |
| ENSOANG00000038735 | ENSOANG00000038735 | O_anatinus | Animals | mono_intergenic | H/ACA | | | intergenic |
| ENSOANG00000038736 | ENSOANG00000038736 | O_anatinus | Animals | mono_intergenic | H/ACA | | | intergenic |
| ENSOANG00000038737 | ENSOANG00000038737 | O_anatinus | Animals | intronic_cluster | C/D | ENSOANG00000008488 | | protein_coding |
| ENSOANG00000038738 | ENSOANG00000038738 | O_anatinus | Animals | mono_intergenic | H/ACA | | | intergenic |
| ENSOANG00000038740 | ENSOANG00000038740 | O_anatinus | Animals | mono_intergenic | H/ACA | | | intergenic |
| ENSOANG00000038742 | ENSOANG00000038742 | O_anatinus | Animals | mono_intronic | H/ACA | ENSOANG00000007021 | ERBB4 | protein_coding |
| ENSOANG00000038743 | ENSOANG00000038743 | O_anatinus | Animals | mono_intergenic | H/ACA | | | intergenic |
| ENSOANG00000038744 | ENSOANG00000038744 | O_anatinus | Animals | mono_intergenic | H/ACA | | | intergenic |
| ENSOANG00000038745 | ENSOANG00000038745 | O_anatinus | Animals | mono_intronic | H/ACA | ENSOANG00000001363 | | protein_coding |
| ENSOANG00000038748 | ENSOANG00000038748 | O_anatinus | Animals | mono_intergenic | H/ACA | | | intergenic |
| ENSOANG00000038752 | ENSOANG00000038752 | O_anatinus | Animals | mono_intergenic | H/ACA | | | intergenic |
| ENSOANG00000038754 | ENSOANG00000038754 | O_anatinus | Animals | mono_intergenic | H/ACA | | | intergenic |
| ENSOANG00000038757 | ENSOANG00000038757 | O_anatinus | Animals | mono_intergenic | H/ACA | | | intergenic |
| ENSOANG00000038760 | ENSOANG00000038760 | O_anatinus | Animals | mono_intergenic | H/ACA | | | intergenic |
| ENSOANG00000038768 | ENSOANG00000038768 | O_anatinus | Animals | mono_intergenic | H/ACA | | | intergenic |
| ENSOANG00000038770 | ENSOANG00000038770 | O_anatinus | Animals | mono_intronic | H/ACA | ENSOANG00000013504 | PREX2 | protein_coding |
| ENSOANG00000038778 | ENSOANG00000038778 | O_anatinus | Animals | mono_intronic | H/ACA | ENSOANG00000012129 | | protein_coding |
| ENSOANG00000038780 | ENSOANG00000038780 | O_anatinus | Animals | intronic_cluster | H/ACA | ENSOANG00000007366 | CNTN1 | protein_coding |
| ENSOANG00000038781 | ENSOANG00000038781 | O_anatinus | Animals | mono_intergenic | H/ACA | | | intergenic |
| ENSOANG00000038783 | ENSOANG00000038783 | O_anatinus | Animals | mono_intronic | C/D | ENSOANG00000014467 | WDR43 | protein_coding |
| ENSOANG00000038784 | ENSOANG00000038784 | O_anatinus | Animals | mono_intergenic | H/ACA | | | intergenic |
| ENSOANG00000038787 | ENSOANG00000038787 | O_anatinus | Animals | mono_intergenic | H/ACA | | | intergenic |
| ENSOANG00000038788 | ENSOANG00000038788 | O_anatinus | Animals | mono_intergenic | H/ACA | | | intergenic |
| ENSOANG00000038789 | ENSOANG00000038789 | O_anatinus | Animals | mono_intergenic | H/ACA | | | intergenic |
| ENSOANG00000038790 | ENSOANG00000038790 | O_anatinus | Animals | mono_intergenic | H/ACA | | | intergenic |
| ENSOANG00000038792 | ENSOANG00000038792 | O_anatinus | Animals | mono_intergenic | H/ACA | | | intergenic |
| ENSOANG00000038793 | ENSOANG00000038793 | O_anatinus | Animals | mono_intergenic | H/ACA | | | intergenic |

| | | | | | | | | |
|---|---|---|---|---|---|---|---|---|
| ENSOANG00000038795 | ENSOANG00000038795 | O_anatinus | Animals | mono_intergenic | H/ACA | | | intergenic |
| ENSOANG00000038803 | ENSOANG00000038803 | O_anatinus | Animals | mono_intergenic | H/ACA | | | intergenic |
| ENSOANG00000038806 | ENSOANG00000038806 | O_anatinus | Animals | mono_intergenic | H/ACA | | | intergenic |
| ENSOANG00000038808 | ENSOANG00000038808 | O_anatinus | Animals | mono_intergenic | H/ACA | | | intergenic |
| ENSOANG00000038809 | ENSOANG00000038809 | O_anatinus | Animals | mono_intergenic | H/ACA | | | intergenic |
| ENSOANG00000038810 | ENSOANG00000038810 | O_anatinus | Animals | mono_intergenic | H/ACA | | | intergenic |
| ENSOANG00000038814 | ENSOANG00000038814 | O_anatinus | Animals | mono_intergenic | H/ACA | | | intergenic |
| ENSOANG00000038815 | ENSOANG00000038815 | O_anatinus | Animals | mono_intergenic | H/ACA | | | intergenic |
| ENSOANG00000038819 | ENSOANG00000038819 | O_anatinus | Animals | mono_intergenic | H/ACA | | | intergenic |
| ENSOANG00000038821 | ENSOANG00000038821 | O_anatinus | Animals | mono_intronic | H/ACA | ENSOANG00000012817 | | protein_coding |
| ENSOANG00000038822 | ENSOANG00000038822 | O_anatinus | Animals | mono_intergenic | H/ACA | | | intergenic |
| ENSOANG00000038833 | ENSOANG00000038833 | O_anatinus | Animals | mono_intergenic | H/ACA | | | intergenic |
| ENSOANG00000038834 | ENSOANG00000038834 | O_anatinus | Animals | mono_intergenic | H/ACA | | | intergenic |
| ENSOANG00000038835 | ENSOANG00000038835 | O_anatinus | Animals | mono_intergenic | H/ACA | | | intergenic |
| ENSOANG00000038837 | ENSOANG00000038837 | O_anatinus | Animals | mono_intergenic | H/ACA | | | intergenic |
| ENSOANG00000038838 | ENSOANG00000038838 | O_anatinus | Animals | mono_intronic | H/ACA | ENSOANG00000038575 | | protein_coding |
| ENSOANG00000038839 | ENSOANG00000038839 | O_anatinus | Animals | mono_intergenic | H/ACA | | | intergenic |
| ENSOANG00000038841 | ENSOANG00000038841 | O_anatinus | Animals | mono_intergenic | H/ACA | | | intergenic |
| ENSOANG00000038843 | ENSOANG00000038843 | O_anatinus | Animals | mono_intergenic | H/ACA | | | intergenic |
| ENSOANG00000038846 | ENSOANG00000038846 | O_anatinus | Animals | mono_intergenic | H/ACA | | | intergenic |
| ENSOANG00000038850 | ENSOANG00000038850 | O_anatinus | Animals | mono_intergenic | H/ACA | | | intergenic |
| ENSOANG00000038852 | ENSOANG00000038852 | O_anatinus | Animals | mono_intergenic | H/ACA | | | intergenic |
| ENSOANG00000038854 | ENSOANG00000038854 | O_anatinus | Animals | mono_intergenic | H/ACA | | | intergenic |
| ENSOANG00000038857 | ENSOANG00000038857 | O_anatinus | Animals | mono_intergenic | H/ACA | | | intergenic |
| ENSOANG00000038858 | ENSOANG00000038858 | O_anatinus | Animals | mono_intergenic | H/ACA | | | intergenic |
| ENSOANG00000038859 | ENSOANG00000038859 | O_anatinus | Animals | mono_intergenic | H/ACA | | | intergenic |
| ENSOANG00000038862 | ENSOANG00000038862 | O_anatinus | Animals | mono_intergenic | H/ACA | | | intergenic |
| ENSOANG00000038863 | ENSOANG00000038863 | O_anatinus | Animals | mono_intergenic | H/ACA | | | intergenic |
| ENSOANG00000038865 | ENSOANG00000038865 | O_anatinus | Animals | mono_intergenic | H/ACA | | | intergenic |
| ENSOANG00000038866 | ENSOANG00000038866 | O_anatinus | Animals | mono_intergenic | H/ACA | | | intergenic |
| ENSOANG00000038868 | ENSOANG00000038868 | O_anatinus | Animals | mono_intergenic | H/ACA | | | intergenic |
| ENSOANG00000038869 | ENSOANG00000038869 | O_anatinus | Animals | mono_intergenic | H/ACA | | | intergenic |
| ENSOANG00000038870 | ENSOANG00000038870 | O_anatinus | Animals | mono_intergenic | H/ACA | | | intergenic |
| ENSOANG00000038873 | ENSOANG00000038873 | O_anatinus | Animals | mono_intergenic | H/ACA | | | intergenic |
| ENSOANG00000038875 | ENSOANG00000038875 | O_anatinus | Animals | mono_intergenic | H/ACA | | | intergenic |
| ENSOANG00000038881 | ENSOANG00000038881 | O_anatinus | Animals | mono_intergenic | H/ACA | | | intergenic |
| ENSOANG00000038882 | ENSOANG00000038882 | O_anatinus | Animals | mono_intergenic | H/ACA | | | intergenic |
| ENSOANG00000038883 | ENSOANG00000038883 | O_anatinus | Animals | mono_intergenic | H/ACA | | | intergenic |
| ENSOANG00000038885 | ENSOANG00000038885 | O_anatinus | Animals | intronic_cluster | H/ACA | ENSOANG00000045577 | NUBP1 | protein_coding |
| ENSOANG00000038887 | ENSOANG00000038887 | O_anatinus | Animals | mono_intergenic | H/ACA | | | intergenic |
| ENSOANG00000038892 | ENSOANG00000038892 | O_anatinus | Animals | mono_intergenic | H/ACA | | | intergenic |

| | | | | | | | | |
|---|---|---|---|---|---|---|---|---|
| ENSOANG00000038893 | ENSOANG00000038893 | O_anatinus | Animals | mono_intergenic | H/ACA | | | intergenic |
| ENSOANG00000038894 | ENSOANG00000038894 | O_anatinus | Animals | mono_intergenic | H/ACA | | | intergenic |
| ENSOANG00000038896 | ENSOANG00000038896 | O_anatinus | Animals | mono_intronic | H/ACA | ENSOANG00000002718 | AKAP13 | protein_coding |
| ENSOANG00000038898 | ENSOANG00000038898 | O_anatinus | Animals | mono_intergenic | H/ACA | | | intergenic |
| ENSOANG00000038900 | ENSOANG00000038900 | O_anatinus | Animals | mono_intergenic | H/ACA | | | intergenic |
| ENSOANG00000038901 | ENSOANG00000038901 | O_anatinus | Animals | mono_intergenic | H/ACA | | | intergenic |
| ENSOANG00000038902 | ENSOANG00000038902 | O_anatinus | Animals | mono_intergenic | H/ACA | | | intergenic |
| ENSOANG00000038904 | ENSOANG00000038904 | O_anatinus | Animals | mono_intergenic | H/ACA | | | intergenic |
| ENSOANG00000038906 | ENSOANG00000038906 | O_anatinus | Animals | mono_intergenic | H/ACA | | | intergenic |
| ENSOANG00000038909 | ENSOANG00000038909 | O_anatinus | Animals | mono_intergenic | H/ACA | | | intergenic |
| ENSOANG00000038911 | ENSOANG00000038911 | O_anatinus | Animals | mono_intergenic | H/ACA | | | intergenic |
| ENSOANG00000038915 | ENSOANG00000038915 | O_anatinus | Animals | mono_intergenic | H/ACA | | | intergenic |
| ENSOANG00000038917 | ENSOANG00000038917 | O_anatinus | Animals | mono_intergenic | H/ACA | | | intergenic |
| ENSOANG00000038920 | ENSOANG00000038920 | O_anatinus | Animals | mono_intergenic | H/ACA | | | intergenic |
| ENSOANG00000038921 | ENSOANG00000038921 | O_anatinus | Animals | mono_intergenic | H/ACA | | | intergenic |
| ENSOANG00000038924 | ENSOANG00000038924 | O_anatinus | Animals | mono_intergenic | H/ACA | | | intergenic |
| ENSOANG00000038925 | ENSOANG00000038925 | O_anatinus | Animals | mono_intergenic | H/ACA | | | intergenic |
| ENSOANG00000038926 | ENSOANG00000038926 | O_anatinus | Animals | mono_intergenic | H/ACA | | | intergenic |
| ENSOANG00000038927 | ENSOANG00000038927 | O_anatinus | Animals | mono_intergenic | H/ACA | | | intergenic |
| ENSOANG00000038929 | ENSOANG00000038929 | O_anatinus | Animals | mono_intergenic | H/ACA | | | intergenic |
| ENSOANG00000038930 | ENSOANG00000038930 | O_anatinus | Animals | mono_intergenic | H/ACA | | | intergenic |
| ENSOANG00000038931 | ENSOANG00000038931 | O_anatinus | Animals | mono_intergenic | H/ACA | | | intergenic |
| ENSOANG00000038932 | ENSOANG00000038932 | O_anatinus | Animals | mono_intronic | H/ACA | ENSOANG00000042748 | AIF1L | protein_coding |
| ENSOANG00000038934 | ENSOANG00000038934 | O_anatinus | Animals | mono_intergenic | H/ACA | | | intergenic |
| ENSOANG00000038935 | ENSOANG00000038935 | O_anatinus | Animals | mono_intergenic | H/ACA | | | intergenic |
| ENSOANG00000038936 | ENSOANG00000038936 | O_anatinus | Animals | mono_intergenic | H/ACA | | | intergenic |
| ENSOANG00000038937 | ENSOANG00000038937 | O_anatinus | Animals | mono_intronic | H/ACA | ENSOANG00000048528 | | protein_coding |
| ENSOANG00000038938 | ENSOANG00000038938 | O_anatinus | Animals | mono_intronic | H/ACA | ENSOANG00000010024 | JADE2 | protein_coding |
| ENSOANG00000038939 | ENSOANG00000038939 | O_anatinus | Animals | mono_intronic | H/ACA | ENSOANG00000038763 | | protein_coding |
| ENSOANG00000038940 | ENSOANG00000038940 | O_anatinus | Animals | mono_intergenic | H/ACA | | | intergenic |
| ENSOANG00000038941 | ENSOANG00000038941 | O_anatinus | Animals | mono_intergenic | H/ACA | | | intergenic |
| ENSOANG00000038942 | ENSOANG00000038942 | O_anatinus | Animals | mono_intergenic | H/ACA | | | intergenic |
| ENSOANG00000038944 | ENSOANG00000038944 | O_anatinus | Animals | mono_intronic | H/ACA | ENSOANG00000001812 | PLOD2 | protein_coding |
| ENSOANG00000038956 | ENSOANG00000038956 | O_anatinus | Animals | mono_intergenic | H/ACA | | | intergenic |
| ENSOANG00000038958 | ENSOANG00000038958 | O_anatinus | Animals | mono_intronic | H/ACA | ENSOANG00000011992 | ADAMTSL3 | protein_coding |
| ENSOANG00000038959 | ENSOANG00000038959 | O_anatinus | Animals | mono_intergenic | H/ACA | | | intergenic |
| ENSOANG00000038961 | ENSOANG00000038961 | O_anatinus | Animals | mono_intronic | H/ACA | ENSOANG00000007157 | GRIN2A | protein_coding |
| ENSOANG00000038963 | ENSOANG00000038963 | O_anatinus | Animals | mono_intergenic | H/ACA | | | intergenic |
| ENSOANG00000038964 | ENSOANG00000038964 | O_anatinus | Animals | mono_intergenic | H/ACA | | | intergenic |
| ENSOANG00000038968 | ENSOANG00000038968 | O_anatinus | Animals | mono_intergenic | H/ACA | | | intergenic |
| ENSOANG00000038973 | ENSOANG00000038973 | O_anatinus | Animals | mono_intergenic | H/ACA | | | intergenic |

| | | | | | | | | |
|---|---|---|---|---|---|---|---|---|
| ENSOANG00000038978 | ENSOANG00000038978 | O_anatinus | Animals | mono_intergenic | H/ACA | | | intergenic |
| ENSOANG00000038980 | ENSOANG00000038980 | O_anatinus | Animals | mono_intergenic | H/ACA | | | intergenic |
| ENSOANG00000038981 | ENSOANG00000038981 | O_anatinus | Animals | mono_intergenic | H/ACA | | | intergenic |
| ENSOANG00000038983 | ENSOANG00000038983 | O_anatinus | Animals | mono_intergenic | H/ACA | | | intergenic |
| ENSOANG00000038984 | ENSOANG00000038984 | O_anatinus | Animals | mono_intergenic | H/ACA | | | intergenic |
| ENSOANG00000038985 | ENSOANG00000038985 | O_anatinus | Animals | mono_intergenic | H/ACA | | | intergenic |
| ENSOANG00000038986 | ENSOANG00000038986 | O_anatinus | Animals | mono_intergenic | H/ACA | | | intergenic |
| ENSOANG00000038988 | ENSOANG00000038988 | O_anatinus | Animals | mono_intergenic | H/ACA | | | intergenic |
| ENSOANG00000038989 | ENSOANG00000038989 | O_anatinus | Animals | mono_intergenic | H/ACA | | | intergenic |
| ENSOANG00000038994 | ENSOANG00000038994 | O_anatinus | Animals | mono_intergenic | H/ACA | | | intergenic |
| ENSOANG00000038996 | ENSOANG00000038996 | O_anatinus | Animals | mono_intergenic | H/ACA | | | intergenic |
| ENSOANG00000038997 | ENSOANG00000038997 | O_anatinus | Animals | mono_intergenic | H/ACA | | | intergenic |
| ENSOANG00000038999 | ENSOANG00000038999 | O_anatinus | Animals | mono_intronic | H/ACA | ENSOANG00000021714 | CABCOCO1 | protein_coding |
| ENSOANG00000039002 | ENSOANG00000039002 | O_anatinus | Animals | mono_intergenic | H/ACA | | | intergenic |
| ENSOANG00000039004 | ENSOANG00000039004 | O_anatinus | Animals | mono_intergenic | H/ACA | | | intergenic |
| ENSOANG00000039005 | ENSOANG00000039005 | O_anatinus | Animals | mono_intergenic | H/ACA | | | intergenic |
| ENSOANG00000039007 | ENSOANG00000039007 | O_anatinus | Animals | mono_intergenic | H/ACA | | | intergenic |
| ENSOANG00000039009 | ENSOANG00000039009 | O_anatinus | Animals | mono_intergenic | H/ACA | | | intergenic |
| ENSOANG00000039011 | ENSOANG00000039011 | O_anatinus | Animals | mono_intergenic | H/ACA | | | intergenic |
| ENSOANG00000039016 | ENSOANG00000039016 | O_anatinus | Animals | mono_intergenic | H/ACA | | | intergenic |
| ENSOANG00000039029 | ENSOANG00000039029 | O_anatinus | Animals | mono_intergenic | H/ACA | | | intergenic |
| ENSOANG00000039032 | ENSOANG00000039032 | O_anatinus | Animals | mono_intergenic | H/ACA | | | intergenic |
| ENSOANG00000039034 | ENSOANG00000039034 | O_anatinus | Animals | mono_intergenic | H/ACA | | | intergenic |
| ENSOANG00000039035 | ENSOANG00000039035 | O_anatinus | Animals | mono_intergenic | H/ACA | | | intergenic |
| ENSOANG00000039036 | ENSOANG00000039036 | O_anatinus | Animals | mono_intergenic | H/ACA | | | intergenic |
| ENSOANG00000039043 | ENSOANG00000039043 | O_anatinus | Animals | mono_intergenic | H/ACA | | | intergenic |
| ENSOANG00000039044 | ENSOANG00000039044 | O_anatinus | Animals | mono_intergenic | H/ACA | | | intergenic |
| ENSOANG00000039045 | ENSOANG00000039045 | O_anatinus | Animals | mono_intergenic | H/ACA | | | intergenic |
| ENSOANG00000039048 | ENSOANG00000039048 | O_anatinus | Animals | mono_intergenic | H/ACA | | | intergenic |
| ENSOANG00000039049 | ENSOANG00000039049 | O_anatinus | Animals | mono_intergenic | H/ACA | | | intergenic |
| ENSOANG00000039051 | ENSOANG00000039051 | O_anatinus | Animals | mono_intergenic | H/ACA | | | intergenic |
| ENSOANG00000039053 | ENSOANG00000039053 | O_anatinus | Animals | mono_intergenic | H/ACA | | | intergenic |
| ENSOANG00000039054 | ENSOANG00000039054 | O_anatinus | Animals | mono_intergenic | H/ACA | | | intergenic |
| ENSOANG00000039056 | ENSOANG00000039056 | O_anatinus | Animals | mono_intronic | H/ACA | ENSOANG00000014120 | MYOM2 | protein_coding |
| ENSOANG00000039057 | ENSOANG00000039057 | O_anatinus | Animals | mono_intergenic | H/ACA | | | intergenic |
| ENSOANG00000039058 | ENSOANG00000039058 | O_anatinus | Animals | mono_intergenic | H/ACA | | | intergenic |
| ENSOANG00000039059 | ENSOANG00000039059 | O_anatinus | Animals | mono_intergenic | H/ACA | | | intergenic |
| ENSOANG00000039061 | ENSOANG00000039061 | O_anatinus | Animals | mono_intergenic | H/ACA | | | intergenic |
| ENSOANG00000039062 | ENSOANG00000039062 | O_anatinus | Animals | mono_intronic | H/ACA | ENSOANG00000007236 | | protein_coding |
| ENSOANG00000039064 | ENSOANG00000039064 | O_anatinus | Animals | mono_intronic | H/ACA | ENSOANG00000044543 | | non_coding |
| ENSOANG00000039066 | ENSOANG00000039066 | O_anatinus | Animals | mono_intergenic | H/ACA | | | intergenic |

| | | | | | | | | |
|---|---|---|---|---|---|---|---|---|
| ENSOANG00000039067 | ENSOANG00000039067 | O_anatinus | Animals | mono_intergenic | H/ACA | | | intergenic |
| ENSOANG00000039069 | ENSOANG00000039069 | O_anatinus | Animals | mono_intergenic | H/ACA | | | intergenic |
| ENSOANG00000039072 | ENSOANG00000039072 | O_anatinus | Animals | mono_intergenic | H/ACA | | | intergenic |
| ENSOANG00000039075 | ENSOANG00000039075 | O_anatinus | Animals | mono_intergenic | H/ACA | | | intergenic |
| ENSOANG00000039076 | ENSOANG00000039076 | O_anatinus | Animals | mono_intergenic | H/ACA | | | intergenic |
| ENSOANG00000039077 | ENSOANG00000039077 | O_anatinus | Animals | mono_intergenic | H/ACA | | | intergenic |
| ENSOANG00000039081 | ENSOANG00000039081 | O_anatinus | Animals | mono_intergenic | H/ACA | | | intergenic |
| ENSOANG00000039092 | ENSOANG00000039092 | O_anatinus | Animals | mono_intergenic | H/ACA | | | intergenic |
| ENSOANG00000039100 | ENSOANG00000039100 | O_anatinus | Animals | mono_intergenic | H/ACA | | | intergenic |
| ENSOANG00000039102 | ENSOANG00000039102 | O_anatinus | Animals | mono_intergenic | H/ACA | | | intergenic |
| ENSOANG00000039103 | ENSOANG00000039103 | O_anatinus | Animals | mono_intergenic | H/ACA | | | intergenic |
| ENSOANG00000039104 | ENSOANG00000039104 | O_anatinus | Animals | mono_intronic | H/ACA | ENSOANG00000040855 | | protein_coding |
| ENSOANG00000039106 | ENSOANG00000039106 | O_anatinus | Animals | mono_intergenic | H/ACA | | | intergenic |
| ENSOANG00000039107 | ENSOANG00000039107 | O_anatinus | Animals | mono_intergenic | H/ACA | | | intergenic |
| ENSOANG00000039110 | ENSOANG00000039110 | O_anatinus | Animals | mono_intergenic | H/ACA | | | intergenic |
| ENSOANG00000039111 | ENSOANG00000039111 | O_anatinus | Animals | mono_intergenic | H/ACA | | | intergenic |
| ENSOANG00000039112 | ENSOANG00000039112 | O_anatinus | Animals | mono_intergenic | H/ACA | | | intergenic |
| ENSOANG00000039113 | ENSOANG00000039113 | O_anatinus | Animals | mono_intergenic | H/ACA | | | intergenic |
| ENSOANG00000039116 | ENSOANG00000039116 | O_anatinus | Animals | mono_intronic | H/ACA | ENSOANG00000015055 | FAM13C | protein_coding |
| ENSOANG00000039117 | ENSOANG00000039117 | O_anatinus | Animals | mono_intronic | H/ACA | ENSOANG00000008475 | KCNH1 | protein_coding |
| ENSOANG00000039118 | ENSOANG00000039118 | O_anatinus | Animals | mono_intergenic | H/ACA | | | intergenic |
| ENSOANG00000039119 | ENSOANG00000039119 | O_anatinus | Animals | mono_intergenic | H/ACA | | | intergenic |
| ENSOANG00000039120 | ENSOANG00000039120 | O_anatinus | Animals | mono_intergenic | H/ACA | | | intergenic |
| ENSOANG00000039122 | ENSOANG00000039122 | O_anatinus | Animals | mono_intergenic | H/ACA | | | intergenic |
| ENSOANG00000039123 | ENSOANG00000039123 | O_anatinus | Animals | mono_intergenic | H/ACA | | | intergenic |
| ENSOANG00000039124 | ENSOANG00000039124 | O_anatinus | Animals | mono_intergenic | H/ACA | | | intergenic |
| ENSOANG00000039133 | ENSOANG00000039133 | O_anatinus | Animals | mono_intergenic | H/ACA | | | intergenic |
| ENSOANG00000039138 | ENSOANG00000039138 | O_anatinus | Animals | mono_intergenic | H/ACA | | | intergenic |
| ENSOANG00000039144 | ENSOANG00000039144 | O_anatinus | Animals | mono_intergenic | H/ACA | | | intergenic |
| ENSOANG00000039145 | ENSOANG00000039145 | O_anatinus | Animals | mono_intergenic | H/ACA | | | intergenic |
| ENSOANG00000039146 | ENSOANG00000039146 | O_anatinus | Animals | mono_intergenic | H/ACA | | | intergenic |
| ENSOANG00000039147 | ENSOANG00000039147 | O_anatinus | Animals | mono_intronic | H/ACA | ENSOANG00000042173 | SOX5 | protein_coding |
| ENSOANG00000039151 | ENSOANG00000039151 | O_anatinus | Animals | mono_intergenic | H/ACA | | | intergenic |
| ENSOANG00000039152 | ENSOANG00000039152 | O_anatinus | Animals | mono_intergenic | H/ACA | | | intergenic |
| ENSOANG00000039155 | ENSOANG00000039155 | O_anatinus | Animals | mono_intergenic | H/ACA | | | intergenic |
| ENSOANG00000039156 | ENSOANG00000039156 | O_anatinus | Animals | mono_intergenic | H/ACA | | | intergenic |
| ENSOANG00000039162 | ENSOANG00000039162 | O_anatinus | Animals | mono_intergenic | H/ACA | | | intergenic |
| ENSOANG00000039169 | ENSOANG00000039169 | O_anatinus | Animals | mono_intergenic | H/ACA | | | intergenic |
| ENSOANG00000039170 | ENSOANG00000039170 | O_anatinus | Animals | mono_intergenic | H/ACA | | | intergenic |
| ENSOANG00000039175 | ENSOANG00000039175 | O_anatinus | Animals | mono_intronic | H/ACA | ENSOANG00000011551 | SSUH2 | protein_coding |
| ENSOANG00000039178 | ENSOANG00000039178 | O_anatinus | Animals | mono_intergenic | H/ACA | | | intergenic |

| | | | | | | | | |
|---|---|---|---|---|---|---|---|---|
| ENSOANG00000039181 | ENSOANG00000039181 | O_anatinus | Animals | intronic_cluster | H/ACA | ENSOANG00000004857 | ZNF281 | protein_coding |
| ENSOANG00000039183 | ENSOANG00000039183 | O_anatinus | Animals | mono_intronic | H/ACA | ENSOANG00000043113 | CAPRIN2 | protein_coding |
| ENSOANG00000039185 | ENSOANG00000039185 | O_anatinus | Animals | mono_intergenic | H/ACA | | | intergenic |
| ENSOANG00000039187 | ENSOANG00000039187 | O_anatinus | Animals | mono_intergenic | H/ACA | | | intergenic |
| ENSOANG00000039188 | ENSOANG00000039188 | O_anatinus | Animals | intronic_cluster | H/ACA | ENSOANG00000013591 | | protein_coding |
| ENSOANG00000039190 | ENSOANG00000039190 | O_anatinus | Animals | mono_intergenic | H/ACA | | | intergenic |
| ENSOANG00000039194 | ENSOANG00000039194 | O_anatinus | Animals | mono_intergenic | H/ACA | | | intergenic |
| ENSOANG00000039195 | ENSOANG00000039195 | O_anatinus | Animals | mono_intergenic | H/ACA | | | intergenic |
| ENSOANG00000039199 | ENSOANG00000039199 | O_anatinus | Animals | mono_intergenic | H/ACA | | | intergenic |
| ENSOANG00000039201 | ENSOANG00000039201 | O_anatinus | Animals | mono_intergenic | H/ACA | | | intergenic |
| ENSOANG00000039202 | ENSOANG00000039202 | O_anatinus | Animals | mono_intergenic | H/ACA | | | intergenic |
| ENSOANG00000039206 | ENSOANG00000039206 | O_anatinus | Animals | mono_intergenic | H/ACA | | | intergenic |
| ENSOANG00000039207 | ENSOANG00000039207 | O_anatinus | Animals | mono_intergenic | H/ACA | | | intergenic |
| ENSOANG00000039208 | ENSOANG00000039208 | O_anatinus | Animals | mono_intronic | H/ACA | ENSOANG00000014727 | | protein_coding |
| ENSOANG00000039211 | ENSOANG00000039211 | O_anatinus | Animals | mono_intergenic | H/ACA | | | intergenic |
| ENSOANG00000039212 | ENSOANG00000039212 | O_anatinus | Animals | mono_intergenic | H/ACA | | | intergenic |
| ENSOANG00000039213 | ENSOANG00000039213 | O_anatinus | Animals | mono_intergenic | H/ACA | | | intergenic |
| ENSOANG00000039218 | ENSOANG00000039218 | O_anatinus | Animals | mono_intergenic | H/ACA | | | intergenic |
| ENSOANG00000039222 | ENSOANG00000039222 | O_anatinus | Animals | mono_intergenic | H/ACA | | | intergenic |
| ENSOANG00000039224 | ENSOANG00000039224 | O_anatinus | Animals | mono_intergenic | H/ACA | | | intergenic |
| ENSOANG00000039225 | ENSOANG00000039225 | O_anatinus | Animals | mono_intronic | H/ACA | ENSOANG00000045565 | | non_coding |
| ENSOANG00000039227 | ENSOANG00000039227 | O_anatinus | Animals | mono_intergenic | H/ACA | | | intergenic |
| ENSOANG00000039229 | ENSOANG00000039229 | O_anatinus | Animals | mono_intergenic | H/ACA | | | intergenic |
| ENSOANG00000039230 | ENSOANG00000039230 | O_anatinus | Animals | mono_intergenic | H/ACA | | | intergenic |
| ENSOANG00000039231 | ENSOANG00000039231 | O_anatinus | Animals | mono_intergenic | H/ACA | | | intergenic |
| ENSOANG00000039237 | ENSOANG00000039237 | O_anatinus | Animals | mono_intergenic | H/ACA | | | intergenic |
| ENSOANG00000039240 | ENSOANG00000039240 | O_anatinus | Animals | mono_intergenic | H/ACA | | | intergenic |
| ENSOANG00000039241 | ENSOANG00000039241 | O_anatinus | Animals | mono_intergenic | H/ACA | | | intergenic |
| ENSOANG00000039244 | ENSOANG00000039244 | O_anatinus | Animals | mono_intergenic | H/ACA | | | intergenic |
| ENSOANG00000039245 | ENSOANG00000039245 | O_anatinus | Animals | mono_intergenic | H/ACA | | | intergenic |
| ENSOANG00000039246 | ENSOANG00000039246 | O_anatinus | Animals | mono_intergenic | H/ACA | | | intergenic |
| ENSOANG00000039254 | ENSOANG00000039254 | O_anatinus | Animals | mono_intergenic | H/ACA | | | intergenic |
| ENSOANG00000039255 | ENSOANG00000039255 | O_anatinus | Animals | mono_intergenic | H/ACA | | | intergenic |
| ENSOANG00000039256 | ENSOANG00000039256 | O_anatinus | Animals | mono_intergenic | H/ACA | | | intergenic |
| ENSOANG00000039258 | ENSOANG00000039258 | O_anatinus | Animals | mono_intergenic | H/ACA | | | intergenic |
| ENSOANG00000039260 | ENSOANG00000039260 | O_anatinus | Animals | mono_intergenic | H/ACA | | | intergenic |
| ENSOANG00000039265 | ENSOANG00000039265 | O_anatinus | Animals | mono_intergenic | H/ACA | | | intergenic |
| ENSOANG00000039268 | ENSOANG00000039268 | O_anatinus | Animals | mono_intergenic | H/ACA | | | intergenic |
| ENSOANG00000039275 | ENSOANG00000039275 | O_anatinus | Animals | mono_intergenic | H/ACA | | | intergenic |
| ENSOANG00000039286 | ENSOANG00000039286 | O_anatinus | Animals | mono_intergenic | H/ACA | | | intergenic |
| ENSOANG00000039287 | ENSOANG00000039287 | O_anatinus | Animals | mono_intergenic | H/ACA | | | intergenic |

| | | | | | | | | | |
|---|---|---|---|---|---|---|---|---|---|
| ENSOANG00000039293 | ENSOANG00000039293 | O_anatinus | Animals | mono_intergenic | H/ACA | | | intergenic |
| ENSOANG00000039302 | ENSOANG00000039302 | O_anatinus | Animals | mono_intergenic | H/ACA | | | intergenic |
| ENSOANG00000039303 | ENSOANG00000039303 | O_anatinus | Animals | mono_intronic | H/ACA | ENSOANG00000045136 | SUPT3H | protein_coding |
| ENSOANG00000039304 | ENSOANG00000039304 | O_anatinus | Animals | mono_intergenic | H/ACA | | | intergenic |
| ENSOANG00000039309 | ENSOANG00000039309 | O_anatinus | Animals | mono_intronic | H/ACA | ENSOANG00000036695 | LIPH | protein_coding |
| ENSOANG00000039311 | ENSOANG00000039311 | O_anatinus | Animals | mono_intergenic | H/ACA | | | intergenic |
| ENSOANG00000039312 | ENSOANG00000039312 | O_anatinus | Animals | mono_intergenic | H/ACA | | | intergenic |
| ENSOANG00000039313 | ENSOANG00000039313 | O_anatinus | Animals | mono_intergenic | H/ACA | | | intergenic |
| ENSOANG00000039316 | ENSOANG00000039316 | O_anatinus | Animals | mono_intronic | H/ACA | ENSOANG00000001427 | SGPP2 | protein_coding |
| ENSOANG00000039321 | ENSOANG00000039321 | O_anatinus | Animals | mono_intronic | H/ACA | ENSOANG00000040124 | SERPINI2 | protein_coding |
| ENSOANG00000039323 | ENSOANG00000039323 | O_anatinus | Animals | mono_intergenic | H/ACA | | | intergenic |
| ENSOANG00000039325 | ENSOANG00000039325 | O_anatinus | Animals | mono_intergenic | H/ACA | | | intergenic |
| ENSOANG00000039326 | ENSOANG00000039326 | O_anatinus | Animals | mono_intergenic | H/ACA | | | intergenic |
| ENSOANG00000039331 | ENSOANG00000039331 | O_anatinus | Animals | mono_intronic | H/ACA | ENSOANG00000004111 | FADS3 | protein_coding |
| ENSOANG00000039333 | ENSOANG00000039333 | O_anatinus | Animals | mono_intergenic | H/ACA | | | intergenic |
| ENSOANG00000039334 | ENSOANG00000039334 | O_anatinus | Animals | mono_intergenic | H/ACA | | | intergenic |
| ENSOANG00000039336 | ENSOANG00000039336 | O_anatinus | Animals | mono_intergenic | H/ACA | | | intergenic |
| ENSOANG00000039337 | ENSOANG00000039337 | O_anatinus | Animals | mono_intronic | H/ACA | ENSOANG00000006203 | VPS33A | protein_coding |
| ENSOANG00000039338 | ENSOANG00000039338 | O_anatinus | Animals | mono_intergenic | H/ACA | | | intergenic |
| ENSOANG00000039343 | U3 | O_anatinus | Animals | intronic_cluster | C/D | ENSOANG00000008488 | | protein_coding |
| ENSOANG00000039344 | ENSOANG00000039344 | O_anatinus | Animals | mono_intergenic | H/ACA | | | intergenic |
| ENSOANG00000039347 | ENSOANG00000039347 | O_anatinus | Animals | mono_intergenic | H/ACA | | | intergenic |
| ENSOANG00000039348 | ENSOANG00000039348 | O_anatinus | Animals | mono_intergenic | H/ACA | | | intergenic |
| ENSOANG00000039353 | ENSOANG00000039353 | O_anatinus | Animals | mono_intronic | H/ACA | ENSOANG00000003443 | MTO1 | protein_coding |
| ENSOANG00000039357 | ENSOANG00000039357 | O_anatinus | Animals | intronic_cluster | H/ACA | ENSOANG00000001361 | KIRREL3 | protein_coding |
| ENSOANG00000039358 | ENSOANG00000039358 | O_anatinus | Animals | mono_intergenic | H/ACA | | | intergenic |
| ENSOANG00000039360 | ENSOANG00000039360 | O_anatinus | Animals | mono_intergenic | H/ACA | | | intergenic |
| ENSOANG00000039365 | ENSOANG00000039365 | O_anatinus | Animals | mono_intergenic | H/ACA | | | intergenic |
| ENSOANG00000039369 | ENSOANG00000039369 | O_anatinus | Animals | mono_intergenic | H/ACA | | | intergenic |
| ENSOANG00000039370 | ENSOANG00000039370 | O_anatinus | Animals | mono_intergenic | H/ACA | | | intergenic |
| ENSOANG00000039371 | ENSOANG00000039371 | O_anatinus | Animals | mono_intergenic | H/ACA | | | intergenic |
| ENSOANG00000039372 | ENSOANG00000039372 | O_anatinus | Animals | mono_intronic | H/ACA | ENSOANG00000002218 | | protein_coding |
| ENSOANG00000039373 | ENSOANG00000039373 | O_anatinus | Animals | mono_intronic | H/ACA | ENSOANG00000003555 | SERPINE2 | protein_coding |
| ENSOANG00000039374 | ENSOANG00000039374 | O_anatinus | Animals | mono_intergenic | H/ACA | | | intergenic |
| ENSOANG00000039378 | ENSOANG00000039378 | O_anatinus | Animals | mono_intergenic | H/ACA | | | intergenic |
| ENSOANG00000039381 | ENSOANG00000039381 | O_anatinus | Animals | mono_intergenic | H/ACA | | | intergenic |
| ENSOANG00000039387 | ENSOANG00000039387 | O_anatinus | Animals | mono_intergenic | H/ACA | | | intergenic |
| ENSOANG00000039389 | ENSOANG00000039389 | O_anatinus | Animals | mono_intronic | H/ACA | ENSOANG00000000684 | BGN | protein_coding |
| ENSOANG00000039390 | ENSOANG00000039390 | O_anatinus | Animals | mono_intergenic | H/ACA | | | intergenic |
| ENSOANG00000039396 | ENSOANG00000039396 | O_anatinus | Animals | mono_intergenic | H/ACA | | | intergenic |
| ENSOANG00000039398 | ENSOANG00000039398 | O_anatinus | Animals | intronic_cluster | H/ACA | ENSOANG00000004857 | ZNF281 | protein_coding |

| | | | | | | | | |
|---|---|---|---|---|---|---|---|---|
| ENSOANG00000039403 | ENSOANG00000039403 | O_anatinus | Animals | mono_intergenic | H/ACA | | | intergenic |
| ENSOANG00000039405 | ENSOANG00000039405 | O_anatinus | Animals | mono_intergenic | H/ACA | | | intergenic |
| ENSOANG00000039406 | ENSOANG00000039406 | O_anatinus | Animals | mono_intergenic | H/ACA | | | intergenic |
| ENSOANG00000039407 | ENSOANG00000039407 | O_anatinus | Animals | mono_intergenic | H/ACA | | | intergenic |
| ENSOANG00000039408 | ENSOANG00000039408 | O_anatinus | Animals | mono_intergenic | H/ACA | | | intergenic |
| ENSOANG00000039410 | ENSOANG00000039410 | O_anatinus | Animals | mono_intergenic | H/ACA | | | intergenic |
| ENSOANG00000039412 | ENSOANG00000039412 | O_anatinus | Animals | mono_intergenic | H/ACA | | | intergenic |
| ENSOANG00000039413 | ENSOANG00000039413 | O_anatinus | Animals | mono_intergenic | H/ACA | | | intergenic |
| ENSOANG00000039414 | ENSOANG00000039414 | O_anatinus | Animals | mono_intergenic | H/ACA | | | intergenic |
| ENSOANG00000039417 | ENSOANG00000039417 | O_anatinus | Animals | mono_intergenic | H/ACA | | | intergenic |
| ENSOANG00000039418 | ENSOANG00000039418 | O_anatinus | Animals | mono_intergenic | C/D | | | intergenic |
| ENSOANG00000039431 | ENSOANG00000039431 | O_anatinus | Animals | mono_intergenic | H/ACA | | | intergenic |
| ENSOANG00000039439 | ENSOANG00000039439 | O_anatinus | Animals | mono_intergenic | H/ACA | | | intergenic |
| ENSOANG00000039440 | ENSOANG00000039440 | O_anatinus | Animals | mono_intergenic | H/ACA | | | intergenic |
| ENSOANG00000039442 | ENSOANG00000039442 | O_anatinus | Animals | mono_intergenic | H/ACA | | | intergenic |
| ENSOANG00000039443 | ENSOANG00000039443 | O_anatinus | Animals | mono_intergenic | H/ACA | | | intergenic |
| ENSOANG00000039447 | ENSOANG00000039447 | O_anatinus | Animals | mono_intergenic | H/ACA | | | intergenic |
| ENSOANG00000039448 | ENSOANG00000039448 | O_anatinus | Animals | mono_intergenic | H/ACA | | | intergenic |
| ENSOANG00000039452 | ENSOANG00000039452 | O_anatinus | Animals | mono_intergenic | H/ACA | | | intergenic |
| ENSOANG00000039453 | ENSOANG00000039453 | O_anatinus | Animals | mono_intergenic | H/ACA | | | intergenic |
| ENSOANG00000039454 | ENSOANG00000039454 | O_anatinus | Animals | mono_intergenic | H/ACA | | | intergenic |
| ENSOANG00000039458 | ENSOANG00000039458 | O_anatinus | Animals | mono_intergenic | H/ACA | | | intergenic |
| ENSOANG00000039460 | ENSOANG00000039460 | O_anatinus | Animals | mono_intergenic | H/ACA | | | intergenic |
| ENSOANG00000039465 | ENSOANG00000039465 | O_anatinus | Animals | mono_intronic | C/D | ENSOANG00000050134 | | non_coding |
| ENSOANG00000039471 | ENSOANG00000039471 | O_anatinus | Animals | mono_intronic | H/ACA | ENSOANG00000013531 | MPDZ | protein_coding |
| ENSOANG00000039474 | ENSOANG00000039474 | O_anatinus | Animals | mono_intergenic | H/ACA | | | intergenic |
| ENSOANG00000039475 | ENSOANG00000039475 | O_anatinus | Animals | mono_intergenic | H/ACA | | | intergenic |
| ENSOANG00000039476 | ENSOANG00000039476 | O_anatinus | Animals | mono_intronic | H/ACA | ENSOANG00000008263 | KCNAB2 | protein_coding |
| ENSOANG00000039478 | ENSOANG00000039478 | O_anatinus | Animals | mono_intergenic | H/ACA | | | intergenic |
| ENSOANG00000039479 | ENSOANG00000039479 | O_anatinus | Animals | mono_intronic | H/ACA | ENSOANG00000000485 | EPHA3 | protein_coding |
| ENSOANG00000039480 | ENSOANG00000039480 | O_anatinus | Animals | mono_intergenic | H/ACA | | | intergenic |
| ENSOANG00000039481 | ENSOANG00000039481 | O_anatinus | Animals | mono_intergenic | H/ACA | | | intergenic |
| ENSOANG00000039482 | ENSOANG00000039482 | O_anatinus | Animals | mono_intergenic | H/ACA | | | intergenic |
| ENSOANG00000039483 | ENSOANG00000039483 | O_anatinus | Animals | mono_intergenic | H/ACA | | | intergenic |
| ENSOANG00000039484 | ENSOANG00000039484 | O_anatinus | Animals | mono_intergenic | H/ACA | | | intergenic |
| ENSOANG00000039489 | ENSOANG00000039489 | O_anatinus | Animals | mono_intronic | H/ACA | ENSOANG00000037227 | | protein_coding |
| ENSOANG00000039492 | ENSOANG00000039492 | O_anatinus | Animals | mono_intergenic | H/ACA | | | intergenic |
| ENSOANG00000039495 | ENSOANG00000039495 | O_anatinus | Animals | mono_intergenic | H/ACA | | | intergenic |
| ENSOANG00000039500 | ENSOANG00000039500 | O_anatinus | Animals | mono_intergenic | H/ACA | | | intergenic |
| ENSOANG00000039501 | ENSOANG00000039501 | O_anatinus | Animals | mono_intronic | H/ACA | ENSOANG00000013258 | RIMS2 | protein_coding |
| ENSOANG00000039505 | ENSOANG00000039505 | O_anatinus | Animals | mono_intergenic | H/ACA | | | intergenic |

| | | | | | | | | |
|---|---|---|---|---|---|---|---|---|
| ENSOANG00000039506 | ENSOANG00000039506 | O_anatinus | Animals | mono_intergenic | H/ACA | | | intergenic |
| ENSOANG00000039512 | ENSOANG00000039512 | O_anatinus | Animals | mono_intergenic | H/ACA | | | intergenic |
| ENSOANG00000039513 | ENSOANG00000039513 | O_anatinus | Animals | mono_intergenic | H/ACA | | | intergenic |
| ENSOANG00000039516 | ENSOANG00000039516 | O_anatinus | Animals | mono_intergenic | H/ACA | | | intergenic |
| ENSOANG00000039519 | ENSOANG00000039519 | O_anatinus | Animals | mono_intergenic | H/ACA | | | intergenic |
| ENSOANG00000039520 | ENSOANG00000039520 | O_anatinus | Animals | mono_intergenic | H/ACA | | | intergenic |
| ENSOANG00000039521 | ENSOANG00000039521 | O_anatinus | Animals | mono_intronic | H/ACA | ENSOANG00000011398 | | protein_coding |
| ENSOANG00000039523 | ENSOANG00000039523 | O_anatinus | Animals | mono_intergenic | H/ACA | | | intergenic |
| ENSOANG00000039524 | ENSOANG00000039524 | O_anatinus | Animals | intronic_cluster | H/ACA | ENSOANG00000004035 | CAMTA1 | protein_coding |
| ENSOANG00000039526 | ENSOANG00000039526 | O_anatinus | Animals | mono_intergenic | H/ACA | | | intergenic |
| ENSOANG00000039528 | ENSOANG00000039528 | O_anatinus | Animals | mono_intergenic | H/ACA | | | intergenic |
| ENSOANG00000039532 | ENSOANG00000039532 | O_anatinus | Animals | mono_intergenic | H/ACA | | | intergenic |
| ENSOANG00000039535 | ENSOANG00000039535 | O_anatinus | Animals | mono_intergenic | H/ACA | | | intergenic |
| ENSOANG00000039536 | ENSOANG00000039536 | O_anatinus | Animals | mono_intergenic | H/ACA | | | intergenic |
| ENSOANG00000039540 | ENSOANG00000039540 | O_anatinus | Animals | mono_intergenic | H/ACA | | | intergenic |
| ENSOANG00000039541 | ENSOANG00000039541 | O_anatinus | Animals | mono_intergenic | H/ACA | | | intergenic |
| ENSOANG00000039547 | ENSOANG00000039547 | O_anatinus | Animals | mono_intergenic | H/ACA | | | intergenic |
| ENSOANG00000039548 | ENSOANG00000039548 | O_anatinus | Animals | mono_intronic | H/ACA | ENSOANG00000011733 | SLC9A9 | protein_coding |
| ENSOANG00000039553 | ENSOANG00000039553 | O_anatinus | Animals | mono_intergenic | H/ACA | | | intergenic |
| ENSOANG00000039555 | ENSOANG00000039555 | O_anatinus | Animals | mono_intergenic | H/ACA | | | intergenic |
| ENSOANG00000039556 | ENSOANG00000039556 | O_anatinus | Animals | mono_intronic | H/ACA | ENSOANG00000009765 | DOP1B | protein_coding |
| ENSOANG00000039557 | ENSOANG00000039557 | O_anatinus | Animals | mono_intergenic | H/ACA | | | intergenic |
| ENSOANG00000039558 | ENSOANG00000039558 | O_anatinus | Animals | mono_intergenic | H/ACA | | | intergenic |
| ENSOANG00000039559 | ENSOANG00000039559 | O_anatinus | Animals | mono_intergenic | H/ACA | | | intergenic |
| ENSOANG00000039561 | ENSOANG00000039561 | O_anatinus | Animals | mono_intronic | H/ACA | ENSOANG00000047400 | DGKG | protein_coding |
| ENSOANG00000039562 | ENSOANG00000039562 | O_anatinus | Animals | mono_intergenic | H/ACA | | | intergenic |
| ENSOANG00000039566 | ENSOANG00000039566 | O_anatinus | Animals | mono_intronic | H/ACA | ENSOANG00000008726 | | protein_coding |
| ENSOANG00000039567 | ENSOANG00000039567 | O_anatinus | Animals | mono_intergenic | H/ACA | | | intergenic |
| ENSOANG00000039570 | ENSOANG00000039570 | O_anatinus | Animals | mono_intergenic | H/ACA | | | intergenic |
| ENSOANG00000039577 | ENSOANG00000039577 | O_anatinus | Animals | mono_intergenic | H/ACA | | | intergenic |
| ENSOANG00000039580 | ENSOANG00000039580 | O_anatinus | Animals | intronic_cluster | H/ACA | ENSOANG00000043186 | STIMATE | protein_coding |
| ENSOANG00000039581 | ENSOANG00000039581 | O_anatinus | Animals | mono_intronic | H/ACA | ENSOANG00000022453 | | protein_coding |
| ENSOANG00000039582 | ENSOANG00000039582 | O_anatinus | Animals | mono_intergenic | H/ACA | | | intergenic |
| ENSOANG00000039585 | ENSOANG00000039585 | O_anatinus | Animals | mono_intergenic | H/ACA | | | intergenic |
| ENSOANG00000039588 | ENSOANG00000039588 | O_anatinus | Animals | mono_intergenic | H/ACA | | | intergenic |
| ENSOANG00000039596 | ENSOANG00000039596 | O_anatinus | Animals | mono_intergenic | H/ACA | | | intergenic |
| ENSOANG00000039597 | ENSOANG00000039597 | O_anatinus | Animals | mono_intergenic | H/ACA | | | intergenic |
| ENSOANG00000039599 | ENSOANG00000039599 | O_anatinus | Animals | mono_intergenic | H/ACA | | | intergenic |
| ENSOANG00000039600 | ENSOANG00000039600 | O_anatinus | Animals | mono_intronic | H/ACA | ENSOANG00000020697 | RSRC1 | protein_coding |
| ENSOANG00000039604 | ENSOANG00000039604 | O_anatinus | Animals | mono_intergenic | H/ACA | | | intergenic |
| ENSOANG00000039605 | ENSOANG00000039605 | O_anatinus | Animals | mono_intergenic | H/ACA | | | intergenic |

| | | | | | | | | |
|---|---|---|---|---|---|---|---|---|
| ENSOANG00000039608 | ENSOANG00000039608 | O_anatinus | Animals | mono_intergenic | H/ACA | | | intergenic |
| ENSOANG00000039610 | ENSOANG00000039610 | O_anatinus | Animals | mono_intergenic | H/ACA | | | intergenic |
| ENSOANG00000039617 | ENSOANG00000039617 | O_anatinus | Animals | mono_intergenic | H/ACA | | | intergenic |
| ENSOANG00000039618 | ENSOANG00000039618 | O_anatinus | Animals | mono_intergenic | H/ACA | | | intergenic |
| ENSOANG00000039621 | ENSOANG00000039621 | O_anatinus | Animals | mono_intergenic | H/ACA | | | intergenic |
| ENSOANG00000039623 | ENSOANG00000039623 | O_anatinus | Animals | mono_intergenic | H/ACA | | | intergenic |
| ENSOANG00000039625 | ENSOANG00000039625 | O_anatinus | Animals | mono_intergenic | H/ACA | | | intergenic |
| ENSOANG00000039627 | ENSOANG00000039627 | O_anatinus | Animals | mono_intergenic | H/ACA | | | intergenic |
| ENSOANG00000039633 | ENSOANG00000039633 | O_anatinus | Animals | mono_intergenic | H/ACA | | | intergenic |
| ENSOANG00000039634 | ENSOANG00000039634 | O_anatinus | Animals | mono_intergenic | H/ACA | | | intergenic |
| ENSOANG00000039635 | ENSOANG00000039635 | O_anatinus | Animals | mono_intergenic | H/ACA | | | intergenic |
| ENSOANG00000039637 | ENSOANG00000039637 | O_anatinus | Animals | mono_intergenic | H/ACA | | | intergenic |
| ENSOANG00000039641 | ENSOANG00000039641 | O_anatinus | Animals | mono_intronic | C/D | ENSOANG00000015252 | EIF4G2 | protein_coding |
| ENSOANG00000039642 | ENSOANG00000039642 | O_anatinus | Animals | mono_intergenic | H/ACA | | | intergenic |
| ENSOANG00000039643 | ENSOANG00000039643 | O_anatinus | Animals | mono_intergenic | H/ACA | | | intergenic |
| ENSOANG00000039644 | ENSOANG00000039644 | O_anatinus | Animals | mono_intergenic | H/ACA | | | intergenic |
| ENSOANG00000039647 | ENSOANG00000039647 | O_anatinus | Animals | mono_intronic | H/ACA | ENSOANG00000015023 | KLK7 | protein_coding |
| ENSOANG00000039650 | ENSOANG00000039650 | O_anatinus | Animals | mono_intergenic | H/ACA | | | intergenic |
| ENSOANG00000039652 | ENSOANG00000039652 | O_anatinus | Animals | mono_intergenic | H/ACA | | | intergenic |
| ENSOANG00000039662 | ENSOANG00000039662 | O_anatinus | Animals | mono_intergenic | H/ACA | | | intergenic |
| ENSOANG00000039664 | ENSOANG00000039664 | O_anatinus | Animals | mono_intergenic | H/ACA | | | intergenic |
| ENSOANG00000039665 | ENSOANG00000039665 | O_anatinus | Animals | mono_intergenic | H/ACA | | | intergenic |
| ENSOANG00000039666 | ENSOANG00000039666 | O_anatinus | Animals | mono_intronic | H/ACA | ENSOANG00000046970 | CELSR2 | protein_coding |
| ENSOANG00000039671 | ENSOANG00000039671 | O_anatinus | Animals | mono_intergenic | H/ACA | | | intergenic |
| ENSOANG00000039673 | ENSOANG00000039673 | O_anatinus | Animals | mono_intergenic | H/ACA | | | intergenic |
| ENSOANG00000039676 | ENSOANG00000039676 | O_anatinus | Animals | mono_intergenic | H/ACA | | | intergenic |
| ENSOANG00000039681 | ENSOANG00000039681 | O_anatinus | Animals | mono_intergenic | H/ACA | | | intergenic |
| ENSOANG00000039684 | ENSOANG00000039684 | O_anatinus | Animals | mono_intergenic | H/ACA | | | intergenic |
| ENSOANG00000039685 | ENSOANG00000039685 | O_anatinus | Animals | mono_intergenic | H/ACA | | | intergenic |
| ENSOANG00000039686 | ENSOANG00000039686 | O_anatinus | Animals | mono_intergenic | H/ACA | | | intergenic |
| ENSOANG00000039690 | ENSOANG00000039690 | O_anatinus | Animals | mono_intergenic | H/ACA | | | intergenic |
| ENSOANG00000039692 | ENSOANG00000039692 | O_anatinus | Animals | mono_intergenic | H/ACA | | | intergenic |
| ENSOANG00000039693 | ENSOANG00000039693 | O_anatinus | Animals | mono_intronic | H/ACA | ENSOANG00000040911 | | non_coding |
| ENSOANG00000039696 | ENSOANG00000039696 | O_anatinus | Animals | mono_intergenic | H/ACA | | | intergenic |
| ENSOANG00000039700 | ENSOANG00000039700 | O_anatinus | Animals | mono_intergenic | H/ACA | | | intergenic |
| ENSOANG00000039705 | ENSOANG00000039705 | O_anatinus | Animals | mono_intergenic | H/ACA | | | intergenic |
| ENSOANG00000039706 | ENSOANG00000039706 | O_anatinus | Animals | mono_intergenic | H/ACA | | | intergenic |
| ENSOANG00000039707 | ENSOANG00000039707 | O_anatinus | Animals | mono_intergenic | H/ACA | | | intergenic |
| ENSOANG00000039708 | ENSOANG00000039708 | O_anatinus | Animals | mono_intronic | H/ACA | ENSOANG00000003117 | DENND5A | protein_coding |
| ENSOANG00000039711 | ENSOANG00000039711 | O_anatinus | Animals | mono_intergenic | H/ACA | | | intergenic |
| ENSOANG00000039713 | ENSOANG00000039713 | O_anatinus | Animals | mono_intergenic | H/ACA | | | intergenic |

| | | | | | | | | |
|---|---|---|---|---|---|---|---|---|
| ENSOANG00000039716 | ENSOANG00000039716 | O_anatinus | Animals | mono_intergenic | H/ACA | | | intergenic |
| ENSOANG00000039718 | ENSOANG00000039718 | O_anatinus | Animals | mono_intergenic | H/ACA | | | intergenic |
| ENSOANG00000039723 | ENSOANG00000039723 | O_anatinus | Animals | mono_intergenic | H/ACA | | | intergenic |
| ENSOANG00000039724 | ENSOANG00000039724 | O_anatinus | Animals | mono_intergenic | H/ACA | | | intergenic |
| ENSOANG00000039725 | ENSOANG00000039725 | O_anatinus | Animals | mono_intergenic | H/ACA | | | intergenic |
| ENSOANG00000039728 | ENSOANG00000039728 | O_anatinus | Animals | mono_intergenic | H/ACA | | | intergenic |
| ENSOANG00000039730 | ENSOANG00000039730 | O_anatinus | Animals | mono_intergenic | H/ACA | | | intergenic |
| ENSOANG00000039732 | ENSOANG00000039732 | O_anatinus | Animals | mono_intergenic | H/ACA | | | intergenic |
| ENSOANG00000039733 | ENSOANG00000039733 | O_anatinus | Animals | mono_intergenic | H/ACA | | | intergenic |
| ENSOANG00000039740 | ENSOANG00000039740 | O_anatinus | Animals | mono_intergenic | H/ACA | | | intergenic |
| ENSOANG00000039742 | ENSOANG00000039742 | O_anatinus | Animals | mono_intronic | H/ACA | ENSOANG00000012517 | PGM5 | protein_coding |
| ENSOANG00000039743 | ENSOANG00000039743 | O_anatinus | Animals | mono_intergenic | H/ACA | | | intergenic |
| ENSOANG00000039744 | ENSOANG00000039744 | O_anatinus | Animals | mono_intergenic | H/ACA | | | intergenic |
| ENSOANG00000039745 | ENSOANG00000039745 | O_anatinus | Animals | mono_intergenic | H/ACA | | | intergenic |
| ENSOANG00000039746 | ENSOANG00000039746 | O_anatinus | Animals | intronic_cluster | H/ACA | ENSOANG00000001034 | NTNG1 | protein_coding |
| ENSOANG00000039748 | ENSOANG00000039748 | O_anatinus | Animals | mono_intergenic | H/ACA | | | intergenic |
| ENSOANG00000039749 | ENSOANG00000039749 | O_anatinus | Animals | mono_intergenic | H/ACA | | | intergenic |
| ENSOANG00000039751 | ENSOANG00000039751 | O_anatinus | Animals | mono_intergenic | H/ACA | | | intergenic |
| ENSOANG00000039754 | ENSOANG00000039754 | O_anatinus | Animals | mono_intergenic | H/ACA | | | intergenic |
| ENSOANG00000039755 | ENSOANG00000039755 | O_anatinus | Animals | mono_intronic | H/ACA | ENSOANG00000044717 | TMEM9 | protein_coding |
| ENSOANG00000039757 | ENSOANG00000039757 | O_anatinus | Animals | mono_intronic | H/ACA | ENSOANG00000007021 | ERBB4 | protein_coding |
| ENSOANG00000039758 | ENSOANG00000039758 | O_anatinus | Animals | mono_intergenic | H/ACA | | | intergenic |
| ENSOANG00000039759 | ENSOANG00000039759 | O_anatinus | Animals | mono_intergenic | H/ACA | | | intergenic |
| ENSOANG00000039760 | ENSOANG00000039760 | O_anatinus | Animals | mono_intergenic | H/ACA | | | intergenic |
| ENSOANG00000039761 | ENSOANG00000039761 | O_anatinus | Animals | mono_intergenic | H/ACA | | | intergenic |
| ENSOANG00000039762 | ENSOANG00000039762 | O_anatinus | Animals | intronic_cluster | H/ACA | ENSOANG00000007366 | CNTN1 | protein_coding |
| ENSOANG00000039763 | ENSOANG00000039763 | O_anatinus | Animals | mono_intergenic | H/ACA | | | intergenic |
| ENSOANG00000039764 | ENSOANG00000039764 | O_anatinus | Animals | intronic_cluster | H/ACA | ENSOANG00000043336 | | protein_coding |
| ENSOANG00000039766 | ENSOANG00000039766 | O_anatinus | Animals | mono_intronic | H/ACA | ENSOANG00000002498 | CDH18 | protein_coding |
| ENSOANG00000039767 | ENSOANG00000039767 | O_anatinus | Animals | mono_intergenic | H/ACA | | | intergenic |
| ENSOANG00000039769 | ENSOANG00000039769 | O_anatinus | Animals | mono_intronic | H/ACA | ENSOANG00000011733 | SLC9A9 | protein_coding |
| ENSOANG00000039770 | ENSOANG00000039770 | O_anatinus | Animals | mono_intergenic | H/ACA | | | intergenic |
| ENSOANG00000039771 | ENSOANG00000039771 | O_anatinus | Animals | mono_intergenic | H/ACA | | | intergenic |
| ENSOANG00000039772 | ENSOANG00000039772 | O_anatinus | Animals | mono_intergenic | H/ACA | | | intergenic |
| ENSOANG00000039774 | ENSOANG00000039774 | O_anatinus | Animals | mono_intergenic | H/ACA | | | intergenic |
| ENSOANG00000039775 | ENSOANG00000039775 | O_anatinus | Animals | intergenic_cluster | H/ACA | | | intergenic |
| ENSOANG00000039778 | ENSOANG00000039778 | O_anatinus | Animals | mono_intronic | H/ACA | ENSOANG00000041359 | | protein_coding |
| ENSOANG00000039779 | ENSOANG00000039779 | O_anatinus | Animals | mono_intergenic | H/ACA | | | intergenic |
| ENSOANG00000039781 | ENSOANG00000039781 | O_anatinus | Animals | mono_intergenic | H/ACA | | | intergenic |
| ENSOANG00000039783 | ENSOANG00000039783 | O_anatinus | Animals | mono_intergenic | H/ACA | | | intergenic |
| ENSOANG00000039789 | ENSOANG00000039789 | O_anatinus | Animals | mono_intergenic | H/ACA | | | intergenic |

| | | | | | | | | |
|---|---|---|---|---|---|---|---|---|
| ENSOANG00000039792 | ENSOANG00000039792 | O_anatinus | Animals | mono_intergenic | H/ACA | | | intergenic |
| ENSOANG00000039797 | ENSOANG00000039797 | O_anatinus | Animals | intronic_cluster | H/ACA | ENSOANG00000011147 | ST6GAL2 | protein_coding |
| ENSOANG00000039800 | ENSOANG00000039800 | O_anatinus | Animals | intronic_cluster | H/ACA | ENSOANG00000049221 | SEPTIN10 | protein_coding |
| ENSOANG00000039802 | ENSOANG00000039802 | O_anatinus | Animals | mono_intergenic | H/ACA | | | intergenic |
| ENSOANG00000039803 | ENSOANG00000039803 | O_anatinus | Animals | mono_intergenic | H/ACA | | | intergenic |
| ENSOANG00000039805 | ENSOANG00000039805 | O_anatinus | Animals | mono_intergenic | H/ACA | | | intergenic |
| ENSOANG00000039808 | ENSOANG00000039808 | O_anatinus | Animals | mono_intergenic | H/ACA | | | intergenic |
| ENSOANG00000039814 | ENSOANG00000039814 | O_anatinus | Animals | mono_intergenic | H/ACA | | | intergenic |
| ENSOANG00000039816 | ENSOANG00000039816 | O_anatinus | Animals | mono_intergenic | H/ACA | | | intergenic |
| ENSOANG00000039818 | ENSOANG00000039818 | O_anatinus | Animals | mono_intergenic | H/ACA | | | intergenic |
| ENSOANG00000039819 | ENSOANG00000039819 | O_anatinus | Animals | mono_intergenic | H/ACA | | | intergenic |
| ENSOANG00000039821 | ENSOANG00000039821 | O_anatinus | Animals | mono_intergenic | H/ACA | | | intergenic |
| ENSOANG00000039822 | ENSOANG00000039822 | O_anatinus | Animals | mono_intergenic | H/ACA | | | intergenic |
| ENSOANG00000039825 | ENSOANG00000039825 | O_anatinus | Animals | mono_intergenic | H/ACA | | | intergenic |
| ENSOANG00000039828 | ENSOANG00000039828 | O_anatinus | Animals | mono_intronic | H/ACA | ENSOANG00000003242 | | protein_coding |
| ENSOANG00000039829 | ENSOANG00000039829 | O_anatinus | Animals | mono_intergenic | H/ACA | | | intergenic |
| ENSOANG00000039831 | ENSOANG00000039831 | O_anatinus | Animals | mono_intergenic | H/ACA | | | intergenic |
| ENSOANG00000039839 | ENSOANG00000039839 | O_anatinus | Animals | mono_intergenic | H/ACA | | | intergenic |
| ENSOANG00000039841 | ENSOANG00000039841 | O_anatinus | Animals | intronic_cluster | H/ACA | ENSOANG00000029079 | | protein_coding |
| ENSOANG00000039843 | ENSOANG00000039843 | O_anatinus | Animals | mono_intergenic | H/ACA | | | intergenic |
| ENSOANG00000039847 | ENSOANG00000039847 | O_anatinus | Animals | mono_intergenic | H/ACA | | | intergenic |
| ENSOANG00000039848 | ENSOANG00000039848 | O_anatinus | Animals | mono_intronic | H/ACA | ENSOANG00000004069 | SH3GL3 | protein_coding |
| ENSOANG00000039849 | ENSOANG00000039849 | O_anatinus | Animals | mono_intergenic | H/ACA | | | intergenic |
| ENSOANG00000039854 | ENSOANG00000039854 | O_anatinus | Animals | mono_intergenic | H/ACA | | | intergenic |
| ENSOANG00000039857 | ENSOANG00000039857 | O_anatinus | Animals | mono_intergenic | H/ACA | | | intergenic |
| ENSOANG00000039860 | ENSOANG00000039860 | O_anatinus | Animals | intronic_cluster | H/ACA | ENSOANG00000040957 | | non_coding |
| ENSOANG00000039866 | ENSOANG00000039866 | O_anatinus | Animals | mono_intergenic | H/ACA | | | intergenic |
| ENSOANG00000039868 | ENSOANG00000039868 | O_anatinus | Animals | mono_intergenic | H/ACA | | | intergenic |
| ENSOANG00000039870 | ENSOANG00000039870 | O_anatinus | Animals | mono_intergenic | H/ACA | | | intergenic |
| ENSOANG00000039872 | ENSOANG00000039872 | O_anatinus | Animals | mono_intergenic | H/ACA | | | intergenic |
| ENSOANG00000039874 | ENSOANG00000039874 | O_anatinus | Animals | mono_intergenic | H/ACA | | | intergenic |
| ENSOANG00000039876 | ENSOANG00000039876 | O_anatinus | Animals | mono_intergenic | H/ACA | | | intergenic |
| ENSOANG00000039883 | ENSOANG00000039883 | O_anatinus | Animals | mono_intergenic | H/ACA | | | intergenic |
| ENSOANG00000039884 | ENSOANG00000039884 | O_anatinus | Animals | mono_intergenic | H/ACA | | | intergenic |
| ENSOANG00000039892 | ENSOANG00000039892 | O_anatinus | Animals | mono_intergenic | H/ACA | | | intergenic |
| ENSOANG00000039895 | ENSOANG00000039895 | O_anatinus | Animals | mono_intergenic | H/ACA | | | intergenic |
| ENSOANG00000039896 | ENSOANG00000039896 | O_anatinus | Animals | mono_intergenic | H/ACA | | | intergenic |
| ENSOANG00000039897 | ENSOANG00000039897 | O_anatinus | Animals | mono_intergenic | H/ACA | | | intergenic |
| ENSOANG00000039899 | ENSOANG00000039899 | O_anatinus | Animals | mono_intergenic | H/ACA | | | intergenic |
| ENSOANG00000039903 | ENSOANG00000039903 | O_anatinus | Animals | mono_intergenic | H/ACA | | | intergenic |
| ENSOANG00000039905 | ENSOANG00000039905 | O_anatinus | Animals | mono_intergenic | H/ACA | | | intergenic |

| | | | | | | | | |
|---|---|---|---|---|---|---|---|---|
| ENSOANG00000039906 | ENSOANG00000039906 | O_anatinus | Animals | mono_intergenic | H/ACA | | | intergenic |
| ENSOANG00000039907 | ENSOANG00000039907 | O_anatinus | Animals | mono_intergenic | H/ACA | | | intergenic |
| ENSOANG00000039908 | ENSOANG00000039908 | O_anatinus | Animals | mono_intergenic | H/ACA | | | intergenic |
| ENSOANG00000039912 | ENSOANG00000039912 | O_anatinus | Animals | mono_intergenic | H/ACA | | | intergenic |
| ENSOANG00000039931 | ENSOANG00000039931 | O_anatinus | Animals | mono_intergenic | H/ACA | | | intergenic |
| ENSOANG00000039937 | ENSOANG00000039937 | O_anatinus | Animals | mono_intergenic | H/ACA | | | intergenic |
| ENSOANG00000039938 | ENSOANG00000039938 | O_anatinus | Animals | mono_intergenic | H/ACA | | | intergenic |
| ENSOANG00000039939 | ENSOANG00000039939 | O_anatinus | Animals | mono_intergenic | H/ACA | | | intergenic |
| ENSOANG00000039940 | ENSOANG00000039940 | O_anatinus | Animals | mono_intergenic | H/ACA | | | intergenic |
| ENSOANG00000039941 | ENSOANG00000039941 | O_anatinus | Animals | mono_intergenic | H/ACA | | | intergenic |
| ENSOANG00000039943 | ENSOANG00000039943 | O_anatinus | Animals | mono_intergenic | H/ACA | | | intergenic |
| ENSOANG00000039944 | ENSOANG00000039944 | O_anatinus | Animals | mono_intronic | H/ACA | ENSOANG00000011283 | | protein_coding |
| ENSOANG00000039945 | ENSOANG00000039945 | O_anatinus | Animals | mono_intergenic | H/ACA | | | intergenic |
| ENSOANG00000039947 | ENSOANG00000039947 | O_anatinus | Animals | mono_intergenic | H/ACA | | | intergenic |
| ENSOANG00000039952 | ENSOANG00000039952 | O_anatinus | Animals | mono_intergenic | H/ACA | | | intergenic |
| ENSOANG00000039958 | ENSOANG00000039958 | O_anatinus | Animals | mono_intergenic | H/ACA | | | intergenic |
| ENSOANG00000039959 | ENSOANG00000039959 | O_anatinus | Animals | mono_intergenic | H/ACA | | | intergenic |
| ENSOANG00000039963 | ENSOANG00000039963 | O_anatinus | Animals | mono_intergenic | H/ACA | | | intergenic |
| ENSOANG00000039966 | ENSOANG00000039966 | O_anatinus | Animals | mono_intergenic | H/ACA | | | intergenic |
| ENSOANG00000039970 | ENSOANG00000039970 | O_anatinus | Animals | mono_intergenic | H/ACA | | | intergenic |
| ENSOANG00000039973 | ENSOANG00000039973 | O_anatinus | Animals | mono_intergenic | H/ACA | | | intergenic |
| ENSOANG00000039975 | ENSOANG00000039975 | O_anatinus | Animals | mono_intergenic | H/ACA | | | intergenic |
| ENSOANG00000039976 | ENSOANG00000039976 | O_anatinus | Animals | mono_intergenic | H/ACA | | | intergenic |
| ENSOANG00000039978 | ENSOANG00000039978 | O_anatinus | Animals | mono_intergenic | H/ACA | | | intergenic |
| ENSOANG00000039980 | ENSOANG00000039980 | O_anatinus | Animals | mono_intergenic | H/ACA | | | intergenic |
| ENSOANG00000039982 | ENSOANG00000039982 | O_anatinus | Animals | mono_intergenic | H/ACA | | | intergenic |
| ENSOANG00000039986 | ENSOANG00000039986 | O_anatinus | Animals | mono_intergenic | H/ACA | | | intergenic |
| ENSOANG00000039987 | ENSOANG00000039987 | O_anatinus | Animals | mono_intergenic | H/ACA | | | intergenic |
| ENSOANG00000039994 | ENSOANG00000039994 | O_anatinus | Animals | mono_intergenic | H/ACA | | | intergenic |
| ENSOANG00000039997 | ENSOANG00000039997 | O_anatinus | Animals | mono_intergenic | H/ACA | | | intergenic |
| ENSOANG00000039999 | ENSOANG00000039999 | O_anatinus | Animals | mono_intergenic | H/ACA | | | intergenic |
| ENSOANG00000040001 | ENSOANG00000040001 | O_anatinus | Animals | mono_intergenic | H/ACA | | | intergenic |
| ENSOANG00000040005 | ENSOANG00000040005 | O_anatinus | Animals | mono_intergenic | H/ACA | | | intergenic |
| ENSOANG00000040011 | ENSOANG00000040011 | O_anatinus | Animals | mono_intergenic | H/ACA | | | intergenic |
| ENSOANG00000040012 | ENSOANG00000040012 | O_anatinus | Animals | mono_intergenic | H/ACA | | | intergenic |
| ENSOANG00000040017 | ENSOANG00000040017 | O_anatinus | Animals | mono_intronic | H/ACA | ENSOANG00000039613 | ENPP3 | protein_coding |
| ENSOANG00000040018 | ENSOANG00000040018 | O_anatinus | Animals | mono_intergenic | H/ACA | | | intergenic |
| ENSOANG00000040021 | ENSOANG00000040021 | O_anatinus | Animals | mono_intergenic | H/ACA | | | intergenic |
| ENSOANG00000040022 | ENSOANG00000040022 | O_anatinus | Animals | mono_intergenic | H/ACA | | | intergenic |
| ENSOANG00000040023 | ENSOANG00000040023 | O_anatinus | Animals | mono_intergenic | H/ACA | | | intergenic |
| ENSOANG00000040028 | ENSOANG00000040028 | O_anatinus | Animals | mono_intergenic | H/ACA | | | intergenic |

| | | | | | | | | |
|---|---|---|---|---|---|---|---|---|
| ENSOANG00000040031 | ENSOANG00000040031 | O_anatinus | Animals | mono_intergenic | H/ACA | | | intergenic |
| ENSOANG00000040032 | ENSOANG00000040032 | O_anatinus | Animals | mono_intergenic | H/ACA | | | intergenic |
| ENSOANG00000040035 | ENSOANG00000040035 | O_anatinus | Animals | mono_intergenic | H/ACA | | | intergenic |
| ENSOANG00000040040 | ENSOANG00000040040 | O_anatinus | Animals | mono_intergenic | H/ACA | | | intergenic |
| ENSOANG00000040042 | ENSOANG00000040042 | O_anatinus | Animals | mono_intergenic | H/ACA | | | intergenic |
| ENSOANG00000040044 | ENSOANG00000040044 | O_anatinus | Animals | mono_intergenic | H/ACA | | | intergenic |
| ENSOANG00000040048 | ENSOANG00000040048 | O_anatinus | Animals | mono_intergenic | H/ACA | | | intergenic |
| ENSOANG00000040050 | ENSOANG00000040050 | O_anatinus | Animals | mono_intergenic | H/ACA | | | intergenic |
| ENSOANG00000040051 | ENSOANG00000040051 | O_anatinus | Animals | mono_intergenic | H/ACA | | | intergenic |
| ENSOANG00000040052 | ENSOANG00000040052 | O_anatinus | Animals | mono_intergenic | H/ACA | | | intergenic |
| ENSOANG00000040054 | ENSOANG00000040054 | O_anatinus | Animals | mono_intergenic | H/ACA | | | intergenic |
| ENSOANG00000040056 | ENSOANG00000040056 | O_anatinus | Animals | mono_intronic | H/ACA | ENSOANG00000000241 | | protein_coding |
| ENSOANG00000040057 | ENSOANG00000040057 | O_anatinus | Animals | mono_intergenic | H/ACA | | | intergenic |
| ENSOANG00000040058 | ENSOANG00000040058 | O_anatinus | Animals | mono_intergenic | H/ACA | | | intergenic |
| ENSOANG00000040064 | ENSOANG00000040064 | O_anatinus | Animals | mono_intergenic | H/ACA | | | intergenic |
| ENSOANG00000040068 | ENSOANG00000040068 | O_anatinus | Animals | mono_intergenic | H/ACA | | | intergenic |
| ENSOANG00000040072 | ENSOANG00000040072 | O_anatinus | Animals | mono_intergenic | H/ACA | | | intergenic |
| ENSOANG00000040074 | ENSOANG00000040074 | O_anatinus | Animals | mono_intergenic | H/ACA | | | intergenic |
| ENSOANG00000040075 | ENSOANG00000040075 | O_anatinus | Animals | mono_intergenic | H/ACA | | | intergenic |
| ENSOANG00000040082 | ENSOANG00000040082 | O_anatinus | Animals | mono_intergenic | H/ACA | | | intergenic |
| ENSOANG00000040088 | ENSOANG00000040088 | O_anatinus | Animals | mono_intergenic | H/ACA | | | intergenic |
| ENSOANG00000040090 | ENSOANG00000040090 | O_anatinus | Animals | mono_intergenic | H/ACA | | | intergenic |
| ENSOANG00000040092 | ENSOANG00000040092 | O_anatinus | Animals | mono_intergenic | H/ACA | | | intergenic |
| ENSOANG00000040094 | ENSOANG00000040094 | O_anatinus | Animals | mono_intronic | H/ACA | ENSOANG00000000134 | IGF1R | protein_coding |
| ENSOANG00000040097 | ENSOANG00000040097 | O_anatinus | Animals | mono_intergenic | H/ACA | | | intergenic |
| ENSOANG00000040099 | ENSOANG00000040099 | O_anatinus | Animals | mono_intergenic | H/ACA | | | intergenic |
| ENSOANG00000040102 | ENSOANG00000040102 | O_anatinus | Animals | mono_intergenic | H/ACA | | | intergenic |
| ENSOANG00000040103 | ENSOANG00000040103 | O_anatinus | Animals | mono_intergenic | H/ACA | | | intergenic |
| ENSOANG00000040106 | ENSOANG00000040106 | O_anatinus | Animals | mono_intergenic | H/ACA | | | intergenic |
| ENSOANG00000040108 | ENSOANG00000040108 | O_anatinus | Animals | mono_intergenic | H/ACA | | | intergenic |
| ENSOANG00000040109 | ENSOANG00000040109 | O_anatinus | Animals | mono_intergenic | H/ACA | | | intergenic |
| ENSOANG00000040116 | ENSOANG00000040116 | O_anatinus | Animals | mono_intergenic | H/ACA | | | intergenic |
| ENSOANG00000040118 | ENSOANG00000040118 | O_anatinus | Animals | mono_intronic | H/ACA | ENSOANG00000050125 | GBX1 | protein_coding |
| ENSOANG00000040121 | ENSOANG00000040121 | O_anatinus | Animals | mono_intronic | C/D | ENSOANG00000047231 | ZNF711 | protein_coding |
| ENSOANG00000040126 | ENSOANG00000040126 | O_anatinus | Animals | mono_intronic | H/ACA | ENSOANG00000044322 | ATXN7 | protein_coding |
| ENSOANG00000040127 | ENSOANG00000040127 | O_anatinus | Animals | mono_intergenic | H/ACA | | | intergenic |
| ENSOANG00000040135 | ENSOANG00000040135 | O_anatinus | Animals | mono_intergenic | H/ACA | | | intergenic |
| ENSOANG00000040137 | ENSOANG00000040137 | O_anatinus | Animals | mono_intergenic | H/ACA | | | intergenic |
| ENSOANG00000040143 | ENSOANG00000040143 | O_anatinus | Animals | mono_intronic | H/ACA | ENSOANG00000043766 | | protein_coding |
| ENSOANG00000040145 | ENSOANG00000040145 | O_anatinus | Animals | mono_intergenic | H/ACA | | | intergenic |
| ENSOANG00000040146 | ENSOANG00000040146 | O_anatinus | Animals | mono_intergenic | H/ACA | | | intergenic |

| | | | | | | | | |
|---|---|---|---|---|---|---|---|---|
| ENSOANG00000040149 | ENSOANG00000040149 | O_anatinus | Animals | mono_intergenic | H/ACA | | | intergenic |
| ENSOANG00000040150 | ENSOANG00000040150 | O_anatinus | Animals | mono_intergenic | H/ACA | | | intergenic |
| ENSOANG00000040151 | ENSOANG00000040151 | O_anatinus | Animals | mono_intergenic | H/ACA | | | intergenic |
| ENSOANG00000040155 | ENSOANG00000040155 | O_anatinus | Animals | mono_intergenic | H/ACA | | | intergenic |
| ENSOANG00000040156 | ENSOANG00000040156 | O_anatinus | Animals | mono_intergenic | H/ACA | | | intergenic |
| ENSOANG00000040157 | ENSOANG00000040157 | O_anatinus | Animals | mono_intergenic | H/ACA | | | intergenic |
| ENSOANG00000040161 | ENSOANG00000040161 | O_anatinus | Animals | mono_intergenic | H/ACA | | | intergenic |
| ENSOANG00000040162 | ENSOANG00000040162 | O_anatinus | Animals | mono_intergenic | H/ACA | | | intergenic |
| ENSOANG00000040163 | ENSOANG00000040163 | O_anatinus | Animals | mono_intergenic | H/ACA | | | intergenic |
| ENSOANG00000040164 | ENSOANG00000040164 | O_anatinus | Animals | mono_intergenic | H/ACA | | | intergenic |
| ENSOANG00000040165 | ENSOANG00000040165 | O_anatinus | Animals | mono_intergenic | H/ACA | | | intergenic |
| ENSOANG00000040166 | ENSOANG00000040166 | O_anatinus | Animals | mono_intergenic | H/ACA | | | intergenic |
| ENSOANG00000040169 | ENSOANG00000040169 | O_anatinus | Animals | mono_intergenic | H/ACA | | | intergenic |
| ENSOANG00000040170 | ENSOANG00000040170 | O_anatinus | Animals | mono_intronic | H/ACA | ENSOANG00000041406 | FGF13 | protein_coding |
| ENSOANG00000040172 | ENSOANG00000040172 | O_anatinus | Animals | mono_intergenic | H/ACA | | | intergenic |
| ENSOANG00000040173 | ENSOANG00000040173 | O_anatinus | Animals | mono_intergenic | H/ACA | | | intergenic |
| ENSOANG00000040175 | ENSOANG00000040175 | O_anatinus | Animals | mono_intergenic | H/ACA | | | intergenic |
| ENSOANG00000040182 | ENSOANG00000040182 | O_anatinus | Animals | mono_intergenic | H/ACA | | | intergenic |
| ENSOANG00000040183 | ENSOANG00000040183 | O_anatinus | Animals | mono_intergenic | H/ACA | | | intergenic |
| ENSOANG00000040185 | ENSOANG00000040185 | O_anatinus | Animals | mono_intergenic | H/ACA | | | intergenic |
| ENSOANG00000040188 | ENSOANG00000040188 | O_anatinus | Animals | intronic_cluster | H/ACA | ENSOANG00000001081 | | protein_coding |
| ENSOANG00000040190 | ENSOANG00000040190 | O_anatinus | Animals | mono_intergenic | H/ACA | | | intergenic |
| ENSOANG00000040192 | ENSOANG00000040192 | O_anatinus | Animals | mono_intronic | H/ACA | ENSOANG00000045136 | SUPT3H | protein_coding |
| ENSOANG00000040194 | ENSOANG00000040194 | O_anatinus | Animals | mono_intronic | H/ACA | ENSOANG00000009129 | RPL18A | protein_coding |
| ENSOANG00000040195 | ENSOANG00000040195 | O_anatinus | Animals | mono_intergenic | H/ACA | | | intergenic |
| ENSOANG00000040200 | ENSOANG00000040200 | O_anatinus | Animals | mono_intergenic | H/ACA | | | intergenic |
| ENSOANG00000040203 | ENSOANG00000040203 | O_anatinus | Animals | mono_intergenic | H/ACA | | | intergenic |
| ENSOANG00000040208 | ENSOANG00000040208 | O_anatinus | Animals | mono_intergenic | H/ACA | | | intergenic |
| ENSOANG00000040209 | ENSOANG00000040209 | O_anatinus | Animals | mono_intergenic | H/ACA | | | intergenic |
| ENSOANG00000040215 | ENSOANG00000040215 | O_anatinus | Animals | mono_intergenic | H/ACA | | | intergenic |
| ENSOANG00000040217 | ENSOANG00000040217 | O_anatinus | Animals | mono_intergenic | H/ACA | | | intergenic |
| ENSOANG00000040221 | ENSOANG00000040221 | O_anatinus | Animals | mono_intergenic | H/ACA | | | intergenic |
| ENSOANG00000040224 | ENSOANG00000040224 | O_anatinus | Animals | mono_intergenic | H/ACA | | | intergenic |
| ENSOANG00000040225 | ENSOANG00000040225 | O_anatinus | Animals | mono_intergenic | H/ACA | | | intergenic |
| ENSOANG00000040226 | ENSOANG00000040226 | O_anatinus | Animals | mono_intergenic | H/ACA | | | intergenic |
| ENSOANG00000040227 | ENSOANG00000040227 | O_anatinus | Animals | mono_intergenic | H/ACA | | | intergenic |
| ENSOANG00000040231 | ENSOANG00000040231 | O_anatinus | Animals | intronic_cluster | H/ACA | ENSOANG00000003052 | KCNC1 | protein_coding |
| ENSOANG00000040232 | ENSOANG00000040232 | O_anatinus | Animals | mono_intergenic | H/ACA | | | intergenic |
| ENSOANG00000040233 | ENSOANG00000040233 | O_anatinus | Animals | mono_intronic | H/ACA | ENSOANG00000012869 | | protein_coding |
| ENSOANG00000040235 | ENSOANG00000040235 | O_anatinus | Animals | mono_intergenic | H/ACA | | | intergenic |
| ENSOANG00000040236 | ENSOANG00000040236 | O_anatinus | Animals | mono_intergenic | H/ACA | | | intergenic |

| | | | | | | | | |
|---|---|---|---|---|---|---|---|---|
| ENSOANG00000040237 | ENSOANG00000040237 | O_anatinus | Animals | mono_intergenic | H/ACA | | | intergenic |
| ENSOANG00000040244 | ENSOANG00000040244 | O_anatinus | Animals | mono_intergenic | H/ACA | | | intergenic |
| ENSOANG00000040245 | ENSOANG00000040245 | O_anatinus | Animals | mono_intergenic | H/ACA | | | intergenic |
| ENSOANG00000040246 | ENSOANG00000040246 | O_anatinus | Animals | mono_intergenic | H/ACA | | | intergenic |
| ENSOANG00000040247 | ENSOANG00000040247 | O_anatinus | Animals | intronic_cluster | H/ACA | ENSOANG00000021517 | AK5 | protein_coding |
| ENSOANG00000040248 | ENSOANG00000040248 | O_anatinus | Animals | mono_intergenic | H/ACA | | | intergenic |
| ENSOANG00000040253 | ENSOANG00000040253 | O_anatinus | Animals | mono_intergenic | H/ACA | | | intergenic |
| ENSOANG00000040255 | ENSOANG00000040255 | O_anatinus | Animals | mono_intergenic | H/ACA | | | intergenic |
| ENSOANG00000040257 | ENSOANG00000040257 | O_anatinus | Animals | mono_intergenic | H/ACA | | | intergenic |
| ENSOANG00000040261 | ENSOANG00000040261 | O_anatinus | Animals | mono_intergenic | H/ACA | | | intergenic |
| ENSOANG00000040263 | ENSOANG00000040263 | O_anatinus | Animals | mono_intergenic | H/ACA | | | intergenic |
| ENSOANG00000040271 | ENSOANG00000040271 | O_anatinus | Animals | mono_intergenic | H/ACA | | | intergenic |
| ENSOANG00000040272 | ENSOANG00000040272 | O_anatinus | Animals | mono_intronic | H/ACA | ENSOANG00000044706 | ARHGEF26 | protein_coding |
| ENSOANG00000040276 | ENSOANG00000040276 | O_anatinus | Animals | mono_intergenic | H/ACA | | | intergenic |
| ENSOANG00000040277 | ENSOANG00000040277 | O_anatinus | Animals | mono_intronic | H/ACA | ENSOANG00000015075 | MAGI2 | protein_coding |
| ENSOANG00000040278 | ENSOANG00000040278 | O_anatinus | Animals | mono_intronic | H/ACA | ENSOANG00000007132 | HNF1B | protein_coding |
| ENSOANG00000040279 | ENSOANG00000040279 | O_anatinus | Animals | mono_intergenic | H/ACA | | | intergenic |
| ENSOANG00000040280 | ENSOANG00000040280 | O_anatinus | Animals | mono_intergenic | H/ACA | | | intergenic |
| ENSOANG00000040281 | ENSOANG00000040281 | O_anatinus | Animals | mono_intergenic | H/ACA | | | intergenic |
| ENSOANG00000040284 | ENSOANG00000040284 | O_anatinus | Animals | mono_intronic | H/ACA | ENSOANG00000047733 | | non_coding |
| ENSOANG00000040287 | ENSOANG00000040287 | O_anatinus | Animals | mono_intergenic | H/ACA | | | intergenic |
| ENSOANG00000040288 | ENSOANG00000040288 | O_anatinus | Animals | mono_intergenic | H/ACA | | | intergenic |
| ENSOANG00000040289 | ENSOANG00000040289 | O_anatinus | Animals | mono_intergenic | H/ACA | | | intergenic |
| ENSOANG00000040293 | ENSOANG00000040293 | O_anatinus | Animals | mono_intergenic | H/ACA | | | intergenic |
| ENSOANG00000040295 | ENSOANG00000040295 | O_anatinus | Animals | mono_intergenic | H/ACA | | | intergenic |
| ENSOANG00000040297 | ENSOANG00000040297 | O_anatinus | Animals | mono_intronic | H/ACA | ENSOANG00000051348 | EGFLAM | protein_coding |
| ENSOANG00000040304 | ENSOANG00000040304 | O_anatinus | Animals | mono_intergenic | H/ACA | | | intergenic |
| ENSOANG00000040305 | ENSOANG00000040305 | O_anatinus | Animals | mono_intronic | H/ACA | ENSOANG00000005257 | IL20RA | protein_coding |
| ENSOANG00000040310 | ENSOANG00000040310 | O_anatinus | Animals | mono_intergenic | H/ACA | | | intergenic |
| ENSOANG00000040311 | ENSOANG00000040311 | O_anatinus | Animals | mono_intergenic | H/ACA | | | intergenic |
| ENSOANG00000040313 | ENSOANG00000040313 | O_anatinus | Animals | intergenic_cluster | H/ACA | | | intergenic |
| ENSOANG00000040314 | ENSOANG00000040314 | O_anatinus | Animals | mono_intergenic | H/ACA | | | intergenic |
| ENSOANG00000040320 | SNORD34 | O_anatinus | Animals | mono_intronic | C/D | ENSOANG00000000828 | RPL13A | protein_coding |
| ENSOANG00000040323 | ENSOANG00000040323 | O_anatinus | Animals | mono_intronic | H/ACA | ENSOANG00000043264 | ALDH4A1 | protein_coding |
| ENSOANG00000040324 | ENSOANG00000040324 | O_anatinus | Animals | intronic_cluster | H/ACA | ENSOANG00000044930 | PARK7 | protein_coding |
| ENSOANG00000040326 | ENSOANG00000040326 | O_anatinus | Animals | mono_intergenic | H/ACA | | | intergenic |
| ENSOANG00000040327 | ENSOANG00000040327 | O_anatinus | Animals | mono_intergenic | H/ACA | | | intergenic |
| ENSOANG00000040331 | ENSOANG00000040331 | O_anatinus | Animals | mono_intergenic | H/ACA | | | intergenic |
| ENSOANG00000040335 | ENSOANG00000040335 | O_anatinus | Animals | mono_intergenic | H/ACA | | | intergenic |
| ENSOANG00000040339 | ENSOANG00000040339 | O_anatinus | Animals | mono_intergenic | H/ACA | | | intergenic |
| ENSOANG00000040341 | ENSOANG00000040341 | O_anatinus | Animals | mono_intergenic | H/ACA | | | intergenic |

| | | | | | | | | |
|---|---|---|---|---|---|---|---|---|
| ENSOANG00000040343 | ENSOANG00000040343 | O_anatinus | Animals | mono_intergenic | H/ACA | | | intergenic |
| ENSOANG00000040344 | ENSOANG00000040344 | O_anatinus | Animals | mono_intergenic | H/ACA | | | intergenic |
| ENSOANG00000040347 | ENSOANG00000040347 | O_anatinus | Animals | mono_intergenic | H/ACA | | | intergenic |
| ENSOANG00000040354 | ENSOANG00000040354 | O_anatinus | Animals | mono_intergenic | H/ACA | | | intergenic |
| ENSOANG00000040355 | ENSOANG00000040355 | O_anatinus | Animals | mono_intergenic | H/ACA | | | intergenic |
| ENSOANG00000040356 | ENSOANG00000040356 | O_anatinus | Animals | mono_intergenic | H/ACA | | | intergenic |
| ENSOANG00000040357 | ENSOANG00000040357 | O_anatinus | Animals | mono_intergenic | H/ACA | | | intergenic |
| ENSOANG00000040361 | ENSOANG00000040361 | O_anatinus | Animals | mono_intergenic | H/ACA | | | intergenic |
| ENSOANG00000040365 | ENSOANG00000040365 | O_anatinus | Animals | mono_intergenic | H/ACA | | | intergenic |
| ENSOANG00000040367 | ENSOANG00000040367 | O_anatinus | Animals | mono_intergenic | H/ACA | | | intergenic |
| ENSOANG00000040369 | ENSOANG00000040369 | O_anatinus | Animals | mono_intergenic | H/ACA | | | intergenic |
| ENSOANG00000040370 | ENSOANG00000040370 | O_anatinus | Animals | mono_intronic | H/ACA | ENSOANG00000005624 | DSC1 | protein_coding |
| ENSOANG00000040373 | ENSOANG00000040373 | O_anatinus | Animals | mono_intergenic | H/ACA | | | intergenic |
| ENSOANG00000040376 | ENSOANG00000040376 | O_anatinus | Animals | mono_intergenic | H/ACA | | | intergenic |
| ENSOANG00000040382 | ENSOANG00000040382 | O_anatinus | Animals | mono_intergenic | H/ACA | | | intergenic |
| ENSOANG00000040385 | ENSOANG00000040385 | O_anatinus | Animals | mono_intergenic | H/ACA | | | intergenic |
| ENSOANG00000040388 | ENSOANG00000040388 | O_anatinus | Animals | mono_intergenic | H/ACA | | | intergenic |
| ENSOANG00000040390 | ENSOANG00000040390 | O_anatinus | Animals | mono_intronic | H/ACA | ENSOANG00000045011 | FAM83H | protein_coding |
| ENSOANG00000040394 | ENSOANG00000040394 | O_anatinus | Animals | mono_intergenic | H/ACA | | | intergenic |
| ENSOANG00000040396 | ENSOANG00000040396 | O_anatinus | Animals | mono_intergenic | H/ACA | | | intergenic |
| ENSOANG00000040397 | ENSOANG00000040397 | O_anatinus | Animals | mono_intergenic | H/ACA | | | intergenic |
| ENSOANG00000040400 | ENSOANG00000040400 | O_anatinus | Animals | mono_intergenic | H/ACA | | | intergenic |
| ENSOANG00000040406 | ENSOANG00000040406 | O_anatinus | Animals | mono_intergenic | H/ACA | | | intergenic |
| ENSOANG00000040413 | ENSOANG00000040413 | O_anatinus | Animals | mono_intergenic | H/ACA | | | intergenic |
| ENSOANG00000040415 | ENSOANG00000040415 | O_anatinus | Animals | mono_intergenic | H/ACA | | | intergenic |
| ENSOANG00000040417 | ENSOANG00000040417 | O_anatinus | Animals | mono_intronic | H/ACA | ENSOANG00000040243 | | protein_coding |
| ENSOANG00000040429 | ENSOANG00000040429 | O_anatinus | Animals | mono_intergenic | H/ACA | | | intergenic |
| ENSOANG00000040431 | ENSOANG00000040431 | O_anatinus | Animals | mono_intergenic | H/ACA | | | intergenic |
| ENSOANG00000040434 | ENSOANG00000040434 | O_anatinus | Animals | mono_intergenic | H/ACA | | | intergenic |
| ENSOANG00000040439 | ENSOANG00000040439 | O_anatinus | Animals | mono_intergenic | H/ACA | | | intergenic |
| ENSOANG00000040440 | ENSOANG00000040440 | O_anatinus | Animals | mono_intergenic | H/ACA | | | intergenic |
| ENSOANG00000040442 | ENSOANG00000040442 | O_anatinus | Animals | mono_intergenic | H/ACA | | | intergenic |
| ENSOANG00000040444 | ENSOANG00000040444 | O_anatinus | Animals | mono_intergenic | H/ACA | | | intergenic |
| ENSOANG00000040445 | ENSOANG00000040445 | O_anatinus | Animals | mono_intergenic | H/ACA | | | intergenic |
| ENSOANG00000040446 | ENSOANG00000040446 | O_anatinus | Animals | mono_intergenic | H/ACA | | | intergenic |
| ENSOANG00000040451 | ENSOANG00000040451 | O_anatinus | Animals | mono_intronic | H/ACA | ENSOANG00000048135 | CADM1 | protein_coding |
| ENSOANG00000040453 | ENSOANG00000040453 | O_anatinus | Animals | mono_intergenic | H/ACA | | | intergenic |
| ENSOANG00000040455 | ENSOANG00000040455 | O_anatinus | Animals | mono_intergenic | H/ACA | | | intergenic |
| ENSOANG00000040458 | ENSOANG00000040458 | O_anatinus | Animals | mono_intergenic | H/ACA | | | intergenic |
| ENSOANG00000040459 | ENSOANG00000040459 | O_anatinus | Animals | mono_intergenic | H/ACA | | | intergenic |
| ENSOANG00000040461 | ENSOANG00000040461 | O_anatinus | Animals | mono_intergenic | H/ACA | | | intergenic |

| | | | | | | | | |
|---|---|---|---|---|---|---|---|---|
| ENSOANG00000040473 | ENSOANG00000040473 | O_anatinus | Animals | mono_intergenic | H/ACA | | | intergenic |
| ENSOANG00000040474 | ENSOANG00000040474 | O_anatinus | Animals | mono_intronic | H/ACA | ENSOANG00000012345 | RORA | protein_coding |
| ENSOANG00000040480 | ENSOANG00000040480 | O_anatinus | Animals | mono_intergenic | H/ACA | | | intergenic |
| ENSOANG00000040481 | ENSOANG00000040481 | O_anatinus | Animals | mono_intergenic | H/ACA | | | intergenic |
| ENSOANG00000040482 | ENSOANG00000040482 | O_anatinus | Animals | mono_intronic | H/ACA | ENSOANG00000014979 | ARHGEF33 | protein_coding |
| ENSOANG00000040483 | ENSOANG00000040483 | O_anatinus | Animals | mono_intergenic | H/ACA | | | intergenic |
| ENSOANG00000040484 | ENSOANG00000040484 | O_anatinus | Animals | mono_intronic | H/ACA | ENSOANG00000013474 | RBBP5 | protein_coding |
| ENSOANG00000040486 | ENSOANG00000040486 | O_anatinus | Animals | mono_intergenic | H/ACA | | | intergenic |
| ENSOANG00000040489 | ENSOANG00000040489 | O_anatinus | Animals | mono_intergenic | H/ACA | | | intergenic |
| ENSOANG00000040493 | ENSOANG00000040493 | O_anatinus | Animals | mono_intergenic | H/ACA | | | intergenic |
| ENSOANG00000040494 | ENSOANG00000040494 | O_anatinus | Animals | mono_intergenic | H/ACA | | | intergenic |
| ENSOANG00000040495 | ENSOANG00000040495 | O_anatinus | Animals | mono_intergenic | H/ACA | | | intergenic |
| ENSOANG00000040496 | ENSOANG00000040496 | O_anatinus | Animals | mono_intergenic | H/ACA | | | intergenic |
| ENSOANG00000040498 | ENSOANG00000040498 | O_anatinus | Animals | mono_intergenic | H/ACA | | | intergenic |
| ENSOANG00000040500 | ENSOANG00000040500 | O_anatinus | Animals | mono_intergenic | H/ACA | | | intergenic |
| ENSOANG00000040507 | ENSOANG00000040507 | O_anatinus | Animals | mono_intergenic | H/ACA | | | intergenic |
| ENSOANG00000040511 | ENSOANG00000040511 | O_anatinus | Animals | mono_intergenic | H/ACA | | | intergenic |
| ENSOANG00000040512 | ENSOANG00000040512 | O_anatinus | Animals | mono_intergenic | H/ACA | | | intergenic |
| ENSOANG00000040513 | ENSOANG00000040513 | O_anatinus | Animals | mono_intergenic | H/ACA | | | intergenic |
| ENSOANG00000040520 | ENSOANG00000040520 | O_anatinus | Animals | mono_intronic | H/ACA | ENSOANG00000006026 | MYO3A | protein_coding |
| ENSOANG00000040525 | ENSOANG00000040525 | O_anatinus | Animals | intronic_cluster | H/ACA | ENSOANG00000015176 | | protein_coding |
| ENSOANG00000040526 | ENSOANG00000040526 | O_anatinus | Animals | mono_intergenic | H/ACA | | | intergenic |
| ENSOANG00000040528 | ENSOANG00000040528 | O_anatinus | Animals | intronic_cluster | H/ACA | ENSOANG00000001761 | HS2ST1 | protein_coding |
| ENSOANG00000040531 | ENSOANG00000040531 | O_anatinus | Animals | mono_intergenic | H/ACA | | | intergenic |
| ENSOANG00000040533 | ENSOANG00000040533 | O_anatinus | Animals | mono_intronic | H/ACA | ENSOANG00000049378 | GSTO1 | protein_coding |
| ENSOANG00000040536 | ENSOANG00000040536 | O_anatinus | Animals | mono_intronic | H/ACA | ENSOANG00000044540 | | protein_coding |
| ENSOANG00000040537 | ENSOANG00000040537 | O_anatinus | Animals | mono_intergenic | H/ACA | | | intergenic |
| ENSOANG00000040538 | ENSOANG00000040538 | O_anatinus | Animals | mono_intronic | H/ACA | ENSOANG00000003396 | TBC1D32 | protein_coding |
| ENSOANG00000040540 | ENSOANG00000040540 | O_anatinus | Animals | mono_intergenic | H/ACA | | | intergenic |
| ENSOANG00000040542 | ENSOANG00000040542 | O_anatinus | Animals | mono_intergenic | H/ACA | | | intergenic |
| ENSOANG00000040544 | ENSOANG00000040544 | O_anatinus | Animals | mono_intergenic | H/ACA | | | intergenic |
| ENSOANG00000040548 | ENSOANG00000040548 | O_anatinus | Animals | mono_intergenic | H/ACA | | | intergenic |
| ENSOANG00000040549 | ENSOANG00000040549 | O_anatinus | Animals | mono_intergenic | H/ACA | | | intergenic |
| ENSOANG00000040553 | ENSOANG00000040553 | O_anatinus | Animals | mono_intergenic | H/ACA | | | intergenic |
| ENSOANG00000040558 | ENSOANG00000040558 | O_anatinus | Animals | mono_intergenic | H/ACA | | | intergenic |
| ENSOANG00000040561 | ENSOANG00000040561 | O_anatinus | Animals | mono_intergenic | H/ACA | | | intergenic |
| ENSOANG00000040562 | ENSOANG00000040562 | O_anatinus | Animals | mono_intergenic | H/ACA | | | intergenic |
| ENSOANG00000040564 | ENSOANG00000040564 | O_anatinus | Animals | mono_intergenic | H/ACA | | | intergenic |
| ENSOANG00000040566 | ENSOANG00000040566 | O_anatinus | Animals | mono_intergenic | H/ACA | | | intergenic |
| ENSOANG00000040579 | ENSOANG00000040579 | O_anatinus | Animals | mono_intergenic | H/ACA | | | intergenic |
| ENSOANG00000040581 | ENSOANG00000040581 | O_anatinus | Animals | mono_intergenic | H/ACA | | | intergenic |

| | | | | | | | | |
|---|---|---|---|---|---|---|---|---|
| ENSOANG00000040582 | ENSOANG00000040582 | O_anatinus | Animals | mono_intergenic | H/ACA | | | intergenic |
| ENSOANG00000040591 | ENSOANG00000040591 | O_anatinus | Animals | mono_intronic | H/ACA | ENSOANG00000002436 | ITSN1 | protein_coding |
| ENSOANG00000040592 | ENSOANG00000040592 | O_anatinus | Animals | mono_intergenic | H/ACA | | | intergenic |
| ENSOANG00000040600 | ENSOANG00000040600 | O_anatinus | Animals | mono_intergenic | H/ACA | | | intergenic |
| ENSOANG00000040601 | ENSOANG00000040601 | O_anatinus | Animals | mono_intergenic | H/ACA | | | intergenic |
| ENSOANG00000040606 | ENSOANG00000040606 | O_anatinus | Animals | mono_intergenic | H/ACA | | | intergenic |
| ENSOANG00000040607 | ENSOANG00000040607 | O_anatinus | Animals | mono_intergenic | H/ACA | | | intergenic |
| ENSOANG00000040610 | ENSOANG00000040610 | O_anatinus | Animals | mono_intergenic | H/ACA | | | intergenic |
| ENSOANG00000040614 | ENSOANG00000040614 | O_anatinus | Animals | mono_intergenic | H/ACA | | | intergenic |
| ENSOANG00000040615 | ENSOANG00000040615 | O_anatinus | Animals | mono_intergenic | H/ACA | | | intergenic |
| ENSOANG00000040616 | ENSOANG00000040616 | O_anatinus | Animals | mono_intronic | H/ACA | ENSOANG00000031637 | | protein_coding |
| ENSOANG00000040620 | ENSOANG00000040620 | O_anatinus | Animals | mono_intergenic | H/ACA | | | intergenic |
| ENSOANG00000040622 | ENSOANG00000040622 | O_anatinus | Animals | mono_intergenic | H/ACA | | | intergenic |
| ENSOANG00000040625 | ENSOANG00000040625 | O_anatinus | Animals | mono_intronic | H/ACA | ENSOANG00000013303 | NR6A1 | protein_coding |
| ENSOANG00000040626 | ENSOANG00000040626 | O_anatinus | Animals | mono_intergenic | H/ACA | | | intergenic |
| ENSOANG00000040630 | ENSOANG00000040630 | O_anatinus | Animals | mono_intergenic | H/ACA | | | intergenic |
| ENSOANG00000040636 | ENSOANG00000040636 | O_anatinus | Animals | mono_intergenic | H/ACA | | | intergenic |
| ENSOANG00000040642 | ENSOANG00000040642 | O_anatinus | Animals | mono_intergenic | H/ACA | | | intergenic |
| ENSOANG00000040644 | ENSOANG00000040644 | O_anatinus | Animals | mono_intergenic | H/ACA | | | intergenic |
| ENSOANG00000040645 | ENSOANG00000040645 | O_anatinus | Animals | mono_intergenic | H/ACA | | | intergenic |
| ENSOANG00000040646 | ENSOANG00000040646 | O_anatinus | Animals | mono_intergenic | H/ACA | | | intergenic |
| ENSOANG00000040647 | ENSOANG00000040647 | O_anatinus | Animals | mono_intergenic | H/ACA | | | intergenic |
| ENSOANG00000040648 | ENSOANG00000040648 | O_anatinus | Animals | mono_intergenic | H/ACA | | | intergenic |
| ENSOANG00000040649 | ENSOANG00000040649 | O_anatinus | Animals | mono_intergenic | H/ACA | | | intergenic |
| ENSOANG00000040651 | ENSOANG00000040651 | O_anatinus | Animals | mono_intronic | H/ACA | ENSOANG00000007797 | | protein_coding |
| ENSOANG00000040653 | ENSOANG00000040653 | O_anatinus | Animals | mono_intronic | H/ACA | ENSOANG00000041406 | FGF13 | protein_coding |
| ENSOANG00000040657 | ENSOANG00000040657 | O_anatinus | Animals | mono_intronic | H/ACA | ENSOANG00000011370 | CNTN4 | protein_coding |
| ENSOANG00000040663 | ENSOANG00000040663 | O_anatinus | Animals | mono_intergenic | H/ACA | | | intergenic |
| ENSOANG00000040666 | ENSOANG00000040666 | O_anatinus | Animals | mono_intronic | H/ACA | ENSOANG00000013832 | FAM83B | protein_coding |
| ENSOANG00000040668 | ENSOANG00000040668 | O_anatinus | Animals | mono_intergenic | H/ACA | | | intergenic |
| ENSOANG00000040670 | ENSOANG00000040670 | O_anatinus | Animals | mono_intergenic | H/ACA | | | intergenic |
| ENSOANG00000040673 | ENSOANG00000040673 | O_anatinus | Animals | mono_intergenic | H/ACA | | | intergenic |
| ENSOANG00000040674 | ENSOANG00000040674 | O_anatinus | Animals | mono_intergenic | H/ACA | | | intergenic |
| ENSOANG00000040679 | ENSOANG00000040679 | O_anatinus | Animals | mono_intergenic | H/ACA | | | intergenic |
| ENSOANG00000040680 | ENSOANG00000040680 | O_anatinus | Animals | mono_intergenic | H/ACA | | | intergenic |
| ENSOANG00000040681 | ENSOANG00000040681 | O_anatinus | Animals | mono_intergenic | H/ACA | | | intergenic |
| ENSOANG00000040682 | ENSOANG00000040682 | O_anatinus | Animals | mono_intergenic | H/ACA | | | intergenic |
| ENSOANG00000040683 | ENSOANG00000040683 | O_anatinus | Animals | mono_intergenic | H/ACA | | | intergenic |
| ENSOANG00000040688 | ENSOANG00000040688 | O_anatinus | Animals | mono_intergenic | H/ACA | | | intergenic |
| ENSOANG00000040690 | ENSOANG00000040690 | O_anatinus | Animals | mono_intergenic | H/ACA | | | intergenic |
| ENSOANG00000040692 | ENSOANG00000040692 | O_anatinus | Animals | mono_intergenic | H/ACA | | | intergenic |

| | | | | | | | | |
|---|---|---|---|---|---|---|---|---|
| ENSOANG00000040693 | ENSOANG00000040693 | O_anatinus | Animals | mono_intergenic | H/ACA | | | intergenic |
| ENSOANG00000040695 | ENSOANG00000040695 | O_anatinus | Animals | mono_intergenic | H/ACA | | | intergenic |
| ENSOANG00000040696 | ENSOANG00000040696 | O_anatinus | Animals | mono_intergenic | H/ACA | | | intergenic |
| ENSOANG00000040697 | ENSOANG00000040697 | O_anatinus | Animals | mono_intergenic | H/ACA | | | intergenic |
| ENSOANG00000040699 | ENSOANG00000040699 | O_anatinus | Animals | mono_intergenic | H/ACA | | | intergenic |
| ENSOANG00000040701 | ENSOANG00000040701 | O_anatinus | Animals | mono_intergenic | H/ACA | | | intergenic |
| ENSOANG00000040708 | ENSOANG00000040708 | O_anatinus | Animals | mono_intergenic | H/ACA | | | intergenic |
| ENSOANG00000040709 | ENSOANG00000040709 | O_anatinus | Animals | mono_intergenic | H/ACA | | | intergenic |
| ENSOANG00000040712 | ENSOANG00000040712 | O_anatinus | Animals | mono_intergenic | H/ACA | | | intergenic |
| ENSOANG00000040713 | ENSOANG00000040713 | O_anatinus | Animals | mono_intergenic | H/ACA | | | intergenic |
| ENSOANG00000040714 | ENSOANG00000040714 | O_anatinus | Animals | mono_intronic | Unknown | ENSOANG00000041837 | RPS8 | protein_coding |
| ENSOANG00000040719 | ENSOANG00000040719 | O_anatinus | Animals | mono_intronic | H/ACA | ENSOANG00000005022 | TRPM3 | protein_coding |
| ENSOANG00000040720 | ENSOANG00000040720 | O_anatinus | Animals | mono_intergenic | H/ACA | | | intergenic |
| ENSOANG00000040722 | ENSOANG00000040722 | O_anatinus | Animals | mono_intergenic | H/ACA | | | intergenic |
| ENSOANG00000040727 | ENSOANG00000040727 | O_anatinus | Animals | mono_intergenic | H/ACA | | | intergenic |
| ENSOANG00000040730 | ENSOANG00000040730 | O_anatinus | Animals | mono_intronic | H/ACA | ENSOANG00000021714 | CABCOCO1 | protein_coding |
| ENSOANG00000040731 | ENSOANG00000040731 | O_anatinus | Animals | mono_intergenic | H/ACA | | | intergenic |
| ENSOANG00000040734 | ENSOANG00000040734 | O_anatinus | Animals | mono_intergenic | H/ACA | | | intergenic |
| ENSOANG00000040736 | ENSOANG00000040736 | O_anatinus | Animals | mono_intergenic | H/ACA | | | intergenic |
| ENSOANG00000040739 | ENSOANG00000040739 | O_anatinus | Animals | mono_intergenic | H/ACA | | | intergenic |
| ENSOANG00000040744 | ENSOANG00000040744 | O_anatinus | Animals | mono_intergenic | H/ACA | | | intergenic |
| ENSOANG00000040746 | ENSOANG00000040746 | O_anatinus | Animals | mono_intergenic | H/ACA | | | intergenic |
| ENSOANG00000040749 | ENSOANG00000040749 | O_anatinus | Animals | intronic_cluster | H/ACA | ENSOANG00000014431 | ADAMTS12 | protein_coding |
| ENSOANG00000040750 | ENSOANG00000040750 | O_anatinus | Animals | mono_intergenic | H/ACA | | | intergenic |
| ENSOANG00000040754 | ENSOANG00000040754 | O_anatinus | Animals | mono_intergenic | H/ACA | | | intergenic |
| ENSOANG00000040757 | ENSOANG00000040757 | O_anatinus | Animals | mono_intronic | H/ACA | ENSOANG00000044895 | NELL2 | protein_coding |
| ENSOANG00000040760 | ENSOANG00000040760 | O_anatinus | Animals | mono_intergenic | H/ACA | | | intergenic |
| ENSOANG00000040761 | ENSOANG00000040761 | O_anatinus | Animals | mono_intergenic | H/ACA | | | intergenic |
| ENSOANG00000040763 | ENSOANG00000040763 | O_anatinus | Animals | mono_intergenic | H/ACA | | | intergenic |
| ENSOANG00000040765 | ENSOANG00000040765 | O_anatinus | Animals | mono_intergenic | H/ACA | | | intergenic |
| ENSOANG00000040768 | ENSOANG00000040768 | O_anatinus | Animals | mono_intronic | H/ACA | ENSOANG00000039346 | SLC2A13 | protein_coding |
| ENSOANG00000040774 | ENSOANG00000040774 | O_anatinus | Animals | mono_intronic | H/ACA | ENSOANG00000010395 | | protein_coding |
| ENSOANG00000040780 | ENSOANG00000040780 | O_anatinus | Animals | mono_intronic | H/ACA | ENSOANG00000012817 | | protein_coding |
| ENSOANG00000040781 | ENSOANG00000040781 | O_anatinus | Animals | mono_intergenic | H/ACA | | | intergenic |
| ENSOANG00000040784 | ENSOANG00000040784 | O_anatinus | Animals | mono_intergenic | H/ACA | | | intergenic |
| ENSOANG00000040786 | ENSOANG00000040786 | O_anatinus | Animals | mono_intergenic | H/ACA | | | intergenic |
| ENSOANG00000040787 | ENSOANG00000040787 | O_anatinus | Animals | mono_intronic | H/ACA | ENSOANG00000048487 | DBX2 | protein_coding |
| ENSOANG00000040793 | ENSOANG00000040793 | O_anatinus | Animals | mono_intergenic | H/ACA | | | intergenic |
| ENSOANG00000040794 | ENSOANG00000040794 | O_anatinus | Animals | mono_intergenic | H/ACA | | | intergenic |
| ENSOANG00000040797 | ENSOANG00000040797 | O_anatinus | Animals | mono_intergenic | H/ACA | | | intergenic |
| ENSOANG00000040798 | ENSOANG00000040798 | O_anatinus | Animals | mono_intergenic | H/ACA | | | intergenic |

| | | | | | | | | |
|---|---|---|---|---|---|---|---|---|
| ENSOANG00000040801 | ENSOANG00000040801 | O_anatinus | Animals | mono_intergenic | H/ACA | | | intergenic |
| ENSOANG00000040802 | ENSOANG00000040802 | O_anatinus | Animals | mono_intergenic | H/ACA | | | intergenic |
| ENSOANG00000040803 | ENSOANG00000040803 | O_anatinus | Animals | mono_intergenic | H/ACA | | | intergenic |
| ENSOANG00000040804 | ENSOANG00000040804 | O_anatinus | Animals | mono_intergenic | H/ACA | | | intergenic |
| ENSOANG00000040805 | ENSOANG00000040805 | O_anatinus | Animals | intronic_cluster | H/ACA | ENSOANG00000004857 | ZNF281 | protein_coding |
| ENSOANG00000040809 | ENSOANG00000040809 | O_anatinus | Animals | mono_intronic | H/ACA | ENSOANG00000022504 | PXYLP1 | protein_coding |
| ENSOANG00000040812 | ENSOANG00000040812 | O_anatinus | Animals | mono_intergenic | H/ACA | | | intergenic |
| ENSOANG00000040815 | ENSOANG00000040815 | O_anatinus | Animals | mono_intergenic | H/ACA | | | intergenic |
| ENSOANG00000040821 | ENSOANG00000040821 | O_anatinus | Animals | mono_intergenic | H/ACA | | | intergenic |
| ENSOANG00000040824 | ENSOANG00000040824 | O_anatinus | Animals | mono_intergenic | H/ACA | | | intergenic |
| ENSOANG00000040826 | ENSOANG00000040826 | O_anatinus | Animals | mono_intergenic | H/ACA | | | intergenic |
| ENSOANG00000040828 | ENSOANG00000040828 | O_anatinus | Animals | mono_intergenic | H/ACA | | | intergenic |
| ENSOANG00000040835 | ENSOANG00000040835 | O_anatinus | Animals | mono_intronic | H/ACA | ENSOANG00000037899 | RAB31 | protein_coding |
| ENSOANG00000040836 | ENSOANG00000040836 | O_anatinus | Animals | mono_intergenic | H/ACA | | | intergenic |
| ENSOANG00000040842 | ENSOANG00000040842 | O_anatinus | Animals | mono_intergenic | H/ACA | | | intergenic |
| ENSOANG00000040845 | ENSOANG00000040845 | O_anatinus | Animals | mono_intergenic | H/ACA | | | intergenic |
| ENSOANG00000040846 | ENSOANG00000040846 | O_anatinus | Animals | mono_intergenic | H/ACA | | | intergenic |
| ENSOANG00000040849 | ENSOANG00000040849 | O_anatinus | Animals | mono_intergenic | H/ACA | | | intergenic |
| ENSOANG00000040850 | ENSOANG00000040850 | O_anatinus | Animals | mono_intronic | H/ACA | ENSOANG00000011309 | ITGBL1 | protein_coding |
| ENSOANG00000040854 | ENSOANG00000040854 | O_anatinus | Animals | mono_intergenic | H/ACA | | | intergenic |
| ENSOANG00000040859 | ENSOANG00000040859 | O_anatinus | Animals | mono_intergenic | H/ACA | | | intergenic |
| ENSOANG00000040860 | ENSOANG00000040860 | O_anatinus | Animals | mono_intronic | H/ACA | ENSOANG00000041611 | CDK19 | protein_coding |
| ENSOANG00000040862 | ENSOANG00000040862 | O_anatinus | Animals | mono_intergenic | H/ACA | | | intergenic |
| ENSOANG00000040864 | ENSOANG00000040864 | O_anatinus | Animals | mono_intergenic | H/ACA | | | intergenic |
| ENSOANG00000040866 | ENSOANG00000040866 | O_anatinus | Animals | mono_intergenic | H/ACA | | | intergenic |
| ENSOANG00000040869 | ENSOANG00000040869 | O_anatinus | Animals | mono_intergenic | H/ACA | | | intergenic |
| ENSOANG00000040871 | ENSOANG00000040871 | O_anatinus | Animals | mono_intronic | H/ACA | ENSOANG00000041478 | VPS26B | protein_coding |
| ENSOANG00000040873 | ENSOANG00000040873 | O_anatinus | Animals | mono_intergenic | H/ACA | | | intergenic |
| ENSOANG00000040874 | ENSOANG00000040874 | O_anatinus | Animals | mono_intergenic | H/ACA | | | intergenic |
| ENSOANG00000040883 | ENSOANG00000040883 | O_anatinus | Animals | mono_intergenic | H/ACA | | | intergenic |
| ENSOANG00000040885 | ENSOANG00000040885 | O_anatinus | Animals | mono_intergenic | H/ACA | | | intergenic |
| ENSOANG00000040891 | ENSOANG00000040891 | O_anatinus | Animals | mono_intronic | H/ACA | ENSOANG00000043881 | TERT | protein_coding |
| ENSOANG00000040892 | ENSOANG00000040892 | O_anatinus | Animals | mono_intergenic | H/ACA | | | intergenic |
| ENSOANG00000040895 | ENSOANG00000040895 | O_anatinus | Animals | mono_intergenic | H/ACA | | | intergenic |
| ENSOANG00000040897 | ENSOANG00000040897 | O_anatinus | Animals | mono_intergenic | H/ACA | | | intergenic |
| ENSOANG00000040898 | ENSOANG00000040898 | O_anatinus | Animals | mono_intergenic | H/ACA | | | intergenic |
| ENSOANG00000040900 | ENSOANG00000040900 | O_anatinus | Animals | mono_intergenic | H/ACA | | | intergenic |
| ENSOANG00000040901 | ENSOANG00000040901 | O_anatinus | Animals | mono_intergenic | H/ACA | | | intergenic |
| ENSOANG00000040904 | ENSOANG00000040904 | O_anatinus | Animals | intronic_cluster | H/ACA | ENSOANG00000011147 | ST6GAL2 | protein_coding |
| ENSOANG00000040905 | ENSOANG00000040905 | O_anatinus | Animals | mono_intergenic | H/ACA | | | intergenic |
| ENSOANG00000040908 | ENSOANG00000040908 | O_anatinus | Animals | mono_intergenic | H/ACA | | | intergenic |

| | | | | | | | |
|---|---|---|---|---|---|---|---|
| ENSOANG00000040910 | ENSOANG00000040910 | O_anatinus | Animals | mono_intergenic | H/ACA | | intergenic |
| ENSOANG00000040912 | ENSOANG00000040912 | O_anatinus | Animals | mono_intergenic | H/ACA | | intergenic |
| ENSOANG00000040913 | ENSOANG00000040913 | O_anatinus | Animals | mono_intergenic | H/ACA | | intergenic |
| ENSOANG00000040914 | ENSOANG00000040914 | O_anatinus | Animals | mono_intergenic | H/ACA | | intergenic |
| ENSOANG00000040919 | ENSOANG00000040919 | O_anatinus | Animals | mono_intergenic | H/ACA | | intergenic |
| ENSOANG00000040920 | ENSOANG00000040920 | O_anatinus | Animals | mono_intergenic | H/ACA | | intergenic |
| ENSOANG00000040923 | ENSOANG00000040923 | O_anatinus | Animals | mono_intergenic | H/ACA | | intergenic |
| ENSOANG00000040924 | ENSOANG00000040924 | O_anatinus | Animals | mono_intergenic | H/ACA | | intergenic |
| ENSOANG00000040927 | ENSOANG00000040927 | O_anatinus | Animals | mono_intergenic | H/ACA | | intergenic |
| ENSOANG00000040928 | ENSOANG00000040928 | O_anatinus | Animals | mono_intergenic | H/ACA | | intergenic |
| ENSOANG00000040929 | ENSOANG00000040929 | O_anatinus | Animals | mono_intergenic | H/ACA | | intergenic |
| ENSOANG00000040930 | ENSOANG00000040930 | O_anatinus | Animals | mono_intergenic | H/ACA | | intergenic |
| ENSOANG00000040931 | ENSOANG00000040931 | O_anatinus | Animals | mono_intergenic | H/ACA | | intergenic |
| ENSOANG00000040932 | ENSOANG00000040932 | O_anatinus | Animals | mono_intergenic | H/ACA | | intergenic |
| ENSOANG00000040933 | ENSOANG00000040933 | O_anatinus | Animals | mono_intergenic | H/ACA | | intergenic |
| ENSOANG00000040934 | ENSOANG00000040934 | O_anatinus | Animals | mono_intergenic | H/ACA | | intergenic |
| ENSOANG00000040935 | ENSOANG00000040935 | O_anatinus | Animals | mono_intergenic | H/ACA | | intergenic |
| ENSOANG00000040936 | ENSOANG00000040936 | O_anatinus | Animals | mono_intergenic | H/ACA | | intergenic |
| ENSOANG00000040938 | ENSOANG00000040938 | O_anatinus | Animals | mono_intergenic | H/ACA | | intergenic |
| ENSOANG00000040943 | ENSOANG00000040943 | O_anatinus | Animals | mono_intergenic | H/ACA | | intergenic |
| ENSOANG00000040945 | ENSOANG00000040945 | O_anatinus | Animals | mono_intronic | H/ACA | ENSOANG00000040433 | protein_coding |
| ENSOANG00000040948 | ENSOANG00000040948 | O_anatinus | Animals | mono_intergenic | H/ACA | | intergenic |
| ENSOANG00000040950 | ENSOANG00000040950 | O_anatinus | Animals | mono_intergenic | H/ACA | | intergenic |
| ENSOANG00000040954 | ENSOANG00000040954 | O_anatinus | Animals | mono_intergenic | H/ACA | | intergenic |
| ENSOANG00000040956 | ENSOANG00000040956 | O_anatinus | Animals | mono_intergenic | H/ACA | | intergenic |
| ENSOANG00000040962 | ENSOANG00000040962 | O_anatinus | Animals | mono_intergenic | H/ACA | | intergenic |
| ENSOANG00000040963 | ENSOANG00000040963 | O_anatinus | Animals | mono_intergenic | H/ACA | | intergenic |
| ENSOANG00000040964 | ENSOANG00000040964 | O_anatinus | Animals | mono_intergenic | H/ACA | | intergenic |
| ENSOANG00000040966 | ENSOANG00000040966 | O_anatinus | Animals | mono_intergenic | H/ACA | | intergenic |
| ENSOANG00000040969 | ENSOANG00000040969 | O_anatinus | Animals | mono_intergenic | H/ACA | | intergenic |
| ENSOANG00000040973 | ENSOANG00000040973 | O_anatinus | Animals | mono_intergenic | H/ACA | | intergenic |
| ENSOANG00000040977 | ENSOANG00000040977 | O_anatinus | Animals | mono_intergenic | H/ACA | | intergenic |
| ENSOANG00000040978 | ENSOANG00000040978 | O_anatinus | Animals | mono_intergenic | H/ACA | | intergenic |
| ENSOANG00000040984 | ENSOANG00000040984 | O_anatinus | Animals | mono_intergenic | H/ACA | | intergenic |
| ENSOANG00000040985 | ENSOANG00000040985 | O_anatinus | Animals | mono_intergenic | H/ACA | | intergenic |
| ENSOANG00000040989 | ENSOANG00000040989 | O_anatinus | Animals | mono_intergenic | H/ACA | | intergenic |
| ENSOANG00000040990 | ENSOANG00000040990 | O_anatinus | Animals | mono_intergenic | H/ACA | | intergenic |
| ENSOANG00000040991 | ENSOANG00000040991 | O_anatinus | Animals | mono_intergenic | H/ACA | | intergenic |
| ENSOANG00000040993 | ENSOANG00000040993 | O_anatinus | Animals | mono_intergenic | H/ACA | | intergenic |
| ENSOANG00000040994 | ENSOANG00000040994 | O_anatinus | Animals | mono_intergenic | H/ACA | | intergenic |
| ENSOANG00000040997 | ENSOANG00000040997 | O_anatinus | Animals | mono_intergenic | H/ACA | | intergenic |

| | | | | | | | | |
|---|---|---|---|---|---|---|---|---|
| ENSOANG00000040998 | ENSOANG00000040998 | O_anatinus | Animals | mono_intergenic | H/ACA | | | intergenic |
| ENSOANG00000041000 | ENSOANG00000041000 | O_anatinus | Animals | mono_intergenic | H/ACA | | | intergenic |
| ENSOANG00000041001 | ENSOANG00000041001 | O_anatinus | Animals | mono_intergenic | H/ACA | | | intergenic |
| ENSOANG00000041002 | ENSOANG00000041002 | O_anatinus | Animals | mono_intronic | H/ACA | ENSOANG00000010444 | CCT6B | protein_coding |
| ENSOANG00000041003 | ENSOANG00000041003 | O_anatinus | Animals | mono_intergenic | H/ACA | | | intergenic |
| ENSOANG00000041005 | ENSOANG00000041005 | O_anatinus | Animals | mono_intergenic | H/ACA | | | intergenic |
| ENSOANG00000041006 | ENSOANG00000041006 | O_anatinus | Animals | mono_intergenic | H/ACA | | | intergenic |
| ENSOANG00000041007 | ENSOANG00000041007 | O_anatinus | Animals | mono_intronic | H/ACA | ENSOANG00000046560 | VEPH1 | protein_coding |
| ENSOANG00000041009 | ENSOANG00000041009 | O_anatinus | Animals | mono_intergenic | H/ACA | | | intergenic |
| ENSOANG00000041015 | ENSOANG00000041015 | O_anatinus | Animals | mono_intergenic | H/ACA | | | intergenic |
| ENSOANG00000041016 | ENSOANG00000041016 | O_anatinus | Animals | mono_intergenic | H/ACA | | | intergenic |
| ENSOANG00000041017 | ENSOANG00000041017 | O_anatinus | Animals | mono_intergenic | H/ACA | | | intergenic |
| ENSOANG00000041018 | ENSOANG00000041018 | O_anatinus | Animals | mono_intergenic | H/ACA | | | intergenic |
| ENSOANG00000041020 | ENSOANG00000041020 | O_anatinus | Animals | mono_intergenic | H/ACA | | | intergenic |
| ENSOANG00000041021 | ENSOANG00000041021 | O_anatinus | Animals | mono_intronic | H/ACA | ENSOANG00000013699 | SLC4A4 | protein_coding |
| ENSOANG00000041022 | ENSOANG00000041022 | O_anatinus | Animals | mono_intergenic | H/ACA | | | intergenic |
| ENSOANG00000041024 | ENSOANG00000041024 | O_anatinus | Animals | mono_intergenic | H/ACA | | | intergenic |
| ENSOANG00000041025 | ENSOANG00000041025 | O_anatinus | Animals | mono_intergenic | H/ACA | | | intergenic |
| ENSOANG00000041026 | ENSOANG00000041026 | O_anatinus | Animals | mono_intergenic | H/ACA | | | intergenic |
| ENSOANG00000041027 | ENSOANG00000041027 | O_anatinus | Animals | intronic_cluster | H/ACA | ENSOANG00000015176 | | protein_coding |
| ENSOANG00000041029 | ENSOANG00000041029 | O_anatinus | Animals | mono_intergenic | H/ACA | | | intergenic |
| ENSOANG00000041031 | ENSOANG00000041031 | O_anatinus | Animals | intronic_cluster | H/ACA | ENSOANG00000040957 | | non_coding |
| ENSOANG00000041033 | ENSOANG00000041033 | O_anatinus | Animals | mono_intergenic | H/ACA | | | intergenic |
| ENSOANG00000041036 | ENSOANG00000041036 | O_anatinus | Animals | mono_intergenic | H/ACA | | | intergenic |
| ENSOANG00000041037 | ENSOANG00000041037 | O_anatinus | Animals | mono_intergenic | H/ACA | | | intergenic |
| ENSOANG00000041039 | ENSOANG00000041039 | O_anatinus | Animals | mono_intergenic | H/ACA | | | intergenic |
| ENSOANG00000041041 | ENSOANG00000041041 | O_anatinus | Animals | mono_intergenic | H/ACA | | | intergenic |
| ENSOANG00000041043 | ENSOANG00000041043 | O_anatinus | Animals | mono_intronic | H/ACA | ENSOANG00000049315 | PLPPR1 | protein_coding |
| ENSOANG00000041046 | ENSOANG00000041046 | O_anatinus | Animals | mono_intronic | H/ACA | ENSOANG00000005267 | ARHGAP10 | protein_coding |
| ENSOANG00000041049 | ENSOANG00000041049 | O_anatinus | Animals | mono_intergenic | H/ACA | | | intergenic |
| ENSOANG00000041051 | ENSOANG00000041051 | O_anatinus | Animals | mono_intergenic | H/ACA | | | intergenic |
| ENSOANG00000041054 | ENSOANG00000041054 | O_anatinus | Animals | mono_intergenic | H/ACA | | | intergenic |
| ENSOANG00000041063 | ENSOANG00000041063 | O_anatinus | Animals | mono_intergenic | H/ACA | | | intergenic |
| ENSOANG00000041064 | ENSOANG00000041064 | O_anatinus | Animals | mono_intergenic | H/ACA | | | intergenic |
| ENSOANG00000041066 | ENSOANG00000041066 | O_anatinus | Animals | mono_intergenic | H/ACA | | | intergenic |
| ENSOANG00000041068 | ENSOANG00000041068 | O_anatinus | Animals | mono_intergenic | H/ACA | | | intergenic |
| ENSOANG00000041071 | ENSOANG00000041071 | O_anatinus | Animals | mono_intergenic | H/ACA | | | intergenic |
| ENSOANG00000041075 | ENSOANG00000041075 | O_anatinus | Animals | mono_intergenic | H/ACA | | | intergenic |
| ENSOANG00000041078 | ENSOANG00000041078 | O_anatinus | Animals | mono_intergenic | H/ACA | | | intergenic |
| ENSOANG00000041080 | ENSOANG00000041080 | O_anatinus | Animals | mono_intergenic | H/ACA | | | intergenic |
| ENSOANG00000041086 | ENSOANG00000041086 | O_anatinus | Animals | mono_intergenic | H/ACA | | | intergenic |

| | | | | | | | | |
|---|---|---|---|---|---|---|---|---|
| ENSOANG00000041087 | ENSOANG00000041087 | O_anatinus | Animals | mono_intergenic | H/ACA | | | intergenic |
| ENSOANG00000041088 | ENSOANG00000041088 | O_anatinus | Animals | mono_intergenic | H/ACA | | | intergenic |
| ENSOANG00000041089 | ENSOANG00000041089 | O_anatinus | Animals | mono_intergenic | H/ACA | | | intergenic |
| ENSOANG00000041090 | ENSOANG00000041090 | O_anatinus | Animals | mono_intergenic | H/ACA | | | intergenic |
| ENSOANG00000041092 | ENSOANG00000041092 | O_anatinus | Animals | mono_intergenic | H/ACA | | | intergenic |
| ENSOANG00000041093 | ENSOANG00000041093 | O_anatinus | Animals | mono_intergenic | H/ACA | | | intergenic |
| ENSOANG00000041096 | ENSOANG00000041096 | O_anatinus | Animals | mono_intergenic | H/ACA | | | intergenic |
| ENSOANG00000041098 | ENSOANG00000041098 | O_anatinus | Animals | mono_intergenic | H/ACA | | | intergenic |
| ENSOANG00000041101 | ENSOANG00000041101 | O_anatinus | Animals | mono_intergenic | H/ACA | | | intergenic |
| ENSOANG00000041102 | ENSOANG00000041102 | O_anatinus | Animals | mono_intergenic | H/ACA | | | intergenic |
| ENSOANG00000041112 | ENSOANG00000041112 | O_anatinus | Animals | mono_intronic | H/ACA | ENSOANG00000043567 | | protein_coding |
| ENSOANG00000041115 | ENSOANG00000041115 | O_anatinus | Animals | mono_intergenic | H/ACA | | | intergenic |
| ENSOANG00000041117 | ENSOANG00000041117 | O_anatinus | Animals | mono_intergenic | H/ACA | | | intergenic |
| ENSOANG00000041120 | ENSOANG00000041120 | O_anatinus | Animals | mono_intronic | H/ACA | ENSOANG00000002683 | RBP1 | protein_coding |
| ENSOANG00000041122 | ENSOANG00000041122 | O_anatinus | Animals | mono_intergenic | H/ACA | | | intergenic |
| ENSOANG00000041124 | ENSOANG00000041124 | O_anatinus | Animals | mono_intronic | H/ACA | ENSOANG00000014126 | DLGAP2 | protein_coding |
| ENSOANG00000041125 | ENSOANG00000041125 | O_anatinus | Animals | mono_intergenic | H/ACA | | | intergenic |
| ENSOANG00000041126 | ENSOANG00000041126 | O_anatinus | Animals | mono_intergenic | H/ACA | | | intergenic |
| ENSOANG00000041127 | ENSOANG00000041127 | O_anatinus | Animals | mono_intronic | H/ACA | ENSOANG00000036705 | DLGAP1 | protein_coding |
| ENSOANG00000041128 | ENSOANG00000041128 | O_anatinus | Animals | mono_intergenic | H/ACA | | | intergenic |
| ENSOANG00000041130 | ENSOANG00000041130 | O_anatinus | Animals | mono_intergenic | H/ACA | | | intergenic |
| ENSOANG00000041133 | ENSOANG00000041133 | O_anatinus | Animals | mono_intergenic | H/ACA | | | intergenic |
| ENSOANG00000041134 | SNORD34 | O_anatinus | Animals | intronic_cluster | C/D | ENSOANG00000000828 | RPL13A | protein_coding |
| ENSOANG00000041136 | ENSOANG00000041136 | O_anatinus | Animals | mono_intronic | H/ACA | ENSOANG00000041794 | DYNC1I1 | protein_coding |
| ENSOANG00000041137 | ENSOANG00000041137 | O_anatinus | Animals | mono_intergenic | H/ACA | | | intergenic |
| ENSOANG00000041138 | ENSOANG00000041138 | O_anatinus | Animals | mono_intergenic | H/ACA | | | intergenic |
| ENSOANG00000041140 | ENSOANG00000041140 | O_anatinus | Animals | mono_intergenic | H/ACA | | | intergenic |
| ENSOANG00000041147 | ENSOANG00000041147 | O_anatinus | Animals | mono_intergenic | H/ACA | | | intergenic |
| ENSOANG00000041155 | ENSOANG00000041155 | O_anatinus | Animals | mono_intergenic | H/ACA | | | intergenic |
| ENSOANG00000041156 | ENSOANG00000041156 | O_anatinus | Animals | mono_intronic | H/ACA | ENSOANG00000001131 | LIPG | protein_coding |
| ENSOANG00000041159 | ENSOANG00000041159 | O_anatinus | Animals | mono_intergenic | H/ACA | | | intergenic |
| ENSOANG00000041160 | ENSOANG00000041160 | O_anatinus | Animals | mono_intergenic | H/ACA | | | intergenic |
| ENSOANG00000041162 | ENSOANG00000041162 | O_anatinus | Animals | mono_intergenic | H/ACA | | | intergenic |
| ENSOANG00000041163 | ENSOANG00000041163 | O_anatinus | Animals | mono_intergenic | H/ACA | | | intergenic |
| ENSOANG00000041164 | ENSOANG00000041164 | O_anatinus | Animals | mono_intergenic | H/ACA | | | intergenic |
| ENSOANG00000041166 | ENSOANG00000041166 | O_anatinus | Animals | mono_intronic | H/ACA | ENSOANG00000036564 | UNG | protein_coding |
| ENSOANG00000041170 | ENSOANG00000041170 | O_anatinus | Animals | mono_intergenic | H/ACA | | | intergenic |
| ENSOANG00000041171 | ENSOANG00000041171 | O_anatinus | Animals | mono_intronic | H/ACA | ENSOANG00000009333 | CSNK1G2 | protein_coding |
| ENSOANG00000041172 | ENSOANG00000041172 | O_anatinus | Animals | mono_intergenic | H/ACA | | | intergenic |
| ENSOANG00000041173 | ENSOANG00000041173 | O_anatinus | Animals | mono_intronic | H/ACA | ENSOANG00000004496 | CDH8 | protein_coding |
| ENSOANG00000041174 | ENSOANG00000041174 | O_anatinus | Animals | mono_intergenic | H/ACA | | | intergenic |

| | | | | | | | | |
|---|---|---|---|---|---|---|---|---|
| ENSOANG00000041179 | ENSOANG00000041179 | O_anatinus | Animals | mono_intronic | H/ACA | ENSOANG00000022715 | | protein_coding |
| ENSOANG00000041184 | ENSOANG00000041184 | O_anatinus | Animals | mono_intergenic | H/ACA | | | intergenic |
| ENSOANG00000041185 | ENSOANG00000041185 | O_anatinus | Animals | mono_intronic | H/ACA | ENSOANG00000001087 | FNDC3B | protein_coding |
| ENSOANG00000041190 | ENSOANG00000041190 | O_anatinus | Animals | mono_intronic | H/ACA | ENSOANG00000001034 | NTNG1 | protein_coding |
| ENSOANG00000041194 | ENSOANG00000041194 | O_anatinus | Animals | mono_intergenic | H/ACA | | | intergenic |
| ENSOANG00000041199 | ENSOANG00000041199 | O_anatinus | Animals | mono_intergenic | H/ACA | | | intergenic |
| ENSOANG00000041200 | ENSOANG00000041200 | O_anatinus | Animals | mono_intronic | H/ACA | ENSOANG00000002277 | OSBPL10 | protein_coding |
| ENSOANG00000041201 | ENSOANG00000041201 | O_anatinus | Animals | mono_intergenic | H/ACA | | | intergenic |
| ENSOANG00000041202 | ENSOANG00000041202 | O_anatinus | Animals | mono_intergenic | H/ACA | | | intergenic |
| ENSOANG00000041211 | SNORD58 | O_anatinus | Animals | mono_intronic | C/D | ENSOANG00000002910 | | protein_coding |
| ENSOANG00000041212 | ENSOANG00000041212 | O_anatinus | Animals | mono_intergenic | H/ACA | | | intergenic |
| ENSOANG00000041213 | ENSOANG00000041213 | O_anatinus | Animals | mono_intergenic | H/ACA | | | intergenic |
| ENSOANG00000041214 | ENSOANG00000041214 | O_anatinus | Animals | mono_intergenic | H/ACA | | | intergenic |
| ENSOANG00000041215 | ENSOANG00000041215 | O_anatinus | Animals | mono_intronic | H/ACA | ENSOANG00000006601 | CFAP54 | protein_coding |
| ENSOANG00000041223 | ENSOANG00000041223 | O_anatinus | Animals | mono_intergenic | H/ACA | | | intergenic |
| ENSOANG00000041224 | ENSOANG00000041224 | O_anatinus | Animals | mono_intergenic | H/ACA | | | intergenic |
| ENSOANG00000041226 | ENSOANG00000041226 | O_anatinus | Animals | mono_intergenic | H/ACA | | | intergenic |
| ENSOANG00000041228 | ENSOANG00000041228 | O_anatinus | Animals | mono_intergenic | H/ACA | | | intergenic |
| ENSOANG00000041231 | ENSOANG00000041231 | O_anatinus | Animals | mono_intergenic | H/ACA | | | intergenic |
| ENSOANG00000041237 | ENSOANG00000041237 | O_anatinus | Animals | mono_intergenic | H/ACA | | | intergenic |
| ENSOANG00000041241 | ENSOANG00000041241 | O_anatinus | Animals | mono_intergenic | H/ACA | | | intergenic |
| ENSOANG00000041246 | ENSOANG00000041246 | O_anatinus | Animals | mono_intergenic | H/ACA | | | intergenic |
| ENSOANG00000041249 | ENSOANG00000041249 | O_anatinus | Animals | mono_intergenic | H/ACA | | | intergenic |
| ENSOANG00000041250 | ENSOANG00000041250 | O_anatinus | Animals | mono_intergenic | H/ACA | | | intergenic |
| ENSOANG00000041256 | ENSOANG00000041256 | O_anatinus | Animals | mono_intergenic | H/ACA | | | intergenic |
| ENSOANG00000041258 | ENSOANG00000041258 | O_anatinus | Animals | mono_intergenic | H/ACA | | | intergenic |
| ENSOANG00000041260 | ENSOANG00000041260 | O_anatinus | Animals | mono_intergenic | H/ACA | | | intergenic |
| ENSOANG00000041262 | ENSOANG00000041262 | O_anatinus | Animals | mono_intergenic | H/ACA | | | intergenic |
| ENSOANG00000041265 | ENSOANG00000041265 | O_anatinus | Animals | mono_intergenic | H/ACA | | | intergenic |
| ENSOANG00000041270 | ENSOANG00000041270 | O_anatinus | Animals | mono_intergenic | H/ACA | | | intergenic |
| ENSOANG00000041272 | ENSOANG00000041272 | O_anatinus | Animals | mono_intergenic | H/ACA | | | intergenic |
| ENSOANG00000041273 | ENSOANG00000041273 | O_anatinus | Animals | mono_intergenic | H/ACA | | | intergenic |
| ENSOANG00000041275 | ENSOANG00000041275 | O_anatinus | Animals | mono_intronic | H/ACA | ENSOANG00000005437 | PTGR1 | protein_coding |
| ENSOANG00000041276 | ENSOANG00000041276 | O_anatinus | Animals | mono_intronic | H/ACA | ENSOANG00000012242 | SLC9A4 | protein_coding |
| ENSOANG00000041280 | ENSOANG00000041280 | O_anatinus | Animals | mono_intergenic | H/ACA | | | intergenic |
| ENSOANG00000041287 | ENSOANG00000041287 | O_anatinus | Animals | mono_intergenic | H/ACA | | | intergenic |
| ENSOANG00000041290 | ENSOANG00000041290 | O_anatinus | Animals | mono_intergenic | H/ACA | | | intergenic |
| ENSOANG00000041293 | ENSOANG00000041293 | O_anatinus | Animals | mono_intergenic | H/ACA | | | intergenic |
| ENSOANG00000041294 | ENSOANG00000041294 | O_anatinus | Animals | mono_intergenic | H/ACA | | | intergenic |
| ENSOANG00000041295 | ENSOANG00000041295 | O_anatinus | Animals | mono_intergenic | H/ACA | | | intergenic |
| ENSOANG00000041298 | ENSOANG00000041298 | O_anatinus | Animals | mono_intergenic | H/ACA | | | intergenic |

| | | | | | | | | |
|---|---|---|---|---|---|---|---|---|
| ENSOANG00000041304 | ENSOANG00000041304 | O_anatinus | Animals | mono_intergenic | H/ACA | | | intergenic |
| ENSOANG00000041305 | ENSOANG00000041305 | O_anatinus | Animals | intronic_cluster | H/ACA | ENSOANG00000007531 | SYT1 | protein_coding |
| ENSOANG00000041308 | ENSOANG00000041308 | O_anatinus | Animals | mono_intergenic | H/ACA | | | intergenic |
| ENSOANG00000041312 | ENSOANG00000041312 | O_anatinus | Animals | mono_intergenic | H/ACA | | | intergenic |
| ENSOANG00000041317 | ENSOANG00000041317 | O_anatinus | Animals | mono_intergenic | H/ACA | | | intergenic |
| ENSOANG00000041318 | ENSOANG00000041318 | O_anatinus | Animals | mono_intergenic | H/ACA | | | intergenic |
| ENSOANG00000041319 | ENSOANG00000041319 | O_anatinus | Animals | mono_intergenic | H/ACA | | | intergenic |
| ENSOANG00000041321 | ENSOANG00000041321 | O_anatinus | Animals | mono_intronic | H/ACA | ENSOANG00000004763 | EDNRB | protein_coding |
| ENSOANG00000041322 | ENSOANG00000041322 | O_anatinus | Animals | mono_intronic | H/ACA | ENSOANG00000004329 | SDK1 | protein_coding |
| ENSOANG00000041324 | ENSOANG00000041324 | O_anatinus | Animals | mono_intergenic | H/ACA | | | intergenic |
| ENSOANG00000041327 | ENSOANG00000041327 | O_anatinus | Animals | mono_intergenic | H/ACA | | | intergenic |
| ENSOANG00000041329 | ENSOANG00000041329 | O_anatinus | Animals | mono_intergenic | H/ACA | | | intergenic |
| ENSOANG00000041334 | ENSOANG00000041334 | O_anatinus | Animals | mono_intergenic | H/ACA | | | intergenic |
| ENSOANG00000041335 | ENSOANG00000041335 | O_anatinus | Animals | mono_intergenic | H/ACA | | | intergenic |
| ENSOANG00000041340 | ENSOANG00000041340 | O_anatinus | Animals | mono_intronic | H/ACA | ENSOANG00000048834 | BMP5 | protein_coding |
| ENSOANG00000041342 | ENSOANG00000041342 | O_anatinus | Animals | mono_intronic | H/ACA | ENSOANG00000004451 | IL1RAP | protein_coding |
| ENSOANG00000041343 | ENSOANG00000041343 | O_anatinus | Animals | mono_intergenic | H/ACA | | | intergenic |
| ENSOANG00000041346 | ENSOANG00000041346 | O_anatinus | Animals | mono_intergenic | H/ACA | | | intergenic |
| ENSOANG00000041347 | ENSOANG00000041347 | O_anatinus | Animals | mono_intergenic | H/ACA | | | intergenic |
| ENSOANG00000041350 | ENSOANG00000041350 | O_anatinus | Animals | mono_intergenic | H/ACA | | | intergenic |
| ENSOANG00000041357 | ENSOANG00000041357 | O_anatinus | Animals | mono_intronic | H/ACA | ENSOANG00000014625 | LRFN2 | protein_coding |
| ENSOANG00000041360 | ENSOANG00000041360 | O_anatinus | Animals | mono_intergenic | H/ACA | | | intergenic |
| ENSOANG00000041362 | ENSOANG00000041362 | O_anatinus | Animals | mono_intergenic | H/ACA | | | intergenic |
| ENSOANG00000041363 | ENSOANG00000041363 | O_anatinus | Animals | mono_intergenic | H/ACA | | | intergenic |
| ENSOANG00000041364 | ENSOANG00000041364 | O_anatinus | Animals | mono_intergenic | H/ACA | | | intergenic |
| ENSOANG00000041366 | ENSOANG00000041366 | O_anatinus | Animals | mono_intergenic | H/ACA | | | intergenic |
| ENSOANG00000041369 | ENSOANG00000041369 | O_anatinus | Animals | mono_intergenic | H/ACA | | | intergenic |
| ENSOANG00000041373 | ENSOANG00000041373 | O_anatinus | Animals | mono_intergenic | H/ACA | | | intergenic |
| ENSOANG00000041374 | ENSOANG00000041374 | O_anatinus | Animals | mono_intergenic | H/ACA | | | intergenic |
| ENSOANG00000041375 | ENSOANG00000041375 | O_anatinus | Animals | mono_intergenic | H/ACA | | | intergenic |
| ENSOANG00000041376 | ENSOANG00000041376 | O_anatinus | Animals | mono_intergenic | H/ACA | | | intergenic |
| ENSOANG00000041377 | ENSOANG00000041377 | O_anatinus | Animals | mono_intergenic | H/ACA | | | intergenic |
| ENSOANG00000041378 | ENSOANG00000041378 | O_anatinus | Animals | mono_intergenic | H/ACA | | | intergenic |
| ENSOANG00000041379 | ENSOANG00000041379 | O_anatinus | Animals | mono_intergenic | H/ACA | | | intergenic |
| ENSOANG00000041384 | ENSOANG00000041384 | O_anatinus | Animals | mono_intergenic | H/ACA | | | intergenic |
| ENSOANG00000041388 | ENSOANG00000041388 | O_anatinus | Animals | mono_intergenic | H/ACA | | | intergenic |
| ENSOANG00000041391 | ENSOANG00000041391 | O_anatinus | Animals | mono_intergenic | H/ACA | | | intergenic |
| ENSOANG00000041395 | ENSOANG00000041395 | O_anatinus | Animals | mono_intergenic | H/ACA | | | intergenic |
| ENSOANG00000041398 | ENSOANG00000041398 | O_anatinus | Animals | mono_intergenic | H/ACA | | | intergenic |
| ENSOANG00000041399 | ENSOANG00000041399 | O_anatinus | Animals | mono_intergenic | H/ACA | | | intergenic |
| ENSOANG00000041400 | ENSOANG00000041400 | O_anatinus | Animals | mono_intronic | H/ACA | ENSOANG00000010591 | CIPC | protein_coding |

| | | | | | | | | |
|---|---|---|---|---|---|---|---|---|
| ENSOANG00000041404 | ENSOANG00000041404 | O_anatinus | Animals | mono_intergenic | H/ACA | | | intergenic |
| ENSOANG00000041407 | ENSOANG00000041407 | O_anatinus | Animals | mono_intergenic | H/ACA | | | intergenic |
| ENSOANG00000041409 | ENSOANG00000041409 | O_anatinus | Animals | mono_intergenic | H/ACA | | | intergenic |
| ENSOANG00000041411 | ENSOANG00000041411 | O_anatinus | Animals | mono_intergenic | H/ACA | | | intergenic |
| ENSOANG00000041412 | ENSOANG00000041412 | O_anatinus | Animals | mono_intergenic | H/ACA | | | intergenic |
| ENSOANG00000041415 | ENSOANG00000041415 | O_anatinus | Animals | mono_intergenic | H/ACA | | | intergenic |
| ENSOANG00000041416 | ENSOANG00000041416 | O_anatinus | Animals | mono_intergenic | H/ACA | | | intergenic |
| ENSOANG00000041419 | ENSOANG00000041419 | O_anatinus | Animals | mono_intergenic | H/ACA | | | intergenic |
| ENSOANG00000041423 | ENSOANG00000041423 | O_anatinus | Animals | mono_intergenic | H/ACA | | | intergenic |
| ENSOANG00000041424 | ENSOANG00000041424 | O_anatinus | Animals | mono_intronic | H/ACA | ENSOANG00000048824 | GOLM1 | protein_coding |
| ENSOANG00000041428 | ENSOANG00000041428 | O_anatinus | Animals | mono_intergenic | H/ACA | | | intergenic |
| ENSOANG00000041429 | ENSOANG00000041429 | O_anatinus | Animals | mono_intronic | H/ACA | ENSOANG00000004623 | AFF3 | protein_coding |
| ENSOANG00000041430 | ENSOANG00000041430 | O_anatinus | Animals | mono_intergenic | H/ACA | | | intergenic |
| ENSOANG00000041431 | ENSOANG00000041431 | O_anatinus | Animals | mono_intergenic | H/ACA | | | intergenic |
| ENSOANG00000041432 | ENSOANG00000041432 | O_anatinus | Animals | mono_intergenic | H/ACA | | | intergenic |
| ENSOANG00000041433 | ENSOANG00000041433 | O_anatinus | Animals | mono_intergenic | H/ACA | | | intergenic |
| ENSOANG00000041437 | ENSOANG00000041437 | O_anatinus | Animals | mono_intergenic | H/ACA | | | intergenic |
| ENSOANG00000041438 | ENSOANG00000041438 | O_anatinus | Animals | mono_intergenic | H/ACA | | | intergenic |
| ENSOANG00000041453 | ENSOANG00000041453 | O_anatinus | Animals | mono_intergenic | H/ACA | | | intergenic |
| ENSOANG00000041455 | ENSOANG00000041455 | O_anatinus | Animals | mono_intergenic | H/ACA | | | intergenic |
| ENSOANG00000041458 | ENSOANG00000041458 | O_anatinus | Animals | mono_intronic | H/ACA | ENSOANG00000041906 | | non_coding |
| ENSOANG00000041459 | ENSOANG00000041459 | O_anatinus | Animals | mono_intronic | H/ACA | ENSOANG00000004866 | LDB2 | protein_coding |
| ENSOANG00000041460 | ENSOANG00000041460 | O_anatinus | Animals | mono_intergenic | H/ACA | | | intergenic |
| ENSOANG00000041461 | ENSOANG00000041461 | O_anatinus | Animals | mono_intergenic | H/ACA | | | intergenic |
| ENSOANG00000041463 | ENSOANG00000041463 | O_anatinus | Animals | mono_intergenic | H/ACA | | | intergenic |
| ENSOANG00000041464 | ENSOANG00000041464 | O_anatinus | Animals | mono_intergenic | H/ACA | | | intergenic |
| ENSOANG00000041465 | ENSOANG00000041465 | O_anatinus | Animals | mono_intergenic | H/ACA | | | intergenic |
| ENSOANG00000041466 | ENSOANG00000041466 | O_anatinus | Animals | mono_intergenic | H/ACA | | | intergenic |
| ENSOANG00000041468 | ENSOANG00000041468 | O_anatinus | Animals | mono_intergenic | H/ACA | | | intergenic |
| ENSOANG00000041473 | ENSOANG00000041473 | O_anatinus | Animals | mono_intergenic | H/ACA | | | intergenic |
| ENSOANG00000041474 | ENSOANG00000041474 | O_anatinus | Animals | mono_intergenic | H/ACA | | | intergenic |
| ENSOANG00000041484 | ENSOANG00000041484 | O_anatinus | Animals | mono_intergenic | H/ACA | | | intergenic |
| ENSOANG00000041490 | ENSOANG00000041490 | O_anatinus | Animals | mono_intergenic | H/ACA | | | intergenic |
| ENSOANG00000041493 | ENSOANG00000041493 | O_anatinus | Animals | mono_intergenic | H/ACA | | | intergenic |
| ENSOANG00000041494 | ENSOANG00000041494 | O_anatinus | Animals | mono_intergenic | H/ACA | | | intergenic |
| ENSOANG00000041501 | ENSOANG00000041501 | O_anatinus | Animals | mono_intergenic | H/ACA | | | intergenic |
| ENSOANG00000041503 | ENSOANG00000041503 | O_anatinus | Animals | mono_intergenic | H/ACA | | | intergenic |
| ENSOANG00000041506 | ENSOANG00000041506 | O_anatinus | Animals | mono_intergenic | H/ACA | | | intergenic |
| ENSOANG00000041507 | ENSOANG00000041507 | O_anatinus | Animals | mono_intergenic | H/ACA | | | intergenic |
| ENSOANG00000041508 | ENSOANG00000041508 | O_anatinus | Animals | mono_intergenic | H/ACA | | | intergenic |
| ENSOANG00000041510 | ENSOANG00000041510 | O_anatinus | Animals | mono_intergenic | H/ACA | | | intergenic |

| | | | | | | | | |
|---|---|---|---|---|---|---|---|---|
| ENSOANG00000041512 | ENSOANG00000041512 | O_anatinus | Animals | mono_intergenic | H/ACA | | | intergenic |
| ENSOANG00000041519 | ENSOANG00000041519 | O_anatinus | Animals | mono_intergenic | H/ACA | | | intergenic |
| ENSOANG00000041522 | ENSOANG00000041522 | O_anatinus | Animals | mono_intergenic | H/ACA | | | intergenic |
| ENSOANG00000041523 | ENSOANG00000041523 | O_anatinus | Animals | mono_intergenic | H/ACA | | | intergenic |
| ENSOANG00000041528 | ENSOANG00000041528 | O_anatinus | Animals | mono_intergenic | H/ACA | | | intergenic |
| ENSOANG00000041530 | ENSOANG00000041530 | O_anatinus | Animals | mono_intergenic | H/ACA | | | intergenic |
| ENSOANG00000041531 | ENSOANG00000041531 | O_anatinus | Animals | mono_intergenic | H/ACA | | | intergenic |
| ENSOANG00000041532 | ENSOANG00000041532 | O_anatinus | Animals | mono_intergenic | H/ACA | | | intergenic |
| ENSOANG00000041533 | ENSOANG00000041533 | O_anatinus | Animals | mono_intergenic | H/ACA | | | intergenic |
| ENSOANG00000041537 | ENSOANG00000041537 | O_anatinus | Animals | mono_intergenic | H/ACA | | | intergenic |
| ENSOANG00000041539 | ENSOANG00000041539 | O_anatinus | Animals | mono_intergenic | H/ACA | | | intergenic |
| ENSOANG00000041540 | ENSOANG00000041540 | O_anatinus | Animals | mono_intergenic | H/ACA | | | intergenic |
| ENSOANG00000041542 | ENSOANG00000041542 | O_anatinus | Animals | mono_intergenic | H/ACA | | | intergenic |
| ENSOANG00000041543 | ENSOANG00000041543 | O_anatinus | Animals | mono_intergenic | H/ACA | | | intergenic |
| ENSOANG00000041546 | ENSOANG00000041546 | O_anatinus | Animals | mono_intergenic | H/ACA | | | intergenic |
| ENSOANG00000041547 | ENSOANG00000041547 | O_anatinus | Animals | mono_intergenic | H/ACA | | | intergenic |
| ENSOANG00000041549 | ENSOANG00000041549 | O_anatinus | Animals | mono_intergenic | H/ACA | | | intergenic |
| ENSOANG00000041550 | ENSOANG00000041550 | O_anatinus | Animals | mono_intergenic | H/ACA | | | intergenic |
| ENSOANG00000041551 | ENSOANG00000041551 | O_anatinus | Animals | mono_intergenic | H/ACA | | | intergenic |
| ENSOANG00000041554 | ENSOANG00000041554 | O_anatinus | Animals | mono_intergenic | H/ACA | | | intergenic |
| ENSOANG00000041555 | ENSOANG00000041555 | O_anatinus | Animals | mono_intergenic | H/ACA | | | intergenic |
| ENSOANG00000041556 | ENSOANG00000041556 | O_anatinus | Animals | mono_intergenic | H/ACA | | | intergenic |
| ENSOANG00000041558 | ENSOANG00000041558 | O_anatinus | Animals | mono_intergenic | H/ACA | | | intergenic |
| ENSOANG00000041561 | ENSOANG00000041561 | O_anatinus | Animals | mono_intergenic | H/ACA | | | intergenic |
| ENSOANG00000041565 | ENSOANG00000041565 | O_anatinus | Animals | mono_intergenic | H/ACA | | | intergenic |
| ENSOANG00000041569 | ENSOANG00000041569 | O_anatinus | Animals | mono_intergenic | H/ACA | | | intergenic |
| ENSOANG00000041570 | ENSOANG00000041570 | O_anatinus | Animals | mono_intergenic | H/ACA | | | intergenic |
| ENSOANG00000041571 | ENSOANG00000041571 | O_anatinus | Animals | mono_intergenic | H/ACA | | | intergenic |
| ENSOANG00000041572 | ENSOANG00000041572 | O_anatinus | Animals | mono_intergenic | H/ACA | | | intergenic |
| ENSOANG00000041573 | ENSOANG00000041573 | O_anatinus | Animals | mono_intergenic | H/ACA | | | intergenic |
| ENSOANG00000041574 | ENSOANG00000041574 | O_anatinus | Animals | mono_intergenic | H/ACA | | | intergenic |
| ENSOANG00000041575 | ENSOANG00000041575 | O_anatinus | Animals | mono_intergenic | H/ACA | | | intergenic |
| ENSOANG00000041584 | ENSOANG00000041584 | O_anatinus | Animals | mono_intergenic | H/ACA | | | intergenic |
| ENSOANG00000041587 | ENSOANG00000041587 | O_anatinus | Animals | mono_intergenic | H/ACA | | | intergenic |
| ENSOANG00000041589 | ENSOANG00000041589 | O_anatinus | Animals | mono_intergenic | H/ACA | | | intergenic |
| ENSOANG00000041590 | ENSOANG00000041590 | O_anatinus | Animals | mono_intergenic | H/ACA | | | intergenic |
| ENSOANG00000041595 | ENSOANG00000041595 | O_anatinus | Animals | mono_intergenic | H/ACA | | | intergenic |
| ENSOANG00000041598 | ENSOANG00000041598 | O_anatinus | Animals | mono_intronic | H/ACA | ENSOANG00000014092 | TES | protein_coding |
| ENSOANG00000041600 | ENSOANG00000041600 | O_anatinus | Animals | mono_intergenic | H/ACA | | | intergenic |
| ENSOANG00000041604 | ENSOANG00000041604 | O_anatinus | Animals | mono_intergenic | H/ACA | | | intergenic |
| ENSOANG00000041606 | ENSOANG00000041606 | O_anatinus | Animals | mono_intergenic | H/ACA | | | intergenic |

| | | | | | | | | |
|---|---|---|---|---|---|---|---|---|
| ENSOANG00000041619 | ENSOANG00000041619 | O_anatinus | Animals | mono_intergenic | H/ACA | | | intergenic |
| ENSOANG00000041623 | ENSOANG00000041623 | O_anatinus | Animals | mono_intergenic | H/ACA | | | intergenic |
| ENSOANG00000041624 | ENSOANG00000041624 | O_anatinus | Animals | mono_intergenic | H/ACA | | | intergenic |
| ENSOANG00000041625 | ENSOANG00000041625 | O_anatinus | Animals | mono_intergenic | H/ACA | | | intergenic |
| ENSOANG00000041626 | ENSOANG00000041626 | O_anatinus | Animals | mono_intergenic | H/ACA | | | intergenic |
| ENSOANG00000041635 | ENSOANG00000041635 | O_anatinus | Animals | mono_intergenic | H/ACA | | | intergenic |
| ENSOANG00000041636 | ENSOANG00000041636 | O_anatinus | Animals | mono_intronic | H/ACA | ENSOANG00000012051 | | protein_coding |
| ENSOANG00000041640 | ENSOANG00000041640 | O_anatinus | Animals | mono_intergenic | H/ACA | | | intergenic |
| ENSOANG00000041643 | ENSOANG00000041643 | O_anatinus | Animals | mono_intergenic | H/ACA | | | intergenic |
| ENSOANG00000041645 | ENSOANG00000041645 | O_anatinus | Animals | mono_intergenic | H/ACA | | | intergenic |
| ENSOANG00000041646 | ENSOANG00000041646 | O_anatinus | Animals | mono_intergenic | H/ACA | | | intergenic |
| ENSOANG00000041652 | ENSOANG00000041652 | O_anatinus | Animals | mono_intergenic | H/ACA | | | intergenic |
| ENSOANG00000041656 | ENSOANG00000041656 | O_anatinus | Animals | mono_intergenic | H/ACA | | | intergenic |
| ENSOANG00000041658 | ENSOANG00000041658 | O_anatinus | Animals | mono_intronic | H/ACA | ENSOANG00000015220 | THEMIS | protein_coding |
| ENSOANG00000041661 | ENSOANG00000041661 | O_anatinus | Animals | mono_intergenic | H/ACA | | | intergenic |
| ENSOANG00000041662 | ENSOANG00000041662 | O_anatinus | Animals | mono_intergenic | H/ACA | | | intergenic |
| ENSOANG00000041665 | ENSOANG00000041665 | O_anatinus | Animals | mono_intergenic | H/ACA | | | intergenic |
| ENSOANG00000041671 | ENSOANG00000041671 | O_anatinus | Animals | mono_intergenic | H/ACA | | | intergenic |
| ENSOANG00000041672 | ENSOANG00000041672 | O_anatinus | Animals | mono_intergenic | H/ACA | | | intergenic |
| ENSOANG00000041673 | ENSOANG00000041673 | O_anatinus | Animals | mono_intergenic | H/ACA | | | intergenic |
| ENSOANG00000041675 | ENSOANG00000041675 | O_anatinus | Animals | mono_intergenic | H/ACA | | | intergenic |
| ENSOANG00000041677 | ENSOANG00000041677 | O_anatinus | Animals | mono_intronic | H/ACA | ENSOANG00000011993 | EFL1 | protein_coding |
| ENSOANG00000041679 | ENSOANG00000041679 | O_anatinus | Animals | mono_intergenic | H/ACA | | | intergenic |
| ENSOANG00000041684 | ENSOANG00000041684 | O_anatinus | Animals | mono_intergenic | H/ACA | | | intergenic |
| ENSOANG00000041685 | ENSOANG00000041685 | O_anatinus | Animals | mono_intergenic | H/ACA | | | intergenic |
| ENSOANG00000041686 | ENSOANG00000041686 | O_anatinus | Animals | mono_intronic | H/ACA | ENSOANG00000003085 | | protein_coding |
| ENSOANG00000041688 | ENSOANG00000041688 | O_anatinus | Animals | mono_intronic | H/ACA | ENSOANG00000037449 | | protein_coding |
| ENSOANG00000041689 | ENSOANG00000041689 | O_anatinus | Animals | mono_intergenic | H/ACA | | | intergenic |
| ENSOANG00000041700 | ENSOANG00000041700 | O_anatinus | Animals | mono_intergenic | H/ACA | | | intergenic |
| ENSOANG00000041704 | ENSOANG00000041704 | O_anatinus | Animals | mono_intergenic | H/ACA | | | intergenic |
| ENSOANG00000041707 | ENSOANG00000041707 | O_anatinus | Animals | mono_intergenic | H/ACA | | | intergenic |
| ENSOANG00000041708 | ENSOANG00000041708 | O_anatinus | Animals | mono_intergenic | H/ACA | | | intergenic |
| ENSOANG00000041711 | ENSOANG00000041711 | O_anatinus | Animals | mono_intergenic | H/ACA | | | intergenic |
| ENSOANG00000041712 | ENSOANG00000041712 | O_anatinus | Animals | mono_intergenic | H/ACA | | | intergenic |
| ENSOANG00000041716 | ENSOANG00000041716 | O_anatinus | Animals | mono_intergenic | H/ACA | | | intergenic |
| ENSOANG00000041725 | ENSOANG00000041725 | O_anatinus | Animals | mono_intronic | H/ACA | ENSOANG00000000204 | CLSTN2 | protein_coding |
| ENSOANG00000041726 | ENSOANG00000041726 | O_anatinus | Animals | mono_intronic | H/ACA | ENSOANG00000008592 | RAPGEF4 | protein_coding |
| ENSOANG00000041732 | ENSOANG00000041732 | O_anatinus | Animals | mono_intergenic | H/ACA | | | intergenic |
| ENSOANG00000041733 | ENSOANG00000041733 | O_anatinus | Animals | mono_intergenic | H/ACA | | | intergenic |
| ENSOANG00000041738 | ENSOANG00000041738 | O_anatinus | Animals | mono_intergenic | H/ACA | | | intergenic |
| ENSOANG00000041739 | ENSOANG00000041739 | O_anatinus | Animals | mono_intergenic | H/ACA | | | intergenic |

| | | | | | | | | |
|---|---|---|---|---|---|---|---|---|
| ENSOANG00000041741 | ENSOANG00000041741 | O_anatinus | Animals | mono_intergenic | H/ACA | | | intergenic |
| ENSOANG00000041742 | ENSOANG00000041742 | O_anatinus | Animals | mono_intergenic | H/ACA | | | intergenic |
| ENSOANG00000041746 | ENSOANG00000041746 | O_anatinus | Animals | mono_intronic | H/ACA | ENSOANG00000001936 | TECPR1 | protein_coding |
| ENSOANG00000041750 | ENSOANG00000041750 | O_anatinus | Animals | mono_intergenic | H/ACA | | | intergenic |
| ENSOANG00000041757 | ENSOANG00000041757 | O_anatinus | Animals | intronic_cluster | H/ACA | ENSOANG00000010900 | GKAP1 | protein_coding |
| ENSOANG00000041760 | ENSOANG00000041760 | O_anatinus | Animals | mono_intergenic | H/ACA | | | intergenic |
| ENSOANG00000041763 | ENSOANG00000041763 | O_anatinus | Animals | mono_intergenic | H/ACA | | | intergenic |
| ENSOANG00000041764 | ENSOANG00000041764 | O_anatinus | Animals | mono_intergenic | H/ACA | | | intergenic |
| ENSOANG00000041765 | ENSOANG00000041765 | O_anatinus | Animals | mono_intergenic | H/ACA | | | intergenic |
| ENSOANG00000041766 | ENSOANG00000041766 | O_anatinus | Animals | mono_intergenic | H/ACA | | | intergenic |
| ENSOANG00000041767 | ENSOANG00000041767 | O_anatinus | Animals | mono_intergenic | H/ACA | | | intergenic |
| ENSOANG00000041769 | ENSOANG00000041769 | O_anatinus | Animals | mono_intronic | H/ACA | ENSOANG00000003405 | SNRPC | protein_coding |
| ENSOANG00000041770 | ENSOANG00000041770 | O_anatinus | Animals | mono_intergenic | H/ACA | | | intergenic |
| ENSOANG00000041774 | ENSOANG00000041774 | O_anatinus | Animals | mono_intergenic | H/ACA | | | intergenic |
| ENSOANG00000041776 | ENSOANG00000041776 | O_anatinus | Animals | mono_intergenic | H/ACA | | | intergenic |
| ENSOANG00000041779 | ENSOANG00000041779 | O_anatinus | Animals | mono_intronic | H/ACA | ENSOANG00000002499 | RALGAPA2 | protein_coding |
| ENSOANG00000041780 | ENSOANG00000041780 | O_anatinus | Animals | mono_intronic | H/ACA | ENSOANG00000050686 | EIF4A1 | protein_coding |
| ENSOANG00000041781 | ENSOANG00000041781 | O_anatinus | Animals | mono_intergenic | H/ACA | | | intergenic |
| ENSOANG00000041785 | ENSOANG00000041785 | O_anatinus | Animals | intronic_cluster | H/ACA | ENSOANG00000045577 | NUBP1 | protein_coding |
| ENSOANG00000041786 | ENSOANG00000041786 | O_anatinus | Animals | mono_intergenic | H/ACA | | | intergenic |
| ENSOANG00000041787 | ENSOANG00000041787 | O_anatinus | Animals | mono_intronic | H/ACA | ENSOANG00000044143 | DOCK8 | protein_coding |
| ENSOANG00000041788 | ENSOANG00000041788 | O_anatinus | Animals | mono_intergenic | H/ACA | | | intergenic |
| ENSOANG00000041789 | ENSOANG00000041789 | O_anatinus | Animals | mono_intergenic | H/ACA | | | intergenic |
| ENSOANG00000041793 | ENSOANG00000041793 | O_anatinus | Animals | mono_intergenic | H/ACA | | | intergenic |
| ENSOANG00000041795 | ENSOANG00000041795 | O_anatinus | Animals | intronic_cluster | H/ACA | ENSOANG00000001492 | PTPRD | protein_coding |
| ENSOANG00000041797 | ENSOANG00000041797 | O_anatinus | Animals | mono_intergenic | H/ACA | | | intergenic |
| ENSOANG00000041798 | ENSOANG00000041798 | O_anatinus | Animals | mono_intergenic | H/ACA | | | intergenic |
| ENSOANG00000041799 | ENSOANG00000041799 | O_anatinus | Animals | mono_intergenic | H/ACA | | | intergenic |
| ENSOANG00000041800 | ENSOANG00000041800 | O_anatinus | Animals | mono_intergenic | H/ACA | | | intergenic |
| ENSOANG00000041803 | ENSOANG00000041803 | O_anatinus | Animals | mono_intergenic | H/ACA | | | intergenic |
| ENSOANG00000041805 | ENSOANG00000041805 | O_anatinus | Animals | mono_intergenic | H/ACA | | | intergenic |
| ENSOANG00000041807 | ENSOANG00000041807 | O_anatinus | Animals | mono_intergenic | H/ACA | | | intergenic |
| ENSOANG00000041808 | ENSOANG00000041808 | O_anatinus | Animals | mono_intergenic | H/ACA | | | intergenic |
| ENSOANG00000041809 | SNORA50A | O_anatinus | Animals | intronic_cluster | H/ACA | ENSOANG00000012109 | CNOT1 | protein_coding |
| ENSOANG00000041810 | ENSOANG00000041810 | O_anatinus | Animals | mono_intergenic | H/ACA | | | intergenic |
| ENSOANG00000041811 | ENSOANG00000041811 | O_anatinus | Animals | mono_intergenic | H/ACA | | | intergenic |
| ENSOANG00000041814 | ENSOANG00000041814 | O_anatinus | Animals | mono_intergenic | H/ACA | | | intergenic |
| ENSOANG00000041816 | ENSOANG00000041816 | O_anatinus | Animals | mono_intergenic | H/ACA | | | intergenic |
| ENSOANG00000041817 | ENSOANG00000041817 | O_anatinus | Animals | mono_intergenic | H/ACA | | | intergenic |
| ENSOANG00000041819 | ENSOANG00000041819 | O_anatinus | Animals | mono_intergenic | H/ACA | | | intergenic |
| ENSOANG00000041820 | ENSOANG00000041820 | O_anatinus | Animals | mono_intergenic | H/ACA | | | intergenic |

| | | | | | | | | |
|---|---|---|---|---|---|---|---|---|
| ENSOANG00000041827 | ENSOANG00000041827 | O_anatinus | Animals | mono_intergenic | H/ACA | | | intergenic |
| ENSOANG00000041828 | ENSOANG00000041828 | O_anatinus | Animals | mono_intergenic | H/ACA | | | intergenic |
| ENSOANG00000041829 | ENSOANG00000041829 | O_anatinus | Animals | mono_intergenic | H/ACA | | | intergenic |
| ENSOANG00000041842 | ENSOANG00000041842 | O_anatinus | Animals | mono_intergenic | H/ACA | | | intergenic |
| ENSOANG00000041844 | ENSOANG00000041844 | O_anatinus | Animals | mono_intergenic | H/ACA | | | intergenic |
| ENSOANG00000041845 | ENSOANG00000041845 | O_anatinus | Animals | mono_intergenic | H/ACA | | | intergenic |
| ENSOANG00000041850 | ENSOANG00000041850 | O_anatinus | Animals | mono_intergenic | H/ACA | | | intergenic |
| ENSOANG00000041852 | ENSOANG00000041852 | O_anatinus | Animals | mono_intronic | H/ACA | ENSOANG00000013232 | IFT74 | protein_coding |
| ENSOANG00000041856 | ENSOANG00000041856 | O_anatinus | Animals | mono_intergenic | H/ACA | | | intergenic |
| ENSOANG00000041858 | ENSOANG00000041858 | O_anatinus | Animals | mono_intergenic | H/ACA | | | intergenic |
| ENSOANG00000041860 | ENSOANG00000041860 | O_anatinus | Animals | mono_intergenic | H/ACA | | | intergenic |
| ENSOANG00000041866 | ENSOANG00000041866 | O_anatinus | Animals | mono_intronic | H/ACA | ENSOANG00000006622 | PLXNC1 | protein_coding |
| ENSOANG00000041867 | ENSOANG00000041867 | O_anatinus | Animals | mono_intergenic | H/ACA | | | intergenic |
| ENSOANG00000041873 | ENSOANG00000041873 | O_anatinus | Animals | mono_intronic | H/ACA | ENSOANG00000002895 | KRT25 | protein_coding |
| ENSOANG00000041874 | ENSOANG00000041874 | O_anatinus | Animals | mono_intergenic | H/ACA | | | intergenic |
| ENSOANG00000041879 | ENSOANG00000041879 | O_anatinus | Animals | mono_intergenic | H/ACA | | | intergenic |
| ENSOANG00000041880 | ENSOANG00000041880 | O_anatinus | Animals | mono_intergenic | H/ACA | | | intergenic |
| ENSOANG00000041882 | ENSOANG00000041882 | O_anatinus | Animals | mono_intergenic | H/ACA | | | intergenic |
| ENSOANG00000041885 | ENSOANG00000041885 | O_anatinus | Animals | mono_intergenic | H/ACA | | | intergenic |
| ENSOANG00000041886 | ENSOANG00000041886 | O_anatinus | Animals | mono_intergenic | H/ACA | | | intergenic |
| ENSOANG00000041888 | ENSOANG00000041888 | O_anatinus | Animals | mono_intergenic | H/ACA | | | intergenic |
| ENSOANG00000041896 | ENSOANG00000041896 | O_anatinus | Animals | mono_intergenic | H/ACA | | | intergenic |
| ENSOANG00000041900 | ENSOANG00000041900 | O_anatinus | Animals | mono_intergenic | H/ACA | | | intergenic |
| ENSOANG00000041901 | ENSOANG00000041901 | O_anatinus | Animals | mono_intergenic | H/ACA | | | intergenic |
| ENSOANG00000041909 | ENSOANG00000041909 | O_anatinus | Animals | mono_intergenic | H/ACA | | | intergenic |
| ENSOANG00000041910 | ENSOANG00000041910 | O_anatinus | Animals | mono_intergenic | H/ACA | | | intergenic |
| ENSOANG00000041915 | ENSOANG00000041915 | O_anatinus | Animals | mono_intronic | H/ACA | ENSOANG00000004134 | | protein_coding |
| ENSOANG00000041917 | ENSOANG00000041917 | O_anatinus | Animals | mono_intergenic | H/ACA | | | intergenic |
| ENSOANG00000041918 | ENSOANG00000041918 | O_anatinus | Animals | mono_intergenic | H/ACA | | | intergenic |
| ENSOANG00000041919 | ENSOANG00000041919 | O_anatinus | Animals | mono_intergenic | H/ACA | | | intergenic |
| ENSOANG00000041922 | ENSOANG00000041922 | O_anatinus | Animals | mono_intergenic | H/ACA | | | intergenic |
| ENSOANG00000041924 | ENSOANG00000041924 | O_anatinus | Animals | mono_intergenic | H/ACA | | | intergenic |
| ENSOANG00000041926 | ENSOANG00000041926 | O_anatinus | Animals | mono_intergenic | H/ACA | | | intergenic |
| ENSOANG00000041927 | ENSOANG00000041927 | O_anatinus | Animals | mono_intergenic | H/ACA | | | intergenic |
| ENSOANG00000041929 | ENSOANG00000041929 | O_anatinus | Animals | mono_intergenic | H/ACA | | | intergenic |
| ENSOANG00000041930 | ENSOANG00000041930 | O_anatinus | Animals | mono_intergenic | H/ACA | | | intergenic |
| ENSOANG00000041933 | ENSOANG00000041933 | O_anatinus | Animals | mono_intergenic | H/ACA | | | intergenic |
| ENSOANG00000041935 | ENSOANG00000041935 | O_anatinus | Animals | mono_intronic | H/ACA | ENSOANG00000036092 | | protein_coding |
| ENSOANG00000041937 | ENSOANG00000041937 | O_anatinus | Animals | mono_intergenic | H/ACA | | | intergenic |
| ENSOANG00000041941 | ENSOANG00000041941 | O_anatinus | Animals | mono_intergenic | H/ACA | | | intergenic |
| ENSOANG00000041945 | ENSOANG00000041945 | O_anatinus | Animals | mono_intergenic | H/ACA | | | intergenic |

| | | | | | | | | |
|---|---|---|---|---|---|---|---|---|
| ENSOANG00000041946 | ENSOANG00000041946 | O_anatinus | Animals | mono_intergenic | H/ACA | | | intergenic |
| ENSOANG00000041948 | ENSOANG00000041948 | O_anatinus | Animals | mono_intergenic | H/ACA | | | intergenic |
| ENSOANG00000041950 | ENSOANG00000041950 | O_anatinus | Animals | mono_intergenic | H/ACA | | | intergenic |
| ENSOANG00000041951 | ENSOANG00000041951 | O_anatinus | Animals | mono_intergenic | H/ACA | | | intergenic |
| ENSOANG00000041953 | ENSOANG00000041953 | O_anatinus | Animals | mono_intergenic | H/ACA | | | intergenic |
| ENSOANG00000041954 | ENSOANG00000041954 | O_anatinus | Animals | mono_intergenic | H/ACA | | | intergenic |
| ENSOANG00000041956 | ENSOANG00000041956 | O_anatinus | Animals | mono_intergenic | H/ACA | | | intergenic |
| ENSOANG00000041960 | ENSOANG00000041960 | O_anatinus | Animals | mono_intergenic | H/ACA | | | intergenic |
| ENSOANG00000041961 | ENSOANG00000041961 | O_anatinus | Animals | mono_intergenic | H/ACA | | | intergenic |
| ENSOANG00000041962 | ENSOANG00000041962 | O_anatinus | Animals | mono_intergenic | H/ACA | | | intergenic |
| ENSOANG00000041966 | ENSOANG00000041966 | O_anatinus | Animals | mono_intronic | H/ACA | ENSOANG00000031935 | | protein_coding |
| ENSOANG00000041967 | ENSOANG00000041967 | O_anatinus | Animals | mono_intergenic | H/ACA | | | intergenic |
| ENSOANG00000041969 | ENSOANG00000041969 | O_anatinus | Animals | mono_intergenic | H/ACA | | | intergenic |
| ENSOANG00000041970 | ENSOANG00000041970 | O_anatinus | Animals | intronic_cluster | H/ACA | ENSOANG00000002334 | LTBP1 | protein_coding |
| ENSOANG00000041972 | ENSOANG00000041972 | O_anatinus | Animals | mono_intergenic | H/ACA | | | intergenic |
| ENSOANG00000041975 | ENSOANG00000041975 | O_anatinus | Animals | mono_intergenic | H/ACA | | | intergenic |
| ENSOANG00000041976 | ENSOANG00000041976 | O_anatinus | Animals | mono_intergenic | H/ACA | | | intergenic |
| ENSOANG00000041981 | ENSOANG00000041981 | O_anatinus | Animals | mono_intergenic | H/ACA | | | intergenic |
| ENSOANG00000041982 | ENSOANG00000041982 | O_anatinus | Animals | mono_intergenic | H/ACA | | | intergenic |
| ENSOANG00000041986 | ENSOANG00000041986 | O_anatinus | Animals | mono_intergenic | H/ACA | | | intergenic |
| ENSOANG00000041987 | ENSOANG00000041987 | O_anatinus | Animals | mono_intergenic | H/ACA | | | intergenic |
| ENSOANG00000041989 | ENSOANG00000041989 | O_anatinus | Animals | mono_intergenic | H/ACA | | | intergenic |
| ENSOANG00000041992 | ENSOANG00000041992 | O_anatinus | Animals | mono_intergenic | H/ACA | | | intergenic |
| ENSOANG00000041993 | ENSOANG00000041993 | O_anatinus | Animals | mono_intergenic | H/ACA | | | intergenic |
| ENSOANG00000041997 | ENSOANG00000041997 | O_anatinus | Animals | mono_intergenic | H/ACA | | | intergenic |
| ENSOANG00000041998 | ENSOANG00000041998 | O_anatinus | Animals | mono_intronic | H/ACA | ENSOANG00000047086 | | non_coding |
| ENSOANG00000042002 | ENSOANG00000042002 | O_anatinus | Animals | mono_intergenic | H/ACA | | | intergenic |
| ENSOANG00000042004 | ENSOANG00000042004 | O_anatinus | Animals | mono_intergenic | H/ACA | | | intergenic |
| ENSOANG00000042005 | ENSOANG00000042005 | O_anatinus | Animals | mono_intergenic | H/ACA | | | intergenic |
| ENSOANG00000042006 | ENSOANG00000042006 | O_anatinus | Animals | mono_intronic | H/ACA | ENSOANG00000002642 | RSBN1 | protein_coding |
| ENSOANG00000042010 | ENSOANG00000042010 | O_anatinus | Animals | mono_intronic | H/ACA | ENSOANG00000000916 | PRDX6 | protein_coding |
| ENSOANG00000042011 | ENSOANG00000042011 | O_anatinus | Animals | mono_intergenic | H/ACA | | | intergenic |
| ENSOANG00000042013 | ENSOANG00000042013 | O_anatinus | Animals | mono_intergenic | H/ACA | | | intergenic |
| ENSOANG00000042024 | ENSOANG00000042024 | O_anatinus | Animals | mono_intergenic | H/ACA | | | intergenic |
| ENSOANG00000042026 | ENSOANG00000042026 | O_anatinus | Animals | mono_intergenic | H/ACA | | | intergenic |
| ENSOANG00000042028 | ENSOANG00000042028 | O_anatinus | Animals | mono_intergenic | H/ACA | | | intergenic |
| ENSOANG00000042030 | ENSOANG00000042030 | O_anatinus | Animals | mono_intergenic | H/ACA | | | intergenic |
| ENSOANG00000042034 | ENSOANG00000042034 | O_anatinus | Animals | mono_intergenic | H/ACA | | | intergenic |
| ENSOANG00000042035 | ENSOANG00000042035 | O_anatinus | Animals | mono_intergenic | H/ACA | | | intergenic |
| ENSOANG00000042036 | ENSOANG00000042036 | O_anatinus | Animals | mono_intergenic | H/ACA | | | intergenic |
| ENSOANG00000042037 | ENSOANG00000042037 | O_anatinus | Animals | mono_intergenic | H/ACA | | | intergenic |

| | | | | | | | | |
|---|---|---|---|---|---|---|---|---|
| ENSOANG00000042039 | ENSOANG00000042039 | O_anatinus | Animals | mono_intergenic | H/ACA | | | intergenic |
| ENSOANG00000042044 | ENSOANG00000042044 | O_anatinus | Animals | mono_intronic | H/ACA | ENSOANG00000002334 | LTBP1 | protein_coding |
| ENSOANG00000042046 | ENSOANG00000042046 | O_anatinus | Animals | mono_intergenic | H/ACA | | | intergenic |
| ENSOANG00000042050 | ENSOANG00000042050 | O_anatinus | Animals | mono_intergenic | H/ACA | | | intergenic |
| ENSOANG00000042053 | ENSOANG00000042053 | O_anatinus | Animals | mono_intergenic | H/ACA | | | intergenic |
| ENSOANG00000042054 | ENSOANG00000042054 | O_anatinus | Animals | mono_intergenic | H/ACA | | | intergenic |
| ENSOANG00000042057 | ENSOANG00000042057 | O_anatinus | Animals | mono_intergenic | H/ACA | | | intergenic |
| ENSOANG00000042060 | ENSOANG00000042060 | O_anatinus | Animals | mono_intergenic | H/ACA | | | intergenic |
| ENSOANG00000042061 | ENSOANG00000042061 | O_anatinus | Animals | mono_intergenic | H/ACA | | | intergenic |
| ENSOANG00000042062 | ENSOANG00000042062 | O_anatinus | Animals | mono_intergenic | H/ACA | | | intergenic |
| ENSOANG00000042064 | ENSOANG00000042064 | O_anatinus | Animals | mono_intergenic | H/ACA | | | intergenic |
| ENSOANG00000042065 | ENSOANG00000042065 | O_anatinus | Animals | mono_intergenic | H/ACA | | | intergenic |
| ENSOANG00000042066 | ENSOANG00000042066 | O_anatinus | Animals | mono_intronic | H/ACA | ENSOANG00000010789 | TMEM181 | protein_coding |
| ENSOANG00000042070 | ENSOANG00000042070 | O_anatinus | Animals | mono_intergenic | H/ACA | | | intergenic |
| ENSOANG00000042073 | ENSOANG00000042073 | O_anatinus | Animals | mono_intergenic | H/ACA | | | intergenic |
| ENSOANG00000042074 | ENSOANG00000042074 | O_anatinus | Animals | mono_intergenic | H/ACA | | | intergenic |
| ENSOANG00000042075 | ENSOANG00000042075 | O_anatinus | Animals | mono_intronic | H/ACA | ENSOANG00000041653 | PPHLN1 | protein_coding |
| ENSOANG00000042088 | ENSOANG00000042088 | O_anatinus | Animals | mono_intergenic | H/ACA | | | intergenic |
| ENSOANG00000042094 | ENSOANG00000042094 | O_anatinus | Animals | mono_intergenic | H/ACA | | | intergenic |
| ENSOANG00000042098 | ENSOANG00000042098 | O_anatinus | Animals | mono_intergenic | H/ACA | | | intergenic |
| ENSOANG00000042099 | ENSOANG00000042099 | O_anatinus | Animals | mono_intergenic | H/ACA | | | intergenic |
| ENSOANG00000042100 | ENSOANG00000042100 | O_anatinus | Animals | mono_intergenic | H/ACA | | | intergenic |
| ENSOANG00000042102 | ENSOANG00000042102 | O_anatinus | Animals | mono_intergenic | H/ACA | | | intergenic |
| ENSOANG00000042105 | ENSOANG00000042105 | O_anatinus | Animals | mono_intergenic | H/ACA | | | intergenic |
| ENSOANG00000042106 | ENSOANG00000042106 | O_anatinus | Animals | mono_intergenic | H/ACA | | | intergenic |
| ENSOANG00000042111 | ENSOANG00000042111 | O_anatinus | Animals | mono_intergenic | H/ACA | | | intergenic |
| ENSOANG00000042114 | ENSOANG00000042114 | O_anatinus | Animals | mono_intergenic | H/ACA | | | intergenic |
| ENSOANG00000042115 | ENSOANG00000042115 | O_anatinus | Animals | mono_intergenic | H/ACA | | | intergenic |
| ENSOANG00000042121 | ENSOANG00000042121 | O_anatinus | Animals | mono_intronic | H/ACA | ENSOANG00000012433 | AFTPH | protein_coding |
| ENSOANG00000042122 | ENSOANG00000042122 | O_anatinus | Animals | mono_intergenic | H/ACA | | | intergenic |
| ENSOANG00000042124 | ENSOANG00000042124 | O_anatinus | Animals | mono_intronic | H/ACA | ENSOANG00000005614 | MEP1B | protein_coding |
| ENSOANG00000042126 | ENSOANG00000042126 | O_anatinus | Animals | mono_intergenic | H/ACA | | | intergenic |
| ENSOANG00000042131 | ENSOANG00000042131 | O_anatinus | Animals | mono_intergenic | H/ACA | | | intergenic |
| ENSOANG00000042132 | ENSOANG00000042132 | O_anatinus | Animals | mono_intergenic | H/ACA | | | intergenic |
| ENSOANG00000042134 | ENSOANG00000042134 | O_anatinus | Animals | mono_intergenic | H/ACA | | | intergenic |
| ENSOANG00000042135 | ENSOANG00000042135 | O_anatinus | Animals | mono_intergenic | H/ACA | | | intergenic |
| ENSOANG00000042136 | ENSOANG00000042136 | O_anatinus | Animals | mono_intergenic | H/ACA | | | intergenic |
| ENSOANG00000042138 | ENSOANG00000042138 | O_anatinus | Animals | mono_intergenic | H/ACA | | | intergenic |
| ENSOANG00000042139 | ENSOANG00000042139 | O_anatinus | Animals | mono_intronic | H/ACA | ENSOANG00000011799 | ELOVL5 | protein_coding |
| ENSOANG00000042143 | ENSOANG00000042143 | O_anatinus | Animals | mono_intergenic | H/ACA | | | intergenic |
| ENSOANG00000042148 | ENSOANG00000042148 | O_anatinus | Animals | mono_intronic | H/ACA | ENSOANG00000050093 | | protein_coding |

| | | | | | | | | |
|---|---|---|---|---|---|---|---|---|
| ENSOANG00000042149 | ENSOANG00000042149 | O_anatinus | Animals | mono_intergenic | H/ACA | | | intergenic |
| ENSOANG00000042150 | ENSOANG00000042150 | O_anatinus | Animals | mono_intronic | H/ACA | ENSOANG00000000925 | RABGAP1L | protein_coding |
| ENSOANG00000042151 | ENSOANG00000042151 | O_anatinus | Animals | mono_intronic | H/ACA | ENSOANG00000001835 | SLC30A7 | protein_coding |
| ENSOANG00000042153 | ENSOANG00000042153 | O_anatinus | Animals | mono_intergenic | H/ACA | | | intergenic |
| ENSOANG00000042154 | SNORA56 | O_anatinus | Animals | mono_intronic | H/ACA | ENSOANG00000008713 | DKC1 | protein_coding |
| ENSOANG00000042161 | ENSOANG00000042161 | O_anatinus | Animals | mono_intergenic | H/ACA | | | intergenic |
| ENSOANG00000042162 | ENSOANG00000042162 | O_anatinus | Animals | mono_intergenic | H/ACA | | | intergenic |
| ENSOANG00000042163 | ENSOANG00000042163 | O_anatinus | Animals | mono_intergenic | H/ACA | | | intergenic |
| ENSOANG00000042166 | ENSOANG00000042166 | O_anatinus | Animals | mono_intergenic | H/ACA | | | intergenic |
| ENSOANG00000042167 | ENSOANG00000042167 | O_anatinus | Animals | mono_intergenic | H/ACA | | | intergenic |
| ENSOANG00000042172 | ENSOANG00000042172 | O_anatinus | Animals | mono_intergenic | H/ACA | | | intergenic |
| ENSOANG00000042176 | ENSOANG00000042176 | O_anatinus | Animals | mono_intergenic | H/ACA | | | intergenic |
| ENSOANG00000042178 | ENSOANG00000042178 | O_anatinus | Animals | mono_intronic | H/ACA | ENSOANG00000004292 | PTBP3 | protein_coding |
| ENSOANG00000042182 | ENSOANG00000042182 | O_anatinus | Animals | mono_intergenic | H/ACA | | | intergenic |
| ENSOANG00000042184 | ENSOANG00000042184 | O_anatinus | Animals | mono_intergenic | H/ACA | | | intergenic |
| ENSOANG00000042190 | ENSOANG00000042190 | O_anatinus | Animals | mono_intergenic | H/ACA | | | intergenic |
| ENSOANG00000042196 | ENSOANG00000042196 | O_anatinus | Animals | mono_intergenic | H/ACA | | | intergenic |
| ENSOANG00000042197 | ENSOANG00000042197 | O_anatinus | Animals | mono_intergenic | H/ACA | | | intergenic |
| ENSOANG00000042199 | ENSOANG00000042199 | O_anatinus | Animals | mono_intergenic | H/ACA | | | intergenic |
| ENSOANG00000042201 | ENSOANG00000042201 | O_anatinus | Animals | mono_intergenic | H/ACA | | | intergenic |
| ENSOANG00000042202 | ENSOANG00000042202 | O_anatinus | Animals | mono_intronic | H/ACA | ENSOANG00000000848 | | protein_coding |
| ENSOANG00000042204 | ENSOANG00000042204 | O_anatinus | Animals | mono_intergenic | H/ACA | | | intergenic |
| ENSOANG00000042208 | ENSOANG00000042208 | O_anatinus | Animals | mono_intergenic | H/ACA | | | intergenic |
| ENSOANG00000042209 | ENSOANG00000042209 | O_anatinus | Animals | mono_intergenic | H/ACA | | | intergenic |
| ENSOANG00000042212 | ENSOANG00000042212 | O_anatinus | Animals | mono_intergenic | H/ACA | | | intergenic |
| ENSOANG00000042214 | ENSOANG00000042214 | O_anatinus | Animals | mono_intergenic | H/ACA | | | intergenic |
| ENSOANG00000042217 | ENSOANG00000042217 | O_anatinus | Animals | mono_intergenic | H/ACA | | | intergenic |
| ENSOANG00000042219 | ENSOANG00000042219 | O_anatinus | Animals | mono_intergenic | H/ACA | | | intergenic |
| ENSOANG00000042220 | ENSOANG00000042220 | O_anatinus | Animals | mono_intergenic | H/ACA | | | intergenic |
| ENSOANG00000042221 | ENSOANG00000042221 | O_anatinus | Animals | mono_intergenic | H/ACA | | | intergenic |
| ENSOANG00000042222 | ENSOANG00000042222 | O_anatinus | Animals | mono_intergenic | H/ACA | | | intergenic |
| ENSOANG00000042223 | ENSOANG00000042223 | O_anatinus | Animals | mono_intergenic | H/ACA | | | intergenic |
| ENSOANG00000042227 | ENSOANG00000042227 | O_anatinus | Animals | mono_intronic | H/ACA | ENSOANG00000010975 | SLC7A6 | protein_coding |
| ENSOANG00000042232 | ENSOANG00000042232 | O_anatinus | Animals | mono_intergenic | H/ACA | | | intergenic |
| ENSOANG00000042233 | ENSOANG00000042233 | O_anatinus | Animals | mono_intergenic | H/ACA | | | intergenic |
| ENSOANG00000042234 | ENSOANG00000042234 | O_anatinus | Animals | mono_intergenic | H/ACA | | | intergenic |
| ENSOANG00000042235 | ENSOANG00000042235 | O_anatinus | Animals | mono_intergenic | H/ACA | | | intergenic |
| ENSOANG00000042239 | ENSOANG00000042239 | O_anatinus | Animals | mono_intergenic | H/ACA | | | intergenic |
| ENSOANG00000042240 | ENSOANG00000042240 | O_anatinus | Animals | mono_intronic | H/ACA | ENSOANG00000042291 | NECAB1 | protein_coding |
| ENSOANG00000042241 | ENSOANG00000042241 | O_anatinus | Animals | mono_intergenic | H/ACA | | | intergenic |
| ENSOANG00000042247 | ENSOANG00000042247 | O_anatinus | Animals | mono_intergenic | H/ACA | | | intergenic |

| | | | | | | | | |
|---|---|---|---|---|---|---|---|---|
| ENSOANG00000042248 | ENSOANG00000042248 | O_anatinus | Animals | mono_intronic | H/ACA | ENSOANG00000047355 | | non_coding |
| ENSOANG00000042250 | ENSOANG00000042250 | O_anatinus | Animals | mono_intergenic | H/ACA | | | intergenic |
| ENSOANG00000042252 | ENSOANG00000042252 | O_anatinus | Animals | mono_intergenic | H/ACA | | | intergenic |
| ENSOANG00000042256 | ENSOANG00000042256 | O_anatinus | Animals | mono_intergenic | H/ACA | | | intergenic |
| ENSOANG00000042258 | ENSOANG00000042258 | O_anatinus | Animals | mono_intergenic | H/ACA | | | intergenic |
| ENSOANG00000042259 | ENSOANG00000042259 | O_anatinus | Animals | mono_intergenic | H/ACA | | | intergenic |
| ENSOANG00000042260 | ENSOANG00000042260 | O_anatinus | Animals | mono_intergenic | H/ACA | | | intergenic |
| ENSOANG00000042261 | ENSOANG00000042261 | O_anatinus | Animals | mono_intergenic | H/ACA | | | intergenic |
| ENSOANG00000042262 | ENSOANG00000042262 | O_anatinus | Animals | mono_intergenic | H/ACA | | | intergenic |
| ENSOANG00000042264 | ENSOANG00000042264 | O_anatinus | Animals | mono_intergenic | H/ACA | | | intergenic |
| ENSOANG00000042266 | ENSOANG00000042266 | O_anatinus | Animals | mono_intergenic | H/ACA | | | intergenic |
| ENSOANG00000042269 | ENSOANG00000042269 | O_anatinus | Animals | mono_intergenic | H/ACA | | | intergenic |
| ENSOANG00000042270 | ENSOANG00000042270 | O_anatinus | Animals | mono_intergenic | H/ACA | | | intergenic |
| ENSOANG00000042272 | ENSOANG00000042272 | O_anatinus | Animals | mono_intergenic | H/ACA | | | intergenic |
| ENSOANG00000042273 | ENSOANG00000042273 | O_anatinus | Animals | mono_intergenic | H/ACA | | | intergenic |
| ENSOANG00000042274 | ENSOANG00000042274 | O_anatinus | Animals | mono_intronic | H/ACA | ENSOANG00000003278 | USP42 | protein_coding |
| ENSOANG00000042278 | ENSOANG00000042278 | O_anatinus | Animals | mono_intergenic | H/ACA | | | intergenic |
| ENSOANG00000042279 | ENSOANG00000042279 | O_anatinus | Animals | mono_intergenic | H/ACA | | | intergenic |
| ENSOANG00000042281 | ENSOANG00000042281 | O_anatinus | Animals | mono_intergenic | H/ACA | | | intergenic |
| ENSOANG00000042283 | ENSOANG00000042283 | O_anatinus | Animals | mono_intronic | H/ACA | ENSOANG00000008795 | C1QTNF7 | protein_coding |
| ENSOANG00000042285 | ENSOANG00000042285 | O_anatinus | Animals | mono_intergenic | H/ACA | | | intergenic |
| ENSOANG00000042289 | ENSOANG00000042289 | O_anatinus | Animals | mono_intergenic | H/ACA | | | intergenic |
| ENSOANG00000042290 | ENSOANG00000042290 | O_anatinus | Animals | mono_intergenic | H/ACA | | | intergenic |
| ENSOANG00000042293 | ENSOANG00000042293 | O_anatinus | Animals | mono_intronic | H/ACA | ENSOANG00000012860 | BNC2 | protein_coding |
| ENSOANG00000042294 | ENSOANG00000042294 | O_anatinus | Animals | mono_intergenic | H/ACA | | | intergenic |
| ENSOANG00000042296 | ENSOANG00000042296 | O_anatinus | Animals | mono_intergenic | H/ACA | | | intergenic |
| ENSOANG00000042301 | ENSOANG00000042301 | O_anatinus | Animals | mono_intergenic | H/ACA | | | intergenic |
| ENSOANG00000042303 | ENSOANG00000042303 | O_anatinus | Animals | mono_intronic | H/ACA | ENSOANG00000011220 | | protein_coding |
| ENSOANG00000042304 | ENSOANG00000042304 | O_anatinus | Animals | mono_intergenic | H/ACA | | | intergenic |
| ENSOANG00000042308 | ENSOANG00000042308 | O_anatinus | Animals | mono_intergenic | H/ACA | | | intergenic |
| ENSOANG00000042310 | ENSOANG00000042310 | O_anatinus | Animals | mono_intergenic | H/ACA | | | intergenic |
| ENSOANG00000042313 | ENSOANG00000042313 | O_anatinus | Animals | mono_intergenic | H/ACA | | | intergenic |
| ENSOANG00000042314 | ENSOANG00000042314 | O_anatinus | Animals | mono_intergenic | H/ACA | | | intergenic |
| ENSOANG00000042316 | ENSOANG00000042316 | O_anatinus | Animals | mono_intergenic | H/ACA | | | intergenic |
| ENSOANG00000042317 | ENSOANG00000042317 | O_anatinus | Animals | mono_intergenic | H/ACA | | | intergenic |
| ENSOANG00000042319 | ENSOANG00000042319 | O_anatinus | Animals | mono_intergenic | H/ACA | | | intergenic |
| ENSOANG00000042321 | ENSOANG00000042321 | O_anatinus | Animals | mono_intergenic | H/ACA | | | intergenic |
| ENSOANG00000042322 | ENSOANG00000042322 | O_anatinus | Animals | mono_intergenic | H/ACA | | | intergenic |
| ENSOANG00000042323 | ENSOANG00000042323 | O_anatinus | Animals | mono_intergenic | H/ACA | | | intergenic |
| ENSOANG00000042324 | ENSOANG00000042324 | O_anatinus | Animals | mono_intergenic | H/ACA | | | intergenic |
| ENSOANG00000042327 | ENSOANG00000042327 | O_anatinus | Animals | mono_intergenic | H/ACA | | | intergenic |

| | | | | | | | | |
|---|---|---|---|---|---|---|---|---|
| ENSOANG00000042331 | ENSOANG00000042331 | O_anatinus | Animals | mono_intergenic | H/ACA | | | intergenic |
| ENSOANG00000042334 | ENSOANG00000042334 | O_anatinus | Animals | mono_intergenic | H/ACA | | | intergenic |
| ENSOANG00000042336 | ENSOANG00000042336 | O_anatinus | Animals | mono_intergenic | H/ACA | | | intergenic |
| ENSOANG00000042339 | ENSOANG00000042339 | O_anatinus | Animals | mono_intronic | H/ACA | ENSOANG00000012965 | INO80D | protein_coding |
| ENSOANG00000042342 | ENSOANG00000042342 | O_anatinus | Animals | mono_intergenic | H/ACA | | | intergenic |
| ENSOANG00000042343 | ENSOANG00000042343 | O_anatinus | Animals | mono_intergenic | H/ACA | | | intergenic |
| ENSOANG00000042351 | ENSOANG00000042351 | O_anatinus | Animals | mono_intronic | H/ACA | ENSOANG00000015335 | CHRDL2 | protein_coding |
| ENSOANG00000042352 | ENSOANG00000042352 | O_anatinus | Animals | mono_intronic | H/ACA | ENSOANG00000006708 | TIAM2 | protein_coding |
| ENSOANG00000042355 | ENSOANG00000042355 | O_anatinus | Animals | mono_intergenic | H/ACA | | | intergenic |
| ENSOANG00000042356 | ENSOANG00000042356 | O_anatinus | Animals | mono_intergenic | H/ACA | | | intergenic |
| ENSOANG00000042360 | ENSOANG00000042360 | O_anatinus | Animals | mono_intergenic | H/ACA | | | intergenic |
| ENSOANG00000042363 | ENSOANG00000042363 | O_anatinus | Animals | mono_intergenic | H/ACA | | | intergenic |
| ENSOANG00000042364 | ENSOANG00000042364 | O_anatinus | Animals | mono_intergenic | H/ACA | | | intergenic |
| ENSOANG00000042372 | ENSOANG00000042372 | O_anatinus | Animals | mono_intergenic | H/ACA | | | intergenic |
| ENSOANG00000042380 | ENSOANG00000042380 | O_anatinus | Animals | mono_intergenic | H/ACA | | | intergenic |
| ENSOANG00000042381 | ENSOANG00000042381 | O_anatinus | Animals | mono_intergenic | H/ACA | | | intergenic |
| ENSOANG00000042382 | ENSOANG00000042382 | O_anatinus | Animals | mono_intergenic | H/ACA | | | intergenic |
| ENSOANG00000042389 | ENSOANG00000042389 | O_anatinus | Animals | mono_intergenic | H/ACA | | | intergenic |
| ENSOANG00000042391 | ENSOANG00000042391 | O_anatinus | Animals | mono_intergenic | H/ACA | | | intergenic |
| ENSOANG00000042394 | ENSOANG00000042394 | O_anatinus | Animals | mono_intronic | H/ACA | ENSOANG00000029206 | HS3ST2 | protein_coding |
| ENSOANG00000042395 | ENSOANG00000042395 | O_anatinus | Animals | mono_intergenic | H/ACA | | | intergenic |
| ENSOANG00000042396 | ENSOANG00000042396 | O_anatinus | Animals | mono_intergenic | H/ACA | | | intergenic |
| ENSOANG00000042397 | ENSOANG00000042397 | O_anatinus | Animals | mono_intergenic | H/ACA | | | intergenic |
| ENSOANG00000042399 | ENSOANG00000042399 | O_anatinus | Animals | mono_intergenic | H/ACA | | | intergenic |
| ENSOANG00000042401 | ENSOANG00000042401 | O_anatinus | Animals | mono_intergenic | H/ACA | | | intergenic |
| ENSOANG00000042405 | ENSOANG00000042405 | O_anatinus | Animals | mono_intergenic | H/ACA | | | intergenic |
| ENSOANG00000042407 | ENSOANG00000042407 | O_anatinus | Animals | mono_intergenic | H/ACA | | | intergenic |
| ENSOANG00000042408 | ENSOANG00000042408 | O_anatinus | Animals | mono_intergenic | H/ACA | | | intergenic |
| ENSOANG00000042412 | ENSOANG00000042412 | O_anatinus | Animals | mono_intergenic | H/ACA | | | intergenic |
| ENSOANG00000042414 | ENSOANG00000042414 | O_anatinus | Animals | mono_intergenic | H/ACA | | | intergenic |
| ENSOANG00000042415 | ENSOANG00000042415 | O_anatinus | Animals | mono_intergenic | H/ACA | | | intergenic |
| ENSOANG00000042417 | ENSOANG00000042417 | O_anatinus | Animals | mono_intergenic | H/ACA | | | intergenic |
| ENSOANG00000042418 | ENSOANG00000042418 | O_anatinus | Animals | mono_intergenic | H/ACA | | | intergenic |
| ENSOANG00000042422 | ENSOANG00000042422 | O_anatinus | Animals | mono_intergenic | H/ACA | | | intergenic |
| ENSOANG00000042425 | ENSOANG00000042425 | O_anatinus | Animals | mono_intergenic | H/ACA | | | intergenic |
| ENSOANG00000042427 | ENSOANG00000042427 | O_anatinus | Animals | mono_intergenic | H/ACA | | | intergenic |
| ENSOANG00000042430 | ENSOANG00000042430 | O_anatinus | Animals | intronic_cluster | H/ACA | ENSOANG00000012629 | | protein_coding |
| ENSOANG00000042433 | ENSOANG00000042433 | O_anatinus | Animals | mono_intergenic | H/ACA | | | intergenic |
| ENSOANG00000042434 | ENSOANG00000042434 | O_anatinus | Animals | mono_intergenic | H/ACA | | | intergenic |
| ENSOANG00000042435 | ENSOANG00000042435 | O_anatinus | Animals | mono_intergenic | H/ACA | | | intergenic |
| ENSOANG00000042436 | ENSOANG00000042436 | O_anatinus | Animals | mono_intergenic | H/ACA | | | intergenic |

| | | | | | | | | |
|---|---|---|---|---|---|---|---|---|
| ENSOANG00000042438 | ENSOANG00000042438 | O_anatinus | Animals | mono_intergenic | H/ACA | | | intergenic |
| ENSOANG00000042443 | ENSOANG00000042443 | O_anatinus | Animals | mono_intergenic | H/ACA | | | intergenic |
| ENSOANG00000042445 | ENSOANG00000042445 | O_anatinus | Animals | mono_intergenic | H/ACA | | | intergenic |
| ENSOANG00000042452 | ENSOANG00000042452 | O_anatinus | Animals | mono_intronic | H/ACA | ENSOANG00000002340 | | protein_coding |
| ENSOANG00000042453 | ENSOANG00000042453 | O_anatinus | Animals | mono_intergenic | H/ACA | | | intergenic |
| ENSOANG00000042454 | ENSOANG00000042454 | O_anatinus | Animals | mono_intergenic | H/ACA | | | intergenic |
| ENSOANG00000042455 | ENSOANG00000042455 | O_anatinus | Animals | mono_intronic | H/ACA | ENSOANG00000005925 | GRIP1 | protein_coding |
| ENSOANG00000042460 | ENSOANG00000042460 | O_anatinus | Animals | mono_intergenic | H/ACA | | | intergenic |
| ENSOANG00000042462 | ENSOANG00000042462 | O_anatinus | Animals | mono_intronic | H/ACA | ENSOANG00000037529 | FGF18 | protein_coding |
| ENSOANG00000042465 | ENSOANG00000042465 | O_anatinus | Animals | mono_intergenic | H/ACA | | | intergenic |
| ENSOANG00000042466 | ENSOANG00000042466 | O_anatinus | Animals | mono_intergenic | H/ACA | | | intergenic |
| ENSOANG00000042468 | SNORA63 | O_anatinus | Animals | mono_intronic | H/ACA | ENSOANG00000042142 | EIF4A2 | protein_coding |
| ENSOANG00000042472 | ENSOANG00000042472 | O_anatinus | Animals | mono_intergenic | H/ACA | | | intergenic |
| ENSOANG00000042474 | ENSOANG00000042474 | O_anatinus | Animals | mono_intergenic | H/ACA | | | intergenic |
| ENSOANG00000042475 | ENSOANG00000042475 | O_anatinus | Animals | mono_intergenic | H/ACA | | | intergenic |
| ENSOANG00000042476 | ENSOANG00000042476 | O_anatinus | Animals | mono_intergenic | H/ACA | | | intergenic |
| ENSOANG00000042477 | SNORD73 | O_anatinus | Animals | mono_intronic | C/D | ENSOANG00000039121 | | protein_coding |
| ENSOANG00000042480 | ENSOANG00000042480 | O_anatinus | Animals | mono_intergenic | H/ACA | | | intergenic |
| ENSOANG00000042484 | ENSOANG00000042484 | O_anatinus | Animals | mono_intergenic | H/ACA | | | intergenic |
| ENSOANG00000042485 | ENSOANG00000042485 | O_anatinus | Animals | mono_intergenic | H/ACA | | | intergenic |
| ENSOANG00000042487 | ENSOANG00000042487 | O_anatinus | Animals | mono_intergenic | H/ACA | | | intergenic |
| ENSOANG00000042490 | ENSOANG00000042490 | O_anatinus | Animals | mono_intergenic | H/ACA | | | intergenic |
| ENSOANG00000042491 | ENSOANG00000042491 | O_anatinus | Animals | mono_intronic | H/ACA | ENSOANG00000015557 | SYNE1 | protein_coding |
| ENSOANG00000042493 | ENSOANG00000042493 | O_anatinus | Animals | mono_intergenic | H/ACA | | | intergenic |
| ENSOANG00000042494 | ENSOANG00000042494 | O_anatinus | Animals | mono_intergenic | H/ACA | | | intergenic |
| ENSOANG00000042500 | ENSOANG00000042500 | O_anatinus | Animals | mono_intergenic | H/ACA | | | intergenic |
| ENSOANG00000042502 | ENSOANG00000042502 | O_anatinus | Animals | mono_intergenic | H/ACA | | | intergenic |
| ENSOANG00000042504 | ENSOANG00000042504 | O_anatinus | Animals | mono_intergenic | H/ACA | | | intergenic |
| ENSOANG00000042507 | ENSOANG00000042507 | O_anatinus | Animals | mono_intronic | H/ACA | ENSOANG00000005603 | GLRA3 | protein_coding |
| ENSOANG00000042509 | ENSOANG00000042509 | O_anatinus | Animals | mono_intergenic | H/ACA | | | intergenic |
| ENSOANG00000042511 | ENSOANG00000042511 | O_anatinus | Animals | mono_intergenic | H/ACA | | | intergenic |
| ENSOANG00000042513 | ENSOANG00000042513 | O_anatinus | Animals | mono_intergenic | H/ACA | | | intergenic |
| ENSOANG00000042521 | ENSOANG00000042521 | O_anatinus | Animals | mono_intergenic | H/ACA | | | intergenic |
| ENSOANG00000042526 | ENSOANG00000042526 | O_anatinus | Animals | mono_intronic | H/ACA | ENSOANG00000020004 | ANKS1B | protein_coding |
| ENSOANG00000042527 | ENSOANG00000042527 | O_anatinus | Animals | mono_intergenic | H/ACA | | | intergenic |
| ENSOANG00000042528 | ENSOANG00000042528 | O_anatinus | Animals | mono_intergenic | H/ACA | | | intergenic |
| ENSOANG00000042531 | ENSOANG00000042531 | O_anatinus | Animals | intronic_cluster | H/ACA | ENSOANG00000021517 | AK5 | protein_coding |
| ENSOANG00000042532 | ENSOANG00000042532 | O_anatinus | Animals | mono_intergenic | H/ACA | | | intergenic |
| ENSOANG00000042537 | ENSOANG00000042537 | O_anatinus | Animals | mono_intergenic | H/ACA | | | intergenic |
| ENSOANG00000042542 | ENSOANG00000042542 | O_anatinus | Animals | mono_intergenic | H/ACA | | | intergenic |
| ENSOANG00000042544 | ENSOANG00000042544 | O_anatinus | Animals | mono_intergenic | H/ACA | | | intergenic |

| | | | | | | | | |
|---|---|---|---|---|---|---|---|---|
| ENSOANG00000042545 | ENSOANG00000042545 | O_anatinus | Animals | mono_intergenic | H/ACA | | | intergenic |
| ENSOANG00000042549 | ENSOANG00000042549 | O_anatinus | Animals | mono_intergenic | H/ACA | | | intergenic |
| ENSOANG00000042551 | ENSOANG00000042551 | O_anatinus | Animals | mono_intergenic | H/ACA | | | intergenic |
| ENSOANG00000042552 | ENSOANG00000042552 | O_anatinus | Animals | mono_intergenic | H/ACA | | | intergenic |
| ENSOANG00000042554 | ENSOANG00000042554 | O_anatinus | Animals | intronic_cluster | H/ACA | ENSOANG00000014155 | USP54 | protein_coding |
| ENSOANG00000042558 | ENSOANG00000042558 | O_anatinus | Animals | mono_intronic | H/ACA | ENSOANG00000012096 | | protein_coding |
| ENSOANG00000042559 | ENSOANG00000042559 | O_anatinus | Animals | mono_intergenic | H/ACA | | | intergenic |
| ENSOANG00000042561 | ENSOANG00000042561 | O_anatinus | Animals | mono_intergenic | H/ACA | | | intergenic |
| ENSOANG00000042562 | ENSOANG00000042562 | O_anatinus | Animals | mono_intergenic | H/ACA | | | intergenic |
| ENSOANG00000042563 | ENSOANG00000042563 | O_anatinus | Animals | mono_intergenic | H/ACA | | | intergenic |
| ENSOANG00000042566 | ENSOANG00000042566 | O_anatinus | Animals | mono_intronic | C/D | ENSOANG00000014467 | WDR43 | protein_coding |
| ENSOANG00000042567 | ENSOANG00000042567 | O_anatinus | Animals | mono_intergenic | H/ACA | | | intergenic |
| ENSOANG00000042569 | ENSOANG00000042569 | O_anatinus | Animals | mono_intergenic | H/ACA | | | intergenic |
| ENSOANG00000042570 | ENSOANG00000042570 | O_anatinus | Animals | mono_intergenic | H/ACA | | | intergenic |
| ENSOANG00000042574 | ENSOANG00000042574 | O_anatinus | Animals | mono_intergenic | H/ACA | | | intergenic |
| ENSOANG00000042575 | ENSOANG00000042575 | O_anatinus | Animals | mono_intergenic | H/ACA | | | intergenic |
| ENSOANG00000042576 | ENSOANG00000042576 | O_anatinus | Animals | mono_intergenic | H/ACA | | | intergenic |
| ENSOANG00000042578 | ENSOANG00000042578 | O_anatinus | Animals | mono_intergenic | H/ACA | | | intergenic |
| ENSOANG00000042579 | ENSOANG00000042579 | O_anatinus | Animals | mono_intergenic | H/ACA | | | intergenic |
| ENSOANG00000042580 | ENSOANG00000042580 | O_anatinus | Animals | mono_intergenic | H/ACA | | | intergenic |
| ENSOANG00000042581 | ENSOANG00000042581 | O_anatinus | Animals | mono_intergenic | H/ACA | | | intergenic |
| ENSOANG00000042582 | ENSOANG00000042582 | O_anatinus | Animals | mono_intergenic | H/ACA | | | intergenic |
| ENSOANG00000042583 | ENSOANG00000042583 | O_anatinus | Animals | mono_intergenic | H/ACA | | | intergenic |
| ENSOANG00000042584 | ENSOANG00000042584 | O_anatinus | Animals | mono_intronic | H/ACA | ENSOANG00000021418 | DOCK2 | protein_coding |
| ENSOANG00000042586 | ENSOANG00000042586 | O_anatinus | Animals | mono_intergenic | H/ACA | | | intergenic |
| ENSOANG00000042587 | ENSOANG00000042587 | O_anatinus | Animals | mono_intergenic | H/ACA | | | intergenic |
| ENSOANG00000042593 | ENSOANG00000042593 | O_anatinus | Animals | mono_intergenic | H/ACA | | | intergenic |
| ENSOANG00000042597 | ENSOANG00000042597 | O_anatinus | Animals | mono_intergenic | H/ACA | | | intergenic |
| ENSOANG00000042599 | ENSOANG00000042599 | O_anatinus | Animals | mono_intronic | H/ACA | ENSOANG00000005515 | LARGE1 | protein_coding |
| ENSOANG00000042601 | ENSOANG00000042601 | O_anatinus | Animals | mono_intergenic | H/ACA | | | intergenic |
| ENSOANG00000042605 | ENSOANG00000042605 | O_anatinus | Animals | mono_intergenic | H/ACA | | | intergenic |
| ENSOANG00000042606 | ENSOANG00000042606 | O_anatinus | Animals | mono_intergenic | H/ACA | | | intergenic |
| ENSOANG00000042610 | ENSOANG00000042610 | O_anatinus | Animals | mono_intergenic | H/ACA | | | intergenic |
| ENSOANG00000042615 | ENSOANG00000042615 | O_anatinus | Animals | mono_intergenic | H/ACA | | | intergenic |
| ENSOANG00000042619 | ENSOANG00000042619 | O_anatinus | Animals | mono_intergenic | H/ACA | | | intergenic |
| ENSOANG00000042621 | ENSOANG00000042621 | O_anatinus | Animals | mono_intergenic | H/ACA | | | intergenic |
| ENSOANG00000042625 | ENSOANG00000042625 | O_anatinus | Animals | mono_intergenic | H/ACA | | | intergenic |
| ENSOANG00000042628 | ENSOANG00000042628 | O_anatinus | Animals | mono_intergenic | H/ACA | | | intergenic |
| ENSOANG00000042631 | ENSOANG00000042631 | O_anatinus | Animals | mono_intergenic | H/ACA | | | intergenic |
| ENSOANG00000042632 | ENSOANG00000042632 | O_anatinus | Animals | mono_intergenic | H/ACA | | | intergenic |
| ENSOANG00000042633 | ENSOANG00000042633 | O_anatinus | Animals | mono_intergenic | H/ACA | | | intergenic |

| | | | | | | | | |
|---|---|---|---|---|---|---|---|---|
| ENSOANG00000042634 | ENSOANG00000042634 | O_anatinus | Animals | mono_intergenic | H/ACA | | | intergenic |
| ENSOANG00000042635 | ENSOANG00000042635 | O_anatinus | Animals | mono_intergenic | H/ACA | | | intergenic |
| ENSOANG00000042637 | ENSOANG00000042637 | O_anatinus | Animals | mono_intergenic | H/ACA | | | intergenic |
| ENSOANG00000042639 | ENSOANG00000042639 | O_anatinus | Animals | mono_intergenic | H/ACA | | | intergenic |
| ENSOANG00000042640 | ENSOANG00000042640 | O_anatinus | Animals | mono_intergenic | H/ACA | | | intergenic |
| ENSOANG00000042642 | ENSOANG00000042642 | O_anatinus | Animals | mono_intergenic | H/ACA | | | intergenic |
| ENSOANG00000042647 | ENSOANG00000042647 | O_anatinus | Animals | mono_intergenic | H/ACA | | | intergenic |
| ENSOANG00000042648 | ENSOANG00000042648 | O_anatinus | Animals | mono_intergenic | H/ACA | | | intergenic |
| ENSOANG00000042654 | ENSOANG00000042654 | O_anatinus | Animals | mono_intergenic | H/ACA | | | intergenic |
| ENSOANG00000042655 | ENSOANG00000042655 | O_anatinus | Animals | mono_intronic | H/ACA | ENSOANG00000009947 | TMEM132C | protein_coding |
| ENSOANG00000042656 | ENSOANG00000042656 | O_anatinus | Animals | mono_intergenic | H/ACA | | | intergenic |
| ENSOANG00000042660 | ENSOANG00000042660 | O_anatinus | Animals | mono_intergenic | H/ACA | | | intergenic |
| ENSOANG00000042661 | ENSOANG00000042661 | O_anatinus | Animals | mono_intergenic | H/ACA | | | intergenic |
| ENSOANG00000042662 | ENSOANG00000042662 | O_anatinus | Animals | mono_intergenic | H/ACA | | | intergenic |
| ENSOANG00000042665 | ENSOANG00000042665 | O_anatinus | Animals | mono_intronic | H/ACA | ENSOANG00000036834 | DMRT1 | protein_coding |
| ENSOANG00000042667 | ENSOANG00000042667 | O_anatinus | Animals | mono_intergenic | H/ACA | | | intergenic |
| ENSOANG00000042672 | ENSOANG00000042672 | O_anatinus | Animals | mono_intronic | H/ACA | ENSOANG00000006064 | PARD3 | protein_coding |
| ENSOANG00000042675 | ENSOANG00000042675 | O_anatinus | Animals | mono_intergenic | H/ACA | | | intergenic |
| ENSOANG00000042680 | ENSOANG00000042680 | O_anatinus | Animals | mono_intergenic | H/ACA | | | intergenic |
| ENSOANG00000042682 | ENSOANG00000042682 | O_anatinus | Animals | mono_intergenic | H/ACA | | | intergenic |
| ENSOANG00000042684 | ENSOANG00000042684 | O_anatinus | Animals | mono_intergenic | H/ACA | | | intergenic |
| ENSOANG00000042688 | ENSOANG00000042688 | O_anatinus | Animals | mono_intergenic | H/ACA | | | intergenic |
| ENSOANG00000042690 | ENSOANG00000042690 | O_anatinus | Animals | mono_intergenic | H/ACA | | | intergenic |
| ENSOANG00000042691 | ENSOANG00000042691 | O_anatinus | Animals | mono_intergenic | H/ACA | | | intergenic |
| ENSOANG00000042692 | ENSOANG00000042692 | O_anatinus | Animals | mono_intergenic | H/ACA | | | intergenic |
| ENSOANG00000042696 | ENSOANG00000042696 | O_anatinus | Animals | mono_intergenic | H/ACA | | | intergenic |
| ENSOANG00000042698 | ENSOANG00000042698 | O_anatinus | Animals | mono_intronic | H/ACA | ENSOANG00000007531 | SYT1 | protein_coding |
| ENSOANG00000042700 | ENSOANG00000042700 | O_anatinus | Animals | mono_intergenic | H/ACA | | | intergenic |
| ENSOANG00000042701 | ENSOANG00000042701 | O_anatinus | Animals | mono_intergenic | H/ACA | | | intergenic |
| ENSOANG00000042702 | ENSOANG00000042702 | O_anatinus | Animals | mono_intergenic | H/ACA | | | intergenic |
| ENSOANG00000042704 | ENSOANG00000042704 | O_anatinus | Animals | mono_intergenic | H/ACA | | | intergenic |
| ENSOANG00000042705 | ENSOANG00000042705 | O_anatinus | Animals | mono_intergenic | H/ACA | | | intergenic |
| ENSOANG00000042708 | ENSOANG00000042708 | O_anatinus | Animals | mono_intergenic | H/ACA | | | intergenic |
| ENSOANG00000042709 | ENSOANG00000042709 | O_anatinus | Animals | mono_intergenic | H/ACA | | | intergenic |
| ENSOANG00000042711 | ENSOANG00000042711 | O_anatinus | Animals | mono_intergenic | H/ACA | | | intergenic |
| ENSOANG00000042712 | ENSOANG00000042712 | O_anatinus | Animals | mono_intergenic | H/ACA | | | intergenic |
| ENSOANG00000042713 | ENSOANG00000042713 | O_anatinus | Animals | mono_intergenic | H/ACA | | | intergenic |
| ENSOANG00000042715 | ENSOANG00000042715 | O_anatinus | Animals | mono_intergenic | H/ACA | | | intergenic |
| ENSOANG00000042716 | ENSOANG00000042716 | O_anatinus | Animals | mono_intergenic | H/ACA | | | intergenic |
| ENSOANG00000042717 | ENSOANG00000042717 | O_anatinus | Animals | mono_intergenic | H/ACA | | | intergenic |
| ENSOANG00000042718 | ENSOANG00000042718 | O_anatinus | Animals | mono_intergenic | H/ACA | | | intergenic |

| | | | | | | | | |
|---|---|---|---|---|---|---|---|---|
| ENSOANG00000042723 | ENSOANG00000042723 | O_anatinus | Animals | mono_intergenic | H/ACA | | | intergenic |
| ENSOANG00000042728 | ENSOANG00000042728 | O_anatinus | Animals | mono_intergenic | H/ACA | | | intergenic |
| ENSOANG00000042729 | ENSOANG00000042729 | O_anatinus | Animals | intronic_cluster | H/ACA | ENSOANG00000015522 | LRRC7 | protein_coding |
| ENSOANG00000042730 | ENSOANG00000042730 | O_anatinus | Animals | mono_intergenic | H/ACA | | | intergenic |
| ENSOANG00000042733 | ENSOANG00000042733 | O_anatinus | Animals | mono_intergenic | H/ACA | | | intergenic |
| ENSOANG00000042736 | ENSOANG00000042736 | O_anatinus | Animals | mono_intergenic | H/ACA | | | intergenic |
| ENSOANG00000042740 | ENSOANG00000042740 | O_anatinus | Animals | mono_intergenic | H/ACA | | | intergenic |
| ENSOANG00000042747 | ENSOANG00000042747 | O_anatinus | Animals | mono_intergenic | H/ACA | | | intergenic |
| ENSOANG00000042750 | ENSOANG00000042750 | O_anatinus | Animals | mono_intronic | H/ACA | ENSOANG00000001738 | TMEM117 | protein_coding |
| ENSOANG00000042761 | ENSOANG00000042761 | O_anatinus | Animals | mono_intergenic | H/ACA | | | intergenic |
| ENSOANG00000042762 | ENSOANG00000042762 | O_anatinus | Animals | mono_intergenic | H/ACA | | | intergenic |
| ENSOANG00000042765 | ENSOANG00000042765 | O_anatinus | Animals | mono_intergenic | H/ACA | | | intergenic |
| ENSOANG00000042766 | ENSOANG00000042766 | O_anatinus | Animals | mono_intergenic | H/ACA | | | intergenic |
| ENSOANG00000042770 | ENSOANG00000042770 | O_anatinus | Animals | mono_intergenic | H/ACA | | | intergenic |
| ENSOANG00000042772 | ENSOANG00000042772 | O_anatinus | Animals | mono_intergenic | H/ACA | | | intergenic |
| ENSOANG00000042774 | ENSOANG00000042774 | O_anatinus | Animals | mono_intergenic | H/ACA | | | intergenic |
| ENSOANG00000042775 | ENSOANG00000042775 | O_anatinus | Animals | mono_intronic | H/ACA | ENSOANG00000003627 | PDE1A | protein_coding |
| ENSOANG00000042778 | ENSOANG00000042778 | O_anatinus | Animals | mono_intergenic | H/ACA | | | intergenic |
| ENSOANG00000042779 | ENSOANG00000042779 | O_anatinus | Animals | intergenic_cluster | H/ACA | | | intergenic |
| ENSOANG00000042781 | ENSOANG00000042781 | O_anatinus | Animals | mono_intergenic | H/ACA | | | intergenic |
| ENSOANG00000042782 | ENSOANG00000042782 | O_anatinus | Animals | mono_intergenic | H/ACA | | | intergenic |
| ENSOANG00000042783 | ENSOANG00000042783 | O_anatinus | Animals | mono_intergenic | H/ACA | | | intergenic |
| ENSOANG00000042784 | ENSOANG00000042784 | O_anatinus | Animals | mono_intergenic | H/ACA | | | intergenic |
| ENSOANG00000042785 | ENSOANG00000042785 | O_anatinus | Animals | mono_intergenic | H/ACA | | | intergenic |
| ENSOANG00000042787 | ENSOANG00000042787 | O_anatinus | Animals | mono_intergenic | H/ACA | | | intergenic |
| ENSOANG00000042788 | ENSOANG00000042788 | O_anatinus | Animals | mono_intergenic | H/ACA | | | intergenic |
| ENSOANG00000042791 | ENSOANG00000042791 | O_anatinus | Animals | mono_intergenic | H/ACA | | | intergenic |
| ENSOANG00000042800 | ENSOANG00000042800 | O_anatinus | Animals | mono_intergenic | H/ACA | | | intergenic |
| ENSOANG00000042802 | ENSOANG00000042802 | O_anatinus | Animals | intronic_cluster | H/ACA | ENSOANG00000003052 | KCNC1 | protein_coding |
| ENSOANG00000042803 | ENSOANG00000042803 | O_anatinus | Animals | mono_intergenic | H/ACA | | | intergenic |
| ENSOANG00000042806 | ENSOANG00000042806 | O_anatinus | Animals | mono_intergenic | H/ACA | | | intergenic |
| ENSOANG00000042813 | ENSOANG00000042813 | O_anatinus | Animals | mono_intergenic | H/ACA | | | intergenic |
| ENSOANG00000042814 | ENSOANG00000042814 | O_anatinus | Animals | mono_intergenic | H/ACA | | | intergenic |
| ENSOANG00000042817 | ENSOANG00000042817 | O_anatinus | Animals | mono_intergenic | H/ACA | | | intergenic |
| ENSOANG00000042818 | ENSOANG00000042818 | O_anatinus | Animals | mono_intergenic | H/ACA | | | intergenic |
| ENSOANG00000042821 | ENSOANG00000042821 | O_anatinus | Animals | mono_intergenic | H/ACA | | | intergenic |
| ENSOANG00000042823 | ENSOANG00000042823 | O_anatinus | Animals | mono_intergenic | H/ACA | | | intergenic |
| ENSOANG00000042827 | ENSOANG00000042827 | O_anatinus | Animals | mono_intergenic | H/ACA | | | intergenic |
| ENSOANG00000042828 | ENSOANG00000042828 | O_anatinus | Animals | mono_intronic | H/ACA | ENSOANG00000005178 | ZMIZ1 | protein_coding |
| ENSOANG00000042829 | ENSOANG00000042829 | O_anatinus | Animals | mono_intronic | C/D | ENSOANG00000008205 | | protein_coding |
| ENSOANG00000042833 | ENSOANG00000042833 | O_anatinus | Animals | mono_intergenic | H/ACA | | | intergenic |

| | | | | | | | | |
|---|---|---|---|---|---|---|---|---|
| ENSOANG00000042836 | ENSOANG00000042836 | O_anatinus | Animals | mono_intergenic | H/ACA | | | intergenic |
| ENSOANG00000042837 | ENSOANG00000042837 | O_anatinus | Animals | mono_intergenic | H/ACA | | | intergenic |
| ENSOANG00000042838 | ENSOANG00000042838 | O_anatinus | Animals | mono_intergenic | H/ACA | | | intergenic |
| ENSOANG00000042839 | ENSOANG00000042839 | O_anatinus | Animals | mono_intergenic | H/ACA | | | intergenic |
| ENSOANG00000042841 | ENSOANG00000042841 | O_anatinus | Animals | mono_intergenic | H/ACA | | | intergenic |
| ENSOANG00000042843 | ENSOANG00000042843 | O_anatinus | Animals | mono_intergenic | H/ACA | | | intergenic |
| ENSOANG00000042844 | ENSOANG00000042844 | O_anatinus | Animals | mono_intergenic | H/ACA | | | intergenic |
| ENSOANG00000042845 | ENSOANG00000042845 | O_anatinus | Animals | mono_intergenic | H/ACA | | | intergenic |
| ENSOANG00000042847 | ENSOANG00000042847 | O_anatinus | Animals | mono_intergenic | H/ACA | | | intergenic |
| ENSOANG00000042848 | ENSOANG00000042848 | O_anatinus | Animals | mono_intergenic | H/ACA | | | intergenic |
| ENSOANG00000042850 | ENSOANG00000042850 | O_anatinus | Animals | mono_intergenic | H/ACA | | | intergenic |
| ENSOANG00000042852 | ENSOANG00000042852 | O_anatinus | Animals | mono_intergenic | H/ACA | | | intergenic |
| ENSOANG00000042854 | ENSOANG00000042854 | O_anatinus | Animals | intergenic_cluster | C/D | | | intergenic |
| ENSOANG00000042857 | ENSOANG00000042857 | O_anatinus | Animals | mono_intergenic | H/ACA | | | intergenic |
| ENSOANG00000042858 | ENSOANG00000042858 | O_anatinus | Animals | mono_intergenic | H/ACA | | | intergenic |
| ENSOANG00000042860 | ENSOANG00000042860 | O_anatinus | Animals | mono_intergenic | H/ACA | | | intergenic |
| ENSOANG00000042861 | ENSOANG00000042861 | O_anatinus | Animals | mono_intergenic | H/ACA | | | intergenic |
| ENSOANG00000042864 | ENSOANG00000042864 | O_anatinus | Animals | mono_intergenic | H/ACA | | | intergenic |
| ENSOANG00000042872 | ENSOANG00000042872 | O_anatinus | Animals | mono_intergenic | H/ACA | | | intergenic |
| ENSOANG00000042874 | ENSOANG00000042874 | O_anatinus | Animals | mono_intergenic | H/ACA | | | intergenic |
| ENSOANG00000042875 | ENSOANG00000042875 | O_anatinus | Animals | mono_intergenic | H/ACA | | | intergenic |
| ENSOANG00000042876 | ENSOANG00000042876 | O_anatinus | Animals | mono_intergenic | H/ACA | | | intergenic |
| ENSOANG00000042879 | ENSOANG00000042879 | O_anatinus | Animals | mono_intronic | H/ACA | ENSOANG00000028698 | | protein_coding |
| ENSOANG00000042880 | ENSOANG00000042880 | O_anatinus | Animals | mono_intergenic | H/ACA | | | intergenic |
| ENSOANG00000042882 | ENSOANG00000042882 | O_anatinus | Animals | mono_intergenic | H/ACA | | | intergenic |
| ENSOANG00000042883 | ENSOANG00000042883 | O_anatinus | Animals | mono_intergenic | H/ACA | | | intergenic |
| ENSOANG00000042884 | ENSOANG00000042884 | O_anatinus | Animals | mono_intergenic | H/ACA | | | intergenic |
| ENSOANG00000042885 | ENSOANG00000042885 | O_anatinus | Animals | mono_intergenic | H/ACA | | | intergenic |
| ENSOANG00000042887 | ENSOANG00000042887 | O_anatinus | Animals | mono_intergenic | H/ACA | | | intergenic |
| ENSOANG00000042888 | ENSOANG00000042888 | O_anatinus | Animals | mono_intergenic | H/ACA | | | intergenic |
| ENSOANG00000042890 | ENSOANG00000042890 | O_anatinus | Animals | mono_intronic | H/ACA | ENSOANG00000043356 | PAWR | protein_coding |
| ENSOANG00000042892 | ENSOANG00000042892 | O_anatinus | Animals | mono_intergenic | H/ACA | | | intergenic |
| ENSOANG00000042893 | ENSOANG00000042893 | O_anatinus | Animals | mono_intergenic | H/ACA | | | intergenic |
| ENSOANG00000042897 | ENSOANG00000042897 | O_anatinus | Animals | mono_intergenic | H/ACA | | | intergenic |
| ENSOANG00000042899 | ENSOANG00000042899 | O_anatinus | Animals | mono_intergenic | H/ACA | | | intergenic |
| ENSOANG00000042900 | ENSOANG00000042900 | O_anatinus | Animals | mono_intergenic | H/ACA | | | intergenic |
| ENSOANG00000042902 | ENSOANG00000042902 | O_anatinus | Animals | mono_intergenic | H/ACA | | | intergenic |
| ENSOANG00000042905 | ENSOANG00000042905 | O_anatinus | Animals | mono_intergenic | H/ACA | | | intergenic |
| ENSOANG00000042906 | ENSOANG00000042906 | O_anatinus | Animals | mono_intergenic | H/ACA | | | intergenic |
| ENSOANG00000042910 | ENSOANG00000042910 | O_anatinus | Animals | mono_intergenic | H/ACA | | | intergenic |
| ENSOANG00000042917 | ENSOANG00000042917 | O_anatinus | Animals | mono_intergenic | H/ACA | | | intergenic |

| | | | | | | | | |
|---|---|---|---|---|---|---|---|---|
| ENSOANG00000042919 | ENSOANG00000042919 | O_anatinus | Animals | mono_intergenic | H/ACA | | | intergenic |
| ENSOANG00000042921 | ENSOANG00000042921 | O_anatinus | Animals | mono_intergenic | H/ACA | | | intergenic |
| ENSOANG00000042922 | ENSOANG00000042922 | O_anatinus | Animals | mono_intergenic | H/ACA | | | intergenic |
| ENSOANG00000042923 | ENSOANG00000042923 | O_anatinus | Animals | mono_intronic | H/ACA | ENSOANG00000013244 | | protein_coding |
| ENSOANG00000042926 | ENSOANG00000042926 | O_anatinus | Animals | mono_intergenic | H/ACA | | | intergenic |
| ENSOANG00000042927 | ENSOANG00000042927 | O_anatinus | Animals | mono_intergenic | H/ACA | | | intergenic |
| ENSOANG00000042929 | ENSOANG00000042929 | O_anatinus | Animals | mono_intergenic | H/ACA | | | intergenic |
| ENSOANG00000042930 | ENSOANG00000042930 | O_anatinus | Animals | mono_intronic | H/ACA | ENSOANG00000046492 | CFAP61 | protein_coding |
| ENSOANG00000042937 | ENSOANG00000042937 | O_anatinus | Animals | mono_intergenic | H/ACA | | | intergenic |
| ENSOANG00000042938 | ENSOANG00000042938 | O_anatinus | Animals | mono_intergenic | H/ACA | | | intergenic |
| ENSOANG00000042944 | ENSOANG00000042944 | O_anatinus | Animals | mono_intergenic | H/ACA | | | intergenic |
| ENSOANG00000042951 | ENSOANG00000042951 | O_anatinus | Animals | mono_intergenic | H/ACA | | | intergenic |
| ENSOANG00000042953 | ENSOANG00000042953 | O_anatinus | Animals | mono_intergenic | H/ACA | | | intergenic |
| ENSOANG00000042954 | ENSOANG00000042954 | O_anatinus | Animals | mono_intergenic | H/ACA | | | intergenic |
| ENSOANG00000042959 | ENSOANG00000042959 | O_anatinus | Animals | mono_intergenic | H/ACA | | | intergenic |
| ENSOANG00000042963 | ENSOANG00000042963 | O_anatinus | Animals | mono_intergenic | H/ACA | | | intergenic |
| ENSOANG00000042973 | ENSOANG00000042973 | O_anatinus | Animals | mono_intergenic | H/ACA | | | intergenic |
| ENSOANG00000042974 | ENSOANG00000042974 | O_anatinus | Animals | mono_intergenic | H/ACA | | | intergenic |
| ENSOANG00000042979 | ENSOANG00000042979 | O_anatinus | Animals | mono_intergenic | H/ACA | | | intergenic |
| ENSOANG00000042980 | ENSOANG00000042980 | O_anatinus | Animals | mono_intergenic | H/ACA | | | intergenic |
| ENSOANG00000042981 | ENSOANG00000042981 | O_anatinus | Animals | mono_intronic | H/ACA | ENSOANG00000001427 | SGPP2 | protein_coding |
| ENSOANG00000042984 | ENSOANG00000042984 | O_anatinus | Animals | mono_intergenic | H/ACA | | | intergenic |
| ENSOANG00000042987 | ENSOANG00000042987 | O_anatinus | Animals | mono_intronic | H/ACA | ENSOANG00000004193 | ZNF462 | protein_coding |
| ENSOANG00000042991 | ENSOANG00000042991 | O_anatinus | Animals | mono_intergenic | H/ACA | | | intergenic |
| ENSOANG00000042994 | ENSOANG00000042994 | O_anatinus | Animals | mono_intergenic | H/ACA | | | intergenic |
| ENSOANG00000042997 | ENSOANG00000042997 | O_anatinus | Animals | mono_intergenic | H/ACA | | | intergenic |
| ENSOANG00000043001 | ENSOANG00000043001 | O_anatinus | Animals | mono_intergenic | H/ACA | | | intergenic |
| ENSOANG00000043005 | ENSOANG00000043005 | O_anatinus | Animals | mono_intergenic | H/ACA | | | intergenic |
| ENSOANG00000043008 | ENSOANG00000043008 | O_anatinus | Animals | mono_intronic | H/ACA | ENSOANG00000013780 | NECAB2 | protein_coding |
| ENSOANG00000043009 | ENSOANG00000043009 | O_anatinus | Animals | mono_intergenic | H/ACA | | | intergenic |
| ENSOANG00000043010 | ENSOANG00000043010 | O_anatinus | Animals | mono_intergenic | H/ACA | | | intergenic |
| ENSOANG00000043014 | ENSOANG00000043014 | O_anatinus | Animals | mono_intergenic | H/ACA | | | intergenic |
| ENSOANG00000043015 | ENSOANG00000043015 | O_anatinus | Animals | mono_intergenic | H/ACA | | | intergenic |
| ENSOANG00000043019 | ENSOANG00000043019 | O_anatinus | Animals | mono_intergenic | H/ACA | | | intergenic |
| ENSOANG00000043024 | ENSOANG00000043024 | O_anatinus | Animals | mono_intronic | H/ACA | ENSOANG00000000140 | TTC23 | protein_coding |
| ENSOANG00000043025 | ENSOANG00000043025 | O_anatinus | Animals | mono_intergenic | H/ACA | | | intergenic |
| ENSOANG00000043029 | ENSOANG00000043029 | O_anatinus | Animals | mono_intergenic | H/ACA | | | intergenic |
| ENSOANG00000043032 | ENSOANG00000043032 | O_anatinus | Animals | mono_intronic | H/ACA | ENSOANG00000014971 | TMEM178A | protein_coding |
| ENSOANG00000043033 | SNORD79 | O_anatinus | Animals | intergenic_cluster | C/D | | | intergenic |
| ENSOANG00000043035 | ENSOANG00000043035 | O_anatinus | Animals | mono_intergenic | H/ACA | | | intergenic |
| ENSOANG00000043039 | ENSOANG00000043039 | O_anatinus | Animals | mono_intergenic | H/ACA | | | intergenic |

| | | | | | | | | |
|---|---|---|---|---|---|---|---|---|
| ENSOANG00000043040 | ENSOANG00000043040 | O_anatinus | Animals | mono_intergenic | H/ACA | | | intergenic |
| ENSOANG00000043041 | ENSOANG00000043041 | O_anatinus | Animals | mono_intergenic | H/ACA | | | intergenic |
| ENSOANG00000043044 | ENSOANG00000043044 | O_anatinus | Animals | mono_intergenic | H/ACA | | | intergenic |
| ENSOANG00000043045 | ENSOANG00000043045 | O_anatinus | Animals | mono_intergenic | H/ACA | | | intergenic |
| ENSOANG00000043048 | ENSOANG00000043048 | O_anatinus | Animals | mono_intergenic | H/ACA | | | intergenic |
| ENSOANG00000043051 | ENSOANG00000043051 | O_anatinus | Animals | mono_intergenic | H/ACA | | | intergenic |
| ENSOANG00000043053 | ENSOANG00000043053 | O_anatinus | Animals | mono_intergenic | H/ACA | | | intergenic |
| ENSOANG00000043054 | ENSOANG00000043054 | O_anatinus | Animals | mono_intergenic | H/ACA | | | intergenic |
| ENSOANG00000043056 | ENSOANG00000043056 | O_anatinus | Animals | mono_intergenic | H/ACA | | | intergenic |
| ENSOANG00000043063 | ENSOANG00000043063 | O_anatinus | Animals | mono_intergenic | H/ACA | | | intergenic |
| ENSOANG00000043065 | ENSOANG00000043065 | O_anatinus | Animals | mono_intronic | H/ACA | ENSOANG00000013307 | DAG1 | protein_coding |
| ENSOANG00000043066 | ENSOANG00000043066 | O_anatinus | Animals | mono_intergenic | H/ACA | | | intergenic |
| ENSOANG00000043069 | ENSOANG00000043069 | O_anatinus | Animals | mono_intergenic | H/ACA | | | intergenic |
| ENSOANG00000043072 | ENSOANG00000043072 | O_anatinus | Animals | mono_intergenic | H/ACA | | | intergenic |
| ENSOANG00000043073 | ENSOANG00000043073 | O_anatinus | Animals | mono_intergenic | H/ACA | | | intergenic |
| ENSOANG00000043075 | ENSOANG00000043075 | O_anatinus | Animals | mono_intergenic | H/ACA | | | intergenic |
| ENSOANG00000043078 | ENSOANG00000043078 | O_anatinus | Animals | mono_intergenic | H/ACA | | | intergenic |
| ENSOANG00000043079 | ENSOANG00000043079 | O_anatinus | Animals | mono_intergenic | H/ACA | | | intergenic |
| ENSOANG00000043080 | ENSOANG00000043080 | O_anatinus | Animals | mono_intergenic | H/ACA | | | intergenic |
| ENSOANG00000043081 | ENSOANG00000043081 | O_anatinus | Animals | mono_intergenic | H/ACA | | | intergenic |
| ENSOANG00000043086 | ENSOANG00000043086 | O_anatinus | Animals | mono_intergenic | H/ACA | | | intergenic |
| ENSOANG00000043090 | ENSOANG00000043090 | O_anatinus | Animals | mono_intergenic | H/ACA | | | intergenic |
| ENSOANG00000043092 | ENSOANG00000043092 | O_anatinus | Animals | mono_intergenic | H/ACA | | | intergenic |
| ENSOANG00000043097 | ENSOANG00000043097 | O_anatinus | Animals | mono_intergenic | H/ACA | | | intergenic |
| ENSOANG00000043104 | ENSOANG00000043104 | O_anatinus | Animals | mono_intergenic | H/ACA | | | intergenic |
| ENSOANG00000043107 | ENSOANG00000043107 | O_anatinus | Animals | mono_intergenic | H/ACA | | | intergenic |
| ENSOANG00000043108 | ENSOANG00000043108 | O_anatinus | Animals | mono_intergenic | H/ACA | | | intergenic |
| ENSOANG00000043109 | ENSOANG00000043109 | O_anatinus | Animals | mono_intergenic | H/ACA | | | intergenic |
| ENSOANG00000043115 | ENSOANG00000043115 | O_anatinus | Animals | mono_intergenic | H/ACA | | | intergenic |
| ENSOANG00000043133 | ENSOANG00000043133 | O_anatinus | Animals | mono_intergenic | H/ACA | | | intergenic |
| ENSOANG00000043134 | ENSOANG00000043134 | O_anatinus | Animals | mono_intergenic | H/ACA | | | intergenic |
| ENSOANG00000043137 | ENSOANG00000043137 | O_anatinus | Animals | mono_intergenic | H/ACA | | | intergenic |
| ENSOANG00000043140 | ENSOANG00000043140 | O_anatinus | Animals | mono_intergenic | H/ACA | | | intergenic |
| ENSOANG00000043143 | ENSOANG00000043143 | O_anatinus | Animals | mono_intergenic | H/ACA | | | intergenic |
| ENSOANG00000043144 | ENSOANG00000043144 | O_anatinus | Animals | mono_intronic | H/ACA | ENSOANG00000020373 | SLCO3A1 | protein_coding |
| ENSOANG00000043146 | ENSOANG00000043146 | O_anatinus | Animals | mono_intergenic | H/ACA | | | intergenic |
| ENSOANG00000043148 | ENSOANG00000043148 | O_anatinus | Animals | mono_intergenic | H/ACA | | | intergenic |
| ENSOANG00000043150 | ENSOANG00000043150 | O_anatinus | Animals | mono_intergenic | H/ACA | | | intergenic |
| ENSOANG00000043152 | ENSOANG00000043152 | O_anatinus | Animals | mono_intergenic | H/ACA | | | intergenic |
| ENSOANG00000043154 | ENSOANG00000043154 | O_anatinus | Animals | mono_intergenic | H/ACA | | | intergenic |
| ENSOANG00000043165 | ENSOANG00000043165 | O_anatinus | Animals | mono_intergenic | H/ACA | | | intergenic |

| | | | | | | | | |
|---|---|---|---|---|---|---|---|---|
| ENSOANG00000043168 | ENSOANG00000043168 | O_anatinus | Animals | mono_intergenic | H/ACA | | | intergenic |
| ENSOANG00000043170 | ENSOANG00000043170 | O_anatinus | Animals | mono_intergenic | H/ACA | | | intergenic |
| ENSOANG00000043171 | ENSOANG00000043171 | O_anatinus | Animals | mono_intergenic | H/ACA | | | intergenic |
| ENSOANG00000043172 | ENSOANG00000043172 | O_anatinus | Animals | mono_intronic | H/ACA | ENSOANG00000048356 | DAPK2 | protein_coding |
| ENSOANG00000043178 | ENSOANG00000043178 | O_anatinus | Animals | mono_intergenic | H/ACA | | | intergenic |
| ENSOANG00000043180 | SNORD31 | O_anatinus | Animals | intergenic_cluster | C/D | | | intergenic |
| ENSOANG00000043182 | ENSOANG00000043182 | O_anatinus | Animals | mono_intergenic | H/ACA | | | intergenic |
| ENSOANG00000043191 | ENSOANG00000043191 | O_anatinus | Animals | mono_intergenic | H/ACA | | | intergenic |
| ENSOANG00000043192 | ENSOANG00000043192 | O_anatinus | Animals | mono_intergenic | H/ACA | | | intergenic |
| ENSOANG00000043195 | ENSOANG00000043195 | O_anatinus | Animals | mono_intergenic | H/ACA | | | intergenic |
| ENSOANG00000043196 | ENSOANG00000043196 | O_anatinus | Animals | mono_intergenic | H/ACA | | | intergenic |
| ENSOANG00000043197 | ENSOANG00000043197 | O_anatinus | Animals | mono_intergenic | H/ACA | | | intergenic |
| ENSOANG00000043206 | ENSOANG00000043206 | O_anatinus | Animals | mono_intergenic | H/ACA | | | intergenic |
| ENSOANG00000043207 | ENSOANG00000043207 | O_anatinus | Animals | mono_intergenic | H/ACA | | | intergenic |
| ENSOANG00000043208 | ENSOANG00000043208 | O_anatinus | Animals | mono_intergenic | H/ACA | | | intergenic |
| ENSOANG00000043209 | ENSOANG00000043209 | O_anatinus | Animals | mono_intronic | H/ACA | ENSOANG00000002281 | RIN2 | protein_coding |
| ENSOANG00000043210 | ENSOANG00000043210 | O_anatinus | Animals | mono_intronic | H/ACA | ENSOANG00000047227 | | protein_coding |
| ENSOANG00000043212 | ENSOANG00000043212 | O_anatinus | Animals | mono_intergenic | H/ACA | | | intergenic |
| ENSOANG00000043215 | ENSOANG00000043215 | O_anatinus | Animals | mono_intergenic | H/ACA | | | intergenic |
| ENSOANG00000043217 | ENSOANG00000043217 | O_anatinus | Animals | mono_intronic | H/ACA | ENSOANG00000020206 | LDAH | protein_coding |
| ENSOANG00000043218 | ENSOANG00000043218 | O_anatinus | Animals | mono_intergenic | H/ACA | | | intergenic |
| ENSOANG00000043219 | ENSOANG00000043219 | O_anatinus | Animals | mono_intronic | H/ACA | ENSOANG00000011575 | TMEM161A | protein_coding |
| ENSOANG00000043221 | ENSOANG00000043221 | O_anatinus | Animals | mono_intergenic | H/ACA | | | intergenic |
| ENSOANG00000043222 | ENSOANG00000043222 | O_anatinus | Animals | mono_intergenic | H/ACA | | | intergenic |
| ENSOANG00000043225 | ENSOANG00000043225 | O_anatinus | Animals | mono_intergenic | H/ACA | | | intergenic |
| ENSOANG00000043226 | ENSOANG00000043226 | O_anatinus | Animals | mono_intergenic | H/ACA | | | intergenic |
| ENSOANG00000043227 | ENSOANG00000043227 | O_anatinus | Animals | mono_intergenic | H/ACA | | | intergenic |
| ENSOANG00000043229 | ENSOANG00000043229 | O_anatinus | Animals | mono_intergenic | H/ACA | | | intergenic |
| ENSOANG00000043230 | ENSOANG00000043230 | O_anatinus | Animals | mono_intergenic | H/ACA | | | intergenic |
| ENSOANG00000043232 | ENSOANG00000043232 | O_anatinus | Animals | mono_intergenic | H/ACA | | | intergenic |
| ENSOANG00000043238 | ENSOANG00000043238 | O_anatinus | Animals | mono_intergenic | H/ACA | | | intergenic |
| ENSOANG00000043239 | ENSOANG00000043239 | O_anatinus | Animals | mono_intergenic | H/ACA | | | intergenic |
| ENSOANG00000043242 | ENSOANG00000043242 | O_anatinus | Animals | mono_intergenic | H/ACA | | | intergenic |
| ENSOANG00000043243 | ENSOANG00000043243 | O_anatinus | Animals | mono_intronic | H/ACA | ENSOANG00000003573 | | protein_coding |
| ENSOANG00000043244 | ENSOANG00000043244 | O_anatinus | Animals | mono_intergenic | H/ACA | | | intergenic |
| ENSOANG00000043245 | ENSOANG00000043245 | O_anatinus | Animals | mono_intronic | H/ACA | ENSOANG00000010369 | APBA2 | protein_coding |
| ENSOANG00000043247 | ENSOANG00000043247 | O_anatinus | Animals | mono_intergenic | H/ACA | | | intergenic |
| ENSOANG00000043251 | ENSOANG00000043251 | O_anatinus | Animals | mono_intergenic | H/ACA | | | intergenic |
| ENSOANG00000043252 | ENSOANG00000043252 | O_anatinus | Animals | mono_intergenic | H/ACA | | | intergenic |
| ENSOANG00000043259 | ENSOANG00000043259 | O_anatinus | Animals | mono_intergenic | H/ACA | | | intergenic |
| ENSOANG00000043260 | ENSOANG00000043260 | O_anatinus | Animals | mono_intergenic | H/ACA | | | intergenic |

| | | | | | | | | |
|---|---|---|---|---|---|---|---|---|
| ENSOANG00000043263 | U8 | O_anatinus | Animals | mono_intergenic | C/D | | | intergenic |
| ENSOANG00000043265 | ENSOANG00000043265 | O_anatinus | Animals | mono_intronic | H/ACA | ENSOANG00000011339 | WFDC1 | protein_coding |
| ENSOANG00000043267 | ENSOANG00000043267 | O_anatinus | Animals | mono_intergenic | H/ACA | | | intergenic |
| ENSOANG00000043268 | ENSOANG00000043268 | O_anatinus | Animals | mono_intergenic | H/ACA | | | intergenic |
| ENSOANG00000043274 | ENSOANG00000043274 | O_anatinus | Animals | mono_intergenic | H/ACA | | | intergenic |
| ENSOANG00000043275 | ENSOANG00000043275 | O_anatinus | Animals | mono_intergenic | H/ACA | | | intergenic |
| ENSOANG00000043278 | ENSOANG00000043278 | O_anatinus | Animals | mono_intergenic | H/ACA | | | intergenic |
| ENSOANG00000043279 | ENSOANG00000043279 | O_anatinus | Animals | mono_intronic | H/ACA | ENSOANG00000050093 | | protein_coding |
| ENSOANG00000043280 | ENSOANG00000043280 | O_anatinus | Animals | mono_intergenic | H/ACA | | | intergenic |
| ENSOANG00000043283 | ENSOANG00000043283 | O_anatinus | Animals | mono_intergenic | H/ACA | | | intergenic |
| ENSOANG00000043284 | ENSOANG00000043284 | O_anatinus | Animals | mono_intergenic | H/ACA | | | intergenic |
| ENSOANG00000043285 | ENSOANG00000043285 | O_anatinus | Animals | mono_intergenic | H/ACA | | | intergenic |
| ENSOANG00000043290 | ENSOANG00000043290 | O_anatinus | Animals | mono_intergenic | H/ACA | | | intergenic |
| ENSOANG00000043291 | ENSOANG00000043291 | O_anatinus | Animals | mono_intronic | H/ACA | ENSOANG00000010371 | | protein_coding |
| ENSOANG00000043292 | ENSOANG00000043292 | O_anatinus | Animals | mono_intergenic | H/ACA | | | intergenic |
| ENSOANG00000043294 | ENSOANG00000043294 | O_anatinus | Animals | mono_intronic | H/ACA | ENSOANG00000005298 | UTRN | protein_coding |
| ENSOANG00000043296 | ENSOANG00000043296 | O_anatinus | Animals | mono_intergenic | H/ACA | | | intergenic |
| ENSOANG00000043298 | ENSOANG00000043298 | O_anatinus | Animals | mono_intergenic | H/ACA | | | intergenic |
| ENSOANG00000043299 | ENSOANG00000043299 | O_anatinus | Animals | mono_intergenic | H/ACA | | | intergenic |
| ENSOANG00000043300 | ENSOANG00000043300 | O_anatinus | Animals | mono_intergenic | H/ACA | | | intergenic |
| ENSOANG00000043301 | ENSOANG00000043301 | O_anatinus | Animals | mono_intronic | H/ACA | ENSOANG00000007021 | ERBB4 | protein_coding |
| ENSOANG00000043302 | ENSOANG00000043302 | O_anatinus | Animals | mono_intergenic | H/ACA | | | intergenic |
| ENSOANG00000043303 | ENSOANG00000043303 | O_anatinus | Animals | mono_intergenic | H/ACA | | | intergenic |
| ENSOANG00000043310 | ENSOANG00000043310 | O_anatinus | Animals | mono_intergenic | H/ACA | | | intergenic |
| ENSOANG00000043315 | ENSOANG00000043315 | O_anatinus | Animals | mono_intergenic | H/ACA | | | intergenic |
| ENSOANG00000043316 | ENSOANG00000043316 | O_anatinus | Animals | mono_intergenic | H/ACA | | | intergenic |
| ENSOANG00000043318 | ENSOANG00000043318 | O_anatinus | Animals | mono_intergenic | H/ACA | | | intergenic |
| ENSOANG00000043319 | ENSOANG00000043319 | O_anatinus | Animals | mono_intronic | H/ACA | ENSOANG00000047862 | SMTNL2 | protein_coding |
| ENSOANG00000043323 | ENSOANG00000043323 | O_anatinus | Animals | mono_intergenic | H/ACA | | | intergenic |
| ENSOANG00000043324 | ENSOANG00000043324 | O_anatinus | Animals | mono_intronic | H/ACA | ENSOANG00000037893 | ENTPD1 | protein_coding |
| ENSOANG00000043326 | ENSOANG00000043326 | O_anatinus | Animals | mono_intergenic | H/ACA | | | intergenic |
| ENSOANG00000043328 | ENSOANG00000043328 | O_anatinus | Animals | intergenic_cluster | H/ACA | | | intergenic |
| ENSOANG00000043334 | ENSOANG00000043334 | O_anatinus | Animals | mono_intergenic | H/ACA | | | intergenic |
| ENSOANG00000043338 | ENSOANG00000043338 | O_anatinus | Animals | mono_intergenic | H/ACA | | | intergenic |
| ENSOANG00000043349 | ENSOANG00000043349 | O_anatinus | Animals | mono_intergenic | H/ACA | | | intergenic |
| ENSOANG00000043350 | ENSOANG00000043350 | O_anatinus | Animals | mono_intergenic | H/ACA | | | intergenic |
| ENSOANG00000043351 | ENSOANG00000043351 | O_anatinus | Animals | mono_intergenic | H/ACA | | | intergenic |
| ENSOANG00000043353 | ENSOANG00000043353 | O_anatinus | Animals | mono_intergenic | H/ACA | | | intergenic |
| ENSOANG00000043357 | ENSOANG00000043357 | O_anatinus | Animals | mono_intergenic | H/ACA | | | intergenic |
| ENSOANG00000043360 | ENSOANG00000043360 | O_anatinus | Animals | mono_intergenic | H/ACA | | | intergenic |
| ENSOANG00000043361 | ENSOANG00000043361 | O_anatinus | Animals | mono_intergenic | H/ACA | | | intergenic |

| | | | | | | | | |
|---|---|---|---|---|---|---|---|---|
| ENSOANG00000043364 | ENSOANG00000043364 | O_anatinus | Animals | mono_intergenic | H/ACA | | | intergenic |
| ENSOANG00000043373 | ENSOANG00000043373 | O_anatinus | Animals | mono_intergenic | H/ACA | | | intergenic |
| ENSOANG00000043374 | ENSOANG00000043374 | O_anatinus | Animals | mono_intergenic | H/ACA | | | intergenic |
| ENSOANG00000043375 | ENSOANG00000043375 | O_anatinus | Animals | mono_intergenic | H/ACA | | | intergenic |
| ENSOANG00000043378 | ENSOANG00000043378 | O_anatinus | Animals | mono_intergenic | H/ACA | | | intergenic |
| ENSOANG00000043384 | ENSOANG00000043384 | O_anatinus | Animals | mono_intergenic | H/ACA | | | intergenic |
| ENSOANG00000043385 | ENSOANG00000043385 | O_anatinus | Animals | mono_intergenic | H/ACA | | | intergenic |
| ENSOANG00000043386 | ENSOANG00000043386 | O_anatinus | Animals | mono_intergenic | H/ACA | | | intergenic |
| ENSOANG00000043387 | ENSOANG00000043387 | O_anatinus | Animals | mono_intergenic | H/ACA | | | intergenic |
| ENSOANG00000043389 | ENSOANG00000043389 | O_anatinus | Animals | mono_intergenic | H/ACA | | | intergenic |
| ENSOANG00000043392 | ENSOANG00000043392 | O_anatinus | Animals | mono_intergenic | H/ACA | | | intergenic |
| ENSOANG00000043396 | ENSOANG00000043396 | O_anatinus | Animals | mono_intronic | H/ACA | ENSOANG00000014655 | DRG1 | protein_coding |
| ENSOANG00000043399 | ENSOANG00000043399 | O_anatinus | Animals | mono_intergenic | H/ACA | | | intergenic |
| ENSOANG00000043401 | ENSOANG00000043401 | O_anatinus | Animals | mono_intergenic | H/ACA | | | intergenic |
| ENSOANG00000043408 | ENSOANG00000043408 | O_anatinus | Animals | mono_intergenic | H/ACA | | | intergenic |
| ENSOANG00000043410 | ENSOANG00000043410 | O_anatinus | Animals | mono_intergenic | H/ACA | | | intergenic |
| ENSOANG00000043414 | ENSOANG00000043414 | O_anatinus | Animals | mono_intergenic | H/ACA | | | intergenic |
| ENSOANG00000043418 | ENSOANG00000043418 | O_anatinus | Animals | mono_intergenic | H/ACA | | | intergenic |
| ENSOANG00000043419 | ENSOANG00000043419 | O_anatinus | Animals | mono_intergenic | H/ACA | | | intergenic |
| ENSOANG00000043421 | ENSOANG00000043421 | O_anatinus | Animals | mono_intergenic | H/ACA | | | intergenic |
| ENSOANG00000043422 | ENSOANG00000043422 | O_anatinus | Animals | mono_intergenic | H/ACA | | | intergenic |
| ENSOANG00000043423 | ENSOANG00000043423 | O_anatinus | Animals | mono_intergenic | H/ACA | | | intergenic |
| ENSOANG00000043424 | ENSOANG00000043424 | O_anatinus | Animals | mono_intergenic | H/ACA | | | intergenic |
| ENSOANG00000043427 | ENSOANG00000043427 | O_anatinus | Animals | mono_intronic | H/ACA | ENSOANG00000039698 | | non_coding |
| ENSOANG00000043429 | ENSOANG00000043429 | O_anatinus | Animals | mono_intergenic | H/ACA | | | intergenic |
| ENSOANG00000043431 | ENSOANG00000043431 | O_anatinus | Animals | mono_intergenic | H/ACA | | | intergenic |
| ENSOANG00000043434 | ENSOANG00000043434 | O_anatinus | Animals | mono_intergenic | H/ACA | | | intergenic |
| ENSOANG00000043435 | ENSOANG00000043435 | O_anatinus | Animals | mono_intergenic | H/ACA | | | intergenic |
| ENSOANG00000043438 | ENSOANG00000043438 | O_anatinus | Animals | mono_intergenic | H/ACA | | | intergenic |
| ENSOANG00000043441 | ENSOANG00000043441 | O_anatinus | Animals | mono_intergenic | H/ACA | | | intergenic |
| ENSOANG00000043443 | ENSOANG00000043443 | O_anatinus | Animals | mono_intergenic | H/ACA | | | intergenic |
| ENSOANG00000043444 | ENSOANG00000043444 | O_anatinus | Animals | mono_intergenic | H/ACA | | | intergenic |
| ENSOANG00000043446 | ENSOANG00000043446 | O_anatinus | Animals | mono_intergenic | H/ACA | | | intergenic |
| ENSOANG00000043453 | ENSOANG00000043453 | O_anatinus | Animals | mono_intergenic | H/ACA | | | intergenic |
| ENSOANG00000043455 | ENSOANG00000043455 | O_anatinus | Animals | mono_intergenic | H/ACA | | | intergenic |
| ENSOANG00000043456 | ENSOANG00000043456 | O_anatinus | Animals | mono_intergenic | H/ACA | | | intergenic |
| ENSOANG00000043459 | ENSOANG00000043459 | O_anatinus | Animals | mono_intergenic | H/ACA | | | intergenic |
| ENSOANG00000043460 | ENSOANG00000043460 | O_anatinus | Animals | mono_intronic | H/ACA | ENSOANG00000013898 | | protein_coding |
| ENSOANG00000043464 | ENSOANG00000043464 | O_anatinus | Animals | mono_intergenic | H/ACA | | | intergenic |
| ENSOANG00000043466 | ENSOANG00000043466 | O_anatinus | Animals | mono_intergenic | H/ACA | | | intergenic |
| ENSOANG00000043467 | ENSOANG00000043467 | O_anatinus | Animals | mono_intergenic | H/ACA | | | intergenic |

| | | | | | | | | |
|---|---|---|---|---|---|---|---|---|
| ENSOANG00000043469 | ENSOANG00000043469 | O_anatinus | Animals | mono_intergenic | H/ACA | | | intergenic |
| ENSOANG00000043471 | ENSOANG00000043471 | O_anatinus | Animals | mono_intergenic | H/ACA | | | intergenic |
| ENSOANG00000043473 | ENSOANG00000043473 | O_anatinus | Animals | mono_intergenic | H/ACA | | | intergenic |
| ENSOANG00000043477 | ENSOANG00000043477 | O_anatinus | Animals | mono_intergenic | H/ACA | | | intergenic |
| ENSOANG00000043478 | ENSOANG00000043478 | O_anatinus | Animals | mono_intergenic | H/ACA | | | intergenic |
| ENSOANG00000043480 | ENSOANG00000043480 | O_anatinus | Animals | mono_intronic | H/ACA | ENSOANG00000004617 | MYPN | protein_coding |
| ENSOANG00000043481 | ENSOANG00000043481 | O_anatinus | Animals | mono_intergenic | H/ACA | | | intergenic |
| ENSOANG00000043484 | ENSOANG00000043484 | O_anatinus | Animals | mono_intronic | H/ACA | ENSOANG00000010807 | MON2 | protein_coding |
| ENSOANG00000043485 | ENSOANG00000043485 | O_anatinus | Animals | mono_intergenic | H/ACA | | | intergenic |
| ENSOANG00000043490 | ENSOANG00000043490 | O_anatinus | Animals | mono_intergenic | H/ACA | | | intergenic |
| ENSOANG00000043496 | ENSOANG00000043496 | O_anatinus | Animals | mono_intronic | H/ACA | ENSOANG00000002862 | SMARCA1 | protein_coding |
| ENSOANG00000043498 | ENSOANG00000043498 | O_anatinus | Animals | mono_intergenic | H/ACA | | | intergenic |
| ENSOANG00000043499 | ENSOANG00000043499 | O_anatinus | Animals | mono_intergenic | H/ACA | | | intergenic |
| ENSOANG00000043502 | ENSOANG00000043502 | O_anatinus | Animals | mono_intergenic | H/ACA | | | intergenic |
| ENSOANG00000043504 | ENSOANG00000043504 | O_anatinus | Animals | mono_intergenic | H/ACA | | | intergenic |
| ENSOANG00000043505 | ENSOANG00000043505 | O_anatinus | Animals | mono_intergenic | H/ACA | | | intergenic |
| ENSOANG00000043510 | ENSOANG00000043510 | O_anatinus | Animals | mono_intergenic | H/ACA | | | intergenic |
| ENSOANG00000043511 | ENSOANG00000043511 | O_anatinus | Animals | mono_intergenic | H/ACA | | | intergenic |
| ENSOANG00000043513 | ENSOANG00000043513 | O_anatinus | Animals | mono_intronic | H/ACA | ENSOANG00000001812 | PLOD2 | protein_coding |
| ENSOANG00000043514 | ENSOANG00000043514 | O_anatinus | Animals | mono_intronic | H/ACA | ENSOANG00000038313 | SRL | protein_coding |
| ENSOANG00000043522 | ENSOANG00000043522 | O_anatinus | Animals | mono_intergenic | H/ACA | | | intergenic |
| ENSOANG00000043523 | ENSOANG00000043523 | O_anatinus | Animals | mono_intergenic | H/ACA | | | intergenic |
| ENSOANG00000043526 | ENSOANG00000043526 | O_anatinus | Animals | mono_intergenic | H/ACA | | | intergenic |
| ENSOANG00000043529 | ENSOANG00000043529 | O_anatinus | Animals | mono_intergenic | H/ACA | | | intergenic |
| ENSOANG00000043532 | ENSOANG00000043532 | O_anatinus | Animals | mono_intergenic | H/ACA | | | intergenic |
| ENSOANG00000043535 | ENSOANG00000043535 | O_anatinus | Animals | mono_intergenic | H/ACA | | | intergenic |
| ENSOANG00000043536 | ENSOANG00000043536 | O_anatinus | Animals | mono_intergenic | H/ACA | | | intergenic |
| ENSOANG00000043538 | ENSOANG00000043538 | O_anatinus | Animals | mono_intergenic | H/ACA | | | intergenic |
| ENSOANG00000043540 | ENSOANG00000043540 | O_anatinus | Animals | mono_intergenic | H/ACA | | | intergenic |
| ENSOANG00000043546 | ENSOANG00000043546 | O_anatinus | Animals | mono_intergenic | H/ACA | | | intergenic |
| ENSOANG00000043547 | U3 | O_anatinus | Animals | intronic_cluster | C/D | ENSOANG00000008488 | | protein_coding |
| ENSOANG00000043555 | ENSOANG00000043555 | O_anatinus | Animals | mono_intronic | H/ACA | ENSOANG00000014760 | HAPLN1 | protein_coding |
| ENSOANG00000043561 | ENSOANG00000043561 | O_anatinus | Animals | mono_intronic | H/ACA | ENSOANG00000011542 | | protein_coding |
| ENSOANG00000043562 | ENSOANG00000043562 | O_anatinus | Animals | mono_intergenic | H/ACA | | | intergenic |
| ENSOANG00000043564 | ENSOANG00000043564 | O_anatinus | Animals | mono_intergenic | H/ACA | | | intergenic |
| ENSOANG00000043565 | ENSOANG00000043565 | O_anatinus | Animals | mono_intergenic | H/ACA | | | intergenic |
| ENSOANG00000043566 | ENSOANG00000043566 | O_anatinus | Animals | mono_intergenic | H/ACA | | | intergenic |
| ENSOANG00000043568 | ENSOANG00000043568 | O_anatinus | Animals | mono_intergenic | H/ACA | | | intergenic |
| ENSOANG00000043573 | ENSOANG00000043573 | O_anatinus | Animals | mono_intergenic | H/ACA | | | intergenic |
| ENSOANG00000043574 | ENSOANG00000043574 | O_anatinus | Animals | mono_intergenic | H/ACA | | | intergenic |
| ENSOANG00000043575 | ENSOANG00000043575 | O_anatinus | Animals | mono_intergenic | H/ACA | | | intergenic |

| | | | | | | | | |
|---|---|---|---|---|---|---|---|---|
| ENSOANG00000043580 | ENSOANG00000043580 | O_anatinus | Animals | mono_intergenic | H/ACA | | | intergenic |
| ENSOANG00000043581 | ENSOANG00000043581 | O_anatinus | Animals | mono_intergenic | H/ACA | | | intergenic |
| ENSOANG00000043583 | ENSOANG00000043583 | O_anatinus | Animals | mono_intergenic | H/ACA | | | intergenic |
| ENSOANG00000043585 | ENSOANG00000043585 | O_anatinus | Animals | mono_intergenic | H/ACA | | | intergenic |
| ENSOANG00000043593 | ENSOANG00000043593 | O_anatinus | Animals | mono_intergenic | H/ACA | | | intergenic |
| ENSOANG00000043594 | ENSOANG00000043594 | O_anatinus | Animals | mono_intergenic | H/ACA | | | intergenic |
| ENSOANG00000043597 | ENSOANG00000043597 | O_anatinus | Animals | mono_intergenic | H/ACA | | | intergenic |
| ENSOANG00000043599 | ENSOANG00000043599 | O_anatinus | Animals | mono_exonic | C/D | ENSOANG00000050134 | | non_coding |
| ENSOANG00000043600 | ENSOANG00000043600 | O_anatinus | Animals | mono_intergenic | H/ACA | | | intergenic |
| ENSOANG00000043604 | ENSOANG00000043604 | O_anatinus | Animals | mono_intronic | H/ACA | ENSOANG00000010801 | AFG3L2 | protein_coding |
| ENSOANG00000043608 | ENSOANG00000043608 | O_anatinus | Animals | mono_intronic | C/D | ENSOANG00000013437 | GNL3 | protein_coding |
| ENSOANG00000043611 | ENSOANG00000043611 | O_anatinus | Animals | mono_intergenic | H/ACA | | | intergenic |
| ENSOANG00000043612 | ENSOANG00000043612 | O_anatinus | Animals | mono_intergenic | H/ACA | | | intergenic |
| ENSOANG00000043614 | ENSOANG00000043614 | O_anatinus | Animals | mono_intergenic | H/ACA | | | intergenic |
| ENSOANG00000043619 | ENSOANG00000043619 | O_anatinus | Animals | mono_intergenic | H/ACA | | | intergenic |
| ENSOANG00000043625 | ENSOANG00000043625 | O_anatinus | Animals | mono_intergenic | H/ACA | | | intergenic |
| ENSOANG00000043629 | ENSOANG00000043629 | O_anatinus | Animals | mono_intergenic | H/ACA | | | intergenic |
| ENSOANG00000043635 | SNORD22 | O_anatinus | Animals | intergenic_cluster | C/D | | | intergenic |
| ENSOANG00000043636 | ENSOANG00000043636 | O_anatinus | Animals | mono_intergenic | H/ACA | | | intergenic |
| ENSOANG00000043638 | ENSOANG00000043638 | O_anatinus | Animals | mono_intergenic | H/ACA | | | intergenic |
| ENSOANG00000043641 | ENSOANG00000043641 | O_anatinus | Animals | mono_intronic | H/ACA | ENSOANG00000015755 | PIEZO2 | protein_coding |
| ENSOANG00000043643 | ENSOANG00000043643 | O_anatinus | Animals | mono_intergenic | H/ACA | | | intergenic |
| ENSOANG00000043644 | ENSOANG00000043644 | O_anatinus | Animals | mono_intergenic | H/ACA | | | intergenic |
| ENSOANG00000043645 | ENSOANG00000043645 | O_anatinus | Animals | mono_intergenic | H/ACA | | | intergenic |
| ENSOANG00000043648 | ENSOANG00000043648 | O_anatinus | Animals | mono_intronic | H/ACA | ENSOANG00000051233 | ARNT2 | protein_coding |
| ENSOANG00000043652 | ENSOANG00000043652 | O_anatinus | Animals | mono_intronic | H/ACA | ENSOANG00000003796 | RFX2 | protein_coding |
| ENSOANG00000043655 | ENSOANG00000043655 | O_anatinus | Animals | mono_intergenic | H/ACA | | | intergenic |
| ENSOANG00000043656 | ENSOANG00000043656 | O_anatinus | Animals | mono_intergenic | H/ACA | | | intergenic |
| ENSOANG00000043659 | ENSOANG00000043659 | O_anatinus | Animals | mono_intronic | H/ACA | ENSOANG00000010323 | | protein_coding |
| ENSOANG00000043666 | ENSOANG00000043666 | O_anatinus | Animals | mono_intergenic | H/ACA | | | intergenic |
| ENSOANG00000043669 | ENSOANG00000043669 | O_anatinus | Animals | mono_intronic | H/ACA | ENSOANG00000021074 | CEP43 | protein_coding |
| ENSOANG00000043670 | ENSOANG00000043670 | O_anatinus | Animals | mono_intergenic | H/ACA | | | intergenic |
| ENSOANG00000043673 | ENSOANG00000043673 | O_anatinus | Animals | mono_intergenic | H/ACA | | | intergenic |
| ENSOANG00000043675 | ENSOANG00000043675 | O_anatinus | Animals | mono_intergenic | H/ACA | | | intergenic |
| ENSOANG00000043680 | ENSOANG00000043680 | O_anatinus | Animals | mono_intergenic | H/ACA | | | intergenic |
| ENSOANG00000043683 | ENSOANG00000043683 | O_anatinus | Animals | mono_intronic | H/ACA | ENSOANG00000008767 | CFTR | protein_coding |
| ENSOANG00000043684 | ENSOANG00000043684 | O_anatinus | Animals | mono_intronic | H/ACA | ENSOANG00000003310 | SEC24D | protein_coding |
| ENSOANG00000043687 | ENSOANG00000043687 | O_anatinus | Animals | mono_intergenic | H/ACA | | | intergenic |
| ENSOANG00000043691 | ENSOANG00000043691 | O_anatinus | Animals | intronic_cluster | H/ACA | ENSOANG00000043246 | | protein_coding |
| ENSOANG00000043695 | ENSOANG00000043695 | O_anatinus | Animals | mono_intergenic | H/ACA | | | intergenic |
| ENSOANG00000043697 | ENSOANG00000043697 | O_anatinus | Animals | mono_intergenic | H/ACA | | | intergenic |

| | | | | | | | | |
|---|---|---|---|---|---|---|---|---|
| ENSOANG00000043699 | ENSOANG00000043699 | O_anatinus | Animals | mono_intergenic | H/ACA | | | intergenic |
| ENSOANG00000043702 | ENSOANG00000043702 | O_anatinus | Animals | mono_intergenic | H/ACA | | | intergenic |
| ENSOANG00000043703 | ENSOANG00000043703 | O_anatinus | Animals | mono_intergenic | H/ACA | | | intergenic |
| ENSOANG00000043706 | ENSOANG00000043706 | O_anatinus | Animals | mono_intergenic | H/ACA | | | intergenic |
| ENSOANG00000043711 | ENSOANG00000043711 | O_anatinus | Animals | mono_intergenic | H/ACA | | | intergenic |
| ENSOANG00000043713 | ENSOANG00000043713 | O_anatinus | Animals | mono_intergenic | H/ACA | | | intergenic |
| ENSOANG00000043714 | ENSOANG00000043714 | O_anatinus | Animals | mono_intronic | H/ACA | ENSOANG00000009947 | TMEM132C | protein_coding |
| ENSOANG00000043715 | ENSOANG00000043715 | O_anatinus | Animals | mono_intergenic | H/ACA | | | intergenic |
| ENSOANG00000043716 | ENSOANG00000043716 | O_anatinus | Animals | mono_intergenic | H/ACA | | | intergenic |
| ENSOANG00000043718 | ENSOANG00000043718 | O_anatinus | Animals | mono_intergenic | H/ACA | | | intergenic |
| ENSOANG00000043722 | ENSOANG00000043722 | O_anatinus | Animals | mono_intergenic | H/ACA | | | intergenic |
| ENSOANG00000043723 | ENSOANG00000043723 | O_anatinus | Animals | mono_intronic | H/ACA | ENSOANG00000015043 | RELN | protein_coding |
| ENSOANG00000043724 | ENSOANG00000043724 | O_anatinus | Animals | mono_intergenic | H/ACA | | | intergenic |
| ENSOANG00000043727 | ENSOANG00000043727 | O_anatinus | Animals | mono_intergenic | H/ACA | | | intergenic |
| ENSOANG00000043728 | ENSOANG00000043728 | O_anatinus | Animals | mono_intergenic | H/ACA | | | intergenic |
| ENSOANG00000043735 | ENSOANG00000043735 | O_anatinus | Animals | mono_intronic | H/ACA | ENSOANG00000047227 | | protein_coding |
| ENSOANG00000043740 | ENSOANG00000043740 | O_anatinus | Animals | mono_intergenic | H/ACA | | | intergenic |
| ENSOANG00000043741 | ENSOANG00000043741 | O_anatinus | Animals | mono_intergenic | H/ACA | | | intergenic |
| ENSOANG00000043742 | SNORD14 | O_anatinus | Animals | mono_intronic | C/D | ENSOANG00000014580 | HSPA8 | protein_coding |
| ENSOANG00000043743 | ENSOANG00000043743 | O_anatinus | Animals | mono_intergenic | H/ACA | | | intergenic |
| ENSOANG00000043744 | ENSOANG00000043744 | O_anatinus | Animals | mono_intergenic | H/ACA | | | intergenic |
| ENSOANG00000043747 | ENSOANG00000043747 | O_anatinus | Animals | mono_intergenic | H/ACA | | | intergenic |
| ENSOANG00000043753 | ENSOANG00000043753 | O_anatinus | Animals | mono_intronic | H/ACA | ENSOANG00000044977 | BANK1 | protein_coding |
| ENSOANG00000043755 | ENSOANG00000043755 | O_anatinus | Animals | mono_intergenic | H/ACA | | | intergenic |
| ENSOANG00000043761 | ENSOANG00000043761 | O_anatinus | Animals | mono_intergenic | H/ACA | | | intergenic |
| ENSOANG00000043762 | ENSOANG00000043762 | O_anatinus | Animals | mono_intergenic | H/ACA | | | intergenic |
| ENSOANG00000043764 | ENSOANG00000043764 | O_anatinus | Animals | mono_intergenic | H/ACA | | | intergenic |
| ENSOANG00000043765 | ENSOANG00000043765 | O_anatinus | Animals | mono_intergenic | H/ACA | | | intergenic |
| ENSOANG00000043771 | ENSOANG00000043771 | O_anatinus | Animals | mono_intergenic | H/ACA | | | intergenic |
| ENSOANG00000043773 | ENSOANG00000043773 | O_anatinus | Animals | mono_intergenic | H/ACA | | | intergenic |
| ENSOANG00000043775 | SNORA50A | O_anatinus | Animals | intronic_cluster | H/ACA | ENSOANG00000012109 | CNOT1 | protein_coding |
| ENSOANG00000043777 | ENSOANG00000043777 | O_anatinus | Animals | mono_intergenic | H/ACA | | | intergenic |
| ENSOANG00000043779 | ENSOANG00000043779 | O_anatinus | Animals | mono_intergenic | H/ACA | | | intergenic |
| ENSOANG00000043781 | ENSOANG00000043781 | O_anatinus | Animals | mono_intergenic | H/ACA | | | intergenic |
| ENSOANG00000043796 | ENSOANG00000043796 | O_anatinus | Animals | mono_intergenic | H/ACA | | | intergenic |
| ENSOANG00000043797 | ENSOANG00000043797 | O_anatinus | Animals | mono_intergenic | H/ACA | | | intergenic |
| ENSOANG00000043799 | ENSOANG00000043799 | O_anatinus | Animals | mono_intergenic | H/ACA | | | intergenic |
| ENSOANG00000043801 | ENSOANG00000043801 | O_anatinus | Animals | mono_intergenic | H/ACA | | | intergenic |
| ENSOANG00000043803 | ENSOANG00000043803 | O_anatinus | Animals | mono_intronic | H/ACA | ENSOANG00000007134 | SYNRG | protein_coding |
| ENSOANG00000043806 | ENSOANG00000043806 | O_anatinus | Animals | mono_intergenic | H/ACA | | | intergenic |
| ENSOANG00000043808 | ENSOANG00000043808 | O_anatinus | Animals | mono_intergenic | H/ACA | | | intergenic |

| | | | | | | | | |
|---|---|---|---|---|---|---|---|---|
| ENSOANG00000043809 | ENSOANG00000043809 | O_anatinus | Animals | mono_intergenic | H/ACA | | | intergenic |
| ENSOANG00000043812 | ENSOANG00000043812 | O_anatinus | Animals | mono_intergenic | H/ACA | | | intergenic |
| ENSOANG00000043813 | ENSOANG00000043813 | O_anatinus | Animals | mono_intergenic | H/ACA | | | intergenic |
| ENSOANG00000043820 | ENSOANG00000043820 | O_anatinus | Animals | intergenic_cluster | H/ACA | | | intergenic |
| ENSOANG00000043825 | ENSOANG00000043825 | O_anatinus | Animals | mono_intergenic | H/ACA | | | intergenic |
| ENSOANG00000043826 | ENSOANG00000043826 | O_anatinus | Animals | mono_intergenic | H/ACA | | | intergenic |
| ENSOANG00000043827 | ENSOANG00000043827 | O_anatinus | Animals | mono_intergenic | H/ACA | | | intergenic |
| ENSOANG00000043828 | ENSOANG00000043828 | O_anatinus | Animals | mono_intergenic | H/ACA | | | intergenic |
| ENSOANG00000043829 | ENSOANG00000043829 | O_anatinus | Animals | mono_intergenic | H/ACA | | | intergenic |
| ENSOANG00000043832 | ENSOANG00000043832 | O_anatinus | Animals | mono_intergenic | H/ACA | | | intergenic |
| ENSOANG00000043836 | ENSOANG00000043836 | O_anatinus | Animals | mono_intronic | H/ACA | ENSOANG00000007283 | | protein_coding |
| ENSOANG00000043839 | ENSOANG00000043839 | O_anatinus | Animals | mono_intergenic | H/ACA | | | intergenic |
| ENSOANG00000043843 | ENSOANG00000043843 | O_anatinus | Animals | mono_intergenic | H/ACA | | | intergenic |
| ENSOANG00000043844 | ENSOANG00000043844 | O_anatinus | Animals | mono_intergenic | H/ACA | | | intergenic |
| ENSOANG00000043845 | ENSOANG00000043845 | O_anatinus | Animals | mono_intergenic | H/ACA | | | intergenic |
| ENSOANG00000043848 | ENSOANG00000043848 | O_anatinus | Animals | mono_intergenic | H/ACA | | | intergenic |
| ENSOANG00000043851 | ENSOANG00000043851 | O_anatinus | Animals | mono_intergenic | H/ACA | | | intergenic |
| ENSOANG00000043853 | ENSOANG00000043853 | O_anatinus | Animals | mono_intergenic | H/ACA | | | intergenic |
| ENSOANG00000043857 | ENSOANG00000043857 | O_anatinus | Animals | mono_intergenic | H/ACA | | | intergenic |
| ENSOANG00000043859 | ENSOANG00000043859 | O_anatinus | Animals | mono_intergenic | H/ACA | | | intergenic |
| ENSOANG00000043860 | ENSOANG00000043860 | O_anatinus | Animals | mono_intergenic | H/ACA | | | intergenic |
| ENSOANG00000043862 | ENSOANG00000043862 | O_anatinus | Animals | intergenic_cluster | H/ACA | | | intergenic |
| ENSOANG00000043863 | SNORD23 | O_anatinus | Animals | mono_intronic | C/D | ENSOANG00000050511 | NOP53 | protein_coding |
| ENSOANG00000043864 | ENSOANG00000043864 | O_anatinus | Animals | mono_intergenic | H/ACA | | | intergenic |
| ENSOANG00000043865 | ENSOANG00000043865 | O_anatinus | Animals | mono_intergenic | H/ACA | | | intergenic |
| ENSOANG00000043866 | ENSOANG00000043866 | O_anatinus | Animals | mono_intergenic | H/ACA | | | intergenic |
| ENSOANG00000043874 | ENSOANG00000043874 | O_anatinus | Animals | mono_intergenic | H/ACA | | | intergenic |
| ENSOANG00000043876 | ENSOANG00000043876 | O_anatinus | Animals | mono_intergenic | H/ACA | | | intergenic |
| ENSOANG00000043877 | ENSOANG00000043877 | O_anatinus | Animals | mono_intergenic | H/ACA | | | intergenic |
| ENSOANG00000043878 | ENSOANG00000043878 | O_anatinus | Animals | mono_intergenic | H/ACA | | | intergenic |
| ENSOANG00000043884 | ENSOANG00000043884 | O_anatinus | Animals | mono_intergenic | H/ACA | | | intergenic |
| ENSOANG00000043889 | ENSOANG00000043889 | O_anatinus | Animals | mono_intergenic | H/ACA | | | intergenic |
| ENSOANG00000043891 | ENSOANG00000043891 | O_anatinus | Animals | mono_intronic | H/ACA | ENSOANG00000039346 | SLC2A13 | protein_coding |
| ENSOANG00000043893 | ENSOANG00000043893 | O_anatinus | Animals | mono_intergenic | H/ACA | | | intergenic |
| ENSOANG00000043896 | ENSOANG00000043896 | O_anatinus | Animals | mono_intergenic | H/ACA | | | intergenic |
| ENSOANG00000043897 | ENSOANG00000043897 | O_anatinus | Animals | mono_intergenic | H/ACA | | | intergenic |
| ENSOANG00000043898 | ENSOANG00000043898 | O_anatinus | Animals | mono_intergenic | H/ACA | | | intergenic |
| ENSOANG00000043899 | ENSOANG00000043899 | O_anatinus | Animals | mono_intergenic | H/ACA | | | intergenic |
| ENSOANG00000043902 | ENSOANG00000043902 | O_anatinus | Animals | mono_intergenic | H/ACA | | | intergenic |
| ENSOANG00000043906 | ENSOANG00000043906 | O_anatinus | Animals | intronic_cluster | H/ACA | ENSOANG00000010461 | CRTAC1 | protein_coding |
| ENSOANG00000043907 | ENSOANG00000043907 | O_anatinus | Animals | mono_intergenic | H/ACA | | | intergenic |

| | | | | | | | | |
|---|---|---|---|---|---|---|---|---|
| ENSOANG00000043909 | ENSOANG00000043909 | O_anatinus | Animals | mono_intergenic | H/ACA | | | intergenic |
| ENSOANG00000043911 | ENSOANG00000043911 | O_anatinus | Animals | mono_intergenic | H/ACA | | | intergenic |
| ENSOANG00000043913 | ENSOANG00000043913 | O_anatinus | Animals | mono_intergenic | H/ACA | | | intergenic |
| ENSOANG00000043916 | ENSOANG00000043916 | O_anatinus | Animals | mono_intergenic | H/ACA | | | intergenic |
| ENSOANG00000043917 | ENSOANG00000043917 | O_anatinus | Animals | mono_intergenic | H/ACA | | | intergenic |
| ENSOANG00000043918 | ENSOANG00000043918 | O_anatinus | Animals | mono_intergenic | H/ACA | | | intergenic |
| ENSOANG00000043919 | ENSOANG00000043919 | O_anatinus | Animals | mono_intergenic | H/ACA | | | intergenic |
| ENSOANG00000043921 | ENSOANG00000043921 | O_anatinus | Animals | mono_intergenic | H/ACA | | | intergenic |
| ENSOANG00000043922 | ENSOANG00000043922 | O_anatinus | Animals | mono_intronic | H/ACA | ENSOANG00000010614 | TSHR | protein_coding |
| ENSOANG00000043923 | ENSOANG00000043923 | O_anatinus | Animals | mono_intergenic | H/ACA | | | intergenic |
| ENSOANG00000043925 | ENSOANG00000043925 | O_anatinus | Animals | mono_intergenic | H/ACA | | | intergenic |
| ENSOANG00000043926 | ENSOANG00000043926 | O_anatinus | Animals | mono_intergenic | H/ACA | | | intergenic |
| ENSOANG00000043929 | ENSOANG00000043929 | O_anatinus | Animals | mono_intergenic | H/ACA | | | intergenic |
| ENSOANG00000043930 | ENSOANG00000043930 | O_anatinus | Animals | mono_intronic | H/ACA | ENSOANG00000006983 | ARSK | protein_coding |
| ENSOANG00000043933 | ENSOANG00000043933 | O_anatinus | Animals | mono_intronic | H/ACA | ENSOANG00000009108 | EEFSEC | protein_coding |
| ENSOANG00000043934 | ENSOANG00000043934 | O_anatinus | Animals | mono_intergenic | H/ACA | | | intergenic |
| ENSOANG00000043935 | ENSOANG00000043935 | O_anatinus | Animals | mono_intergenic | H/ACA | | | intergenic |
| ENSOANG00000043936 | ENSOANG00000043936 | O_anatinus | Animals | mono_intergenic | H/ACA | | | intergenic |
| ENSOANG00000043943 | ENSOANG00000043943 | O_anatinus | Animals | mono_intergenic | H/ACA | | | intergenic |
| ENSOANG00000043944 | ENSOANG00000043944 | O_anatinus | Animals | mono_intronic | H/ACA | ENSOANG00000050684 | | protein_coding |
| ENSOANG00000043945 | ENSOANG00000043945 | O_anatinus | Animals | mono_intergenic | H/ACA | | | intergenic |
| ENSOANG00000043947 | ENSOANG00000043947 | O_anatinus | Animals | mono_intergenic | H/ACA | | | intergenic |
| ENSOANG00000043951 | ENSOANG00000043951 | O_anatinus | Animals | mono_intergenic | H/ACA | | | intergenic |
| ENSOANG00000043952 | ENSOANG00000043952 | O_anatinus | Animals | mono_intergenic | H/ACA | | | intergenic |
| ENSOANG00000043953 | ENSOANG00000043953 | O_anatinus | Animals | mono_intergenic | H/ACA | | | intergenic |
| ENSOANG00000043954 | ENSOANG00000043954 | O_anatinus | Animals | mono_intergenic | H/ACA | | | intergenic |
| ENSOANG00000043955 | ENSOANG00000043955 | O_anatinus | Animals | mono_intronic | H/ACA | ENSOANG00000010413 | ADK | protein_coding |
| ENSOANG00000043956 | ENSOANG00000043956 | O_anatinus | Animals | mono_intergenic | H/ACA | | | intergenic |
| ENSOANG00000043959 | ENSOANG00000043959 | O_anatinus | Animals | mono_intergenic | H/ACA | | | intergenic |
| ENSOANG00000043960 | ENSOANG00000043960 | O_anatinus | Animals | mono_intergenic | H/ACA | | | intergenic |
| ENSOANG00000043964 | ENSOANG00000043964 | O_anatinus | Animals | mono_intergenic | H/ACA | | | intergenic |
| ENSOANG00000043966 | ENSOANG00000043966 | O_anatinus | Animals | mono_intergenic | H/ACA | | | intergenic |
| ENSOANG00000043967 | ENSOANG00000043967 | O_anatinus | Animals | mono_intergenic | H/ACA | | | intergenic |
| ENSOANG00000043968 | ENSOANG00000043968 | O_anatinus | Animals | mono_intergenic | H/ACA | | | intergenic |
| ENSOANG00000043972 | ENSOANG00000043972 | O_anatinus | Animals | mono_intergenic | H/ACA | | | intergenic |
| ENSOANG00000043976 | ENSOANG00000043976 | O_anatinus | Animals | mono_intergenic | H/ACA | | | intergenic |
| ENSOANG00000043979 | ENSOANG00000043979 | O_anatinus | Animals | mono_intergenic | H/ACA | | | intergenic |
| ENSOANG00000043980 | ENSOANG00000043980 | O_anatinus | Animals | mono_intergenic | H/ACA | | | intergenic |
| ENSOANG00000043981 | ENSOANG00000043981 | O_anatinus | Animals | mono_intergenic | H/ACA | | | intergenic |
| ENSOANG00000043984 | SNORD98 | O_anatinus | Animals | mono_intronic | C/D | ENSOANG00000015048 | CCAR1 | protein_coding |
| ENSOANG00000043989 | ENSOANG00000043989 | O_anatinus | Animals | mono_intergenic | H/ACA | | | intergenic |

| | | | | | | | | |
|---|---|---|---|---|---|---|---|---|
| ENSOANG00000043993 | ENSOANG00000043993 | O_anatinus | Animals | mono_intergenic | H/ACA | | | intergenic |
| ENSOANG00000043999 | ENSOANG00000043999 | O_anatinus | Animals | mono_intronic | H/ACA | ENSOANG00000008767 | CFTR | protein_coding |
| ENSOANG00000044009 | ENSOANG00000044009 | O_anatinus | Animals | mono_intergenic | H/ACA | | | intergenic |
| ENSOANG00000044011 | ENSOANG00000044011 | O_anatinus | Animals | mono_intergenic | H/ACA | | | intergenic |
| ENSOANG00000044017 | ENSOANG00000044017 | O_anatinus | Animals | mono_intergenic | H/ACA | | | intergenic |
| ENSOANG00000044022 | ENSOANG00000044022 | O_anatinus | Animals | mono_intergenic | H/ACA | | | intergenic |
| ENSOANG00000044023 | ENSOANG00000044023 | O_anatinus | Animals | mono_intergenic | H/ACA | | | intergenic |
| ENSOANG00000044024 | ENSOANG00000044024 | O_anatinus | Animals | mono_intergenic | H/ACA | | | intergenic |
| ENSOANG00000044029 | ENSOANG00000044029 | O_anatinus | Animals | mono_intergenic | H/ACA | | | intergenic |
| ENSOANG00000044031 | ENSOANG00000044031 | O_anatinus | Animals | mono_intergenic | H/ACA | | | intergenic |
| ENSOANG00000044032 | ENSOANG00000044032 | O_anatinus | Animals | mono_intergenic | H/ACA | | | intergenic |
| ENSOANG00000044034 | ENSOANG00000044034 | O_anatinus | Animals | mono_intergenic | H/ACA | | | intergenic |
| ENSOANG00000044035 | ENSOANG00000044035 | O_anatinus | Animals | mono_intergenic | H/ACA | | | intergenic |
| ENSOANG00000044042 | ENSOANG00000044042 | O_anatinus | Animals | mono_intergenic | H/ACA | | | intergenic |
| ENSOANG00000044046 | ENSOANG00000044046 | O_anatinus | Animals | mono_intergenic | H/ACA | | | intergenic |
| ENSOANG00000044051 | ENSOANG00000044051 | O_anatinus | Animals | mono_intergenic | H/ACA | | | intergenic |
| ENSOANG00000044053 | ENSOANG00000044053 | O_anatinus | Animals | mono_intergenic | H/ACA | | | intergenic |
| ENSOANG00000044055 | ENSOANG00000044055 | O_anatinus | Animals | mono_intronic | H/ACA | ENSOANG00000003090 | | protein_coding |
| ENSOANG00000044057 | ENSOANG00000044057 | O_anatinus | Animals | mono_intergenic | H/ACA | | | intergenic |
| ENSOANG00000044058 | ENSOANG00000044058 | O_anatinus | Animals | mono_intergenic | H/ACA | | | intergenic |
| ENSOANG00000044060 | ENSOANG00000044060 | O_anatinus | Animals | mono_intergenic | H/ACA | | | intergenic |
| ENSOANG00000044062 | ENSOANG00000044062 | O_anatinus | Animals | mono_intergenic | H/ACA | | | intergenic |
| ENSOANG00000044065 | ENSOANG00000044065 | O_anatinus | Animals | mono_intergenic | H/ACA | | | intergenic |
| ENSOANG00000044066 | ENSOANG00000044066 | O_anatinus | Animals | mono_intergenic | H/ACA | | | intergenic |
| ENSOANG00000044069 | ENSOANG00000044069 | O_anatinus | Animals | mono_intergenic | H/ACA | | | intergenic |
| ENSOANG00000044070 | ENSOANG00000044070 | O_anatinus | Animals | mono_intergenic | H/ACA | | | intergenic |
| ENSOANG00000044071 | ENSOANG00000044071 | O_anatinus | Animals | mono_intergenic | H/ACA | | | intergenic |
| ENSOANG00000044072 | ENSOANG00000044072 | O_anatinus | Animals | mono_intergenic | H/ACA | | | intergenic |
| ENSOANG00000044074 | ENSOANG00000044074 | O_anatinus | Animals | mono_intergenic | H/ACA | | | intergenic |
| ENSOANG00000044075 | ENSOANG00000044075 | O_anatinus | Animals | mono_intergenic | H/ACA | | | intergenic |
| ENSOANG00000044076 | ENSOANG00000044076 | O_anatinus | Animals | mono_intergenic | H/ACA | | | intergenic |
| ENSOANG00000044077 | ENSOANG00000044077 | O_anatinus | Animals | mono_intergenic | H/ACA | | | intergenic |
| ENSOANG00000044079 | ENSOANG00000044079 | O_anatinus | Animals | mono_intergenic | H/ACA | | | intergenic |
| ENSOANG00000044081 | ENSOANG00000044081 | O_anatinus | Animals | mono_intergenic | H/ACA | | | intergenic |
| ENSOANG00000044082 | ENSOANG00000044082 | O_anatinus | Animals | mono_intergenic | H/ACA | | | intergenic |
| ENSOANG00000044089 | ENSOANG00000044089 | O_anatinus | Animals | mono_intergenic | H/ACA | | | intergenic |
| ENSOANG00000044091 | ENSOANG00000044091 | O_anatinus | Animals | mono_intergenic | H/ACA | | | intergenic |
| ENSOANG00000044092 | ENSOANG00000044092 | O_anatinus | Animals | mono_intergenic | H/ACA | | | intergenic |
| ENSOANG00000044093 | ENSOANG00000044093 | O_anatinus | Animals | mono_intergenic | H/ACA | | | intergenic |
| ENSOANG00000044095 | ENSOANG00000044095 | O_anatinus | Animals | mono_intergenic | H/ACA | | | intergenic |
| ENSOANG00000044099 | ENSOANG00000044099 | O_anatinus | Animals | mono_intergenic | H/ACA | | | intergenic |

| | | | | | | | | |
|---|---|---|---|---|---|---|---|---|
| ENSOANG00000044100 | ENSOANG00000044100 | O_anatinus | Animals | mono_intergenic | H/ACA | | | intergenic |
| ENSOANG00000044101 | ENSOANG00000044101 | O_anatinus | Animals | mono_intergenic | H/ACA | | | intergenic |
| ENSOANG00000044102 | ENSOANG00000044102 | O_anatinus | Animals | mono_intergenic | H/ACA | | | intergenic |
| ENSOANG00000044104 | ENSOANG00000044104 | O_anatinus | Animals | mono_intergenic | H/ACA | | | intergenic |
| ENSOANG00000044107 | ENSOANG00000044107 | O_anatinus | Animals | mono_intergenic | H/ACA | | | intergenic |
| ENSOANG00000044117 | ENSOANG00000044117 | O_anatinus | Animals | mono_intronic | H/ACA | ENSOANG00000005136 | SORBS1 | protein_coding |
| ENSOANG00000044118 | ENSOANG00000044118 | O_anatinus | Animals | mono_intergenic | H/ACA | | | intergenic |
| ENSOANG00000044122 | ENSOANG00000044122 | O_anatinus | Animals | mono_intergenic | H/ACA | | | intergenic |
| ENSOANG00000044126 | ENSOANG00000044126 | O_anatinus | Animals | mono_intergenic | H/ACA | | | intergenic |
| ENSOANG00000044133 | ENSOANG00000044133 | O_anatinus | Animals | mono_intergenic | H/ACA | | | intergenic |
| ENSOANG00000044135 | ENSOANG00000044135 | O_anatinus | Animals | mono_intergenic | H/ACA | | | intergenic |
| ENSOANG00000044139 | ENSOANG00000044139 | O_anatinus | Animals | mono_intergenic | H/ACA | | | intergenic |
| ENSOANG00000044142 | ENSOANG00000044142 | O_anatinus | Animals | mono_intergenic | H/ACA | | | intergenic |
| ENSOANG00000044153 | ENSOANG00000044153 | O_anatinus | Animals | mono_intergenic | H/ACA | | | intergenic |
| ENSOANG00000044156 | ENSOANG00000044156 | O_anatinus | Animals | mono_intergenic | H/ACA | | | intergenic |
| ENSOANG00000044160 | ENSOANG00000044160 | O_anatinus | Animals | mono_intergenic | H/ACA | | | intergenic |
| ENSOANG00000044161 | ENSOANG00000044161 | O_anatinus | Animals | mono_intergenic | H/ACA | | | intergenic |
| ENSOANG00000044162 | ENSOANG00000044162 | O_anatinus | Animals | mono_intergenic | H/ACA | | | intergenic |
| ENSOANG00000044165 | ENSOANG00000044165 | O_anatinus | Animals | mono_intergenic | H/ACA | | | intergenic |
| ENSOANG00000044166 | ENSOANG00000044166 | O_anatinus | Animals | mono_intronic | H/ACA | ENSOANG00000041655 | PEX5L | protein_coding |
| ENSOANG00000044167 | ENSOANG00000044167 | O_anatinus | Animals | intronic_cluster | H/ACA | ENSOANG00000005417 | | protein_coding |
| ENSOANG00000044168 | ENSOANG00000044168 | O_anatinus | Animals | mono_intergenic | H/ACA | | | intergenic |
| ENSOANG00000044169 | ENSOANG00000044169 | O_anatinus | Animals | mono_intergenic | H/ACA | | | intergenic |
| ENSOANG00000044170 | ENSOANG00000044170 | O_anatinus | Animals | mono_intergenic | H/ACA | | | intergenic |
| ENSOANG00000044174 | ENSOANG00000044174 | O_anatinus | Animals | mono_intergenic | H/ACA | | | intergenic |
| ENSOANG00000044177 | ENSOANG00000044177 | O_anatinus | Animals | mono_intergenic | H/ACA | | | intergenic |
| ENSOANG00000044178 | ENSOANG00000044178 | O_anatinus | Animals | mono_intergenic | H/ACA | | | intergenic |
| ENSOANG00000044180 | ENSOANG00000044180 | O_anatinus | Animals | mono_intergenic | H/ACA | | | intergenic |
| ENSOANG00000044181 | ENSOANG00000044181 | O_anatinus | Animals | mono_intergenic | H/ACA | | | intergenic |
| ENSOANG00000044183 | ENSOANG00000044183 | O_anatinus | Animals | mono_intergenic | H/ACA | | | intergenic |
| ENSOANG00000044184 | ENSOANG00000044184 | O_anatinus | Animals | mono_intergenic | H/ACA | | | intergenic |
| ENSOANG00000044187 | ENSOANG00000044187 | O_anatinus | Animals | mono_intergenic | H/ACA | | | intergenic |
| ENSOANG00000044190 | ENSOANG00000044190 | O_anatinus | Animals | mono_intergenic | H/ACA | | | intergenic |
| ENSOANG00000044191 | ENSOANG00000044191 | O_anatinus | Animals | mono_intronic | H/ACA | ENSOANG00000002313 | SYNPO | protein_coding |
| ENSOANG00000044195 | ENSOANG00000044195 | O_anatinus | Animals | mono_intergenic | H/ACA | | | intergenic |
| ENSOANG00000044201 | ENSOANG00000044201 | O_anatinus | Animals | mono_intergenic | H/ACA | | | intergenic |
| ENSOANG00000044203 | ENSOANG00000044203 | O_anatinus | Animals | mono_intronic | H/ACA | ENSOANG00000049145 | NAALADL2 | protein_coding |
| ENSOANG00000044204 | ENSOANG00000044204 | O_anatinus | Animals | mono_intronic | H/ACA | ENSOANG00000046623 | FADS1 | protein_coding |
| ENSOANG00000044207 | ENSOANG00000044207 | O_anatinus | Animals | mono_intergenic | H/ACA | | | intergenic |
| ENSOANG00000044208 | ENSOANG00000044208 | O_anatinus | Animals | mono_intergenic | H/ACA | | | intergenic |
| ENSOANG00000044209 | SCARNA10 | O_anatinus | Animals | mono_intronic | C/D | ENSOANG00000003013 | NCAPD2 | protein_coding |

| | | | | | | | | |
|---|---|---|---|---|---|---|---|---|
| ENSOANG00000044210 | ENSOANG00000044210 | O_anatinus | Animals | mono_intergenic | H/ACA | | | intergenic |
| ENSOANG00000044214 | ENSOANG00000044214 | O_anatinus | Animals | mono_intergenic | H/ACA | | | intergenic |
| ENSOANG00000044216 | ENSOANG00000044216 | O_anatinus | Animals | mono_intergenic | H/ACA | | | intergenic |
| ENSOANG00000044218 | ENSOANG00000044218 | O_anatinus | Animals | mono_intergenic | H/ACA | | | intergenic |
| ENSOANG00000044219 | ENSOANG00000044219 | O_anatinus | Animals | mono_intergenic | H/ACA | | | intergenic |
| ENSOANG00000044222 | ENSOANG00000044222 | O_anatinus | Animals | mono_intergenic | H/ACA | | | intergenic |
| ENSOANG00000044230 | ENSOANG00000044230 | O_anatinus | Animals | mono_intergenic | H/ACA | | | intergenic |
| ENSOANG00000044233 | ENSOANG00000044233 | O_anatinus | Animals | mono_intergenic | H/ACA | | | intergenic |
| ENSOANG00000044235 | ENSOANG00000044235 | O_anatinus | Animals | mono_intergenic | H/ACA | | | intergenic |
| ENSOANG00000044240 | ENSOANG00000044240 | O_anatinus | Animals | mono_intergenic | H/ACA | | | intergenic |
| ENSOANG00000044241 | ENSOANG00000044241 | O_anatinus | Animals | mono_intergenic | H/ACA | | | intergenic |
| ENSOANG00000044243 | ENSOANG00000044243 | O_anatinus | Animals | mono_intergenic | H/ACA | | | intergenic |
| ENSOANG00000044244 | ENSOANG00000044244 | O_anatinus | Animals | mono_intergenic | H/ACA | | | intergenic |
| ENSOANG00000044245 | ENSOANG00000044245 | O_anatinus | Animals | mono_intergenic | H/ACA | | | intergenic |
| ENSOANG00000044248 | ENSOANG00000044248 | O_anatinus | Animals | mono_intergenic | H/ACA | | | intergenic |
| ENSOANG00000044249 | ENSOANG00000044249 | O_anatinus | Animals | mono_intergenic | H/ACA | | | intergenic |
| ENSOANG00000044250 | ENSOANG00000044250 | O_anatinus | Animals | mono_intergenic | H/ACA | | | intergenic |
| ENSOANG00000044251 | ENSOANG00000044251 | O_anatinus | Animals | mono_intergenic | H/ACA | | | intergenic |
| ENSOANG00000044252 | ENSOANG00000044252 | O_anatinus | Animals | mono_intergenic | H/ACA | | | intergenic |
| ENSOANG00000044253 | ENSOANG00000044253 | O_anatinus | Animals | mono_intergenic | H/ACA | | | intergenic |
| ENSOANG00000044258 | ENSOANG00000044258 | O_anatinus | Animals | mono_intergenic | H/ACA | | | intergenic |
| ENSOANG00000044259 | ENSOANG00000044259 | O_anatinus | Animals | mono_intergenic | H/ACA | | | intergenic |
| ENSOANG00000044263 | ENSOANG00000044263 | O_anatinus | Animals | mono_intergenic | H/ACA | | | intergenic |
| ENSOANG00000044264 | ENSOANG00000044264 | O_anatinus | Animals | mono_intergenic | H/ACA | | | intergenic |
| ENSOANG00000044265 | ENSOANG00000044265 | O_anatinus | Animals | mono_intergenic | H/ACA | | | intergenic |
| ENSOANG00000044266 | ENSOANG00000044266 | O_anatinus | Animals | mono_intergenic | H/ACA | | | intergenic |
| ENSOANG00000044267 | ENSOANG00000044267 | O_anatinus | Animals | mono_intergenic | H/ACA | | | intergenic |
| ENSOANG00000044268 | ENSOANG00000044268 | O_anatinus | Animals | mono_intergenic | H/ACA | | | intergenic |
| ENSOANG00000044269 | ENSOANG00000044269 | O_anatinus | Animals | mono_intronic | H/ACA | ENSOANG00000006018 | KLHL1 | protein_coding |
| ENSOANG00000044270 | ENSOANG00000044270 | O_anatinus | Animals | mono_intronic | H/ACA | ENSOANG00000045084 | GNAI2 | protein_coding |
| ENSOANG00000044272 | ENSOANG00000044272 | O_anatinus | Animals | mono_intergenic | H/ACA | | | intergenic |
| ENSOANG00000044273 | ENSOANG00000044273 | O_anatinus | Animals | mono_intergenic | H/ACA | | | intergenic |
| ENSOANG00000044274 | ENSOANG00000044274 | O_anatinus | Animals | mono_intergenic | H/ACA | | | intergenic |
| ENSOANG00000044280 | ENSOANG00000044280 | O_anatinus | Animals | mono_intergenic | H/ACA | | | intergenic |
| ENSOANG00000044281 | ENSOANG00000044281 | O_anatinus | Animals | mono_intronic | H/ACA | ENSOANG00000013403 | FSTL4 | protein_coding |
| ENSOANG00000044282 | ENSOANG00000044282 | O_anatinus | Animals | mono_intronic | H/ACA | ENSOANG00000006102 | | protein_coding |
| ENSOANG00000044284 | ENSOANG00000044284 | O_anatinus | Animals | mono_intergenic | H/ACA | | | intergenic |
| ENSOANG00000044286 | ENSOANG00000044286 | O_anatinus | Animals | mono_intronic | H/ACA | ENSOANG00000007585 | MECOM | protein_coding |
| ENSOANG00000044287 | ENSOANG00000044287 | O_anatinus | Animals | mono_intergenic | H/ACA | | | intergenic |
| ENSOANG00000044289 | ENSOANG00000044289 | O_anatinus | Animals | mono_intergenic | H/ACA | | | intergenic |
| ENSOANG00000044292 | ENSOANG00000044292 | O_anatinus | Animals | mono_intergenic | H/ACA | | | intergenic |

| | | | | | | | | |
|---|---|---|---|---|---|---|---|---|
| ENSOANG00000044294 | ENSOANG00000044294 | O_anatinus | Animals | mono_intergenic | H/ACA | | | intergenic |
| ENSOANG00000044296 | ENSOANG00000044296 | O_anatinus | Animals | mono_intergenic | H/ACA | | | intergenic |
| ENSOANG00000044298 | ENSOANG00000044298 | O_anatinus | Animals | mono_intergenic | H/ACA | | | intergenic |
| ENSOANG00000044299 | ENSOANG00000044299 | O_anatinus | Animals | mono_intergenic | H/ACA | | | intergenic |
| ENSOANG00000044301 | ENSOANG00000044301 | O_anatinus | Animals | mono_intergenic | H/ACA | | | intergenic |
| ENSOANG00000044310 | ENSOANG00000044310 | O_anatinus | Animals | mono_intronic | H/ACA | ENSOANG00000000786 | KLC1 | protein_coding |
| ENSOANG00000044315 | ENSOANG00000044315 | O_anatinus | Animals | mono_intergenic | H/ACA | | | intergenic |
| ENSOANG00000044317 | ENSOANG00000044317 | O_anatinus | Animals | mono_intergenic | H/ACA | | | intergenic |
| ENSOANG00000044320 | ENSOANG00000044320 | O_anatinus | Animals | mono_intergenic | H/ACA | | | intergenic |
| ENSOANG00000044321 | ENSOANG00000044321 | O_anatinus | Animals | mono_intergenic | H/ACA | | | intergenic |
| ENSOANG00000044324 | ENSOANG00000044324 | O_anatinus | Animals | mono_intergenic | H/ACA | | | intergenic |
| ENSOANG00000044325 | ENSOANG00000044325 | O_anatinus | Animals | mono_intergenic | H/ACA | | | intergenic |
| ENSOANG00000044326 | ENSOANG00000044326 | O_anatinus | Animals | mono_intergenic | H/ACA | | | intergenic |
| ENSOANG00000044328 | ENSOANG00000044328 | O_anatinus | Animals | mono_intergenic | H/ACA | | | intergenic |
| ENSOANG00000044329 | ENSOANG00000044329 | O_anatinus | Animals | mono_intergenic | H/ACA | | | intergenic |
| ENSOANG00000044330 | ENSOANG00000044330 | O_anatinus | Animals | mono_intergenic | H/ACA | | | intergenic |
| ENSOANG00000044332 | ENSOANG00000044332 | O_anatinus | Animals | mono_intergenic | H/ACA | | | intergenic |
| ENSOANG00000044333 | ENSOANG00000044333 | O_anatinus | Animals | mono_intergenic | H/ACA | | | intergenic |
| ENSOANG00000044339 | ENSOANG00000044339 | O_anatinus | Animals | mono_intergenic | H/ACA | | | intergenic |
| ENSOANG00000044341 | ENSOANG00000044341 | O_anatinus | Animals | mono_intergenic | H/ACA | | | intergenic |
| ENSOANG00000044343 | ENSOANG00000044343 | O_anatinus | Animals | mono_intergenic | H/ACA | | | intergenic |
| ENSOANG00000044344 | ENSOANG00000044344 | O_anatinus | Animals | mono_intergenic | H/ACA | | | intergenic |
| ENSOANG00000044346 | ENSOANG00000044346 | O_anatinus | Animals | mono_intergenic | H/ACA | | | intergenic |
| ENSOANG00000044347 | ENSOANG00000044347 | O_anatinus | Animals | mono_intergenic | H/ACA | | | intergenic |
| ENSOANG00000044348 | ENSOANG00000044348 | O_anatinus | Animals | mono_intronic | H/ACA | ENSOANG00000013116 | CNTNAP5 | protein_coding |
| ENSOANG00000044350 | ENSOANG00000044350 | O_anatinus | Animals | mono_intergenic | H/ACA | | | intergenic |
| ENSOANG00000044352 | ENSOANG00000044352 | O_anatinus | Animals | mono_intergenic | H/ACA | | | intergenic |
| ENSOANG00000044354 | ENSOANG00000044354 | O_anatinus | Animals | mono_intronic | H/ACA | ENSOANG00000004962 | ADAMTS20 | protein_coding |
| ENSOANG00000044358 | ENSOANG00000044358 | O_anatinus | Animals | mono_intergenic | H/ACA | | | intergenic |
| ENSOANG00000044360 | ENSOANG00000044360 | O_anatinus | Animals | mono_intergenic | H/ACA | | | intergenic |
| ENSOANG00000044364 | ENSOANG00000044364 | O_anatinus | Animals | mono_intergenic | H/ACA | | | intergenic |
| ENSOANG00000044365 | ENSOANG00000044365 | O_anatinus | Animals | mono_intergenic | H/ACA | | | intergenic |
| ENSOANG00000044367 | SNORD15 | O_anatinus | Animals | mono_intronic | C/D | ENSOANG00000044114 | | protein_coding |
| ENSOANG00000044370 | ENSOANG00000044370 | O_anatinus | Animals | mono_intergenic | H/ACA | | | intergenic |
| ENSOANG00000044372 | ENSOANG00000044372 | O_anatinus | Animals | mono_intergenic | H/ACA | | | intergenic |
| ENSOANG00000044376 | ENSOANG00000044376 | O_anatinus | Animals | mono_intergenic | H/ACA | | | intergenic |
| ENSOANG00000044377 | ENSOANG00000044377 | O_anatinus | Animals | mono_intronic | H/ACA | ENSOANG00000006437 | | protein_coding |
| ENSOANG00000044380 | ENSOANG00000044380 | O_anatinus | Animals | mono_intergenic | H/ACA | | | intergenic |
| ENSOANG00000044384 | ENSOANG00000044384 | O_anatinus | Animals | mono_intergenic | H/ACA | | | intergenic |
| ENSOANG00000044385 | ENSOANG00000044385 | O_anatinus | Animals | mono_intergenic | H/ACA | | | intergenic |
| ENSOANG00000044386 | ENSOANG00000044386 | O_anatinus | Animals | mono_intergenic | H/ACA | | | intergenic |

| | | | | | | | | |
|---|---|---|---|---|---|---|---|---|
| ENSOANG00000044388 | ENSOANG00000044388 | O_anatinus | Animals | mono_intergenic | H/ACA | | | intergenic |
| ENSOANG00000044392 | ENSOANG00000044392 | O_anatinus | Animals | mono_intergenic | H/ACA | | | intergenic |
| ENSOANG00000044393 | ENSOANG00000044393 | O_anatinus | Animals | mono_intergenic | H/ACA | | | intergenic |
| ENSOANG00000044395 | ENSOANG00000044395 | O_anatinus | Animals | mono_intergenic | H/ACA | | | intergenic |
| ENSOANG00000044397 | ENSOANG00000044397 | O_anatinus | Animals | mono_intergenic | H/ACA | | | intergenic |
| ENSOANG00000044399 | ENSOANG00000044399 | O_anatinus | Animals | mono_intergenic | H/ACA | | | intergenic |
| ENSOANG00000044401 | ENSOANG00000044401 | O_anatinus | Animals | mono_intergenic | H/ACA | | | intergenic |
| ENSOANG00000044404 | ENSOANG00000044404 | O_anatinus | Animals | mono_intergenic | H/ACA | | | intergenic |
| ENSOANG00000044407 | ENSOANG00000044407 | O_anatinus | Animals | mono_intergenic | H/ACA | | | intergenic |
| ENSOANG00000044408 | ENSOANG00000044408 | O_anatinus | Animals | mono_intergenic | H/ACA | | | intergenic |
| ENSOANG00000044409 | ENSOANG00000044409 | O_anatinus | Animals | mono_intergenic | H/ACA | | | intergenic |
| ENSOANG00000044410 | ENSOANG00000044410 | O_anatinus | Animals | mono_intergenic | H/ACA | | | intergenic |
| ENSOANG00000044413 | ENSOANG00000044413 | O_anatinus | Animals | mono_intergenic | H/ACA | | | intergenic |
| ENSOANG00000044414 | ENSOANG00000044414 | O_anatinus | Animals | mono_intergenic | H/ACA | | | intergenic |
| ENSOANG00000044416 | ENSOANG00000044416 | O_anatinus | Animals | mono_intergenic | H/ACA | | | intergenic |
| ENSOANG00000044419 | ENSOANG00000044419 | O_anatinus | Animals | mono_intergenic | H/ACA | | | intergenic |
| ENSOANG00000044427 | ENSOANG00000044427 | O_anatinus | Animals | mono_intergenic | H/ACA | | | intergenic |
| ENSOANG00000044428 | ENSOANG00000044428 | O_anatinus | Animals | mono_intergenic | H/ACA | | | intergenic |
| ENSOANG00000044429 | ENSOANG00000044429 | O_anatinus | Animals | mono_intergenic | H/ACA | | | intergenic |
| ENSOANG00000044434 | ENSOANG00000044434 | O_anatinus | Animals | mono_intergenic | H/ACA | | | intergenic |
| ENSOANG00000044442 | ENSOANG00000044442 | O_anatinus | Animals | mono_intergenic | H/ACA | | | intergenic |
| ENSOANG00000044443 | ENSOANG00000044443 | O_anatinus | Animals | mono_intergenic | H/ACA | | | intergenic |
| ENSOANG00000044449 | ENSOANG00000044449 | O_anatinus | Animals | mono_intergenic | H/ACA | | | intergenic |
| ENSOANG00000044450 | ENSOANG00000044450 | O_anatinus | Animals | mono_intergenic | H/ACA | | | intergenic |
| ENSOANG00000044455 | ENSOANG00000044455 | O_anatinus | Animals | mono_intergenic | H/ACA | | | intergenic |
| ENSOANG00000044456 | ENSOANG00000044456 | O_anatinus | Animals | mono_intergenic | H/ACA | | | intergenic |
| ENSOANG00000044460 | ENSOANG00000044460 | O_anatinus | Animals | mono_intergenic | H/ACA | | | intergenic |
| ENSOANG00000044464 | ENSOANG00000044464 | O_anatinus | Animals | mono_intergenic | H/ACA | | | intergenic |
| ENSOANG00000044465 | ENSOANG00000044465 | O_anatinus | Animals | mono_intergenic | H/ACA | | | intergenic |
| ENSOANG00000044466 | ENSOANG00000044466 | O_anatinus | Animals | mono_intergenic | H/ACA | | | intergenic |
| ENSOANG00000044474 | ENSOANG00000044474 | O_anatinus | Animals | mono_intergenic | H/ACA | | | intergenic |
| ENSOANG00000044478 | ENSOANG00000044478 | O_anatinus | Animals | mono_intergenic | H/ACA | | | intergenic |
| ENSOANG00000044483 | ENSOANG00000044483 | O_anatinus | Animals | mono_intergenic | H/ACA | | | intergenic |
| ENSOANG00000044484 | ENSOANG00000044484 | O_anatinus | Animals | mono_intergenic | H/ACA | | | intergenic |
| ENSOANG00000044485 | ENSOANG00000044485 | O_anatinus | Animals | mono_intronic | H/ACA | ENSOANG00000014549 | TENM1 | protein_coding |
| ENSOANG00000044495 | ENSOANG00000044495 | O_anatinus | Animals | mono_intergenic | H/ACA | | | intergenic |
| ENSOANG00000044496 | ENSOANG00000044496 | O_anatinus | Animals | mono_intergenic | H/ACA | | | intergenic |
| ENSOANG00000044497 | ENSOANG00000044497 | O_anatinus | Animals | mono_intergenic | H/ACA | | | intergenic |
| ENSOANG00000044498 | ENSOANG00000044498 | O_anatinus | Animals | mono_intergenic | H/ACA | | | intergenic |
| ENSOANG00000044499 | ENSOANG00000044499 | O_anatinus | Animals | mono_intronic | H/ACA | ENSOANG00000003170 | RASGEF1A | protein_coding |
| ENSOANG00000044502 | ENSOANG00000044502 | O_anatinus | Animals | mono_intergenic | H/ACA | | | intergenic |

| | | | | | | | | |
|---|---|---|---|---|---|---|---|---|
| ENSOANG00000044505 | ENSOANG00000044505 | O_anatinus | Animals | mono_intergenic | H/ACA | | | intergenic |
| ENSOANG00000044508 | ENSOANG00000044508 | O_anatinus | Animals | mono_intergenic | H/ACA | | | intergenic |
| ENSOANG00000044509 | ENSOANG00000044509 | O_anatinus | Animals | mono_intergenic | H/ACA | | | intergenic |
| ENSOANG00000044511 | ENSOANG00000044511 | O_anatinus | Animals | mono_intergenic | H/ACA | | | intergenic |
| ENSOANG00000044515 | ENSOANG00000044515 | O_anatinus | Animals | mono_intergenic | H/ACA | | | intergenic |
| ENSOANG00000044516 | ENSOANG00000044516 | O_anatinus | Animals | mono_intergenic | H/ACA | | | intergenic |
| ENSOANG00000044522 | ENSOANG00000044522 | O_anatinus | Animals | mono_intergenic | H/ACA | | | intergenic |
| ENSOANG00000044525 | ENSOANG00000044525 | O_anatinus | Animals | mono_intergenic | H/ACA | | | intergenic |
| ENSOANG00000044530 | ENSOANG00000044530 | O_anatinus | Animals | mono_intergenic | H/ACA | | | intergenic |
| ENSOANG00000044531 | ENSOANG00000044531 | O_anatinus | Animals | mono_intergenic | H/ACA | | | intergenic |
| ENSOANG00000044533 | ENSOANG00000044533 | O_anatinus | Animals | mono_intergenic | H/ACA | | | intergenic |
| ENSOANG00000044534 | ENSOANG00000044534 | O_anatinus | Animals | mono_intergenic | H/ACA | | | intergenic |
| ENSOANG00000044538 | ENSOANG00000044538 | O_anatinus | Animals | mono_intergenic | H/ACA | | | intergenic |
| ENSOANG00000044542 | ENSOANG00000044542 | O_anatinus | Animals | mono_intergenic | H/ACA | | | intergenic |
| ENSOANG00000044546 | ENSOANG00000044546 | O_anatinus | Animals | mono_intergenic | H/ACA | | | intergenic |
| ENSOANG00000044547 | ENSOANG00000044547 | O_anatinus | Animals | mono_intergenic | H/ACA | | | intergenic |
| ENSOANG00000044548 | ENSOANG00000044548 | O_anatinus | Animals | mono_intergenic | H/ACA | | | intergenic |
| ENSOANG00000044552 | ENSOANG00000044552 | O_anatinus | Animals | mono_intergenic | H/ACA | | | intergenic |
| ENSOANG00000044554 | ENSOANG00000044554 | O_anatinus | Animals | mono_intergenic | H/ACA | | | intergenic |
| ENSOANG00000044555 | ENSOANG00000044555 | O_anatinus | Animals | mono_intergenic | H/ACA | | | intergenic |
| ENSOANG00000044558 | ENSOANG00000044558 | O_anatinus | Animals | mono_intergenic | H/ACA | | | intergenic |
| ENSOANG00000044562 | ENSOANG00000044562 | O_anatinus | Animals | mono_intergenic | H/ACA | | | intergenic |
| ENSOANG00000044567 | ENSOANG00000044567 | O_anatinus | Animals | mono_intergenic | H/ACA | | | intergenic |
| ENSOANG00000044571 | ENSOANG00000044571 | O_anatinus | Animals | mono_intergenic | H/ACA | | | intergenic |
| ENSOANG00000044575 | ENSOANG00000044575 | O_anatinus | Animals | mono_intergenic | H/ACA | | | intergenic |
| ENSOANG00000044576 | ENSOANG00000044576 | O_anatinus | Animals | mono_intergenic | H/ACA | | | intergenic |
| ENSOANG00000044577 | ENSOANG00000044577 | O_anatinus | Animals | mono_intergenic | H/ACA | | | intergenic |
| ENSOANG00000044579 | ENSOANG00000044579 | O_anatinus | Animals | mono_intronic | H/ACA | ENSOANG00000002667 | GREB1 | protein_coding |
| ENSOANG00000044580 | ENSOANG00000044580 | O_anatinus | Animals | mono_intergenic | H/ACA | | | intergenic |
| ENSOANG00000044582 | ENSOANG00000044582 | O_anatinus | Animals | mono_intergenic | H/ACA | | | intergenic |
| ENSOANG00000044587 | ENSOANG00000044587 | O_anatinus | Animals | mono_intergenic | H/ACA | | | intergenic |
| ENSOANG00000044589 | ENSOANG00000044589 | O_anatinus | Animals | mono_intergenic | H/ACA | | | intergenic |
| ENSOANG00000044596 | ENSOANG00000044596 | O_anatinus | Animals | mono_intergenic | H/ACA | | | intergenic |
| ENSOANG00000044601 | ENSOANG00000044601 | O_anatinus | Animals | mono_intergenic | H/ACA | | | intergenic |
| ENSOANG00000044602 | ENSOANG00000044602 | O_anatinus | Animals | mono_intergenic | H/ACA | | | intergenic |
| ENSOANG00000044610 | ENSOANG00000044610 | O_anatinus | Animals | mono_intergenic | H/ACA | | | intergenic |
| ENSOANG00000044615 | ENSOANG00000044615 | O_anatinus | Animals | mono_intronic | H/ACA | ENSOANG00000050040 | SLC25A36 | protein_coding |
| ENSOANG00000044616 | ENSOANG00000044616 | O_anatinus | Animals | mono_intergenic | H/ACA | | | intergenic |
| ENSOANG00000044617 | ENSOANG00000044617 | O_anatinus | Animals | mono_intergenic | H/ACA | | | intergenic |
| ENSOANG00000044620 | ENSOANG00000044620 | O_anatinus | Animals | mono_intergenic | H/ACA | | | intergenic |
| ENSOANG00000044621 | ENSOANG00000044621 | O_anatinus | Animals | mono_intergenic | H/ACA | | | intergenic |

| | | | | | | | | |
|---|---|---|---|---|---|---|---|---|
| ENSOANG00000044624 | ENSOANG00000044624 | O_anatinus | Animals | mono_intergenic | H/ACA | | | intergenic |
| ENSOANG00000044626 | ENSOANG00000044626 | O_anatinus | Animals | mono_intergenic | H/ACA | | | intergenic |
| ENSOANG00000044629 | ENSOANG00000044629 | O_anatinus | Animals | mono_intergenic | H/ACA | | | intergenic |
| ENSOANG00000044630 | ENSOANG00000044630 | O_anatinus | Animals | mono_intergenic | H/ACA | | | intergenic |
| ENSOANG00000044633 | ENSOANG00000044633 | O_anatinus | Animals | mono_intergenic | H/ACA | | | intergenic |
| ENSOANG00000044634 | ENSOANG00000044634 | O_anatinus | Animals | mono_intergenic | H/ACA | | | intergenic |
| ENSOANG00000044637 | ENSOANG00000044637 | O_anatinus | Animals | mono_intergenic | H/ACA | | | intergenic |
| ENSOANG00000044639 | ENSOANG00000044639 | O_anatinus | Animals | mono_intergenic | H/ACA | | | intergenic |
| ENSOANG00000044644 | ENSOANG00000044644 | O_anatinus | Animals | mono_intergenic | H/ACA | | | intergenic |
| ENSOANG00000044646 | ENSOANG00000044646 | O_anatinus | Animals | mono_intergenic | H/ACA | | | intergenic |
| ENSOANG00000044647 | ENSOANG00000044647 | O_anatinus | Animals | mono_intronic | H/ACA | ENSOANG00000014947 | CCSER1 | protein_coding |
| ENSOANG00000044649 | ENSOANG00000044649 | O_anatinus | Animals | mono_intergenic | H/ACA | | | intergenic |
| ENSOANG00000044658 | ENSOANG00000044658 | O_anatinus | Animals | mono_intergenic | H/ACA | | | intergenic |
| ENSOANG00000044659 | ENSOANG00000044659 | O_anatinus | Animals | mono_intergenic | H/ACA | | | intergenic |
| ENSOANG00000044662 | ENSOANG00000044662 | O_anatinus | Animals | mono_intronic | H/ACA | ENSOANG00000044188 | RABGAP1 | protein_coding |
| ENSOANG00000044664 | ENSOANG00000044664 | O_anatinus | Animals | mono_intergenic | H/ACA | | | intergenic |
| ENSOANG00000044671 | ENSOANG00000044671 | O_anatinus | Animals | mono_intergenic | H/ACA | | | intergenic |
| ENSOANG00000044673 | ENSOANG00000044673 | O_anatinus | Animals | mono_intergenic | H/ACA | | | intergenic |
| ENSOANG00000044674 | ENSOANG00000044674 | O_anatinus | Animals | mono_intergenic | H/ACA | | | intergenic |
| ENSOANG00000044676 | ENSOANG00000044676 | O_anatinus | Animals | mono_intronic | C/D | ENSOANG00000001161 | SF3B3 | protein_coding |
| ENSOANG00000044677 | ENSOANG00000044677 | O_anatinus | Animals | mono_intergenic | H/ACA | | | intergenic |
| ENSOANG00000044681 | ENSOANG00000044681 | O_anatinus | Animals | mono_intergenic | H/ACA | | | intergenic |
| ENSOANG00000044686 | ENSOANG00000044686 | O_anatinus | Animals | mono_intergenic | H/ACA | | | intergenic |
| ENSOANG00000044688 | ENSOANG00000044688 | O_anatinus | Animals | mono_intergenic | H/ACA | | | intergenic |
| ENSOANG00000044690 | ENSOANG00000044690 | O_anatinus | Animals | mono_intergenic | H/ACA | | | intergenic |
| ENSOANG00000044691 | ENSOANG00000044691 | O_anatinus | Animals | mono_intronic | H/ACA | ENSOANG00000001886 | RPS12 | protein_coding |
| ENSOANG00000044693 | ENSOANG00000044693 | O_anatinus | Animals | mono_intergenic | H/ACA | | | intergenic |
| ENSOANG00000044694 | ENSOANG00000044694 | O_anatinus | Animals | mono_intronic | H/ACA | ENSOANG00000013095 | SGK1 | protein_coding |
| ENSOANG00000044695 | ENSOANG00000044695 | O_anatinus | Animals | mono_intronic | H/ACA | ENSOANG00000038563 | | non_coding |
| ENSOANG00000044703 | ENSOANG00000044703 | O_anatinus | Animals | mono_intergenic | H/ACA | | | intergenic |
| ENSOANG00000044707 | ENSOANG00000044707 | O_anatinus | Animals | mono_intergenic | H/ACA | | | intergenic |
| ENSOANG00000044711 | ENSOANG00000044711 | O_anatinus | Animals | mono_intergenic | H/ACA | | | intergenic |
| ENSOANG00000044714 | ENSOANG00000044714 | O_anatinus | Animals | mono_intergenic | H/ACA | | | intergenic |
| ENSOANG00000044715 | ENSOANG00000044715 | O_anatinus | Animals | mono_intronic | H/ACA | ENSOANG00000046180 | RET | protein_coding |
| ENSOANG00000044721 | ENSOANG00000044721 | O_anatinus | Animals | mono_intergenic | H/ACA | | | intergenic |
| ENSOANG00000044722 | ENSOANG00000044722 | O_anatinus | Animals | mono_intergenic | H/ACA | | | intergenic |
| ENSOANG00000044728 | ENSOANG00000044728 | O_anatinus | Animals | mono_intergenic | H/ACA | | | intergenic |
| ENSOANG00000044734 | ENSOANG00000044734 | O_anatinus | Animals | mono_intergenic | H/ACA | | | intergenic |
| ENSOANG00000044736 | ENSOANG00000044736 | O_anatinus | Animals | mono_intronic | H/ACA | ENSOANG00000021347 | SLC25A26 | protein_coding |
| ENSOANG00000044740 | ENSOANG00000044740 | O_anatinus | Animals | mono_intronic | H/ACA | ENSOANG00000044568 | WDR70 | protein_coding |
| ENSOANG00000044741 | ENSOANG00000044741 | O_anatinus | Animals | mono_intergenic | H/ACA | | | intergenic |

| | | | | | | | | |
|---|---|---|---|---|---|---|---|---|
| ENSOANG00000044744 | ENSOANG00000044744 | O_anatinus | Animals | mono_intronic | H/ACA | ENSOANG00000009682 | AGBL1 | protein_coding |
| ENSOANG00000044753 | ENSOANG00000044753 | O_anatinus | Animals | mono_intergenic | H/ACA | | | intergenic |
| ENSOANG00000044757 | ENSOANG00000044757 | O_anatinus | Animals | mono_intergenic | H/ACA | | | intergenic |
| ENSOANG00000044758 | ENSOANG00000044758 | O_anatinus | Animals | mono_intergenic | H/ACA | | | intergenic |
| ENSOANG00000044761 | ENSOANG00000044761 | O_anatinus | Animals | mono_intergenic | H/ACA | | | intergenic |
| ENSOANG00000044762 | ENSOANG00000044762 | O_anatinus | Animals | mono_intergenic | H/ACA | | | intergenic |
| ENSOANG00000044767 | ENSOANG00000044767 | O_anatinus | Animals | mono_intergenic | H/ACA | | | intergenic |
| ENSOANG00000044771 | ENSOANG00000044771 | O_anatinus | Animals | mono_intergenic | H/ACA | | | intergenic |
| ENSOANG00000044774 | ENSOANG00000044774 | O_anatinus | Animals | mono_intronic | H/ACA | ENSOANG00000006736 | ACSF3 | protein_coding |
| ENSOANG00000044778 | ENSOANG00000044778 | O_anatinus | Animals | mono_intergenic | H/ACA | | | intergenic |
| ENSOANG00000044780 | ENSOANG00000044780 | O_anatinus | Animals | mono_intronic | H/ACA | ENSOANG00000001739 | FLT4 | protein_coding |
| ENSOANG00000044781 | ENSOANG00000044781 | O_anatinus | Animals | mono_intergenic | H/ACA | | | intergenic |
| ENSOANG00000044782 | ENSOANG00000044782 | O_anatinus | Animals | mono_intergenic | H/ACA | | | intergenic |
| ENSOANG00000044783 | ENSOANG00000044783 | O_anatinus | Animals | mono_intergenic | H/ACA | | | intergenic |
| ENSOANG00000044786 | ENSOANG00000044786 | O_anatinus | Animals | mono_intergenic | H/ACA | | | intergenic |
| ENSOANG00000044789 | ENSOANG00000044789 | O_anatinus | Animals | mono_intergenic | H/ACA | | | intergenic |
| ENSOANG00000044790 | ENSOANG00000044790 | O_anatinus | Animals | mono_intergenic | H/ACA | | | intergenic |
| ENSOANG00000044791 | ENSOANG00000044791 | O_anatinus | Animals | mono_intergenic | H/ACA | | | intergenic |
| ENSOANG00000044795 | ENSOANG00000044795 | O_anatinus | Animals | mono_intergenic | H/ACA | | | intergenic |
| ENSOANG00000044797 | ENSOANG00000044797 | O_anatinus | Animals | mono_intergenic | H/ACA | | | intergenic |
| ENSOANG00000044799 | ENSOANG00000044799 | O_anatinus | Animals | intronic_cluster | H/ACA | ENSOANG00000039168 | SLC6A11 | protein_coding |
| ENSOANG00000044801 | ENSOANG00000044801 | O_anatinus | Animals | mono_intergenic | H/ACA | | | intergenic |
| ENSOANG00000044802 | ENSOANG00000044802 | O_anatinus | Animals | mono_intergenic | H/ACA | | | intergenic |
| ENSOANG00000044803 | ENSOANG00000044803 | O_anatinus | Animals | mono_intergenic | H/ACA | | | intergenic |
| ENSOANG00000044804 | ENSOANG00000044804 | O_anatinus | Animals | mono_intergenic | H/ACA | | | intergenic |
| ENSOANG00000044806 | ENSOANG00000044806 | O_anatinus | Animals | mono_intergenic | H/ACA | | | intergenic |
| ENSOANG00000044811 | ENSOANG00000044811 | O_anatinus | Animals | mono_intergenic | H/ACA | | | intergenic |
| ENSOANG00000044813 | ENSOANG00000044813 | O_anatinus | Animals | mono_intergenic | H/ACA | | | intergenic |
| ENSOANG00000044814 | ENSOANG00000044814 | O_anatinus | Animals | mono_intergenic | H/ACA | | | intergenic |
| ENSOANG00000044815 | ENSOANG00000044815 | O_anatinus | Animals | mono_intergenic | H/ACA | | | intergenic |
| ENSOANG00000044816 | ENSOANG00000044816 | O_anatinus | Animals | mono_intronic | H/ACA | ENSOANG00000013668 | | protein_coding |
| ENSOANG00000044817 | ENSOANG00000044817 | O_anatinus | Animals | mono_intergenic | H/ACA | | | intergenic |
| ENSOANG00000044818 | ENSOANG00000044818 | O_anatinus | Animals | mono_intergenic | H/ACA | | | intergenic |
| ENSOANG00000044819 | ENSOANG00000044819 | O_anatinus | Animals | mono_intergenic | H/ACA | | | intergenic |
| ENSOANG00000044826 | ENSOANG00000044826 | O_anatinus | Animals | mono_intergenic | H/ACA | | | intergenic |
| ENSOANG00000044830 | ENSOANG00000044830 | O_anatinus | Animals | mono_intergenic | H/ACA | | | intergenic |
| ENSOANG00000044831 | ENSOANG00000044831 | O_anatinus | Animals | mono_intronic | H/ACA | ENSOANG00000040340 | IMMP2L | protein_coding |
| ENSOANG00000044833 | ENSOANG00000044833 | O_anatinus | Animals | mono_intergenic | H/ACA | | | intergenic |
| ENSOANG00000044834 | ENSOANG00000044834 | O_anatinus | Animals | mono_intronic | H/ACA | ENSOANG00000040502 | TEX264 | protein_coding |
| ENSOANG00000044836 | ENSOANG00000044836 | O_anatinus | Animals | mono_intergenic | H/ACA | | | intergenic |
| ENSOANG00000044843 | ENSOANG00000044843 | O_anatinus | Animals | mono_intergenic | H/ACA | | | intergenic |

| | | | | | | | | | |
|---|---|---|---|---|---|---|---|---|---|
| ENSOANG00000044844 | ENSOANG00000044844 | O_anatinus | Animals | mono_intronic | H/ACA | ENSOANG00000046492 | CFAP61 | protein_coding |
| ENSOANG00000044849 | ENSOANG00000044849 | O_anatinus | Animals | intronic_cluster | H/ACA | ENSOANG00000032096 | | protein_coding |
| ENSOANG00000044850 | ENSOANG00000044850 | O_anatinus | Animals | mono_intergenic | H/ACA | | | intergenic |
| ENSOANG00000044851 | ENSOANG00000044851 | O_anatinus | Animals | mono_intergenic | H/ACA | | | intergenic |
| ENSOANG00000044854 | ENSOANG00000044854 | O_anatinus | Animals | mono_intergenic | H/ACA | | | intergenic |
| ENSOANG00000044859 | ENSOANG00000044859 | O_anatinus | Animals | mono_intergenic | H/ACA | | | intergenic |
| ENSOANG00000044863 | ENSOANG00000044863 | O_anatinus | Animals | mono_intergenic | H/ACA | | | intergenic |
| ENSOANG00000044866 | ENSOANG00000044866 | O_anatinus | Animals | mono_intergenic | H/ACA | | | intergenic |
| ENSOANG00000044869 | ENSOANG00000044869 | O_anatinus | Animals | mono_intergenic | H/ACA | | | intergenic |
| ENSOANG00000044871 | ENSOANG00000044871 | O_anatinus | Animals | mono_intergenic | H/ACA | | | intergenic |
| ENSOANG00000044873 | ENSOANG00000044873 | O_anatinus | Animals | mono_intergenic | H/ACA | | | intergenic |
| ENSOANG00000044877 | ENSOANG00000044877 | O_anatinus | Animals | mono_intergenic | H/ACA | | | intergenic |
| ENSOANG00000044880 | ENSOANG00000044880 | O_anatinus | Animals | mono_intergenic | H/ACA | | | intergenic |
| ENSOANG00000044885 | ENSOANG00000044885 | O_anatinus | Animals | mono_intronic | H/ACA | ENSOANG00000008700 | | protein_coding |
| ENSOANG00000044886 | ENSOANG00000044886 | O_anatinus | Animals | mono_intergenic | H/ACA | | | intergenic |
| ENSOANG00000044887 | ENSOANG00000044887 | O_anatinus | Animals | mono_intergenic | H/ACA | | | intergenic |
| ENSOANG00000044890 | ENSOANG00000044890 | O_anatinus | Animals | mono_intergenic | H/ACA | | | intergenic |
| ENSOANG00000044891 | ENSOANG00000044891 | O_anatinus | Animals | mono_intergenic | H/ACA | | | intergenic |
| ENSOANG00000044892 | ENSOANG00000044892 | O_anatinus | Animals | mono_intergenic | H/ACA | | | intergenic |
| ENSOANG00000044893 | ENSOANG00000044893 | O_anatinus | Animals | mono_intergenic | H/ACA | | | intergenic |
| ENSOANG00000044894 | ENSOANG00000044894 | O_anatinus | Animals | mono_intergenic | H/ACA | | | intergenic |
| ENSOANG00000044896 | ENSOANG00000044896 | O_anatinus | Animals | mono_intergenic | H/ACA | | | intergenic |
| ENSOANG00000044903 | ENSOANG00000044903 | O_anatinus | Animals | mono_intergenic | H/ACA | | | intergenic |
| ENSOANG00000044904 | ENSOANG00000044904 | O_anatinus | Animals | mono_intergenic | H/ACA | | | intergenic |
| ENSOANG00000044907 | ENSOANG00000044907 | O_anatinus | Animals | mono_intergenic | H/ACA | | | intergenic |
| ENSOANG00000044910 | ENSOANG00000044910 | O_anatinus | Animals | mono_intergenic | H/ACA | | | intergenic |
| ENSOANG00000044913 | ENSOANG00000044913 | O_anatinus | Animals | mono_intergenic | H/ACA | | | intergenic |
| ENSOANG00000044914 | ENSOANG00000044914 | O_anatinus | Animals | mono_intergenic | H/ACA | | | intergenic |
| ENSOANG00000044920 | ENSOANG00000044920 | O_anatinus | Animals | mono_intergenic | H/ACA | | | intergenic |
| ENSOANG00000044922 | ENSOANG00000044922 | O_anatinus | Animals | mono_intergenic | H/ACA | | | intergenic |
| ENSOANG00000044924 | ENSOANG00000044924 | O_anatinus | Animals | mono_intergenic | H/ACA | | | intergenic |
| ENSOANG00000044925 | ENSOANG00000044925 | O_anatinus | Animals | mono_intergenic | H/ACA | | | intergenic |
| ENSOANG00000044927 | ENSOANG00000044927 | O_anatinus | Animals | mono_intergenic | H/ACA | | | intergenic |
| ENSOANG00000044932 | ENSOANG00000044932 | O_anatinus | Animals | mono_intergenic | H/ACA | | | intergenic |
| ENSOANG00000044933 | ENSOANG00000044933 | O_anatinus | Animals | mono_intergenic | H/ACA | | | intergenic |
| ENSOANG00000044934 | ENSOANG00000044934 | O_anatinus | Animals | mono_intronic | H/ACA | ENSOANG00000022711 | | protein_coding |
| ENSOANG00000044938 | ENSOANG00000044938 | O_anatinus | Animals | mono_intergenic | H/ACA | | | intergenic |
| ENSOANG00000044939 | ENSOANG00000044939 | O_anatinus | Animals | mono_intergenic | H/ACA | | | intergenic |
| ENSOANG00000044940 | ENSOANG00000044940 | O_anatinus | Animals | mono_intergenic | H/ACA | | | intergenic |
| ENSOANG00000044941 | ENSOANG00000044941 | O_anatinus | Animals | mono_intergenic | H/ACA | | | intergenic |
| ENSOANG00000044943 | ENSOANG00000044943 | O_anatinus | Animals | mono_intronic | H/ACA | ENSOANG00000035779 | | non_coding |

| | | | | | | | | |
|---|---|---|---|---|---|---|---|---|
| ENSOANG00000044945 | ENSOANG00000044945 | O_anatinus | Animals | mono_intergenic | H/ACA | | | intergenic |
| ENSOANG00000044947 | ENSOANG00000044947 | O_anatinus | Animals | mono_intergenic | H/ACA | | | intergenic |
| ENSOANG00000044951 | ENSOANG00000044951 | O_anatinus | Animals | mono_intergenic | H/ACA | | | intergenic |
| ENSOANG00000044952 | ENSOANG00000044952 | O_anatinus | Animals | mono_intergenic | H/ACA | | | intergenic |
| ENSOANG00000044953 | ENSOANG00000044953 | O_anatinus | Animals | mono_intergenic | H/ACA | | | intergenic |
| ENSOANG00000044963 | ENSOANG00000044963 | O_anatinus | Animals | mono_intergenic | H/ACA | | | intergenic |
| ENSOANG00000044964 | ENSOANG00000044964 | O_anatinus | Animals | mono_intronic | H/ACA | ENSOANG00000007692 | CYP46A1 | protein_coding |
| ENSOANG00000044974 | ENSOANG00000044974 | O_anatinus | Animals | mono_intergenic | H/ACA | | | intergenic |
| ENSOANG00000044980 | ENSOANG00000044980 | O_anatinus | Animals | mono_intergenic | H/ACA | | | intergenic |
| ENSOANG00000044981 | ENSOANG00000044981 | O_anatinus | Animals | mono_intergenic | H/ACA | | | intergenic |
| ENSOANG00000044982 | ENSOANG00000044982 | O_anatinus | Animals | mono_intergenic | H/ACA | | | intergenic |
| ENSOANG00000044985 | ENSOANG00000044985 | O_anatinus | Animals | mono_intronic | H/ACA | ENSOANG00000000581 | ST14 | protein_coding |
| ENSOANG00000044987 | ENSOANG00000044987 | O_anatinus | Animals | mono_intergenic | H/ACA | | | intergenic |
| ENSOANG00000044991 | ENSOANG00000044991 | O_anatinus | Animals | mono_intronic | H/ACA | ENSOANG00000012888 | DENND6A | protein_coding |
| ENSOANG00000044993 | ENSOANG00000044993 | O_anatinus | Animals | mono_intergenic | H/ACA | | | intergenic |
| ENSOANG00000044995 | ENSOANG00000044995 | O_anatinus | Animals | mono_intergenic | H/ACA | | | intergenic |
| ENSOANG00000044996 | ENSOANG00000044996 | O_anatinus | Animals | mono_intergenic | H/ACA | | | intergenic |
| ENSOANG00000044999 | ENSOANG00000044999 | O_anatinus | Animals | mono_intronic | H/ACA | ENSOANG00000001286 | BCKDHB | protein_coding |
| ENSOANG00000045000 | ENSOANG00000045000 | O_anatinus | Animals | intronic_cluster | H/ACA | ENSOANG00000007366 | CNTN1 | protein_coding |
| ENSOANG00000045007 | ENSOANG00000045007 | O_anatinus | Animals | mono_intergenic | H/ACA | | | intergenic |
| ENSOANG00000045008 | ENSOANG00000045008 | O_anatinus | Animals | mono_intergenic | C/D | | | intergenic |
| ENSOANG00000045012 | ENSOANG00000045012 | O_anatinus | Animals | mono_intergenic | H/ACA | | | intergenic |
| ENSOANG00000045013 | ENSOANG00000045013 | O_anatinus | Animals | mono_intergenic | H/ACA | | | intergenic |
| ENSOANG00000045014 | ENSOANG00000045014 | O_anatinus | Animals | mono_intergenic | H/ACA | | | intergenic |
| ENSOANG00000045016 | ENSOANG00000045016 | O_anatinus | Animals | mono_intergenic | H/ACA | | | intergenic |
| ENSOANG00000045019 | ENSOANG00000045019 | O_anatinus | Animals | mono_intergenic | H/ACA | | | intergenic |
| ENSOANG00000045020 | ENSOANG00000045020 | O_anatinus | Animals | mono_intergenic | H/ACA | | | intergenic |
| ENSOANG00000045024 | ENSOANG00000045024 | O_anatinus | Animals | mono_intergenic | H/ACA | | | intergenic |
| ENSOANG00000045028 | ENSOANG00000045028 | O_anatinus | Animals | mono_intronic | H/ACA | ENSOANG00000015024 | GHR | protein_coding |
| ENSOANG00000045030 | ENSOANG00000045030 | O_anatinus | Animals | mono_intergenic | H/ACA | | | intergenic |
| ENSOANG00000045031 | ENSOANG00000045031 | O_anatinus | Animals | mono_intergenic | H/ACA | | | intergenic |
| ENSOANG00000045036 | ENSOANG00000045036 | O_anatinus | Animals | mono_intergenic | H/ACA | | | intergenic |
| ENSOANG00000045040 | ENSOANG00000045040 | O_anatinus | Animals | intergenic_cluster | H/ACA | | | intergenic |
| ENSOANG00000045042 | ENSOANG00000045042 | O_anatinus | Animals | mono_intergenic | H/ACA | | | intergenic |
| ENSOANG00000045047 | ENSOANG00000045047 | O_anatinus | Animals | mono_intergenic | H/ACA | | | intergenic |
| ENSOANG00000045049 | ENSOANG00000045049 | O_anatinus | Animals | mono_intergenic | H/ACA | | | intergenic |
| ENSOANG00000045050 | ENSOANG00000045050 | O_anatinus | Animals | mono_intronic | H/ACA | ENSOANG00000006164 | TNFAIP8 | protein_coding |
| ENSOANG00000045051 | ENSOANG00000045051 | O_anatinus | Animals | mono_intergenic | H/ACA | | | intergenic |
| ENSOANG00000045053 | ENSOANG00000045053 | O_anatinus | Animals | mono_intergenic | H/ACA | | | intergenic |
| ENSOANG00000045055 | ENSOANG00000045055 | O_anatinus | Animals | mono_intergenic | H/ACA | | | intergenic |
| ENSOANG00000045057 | ENSOANG00000045057 | O_anatinus | Animals | mono_intergenic | H/ACA | | | intergenic |

| | | | | | | | | |
|---|---|---|---|---|---|---|---|---|
| ENSOANG00000045066 | ENSOANG00000045066 | O_anatinus | Animals | mono_intergenic | H/ACA | | | intergenic |
| ENSOANG00000045067 | ENSOANG00000045067 | O_anatinus | Animals | mono_intergenic | H/ACA | | | intergenic |
| ENSOANG00000045068 | ENSOANG00000045068 | O_anatinus | Animals | mono_intergenic | H/ACA | | | intergenic |
| ENSOANG00000045070 | ENSOANG00000045070 | O_anatinus | Animals | mono_intergenic | H/ACA | | | intergenic |
| ENSOANG00000045072 | ENSOANG00000045072 | O_anatinus | Animals | mono_intergenic | H/ACA | | | intergenic |
| ENSOANG00000045076 | ENSOANG00000045076 | O_anatinus | Animals | mono_intergenic | H/ACA | | | intergenic |
| ENSOANG00000045077 | ENSOANG00000045077 | O_anatinus | Animals | mono_intergenic | H/ACA | | | intergenic |
| ENSOANG00000045081 | ENSOANG00000045081 | O_anatinus | Animals | mono_intergenic | H/ACA | | | intergenic |
| ENSOANG00000045083 | ENSOANG00000045083 | O_anatinus | Animals | mono_intergenic | H/ACA | | | intergenic |
| ENSOANG00000045086 | ENSOANG00000045086 | O_anatinus | Animals | mono_intergenic | H/ACA | | | intergenic |
| ENSOANG00000045087 | ENSOANG00000045087 | O_anatinus | Animals | mono_intergenic | H/ACA | | | intergenic |
| ENSOANG00000045088 | ENSOANG00000045088 | O_anatinus | Animals | mono_intergenic | H/ACA | | | intergenic |
| ENSOANG00000045091 | ENSOANG00000045091 | O_anatinus | Animals | mono_intergenic | H/ACA | | | intergenic |
| ENSOANG00000045095 | ENSOANG00000045095 | O_anatinus | Animals | mono_intergenic | H/ACA | | | intergenic |
| ENSOANG00000045098 | ENSOANG00000045098 | O_anatinus | Animals | mono_intergenic | H/ACA | | | intergenic |
| ENSOANG00000045099 | ENSOANG00000045099 | O_anatinus | Animals | mono_intergenic | H/ACA | | | intergenic |
| ENSOANG00000045102 | ENSOANG00000045102 | O_anatinus | Animals | mono_intergenic | H/ACA | | | intergenic |
| ENSOANG00000045104 | ENSOANG00000045104 | O_anatinus | Animals | mono_intergenic | H/ACA | | | intergenic |
| ENSOANG00000045111 | ENSOANG00000045111 | O_anatinus | Animals | mono_intronic | H/ACA | ENSOANG00000014699 | GARNL3 | protein_coding |
| ENSOANG00000045115 | ENSOANG00000045115 | O_anatinus | Animals | mono_intergenic | H/ACA | | | intergenic |
| ENSOANG00000045117 | ENSOANG00000045117 | O_anatinus | Animals | mono_intergenic | H/ACA | | | intergenic |
| ENSOANG00000045121 | ENSOANG00000045121 | O_anatinus | Animals | mono_intergenic | H/ACA | | | intergenic |
| ENSOANG00000045123 | ENSOANG00000045123 | O_anatinus | Animals | mono_intergenic | H/ACA | | | intergenic |
| ENSOANG00000045124 | ENSOANG00000045124 | O_anatinus | Animals | mono_intergenic | H/ACA | | | intergenic |
| ENSOANG00000045125 | ENSOANG00000045125 | O_anatinus | Animals | mono_intergenic | H/ACA | | | intergenic |
| ENSOANG00000045126 | ENSOANG00000045126 | O_anatinus | Animals | mono_intergenic | H/ACA | | | intergenic |
| ENSOANG00000045127 | ENSOANG00000045127 | O_anatinus | Animals | mono_intergenic | H/ACA | | | intergenic |
| ENSOANG00000045130 | ENSOANG00000045130 | O_anatinus | Animals | intergenic_cluster | H/ACA | | | intergenic |
| ENSOANG00000045131 | ENSOANG00000045131 | O_anatinus | Animals | mono_intronic | H/ACA | ENSOANG00000015382 | IL5RA | protein_coding |
| ENSOANG00000045132 | ENSOANG00000045132 | O_anatinus | Animals | mono_intronic | H/ACA | ENSOANG00000007549 | PSTPIP1 | protein_coding |
| ENSOANG00000045134 | ENSOANG00000045134 | O_anatinus | Animals | mono_intergenic | H/ACA | | | intergenic |
| ENSOANG00000045137 | ENSOANG00000045137 | O_anatinus | Animals | mono_intergenic | H/ACA | | | intergenic |
| ENSOANG00000045141 | ENSOANG00000045141 | O_anatinus | Animals | mono_intergenic | H/ACA | | | intergenic |
| ENSOANG00000045142 | ENSOANG00000045142 | O_anatinus | Animals | mono_intergenic | H/ACA | | | intergenic |
| ENSOANG00000045145 | ENSOANG00000045145 | O_anatinus | Animals | mono_intergenic | H/ACA | | | intergenic |
| ENSOANG00000045147 | ENSOANG00000045147 | O_anatinus | Animals | mono_intergenic | H/ACA | | | intergenic |
| ENSOANG00000045149 | ENSOANG00000045149 | O_anatinus | Animals | mono_intergenic | H/ACA | | | intergenic |
| ENSOANG00000045155 | ENSOANG00000045155 | O_anatinus | Animals | mono_intergenic | H/ACA | | | intergenic |
| ENSOANG00000045158 | ENSOANG00000045158 | O_anatinus | Animals | mono_intergenic | H/ACA | | | intergenic |
| ENSOANG00000045159 | ENSOANG00000045159 | O_anatinus | Animals | mono_intergenic | H/ACA | | | intergenic |
| ENSOANG00000045160 | ENSOANG00000045160 | O_anatinus | Animals | mono_intergenic | H/ACA | | | intergenic |

| | | | | | | | | |
|---|---|---|---|---|---|---|---|---|
| ENSOANG00000045161 | ENSOANG00000045161 | O_anatinus | Animals | mono_intergenic | H/ACA | | | intergenic |
| ENSOANG00000045169 | ENSOANG00000045169 | O_anatinus | Animals | mono_intergenic | H/ACA | | | intergenic |
| ENSOANG00000045173 | ENSOANG00000045173 | O_anatinus | Animals | mono_intergenic | H/ACA | | | intergenic |
| ENSOANG00000045174 | ENSOANG00000045174 | O_anatinus | Animals | mono_intergenic | H/ACA | | | intergenic |
| ENSOANG00000045176 | ENSOANG00000045176 | O_anatinus | Animals | mono_intergenic | H/ACA | | | intergenic |
| ENSOANG00000045190 | ENSOANG00000045190 | O_anatinus | Animals | mono_intronic | H/ACA | ENSOANG00000042812 | PDZRN3 | protein_coding |
| ENSOANG00000045193 | ENSOANG00000045193 | O_anatinus | Animals | mono_intergenic | H/ACA | | | intergenic |
| ENSOANG00000045195 | ENSOANG00000045195 | O_anatinus | Animals | mono_intergenic | H/ACA | | | intergenic |
| ENSOANG00000045198 | ENSOANG00000045198 | O_anatinus | Animals | mono_intronic | H/ACA | ENSOANG00000003946 | SRD5A2 | protein_coding |
| ENSOANG00000045206 | ENSOANG00000045206 | O_anatinus | Animals | mono_intronic | H/ACA | ENSOANG00000004035 | CAMTA1 | protein_coding |
| ENSOANG00000045209 | ENSOANG00000045209 | O_anatinus | Animals | mono_intronic | H/ACA | ENSOANG00000047048 | | non_coding |
| ENSOANG00000045216 | ENSOANG00000045216 | O_anatinus | Animals | mono_intergenic | H/ACA | | | intergenic |
| ENSOANG00000045218 | ENSOANG00000045218 | O_anatinus | Animals | mono_intergenic | H/ACA | | | intergenic |
| ENSOANG00000045219 | ENSOANG00000045219 | O_anatinus | Animals | mono_intergenic | H/ACA | | | intergenic |
| ENSOANG00000045222 | ENSOANG00000045222 | O_anatinus | Animals | mono_intergenic | H/ACA | | | intergenic |
| ENSOANG00000045225 | ENSOANG00000045225 | O_anatinus | Animals | mono_intergenic | H/ACA | | | intergenic |
| ENSOANG00000045229 | ENSOANG00000045229 | O_anatinus | Animals | mono_intronic | H/ACA | ENSOANG00000002405 | PTPRM | protein_coding |
| ENSOANG00000045231 | ENSOANG00000045231 | O_anatinus | Animals | mono_intergenic | H/ACA | | | intergenic |
| ENSOANG00000045235 | ENSOANG00000045235 | O_anatinus | Animals | mono_intergenic | H/ACA | | | intergenic |
| ENSOANG00000045236 | ENSOANG00000045236 | O_anatinus | Animals | mono_intergenic | H/ACA | | | intergenic |
| ENSOANG00000045238 | ENSOANG00000045238 | O_anatinus | Animals | mono_intergenic | H/ACA | | | intergenic |
| ENSOANG00000045239 | ENSOANG00000045239 | O_anatinus | Animals | mono_intergenic | H/ACA | | | intergenic |
| ENSOANG00000045241 | ENSOANG00000045241 | O_anatinus | Animals | mono_intergenic | H/ACA | | | intergenic |
| ENSOANG00000045244 | ENSOANG00000045244 | O_anatinus | Animals | mono_intergenic | H/ACA | | | intergenic |
| ENSOANG00000045250 | ENSOANG00000045250 | O_anatinus | Animals | mono_intergenic | H/ACA | | | intergenic |
| ENSOANG00000045254 | ENSOANG00000045254 | O_anatinus | Animals | mono_intronic | H/ACA | ENSOANG00000000856 | NME7 | protein_coding |
| ENSOANG00000045255 | ENSOANG00000045255 | O_anatinus | Animals | mono_intergenic | H/ACA | | | intergenic |
| ENSOANG00000045263 | ENSOANG00000045263 | O_anatinus | Animals | mono_intergenic | H/ACA | | | intergenic |
| ENSOANG00000045265 | ENSOANG00000045265 | O_anatinus | Animals | mono_intergenic | H/ACA | | | intergenic |
| ENSOANG00000045266 | ENSOANG00000045266 | O_anatinus | Animals | mono_intergenic | H/ACA | | | intergenic |
| ENSOANG00000045269 | ENSOANG00000045269 | O_anatinus | Animals | mono_intergenic | H/ACA | | | intergenic |
| ENSOANG00000045270 | ENSOANG00000045270 | O_anatinus | Animals | mono_intronic | H/ACA | ENSOANG00000047756 | | protein_coding |
| ENSOANG00000045273 | ENSOANG00000045273 | O_anatinus | Animals | mono_intronic | H/ACA | ENSOANG00000000846 | SNX30 | protein_coding |
| ENSOANG00000045274 | ENSOANG00000045274 | O_anatinus | Animals | mono_intergenic | H/ACA | | | intergenic |
| ENSOANG00000045277 | ENSOANG00000045277 | O_anatinus | Animals | mono_intergenic | H/ACA | | | intergenic |
| ENSOANG00000045280 | ENSOANG00000045280 | O_anatinus | Animals | mono_intergenic | H/ACA | | | intergenic |
| ENSOANG00000045282 | ENSOANG00000045282 | O_anatinus | Animals | mono_intergenic | H/ACA | | | intergenic |
| ENSOANG00000045283 | ENSOANG00000045283 | O_anatinus | Animals | mono_intergenic | H/ACA | | | intergenic |
| ENSOANG00000045286 | ENSOANG00000045286 | O_anatinus | Animals | mono_intergenic | H/ACA | | | intergenic |
| ENSOANG00000045287 | ENSOANG00000045287 | O_anatinus | Animals | mono_intergenic | H/ACA | | | intergenic |
| ENSOANG00000045291 | ENSOANG00000045291 | O_anatinus | Animals | mono_intergenic | H/ACA | | | intergenic |

| | | | | | | | | |
|---|---|---|---|---|---|---|---|---|
| ENSOANG00000045293 | ENSOANG00000045293 | O_anatinus | Animals | mono_intergenic | H/ACA | | | intergenic |
| ENSOANG00000045294 | ENSOANG00000045294 | O_anatinus | Animals | mono_intergenic | H/ACA | | | intergenic |
| ENSOANG00000045295 | ENSOANG00000045295 | O_anatinus | Animals | mono_intergenic | C/D | | | intergenic |
| ENSOANG00000045297 | ENSOANG00000045297 | O_anatinus | Animals | mono_intergenic | H/ACA | | | intergenic |
| ENSOANG00000045298 | ENSOANG00000045298 | O_anatinus | Animals | mono_intergenic | H/ACA | | | intergenic |
| ENSOANG00000045300 | ENSOANG00000045300 | O_anatinus | Animals | mono_intergenic | H/ACA | | | intergenic |
| ENSOANG00000045301 | ENSOANG00000045301 | O_anatinus | Animals | mono_intergenic | H/ACA | | | intergenic |
| ENSOANG00000045303 | ENSOANG00000045303 | O_anatinus | Animals | mono_intergenic | H/ACA | | | intergenic |
| ENSOANG00000045305 | ENSOANG00000045305 | O_anatinus | Animals | mono_intergenic | H/ACA | | | intergenic |
| ENSOANG00000045307 | ENSOANG00000045307 | O_anatinus | Animals | mono_intergenic | H/ACA | | | intergenic |
| ENSOANG00000045308 | ENSOANG00000045308 | O_anatinus | Animals | mono_intergenic | H/ACA | | | intergenic |
| ENSOANG00000045310 | ENSOANG00000045310 | O_anatinus | Animals | mono_intergenic | H/ACA | | | intergenic |
| ENSOANG00000045311 | ENSOANG00000045311 | O_anatinus | Animals | mono_intergenic | H/ACA | | | intergenic |
| ENSOANG00000045316 | ENSOANG00000045316 | O_anatinus | Animals | mono_intergenic | H/ACA | | | intergenic |
| ENSOANG00000045317 | ENSOANG00000045317 | O_anatinus | Animals | mono_intergenic | H/ACA | | | intergenic |
| ENSOANG00000045324 | ENSOANG00000045324 | O_anatinus | Animals | mono_intergenic | H/ACA | | | intergenic |
| ENSOANG00000045325 | ENSOANG00000045325 | O_anatinus | Animals | mono_intergenic | H/ACA | | | intergenic |
| ENSOANG00000045328 | ENSOANG00000045328 | O_anatinus | Animals | mono_intergenic | H/ACA | | | intergenic |
| ENSOANG00000045331 | ENSOANG00000045331 | O_anatinus | Animals | mono_intergenic | H/ACA | | | intergenic |
| ENSOANG00000045332 | ENSOANG00000045332 | O_anatinus | Animals | mono_intergenic | H/ACA | | | intergenic |
| ENSOANG00000045335 | ENSOANG00000045335 | O_anatinus | Animals | mono_intergenic | H/ACA | | | intergenic |
| ENSOANG00000045337 | ENSOANG00000045337 | O_anatinus | Animals | mono_intergenic | H/ACA | | | intergenic |
| ENSOANG00000045339 | ENSOANG00000045339 | O_anatinus | Animals | mono_intergenic | H/ACA | | | intergenic |
| ENSOANG00000045343 | ENSOANG00000045343 | O_anatinus | Animals | mono_intergenic | H/ACA | | | intergenic |
| ENSOANG00000045347 | ENSOANG00000045347 | O_anatinus | Animals | mono_intergenic | H/ACA | | | intergenic |
| ENSOANG00000045351 | ENSOANG00000045351 | O_anatinus | Animals | mono_intergenic | H/ACA | | | intergenic |
| ENSOANG00000045352 | ENSOANG00000045352 | O_anatinus | Animals | intronic_cluster | H/ACA | ENSOANG00000037357 | LRRC1 | protein_coding |
| ENSOANG00000045353 | ENSOANG00000045353 | O_anatinus | Animals | mono_intergenic | H/ACA | | | intergenic |
| ENSOANG00000045354 | ENSOANG00000045354 | O_anatinus | Animals | mono_intergenic | H/ACA | | | intergenic |
| ENSOANG00000045357 | ENSOANG00000045357 | O_anatinus | Animals | mono_intergenic | H/ACA | | | intergenic |
| ENSOANG00000045358 | ENSOANG00000045358 | O_anatinus | Animals | mono_intergenic | H/ACA | | | intergenic |
| ENSOANG00000045359 | ENSOANG00000045359 | O_anatinus | Animals | mono_intergenic | H/ACA | | | intergenic |
| ENSOANG00000045361 | ENSOANG00000045361 | O_anatinus | Animals | mono_intergenic | H/ACA | | | intergenic |
| ENSOANG00000045366 | ENSOANG00000045366 | O_anatinus | Animals | mono_intergenic | H/ACA | | | intergenic |
| ENSOANG00000045371 | ENSOANG00000045371 | O_anatinus | Animals | mono_intergenic | H/ACA | | | intergenic |
| ENSOANG00000045372 | ENSOANG00000045372 | O_anatinus | Animals | mono_intergenic | H/ACA | | | intergenic |
| ENSOANG00000045374 | ENSOANG00000045374 | O_anatinus | Animals | mono_intergenic | H/ACA | | | intergenic |
| ENSOANG00000045375 | ENSOANG00000045375 | O_anatinus | Animals | mono_intergenic | H/ACA | | | intergenic |
| ENSOANG00000045376 | ENSOANG00000045376 | O_anatinus | Animals | mono_intergenic | H/ACA | | | intergenic |
| ENSOANG00000045377 | ENSOANG00000045377 | O_anatinus | Animals | mono_intergenic | H/ACA | | | intergenic |
| ENSOANG00000045378 | ENSOANG00000045378 | O_anatinus | Animals | mono_intergenic | H/ACA | | | intergenic |

| | | | | | | | | |
|---|---|---|---|---|---|---|---|---|
| ENSOANG00000045379 | ENSOANG00000045379 | O_anatinus | Animals | mono_intergenic | H/ACA | | | intergenic |
| ENSOANG00000045380 | ENSOANG00000045380 | O_anatinus | Animals | mono_intergenic | H/ACA | | | intergenic |
| ENSOANG00000045382 | ENSOANG00000045382 | O_anatinus | Animals | mono_intergenic | H/ACA | | | intergenic |
| ENSOANG00000045386 | ENSOANG00000045386 | O_anatinus | Animals | intronic_cluster | H/ACA | ENSOANG00000001361 | KIRREL3 | protein_coding |
| ENSOANG00000045387 | ENSOANG00000045387 | O_anatinus | Animals | mono_intergenic | H/ACA | | | intergenic |
| ENSOANG00000045390 | ENSOANG00000045390 | O_anatinus | Animals | mono_intergenic | H/ACA | | | intergenic |
| ENSOANG00000045393 | ENSOANG00000045393 | O_anatinus | Animals | mono_intergenic | H/ACA | | | intergenic |
| ENSOANG00000045394 | ENSOANG00000045394 | O_anatinus | Animals | mono_intergenic | H/ACA | | | intergenic |
| ENSOANG00000045397 | ENSOANG00000045397 | O_anatinus | Animals | mono_intergenic | H/ACA | | | intergenic |
| ENSOANG00000045398 | ENSOANG00000045398 | O_anatinus | Animals | mono_intergenic | H/ACA | | | intergenic |
| ENSOANG00000045399 | ENSOANG00000045399 | O_anatinus | Animals | mono_intergenic | H/ACA | | | intergenic |
| ENSOANG00000045402 | ENSOANG00000045402 | O_anatinus | Animals | mono_intergenic | H/ACA | | | intergenic |
| ENSOANG00000045408 | ENSOANG00000045408 | O_anatinus | Animals | mono_intergenic | H/ACA | | | intergenic |
| ENSOANG00000045410 | ENSOANG00000045410 | O_anatinus | Animals | mono_intergenic | H/ACA | | | intergenic |
| ENSOANG00000045413 | ENSOANG00000045413 | O_anatinus | Animals | mono_intergenic | H/ACA | | | intergenic |
| ENSOANG00000045414 | ENSOANG00000045414 | O_anatinus | Animals | mono_intergenic | H/ACA | | | intergenic |
| ENSOANG00000045415 | ENSOANG00000045415 | O_anatinus | Animals | mono_intergenic | H/ACA | | | intergenic |
| ENSOANG00000045419 | ENSOANG00000045419 | O_anatinus | Animals | mono_intergenic | H/ACA | | | intergenic |
| ENSOANG00000045422 | ENSOANG00000045422 | O_anatinus | Animals | mono_intergenic | H/ACA | | | intergenic |
| ENSOANG00000045426 | ENSOANG00000045426 | O_anatinus | Animals | mono_intergenic | H/ACA | | | intergenic |
| ENSOANG00000045429 | ENSOANG00000045429 | O_anatinus | Animals | mono_intergenic | H/ACA | | | intergenic |
| ENSOANG00000045431 | ENSOANG00000045431 | O_anatinus | Animals | mono_intergenic | H/ACA | | | intergenic |
| ENSOANG00000045436 | ENSOANG00000045436 | O_anatinus | Animals | mono_intergenic | H/ACA | | | intergenic |
| ENSOANG00000045441 | ENSOANG00000045441 | O_anatinus | Animals | mono_intergenic | H/ACA | | | intergenic |
| ENSOANG00000045444 | ENSOANG00000045444 | O_anatinus | Animals | mono_intergenic | H/ACA | | | intergenic |
| ENSOANG00000045445 | ENSOANG00000045445 | O_anatinus | Animals | mono_intergenic | H/ACA | | | intergenic |
| ENSOANG00000045451 | ENSOANG00000045451 | O_anatinus | Animals | mono_intergenic | H/ACA | | | intergenic |
| ENSOANG00000045453 | ENSOANG00000045453 | O_anatinus | Animals | mono_intronic | H/ACA | ENSOANG00000011213 | RHBDD1 | protein_coding |
| ENSOANG00000045454 | ENSOANG00000045454 | O_anatinus | Animals | mono_intergenic | H/ACA | | | intergenic |
| ENSOANG00000045457 | ENSOANG00000045457 | O_anatinus | Animals | mono_intergenic | H/ACA | | | intergenic |
| ENSOANG00000045458 | ENSOANG00000045458 | O_anatinus | Animals | mono_intergenic | H/ACA | | | intergenic |
| ENSOANG00000045459 | ENSOANG00000045459 | O_anatinus | Animals | mono_intronic | H/ACA | ENSOANG00000007313 | FBXW4 | protein_coding |
| ENSOANG00000045465 | ENSOANG00000045465 | O_anatinus | Animals | mono_intergenic | H/ACA | | | intergenic |
| ENSOANG00000045468 | ENSOANG00000045468 | O_anatinus | Animals | mono_intergenic | H/ACA | | | intergenic |
| ENSOANG00000045471 | ENSOANG00000045471 | O_anatinus | Animals | mono_intergenic | H/ACA | | | intergenic |
| ENSOANG00000045473 | ENSOANG00000045473 | O_anatinus | Animals | mono_intronic | H/ACA | ENSOANG00000006974 | | protein_coding |
| ENSOANG00000045482 | ENSOANG00000045482 | O_anatinus | Animals | mono_intergenic | H/ACA | | | intergenic |
| ENSOANG00000045485 | ENSOANG00000045485 | O_anatinus | Animals | mono_intergenic | H/ACA | | | intergenic |
| ENSOANG00000045489 | ENSOANG00000045489 | O_anatinus | Animals | mono_intergenic | H/ACA | | | intergenic |
| ENSOANG00000045490 | ENSOANG00000045490 | O_anatinus | Animals | mono_intergenic | H/ACA | | | intergenic |
| ENSOANG00000045494 | ENSOANG00000045494 | O_anatinus | Animals | mono_intronic | H/ACA | ENSOANG00000046175 | SDC2 | protein_coding |

| | | | | | | | | |
|---|---|---|---|---|---|---|---|---|
| ENSOANG00000045496 | ENSOANG00000045496 | O_anatinus | Animals | mono_intergenic | H/ACA | | | intergenic |
| ENSOANG00000045498 | ENSOANG00000045498 | O_anatinus | Animals | mono_intergenic | H/ACA | | | intergenic |
| ENSOANG00000045499 | ENSOANG00000045499 | O_anatinus | Animals | mono_intergenic | H/ACA | | | intergenic |
| ENSOANG00000045500 | ENSOANG00000045500 | O_anatinus | Animals | mono_intergenic | H/ACA | | | intergenic |
| ENSOANG00000045501 | ENSOANG00000045501 | O_anatinus | Animals | mono_intergenic | H/ACA | | | intergenic |
| ENSOANG00000045502 | ENSOANG00000045502 | O_anatinus | Animals | mono_intergenic | H/ACA | | | intergenic |
| ENSOANG00000045506 | ENSOANG00000045506 | O_anatinus | Animals | mono_intergenic | H/ACA | | | intergenic |
| ENSOANG00000045514 | ENSOANG00000045514 | O_anatinus | Animals | mono_intergenic | H/ACA | | | intergenic |
| ENSOANG00000045517 | ENSOANG00000045517 | O_anatinus | Animals | mono_intergenic | H/ACA | | | intergenic |
| ENSOANG00000045518 | ENSOANG00000045518 | O_anatinus | Animals | mono_intronic | H/ACA | ENSOANG00000030027 | SLC35F1 | protein_coding |
| ENSOANG00000045520 | ENSOANG00000045520 | O_anatinus | Animals | mono_intergenic | H/ACA | | | intergenic |
| ENSOANG00000045521 | ENSOANG00000045521 | O_anatinus | Animals | mono_intergenic | H/ACA | | | intergenic |
| ENSOANG00000045528 | ENSOANG00000045528 | O_anatinus | Animals | mono_intergenic | H/ACA | | | intergenic |
| ENSOANG00000045529 | ENSOANG00000045529 | O_anatinus | Animals | mono_intergenic | H/ACA | | | intergenic |
| ENSOANG00000045532 | ENSOANG00000045532 | O_anatinus | Animals | mono_intergenic | H/ACA | | | intergenic |
| ENSOANG00000045533 | ENSOANG00000045533 | O_anatinus | Animals | mono_intergenic | H/ACA | | | intergenic |
| ENSOANG00000045536 | ENSOANG00000045536 | O_anatinus | Animals | mono_intergenic | H/ACA | | | intergenic |
| ENSOANG00000045538 | ENSOANG00000045538 | O_anatinus | Animals | mono_intronic | H/ACA | ENSOANG00000005261 | | protein_coding |
| ENSOANG00000045547 | ENSOANG00000045547 | O_anatinus | Animals | mono_intergenic | H/ACA | | | intergenic |
| ENSOANG00000045549 | ENSOANG00000045549 | O_anatinus | Animals | mono_intergenic | H/ACA | | | intergenic |
| ENSOANG00000045552 | ENSOANG00000045552 | O_anatinus | Animals | mono_intergenic | H/ACA | | | intergenic |
| ENSOANG00000045553 | ENSOANG00000045553 | O_anatinus | Animals | mono_intronic | H/ACA | ENSOANG00000035582 | | protein_coding |
| ENSOANG00000045555 | ENSOANG00000045555 | O_anatinus | Animals | mono_intergenic | H/ACA | | | intergenic |
| ENSOANG00000045556 | ENSOANG00000045556 | O_anatinus | Animals | mono_intronic | H/ACA | ENSOANG00000048215 | CFAP74 | protein_coding |
| ENSOANG00000045558 | ENSOANG00000045558 | O_anatinus | Animals | mono_intergenic | H/ACA | | | intergenic |
| ENSOANG00000045561 | ENSOANG00000045561 | O_anatinus | Animals | mono_intergenic | H/ACA | | | intergenic |
| ENSOANG00000045562 | ENSOANG00000045562 | O_anatinus | Animals | mono_intergenic | H/ACA | | | intergenic |
| ENSOANG00000045563 | ENSOANG00000045563 | O_anatinus | Animals | intergenic_cluster | H/ACA | | | intergenic |
| ENSOANG00000045570 | ENSOANG00000045570 | O_anatinus | Animals | mono_intergenic | H/ACA | | | intergenic |
| ENSOANG00000045578 | ENSOANG00000045578 | O_anatinus | Animals | mono_intergenic | H/ACA | | | intergenic |
| ENSOANG00000045581 | ENSOANG00000045581 | O_anatinus | Animals | mono_intergenic | H/ACA | | | intergenic |
| ENSOANG00000045586 | ENSOANG00000045586 | O_anatinus | Animals | mono_intergenic | H/ACA | | | intergenic |
| ENSOANG00000045587 | ENSOANG00000045587 | O_anatinus | Animals | mono_intergenic | H/ACA | | | intergenic |
| ENSOANG00000045589 | ENSOANG00000045589 | O_anatinus | Animals | mono_intergenic | H/ACA | | | intergenic |
| ENSOANG00000045595 | ENSOANG00000045595 | O_anatinus | Animals | mono_intergenic | H/ACA | | | intergenic |
| ENSOANG00000045596 | ENSOANG00000045596 | O_anatinus | Animals | mono_intergenic | H/ACA | | | intergenic |
| ENSOANG00000045598 | ENSOANG00000045598 | O_anatinus | Animals | mono_intronic | H/ACA | ENSOANG00000039954 | SYT13 | protein_coding |
| ENSOANG00000045599 | ENSOANG00000045599 | O_anatinus | Animals | mono_intergenic | H/ACA | | | intergenic |
| ENSOANG00000045601 | ENSOANG00000045601 | O_anatinus | Animals | mono_intergenic | H/ACA | | | intergenic |
| ENSOANG00000045602 | ENSOANG00000045602 | O_anatinus | Animals | mono_intergenic | H/ACA | | | intergenic |
| ENSOANG00000045606 | ENSOANG00000045606 | O_anatinus | Animals | mono_intergenic | H/ACA | | | intergenic |

| | | | | | | | | |
|---|---|---|---|---|---|---|---|---|
| ENSOANG00000045611 | ENSOANG00000045611 | O_anatinus | Animals | mono_intergenic | H/ACA | | | intergenic |
| ENSOANG00000045612 | ENSOANG00000045612 | O_anatinus | Animals | mono_intergenic | H/ACA | | | intergenic |
| ENSOANG00000045619 | ENSOANG00000045619 | O_anatinus | Animals | mono_intergenic | H/ACA | | | intergenic |
| ENSOANG00000045620 | ENSOANG00000045620 | O_anatinus | Animals | mono_intronic | H/ACA | ENSOANG00000047400 | DGKG | protein_coding |
| ENSOANG00000045621 | ENSOANG00000045621 | O_anatinus | Animals | mono_intergenic | H/ACA | | | intergenic |
| ENSOANG00000045622 | ENSOANG00000045622 | O_anatinus | Animals | mono_intergenic | H/ACA | | | intergenic |
| ENSOANG00000045623 | ENSOANG00000045623 | O_anatinus | Animals | mono_intergenic | H/ACA | | | intergenic |
| ENSOANG00000045626 | ENSOANG00000045626 | O_anatinus | Animals | mono_intergenic | H/ACA | | | intergenic |
| ENSOANG00000045629 | ENSOANG00000045629 | O_anatinus | Animals | mono_intergenic | H/ACA | | | intergenic |
| ENSOANG00000045631 | ENSOANG00000045631 | O_anatinus | Animals | mono_intergenic | H/ACA | | | intergenic |
| ENSOANG00000045635 | ENSOANG00000045635 | O_anatinus | Animals | mono_intergenic | H/ACA | | | intergenic |
| ENSOANG00000045640 | ENSOANG00000045640 | O_anatinus | Animals | mono_intronic | H/ACA | ENSOANG00000039346 | SLC2A13 | protein_coding |
| ENSOANG00000045642 | ENSOANG00000045642 | O_anatinus | Animals | mono_intergenic | H/ACA | | | intergenic |
| ENSOANG00000045646 | ENSOANG00000045646 | O_anatinus | Animals | mono_intergenic | H/ACA | | | intergenic |
| ENSOANG00000045650 | ENSOANG00000045650 | O_anatinus | Animals | mono_intergenic | H/ACA | | | intergenic |
| ENSOANG00000045655 | ENSOANG00000045655 | O_anatinus | Animals | mono_intergenic | H/ACA | | | intergenic |
| ENSOANG00000045657 | ENSOANG00000045657 | O_anatinus | Animals | mono_intergenic | H/ACA | | | intergenic |
| ENSOANG00000045667 | ENSOANG00000045667 | O_anatinus | Animals | mono_intergenic | H/ACA | | | intergenic |
| ENSOANG00000045668 | ENSOANG00000045668 | O_anatinus | Animals | mono_intergenic | H/ACA | | | intergenic |
| ENSOANG00000045672 | ENSOANG00000045672 | O_anatinus | Animals | mono_intergenic | H/ACA | | | intergenic |
| ENSOANG00000045674 | ENSOANG00000045674 | O_anatinus | Animals | mono_intergenic | H/ACA | | | intergenic |
| ENSOANG00000045675 | ENSOANG00000045675 | O_anatinus | Animals | mono_intergenic | H/ACA | | | intergenic |
| ENSOANG00000045676 | ENSOANG00000045676 | O_anatinus | Animals | mono_intergenic | H/ACA | | | intergenic |
| ENSOANG00000045678 | ENSOANG00000045678 | O_anatinus | Animals | mono_intergenic | H/ACA | | | intergenic |
| ENSOANG00000045679 | ENSOANG00000045679 | O_anatinus | Animals | mono_intergenic | H/ACA | | | intergenic |
| ENSOANG00000045683 | ENSOANG00000045683 | O_anatinus | Animals | intronic_cluster | H/ACA | ENSOANG00000047521 | HCN4 | protein_coding |
| ENSOANG00000045689 | ENSOANG00000045689 | O_anatinus | Animals | mono_intergenic | H/ACA | | | intergenic |
| ENSOANG00000045691 | ENSOANG00000045691 | O_anatinus | Animals | mono_intergenic | H/ACA | | | intergenic |
| ENSOANG00000045692 | ENSOANG00000045692 | O_anatinus | Animals | mono_intergenic | H/ACA | | | intergenic |
| ENSOANG00000045693 | ENSOANG00000045693 | O_anatinus | Animals | mono_intergenic | H/ACA | | | intergenic |
| ENSOANG00000045695 | ENSOANG00000045695 | O_anatinus | Animals | mono_intergenic | H/ACA | | | intergenic |
| ENSOANG00000045696 | ENSOANG00000045696 | O_anatinus | Animals | mono_intergenic | H/ACA | | | intergenic |
| ENSOANG00000045697 | ENSOANG00000045697 | O_anatinus | Animals | mono_intergenic | H/ACA | | | intergenic |
| ENSOANG00000045699 | ENSOANG00000045699 | O_anatinus | Animals | mono_intergenic | H/ACA | | | intergenic |
| ENSOANG00000045702 | ENSOANG00000045702 | O_anatinus | Animals | mono_intergenic | H/ACA | | | intergenic |
| ENSOANG00000045703 | ENSOANG00000045703 | O_anatinus | Animals | mono_intergenic | H/ACA | | | intergenic |
| ENSOANG00000045706 | ENSOANG00000045706 | O_anatinus | Animals | mono_intronic | H/ACA | ENSOANG00000011655 | RORB | protein_coding |
| ENSOANG00000045707 | ENSOANG00000045707 | O_anatinus | Animals | mono_intergenic | H/ACA | | | intergenic |
| ENSOANG00000045712 | ENSOANG00000045712 | O_anatinus | Animals | mono_intergenic | H/ACA | | | intergenic |
| ENSOANG00000045713 | ENSOANG00000045713 | O_anatinus | Animals | mono_intergenic | H/ACA | | | intergenic |
| ENSOANG00000045718 | ENSOANG00000045718 | O_anatinus | Animals | mono_intergenic | H/ACA | | | intergenic |

| | | | | | | | | |
|---|---|---|---|---|---|---|---|---|
| ENSOANG00000045719 | SNORD33 | O_anatinus | Animals | mono_intronic | C/D | ENSOANG00000000828 | RPL13A | protein_coding |
| ENSOANG00000045720 | ENSOANG00000045720 | O_anatinus | Animals | mono_intergenic | H/ACA | | | intergenic |
| ENSOANG00000045721 | ENSOANG00000045721 | O_anatinus | Animals | mono_intergenic | H/ACA | | | intergenic |
| ENSOANG00000045724 | ENSOANG00000045724 | O_anatinus | Animals | mono_intronic | H/ACA | ENSOANG00000046247 | TGFBR3 | protein_coding |
| ENSOANG00000045731 | ENSOANG00000045731 | O_anatinus | Animals | mono_intergenic | H/ACA | | | intergenic |
| ENSOANG00000045736 | ENSOANG00000045736 | O_anatinus | Animals | mono_intergenic | H/ACA | | | intergenic |
| ENSOANG00000045740 | ENSOANG00000045740 | O_anatinus | Animals | mono_intergenic | H/ACA | | | intergenic |
| ENSOANG00000045741 | ENSOANG00000045741 | O_anatinus | Animals | mono_intergenic | H/ACA | | | intergenic |
| ENSOANG00000045743 | ENSOANG00000045743 | O_anatinus | Animals | mono_intergenic | H/ACA | | | intergenic |
| ENSOANG00000045745 | ENSOANG00000045745 | O_anatinus | Animals | mono_intergenic | H/ACA | | | intergenic |
| ENSOANG00000045747 | ENSOANG00000045747 | O_anatinus | Animals | mono_intergenic | H/ACA | | | intergenic |
| ENSOANG00000045750 | ENSOANG00000045750 | O_anatinus | Animals | mono_intergenic | H/ACA | | | intergenic |
| ENSOANG00000045756 | ENSOANG00000045756 | O_anatinus | Animals | mono_intronic | H/ACA | ENSOANG00000002495 | KLHL6 | protein_coding |
| ENSOANG00000045762 | ENSOANG00000045762 | O_anatinus | Animals | mono_intergenic | H/ACA | | | intergenic |
| ENSOANG00000045763 | ENSOANG00000045763 | O_anatinus | Animals | mono_intergenic | H/ACA | | | intergenic |
| ENSOANG00000045765 | ENSOANG00000045765 | O_anatinus | Animals | mono_intergenic | H/ACA | | | intergenic |
| ENSOANG00000045769 | ENSOANG00000045769 | O_anatinus | Animals | mono_intergenic | H/ACA | | | intergenic |
| ENSOANG00000045771 | ENSOANG00000045771 | O_anatinus | Animals | mono_intergenic | H/ACA | | | intergenic |
| ENSOANG00000045774 | ENSOANG00000045774 | O_anatinus | Animals | mono_intergenic | H/ACA | | | intergenic |
| ENSOANG00000045781 | ENSOANG00000045781 | O_anatinus | Animals | mono_intergenic | H/ACA | | | intergenic |
| ENSOANG00000045783 | ENSOANG00000045783 | O_anatinus | Animals | mono_intergenic | H/ACA | | | intergenic |
| ENSOANG00000045784 | ENSOANG00000045784 | O_anatinus | Animals | mono_intergenic | H/ACA | | | intergenic |
| ENSOANG00000045788 | ENSOANG00000045788 | O_anatinus | Animals | mono_intergenic | H/ACA | | | intergenic |
| ENSOANG00000045793 | ENSOANG00000045793 | O_anatinus | Animals | mono_intergenic | H/ACA | | | intergenic |
| ENSOANG00000045798 | ENSOANG00000045798 | O_anatinus | Animals | mono_intergenic | H/ACA | | | intergenic |
| ENSOANG00000045799 | ENSOANG00000045799 | O_anatinus | Animals | mono_intergenic | H/ACA | | | intergenic |
| ENSOANG00000045800 | ENSOANG00000045800 | O_anatinus | Animals | mono_intergenic | H/ACA | | | intergenic |
| ENSOANG00000045804 | ENSOANG00000045804 | O_anatinus | Animals | mono_intergenic | H/ACA | | | intergenic |
| ENSOANG00000045806 | ENSOANG00000045806 | O_anatinus | Animals | mono_intronic | H/ACA | ENSOANG00000039488 | | protein_coding |
| ENSOANG00000045808 | ENSOANG00000045808 | O_anatinus | Animals | mono_intergenic | H/ACA | | | intergenic |
| ENSOANG00000045809 | ENSOANG00000045809 | O_anatinus | Animals | mono_intergenic | H/ACA | | | intergenic |
| ENSOANG00000045812 | ENSOANG00000045812 | O_anatinus | Animals | mono_intergenic | H/ACA | | | intergenic |
| ENSOANG00000045813 | ENSOANG00000045813 | O_anatinus | Animals | mono_intergenic | H/ACA | | | intergenic |
| ENSOANG00000045814 | ENSOANG00000045814 | O_anatinus | Animals | mono_intergenic | H/ACA | | | intergenic |
| ENSOANG00000045816 | ENSOANG00000045816 | O_anatinus | Animals | mono_intergenic | H/ACA | | | intergenic |
| ENSOANG00000045819 | ENSOANG00000045819 | O_anatinus | Animals | mono_intergenic | H/ACA | | | intergenic |
| ENSOANG00000045823 | ENSOANG00000045823 | O_anatinus | Animals | mono_intergenic | H/ACA | | | intergenic |
| ENSOANG00000045827 | ENSOANG00000045827 | O_anatinus | Animals | mono_intergenic | H/ACA | | | intergenic |
| ENSOANG00000045829 | ENSOANG00000045829 | O_anatinus | Animals | mono_intergenic | H/ACA | | | intergenic |
| ENSOANG00000045832 | ENSOANG00000045832 | O_anatinus | Animals | mono_intergenic | H/ACA | | | intergenic |
| ENSOANG00000045834 | ENSOANG00000045834 | O_anatinus | Animals | mono_intergenic | H/ACA | | | intergenic |

| | | | | | | | | |
|---|---|---|---|---|---|---|---|---|
| ENSOANG00000045835 | ENSOANG00000045835 | O_anatinus | Animals | mono_intergenic | H/ACA | | | intergenic |
| ENSOANG00000045838 | ENSOANG00000045838 | O_anatinus | Animals | mono_intergenic | H/ACA | | | intergenic |
| ENSOANG00000045842 | ENSOANG00000045842 | O_anatinus | Animals | mono_intergenic | H/ACA | | | intergenic |
| ENSOANG00000045843 | ENSOANG00000045843 | O_anatinus | Animals | mono_intergenic | H/ACA | | | intergenic |
| ENSOANG00000045845 | ENSOANG00000045845 | O_anatinus | Animals | mono_intergenic | H/ACA | | | intergenic |
| ENSOANG00000045847 | SNORA73 | O_anatinus | Animals | intergenic_cluster | H/ACA | | | intergenic |
| ENSOANG00000045848 | SNORD24 | O_anatinus | Animals | mono_intronic | C/D | ENSOANG00000007060 | | protein_coding |
| ENSOANG00000045849 | ENSOANG00000045849 | O_anatinus | Animals | mono_intergenic | H/ACA | | | intergenic |
| ENSOANG00000045850 | ENSOANG00000045850 | O_anatinus | Animals | mono_intergenic | H/ACA | | | intergenic |
| ENSOANG00000045851 | ENSOANG00000045851 | O_anatinus | Animals | mono_intergenic | H/ACA | | | intergenic |
| ENSOANG00000045855 | ENSOANG00000045855 | O_anatinus | Animals | mono_intergenic | H/ACA | | | intergenic |
| ENSOANG00000045857 | ENSOANG00000045857 | O_anatinus | Animals | mono_intergenic | H/ACA | | | intergenic |
| ENSOANG00000045858 | ENSOANG00000045858 | O_anatinus | Animals | mono_intergenic | H/ACA | | | intergenic |
| ENSOANG00000045859 | ENSOANG00000045859 | O_anatinus | Animals | mono_intergenic | H/ACA | | | intergenic |
| ENSOANG00000045860 | ENSOANG00000045860 | O_anatinus | Animals | mono_intergenic | H/ACA | | | intergenic |
| ENSOANG00000045862 | ENSOANG00000045862 | O_anatinus | Animals | mono_intergenic | H/ACA | | | intergenic |
| ENSOANG00000045865 | ENSOANG00000045865 | O_anatinus | Animals | mono_intergenic | H/ACA | | | intergenic |
| ENSOANG00000045866 | ENSOANG00000045866 | O_anatinus | Animals | mono_intergenic | H/ACA | | | intergenic |
| ENSOANG00000045867 | ENSOANG00000045867 | O_anatinus | Animals | mono_intronic | H/ACA | ENSOANG00000038424 | | protein_coding |
| ENSOANG00000045869 | ENSOANG00000045869 | O_anatinus | Animals | mono_intronic | H/ACA | ENSOANG00000014279 | ARHGEF3 | protein_coding |
| ENSOANG00000045871 | ENSOANG00000045871 | O_anatinus | Animals | mono_intergenic | H/ACA | | | intergenic |
| ENSOANG00000045872 | ENSOANG00000045872 | O_anatinus | Animals | mono_intergenic | H/ACA | | | intergenic |
| ENSOANG00000045880 | ENSOANG00000045880 | O_anatinus | Animals | mono_intergenic | H/ACA | | | intergenic |
| ENSOANG00000045881 | ENSOANG00000045881 | O_anatinus | Animals | mono_intronic | H/ACA | ENSOANG00000014105 | NEDD4L | protein_coding |
| ENSOANG00000045882 | ENSOANG00000045882 | O_anatinus | Animals | mono_intergenic | H/ACA | | | intergenic |
| ENSOANG00000045883 | ENSOANG00000045883 | O_anatinus | Animals | mono_intergenic | H/ACA | | | intergenic |
| ENSOANG00000045886 | ENSOANG00000045886 | O_anatinus | Animals | mono_intergenic | H/ACA | | | intergenic |
| ENSOANG00000045889 | ENSOANG00000045889 | O_anatinus | Animals | mono_intergenic | H/ACA | | | intergenic |
| ENSOANG00000045890 | ENSOANG00000045890 | O_anatinus | Animals | mono_intronic | H/ACA | ENSOANG00000037097 | | protein_coding |
| ENSOANG00000045891 | ENSOANG00000045891 | O_anatinus | Animals | mono_intergenic | H/ACA | | | intergenic |
| ENSOANG00000045893 | ENSOANG00000045893 | O_anatinus | Animals | mono_intergenic | H/ACA | | | intergenic |
| ENSOANG00000045894 | ENSOANG00000045894 | O_anatinus | Animals | mono_intergenic | H/ACA | | | intergenic |
| ENSOANG00000045897 | ENSOANG00000045897 | O_anatinus | Animals | mono_intergenic | H/ACA | | | intergenic |
| ENSOANG00000045900 | ENSOANG00000045900 | O_anatinus | Animals | mono_intergenic | H/ACA | | | intergenic |
| ENSOANG00000045908 | ENSOANG00000045908 | O_anatinus | Animals | mono_intergenic | H/ACA | | | intergenic |
| ENSOANG00000045914 | ENSOANG00000045914 | O_anatinus | Animals | mono_intergenic | H/ACA | | | intergenic |
| ENSOANG00000045919 | ENSOANG00000045919 | O_anatinus | Animals | mono_intronic | H/ACA | ENSOANG00000010786 | LMBRD2 | protein_coding |
| ENSOANG00000045922 | ENSOANG00000045922 | O_anatinus | Animals | mono_intronic | H/ACA | ENSOANG00000013258 | RIMS2 | protein_coding |
| ENSOANG00000045925 | ENSOANG00000045925 | O_anatinus | Animals | mono_intergenic | H/ACA | | | intergenic |
| ENSOANG00000045929 | ENSOANG00000045929 | O_anatinus | Animals | mono_intronic | H/ACA | ENSOANG00000010503 | SESN3 | protein_coding |
| ENSOANG00000045931 | ENSOANG00000045931 | O_anatinus | Animals | mono_intergenic | H/ACA | | | intergenic |

| | | | | | | | | |
|---|---|---|---|---|---|---|---|---|
| ENSOANG00000045932 | ENSOANG00000045932 | O_anatinus | Animals | mono_intergenic | H/ACA | | | intergenic |
| ENSOANG00000045937 | ENSOANG00000045937 | O_anatinus | Animals | intronic_cluster | H/ACA | ENSOANG00000047093 | RAB6B | protein_coding |
| ENSOANG00000045938 | ENSOANG00000045938 | O_anatinus | Animals | mono_intergenic | H/ACA | | | intergenic |
| ENSOANG00000045940 | ENSOANG00000045940 | O_anatinus | Animals | mono_intergenic | H/ACA | | | intergenic |
| ENSOANG00000045946 | ENSOANG00000045946 | O_anatinus | Animals | mono_intergenic | H/ACA | | | intergenic |
| ENSOANG00000045949 | ENSOANG00000045949 | O_anatinus | Animals | mono_intergenic | H/ACA | | | intergenic |
| ENSOANG00000045955 | ENSOANG00000045955 | O_anatinus | Animals | mono_intergenic | H/ACA | | | intergenic |
| ENSOANG00000045957 | ENSOANG00000045957 | O_anatinus | Animals | intronic_cluster | H/ACA | ENSOANG00000009950 | TMEM132D | protein_coding |
| ENSOANG00000045958 | ENSOANG00000045958 | O_anatinus | Animals | mono_intergenic | H/ACA | | | intergenic |
| ENSOANG00000045961 | ENSOANG00000045961 | O_anatinus | Animals | mono_intergenic | H/ACA | | | intergenic |
| ENSOANG00000045964 | ENSOANG00000045964 | O_anatinus | Animals | mono_intergenic | H/ACA | | | intergenic |
| ENSOANG00000045968 | ENSOANG00000045968 | O_anatinus | Animals | mono_intergenic | H/ACA | | | intergenic |
| ENSOANG00000045972 | ENSOANG00000045972 | O_anatinus | Animals | mono_intergenic | H/ACA | | | intergenic |
| ENSOANG00000045974 | ENSOANG00000045974 | O_anatinus | Animals | mono_intergenic | H/ACA | | | intergenic |
| ENSOANG00000045982 | ENSOANG00000045982 | O_anatinus | Animals | mono_intronic | H/ACA | ENSOANG00000007998 | LRP1B | protein_coding |
| ENSOANG00000045985 | ENSOANG00000045985 | O_anatinus | Animals | mono_intergenic | H/ACA | | | intergenic |
| ENSOANG00000045987 | ENSOANG00000045987 | O_anatinus | Animals | mono_intergenic | H/ACA | | | intergenic |
| ENSOANG00000045992 | ENSOANG00000045992 | O_anatinus | Animals | mono_intronic | H/ACA | ENSOANG00000004993 | TBC1D5 | protein_coding |
| ENSOANG00000045993 | ENSOANG00000045993 | O_anatinus | Animals | mono_intergenic | H/ACA | | | intergenic |
| ENSOANG00000045995 | ENSOANG00000045995 | O_anatinus | Animals | mono_intergenic | H/ACA | | | intergenic |
| ENSOANG00000045996 | ENSOANG00000045996 | O_anatinus | Animals | mono_intergenic | H/ACA | | | intergenic |
| ENSOANG00000046003 | ENSOANG00000046003 | O_anatinus | Animals | mono_intergenic | H/ACA | | | intergenic |
| ENSOANG00000046005 | ENSOANG00000046005 | O_anatinus | Animals | mono_intergenic | H/ACA | | | intergenic |
| ENSOANG00000046010 | ENSOANG00000046010 | O_anatinus | Animals | mono_intergenic | H/ACA | | | intergenic |
| ENSOANG00000046012 | ENSOANG00000046012 | O_anatinus | Animals | mono_intergenic | H/ACA | | | intergenic |
| ENSOANG00000046013 | ENSOANG00000046013 | O_anatinus | Animals | mono_intergenic | H/ACA | | | intergenic |
| ENSOANG00000046014 | ENSOANG00000046014 | O_anatinus | Animals | mono_intergenic | H/ACA | | | intergenic |
| ENSOANG00000046018 | ENSOANG00000046018 | O_anatinus | Animals | mono_intronic | H/ACA | ENSOANG00000031640 | AHI1 | protein_coding |
| ENSOANG00000046019 | ENSOANG00000046019 | O_anatinus | Animals | mono_intergenic | H/ACA | | | intergenic |
| ENSOANG00000046021 | ENSOANG00000046021 | O_anatinus | Animals | mono_intergenic | H/ACA | | | intergenic |
| ENSOANG00000046022 | ENSOANG00000046022 | O_anatinus | Animals | mono_intronic | H/ACA | ENSOANG00000014835 | ST8SIA5 | protein_coding |
| ENSOANG00000046026 | ENSOANG00000046026 | O_anatinus | Animals | mono_intergenic | H/ACA | | | intergenic |
| ENSOANG00000046029 | ENSOANG00000046029 | O_anatinus | Animals | intronic_cluster | H/ACA | ENSOANG00000012013 | CRB1 | protein_coding |
| ENSOANG00000046036 | ENSOANG00000046036 | O_anatinus | Animals | mono_intergenic | H/ACA | | | intergenic |
| ENSOANG00000046040 | ENSOANG00000046040 | O_anatinus | Animals | mono_intergenic | H/ACA | | | intergenic |
| ENSOANG00000046045 | ENSOANG00000046045 | O_anatinus | Animals | mono_intronic | H/ACA | ENSOANG00000048340 | WHRN | protein_coding |
| ENSOANG00000046046 | ENSOANG00000046046 | O_anatinus | Animals | mono_intergenic | H/ACA | | | intergenic |
| ENSOANG00000046047 | ENSOANG00000046047 | O_anatinus | Animals | mono_intergenic | H/ACA | | | intergenic |
| ENSOANG00000046048 | ENSOANG00000046048 | O_anatinus | Animals | mono_intergenic | H/ACA | | | intergenic |
| ENSOANG00000046051 | ENSOANG00000046051 | O_anatinus | Animals | mono_intergenic | H/ACA | | | intergenic |
| ENSOANG00000046053 | ENSOANG00000046053 | O_anatinus | Animals | mono_intergenic | H/ACA | | | intergenic |

| | | | | | | | | |
|---|---|---|---|---|---|---|---|---|
| ENSOANG00000046055 | ENSOANG00000046055 | O_anatinus | Animals | mono_intergenic | H/ACA | | | intergenic |
| ENSOANG00000046056 | ENSOANG00000046056 | O_anatinus | Animals | mono_intronic | H/ACA | ENSOANG00000004741 | | protein_coding |
| ENSOANG00000046057 | ENSOANG00000046057 | O_anatinus | Animals | mono_intergenic | H/ACA | | | intergenic |
| ENSOANG00000046060 | ENSOANG00000046060 | O_anatinus | Animals | mono_intergenic | H/ACA | | | intergenic |
| ENSOANG00000046063 | ENSOANG00000046063 | O_anatinus | Animals | mono_intergenic | H/ACA | | | intergenic |
| ENSOANG00000046065 | ENSOANG00000046065 | O_anatinus | Animals | mono_intergenic | H/ACA | | | intergenic |
| ENSOANG00000046067 | ENSOANG00000046067 | O_anatinus | Animals | mono_intronic | H/ACA | ENSOANG00000014345 | TOX2 | protein_coding |
| ENSOANG00000046068 | ENSOANG00000046068 | O_anatinus | Animals | mono_intergenic | H/ACA | | | intergenic |
| ENSOANG00000046069 | ENSOANG00000046069 | O_anatinus | Animals | mono_intronic | H/ACA | ENSOANG00000046982 | DOK6 | protein_coding |
| ENSOANG00000046074 | ENSOANG00000046074 | O_anatinus | Animals | mono_intergenic | H/ACA | | | intergenic |
| ENSOANG00000046075 | ENSOANG00000046075 | O_anatinus | Animals | mono_intergenic | H/ACA | | | intergenic |
| ENSOANG00000046078 | ENSOANG00000046078 | O_anatinus | Animals | mono_intergenic | H/ACA | | | intergenic |
| ENSOANG00000046079 | ENSOANG00000046079 | O_anatinus | Animals | mono_intergenic | H/ACA | | | intergenic |
| ENSOANG00000046080 | ENSOANG00000046080 | O_anatinus | Animals | mono_intergenic | H/ACA | | | intergenic |
| ENSOANG00000046081 | ENSOANG00000046081 | O_anatinus | Animals | mono_intronic | H/ACA | ENSOANG00000040706 | EPHB2 | protein_coding |
| ENSOANG00000046082 | ENSOANG00000046082 | O_anatinus | Animals | mono_intergenic | H/ACA | | | intergenic |
| ENSOANG00000046086 | ENSOANG00000046086 | O_anatinus | Animals | mono_intergenic | H/ACA | | | intergenic |
| ENSOANG00000046089 | ENSOANG00000046089 | O_anatinus | Animals | mono_intergenic | H/ACA | | | intergenic |
| ENSOANG00000046094 | ENSOANG00000046094 | O_anatinus | Animals | mono_intergenic | H/ACA | | | intergenic |
| ENSOANG00000046096 | ENSOANG00000046096 | O_anatinus | Animals | mono_intergenic | H/ACA | | | intergenic |
| ENSOANG00000046100 | ENSOANG00000046100 | O_anatinus | Animals | mono_intergenic | H/ACA | | | intergenic |
| ENSOANG00000046101 | ENSOANG00000046101 | O_anatinus | Animals | mono_intergenic | H/ACA | | | intergenic |
| ENSOANG00000046102 | ENSOANG00000046102 | O_anatinus | Animals | mono_intronic | H/ACA | ENSOANG00000012096 | | protein_coding |
| ENSOANG00000046106 | ENSOANG00000046106 | O_anatinus | Animals | mono_intronic | H/ACA | ENSOANG00000005621 | DSG4 | protein_coding |
| ENSOANG00000046109 | ENSOANG00000046109 | O_anatinus | Animals | mono_intergenic | H/ACA | | | intergenic |
| ENSOANG00000046110 | ENSOANG00000046110 | O_anatinus | Animals | mono_intergenic | H/ACA | | | intergenic |
| ENSOANG00000046111 | ENSOANG00000046111 | O_anatinus | Animals | mono_intergenic | H/ACA | | | intergenic |
| ENSOANG00000046112 | ENSOANG00000046112 | O_anatinus | Animals | mono_intergenic | H/ACA | | | intergenic |
| ENSOANG00000046119 | ENSOANG00000046119 | O_anatinus | Animals | mono_intergenic | H/ACA | | | intergenic |
| ENSOANG00000046120 | ENSOANG00000046120 | O_anatinus | Animals | mono_intergenic | H/ACA | | | intergenic |
| ENSOANG00000046121 | ENSOANG00000046121 | O_anatinus | Animals | mono_intergenic | H/ACA | | | intergenic |
| ENSOANG00000046122 | ENSOANG00000046122 | O_anatinus | Animals | mono_intronic | H/ACA | ENSOANG00000021517 | AK5 | protein_coding |
| ENSOANG00000046128 | ENSOANG00000046128 | O_anatinus | Animals | mono_intergenic | H/ACA | | | intergenic |
| ENSOANG00000046129 | ENSOANG00000046129 | O_anatinus | Animals | mono_intergenic | H/ACA | | | intergenic |
| ENSOANG00000046130 | ENSOANG00000046130 | O_anatinus | Animals | mono_intergenic | H/ACA | | | intergenic |
| ENSOANG00000046131 | ENSOANG00000046131 | O_anatinus | Animals | mono_intergenic | H/ACA | | | intergenic |
| ENSOANG00000046132 | ENSOANG00000046132 | O_anatinus | Animals | intronic_cluster | H/ACA | ENSOANG00000012013 | CRB1 | protein_coding |
| ENSOANG00000046134 | ENSOANG00000046134 | O_anatinus | Animals | mono_intergenic | H/ACA | | | intergenic |
| ENSOANG00000046136 | ENSOANG00000046136 | O_anatinus | Animals | mono_intergenic | H/ACA | | | intergenic |
| ENSOANG00000046138 | ENSOANG00000046138 | O_anatinus | Animals | mono_intergenic | H/ACA | | | intergenic |
| ENSOANG00000046140 | ENSOANG00000046140 | O_anatinus | Animals | mono_intergenic | H/ACA | | | intergenic |

| | | | | | | | | |
|---|---|---|---|---|---|---|---|---|
| ENSOANG00000046143 | ENSOANG00000046143 | O_anatinus | Animals | mono_intergenic | H/ACA | | | intergenic |
| ENSOANG00000046145 | ENSOANG00000046145 | O_anatinus | Animals | mono_intergenic | H/ACA | | | intergenic |
| ENSOANG00000046147 | ENSOANG00000046147 | O_anatinus | Animals | mono_intergenic | H/ACA | | | intergenic |
| ENSOANG00000046148 | ENSOANG00000046148 | O_anatinus | Animals | mono_intergenic | H/ACA | | | intergenic |
| ENSOANG00000046149 | ENSOANG00000046149 | O_anatinus | Animals | mono_intergenic | H/ACA | | | intergenic |
| ENSOANG00000046151 | ENSOANG00000046151 | O_anatinus | Animals | mono_intergenic | H/ACA | | | intergenic |
| ENSOANG00000046152 | ENSOANG00000046152 | O_anatinus | Animals | mono_intronic | H/ACA | ENSOANG00000045073 | SCN2B | protein_coding |
| ENSOANG00000046153 | ENSOANG00000046153 | O_anatinus | Animals | mono_intergenic | H/ACA | | | intergenic |
| ENSOANG00000046157 | ENSOANG00000046157 | O_anatinus | Animals | mono_intergenic | H/ACA | | | intergenic |
| ENSOANG00000046158 | ENSOANG00000046158 | O_anatinus | Animals | mono_intronic | H/ACA | ENSOANG00000029705 | CPQ | protein_coding |
| ENSOANG00000046160 | ENSOANG00000046160 | O_anatinus | Animals | mono_intergenic | H/ACA | | | intergenic |
| ENSOANG00000046161 | ENSOANG00000046161 | O_anatinus | Animals | mono_intergenic | H/ACA | | | intergenic |
| ENSOANG00000046163 | ENSOANG00000046163 | O_anatinus | Animals | mono_intergenic | H/ACA | | | intergenic |
| ENSOANG00000046165 | ENSOANG00000046165 | O_anatinus | Animals | mono_intergenic | H/ACA | | | intergenic |
| ENSOANG00000046166 | ENSOANG00000046166 | O_anatinus | Animals | mono_intergenic | H/ACA | | | intergenic |
| ENSOANG00000046167 | ENSOANG00000046167 | O_anatinus | Animals | mono_intergenic | H/ACA | | | intergenic |
| ENSOANG00000046170 | ENSOANG00000046170 | O_anatinus | Animals | mono_intergenic | H/ACA | | | intergenic |
| ENSOANG00000046171 | ENSOANG00000046171 | O_anatinus | Animals | mono_intergenic | H/ACA | | | intergenic |
| ENSOANG00000046177 | ENSOANG00000046177 | O_anatinus | Animals | mono_intergenic | H/ACA | | | intergenic |
| ENSOANG00000046178 | ENSOANG00000046178 | O_anatinus | Animals | mono_intergenic | H/ACA | | | intergenic |
| ENSOANG00000046179 | ENSOANG00000046179 | O_anatinus | Animals | mono_intergenic | H/ACA | | | intergenic |
| ENSOANG00000046182 | ENSOANG00000046182 | O_anatinus | Animals | mono_intergenic | H/ACA | | | intergenic |
| ENSOANG00000046184 | ENSOANG00000046184 | O_anatinus | Animals | mono_intergenic | H/ACA | | | intergenic |
| ENSOANG00000046187 | ENSOANG00000046187 | O_anatinus | Animals | mono_intergenic | H/ACA | | | intergenic |
| ENSOANG00000046191 | ENSOANG00000046191 | O_anatinus | Animals | mono_intergenic | H/ACA | | | intergenic |
| ENSOANG00000046192 | ENSOANG00000046192 | O_anatinus | Animals | mono_intronic | H/ACA | ENSOANG00000043085 | | protein_coding |
| ENSOANG00000046200 | ENSOANG00000046200 | O_anatinus | Animals | mono_intronic | H/ACA | ENSOANG00000003698 | TKT | protein_coding |
| ENSOANG00000046201 | ENSOANG00000046201 | O_anatinus | Animals | mono_intergenic | H/ACA | | | intergenic |
| ENSOANG00000046204 | ENSOANG00000046204 | O_anatinus | Animals | mono_intergenic | H/ACA | | | intergenic |
| ENSOANG00000046206 | ENSOANG00000046206 | O_anatinus | Animals | mono_intergenic | H/ACA | | | intergenic |
| ENSOANG00000046209 | ENSOANG00000046209 | O_anatinus | Animals | mono_intergenic | H/ACA | | | intergenic |
| ENSOANG00000046215 | ENSOANG00000046215 | O_anatinus | Animals | mono_intergenic | H/ACA | | | intergenic |
| ENSOANG00000046216 | ENSOANG00000046216 | O_anatinus | Animals | mono_intergenic | H/ACA | | | intergenic |
| ENSOANG00000046219 | ENSOANG00000046219 | O_anatinus | Animals | mono_intergenic | H/ACA | | | intergenic |
| ENSOANG00000046220 | ENSOANG00000046220 | O_anatinus | Animals | mono_intronic | H/ACA | ENSOANG00000020697 | RSRC1 | protein_coding |
| ENSOANG00000046223 | ENSOANG00000046223 | O_anatinus | Animals | mono_intronic | H/ACA | ENSOANG00000001169 | HYDIN | protein_coding |
| ENSOANG00000046226 | ENSOANG00000046226 | O_anatinus | Animals | mono_intergenic | H/ACA | | | intergenic |
| ENSOANG00000046227 | ENSOANG00000046227 | O_anatinus | Animals | mono_intergenic | H/ACA | | | intergenic |
| ENSOANG00000046230 | ENSOANG00000046230 | O_anatinus | Animals | mono_intergenic | H/ACA | | | intergenic |
| ENSOANG00000046231 | ENSOANG00000046231 | O_anatinus | Animals | mono_intergenic | H/ACA | | | intergenic |
| ENSOANG00000046233 | ENSOANG00000046233 | O_anatinus | Animals | mono_intergenic | H/ACA | | | intergenic |

| | | | | | | | | |
|---|---|---|---|---|---|---|---|---|
| ENSOANG00000046238 | ENSOANG00000046238 | O_anatinus | Animals | mono_intergenic | H/ACA | | | intergenic |
| ENSOANG00000046246 | ENSOANG00000046246 | O_anatinus | Animals | mono_intergenic | H/ACA | | | intergenic |
| ENSOANG00000046249 | ENSOANG00000046249 | O_anatinus | Animals | mono_intergenic | H/ACA | | | intergenic |
| ENSOANG00000046253 | ENSOANG00000046253 | O_anatinus | Animals | mono_intergenic | H/ACA | | | intergenic |
| ENSOANG00000046254 | ENSOANG00000046254 | O_anatinus | Animals | mono_intergenic | H/ACA | | | intergenic |
| ENSOANG00000046255 | ENSOANG00000046255 | O_anatinus | Animals | mono_intergenic | H/ACA | | | intergenic |
| ENSOANG00000046256 | ENSOANG00000046256 | O_anatinus | Animals | mono_intergenic | H/ACA | | | intergenic |
| ENSOANG00000046258 | ENSOANG00000046258 | O_anatinus | Animals | mono_intergenic | H/ACA | | | intergenic |
| ENSOANG00000046261 | ENSOANG00000046261 | O_anatinus | Animals | mono_intergenic | H/ACA | | | intergenic |
| ENSOANG00000046263 | ENSOANG00000046263 | O_anatinus | Animals | mono_intergenic | H/ACA | | | intergenic |
| ENSOANG00000046264 | ENSOANG00000046264 | O_anatinus | Animals | intronic_cluster | H/ACA | ENSOANG00000014155 | USP54 | protein_coding |
| ENSOANG00000046269 | ENSOANG00000046269 | O_anatinus | Animals | mono_intergenic | H/ACA | | | intergenic |
| ENSOANG00000046270 | ENSOANG00000046270 | O_anatinus | Animals | mono_intergenic | H/ACA | | | intergenic |
| ENSOANG00000046273 | ENSOANG00000046273 | O_anatinus | Animals | mono_intergenic | H/ACA | | | intergenic |
| ENSOANG00000046275 | ENSOANG00000046275 | O_anatinus | Animals | mono_intergenic | H/ACA | | | intergenic |
| ENSOANG00000046276 | ENSOANG00000046276 | O_anatinus | Animals | mono_intergenic | H/ACA | | | intergenic |
| ENSOANG00000046278 | ENSOANG00000046278 | O_anatinus | Animals | mono_intergenic | H/ACA | | | intergenic |
| ENSOANG00000046279 | ENSOANG00000046279 | O_anatinus | Animals | mono_intergenic | H/ACA | | | intergenic |
| ENSOANG00000046281 | ENSOANG00000046281 | O_anatinus | Animals | mono_intergenic | H/ACA | | | intergenic |
| ENSOANG00000046290 | ENSOANG00000046290 | O_anatinus | Animals | mono_intergenic | H/ACA | | | intergenic |
| ENSOANG00000046291 | ENSOANG00000046291 | O_anatinus | Animals | mono_intergenic | H/ACA | | | intergenic |
| ENSOANG00000046296 | ENSOANG00000046296 | O_anatinus | Animals | mono_intergenic | H/ACA | | | intergenic |
| ENSOANG00000046300 | ENSOANG00000046300 | O_anatinus | Animals | mono_intergenic | H/ACA | | | intergenic |
| ENSOANG00000046301 | ENSOANG00000046301 | O_anatinus | Animals | mono_intergenic | H/ACA | | | intergenic |
| ENSOANG00000046306 | ENSOANG00000046306 | O_anatinus | Animals | mono_intergenic | H/ACA | | | intergenic |
| ENSOANG00000046309 | ENSOANG00000046309 | O_anatinus | Animals | mono_intergenic | H/ACA | | | intergenic |
| ENSOANG00000046312 | ENSOANG00000046312 | O_anatinus | Animals | mono_intergenic | H/ACA | | | intergenic |
| ENSOANG00000046314 | ENSOANG00000046314 | O_anatinus | Animals | mono_intergenic | H/ACA | | | intergenic |
| ENSOANG00000046317 | ENSOANG00000046317 | O_anatinus | Animals | mono_intergenic | H/ACA | | | intergenic |
| ENSOANG00000046319 | ENSOANG00000046319 | O_anatinus | Animals | mono_intergenic | H/ACA | | | intergenic |
| ENSOANG00000046322 | ENSOANG00000046322 | O_anatinus | Animals | mono_intergenic | H/ACA | | | intergenic |
| ENSOANG00000046328 | ENSOANG00000046328 | O_anatinus | Animals | mono_intergenic | H/ACA | | | intergenic |
| ENSOANG00000046329 | ENSOANG00000046329 | O_anatinus | Animals | mono_intergenic | H/ACA | | | intergenic |
| ENSOANG00000046332 | ENSOANG00000046332 | O_anatinus | Animals | mono_intronic | H/ACA | ENSOANG00000014833 | LOXHD1 | protein_coding |
| ENSOANG00000046333 | ENSOANG00000046333 | O_anatinus | Animals | mono_intergenic | H/ACA | | | intergenic |
| ENSOANG00000046339 | ENSOANG00000046339 | O_anatinus | Animals | mono_intergenic | H/ACA | | | intergenic |
| ENSOANG00000046340 | ENSOANG00000046340 | O_anatinus | Animals | mono_intergenic | H/ACA | | | intergenic |
| ENSOANG00000046343 | ENSOANG00000046343 | O_anatinus | Animals | mono_intergenic | H/ACA | | | intergenic |
| ENSOANG00000046345 | ENSOANG00000046345 | O_anatinus | Animals | mono_intergenic | H/ACA | | | intergenic |
| ENSOANG00000046348 | ENSOANG00000046348 | O_anatinus | Animals | mono_intergenic | H/ACA | | | intergenic |
| ENSOANG00000046350 | ENSOANG00000046350 | O_anatinus | Animals | mono_intronic | H/ACA | ENSOANG00000011092 | SLC6A2 | protein_coding |

| | | | | | | | | |
|---|---|---|---|---|---|---|---|---|
| ENSOANG00000046352 | ENSOANG00000046352 | O_anatinus | Animals | mono_intergenic | H/ACA | | | intergenic |
| ENSOANG00000046353 | ENSOANG00000046353 | O_anatinus | Animals | mono_intergenic | H/ACA | | | intergenic |
| ENSOANG00000046354 | ENSOANG00000046354 | O_anatinus | Animals | mono_intergenic | H/ACA | | | intergenic |
| ENSOANG00000046355 | ENSOANG00000046355 | O_anatinus | Animals | mono_intergenic | H/ACA | | | intergenic |
| ENSOANG00000046357 | ENSOANG00000046357 | O_anatinus | Animals | mono_intergenic | H/ACA | | | intergenic |
| ENSOANG00000046362 | ENSOANG00000046362 | O_anatinus | Animals | mono_intergenic | H/ACA | | | intergenic |
| ENSOANG00000046363 | ENSOANG00000046363 | O_anatinus | Animals | mono_intergenic | H/ACA | | | intergenic |
| ENSOANG00000046364 | ENSOANG00000046364 | O_anatinus | Animals | mono_intronic | H/ACA | ENSOANG00000021604 | VSTM4 | protein_coding |
| ENSOANG00000046367 | ENSOANG00000046367 | O_anatinus | Animals | mono_intergenic | H/ACA | | | intergenic |
| ENSOANG00000046368 | ENSOANG00000046368 | O_anatinus | Animals | mono_intergenic | H/ACA | | | intergenic |
| ENSOANG00000046370 | ENSOANG00000046370 | O_anatinus | Animals | mono_intergenic | H/ACA | | | intergenic |
| ENSOANG00000046371 | ENSOANG00000046371 | O_anatinus | Animals | mono_intronic | H/ACA | ENSOANG00000044559 | | protein_coding |
| ENSOANG00000046380 | ENSOANG00000046380 | O_anatinus | Animals | mono_intergenic | H/ACA | | | intergenic |
| ENSOANG00000046381 | ENSOANG00000046381 | O_anatinus | Animals | mono_intergenic | H/ACA | | | intergenic |
| ENSOANG00000046383 | ENSOANG00000046383 | O_anatinus | Animals | mono_intronic | C/D | ENSOANG00000005781 | EIF4G1 | protein_coding |
| ENSOANG00000046385 | ENSOANG00000046385 | O_anatinus | Animals | mono_intergenic | H/ACA | | | intergenic |
| ENSOANG00000046386 | ENSOANG00000046386 | O_anatinus | Animals | mono_intergenic | H/ACA | | | intergenic |
| ENSOANG00000046390 | ENSOANG00000046390 | O_anatinus | Animals | mono_intergenic | H/ACA | | | intergenic |
| ENSOANG00000046395 | ENSOANG00000046395 | O_anatinus | Animals | mono_intergenic | H/ACA | | | intergenic |
| ENSOANG00000046398 | ENSOANG00000046398 | O_anatinus | Animals | mono_intergenic | H/ACA | | | intergenic |
| ENSOANG00000046399 | ENSOANG00000046399 | O_anatinus | Animals | mono_intergenic | H/ACA | | | intergenic |
| ENSOANG00000046404 | ENSOANG00000046404 | O_anatinus | Animals | mono_intergenic | H/ACA | | | intergenic |
| ENSOANG00000046405 | ENSOANG00000046405 | O_anatinus | Animals | mono_intergenic | H/ACA | | | intergenic |
| ENSOANG00000046410 | ENSOANG00000046410 | O_anatinus | Animals | mono_intergenic | H/ACA | | | intergenic |
| ENSOANG00000046412 | ENSOANG00000046412 | O_anatinus | Animals | mono_intergenic | H/ACA | | | intergenic |
| ENSOANG00000046415 | ENSOANG00000046415 | O_anatinus | Animals | mono_intergenic | H/ACA | | | intergenic |
| ENSOANG00000046417 | ENSOANG00000046417 | O_anatinus | Animals | mono_intronic | H/ACA | ENSOANG00000004962 | ADAMTS20 | protein_coding |
| ENSOANG00000046419 | ENSOANG00000046419 | O_anatinus | Animals | mono_intronic | H/ACA | ENSOANG00000041311 | | protein_coding |
| ENSOANG00000046420 | ENSOANG00000046420 | O_anatinus | Animals | mono_intergenic | H/ACA | | | intergenic |
| ENSOANG00000046424 | ENSOANG00000046424 | O_anatinus | Animals | mono_intergenic | H/ACA | | | intergenic |
| ENSOANG00000046425 | ENSOANG00000046425 | O_anatinus | Animals | mono_intergenic | H/ACA | | | intergenic |
| ENSOANG00000046430 | ENSOANG00000046430 | O_anatinus | Animals | mono_intergenic | H/ACA | | | intergenic |
| ENSOANG00000046434 | ENSOANG00000046434 | O_anatinus | Animals | mono_intronic | H/ACA | ENSOANG00000003440 | MALT1 | protein_coding |
| ENSOANG00000046436 | ENSOANG00000046436 | O_anatinus | Animals | mono_intergenic | H/ACA | | | intergenic |
| ENSOANG00000046440 | ENSOANG00000046440 | O_anatinus | Animals | mono_intergenic | H/ACA | | | intergenic |
| ENSOANG00000046441 | ENSOANG00000046441 | O_anatinus | Animals | mono_intergenic | H/ACA | | | intergenic |
| ENSOANG00000046446 | ENSOANG00000046446 | O_anatinus | Animals | mono_intergenic | H/ACA | | | intergenic |
| ENSOANG00000046447 | ENSOANG00000046447 | O_anatinus | Animals | mono_intergenic | H/ACA | | | intergenic |
| ENSOANG00000046448 | ENSOANG00000046448 | O_anatinus | Animals | mono_intergenic | H/ACA | | | intergenic |
| ENSOANG00000046449 | ENSOANG00000046449 | O_anatinus | Animals | mono_intergenic | H/ACA | | | intergenic |
| ENSOANG00000046450 | ENSOANG00000046450 | O_anatinus | Animals | mono_intergenic | H/ACA | | | intergenic |

| | | | | | | | | |
|---|---|---|---|---|---|---|---|---|
| ENSOANG00000046455 | ENSOANG00000046455 | O_anatinus | Animals | mono_intergenic | H/ACA | | | intergenic |
| ENSOANG00000046460 | ENSOANG00000046460 | O_anatinus | Animals | mono_intergenic | H/ACA | | | intergenic |
| ENSOANG00000046461 | ENSOANG00000046461 | O_anatinus | Animals | mono_intergenic | H/ACA | | | intergenic |
| ENSOANG00000046462 | ENSOANG00000046462 | O_anatinus | Animals | mono_intergenic | H/ACA | | | intergenic |
| ENSOANG00000046465 | ENSOANG00000046465 | O_anatinus | Animals | mono_intergenic | H/ACA | | | intergenic |
| ENSOANG00000046467 | ENSOANG00000046467 | O_anatinus | Animals | mono_intergenic | H/ACA | | | intergenic |
| ENSOANG00000046470 | ENSOANG00000046470 | O_anatinus | Animals | mono_intergenic | H/ACA | | | intergenic |
| ENSOANG00000046473 | ENSOANG00000046473 | O_anatinus | Animals | mono_intergenic | H/ACA | | | intergenic |
| ENSOANG00000046475 | ENSOANG00000046475 | O_anatinus | Animals | mono_intergenic | H/ACA | | | intergenic |
| ENSOANG00000046479 | ENSOANG00000046479 | O_anatinus | Animals | mono_intronic | H/ACA | ENSOANG00000005363 | SH2D4A | protein_coding |
| ENSOANG00000046481 | ENSOANG00000046481 | O_anatinus | Animals | mono_intronic | H/ACA | ENSOANG00000012872 | | protein_coding |
| ENSOANG00000046484 | ENSOANG00000046484 | O_anatinus | Animals | mono_intronic | H/ACA | ENSOANG00000015082 | PTPRT | protein_coding |
| ENSOANG00000046486 | ENSOANG00000046486 | O_anatinus | Animals | mono_intergenic | H/ACA | | | intergenic |
| ENSOANG00000046488 | ENSOANG00000046488 | O_anatinus | Animals | mono_intronic | H/ACA | ENSOANG00000029705 | CPQ | protein_coding |
| ENSOANG00000046491 | ENSOANG00000046491 | O_anatinus | Animals | mono_intergenic | H/ACA | | | intergenic |
| ENSOANG00000046493 | ENSOANG00000046493 | O_anatinus | Animals | mono_intergenic | H/ACA | | | intergenic |
| ENSOANG00000046497 | ENSOANG00000046497 | O_anatinus | Animals | mono_intergenic | H/ACA | | | intergenic |
| ENSOANG00000046498 | ENSOANG00000046498 | O_anatinus | Animals | mono_intergenic | H/ACA | | | intergenic |
| ENSOANG00000046500 | ENSOANG00000046500 | O_anatinus | Animals | intronic_cluster | H/ACA | ENSOANG00000029848 | | protein_coding |
| ENSOANG00000046501 | ENSOANG00000046501 | O_anatinus | Animals | mono_intergenic | H/ACA | | | intergenic |
| ENSOANG00000046503 | ENSOANG00000046503 | O_anatinus | Animals | mono_intergenic | H/ACA | | | intergenic |
| ENSOANG00000046505 | ENSOANG00000046505 | O_anatinus | Animals | mono_intergenic | H/ACA | | | intergenic |
| ENSOANG00000046506 | ENSOANG00000046506 | O_anatinus | Animals | mono_intergenic | H/ACA | | | intergenic |
| ENSOANG00000046510 | ENSOANG00000046510 | O_anatinus | Animals | mono_intergenic | H/ACA | | | intergenic |
| ENSOANG00000046514 | ENSOANG00000046514 | O_anatinus | Animals | mono_intergenic | H/ACA | | | intergenic |
| ENSOANG00000046515 | ENSOANG00000046515 | O_anatinus | Animals | mono_intergenic | H/ACA | | | intergenic |
| ENSOANG00000046517 | ENSOANG00000046517 | O_anatinus | Animals | mono_intergenic | H/ACA | | | intergenic |
| ENSOANG00000046518 | ENSOANG00000046518 | O_anatinus | Animals | mono_intergenic | H/ACA | | | intergenic |
| ENSOANG00000046519 | ENSOANG00000046519 | O_anatinus | Animals | mono_intergenic | H/ACA | | | intergenic |
| ENSOANG00000046520 | ENSOANG00000046520 | O_anatinus | Animals | mono_intergenic | H/ACA | | | intergenic |
| ENSOANG00000046521 | ENSOANG00000046521 | O_anatinus | Animals | mono_intergenic | H/ACA | | | intergenic |
| ENSOANG00000046523 | ENSOANG00000046523 | O_anatinus | Animals | mono_intergenic | H/ACA | | | intergenic |
| ENSOANG00000046524 | ENSOANG00000046524 | O_anatinus | Animals | mono_intergenic | H/ACA | | | intergenic |
| ENSOANG00000046525 | ENSOANG00000046525 | O_anatinus | Animals | mono_intergenic | H/ACA | | | intergenic |
| ENSOANG00000046526 | ENSOANG00000046526 | O_anatinus | Animals | mono_intergenic | H/ACA | | | intergenic |
| ENSOANG00000046529 | ENSOANG00000046529 | O_anatinus | Animals | mono_intergenic | H/ACA | | | intergenic |
| ENSOANG00000046533 | ENSOANG00000046533 | O_anatinus | Animals | mono_intergenic | H/ACA | | | intergenic |
| ENSOANG00000046535 | ENSOANG00000046535 | O_anatinus | Animals | mono_intergenic | H/ACA | | | intergenic |
| ENSOANG00000046536 | ENSOANG00000046536 | O_anatinus | Animals | mono_intergenic | H/ACA | | | intergenic |
| ENSOANG00000046537 | ENSOANG00000046537 | O_anatinus | Animals | mono_intergenic | H/ACA | | | intergenic |
| ENSOANG00000046538 | ENSOANG00000046538 | O_anatinus | Animals | mono_intergenic | H/ACA | | | intergenic |

| | | | | | | | | |
|---|---|---|---|---|---|---|---|---|
| ENSOANG00000046540 | ENSOANG00000046540 | O_anatinus | Animals | mono_intronic | H/ACA | ENSOANG00000006103 | VPS13D | protein_coding |
| ENSOANG00000046541 | ENSOANG00000046541 | O_anatinus | Animals | mono_intergenic | H/ACA | | | intergenic |
| ENSOANG00000046543 | ENSOANG00000046543 | O_anatinus | Animals | mono_intergenic | H/ACA | | | intergenic |
| ENSOANG00000046549 | ENSOANG00000046549 | O_anatinus | Animals | mono_intergenic | H/ACA | | | intergenic |
| ENSOANG00000046550 | ENSOANG00000046550 | O_anatinus | Animals | mono_intergenic | H/ACA | | | intergenic |
| ENSOANG00000046551 | ENSOANG00000046551 | O_anatinus | Animals | mono_intergenic | H/ACA | | | intergenic |
| ENSOANG00000046552 | ENSOANG00000046552 | O_anatinus | Animals | mono_intergenic | H/ACA | | | intergenic |
| ENSOANG00000046555 | ENSOANG00000046555 | O_anatinus | Animals | mono_intronic | H/ACA | ENSOANG00000001908 | CLCN5 | protein_coding |
| ENSOANG00000046558 | ENSOANG00000046558 | O_anatinus | Animals | mono_intergenic | H/ACA | | | intergenic |
| ENSOANG00000046559 | ENSOANG00000046559 | O_anatinus | Animals | mono_intergenic | H/ACA | | | intergenic |
| ENSOANG00000046561 | ENSOANG00000046561 | O_anatinus | Animals | mono_intergenic | H/ACA | | | intergenic |
| ENSOANG00000046562 | ENSOANG00000046562 | O_anatinus | Animals | intronic_cluster | H/ACA | ENSOANG00000012559 | | protein_coding |
| ENSOANG00000046563 | ENSOANG00000046563 | O_anatinus | Animals | mono_intergenic | H/ACA | | | intergenic |
| ENSOANG00000046565 | ENSOANG00000046565 | O_anatinus | Animals | mono_intergenic | H/ACA | | | intergenic |
| ENSOANG00000046569 | ENSOANG00000046569 | O_anatinus | Animals | mono_intronic | H/ACA | ENSOANG00000002822 | SMOC2 | protein_coding |
| ENSOANG00000046570 | ENSOANG00000046570 | O_anatinus | Animals | mono_intergenic | H/ACA | | | intergenic |
| ENSOANG00000046572 | ENSOANG00000046572 | O_anatinus | Animals | mono_intergenic | H/ACA | | | intergenic |
| ENSOANG00000046574 | ENSOANG00000046574 | O_anatinus | Animals | mono_intergenic | H/ACA | | | intergenic |
| ENSOANG00000046576 | ENSOANG00000046576 | O_anatinus | Animals | mono_intergenic | H/ACA | | | intergenic |
| ENSOANG00000046577 | ENSOANG00000046577 | O_anatinus | Animals | mono_intronic | H/ACA | ENSOANG00000007620 | KLHL29 | protein_coding |
| ENSOANG00000046578 | ENSOANG00000046578 | O_anatinus | Animals | mono_intergenic | H/ACA | | | intergenic |
| ENSOANG00000046580 | ENSOANG00000046580 | O_anatinus | Animals | mono_intergenic | H/ACA | | | intergenic |
| ENSOANG00000046590 | ENSOANG00000046590 | O_anatinus | Animals | mono_intergenic | H/ACA | | | intergenic |
| ENSOANG00000046591 | ENSOANG00000046591 | O_anatinus | Animals | mono_intergenic | H/ACA | | | intergenic |
| ENSOANG00000046592 | ENSOANG00000046592 | O_anatinus | Animals | mono_intergenic | H/ACA | | | intergenic |
| ENSOANG00000046595 | ENSOANG00000046595 | O_anatinus | Animals | mono_intronic | H/ACA | ENSOANG00000007703 | FAM168B | protein_coding |
| ENSOANG00000046597 | ENSOANG00000046597 | O_anatinus | Animals | mono_intergenic | H/ACA | | | intergenic |
| ENSOANG00000046604 | ENSOANG00000046604 | O_anatinus | Animals | mono_intergenic | H/ACA | | | intergenic |
| ENSOANG00000046606 | ENSOANG00000046606 | O_anatinus | Animals | mono_intergenic | H/ACA | | | intergenic |
| ENSOANG00000046615 | ENSOANG00000046615 | O_anatinus | Animals | mono_intergenic | H/ACA | | | intergenic |
| ENSOANG00000046616 | ENSOANG00000046616 | O_anatinus | Animals | mono_intergenic | H/ACA | | | intergenic |
| ENSOANG00000046622 | ENSOANG00000046622 | O_anatinus | Animals | mono_intergenic | H/ACA | | | intergenic |
| ENSOANG00000046624 | ENSOANG00000046624 | O_anatinus | Animals | mono_intergenic | H/ACA | | | intergenic |
| ENSOANG00000046627 | ENSOANG00000046627 | O_anatinus | Animals | mono_intergenic | H/ACA | | | intergenic |
| ENSOANG00000046630 | ENSOANG00000046630 | O_anatinus | Animals | mono_intergenic | H/ACA | | | intergenic |
| ENSOANG00000046633 | ENSOANG00000046633 | O_anatinus | Animals | mono_intergenic | H/ACA | | | intergenic |
| ENSOANG00000046634 | ENSOANG00000046634 | O_anatinus | Animals | mono_intergenic | H/ACA | | | intergenic |
| ENSOANG00000046636 | ENSOANG00000046636 | O_anatinus | Animals | mono_intergenic | H/ACA | | | intergenic |
| ENSOANG00000046638 | ENSOANG00000046638 | O_anatinus | Animals | mono_intergenic | H/ACA | | | intergenic |
| ENSOANG00000046639 | ENSOANG00000046639 | O_anatinus | Animals | mono_intergenic | H/ACA | | | intergenic |
| ENSOANG00000046642 | ENSOANG00000046642 | O_anatinus | Animals | mono_intergenic | H/ACA | | | intergenic |

| | | | | | | | | |
|---|---|---|---|---|---|---|---|---|
| ENSOANG00000046646 | ENSOANG00000046646 | O_anatinus | Animals | mono_intergenic | H/ACA | | | intergenic |
| ENSOANG00000046647 | ENSOANG00000046647 | O_anatinus | Animals | mono_intergenic | H/ACA | | | intergenic |
| ENSOANG00000046648 | ENSOANG00000046648 | O_anatinus | Animals | mono_intergenic | H/ACA | | | intergenic |
| ENSOANG00000046649 | ENSOANG00000046649 | O_anatinus | Animals | mono_intergenic | H/ACA | | | intergenic |
| ENSOANG00000046650 | ENSOANG00000046650 | O_anatinus | Animals | mono_intergenic | H/ACA | | | intergenic |
| ENSOANG00000046654 | ENSOANG00000046654 | O_anatinus | Animals | mono_intergenic | H/ACA | | | intergenic |
| ENSOANG00000046656 | ENSOANG00000046656 | O_anatinus | Animals | mono_intronic | H/ACA | ENSOANG00000008694 | BRD4 | protein_coding |
| ENSOANG00000046657 | ENSOANG00000046657 | O_anatinus | Animals | mono_intergenic | H/ACA | | | intergenic |
| ENSOANG00000046659 | ENSOANG00000046659 | O_anatinus | Animals | mono_intergenic | H/ACA | | | intergenic |
| ENSOANG00000046661 | ENSOANG00000046661 | O_anatinus | Animals | mono_intergenic | H/ACA | | | intergenic |
| ENSOANG00000046668 | ENSOANG00000046668 | O_anatinus | Animals | mono_intronic | H/ACA | ENSOANG00000046302 | | protein_coding |
| ENSOANG00000046671 | ENSOANG00000046671 | O_anatinus | Animals | mono_intronic | H/ACA | ENSOANG00000001913 | RUNX1T1 | protein_coding |
| ENSOANG00000046672 | ENSOANG00000046672 | O_anatinus | Animals | mono_intergenic | H/ACA | | | intergenic |
| ENSOANG00000046674 | ENSOANG00000046674 | O_anatinus | Animals | mono_intergenic | H/ACA | | | intergenic |
| ENSOANG00000046681 | ENSOANG00000046681 | O_anatinus | Animals | mono_intergenic | H/ACA | | | intergenic |
| ENSOANG00000046682 | ENSOANG00000046682 | O_anatinus | Animals | mono_intergenic | H/ACA | | | intergenic |
| ENSOANG00000046684 | ENSOANG00000046684 | O_anatinus | Animals | mono_intergenic | H/ACA | | | intergenic |
| ENSOANG00000046686 | SNORD100 | O_anatinus | Animals | mono_intronic | C/D | ENSOANG00000001886 | RPS12 | protein_coding |
| ENSOANG00000046687 | ENSOANG00000046687 | O_anatinus | Animals | mono_intergenic | H/ACA | | | intergenic |
| ENSOANG00000046688 | ENSOANG00000046688 | O_anatinus | Animals | mono_intronic | H/ACA | ENSOANG00000001882 | SLC19A3 | protein_coding |
| ENSOANG00000046690 | ENSOANG00000046690 | O_anatinus | Animals | mono_intergenic | H/ACA | | | intergenic |
| ENSOANG00000046691 | ENSOANG00000046691 | O_anatinus | Animals | mono_intergenic | H/ACA | | | intergenic |
| ENSOANG00000046693 | ENSOANG00000046693 | O_anatinus | Animals | mono_intergenic | H/ACA | | | intergenic |
| ENSOANG00000046698 | ENSOANG00000046698 | O_anatinus | Animals | mono_intronic | H/ACA | ENSOANG00000003340 | SH3GL2 | protein_coding |
| ENSOANG00000046699 | ENSOANG00000046699 | O_anatinus | Animals | mono_intergenic | H/ACA | | | intergenic |
| ENSOANG00000046704 | ENSOANG00000046704 | O_anatinus | Animals | mono_intronic | H/ACA | ENSOANG00000015366 | TBRG4 | protein_coding |
| ENSOANG00000046706 | ENSOANG00000046706 | O_anatinus | Animals | mono_intergenic | H/ACA | | | intergenic |
| ENSOANG00000046707 | ENSOANG00000046707 | O_anatinus | Animals | mono_intergenic | H/ACA | | | intergenic |
| ENSOANG00000046708 | ENSOANG00000046708 | O_anatinus | Animals | mono_intergenic | H/ACA | | | intergenic |
| ENSOANG00000046711 | ENSOANG00000046711 | O_anatinus | Animals | mono_intergenic | H/ACA | | | intergenic |
| ENSOANG00000046715 | ENSOANG00000046715 | O_anatinus | Animals | mono_intergenic | H/ACA | | | intergenic |
| ENSOANG00000046716 | ENSOANG00000046716 | O_anatinus | Animals | mono_intergenic | H/ACA | | | intergenic |
| ENSOANG00000046719 | ENSOANG00000046719 | O_anatinus | Animals | mono_intergenic | H/ACA | | | intergenic |
| ENSOANG00000046731 | ENSOANG00000046731 | O_anatinus | Animals | mono_intergenic | H/ACA | | | intergenic |
| ENSOANG00000046743 | ENSOANG00000046743 | O_anatinus | Animals | mono_intergenic | H/ACA | | | intergenic |
| ENSOANG00000046744 | ENSOANG00000046744 | O_anatinus | Animals | mono_intergenic | H/ACA | | | intergenic |
| ENSOANG00000046745 | ENSOANG00000046745 | O_anatinus | Animals | mono_intronic | H/ACA | ENSOANG00000047423 | ARHGAP32 | protein_coding |
| ENSOANG00000046746 | ENSOANG00000046746 | O_anatinus | Animals | mono_intergenic | H/ACA | | | intergenic |
| ENSOANG00000046751 | ENSOANG00000046751 | O_anatinus | Animals | mono_intergenic | H/ACA | | | intergenic |
| ENSOANG00000046753 | ENSOANG00000046753 | O_anatinus | Animals | mono_intronic | H/ACA | ENSOANG00000001492 | PTPRD | protein_coding |
| ENSOANG00000046754 | ENSOANG00000046754 | O_anatinus | Animals | mono_intergenic | H/ACA | | | intergenic |

| | | | | | | | | |
|---|---|---|---|---|---|---|---|---|
| ENSOANG00000046755 | ENSOANG00000046755 | O_anatinus | Animals | mono_intergenic | H/ACA | | | intergenic |
| ENSOANG00000046760 | ENSOANG00000046760 | O_anatinus | Animals | mono_intergenic | H/ACA | | | intergenic |
| ENSOANG00000046762 | ENSOANG00000046762 | O_anatinus | Animals | mono_intronic | H/ACA | ENSOANG00000007787 | | protein_coding |
| ENSOANG00000046763 | ENSOANG00000046763 | O_anatinus | Animals | mono_intergenic | H/ACA | | | intergenic |
| ENSOANG00000046768 | ENSOANG00000046768 | O_anatinus | Animals | mono_intergenic | H/ACA | | | intergenic |
| ENSOANG00000046770 | ENSOANG00000046770 | O_anatinus | Animals | mono_intergenic | H/ACA | | | intergenic |
| ENSOANG00000046771 | ENSOANG00000046771 | O_anatinus | Animals | mono_intergenic | H/ACA | | | intergenic |
| ENSOANG00000046772 | ENSOANG00000046772 | O_anatinus | Animals | mono_intergenic | H/ACA | | | intergenic |
| ENSOANG00000046775 | ENSOANG00000046775 | O_anatinus | Animals | mono_intergenic | H/ACA | | | intergenic |
| ENSOANG00000046776 | ENSOANG00000046776 | O_anatinus | Animals | mono_intronic | H/ACA | ENSOANG00000011714 | ABHD2 | protein_coding |
| ENSOANG00000046777 | ENSOANG00000046777 | O_anatinus | Animals | mono_intergenic | H/ACA | | | intergenic |
| ENSOANG00000046778 | ENSOANG00000046778 | O_anatinus | Animals | mono_intergenic | H/ACA | | | intergenic |
| ENSOANG00000046780 | ENSOANG00000046780 | O_anatinus | Animals | mono_intronic | H/ACA | ENSOANG00000015092 | ARHGAP40 | protein_coding |
| ENSOANG00000046782 | ENSOANG00000046782 | O_anatinus | Animals | intronic_cluster | H/ACA | ENSOANG00000029848 | | protein_coding |
| ENSOANG00000046786 | ENSOANG00000046786 | O_anatinus | Animals | mono_intergenic | H/ACA | | | intergenic |
| ENSOANG00000046794 | ENSOANG00000046794 | O_anatinus | Animals | mono_intergenic | H/ACA | | | intergenic |
| ENSOANG00000046796 | ENSOANG00000046796 | O_anatinus | Animals | mono_intergenic | H/ACA | | | intergenic |
| ENSOANG00000046798 | ENSOANG00000046798 | O_anatinus | Animals | mono_intergenic | H/ACA | | | intergenic |
| ENSOANG00000046799 | ENSOANG00000046799 | O_anatinus | Animals | mono_intergenic | H/ACA | | | intergenic |
| ENSOANG00000046800 | ENSOANG00000046800 | O_anatinus | Animals | mono_intergenic | H/ACA | | | intergenic |
| ENSOANG00000046802 | ENSOANG00000046802 | O_anatinus | Animals | mono_intergenic | H/ACA | | | intergenic |
| ENSOANG00000046803 | ENSOANG00000046803 | O_anatinus | Animals | mono_intergenic | H/ACA | | | intergenic |
| ENSOANG00000046807 | ENSOANG00000046807 | O_anatinus | Animals | mono_intronic | H/ACA | ENSOANG00000006938 | RCC2 | protein_coding |
| ENSOANG00000046808 | ENSOANG00000046808 | O_anatinus | Animals | mono_intergenic | H/ACA | | | intergenic |
| ENSOANG00000046810 | ENSOANG00000046810 | O_anatinus | Animals | mono_intergenic | H/ACA | | | intergenic |
| ENSOANG00000046811 | ENSOANG00000046811 | O_anatinus | Animals | mono_intergenic | H/ACA | | | intergenic |
| ENSOANG00000046812 | ENSOANG00000046812 | O_anatinus | Animals | mono_intergenic | H/ACA | | | intergenic |
| ENSOANG00000046815 | ENSOANG00000046815 | O_anatinus | Animals | mono_intergenic | H/ACA | | | intergenic |
| ENSOANG00000046818 | ENSOANG00000046818 | O_anatinus | Animals | mono_intergenic | H/ACA | | | intergenic |
| ENSOANG00000046819 | ENSOANG00000046819 | O_anatinus | Animals | mono_intergenic | H/ACA | | | intergenic |
| ENSOANG00000046822 | ENSOANG00000046822 | O_anatinus | Animals | mono_intergenic | H/ACA | | | intergenic |
| ENSOANG00000046827 | ENSOANG00000046827 | O_anatinus | Animals | mono_intergenic | H/ACA | | | intergenic |
| ENSOANG00000046829 | ENSOANG00000046829 | O_anatinus | Animals | mono_intergenic | H/ACA | | | intergenic |
| ENSOANG00000046831 | ENSOANG00000046831 | O_anatinus | Animals | mono_intergenic | H/ACA | | | intergenic |
| ENSOANG00000046834 | ENSOANG00000046834 | O_anatinus | Animals | mono_intergenic | H/ACA | | | intergenic |
| ENSOANG00000046835 | ENSOANG00000046835 | O_anatinus | Animals | mono_intergenic | H/ACA | | | intergenic |
| ENSOANG00000046836 | ENSOANG00000046836 | O_anatinus | Animals | mono_intergenic | H/ACA | | | intergenic |
| ENSOANG00000046837 | ENSOANG00000046837 | O_anatinus | Animals | mono_intronic | H/ACA | ENSOANG00000041210 | ATP6V0D1 | protein_coding |
| ENSOANG00000046838 | ENSOANG00000046838 | O_anatinus | Animals | intronic_cluster | H/ACA | ENSOANG00000043336 | | protein_coding |
| ENSOANG00000046843 | ENSOANG00000046843 | O_anatinus | Animals | mono_intergenic | H/ACA | | | intergenic |
| ENSOANG00000046846 | ENSOANG00000046846 | O_anatinus | Animals | mono_intergenic | H/ACA | | | intergenic |

| | | | | | | | | |
|---|---|---|---|---|---|---|---|---|
| ENSOANG00000046850 | ENSOANG00000046850 | O_anatinus | Animals | mono_intergenic | H/ACA | | | intergenic |
| ENSOANG00000046851 | ENSOANG00000046851 | O_anatinus | Animals | mono_intergenic | H/ACA | | | intergenic |
| ENSOANG00000046852 | ENSOANG00000046852 | O_anatinus | Animals | mono_intergenic | H/ACA | | | intergenic |
| ENSOANG00000046859 | ENSOANG00000046859 | O_anatinus | Animals | mono_intergenic | H/ACA | | | intergenic |
| ENSOANG00000046863 | ENSOANG00000046863 | O_anatinus | Animals | mono_intergenic | H/ACA | | | intergenic |
| ENSOANG00000046866 | ENSOANG00000046866 | O_anatinus | Animals | mono_intergenic | H/ACA | | | intergenic |
| ENSOANG00000046870 | ENSOANG00000046870 | O_anatinus | Animals | intergenic_cluster | H/ACA | | | intergenic |
| ENSOANG00000046873 | ENSOANG00000046873 | O_anatinus | Animals | mono_intergenic | H/ACA | | | intergenic |
| ENSOANG00000046877 | ENSOANG00000046877 | O_anatinus | Animals | mono_intergenic | H/ACA | | | intergenic |
| ENSOANG00000046879 | ENSOANG00000046879 | O_anatinus | Animals | mono_intergenic | H/ACA | | | intergenic |
| ENSOANG00000046882 | ENSOANG00000046882 | O_anatinus | Animals | mono_intergenic | H/ACA | | | intergenic |
| ENSOANG00000046884 | ENSOANG00000046884 | O_anatinus | Animals | mono_intergenic | H/ACA | | | intergenic |
| ENSOANG00000046886 | ENSOANG00000046886 | O_anatinus | Animals | mono_intergenic | H/ACA | | | intergenic |
| ENSOANG00000046896 | ENSOANG00000046896 | O_anatinus | Animals | mono_intergenic | H/ACA | | | intergenic |
| ENSOANG00000046897 | ENSOANG00000046897 | O_anatinus | Animals | mono_intergenic | H/ACA | | | intergenic |
| ENSOANG00000046902 | ENSOANG00000046902 | O_anatinus | Animals | mono_intergenic | H/ACA | | | intergenic |
| ENSOANG00000046903 | ENSOANG00000046903 | O_anatinus | Animals | mono_intergenic | H/ACA | | | intergenic |
| ENSOANG00000046904 | ENSOANG00000046904 | O_anatinus | Animals | mono_intronic | H/ACA | ENSOANG00000011608 | PDE1C | protein_coding |
| ENSOANG00000046906 | ENSOANG00000046906 | O_anatinus | Animals | mono_intergenic | H/ACA | | | intergenic |
| ENSOANG00000046907 | ENSOANG00000046907 | O_anatinus | Animals | mono_intergenic | H/ACA | | | intergenic |
| ENSOANG00000046908 | ENSOANG00000046908 | O_anatinus | Animals | mono_intergenic | H/ACA | | | intergenic |
| ENSOANG00000046909 | ENSOANG00000046909 | O_anatinus | Animals | mono_intergenic | H/ACA | | | intergenic |
| ENSOANG00000046915 | ENSOANG00000046915 | O_anatinus | Animals | mono_intergenic | H/ACA | | | intergenic |
| ENSOANG00000046917 | ENSOANG00000046917 | O_anatinus | Animals | mono_intergenic | H/ACA | | | intergenic |
| ENSOANG00000046918 | ENSOANG00000046918 | O_anatinus | Animals | mono_intergenic | H/ACA | | | intergenic |
| ENSOANG00000046919 | ENSOANG00000046919 | O_anatinus | Animals | mono_intronic | H/ACA | ENSOANG00000012491 | ADARB1 | protein_coding |
| ENSOANG00000046920 | ENSOANG00000046920 | O_anatinus | Animals | mono_intronic | H/ACA | ENSOANG00000011183 | F13B | protein_coding |
| ENSOANG00000046925 | ENSOANG00000046925 | O_anatinus | Animals | mono_intergenic | H/ACA | | | intergenic |
| ENSOANG00000046928 | ENSOANG00000046928 | O_anatinus | Animals | mono_intergenic | H/ACA | | | intergenic |
| ENSOANG00000046930 | ENSOANG00000046930 | O_anatinus | Animals | mono_intronic | H/ACA | ENSOANG00000014759 | EDIL3 | protein_coding |
| ENSOANG00000046931 | ENSOANG00000046931 | O_anatinus | Animals | mono_intergenic | H/ACA | | | intergenic |
| ENSOANG00000046932 | ENSOANG00000046932 | O_anatinus | Animals | mono_intergenic | H/ACA | | | intergenic |
| ENSOANG00000046936 | ENSOANG00000046936 | O_anatinus | Animals | mono_intergenic | H/ACA | | | intergenic |
| ENSOANG00000046937 | ENSOANG00000046937 | O_anatinus | Animals | mono_intergenic | H/ACA | | | intergenic |
| ENSOANG00000046938 | ENSOANG00000046938 | O_anatinus | Animals | mono_intergenic | H/ACA | | | intergenic |
| ENSOANG00000046939 | ENSOANG00000046939 | O_anatinus | Animals | mono_intergenic | H/ACA | | | intergenic |
| ENSOANG00000046940 | ENSOANG00000046940 | O_anatinus | Animals | mono_intergenic | H/ACA | | | intergenic |
| ENSOANG00000046942 | ENSOANG00000046942 | O_anatinus | Animals | mono_intergenic | H/ACA | | | intergenic |
| ENSOANG00000046945 | ENSOANG00000046945 | O_anatinus | Animals | mono_intronic | H/ACA | ENSOANG00000009050 | CDK8 | protein_coding |
| ENSOANG00000046947 | ENSOANG00000046947 | O_anatinus | Animals | mono_intergenic | H/ACA | | | intergenic |
| ENSOANG00000046950 | ENSOANG00000046950 | O_anatinus | Animals | mono_intergenic | H/ACA | | | intergenic |

| | | | | | | | | |
|---|---|---|---|---|---|---|---|---|
| ENSOANG00000046951 | ENSOANG00000046951 | O_anatinus | Animals | mono_intergenic | H/ACA | | | intergenic |
| ENSOANG00000046952 | ENSOANG00000046952 | O_anatinus | Animals | mono_intergenic | H/ACA | | | intergenic |
| ENSOANG00000046955 | ENSOANG00000046955 | O_anatinus | Animals | mono_intergenic | H/ACA | | | intergenic |
| ENSOANG00000046958 | ENSOANG00000046958 | O_anatinus | Animals | mono_intergenic | H/ACA | | | intergenic |
| ENSOANG00000046960 | ENSOANG00000046960 | O_anatinus | Animals | mono_intergenic | H/ACA | | | intergenic |
| ENSOANG00000046961 | ENSOANG00000046961 | O_anatinus | Animals | mono_intronic | C/D | ENSOANG00000014467 | WDR43 | protein_coding |
| ENSOANG00000046962 | ENSOANG00000046962 | O_anatinus | Animals | mono_intergenic | H/ACA | | | intergenic |
| ENSOANG00000046963 | ENSOANG00000046963 | O_anatinus | Animals | mono_intergenic | H/ACA | | | intergenic |
| ENSOANG00000046965 | ENSOANG00000046965 | O_anatinus | Animals | mono_intergenic | H/ACA | | | intergenic |
| ENSOANG00000046967 | ENSOANG00000046967 | O_anatinus | Animals | mono_intronic | H/ACA | ENSOANG00000049164 | | non_coding |
| ENSOANG00000046969 | ENSOANG00000046969 | O_anatinus | Animals | mono_intergenic | H/ACA | | | intergenic |
| ENSOANG00000046971 | ENSOANG00000046971 | O_anatinus | Animals | mono_intergenic | H/ACA | | | intergenic |
| ENSOANG00000046974 | ENSOANG00000046974 | O_anatinus | Animals | mono_intergenic | H/ACA | | | intergenic |
| ENSOANG00000046975 | ENSOANG00000046975 | O_anatinus | Animals | mono_intergenic | H/ACA | | | intergenic |
| ENSOANG00000046976 | ENSOANG00000046976 | O_anatinus | Animals | mono_intronic | H/ACA | ENSOANG00000040340 | IMMP2L | protein_coding |
| ENSOANG00000046977 | ENSOANG00000046977 | O_anatinus | Animals | mono_intergenic | H/ACA | | | intergenic |
| ENSOANG00000046978 | ENSOANG00000046978 | O_anatinus | Animals | mono_intergenic | H/ACA | | | intergenic |
| ENSOANG00000046981 | ENSOANG00000046981 | O_anatinus | Animals | mono_intergenic | H/ACA | | | intergenic |
| ENSOANG00000046983 | ENSOANG00000046983 | O_anatinus | Animals | mono_intergenic | H/ACA | | | intergenic |
| ENSOANG00000046988 | ENSOANG00000046988 | O_anatinus | Animals | mono_intergenic | H/ACA | | | intergenic |
| ENSOANG00000046989 | ENSOANG00000046989 | O_anatinus | Animals | mono_intronic | H/ACA | ENSOANG00000050672 | LHFPL3 | protein_coding |
| ENSOANG00000046991 | ENSOANG00000046991 | O_anatinus | Animals | mono_intergenic | H/ACA | | | intergenic |
| ENSOANG00000046992 | ENSOANG00000046992 | O_anatinus | Animals | mono_intergenic | H/ACA | | | intergenic |
| ENSOANG00000046995 | ENSOANG00000046995 | O_anatinus | Animals | mono_intergenic | H/ACA | | | intergenic |
| ENSOANG00000046998 | ENSOANG00000046998 | O_anatinus | Animals | mono_intergenic | H/ACA | | | intergenic |
| ENSOANG00000047000 | ENSOANG00000047000 | O_anatinus | Animals | mono_intergenic | H/ACA | | | intergenic |
| ENSOANG00000047001 | ENSOANG00000047001 | O_anatinus | Animals | mono_intergenic | H/ACA | | | intergenic |
| ENSOANG00000047004 | ENSOANG00000047004 | O_anatinus | Animals | mono_intergenic | H/ACA | | | intergenic |
| ENSOANG00000047015 | ENSOANG00000047015 | O_anatinus | Animals | mono_intronic | H/ACA | ENSOANG00000002718 | AKAP13 | protein_coding |
| ENSOANG00000047018 | SNORA73 | O_anatinus | Animals | intergenic_cluster | H/ACA | | | intergenic |
| ENSOANG00000047020 | ENSOANG00000047020 | O_anatinus | Animals | mono_intergenic | H/ACA | | | intergenic |
| ENSOANG00000047021 | ENSOANG00000047021 | O_anatinus | Animals | mono_intergenic | H/ACA | | | intergenic |
| ENSOANG00000047023 | ENSOANG00000047023 | O_anatinus | Animals | mono_intergenic | H/ACA | | | intergenic |
| ENSOANG00000047024 | ENSOANG00000047024 | O_anatinus | Animals | mono_intergenic | H/ACA | | | intergenic |
| ENSOANG00000047026 | ENSOANG00000047026 | O_anatinus | Animals | mono_intergenic | H/ACA | | | intergenic |
| ENSOANG00000047027 | ENSOANG00000047027 | O_anatinus | Animals | mono_intergenic | H/ACA | | | intergenic |
| ENSOANG00000047028 | ENSOANG00000047028 | O_anatinus | Animals | mono_intergenic | H/ACA | | | intergenic |
| ENSOANG00000047031 | ENSOANG00000047031 | O_anatinus | Animals | mono_intergenic | H/ACA | | | intergenic |
| ENSOANG00000047032 | ENSOANG00000047032 | O_anatinus | Animals | mono_intergenic | H/ACA | | | intergenic |
| ENSOANG00000047033 | ENSOANG00000047033 | O_anatinus | Animals | mono_intergenic | H/ACA | | | intergenic |
| ENSOANG00000047035 | ENSOANG00000047035 | O_anatinus | Animals | mono_intergenic | H/ACA | | | intergenic |

| | | | | | | | | |
|---|---|---|---|---|---|---|---|---|
| ENSOANG00000047043 | ENSOANG00000047043 | O_anatinus | Animals | mono_intergenic | H/ACA | | | intergenic |
| ENSOANG00000047045 | ENSOANG00000047045 | O_anatinus | Animals | mono_exonic | H/ACA | ENSOANG00000050217 | | non_coding |
| ENSOANG00000047046 | ENSOANG00000047046 | O_anatinus | Animals | mono_intergenic | H/ACA | | | intergenic |
| ENSOANG00000047050 | ENSOANG00000047050 | O_anatinus | Animals | mono_intergenic | H/ACA | | | intergenic |
| ENSOANG00000047052 | ENSOANG00000047052 | O_anatinus | Animals | mono_intronic | H/ACA | ENSOANG00000036747 | VPS8 | protein_coding |
| ENSOANG00000047053 | ENSOANG00000047053 | O_anatinus | Animals | mono_intergenic | H/ACA | | | intergenic |
| ENSOANG00000047056 | ENSOANG00000047056 | O_anatinus | Animals | mono_intergenic | H/ACA | | | intergenic |
| ENSOANG00000047059 | ENSOANG00000047059 | O_anatinus | Animals | mono_intergenic | H/ACA | | | intergenic |
| ENSOANG00000047063 | ENSOANG00000047063 | O_anatinus | Animals | mono_intergenic | H/ACA | | | intergenic |
| ENSOANG00000047067 | ENSOANG00000047067 | O_anatinus | Animals | mono_intergenic | H/ACA | | | intergenic |
| ENSOANG00000047068 | ENSOANG00000047068 | O_anatinus | Animals | mono_intergenic | H/ACA | | | intergenic |
| ENSOANG00000047069 | ENSOANG00000047069 | O_anatinus | Animals | mono_intergenic | H/ACA | | | intergenic |
| ENSOANG00000047074 | ENSOANG00000047074 | O_anatinus | Animals | mono_intergenic | H/ACA | | | intergenic |
| ENSOANG00000047075 | ENSOANG00000047075 | O_anatinus | Animals | mono_intergenic | H/ACA | | | intergenic |
| ENSOANG00000047080 | ENSOANG00000047080 | O_anatinus | Animals | mono_intergenic | H/ACA | | | intergenic |
| ENSOANG00000047081 | ENSOANG00000047081 | O_anatinus | Animals | mono_intergenic | H/ACA | | | intergenic |
| ENSOANG00000047083 | ENSOANG00000047083 | O_anatinus | Animals | mono_intergenic | H/ACA | | | intergenic |
| ENSOANG00000047087 | ENSOANG00000047087 | O_anatinus | Animals | mono_intergenic | H/ACA | | | intergenic |
| ENSOANG00000047098 | ENSOANG00000047098 | O_anatinus | Animals | mono_intergenic | H/ACA | | | intergenic |
| ENSOANG00000047100 | ENSOANG00000047100 | O_anatinus | Animals | mono_intergenic | H/ACA | | | intergenic |
| ENSOANG00000047107 | ENSOANG00000047107 | O_anatinus | Animals | mono_intergenic | H/ACA | | | intergenic |
| ENSOANG00000047109 | ENSOANG00000047109 | O_anatinus | Animals | mono_intergenic | H/ACA | | | intergenic |
| ENSOANG00000047111 | ENSOANG00000047111 | O_anatinus | Animals | mono_intergenic | H/ACA | | | intergenic |
| ENSOANG00000047113 | ENSOANG00000047113 | O_anatinus | Animals | mono_intergenic | H/ACA | | | intergenic |
| ENSOANG00000047115 | U3 | O_anatinus | Animals | mono_intergenic | C/D | | | intergenic |
| ENSOANG00000047118 | ENSOANG00000047118 | O_anatinus | Animals | mono_intergenic | H/ACA | | | intergenic |
| ENSOANG00000047124 | ENSOANG00000047124 | O_anatinus | Animals | mono_intergenic | H/ACA | | | intergenic |
| ENSOANG00000047126 | ENSOANG00000047126 | O_anatinus | Animals | mono_intergenic | H/ACA | | | intergenic |
| ENSOANG00000047127 | ENSOANG00000047127 | O_anatinus | Animals | mono_intergenic | H/ACA | | | intergenic |
| ENSOANG00000047130 | ENSOANG00000047130 | O_anatinus | Animals | mono_intergenic | H/ACA | | | intergenic |
| ENSOANG00000047131 | ENSOANG00000047131 | O_anatinus | Animals | mono_intergenic | H/ACA | | | intergenic |
| ENSOANG00000047132 | ENSOANG00000047132 | O_anatinus | Animals | mono_intergenic | H/ACA | | | intergenic |
| ENSOANG00000047135 | ENSOANG00000047135 | O_anatinus | Animals | mono_intergenic | H/ACA | | | intergenic |
| ENSOANG00000047137 | ENSOANG00000047137 | O_anatinus | Animals | mono_intergenic | H/ACA | | | intergenic |
| ENSOANG00000047138 | ENSOANG00000047138 | O_anatinus | Animals | mono_intergenic | H/ACA | | | intergenic |
| ENSOANG00000047140 | ENSOANG00000047140 | O_anatinus | Animals | mono_intergenic | H/ACA | | | intergenic |
| ENSOANG00000047144 | ENSOANG00000047144 | O_anatinus | Animals | mono_intergenic | H/ACA | | | intergenic |
| ENSOANG00000047146 | ENSOANG00000047146 | O_anatinus | Animals | mono_intergenic | H/ACA | | | intergenic |
| ENSOANG00000047150 | ENSOANG00000047150 | O_anatinus | Animals | mono_intergenic | H/ACA | | | intergenic |
| ENSOANG00000047152 | ENSOANG00000047152 | O_anatinus | Animals | mono_intronic | H/ACA | ENSOANG00000043203 | ADCY1 | protein_coding |
| ENSOANG00000047156 | ENSOANG00000047156 | O_anatinus | Animals | mono_intergenic | H/ACA | | | intergenic |

| | | | | | | | | |
|---|---|---|---|---|---|---|---|---|
| ENSOANG00000047157 | ENSOANG00000047157 | O_anatinus | Animals | mono_intergenic | H/ACA | | | intergenic |
| ENSOANG00000047158 | ENSOANG00000047158 | O_anatinus | Animals | mono_intergenic | H/ACA | | | intergenic |
| ENSOANG00000047161 | ENSOANG00000047161 | O_anatinus | Animals | mono_intergenic | H/ACA | | | intergenic |
| ENSOANG00000047164 | ENSOANG00000047164 | O_anatinus | Animals | mono_intergenic | H/ACA | | | intergenic |
| ENSOANG00000047165 | ENSOANG00000047165 | O_anatinus | Animals | mono_intergenic | H/ACA | | | intergenic |
| ENSOANG00000047167 | ENSOANG00000047167 | O_anatinus | Animals | mono_intergenic | H/ACA | | | intergenic |
| ENSOANG00000047170 | ENSOANG00000047170 | O_anatinus | Animals | mono_intergenic | H/ACA | | | intergenic |
| ENSOANG00000047173 | ENSOANG00000047173 | O_anatinus | Animals | intronic_cluster | H/ACA | ENSOANG00000012629 | | protein_coding |
| ENSOANG00000047174 | ENSOANG00000047174 | O_anatinus | Animals | mono_intergenic | H/ACA | | | intergenic |
| ENSOANG00000047175 | ENSOANG00000047175 | O_anatinus | Animals | mono_intergenic | H/ACA | | | intergenic |
| ENSOANG00000047176 | ENSOANG00000047176 | O_anatinus | Animals | mono_intergenic | H/ACA | | | intergenic |
| ENSOANG00000047182 | ENSOANG00000047182 | O_anatinus | Animals | mono_intronic | H/ACA | ENSOANG00000047582 | | non_coding |
| ENSOANG00000047183 | ENSOANG00000047183 | O_anatinus | Animals | mono_intergenic | H/ACA | | | intergenic |
| ENSOANG00000047184 | ENSOANG00000047184 | O_anatinus | Animals | mono_intergenic | H/ACA | | | intergenic |
| ENSOANG00000047187 | ENSOANG00000047187 | O_anatinus | Animals | mono_intergenic | H/ACA | | | intergenic |
| ENSOANG00000047190 | ENSOANG00000047190 | O_anatinus | Animals | mono_intergenic | H/ACA | | | intergenic |
| ENSOANG00000047191 | ENSOANG00000047191 | O_anatinus | Animals | mono_intergenic | H/ACA | | | intergenic |
| ENSOANG00000047194 | ENSOANG00000047194 | O_anatinus | Animals | mono_intergenic | H/ACA | | | intergenic |
| ENSOANG00000047196 | ENSOANG00000047196 | O_anatinus | Animals | mono_intergenic | H/ACA | | | intergenic |
| ENSOANG00000047197 | ENSOANG00000047197 | O_anatinus | Animals | mono_intronic | H/ACA | ENSOANG00000003513 | MCOLN2 | protein_coding |
| ENSOANG00000047198 | ENSOANG00000047198 | O_anatinus | Animals | mono_intergenic | H/ACA | | | intergenic |
| ENSOANG00000047204 | ENSOANG00000047204 | O_anatinus | Animals | mono_intergenic | H/ACA | | | intergenic |
| ENSOANG00000047212 | ENSOANG00000047212 | O_anatinus | Animals | mono_intergenic | H/ACA | | | intergenic |
| ENSOANG00000047218 | ENSOANG00000047218 | O_anatinus | Animals | mono_intronic | H/ACA | ENSOANG00000038522 | PROK1 | protein_coding |
| ENSOANG00000047219 | ENSOANG00000047219 | O_anatinus | Animals | mono_intronic | H/ACA | ENSOANG00000050223 | KY | protein_coding |
| ENSOANG00000047222 | ENSOANG00000047222 | O_anatinus | Animals | mono_intergenic | H/ACA | | | intergenic |
| ENSOANG00000047228 | ENSOANG00000047228 | O_anatinus | Animals | mono_intergenic | H/ACA | | | intergenic |
| ENSOANG00000047234 | ENSOANG00000047234 | O_anatinus | Animals | mono_intergenic | H/ACA | | | intergenic |
| ENSOANG00000047235 | ENSOANG00000047235 | O_anatinus | Animals | mono_intronic | H/ACA | ENSOANG00000005256 | | protein_coding |
| ENSOANG00000047236 | ENSOANG00000047236 | O_anatinus | Animals | mono_intergenic | H/ACA | | | intergenic |
| ENSOANG00000047238 | ENSOANG00000047238 | O_anatinus | Animals | mono_intergenic | H/ACA | | | intergenic |
| ENSOANG00000047244 | ENSOANG00000047244 | O_anatinus | Animals | mono_intergenic | H/ACA | | | intergenic |
| ENSOANG00000047245 | ENSOANG00000047245 | O_anatinus | Animals | mono_intergenic | H/ACA | | | intergenic |
| ENSOANG00000047247 | ENSOANG00000047247 | O_anatinus | Animals | intergenic_cluster | H/ACA | | | intergenic |
| ENSOANG00000047251 | ENSOANG00000047251 | O_anatinus | Animals | mono_intergenic | H/ACA | | | intergenic |
| ENSOANG00000047252 | ENSOANG00000047252 | O_anatinus | Animals | mono_intergenic | H/ACA | | | intergenic |
| ENSOANG00000047256 | ENSOANG00000047256 | O_anatinus | Animals | mono_intergenic | H/ACA | | | intergenic |
| ENSOANG00000047257 | ENSOANG00000047257 | O_anatinus | Animals | mono_intergenic | H/ACA | | | intergenic |
| ENSOANG00000047258 | ENSOANG00000047258 | O_anatinus | Animals | mono_intronic | H/ACA | ENSOANG00000013415 | AGAP1 | protein_coding |
| ENSOANG00000047262 | ENSOANG00000047262 | O_anatinus | Animals | mono_intronic | H/ACA | ENSOANG00000002822 | SMOC2 | protein_coding |
| ENSOANG00000047263 | ENSOANG00000047263 | O_anatinus | Animals | mono_intergenic | H/ACA | | | intergenic |

| | | | | | | | | |
|---|---|---|---|---|---|---|---|---|
| ENSOANG00000047268 | ENSOANG00000047268 | O_anatinus | Animals | mono_intronic | H/ACA | ENSOANG00000038709 | NCOA2 | protein_coding |
| ENSOANG00000047269 | ENSOANG00000047269 | O_anatinus | Animals | mono_intergenic | H/ACA | | | intergenic |
| ENSOANG00000047273 | ENSOANG00000047273 | O_anatinus | Animals | mono_intergenic | H/ACA | | | intergenic |
| ENSOANG00000047274 | ENSOANG00000047274 | O_anatinus | Animals | mono_intronic | H/ACA | ENSOANG00000048909 | C9orf85 | protein_coding |
| ENSOANG00000047275 | ENSOANG00000047275 | O_anatinus | Animals | mono_intergenic | H/ACA | | | intergenic |
| ENSOANG00000047276 | ENSOANG00000047276 | O_anatinus | Animals | mono_intergenic | H/ACA | | | intergenic |
| ENSOANG00000047285 | ENSOANG00000047285 | O_anatinus | Animals | mono_intergenic | H/ACA | | | intergenic |
| ENSOANG00000047286 | ENSOANG00000047286 | O_anatinus | Animals | mono_intergenic | H/ACA | | | intergenic |
| ENSOANG00000047289 | ENSOANG00000047289 | O_anatinus | Animals | mono_intergenic | H/ACA | | | intergenic |
| ENSOANG00000047293 | ENSOANG00000047293 | O_anatinus | Animals | mono_intergenic | H/ACA | | | intergenic |
| ENSOANG00000047294 | ENSOANG00000047294 | O_anatinus | Animals | mono_intergenic | H/ACA | | | intergenic |
| ENSOANG00000047298 | ENSOANG00000047298 | O_anatinus | Animals | mono_intergenic | H/ACA | | | intergenic |
| ENSOANG00000047302 | ENSOANG00000047302 | O_anatinus | Animals | mono_intergenic | H/ACA | | | intergenic |
| ENSOANG00000047303 | ENSOANG00000047303 | O_anatinus | Animals | mono_intergenic | H/ACA | | | intergenic |
| ENSOANG00000047304 | ENSOANG00000047304 | O_anatinus | Animals | mono_intergenic | H/ACA | | | intergenic |
| ENSOANG00000047305 | ENSOANG00000047305 | O_anatinus | Animals | mono_intronic | H/ACA | ENSOANG00000005703 | PLXDC2 | protein_coding |
| ENSOANG00000047306 | ENSOANG00000047306 | O_anatinus | Animals | mono_intronic | H/ACA | ENSOANG00000005929 | ZBTB7C | protein_coding |
| ENSOANG00000047309 | ENSOANG00000047309 | O_anatinus | Animals | mono_intergenic | H/ACA | | | intergenic |
| ENSOANG00000047311 | ENSOANG00000047311 | O_anatinus | Animals | mono_intergenic | H/ACA | | | intergenic |
| ENSOANG00000047312 | ENSOANG00000047312 | O_anatinus | Animals | mono_intergenic | H/ACA | | | intergenic |
| ENSOANG00000047314 | ENSOANG00000047314 | O_anatinus | Animals | mono_intronic | H/ACA | ENSOANG00000046027 | SLC10A1 | protein_coding |
| ENSOANG00000047315 | ENSOANG00000047315 | O_anatinus | Animals | mono_intergenic | H/ACA | | | intergenic |
| ENSOANG00000047317 | ENSOANG00000047317 | O_anatinus | Animals | mono_intergenic | H/ACA | | | intergenic |
| ENSOANG00000047322 | ENSOANG00000047322 | O_anatinus | Animals | mono_intergenic | H/ACA | | | intergenic |
| ENSOANG00000047325 | ENSOANG00000047325 | O_anatinus | Animals | mono_intergenic | H/ACA | | | intergenic |
| ENSOANG00000047329 | ENSOANG00000047329 | O_anatinus | Animals | intronic_cluster | H/ACA | ENSOANG00000010461 | CRTAC1 | protein_coding |
| ENSOANG00000047332 | ENSOANG00000047332 | O_anatinus | Animals | mono_intergenic | H/ACA | | | intergenic |
| ENSOANG00000047334 | ENSOANG00000047334 | O_anatinus | Animals | mono_intergenic | H/ACA | | | intergenic |
| ENSOANG00000047342 | ENSOANG00000047342 | O_anatinus | Animals | mono_intergenic | H/ACA | | | intergenic |
| ENSOANG00000047344 | U3 | O_anatinus | Animals | intronic_cluster | C/D | ENSOANG00000008488 | | protein_coding |
| ENSOANG00000047345 | ENSOANG00000047345 | O_anatinus | Animals | intergenic_cluster | H/ACA | | | intergenic |
| ENSOANG00000047347 | ENSOANG00000047347 | O_anatinus | Animals | mono_intergenic | H/ACA | | | intergenic |
| ENSOANG00000047348 | ENSOANG00000047348 | O_anatinus | Animals | mono_intergenic | H/ACA | | | intergenic |
| ENSOANG00000047349 | ENSOANG00000047349 | O_anatinus | Animals | mono_intergenic | H/ACA | | | intergenic |
| ENSOANG00000047362 | ENSOANG00000047362 | O_anatinus | Animals | mono_intergenic | H/ACA | | | intergenic |
| ENSOANG00000047364 | ENSOANG00000047364 | O_anatinus | Animals | mono_intergenic | H/ACA | | | intergenic |
| ENSOANG00000047365 | ENSOANG00000047365 | O_anatinus | Animals | mono_intergenic | H/ACA | | | intergenic |
| ENSOANG00000047368 | ENSOANG00000047368 | O_anatinus | Animals | mono_intergenic | H/ACA | | | intergenic |
| ENSOANG00000047372 | ENSOANG00000047372 | O_anatinus | Animals | mono_intergenic | H/ACA | | | intergenic |
| ENSOANG00000047377 | ENSOANG00000047377 | O_anatinus | Animals | mono_intergenic | H/ACA | | | intergenic |
| ENSOANG00000047380 | ENSOANG00000047380 | O_anatinus | Animals | mono_intergenic | H/ACA | | | intergenic |

| | | | | | | | | |
|---|---|---|---|---|---|---|---|---|
| ENSOANG00000047382 | ENSOANG00000047382 | O_anatinus | Animals | mono_intergenic | H/ACA | | | intergenic |
| ENSOANG00000047390 | ENSOANG00000047390 | O_anatinus | Animals | mono_intergenic | H/ACA | | | intergenic |
| ENSOANG00000047392 | ENSOANG00000047392 | O_anatinus | Animals | mono_intergenic | H/ACA | | | intergenic |
| ENSOANG00000047395 | ENSOANG00000047395 | O_anatinus | Animals | mono_intergenic | H/ACA | | | intergenic |
| ENSOANG00000047397 | ENSOANG00000047397 | O_anatinus | Animals | mono_intronic | H/ACA | ENSOANG00000038732 | KCNAB1 | protein_coding |
| ENSOANG00000047401 | ENSOANG00000047401 | O_anatinus | Animals | mono_intergenic | H/ACA | | | intergenic |
| ENSOANG00000047402 | ENSOANG00000047402 | O_anatinus | Animals | mono_intergenic | H/ACA | | | intergenic |
| ENSOANG00000047406 | ENSOANG00000047406 | O_anatinus | Animals | mono_intronic | H/ACA | ENSOANG00000012684 | TNIK | protein_coding |
| ENSOANG00000047411 | ENSOANG00000047411 | O_anatinus | Animals | mono_intergenic | H/ACA | | | intergenic |
| ENSOANG00000047413 | ENSOANG00000047413 | O_anatinus | Animals | mono_intergenic | H/ACA | | | intergenic |
| ENSOANG00000047414 | ENSOANG00000047414 | O_anatinus | Animals | mono_intergenic | H/ACA | | | intergenic |
| ENSOANG00000047417 | ENSOANG00000047417 | O_anatinus | Animals | mono_intronic | H/ACA | ENSOANG00000046247 | TGFBR3 | protein_coding |
| ENSOANG00000047418 | ENSOANG00000047418 | O_anatinus | Animals | mono_intergenic | H/ACA | | | intergenic |
| ENSOANG00000047419 | ENSOANG00000047419 | O_anatinus | Animals | mono_intergenic | H/ACA | | | intergenic |
| ENSOANG00000047421 | ENSOANG00000047421 | O_anatinus | Animals | mono_intergenic | H/ACA | | | intergenic |
| ENSOANG00000047422 | ENSOANG00000047422 | O_anatinus | Animals | mono_intergenic | H/ACA | | | intergenic |
| ENSOANG00000047425 | ENSOANG00000047425 | O_anatinus | Animals | mono_intergenic | H/ACA | | | intergenic |
| ENSOANG00000047429 | ENSOANG00000047429 | O_anatinus | Animals | mono_intergenic | H/ACA | | | intergenic |
| ENSOANG00000047433 | ENSOANG00000047433 | O_anatinus | Animals | mono_intergenic | H/ACA | | | intergenic |
| ENSOANG00000047435 | ENSOANG00000047435 | O_anatinus | Animals | mono_intergenic | H/ACA | | | intergenic |
| ENSOANG00000047436 | ENSOANG00000047436 | O_anatinus | Animals | mono_intergenic | H/ACA | | | intergenic |
| ENSOANG00000047439 | ENSOANG00000047439 | O_anatinus | Animals | mono_intergenic | H/ACA | | | intergenic |
| ENSOANG00000047441 | ENSOANG00000047441 | O_anatinus | Animals | mono_intergenic | H/ACA | | | intergenic |
| ENSOANG00000047442 | ENSOANG00000047442 | O_anatinus | Animals | mono_intronic | H/ACA | ENSOANG00000021805 | NTRK3 | protein_coding |
| ENSOANG00000047445 | ENSOANG00000047445 | O_anatinus | Animals | mono_intergenic | H/ACA | | | intergenic |
| ENSOANG00000047449 | ENSOANG00000047449 | O_anatinus | Animals | mono_intergenic | H/ACA | | | intergenic |
| ENSOANG00000047450 | ENSOANG00000047450 | O_anatinus | Animals | mono_intergenic | H/ACA | | | intergenic |
| ENSOANG00000047460 | ENSOANG00000047460 | O_anatinus | Animals | mono_intronic | H/ACA | ENSOANG00000043974 | SSRP1 | protein_coding |
| ENSOANG00000047462 | ENSOANG00000047462 | O_anatinus | Animals | mono_intergenic | H/ACA | | | intergenic |
| ENSOANG00000047463 | ENSOANG00000047463 | O_anatinus | Animals | mono_intergenic | H/ACA | | | intergenic |
| ENSOANG00000047465 | ENSOANG00000047465 | O_anatinus | Animals | mono_intergenic | H/ACA | | | intergenic |
| ENSOANG00000047466 | ENSOANG00000047466 | O_anatinus | Animals | mono_intergenic | H/ACA | | | intergenic |
| ENSOANG00000047468 | ENSOANG00000047468 | O_anatinus | Animals | mono_intronic | H/ACA | ENSOANG00000047501 | | non_coding |
| ENSOANG00000047469 | ENSOANG00000047469 | O_anatinus | Animals | mono_intergenic | H/ACA | | | intergenic |
| ENSOANG00000047473 | SNORD83A | O_anatinus | Animals | mono_intronic | C/D | ENSOANG00000013665 | RPL3 | protein_coding |
| ENSOANG00000047474 | ENSOANG00000047474 | O_anatinus | Animals | mono_intergenic | H/ACA | | | intergenic |
| ENSOANG00000047478 | ENSOANG00000047478 | O_anatinus | Animals | mono_intergenic | H/ACA | | | intergenic |
| ENSOANG00000047480 | ENSOANG00000047480 | O_anatinus | Animals | mono_intergenic | H/ACA | | | intergenic |
| ENSOANG00000047483 | ENSOANG00000047483 | O_anatinus | Animals | mono_intergenic | H/ACA | | | intergenic |
| ENSOANG00000047484 | ENSOANG00000047484 | O_anatinus | Animals | mono_intergenic | H/ACA | | | intergenic |
| ENSOANG00000047485 | ENSOANG00000047485 | O_anatinus | Animals | intronic_cluster | H/ACA | ENSOANG00000008229 | | protein_coding |

| | | | | | | | | |
|---|---|---|---|---|---|---|---|---|
| ENSOANG00000047486 | ENSOANG00000047486 | O_anatinus | Animals | mono_intergenic | H/ACA | | | intergenic |
| ENSOANG00000047487 | ENSOANG00000047487 | O_anatinus | Animals | mono_intergenic | H/ACA | | | intergenic |
| ENSOANG00000047489 | ENSOANG00000047489 | O_anatinus | Animals | mono_intergenic | H/ACA | | | intergenic |
| ENSOANG00000047491 | ENSOANG00000047491 | O_anatinus | Animals | mono_intergenic | H/ACA | | | intergenic |
| ENSOANG00000047496 | ENSOANG00000047496 | O_anatinus | Animals | mono_intergenic | H/ACA | | | intergenic |
| ENSOANG00000047497 | ENSOANG00000047497 | O_anatinus | Animals | mono_intergenic | H/ACA | | | intergenic |
| ENSOANG00000047498 | ENSOANG00000047498 | O_anatinus | Animals | mono_intergenic | H/ACA | | | intergenic |
| ENSOANG00000047500 | ENSOANG00000047500 | O_anatinus | Animals | mono_intronic | H/ACA | ENSOANG00000007366 | CNTN1 | protein_coding |
| ENSOANG00000047502 | ENSOANG00000047502 | O_anatinus | Animals | mono_intergenic | H/ACA | | | intergenic |
| ENSOANG00000047503 | ENSOANG00000047503 | O_anatinus | Animals | mono_intronic | H/ACA | ENSOANG00000013454 | GPSM1 | protein_coding |
| ENSOANG00000047504 | ENSOANG00000047504 | O_anatinus | Animals | mono_intronic | H/ACA | ENSOANG00000008701 | SERAC1 | protein_coding |
| ENSOANG00000047505 | ENSOANG00000047505 | O_anatinus | Animals | mono_intergenic | H/ACA | | | intergenic |
| ENSOANG00000047506 | ENSOANG00000047506 | O_anatinus | Animals | mono_intergenic | H/ACA | | | intergenic |
| ENSOANG00000047507 | ENSOANG00000047507 | O_anatinus | Animals | mono_intergenic | H/ACA | | | intergenic |
| ENSOANG00000047508 | ENSOANG00000047508 | O_anatinus | Animals | mono_intergenic | H/ACA | | | intergenic |
| ENSOANG00000047512 | ENSOANG00000047512 | O_anatinus | Animals | intronic_cluster | H/ACA | ENSOANG00000044098 | | protein_coding |
| ENSOANG00000047519 | ENSOANG00000047519 | O_anatinus | Animals | intronic_cluster | H/ACA | ENSOANG00000008416 | PLCB1 | protein_coding |
| ENSOANG00000047520 | ENSOANG00000047520 | O_anatinus | Animals | mono_intergenic | H/ACA | | | intergenic |
| ENSOANG00000047523 | ENSOANG00000047523 | O_anatinus | Animals | mono_intergenic | H/ACA | | | intergenic |
| ENSOANG00000047525 | ENSOANG00000047525 | O_anatinus | Animals | mono_intergenic | H/ACA | | | intergenic |
| ENSOANG00000047526 | ENSOANG00000047526 | O_anatinus | Animals | mono_intergenic | H/ACA | | | intergenic |
| ENSOANG00000047527 | ENSOANG00000047527 | O_anatinus | Animals | mono_intergenic | H/ACA | | | intergenic |
| ENSOANG00000047530 | ENSOANG00000047530 | O_anatinus | Animals | mono_intergenic | H/ACA | | | intergenic |
| ENSOANG00000047533 | ENSOANG00000047533 | O_anatinus | Animals | mono_intergenic | H/ACA | | | intergenic |
| ENSOANG00000047534 | ENSOANG00000047534 | O_anatinus | Animals | mono_intergenic | H/ACA | | | intergenic |
| ENSOANG00000047537 | ENSOANG00000047537 | O_anatinus | Animals | mono_intergenic | H/ACA | | | intergenic |
| ENSOANG00000047539 | ENSOANG00000047539 | O_anatinus | Animals | intronic_cluster | H/ACA | ENSOANG00000046123 | | non_coding |
| ENSOANG00000047541 | ENSOANG00000047541 | O_anatinus | Animals | mono_intergenic | H/ACA | | | intergenic |
| ENSOANG00000047542 | ENSOANG00000047542 | O_anatinus | Animals | mono_intergenic | H/ACA | | | intergenic |
| ENSOANG00000047543 | ENSOANG00000047543 | O_anatinus | Animals | mono_intronic | H/ACA | ENSOANG00000029848 | | protein_coding |
| ENSOANG00000047547 | ENSOANG00000047547 | O_anatinus | Animals | mono_intergenic | H/ACA | | | intergenic |
| ENSOANG00000047549 | ENSOANG00000047549 | O_anatinus | Animals | mono_intergenic | H/ACA | | | intergenic |
| ENSOANG00000047550 | ENSOANG00000047550 | O_anatinus | Animals | mono_intergenic | H/ACA | | | intergenic |
| ENSOANG00000047554 | ENSOANG00000047554 | O_anatinus | Animals | mono_intergenic | H/ACA | | | intergenic |
| ENSOANG00000047555 | ENSOANG00000047555 | O_anatinus | Animals | mono_intergenic | H/ACA | | | intergenic |
| ENSOANG00000047563 | ENSOANG00000047563 | O_anatinus | Animals | mono_intergenic | H/ACA | | | intergenic |
| ENSOANG00000047569 | ENSOANG00000047569 | O_anatinus | Animals | mono_intergenic | H/ACA | | | intergenic |
| ENSOANG00000047573 | ENSOANG00000047573 | O_anatinus | Animals | mono_intergenic | H/ACA | | | intergenic |
| ENSOANG00000047576 | ENSOANG00000047576 | O_anatinus | Animals | mono_intergenic | H/ACA | | | intergenic |
| ENSOANG00000047578 | ENSOANG00000047578 | O_anatinus | Animals | mono_intergenic | H/ACA | | | intergenic |
| ENSOANG00000047579 | ENSOANG00000047579 | O_anatinus | Animals | mono_intergenic | H/ACA | | | intergenic |

| | | | | | | | | |
|---|---|---|---|---|---|---|---|---|
| ENSOANG00000047585 | ENSOANG00000047585 | O_anatinus | Animals | mono_intronic | H/ACA | ENSOANG00000050223 | KY | protein_coding |
| ENSOANG00000047587 | ENSOANG00000047587 | O_anatinus | Animals | mono_intronic | H/ACA | ENSOANG00000021517 | AK5 | protein_coding |
| ENSOANG00000047589 | ENSOANG00000047589 | O_anatinus | Animals | mono_intergenic | H/ACA | | | intergenic |
| ENSOANG00000047591 | ENSOANG00000047591 | O_anatinus | Animals | mono_intergenic | H/ACA | | | intergenic |
| ENSOANG00000047593 | ENSOANG00000047593 | O_anatinus | Animals | intronic_cluster | H/ACA | ENSOANG00000014431 | ADAMTS12 | protein_coding |
| ENSOANG00000047594 | ENSOANG00000047594 | O_anatinus | Animals | mono_intergenic | H/ACA | | | intergenic |
| ENSOANG00000047595 | ENSOANG00000047595 | O_anatinus | Animals | mono_intergenic | H/ACA | | | intergenic |
| ENSOANG00000047596 | ENSOANG00000047596 | O_anatinus | Animals | mono_intergenic | H/ACA | | | intergenic |
| ENSOANG00000047597 | ENSOANG00000047597 | O_anatinus | Animals | mono_intergenic | H/ACA | | | intergenic |
| ENSOANG00000047598 | ENSOANG00000047598 | O_anatinus | Animals | mono_intergenic | H/ACA | | | intergenic |
| ENSOANG00000047600 | ENSOANG00000047600 | O_anatinus | Animals | mono_intergenic | H/ACA | | | intergenic |
| ENSOANG00000047601 | ENSOANG00000047601 | O_anatinus | Animals | mono_intergenic | H/ACA | | | intergenic |
| ENSOANG00000047603 | ENSOANG00000047603 | O_anatinus | Animals | mono_intergenic | H/ACA | | | intergenic |
| ENSOANG00000047606 | ENSOANG00000047606 | O_anatinus | Animals | mono_intergenic | H/ACA | | | intergenic |
| ENSOANG00000047607 | ENSOANG00000047607 | O_anatinus | Animals | mono_intronic | H/ACA | ENSOANG00000015031 | MET | protein_coding |
| ENSOANG00000047609 | ENSOANG00000047609 | O_anatinus | Animals | mono_intergenic | H/ACA | | | intergenic |
| ENSOANG00000047610 | ENSOANG00000047610 | O_anatinus | Animals | mono_intergenic | H/ACA | | | intergenic |
| ENSOANG00000047611 | ENSOANG00000047611 | O_anatinus | Animals | mono_intergenic | H/ACA | | | intergenic |
| ENSOANG00000047612 | ENSOANG00000047612 | O_anatinus | Animals | mono_intergenic | H/ACA | | | intergenic |
| ENSOANG00000047616 | ENSOANG00000047616 | O_anatinus | Animals | mono_intergenic | H/ACA | | | intergenic |
| ENSOANG00000047624 | ENSOANG00000047624 | O_anatinus | Animals | mono_intergenic | H/ACA | | | intergenic |
| ENSOANG00000047625 | ENSOANG00000047625 | O_anatinus | Animals | mono_intergenic | H/ACA | | | intergenic |
| ENSOANG00000047630 | ENSOANG00000047630 | O_anatinus | Animals | mono_intergenic | H/ACA | | | intergenic |
| ENSOANG00000047631 | ENSOANG00000047631 | O_anatinus | Animals | mono_intergenic | H/ACA | | | intergenic |
| ENSOANG00000047634 | ENSOANG00000047634 | O_anatinus | Animals | mono_intergenic | H/ACA | | | intergenic |
| ENSOANG00000047635 | ENSOANG00000047635 | O_anatinus | Animals | mono_intergenic | H/ACA | | | intergenic |
| ENSOANG00000047636 | ENSOANG00000047636 | O_anatinus | Animals | mono_intergenic | H/ACA | | | intergenic |
| ENSOANG00000047637 | ENSOANG00000047637 | O_anatinus | Animals | mono_intergenic | H/ACA | | | intergenic |
| ENSOANG00000047644 | ENSOANG00000047644 | O_anatinus | Animals | mono_intergenic | H/ACA | | | intergenic |
| ENSOANG00000047646 | ENSOANG00000047646 | O_anatinus | Animals | mono_intergenic | H/ACA | | | intergenic |
| ENSOANG00000047647 | ENSOANG00000047647 | O_anatinus | Animals | mono_intronic | H/ACA | ENSOANG00000028726 | | protein_coding |
| ENSOANG00000047648 | ENSOANG00000047648 | O_anatinus | Animals | mono_intergenic | H/ACA | | | intergenic |
| ENSOANG00000047652 | ENSOANG00000047652 | O_anatinus | Animals | mono_intergenic | H/ACA | | | intergenic |
| ENSOANG00000047655 | ENSOANG00000047655 | O_anatinus | Animals | mono_intergenic | H/ACA | | | intergenic |
| ENSOANG00000047664 | ENSOANG00000047664 | O_anatinus | Animals | mono_intergenic | H/ACA | | | intergenic |
| ENSOANG00000047665 | ENSOANG00000047665 | O_anatinus | Animals | mono_intergenic | H/ACA | | | intergenic |
| ENSOANG00000047668 | ENSOANG00000047668 | O_anatinus | Animals | mono_intronic | H/ACA | ENSOANG00000014787 | GRID1 | protein_coding |
| ENSOANG00000047669 | ENSOANG00000047669 | O_anatinus | Animals | mono_intergenic | H/ACA | | | intergenic |
| ENSOANG00000047673 | ENSOANG00000047673 | O_anatinus | Animals | mono_intergenic | H/ACA | | | intergenic |
| ENSOANG00000047676 | ENSOANG00000047676 | O_anatinus | Animals | mono_intergenic | H/ACA | | | intergenic |
| ENSOANG00000047682 | SNORA66 | O_anatinus | Animals | mono_intronic | H/ACA | ENSOANG00000004669 | RPL5 | protein_coding |

| | | | | | | | | |
|---|---|---|---|---|---|---|---|---|
| ENSOANG00000047685 | ENSOANG00000047685 | O_anatinus | Animals | mono_intergenic | H/ACA | | | intergenic |
| ENSOANG00000047688 | ENSOANG00000047688 | O_anatinus | Animals | intronic_cluster | H/ACA | ENSOANG00000001761 | HS2ST1 | protein_coding |
| ENSOANG00000047690 | ENSOANG00000047690 | O_anatinus | Animals | mono_intergenic | H/ACA | | | intergenic |
| ENSOANG00000047691 | ENSOANG00000047691 | O_anatinus | Animals | mono_intergenic | H/ACA | | | intergenic |
| ENSOANG00000047694 | ENSOANG00000047694 | O_anatinus | Animals | mono_intergenic | H/ACA | | | intergenic |
| ENSOANG00000047696 | ENSOANG00000047696 | O_anatinus | Animals | mono_intergenic | H/ACA | | | intergenic |
| ENSOANG00000047698 | ENSOANG00000047698 | O_anatinus | Animals | mono_intergenic | H/ACA | | | intergenic |
| ENSOANG00000047704 | ENSOANG00000047704 | O_anatinus | Animals | mono_intergenic | H/ACA | | | intergenic |
| ENSOANG00000047705 | ENSOANG00000047705 | O_anatinus | Animals | intronic_cluster | H/ACA | ENSOANG00000044930 | PARK7 | protein_coding |
| ENSOANG00000047706 | ENSOANG00000047706 | O_anatinus | Animals | mono_intergenic | H/ACA | | | intergenic |
| ENSOANG00000047707 | ENSOANG00000047707 | O_anatinus | Animals | mono_intergenic | H/ACA | | | intergenic |
| ENSOANG00000047708 | ENSOANG00000047708 | O_anatinus | Animals | mono_intergenic | H/ACA | | | intergenic |
| ENSOANG00000047709 | ENSOANG00000047709 | O_anatinus | Animals | mono_intergenic | H/ACA | | | intergenic |
| ENSOANG00000047714 | ENSOANG00000047714 | O_anatinus | Animals | mono_intergenic | H/ACA | | | intergenic |
| ENSOANG00000047715 | ENSOANG00000047715 | O_anatinus | Animals | mono_intergenic | H/ACA | | | intergenic |
| ENSOANG00000047719 | ENSOANG00000047719 | O_anatinus | Animals | mono_intergenic | H/ACA | | | intergenic |
| ENSOANG00000047724 | ENSOANG00000047724 | O_anatinus | Animals | mono_intergenic | H/ACA | | | intergenic |
| ENSOANG00000047727 | ENSOANG00000047727 | O_anatinus | Animals | mono_intergenic | H/ACA | | | intergenic |
| ENSOANG00000047732 | ENSOANG00000047732 | O_anatinus | Animals | mono_intergenic | H/ACA | | | intergenic |
| ENSOANG00000047736 | ENSOANG00000047736 | O_anatinus | Animals | mono_intergenic | H/ACA | | | intergenic |
| ENSOANG00000047739 | ENSOANG00000047739 | O_anatinus | Animals | mono_intronic | H/ACA | ENSOANG00000010870 | SYNDIG1 | protein_coding |
| ENSOANG00000047740 | ENSOANG00000047740 | O_anatinus | Animals | mono_intergenic | H/ACA | | | intergenic |
| ENSOANG00000047741 | ENSOANG00000047741 | O_anatinus | Animals | mono_intronic | H/ACA | ENSOANG00000007026 | PRKG1 | protein_coding |
| ENSOANG00000047742 | ENSOANG00000047742 | O_anatinus | Animals | mono_intergenic | H/ACA | | | intergenic |
| ENSOANG00000047746 | ENSOANG00000047746 | O_anatinus | Animals | mono_intergenic | H/ACA | | | intergenic |
| ENSOANG00000047754 | ENSOANG00000047754 | O_anatinus | Animals | mono_intergenic | H/ACA | | | intergenic |
| ENSOANG00000047755 | ENSOANG00000047755 | O_anatinus | Animals | mono_intergenic | H/ACA | | | intergenic |
| ENSOANG00000047760 | ENSOANG00000047760 | O_anatinus | Animals | mono_intronic | H/ACA | ENSOANG00000005462 | STK24 | protein_coding |
| ENSOANG00000047765 | ENSOANG00000047765 | O_anatinus | Animals | mono_intergenic | H/ACA | | | intergenic |
| ENSOANG00000047766 | ENSOANG00000047766 | O_anatinus | Animals | mono_intergenic | H/ACA | | | intergenic |
| ENSOANG00000047768 | ENSOANG00000047768 | O_anatinus | Animals | mono_intronic | H/ACA | ENSOANG00000002156 | PHKB | protein_coding |
| ENSOANG00000047771 | ENSOANG00000047771 | O_anatinus | Animals | mono_intergenic | H/ACA | | | intergenic |
| ENSOANG00000047772 | ENSOANG00000047772 | O_anatinus | Animals | mono_intergenic | H/ACA | | | intergenic |
| ENSOANG00000047776 | ENSOANG00000047776 | O_anatinus | Animals | mono_intergenic | H/ACA | | | intergenic |
| ENSOANG00000047777 | ENSOANG00000047777 | O_anatinus | Animals | mono_intergenic | H/ACA | | | intergenic |
| ENSOANG00000047780 | ENSOANG00000047780 | O_anatinus | Animals | mono_intergenic | H/ACA | | | intergenic |
| ENSOANG00000047782 | ENSOANG00000047782 | O_anatinus | Animals | mono_intronic | H/ACA | ENSOANG00000015749 | WDFY4 | protein_coding |
| ENSOANG00000047783 | ENSOANG00000047783 | O_anatinus | Animals | mono_intergenic | H/ACA | | | intergenic |
| ENSOANG00000047784 | ENSOANG00000047784 | O_anatinus | Animals | mono_intronic | H/ACA | ENSOANG00000007450 | E2F7 | protein_coding |
| ENSOANG00000047785 | ENSOANG00000047785 | O_anatinus | Animals | mono_intergenic | H/ACA | | | intergenic |
| ENSOANG00000047786 | ENSOANG00000047786 | O_anatinus | Animals | mono_intergenic | H/ACA | | | intergenic |

| | | | | | | | | |
|---|---|---|---|---|---|---|---|---|
| ENSOANG00000047787 | ENSOANG00000047787 | O_anatinus | Animals | mono_intergenic | H/ACA | | | intergenic |
| ENSOANG00000047789 | ENSOANG00000047789 | O_anatinus | Animals | mono_intergenic | H/ACA | | | intergenic |
| ENSOANG00000047794 | ENSOANG00000047794 | O_anatinus | Animals | mono_intronic | H/ACA | ENSOANG00000022364 | | protein_coding |
| ENSOANG00000047796 | ENSOANG00000047796 | O_anatinus | Animals | mono_intergenic | H/ACA | | | intergenic |
| ENSOANG00000047801 | ENSOANG00000047801 | O_anatinus | Animals | mono_intergenic | H/ACA | | | intergenic |
| ENSOANG00000047806 | ENSOANG00000047806 | O_anatinus | Animals | intronic_cluster | H/ACA | ENSOANG00000039168 | SLC6A11 | protein_coding |
| ENSOANG00000047807 | ENSOANG00000047807 | O_anatinus | Animals | mono_intergenic | H/ACA | | | intergenic |
| ENSOANG00000047809 | ENSOANG00000047809 | O_anatinus | Animals | mono_intergenic | H/ACA | | | intergenic |
| ENSOANG00000047811 | ENSOANG00000047811 | O_anatinus | Animals | mono_intronic | H/ACA | ENSOANG00000008127 | MYO5B | protein_coding |
| ENSOANG00000047814 | ENSOANG00000047814 | O_anatinus | Animals | mono_intergenic | H/ACA | | | intergenic |
| ENSOANG00000047820 | ENSOANG00000047820 | O_anatinus | Animals | mono_intergenic | H/ACA | | | intergenic |
| ENSOANG00000047824 | ENSOANG00000047824 | O_anatinus | Animals | mono_intronic | H/ACA | ENSOANG00000010656 | CNKSR2 | protein_coding |
| ENSOANG00000047827 | ENSOANG00000047827 | O_anatinus | Animals | mono_intergenic | H/ACA | | | intergenic |
| ENSOANG00000047829 | ENSOANG00000047829 | O_anatinus | Animals | mono_intergenic | H/ACA | | | intergenic |
| ENSOANG00000047831 | ENSOANG00000047831 | O_anatinus | Animals | mono_intergenic | H/ACA | | | intergenic |
| ENSOANG00000047832 | ENSOANG00000047832 | O_anatinus | Animals | mono_intergenic | H/ACA | | | intergenic |
| ENSOANG00000047833 | ENSOANG00000047833 | O_anatinus | Animals | intronic_cluster | H/ACA | ENSOANG00000001034 | NTNG1 | protein_coding |
| ENSOANG00000047834 | ENSOANG00000047834 | O_anatinus | Animals | mono_intronic | H/ACA | ENSOANG00000010001 | PABPC4 | protein_coding |
| ENSOANG00000047839 | ENSOANG00000047839 | O_anatinus | Animals | mono_intergenic | H/ACA | | | intergenic |
| ENSOANG00000047840 | ENSOANG00000047840 | O_anatinus | Animals | mono_intergenic | H/ACA | | | intergenic |
| ENSOANG00000047845 | ENSOANG00000047845 | O_anatinus | Animals | mono_intergenic | H/ACA | | | intergenic |
| ENSOANG00000047850 | ENSOANG00000047850 | O_anatinus | Animals | mono_intergenic | H/ACA | | | intergenic |
| ENSOANG00000047851 | ENSOANG00000047851 | O_anatinus | Animals | mono_intronic | H/ACA | ENSOANG00000006974 | | protein_coding |
| ENSOANG00000047852 | ENSOANG00000047852 | O_anatinus | Animals | mono_intergenic | H/ACA | | | intergenic |
| ENSOANG00000047855 | ENSOANG00000047855 | O_anatinus | Animals | mono_intergenic | H/ACA | | | intergenic |
| ENSOANG00000047858 | ENSOANG00000047858 | O_anatinus | Animals | mono_intergenic | H/ACA | | | intergenic |
| ENSOANG00000047860 | ENSOANG00000047860 | O_anatinus | Animals | mono_intergenic | H/ACA | | | intergenic |
| ENSOANG00000047866 | ENSOANG00000047866 | O_anatinus | Animals | mono_intergenic | H/ACA | | | intergenic |
| ENSOANG00000047867 | ENSOANG00000047867 | O_anatinus | Animals | mono_intergenic | H/ACA | | | intergenic |
| ENSOANG00000047874 | ENSOANG00000047874 | O_anatinus | Animals | mono_intergenic | H/ACA | | | intergenic |
| ENSOANG00000047875 | ENSOANG00000047875 | O_anatinus | Animals | mono_intergenic | H/ACA | | | intergenic |
| ENSOANG00000047877 | ENSOANG00000047877 | O_anatinus | Animals | mono_intergenic | H/ACA | | | intergenic |
| ENSOANG00000047878 | ENSOANG00000047878 | O_anatinus | Animals | mono_intergenic | H/ACA | | | intergenic |
| ENSOANG00000047879 | ENSOANG00000047879 | O_anatinus | Animals | mono_intergenic | H/ACA | | | intergenic |
| ENSOANG00000047881 | ENSOANG00000047881 | O_anatinus | Animals | mono_intergenic | H/ACA | | | intergenic |
| ENSOANG00000047885 | ENSOANG00000047885 | O_anatinus | Animals | mono_intergenic | H/ACA | | | intergenic |
| ENSOANG00000047889 | ENSOANG00000047889 | O_anatinus | Animals | mono_intronic | H/ACA | ENSOANG00000014878 | TMEM132B | protein_coding |
| ENSOANG00000047890 | ENSOANG00000047890 | O_anatinus | Animals | mono_intronic | H/ACA | ENSOANG00000046889 | | protein_coding |
| ENSOANG00000047893 | ENSOANG00000047893 | O_anatinus | Animals | mono_intronic | H/ACA | ENSOANG00000003718 | LRRC31 | protein_coding |
| ENSOANG00000047894 | ENSOANG00000047894 | O_anatinus | Animals | mono_intronic | C/D | ENSOANG00000050134 | | non_coding |
| ENSOANG00000047898 | ENSOANG00000047898 | O_anatinus | Animals | mono_intronic | H/ACA | ENSOANG00000050997 | | protein_coding |

| | | | | | | | | |
|---|---|---|---|---|---|---|---|---|
| ENSOANG00000047899 | ENSOANG00000047899 | O_anatinus | Animals | mono_intronic | H/ACA | ENSOANG00000046360 | | protein_coding |
| ENSOANG00000047900 | ENSOANG00000047900 | O_anatinus | Animals | mono_intergenic | H/ACA | | | intergenic |
| ENSOANG00000047902 | ENSOANG00000047902 | O_anatinus | Animals | mono_intergenic | H/ACA | | | intergenic |
| ENSOANG00000047903 | ENSOANG00000047903 | O_anatinus | Animals | mono_intergenic | H/ACA | | | intergenic |
| ENSOANG00000047904 | ENSOANG00000047904 | O_anatinus | Animals | mono_intergenic | H/ACA | | | intergenic |
| ENSOANG00000047905 | ENSOANG00000047905 | O_anatinus | Animals | mono_intergenic | H/ACA | | | intergenic |
| ENSOANG00000047906 | ENSOANG00000047906 | O_anatinus | Animals | mono_intergenic | H/ACA | | | intergenic |
| ENSOANG00000047907 | ENSOANG00000047907 | O_anatinus | Animals | mono_intergenic | H/ACA | | | intergenic |
| ENSOANG00000047908 | ENSOANG00000047908 | O_anatinus | Animals | mono_intergenic | H/ACA | | | intergenic |
| ENSOANG00000047912 | ENSOANG00000047912 | O_anatinus | Animals | mono_intergenic | H/ACA | | | intergenic |
| ENSOANG00000047913 | ENSOANG00000047913 | O_anatinus | Animals | mono_intergenic | H/ACA | | | intergenic |
| ENSOANG00000047918 | ENSOANG00000047918 | O_anatinus | Animals | mono_intronic | H/ACA | ENSOANG00000004866 | LDB2 | protein_coding |
| ENSOANG00000047919 | ENSOANG00000047919 | O_anatinus | Animals | mono_intergenic | H/ACA | | | intergenic |
| ENSOANG00000047922 | ENSOANG00000047922 | O_anatinus | Animals | mono_intergenic | H/ACA | | | intergenic |
| ENSOANG00000047923 | ENSOANG00000047923 | O_anatinus | Animals | mono_intergenic | H/ACA | | | intergenic |
| ENSOANG00000047924 | ENSOANG00000047924 | O_anatinus | Animals | mono_intergenic | H/ACA | | | intergenic |
| ENSOANG00000047926 | ENSOANG00000047926 | O_anatinus | Animals | mono_intergenic | H/ACA | | | intergenic |
| ENSOANG00000047927 | ENSOANG00000047927 | O_anatinus | Animals | mono_intronic | H/ACA | ENSOANG00000004024 | SLC16A4 | protein_coding |
| ENSOANG00000047930 | ENSOANG00000047930 | O_anatinus | Animals | mono_intergenic | H/ACA | | | intergenic |
| ENSOANG00000047931 | ENSOANG00000047931 | O_anatinus | Animals | mono_intronic | H/ACA | ENSOANG00000050538 | HPCAL1 | protein_coding |
| ENSOANG00000047932 | ENSOANG00000047932 | O_anatinus | Animals | mono_intergenic | H/ACA | | | intergenic |
| ENSOANG00000047935 | ENSOANG00000047935 | O_anatinus | Animals | mono_intergenic | H/ACA | | | intergenic |
| ENSOANG00000047937 | ENSOANG00000047937 | O_anatinus | Animals | mono_intergenic | H/ACA | | | intergenic |
| ENSOANG00000047938 | ENSOANG00000047938 | O_anatinus | Animals | mono_intergenic | H/ACA | | | intergenic |
| ENSOANG00000047941 | ENSOANG00000047941 | O_anatinus | Animals | mono_intergenic | H/ACA | | | intergenic |
| ENSOANG00000047942 | ENSOANG00000047942 | O_anatinus | Animals | mono_intergenic | H/ACA | | | intergenic |
| ENSOANG00000047944 | ENSOANG00000047944 | O_anatinus | Animals | mono_intergenic | H/ACA | | | intergenic |
| ENSOANG00000047945 | ENSOANG00000047945 | O_anatinus | Animals | mono_intergenic | H/ACA | | | intergenic |
| ENSOANG00000047947 | ENSOANG00000047947 | O_anatinus | Animals | mono_intergenic | H/ACA | | | intergenic |
| ENSOANG00000047951 | ENSOANG00000047951 | O_anatinus | Animals | mono_intergenic | H/ACA | | | intergenic |
| ENSOANG00000047952 | ENSOANG00000047952 | O_anatinus | Animals | mono_intergenic | H/ACA | | | intergenic |
| ENSOANG00000047954 | ENSOANG00000047954 | O_anatinus | Animals | mono_intergenic | H/ACA | | | intergenic |
| ENSOANG00000047956 | ENSOANG00000047956 | O_anatinus | Animals | mono_intergenic | H/ACA | | | intergenic |
| ENSOANG00000047970 | ENSOANG00000047970 | O_anatinus | Animals | mono_intergenic | H/ACA | | | intergenic |
| ENSOANG00000047975 | ENSOANG00000047975 | O_anatinus | Animals | mono_intergenic | H/ACA | | | intergenic |
| ENSOANG00000047976 | ENSOANG00000047976 | O_anatinus | Animals | mono_intergenic | H/ACA | | | intergenic |
| ENSOANG00000047978 | ENSOANG00000047978 | O_anatinus | Animals | mono_intergenic | H/ACA | | | intergenic |
| ENSOANG00000047986 | ENSOANG00000047986 | O_anatinus | Animals | mono_intergenic | H/ACA | | | intergenic |
| ENSOANG00000047987 | ENSOANG00000047987 | O_anatinus | Animals | mono_intergenic | H/ACA | | | intergenic |
| ENSOANG00000047990 | ENSOANG00000047990 | O_anatinus | Animals | mono_intergenic | H/ACA | | | intergenic |
| ENSOANG00000047994 | ENSOANG00000047994 | O_anatinus | Animals | mono_intergenic | H/ACA | | | intergenic |

| | | | | | | | | |
|---|---|---|---|---|---|---|---|---|
| ENSOANG00000048000 | ENSOANG00000048000 | O_anatinus | Animals | mono_intergenic | H/ACA | | | intergenic |
| ENSOANG00000048008 | ENSOANG00000048008 | O_anatinus | Animals | mono_intergenic | H/ACA | | | intergenic |
| ENSOANG00000048009 | ENSOANG00000048009 | O_anatinus | Animals | mono_intergenic | H/ACA | | | intergenic |
| ENSOANG00000048015 | ENSOANG00000048015 | O_anatinus | Animals | mono_intergenic | H/ACA | | | intergenic |
| ENSOANG00000048017 | ENSOANG00000048017 | O_anatinus | Animals | mono_intergenic | H/ACA | | | intergenic |
| ENSOANG00000048023 | ENSOANG00000048023 | O_anatinus | Animals | mono_intergenic | H/ACA | | | intergenic |
| ENSOANG00000048024 | ENSOANG00000048024 | O_anatinus | Animals | mono_intergenic | H/ACA | | | intergenic |
| ENSOANG00000048025 | ENSOANG00000048025 | O_anatinus | Animals | mono_intergenic | H/ACA | | | intergenic |
| ENSOANG00000048026 | ENSOANG00000048026 | O_anatinus | Animals | mono_intergenic | H/ACA | | | intergenic |
| ENSOANG00000048027 | ENSOANG00000048027 | O_anatinus | Animals | mono_intergenic | H/ACA | | | intergenic |
| ENSOANG00000048031 | ENSOANG00000048031 | O_anatinus | Animals | mono_intergenic | H/ACA | | | intergenic |
| ENSOANG00000048032 | ENSOANG00000048032 | O_anatinus | Animals | mono_intronic | H/ACA | ENSOANG00000004629 | VWA8 | protein_coding |
| ENSOANG00000048037 | ENSOANG00000048037 | O_anatinus | Animals | mono_intergenic | H/ACA | | | intergenic |
| ENSOANG00000048038 | ENSOANG00000048038 | O_anatinus | Animals | mono_intergenic | H/ACA | | | intergenic |
| ENSOANG00000048045 | ENSOANG00000048045 | O_anatinus | Animals | mono_intergenic | H/ACA | | | intergenic |
| ENSOANG00000048046 | ENSOANG00000048046 | O_anatinus | Animals | mono_intergenic | H/ACA | | | intergenic |
| ENSOANG00000048053 | ENSOANG00000048053 | O_anatinus | Animals | mono_intergenic | H/ACA | | | intergenic |
| ENSOANG00000048054 | ENSOANG00000048054 | O_anatinus | Animals | mono_intergenic | H/ACA | | | intergenic |
| ENSOANG00000048055 | ENSOANG00000048055 | O_anatinus | Animals | mono_intergenic | H/ACA | | | intergenic |
| ENSOANG00000048057 | ENSOANG00000048057 | O_anatinus | Animals | mono_intergenic | H/ACA | | | intergenic |
| ENSOANG00000048061 | ENSOANG00000048061 | O_anatinus | Animals | mono_intergenic | H/ACA | | | intergenic |
| ENSOANG00000048064 | ENSOANG00000048064 | O_anatinus | Animals | mono_intergenic | H/ACA | | | intergenic |
| ENSOANG00000048066 | ENSOANG00000048066 | O_anatinus | Animals | mono_intergenic | H/ACA | | | intergenic |
| ENSOANG00000048068 | ENSOANG00000048068 | O_anatinus | Animals | mono_intergenic | H/ACA | | | intergenic |
| ENSOANG00000048070 | ENSOANG00000048070 | O_anatinus | Animals | mono_intergenic | H/ACA | | | intergenic |
| ENSOANG00000048072 | ENSOANG00000048072 | O_anatinus | Animals | mono_intergenic | H/ACA | | | intergenic |
| ENSOANG00000048074 | ENSOANG00000048074 | O_anatinus | Animals | mono_intronic | H/ACA | ENSOANG00000004432 | ITGA8 | protein_coding |
| ENSOANG00000048079 | ENSOANG00000048079 | O_anatinus | Animals | mono_intergenic | H/ACA | | | intergenic |
| ENSOANG00000048080 | ENSOANG00000048080 | O_anatinus | Animals | mono_intronic | H/ACA | ENSOANG00000013540 | TBC1D9 | protein_coding |
| ENSOANG00000048081 | ENSOANG00000048081 | O_anatinus | Animals | mono_intronic | H/ACA | ENSOANG00000039953 | | protein_coding |
| ENSOANG00000048087 | ENSOANG00000048087 | O_anatinus | Animals | mono_intergenic | H/ACA | | | intergenic |
| ENSOANG00000048089 | ENSOANG00000048089 | O_anatinus | Animals | mono_intergenic | H/ACA | | | intergenic |
| ENSOANG00000048091 | ENSOANG00000048091 | O_anatinus | Animals | mono_intronic | H/ACA | ENSOANG00000004754 | | protein_coding |
| ENSOANG00000048093 | ENSOANG00000048093 | O_anatinus | Animals | mono_intergenic | H/ACA | | | intergenic |
| ENSOANG00000048094 | ENSOANG00000048094 | O_anatinus | Animals | mono_intergenic | H/ACA | | | intergenic |
| ENSOANG00000048095 | ENSOANG00000048095 | O_anatinus | Animals | mono_intronic | H/ACA | ENSOANG00000050709 | FBXL17 | protein_coding |
| ENSOANG00000048100 | ENSOANG00000048100 | O_anatinus | Animals | mono_intergenic | H/ACA | | | intergenic |
| ENSOANG00000048105 | ENSOANG00000048105 | O_anatinus | Animals | mono_intergenic | H/ACA | | | intergenic |
| ENSOANG00000048108 | ENSOANG00000048108 | O_anatinus | Animals | mono_intergenic | H/ACA | | | intergenic |
| ENSOANG00000048110 | ENSOANG00000048110 | O_anatinus | Animals | mono_intergenic | H/ACA | | | intergenic |
| ENSOANG00000048114 | ENSOANG00000048114 | O_anatinus | Animals | mono_intergenic | H/ACA | | | intergenic |

| | | | | | | | | |
|---|---|---|---|---|---|---|---|---|
| ENSOANG00000048115 | ENSOANG00000048115 | O_anatinus | Animals | mono_intergenic | H/ACA | | | intergenic |
| ENSOANG00000048120 | ENSOANG00000048120 | O_anatinus | Animals | mono_intergenic | H/ACA | | | intergenic |
| ENSOANG00000048123 | ENSOANG00000048123 | O_anatinus | Animals | mono_intronic | H/ACA | ENSOANG00000004946 | DLGAP4 | protein_coding |
| ENSOANG00000048124 | ENSOANG00000048124 | O_anatinus | Animals | mono_intergenic | H/ACA | | | intergenic |
| ENSOANG00000048126 | ENSOANG00000048126 | O_anatinus | Animals | mono_intergenic | H/ACA | | | intergenic |
| ENSOANG00000048128 | ENSOANG00000048128 | O_anatinus | Animals | mono_intergenic | H/ACA | | | intergenic |
| ENSOANG00000048131 | ENSOANG00000048131 | O_anatinus | Animals | mono_intronic | H/ACA | ENSOANG00000004294 | PDE10A | protein_coding |
| ENSOANG00000048134 | ENSOANG00000048134 | O_anatinus | Animals | mono_intergenic | H/ACA | | | intergenic |
| ENSOANG00000048136 | ENSOANG00000048136 | O_anatinus | Animals | mono_intergenic | H/ACA | | | intergenic |
| ENSOANG00000048137 | ENSOANG00000048137 | O_anatinus | Animals | mono_intergenic | H/ACA | | | intergenic |
| ENSOANG00000048144 | ENSOANG00000048144 | O_anatinus | Animals | mono_intergenic | H/ACA | | | intergenic |
| ENSOANG00000048145 | ENSOANG00000048145 | O_anatinus | Animals | mono_intergenic | H/ACA | | | intergenic |
| ENSOANG00000048146 | ENSOANG00000048146 | O_anatinus | Animals | mono_intronic | H/ACA | ENSOANG00000013156 | KIF6 | protein_coding |
| ENSOANG00000048147 | ENSOANG00000048147 | O_anatinus | Animals | mono_intergenic | H/ACA | | | intergenic |
| ENSOANG00000048150 | ENSOANG00000048150 | O_anatinus | Animals | mono_intergenic | H/ACA | | | intergenic |
| ENSOANG00000048156 | ENSOANG00000048156 | O_anatinus | Animals | mono_intronic | H/ACA | ENSOANG00000001738 | TMEM117 | protein_coding |
| ENSOANG00000048158 | ENSOANG00000048158 | O_anatinus | Animals | mono_intergenic | H/ACA | | | intergenic |
| ENSOANG00000048160 | ENSOANG00000048160 | O_anatinus | Animals | mono_intronic | H/ACA | ENSOANG00000050093 | | protein_coding |
| ENSOANG00000048166 | ENSOANG00000048166 | O_anatinus | Animals | mono_intergenic | H/ACA | | | intergenic |
| ENSOANG00000048170 | ENSOANG00000048170 | O_anatinus | Animals | mono_intronic | H/ACA | ENSOANG00000015699 | SLC24A3 | protein_coding |
| ENSOANG00000048171 | ENSOANG00000048171 | O_anatinus | Animals | mono_intergenic | H/ACA | | | intergenic |
| ENSOANG00000048172 | ENSOANG00000048172 | O_anatinus | Animals | mono_intergenic | H/ACA | | | intergenic |
| ENSOANG00000048173 | ENSOANG00000048173 | O_anatinus | Animals | mono_intergenic | H/ACA | | | intergenic |
| ENSOANG00000048174 | ENSOANG00000048174 | O_anatinus | Animals | intergenic_cluster | H/ACA | | | intergenic |
| ENSOANG00000048175 | ENSOANG00000048175 | O_anatinus | Animals | mono_intergenic | H/ACA | | | intergenic |
| ENSOANG00000048176 | ENSOANG00000048176 | O_anatinus | Animals | mono_intergenic | H/ACA | | | intergenic |
| ENSOANG00000048177 | ENSOANG00000048177 | O_anatinus | Animals | mono_intergenic | H/ACA | | | intergenic |
| ENSOANG00000048178 | ENSOANG00000048178 | O_anatinus | Animals | mono_intergenic | H/ACA | | | intergenic |
| ENSOANG00000048179 | ENSOANG00000048179 | O_anatinus | Animals | mono_intergenic | H/ACA | | | intergenic |
| ENSOANG00000048180 | ENSOANG00000048180 | O_anatinus | Animals | mono_intergenic | H/ACA | | | intergenic |
| ENSOANG00000048181 | ENSOANG00000048181 | O_anatinus | Animals | mono_intergenic | H/ACA | | | intergenic |
| ENSOANG00000048184 | ENSOANG00000048184 | O_anatinus | Animals | mono_intergenic | H/ACA | | | intergenic |
| ENSOANG00000048185 | ENSOANG00000048185 | O_anatinus | Animals | mono_intergenic | H/ACA | | | intergenic |
| ENSOANG00000048186 | ENSOANG00000048186 | O_anatinus | Animals | mono_intergenic | H/ACA | | | intergenic |
| ENSOANG00000048188 | ENSOANG00000048188 | O_anatinus | Animals | mono_intergenic | H/ACA | | | intergenic |
| ENSOANG00000048189 | ENSOANG00000048189 | O_anatinus | Animals | mono_intergenic | H/ACA | | | intergenic |
| ENSOANG00000048191 | ENSOANG00000048191 | O_anatinus | Animals | mono_intergenic | H/ACA | | | intergenic |
| ENSOANG00000048192 | ENSOANG00000048192 | O_anatinus | Animals | mono_intergenic | H/ACA | | | intergenic |
| ENSOANG00000048194 | ENSOANG00000048194 | O_anatinus | Animals | mono_intergenic | H/ACA | | | intergenic |
| ENSOANG00000048196 | ENSOANG00000048196 | O_anatinus | Animals | mono_intergenic | H/ACA | | | intergenic |
| ENSOANG00000048198 | ENSOANG00000048198 | O_anatinus | Animals | mono_intergenic | H/ACA | | | intergenic |

| | | | | | | | | | |
|---|---|---|---|---|---|---|---|---|---|
| ENSOANG00000048200 | ENSOANG00000048200 | O_anatinus | Animals | mono_intergenic | H/ACA | | | | intergenic |
| ENSOANG00000048201 | ENSOANG00000048201 | O_anatinus | Animals | mono_intergenic | H/ACA | | | | intergenic |
| ENSOANG00000048203 | ENSOANG00000048203 | O_anatinus | Animals | mono_intergenic | H/ACA | | | | intergenic |
| ENSOANG00000048205 | ENSOANG00000048205 | O_anatinus | Animals | mono_intergenic | H/ACA | | | | intergenic |
| ENSOANG00000048206 | ENSOANG00000048206 | O_anatinus | Animals | mono_intergenic | H/ACA | | | | intergenic |
| ENSOANG00000048210 | ENSOANG00000048210 | O_anatinus | Animals | mono_intergenic | H/ACA | | | | intergenic |
| ENSOANG00000048216 | ENSOANG00000048216 | O_anatinus | Animals | mono_intergenic | H/ACA | | | | intergenic |
| ENSOANG00000048219 | ENSOANG00000048219 | O_anatinus | Animals | mono_intergenic | H/ACA | | | | intergenic |
| ENSOANG00000048220 | ENSOANG00000048220 | O_anatinus | Animals | mono_intergenic | H/ACA | | | | intergenic |
| ENSOANG00000048226 | ENSOANG00000048226 | O_anatinus | Animals | mono_intergenic | H/ACA | | | | intergenic |
| ENSOANG00000048227 | ENSOANG00000048227 | O_anatinus | Animals | mono_intergenic | H/ACA | | | | intergenic |
| ENSOANG00000048228 | ENSOANG00000048228 | O_anatinus | Animals | mono_intergenic | H/ACA | | | | intergenic |
| ENSOANG00000048230 | ENSOANG00000048230 | O_anatinus | Animals | mono_intergenic | H/ACA | | | | intergenic |
| ENSOANG00000048232 | ENSOANG00000048232 | O_anatinus | Animals | mono_intergenic | H/ACA | | | | intergenic |
| ENSOANG00000048234 | ENSOANG00000048234 | O_anatinus | Animals | mono_intronic | H/ACA | ENSOANG00000008229 | | | protein_coding |
| ENSOANG00000048240 | ENSOANG00000048240 | O_anatinus | Animals | mono_intergenic | H/ACA | | | | intergenic |
| ENSOANG00000048243 | ENSOANG00000048243 | O_anatinus | Animals | mono_intronic | H/ACA | ENSOANG00000042941 | | NTM | protein_coding |
| ENSOANG00000048244 | ENSOANG00000048244 | O_anatinus | Animals | mono_intergenic | H/ACA | | | | intergenic |
| ENSOANG00000048247 | ENSOANG00000048247 | O_anatinus | Animals | mono_intergenic | H/ACA | | | | intergenic |
| ENSOANG00000048251 | ENSOANG00000048251 | O_anatinus | Animals | mono_intergenic | H/ACA | | | | intergenic |
| ENSOANG00000048253 | ENSOANG00000048253 | O_anatinus | Animals | mono_intergenic | H/ACA | | | | intergenic |
| ENSOANG00000048256 | ENSOANG00000048256 | O_anatinus | Animals | mono_intergenic | H/ACA | | | | intergenic |
| ENSOANG00000048257 | ENSOANG00000048257 | O_anatinus | Animals | mono_intergenic | H/ACA | | | | intergenic |
| ENSOANG00000048259 | ENSOANG00000048259 | O_anatinus | Animals | mono_intergenic | H/ACA | | | | intergenic |
| ENSOANG00000048261 | ENSOANG00000048261 | O_anatinus | Animals | mono_intergenic | H/ACA | | | | intergenic |
| ENSOANG00000048264 | ENSOANG00000048264 | O_anatinus | Animals | mono_intronic | H/ACA | ENSOANG00000050479 | | | protein_coding |
| ENSOANG00000048265 | ENSOANG00000048265 | O_anatinus | Animals | mono_intergenic | H/ACA | | | | intergenic |
| ENSOANG00000048270 | ENSOANG00000048270 | O_anatinus | Animals | mono_intergenic | H/ACA | | | | intergenic |
| ENSOANG00000048272 | ENSOANG00000048272 | O_anatinus | Animals | mono_intergenic | H/ACA | | | | intergenic |
| ENSOANG00000048282 | ENSOANG00000048282 | O_anatinus | Animals | mono_intronic | H/ACA | ENSOANG00000038256 | | IRAG1 | protein_coding |
| ENSOANG00000048283 | ENSOANG00000048283 | O_anatinus | Animals | mono_intergenic | H/ACA | | | | intergenic |
| ENSOANG00000048285 | ENSOANG00000048285 | O_anatinus | Animals | mono_intergenic | H/ACA | | | | intergenic |
| ENSOANG00000048287 | ENSOANG00000048287 | O_anatinus | Animals | mono_intergenic | H/ACA | | | | intergenic |
| ENSOANG00000048289 | ENSOANG00000048289 | O_anatinus | Animals | mono_intergenic | H/ACA | | | | intergenic |
| ENSOANG00000048290 | ENSOANG00000048290 | O_anatinus | Animals | mono_intergenic | H/ACA | | | | intergenic |
| ENSOANG00000048294 | ENSOANG00000048294 | O_anatinus | Animals | mono_intergenic | H/ACA | | | | intergenic |
| ENSOANG00000048297 | ENSOANG00000048297 | O_anatinus | Animals | mono_intergenic | H/ACA | | | | intergenic |
| ENSOANG00000048299 | ENSOANG00000048299 | O_anatinus | Animals | mono_intergenic | H/ACA | | | | intergenic |
| ENSOANG00000048301 | ENSOANG00000048301 | O_anatinus | Animals | mono_intronic | H/ACA | ENSOANG00000041176 | | | protein_coding |
| ENSOANG00000048304 | ENSOANG00000048304 | O_anatinus | Animals | mono_intergenic | H/ACA | | | | intergenic |
| ENSOANG00000048305 | ENSOANG00000048305 | O_anatinus | Animals | mono_intronic | H/ACA | ENSOANG00000037357 | | LRRC1 | protein_coding |

| | | | | | | | | |
|---|---|---|---|---|---|---|---|---|
| ENSOANG00000048306 | ENSOANG00000048306 | O_anatinus | Animals | mono_intergenic | H/ACA | | | intergenic |
| ENSOANG00000048308 | ENSOANG00000048308 | O_anatinus | Animals | mono_intergenic | H/ACA | | | intergenic |
| ENSOANG00000048309 | ENSOANG00000048309 | O_anatinus | Animals | mono_intergenic | H/ACA | | | intergenic |
| ENSOANG00000048312 | ENSOANG00000048312 | O_anatinus | Animals | mono_intronic | H/ACA | ENSOANG00000004292 | PTBP3 | protein_coding |
| ENSOANG00000048319 | ENSOANG00000048319 | O_anatinus | Animals | mono_intronic | H/ACA | ENSOANG00000006832 | TJP1 | protein_coding |
| ENSOANG00000048320 | ENSOANG00000048320 | O_anatinus | Animals | mono_intergenic | H/ACA | | | intergenic |
| ENSOANG00000048322 | ENSOANG00000048322 | O_anatinus | Animals | intronic_cluster | H/ACA | ENSOANG00000047521 | HCN4 | protein_coding |
| ENSOANG00000048324 | ENSOANG00000048324 | O_anatinus | Animals | mono_intergenic | H/ACA | | | intergenic |
| ENSOANG00000048327 | ENSOANG00000048327 | O_anatinus | Animals | mono_intronic | H/ACA | ENSOANG00000014727 | | protein_coding |
| ENSOANG00000048329 | ENSOANG00000048329 | O_anatinus | Animals | mono_intronic | H/ACA | ENSOANG00000045064 | | protein_coding |
| ENSOANG00000048330 | ENSOANG00000048330 | O_anatinus | Animals | mono_intergenic | H/ACA | | | intergenic |
| ENSOANG00000048331 | ENSOANG00000048331 | O_anatinus | Animals | mono_intergenic | H/ACA | | | intergenic |
| ENSOANG00000048333 | ENSOANG00000048333 | O_anatinus | Animals | mono_intronic | H/ACA | ENSOANG00000048890 | | non_coding |
| ENSOANG00000048334 | ENSOANG00000048334 | O_anatinus | Animals | mono_intergenic | H/ACA | | | intergenic |
| ENSOANG00000048337 | ENSOANG00000048337 | O_anatinus | Animals | mono_intergenic | H/ACA | | | intergenic |
| ENSOANG00000048338 | ENSOANG00000048338 | O_anatinus | Animals | mono_intergenic | H/ACA | | | intergenic |
| ENSOANG00000048339 | ENSOANG00000048339 | O_anatinus | Animals | mono_intergenic | H/ACA | | | intergenic |
| ENSOANG00000048341 | ENSOANG00000048341 | O_anatinus | Animals | mono_intergenic | H/ACA | | | intergenic |
| ENSOANG00000048342 | ENSOANG00000048342 | O_anatinus | Animals | mono_intergenic | H/ACA | | | intergenic |
| ENSOANG00000048343 | ENSOANG00000048343 | O_anatinus | Animals | mono_intergenic | H/ACA | | | intergenic |
| ENSOANG00000048345 | ENSOANG00000048345 | O_anatinus | Animals | mono_intergenic | H/ACA | | | intergenic |
| ENSOANG00000048351 | ENSOANG00000048351 | O_anatinus | Animals | mono_intergenic | H/ACA | | | intergenic |
| ENSOANG00000048352 | ENSOANG00000048352 | O_anatinus | Animals | mono_intergenic | H/ACA | | | intergenic |
| ENSOANG00000048354 | ENSOANG00000048354 | O_anatinus | Animals | mono_intergenic | H/ACA | | | intergenic |
| ENSOANG00000048357 | ENSOANG00000048357 | O_anatinus | Animals | mono_intergenic | H/ACA | | | intergenic |
| ENSOANG00000048363 | ENSOANG00000048363 | O_anatinus | Animals | mono_intergenic | H/ACA | | | intergenic |
| ENSOANG00000048364 | ENSOANG00000048364 | O_anatinus | Animals | mono_intergenic | H/ACA | | | intergenic |
| ENSOANG00000048365 | ENSOANG00000048365 | O_anatinus | Animals | mono_intergenic | H/ACA | | | intergenic |
| ENSOANG00000048368 | ENSOANG00000048368 | O_anatinus | Animals | mono_intergenic | H/ACA | | | intergenic |
| ENSOANG00000048370 | ENSOANG00000048370 | O_anatinus | Animals | mono_intergenic | H/ACA | | | intergenic |
| ENSOANG00000048375 | ENSOANG00000048375 | O_anatinus | Animals | mono_intergenic | H/ACA | | | intergenic |
| ENSOANG00000048379 | ENSOANG00000048379 | O_anatinus | Animals | mono_intergenic | H/ACA | | | intergenic |
| ENSOANG00000048385 | ENSOANG00000048385 | O_anatinus | Animals | mono_intergenic | H/ACA | | | intergenic |
| ENSOANG00000048386 | ENSOANG00000048386 | O_anatinus | Animals | mono_intergenic | H/ACA | | | intergenic |
| ENSOANG00000048388 | ENSOANG00000048388 | O_anatinus | Animals | mono_intergenic | H/ACA | | | intergenic |
| ENSOANG00000048389 | ENSOANG00000048389 | O_anatinus | Animals | mono_intronic | C/D | ENSOANG00000003235 | ATP5F1B | protein_coding |
| ENSOANG00000048392 | ENSOANG00000048392 | O_anatinus | Animals | mono_intergenic | H/ACA | | | intergenic |
| ENSOANG00000048393 | ENSOANG00000048393 | O_anatinus | Animals | intronic_cluster | H/ACA | ENSOANG00000001903 | PCDH15 | protein_coding |
| ENSOANG00000048395 | ENSOANG00000048395 | O_anatinus | Animals | mono_intergenic | H/ACA | | | intergenic |
| ENSOANG00000048397 | ENSOANG00000048397 | O_anatinus | Animals | mono_intergenic | H/ACA | | | intergenic |
| ENSOANG00000048398 | ENSOANG00000048398 | O_anatinus | Animals | mono_intergenic | H/ACA | | | intergenic |

| | | | | | | | | |
|---|---|---|---|---|---|---|---|---|
| ENSOANG00000048400 | ENSOANG00000048400 | O_anatinus | Animals | mono_intergenic | H/ACA | | | intergenic |
| ENSOANG00000048402 | ENSOANG00000048402 | O_anatinus | Animals | mono_intergenic | H/ACA | | | intergenic |
| ENSOANG00000048403 | ENSOANG00000048403 | O_anatinus | Animals | mono_intergenic | H/ACA | | | intergenic |
| ENSOANG00000048408 | ENSOANG00000048408 | O_anatinus | Animals | mono_intergenic | H/ACA | | | intergenic |
| ENSOANG00000048412 | ENSOANG00000048412 | O_anatinus | Animals | mono_intergenic | H/ACA | | | intergenic |
| ENSOANG00000048413 | ENSOANG00000048413 | O_anatinus | Animals | mono_intergenic | H/ACA | | | intergenic |
| ENSOANG00000048418 | ENSOANG00000048418 | O_anatinus | Animals | mono_intergenic | H/ACA | | | intergenic |
| ENSOANG00000048421 | ENSOANG00000048421 | O_anatinus | Animals | mono_intergenic | H/ACA | | | intergenic |
| ENSOANG00000048424 | ENSOANG00000048424 | O_anatinus | Animals | mono_intergenic | H/ACA | | | intergenic |
| ENSOANG00000048427 | ENSOANG00000048427 | O_anatinus | Animals | mono_intergenic | H/ACA | | | intergenic |
| ENSOANG00000048428 | ENSOANG00000048428 | O_anatinus | Animals | mono_intergenic | H/ACA | | | intergenic |
| ENSOANG00000048431 | ENSOANG00000048431 | O_anatinus | Animals | mono_intergenic | H/ACA | | | intergenic |
| ENSOANG00000048432 | ENSOANG00000048432 | O_anatinus | Animals | mono_intergenic | H/ACA | | | intergenic |
| ENSOANG00000048443 | ENSOANG00000048443 | O_anatinus | Animals | mono_intergenic | H/ACA | | | intergenic |
| ENSOANG00000048445 | ENSOANG00000048445 | O_anatinus | Animals | mono_intergenic | H/ACA | | | intergenic |
| ENSOANG00000048446 | ENSOANG00000048446 | O_anatinus | Animals | mono_intergenic | H/ACA | | | intergenic |
| ENSOANG00000048448 | ENSOANG00000048448 | O_anatinus | Animals | mono_intergenic | H/ACA | | | intergenic |
| ENSOANG00000048450 | ENSOANG00000048450 | O_anatinus | Animals | mono_intergenic | H/ACA | | | intergenic |
| ENSOANG00000048454 | ENSOANG00000048454 | O_anatinus | Animals | mono_intergenic | H/ACA | | | intergenic |
| ENSOANG00000048458 | ENSOANG00000048458 | O_anatinus | Animals | mono_intergenic | C/D | | | intergenic |
| ENSOANG00000048462 | ENSOANG00000048462 | O_anatinus | Animals | mono_intergenic | H/ACA | | | intergenic |
| ENSOANG00000048464 | ENSOANG00000048464 | O_anatinus | Animals | mono_intergenic | H/ACA | | | intergenic |
| ENSOANG00000048466 | ENSOANG00000048466 | O_anatinus | Animals | mono_intergenic | H/ACA | | | intergenic |
| ENSOANG00000048467 | ENSOANG00000048467 | O_anatinus | Animals | mono_intergenic | H/ACA | | | intergenic |
| ENSOANG00000048469 | ENSOANG00000048469 | O_anatinus | Animals | mono_intergenic | H/ACA | | | intergenic |
| ENSOANG00000048470 | ENSOANG00000048470 | O_anatinus | Animals | mono_intergenic | H/ACA | | | intergenic |
| ENSOANG00000048472 | ENSOANG00000048472 | O_anatinus | Animals | mono_intronic | H/ACA | ENSOANG00000039006 | | protein_coding |
| ENSOANG00000048474 | ENSOANG00000048474 | O_anatinus | Animals | mono_intergenic | H/ACA | | | intergenic |
| ENSOANG00000048475 | ENSOANG00000048475 | O_anatinus | Animals | mono_intergenic | H/ACA | | | intergenic |
| ENSOANG00000048479 | ENSOANG00000048479 | O_anatinus | Animals | mono_intronic | H/ACA | ENSOANG00000009071 | FLT1 | protein_coding |
| ENSOANG00000048482 | ENSOANG00000048482 | O_anatinus | Animals | mono_intergenic | H/ACA | | | intergenic |
| ENSOANG00000048488 | ENSOANG00000048488 | O_anatinus | Animals | mono_intergenic | H/ACA | | | intergenic |
| ENSOANG00000048490 | ENSOANG00000048490 | O_anatinus | Animals | mono_intergenic | H/ACA | | | intergenic |
| ENSOANG00000048497 | ENSOANG00000048497 | O_anatinus | Animals | mono_intergenic | H/ACA | | | intergenic |
| ENSOANG00000048498 | ENSOANG00000048498 | O_anatinus | Animals | mono_intergenic | H/ACA | | | intergenic |
| ENSOANG00000048502 | ENSOANG00000048502 | O_anatinus | Animals | mono_intergenic | H/ACA | | | intergenic |
| ENSOANG00000048503 | ENSOANG00000048503 | O_anatinus | Animals | mono_intergenic | H/ACA | | | intergenic |
| ENSOANG00000048513 | ENSOANG00000048513 | O_anatinus | Animals | mono_intergenic | H/ACA | | | intergenic |
| ENSOANG00000048517 | ENSOANG00000048517 | O_anatinus | Animals | mono_intergenic | H/ACA | | | intergenic |
| ENSOANG00000048519 | ENSOANG00000048519 | O_anatinus | Animals | mono_intergenic | H/ACA | | | intergenic |
| ENSOANG00000048522 | ENSOANG00000048522 | O_anatinus | Animals | mono_intergenic | H/ACA | | | intergenic |

| | | | | | | | | |
|---|---|---|---|---|---|---|---|---|
| ENSOANG00000048523 | ENSOANG00000048523 | O_anatinus | Animals | mono_intergenic | H/ACA | | | intergenic |
| ENSOANG00000048526 | ENSOANG00000048526 | O_anatinus | Animals | mono_intergenic | H/ACA | | | intergenic |
| ENSOANG00000048531 | ENSOANG00000048531 | O_anatinus | Animals | mono_intronic | H/ACA | ENSOANG00000001603 | RASGRF2 | protein_coding |
| ENSOANG00000048534 | ENSOANG00000048534 | O_anatinus | Animals | mono_intergenic | H/ACA | | | intergenic |
| ENSOANG00000048535 | ENSOANG00000048535 | O_anatinus | Animals | mono_intergenic | H/ACA | | | intergenic |
| ENSOANG00000048537 | SNORA57 | O_anatinus | Animals | mono_intergenic | H/ACA | | | intergenic |
| ENSOANG00000048538 | ENSOANG00000048538 | O_anatinus | Animals | mono_intergenic | H/ACA | | | intergenic |
| ENSOANG00000048540 | ENSOANG00000048540 | O_anatinus | Animals | mono_intergenic | H/ACA | | | intergenic |
| ENSOANG00000048546 | ENSOANG00000048546 | O_anatinus | Animals | mono_intergenic | H/ACA | | | intergenic |
| ENSOANG00000048549 | ENSOANG00000048549 | O_anatinus | Animals | mono_intergenic | H/ACA | | | intergenic |
| ENSOANG00000048552 | ENSOANG00000048552 | O_anatinus | Animals | mono_intergenic | H/ACA | | | intergenic |
| ENSOANG00000048554 | ENSOANG00000048554 | O_anatinus | Animals | mono_intergenic | H/ACA | | | intergenic |
| ENSOANG00000048555 | ENSOANG00000048555 | O_anatinus | Animals | mono_intergenic | H/ACA | | | intergenic |
| ENSOANG00000048556 | ENSOANG00000048556 | O_anatinus | Animals | mono_intergenic | H/ACA | | | intergenic |
| ENSOANG00000048558 | ENSOANG00000048558 | O_anatinus | Animals | mono_intergenic | H/ACA | | | intergenic |
| ENSOANG00000048559 | ENSOANG00000048559 | O_anatinus | Animals | mono_intergenic | H/ACA | | | intergenic |
| ENSOANG00000048560 | ENSOANG00000048560 | O_anatinus | Animals | mono_intergenic | H/ACA | | | intergenic |
| ENSOANG00000048562 | ENSOANG00000048562 | O_anatinus | Animals | mono_intergenic | H/ACA | | | intergenic |
| ENSOANG00000048567 | ENSOANG00000048567 | O_anatinus | Animals | mono_intergenic | H/ACA | | | intergenic |
| ENSOANG00000048568 | ENSOANG00000048568 | O_anatinus | Animals | mono_intergenic | H/ACA | | | intergenic |
| ENSOANG00000048569 | ENSOANG00000048569 | O_anatinus | Animals | mono_intergenic | H/ACA | | | intergenic |
| ENSOANG00000048571 | ENSOANG00000048571 | O_anatinus | Animals | mono_intergenic | H/ACA | | | intergenic |
| ENSOANG00000048578 | ENSOANG00000048578 | O_anatinus | Animals | mono_intronic | H/ACA | ENSOANG00000000325 | RAB36 | protein_coding |
| ENSOANG00000048580 | ENSOANG00000048580 | O_anatinus | Animals | mono_intergenic | H/ACA | | | intergenic |
| ENSOANG00000048581 | ENSOANG00000048581 | O_anatinus | Animals | mono_intergenic | H/ACA | | | intergenic |
| ENSOANG00000048583 | ENSOANG00000048583 | O_anatinus | Animals | mono_intergenic | H/ACA | | | intergenic |
| ENSOANG00000048585 | ENSOANG00000048585 | O_anatinus | Animals | mono_intronic | H/ACA | ENSOANG00000038272 | | non_coding |
| ENSOANG00000048586 | ENSOANG00000048586 | O_anatinus | Animals | mono_intergenic | H/ACA | | | intergenic |
| ENSOANG00000048594 | ENSOANG00000048594 | O_anatinus | Animals | mono_intergenic | H/ACA | | | intergenic |
| ENSOANG00000048596 | ENSOANG00000048596 | O_anatinus | Animals | mono_intergenic | H/ACA | | | intergenic |
| ENSOANG00000048598 | ENSOANG00000048598 | O_anatinus | Animals | mono_intergenic | H/ACA | | | intergenic |
| ENSOANG00000048599 | ENSOANG00000048599 | O_anatinus | Animals | mono_intergenic | H/ACA | | | intergenic |
| ENSOANG00000048607 | ENSOANG00000048607 | O_anatinus | Animals | mono_intergenic | H/ACA | | | intergenic |
| ENSOANG00000048608 | SNORD39 | O_anatinus | Animals | mono_intronic | C/D | ENSOANG00000041837 | RPS8 | protein_coding |
| ENSOANG00000048609 | ENSOANG00000048609 | O_anatinus | Animals | mono_intronic | H/ACA | ENSOANG00000005919 | MSRB3 | protein_coding |
| ENSOANG00000048611 | ENSOANG00000048611 | O_anatinus | Animals | mono_intergenic | H/ACA | | | intergenic |
| ENSOANG00000048616 | ENSOANG00000048616 | O_anatinus | Animals | mono_intronic | H/ACA | ENSOANG00000003444 | KCNQ5 | protein_coding |
| ENSOANG00000048618 | ENSOANG00000048618 | O_anatinus | Animals | mono_intergenic | H/ACA | | | intergenic |
| ENSOANG00000048622 | ENSOANG00000048622 | O_anatinus | Animals | mono_intergenic | H/ACA | | | intergenic |
| ENSOANG00000048624 | ENSOANG00000048624 | O_anatinus | Animals | mono_intergenic | H/ACA | | | intergenic |
| ENSOANG00000048627 | ENSOANG00000048627 | O_anatinus | Animals | mono_intronic | H/ACA | ENSOANG00000006938 | RCC2 | protein_coding |

| | | | | | | | | |
|---|---|---|---|---|---|---|---|---|
| ENSOANG00000048629 | ENSOANG00000048629 | O_anatinus | Animals | mono_intergenic | H/ACA | | | intergenic |
| ENSOANG00000048635 | ENSOANG00000048635 | O_anatinus | Animals | mono_intergenic | H/ACA | | | intergenic |
| ENSOANG00000048637 | ENSOANG00000048637 | O_anatinus | Animals | mono_intronic | H/ACA | ENSOANG00000006717 | PIK3AP1 | protein_coding |
| ENSOANG00000048642 | ENSOANG00000048642 | O_anatinus | Animals | mono_intergenic | H/ACA | | | intergenic |
| ENSOANG00000048643 | ENSOANG00000048643 | O_anatinus | Animals | mono_intronic | H/ACA | ENSOANG00000041706 | | non_coding |
| ENSOANG00000048645 | ENSOANG00000048645 | O_anatinus | Animals | mono_intergenic | H/ACA | | | intergenic |
| ENSOANG00000048646 | ENSOANG00000048646 | O_anatinus | Animals | mono_intergenic | H/ACA | | | intergenic |
| ENSOANG00000048647 | ENSOANG00000048647 | O_anatinus | Animals | mono_intergenic | H/ACA | | | intergenic |
| ENSOANG00000048649 | ENSOANG00000048649 | O_anatinus | Animals | mono_intergenic | H/ACA | | | intergenic |
| ENSOANG00000048654 | ENSOANG00000048654 | O_anatinus | Animals | mono_intergenic | H/ACA | | | intergenic |
| ENSOANG00000048657 | ENSOANG00000048657 | O_anatinus | Animals | mono_intergenic | H/ACA | | | intergenic |
| ENSOANG00000048658 | ENSOANG00000048658 | O_anatinus | Animals | mono_intergenic | H/ACA | | | intergenic |
| ENSOANG00000048659 | ENSOANG00000048659 | O_anatinus | Animals | mono_intronic | H/ACA | ENSOANG00000007157 | GRIN2A | protein_coding |
| ENSOANG00000048661 | ENSOANG00000048661 | O_anatinus | Animals | mono_intergenic | H/ACA | | | intergenic |
| ENSOANG00000048665 | ENSOANG00000048665 | O_anatinus | Animals | mono_intergenic | H/ACA | | | intergenic |
| ENSOANG00000048667 | ENSOANG00000048667 | O_anatinus | Animals | mono_intronic | H/ACA | ENSOANG00000014549 | TENM1 | protein_coding |
| ENSOANG00000048668 | ENSOANG00000048668 | O_anatinus | Animals | mono_intergenic | H/ACA | | | intergenic |
| ENSOANG00000048674 | ENSOANG00000048674 | O_anatinus | Animals | mono_intergenic | H/ACA | | | intergenic |
| ENSOANG00000048680 | ENSOANG00000048680 | O_anatinus | Animals | mono_intergenic | H/ACA | | | intergenic |
| ENSOANG00000048687 | ENSOANG00000048687 | O_anatinus | Animals | mono_intergenic | H/ACA | | | intergenic |
| ENSOANG00000048688 | ENSOANG00000048688 | O_anatinus | Animals | intronic_cluster | H/ACA | ENSOANG00000029831 | AGMO | protein_coding |
| ENSOANG00000048690 | ENSOANG00000048690 | O_anatinus | Animals | mono_intergenic | H/ACA | | | intergenic |
| ENSOANG00000048694 | ENSOANG00000048694 | O_anatinus | Animals | mono_intergenic | H/ACA | | | intergenic |
| ENSOANG00000048695 | ENSOANG00000048695 | O_anatinus | Animals | mono_intergenic | H/ACA | | | intergenic |
| ENSOANG00000048697 | ENSOANG00000048697 | O_anatinus | Animals | mono_intergenic | H/ACA | | | intergenic |
| ENSOANG00000048699 | ENSOANG00000048699 | O_anatinus | Animals | mono_intergenic | H/ACA | | | intergenic |
| ENSOANG00000048701 | ENSOANG00000048701 | O_anatinus | Animals | mono_intergenic | H/ACA | | | intergenic |
| ENSOANG00000048712 | ENSOANG00000048712 | O_anatinus | Animals | mono_intronic | H/ACA | ENSOANG00000003166 | PRKAR2B | protein_coding |
| ENSOANG00000048720 | ENSOANG00000048720 | O_anatinus | Animals | mono_intronic | H/ACA | ENSOANG00000043726 | CPN1 | protein_coding |
| ENSOANG00000048721 | ENSOANG00000048721 | O_anatinus | Animals | mono_intergenic | H/ACA | | | intergenic |
| ENSOANG00000048723 | ENSOANG00000048723 | O_anatinus | Animals | mono_intergenic | H/ACA | | | intergenic |
| ENSOANG00000048727 | ENSOANG00000048727 | O_anatinus | Animals | mono_intergenic | H/ACA | | | intergenic |
| ENSOANG00000048728 | ENSOANG00000048728 | O_anatinus | Animals | mono_intergenic | H/ACA | | | intergenic |
| ENSOANG00000048729 | ENSOANG00000048729 | O_anatinus | Animals | mono_intergenic | H/ACA | | | intergenic |
| ENSOANG00000048732 | ENSOANG00000048732 | O_anatinus | Animals | mono_intergenic | H/ACA | | | intergenic |
| ENSOANG00000048734 | ENSOANG00000048734 | O_anatinus | Animals | mono_intergenic | H/ACA | | | intergenic |
| ENSOANG00000048736 | ENSOANG00000048736 | O_anatinus | Animals | mono_intergenic | H/ACA | | | intergenic |
| ENSOANG00000048738 | ENSOANG00000048738 | O_anatinus | Animals | mono_intergenic | H/ACA | | | intergenic |
| ENSOANG00000048739 | ENSOANG00000048739 | O_anatinus | Animals | mono_intronic | H/ACA | ENSOANG00000049291 | KCNT2 | protein_coding |
| ENSOANG00000048740 | ENSOANG00000048740 | O_anatinus | Animals | mono_intergenic | H/ACA | | | intergenic |
| ENSOANG00000048741 | ENSOANG00000048741 | O_anatinus | Animals | mono_intergenic | H/ACA | | | intergenic |

| | | | | | | | | |
|---|---|---|---|---|---|---|---|---|
| ENSOANG00000048742 | ENSOANG00000048742 | O_anatinus | Animals | mono_intergenic | H/ACA | | | intergenic |
| ENSOANG00000048747 | ENSOANG00000048747 | O_anatinus | Animals | mono_intergenic | H/ACA | | | intergenic |
| ENSOANG00000048748 | ENSOANG00000048748 | O_anatinus | Animals | mono_intergenic | H/ACA | | | intergenic |
| ENSOANG00000048751 | ENSOANG00000048751 | O_anatinus | Animals | mono_intergenic | H/ACA | | | intergenic |
| ENSOANG00000048754 | ENSOANG00000048754 | O_anatinus | Animals | mono_intergenic | H/ACA | | | intergenic |
| ENSOANG00000048755 | ENSOANG00000048755 | O_anatinus | Animals | mono_intergenic | H/ACA | | | intergenic |
| ENSOANG00000048756 | ENSOANG00000048756 | O_anatinus | Animals | mono_intergenic | H/ACA | | | intergenic |
| ENSOANG00000048764 | ENSOANG00000048764 | O_anatinus | Animals | mono_intergenic | H/ACA | | | intergenic |
| ENSOANG00000048772 | ENSOANG00000048772 | O_anatinus | Animals | mono_intergenic | H/ACA | | | intergenic |
| ENSOANG00000048773 | ENSOANG00000048773 | O_anatinus | Animals | mono_intergenic | H/ACA | | | intergenic |
| ENSOANG00000048777 | ENSOANG00000048777 | O_anatinus | Animals | mono_intergenic | H/ACA | | | intergenic |
| ENSOANG00000048783 | ENSOANG00000048783 | O_anatinus | Animals | mono_intergenic | H/ACA | | | intergenic |
| ENSOANG00000048784 | ENSOANG00000048784 | O_anatinus | Animals | mono_intergenic | H/ACA | | | intergenic |
| ENSOANG00000048787 | ENSOANG00000048787 | O_anatinus | Animals | mono_intronic | H/ACA | ENSOANG00000001480 | | protein_coding |
| ENSOANG00000048788 | ENSOANG00000048788 | O_anatinus | Animals | mono_intergenic | H/ACA | | | intergenic |
| ENSOANG00000048790 | ENSOANG00000048790 | O_anatinus | Animals | mono_intronic | H/ACA | ENSOANG00000008811 | MYO1H | protein_coding |
| ENSOANG00000048794 | ENSOANG00000048794 | O_anatinus | Animals | mono_intergenic | H/ACA | | | intergenic |
| ENSOANG00000048795 | ENSOANG00000048795 | O_anatinus | Animals | mono_intergenic | H/ACA | | | intergenic |
| ENSOANG00000048796 | ENSOANG00000048796 | O_anatinus | Animals | mono_intergenic | H/ACA | | | intergenic |
| ENSOANG00000048799 | ENSOANG00000048799 | O_anatinus | Animals | mono_intergenic | H/ACA | | | intergenic |
| ENSOANG00000048800 | ENSOANG00000048800 | O_anatinus | Animals | mono_intergenic | H/ACA | | | intergenic |
| ENSOANG00000048803 | ENSOANG00000048803 | O_anatinus | Animals | mono_intergenic | H/ACA | | | intergenic |
| ENSOANG00000048804 | ENSOANG00000048804 | O_anatinus | Animals | mono_intergenic | H/ACA | | | intergenic |
| ENSOANG00000048812 | ENSOANG00000048812 | O_anatinus | Animals | mono_intronic | H/ACA | ENSOANG00000041309 | | protein_coding |
| ENSOANG00000048815 | ENSOANG00000048815 | O_anatinus | Animals | mono_intergenic | H/ACA | | | intergenic |
| ENSOANG00000048821 | ENSOANG00000048821 | O_anatinus | Animals | mono_intergenic | H/ACA | | | intergenic |
| ENSOANG00000048823 | ENSOANG00000048823 | O_anatinus | Animals | mono_intergenic | H/ACA | | | intergenic |
| ENSOANG00000048826 | ENSOANG00000048826 | O_anatinus | Animals | mono_intergenic | H/ACA | | | intergenic |
| ENSOANG00000048829 | ENSOANG00000048829 | O_anatinus | Animals | mono_intergenic | H/ACA | | | intergenic |
| ENSOANG00000048831 | ENSOANG00000048831 | O_anatinus | Animals | mono_intergenic | H/ACA | | | intergenic |
| ENSOANG00000048833 | ENSOANG00000048833 | O_anatinus | Animals | mono_intergenic | H/ACA | | | intergenic |
| ENSOANG00000048835 | ENSOANG00000048835 | O_anatinus | Animals | mono_intronic | H/ACA | ENSOANG00000012869 | | protein_coding |
| ENSOANG00000048838 | ENSOANG00000048838 | O_anatinus | Animals | mono_intergenic | H/ACA | | | intergenic |
| ENSOANG00000048840 | ENSOANG00000048840 | O_anatinus | Animals | mono_intergenic | H/ACA | | | intergenic |
| ENSOANG00000048841 | ENSOANG00000048841 | O_anatinus | Animals | mono_intergenic | H/ACA | | | intergenic |
| ENSOANG00000048850 | ENSOANG00000048850 | O_anatinus | Animals | mono_intergenic | H/ACA | | | intergenic |
| ENSOANG00000048853 | ENSOANG00000048853 | O_anatinus | Animals | mono_intronic | H/ACA | ENSOANG00000015577 | | protein_coding |
| ENSOANG00000048855 | ENSOANG00000048855 | O_anatinus | Animals | mono_intergenic | H/ACA | | | intergenic |
| ENSOANG00000048856 | ENSOANG00000048856 | O_anatinus | Animals | intronic_cluster | H/ACA | ENSOANG00000015522 | LRRC7 | protein_coding |
| ENSOANG00000048858 | ENSOANG00000048858 | O_anatinus | Animals | mono_intergenic | H/ACA | | | intergenic |
| ENSOANG00000048859 | ENSOANG00000048859 | O_anatinus | Animals | mono_intergenic | H/ACA | | | intergenic |

| | | | | | | | | |
|---|---|---|---|---|---|---|---|---|
| ENSOANG00000048860 | ENSOANG00000048860 | O_anatinus | Animals | mono_intergenic | H/ACA | | | intergenic |
| ENSOANG00000048862 | ENSOANG00000048862 | O_anatinus | Animals | mono_intergenic | H/ACA | | | intergenic |
| ENSOANG00000048863 | ENSOANG00000048863 | O_anatinus | Animals | mono_intergenic | H/ACA | | | intergenic |
| ENSOANG00000048864 | ENSOANG00000048864 | O_anatinus | Animals | mono_intergenic | H/ACA | | | intergenic |
| ENSOANG00000048869 | ENSOANG00000048869 | O_anatinus | Animals | mono_intergenic | H/ACA | | | intergenic |
| ENSOANG00000048875 | ENSOANG00000048875 | O_anatinus | Animals | mono_intergenic | H/ACA | | | intergenic |
| ENSOANG00000048876 | ENSOANG00000048876 | O_anatinus | Animals | mono_intergenic | H/ACA | | | intergenic |
| ENSOANG00000048878 | ENSOANG00000048878 | O_anatinus | Animals | mono_intronic | H/ACA | ENSOANG00000003647 | ATR | protein_coding |
| ENSOANG00000048881 | ENSOANG00000048881 | O_anatinus | Animals | mono_intronic | H/ACA | ENSOANG00000050506 | | non_coding |
| ENSOANG00000048889 | ENSOANG00000048889 | O_anatinus | Animals | mono_intergenic | H/ACA | | | intergenic |
| ENSOANG00000048891 | ENSOANG00000048891 | O_anatinus | Animals | mono_intronic | H/ACA | ENSOANG00000041984 | DIS3L2 | protein_coding |
| ENSOANG00000048892 | U3 | O_anatinus | Animals | mono_intergenic | C/D | | | intergenic |
| ENSOANG00000048893 | ENSOANG00000048893 | O_anatinus | Animals | mono_intergenic | H/ACA | | | intergenic |
| ENSOANG00000048896 | ENSOANG00000048896 | O_anatinus | Animals | mono_intergenic | H/ACA | | | intergenic |
| ENSOANG00000048898 | ENSOANG00000048898 | O_anatinus | Animals | mono_intergenic | H/ACA | | | intergenic |
| ENSOANG00000048899 | ENSOANG00000048899 | O_anatinus | Animals | mono_intergenic | H/ACA | | | intergenic |
| ENSOANG00000048902 | ENSOANG00000048902 | O_anatinus | Animals | mono_intergenic | H/ACA | | | intergenic |
| ENSOANG00000048903 | ENSOANG00000048903 | O_anatinus | Animals | mono_intronic | H/ACA | ENSOANG00000014878 | TMEM132B | protein_coding |
| ENSOANG00000048907 | ENSOANG00000048907 | O_anatinus | Animals | mono_intergenic | H/ACA | | | intergenic |
| ENSOANG00000048913 | ENSOANG00000048913 | O_anatinus | Animals | mono_intronic | H/ACA | ENSOANG00000005622 | DSG1 | protein_coding |
| ENSOANG00000048914 | ENSOANG00000048914 | O_anatinus | Animals | mono_intergenic | H/ACA | | | intergenic |
| ENSOANG00000048921 | ENSOANG00000048921 | O_anatinus | Animals | mono_intergenic | H/ACA | | | intergenic |
| ENSOANG00000048922 | ENSOANG00000048922 | O_anatinus | Animals | mono_intergenic | H/ACA | | | intergenic |
| ENSOANG00000048925 | ENSOANG00000048925 | O_anatinus | Animals | mono_intergenic | H/ACA | | | intergenic |
| ENSOANG00000048926 | ENSOANG00000048926 | O_anatinus | Animals | mono_intronic | H/ACA | ENSOANG00000014483 | SRGAP3 | protein_coding |
| ENSOANG00000048931 | ENSOANG00000048931 | O_anatinus | Animals | mono_intergenic | H/ACA | | | intergenic |
| ENSOANG00000048941 | ENSOANG00000048941 | O_anatinus | Animals | mono_intronic | H/ACA | ENSOANG00000015645 | GAS6 | protein_coding |
| ENSOANG00000048945 | ENSOANG00000048945 | O_anatinus | Animals | mono_intergenic | H/ACA | | | intergenic |
| ENSOANG00000048946 | ENSOANG00000048946 | O_anatinus | Animals | mono_intronic | H/ACA | ENSOANG00000010322 | KLHL5 | protein_coding |
| ENSOANG00000048947 | ENSOANG00000048947 | O_anatinus | Animals | mono_intergenic | H/ACA | | | intergenic |
| ENSOANG00000048948 | ENSOANG00000048948 | O_anatinus | Animals | mono_intergenic | H/ACA | | | intergenic |
| ENSOANG00000048949 | ENSOANG00000048949 | O_anatinus | Animals | mono_intergenic | H/ACA | | | intergenic |
| ENSOANG00000048952 | ENSOANG00000048952 | O_anatinus | Animals | mono_intergenic | H/ACA | | | intergenic |
| ENSOANG00000048953 | ENSOANG00000048953 | O_anatinus | Animals | mono_intergenic | H/ACA | | | intergenic |
| ENSOANG00000048956 | ENSOANG00000048956 | O_anatinus | Animals | mono_intergenic | H/ACA | | | intergenic |
| ENSOANG00000048958 | ENSOANG00000048958 | O_anatinus | Animals | mono_intergenic | H/ACA | | | intergenic |
| ENSOANG00000048959 | ENSOANG00000048959 | O_anatinus | Animals | mono_intergenic | H/ACA | | | intergenic |
| ENSOANG00000048961 | ENSOANG00000048961 | O_anatinus | Animals | mono_intergenic | H/ACA | | | intergenic |
| ENSOANG00000048962 | ENSOANG00000048962 | O_anatinus | Animals | mono_intergenic | H/ACA | | | intergenic |
| ENSOANG00000048963 | ENSOANG00000048963 | O_anatinus | Animals | mono_intergenic | H/ACA | | | intergenic |
| ENSOANG00000048964 | ENSOANG00000048964 | O_anatinus | Animals | intronic_cluster | H/ACA | ENSOANG00000001081 | | protein_coding |

| | | | | | | | | |
|---|---|---|---|---|---|---|---|---|
| ENSOANG00000048965 | ENSOANG00000048965 | O_anatinus | Animals | mono_intergenic | H/ACA | | | intergenic |
| ENSOANG00000048966 | ENSOANG00000048966 | O_anatinus | Animals | mono_intergenic | H/ACA | | | intergenic |
| ENSOANG00000048967 | ENSOANG00000048967 | O_anatinus | Animals | mono_intergenic | H/ACA | | | intergenic |
| ENSOANG00000048969 | ENSOANG00000048969 | O_anatinus | Animals | mono_intronic | H/ACA | ENSOANG00000009560 | CSMD3 | protein_coding |
| ENSOANG00000048970 | ENSOANG00000048970 | O_anatinus | Animals | mono_intergenic | H/ACA | | | intergenic |
| ENSOANG00000048971 | ENSOANG00000048971 | O_anatinus | Animals | mono_intergenic | H/ACA | | | intergenic |
| ENSOANG00000048972 | ENSOANG00000048972 | O_anatinus | Animals | mono_intergenic | H/ACA | | | intergenic |
| ENSOANG00000048976 | ENSOANG00000048976 | O_anatinus | Animals | mono_intergenic | H/ACA | | | intergenic |
| ENSOANG00000048980 | ENSOANG00000048980 | O_anatinus | Animals | mono_intergenic | H/ACA | | | intergenic |
| ENSOANG00000048981 | ENSOANG00000048981 | O_anatinus | Animals | mono_intergenic | H/ACA | | | intergenic |
| ENSOANG00000048983 | ENSOANG00000048983 | O_anatinus | Animals | mono_intergenic | H/ACA | | | intergenic |
| ENSOANG00000048984 | ENSOANG00000048984 | O_anatinus | Animals | intronic_cluster | H/ACA | ENSOANG00000029831 | AGMO | protein_coding |
| ENSOANG00000048985 | ENSOANG00000048985 | O_anatinus | Animals | mono_intergenic | H/ACA | | | intergenic |
| ENSOANG00000048986 | ENSOANG00000048986 | O_anatinus | Animals | mono_intergenic | H/ACA | | | intergenic |
| ENSOANG00000048987 | ENSOANG00000048987 | O_anatinus | Animals | mono_intergenic | H/ACA | | | intergenic |
| ENSOANG00000048988 | ENSOANG00000048988 | O_anatinus | Animals | intronic_cluster | H/ACA | ENSOANG00000009950 | TMEM132D | protein_coding |
| ENSOANG00000048996 | ENSOANG00000048996 | O_anatinus | Animals | mono_intronic | H/ACA | ENSOANG00000020878 | | protein_coding |
| ENSOANG00000048998 | ENSOANG00000048998 | O_anatinus | Animals | mono_intronic | H/ACA | ENSOANG00000001217 | ERAP2 | protein_coding |
| ENSOANG00000049001 | ENSOANG00000049001 | O_anatinus | Animals | mono_intergenic | H/ACA | | | intergenic |
| ENSOANG00000049008 | ENSOANG00000049008 | O_anatinus | Animals | mono_intergenic | H/ACA | | | intergenic |
| ENSOANG00000049010 | ENSOANG00000049010 | O_anatinus | Animals | mono_intergenic | H/ACA | | | intergenic |
| ENSOANG00000049011 | ENSOANG00000049011 | O_anatinus | Animals | mono_intergenic | H/ACA | | | intergenic |
| ENSOANG00000049012 | ENSOANG00000049012 | O_anatinus | Animals | mono_intergenic | H/ACA | | | intergenic |
| ENSOANG00000049015 | ENSOANG00000049015 | O_anatinus | Animals | mono_intergenic | H/ACA | | | intergenic |
| ENSOANG00000049017 | ENSOANG00000049017 | O_anatinus | Animals | mono_intergenic | H/ACA | | | intergenic |
| ENSOANG00000049019 | ENSOANG00000049019 | O_anatinus | Animals | mono_intergenic | H/ACA | | | intergenic |
| ENSOANG00000049020 | ENSOANG00000049020 | O_anatinus | Animals | mono_intergenic | H/ACA | | | intergenic |
| ENSOANG00000049024 | ENSOANG00000049024 | O_anatinus | Animals | mono_intergenic | H/ACA | | | intergenic |
| ENSOANG00000049026 | ENSOANG00000049026 | O_anatinus | Animals | mono_intergenic | H/ACA | | | intergenic |
| ENSOANG00000049027 | ENSOANG00000049027 | O_anatinus | Animals | mono_intergenic | H/ACA | | | intergenic |
| ENSOANG00000049028 | ENSOANG00000049028 | O_anatinus | Animals | mono_intronic | H/ACA | ENSOANG00000050649 | | non_coding |
| ENSOANG00000049029 | ENSOANG00000049029 | O_anatinus | Animals | mono_intergenic | H/ACA | | | intergenic |
| ENSOANG00000049030 | ENSOANG00000049030 | O_anatinus | Animals | mono_intergenic | H/ACA | | | intergenic |
| ENSOANG00000049035 | ENSOANG00000049035 | O_anatinus | Animals | mono_intergenic | H/ACA | | | intergenic |
| ENSOANG00000049036 | ENSOANG00000049036 | O_anatinus | Animals | mono_intronic | C/D | ENSOANG00000014467 | WDR43 | protein_coding |
| ENSOANG00000049042 | ENSOANG00000049042 | O_anatinus | Animals | mono_intronic | H/ACA | ENSOANG00000014468 | | protein_coding |
| ENSOANG00000049047 | ENSOANG00000049047 | O_anatinus | Animals | mono_intergenic | H/ACA | | | intergenic |
| ENSOANG00000049048 | ENSOANG00000049048 | O_anatinus | Animals | mono_intergenic | H/ACA | | | intergenic |
| ENSOANG00000049049 | ENSOANG00000049049 | O_anatinus | Animals | mono_intergenic | H/ACA | | | intergenic |
| ENSOANG00000049053 | ENSOANG00000049053 | O_anatinus | Animals | mono_intergenic | H/ACA | | | intergenic |
| ENSOANG00000049058 | ENSOANG00000049058 | O_anatinus | Animals | mono_intergenic | H/ACA | | | intergenic |

| | | | | | | | | |
|---|---|---|---|---|---|---|---|---|
| ENSOANG00000049062 | SNORD73 | O_anatinus | Animals | mono_intronic | C/D | ENSOANG00000000209 | | protein_coding |
| ENSOANG00000049063 | ENSOANG00000049063 | O_anatinus | Animals | mono_intergenic | H/ACA | | | intergenic |
| ENSOANG00000049064 | ENSOANG00000049064 | O_anatinus | Animals | mono_intergenic | H/ACA | | | intergenic |
| ENSOANG00000049065 | ENSOANG00000049065 | O_anatinus | Animals | mono_intergenic | H/ACA | | | intergenic |
| ENSOANG00000049066 | ENSOANG00000049066 | O_anatinus | Animals | mono_intergenic | H/ACA | | | intergenic |
| ENSOANG00000049070 | ENSOANG00000049070 | O_anatinus | Animals | mono_intergenic | H/ACA | | | intergenic |
| ENSOANG00000049075 | ENSOANG00000049075 | O_anatinus | Animals | mono_intergenic | H/ACA | | | intergenic |
| ENSOANG00000049081 | ENSOANG00000049081 | O_anatinus | Animals | mono_intergenic | H/ACA | | | intergenic |
| ENSOANG00000049084 | ENSOANG00000049084 | O_anatinus | Animals | mono_intergenic | H/ACA | | | intergenic |
| ENSOANG00000049086 | ENSOANG00000049086 | O_anatinus | Animals | mono_intergenic | H/ACA | | | intergenic |
| ENSOANG00000049087 | ENSOANG00000049087 | O_anatinus | Animals | mono_intergenic | H/ACA | | | intergenic |
| ENSOANG00000049092 | ENSOANG00000049092 | O_anatinus | Animals | mono_intergenic | H/ACA | | | intergenic |
| ENSOANG00000049094 | ENSOANG00000049094 | O_anatinus | Animals | mono_intergenic | H/ACA | | | intergenic |
| ENSOANG00000049095 | ENSOANG00000049095 | O_anatinus | Animals | mono_intergenic | H/ACA | | | intergenic |
| ENSOANG00000049097 | ENSOANG00000049097 | O_anatinus | Animals | mono_intergenic | H/ACA | | | intergenic |
| ENSOANG00000049098 | ENSOANG00000049098 | O_anatinus | Animals | mono_intergenic | H/ACA | | | intergenic |
| ENSOANG00000049099 | ENSOANG00000049099 | O_anatinus | Animals | mono_intergenic | H/ACA | | | intergenic |
| ENSOANG00000049100 | ENSOANG00000049100 | O_anatinus | Animals | mono_intronic | H/ACA | ENSOANG00000001775 | ZFAND4 | protein_coding |
| ENSOANG00000049105 | ENSOANG00000049105 | O_anatinus | Animals | mono_intergenic | H/ACA | | | intergenic |
| ENSOANG00000049106 | ENSOANG00000049106 | O_anatinus | Animals | mono_intergenic | H/ACA | | | intergenic |
| ENSOANG00000049107 | ENSOANG00000049107 | O_anatinus | Animals | mono_intergenic | H/ACA | | | intergenic |
| ENSOANG00000049109 | ENSOANG00000049109 | O_anatinus | Animals | mono_intronic | H/ACA | ENSOANG00000041205 | | non_coding |
| ENSOANG00000049116 | ENSOANG00000049116 | O_anatinus | Animals | mono_intergenic | H/ACA | | | intergenic |
| ENSOANG00000049118 | ENSOANG00000049118 | O_anatinus | Animals | mono_intergenic | H/ACA | | | intergenic |
| ENSOANG00000049120 | ENSOANG00000049120 | O_anatinus | Animals | mono_intergenic | H/ACA | | | intergenic |
| ENSOANG00000049121 | ENSOANG00000049121 | O_anatinus | Animals | mono_intergenic | H/ACA | | | intergenic |
| ENSOANG00000049124 | ENSOANG00000049124 | O_anatinus | Animals | mono_intronic | H/ACA | ENSOANG00000012398 | SLC24A2 | protein_coding |
| ENSOANG00000049126 | ENSOANG00000049126 | O_anatinus | Animals | mono_intergenic | H/ACA | | | intergenic |
| ENSOANG00000049129 | ENSOANG00000049129 | O_anatinus | Animals | intergenic_cluster | C/D | | | intergenic |
| ENSOANG00000049133 | SNORA65 | O_anatinus | Animals | mono_intronic | H/ACA | ENSOANG00000013186 | RPL12 | protein_coding |
| ENSOANG00000049148 | ENSOANG00000049148 | O_anatinus | Animals | mono_intronic | H/ACA | ENSOANG00000010621 | CEP128 | protein_coding |
| ENSOANG00000049150 | ENSOANG00000049150 | O_anatinus | Animals | mono_intergenic | H/ACA | | | intergenic |
| ENSOANG00000049151 | ENSOANG00000049151 | O_anatinus | Animals | mono_intergenic | H/ACA | | | intergenic |
| ENSOANG00000049152 | ENSOANG00000049152 | O_anatinus | Animals | mono_intergenic | H/ACA | | | intergenic |
| ENSOANG00000049155 | ENSOANG00000049155 | O_anatinus | Animals | mono_intergenic | H/ACA | | | intergenic |
| ENSOANG00000049160 | ENSOANG00000049160 | O_anatinus | Animals | mono_intergenic | H/ACA | | | intergenic |
| ENSOANG00000049163 | ENSOANG00000049163 | O_anatinus | Animals | mono_intergenic | H/ACA | | | intergenic |
| ENSOANG00000049176 | ENSOANG00000049176 | O_anatinus | Animals | mono_intergenic | H/ACA | | | intergenic |
| ENSOANG00000049180 | ENSOANG00000049180 | O_anatinus | Animals | mono_intronic | H/ACA | ENSOANG00000005612 | KLHL14 | protein_coding |
| ENSOANG00000049182 | ENSOANG00000049182 | O_anatinus | Animals | mono_intergenic | H/ACA | | | intergenic |
| ENSOANG00000049183 | ENSOANG00000049183 | O_anatinus | Animals | mono_intergenic | H/ACA | | | intergenic |

| | | | | | | | | |
|---|---|---|---|---|---|---|---|---|
| ENSOANG00000049186 | ENSOANG00000049186 | O_anatinus | Animals | mono_intergenic | H/ACA | | | intergenic |
| ENSOANG00000049192 | ENSOANG00000049192 | O_anatinus | Animals | mono_intergenic | H/ACA | | | intergenic |
| ENSOANG00000049193 | ENSOANG00000049193 | O_anatinus | Animals | mono_intergenic | H/ACA | | | intergenic |
| ENSOANG00000049195 | ENSOANG00000049195 | O_anatinus | Animals | mono_intergenic | H/ACA | | | intergenic |
| ENSOANG00000049196 | ENSOANG00000049196 | O_anatinus | Animals | mono_intronic | H/ACA | ENSOANG00000048416 | GCM1 | protein_coding |
| ENSOANG00000049198 | ENSOANG00000049198 | O_anatinus | Animals | mono_intronic | H/ACA | ENSOANG00000011351 | VIRMA | protein_coding |
| ENSOANG00000049202 | ENSOANG00000049202 | O_anatinus | Animals | mono_intergenic | H/ACA | | | intergenic |
| ENSOANG00000049207 | ENSOANG00000049207 | O_anatinus | Animals | mono_intergenic | H/ACA | | | intergenic |
| ENSOANG00000049208 | ENSOANG00000049208 | O_anatinus | Animals | mono_intergenic | H/ACA | | | intergenic |
| ENSOANG00000049209 | ENSOANG00000049209 | O_anatinus | Animals | mono_intergenic | H/ACA | | | intergenic |
| ENSOANG00000049210 | ENSOANG00000049210 | O_anatinus | Animals | mono_intergenic | H/ACA | | | intergenic |
| ENSOANG00000049213 | ENSOANG00000049213 | O_anatinus | Animals | mono_intergenic | H/ACA | | | intergenic |
| ENSOANG00000049214 | ENSOANG00000049214 | O_anatinus | Animals | mono_intronic | H/ACA | ENSOANG00000004496 | CDH8 | protein_coding |
| ENSOANG00000049215 | ENSOANG00000049215 | O_anatinus | Animals | mono_intergenic | H/ACA | | | intergenic |
| ENSOANG00000049216 | ENSOANG00000049216 | O_anatinus | Animals | mono_intergenic | H/ACA | | | intergenic |
| ENSOANG00000049219 | ENSOANG00000049219 | O_anatinus | Animals | mono_intergenic | H/ACA | | | intergenic |
| ENSOANG00000049223 | ENSOANG00000049223 | O_anatinus | Animals | mono_intergenic | H/ACA | | | intergenic |
| ENSOANG00000049227 | ENSOANG00000049227 | O_anatinus | Animals | mono_intergenic | H/ACA | | | intergenic |
| ENSOANG00000049230 | ENSOANG00000049230 | O_anatinus | Animals | mono_intergenic | H/ACA | | | intergenic |
| ENSOANG00000049231 | ENSOANG00000049231 | O_anatinus | Animals | mono_intergenic | H/ACA | | | intergenic |
| ENSOANG00000049233 | ENSOANG00000049233 | O_anatinus | Animals | mono_intergenic | H/ACA | | | intergenic |
| ENSOANG00000049235 | ENSOANG00000049235 | O_anatinus | Animals | mono_intronic | H/ACA | ENSOANG00000003850 | DPYD | protein_coding |
| ENSOANG00000049236 | ENSOANG00000049236 | O_anatinus | Animals | mono_intergenic | H/ACA | | | intergenic |
| ENSOANG00000049239 | ENSOANG00000049239 | O_anatinus | Animals | mono_intergenic | H/ACA | | | intergenic |
| ENSOANG00000049244 | ENSOANG00000049244 | O_anatinus | Animals | mono_intergenic | H/ACA | | | intergenic |
| ENSOANG00000049247 | ENSOANG00000049247 | O_anatinus | Animals | mono_intergenic | H/ACA | | | intergenic |
| ENSOANG00000049248 | ENSOANG00000049248 | O_anatinus | Animals | mono_intergenic | H/ACA | | | intergenic |
| ENSOANG00000049249 | ENSOANG00000049249 | O_anatinus | Animals | mono_intergenic | H/ACA | | | intergenic |
| ENSOANG00000049251 | ENSOANG00000049251 | O_anatinus | Animals | mono_intergenic | H/ACA | | | intergenic |
| ENSOANG00000049258 | ENSOANG00000049258 | O_anatinus | Animals | mono_intergenic | H/ACA | | | intergenic |
| ENSOANG00000049263 | ENSOANG00000049263 | O_anatinus | Animals | intronic_cluster | H/ACA | ENSOANG00000013591 | | protein_coding |
| ENSOANG00000049267 | ENSOANG00000049267 | O_anatinus | Animals | mono_intronic | H/ACA | ENSOANG00000006816 | | protein_coding |
| ENSOANG00000049269 | ENSOANG00000049269 | O_anatinus | Animals | mono_intergenic | H/ACA | | | intergenic |
| ENSOANG00000049270 | ENSOANG00000049270 | O_anatinus | Animals | mono_intergenic | H/ACA | | | intergenic |
| ENSOANG00000049272 | ENSOANG00000049272 | O_anatinus | Animals | mono_intergenic | H/ACA | | | intergenic |
| ENSOANG00000049275 | ENSOANG00000049275 | O_anatinus | Animals | mono_intronic | H/ACA | ENSOANG00000043031 | | protein_coding |
| ENSOANG00000049276 | ENSOANG00000049276 | O_anatinus | Animals | mono_intronic | H/ACA | ENSOANG00000014120 | MYOM2 | protein_coding |
| ENSOANG00000049277 | ENSOANG00000049277 | O_anatinus | Animals | mono_intronic | C/D | ENSOANG00000001886 | RPS12 | protein_coding |
| ENSOANG00000049278 | ENSOANG00000049278 | O_anatinus | Animals | mono_intergenic | H/ACA | | | intergenic |
| ENSOANG00000049286 | ENSOANG00000049286 | O_anatinus | Animals | mono_intergenic | H/ACA | | | intergenic |
| ENSOANG00000049287 | ENSOANG00000049287 | O_anatinus | Animals | mono_intergenic | H/ACA | | | intergenic |

| | | | | | | | | |
|---|---|---|---|---|---|---|---|---|
| ENSOANG00000049292 | ENSOANG00000049292 | O_anatinus | Animals | mono_intergenic | H/ACA | | | intergenic |
| ENSOANG00000049299 | ENSOANG00000049299 | O_anatinus | Animals | mono_intergenic | H/ACA | | | intergenic |
| ENSOANG00000049303 | ENSOANG00000049303 | O_anatinus | Animals | mono_intergenic | H/ACA | | | intergenic |
| ENSOANG00000049306 | ENSOANG00000049306 | O_anatinus | Animals | mono_intergenic | H/ACA | | | intergenic |
| ENSOANG00000049307 | ENSOANG00000049307 | O_anatinus | Animals | mono_intergenic | H/ACA | | | intergenic |
| ENSOANG00000049309 | ENSOANG00000049309 | O_anatinus | Animals | mono_intergenic | H/ACA | | | intergenic |
| ENSOANG00000049316 | ENSOANG00000049316 | O_anatinus | Animals | mono_intergenic | H/ACA | | | intergenic |
| ENSOANG00000049317 | ENSOANG00000049317 | O_anatinus | Animals | mono_intergenic | H/ACA | | | intergenic |
| ENSOANG00000049318 | ENSOANG00000049318 | O_anatinus | Animals | mono_intergenic | H/ACA | | | intergenic |
| ENSOANG00000049320 | ENSOANG00000049320 | O_anatinus | Animals | mono_intergenic | H/ACA | | | intergenic |
| ENSOANG00000049323 | ENSOANG00000049323 | O_anatinus | Animals | mono_intergenic | H/ACA | | | intergenic |
| ENSOANG00000049327 | ENSOANG00000049327 | O_anatinus | Animals | mono_intergenic | H/ACA | | | intergenic |
| ENSOANG00000049331 | ENSOANG00000049331 | O_anatinus | Animals | mono_intergenic | H/ACA | | | intergenic |
| ENSOANG00000049332 | ENSOANG00000049332 | O_anatinus | Animals | mono_intronic | H/ACA | ENSOANG00000007407 | SHANK2 | protein_coding |
| ENSOANG00000049333 | ENSOANG00000049333 | O_anatinus | Animals | mono_intronic | H/ACA | ENSOANG00000014549 | TENM1 | protein_coding |
| ENSOANG00000049339 | ENSOANG00000049339 | O_anatinus | Animals | mono_intergenic | H/ACA | | | intergenic |
| ENSOANG00000049340 | ENSOANG00000049340 | O_anatinus | Animals | intronic_cluster | H/ACA | ENSOANG00000043246 | | protein_coding |
| ENSOANG00000049341 | ENSOANG00000049341 | O_anatinus | Animals | mono_intronic | H/ACA | ENSOANG00000050772 | KCNN2 | protein_coding |
| ENSOANG00000049346 | ENSOANG00000049346 | O_anatinus | Animals | mono_intronic | H/ACA | ENSOANG00000007867 | | protein_coding |
| ENSOANG00000049353 | ENSOANG00000049353 | O_anatinus | Animals | mono_intronic | H/ACA | ENSOANG00000003682 | MATN2 | protein_coding |
| ENSOANG00000049355 | ENSOANG00000049355 | O_anatinus | Animals | mono_intergenic | H/ACA | | | intergenic |
| ENSOANG00000049356 | ENSOANG00000049356 | O_anatinus | Animals | mono_intergenic | H/ACA | | | intergenic |
| ENSOANG00000049357 | ENSOANG00000049357 | O_anatinus | Animals | mono_intergenic | H/ACA | | | intergenic |
| ENSOANG00000049360 | ENSOANG00000049360 | O_anatinus | Animals | mono_intergenic | H/ACA | | | intergenic |
| ENSOANG00000049361 | ENSOANG00000049361 | O_anatinus | Animals | mono_intronic | H/ACA | ENSOANG00000049985 | RDH14 | protein_coding |
| ENSOANG00000049362 | ENSOANG00000049362 | O_anatinus | Animals | mono_intronic | H/ACA | ENSOANG00000015432 | CELF2 | protein_coding |
| ENSOANG00000049363 | ENSOANG00000049363 | O_anatinus | Animals | mono_intergenic | H/ACA | | | intergenic |
| ENSOANG00000049365 | ENSOANG00000049365 | O_anatinus | Animals | mono_intergenic | H/ACA | | | intergenic |
| ENSOANG00000049367 | ENSOANG00000049367 | O_anatinus | Animals | mono_intergenic | H/ACA | | | intergenic |
| ENSOANG00000049368 | ENSOANG00000049368 | O_anatinus | Animals | mono_intergenic | H/ACA | | | intergenic |
| ENSOANG00000049369 | ENSOANG00000049369 | O_anatinus | Animals | mono_intergenic | H/ACA | | | intergenic |
| ENSOANG00000049372 | ENSOANG00000049372 | O_anatinus | Animals | mono_intergenic | H/ACA | | | intergenic |
| ENSOANG00000049374 | ENSOANG00000049374 | O_anatinus | Animals | mono_intergenic | H/ACA | | | intergenic |
| ENSOANG00000049376 | ENSOANG00000049376 | O_anatinus | Animals | mono_intergenic | H/ACA | | | intergenic |
| ENSOANG00000049377 | ENSOANG00000049377 | O_anatinus | Animals | mono_intergenic | H/ACA | | | intergenic |
| ENSOANG00000049381 | ENSOANG00000049381 | O_anatinus | Animals | mono_intergenic | H/ACA | | | intergenic |
| ENSOANG00000049383 | ENSOANG00000049383 | O_anatinus | Animals | mono_intergenic | H/ACA | | | intergenic |
| ENSOANG00000049386 | ENSOANG00000049386 | O_anatinus | Animals | mono_intergenic | H/ACA | | | intergenic |
| ENSOANG00000049387 | ENSOANG00000049387 | O_anatinus | Animals | mono_intergenic | H/ACA | | | intergenic |
| ENSOANG00000049390 | ENSOANG00000049390 | O_anatinus | Animals | mono_intergenic | H/ACA | | | intergenic |
| ENSOANG00000049392 | ENSOANG00000049392 | O_anatinus | Animals | mono_intronic | H/ACA | ENSOANG00000038954 | | protein_coding |

| | | | | | | | | |
|---|---|---|---|---|---|---|---|---|
| ENSOANG00000049393 | ENSOANG00000049393 | O_anatinus | Animals | mono_intergenic | H/ACA | | | intergenic |
| ENSOANG00000049394 | ENSOANG00000049394 | O_anatinus | Animals | mono_intergenic | H/ACA | | | intergenic |
| ENSOANG00000049396 | ENSOANG00000049396 | O_anatinus | Animals | mono_intergenic | H/ACA | | | intergenic |
| ENSOANG00000049397 | ENSOANG00000049397 | O_anatinus | Animals | mono_intergenic | H/ACA | | | intergenic |
| ENSOANG00000049398 | ENSOANG00000049398 | O_anatinus | Animals | mono_intergenic | H/ACA | | | intergenic |
| ENSOANG00000049399 | ENSOANG00000049399 | O_anatinus | Animals | mono_intronic | H/ACA | ENSOANG00000008490 | STK39 | protein_coding |
| ENSOANG00000049403 | ENSOANG00000049403 | O_anatinus | Animals | mono_intronic | H/ACA | ENSOANG00000013116 | CNTNAP5 | protein_coding |
| ENSOANG00000049404 | ENSOANG00000049404 | O_anatinus | Animals | mono_intergenic | H/ACA | | | intergenic |
| ENSOANG00000049409 | ENSOANG00000049409 | O_anatinus | Animals | mono_intergenic | H/ACA | | | intergenic |
| ENSOANG00000049412 | ENSOANG00000049412 | O_anatinus | Animals | mono_intergenic | H/ACA | | | intergenic |
| ENSOANG00000049413 | ENSOANG00000049413 | O_anatinus | Animals | mono_intronic | H/ACA | ENSOANG00000014979 | ARHGEF33 | protein_coding |
| ENSOANG00000049416 | ENSOANG00000049416 | O_anatinus | Animals | mono_intergenic | H/ACA | | | intergenic |
| ENSOANG00000049417 | ENSOANG00000049417 | O_anatinus | Animals | mono_intergenic | H/ACA | | | intergenic |
| ENSOANG00000049420 | ENSOANG00000049420 | O_anatinus | Animals | mono_intergenic | H/ACA | | | intergenic |
| ENSOANG00000049427 | ENSOANG00000049427 | O_anatinus | Animals | mono_intergenic | H/ACA | | | intergenic |
| ENSOANG00000049428 | ENSOANG00000049428 | O_anatinus | Animals | mono_intergenic | H/ACA | | | intergenic |
| ENSOANG00000049429 | ENSOANG00000049429 | O_anatinus | Animals | mono_intergenic | H/ACA | | | intergenic |
| ENSOANG00000049430 | ENSOANG00000049430 | O_anatinus | Animals | mono_intergenic | H/ACA | | | intergenic |
| ENSOANG00000049433 | ENSOANG00000049433 | O_anatinus | Animals | mono_intergenic | H/ACA | | | intergenic |
| ENSOANG00000049439 | ENSOANG00000049439 | O_anatinus | Animals | mono_intergenic | H/ACA | | | intergenic |
| ENSOANG00000049442 | ENSOANG00000049442 | O_anatinus | Animals | mono_intergenic | H/ACA | | | intergenic |
| ENSOANG00000049443 | ENSOANG00000049443 | O_anatinus | Animals | mono_intergenic | H/ACA | | | intergenic |
| ENSOANG00000049446 | ENSOANG00000049446 | O_anatinus | Animals | mono_intergenic | H/ACA | | | intergenic |
| ENSOANG00000049447 | ENSOANG00000049447 | O_anatinus | Animals | mono_intergenic | H/ACA | | | intergenic |
| ENSOANG00000049449 | ENSOANG00000049449 | O_anatinus | Animals | mono_intergenic | H/ACA | | | intergenic |
| ENSOANG00000049450 | ENSOANG00000049450 | O_anatinus | Animals | mono_intergenic | H/ACA | | | intergenic |
| ENSOANG00000049451 | ENSOANG00000049451 | O_anatinus | Animals | mono_intergenic | H/ACA | | | intergenic |
| ENSOANG00000049453 | ENSOANG00000049453 | O_anatinus | Animals | mono_intergenic | H/ACA | | | intergenic |
| ENSOANG00000049461 | ENSOANG00000049461 | O_anatinus | Animals | mono_intergenic | H/ACA | | | intergenic |
| ENSOANG00000049462 | ENSOANG00000049462 | O_anatinus | Animals | mono_intergenic | H/ACA | | | intergenic |
| ENSOANG00000049464 | ENSOANG00000049464 | O_anatinus | Animals | mono_intergenic | H/ACA | | | intergenic |
| ENSOANG00000049467 | ENSOANG00000049467 | O_anatinus | Animals | intronic_cluster | H/ACA | ENSOANG00000005642 | NR5A2 | protein_coding |
| ENSOANG00000049468 | ENSOANG00000049468 | O_anatinus | Animals | mono_intergenic | H/ACA | | | intergenic |
| ENSOANG00000049469 | ENSOANG00000049469 | O_anatinus | Animals | mono_intergenic | H/ACA | | | intergenic |
| ENSOANG00000049472 | ENSOANG00000049472 | O_anatinus | Animals | mono_intergenic | H/ACA | | | intergenic |
| ENSOANG00000049473 | ENSOANG00000049473 | O_anatinus | Animals | mono_intergenic | H/ACA | | | intergenic |
| ENSOANG00000049474 | ENSOANG00000049474 | O_anatinus | Animals | mono_intergenic | H/ACA | | | intergenic |
| ENSOANG00000049475 | ENSOANG00000049475 | O_anatinus | Animals | mono_intergenic | H/ACA | | | intergenic |
| ENSOANG00000049476 | ENSOANG00000049476 | O_anatinus | Animals | mono_intergenic | H/ACA | | | intergenic |
| ENSOANG00000049479 | ENSOANG00000049479 | O_anatinus | Animals | mono_intergenic | H/ACA | | | intergenic |
| ENSOANG00000049480 | ENSOANG00000049480 | O_anatinus | Animals | mono_intergenic | H/ACA | | | intergenic |

| | | | | | | | | |
|---|---|---|---|---|---|---|---|---|
| ENSOANG00000049482 | ENSOANG00000049482 | O_anatinus | Animals | mono_intergenic | H/ACA | | | intergenic |
| ENSOANG00000049486 | ENSOANG00000049486 | O_anatinus | Animals | mono_intergenic | H/ACA | | | intergenic |
| ENSOANG00000049488 | ENSOANG00000049488 | O_anatinus | Animals | mono_intergenic | H/ACA | | | intergenic |
| ENSOANG00000049490 | ENSOANG00000049490 | O_anatinus | Animals | mono_intronic | H/ACA | ENSOANG00000004341 | HP1BP3 | protein_coding |
| ENSOANG00000049491 | ENSOANG00000049491 | O_anatinus | Animals | mono_intergenic | H/ACA | | | intergenic |
| ENSOANG00000049492 | ENSOANG00000049492 | O_anatinus | Animals | mono_intergenic | H/ACA | | | intergenic |
| ENSOANG00000049493 | ENSOANG00000049493 | O_anatinus | Animals | mono_intergenic | H/ACA | | | intergenic |
| ENSOANG00000049494 | ENSOANG00000049494 | O_anatinus | Animals | mono_intronic | H/ACA | ENSOANG00000001686 | FAM3D | protein_coding |
| ENSOANG00000049495 | ENSOANG00000049495 | O_anatinus | Animals | mono_intergenic | H/ACA | | | intergenic |
| ENSOANG00000049502 | ENSOANG00000049502 | O_anatinus | Animals | mono_intergenic | H/ACA | | | intergenic |
| ENSOANG00000049505 | ENSOANG00000049505 | O_anatinus | Animals | mono_intergenic | H/ACA | | | intergenic |
| ENSOANG00000049506 | ENSOANG00000049506 | O_anatinus | Animals | mono_intergenic | H/ACA | | | intergenic |
| ENSOANG00000049508 | ENSOANG00000049508 | O_anatinus | Animals | mono_intergenic | H/ACA | | | intergenic |
| ENSOANG00000049512 | ENSOANG00000049512 | O_anatinus | Animals | mono_intergenic | H/ACA | | | intergenic |
| ENSOANG00000049520 | ENSOANG00000049520 | O_anatinus | Animals | mono_intergenic | H/ACA | | | intergenic |
| ENSOANG00000049523 | ENSOANG00000049523 | O_anatinus | Animals | mono_intergenic | H/ACA | | | intergenic |
| ENSOANG00000049525 | ENSOANG00000049525 | O_anatinus | Animals | mono_intergenic | H/ACA | | | intergenic |
| ENSOANG00000049526 | ENSOANG00000049526 | O_anatinus | Animals | mono_intergenic | H/ACA | | | intergenic |
| ENSOANG00000049529 | ENSOANG00000049529 | O_anatinus | Animals | mono_intergenic | H/ACA | | | intergenic |
| ENSOANG00000049532 | ENSOANG00000049532 | O_anatinus | Animals | mono_intronic | H/ACA | ENSOANG00000049141 | FBXO36 | protein_coding |
| ENSOANG00000049535 | ENSOANG00000049535 | O_anatinus | Animals | intergenic_cluster | H/ACA | | | intergenic |
| ENSOANG00000049536 | ENSOANG00000049536 | O_anatinus | Animals | mono_intergenic | H/ACA | | | intergenic |
| ENSOANG00000049537 | ENSOANG00000049537 | O_anatinus | Animals | mono_intergenic | H/ACA | | | intergenic |
| ENSOANG00000049539 | ENSOANG00000049539 | O_anatinus | Animals | mono_intergenic | H/ACA | | | intergenic |
| ENSOANG00000049540 | ENSOANG00000049540 | O_anatinus | Animals | mono_intergenic | H/ACA | | | intergenic |
| ENSOANG00000049542 | ENSOANG00000049542 | O_anatinus | Animals | mono_intergenic | H/ACA | | | intergenic |
| ENSOANG00000049543 | ENSOANG00000049543 | O_anatinus | Animals | mono_intergenic | H/ACA | | | intergenic |
| ENSOANG00000049550 | ENSOANG00000049550 | O_anatinus | Animals | mono_intergenic | H/ACA | | | intergenic |
| ENSOANG00000049551 | ENSOANG00000049551 | O_anatinus | Animals | mono_intergenic | H/ACA | | | intergenic |
| ENSOANG00000049553 | ENSOANG00000049553 | O_anatinus | Animals | mono_intergenic | H/ACA | | | intergenic |
| ENSOANG00000049557 | ENSOANG00000049557 | O_anatinus | Animals | mono_intergenic | H/ACA | | | intergenic |
| ENSOANG00000049558 | ENSOANG00000049558 | O_anatinus | Animals | mono_intergenic | H/ACA | | | intergenic |
| ENSOANG00000049560 | ENSOANG00000049560 | O_anatinus | Animals | mono_intergenic | H/ACA | | | intergenic |
| ENSOANG00000049564 | ENSOANG00000049564 | O_anatinus | Animals | mono_intergenic | H/ACA | | | intergenic |
| ENSOANG00000049565 | ENSOANG00000049565 | O_anatinus | Animals | mono_intergenic | H/ACA | | | intergenic |
| ENSOANG00000049567 | ENSOANG00000049567 | O_anatinus | Animals | mono_intergenic | H/ACA | | | intergenic |
| ENSOANG00000049573 | ENSOANG00000049573 | O_anatinus | Animals | mono_intergenic | H/ACA | | | intergenic |
| ENSOANG00000049575 | ENSOANG00000049575 | O_anatinus | Animals | mono_intronic | H/ACA | ENSOANG00000003152 | LMO7 | protein_coding |
| ENSOANG00000049577 | ENSOANG00000049577 | O_anatinus | Animals | mono_intergenic | H/ACA | | | intergenic |
| ENSOANG00000049580 | ENSOANG00000049580 | O_anatinus | Animals | mono_intronic | H/ACA | ENSOANG00000038864 | ELMO1 | protein_coding |
| ENSOANG00000049581 | ENSOANG00000049581 | O_anatinus | Animals | mono_intergenic | H/ACA | | | intergenic |

| | | | | | | | | |
|---|---|---|---|---|---|---|---|---|
| ENSOANG00000049583 | ENSOANG00000049583 | O_anatinus | Animals | mono_intergenic | H/ACA | | | intergenic |
| ENSOANG00000049585 | ENSOANG00000049585 | O_anatinus | Animals | mono_intergenic | H/ACA | | | intergenic |
| ENSOANG00000049588 | ENSOANG00000049588 | O_anatinus | Animals | mono_intergenic | H/ACA | | | intergenic |
| ENSOANG00000049589 | ENSOANG00000049589 | O_anatinus | Animals | mono_intergenic | H/ACA | | | intergenic |
| ENSOANG00000049590 | ENSOANG00000049590 | O_anatinus | Animals | mono_intronic | H/ACA | ENSOANG00000011697 | MBNL3 | protein_coding |
| ENSOANG00000049592 | ENSOANG00000049592 | O_anatinus | Animals | mono_intergenic | H/ACA | | | intergenic |
| ENSOANG00000049593 | ENSOANG00000049593 | O_anatinus | Animals | mono_intergenic | H/ACA | | | intergenic |
| ENSOANG00000049597 | ENSOANG00000049597 | O_anatinus | Animals | mono_intergenic | H/ACA | | | intergenic |
| ENSOANG00000049601 | ENSOANG00000049601 | O_anatinus | Animals | mono_intergenic | H/ACA | | | intergenic |
| ENSOANG00000049605 | ENSOANG00000049605 | O_anatinus | Animals | mono_intergenic | H/ACA | | | intergenic |
| ENSOANG00000049608 | ENSOANG00000049608 | O_anatinus | Animals | mono_intergenic | H/ACA | | | intergenic |
| ENSOANG00000049609 | ENSOANG00000049609 | O_anatinus | Animals | mono_intronic | H/ACA | ENSOANG00000011147 | ST6GAL2 | protein_coding |
| ENSOANG00000049610 | ENSOANG00000049610 | O_anatinus | Animals | mono_intergenic | H/ACA | | | intergenic |
| ENSOANG00000049613 | ENSOANG00000049613 | O_anatinus | Animals | mono_intergenic | H/ACA | | | intergenic |
| ENSOANG00000049616 | ENSOANG00000049616 | O_anatinus | Animals | mono_intergenic | H/ACA | | | intergenic |
| ENSOANG00000049619 | ENSOANG00000049619 | O_anatinus | Animals | mono_intronic | H/ACA | ENSOANG00000007132 | HNF1B | protein_coding |
| ENSOANG00000049626 | ENSOANG00000049626 | O_anatinus | Animals | mono_intergenic | H/ACA | | | intergenic |
| ENSOANG00000049627 | ENSOANG00000049627 | O_anatinus | Animals | mono_intergenic | H/ACA | | | intergenic |
| ENSOANG00000049628 | ENSOANG00000049628 | O_anatinus | Animals | mono_intergenic | H/ACA | | | intergenic |
| ENSOANG00000049629 | ENSOANG00000049629 | O_anatinus | Animals | mono_intergenic | H/ACA | | | intergenic |
| ENSOANG00000049631 | ENSOANG00000049631 | O_anatinus | Animals | mono_intergenic | H/ACA | | | intergenic |
| ENSOANG00000049632 | ENSOANG00000049632 | O_anatinus | Animals | mono_intergenic | H/ACA | | | intergenic |
| ENSOANG00000049633 | ENSOANG00000049633 | O_anatinus | Animals | mono_intergenic | H/ACA | | | intergenic |
| ENSOANG00000049638 | ENSOANG00000049638 | O_anatinus | Animals | mono_intergenic | H/ACA | | | intergenic |
| ENSOANG00000049641 | ENSOANG00000049641 | O_anatinus | Animals | mono_intergenic | H/ACA | | | intergenic |
| ENSOANG00000049642 | ENSOANG00000049642 | O_anatinus | Animals | mono_intergenic | H/ACA | | | intergenic |
| ENSOANG00000049645 | ENSOANG00000049645 | O_anatinus | Animals | mono_intergenic | H/ACA | | | intergenic |
| ENSOANG00000049651 | ENSOANG00000049651 | O_anatinus | Animals | mono_intronic | H/ACA | ENSOANG00000020908 | | protein_coding |
| ENSOANG00000049655 | ENSOANG00000049655 | O_anatinus | Animals | mono_intergenic | H/ACA | | | intergenic |
| ENSOANG00000049656 | ENSOANG00000049656 | O_anatinus | Animals | mono_intergenic | H/ACA | | | intergenic |
| ENSOANG00000049657 | ENSOANG00000049657 | O_anatinus | Animals | mono_intergenic | H/ACA | | | intergenic |
| ENSOANG00000049659 | ENSOANG00000049659 | O_anatinus | Animals | mono_intergenic | H/ACA | | | intergenic |
| ENSOANG00000049661 | ENSOANG00000049661 | O_anatinus | Animals | mono_intergenic | H/ACA | | | intergenic |
| ENSOANG00000049662 | ENSOANG00000049662 | O_anatinus | Animals | mono_intergenic | H/ACA | | | intergenic |
| ENSOANG00000049663 | ENSOANG00000049663 | O_anatinus | Animals | mono_intronic | H/ACA | ENSOANG00000000983 | HRH1 | protein_coding |
| ENSOANG00000049665 | ENSOANG00000049665 | O_anatinus | Animals | mono_intergenic | H/ACA | | | intergenic |
| ENSOANG00000049668 | ENSOANG00000049668 | O_anatinus | Animals | mono_intergenic | H/ACA | | | intergenic |
| ENSOANG00000049669 | ENSOANG00000049669 | O_anatinus | Animals | mono_intergenic | H/ACA | | | intergenic |
| ENSOANG00000049672 | ENSOANG00000049672 | O_anatinus | Animals | mono_intronic | H/ACA | ENSOANG00000015755 | PIEZO2 | protein_coding |
| ENSOANG00000049674 | ENSOANG00000049674 | O_anatinus | Animals | mono_intronic | H/ACA | ENSOANG00000006132 | DNAJB6 | protein_coding |
| ENSOANG00000049675 | ENSOANG00000049675 | O_anatinus | Animals | mono_intergenic | H/ACA | | | intergenic |

| | | | | | | | | |
|---|---|---|---|---|---|---|---|---|
| ENSOANG00000049676 | ENSOANG00000049676 | O_anatinus | Animals | mono_intergenic | H/ACA | | | intergenic |
| ENSOANG00000049680 | ENSOANG00000049680 | O_anatinus | Animals | mono_intronic | H/ACA | ENSOANG00000001265 | MPRIP | protein_coding |
| ENSOANG00000049681 | ENSOANG00000049681 | O_anatinus | Animals | mono_intronic | H/ACA | ENSOANG00000005372 | SLC16A9 | protein_coding |
| ENSOANG00000049685 | ENSOANG00000049685 | O_anatinus | Animals | mono_intronic | H/ACA | ENSOANG00000045001 | HTRA1 | protein_coding |
| ENSOANG00000049688 | ENSOANG00000049688 | O_anatinus | Animals | mono_intergenic | H/ACA | | | intergenic |
| ENSOANG00000049697 | ENSOANG00000049697 | O_anatinus | Animals | mono_intergenic | H/ACA | | | intergenic |
| ENSOANG00000049700 | SNORD125 | O_anatinus | Animals | mono_intronic | C/D | ENSOANG00000038796 | AP1B1 | protein_coding |
| ENSOANG00000049703 | ENSOANG00000049703 | O_anatinus | Animals | mono_intergenic | H/ACA | | | intergenic |
| ENSOANG00000049706 | ENSOANG00000049706 | O_anatinus | Animals | mono_intergenic | H/ACA | | | intergenic |
| ENSOANG00000049710 | ENSOANG00000049710 | O_anatinus | Animals | mono_intergenic | H/ACA | | | intergenic |
| ENSOANG00000049715 | ENSOANG00000049715 | O_anatinus | Animals | mono_intergenic | H/ACA | | | intergenic |
| ENSOANG00000049719 | ENSOANG00000049719 | O_anatinus | Animals | mono_intergenic | H/ACA | | | intergenic |
| ENSOANG00000049721 | ENSOANG00000049721 | O_anatinus | Animals | mono_intergenic | H/ACA | | | intergenic |
| ENSOANG00000049723 | ENSOANG00000049723 | O_anatinus | Animals | mono_intergenic | H/ACA | | | intergenic |
| ENSOANG00000049724 | ENSOANG00000049724 | O_anatinus | Animals | mono_intergenic | H/ACA | | | intergenic |
| ENSOANG00000049728 | ENSOANG00000049728 | O_anatinus | Animals | mono_intergenic | H/ACA | | | intergenic |
| ENSOANG00000049729 | ENSOANG00000049729 | O_anatinus | Animals | mono_intergenic | H/ACA | | | intergenic |
| ENSOANG00000049731 | ENSOANG00000049731 | O_anatinus | Animals | mono_intergenic | H/ACA | | | intergenic |
| ENSOANG00000049733 | ENSOANG00000049733 | O_anatinus | Animals | mono_intergenic | H/ACA | | | intergenic |
| ENSOANG00000049737 | ENSOANG00000049737 | O_anatinus | Animals | mono_intergenic | H/ACA | | | intergenic |
| ENSOANG00000049740 | ENSOANG00000049740 | O_anatinus | Animals | mono_intergenic | H/ACA | | | intergenic |
| ENSOANG00000049741 | ENSOANG00000049741 | O_anatinus | Animals | mono_intergenic | H/ACA | | | intergenic |
| ENSOANG00000049744 | ENSOANG00000049744 | O_anatinus | Animals | mono_intergenic | H/ACA | | | intergenic |
| ENSOANG00000049747 | ENSOANG00000049747 | O_anatinus | Animals | mono_intergenic | H/ACA | | | intergenic |
| ENSOANG00000049748 | ENSOANG00000049748 | O_anatinus | Animals | mono_intergenic | H/ACA | | | intergenic |
| ENSOANG00000049749 | ENSOANG00000049749 | O_anatinus | Animals | mono_intergenic | H/ACA | | | intergenic |
| ENSOANG00000049751 | ENSOANG00000049751 | O_anatinus | Animals | mono_intergenic | H/ACA | | | intergenic |
| ENSOANG00000049753 | ENSOANG00000049753 | O_anatinus | Animals | mono_intergenic | H/ACA | | | intergenic |
| ENSOANG00000049755 | ENSOANG00000049755 | O_anatinus | Animals | mono_intergenic | H/ACA | | | intergenic |
| ENSOANG00000049756 | ENSOANG00000049756 | O_anatinus | Animals | mono_intergenic | H/ACA | | | intergenic |
| ENSOANG00000049757 | ENSOANG00000049757 | O_anatinus | Animals | mono_intergenic | H/ACA | | | intergenic |
| ENSOANG00000049761 | ENSOANG00000049761 | O_anatinus | Animals | mono_intergenic | H/ACA | | | intergenic |
| ENSOANG00000049764 | ENSOANG00000049764 | O_anatinus | Animals | mono_intronic | H/ACA | ENSOANG00000039639 | ARHGEF37 | protein_coding |
| ENSOANG00000049768 | ENSOANG00000049768 | O_anatinus | Animals | intergenic_cluster | H/ACA | | | intergenic |
| ENSOANG00000049770 | ENSOANG00000049770 | O_anatinus | Animals | mono_intergenic | H/ACA | | | intergenic |
| ENSOANG00000049771 | ENSOANG00000049771 | O_anatinus | Animals | mono_intronic | H/ACA | ENSOANG00000013846 | ARHGAP22 | protein_coding |
| ENSOANG00000049772 | ENSOANG00000049772 | O_anatinus | Animals | mono_intronic | H/ACA | ENSOANG00000002095 | ASTN2 | protein_coding |
| ENSOANG00000049775 | ENSOANG00000049775 | O_anatinus | Animals | mono_intergenic | H/ACA | | | intergenic |
| ENSOANG00000049778 | ENSOANG00000049778 | O_anatinus | Animals | mono_intergenic | H/ACA | | | intergenic |
| ENSOANG00000049779 | ENSOANG00000049779 | O_anatinus | Animals | mono_intergenic | H/ACA | | | intergenic |
| ENSOANG00000049781 | ENSOANG00000049781 | O_anatinus | Animals | intronic_cluster | H/ACA | ENSOANG00000049221 | SEPTIN10 | protein_coding |

| | | | | | | | | |
|---|---|---|---|---|---|---|---|---|
| ENSOANG00000049782 | ENSOANG00000049782 | O_anatinus | Animals | mono_intergenic | H/ACA | | | intergenic |
| ENSOANG00000049783 | ENSOANG00000049783 | O_anatinus | Animals | mono_intergenic | H/ACA | | | intergenic |
| ENSOANG00000049784 | ENSOANG00000049784 | O_anatinus | Animals | mono_intergenic | H/ACA | | | intergenic |
| ENSOANG00000049785 | ENSOANG00000049785 | O_anatinus | Animals | mono_intronic | H/ACA | ENSOANG00000006700 | TBC1D19 | protein_coding |
| ENSOANG00000049786 | ENSOANG00000049786 | O_anatinus | Animals | mono_intergenic | H/ACA | | | intergenic |
| ENSOANG00000049788 | ENSOANG00000049788 | O_anatinus | Animals | mono_intergenic | H/ACA | | | intergenic |
| ENSOANG00000049793 | ENSOANG00000049793 | O_anatinus | Animals | mono_intergenic | H/ACA | | | intergenic |
| ENSOANG00000049797 | ENSOANG00000049797 | O_anatinus | Animals | mono_intronic | H/ACA | ENSOANG00000013296 | SCAI | protein_coding |
| ENSOANG00000049798 | ENSOANG00000049798 | O_anatinus | Animals | mono_intergenic | H/ACA | | | intergenic |
| ENSOANG00000049799 | ENSOANG00000049799 | O_anatinus | Animals | mono_intergenic | H/ACA | | | intergenic |
| ENSOANG00000049801 | ENSOANG00000049801 | O_anatinus | Animals | intronic_cluster | H/ACA | ENSOANG00000046123 | | non_coding |
| ENSOANG00000049802 | ENSOANG00000049802 | O_anatinus | Animals | mono_intronic | H/ACA | ENSOANG00000013588 | MGAT4B | protein_coding |
| ENSOANG00000049803 | ENSOANG00000049803 | O_anatinus | Animals | mono_intronic | C/D | ENSOANG00000050134 | | non_coding |
| ENSOANG00000049805 | ENSOANG00000049805 | O_anatinus | Animals | mono_intergenic | H/ACA | | | intergenic |
| ENSOANG00000049810 | ENSOANG00000049810 | O_anatinus | Animals | mono_intergenic | H/ACA | | | intergenic |
| ENSOANG00000049811 | ENSOANG00000049811 | O_anatinus | Animals | mono_intergenic | H/ACA | | | intergenic |
| ENSOANG00000049813 | ENSOANG00000049813 | O_anatinus | Animals | mono_intergenic | H/ACA | | | intergenic |
| ENSOANG00000049814 | ENSOANG00000049814 | O_anatinus | Animals | mono_intronic | H/ACA | ENSOANG00000003765 | ITPR1 | protein_coding |
| ENSOANG00000049815 | ENSOANG00000049815 | O_anatinus | Animals | mono_intergenic | H/ACA | | | intergenic |
| ENSOANG00000049817 | ENSOANG00000049817 | O_anatinus | Animals | mono_intergenic | H/ACA | | | intergenic |
| ENSOANG00000049818 | ENSOANG00000049818 | O_anatinus | Animals | mono_intergenic | H/ACA | | | intergenic |
| ENSOANG00000049820 | ENSOANG00000049820 | O_anatinus | Animals | mono_intergenic | H/ACA | | | intergenic |
| ENSOANG00000049822 | ENSOANG00000049822 | O_anatinus | Animals | mono_intergenic | H/ACA | | | intergenic |
| ENSOANG00000049824 | ENSOANG00000049824 | O_anatinus | Animals | mono_intergenic | H/ACA | | | intergenic |
| ENSOANG00000049826 | ENSOANG00000049826 | O_anatinus | Animals | mono_intergenic | H/ACA | | | intergenic |
| ENSOANG00000049827 | ENSOANG00000049827 | O_anatinus | Animals | mono_intergenic | H/ACA | | | intergenic |
| ENSOANG00000049828 | SNORA73 | O_anatinus | Animals | intergenic_cluster | H/ACA | | | intergenic |
| ENSOANG00000049829 | ENSOANG00000049829 | O_anatinus | Animals | mono_intergenic | H/ACA | | | intergenic |
| ENSOANG00000049832 | ENSOANG00000049832 | O_anatinus | Animals | mono_intergenic | H/ACA | | | intergenic |
| ENSOANG00000049838 | ENSOANG00000049838 | O_anatinus | Animals | mono_intergenic | H/ACA | | | intergenic |
| ENSOANG00000049839 | ENSOANG00000049839 | O_anatinus | Animals | mono_intergenic | H/ACA | | | intergenic |
| ENSOANG00000049842 | ENSOANG00000049842 | O_anatinus | Animals | mono_intergenic | H/ACA | | | intergenic |
| ENSOANG00000049845 | ENSOANG00000049845 | O_anatinus | Animals | mono_intergenic | H/ACA | | | intergenic |
| ENSOANG00000049846 | ENSOANG00000049846 | O_anatinus | Animals | mono_intergenic | H/ACA | | | intergenic |
| ENSOANG00000049853 | ENSOANG00000049853 | O_anatinus | Animals | mono_intronic | H/ACA | ENSOANG00000031582 | OCA2 | protein_coding |
| ENSOANG00000049854 | ENSOANG00000049854 | O_anatinus | Animals | mono_intergenic | H/ACA | | | intergenic |
| ENSOANG00000049856 | ENSOANG00000049856 | O_anatinus | Animals | mono_intergenic | H/ACA | | | intergenic |
| ENSOANG00000049865 | ENSOANG00000049865 | O_anatinus | Animals | mono_intronic | H/ACA | ENSOANG00000047282 | | non_coding |
| ENSOANG00000049866 | ENSOANG00000049866 | O_anatinus | Animals | mono_intergenic | H/ACA | | | intergenic |
| ENSOANG00000049868 | ENSOANG00000049868 | O_anatinus | Animals | mono_intergenic | H/ACA | | | intergenic |
| ENSOANG00000049869 | ENSOANG00000049869 | O_anatinus | Animals | mono_intergenic | H/ACA | | | intergenic |

| | | | | | | | | |
|---|---|---|---|---|---|---|---|---|
| ENSOANG00000049873 | ENSOANG00000049873 | O_anatinus | Animals | mono_intergenic | H/ACA | | | intergenic |
| ENSOANG00000049874 | ENSOANG00000049874 | O_anatinus | Animals | mono_intergenic | H/ACA | | | intergenic |
| ENSOANG00000049878 | ENSOANG00000049878 | O_anatinus | Animals | mono_intergenic | H/ACA | | | intergenic |
| ENSOANG00000049882 | ENSOANG00000049882 | O_anatinus | Animals | mono_intergenic | H/ACA | | | intergenic |
| ENSOANG00000049883 | ENSOANG00000049883 | O_anatinus | Animals | mono_intergenic | H/ACA | | | intergenic |
| ENSOANG00000049886 | ENSOANG00000049886 | O_anatinus | Animals | mono_intergenic | H/ACA | | | intergenic |
| ENSOANG00000049887 | ENSOANG00000049887 | O_anatinus | Animals | mono_intergenic | H/ACA | | | intergenic |
| ENSOANG00000049888 | ENSOANG00000049888 | O_anatinus | Animals | mono_intergenic | H/ACA | | | intergenic |
| ENSOANG00000049889 | ENSOANG00000049889 | O_anatinus | Animals | mono_intergenic | H/ACA | | | intergenic |
| ENSOANG00000049890 | ENSOANG00000049890 | O_anatinus | Animals | mono_intergenic | H/ACA | | | intergenic |
| ENSOANG00000049891 | ENSOANG00000049891 | O_anatinus | Animals | mono_intronic | H/ACA | ENSOANG00000041311 | | protein_coding |
| ENSOANG00000049894 | ENSOANG00000049894 | O_anatinus | Animals | mono_intergenic | H/ACA | | | intergenic |
| ENSOANG00000049895 | ENSOANG00000049895 | O_anatinus | Animals | mono_intronic | C/D | ENSOANG00000041245 | CHD8 | protein_coding |
| ENSOANG00000049898 | ENSOANG00000049898 | O_anatinus | Animals | mono_intergenic | H/ACA | | | intergenic |
| ENSOANG00000049901 | ENSOANG00000049901 | O_anatinus | Animals | mono_intergenic | H/ACA | | | intergenic |
| ENSOANG00000049904 | ENSOANG00000049904 | O_anatinus | Animals | mono_intergenic | H/ACA | | | intergenic |
| ENSOANG00000049906 | ENSOANG00000049906 | O_anatinus | Animals | mono_intergenic | H/ACA | | | intergenic |
| ENSOANG00000049911 | ENSOANG00000049911 | O_anatinus | Animals | mono_intergenic | H/ACA | | | intergenic |
| ENSOANG00000049912 | ENSOANG00000049912 | O_anatinus | Animals | mono_intergenic | H/ACA | | | intergenic |
| ENSOANG00000049918 | ENSOANG00000049918 | O_anatinus | Animals | mono_intergenic | H/ACA | | | intergenic |
| ENSOANG00000049919 | ENSOANG00000049919 | O_anatinus | Animals | mono_intergenic | H/ACA | | | intergenic |
| ENSOANG00000049922 | ENSOANG00000049922 | O_anatinus | Animals | mono_intergenic | H/ACA | | | intergenic |
| ENSOANG00000049923 | SNORD54 | O_anatinus | Animals | mono_intronic | C/D | ENSOANG00000005502 | RPS20 | protein_coding |
| ENSOANG00000049925 | ENSOANG00000049925 | O_anatinus | Animals | mono_intergenic | H/ACA | | | intergenic |
| ENSOANG00000049926 | ENSOANG00000049926 | O_anatinus | Animals | mono_intergenic | H/ACA | | | intergenic |
| ENSOANG00000049929 | ENSOANG00000049929 | O_anatinus | Animals | mono_intergenic | H/ACA | | | intergenic |
| ENSOANG00000049936 | ENSOANG00000049936 | O_anatinus | Animals | mono_intergenic | H/ACA | | | intergenic |
| ENSOANG00000049938 | ENSOANG00000049938 | O_anatinus | Animals | mono_intergenic | H/ACA | | | intergenic |
| ENSOANG00000049941 | ENSOANG00000049941 | O_anatinus | Animals | mono_intergenic | H/ACA | | | intergenic |
| ENSOANG00000049945 | ENSOANG00000049945 | O_anatinus | Animals | mono_intronic | H/ACA | ENSOANG00000005353 | GDNF | protein_coding |
| ENSOANG00000049946 | ENSOANG00000049946 | O_anatinus | Animals | mono_intergenic | H/ACA | | | intergenic |
| ENSOANG00000049947 | ENSOANG00000049947 | O_anatinus | Animals | mono_intergenic | H/ACA | | | intergenic |
| ENSOANG00000049952 | ENSOANG00000049952 | O_anatinus | Animals | mono_intergenic | H/ACA | | | intergenic |
| ENSOANG00000049955 | ENSOANG00000049955 | O_anatinus | Animals | mono_intergenic | H/ACA | | | intergenic |
| ENSOANG00000049956 | ENSOANG00000049956 | O_anatinus | Animals | mono_intergenic | H/ACA | | | intergenic |
| ENSOANG00000049957 | ENSOANG00000049957 | O_anatinus | Animals | mono_intergenic | H/ACA | | | intergenic |
| ENSOANG00000049958 | ENSOANG00000049958 | O_anatinus | Animals | mono_intronic | H/ACA | ENSOANG00000012740 | | protein_coding |
| ENSOANG00000049959 | ENSOANG00000049959 | O_anatinus | Animals | mono_intergenic | H/ACA | | | intergenic |
| ENSOANG00000049961 | ENSOANG00000049961 | O_anatinus | Animals | mono_intergenic | H/ACA | | | intergenic |
| ENSOANG00000049962 | ENSOANG00000049962 | O_anatinus | Animals | mono_intergenic | H/ACA | | | intergenic |
| ENSOANG00000049965 | ENSOANG00000049965 | O_anatinus | Animals | mono_intronic | H/ACA | ENSOANG00000028875 | UNC80 | protein_coding |

| | | | | | | | | |
|---|---|---|---|---|---|---|---|---|
| ENSOANG00000049967 | ENSOANG00000049967 | O_anatinus | Animals | mono_intergenic | H/ACA | | | intergenic |
| ENSOANG00000049969 | ENSOANG00000049969 | O_anatinus | Animals | mono_intronic | H/ACA | ENSOANG00000008387 | | protein_coding |
| ENSOANG00000049971 | ENSOANG00000049971 | O_anatinus | Animals | mono_intergenic | H/ACA | | | intergenic |
| ENSOANG00000049972 | SNORD94 | O_anatinus | Animals | mono_intronic | C/D | ENSOANG00000012383 | PTCD3 | protein_coding |
| ENSOANG00000049973 | ENSOANG00000049973 | O_anatinus | Animals | mono_intergenic | H/ACA | | | intergenic |
| ENSOANG00000049978 | ENSOANG00000049978 | O_anatinus | Animals | mono_intergenic | H/ACA | | | intergenic |
| ENSOANG00000049979 | ENSOANG00000049979 | O_anatinus | Animals | mono_intergenic | H/ACA | | | intergenic |
| ENSOANG00000049980 | ENSOANG00000049980 | O_anatinus | Animals | mono_intronic | H/ACA | ENSOANG00000004697 | DGKD | protein_coding |
| ENSOANG00000049981 | ENSOANG00000049981 | O_anatinus | Animals | mono_intergenic | H/ACA | | | intergenic |
| ENSOANG00000049984 | ENSOANG00000049984 | O_anatinus | Animals | mono_intergenic | H/ACA | | | intergenic |
| ENSOANG00000049987 | ENSOANG00000049987 | O_anatinus | Animals | mono_intergenic | H/ACA | | | intergenic |
| ENSOANG00000049989 | ENSOANG00000049989 | O_anatinus | Animals | mono_intergenic | H/ACA | | | intergenic |
| ENSOANG00000049992 | ENSOANG00000049992 | O_anatinus | Animals | mono_intronic | H/ACA | ENSOANG00000047963 | | protein_coding |
| ENSOANG00000049996 | ENSOANG00000049996 | O_anatinus | Animals | mono_intergenic | H/ACA | | | intergenic |
| ENSOANG00000050000 | ENSOANG00000050000 | O_anatinus | Animals | mono_intergenic | H/ACA | | | intergenic |
| ENSOANG00000050004 | ENSOANG00000050004 | O_anatinus | Animals | mono_intergenic | H/ACA | | | intergenic |
| ENSOANG00000050005 | ENSOANG00000050005 | O_anatinus | Animals | mono_intergenic | H/ACA | | | intergenic |
| ENSOANG00000050009 | ENSOANG00000050009 | O_anatinus | Animals | mono_intergenic | H/ACA | | | intergenic |
| ENSOANG00000050011 | ENSOANG00000050011 | O_anatinus | Animals | mono_intergenic | H/ACA | | | intergenic |
| ENSOANG00000050015 | ENSOANG00000050015 | O_anatinus | Animals | mono_intergenic | H/ACA | | | intergenic |
| ENSOANG00000050019 | ENSOANG00000050019 | O_anatinus | Animals | mono_intergenic | H/ACA | | | intergenic |
| ENSOANG00000050021 | ENSOANG00000050021 | O_anatinus | Animals | mono_intergenic | H/ACA | | | intergenic |
| ENSOANG00000050023 | ENSOANG00000050023 | O_anatinus | Animals | mono_intergenic | H/ACA | | | intergenic |
| ENSOANG00000050024 | ENSOANG00000050024 | O_anatinus | Animals | mono_intronic | H/ACA | ENSOANG00000013743 | WDR49 | protein_coding |
| ENSOANG00000050026 | ENSOANG00000050026 | O_anatinus | Animals | mono_intergenic | H/ACA | | | intergenic |
| ENSOANG00000050032 | ENSOANG00000050032 | O_anatinus | Animals | mono_intergenic | H/ACA | | | intergenic |
| ENSOANG00000050033 | ENSOANG00000050033 | O_anatinus | Animals | mono_intergenic | H/ACA | | | intergenic |
| ENSOANG00000050035 | ENSOANG00000050035 | O_anatinus | Animals | mono_intergenic | H/ACA | | | intergenic |
| ENSOANG00000050044 | ENSOANG00000050044 | O_anatinus | Animals | mono_intergenic | H/ACA | | | intergenic |
| ENSOANG00000050048 | ENSOANG00000050048 | O_anatinus | Animals | mono_intergenic | H/ACA | | | intergenic |
| ENSOANG00000050050 | ENSOANG00000050050 | O_anatinus | Animals | mono_intergenic | H/ACA | | | intergenic |
| ENSOANG00000050051 | ENSOANG00000050051 | O_anatinus | Animals | mono_intergenic | H/ACA | | | intergenic |
| ENSOANG00000050055 | ENSOANG00000050055 | O_anatinus | Animals | mono_intergenic | H/ACA | | | intergenic |
| ENSOANG00000050060 | ENSOANG00000050060 | O_anatinus | Animals | mono_intergenic | H/ACA | | | intergenic |
| ENSOANG00000050064 | ENSOANG00000050064 | O_anatinus | Animals | intronic_cluster | C/D | ENSOANG00000008488 | | protein_coding |
| ENSOANG00000050065 | ENSOANG00000050065 | O_anatinus | Animals | intronic_cluster | H/ACA | ENSOANG00000049189 | UGCG | protein_coding |
| ENSOANG00000050068 | ENSOANG00000050068 | O_anatinus | Animals | mono_intergenic | H/ACA | | | intergenic |
| ENSOANG00000050069 | ENSOANG00000050069 | O_anatinus | Animals | mono_intronic | H/ACA | ENSOANG00000047756 | | protein_coding |
| ENSOANG00000050071 | ENSOANG00000050071 | O_anatinus | Animals | mono_intergenic | H/ACA | | | intergenic |
| ENSOANG00000050073 | ENSOANG00000050073 | O_anatinus | Animals | mono_intergenic | H/ACA | | | intergenic |
| ENSOANG00000050075 | ENSOANG00000050075 | O_anatinus | Animals | mono_intergenic | H/ACA | | | intergenic |

| | | | | | | | | |
|---|---|---|---|---|---|---|---|---|
| ENSOANG00000050076 | ENSOANG00000050076 | O_anatinus | Animals | mono_intronic | H/ACA | ENSOANG00000013095 | SGK1 | protein_coding |
| ENSOANG00000050079 | ENSOANG00000050079 | O_anatinus | Animals | mono_intergenic | H/ACA | | | intergenic |
| ENSOANG00000050080 | ENSOANG00000050080 | O_anatinus | Animals | intronic_cluster | C/D | ENSOANG00000000828 | RPL13A | protein_coding |
| ENSOANG00000050081 | ENSOANG00000050081 | O_anatinus | Animals | mono_intronic | H/ACA | ENSOANG00000040181 | OPCML | protein_coding |
| ENSOANG00000050084 | ENSOANG00000050084 | O_anatinus | Animals | mono_intergenic | H/ACA | | | intergenic |
| ENSOANG00000050086 | ENSOANG00000050086 | O_anatinus | Animals | mono_intergenic | H/ACA | | | intergenic |
| ENSOANG00000050087 | ENSOANG00000050087 | O_anatinus | Animals | mono_intergenic | H/ACA | | | intergenic |
| ENSOANG00000050088 | ENSOANG00000050088 | O_anatinus | Animals | mono_intergenic | H/ACA | | | intergenic |
| ENSOANG00000050089 | ENSOANG00000050089 | O_anatinus | Animals | mono_intergenic | H/ACA | | | intergenic |
| ENSOANG00000050091 | ENSOANG00000050091 | O_anatinus | Animals | mono_intergenic | H/ACA | | | intergenic |
| ENSOANG00000050094 | ENSOANG00000050094 | O_anatinus | Animals | mono_intronic | H/ACA | ENSOANG00000007661 | | protein_coding |
| ENSOANG00000050095 | ENSOANG00000050095 | O_anatinus | Animals | mono_intronic | H/ACA | ENSOANG00000013449 | | protein_coding |
| ENSOANG00000050097 | ENSOANG00000050097 | O_anatinus | Animals | mono_intergenic | H/ACA | | | intergenic |
| ENSOANG00000050099 | ENSOANG00000050099 | O_anatinus | Animals | mono_intergenic | H/ACA | | | intergenic |
| ENSOANG00000050104 | ENSOANG00000050104 | O_anatinus | Animals | mono_intronic | H/ACA | ENSOANG00000000584 | ZNF423 | protein_coding |
| ENSOANG00000050106 | ENSOANG00000050106 | O_anatinus | Animals | mono_intergenic | H/ACA | | | intergenic |
| ENSOANG00000050110 | ENSOANG00000050110 | O_anatinus | Animals | mono_intergenic | H/ACA | | | intergenic |
| ENSOANG00000050113 | ENSOANG00000050113 | O_anatinus | Animals | mono_intronic | H/ACA | ENSOANG00000002823 | PLCXD3 | protein_coding |
| ENSOANG00000050115 | ENSOANG00000050115 | O_anatinus | Animals | mono_intergenic | H/ACA | | | intergenic |
| ENSOANG00000050116 | ENSOANG00000050116 | O_anatinus | Animals | mono_intergenic | H/ACA | | | intergenic |
| ENSOANG00000050118 | ENSOANG00000050118 | O_anatinus | Animals | mono_intronic | H/ACA | ENSOANG00000005412 | GPHN | protein_coding |
| ENSOANG00000050121 | ENSOANG00000050121 | O_anatinus | Animals | mono_intergenic | H/ACA | | | intergenic |
| ENSOANG00000050123 | ENSOANG00000050123 | O_anatinus | Animals | mono_intergenic | H/ACA | | | intergenic |
| ENSOANG00000050126 | ENSOANG00000050126 | O_anatinus | Animals | mono_intergenic | H/ACA | | | intergenic |
| ENSOANG00000050132 | ENSOANG00000050132 | O_anatinus | Animals | mono_intergenic | H/ACA | | | intergenic |
| ENSOANG00000050137 | ENSOANG00000050137 | O_anatinus | Animals | mono_intergenic | H/ACA | | | intergenic |
| ENSOANG00000050138 | ENSOANG00000050138 | O_anatinus | Animals | mono_intergenic | H/ACA | | | intergenic |
| ENSOANG00000050141 | ENSOANG00000050141 | O_anatinus | Animals | mono_intergenic | H/ACA | | | intergenic |
| ENSOANG00000050145 | ENSOANG00000050145 | O_anatinus | Animals | mono_intronic | H/ACA | ENSOANG00000015366 | TBRG4 | protein_coding |
| ENSOANG00000050146 | ENSOANG00000050146 | O_anatinus | Animals | mono_intergenic | H/ACA | | | intergenic |
| ENSOANG00000050149 | ENSOANG00000050149 | O_anatinus | Animals | mono_intergenic | H/ACA | | | intergenic |
| ENSOANG00000050153 | ENSOANG00000050153 | O_anatinus | Animals | mono_intergenic | H/ACA | | | intergenic |
| ENSOANG00000050158 | ENSOANG00000050158 | O_anatinus | Animals | mono_intronic | H/ACA | ENSOANG00000010210 | | protein_coding |
| ENSOANG00000050162 | ENSOANG00000050162 | O_anatinus | Animals | mono_intergenic | H/ACA | | | intergenic |
| ENSOANG00000050164 | ENSOANG00000050164 | O_anatinus | Animals | mono_intergenic | H/ACA | | | intergenic |
| ENSOANG00000050165 | ENSOANG00000050165 | O_anatinus | Animals | mono_intergenic | H/ACA | | | intergenic |
| ENSOANG00000050167 | ENSOANG00000050167 | O_anatinus | Animals | mono_intergenic | H/ACA | | | intergenic |
| ENSOANG00000050169 | ENSOANG00000050169 | O_anatinus | Animals | mono_intergenic | H/ACA | | | intergenic |
| ENSOANG00000050172 | ENSOANG00000050172 | O_anatinus | Animals | mono_intergenic | H/ACA | | | intergenic |
| ENSOANG00000050177 | ENSOANG00000050177 | O_anatinus | Animals | mono_intergenic | H/ACA | | | intergenic |
| ENSOANG00000050179 | ENSOANG00000050179 | O_anatinus | Animals | mono_intergenic | H/ACA | | | intergenic |

| | | | | | | | | |
|---|---|---|---|---|---|---|---|---|
| ENSOANG00000050180 | ENSOANG00000050180 | O_anatinus | Animals | mono_intergenic | H/ACA | | | intergenic |
| ENSOANG00000050181 | ENSOANG00000050181 | O_anatinus | Animals | mono_intronic | H/ACA | ENSOANG00000008713 | DKC1 | protein_coding |
| ENSOANG00000050184 | ENSOANG00000050184 | O_anatinus | Animals | mono_intergenic | H/ACA | | | intergenic |
| ENSOANG00000050185 | ENSOANG00000050185 | O_anatinus | Animals | mono_intergenic | H/ACA | | | intergenic |
| ENSOANG00000050187 | ENSOANG00000050187 | O_anatinus | Animals | mono_intergenic | H/ACA | | | intergenic |
| ENSOANG00000050192 | ENSOANG00000050192 | O_anatinus | Animals | mono_intergenic | H/ACA | | | intergenic |
| ENSOANG00000050193 | ENSOANG00000050193 | O_anatinus | Animals | mono_intronic | H/ACA | ENSOANG00000041352 | | non_coding |
| ENSOANG00000050198 | ENSOANG00000050198 | O_anatinus | Animals | mono_intergenic | H/ACA | | | intergenic |
| ENSOANG00000050200 | ENSOANG00000050200 | O_anatinus | Animals | mono_intronic | H/ACA | ENSOANG00000046007 | | protein_coding |
| ENSOANG00000050201 | ENSOANG00000050201 | O_anatinus | Animals | mono_intronic | H/ACA | ENSOANG00000010082 | ANO10 | protein_coding |
| ENSOANG00000050202 | ENSOANG00000050202 | O_anatinus | Animals | mono_intergenic | H/ACA | | | intergenic |
| ENSOANG00000050203 | ENSOANG00000050203 | O_anatinus | Animals | mono_intergenic | H/ACA | | | intergenic |
| ENSOANG00000050204 | ENSOANG00000050204 | O_anatinus | Animals | mono_intergenic | H/ACA | | | intergenic |
| ENSOANG00000050206 | ENSOANG00000050206 | O_anatinus | Animals | mono_intergenic | H/ACA | | | intergenic |
| ENSOANG00000050208 | ENSOANG00000050208 | O_anatinus | Animals | mono_intergenic | H/ACA | | | intergenic |
| ENSOANG00000050210 | ENSOANG00000050210 | O_anatinus | Animals | mono_intergenic | H/ACA | | | intergenic |
| ENSOANG00000050211 | ENSOANG00000050211 | O_anatinus | Animals | mono_intergenic | H/ACA | | | intergenic |
| ENSOANG00000050212 | ENSOANG00000050212 | O_anatinus | Animals | mono_intergenic | H/ACA | | | intergenic |
| ENSOANG00000050216 | ENSOANG00000050216 | O_anatinus | Animals | mono_intergenic | H/ACA | | | intergenic |
| ENSOANG00000050227 | ENSOANG00000050227 | O_anatinus | Animals | mono_intergenic | H/ACA | | | intergenic |
| ENSOANG00000050229 | ENSOANG00000050229 | O_anatinus | Animals | mono_intergenic | H/ACA | | | intergenic |
| ENSOANG00000050233 | ENSOANG00000050233 | O_anatinus | Animals | mono_intergenic | H/ACA | | | intergenic |
| ENSOANG00000050234 | ENSOANG00000050234 | O_anatinus | Animals | mono_intergenic | H/ACA | | | intergenic |
| ENSOANG00000050235 | ENSOANG00000050235 | O_anatinus | Animals | mono_intergenic | H/ACA | | | intergenic |
| ENSOANG00000050241 | ENSOANG00000050241 | O_anatinus | Animals | mono_intergenic | H/ACA | | | intergenic |
| ENSOANG00000050244 | ENSOANG00000050244 | O_anatinus | Animals | mono_intergenic | H/ACA | | | intergenic |
| ENSOANG00000050245 | ENSOANG00000050245 | O_anatinus | Animals | mono_intergenic | H/ACA | | | intergenic |
| ENSOANG00000050249 | ENSOANG00000050249 | O_anatinus | Animals | mono_intergenic | H/ACA | | | intergenic |
| ENSOANG00000050253 | ENSOANG00000050253 | O_anatinus | Animals | mono_intergenic | H/ACA | | | intergenic |
| ENSOANG00000050254 | ENSOANG00000050254 | O_anatinus | Animals | mono_intergenic | H/ACA | | | intergenic |
| ENSOANG00000050255 | ENSOANG00000050255 | O_anatinus | Animals | mono_intronic | C/D | ENSOANG00000001930 | TRRAP | protein_coding |
| ENSOANG00000050256 | ENSOANG00000050256 | O_anatinus | Animals | mono_intergenic | H/ACA | | | intergenic |
| ENSOANG00000050259 | ENSOANG00000050259 | O_anatinus | Animals | mono_intergenic | H/ACA | | | intergenic |
| ENSOANG00000050260 | ENSOANG00000050260 | O_anatinus | Animals | mono_intergenic | H/ACA | | | intergenic |
| ENSOANG00000050261 | ENSOANG00000050261 | O_anatinus | Animals | mono_intergenic | H/ACA | | | intergenic |
| ENSOANG00000050265 | ENSOANG00000050265 | O_anatinus | Animals | mono_intergenic | H/ACA | | | intergenic |
| ENSOANG00000050267 | ENSOANG00000050267 | O_anatinus | Animals | mono_intergenic | H/ACA | | | intergenic |
| ENSOANG00000050268 | ENSOANG00000050268 | O_anatinus | Animals | mono_intergenic | H/ACA | | | intergenic |
| ENSOANG00000050269 | ENSOANG00000050269 | O_anatinus | Animals | mono_intergenic | H/ACA | | | intergenic |
| ENSOANG00000050271 | ENSOANG00000050271 | O_anatinus | Animals | mono_intergenic | H/ACA | | | intergenic |
| ENSOANG00000050282 | ENSOANG00000050282 | O_anatinus | Animals | mono_intergenic | H/ACA | | | intergenic |

| | | | | | | | | |
|---|---|---|---|---|---|---|---|---|
| ENSOANG00000050283 | ENSOANG00000050283 | O_anatinus | Animals | mono_intergenic | H/ACA | | | intergenic |
| ENSOANG00000050285 | ENSOANG00000050285 | O_anatinus | Animals | mono_intergenic | H/ACA | | | intergenic |
| ENSOANG00000050287 | ENSOANG00000050287 | O_anatinus | Animals | mono_intronic | H/ACA | ENSOANG00000039659 | WBP4 | protein_coding |
| ENSOANG00000050289 | ENSOANG00000050289 | O_anatinus | Animals | mono_intronic | H/ACA | ENSOANG00000014323 | PHKA1 | protein_coding |
| ENSOANG00000050291 | ENSOANG00000050291 | O_anatinus | Animals | mono_intergenic | H/ACA | | | intergenic |
| ENSOANG00000050292 | ENSOANG00000050292 | O_anatinus | Animals | mono_intergenic | H/ACA | | | intergenic |
| ENSOANG00000050293 | ENSOANG00000050293 | O_anatinus | Animals | mono_intronic | H/ACA | ENSOANG00000012175 | CNTN6 | protein_coding |
| ENSOANG00000050294 | ENSOANG00000050294 | O_anatinus | Animals | mono_intronic | H/ACA | ENSOANG00000002187 | | protein_coding |
| ENSOANG00000050295 | ENSOANG00000050295 | O_anatinus | Animals | mono_intergenic | H/ACA | | | intergenic |
| ENSOANG00000050296 | ENSOANG00000050296 | O_anatinus | Animals | mono_intergenic | H/ACA | | | intergenic |
| ENSOANG00000050300 | ENSOANG00000050300 | O_anatinus | Animals | mono_intergenic | H/ACA | | | intergenic |
| ENSOANG00000050301 | ENSOANG00000050301 | O_anatinus | Animals | mono_intergenic | H/ACA | | | intergenic |
| ENSOANG00000050302 | ENSOANG00000050302 | O_anatinus | Animals | mono_intergenic | H/ACA | | | intergenic |
| ENSOANG00000050310 | ENSOANG00000050310 | O_anatinus | Animals | mono_intergenic | H/ACA | | | intergenic |
| ENSOANG00000050311 | ENSOANG00000050311 | O_anatinus | Animals | mono_intergenic | H/ACA | | | intergenic |
| ENSOANG00000050314 | ENSOANG00000050314 | O_anatinus | Animals | mono_intergenic | H/ACA | | | intergenic |
| ENSOANG00000050315 | ENSOANG00000050315 | O_anatinus | Animals | mono_intergenic | H/ACA | | | intergenic |
| ENSOANG00000050318 | ENSOANG00000050318 | O_anatinus | Animals | mono_intronic | H/ACA | ENSOANG00000010703 | DCDC1 | protein_coding |
| ENSOANG00000050319 | ENSOANG00000050319 | O_anatinus | Animals | mono_intronic | H/ACA | ENSOANG00000040769 | | protein_coding |
| ENSOANG00000050323 | ENSOANG00000050323 | O_anatinus | Animals | mono_intergenic | H/ACA | | | intergenic |
| ENSOANG00000050332 | ENSOANG00000050332 | O_anatinus | Animals | mono_intergenic | H/ACA | | | intergenic |
| ENSOANG00000050333 | ENSOANG00000050333 | O_anatinus | Animals | mono_intergenic | H/ACA | | | intergenic |
| ENSOANG00000050336 | ENSOANG00000050336 | O_anatinus | Animals | mono_intronic | H/ACA | ENSOANG00000010799 | SPIRE1 | protein_coding |
| ENSOANG00000050337 | ENSOANG00000050337 | O_anatinus | Animals | mono_intergenic | H/ACA | | | intergenic |
| ENSOANG00000050339 | ENSOANG00000050339 | O_anatinus | Animals | mono_intergenic | H/ACA | | | intergenic |
| ENSOANG00000050345 | ENSOANG00000050345 | O_anatinus | Animals | mono_intergenic | H/ACA | | | intergenic |
| ENSOANG00000050346 | ENSOANG00000050346 | O_anatinus | Animals | mono_intergenic | H/ACA | | | intergenic |
| ENSOANG00000050349 | ENSOANG00000050349 | O_anatinus | Animals | mono_intronic | H/ACA | ENSOANG00000022095 | | protein_coding |
| ENSOANG00000050354 | ENSOANG00000050354 | O_anatinus | Animals | mono_intergenic | H/ACA | | | intergenic |
| ENSOANG00000050355 | ENSOANG00000050355 | O_anatinus | Animals | mono_intergenic | H/ACA | | | intergenic |
| ENSOANG00000050357 | ENSOANG00000050357 | O_anatinus | Animals | mono_intergenic | H/ACA | | | intergenic |
| ENSOANG00000050359 | ENSOANG00000050359 | O_anatinus | Animals | mono_intergenic | H/ACA | | | intergenic |
| ENSOANG00000050361 | ENSOANG00000050361 | O_anatinus | Animals | mono_intronic | H/ACA | ENSOANG00000005026 | MAMDC2 | protein_coding |
| ENSOANG00000050362 | ENSOANG00000050362 | O_anatinus | Animals | mono_intergenic | H/ACA | | | intergenic |
| ENSOANG00000050365 | ENSOANG00000050365 | O_anatinus | Animals | mono_intergenic | H/ACA | | | intergenic |
| ENSOANG00000050367 | ENSOANG00000050367 | O_anatinus | Animals | mono_intergenic | H/ACA | | | intergenic |
| ENSOANG00000050369 | ENSOANG00000050369 | O_anatinus | Animals | intronic_cluster | H/ACA | ENSOANG00000010900 | GKAP1 | protein_coding |
| ENSOANG00000050374 | ENSOANG00000050374 | O_anatinus | Animals | mono_intronic | H/ACA | ENSOANG00000015746 | CDH6 | protein_coding |
| ENSOANG00000050376 | ENSOANG00000050376 | O_anatinus | Animals | mono_intergenic | H/ACA | | | intergenic |
| ENSOANG00000050378 | ENSOANG00000050378 | O_anatinus | Animals | mono_intronic | H/ACA | ENSOANG00000047654 | | non_coding |
| ENSOANG00000050381 | ENSOANG00000050381 | O_anatinus | Animals | mono_intergenic | H/ACA | | | intergenic |

| | | | | | | | | |
|---|---|---|---|---|---|---|---|---|
| ENSOANG00000050382 | ENSOANG00000050382 | O_anatinus | Animals | mono_intergenic | H/ACA | | | intergenic |
| ENSOANG00000050385 | ENSOANG00000050385 | O_anatinus | Animals | mono_intergenic | H/ACA | | | intergenic |
| ENSOANG00000050387 | ENSOANG00000050387 | O_anatinus | Animals | mono_intergenic | H/ACA | | | intergenic |
| ENSOANG00000050392 | ENSOANG00000050392 | O_anatinus | Animals | mono_intergenic | H/ACA | | | intergenic |
| ENSOANG00000050394 | ENSOANG00000050394 | O_anatinus | Animals | mono_intergenic | H/ACA | | | intergenic |
| ENSOANG00000050396 | ENSOANG00000050396 | O_anatinus | Animals | mono_intergenic | H/ACA | | | intergenic |
| ENSOANG00000050399 | ENSOANG00000050399 | O_anatinus | Animals | mono_intergenic | H/ACA | | | intergenic |
| ENSOANG00000050409 | ENSOANG00000050409 | O_anatinus | Animals | mono_intergenic | H/ACA | | | intergenic |
| ENSOANG00000050410 | ENSOANG00000050410 | O_anatinus | Animals | mono_intergenic | H/ACA | | | intergenic |
| ENSOANG00000050412 | ENSOANG00000050412 | O_anatinus | Animals | mono_intergenic | H/ACA | | | intergenic |
| ENSOANG00000050413 | ENSOANG00000050413 | O_anatinus | Animals | mono_intergenic | H/ACA | | | intergenic |
| ENSOANG00000050414 | ENSOANG00000050414 | O_anatinus | Animals | mono_intergenic | H/ACA | | | intergenic |
| ENSOANG00000050416 | ENSOANG00000050416 | O_anatinus | Animals | mono_intergenic | H/ACA | | | intergenic |
| ENSOANG00000050417 | ENSOANG00000050417 | O_anatinus | Animals | mono_intergenic | H/ACA | | | intergenic |
| ENSOANG00000050418 | ENSOANG00000050418 | O_anatinus | Animals | mono_intergenic | H/ACA | | | intergenic |
| ENSOANG00000050419 | ENSOANG00000050419 | O_anatinus | Animals | mono_intronic | H/ACA | ENSOANG00000051201 | RAB27B | protein_coding |
| ENSOANG00000050421 | ENSOANG00000050421 | O_anatinus | Animals | mono_intergenic | H/ACA | | | intergenic |
| ENSOANG00000050424 | ENSOANG00000050424 | O_anatinus | Animals | mono_intergenic | H/ACA | | | intergenic |
| ENSOANG00000050425 | ENSOANG00000050425 | O_anatinus | Animals | mono_intergenic | H/ACA | | | intergenic |
| ENSOANG00000050429 | ENSOANG00000050429 | O_anatinus | Animals | mono_intergenic | H/ACA | | | intergenic |
| ENSOANG00000050434 | ENSOANG00000050434 | O_anatinus | Animals | mono_intergenic | H/ACA | | | intergenic |
| ENSOANG00000050435 | ENSOANG00000050435 | O_anatinus | Animals | mono_intronic | H/ACA | ENSOANG00000050502 | HMGCLL1 | protein_coding |
| ENSOANG00000050436 | ENSOANG00000050436 | O_anatinus | Animals | mono_intronic | H/ACA | ENSOANG00000044928 | RNF13 | protein_coding |
| ENSOANG00000050437 | ENSOANG00000050437 | O_anatinus | Animals | mono_intergenic | H/ACA | | | intergenic |
| ENSOANG00000050438 | ENSOANG00000050438 | O_anatinus | Animals | mono_intronic | H/ACA | ENSOANG00000020240 | FGD3 | protein_coding |
| ENSOANG00000050442 | ENSOANG00000050442 | O_anatinus | Animals | mono_intergenic | H/ACA | | | intergenic |
| ENSOANG00000050444 | ENSOANG00000050444 | O_anatinus | Animals | mono_intergenic | H/ACA | | | intergenic |
| ENSOANG00000050445 | ENSOANG00000050445 | O_anatinus | Animals | mono_intergenic | H/ACA | | | intergenic |
| ENSOANG00000050446 | ENSOANG00000050446 | O_anatinus | Animals | mono_intergenic | H/ACA | | | intergenic |
| ENSOANG00000050450 | ENSOANG00000050450 | O_anatinus | Animals | mono_intergenic | H/ACA | | | intergenic |
| ENSOANG00000050452 | ENSOANG00000050452 | O_anatinus | Animals | mono_intergenic | H/ACA | | | intergenic |
| ENSOANG00000050453 | ENSOANG00000050453 | O_anatinus | Animals | mono_intergenic | H/ACA | | | intergenic |
| ENSOANG00000050457 | ENSOANG00000050457 | O_anatinus | Animals | mono_intergenic | H/ACA | | | intergenic |
| ENSOANG00000050458 | ENSOANG00000050458 | O_anatinus | Animals | mono_intergenic | H/ACA | | | intergenic |
| ENSOANG00000050461 | ENSOANG00000050461 | O_anatinus | Animals | mono_intergenic | H/ACA | | | intergenic |
| ENSOANG00000050462 | ENSOANG00000050462 | O_anatinus | Animals | mono_intergenic | H/ACA | | | intergenic |
| ENSOANG00000050470 | ENSOANG00000050470 | O_anatinus | Animals | mono_intergenic | H/ACA | | | intergenic |
| ENSOANG00000050473 | ENSOANG00000050473 | O_anatinus | Animals | mono_intergenic | H/ACA | | | intergenic |
| ENSOANG00000050480 | ENSOANG00000050480 | O_anatinus | Animals | mono_intronic | C/D | ENSOANG00000050134 | | non_coding |
| ENSOANG00000050488 | ENSOANG00000050488 | O_anatinus | Animals | mono_intergenic | H/ACA | | | intergenic |
| ENSOANG00000050490 | ENSOANG00000050490 | O_anatinus | Animals | mono_intronic | H/ACA | ENSOANG00000000861 | | protein_coding |

| | | | | | | | | |
|---|---|---|---|---|---|---|---|---|
| ENSOANG00000050491 | ENSOANG00000050491 | O_anatinus | Animals | mono_intergenic | H/ACA | | | intergenic |
| ENSOANG00000050492 | ENSOANG00000050492 | O_anatinus | Animals | mono_intergenic | H/ACA | | | intergenic |
| ENSOANG00000050493 | ENSOANG00000050493 | O_anatinus | Animals | mono_intronic | H/ACA | ENSOANG00000009950 | TMEM132D | protein_coding |
| ENSOANG00000050497 | ENSOANG00000050497 | O_anatinus | Animals | mono_intergenic | H/ACA | | | intergenic |
| ENSOANG00000050499 | ENSOANG00000050499 | O_anatinus | Animals | mono_intergenic | H/ACA | | | intergenic |
| ENSOANG00000050508 | ENSOANG00000050508 | O_anatinus | Animals | mono_intergenic | H/ACA | | | intergenic |
| ENSOANG00000050515 | ENSOANG00000050515 | O_anatinus | Animals | mono_intergenic | H/ACA | | | intergenic |
| ENSOANG00000050520 | ENSOANG00000050520 | O_anatinus | Animals | mono_intergenic | H/ACA | | | intergenic |
| ENSOANG00000050521 | ENSOANG00000050521 | O_anatinus | Animals | mono_intergenic | H/ACA | | | intergenic |
| ENSOANG00000050523 | ENSOANG00000050523 | O_anatinus | Animals | mono_intergenic | H/ACA | | | intergenic |
| ENSOANG00000050526 | ENSOANG00000050526 | O_anatinus | Animals | mono_intergenic | H/ACA | | | intergenic |
| ENSOANG00000050530 | ENSOANG00000050530 | O_anatinus | Animals | mono_intergenic | H/ACA | | | intergenic |
| ENSOANG00000050532 | ENSOANG00000050532 | O_anatinus | Animals | mono_intergenic | H/ACA | | | intergenic |
| ENSOANG00000050541 | ENSOANG00000050541 | O_anatinus | Animals | mono_intergenic | H/ACA | | | intergenic |
| ENSOANG00000050543 | ENSOANG00000050543 | O_anatinus | Animals | mono_intergenic | H/ACA | | | intergenic |
| ENSOANG00000050550 | ENSOANG00000050550 | O_anatinus | Animals | mono_intergenic | H/ACA | | | intergenic |
| ENSOANG00000050551 | ENSOANG00000050551 | O_anatinus | Animals | mono_intergenic | H/ACA | | | intergenic |
| ENSOANG00000050552 | ENSOANG00000050552 | O_anatinus | Animals | mono_intergenic | H/ACA | | | intergenic |
| ENSOANG00000050554 | ENSOANG00000050554 | O_anatinus | Animals | mono_intergenic | H/ACA | | | intergenic |
| ENSOANG00000050555 | ENSOANG00000050555 | O_anatinus | Animals | mono_intergenic | H/ACA | | | intergenic |
| ENSOANG00000050557 | ENSOANG00000050557 | O_anatinus | Animals | mono_intronic | H/ACA | ENSOANG00000015049 | | protein_coding |
| ENSOANG00000050566 | ENSOANG00000050566 | O_anatinus | Animals | mono_intronic | H/ACA | ENSOANG00000016001 | HTR7 | protein_coding |
| ENSOANG00000050567 | ENSOANG00000050567 | O_anatinus | Animals | mono_intergenic | H/ACA | | | intergenic |
| ENSOANG00000050571 | ENSOANG00000050571 | O_anatinus | Animals | mono_intronic | H/ACA | ENSOANG00000014928 | | protein_coding |
| ENSOANG00000050574 | ENSOANG00000050574 | O_anatinus | Animals | mono_intergenic | H/ACA | | | intergenic |
| ENSOANG00000050578 | ENSOANG00000050578 | O_anatinus | Animals | mono_intronic | H/ACA | ENSOANG00000043457 | TVP23A | protein_coding |
| ENSOANG00000050580 | ENSOANG00000050580 | O_anatinus | Animals | mono_intergenic | H/ACA | | | intergenic |
| ENSOANG00000050583 | ENSOANG00000050583 | O_anatinus | Animals | mono_intergenic | H/ACA | | | intergenic |
| ENSOANG00000050584 | ENSOANG00000050584 | O_anatinus | Animals | mono_intergenic | H/ACA | | | intergenic |
| ENSOANG00000050586 | ENSOANG00000050586 | O_anatinus | Animals | mono_intergenic | H/ACA | | | intergenic |
| ENSOANG00000050587 | ENSOANG00000050587 | O_anatinus | Animals | mono_intergenic | H/ACA | | | intergenic |
| ENSOANG00000050590 | ENSOANG00000050590 | O_anatinus | Animals | mono_intergenic | H/ACA | | | intergenic |
| ENSOANG00000050592 | ENSOANG00000050592 | O_anatinus | Animals | mono_intergenic | H/ACA | | | intergenic |
| ENSOANG00000050593 | ENSOANG00000050593 | O_anatinus | Animals | mono_intergenic | H/ACA | | | intergenic |
| ENSOANG00000050598 | ENSOANG00000050598 | O_anatinus | Animals | mono_intronic | H/ACA | ENSOANG00000010395 | | protein_coding |
| ENSOANG00000050603 | ENSOANG00000050603 | O_anatinus | Animals | mono_intergenic | H/ACA | | | intergenic |
| ENSOANG00000050604 | ENSOANG00000050604 | O_anatinus | Animals | mono_intergenic | H/ACA | | | intergenic |
| ENSOANG00000050609 | ENSOANG00000050609 | O_anatinus | Animals | mono_intergenic | H/ACA | | | intergenic |
| ENSOANG00000050610 | ENSOANG00000050610 | O_anatinus | Animals | mono_intergenic | H/ACA | | | intergenic |
| ENSOANG00000050611 | ENSOANG00000050611 | O_anatinus | Animals | mono_intergenic | H/ACA | | | intergenic |
| ENSOANG00000050612 | ENSOANG00000050612 | O_anatinus | Animals | mono_intergenic | H/ACA | | | intergenic |

| | | | | | | | | |
|---|---|---|---|---|---|---|---|---|
| ENSOANG00000050613 | ENSOANG00000050613 | O_anatinus | Animals | mono_intergenic | H/ACA | | | intergenic |
| ENSOANG00000050617 | ENSOANG00000050617 | O_anatinus | Animals | intronic_cluster | H/ACA | ENSOANG00000045478 | LRMDA | protein_coding |
| ENSOANG00000050618 | ENSOANG00000050618 | O_anatinus | Animals | intronic_cluster | H/ACA | ENSOANG00000047093 | RAB6B | protein_coding |
| ENSOANG00000050619 | ENSOANG00000050619 | O_anatinus | Animals | mono_intergenic | H/ACA | | | intergenic |
| ENSOANG00000050620 | ENSOANG00000050620 | O_anatinus | Animals | mono_intergenic | H/ACA | | | intergenic |
| ENSOANG00000050621 | ENSOANG00000050621 | O_anatinus | Animals | mono_intergenic | H/ACA | | | intergenic |
| ENSOANG00000050624 | ENSOANG00000050624 | O_anatinus | Animals | mono_intergenic | H/ACA | | | intergenic |
| ENSOANG00000050625 | ENSOANG00000050625 | O_anatinus | Animals | mono_intergenic | H/ACA | | | intergenic |
| ENSOANG00000050626 | ENSOANG00000050626 | O_anatinus | Animals | mono_intergenic | H/ACA | | | intergenic |
| ENSOANG00000050629 | ENSOANG00000050629 | O_anatinus | Animals | mono_intergenic | H/ACA | | | intergenic |
| ENSOANG00000050630 | ENSOANG00000050630 | O_anatinus | Animals | mono_intergenic | H/ACA | | | intergenic |
| ENSOANG00000050631 | ENSOANG00000050631 | O_anatinus | Animals | mono_intergenic | H/ACA | | | intergenic |
| ENSOANG00000050634 | ENSOANG00000050634 | O_anatinus | Animals | mono_intergenic | H/ACA | | | intergenic |
| ENSOANG00000050642 | ENSOANG00000050642 | O_anatinus | Animals | mono_intronic | C/D | ENSOANG00000014467 | WDR43 | protein_coding |
| ENSOANG00000050643 | ENSOANG00000050643 | O_anatinus | Animals | intronic_cluster | H/ACA | ENSOANG00000001903 | PCDH15 | protein_coding |
| ENSOANG00000050651 | ENSOANG00000050651 | O_anatinus | Animals | mono_intergenic | H/ACA | | | intergenic |
| ENSOANG00000050652 | ENSOANG00000050652 | O_anatinus | Animals | mono_intergenic | H/ACA | | | intergenic |
| ENSOANG00000050654 | ENSOANG00000050654 | O_anatinus | Animals | mono_intergenic | H/ACA | | | intergenic |
| ENSOANG00000050655 | ENSOANG00000050655 | O_anatinus | Animals | mono_intergenic | H/ACA | | | intergenic |
| ENSOANG00000050656 | ENSOANG00000050656 | O_anatinus | Animals | mono_intronic | H/ACA | ENSOANG00000003214 | | protein_coding |
| ENSOANG00000050658 | ENSOANG00000050658 | O_anatinus | Animals | intronic_cluster | H/ACA | ENSOANG00000040015 | KCNMB2 | protein_coding |
| ENSOANG00000050660 | ENSOANG00000050660 | O_anatinus | Animals | mono_intergenic | H/ACA | | | intergenic |
| ENSOANG00000050661 | ENSOANG00000050661 | O_anatinus | Animals | mono_intergenic | H/ACA | | | intergenic |
| ENSOANG00000050665 | ENSOANG00000050665 | O_anatinus | Animals | intergenic_cluster | H/ACA | | | intergenic |
| ENSOANG00000050670 | ENSOANG00000050670 | O_anatinus | Animals | mono_intergenic | H/ACA | | | intergenic |
| ENSOANG00000050673 | ENSOANG00000050673 | O_anatinus | Animals | mono_intergenic | H/ACA | | | intergenic |
| ENSOANG00000050674 | ENSOANG00000050674 | O_anatinus | Animals | mono_intergenic | H/ACA | | | intergenic |
| ENSOANG00000050675 | ENSOANG00000050675 | O_anatinus | Animals | mono_intergenic | H/ACA | | | intergenic |
| ENSOANG00000050679 | ENSOANG00000050679 | O_anatinus | Animals | mono_intergenic | H/ACA | | | intergenic |
| ENSOANG00000050680 | ENSOANG00000050680 | O_anatinus | Animals | mono_intronic | H/ACA | ENSOANG00000012776 | KLHDC8A | protein_coding |
| ENSOANG00000050682 | ENSOANG00000050682 | O_anatinus | Animals | mono_intronic | H/ACA | ENSOANG00000046973 | | protein_coding |
| ENSOANG00000050685 | ENSOANG00000050685 | O_anatinus | Animals | mono_intergenic | H/ACA | | | intergenic |
| ENSOANG00000050688 | ENSOANG00000050688 | O_anatinus | Animals | mono_intergenic | H/ACA | | | intergenic |
| ENSOANG00000050690 | ENSOANG00000050690 | O_anatinus | Animals | mono_intronic | H/ACA | ENSOANG00000011283 | | protein_coding |
| ENSOANG00000050692 | ENSOANG00000050692 | O_anatinus | Animals | mono_exonic | H/ACA | ENSOANG00000015144 | PRRG4 | protein_coding |
| ENSOANG00000050693 | ENSOANG00000050693 | O_anatinus | Animals | mono_intergenic | H/ACA | | | intergenic |
| ENSOANG00000050694 | ENSOANG00000050694 | O_anatinus | Animals | mono_intergenic | H/ACA | | | intergenic |
| ENSOANG00000050704 | ENSOANG00000050704 | O_anatinus | Animals | mono_intergenic | H/ACA | | | intergenic |
| ENSOANG00000050708 | ENSOANG00000050708 | O_anatinus | Animals | mono_intergenic | H/ACA | | | intergenic |
| ENSOANG00000050712 | ENSOANG00000050712 | O_anatinus | Animals | mono_intergenic | H/ACA | | | intergenic |
| ENSOANG00000050715 | ENSOANG00000050715 | O_anatinus | Animals | mono_intergenic | H/ACA | | | intergenic |

| | | | | | | | | |
|---|---|---|---|---|---|---|---|---|
| ENSOANG00000050722 | ENSOANG00000050722 | O_anatinus | Animals | mono_intergenic | H/ACA | | | intergenic |
| ENSOANG00000050726 | ENSOANG00000050726 | O_anatinus | Animals | mono_intronic | H/ACA | ENSOANG00000051233 | ARNT2 | protein_coding |
| ENSOANG00000050727 | ENSOANG00000050727 | O_anatinus | Animals | mono_intergenic | H/ACA | | | intergenic |
| ENSOANG00000050728 | ENSOANG00000050728 | O_anatinus | Animals | mono_intergenic | H/ACA | | | intergenic |
| ENSOANG00000050729 | ENSOANG00000050729 | O_anatinus | Animals | mono_intergenic | H/ACA | | | intergenic |
| ENSOANG00000050735 | ENSOANG00000050735 | O_anatinus | Animals | mono_intronic | H/ACA | ENSOANG00000005963 | PTPRR | protein_coding |
| ENSOANG00000050737 | ENSOANG00000050737 | O_anatinus | Animals | mono_intergenic | H/ACA | | | intergenic |
| ENSOANG00000050739 | ENSOANG00000050739 | O_anatinus | Animals | mono_intergenic | H/ACA | | | intergenic |
| ENSOANG00000050742 | ENSOANG00000050742 | O_anatinus | Animals | mono_intergenic | H/ACA | | | intergenic |
| ENSOANG00000050744 | ENSOANG00000050744 | O_anatinus | Animals | mono_intergenic | H/ACA | | | intergenic |
| ENSOANG00000050745 | ENSOANG00000050745 | O_anatinus | Animals | mono_intergenic | H/ACA | | | intergenic |
| ENSOANG00000050747 | ENSOANG00000050747 | O_anatinus | Animals | mono_intergenic | H/ACA | | | intergenic |
| ENSOANG00000050754 | ENSOANG00000050754 | O_anatinus | Animals | mono_intergenic | H/ACA | | | intergenic |
| ENSOANG00000050757 | ENSOANG00000050757 | O_anatinus | Animals | mono_intergenic | H/ACA | | | intergenic |
| ENSOANG00000050762 | ENSOANG00000050762 | O_anatinus | Animals | mono_intergenic | H/ACA | | | intergenic |
| ENSOANG00000050765 | ENSOANG00000050765 | O_anatinus | Animals | mono_intergenic | H/ACA | | | intergenic |
| ENSOANG00000050766 | ENSOANG00000050766 | O_anatinus | Animals | mono_intergenic | H/ACA | | | intergenic |
| ENSOANG00000050769 | ENSOANG00000050769 | O_anatinus | Animals | intronic_cluster | H/ACA | ENSOANG00000002334 | LTBP1 | protein_coding |
| ENSOANG00000050770 | ENSOANG00000050770 | O_anatinus | Animals | mono_intergenic | H/ACA | | | intergenic |
| ENSOANG00000050771 | ENSOANG00000050771 | O_anatinus | Animals | mono_intronic | H/ACA | ENSOANG00000034291 | | non_coding |
| ENSOANG00000050780 | ENSOANG00000050780 | O_anatinus | Animals | mono_intergenic | H/ACA | | | intergenic |
| ENSOANG00000050781 | ENSOANG00000050781 | O_anatinus | Animals | mono_intergenic | H/ACA | | | intergenic |
| ENSOANG00000050782 | ENSOANG00000050782 | O_anatinus | Animals | mono_intergenic | H/ACA | | | intergenic |
| ENSOANG00000050783 | ENSOANG00000050783 | O_anatinus | Animals | mono_intergenic | H/ACA | | | intergenic |
| ENSOANG00000050787 | ENSOANG00000050787 | O_anatinus | Animals | mono_intergenic | H/ACA | | | intergenic |
| ENSOANG00000050789 | ENSOANG00000050789 | O_anatinus | Animals | mono_intergenic | H/ACA | | | intergenic |
| ENSOANG00000050795 | ENSOANG00000050795 | O_anatinus | Animals | mono_intergenic | H/ACA | | | intergenic |
| ENSOANG00000050799 | ENSOANG00000050799 | O_anatinus | Animals | mono_intergenic | H/ACA | | | intergenic |
| ENSOANG00000050802 | ENSOANG00000050802 | O_anatinus | Animals | mono_intergenic | H/ACA | | | intergenic |
| ENSOANG00000050803 | ENSOANG00000050803 | O_anatinus | Animals | mono_intergenic | H/ACA | | | intergenic |
| ENSOANG00000050804 | ENSOANG00000050804 | O_anatinus | Animals | mono_intergenic | H/ACA | | | intergenic |
| ENSOANG00000050808 | ENSOANG00000050808 | O_anatinus | Animals | mono_intergenic | H/ACA | | | intergenic |
| ENSOANG00000050809 | ENSOANG00000050809 | O_anatinus | Animals | mono_intergenic | H/ACA | | | intergenic |
| ENSOANG00000050810 | ENSOANG00000050810 | O_anatinus | Animals | mono_intergenic | H/ACA | | | intergenic |
| ENSOANG00000050815 | ENSOANG00000050815 | O_anatinus | Animals | mono_intergenic | H/ACA | | | intergenic |
| ENSOANG00000050816 | ENSOANG00000050816 | O_anatinus | Animals | mono_intergenic | H/ACA | | | intergenic |
| ENSOANG00000050820 | ENSOANG00000050820 | O_anatinus | Animals | mono_intergenic | H/ACA | | | intergenic |
| ENSOANG00000050823 | ENSOANG00000050823 | O_anatinus | Animals | mono_intergenic | H/ACA | | | intergenic |
| ENSOANG00000050826 | ENSOANG00000050826 | O_anatinus | Animals | mono_intergenic | H/ACA | | | intergenic |
| ENSOANG00000050829 | ENSOANG00000050829 | O_anatinus | Animals | mono_intergenic | H/ACA | | | intergenic |
| ENSOANG00000050831 | ENSOANG00000050831 | O_anatinus | Animals | mono_intergenic | H/ACA | | | intergenic |

| | | | | | | | | |
|---|---|---|---|---|---|---|---|---|
| ENSOANG00000050834 | ENSOANG00000050834 | O_anatinus | Animals | mono_intergenic | H/ACA | | | intergenic |
| ENSOANG00000050841 | ENSOANG00000050841 | O_anatinus | Animals | mono_intronic | H/ACA | ENSOANG00000008595 | MAP3K20 | protein_coding |
| ENSOANG00000050844 | ENSOANG00000050844 | O_anatinus | Animals | mono_intergenic | H/ACA | | | intergenic |
| ENSOANG00000050846 | ENSOANG00000050846 | O_anatinus | Animals | mono_intergenic | H/ACA | | | intergenic |
| ENSOANG00000050847 | ENSOANG00000050847 | O_anatinus | Animals | mono_intergenic | H/ACA | | | intergenic |
| ENSOANG00000050851 | ENSOANG00000050851 | O_anatinus | Animals | mono_intergenic | H/ACA | | | intergenic |
| ENSOANG00000050852 | ENSOANG00000050852 | O_anatinus | Animals | mono_intergenic | H/ACA | | | intergenic |
| ENSOANG00000050853 | ENSOANG00000050853 | O_anatinus | Animals | mono_intergenic | H/ACA | | | intergenic |
| ENSOANG00000050856 | ENSOANG00000050856 | O_anatinus | Animals | mono_intergenic | H/ACA | | | intergenic |
| ENSOANG00000050857 | ENSOANG00000050857 | O_anatinus | Animals | mono_intergenic | H/ACA | | | intergenic |
| ENSOANG00000050860 | ENSOANG00000050860 | O_anatinus | Animals | mono_intergenic | H/ACA | | | intergenic |
| ENSOANG00000050862 | ENSOANG00000050862 | O_anatinus | Animals | mono_intergenic | H/ACA | | | intergenic |
| ENSOANG00000050863 | ENSOANG00000050863 | O_anatinus | Animals | mono_intergenic | H/ACA | | | intergenic |
| ENSOANG00000050868 | ENSOANG00000050868 | O_anatinus | Animals | mono_intergenic | H/ACA | | | intergenic |
| ENSOANG00000050871 | ENSOANG00000050871 | O_anatinus | Animals | mono_intergenic | H/ACA | | | intergenic |
| ENSOANG00000050873 | ENSOANG00000050873 | O_anatinus | Animals | mono_intergenic | H/ACA | | | intergenic |
| ENSOANG00000050878 | ENSOANG00000050878 | O_anatinus | Animals | mono_intergenic | H/ACA | | | intergenic |
| ENSOANG00000050881 | ENSOANG00000050881 | O_anatinus | Animals | mono_intergenic | H/ACA | | | intergenic |
| ENSOANG00000050882 | ENSOANG00000050882 | O_anatinus | Animals | mono_intronic | H/ACA | ENSOANG00000038995 | FBLN7 | protein_coding |
| ENSOANG00000050886 | ENSOANG00000050886 | O_anatinus | Animals | mono_intergenic | H/ACA | | | intergenic |
| ENSOANG00000050889 | ENSOANG00000050889 | O_anatinus | Animals | mono_intergenic | H/ACA | | | intergenic |
| ENSOANG00000050894 | ENSOANG00000050894 | O_anatinus | Animals | mono_intergenic | H/ACA | | | intergenic |
| ENSOANG00000050898 | ENSOANG00000050898 | O_anatinus | Animals | mono_intergenic | H/ACA | | | intergenic |
| ENSOANG00000050901 | ENSOANG00000050901 | O_anatinus | Animals | mono_intergenic | H/ACA | | | intergenic |
| ENSOANG00000050902 | ENSOANG00000050902 | O_anatinus | Animals | mono_intergenic | H/ACA | | | intergenic |
| ENSOANG00000050903 | ENSOANG00000050903 | O_anatinus | Animals | mono_intronic | H/ACA | ENSOANG00000043047 | | protein_coding |
| ENSOANG00000050904 | ENSOANG00000050904 | O_anatinus | Animals | intronic_cluster | H/ACA | ENSOANG00000013547 | PIAS1 | protein_coding |
| ENSOANG00000050905 | ENSOANG00000050905 | O_anatinus | Animals | mono_intronic | H/ACA | ENSOANG00000013182 | GFRA2 | protein_coding |
| ENSOANG00000050906 | SCARNA12 | O_anatinus | Animals | mono_intronic | C/D | ENSOANG00000037102 | PHB2 | protein_coding |
| ENSOANG00000050908 | ENSOANG00000050908 | O_anatinus | Animals | mono_intergenic | H/ACA | | | intergenic |
| ENSOANG00000050909 | ENSOANG00000050909 | O_anatinus | Animals | mono_intergenic | H/ACA | | | intergenic |
| ENSOANG00000050915 | ENSOANG00000050915 | O_anatinus | Animals | mono_intergenic | H/ACA | | | intergenic |
| ENSOANG00000050921 | ENSOANG00000050921 | O_anatinus | Animals | mono_intergenic | H/ACA | | | intergenic |
| ENSOANG00000050925 | ENSOANG00000050925 | O_anatinus | Animals | mono_intergenic | H/ACA | | | intergenic |
| ENSOANG00000050927 | ENSOANG00000050927 | O_anatinus | Animals | mono_intergenic | H/ACA | | | intergenic |
| ENSOANG00000050930 | ENSOANG00000050930 | O_anatinus | Animals | mono_intronic | H/ACA | ENSOANG00000011207 | COL4A3 | protein_coding |
| ENSOANG00000050931 | ENSOANG00000050931 | O_anatinus | Animals | mono_intergenic | H/ACA | | | intergenic |
| ENSOANG00000050933 | ENSOANG00000050933 | O_anatinus | Animals | mono_intergenic | H/ACA | | | intergenic |
| ENSOANG00000050935 | ENSOANG00000050935 | O_anatinus | Animals | mono_intergenic | H/ACA | | | intergenic |
| ENSOANG00000050937 | ENSOANG00000050937 | O_anatinus | Animals | mono_intergenic | H/ACA | | | intergenic |
| ENSOANG00000050940 | ENSOANG00000050940 | O_anatinus | Animals | mono_intronic | H/ACA | ENSOANG00000000486 | FRMD4B | protein_coding |

| | | | | | | | | |
|---|---|---|---|---|---|---|---|---|
| ENSOANG00000050943 | ENSOANG00000050943 | O_anatinus | Animals | mono_intergenic | H/ACA | | | intergenic |
| ENSOANG00000050944 | ENSOANG00000050944 | O_anatinus | Animals | mono_intergenic | H/ACA | | | intergenic |
| ENSOANG00000050945 | ENSOANG00000050945 | O_anatinus | Animals | mono_intergenic | H/ACA | | | intergenic |
| ENSOANG00000050946 | ENSOANG00000050946 | O_anatinus | Animals | mono_intergenic | H/ACA | | | intergenic |
| ENSOANG00000050947 | ENSOANG00000050947 | O_anatinus | Animals | mono_intronic | H/ACA | ENSOANG00000001477 | PRKCB | protein_coding |
| ENSOANG00000050949 | ENSOANG00000050949 | O_anatinus | Animals | mono_intergenic | H/ACA | | | intergenic |
| ENSOANG00000050951 | ENSOANG00000050951 | O_anatinus | Animals | mono_intergenic | H/ACA | | | intergenic |
| ENSOANG00000050953 | ENSOANG00000050953 | O_anatinus | Animals | mono_intergenic | H/ACA | | | intergenic |
| ENSOANG00000050954 | ENSOANG00000050954 | O_anatinus | Animals | mono_intergenic | H/ACA | | | intergenic |
| ENSOANG00000050957 | ENSOANG00000050957 | O_anatinus | Animals | mono_intronic | H/ACA | ENSOANG00000014942 | NDST3 | protein_coding |
| ENSOANG00000050960 | ENSOANG00000050960 | O_anatinus | Animals | mono_intergenic | H/ACA | | | intergenic |
| ENSOANG00000050964 | ENSOANG00000050964 | O_anatinus | Animals | mono_intergenic | H/ACA | | | intergenic |
| ENSOANG00000050966 | ENSOANG00000050966 | O_anatinus | Animals | mono_intergenic | H/ACA | | | intergenic |
| ENSOANG00000050968 | ENSOANG00000050968 | O_anatinus | Animals | mono_intergenic | H/ACA | | | intergenic |
| ENSOANG00000050969 | ENSOANG00000050969 | O_anatinus | Animals | mono_intergenic | H/ACA | | | intergenic |
| ENSOANG00000050972 | ENSOANG00000050972 | O_anatinus | Animals | mono_intergenic | H/ACA | | | intergenic |
| ENSOANG00000050978 | ENSOANG00000050978 | O_anatinus | Animals | mono_intergenic | H/ACA | | | intergenic |
| ENSOANG00000050982 | ENSOANG00000050982 | O_anatinus | Animals | mono_intergenic | H/ACA | | | intergenic |
| ENSOANG00000050985 | ENSOANG00000050985 | O_anatinus | Animals | mono_intergenic | H/ACA | | | intergenic |
| ENSOANG00000050986 | ENSOANG00000050986 | O_anatinus | Animals | intronic_cluster | H/ACA | ENSOANG00000008416 | PLCB1 | protein_coding |
| ENSOANG00000050987 | ENSOANG00000050987 | O_anatinus | Animals | mono_intergenic | H/ACA | | | intergenic |
| ENSOANG00000050988 | ENSOANG00000050988 | O_anatinus | Animals | mono_intergenic | H/ACA | | | intergenic |
| ENSOANG00000050989 | ENSOANG00000050989 | O_anatinus | Animals | mono_intergenic | H/ACA | | | intergenic |
| ENSOANG00000050990 | ENSOANG00000050990 | O_anatinus | Animals | mono_intronic | H/ACA | ENSOANG00000030635 | ANKUB1 | protein_coding |
| ENSOANG00000050995 | ENSOANG00000050995 | O_anatinus | Animals | mono_intergenic | H/ACA | | | intergenic |
| ENSOANG00000050996 | ENSOANG00000050996 | O_anatinus | Animals | mono_intergenic | H/ACA | | | intergenic |
| ENSOANG00000050998 | ENSOANG00000050998 | O_anatinus | Animals | mono_intergenic | H/ACA | | | intergenic |
| ENSOANG00000051000 | ENSOANG00000051000 | O_anatinus | Animals | mono_intergenic | H/ACA | | | intergenic |
| ENSOANG00000051001 | ENSOANG00000051001 | O_anatinus | Animals | mono_intergenic | H/ACA | | | intergenic |
| ENSOANG00000051002 | ENSOANG00000051002 | O_anatinus | Animals | mono_intergenic | H/ACA | | | intergenic |
| ENSOANG00000051004 | ENSOANG00000051004 | O_anatinus | Animals | mono_intergenic | H/ACA | | | intergenic |
| ENSOANG00000051007 | ENSOANG00000051007 | O_anatinus | Animals | mono_intergenic | H/ACA | | | intergenic |
| ENSOANG00000051011 | ENSOANG00000051011 | O_anatinus | Animals | mono_intergenic | H/ACA | | | intergenic |
| ENSOANG00000051015 | ENSOANG00000051015 | O_anatinus | Animals | mono_intergenic | H/ACA | | | intergenic |
| ENSOANG00000051016 | ENSOANG00000051016 | O_anatinus | Animals | mono_intronic | H/ACA | ENSOANG00000002626 | NPAS2 | protein_coding |
| ENSOANG00000051022 | ENSOANG00000051022 | O_anatinus | Animals | mono_intergenic | H/ACA | | | intergenic |
| ENSOANG00000051024 | ENSOANG00000051024 | O_anatinus | Animals | mono_intergenic | H/ACA | | | intergenic |
| ENSOANG00000051025 | ENSOANG00000051025 | O_anatinus | Animals | mono_intronic | H/ACA | ENSOANG00000015571 | TSPEAR | protein_coding |
| ENSOANG00000051026 | ENSOANG00000051026 | O_anatinus | Animals | mono_intergenic | H/ACA | | | intergenic |
| ENSOANG00000051027 | ENSOANG00000051027 | O_anatinus | Animals | mono_intergenic | H/ACA | | | intergenic |
| ENSOANG00000051030 | ENSOANG00000051030 | O_anatinus | Animals | mono_intergenic | H/ACA | | | intergenic |

| | | | | | | | | |
|---|---|---|---|---|---|---|---|---|
| ENSOANG00000051031 | ENSOANG00000051031 | O_anatinus | Animals | mono_intergenic | H/ACA | | | intergenic |
| ENSOANG00000051033 | ENSOANG00000051033 | O_anatinus | Animals | mono_intergenic | H/ACA | | | intergenic |
| ENSOANG00000051034 | ENSOANG00000051034 | O_anatinus | Animals | mono_intergenic | H/ACA | | | intergenic |
| ENSOANG00000051037 | ENSOANG00000051037 | O_anatinus | Animals | mono_intergenic | H/ACA | | | intergenic |
| ENSOANG00000051038 | ENSOANG00000051038 | O_anatinus | Animals | mono_intergenic | H/ACA | | | intergenic |
| ENSOANG00000051040 | ENSOANG00000051040 | O_anatinus | Animals | mono_intergenic | H/ACA | | | intergenic |
| ENSOANG00000051043 | ENSOANG00000051043 | O_anatinus | Animals | mono_intergenic | H/ACA | | | intergenic |
| ENSOANG00000051048 | ENSOANG00000051048 | O_anatinus | Animals | mono_intergenic | H/ACA | | | intergenic |
| ENSOANG00000051050 | ENSOANG00000051050 | O_anatinus | Animals | mono_intergenic | H/ACA | | | intergenic |
| ENSOANG00000051051 | ENSOANG00000051051 | O_anatinus | Animals | mono_intronic | H/ACA | ENSOANG00000015364 | TTC7B | protein_coding |
| ENSOANG00000051052 | ENSOANG00000051052 | O_anatinus | Animals | mono_intergenic | H/ACA | | | intergenic |
| ENSOANG00000051053 | ENSOANG00000051053 | O_anatinus | Animals | mono_intergenic | H/ACA | | | intergenic |
| ENSOANG00000051057 | ENSOANG00000051057 | O_anatinus | Animals | mono_intergenic | H/ACA | | | intergenic |
| ENSOANG00000051059 | ENSOANG00000051059 | O_anatinus | Animals | mono_intergenic | H/ACA | | | intergenic |
| ENSOANG00000051060 | ENSOANG00000051060 | O_anatinus | Animals | mono_intergenic | H/ACA | | | intergenic |
| ENSOANG00000051062 | ENSOANG00000051062 | O_anatinus | Animals | mono_intergenic | H/ACA | | | intergenic |
| ENSOANG00000051065 | ENSOANG00000051065 | O_anatinus | Animals | mono_intronic | H/ACA | ENSOANG00000003100 | AP3B1 | protein_coding |
| ENSOANG00000051066 | ENSOANG00000051066 | O_anatinus | Animals | mono_intergenic | H/ACA | | | intergenic |
| ENSOANG00000051067 | ENSOANG00000051067 | O_anatinus | Animals | mono_intergenic | H/ACA | | | intergenic |
| ENSOANG00000051068 | ENSOANG00000051068 | O_anatinus | Animals | intronic_cluster | H/ACA | ENSOANG00000001492 | PTPRD | protein_coding |
| ENSOANG00000051069 | ENSOANG00000051069 | O_anatinus | Animals | mono_intergenic | H/ACA | | | intergenic |
| ENSOANG00000051072 | ENSOANG00000051072 | O_anatinus | Animals | mono_intergenic | H/ACA | | | intergenic |
| ENSOANG00000051073 | ENSOANG00000051073 | O_anatinus | Animals | mono_intronic | H/ACA | ENSOANG00000044353 | | protein_coding |
| ENSOANG00000051077 | ENSOANG00000051077 | O_anatinus | Animals | mono_intergenic | H/ACA | | | intergenic |
| ENSOANG00000051082 | ENSOANG00000051082 | O_anatinus | Animals | mono_intronic | H/ACA | ENSOANG00000012553 | RIOK1 | protein_coding |
| ENSOANG00000051083 | ENSOANG00000051083 | O_anatinus | Animals | mono_intergenic | H/ACA | | | intergenic |
| ENSOANG00000051085 | ENSOANG00000051085 | O_anatinus | Animals | mono_intergenic | H/ACA | | | intergenic |
| ENSOANG00000051086 | ENSOANG00000051086 | O_anatinus | Animals | mono_intergenic | H/ACA | | | intergenic |
| ENSOANG00000051089 | ENSOANG00000051089 | O_anatinus | Animals | mono_intergenic | H/ACA | | | intergenic |
| ENSOANG00000051092 | ENSOANG00000051092 | O_anatinus | Animals | mono_intergenic | H/ACA | | | intergenic |
| ENSOANG00000051093 | ENSOANG00000051093 | O_anatinus | Animals | mono_intergenic | H/ACA | | | intergenic |
| ENSOANG00000051097 | ENSOANG00000051097 | O_anatinus | Animals | mono_intergenic | H/ACA | | | intergenic |
| ENSOANG00000051098 | ENSOANG00000051098 | O_anatinus | Animals | mono_intronic | H/ACA | ENSOANG00000041958 | | protein_coding |
| ENSOANG00000051100 | ENSOANG00000051100 | O_anatinus | Animals | mono_intergenic | H/ACA | | | intergenic |
| ENSOANG00000051101 | ENSOANG00000051101 | O_anatinus | Animals | mono_intergenic | H/ACA | | | intergenic |
| ENSOANG00000051103 | ENSOANG00000051103 | O_anatinus | Animals | mono_intergenic | H/ACA | | | intergenic |
| ENSOANG00000051106 | ENSOANG00000051106 | O_anatinus | Animals | mono_intergenic | H/ACA | | | intergenic |
| ENSOANG00000051107 | ENSOANG00000051107 | O_anatinus | Animals | mono_intergenic | H/ACA | | | intergenic |
| ENSOANG00000051108 | ENSOANG00000051108 | O_anatinus | Animals | mono_intergenic | H/ACA | | | intergenic |
| ENSOANG00000051115 | ENSOANG00000051115 | O_anatinus | Animals | mono_intergenic | H/ACA | | | intergenic |
| ENSOANG00000051119 | ENSOANG00000051119 | O_anatinus | Animals | mono_intergenic | H/ACA | | | intergenic |

| | | | | | | | | |
|---|---|---|---|---|---|---|---|---|
| ENSOANG00000051124 | ENSOANG00000051124 | O_anatinus | Animals | mono_intergenic | H/ACA | | | intergenic |
| ENSOANG00000051125 | ENSOANG00000051125 | O_anatinus | Animals | mono_intergenic | H/ACA | | | intergenic |
| ENSOANG00000051126 | ENSOANG00000051126 | O_anatinus | Animals | mono_intergenic | H/ACA | | | intergenic |
| ENSOANG00000051136 | ENSOANG00000051136 | O_anatinus | Animals | mono_intergenic | H/ACA | | | intergenic |
| ENSOANG00000051137 | U3 | O_anatinus | Animals | mono_intronic | C/D | ENSOANG00000043317 | | protein_coding |
| ENSOANG00000051141 | ENSOANG00000051141 | O_anatinus | Animals | mono_intergenic | H/ACA | | | intergenic |
| ENSOANG00000051142 | ENSOANG00000051142 | O_anatinus | Animals | mono_intergenic | H/ACA | | | intergenic |
| ENSOANG00000051144 | SNORD127 | O_anatinus | Animals | mono_intronic | C/D | ENSOANG00000014000 | PRPF39 | protein_coding |
| ENSOANG00000051145 | ENSOANG00000051145 | O_anatinus | Animals | mono_intergenic | H/ACA | | | intergenic |
| ENSOANG00000051148 | ENSOANG00000051148 | O_anatinus | Animals | mono_intergenic | H/ACA | | | intergenic |
| ENSOANG00000051149 | ENSOANG00000051149 | O_anatinus | Animals | mono_intronic | H/ACA | ENSOANG00000009178 | CREB5 | protein_coding |
| ENSOANG00000051150 | ENSOANG00000051150 | O_anatinus | Animals | mono_intergenic | H/ACA | | | intergenic |
| ENSOANG00000051153 | ENSOANG00000051153 | O_anatinus | Animals | mono_intergenic | H/ACA | | | intergenic |
| ENSOANG00000051159 | ENSOANG00000051159 | O_anatinus | Animals | mono_intronic | H/ACA | ENSOANG00000003131 | PKHD1 | protein_coding |
| ENSOANG00000051160 | ENSOANG00000051160 | O_anatinus | Animals | mono_intergenic | H/ACA | | | intergenic |
| ENSOANG00000051166 | ENSOANG00000051166 | O_anatinus | Animals | mono_intergenic | H/ACA | | | intergenic |
| ENSOANG00000051168 | ENSOANG00000051168 | O_anatinus | Animals | mono_intergenic | H/ACA | | | intergenic |
| ENSOANG00000051169 | ENSOANG00000051169 | O_anatinus | Animals | mono_intergenic | H/ACA | | | intergenic |
| ENSOANG00000051172 | ENSOANG00000051172 | O_anatinus | Animals | mono_intergenic | H/ACA | | | intergenic |
| ENSOANG00000051177 | ENSOANG00000051177 | O_anatinus | Animals | mono_intergenic | H/ACA | | | intergenic |
| ENSOANG00000051180 | ENSOANG00000051180 | O_anatinus | Animals | mono_intergenic | H/ACA | | | intergenic |
| ENSOANG00000051181 | ENSOANG00000051181 | O_anatinus | Animals | intergenic_cluster | H/ACA | | | intergenic |
| ENSOANG00000051186 | ENSOANG00000051186 | O_anatinus | Animals | mono_intergenic | H/ACA | | | intergenic |
| ENSOANG00000051187 | ENSOANG00000051187 | O_anatinus | Animals | mono_intergenic | H/ACA | | | intergenic |
| ENSOANG00000051190 | ENSOANG00000051190 | O_anatinus | Animals | mono_intronic | H/ACA | ENSOANG00000008721 | | protein_coding |
| ENSOANG00000051191 | ENSOANG00000051191 | O_anatinus | Animals | mono_intergenic | H/ACA | | | intergenic |
| ENSOANG00000051192 | ENSOANG00000051192 | O_anatinus | Animals | mono_intergenic | H/ACA | | | intergenic |
| ENSOANG00000051193 | ENSOANG00000051193 | O_anatinus | Animals | mono_intergenic | H/ACA | | | intergenic |
| ENSOANG00000051195 | ENSOANG00000051195 | O_anatinus | Animals | mono_intergenic | H/ACA | | | intergenic |
| ENSOANG00000051196 | ENSOANG00000051196 | O_anatinus | Animals | mono_intergenic | H/ACA | | | intergenic |
| ENSOANG00000051206 | ENSOANG00000051206 | O_anatinus | Animals | mono_intergenic | H/ACA | | | intergenic |
| ENSOANG00000051211 | ENSOANG00000051211 | O_anatinus | Animals | mono_intergenic | H/ACA | | | intergenic |
| ENSOANG00000051215 | ENSOANG00000051215 | O_anatinus | Animals | mono_intergenic | H/ACA | | | intergenic |
| ENSOANG00000051217 | ENSOANG00000051217 | O_anatinus | Animals | mono_intronic | H/ACA | ENSOANG00000051152 | MDFIC2 | protein_coding |
| ENSOANG00000051219 | ENSOANG00000051219 | O_anatinus | Animals | mono_intergenic | H/ACA | | | intergenic |
| ENSOANG00000051220 | ENSOANG00000051220 | O_anatinus | Animals | mono_intergenic | H/ACA | | | intergenic |
| ENSOANG00000051222 | ENSOANG00000051222 | O_anatinus | Animals | mono_intergenic | H/ACA | | | intergenic |
| ENSOANG00000051223 | ENSOANG00000051223 | O_anatinus | Animals | mono_intergenic | H/ACA | | | intergenic |
| ENSOANG00000051225 | ENSOANG00000051225 | O_anatinus | Animals | mono_intergenic | H/ACA | | | intergenic |
| ENSOANG00000051227 | ENSOANG00000051227 | O_anatinus | Animals | mono_intronic | H/ACA | ENSOANG00000006824 | | protein_coding |
| ENSOANG00000051228 | SNORD58 | O_anatinus | Animals | mono_intronic | C/D | ENSOANG00000002910 | | protein_coding |

| | | | | | | | | |
|---|---|---|---|---|---|---|---|---|
| ENSOANG00000051229 | ENSOANG00000051229 | O_anatinus | Animals | mono_intronic | H/ACA | ENSOANG00000011102 | FNDC7 | protein_coding |
| ENSOANG00000051236 | ENSOANG00000051236 | O_anatinus | Animals | mono_intergenic | H/ACA | | | intergenic |
| ENSOANG00000051238 | ENSOANG00000051238 | O_anatinus | Animals | mono_intergenic | H/ACA | | | intergenic |
| ENSOANG00000051239 | ENSOANG00000051239 | O_anatinus | Animals | mono_intergenic | H/ACA | | | intergenic |
| ENSOANG00000051241 | ENSOANG00000051241 | O_anatinus | Animals | mono_intergenic | H/ACA | | | intergenic |
| ENSOANG00000051250 | ENSOANG00000051250 | O_anatinus | Animals | mono_intergenic | H/ACA | | | intergenic |
| ENSOANG00000051252 | ENSOANG00000051252 | O_anatinus | Animals | mono_intergenic | H/ACA | | | intergenic |
| ENSOANG00000051254 | ENSOANG00000051254 | O_anatinus | Animals | mono_intergenic | H/ACA | | | intergenic |
| ENSOANG00000051255 | ENSOANG00000051255 | O_anatinus | Animals | mono_intergenic | H/ACA | | | intergenic |
| ENSOANG00000051256 | ENSOANG00000051256 | O_anatinus | Animals | mono_intergenic | H/ACA | | | intergenic |
| ENSOANG00000051259 | ENSOANG00000051259 | O_anatinus | Animals | mono_intergenic | H/ACA | | | intergenic |
| ENSOANG00000051260 | ENSOANG00000051260 | O_anatinus | Animals | mono_intergenic | H/ACA | | | intergenic |
| ENSOANG00000051261 | ENSOANG00000051261 | O_anatinus | Animals | mono_intergenic | H/ACA | | | intergenic |
| ENSOANG00000051262 | ENSOANG00000051262 | O_anatinus | Animals | mono_intergenic | H/ACA | | | intergenic |
| ENSOANG00000051264 | ENSOANG00000051264 | O_anatinus | Animals | mono_intergenic | H/ACA | | | intergenic |
| ENSOANG00000051270 | ENSOANG00000051270 | O_anatinus | Animals | mono_intronic | H/ACA | ENSOANG00000040269 | | protein_coding |
| ENSOANG00000051273 | ENSOANG00000051273 | O_anatinus | Animals | mono_intergenic | H/ACA | | | intergenic |
| ENSOANG00000051277 | ENSOANG00000051277 | O_anatinus | Animals | mono_intergenic | H/ACA | | | intergenic |
| ENSOANG00000051281 | ENSOANG00000051281 | O_anatinus | Animals | mono_intronic | H/ACA | ENSOANG00000008490 | STK39 | protein_coding |
| ENSOANG00000051283 | ENSOANG00000051283 | O_anatinus | Animals | intronic_cluster | H/ACA | ENSOANG00000005642 | NR5A2 | protein_coding |
| ENSOANG00000051284 | ENSOANG00000051284 | O_anatinus | Animals | mono_intergenic | H/ACA | | | intergenic |
| ENSOANG00000051285 | ENSOANG00000051285 | O_anatinus | Animals | mono_intergenic | H/ACA | | | intergenic |
| ENSOANG00000051286 | ENSOANG00000051286 | O_anatinus | Animals | mono_intergenic | H/ACA | | | intergenic |
| ENSOANG00000051287 | ENSOANG00000051287 | O_anatinus | Animals | mono_intergenic | H/ACA | | | intergenic |
| ENSOANG00000051290 | ENSOANG00000051290 | O_anatinus | Animals | mono_intergenic | H/ACA | | | intergenic |
| ENSOANG00000051291 | ENSOANG00000051291 | O_anatinus | Animals | mono_intergenic | H/ACA | | | intergenic |
| ENSOANG00000051293 | ENSOANG00000051293 | O_anatinus | Animals | mono_intronic | H/ACA | ENSOANG00000007407 | SHANK2 | protein_coding |
| ENSOANG00000051294 | ENSOANG00000051294 | O_anatinus | Animals | mono_intergenic | H/ACA | | | intergenic |
| ENSOANG00000051297 | ENSOANG00000051297 | O_anatinus | Animals | mono_intergenic | H/ACA | | | intergenic |
| ENSOANG00000051298 | ENSOANG00000051298 | O_anatinus | Animals | mono_intronic | H/ACA | ENSOANG00000007273 | PCMTD1 | protein_coding |
| ENSOANG00000051302 | ENSOANG00000051302 | O_anatinus | Animals | mono_intergenic | H/ACA | | | intergenic |
| ENSOANG00000051303 | ENSOANG00000051303 | O_anatinus | Animals | mono_intergenic | H/ACA | | | intergenic |
| ENSOANG00000051307 | ENSOANG00000051307 | O_anatinus | Animals | mono_intergenic | H/ACA | | | intergenic |
| ENSOANG00000051308 | ENSOANG00000051308 | O_anatinus | Animals | mono_intergenic | H/ACA | | | intergenic |
| ENSOANG00000051310 | ENSOANG00000051310 | O_anatinus | Animals | mono_intergenic | H/ACA | | | intergenic |
| ENSOANG00000051312 | ENSOANG00000051312 | O_anatinus | Animals | mono_intronic | H/ACA | ENSOANG00000014784 | MED27 | protein_coding |
| ENSOANG00000051313 | ENSOANG00000051313 | O_anatinus | Animals | mono_intergenic | H/ACA | | | intergenic |
| ENSOANG00000051314 | ENSOANG00000051314 | O_anatinus | Animals | mono_intronic | H/ACA | ENSOANG00000007906 | AGBL2 | protein_coding |
| ENSOANG00000051315 | ENSOANG00000051315 | O_anatinus | Animals | mono_intergenic | H/ACA | | | intergenic |
| ENSOANG00000051317 | ENSOANG00000051317 | O_anatinus | Animals | mono_intergenic | H/ACA | | | intergenic |
| ENSOANG00000051324 | ENSOANG00000051324 | O_anatinus | Animals | mono_intergenic | H/ACA | | | intergenic |

| | | | | | | | | |
|---|---|---|---|---|---|---|---|---|
| ENSOANG00000051325 | ENSOANG00000051325 | O_anatinus | Animals | mono_intergenic | H/ACA | | | intergenic |
| ENSOANG00000051328 | ENSOANG00000051328 | O_anatinus | Animals | mono_intergenic | H/ACA | | | intergenic |
| ENSOANG00000051329 | ENSOANG00000051329 | O_anatinus | Animals | mono_intergenic | H/ACA | | | intergenic |
| ENSOANG00000051331 | ENSOANG00000051331 | O_anatinus | Animals | mono_intergenic | H/ACA | | | intergenic |
| ENSOANG00000051332 | ENSOANG00000051332 | O_anatinus | Animals | mono_intergenic | H/ACA | | | intergenic |
| ENSOANG00000051336 | ENSOANG00000051336 | O_anatinus | Animals | mono_intronic | H/ACA | ENSOANG00000021911 | | protein_coding |
| ENSOANG00000051338 | ENSOANG00000051338 | O_anatinus | Animals | mono_intergenic | H/ACA | | | intergenic |
| ENSOANG00000051343 | ENSOANG00000051343 | O_anatinus | Animals | mono_intergenic | H/ACA | | | intergenic |
| ENSOANG00000051344 | ENSOANG00000051344 | O_anatinus | Animals | mono_intergenic | H/ACA | | | intergenic |
| ENSOANG00000051345 | ENSOANG00000051345 | O_anatinus | Animals | mono_intergenic | H/ACA | | | intergenic |
| ENSOANG00000051346 | ENSOANG00000051346 | O_anatinus | Animals | mono_intergenic | H/ACA | | | intergenic |
| ENSOANG00000051349 | ENSOANG00000051349 | O_anatinus | Animals | mono_intergenic | H/ACA | | | intergenic |
| ENSOANG00000051350 | ENSOANG00000051350 | O_anatinus | Animals | mono_intergenic | H/ACA | | | intergenic |
| ENSOANG00000051351 | ENSOANG00000051351 | O_anatinus | Animals | mono_intergenic | H/ACA | | | intergenic |
| ENSOANG00000051352 | ENSOANG00000051352 | O_anatinus | Animals | mono_intergenic | H/ACA | | | intergenic |
| ENSOANG00000051354 | ENSOANG00000051354 | O_anatinus | Animals | mono_intergenic | H/ACA | | | intergenic |
| ENSOANG00000051355 | ENSOANG00000051355 | O_anatinus | Animals | mono_intergenic | H/ACA | | | intergenic |
| ENSOANG00000051356 | ENSOANG00000051356 | O_anatinus | Animals | mono_intronic | H/ACA | ENSOANG00000047654 | | non_coding |
| ENSRNOG00000040595 | LOC120096376 | R_norvegicus | Animals | intergenic_cluster | C/D | | | intergenic |
| ENSRNOG00000044194 | Snora47 | R_norvegicus | Animals | mono_intronic | H/ACA | ENSRNOG00000028941 | Zbed3 | protein_coding |
| ENSRNOG00000051367 | LOC120094943 | R_norvegicus | Animals | mono_intronic | C/D | ENSRNOG00000016486 | Nop58 | protein_coding |
| ENSRNOG00000051373 | LOC120096411 | R_norvegicus | Animals | mono_intergenic | H/ACA | | | intergenic |
| ENSRNOG00000051388 | LOC120101321 | R_norvegicus | Animals | mono_intergenic | H/ACA | | | intergenic |
| ENSRNOG00000051403 | LOC120097219 | R_norvegicus | Animals | mono_intergenic | H/ACA | | | intergenic |
| ENSRNOG00000051409 | LOC120100372 | R_norvegicus | Animals | mono_intergenic | H/ACA | | | intergenic |
| ENSRNOG00000051411 | LOC120094514 | R_norvegicus | Animals | mono_intergenic | H/ACA | | | intergenic |
| ENSRNOG00000051415 | LOC120100379 | R_norvegicus | Animals | mono_intergenic | H/ACA | | | intergenic |
| ENSRNOG00000051422 | LOC120094941 | R_norvegicus | Animals | mono_intergenic | H/ACA | | | intergenic |
| ENSRNOG00000051427 | LOC120097999 | R_norvegicus | Animals | mono_intronic | H/ACA | ENSRNOG00000059705 | Elmo1 | protein_coding |
| ENSRNOG00000051428 | LOC120097974 | R_norvegicus | Animals | mono_intergenic | H/ACA | | | intergenic |
| ENSRNOG00000051434 | LOC120095756 | R_norvegicus | Animals | mono_intergenic | H/ACA | | | intergenic |
| ENSRNOG00000051445 | LOC120102629 | R_norvegicus | Animals | mono_intergenic | H/ACA | | | intergenic |
| ENSRNOG00000051455 | LOC120102102 | R_norvegicus | Animals | mono_intergenic | H/ACA | | | intergenic |
| ENSRNOG00000051473 | LOC120099079 | R_norvegicus | Animals | mono_intergenic | H/ACA | | | intergenic |
| ENSRNOG00000051475 | LOC120097623 | R_norvegicus | Animals | mono_intergenic | H/ACA | | | intergenic |
| ENSRNOG00000051492 | Snora74a | R_norvegicus | Animals | mono_exonic | H/ACA | ENSRNOG00000070938 | | non_coding |
| ENSRNOG00000051511 | LOC120103253 | R_norvegicus | Animals | mono_intergenic | H/ACA | | | intergenic |
| ENSRNOG00000051515 | LOC120096836 | R_norvegicus | Animals | mono_intronic | C/D | ENSRNOG00000008786 | Ap1b1 | protein_coding |
| ENSRNOG00000051532 | LOC120097581 | R_norvegicus | Animals | mono_intronic | H/ACA | ENSRNOG00000018795 | Rpl18a | protein_coding |
| ENSRNOG00000051544 | LOC120099460 | R_norvegicus | Animals | mono_intergenic | H/ACA | | | intergenic |
| ENSRNOG00000051550 | LOC120097608 | R_norvegicus | Animals | mono_intergenic | H/ACA | | | intergenic |

| | | | | | | | | |
|---|---|---|---|---|---|---|---|---|
| ENSRNOG00000051556 | LOC120102050 | R_norvegicus | Animals | mono_intergenic | H/ACA | | | intergenic |
| ENSRNOG00000051561 | LOC120100354 | R_norvegicus | Animals | mono_intronic | H/ACA | ENSRNOG00000020394 | Mcmbp | protein_coding |
| ENSRNOG00000051585 | Snora22c | R_norvegicus | Animals | mono_intronic | H/ACA | ENSRNOG00000000923 | Cct6a | protein_coding |
| ENSRNOG00000051587 | LOC120097604 | R_norvegicus | Animals | mono_intergenic | H/ACA | | | intergenic |
| ENSRNOG00000051605 | Snord86 | R_norvegicus | Animals | mono_intronic | C/D | ENSRNOG00000007128 | Nop56 | protein_coding |
| ENSRNOG00000051642 | LOC120096829 | R_norvegicus | Animals | mono_intergenic | H/ACA | | | intergenic |
| ENSRNOG00000051643 | LOC120103326 | R_norvegicus | Animals | mono_intergenic | H/ACA | | | intergenic |
| ENSRNOG00000051644 | LOC120099468 | R_norvegicus | Animals | mono_intergenic | H/ACA | | | intergenic |
| ENSRNOG00000051647 | LOC120094486 | R_norvegicus | Animals | mono_intergenic | H/ACA | | | intergenic |
| ENSRNOG00000051658 | LOC120094939 | R_norvegicus | Animals | mono_intergenic | H/ACA | | | intergenic |
| ENSRNOG00000051665 | LOC120102075 | R_norvegicus | Animals | mono_intergenic | H/ACA | | | intergenic |
| ENSRNOG00000051673 | LOC120101323 | R_norvegicus | Animals | mono_intergenic | H/ACA | | | intergenic |
| ENSRNOG00000051692 | LOC120093954 | R_norvegicus | Animals | mono_intergenic | H/ACA | | | intergenic |
| ENSRNOG00000051714 | LOC120102625 | R_norvegicus | Animals | mono_intronic | H/ACA | ENSRNOG00000005480 | Ybx3 | protein_coding |
| ENSRNOG00000051715 | LOC120098367 | R_norvegicus | Animals | mono_intergenic | H/ACA | | | intergenic |
| ENSRNOG00000051717 | LOC120102704 | R_norvegicus | Animals | mono_intergenic | H/ACA | | | intergenic |
| ENSRNOG00000051718 | LOC120102692 | R_norvegicus | Animals | mono_intergenic | H/ACA | | | intergenic |
| ENSRNOG00000051730 | LOC120093045 | R_norvegicus | Animals | mono_intergenic | H/ACA | | | intergenic |
| ENSRNOG00000051736 | LOC120096832 | R_norvegicus | Animals | mono_intergenic | H/ACA | | | intergenic |
| ENSRNOG00000051738 | LOC120094490 | R_norvegicus | Animals | mono_intergenic | H/ACA | | | intergenic |
| ENSRNOG00000051751 | LOC120095761 | R_norvegicus | Animals | mono_intergenic | H/ACA | | | intergenic |
| ENSRNOG00000051769 | LOC120099506 | R_norvegicus | Animals | mono_intergenic | H/ACA | | | intergenic |
| ENSRNOG00000051780 | LOC120100462 | R_norvegicus | Animals | mono_intergenic | H/ACA | | | intergenic |
| ENSRNOG00000051790 | LOC120093991 | R_norvegicus | Animals | mono_intergenic | H/ACA | | | intergenic |
| ENSRNOG00000051794 | LOC120102044 | R_norvegicus | Animals | mono_intergenic | H/ACA | | | intergenic |
| ENSRNOG00000051807 | LOC120096119 | R_norvegicus | Animals | mono_intergenic | H/ACA | | | intergenic |
| ENSRNOG00000051820 | LOC120099510 | R_norvegicus | Animals | mono_intergenic | H/ACA | | | intergenic |
| ENSRNOG00000051833 | LOC120098006 | R_norvegicus | Animals | mono_intergenic | H/ACA | | | intergenic |
| ENSRNOG00000051842 | LOC120103267 | R_norvegicus | Animals | mono_intronic | C/D | ENSRNOG00000029427 | Grhl3 | protein_coding |
| ENSRNOG00000051864 | LOC120099017 | R_norvegicus | Animals | mono_intergenic | H/ACA | | | intergenic |
| ENSRNOG00000051870 | LOC120096125 | R_norvegicus | Animals | mono_intergenic | H/ACA | | | intergenic |
| ENSRNOG00000051871 | LOC120101329 | R_norvegicus | Animals | mono_intergenic | H/ACA | | | intergenic |
| ENSRNOG00000051873 | LOC120101331 | R_norvegicus | Animals | mono_intergenic | H/ACA | | | intergenic |
| ENSRNOG00000051897 | LOC120094909 | R_norvegicus | Animals | mono_intergenic | H/ACA | | | intergenic |
| ENSRNOG00000051898 | LOC120095771 | R_norvegicus | Animals | mono_intergenic | H/ACA | | | intergenic |
| ENSRNOG00000051900 | LOC120094891 | R_norvegicus | Animals | mono_intergenic | H/ACA | | | intergenic |
| ENSRNOG00000051908 | LOC120102054 | R_norvegicus | Animals | mono_intergenic | H/ACA | | | intergenic |
| ENSRNOG00000051910 | LOC120094930 | R_norvegicus | Animals | mono_intergenic | H/ACA | | | intergenic |
| ENSRNOG00000051914 | LOC120102718 | R_norvegicus | Animals | mono_intergenic | H/ACA | | | intergenic |
| ENSRNOG00000051919 | LOC120102064 | R_norvegicus | Animals | mono_intergenic | H/ACA | | | intergenic |
| ENSRNOG00000051923 | LOC120093413 | R_norvegicus | Animals | mono_intergenic | C/D | | | intergenic |

| | | | | | | | | |
|---|---|---|---|---|---|---|---|---|
| ENSRNOG00000051927 | Snord98 | R_norvegicus | Animals | mono_intronic | C/D | ENSRNOG00000000397 | Ccar1 | protein_coding |
| ENSRNOG00000051930 | LOC120101375 | R_norvegicus | Animals | mono_intergenic | C/D | | | intergenic |
| ENSRNOG00000051969 | Snora29 | R_norvegicus | Animals | mono_intronic | H/ACA | ENSRNOG00000014160 | Tcp1 | protein_coding |
| ENSRNOG00000051976 | LOC120093410 | R_norvegicus | Animals | mono_intergenic | C/D | | | intergenic |
| ENSRNOG00000051981 | LOC120103331 | R_norvegicus | Animals | mono_intergenic | H/ACA | | | intergenic |
| ENSRNOG00000051987 | LOC120103346 | R_norvegicus | Animals | mono_intergenic | H/ACA | | | intergenic |
| ENSRNOG00000051992 | LOC120094900 | R_norvegicus | Animals | mono_exonic | H/ACA | ENSRNOG00000045605 | Uxs1 | protein_coding |
| ENSRNOG00000051998 | LOC120096823 | R_norvegicus | Animals | mono_intergenic | H/ACA | | | intergenic |
| ENSRNOG00000052013 | Snord81 | R_norvegicus | Animals | intergenic_cluster | C/D | | | intergenic |
| ENSRNOG00000052026 | LOC120095775 | R_norvegicus | Animals | mono_intronic | H/ACA | ENSRNOG00000001884 | Ranbp1 | protein_coding |
| ENSRNOG00000052033 | LOC120102608 | R_norvegicus | Animals | mono_intronic | H/ACA | ENSRNOG00000021468 | Grm8 | protein_coding |
| ENSRNOG00000052045 | LOC120094924 | R_norvegicus | Animals | mono_intergenic | H/ACA | | | intergenic |
| ENSRNOG00000052060 | LOC120103343 | R_norvegicus | Animals | mono_intergenic | H/ACA | | | intergenic |
| ENSRNOG00000052065 | LOC120096401 | R_norvegicus | Animals | mono_intergenic | H/ACA | | | intergenic |
| ENSRNOG00000052072 | LOC120098697 | R_norvegicus | Animals | mono_intergenic | H/ACA | | | intergenic |
| ENSRNOG00000052082 | LOC120094515 | R_norvegicus | Animals | mono_intergenic | H/ACA | | | intergenic |
| ENSRNOG00000052098 | LOC120095760 | R_norvegicus | Animals | mono_intergenic | H/ACA | | | intergenic |
| ENSRNOG00000052115 | LOC120095401 | R_norvegicus | Animals | mono_intronic | H/ACA | ENSRNOG00000030628 | Eif4a1 | protein_coding |
| ENSRNOG00000052126 | LOC120094498 | R_norvegicus | Animals | mono_intergenic | H/ACA | | | intergenic |
| ENSRNOG00000052131 | LOC120102660 | R_norvegicus | Animals | mono_intronic | H/ACA | ENSRNOG00000014713 | Csgalnact2 | protein_coding |
| ENSRNOG00000052146 | LOC120096405 | R_norvegicus | Animals | mono_intergenic | H/ACA | | | intergenic |
| ENSRNOG00000052156 | LOC120094886 | R_norvegicus | Animals | mono_intronic | C/D | ENSRNOG00000024186 | Eef1b2 | protein_coding |
| ENSRNOG00000052158 | LOC120094511 | R_norvegicus | Animals | mono_intergenic | H/ACA | | | intergenic |
| ENSRNOG00000052161 | LOC120094526 | R_norvegicus | Animals | mono_intronic | C/D | ENSRNOG00000010921 | Taf1d | protein_coding |
| ENSRNOG00000052172 | LOC120098676 | R_norvegicus | Animals | mono_intergenic | C/D | | | intergenic |
| ENSRNOG00000052179 | LOC120100397 | R_norvegicus | Animals | mono_intergenic | H/ACA | | | intergenic |
| ENSRNOG00000052183 | LOC120093473 | R_norvegicus | Animals | mono_intronic | H/ACA | ENSRNOG00000034026 | Lclat1 | protein_coding |
| ENSRNOG00000052197 | LOC120102013 | R_norvegicus | Animals | mono_intergenic | H/ACA | | | intergenic |
| ENSRNOG00000052201 | LOC120102095 | R_norvegicus | Animals | mono_intronic | H/ACA | ENSRNOG00000006419 | Aven | protein_coding |
| ENSRNOG00000052202 | LOC120097213 | R_norvegicus | Animals | mono_intergenic | C/D | | | intergenic |
| ENSRNOG00000052210 | LOC120099020 | R_norvegicus | Animals | mono_intergenic | H/ACA | | | intergenic |
| ENSRNOG00000052213 | LOC120102735 | R_norvegicus | Animals | mono_intergenic | H/ACA | | | intergenic |
| ENSRNOG00000052215 | LOC120093430 | R_norvegicus | Animals | mono_intergenic | H/ACA | | | intergenic |
| ENSRNOG00000052218 | LOC120093994 | R_norvegicus | Animals | mono_intronic | H/ACA | ENSRNOG00000025344 | Enthd1 | non_coding |
| ENSRNOG00000052250 | LOC120096404 | R_norvegicus | Animals | mono_intergenic | H/ACA | | | intergenic |
| ENSRNOG00000052252 | LOC120096419 | R_norvegicus | Animals | intergenic_cluster | H/ACA | | | intergenic |
| ENSRNOG00000052262 | LOC120097622 | R_norvegicus | Animals | mono_intergenic | H/ACA | | | intergenic |
| ENSRNOG00000052274 | LOC120100342 | R_norvegicus | Animals | mono_intergenic | H/ACA | | | intergenic |
| ENSRNOG00000052285 | LOC120093429 | R_norvegicus | Animals | mono_intergenic | H/ACA | | | intergenic |
| ENSRNOG00000052293 | Snord15a | R_norvegicus | Animals | mono_intronic | C/D | ENSRNOG00000017418 | Rps3 | protein_coding |
| ENSRNOG00000052295 | LOC120102058 | R_norvegicus | Animals | mono_intergenic | H/ACA | | | intergenic |

| | | | | | | | | |
|---|---|---|---|---|---|---|---|---|
| ENSRNOG00000052300 | Snord35b | R_norvegicus | Animals | mono_intronic | C/D | ENSRNOG00000020595 | Rps11 | protein_coding |
| ENSRNOG00000052312 | LOC120098356 | R_norvegicus | Animals | mono_intronic | H/ACA | ENSRNOG00000013281 | Mib1 | protein_coding |
| ENSRNOG00000052314 | LOC120101253 | R_norvegicus | Animals | mono_intergenic | H/ACA | | | intergenic |
| ENSRNOG00000052338 | LOC120095736 | R_norvegicus | Animals | mono_intergenic | H/ACA | | | intergenic |
| ENSRNOG00000052344 | LOC120095463 | R_norvegicus | Animals | intergenic_cluster | H/ACA | | | intergenic |
| ENSRNOG00000052370 | LOC120094489 | R_norvegicus | Animals | mono_intergenic | H/ACA | | | intergenic |
| ENSRNOG00000052375 | LOC120100435 | R_norvegicus | Animals | mono_intergenic | H/ACA | | | intergenic |
| ENSRNOG00000052403 | LOC120096399 | R_norvegicus | Animals | mono_intergenic | H/ACA | | | intergenic |
| ENSRNOG00000052404 | LOC120099026 | R_norvegicus | Animals | mono_intergenic | H/ACA | | | intergenic |
| ENSRNOG00000052408 | LOC120102673 | R_norvegicus | Animals | mono_intergenic | H/ACA | | | intergenic |
| ENSRNOG00000052410 | LOC120103301 | R_norvegicus | Animals | mono_intergenic | H/ACA | | | intergenic |
| ENSRNOG00000052416 | LOC120094520 | R_norvegicus | Animals | mono_intergenic | H/ACA | | | intergenic |
| ENSRNOG00000052417 | LOC120102698 | R_norvegicus | Animals | mono_intergenic | H/ACA | | | intergenic |
| ENSRNOG00000052432 | LOC120096824 | R_norvegicus | Animals | mono_intergenic | H/ACA | | | intergenic |
| ENSRNOG00000052438 | LOC120093440 | R_norvegicus | Animals | mono_intergenic | H/ACA | | | intergenic |
| ENSRNOG00000052439 | Snora73bl1 | R_norvegicus | Animals | mono_intronic | H/ACA | ENSRNOG00000018645 | Rpsa | protein_coding |
| ENSRNOG00000052446 | LOC120103278 | R_norvegicus | Animals | mono_intergenic | H/ACA | | | intergenic |
| ENSRNOG00000052454 | LOC120102053 | R_norvegicus | Animals | mono_intronic | H/ACA | ENSRNOG00000007528 | Kcnh7 | protein_coding |
| ENSRNOG00000052462 | LOC120093456 | R_norvegicus | Animals | mono_intergenic | H/ACA | | | intergenic |
| ENSRNOG00000052476 | LOC120103321 | R_norvegicus | Animals | mono_intronic | H/ACA | ENSRNOG00000007894 | Mpdz | protein_coding |
| ENSRNOG00000052482 | LOC120096116 | R_norvegicus | Animals | mono_intergenic | H/ACA | | | intergenic |
| ENSRNOG00000052485 | LOC120095456 | R_norvegicus | Animals | mono_intergenic | H/ACA | | | intergenic |
| ENSRNOG00000052487 | Snord73b | R_norvegicus | Animals | mono_intronic | C/D | ENSRNOG00000011893 | Rps3a | protein_coding |
| ENSRNOG00000052517 | LOC120096378 | R_norvegicus | Animals | mono_intergenic | H/ACA | | | intergenic |
| ENSRNOG00000052528 | Rnu105c | R_norvegicus | Animals | mono_intronic | H/ACA | ENSRNOG00000015018 | Pdia3 | protein_coding |
| ENSRNOG00000052543 | LOC120096406 | R_norvegicus | Animals | mono_intergenic | H/ACA | | | intergenic |
| ENSRNOG00000052544 | LOC120096783 | R_norvegicus | Animals | mono_intronic | H/ACA | ENSRNOG00000065000 | Lrrc8c | protein_coding |
| ENSRNOG00000052548 | SNORA73 | R_norvegicus | Animals | mono_intronic | H/ACA | ENSRNOG00000027864 | Ppia | protein_coding |
| ENSRNOG00000052562 | LOC120095737 | R_norvegicus | Animals | mono_intergenic | H/ACA | | | intergenic |
| ENSRNOG00000052563 | LOC120102747 | R_norvegicus | Animals | mono_intronic | C/D | ENSRNOG00000012999 | Phb2 | protein_coding |
| ENSRNOG00000052573 | Snord17 | R_norvegicus | Animals | mono_intronic | C/D | ENSRNOG00000006077 | Snx5 | protein_coding |
| ENSRNOG00000052577 | Snora54 | R_norvegicus | Animals | mono_intronic | H/ACA | ENSRNOG00000020615 | Nap1l4 | protein_coding |
| ENSRNOG00000052580 | LOC120093956 | R_norvegicus | Animals | mono_intergenic | H/ACA | | | intergenic |
| ENSRNOG00000052607 | Snora12 | R_norvegicus | Animals | mono_intronic | H/ACA | ENSRNOG00000012763 | Cwf19l1 | protein_coding |
| ENSRNOG00000052611 | Snord43l1 | R_norvegicus | Animals | mono_exonic | C/D | ENSRNOG00000016896 | Rpl3 | protein_coding |
| ENSRNOG00000052616 | LOC120094468 | R_norvegicus | Animals | mono_intergenic | H/ACA | | | intergenic |
| ENSRNOG00000052617 | Snord67 | R_norvegicus | Animals | mono_intronic | C/D | ENSRNOG00000016067 | Ckap5 | protein_coding |
| ENSRNOG00000052635 | LOC120099439 | R_norvegicus | Animals | mono_intergenic | H/ACA | | | intergenic |
| ENSRNOG00000052644 | LOC120100362 | R_norvegicus | Animals | mono_intergenic | H/ACA | | | intergenic |
| ENSRNOG00000052658 | LOC120096811 | R_norvegicus | Animals | mono_intergenic | H/ACA | | | intergenic |
| ENSRNOG00000052660 | LOC120095394 | R_norvegicus | Animals | mono_intronic | C/D | ENSRNOG00000052620 | Rack1 | protein_coding |

| | | | | | | | | |
|---|---|---|---|---|---|---|---|---|
| ENSRNOG00000052672 | LOC120101257 | R_norvegicus | Animals | mono_intergenic | H/ACA | | | intergenic |
| ENSRNOG00000052679 | LOC120103323 | R_norvegicus | Animals | mono_intergenic | H/ACA | | | intergenic |
| ENSRNOG00000052692 | LOC120097227 | R_norvegicus | Animals | intergenic_cluster | H/ACA | | | intergenic |
| ENSRNOG00000052698 | LOC120099023 | R_norvegicus | Animals | mono_intergenic | H/ACA | | | intergenic |
| ENSRNOG00000052712 | LOC120100455 | R_norvegicus | Animals | mono_intergenic | H/ACA | | | intergenic |
| ENSRNOG00000052714 | LOC120095727 | R_norvegicus | Animals | mono_intronic | H/ACA | ENSRNOG00000001815 | Eif4a2 | protein_coding |
| ENSRNOG00000052715 | LOC120102726 | R_norvegicus | Animals | mono_intergenic | H/ACA | | | intergenic |
| ENSRNOG00000052723 | LOC120098390 | R_norvegicus | Animals | mono_intergenic | H/ACA | | | intergenic |
| ENSRNOG00000052741 | Snora21 | R_norvegicus | Animals | mono_intronic | H/ACA | ENSRNOG00000004107 | Rpl23 | protein_coding |
| ENSRNOG00000052744 | LOC120101122 | R_norvegicus | Animals | mono_intergenic | C/D | | | intergenic |
| ENSRNOG00000052747 | LOC120102720 | R_norvegicus | Animals | mono_intergenic | H/ACA | | | intergenic |
| ENSRNOG00000052748 | LOC120094881 | R_norvegicus | Animals | mono_intronic | H/ACA | ENSRNOG00000018273 | Ncl | protein_coding |
| ENSRNOG00000052763 | LOC120101256 | R_norvegicus | Animals | mono_intronic | H/ACA | ENSRNOG00000032630 | Mrps28 | protein_coding |
| ENSRNOG00000052780 | LOC120099067 | R_norvegicus | Animals | mono_intergenic | H/ACA | | | intergenic |
| ENSRNOG00000052786 | LOC120103335 | R_norvegicus | Animals | mono_intergenic | H/ACA | | | intergenic |
| ENSRNOG00000052788 | LOC120100357 | R_norvegicus | Animals | mono_intronic | H/ACA | ENSRNOG00000013160 | Sash1 | protein_coding |
| ENSRNOG00000052797 | LOC120094495 | R_norvegicus | Animals | mono_intergenic | H/ACA | | | intergenic |
| ENSRNOG00000052806 | LOC120094922 | R_norvegicus | Animals | intergenic_cluster | H/ACA | | | intergenic |
| ENSRNOG00000052815 | LOC120096379 | R_norvegicus | Animals | mono_intergenic | C/D | | | intergenic |
| ENSRNOG00000052816 | LOC120095445 | R_norvegicus | Animals | mono_intronic | H/ACA | ENSRNOG00000007377 | Slit3 | protein_coding |
| ENSRNOG00000052832 | LOC120100391 | R_norvegicus | Animals | mono_intronic | H/ACA | ENSRNOG00000014486 | Rfx3 | protein_coding |
| ENSRNOG00000052849 | LOC120102725 | R_norvegicus | Animals | mono_intergenic | H/ACA | | | intergenic |
| ENSRNOG00000052850 | LOC120094444 | R_norvegicus | Animals | mono_intergenic | H/ACA | | | intergenic |
| ENSRNOG00000052853 | LOC120098696 | R_norvegicus | Animals | mono_intergenic | H/ACA | | | intergenic |
| ENSRNOG00000052855 | LOC120097605 | R_norvegicus | Animals | mono_intergenic | H/ACA | | | intergenic |
| ENSRNOG00000052871 | LOC120100439 | R_norvegicus | Animals | mono_intergenic | H/ACA | | | intergenic |
| ENSRNOG00000052872 | LOC120095765 | R_norvegicus | Animals | mono_intergenic | H/ACA | | | intergenic |
| ENSRNOG00000052874 | LOC120101324 | R_norvegicus | Animals | mono_intergenic | H/ACA | | | intergenic |
| ENSRNOG00000052875 | LOC120094932 | R_norvegicus | Animals | mono_intergenic | H/ACA | | | intergenic |
| ENSRNOG00000052881 | LOC120101345 | R_norvegicus | Animals | mono_intergenic | H/ACA | | | intergenic |
| ENSRNOG00000052888 | LOC120098376 | R_norvegicus | Animals | mono_intergenic | H/ACA | | | intergenic |
| ENSRNOG00000052892 | LOC120101284 | R_norvegicus | Animals | mono_intergenic | H/ACA | | | intergenic |
| ENSRNOG00000052900 | LOC120097617 | R_norvegicus | Animals | mono_intergenic | H/ACA | | | intergenic |
| ENSRNOG00000052933 | LOC120098705 | R_norvegicus | Animals | mono_intronic | H/ACA | ENSRNOG00000011575 | Adamts18 | protein_coding |
| ENSRNOG00000052937 | LOC120100460 | R_norvegicus | Animals | mono_intergenic | H/ACA | | | intergenic |
| ENSRNOG00000052983 | LOC120094440 | R_norvegicus | Animals | mono_intronic | C/D | ENSRNOG00000009378 | Rpl4 | protein_coding |
| ENSRNOG00000052988 | LOC120094499 | R_norvegicus | Animals | mono_intergenic | H/ACA | | | intergenic |
| ENSRNOG00000052997 | LOC120094890 | R_norvegicus | Animals | mono_intergenic | H/ACA | | | intergenic |
| ENSRNOG00000053003 | LOC120094946 | R_norvegicus | Animals | mono_intronic | C/D | ENSRNOG00000016486 | Nop58 | protein_coding |
| ENSRNOG00000053012 | LOC120100427 | R_norvegicus | Animals | mono_intergenic | H/ACA | | | intergenic |
| ENSRNOG00000053016 | LOC120096381 | R_norvegicus | Animals | mono_intergenic | C/D | | | intergenic |

| | | | | | | | | |
|---|---|---|---|---|---|---|---|---|
| ENSRNOG00000053018 | LOC120095388 | R_norvegicus | Animals | mono_intergenic | H/ACA | | | intergenic |
| ENSRNOG00000053020 | LOC120093462 | R_norvegicus | Animals | mono_intergenic | H/ACA | | | intergenic |
| ENSRNOG00000053065 | LOC120097210 | R_norvegicus | Animals | mono_intergenic | H/ACA | | | intergenic |
| ENSRNOG00000053071 | LOC120103254 | R_norvegicus | Animals | mono_intergenic | H/ACA | | | intergenic |
| ENSRNOG00000053082 | LOC120095429 | R_norvegicus | Animals | mono_intergenic | H/ACA | | | intergenic |
| ENSRNOG00000053089 | LOC120100352 | R_norvegicus | Animals | mono_intergenic | H/ACA | | | intergenic |
| ENSRNOG00000053094 | LOC120099457 | R_norvegicus | Animals | mono_intergenic | H/ACA | | | intergenic |
| ENSRNOG00000053107 | LOC120093426 | R_norvegicus | Animals | mono_intergenic | H/ACA | | | intergenic |
| ENSRNOG00000053111 | LOC120102740 | R_norvegicus | Animals | mono_intergenic | H/ACA | | | intergenic |
| ENSRNOG00000053117 | LOC120099504 | R_norvegicus | Animals | mono_intergenic | H/ACA | | | intergenic |
| ENSRNOG00000053119 | LOC120096807 | R_norvegicus | Animals | mono_intergenic | H/ACA | | | intergenic |
| ENSRNOG00000053121 | LOC120093472 | R_norvegicus | Animals | mono_intergenic | H/ACA | | | intergenic |
| ENSRNOG00000053138 | LOC120094902 | R_norvegicus | Animals | mono_intergenic | H/ACA | | | intergenic |
| ENSRNOG00000053139 | AABR07048648.1 | R_norvegicus | Animals | mono_intergenic | H/ACA | | | intergenic |
| ENSRNOG00000053149 | LOC120102689 | R_norvegicus | Animals | mono_intergenic | H/ACA | | | intergenic |
| ENSRNOG00000053152 | LOC120095389 | R_norvegicus | Animals | mono_intergenic | H/ACA | | | intergenic |
| ENSRNOG00000053158 | LOC120100341 | R_norvegicus | Animals | mono_intronic | H/ACA | ENSRNOG00000023274 | Lrrc28 | protein_coding |
| ENSRNOG00000053163 | LOC120103261 | R_norvegicus | Animals | mono_intronic | H/ACA | ENSRNOG00000071131 | LOC120102992 | non_coding |
| ENSRNOG00000053177 | LOC120102699 | R_norvegicus | Animals | mono_intergenic | H/ACA | | | intergenic |
| ENSRNOG00000053189 | LOC120103316 | R_norvegicus | Animals | mono_intergenic | H/ACA | | | intergenic |
| ENSRNOG00000053191 | LOC120099486 | R_norvegicus | Animals | mono_intergenic | H/ACA | | | intergenic |
| ENSRNOG00000053202 | LOC120100337 | R_norvegicus | Animals | mono_intergenic | C/D | | | intergenic |
| ENSRNOG00000053212 | LOC120102015 | R_norvegicus | Animals | mono_intronic | H/ACA | ENSRNOG00000005335 | Galnt13 | protein_coding |
| ENSRNOG00000053214 | LOC120098008 | R_norvegicus | Animals | mono_intergenic | H/ACA | | | intergenic |
| ENSRNOG00000053218 | LOC120096388 | R_norvegicus | Animals | mono_intronic | C/D | ENSRNOG00000003614 | Mgat5 | protein_coding |
| ENSRNOG00000053219 | LOC120103305 | R_norvegicus | Animals | mono_intergenic | H/ACA | | | intergenic |
| ENSRNOG00000053220 | LOC120101244 | R_norvegicus | Animals | mono_intergenic | H/ACA | | | intergenic |
| ENSRNOG00000053237 | LOC120102672 | R_norvegicus | Animals | mono_intergenic | H/ACA | | | intergenic |
| ENSRNOG00000053243 | LOC120101242 | R_norvegicus | Animals | mono_intergenic | H/ACA | | | intergenic |
| ENSRNOG00000053246 | LOC120095764 | R_norvegicus | Animals | mono_intergenic | H/ACA | | | intergenic |
| ENSRNOG00000053261 | LOC120099461 | R_norvegicus | Animals | mono_intergenic | H/ACA | | | intergenic |
| ENSRNOG00000053278 | LOC120103350 | R_norvegicus | Animals | mono_intergenic | H/ACA | | | intergenic |
| ENSRNOG00000053279 | LOC120100359 | R_norvegicus | Animals | mono_intergenic | H/ACA | | | intergenic |
| ENSRNOG00000053280 | LOC120094934 | R_norvegicus | Animals | mono_intergenic | H/ACA | | | intergenic |
| ENSRNOG00000053295 | LOC120098405 | R_norvegicus | Animals | mono_intergenic | H/ACA | | | intergenic |
| ENSRNOG00000053310 | LOC120102628 | R_norvegicus | Animals | mono_intergenic | H/ACA | | | intergenic |
| ENSRNOG00000053313 | LOC120095421 | R_norvegicus | Animals | mono_intergenic | H/ACA | | | intergenic |
| ENSRNOG00000053318 | LOC120102074 | R_norvegicus | Animals | mono_intergenic | H/ACA | | | intergenic |
| ENSRNOG00000053352 | LOC120096416 | R_norvegicus | Animals | mono_intergenic | H/ACA | | | intergenic |
| ENSRNOG00000053353 | Snord7 | R_norvegicus | Animals | mono_intergenic | C/D | | | intergenic |
| ENSRNOG00000053385 | LOC120096420 | R_norvegicus | Animals | mono_intergenic | H/ACA | | | intergenic |

| | | | | | | | | |
|---|---|---|---|---|---|---|---|---|
| ENSRNOG00000053390 | LOC120102678 | R_norvegicus | Animals | mono_intergenic | H/ACA | | | intergenic |
| ENSRNOG00000053398 | LOC120093428 | R_norvegicus | Animals | mono_intergenic | H/ACA | | | intergenic |
| ENSRNOG00000053403 | LOC120094488 | R_norvegicus | Animals | mono_intergenic | H/ACA | | | intergenic |
| ENSRNOG00000053414 | LOC120102674 | R_norvegicus | Animals | mono_intergenic | H/ACA | | | intergenic |
| ENSRNOG00000053421 | LOC120096117 | R_norvegicus | Animals | mono_intergenic | H/ACA | | | intergenic |
| ENSRNOG00000053426 | LOC120098695 | R_norvegicus | Animals | mono_intergenic | H/ACA | | | intergenic |
| ENSRNOG00000053436 | LOC120102016 | R_norvegicus | Animals | mono_intergenic | H/ACA | | | intergenic |
| ENSRNOG00000053438 | LOC120102637 | R_norvegicus | Animals | mono_intronic | H/ACA | ENSRNOG00000010746 | Rpl32 | protein_coding |
| ENSRNOG00000053462 | LOC120098679 | R_norvegicus | Animals | mono_intronic | H/ACA | ENSRNOG00000019980 | Tomm20 | protein_coding |
| ENSRNOG00000053464 | LOC120100418 | R_norvegicus | Animals | mono_intergenic | H/ACA | | | intergenic |
| ENSRNOG00000053480 | LOC120102017 | R_norvegicus | Animals | intergenic_cluster | H/ACA | | | intergenic |
| ENSRNOG00000053482 | LOC120094023 | R_norvegicus | Animals | mono_intergenic | H/ACA | | | intergenic |
| ENSRNOG00000053492 | LOC120103313 | R_norvegicus | Animals | mono_intergenic | H/ACA | | | intergenic |
| ENSRNOG00000053495 | LOC120097221 | R_norvegicus | Animals | mono_intergenic | H/ACA | | | intergenic |
| ENSRNOG00000053499 | LOC120101349 | R_norvegicus | Animals | mono_intergenic | H/ACA | | | intergenic |
| ENSRNOG00000053500 | LOC120103270 | R_norvegicus | Animals | mono_intronic | H/ACA | ENSRNOG00000006833 | Rb1cc1 | protein_coding |
| ENSRNOG00000053512 | LOC120100487 | R_norvegicus | Animals | mono_intergenic | H/ACA | | | intergenic |
| ENSRNOG00000053533 | LOC120100208 | R_norvegicus | Animals | mono_intronic | H/ACA | ENSRNOG00000036603 | Ifit1bl | protein_coding |
| ENSRNOG00000053534 | LOC120095472 | R_norvegicus | Animals | mono_intergenic | H/ACA | | | intergenic |
| ENSRNOG00000053545 | LOC120095410 | R_norvegicus | Animals | mono_intergenic | H/ACA | | | intergenic |
| ENSRNOG00000053555 | LOC120099066 | R_norvegicus | Animals | mono_intergenic | H/ACA | | | intergenic |
| ENSRNOG00000053556 | LOC120101305 | R_norvegicus | Animals | mono_intergenic | H/ACA | | | intergenic |
| ENSRNOG00000053558 | LOC120098005 | R_norvegicus | Animals | mono_intergenic | H/ACA | | | intergenic |
| ENSRNOG00000053569 | LOC120095413 | R_norvegicus | Animals | mono_intergenic | H/ACA | | | intergenic |
| ENSRNOG00000053584 | LOC120099505 | R_norvegicus | Animals | mono_intergenic | H/ACA | | | intergenic |
| ENSRNOG00000053586 | Snord19 | R_norvegicus | Animals | mono_intronic | C/D | ENSRNOG00000028461 | Gnl3 | protein_coding |
| ENSRNOG00000053594 | LOC120094893 | R_norvegicus | Animals | mono_intergenic | H/ACA | | | intergenic |
| ENSRNOG00000053611 | LOC120103297 | R_norvegicus | Animals | mono_intergenic | H/ACA | | | intergenic |
| ENSRNOG00000053612 | LOC120099073 | R_norvegicus | Animals | mono_intergenic | H/ACA | | | intergenic |
| ENSRNOG00000053621 | Snord2 | R_norvegicus | Animals | mono_intronic | C/D | ENSRNOG00000001815 | Eif4a2 | protein_coding |
| ENSRNOG00000053627 | LOC120097977 | R_norvegicus | Animals | mono_intergenic | H/ACA | | | intergenic |
| ENSRNOG00000053629 | LOC120095749 | R_norvegicus | Animals | mono_intergenic | H/ACA | | | intergenic |
| ENSRNOG00000053637 | LOC120096114 | R_norvegicus | Animals | mono_intergenic | H/ACA | | | intergenic |
| ENSRNOG00000053651 | LOC120101251 | R_norvegicus | Animals | mono_intronic | H/ACA | ENSRNOG00000019090 | Cct3 | protein_coding |
| ENSRNOG00000053670 | LOC120098404 | R_norvegicus | Animals | mono_intergenic | H/ACA | | | intergenic |
| ENSRNOG00000053692 | Snora3l1 | R_norvegicus | Animals | mono_intronic | H/ACA | ENSRNOG00000022844 | Pdzrn4 | protein_coding |
| ENSRNOG00000053698 | LOC120102609 | R_norvegicus | Animals | mono_intergenic | H/ACA | | | intergenic |
| ENSRNOG00000053707 | LOC120101300 | R_norvegicus | Animals | mono_intergenic | H/ACA | | | intergenic |
| ENSRNOG00000053713 | LOC120094925 | R_norvegicus | Animals | mono_intergenic | H/ACA | | | intergenic |
| ENSRNOG00000053726 | LOC120100335 | R_norvegicus | Animals | mono_intergenic | C/D | | | intergenic |
| ENSRNOG00000053750 | LOC120094921 | R_norvegicus | Animals | mono_intergenic | H/ACA | | | intergenic |

| | | | | | | | | |
|---|---|---|---|---|---|---|---|---|
| ENSRNOG00000053757 | Snora51 | R_norvegicus | Animals | mono_intronic | H/ACA | ENSRNOG00000007128 | Nop56 | protein_coding |
| ENSRNOG00000053761 | Snord113l4 | R_norvegicus | Animals | intergenic_cluster | C/D | | | intergenic |
| ENSRNOG00000053764 | Snord33 | R_norvegicus | Animals | mono_intronic | C/D | ENSRNOG00000020618 | Rpl13a | protein_coding |
| ENSRNOG00000053770 | LOC120096108 | R_norvegicus | Animals | mono_intronic | H/ACA | ENSRNOG00000000923 | Cct6a | protein_coding |
| ENSRNOG00000053777 | LOC120101346 | R_norvegicus | Animals | mono_intronic | H/ACA | ENSRNOG00000026392 | Cdh12 | protein_coding |
| ENSRNOG00000053784 | LOC120094494 | R_norvegicus | Animals | mono_intergenic | H/ACA | | | intergenic |
| ENSRNOG00000053785 | LOC120100363 | R_norvegicus | Animals | mono_intergenic | H/ACA | | | intergenic |
| ENSRNOG00000053795 | Snord45a | R_norvegicus | Animals | mono_intronic | C/D | ENSRNOG00000009992 | Rabggtb | protein_coding |
| ENSRNOG00000053796 | LOC120095492 | R_norvegicus | Animals | intronic_cluster | C/D | ENSRNOG00000037418 | Inca1 | protein_coding |
| ENSRNOG00000053801 | LOC120093962 | R_norvegicus | Animals | mono_intergenic | H/ACA | | | intergenic |
| ENSRNOG00000053830 | LOC120095430 | R_norvegicus | Animals | mono_intronic | H/ACA | ENSRNOG00000010038 | Psmc5 | protein_coding |
| ENSRNOG00000053838 | LOC120100288 | R_norvegicus | Animals | intergenic_cluster | C/D | | | intergenic |
| ENSRNOG00000053854 | LOC120093391 | R_norvegicus | Animals | mono_intergenic | C/D | | | intergenic |
| ENSRNOG00000053871 | LOC120100456 | R_norvegicus | Animals | mono_intergenic | H/ACA | | | intergenic |
| ENSRNOG00000053892 | LOC120101308 | R_norvegicus | Animals | mono_intergenic | H/ACA | | | intergenic |
| ENSRNOG00000053898 | LOC120101240 | R_norvegicus | Animals | mono_intergenic | H/ACA | | | intergenic |
| ENSRNOG00000053907 | LOC120093463 | R_norvegicus | Animals | mono_intergenic | H/ACA | | | intergenic |
| ENSRNOG00000053912 | LOC120102681 | R_norvegicus | Animals | mono_intergenic | H/ACA | | | intergenic |
| ENSRNOG00000053921 | LOC120093990 | R_norvegicus | Animals | mono_intronic | H/ACA | ENSRNOG00000021681 | Eea1 | protein_coding |
| ENSRNOG00000053926 | LOC120098382 | R_norvegicus | Animals | mono_intergenic | H/ACA | | | intergenic |
| ENSRNOG00000053933 | LOC120098000 | R_norvegicus | Animals | mono_intergenic | H/ACA | | | intergenic |
| ENSRNOG00000053941 | LOC120097215 | R_norvegicus | Animals | mono_intergenic | C/D | | | intergenic |
| ENSRNOG00000053947 | LOC120094936 | R_norvegicus | Animals | mono_intergenic | H/ACA | | | intergenic |
| ENSRNOG00000053958 | LOC120099019 | R_norvegicus | Animals | mono_intergenic | H/ACA | | | intergenic |
| ENSRNOG00000053973 | Snord121a | R_norvegicus | Animals | mono_intronic | C/D | ENSRNOG00000052087 | Ubap2 | protein_coding |
| ENSRNOG00000053976 | LOC120097233 | R_norvegicus | Animals | mono_intergenic | H/ACA | | | intergenic |
| ENSRNOG00000053988 | Snord52 | R_norvegicus | Animals | mono_intergenic | C/D | | | intergenic |
| ENSRNOG00000053994 | LOC120100425 | R_norvegicus | Animals | mono_intergenic | H/ACA | | | intergenic |
| ENSRNOG00000054004 | LOC120099018 | R_norvegicus | Animals | mono_intergenic | H/ACA | | | intergenic |
| ENSRNOG00000054019 | LOC120093944 | R_norvegicus | Animals | mono_intronic | H/ACA | ENSRNOG00000068679 | Rpl30 | non_coding |
| ENSRNOG00000054036 | LOC120103309 | R_norvegicus | Animals | mono_intergenic | H/ACA | | | intergenic |
| ENSRNOG00000054041 | LOC120097214 | R_norvegicus | Animals | mono_intergenic | H/ACA | | | intergenic |
| ENSRNOG00000054043 | LOC120097613 | R_norvegicus | Animals | mono_intergenic | H/ACA | | | intergenic |
| ENSRNOG00000054044 | LOC120101347 | R_norvegicus | Animals | mono_intergenic | H/ACA | | | intergenic |
| ENSRNOG00000054081 | Snord12 | R_norvegicus | Animals | intergenic_cluster | C/D | | | intergenic |
| ENSRNOG00000054091 | Snora38 | R_norvegicus | Animals | mono_intronic | H/ACA | ENSRNOG00000000852 | Prrc2a | protein_coding |
| ENSRNOG00000054092 | LOC120098393 | R_norvegicus | Animals | mono_intergenic | H/ACA | | | intergenic |
| ENSRNOG00000054095 | LOC120096418 | R_norvegicus | Animals | mono_intergenic | H/ACA | | | intergenic |
| ENSRNOG00000054099 | LOC120099071 | R_norvegicus | Animals | mono_intergenic | H/ACA | | | intergenic |
| ENSRNOG00000054102 | LOC120100468 | R_norvegicus | Animals | mono_intergenic | H/ACA | | | intergenic |
| ENSRNOG00000054115 | LOC120101231 | R_norvegicus | Animals | mono_intergenic | H/ACA | | | intergenic |

| | | | | | | | | |
|---|---|---|---|---|---|---|---|---|
| ENSRNOG00000054116 | LOC120102052 | R_norvegicus | Animals | mono_intergenic | H/ACA | | | intergenic |
| ENSRNOG00000054117 | LOC120103329 | R_norvegicus | Animals | mono_intronic | H/ACA | ENSRNOG00000005641 | Fbxl4 | protein_coding |
| ENSRNOG00000054161 | LOC120103328 | R_norvegicus | Animals | mono_intergenic | H/ACA | | | intergenic |
| ENSRNOG00000054178 | LOC120102065 | R_norvegicus | Animals | mono_intergenic | H/ACA | | | intergenic |
| ENSRNOG00000054211 | LOC120102671 | R_norvegicus | Animals | mono_intergenic | H/ACA | | | intergenic |
| ENSRNOG00000054237 | LOC120096387 | R_norvegicus | Animals | mono_intronic | H/ACA | ENSRNOG00000003953 | Rab3gap1 | protein_coding |
| ENSRNOG00000054243 | LOC120095452 | R_norvegicus | Animals | mono_intergenic | H/ACA | | | intergenic |
| ENSRNOG00000054258 | LOC120101226 | R_norvegicus | Animals | mono_intergenic | C/D | | | intergenic |
| ENSRNOG00000054310 | LOC120098677 | R_norvegicus | Animals | mono_intronic | C/D | ENSRNOG00000015335 | Rpl13 | protein_coding |
| ENSRNOG00000054315 | Snord27 | R_norvegicus | Animals | mono_intronic | C/D | ENSRNOG00000067892 | LOC100912041 | non_coding |
| ENSRNOG00000054318 | LOC120101236 | R_norvegicus | Animals | mono_intergenic | C/D | | | intergenic |
| ENSRNOG00000054323 | LOC120094512 | R_norvegicus | Animals | mono_intergenic | H/ACA | | | intergenic |
| ENSRNOG00000054324 | Snord55 | R_norvegicus | Animals | mono_exonic | C/D | ENSRNOG00000054626 | Rps8 | protein_coding |
| ENSRNOG00000054326 | Snord88b | R_norvegicus | Animals | mono_intronic | C/D | ENSRNOG00000019132 | Fv1 | protein_coding |
| ENSRNOG00000054339 | LOC120099441 | R_norvegicus | Animals | mono_intronic | H/ACA | ENSRNOG00000056765 | Rpl10 | protein_coding |
| ENSRNOG00000054341 | LOC120102736 | R_norvegicus | Animals | mono_intergenic | H/ACA | | | intergenic |
| ENSRNOG00000054347 | LOC120096384 | R_norvegicus | Animals | intergenic_cluster | C/D | | | intergenic |
| ENSRNOG00000054350 | LOC120101287 | R_norvegicus | Animals | mono_intergenic | H/ACA | | | intergenic |
| ENSRNOG00000054357 | LOC120094496 | R_norvegicus | Animals | mono_intergenic | H/ACA | | | intergenic |
| ENSRNOG00000054365 | LOC120103324 | R_norvegicus | Animals | mono_intergenic | H/ACA | | | intergenic |
| ENSRNOG00000054369 | LOC120101306 | R_norvegicus | Animals | mono_intergenic | H/ACA | | | intergenic |
| ENSRNOG00000054381 | AC136161.1 | R_norvegicus | Animals | mono_intergenic | H/ACA | | | intergenic |
| ENSRNOG00000054406 | LOC120097975 | R_norvegicus | Animals | mono_intronic | H/ACA | ENSRNOG00000018381 | Cdkal1 | protein_coding |
| ENSRNOG00000054422 | LOC120103265 | R_norvegicus | Animals | mono_intronic | H/ACA | ENSRNOG00000016334 | Ptbp3 | protein_coding |
| ENSRNOG00000054434 | LOC120098674 | R_norvegicus | Animals | mono_intergenic | H/ACA | | | intergenic |
| ENSRNOG00000054443 | LOC120099027 | R_norvegicus | Animals | mono_intergenic | H/ACA | | | intergenic |
| ENSRNOG00000054449 | LOC120096395 | R_norvegicus | Animals | mono_intergenic | H/ACA | | | intergenic |
| ENSRNOG00000054451 | LOC120098387 | R_norvegicus | Animals | mono_intergenic | H/ACA | | | intergenic |
| ENSRNOG00000054452 | LOC120100374 | R_norvegicus | Animals | mono_intergenic | H/ACA | | | intergenic |
| ENSRNOG00000054457 | LOC120094458 | R_norvegicus | Animals | mono_intronic | H/ACA | ENSRNOG00000010921 | Taf1d | protein_coding |
| ENSRNOG00000054517 | Snora33 | R_norvegicus | Animals | mono_intronic | H/ACA | ENSRNOG00000016411 | Rps12 | protein_coding |
| ENSRNOG00000054518 | LOC120096779 | R_norvegicus | Animals | mono_intronic | H/ACA | ENSRNOG00000070345 | | non_coding |
| ENSRNOG00000054528 | LOC120103288 | R_norvegicus | Animals | mono_intergenic | H/ACA | | | intergenic |
| ENSRNOG00000054530 | LOC120102638 | R_norvegicus | Animals | mono_intergenic | H/ACA | | | intergenic |
| ENSRNOG00000054533 | LOC120100346 | R_norvegicus | Animals | mono_intergenic | H/ACA | | | intergenic |
| ENSRNOG00000054541 | LOC120101326 | R_norvegicus | Animals | mono_intergenic | H/ACA | | | intergenic |
| ENSRNOG00000054556 | LOC120102048 | R_norvegicus | Animals | mono_intergenic | H/ACA | | | intergenic |
| ENSRNOG00000054559 | Snord61 | R_norvegicus | Animals | mono_intronic | C/D | ENSRNOG00000000866 | Rbmx | protein_coding |
| ENSRNOG00000054583 | LOC120101291 | R_norvegicus | Animals | mono_intergenic | H/ACA | | | intergenic |
| ENSRNOG00000054601 | LOC120097598 | R_norvegicus | Animals | mono_intergenic | H/ACA | | | intergenic |
| ENSRNOG00000054606 | LOC120096412 | R_norvegicus | Animals | mono_intergenic | H/ACA | | | intergenic |

| | | | | | | | | |
|---|---|---|---|---|---|---|---|---|
| ENSRNOG00000054607 | Snord56 | R_norvegicus | Animals | mono_intronic | C/D | ENSRNOG00000007128 | Nop56 | protein_coding |
| ENSRNOG00000054619 | LOC120095453 | R_norvegicus | Animals | mono_intergenic | H/ACA | | | intergenic |
| ENSRNOG00000054621 | LOC120093458 | R_norvegicus | Animals | mono_intergenic | H/ACA | | | intergenic |
| ENSRNOG00000054644 | LOC120099455 | R_norvegicus | Animals | mono_intergenic | H/ACA | | | intergenic |
| ENSRNOG00000054649 | LOC120095416 | R_norvegicus | Animals | mono_exonic | H/ACA | ENSRNOG00000070910 | | non_coding |
| ENSRNOG00000054654 | LOC120094933 | R_norvegicus | Animals | mono_intergenic | H/ACA | | | intergenic |
| ENSRNOG00000054659 | LOC120102645 | R_norvegicus | Animals | mono_intronic | H/ACA | ENSRNOG00000012392 | Dnajc2 | protein_coding |
| ENSRNOG00000054660 | LOC120095434 | R_norvegicus | Animals | mono_intronic | H/ACA | ENSRNOG00000046946 | Ptchd3 | protein_coding |
| ENSRNOG00000054664 | LOC120094509 | R_norvegicus | Animals | mono_intergenic | H/ACA | | | intergenic |
| ENSRNOG00000054676 | LOC120098732 | R_norvegicus | Animals | mono_intergenic | C/D | | | intergenic |
| ENSRNOG00000054684 | LOC120103302 | R_norvegicus | Animals | mono_intergenic | H/ACA | | | intergenic |
| ENSRNOG00000054687 | LOC120096127 | R_norvegicus | Animals | mono_intergenic | H/ACA | | | intergenic |
| ENSRNOG00000054705 | LOC120093446 | R_norvegicus | Animals | mono_intergenic | H/ACA | | | intergenic |
| ENSRNOG00000054717 | LOC120097601 | R_norvegicus | Animals | mono_intergenic | H/ACA | | | intergenic |
| ENSRNOG00000054720 | LOC120098703 | R_norvegicus | Animals | mono_intergenic | H/ACA | | | intergenic |
| ENSRNOG00000054735 | LOC120097252 | R_norvegicus | Animals | mono_intergenic | H/ACA | | | intergenic |
| ENSRNOG00000054739 | Snord110 | R_norvegicus | Animals | mono_intronic | C/D | ENSRNOG00000007128 | Nop56 | protein_coding |
| ENSRNOG00000054741 | LOC120102716 | R_norvegicus | Animals | mono_intergenic | H/ACA | | | intergenic |
| ENSRNOG00000054746 | Snora49 | R_norvegicus | Animals | mono_intronic | H/ACA | ENSRNOG00000037483 | Ep400 | protein_coding |
| ENSRNOG00000054766 | LOC120097257 | R_norvegicus | Animals | mono_intergenic | H/ACA | | | intergenic |
| ENSRNOG00000054767 | LOC252890 | R_norvegicus | Animals | mono_intronic | C/D | ENSRNOG00000030237 | Cox7c | protein_coding |
| ENSRNOG00000054771 | LOC120100336 | R_norvegicus | Animals | mono_intergenic | C/D | | | intergenic |
| ENSRNOG00000054789 | LOC120102022 | R_norvegicus | Animals | mono_intergenic | H/ACA | | | intergenic |
| ENSRNOG00000054830 | LOC120093438 | R_norvegicus | Animals | mono_intergenic | H/ACA | | | intergenic |
| ENSRNOG00000054837 | LOC120094502 | R_norvegicus | Animals | mono_intergenic | H/ACA | | | intergenic |
| ENSRNOG00000054845 | LOC120100446 | R_norvegicus | Animals | mono_intergenic | H/ACA | | | intergenic |
| ENSRNOG00000054858 | LOC120098399 | R_norvegicus | Animals | mono_intergenic | H/ACA | | | intergenic |
| ENSRNOG00000054864 | LOC120101365 | R_norvegicus | Animals | mono_intergenic | C/D | | | intergenic |
| ENSRNOG00000054868 | LOC120099465 | R_norvegicus | Animals | mono_intergenic | H/ACA | | | intergenic |
| ENSRNOG00000054874 | LOC120097273 | R_norvegicus | Animals | mono_intronic | H/ACA | ENSRNOG00000008989 | Cryl1 | protein_coding |
| ENSRNOG00000054884 | LOC120102630 | R_norvegicus | Animals | mono_intronic | H/ACA | ENSRNOG00000056462 | Tmf1 | protein_coding |
| ENSRNOG00000054892 | Snord113l1 | R_norvegicus | Animals | mono_intergenic | C/D | | | intergenic |
| ENSRNOG00000054906 | LOC120100424 | R_norvegicus | Animals | mono_intergenic | H/ACA | | | intergenic |
| ENSRNOG00000054911 | LOC120094518 | R_norvegicus | Animals | mono_intergenic | H/ACA | | | intergenic |
| ENSRNOG00000054921 | LOC120100438 | R_norvegicus | Animals | mono_intronic | H/ACA | ENSRNOG00000013202 | Akap7 | protein_coding |
| ENSRNOG00000054933 | LOC120098699 | R_norvegicus | Animals | mono_intergenic | H/ACA | | | intergenic |
| ENSRNOG00000054970 | Snord53l1 | R_norvegicus | Animals | mono_intronic | C/D | ENSRNOG00000026316 | Wdr43 | protein_coding |
| ENSRNOG00000054973 | Snora65 | R_norvegicus | Animals | mono_intronic | H/ACA | ENSRNOG00000016220 | Rpl12 | protein_coding |
| ENSRNOG00000054981 | LOC120098694 | R_norvegicus | Animals | mono_intergenic | H/ACA | | | intergenic |
| ENSRNOG00000054985 | LOC120093468 | R_norvegicus | Animals | mono_intergenic | H/ACA | | | intergenic |
| ENSRNOG00000054994 | LOC120093968 | R_norvegicus | Animals | mono_intergenic | H/ACA | | | intergenic |

| | | | | | | | | |
|---|---|---|---|---|---|---|---|---|
| ENSRNOG00000054998 | LOC120103272 | R_norvegicus | Animals | mono_intronic | H/ACA | ENSRNOG00000006327 | Rcc2 | protein_coding |
| ENSRNOG00000055001 | Snora3 | R_norvegicus | Animals | mono_intergenic | H/ACA | | | intergenic |
| ENSRNOG00000055005 | LOC120103358 | R_norvegicus | Animals | mono_intronic | C/D | ENSRNOG00000052087 | Ubap2 | protein_coding |
| ENSRNOG00000055018 | LOC120103351 | R_norvegicus | Animals | mono_intergenic | H/ACA | | | intergenic |
| ENSRNOG00000055019 | LOC120094944 | R_norvegicus | Animals | mono_intronic | C/D | ENSRNOG00000067231 | | non_coding |
| ENSRNOG00000055027 | Snord83 | R_norvegicus | Animals | mono_intronic | C/D | ENSRNOG00000000841 | Ddx39b | protein_coding |
| ENSRNOG00000055065 | LOC120102730 | R_norvegicus | Animals | mono_intergenic | H/ACA | | | intergenic |
| ENSRNOG00000055077 | LOC120094915 | R_norvegicus | Animals | mono_intergenic | H/ACA | | | intergenic |
| ENSRNOG00000055084 | LOC120101230 | R_norvegicus | Animals | mono_intronic | H/ACA | ENSRNOG00000065234 | | protein_coding |
| ENSRNOG00000055086 | LOC120103320 | R_norvegicus | Animals | mono_intergenic | H/ACA | | | intergenic |
| ENSRNOG00000055087 | LOC120098686 | R_norvegicus | Animals | mono_intergenic | H/ACA | | | intergenic |
| ENSRNOG00000055100 | LOC120093967 | R_norvegicus | Animals | mono_intronic | H/ACA | ENSRNOG00000027468 | Slc6a15 | protein_coding |
| ENSRNOG00000055101 | LOC120097242 | R_norvegicus | Animals | mono_intergenic | H/ACA | | | intergenic |
| ENSRNOG00000055104 | LOC120102047 | R_norvegicus | Animals | mono_intergenic | H/ACA | | | intergenic |
| ENSRNOG00000055125 | LOC120095455 | R_norvegicus | Animals | mono_intronic | H/ACA | ENSRNOG00000003781 | Atp10b | protein_coding |
| ENSRNOG00000055133 | LOC120100447 | R_norvegicus | Animals | mono_intergenic | H/ACA | | | intergenic |
| ENSRNOG00000055151 | LOC120094443 | R_norvegicus | Animals | mono_intergenic | C/D | | | intergenic |
| ENSRNOG00000055161 | LOC120100394 | R_norvegicus | Animals | intergenic_cluster | C/D | | | intergenic |
| ENSRNOG00000055163 | LOC120096407 | R_norvegicus | Animals | mono_intergenic | H/ACA | | | intergenic |
| ENSRNOG00000055165 | LOC120102070 | R_norvegicus | Animals | mono_intergenic | H/ACA | | | intergenic |
| ENSRNOG00000055167 | LOC120093419 | R_norvegicus | Animals | mono_intergenic | H/ACA | | | intergenic |
| ENSRNOG00000055168 | LOC120102092 | R_norvegicus | Animals | mono_intergenic | H/ACA | | | intergenic |
| ENSRNOG00000055172 | LOC120094503 | R_norvegicus | Animals | mono_intergenic | H/ACA | | | intergenic |
| ENSRNOG00000055177 | LOC120101282 | R_norvegicus | Animals | mono_intergenic | H/ACA | | | intergenic |
| ENSRNOG00000055183 | LOC120093988 | R_norvegicus | Animals | mono_intronic | H/ACA | ENSRNOG00000070536 | | non_coding |
| ENSRNOG00000055195 | LOC120098690 | R_norvegicus | Animals | mono_intergenic | H/ACA | | | intergenic |
| ENSRNOG00000055199 | LOC120094513 | R_norvegicus | Animals | mono_intergenic | H/ACA | | | intergenic |
| ENSRNOG00000055214 | LOC120093978 | R_norvegicus | Animals | mono_intergenic | H/ACA | | | intergenic |
| ENSRNOG00000055217 | Snora80l1 | R_norvegicus | Animals | mono_intronic | H/ACA | ENSRNOG00000005424 | Odc1 | protein_coding |
| ENSRNOG00000055223 | LOC120100474 | R_norvegicus | Animals | mono_intergenic | C/D | | | intergenic |
| ENSRNOG00000055237 | LOC120093952 | R_norvegicus | Animals | mono_intergenic | H/ACA | | | intergenic |
| ENSRNOG00000055243 | LOC120102024 | R_norvegicus | Animals | mono_intergenic | H/ACA | | | intergenic |
| ENSRNOG00000055249 | LOC120102643 | R_norvegicus | Animals | mono_intergenic | H/ACA | | | intergenic |
| ENSRNOG00000055273 | LOC120094001 | R_norvegicus | Animals | mono_intergenic | H/ACA | | | intergenic |
| ENSRNOG00000055274 | LOC120093445 | R_norvegicus | Animals | mono_intergenic | H/ACA | | | intergenic |
| ENSRNOG00000055283 | LOC120096118 | R_norvegicus | Animals | mono_intronic | H/ACA | ENSRNOG00000001123 | Spring1 | protein_coding |
| ENSRNOG00000055303 | LOC120102063 | R_norvegicus | Animals | mono_intergenic | H/ACA | | | intergenic |
| ENSRNOG00000055349 | LOC120098397 | R_norvegicus | Animals | mono_intergenic | H/ACA | | | intergenic |
| ENSRNOG00000055351 | LOC120100434 | R_norvegicus | Animals | mono_intergenic | H/ACA | | | intergenic |
| ENSRNOG00000055361 | LOC120094437 | R_norvegicus | Animals | mono_intronic | C/D | ENSRNOG00000009378 | Rpl4 | protein_coding |
| ENSRNOG00000055363 | LOC120093439 | R_norvegicus | Animals | mono_intergenic | H/ACA | | | intergenic |

| | | | | | | | | |
|---|---|---|---|---|---|---|---|---|
| ENSRNOG00000055366 | LOC120100416 | R_norvegicus | Animals | mono_intergenic | H/ACA | | | intergenic |
| ENSRNOG00000055378 | Snord38a | R_norvegicus | Animals | mono_intronic | C/D | ENSRNOG00000054626 | Rps8 | protein_coding |
| ENSRNOG00000055386 | LOC120100364 | R_norvegicus | Animals | mono_intronic | H/ACA | ENSRNOG00000016063 | Vps35l | protein_coding |
| ENSRNOG00000055387 | LOC120100461 | R_norvegicus | Animals | mono_intronic | H/ACA | ENSRNOG00000019578 | Rps16 | protein_coding |
| ENSRNOG00000055390 | Snord10 | R_norvegicus | Animals | mono_intronic | C/D | ENSRNOG00000030628 | Eif4a1 | protein_coding |
| ENSRNOG00000055418 | LOC120093958 | R_norvegicus | Animals | mono_intronic | H/ACA | ENSRNOG00000064401 | LOC102549144 | non_coding |
| ENSRNOG00000055429 | Snord4a | R_norvegicus | Animals | mono_intronic | C/D | ENSRNOG00000023344 | Rpl23a | protein_coding |
| ENSRNOG00000055442 | LOC120096818 | R_norvegicus | Animals | mono_intergenic | H/ACA | | | intergenic |
| ENSRNOG00000055445 | LOC120096110 | R_norvegicus | Animals | mono_intergenic | H/ACA | | | intergenic |
| ENSRNOG00000055463 | LOC120097987 | R_norvegicus | Animals | mono_intergenic | H/ACA | | | intergenic |
| ENSRNOG00000055464 | LOC120094883 | R_norvegicus | Animals | mono_intronic | C/D | ENSRNOG00000018273 | Ncl | protein_coding |
| ENSRNOG00000055465 | Snora62l1 | R_norvegicus | Animals | mono_intronic | H/ACA | ENSRNOG00000018645 | Rpsa | protein_coding |
| ENSRNOG00000055491 | LOC120095758 | R_norvegicus | Animals | intergenic_cluster | H/ACA | | | intergenic |
| ENSRNOG00000055511 | LOC120096780 | R_norvegicus | Animals | mono_intergenic | H/ACA | | | intergenic |
| ENSRNOG00000055514 | LOC120099512 | R_norvegicus | Animals | mono_intergenic | H/ACA | | | intergenic |
| ENSRNOG00000055516 | LOC120095417 | R_norvegicus | Animals | mono_intronic | H/ACA | ENSRNOG00000069160 | Pitpnc1 | protein_coding |
| ENSRNOG00000055520 | LOC120094463 | R_norvegicus | Animals | mono_intergenic | H/ACA | | | intergenic |
| ENSRNOG00000055522 | LOC120094485 | R_norvegicus | Animals | mono_intergenic | H/ACA | | | intergenic |
| ENSRNOG00000055553 | Snora31 | R_norvegicus | Animals | mono_intronic | H/ACA | ENSRNOG00000001049 | Tpt1 | protein_coding |
| ENSRNOG00000055556 | LOC120101356 | R_norvegicus | Animals | mono_intergenic | H/ACA | | | intergenic |
| ENSRNOG00000055559 | LOC120096787 | R_norvegicus | Animals | mono_intergenic | H/ACA | | | intergenic |
| ENSRNOG00000055560 | LOC120100339 | R_norvegicus | Animals | mono_intronic | C/D | ENSRNOG00000020618 | Rpl13a | protein_coding |
| ENSRNOG00000055561 | LOC120098351 | R_norvegicus | Animals | mono_intronic | C/D | ENSRNOG00000018680 | Rpl17 | protein_coding |
| ENSRNOG00000055569 | Snord14b | R_norvegicus | Animals | mono_intronic | C/D | ENSRNOG00000017418 | Rps3 | protein_coding |
| ENSRNOG00000055571 | LOC120102076 | R_norvegicus | Animals | mono_intergenic | H/ACA | | | intergenic |
| ENSRNOG00000055576 | LOC120102056 | R_norvegicus | Animals | mono_intergenic | H/ACA | | | intergenic |
| ENSRNOG00000055593 | LOC120102694 | R_norvegicus | Animals | mono_intergenic | H/ACA | | | intergenic |
| ENSRNOG00000055602 | LOC120102640 | R_norvegicus | Animals | mono_intergenic | H/ACA | | | intergenic |
| ENSRNOG00000055618 | LOC120103337 | R_norvegicus | Animals | mono_intergenic | H/ACA | | | intergenic |
| ENSRNOG00000055640 | Snord65 | R_norvegicus | Animals | mono_intergenic | C/D | | | intergenic |
| ENSRNOG00000055652 | LOC120096385 | R_norvegicus | Animals | intergenic_cluster | C/D | | | intergenic |
| ENSRNOG00000055656 | LOC120093453 | R_norvegicus | Animals | mono_intergenic | H/ACA | | | intergenic |
| ENSRNOG00000055658 | Snord69 | R_norvegicus | Animals | mono_intronic | C/D | ENSRNOG00000028461 | Gnl3 | protein_coding |
| ENSRNOG00000055664 | LOC120096805 | R_norvegicus | Animals | mono_intergenic | H/ACA | | | intergenic |
| ENSRNOG00000055668 | LOC120101360 | R_norvegicus | Animals | mono_intergenic | H/ACA | | | intergenic |
| ENSRNOG00000055675 | Snord94 | R_norvegicus | Animals | mono_intronic | C/D | ENSRNOG00000009484 | Ptcd3 | protein_coding |
| ENSRNOG00000055690 | LOC120093466 | R_norvegicus | Animals | mono_intergenic | H/ACA | | | intergenic |
| ENSRNOG00000055703 | LOC120102685 | R_norvegicus | Animals | mono_intergenic | H/ACA | | | intergenic |
| ENSRNOG00000055704 | LOC120103319 | R_norvegicus | Animals | mono_intergenic | H/ACA | | | intergenic |
| ENSRNOG00000055710 | LOC120096826 | R_norvegicus | Animals | mono_intergenic | H/ACA | | | intergenic |
| ENSRNOG00000055722 | Snord42b | R_norvegicus | Animals | mono_intronic | C/D | ENSRNOG00000023344 | Rpl23a | protein_coding |

| | | | | | | | | |
|---|---|---|---|---|---|---|---|---|
| ENSRNOG00000055743 | LOC120095489 | R_norvegicus | Animals | intronic_cluster | C/D | ENSRNOG00000023698 | Tex14 | protein_coding |
| ENSRNOG00000055752 | LOC120096815 | R_norvegicus | Animals | mono_intronic | H/ACA | ENSRNOG00000067409 | Cfap299 | protein_coding |
| ENSRNOG00000055772 | LOC120101290 | R_norvegicus | Animals | mono_intergenic | H/ACA | | | intergenic |
| ENSRNOG00000055787 | Snora84 | R_norvegicus | Animals | mono_intergenic | H/ACA | | | intergenic |
| ENSRNOG00000055792 | LOC120095471 | R_norvegicus | Animals | mono_intronic | C/D | ENSRNOG00000008711 | Med24 | protein_coding |
| ENSRNOG00000055808 | LOC120094508 | R_norvegicus | Animals | mono_intergenic | H/ACA | | | intergenic |
| ENSRNOG00000055813 | LOC120101241 | R_norvegicus | Animals | mono_intronic | H/ACA | ENSRNOG00000011150 | Arsb | protein_coding |
| ENSRNOG00000055815 | LOC120099068 | R_norvegicus | Animals | mono_intergenic | H/ACA | | | intergenic |
| ENSRNOG00000055839 | LOC120101362 | R_norvegicus | Animals | mono_intergenic | H/ACA | | | intergenic |
| ENSRNOG00000055844 | LOC120093957 | R_norvegicus | Animals | mono_exonic | H/ACA | ENSRNOG00000068679 | Rpl30 | non_coding |
| ENSRNOG00000055845 | LOC120094474 | R_norvegicus | Animals | intronic_cluster | C/D | ENSRNOG00000010999 | Cep295 | protein_coding |
| ENSRNOG00000055857 | LOC120101259 | R_norvegicus | Animals | mono_intergenic | H/ACA | | | intergenic |
| ENSRNOG00000055876 | LOC120094895 | R_norvegicus | Animals | mono_intergenic | H/ACA | | | intergenic |
| ENSRNOG00000055881 | LOC120100444 | R_norvegicus | Animals | mono_intergenic | H/ACA | | | intergenic |
| ENSRNOG00000055902 | LOC120096782 | R_norvegicus | Animals | mono_intronic | H/ACA | ENSRNOG00000002301 | Uso1 | protein_coding |
| ENSRNOG00000055905 | LOC120101335 | R_norvegicus | Animals | mono_intergenic | H/ACA | | | intergenic |
| ENSRNOG00000055908 | Snora28l1 | R_norvegicus | Animals | mono_intronic | H/ACA | ENSRNOG00000010218 | Eif5 | protein_coding |
| ENSRNOG00000055924 | LOC120100392 | R_norvegicus | Animals | mono_intergenic | H/ACA | | | intergenic |
| ENSRNOG00000055927 | LOC120098378 | R_norvegicus | Animals | mono_intergenic | H/ACA | | | intergenic |
| ENSRNOG00000055932 | LOC120097226 | R_norvegicus | Animals | intergenic_cluster | H/ACA | | | intergenic |
| ENSRNOG00000055941 | Snord127 | R_norvegicus | Animals | mono_intronic | C/D | ENSRNOG00000004521 | Prpf39 | protein_coding |
| ENSRNOG00000055949 | LOC120094528 | R_norvegicus | Animals | mono_intronic | C/D | ENSRNOG00000020608 | Ppan | protein_coding |
| ENSRNOG00000055952 | LOC120096422 | R_norvegicus | Animals | intergenic_cluster | C/D | | | intergenic |
| ENSRNOG00000055961 | LOC120095423 | R_norvegicus | Animals | mono_intronic | H/ACA | ENSRNOG00000053680 | Kansl1 | protein_coding |
| ENSRNOG00000055988 | LOC120102062 | R_norvegicus | Animals | mono_intergenic | H/ACA | | | intergenic |
| ENSRNOG00000055990 | Snord78 | R_norvegicus | Animals | intergenic_cluster | C/D | | | intergenic |
| ENSRNOG00000056003 | LOC120098678 | R_norvegicus | Animals | mono_intronic | H/ACA | ENSRNOG00000017841 | Psme3ip1 | protein_coding |
| ENSRNOG00000056004 | LOC120097672 | R_norvegicus | Animals | mono_intergenic | H/ACA | | | intergenic |
| ENSRNOG00000056006 | LOC120099507 | R_norvegicus | Animals | mono_intergenic | H/ACA | | | intergenic |
| ENSRNOG00000056025 | Gm25617 | R_norvegicus | Animals | intronic_cluster | H/ACA | ENSRNOG00000002833 | Gsk3b | protein_coding |
| ENSRNOG00000056026 | Snora4 | R_norvegicus | Animals | mono_intronic | H/ACA | ENSRNOG00000001815 | Eif4a2 | protein_coding |
| ENSRNOG00000056039 | LOC120103308 | R_norvegicus | Animals | mono_intergenic | H/ACA | | | intergenic |
| ENSRNOG00000056042 | LOC120099451 | R_norvegicus | Animals | mono_intronic | H/ACA | ENSRNOG00000004869 | Dach2 | protein_coding |
| ENSRNOG00000056048 | LOC120096825 | R_norvegicus | Animals | mono_intergenic | H/ACA | | | intergenic |
| ENSRNOG00000056053 | LOC120098401 | R_norvegicus | Animals | mono_intergenic | H/ACA | | | intergenic |
| ENSRNOG00000056054 | Snord118 | R_norvegicus | Animals | mono_intergenic | C/D | | | intergenic |
| ENSRNOG00000056058 | LOC120097976 | R_norvegicus | Animals | mono_intronic | H/ACA | ENSRNOG00000014616 | Iars1 | protein_coding |
| ENSRNOG00000056072 | LOC120096103 | R_norvegicus | Animals | mono_intronic | H/ACA | ENSRNOG00000025244 | Trrap | protein_coding |
| ENSRNOG00000056074 | LOC120099024 | R_norvegicus | Animals | mono_intergenic | H/ACA | | | intergenic |
| ENSRNOG00000056080 | LOC120094919 | R_norvegicus | Animals | mono_intergenic | H/ACA | | | intergenic |
| ENSRNOG00000056084 | LOC120099525 | R_norvegicus | Animals | mono_intronic | H/ACA | ENSRNOG00000002449 | Maged2 | protein_coding |

| | | | | | | | | |
|---|---|---|---|---|---|---|---|---|
| ENSRNOG00000056085 | LOC120095766 | R_norvegicus | Animals | mono_intergenic | H/ACA | | | intergenic |
| ENSRNOG00000056090 | LOC120102706 | R_norvegicus | Animals | mono_intergenic | H/ACA | | | intergenic |
| ENSRNOG00000056100 | LOC120101292 | R_norvegicus | Animals | mono_intergenic | H/ACA | | | intergenic |
| ENSRNOG00000056104 | LOC120096122 | R_norvegicus | Animals | mono_intergenic | H/ACA | | | intergenic |
| ENSRNOG00000056111 | LOC120097270 | R_norvegicus | Animals | mono_intergenic | H/ACA | | | intergenic |
| ENSRNOG00000056117 | LOC120096827 | R_norvegicus | Animals | mono_intergenic | H/ACA | | | intergenic |
| ENSRNOG00000056119 | Snord66 | R_norvegicus | Animals | mono_intronic | C/D | ENSRNOG00000001738 | Eif4g1 | protein_coding |
| ENSRNOG00000056120 | LOC120101337 | R_norvegicus | Animals | mono_intergenic | H/ACA | | | intergenic |
| ENSRNOG00000056123 | LOC120101307 | R_norvegicus | Animals | mono_intergenic | H/ACA | | | intergenic |
| ENSRNOG00000056152 | LOC120098700 | R_norvegicus | Animals | mono_intronic | H/ACA | ENSRNOG00000014371 | Cdh13 | protein_coding |
| ENSRNOG00000056159 | LOC120099493 | R_norvegicus | Animals | mono_intergenic | H/ACA | | | intergenic |
| ENSRNOG00000056164 | LOC120102011 | R_norvegicus | Animals | mono_intergenic | H/ACA | | | intergenic |
| ENSRNOG00000056178 | LOC120093424 | R_norvegicus | Animals | mono_intronic | H/ACA | ENSRNOG00000068162 | Npas3 | protein_coding |
| ENSRNOG00000056181 | LOC120102009 | R_norvegicus | Animals | mono_intergenic | H/ACA | | | intergenic |
| ENSRNOG00000056183 | LOC120093044 | R_norvegicus | Animals | mono_intronic | H/ACA | ENSRNOG00000003661 | Sumo2 | protein_coding |
| ENSRNOG00000056198 | U3l1 | R_norvegicus | Animals | mono_intergenic | C/D | | | intergenic |
| ENSRNOG00000056200 | Snord12c | R_norvegicus | Animals | mono_intergenic | C/D | | | intergenic |
| ENSRNOG00000056215 | LOC120095745 | R_norvegicus | Animals | mono_intergenic | H/ACA | | | intergenic |
| ENSRNOG00000056217 | LOC120093965 | R_norvegicus | Animals | mono_intronic | H/ACA | ENSRNOG00000003890 | Nap1l1 | protein_coding |
| ENSRNOG00000056237 | Snord57 | R_norvegicus | Animals | mono_intronic | C/D | ENSRNOG00000007128 | Nop56 | protein_coding |
| ENSRNOG00000056252 | LOC120100399 | R_norvegicus | Animals | mono_intergenic | H/ACA | | | intergenic |
| ENSRNOG00000056264 | LOC120096123 | R_norvegicus | Animals | mono_intronic | H/ACA | ENSRNOG00000023919 | Nipsnap2 | protein_coding |
| ENSRNOG00000056286 | LOC120095748 | R_norvegicus | Animals | mono_intergenic | H/ACA | | | intergenic |
| ENSRNOG00000056287 | LOC120096831 | R_norvegicus | Animals | mono_intergenic | H/ACA | | | intergenic |
| ENSRNOG00000056300 | LOC120099494 | R_norvegicus | Animals | mono_intergenic | H/ACA | | | intergenic |
| ENSRNOG00000056306 | LOC120097254 | R_norvegicus | Animals | mono_intergenic | H/ACA | | | intergenic |
| ENSRNOG00000056317 | LOC120098384 | R_norvegicus | Animals | mono_intergenic | H/ACA | | | intergenic |
| ENSRNOG00000056323 | LOC120093422 | R_norvegicus | Animals | mono_intergenic | H/ACA | | | intergenic |
| ENSRNOG00000056340 | LOC120094006 | R_norvegicus | Animals | mono_intergenic | H/ACA | | | intergenic |
| ENSRNOG00000056377 | Snord42a | R_norvegicus | Animals | mono_intronic | C/D | ENSRNOG00000023344 | Rpl23a | protein_coding |
| ENSRNOG00000056379 | LOC120096104 | R_norvegicus | Animals | mono_intronic | H/ACA | ENSRNOG00000037449 | Pole | protein_coding |
| ENSRNOG00000056381 | LOC120095744 | R_norvegicus | Animals | intronic_cluster | H/ACA | ENSRNOG00000002833 | Gsk3b | protein_coding |
| ENSRNOG00000056382 | LOC120096790 | R_norvegicus | Animals | mono_exonic | H/ACA | ENSRNOG00000063974 | LOC102547700 | non_coding |
| ENSRNOG00000056385 | LOC120101987 | R_norvegicus | Animals | mono_intergenic | H/ACA | | | intergenic |
| ENSRNOG00000056397 | LOC120100233 | R_norvegicus | Animals | mono_intronic | C/D | ENSRNOG00000067892 | LOC100912041 | non_coding |
| ENSRNOG00000056402 | LOC120102627 | R_norvegicus | Animals | mono_intergenic | H/ACA | | | intergenic |
| ENSRNOG00000056408 | LOC120093459 | R_norvegicus | Animals | mono_intergenic | H/ACA | | | intergenic |
| ENSRNOG00000056416 | LOC120101297 | R_norvegicus | Animals | mono_intergenic | H/ACA | | | intergenic |
| ENSRNOG00000056420 | Snord45c | R_norvegicus | Animals | mono_intronic | C/D | ENSRNOG00000009992 | Rabggtb | protein_coding |
| ENSRNOG00000056422 | Snord104 | R_norvegicus | Animals | intergenic_cluster | C/D | | | intergenic |
| ENSRNOG00000056426 | Snora69 | R_norvegicus | Animals | mono_intergenic | H/ACA | | | intergenic |
| ENSRNOG00000056430 | LOC120093450 | R_norvegicus | Animals | mono_intergenic | H/ACA | | | intergenic |
| ENSRNOG00000056434 | Scarna18b | R_norvegicus | Animals | mono_intronic | H/ACA | ENSRNOG00000003545 | Uchl5 | protein_coding |

| | | | | | | | | |
|---|---|---|---|---|---|---|---|---|
| ENSRNOG00000056455 | LOC120097580 | R_norvegicus | Animals | mono_intergenic | H/ACA | | | intergenic |
| ENSRNOG00000056465 | LOC120099453 | R_norvegicus | Animals | mono_intergenic | H/ACA | | | intergenic |
| ENSRNOG00000056475 | Snord41 | R_norvegicus | Animals | mono_intronic | C/D | ENSRNOG00000003970 | Tnpo2 | protein_coding |
| ENSRNOG00000056479 | LOC120095457 | R_norvegicus | Animals | mono_intronic | H/ACA | ENSRNOG00000036667 | Hexd | protein_coding |
| ENSRNOG00000056492 | LOC120099490 | R_norvegicus | Animals | mono_intergenic | H/ACA | | | intergenic |
| ENSRNOG00000056494 | LOC120100441 | R_norvegicus | Animals | mono_intergenic | H/ACA | | | intergenic |
| ENSRNOG00000056499 | LOC120102687 | R_norvegicus | Animals | mono_intergenic | H/ACA | | | intergenic |
| ENSRNOG00000056505 | LOC120102697 | R_norvegicus | Animals | mono_intergenic | H/ACA | | | intergenic |
| ENSRNOG00000056510 | LOC120098391 | R_norvegicus | Animals | mono_intronic | H/ACA | ENSRNOG00000023412 | Tmem241 | protein_coding |
| ENSRNOG00000056514 | LOC120097998 | R_norvegicus | Animals | mono_intergenic | H/ACA | | | intergenic |
| ENSRNOG00000056518 | Snora60 | R_norvegicus | Animals | intronic_cluster | H/ACA | ENSRNOG00000069588 | | non_coding |
| ENSRNOG00000056543 | LOC120095424 | R_norvegicus | Animals | mono_intergenic | H/ACA | | | intergenic |
| ENSRNOG00000056544 | LOC120102631 | R_norvegicus | Animals | mono_intergenic | H/ACA | | | intergenic |
| ENSRNOG00000056549 | LOC120101339 | R_norvegicus | Animals | mono_intergenic | H/ACA | | | intergenic |
| ENSRNOG00000056593 | LOC120099545 | R_norvegicus | Animals | mono_intergenic | C/D | | | intergenic |
| ENSRNOG00000056599 | Snora53 | R_norvegicus | Animals | mono_intronic | H/ACA | ENSRNOG00000008797 | Tmpo | protein_coding |
| ENSRNOG00000056605 | LOC120100453 | R_norvegicus | Animals | mono_intergenic | H/ACA | | | intergenic |
| ENSRNOG00000056607 | LOC120101315 | R_norvegicus | Animals | mono_intronic | H/ACA | ENSRNOG00000021410 | Negr1 | protein_coding |
| ENSRNOG00000056626 | LOC120099078 | R_norvegicus | Animals | mono_intergenic | H/ACA | | | intergenic |
| ENSRNOG00000056667 | LOC120100450 | R_norvegicus | Animals | mono_intergenic | H/ACA | | | intergenic |
| ENSRNOG00000056677 | LOC120103327 | R_norvegicus | Animals | mono_intergenic | H/ACA | | | intergenic |
| ENSRNOG00000056681 | LOC120093464 | R_norvegicus | Animals | mono_intergenic | H/ACA | | | intergenic |
| ENSRNOG00000056695 | LOC120100442 | R_norvegicus | Animals | mono_intergenic | H/ACA | | | intergenic |
| ENSRNOG00000056726 | LOC120093955 | R_norvegicus | Animals | mono_intergenic | H/ACA | | | intergenic |
| ENSRNOG00000056736 | LOC120095435 | R_norvegicus | Animals | mono_intergenic | H/ACA | | | intergenic |
| ENSRNOG00000056759 | LOC120097218 | R_norvegicus | Animals | mono_intronic | H/ACA | ENSRNOG00000029316 | Gtf2f2 | protein_coding |
| ENSRNOG00000056766 | LOC120097269 | R_norvegicus | Animals | mono_intergenic | H/ACA | | | intergenic |
| ENSRNOG00000056775 | LOC120102045 | R_norvegicus | Animals | mono_intergenic | H/ACA | | | intergenic |
| ENSRNOG00000056779 | LOC120094947 | R_norvegicus | Animals | mono_intronic | C/D | ENSRNOG00000016486 | Nop58 | protein_coding |
| ENSRNOG00000056813 | LOC120102071 | R_norvegicus | Animals | mono_intergenic | H/ACA | | | intergenic |
| ENSRNOG00000056815 | LOC120098380 | R_norvegicus | Animals | mono_intergenic | H/ACA | | | intergenic |
| ENSRNOG00000056818 | LOC120093471 | R_norvegicus | Animals | mono_intergenic | H/ACA | | | intergenic |
| ENSRNOG00000056834 | LOC120095726 | R_norvegicus | Animals | mono_intronic | H/ACA | ENSRNOG00000001815 | Eif4a2 | protein_coding |
| ENSRNOG00000056839 | LOC120094892 | R_norvegicus | Animals | mono_intergenic | H/ACA | | | intergenic |
| ENSRNOG00000056857 | LOC120093992 | R_norvegicus | Animals | mono_intergenic | H/ACA | | | intergenic |
| ENSRNOG00000056879 | LOC120099069 | R_norvegicus | Animals | mono_intergenic | H/ACA | | | intergenic |
| ENSRNOG00000056880 | LOC120101239 | R_norvegicus | Animals | mono_intergenic | H/ACA | | | intergenic |
| ENSRNOG00000056885 | LOC120093993 | R_norvegicus | Animals | mono_intergenic | H/ACA | | | intergenic |
| ENSRNOG00000056888 | LOC120094457 | R_norvegicus | Animals | mono_intergenic | H/ACA | | | intergenic |
| ENSRNOG00000056909 | LOC120098701 | R_norvegicus | Animals | mono_intergenic | H/ACA | | | intergenic |
| ENSRNOG00000056913 | LOC120095461 | R_norvegicus | Animals | mono_intronic | C/D | ENSRNOG00000002980 | Tsr1 | protein_coding |
| ENSRNOG00000056924 | LOC120099497 | R_norvegicus | Animals | mono_intergenic | H/ACA | | | intergenic |
| ENSRNOG00000056942 | LOC120100232 | R_norvegicus | Animals | mono_intronic | C/D | ENSRNOG00000067892 | LOC100912041 | non_coding |

| | | | | | | | | |
|---|---|---|---|---|---|---|---|---|
| ENSRNOG00000056958 | LOC120098364 | R_norvegicus | Animals | mono_intergenic | H/ACA | | | intergenic |
| ENSRNOG00000056973 | LOC120102039 | R_norvegicus | Animals | mono_intergenic | H/ACA | | | intergenic |
| ENSRNOG00000056974 | LOC120103341 | R_norvegicus | Animals | mono_intergenic | H/ACA | | | intergenic |
| ENSRNOG00000056978 | LOC120096111 | R_norvegicus | Animals | mono_intronic | H/ACA | ENSRNOG00000025936 | Rpl6 | protein_coding |
| ENSRNOG00000056990 | LOC120100343 | R_norvegicus | Animals | mono_intronic | H/ACA | ENSRNOG00000022521 | Ddias | protein_coding |
| ENSRNOG00000056995 | LOC120095752 | R_norvegicus | Animals | mono_intronic | H/ACA | ENSRNOG00000069351 | | non_coding |
| ENSRNOG00000057004 | LOC120094013 | R_norvegicus | Animals | mono_intergenic | H/ACA | | | intergenic |
| ENSRNOG00000057012 | Snord95 | R_norvegicus | Animals | mono_intronic | C/D | ENSRNOG00000052620 | Rack1 | protein_coding |
| ENSRNOG00000057029 | LOC120096390 | R_norvegicus | Animals | mono_intergenic | H/ACA | | | intergenic |
| ENSRNOG00000057046 | LOC120097996 | R_norvegicus | Animals | mono_intergenic | H/ACA | | | intergenic |
| ENSRNOG00000057066 | LOC120095735 | R_norvegicus | Animals | mono_intergenic | H/ACA | | | intergenic |
| ENSRNOG00000057075 | Snora46 | R_norvegicus | Animals | mono_intronic | H/ACA | ENSRNOG00000012271 | Cnot1 | protein_coding |
| ENSRNOG00000057084 | LOC120094911 | R_norvegicus | Animals | mono_intergenic | H/ACA | | | intergenic |
| ENSRNOG00000057108 | LOC120094889 | R_norvegicus | Animals | mono_intergenic | H/ACA | | | intergenic |
| ENSRNOG00000057109 | LOC120097225 | R_norvegicus | Animals | mono_intergenic | H/ACA | | | intergenic |
| ENSRNOG00000057114 | LOC120098361 | R_norvegicus | Animals | mono_intergenic | H/ACA | | | intergenic |
| ENSRNOG00000057120 | LOC120095420 | R_norvegicus | Animals | mono_intergenic | H/ACA | | | intergenic |
| ENSRNOG00000057142 | LOC120102688 | R_norvegicus | Animals | intronic_cluster | H/ACA | ENSRNOG00000012337 | Pde1c | protein_coding |
| ENSRNOG00000057168 | LOC120097253 | R_norvegicus | Animals | mono_intronic | H/ACA | ENSRNOG00000007206 | Cfap20dc | protein_coding |
| ENSRNOG00000057170 | LOC120102669 | R_norvegicus | Animals | mono_intergenic | H/ACA | | | intergenic |
| ENSRNOG00000057172 | LOC120094476 | R_norvegicus | Animals | mono_intergenic | H/ACA | | | intergenic |
| ENSRNOG00000057181 | LOC120102010 | R_norvegicus | Animals | mono_intergenic | H/ACA | | | intergenic |
| ENSRNOG00000057202 | LOC120103303 | R_norvegicus | Animals | mono_intergenic | H/ACA | | | intergenic |
| ENSRNOG00000057204 | LOC120094475 | R_norvegicus | Animals | intronic_cluster | C/D | ENSRNOG00000010999 | Cep295 | protein_coding |
| ENSRNOG00000057229 | LOC120103271 | R_norvegicus | Animals | mono_intergenic | H/ACA | | | intergenic |
| ENSRNOG00000057232 | LOC120095450 | R_norvegicus | Animals | mono_intergenic | H/ACA | | | intergenic |
| ENSRNOG00000057240 | Snord49b | R_norvegicus | Animals | intergenic_cluster | C/D | | | intergenic |
| ENSRNOG00000057249 | LOC120093948 | R_norvegicus | Animals | mono_intronic | C/D | ENSRNOG00000002840 | Atp5f1b | protein_coding |
| ENSRNOG00000057251 | LOC120097262 | R_norvegicus | Animals | mono_intergenic | H/ACA | | | intergenic |
| ENSRNOG00000057259 | LOC120094928 | R_norvegicus | Animals | mono_intergenic | H/ACA | | | intergenic |
| ENSRNOG00000057268 | LOC120098001 | R_norvegicus | Animals | mono_intergenic | H/ACA | | | intergenic |
| ENSRNOG00000057278 | LOC120096383 | R_norvegicus | Animals | intergenic_cluster | C/D | | | intergenic |
| ENSRNOG00000057283 | LOC120093467 | R_norvegicus | Animals | mono_intergenic | H/ACA | | | intergenic |
| ENSRNOG00000057287 | LOC120100423 | R_norvegicus | Animals | mono_intergenic | H/ACA | | | intergenic |
| ENSRNOG00000057288 | LOC120094901 | R_norvegicus | Animals | mono_intergenic | H/ACA | | | intergenic |
| ENSRNOG00000057302 | LOC120100440 | R_norvegicus | Animals | mono_intergenic | H/ACA | | | intergenic |
| ENSRNOG00000057313 | LOC120101999 | R_norvegicus | Animals | mono_intergenic | C/D | | | intergenic |
| ENSRNOG00000057316 | Snord88c | R_norvegicus | Animals | mono_intronic | C/D | ENSRNOG00000019132 | Fv1 | protein_coding |
| ENSRNOG00000057324 | LOC120095720 | R_norvegicus | Animals | mono_intergenic | H/ACA | | | intergenic |
| ENSRNOG00000057342 | LOC120101309 | R_norvegicus | Animals | mono_intergenic | H/ACA | | | intergenic |
| ENSRNOG00000057346 | LOC120097592 | R_norvegicus | Animals | mono_intergenic | H/ACA | | | intergenic |
| ENSRNOG00000057361 | LOC120096789 | R_norvegicus | Animals | mono_intergenic | H/ACA | | | intergenic |
| ENSRNOG00000057363 | LOC120097255 | R_norvegicus | Animals | mono_intergenic | H/ACA | | | intergenic |

| | | | | | | | | |
|---|---|---|---|---|---|---|---|---|
| ENSRNOG00000057368 | LOC120101314 | R_norvegicus | Animals | mono_intergenic | H/ACA | | | intergenic |
| ENSRNOG00000057375 | Snora57 | R_norvegicus | Animals | mono_intergenic | H/ACA | | | intergenic |
| ENSRNOG00000057377 | LOC120096113 | R_norvegicus | Animals | mono_intergenic | H/ACA | | | intergenic |
| ENSRNOG00000057394 | LOC120098698 | R_norvegicus | Animals | mono_intergenic | H/ACA | | | intergenic |
| ENSRNOG00000057398 | Snord19b | R_norvegicus | Animals | intronic_cluster | C/D | ENSRNOG00000028461 | Gnl3 | protein_coding |
| ENSRNOG00000057405 | LOC120094931 | R_norvegicus | Animals | mono_intergenic | H/ACA | | | intergenic |
| ENSRNOG00000057420 | LOC120093969 | R_norvegicus | Animals | mono_intergenic | H/ACA | | | intergenic |
| ENSRNOG00000057424 | LOC120093981 | R_norvegicus | Animals | mono_intergenic | H/ACA | | | intergenic |
| ENSRNOG00000057431 | LOC120101301 | R_norvegicus | Animals | mono_intergenic | H/ACA | | | intergenic |
| ENSRNOG00000057432 | LOC120098381 | R_norvegicus | Animals | mono_intergenic | H/ACA | | | intergenic |
| ENSRNOG00000057475 | LOC120102072 | R_norvegicus | Animals | mono_intergenic | H/ACA | | | intergenic |
| ENSRNOG00000057479 | LOC120097624 | R_norvegicus | Animals | mono_intronic | H/ACA | ENSRNOG00000033318 | Rfxank | protein_coding |
| ENSRNOG00000057485 | LOC120099065 | R_norvegicus | Animals | mono_exonic | C/D | ENSRNOG00000001216 | Trpm2 | protein_coding |
| ENSRNOG00000057492 | LOC120097582 | R_norvegicus | Animals | mono_intergenic | C/D | | | intergenic |
| ENSRNOG00000057500 | LOC120093479 | R_norvegicus | Animals | mono_intronic | H/ACA | ENSRNOG00000004300 | Gtf2a1 | protein_coding |
| ENSRNOG00000057503 | LOC120097603 | R_norvegicus | Animals | mono_intergenic | H/ACA | | | intergenic |
| ENSRNOG00000057513 | LOC120093317 | R_norvegicus | Animals | mono_intergenic | H/ACA | | | intergenic |
| ENSRNOG00000057533 | LOC120098349 | R_norvegicus | Animals | mono_intergenic | H/ACA | | | intergenic |
| ENSRNOG00000057537 | Snora5c | R_norvegicus | Animals | mono_intronic | H/ACA | ENSRNOG00000052477 | Tbrg4 | protein_coding |
| ENSRNOG00000057538 | LOC120095451 | R_norvegicus | Animals | mono_intergenic | H/ACA | | | intergenic |
| ENSRNOG00000057539 | LOC120093452 | R_norvegicus | Animals | mono_intergenic | H/ACA | | | intergenic |
| ENSRNOG00000057549 | LOC120094937 | R_norvegicus | Animals | mono_intergenic | H/ACA | | | intergenic |
| ENSRNOG00000057559 | LOC120097620 | R_norvegicus | Animals | mono_intergenic | H/ACA | | | intergenic |
| ENSRNOG00000057567 | LOC120093398 | R_norvegicus | Animals | mono_intergenic | C/D | | | intergenic |
| ENSRNOG00000057568 | LOC120095780 | R_norvegicus | Animals | mono_intronic | H/ACA | ENSRNOG00000060729 | Qtrt2 | protein_coding |
| ENSRNOG00000057576 | LOC120097260 | R_norvegicus | Animals | mono_intergenic | H/ACA | | | intergenic |
| ENSRNOG00000057593 | LOC120099487 | R_norvegicus | Animals | mono_intergenic | H/ACA | | | intergenic |
| ENSRNOG00000057599 | Snora35 | R_norvegicus | Animals | mono_intronic | H/ACA | ENSRNOG00000030877 | Htr2c | protein_coding |
| ENSRNOG00000057605 | LOC120095391 | R_norvegicus | Animals | mono_intergenic | H/ACA | | | intergenic |
| ENSRNOG00000057621 | LOC120098394 | R_norvegicus | Animals | mono_intergenic | H/ACA | | | intergenic |
| ENSRNOG00000057624 | LOC120094009 | R_norvegicus | Animals | mono_intergenic | H/ACA | | | intergenic |
| ENSRNOG00000057673 | LOC120094910 | R_norvegicus | Animals | mono_intergenic | H/ACA | | | intergenic |
| ENSRNOG00000057682 | LOC120094453 | R_norvegicus | Animals | mono_intronic | C/D | ENSRNOG00000010921 | Taf1d | protein_coding |
| ENSRNOG00000057709 | Snord8 | R_norvegicus | Animals | mono_intronic | C/D | ENSRNOG00000025011 | Chd8 | protein_coding |
| ENSRNOG00000057712 | LOC120094446 | R_norvegicus | Animals | mono_intergenic | C/D | | | intergenic |
| ENSRNOG00000057743 | LOC120102683 | R_norvegicus | Animals | mono_intergenic | H/ACA | | | intergenic |
| ENSRNOG00000057745 | LOC120099517 | R_norvegicus | Animals | mono_intronic | H/ACA | ENSRNOG00000029582 | Lrch2 | protein_coding |
| ENSRNOG00000057748 | LOC120095738 | R_norvegicus | Animals | mono_intronic | H/ACA | ENSRNOG00000001613 | Zbtb11 | protein_coding |
| ENSRNOG00000057749 | Gm22598 | R_norvegicus | Animals | mono_intergenic | H/ACA | | | intergenic |
| ENSRNOG00000057752 | LOC120101357 | R_norvegicus | Animals | mono_intergenic | H/ACA | | | intergenic |
| ENSRNOG00000057762 | Snord14a | R_norvegicus | Animals | mono_intronic | C/D | ENSRNOG00000068955 | Rps13 | protein_coding |
| ENSRNOG00000057766 | LOC120093946 | R_norvegicus | Animals | mono_intergenic | H/ACA | | | intergenic |
| ENSRNOG00000057770 | LOC120098709 | R_norvegicus | Animals | mono_intronic | C/D | ENSRNOG00000017724 | Sf3b3 | protein_coding |

| | | | | | | | | |
|---|---|---|---|---|---|---|---|---|
| ENSRNOG00000057777 | LOC120094005 | R_norvegicus | Animals | mono_intergenic | H/ACA | | | intergenic |
| ENSRNOG00000057784 | LOC120099503 | R_norvegicus | Animals | mono_intergenic | H/ACA | | | intergenic |
| ENSRNOG00000057805 | LOC120094492 | R_norvegicus | Animals | mono_intergenic | H/ACA | | | intergenic |
| ENSRNOG00000057812 | Snora72 | R_norvegicus | Animals | mono_intergenic | H/ACA | | | intergenic |
| ENSRNOG00000057816 | LOC120096784 | R_norvegicus | Animals | mono_intronic | H/ACA | ENSRNOG00000052477 | Tbrg4 | protein_coding |
| ENSRNOG00000057873 | LOC120094913 | R_norvegicus | Animals | intergenic_cluster | H/ACA | | | intergenic |
| ENSRNOG00000057896 | LOC120102014 | R_norvegicus | Animals | mono_intergenic | H/ACA | | | intergenic |
| ENSRNOG00000057898 | LOC120095390 | R_norvegicus | Animals | mono_intergenic | H/ACA | | | intergenic |
| ENSRNOG00000057905 | Snord60 | R_norvegicus | Animals | mono_intergenic | C/D | | | intergenic |
| ENSRNOG00000057907 | LOC120099511 | R_norvegicus | Animals | mono_intergenic | H/ACA | | | intergenic |
| ENSRNOG00000057911 | LOC120099063 | R_norvegicus | Animals | mono_exonic | C/D | ENSRNOG00000001216 | Trpm2 | protein_coding |
| ENSRNOG00000057912 | LOC120100283 | R_norvegicus | Animals | mono_intergenic | C/D | | | intergenic |
| ENSRNOG00000057916 | LOC120101338 | R_norvegicus | Animals | mono_intergenic | H/ACA | | | intergenic |
| ENSRNOG00000057918 | LOC120101320 | R_norvegicus | Animals | mono_intergenic | H/ACA | | | intergenic |
| ENSRNOG00000057923 | Snora23 | R_norvegicus | Animals | mono_intronic | H/ACA | ENSRNOG00000010427 | Ipo7 | protein_coding |
| ENSRNOG00000057925 | LOC120095462 | R_norvegicus | Animals | mono_intronic | C/D | ENSRNOG00000002980 | Tsr1 | protein_coding |
| ENSRNOG00000057947 | LOC120097263 | R_norvegicus | Animals | mono_intergenic | H/ACA | | | intergenic |
| ENSRNOG00000057950 | LOC120100413 | R_norvegicus | Animals | mono_intergenic | H/ACA | | | intergenic |
| ENSRNOG00000057952 | LOC120100344 | R_norvegicus | Animals | mono_intronic | C/D | ENSRNOG00000016411 | Rps12 | protein_coding |
| ENSRNOG00000057956 | LOC120103330 | R_norvegicus | Animals | mono_intergenic | H/ACA | | | intergenic |
| ENSRNOG00000057962 | LOC120096810 | R_norvegicus | Animals | mono_intergenic | H/ACA | | | intergenic |
| ENSRNOG00000057969 | LOC120095454 | R_norvegicus | Animals | mono_intronic | H/ACA | ENSRNOG00000070177 | | non_coding |
| ENSRNOG00000057970 | LOC120097224 | R_norvegicus | Animals | mono_intergenic | H/ACA | | | intergenic |
| ENSRNOG00000057985 | LOC120096410 | R_norvegicus | Animals | mono_intergenic | H/ACA | | | intergenic |
| ENSRNOG00000057993 | Snord38b | R_norvegicus | Animals | mono_intronic | C/D | ENSRNOG00000054626 | Rps8 | protein_coding |
| ENSRNOG00000057997 | LOC120102741 | R_norvegicus | Animals | mono_intronic | C/D | ENSRNOG00000063089 | Tmem139 | protein_coding |
| ENSRNOG00000058009 | LOC120095729 | R_norvegicus | Animals | mono_intronic | H/ACA | ENSRNOG00000039567 | Trmt10c | protein_coding |
| ENSRNOG00000058017 | Snord90 | R_norvegicus | Animals | mono_intronic | C/D | ENSRNOG00000009196 | Rc3h2 | protein_coding |
| ENSRNOG00000058025 | LOC120100414 | R_norvegicus | Animals | mono_intergenic | H/ACA | | | intergenic |
| ENSRNOG00000058027 | LOC120101237 | R_norvegicus | Animals | mono_intronic | H/ACA | ENSRNOG00000033560 | Polr3c | protein_coding |
| ENSRNOG00000058033 | Snora27 | R_norvegicus | Animals | mono_intronic | H/ACA | ENSRNOG00000000957 | Rpl21 | protein_coding |
| ENSRNOG00000058048 | LOC120100351 | R_norvegicus | Animals | mono_intergenic | H/ACA | | | intergenic |
| ENSRNOG00000058052 | LOC120102632 | R_norvegicus | Animals | mono_intronic | H/ACA | ENSRNOG00000015097 | Kcmf1 | protein_coding |
| ENSRNOG00000058064 | LOC120093460 | R_norvegicus | Animals | mono_intronic | H/ACA | ENSRNOG00000000618 | Mdga2 | protein_coding |
| ENSRNOG00000058065 | LOC120102724 | R_norvegicus | Animals | mono_intergenic | H/ACA | | | intergenic |
| ENSRNOG00000058074 | LOC120093461 | R_norvegicus | Animals | mono_intergenic | H/ACA | | | intergenic |
| ENSRNOG00000058076 | LOC120094935 | R_norvegicus | Animals | mono_intergenic | H/ACA | | | intergenic |
| ENSRNOG00000058078 | LOC120096377 | R_norvegicus | Animals | mono_intergenic | H/ACA | | | intergenic |
| ENSRNOG00000058085 | LOC120097973 | R_norvegicus | Animals | mono_intergenic | C/D | | | intergenic |
| ENSRNOG00000058093 | Snord59al1 | R_norvegicus | Animals | mono_intronic | C/D | ENSRNOG00000002840 | Atp5f1b | protein_coding |
| ENSRNOG00000058095 | LOC120102708 | R_norvegicus | Animals | mono_intergenic | H/ACA | | | intergenic |
| ENSRNOG00000058104 | LOC120098675 | R_norvegicus | Animals | mono_intergenic | H/ACA | | | intergenic |
| ENSRNOG00000058113 | LOC120101212 | R_norvegicus | Animals | mono_intergenic | H/ACA | | | intergenic |

| | | | | | | | | |
|---|---|---|---|---|---|---|---|---|
| ENSRNOG00000058131 | LOC120101359 | R_norvegicus | Animals | mono_intergenic | H/ACA | | | intergenic |
| ENSRNOG00000058132 | LOC120100227 | R_norvegicus | Animals | mono_intronic | C/D | ENSRNOG00000067892 | LOC100912041 | non_coding |
| ENSRNOG00000058148 | Snord58b | R_norvegicus | Animals | mono_intronic | C/D | ENSRNOG00000018680 | Rpl17 | protein_coding |
| ENSRNOG00000058150 | LOC120101342 | R_norvegicus | Animals | mono_intronic | H/ACA | ENSRNOG00000043329 | Rnf180 | protein_coding |
| ENSRNOG00000058155 | LOC120098692 | R_norvegicus | Animals | mono_intergenic | H/ACA | | | intergenic |
| ENSRNOG00000058161 | LOC120102080 | R_norvegicus | Animals | mono_intergenic | H/ACA | | | intergenic |
| ENSRNOG00000058167 | LOC120095777 | R_norvegicus | Animals | mono_intergenic | C/D | | | intergenic |
| ENSRNOG00000058175 | LOC120101249 | R_norvegicus | Animals | mono_intergenic | H/ACA | | | intergenic |
| ENSRNOG00000058181 | LOC120097237 | R_norvegicus | Animals | mono_intergenic | H/ACA | | | intergenic |
| ENSRNOG00000058196 | LOC120093455 | R_norvegicus | Animals | mono_intergenic | H/ACA | | | intergenic |
| ENSRNOG00000058210 | LOC120103340 | R_norvegicus | Animals | mono_intergenic | H/ACA | | | intergenic |
| ENSRNOG00000058213 | LOC120094459 | R_norvegicus | Animals | mono_intergenic | H/ACA | | | intergenic |
| ENSRNOG00000058232 | LOC120101211 | R_norvegicus | Animals | mono_intergenic | C/D | | | intergenic |
| ENSRNOG00000058239 | LOC120095755 | R_norvegicus | Animals | mono_intergenic | H/ACA | | | intergenic |
| ENSRNOG00000058244 | LOC120095419 | R_norvegicus | Animals | mono_intergenic | H/ACA | | | intergenic |
| ENSRNOG00000058255 | LOC120101313 | R_norvegicus | Animals | mono_intergenic | H/ACA | | | intergenic |
| ENSRNOG00000058269 | Snord83b | R_norvegicus | Animals | mono_intronic | C/D | ENSRNOG00000016896 | Rpl3 | protein_coding |
| ENSRNOG00000058279 | LOC120102675 | R_norvegicus | Animals | mono_intergenic | H/ACA | | | intergenic |
| ENSRNOG00000058283 | Snora6l1 | R_norvegicus | Animals | mono_intronic | H/ACA | ENSRNOG00000018645 | Rpsa | protein_coding |
| ENSRNOG00000058294 | LOC120095753 | R_norvegicus | Animals | mono_intergenic | H/ACA | | | intergenic |
| ENSRNOG00000058302 | LOC120096800 | R_norvegicus | Animals | mono_intergenic | H/ACA | | | intergenic |
| ENSRNOG00000058310 | LOC120102079 | R_norvegicus | Animals | mono_intergenic | H/ACA | | | intergenic |
| ENSRNOG00000058339 | LOC120094914 | R_norvegicus | Animals | mono_intergenic | H/ACA | | | intergenic |
| ENSRNOG00000058366 | LOC120098681 | R_norvegicus | Animals | mono_intronic | H/ACA | ENSRNOG00000013481 | Cdh11 | protein_coding |
| ENSRNOG00000058367 | LOC120100433 | R_norvegicus | Animals | mono_intergenic | H/ACA | | | intergenic |
| ENSRNOG00000058368 | LOC120099022 | R_norvegicus | Animals | mono_intergenic | H/ACA | | | intergenic |
| ENSRNOG00000058376 | Snora19 | R_norvegicus | Animals | mono_intergenic | H/ACA | | | intergenic |
| ENSRNOG00000058384 | LOC120098396 | R_norvegicus | Animals | mono_intergenic | H/ACA | | | intergenic |
| ENSRNOG00000058387 | LOC120100436 | R_norvegicus | Animals | mono_intergenic | H/ACA | | | intergenic |
| ENSRNOG00000058390 | LOC120096389 | R_norvegicus | Animals | mono_intergenic | H/ACA | | | intergenic |
| ENSRNOG00000058395 | LOC120097673 | R_norvegicus | Animals | mono_intronic | H/ACA | ENSRNOG00000069017 | Plpp5 | protein_coding |
| ENSRNOG00000058396 | LOC120095767 | R_norvegicus | Animals | intergenic_cluster | H/ACA | | | intergenic |
| ENSRNOG00000058403 | LOC120102732 | R_norvegicus | Animals | mono_intergenic | H/ACA | | | intergenic |
| ENSRNOG00000058433 | LOC120096804 | R_norvegicus | Animals | mono_intergenic | H/ACA | | | intergenic |
| ENSRNOG00000058445 | LOC120094456 | R_norvegicus | Animals | mono_intergenic | H/ACA | | | intergenic |
| ENSRNOG00000058449 | LOC120094888 | R_norvegicus | Animals | mono_intronic | H/ACA | ENSRNOG00000024186 | Eef1b2 | protein_coding |
| ENSRNOG00000058459 | LOC120098402 | R_norvegicus | Animals | mono_intergenic | H/ACA | | | intergenic |
| ENSRNOG00000058468 | LOC120095759 | R_norvegicus | Animals | mono_intergenic | H/ACA | | | intergenic |
| ENSRNOG00000058479 | LOC120097276 | R_norvegicus | Animals | mono_intronic | C/D | ENSRNOG00000026056 | Ccnb1ip1 | protein_coding |
| ENSRNOG00000058488 | Snord53l2 | R_norvegicus | Animals | mono_intronic | C/D | ENSRNOG00000026316 | Wdr43 | protein_coding |
| ENSRNOG00000058492 | LOC120096793 | R_norvegicus | Animals | mono_intronic | H/ACA | ENSRNOG00000017259 | Tacc3 | protein_coding |
| ENSRNOG00000058502 | LOC120101351 | R_norvegicus | Animals | mono_intergenic | H/ACA | | | intergenic |
| ENSRNOG00000058518 | LOC120100369 | R_norvegicus | Animals | mono_intergenic | H/ACA | | | intergenic |

| | | | | | | | | |
|---|---|---|---|---|---|---|---|---|
| ENSRNOG00000058525 | LOC120097261 | R_norvegicus | Animals | mono_intergenic | H/ACA | | | intergenic |
| ENSRNOG00000058533 | LOC120103284 | R_norvegicus | Animals | mono_intronic | H/ACA | ENSRNOG00000015642 | Pabpc4 | protein_coding |
| ENSRNOG00000058535 | LOC120099029 | R_norvegicus | Animals | mono_intergenic | H/ACA | | | intergenic |
| ENSRNOG00000058540 | LOC120099440 | R_norvegicus | Animals | mono_intronic | H/ACA | ENSRNOG00000004036 | Snx12 | protein_coding |
| ENSRNOG00000058556 | LOC120095384 | R_norvegicus | Animals | mono_intergenic | H/ACA | | | intergenic |
| ENSRNOG00000058594 | LOC120093484 | R_norvegicus | Animals | mono_intergenic | H/ACA | | | intergenic |
| ENSRNOG00000058595 | LOC120103310 | R_norvegicus | Animals | mono_intergenic | H/ACA | | | intergenic |
| ENSRNOG00000058605 | LOC120102069 | R_norvegicus | Animals | mono_intergenic | H/ACA | | | intergenic |
| ENSRNOG00000058606 | LOC120094464 | R_norvegicus | Animals | mono_intronic | H/ACA | ENSRNOG00000010921 | Taf1d | protein_coding |
| ENSRNOG00000058607 | LOC120103312 | R_norvegicus | Animals | mono_intergenic | H/ACA | | | intergenic |
| ENSRNOG00000058614 | LOC120094519 | R_norvegicus | Animals | mono_intergenic | H/ACA | | | intergenic |
| ENSRNOG00000058631 | LOC120099059 | R_norvegicus | Animals | mono_intergenic | C/D | | | intergenic |
| ENSRNOG00000058635 | LOC120102059 | R_norvegicus | Animals | mono_intergenic | H/ACA | | | intergenic |
| ENSRNOG00000058641 | LOC120102003 | R_norvegicus | Animals | mono_intergenic | H/ACA | | | intergenic |
| ENSRNOG00000058651 | LOC120100452 | R_norvegicus | Animals | mono_intergenic | H/ACA | | | intergenic |
| ENSRNOG00000058657 | LOC120103256 | R_norvegicus | Animals | mono_intronic | C/D | ENSRNOG00000007575 | Plppr1 | protein_coding |
| ENSRNOG00000058660 | LOC120095776 | R_norvegicus | Animals | mono_intronic | H/ACA | ENSRNOG00000067589 | Fbxo45 | protein_coding |
| ENSRNOG00000058679 | Snord21 | R_norvegicus | Animals | mono_intronic | C/D | ENSRNOG00000023529 | Rpl5 | protein_coding |
| ENSRNOG00000058687 | LOC120095746 | R_norvegicus | Animals | mono_intergenic | H/ACA | | | intergenic |
| ENSRNOG00000058689 | AC111292.6 | R_norvegicus | Animals | intergenic_cluster | H/ACA | | | intergenic |
| ENSRNOG00000058700 | LOC120101318 | R_norvegicus | Animals | mono_intergenic | H/ACA | | | intergenic |
| ENSRNOG00000058701 | Snord107 | R_norvegicus | Animals | mono_intronic | C/D | ENSRNOG00000065497 | LOC102547183 | non_coding |
| ENSRNOG00000058702 | LOC120095763 | R_norvegicus | Animals | mono_intronic | H/ACA | ENSRNOG00000031669 | Lpp | protein_coding |
| ENSRNOG00000058703 | LOC120099520 | R_norvegicus | Animals | mono_intergenic | H/ACA | | | intergenic |
| ENSRNOG00000058707 | LOC120094017 | R_norvegicus | Animals | mono_intronic | H/ACA | ENSRNOG00000006700 | Ntaq1 | protein_coding |
| ENSRNOG00000058715 | LOC120100264 | R_norvegicus | Animals | mono_intergenic | C/D | | | intergenic |
| ENSRNOG00000058729 | LOC120101311 | R_norvegicus | Animals | mono_intergenic | H/ACA | | | intergenic |
| ENSRNOG00000058734 | LOC120102019 | R_norvegicus | Animals | mono_intergenic | H/ACA | | | intergenic |
| ENSRNOG00000058743 | LOC120099471 | R_norvegicus | Animals | mono_intergenic | H/ACA | | | intergenic |
| ENSRNOG00000058757 | LOC120102670 | R_norvegicus | Animals | mono_intergenic | H/ACA | | | intergenic |
| ENSRNOG00000058759 | LOC120097615 | R_norvegicus | Animals | mono_intergenic | H/ACA | | | intergenic |
| ENSRNOG00000058768 | LOC120103348 | R_norvegicus | Animals | mono_intergenic | H/ACA | | | intergenic |
| ENSRNOG00000058770 | LOC120101298 | R_norvegicus | Animals | mono_intergenic | H/ACA | | | intergenic |
| ENSRNOG00000058773 | LOC120102104 | R_norvegicus | Animals | mono_intergenic | C/D | | | intergenic |
| ENSRNOG00000058792 | LOC120099518 | R_norvegicus | Animals | mono_intronic | H/ACA | ENSRNOG00000002565 | Taf1 | protein_coding |
| ENSRNOG00000058797 | LOC120103332 | R_norvegicus | Animals | mono_intergenic | H/ACA | | | intergenic |
| ENSRNOG00000058802 | LOC120102084 | R_norvegicus | Animals | mono_intergenic | H/ACA | | | intergenic |
| ENSRNOG00000058804 | LOC120094015 | R_norvegicus | Animals | mono_intronic | H/ACA | ENSRNOG00000004011 | Nedd1 | protein_coding |
| ENSRNOG00000058806 | LOC120102067 | R_norvegicus | Animals | mono_intronic | H/ACA | ENSRNOG00000034258 | Xirp2 | protein_coding |
| ENSRNOG00000058807 | LOC120101353 | R_norvegicus | Animals | mono_intergenic | H/ACA | | | intergenic |
| ENSRNOG00000058810 | LOC120097583 | R_norvegicus | Animals | mono_intergenic | H/ACA | | | intergenic |
| ENSRNOG00000058816 | Snord73a | R_norvegicus | Animals | mono_intronic | C/D | ENSRNOG00000011893 | Rps3a | protein_coding |
| ENSRNOG00000058823 | LOC120098395 | R_norvegicus | Animals | mono_intergenic | H/ACA | | | intergenic |

| | | | | | | | | |
|---|---|---|---|---|---|---|---|---|
| ENSRNOG00000058828 | LOC120094012 | R_norvegicus | Animals | mono_intergenic | H/ACA | | | intergenic |
| ENSRNOG00000058838 | LOC120095750 | R_norvegicus | Animals | mono_intergenic | H/ACA | | | intergenic |
| ENSRNOG00000058851 | LOC120101332 | R_norvegicus | Animals | mono_intergenic | H/ACA | | | intergenic |
| ENSRNOG00000058865 | LOC120097259 | R_norvegicus | Animals | mono_intergenic | H/ACA | | | intergenic |
| ENSRNOG00000058881 | LOC120094899 | R_norvegicus | Animals | mono_intronic | H/ACA | ENSRNOG00000016486 | Nop58 | protein_coding |
| ENSRNOG00000058895 | LOC120098002 | R_norvegicus | Animals | mono_intergenic | H/ACA | | | intergenic |
| ENSRNOG00000058899 | Snord49a | R_norvegicus | Animals | intergenic_cluster | C/D | | | intergenic |
| ENSRNOG00000058901 | LOC120096396 | R_norvegicus | Animals | mono_intronic | H/ACA | ENSRNOG00000002595 | Dpp10 | protein_coding |
| ENSRNOG00000058905 | LOC120102662 | R_norvegicus | Animals | mono_intergenic | H/ACA | | | intergenic |
| ENSRNOG00000058913 | LOC120100412 | R_norvegicus | Animals | mono_intergenic | H/ACA | | | intergenic |
| ENSRNOG00000058915 | Snord45l1 | R_norvegicus | Animals | mono_intergenic | C/D | | | intergenic |
| ENSRNOG00000058919 | LOC120102077 | R_norvegicus | Animals | mono_intergenic | H/ACA | | | intergenic |
| ENSRNOG00000058929 | LOC120096380 | R_norvegicus | Animals | mono_intronic | H/ACA | ENSRNOG00000002736 | Rabgap1l | protein_coding |
| ENSRNOG00000058942 | LOC120099467 | R_norvegicus | Animals | mono_intergenic | H/ACA | | | intergenic |
| ENSRNOG00000058944 | Snora71c | R_norvegicus | Animals | mono_intergenic | H/ACA | | | intergenic |
| ENSRNOG00000058946 | LOC120100377 | R_norvegicus | Animals | mono_intronic | H/ACA | ENSRNOG00000017260 | Cdr2 | protein_coding |
| ENSRNOG00000058961 | LOC120097995 | R_norvegicus | Animals | mono_intergenic | H/ACA | | | intergenic |
| ENSRNOG00000058963 | LOC120099456 | R_norvegicus | Animals | mono_intergenic | H/ACA | | | intergenic |
| ENSRNOG00000058965 | LOC120100458 | R_norvegicus | Animals | mono_intergenic | H/ACA | | | intergenic |
| ENSRNOG00000058966 | LOC120101229 | R_norvegicus | Animals | mono_intergenic | H/ACA | | | intergenic |
| ENSRNOG00000058974 | LOC120102042 | R_norvegicus | Animals | mono_intergenic | H/ACA | | | intergenic |
| ENSRNOG00000058981 | LOC120098360 | R_norvegicus | Animals | mono_intronic | H/ACA | ENSRNOG00000066854 | LOC120098269 | non_coding |
| ENSRNOG00000059000 | LOC120099442 | R_norvegicus | Animals | mono_intergenic | H/ACA | | | intergenic |
| ENSRNOG00000059024 | LOC120102055 | R_norvegicus | Animals | mono_intergenic | H/ACA | | | intergenic |
| ENSRNOG00000059034 | LOC120097240 | R_norvegicus | Animals | mono_intergenic | H/ACA | | | intergenic |
| ENSRNOG00000059035 | LOC120098369 | R_norvegicus | Animals | mono_intergenic | H/ACA | | | intergenic |
| ENSRNOG00000059042 | LOC120097972 | R_norvegicus | Animals | mono_intergenic | C/D | | | intergenic |
| ENSRNOG00000059045 | LOC120094461 | R_norvegicus | Animals | mono_intergenic | H/ACA | | | intergenic |
| ENSRNOG00000059060 | LOC120095747 | R_norvegicus | Animals | mono_intergenic | H/ACA | | | intergenic |
| ENSRNOG00000059077 | LOC120093945 | R_norvegicus | Animals | mono_exonic | H/ACA | ENSRNOG00000069806 | RGD1564963 | protein_coding |
| ENSRNOG00000059078 | LOC120101303 | R_norvegicus | Animals | mono_intergenic | H/ACA | | | intergenic |
| ENSRNOG00000059083 | LOC120100370 | R_norvegicus | Animals | mono_intergenic | H/ACA | | | intergenic |
| ENSRNOG00000059086 | LOC120099464 | R_norvegicus | Animals | mono_intergenic | H/ACA | | | intergenic |
| ENSRNOG00000059106 | Snord113l3 | R_norvegicus | Animals | mono_intergenic | C/D | | | intergenic |
| ENSRNOG00000059119 | LOC120099074 | R_norvegicus | Animals | mono_intergenic | H/ACA | | | intergenic |
| ENSRNOG00000059132 | LOC120102073 | R_norvegicus | Animals | mono_intergenic | H/ACA | | | intergenic |
| ENSRNOG00000059156 | LOC120097994 | R_norvegicus | Animals | mono_intergenic | H/ACA | | | intergenic |
| ENSRNOG00000059158 | LOC120094002 | R_norvegicus | Animals | mono_intergenic | H/ACA | | | intergenic |
| ENSRNOG00000059161 | LOC120093441 | R_norvegicus | Animals | mono_intergenic | H/ACA | | | intergenic |
| ENSRNOG00000059171 | LOC120098352 | R_norvegicus | Animals | mono_intronic | C/D | ENSRNOG00000018680 | Rpl17 | protein_coding |
| ENSRNOG00000059173 | LOC120100415 | R_norvegicus | Animals | mono_intergenic | H/ACA | | | intergenic |
| ENSRNOG00000059204 | LOC120103275 | R_norvegicus | Animals | mono_intergenic | H/ACA | | | intergenic |
| ENSRNOG00000059213 | LOC120096781 | R_norvegicus | Animals | mono_intergenic | C/D | | | intergenic |

| | | | | | | | | |
|---|---|---|---|---|---|---|---|---|
| ENSRNOG00000059221 | LOC120101295 | R_norvegicus | Animals | mono_intergenic | H/ACA | | | intergenic |
| ENSRNOG00000059226 | LOC120100371 | R_norvegicus | Animals | mono_intergenic | H/ACA | | | intergenic |
| ENSRNOG00000059239 | LOC120100361 | R_norvegicus | Animals | mono_intergenic | H/ACA | | | intergenic |
| ENSRNOG00000059242 | LOC120096409 | R_norvegicus | Animals | mono_intergenic | H/ACA | | | intergenic |
| ENSRNOG00000059253 | LOC120099519 | R_norvegicus | Animals | mono_intergenic | H/ACA | | | intergenic |
| ENSRNOG00000059267 | LOC120093316 | R_norvegicus | Animals | mono_intronic | H/ACA | ENSRNOG00000005589 | Dhrs7 | protein_coding |
| ENSRNOG00000059275 | LOC120101325 | R_norvegicus | Animals | mono_intergenic | H/ACA | | | intergenic |
| ENSRNOG00000059287 | LOC120102066 | R_norvegicus | Animals | mono_intergenic | H/ACA | | | intergenic |
| ENSRNOG00000059289 | LOC120093049 | R_norvegicus | Animals | mono_intergenic | H/ACA | | | intergenic |
| ENSRNOG00000059290 | LOC120101281 | R_norvegicus | Animals | mono_intergenic | H/ACA | | | intergenic |
| ENSRNOG00000059300 | LOC120095414 | R_norvegicus | Animals | mono_intergenic | H/ACA | | | intergenic |
| ENSRNOG00000059305 | LOC120094010 | R_norvegicus | Animals | mono_intergenic | H/ACA | | | intergenic |
| ENSRNOG00000059307 | LOC120101343 | R_norvegicus | Animals | mono_intergenic | H/ACA | | | intergenic |
| ENSRNOG00000059321 | LOC120100395 | R_norvegicus | Animals | intergenic_cluster | C/D | | | intergenic |
| ENSRNOG00000059323 | LOC120100373 | R_norvegicus | Animals | mono_intergenic | H/ACA | | | intergenic |
| ENSRNOG00000059324 | LOC120094507 | R_norvegicus | Animals | mono_intergenic | H/ACA | | | intergenic |
| ENSRNOG00000059339 | LOC120094454 | R_norvegicus | Animals | mono_intronic | H/ACA | ENSRNOG00000010921 | Taf1d | protein_coding |
| ENSRNOG00000059346 | LOC120094448 | R_norvegicus | Animals | mono_intergenic | H/ACA | | | intergenic |
| ENSRNOG00000059370 | LOC120093474 | R_norvegicus | Animals | mono_intronic | H/ACA | ENSRNOG00000003951 | Psmc1 | protein_coding |
| ENSRNOG00000059371 | LOC120093470 | R_norvegicus | Animals | mono_intergenic | H/ACA | | | intergenic |
| ENSRNOG00000059399 | LOC120095444 | R_norvegicus | Animals | mono_intergenic | H/ACA | | | intergenic |
| ENSRNOG00000059404 | Snora64 | R_norvegicus | Animals | mono_intronic | H/ACA | ENSRNOG00000014179 | Rps2 | protein_coding |
| ENSRNOG00000059407 | LOC120101255 | R_norvegicus | Animals | mono_intergenic | H/ACA | | | intergenic |
| ENSRNOG00000059409 | LOC120093469 | R_norvegicus | Animals | mono_intergenic | H/ACA | | | intergenic |
| ENSRNOG00000059410 | LOC120094918 | R_norvegicus | Animals | mono_intergenic | H/ACA | | | intergenic |
| ENSRNOG00000059422 | Snora71a | R_norvegicus | Animals | mono_intergenic | H/ACA | | | intergenic |
| ENSRNOG00000059423 | LOC120094008 | R_norvegicus | Animals | mono_intergenic | H/ACA | | | intergenic |
| ENSRNOG00000059435 | LOC120094439 | R_norvegicus | Animals | mono_intronic | C/D | ENSRNOG00000009378 | Rpl4 | protein_coding |
| ENSRNOG00000059444 | Snord23 | R_norvegicus | Animals | mono_intronic | C/D | ENSRNOG00000013023 | Nop53 | protein_coding |
| ENSRNOG00000059446 | LOC120103317 | R_norvegicus | Animals | mono_intergenic | H/ACA | | | intergenic |
| ENSRNOG00000059451 | LOC120100320 | R_norvegicus | Animals | mono_intergenic | C/D | | | intergenic |
| ENSRNOG00000059462 | LOC120098691 | R_norvegicus | Animals | mono_intergenic | H/ACA | | | intergenic |
| ENSRNOG00000059471 | LOC120102714 | R_norvegicus | Animals | mono_intergenic | H/ACA | | | intergenic |
| ENSRNOG00000059477 | LOC120097988 | R_norvegicus | Animals | mono_intronic | H/ACA | ENSRNOG00000019223 | Atp5f1c | protein_coding |
| ENSRNOG00000059478 | Snord54 | R_norvegicus | Animals | mono_intronic | C/D | ENSRNOG00000008555 | Rps20-ps10 | protein_coding |
| ENSRNOG00000059486 | LOC120102639 | R_norvegicus | Animals | mono_intronic | H/ACA | ENSRNOG00000016507 | LOC687679 | protein_coding |
| ENSRNOG00000059499 | LOC120097992 | R_norvegicus | Animals | mono_intergenic | H/ACA | | | intergenic |
| ENSRNOG00000059502 | LOC120103252 | R_norvegicus | Animals | mono_intronic | H/ACA | ENSRNOG00000026299 | Mysm1 | protein_coding |
| ENSRNOG00000059509 | LOC120096386 | R_norvegicus | Animals | intergenic_cluster | C/D | | | intergenic |
| ENSRNOG00000059511 | LOC120102693 | R_norvegicus | Animals | mono_intergenic | H/ACA | | | intergenic |
| ENSRNOG00000059526 | LOC120103315 | R_norvegicus | Animals | mono_intergenic | H/ACA | | | intergenic |
| ENSRNOG00000059556 | Snora36b | R_norvegicus | Animals | mono_intronic | H/ACA | ENSRNOG00000002353 | Rab3gap2 | protein_coding |
| ENSRNOG00000059557 | LOC120103339 | R_norvegicus | Animals | mono_intergenic | H/ACA | | | intergenic |

| | | | | | | | | |
|---|---|---|---|---|---|---|---|---|
| ENSRNOG00000059571 | LOC120102622 | R_norvegicus | Animals | mono_intergenic | H/ACA | | | intergenic |
| ENSRNOG00000059573 | LOC120101354 | R_norvegicus | Animals | mono_intergenic | H/ACA | | | intergenic |
| ENSRNOG00000059576 | LOC120093478 | R_norvegicus | Animals | mono_intergenic | H/ACA | | | intergenic |
| ENSRNOG00000059581 | LOC120094467 | R_norvegicus | Animals | mono_intergenic | H/ACA | | | intergenic |
| ENSRNOG00000059598 | LOC120102626 | R_norvegicus | Animals | mono_intergenic | H/ACA | | | intergenic |
| ENSRNOG00000059609 | LOC120101358 | R_norvegicus | Animals | mono_intergenic | H/ACA | | | intergenic |
| ENSRNOG00000059610 | LOC120095412 | R_norvegicus | Animals | mono_intergenic | H/ACA | | | intergenic |
| ENSRNOG00000059627 | LOC120097264 | R_norvegicus | Animals | mono_intronic | H/ACA | ENSRNOG00000004516 | Itgbl1 | protein_coding |
| ENSRNOG00000059631 | LOC120102677 | R_norvegicus | Animals | mono_intergenic | H/ACA | | | intergenic |
| ENSRNOG00000059636 | LOC120101252 | R_norvegicus | Animals | mono_intergenic | H/ACA | | | intergenic |
| ENSRNOG00000059637 | LOC120100451 | R_norvegicus | Animals | mono_intronic | H/ACA | ENSRNOG00000012641 | Sfr1 | protein_coding |
| ENSRNOG00000059639 | LOC120102634 | R_norvegicus | Animals | mono_intergenic | H/ACA | | | intergenic |
| ENSRNOG00000059646 | LOC120096809 | R_norvegicus | Animals | mono_intergenic | H/ACA | | | intergenic |
| ENSRNOG00000059648 | LOC120093449 | R_norvegicus | Animals | mono_intergenic | H/ACA | | | intergenic |
| ENSRNOG00000059650 | LOC120097621 | R_norvegicus | Animals | mono_intergenic | H/ACA | | | intergenic |
| ENSRNOG00000059658 | LOC120099080 | R_norvegicus | Animals | mono_intergenic | H/ACA | | | intergenic |
| ENSRNOG00000059684 | LOC120096398 | R_norvegicus | Animals | mono_intergenic | H/ACA | | | intergenic |
| ENSRNOG00000059686 | LOC120103280 | R_norvegicus | Animals | mono_intronic | H/ACA | ENSRNOG00000012455 | Tardbp | protein_coding |
| ENSRNOG00000059688 | LOC120095411 | R_norvegicus | Animals | mono_intronic | H/ACA | ENSRNOG00000004686 | Spop | protein_coding |
| ENSRNOG00000059707 | LOC120097618 | R_norvegicus | Animals | mono_intergenic | H/ACA | | | intergenic |
| ENSRNOG00000059716 | LOC120103338 | R_norvegicus | Animals | mono_intergenic | H/ACA | | | intergenic |
| ENSRNOG00000059726 | LOC120094452 | R_norvegicus | Animals | mono_intronic | H/ACA | ENSRNOG00000053404 | Ppcdc | protein_coding |
| ENSRNOG00000059732 | LOC120102041 | R_norvegicus | Animals | mono_intergenic | H/ACA | | | intergenic |
| ENSRNOG00000059747 | LOC120096814 | R_norvegicus | Animals | mono_intergenic | H/ACA | | | intergenic |
| ENSRNOG00000059797 | LOC120096402 | R_norvegicus | Animals | mono_intronic | H/ACA | ENSRNOG00000003698 | Mroh9 | protein_coding |
| ENSRNOG00000059800 | LOC120101334 | R_norvegicus | Animals | mono_intergenic | H/ACA | | | intergenic |
| ENSRNOG00000059804 | Snord102 | R_norvegicus | Animals | mono_intronic | C/D | ENSRNOG00000000957 | Rpl21 | protein_coding |
| ENSRNOG00000059814 | LOC120099445 | R_norvegicus | Animals | mono_intergenic | H/ACA | | | intergenic |
| ENSRNOG00000059820 | LOC120100360 | R_norvegicus | Animals | mono_intergenic | H/ACA | | | intergenic |
| ENSRNOG00000059833 | LOC120094016 | R_norvegicus | Animals | mono_intronic | H/ACA | ENSRNOG00000023480 | Nabp2 | protein_coding |
| ENSRNOG00000059864 | LOC120100432 | R_norvegicus | Animals | mono_intergenic | H/ACA | | | intergenic |
| ENSRNOG00000059871 | LOC120094497 | R_norvegicus | Animals | mono_intergenic | H/ACA | | | intergenic |
| ENSRNOG00000059872 | Gm50450 | R_norvegicus | Animals | mono_intronic | H/ACA | ENSRNOG00000012264 | Alkbh1 | protein_coding |
| ENSRNOG00000059879 | LOC120097614 | R_norvegicus | Animals | mono_intergenic | H/ACA | | | intergenic |
| ENSRNOG00000059892 | LOC120094917 | R_norvegicus | Animals | mono_intergenic | H/ACA | | | intergenic |
| ENSRNOG00000059895 | LOC120097674 | R_norvegicus | Animals | mono_intergenic | H/ACA | | | intergenic |
| ENSRNOG00000059902 | LOC120097625 | R_norvegicus | Animals | mono_intergenic | H/ACA | | | intergenic |
| ENSRNOG00000059926 | Snord71 | R_norvegicus | Animals | mono_intronic | C/D | ENSRNOG00000069458 | Ap1g1 | protein_coding |
| ENSRNOG00000059930 | LOC120100430 | R_norvegicus | Animals | mono_intergenic | H/ACA | | | intergenic |
| ENSRNOG00000059932 | LOC120096816 | R_norvegicus | Animals | mono_intergenic | H/ACA | | | intergenic |
| ENSRNOG00000059948 | LOC120100266 | R_norvegicus | Animals | mono_intergenic | C/D | | | intergenic |
| ENSRNOG00000059951 | LOC120101350 | R_norvegicus | Animals | mono_intergenic | H/ACA | | | intergenic |
| ENSRNOG00000059967 | LOC120103325 | R_norvegicus | Animals | mono_intergenic | H/ACA | | | intergenic |

| | | | | | | | | |
|---|---|---|---|---|---|---|---|---|
| ENSRNOG00000059972 | LOC120093447 | R_norvegicus | Animals | mono_intergenic | H/ACA | | | intergenic |
| ENSRNOG00000059985 | LOC120097989 | R_norvegicus | Animals | mono_intergenic | H/ACA | | | intergenic |
| ENSRNOG00000059989 | LOC120094501 | R_norvegicus | Animals | mono_intergenic | H/ACA | | | intergenic |
| ENSRNOG00000059991 | LOC120103307 | R_norvegicus | Animals | mono_intergenic | H/ACA | | | intergenic |
| ENSRNOG00000060026 | LOC120102004 | R_norvegicus | Animals | mono_intergenic | H/ACA | | | intergenic |
| ENSRNOG00000060028 | LOC120101336 | R_norvegicus | Animals | mono_intergenic | H/ACA | | | intergenic |
| ENSRNOG00000060031 | Snord35a | R_norvegicus | Animals | mono_intronic | C/D | ENSRNOG00000020618 | Rpl13a | protein_coding |
| ENSRNOG00000060033 | LOC120100449 | R_norvegicus | Animals | mono_intergenic | H/ACA | | | intergenic |
| ENSRNOG00000060035 | LOC120095739 | R_norvegicus | Animals | mono_intronic | H/ACA | ENSRNOG00000001862 | Ube2l3 | protein_coding |
| ENSRNOG00000060046 | LOC120095409 | R_norvegicus | Animals | mono_intronic | H/ACA | ENSRNOG00000027008 | Igtp | protein_coding |
| ENSRNOG00000060059 | LOC120095392 | R_norvegicus | Animals | mono_intronic | H/ACA | ENSRNOG00000008704 | Tmem97 | protein_coding |
| ENSRNOG00000060068 | LOC120096394 | R_norvegicus | Animals | mono_intergenic | H/ACA | | | intergenic |
| ENSRNOG00000060079 | Snord26 | R_norvegicus | Animals | mono_intronic | C/D | ENSRNOG00000067892 | LOC100912041 | non_coding |
| ENSRNOG00000060082 | LOC120095768 | R_norvegicus | Animals | mono_intergenic | H/ACA | | | intergenic |
| ENSRNOG00000060083 | LOC120098392 | R_norvegicus | Animals | mono_intergenic | H/ACA | | | intergenic |
| ENSRNOG00000060084 | LOC120102707 | R_norvegicus | Animals | mono_intergenic | H/ACA | | | intergenic |
| ENSRNOG00000060097 | LOC120098365 | R_norvegicus | Animals | mono_intergenic | H/ACA | | | intergenic |
| ENSRNOG00000060119 | LOC120100419 | R_norvegicus | Animals | mono_intergenic | H/ACA | | | intergenic |
| ENSRNOG00000060124 | LOC120101248 | R_norvegicus | Animals | mono_intronic | H/ACA | ENSRNOG00000058111 | Itga2 | protein_coding |
| ENSRNOG00000060127 | LOC120101312 | R_norvegicus | Animals | mono_intergenic | H/ACA | | | intergenic |
| ENSRNOG00000060151 | LOC120102695 | R_norvegicus | Animals | mono_intergenic | H/ACA | | | intergenic |
| ENSRNOG00000060156 | Snord28b | R_norvegicus | Animals | mono_intronic | C/D | ENSRNOG00000067892 | LOC100912041 | non_coding |
| ENSRNOG00000060186 | LOC120096812 | R_norvegicus | Animals | mono_intergenic | H/ACA | | | intergenic |
| ENSRNOG00000060204 | LOC120094462 | R_norvegicus | Animals | mono_intergenic | H/ACA | | | intergenic |
| ENSRNOG00000060216 | LOC120094885 | R_norvegicus | Animals | mono_intronic | C/D | ENSRNOG00000018273 | Ncl | protein_coding |
| ENSRNOG00000060247 | LOC120095403 | R_norvegicus | Animals | mono_intergenic | H/ACA | | | intergenic |
| ENSRNOG00000060253 | LOC120096397 | R_norvegicus | Animals | mono_intergenic | H/ACA | | | intergenic |
| ENSRNOG00000060257 | LOC120093959 | R_norvegicus | Animals | mono_intergenic | H/ACA | | | intergenic |
| ENSRNOG00000060263 | LOC120097665 | R_norvegicus | Animals | mono_intergenic | H/ACA | | | intergenic |
| ENSRNOG00000060273 | LOC120100386 | R_norvegicus | Animals | mono_intergenic | H/ACA | | | intergenic |
| ENSRNOG00000060279 | LOC120096400 | R_norvegicus | Animals | mono_intergenic | H/ACA | | | intergenic |
| ENSRNOG00000060290 | Snord87 | R_norvegicus | Animals | mono_intergenic | C/D | | | intergenic |
| ENSRNOG00000060311 | LOC120100340 | R_norvegicus | Animals | mono_intronic | C/D | ENSRNOG00000020618 | Rpl13a | protein_coding |
| ENSRNOG00000060316 | LOC120103345 | R_norvegicus | Animals | mono_intergenic | H/ACA | | | intergenic |
| ENSRNOG00000060321 | LOC120100417 | R_norvegicus | Animals | mono_intergenic | H/ACA | | | intergenic |
| ENSRNOG00000060327 | LOC120102049 | R_norvegicus | Animals | mono_intergenic | H/ACA | | | intergenic |
| ENSRNOG00000060330 | LOC120094000 | R_norvegicus | Animals | mono_intergenic | H/ACA | | | intergenic |
| ENSRNOG00000060362 | LOC120098359 | R_norvegicus | Animals | mono_intergenic | H/ACA | | | intergenic |
| ENSRNOG00000060375 | LOC120096408 | R_norvegicus | Animals | mono_intergenic | H/ACA | | | intergenic |
| ENSRNOG00000060388 | LOC120101235 | R_norvegicus | Animals | mono_intronic | C/D | ENSRNOG00000009992 | Rabggtb | protein_coding |
| ENSRNOG00000060397 | LOC120095757 | R_norvegicus | Animals | mono_intergenic | H/ACA | | | intergenic |
| ENSRNOG00000060400 | LOC120103336 | R_norvegicus | Animals | mono_intergenic | H/ACA | | | intergenic |
| ENSRNOG00000060403 | LOC120101296 | R_norvegicus | Animals | mono_intergenic | H/ACA | | | intergenic |

| | | | | | | | | |
|---|---|---|---|---|---|---|---|---|
| ENSRNOG00000060415 | LOC120102719 | R_norvegicus | Animals | mono_intergenic | H/ACA | | | intergenic |
| ENSRNOG00000060425 | LOC120103306 | R_norvegicus | Animals | mono_intergenic | H/ACA | | | intergenic |
| ENSRNOG00000060456 | LOC120093995 | R_norvegicus | Animals | mono_intergenic | H/ACA | | | intergenic |
| ENSRNOG00000060457 | LOC120102633 | R_norvegicus | Animals | mono_intronic | H/ACA | ENSRNOG00000014501 | Zfp638 | protein_coding |
| ENSRNOG00000060473 | LOC120100368 | R_norvegicus | Animals | mono_intronic | H/ACA | ENSRNOG00000015339 | Shoc2 | protein_coding |
| ENSRNOG00000060476 | LOC120095769 | R_norvegicus | Animals | mono_intergenic | H/ACA | | | intergenic |
| ENSRNOG00000060502 | Snord96a | R_norvegicus | Animals | mono_intronic | C/D | ENSRNOG00000052620 | Rack1 | protein_coding |
| ENSRNOG00000060532 | LOC120094524 | R_norvegicus | Animals | mono_intronic | C/D | ENSRNOG00000020608 | Ppan | protein_coding |
| ENSRNOG00000060537 | LOC120097616 | R_norvegicus | Animals | mono_intergenic | H/ACA | | | intergenic |
| ENSRNOG00000060539 | Snora81 | R_norvegicus | Animals | mono_intronic | H/ACA | ENSRNOG00000001815 | Eif4a2 | protein_coding |
| ENSRNOG00000060546 | LOC120099459 | R_norvegicus | Animals | mono_intergenic | H/ACA | | | intergenic |
| ENSRNOG00000060559 | Scarna10 | R_norvegicus | Animals | mono_intronic | C/D | ENSRNOG00000055300 | Ncapd2 | protein_coding |
| ENSRNOG00000060569 | LOC120102020 | R_norvegicus | Animals | mono_intronic | H/ACA | ENSRNOG00000009399 | Fads2b | protein_coding |
| ENSRNOG00000060576 | LOC120094487 | R_norvegicus | Animals | mono_intergenic | H/ACA | | | intergenic |
| ENSRNOG00000060578 | LOC120094942 | R_norvegicus | Animals | mono_intronic | C/D | ENSRNOG00000016486 | Nop58 | protein_coding |
| ENSRNOG00000060589 | LOC120093417 | R_norvegicus | Animals | mono_intronic | C/D | ENSRNOG00000026316 | Wdr43 | protein_coding |
| ENSRNOG00000060592 | LOC120102686 | R_norvegicus | Animals | mono_intergenic | H/ACA | | | intergenic |
| ENSRNOG00000060593 | LOC120098685 | R_norvegicus | Animals | mono_intergenic | H/ACA | | | intergenic |
| ENSRNOG00000060595 | LOC120097588 | R_norvegicus | Animals | mono_intergenic | H/ACA | | | intergenic |
| ENSRNOG00000060616 | LOC120095428 | R_norvegicus | Animals | mono_intronic | H/ACA | ENSRNOG00000013186 | G3bp1 | protein_coding |
| ENSRNOG00000060637 | AC112801.2 | R_norvegicus | Animals | mono_intronic | H/ACA | ENSRNOG00000017724 | Sf3b3 | protein_coding |
| ENSRNOG00000060638 | LOC120094022 | R_norvegicus | Animals | mono_intergenic | H/ACA | | | intergenic |
| ENSRNOG00000060641 | LOC120096821 | R_norvegicus | Animals | mono_intergenic | H/ACA | | | intergenic |
| ENSRNOG00000060642 | LOC120099452 | R_norvegicus | Animals | mono_intronic | H/ACA | ENSRNOG00000012756 | Slc25a43 | protein_coding |
| ENSRNOG00000060649 | LOC120096374 | R_norvegicus | Animals | intergenic_cluster | C/D | | | intergenic |
| ENSRNOG00000060653 | LOC120100457 | R_norvegicus | Animals | mono_intergenic | H/ACA | | | intergenic |
| ENSRNOG00000060660 | LOC120102642 | R_norvegicus | Animals | mono_intronic | H/ACA | ENSRNOG00000012954 | Eefsec | protein_coding |
| ENSRNOG00000060664 | LOC120103349 | R_norvegicus | Animals | mono_intronic | H/ACA | ENSRNOG00000052062 | Tut4 | protein_coding |
| ENSRNOG00000060666 | LOC120095415 | R_norvegicus | Animals | mono_intergenic | H/ACA | | | intergenic |
| ENSRNOG00000060673 | LOC120096820 | R_norvegicus | Animals | mono_intergenic | H/ACA | | | intergenic |
| ENSRNOG00000060692 | LOC120097600 | R_norvegicus | Animals | mono_intergenic | H/ACA | | | intergenic |
| ENSRNOG00000060706 | LOC120093406 | R_norvegicus | Animals | mono_intergenic | C/D | | | intergenic |
| ENSRNOG00000060710 | LOC120097612 | R_norvegicus | Animals | mono_intergenic | H/ACA | | | intergenic |
| ENSRNOG00000060712 | LOC120099515 | R_norvegicus | Animals | mono_intergenic | H/ACA | | | intergenic |
| ENSRNOG00000060721 | LOC120093411 | R_norvegicus | Animals | mono_intergenic | C/D | | | intergenic |
| ENSRNOG00000060734 | LOC120096417 | R_norvegicus | Animals | mono_intergenic | H/ACA | | | intergenic |
| ENSRNOG00000060741 | LOC120099028 | R_norvegicus | Animals | mono_intergenic | H/ACA | | | intergenic |
| ENSRNOG00000060744 | Snora44 | R_norvegicus | Animals | mono_intergenic | H/ACA | | | intergenic |
| ENSRNOG00000060748 | LOC120103314 | R_norvegicus | Animals | mono_intergenic | H/ACA | | | intergenic |
| ENSRNOG00000060754 | LOC120099484 | R_norvegicus | Animals | mono_intergenic | H/ACA | | | intergenic |
| ENSRNOG00000060757 | LOC120096813 | R_norvegicus | Animals | mono_intergenic | H/ACA | | | intergenic |
| ENSRNOG00000060760 | Snord22 | R_norvegicus | Animals | mono_intergenic | C/D | | | intergenic |
| ENSRNOG00000060763 | LOC120099496 | R_norvegicus | Animals | mono_intergenic | H/ACA | | | intergenic |

| | | | | | | | | |
|---|---|---|---|---|---|---|---|---|
| ENSRNOG00000060772 | LOC120102711 | R_norvegicus | Animals | mono_intergenic | H/ACA | | | intergenic |
| ENSRNOG00000060780 | LOC120101238 | R_norvegicus | Animals | mono_intronic | H/ACA | ENSRNOG00000027002 | Mast4 | protein_coding |
| ENSRNOG00000060795 | LOC120102703 | R_norvegicus | Animals | mono_intergenic | H/ACA | | | intergenic |
| ENSRNOG00000060810 | LOC120103283 | R_norvegicus | Animals | mono_intronic | H/ACA | ENSRNOG00000015642 | Pabpc4 | protein_coding |
| ENSRNOG00000060815 | LOC120093989 | R_norvegicus | Animals | mono_intergenic | H/ACA | | | intergenic |
| ENSRNOG00000060816 | LOC120095433 | R_norvegicus | Animals | mono_intronic | H/ACA | ENSRNOG00000030628 | Eif4a1 | protein_coding |
| ENSRNOG00000060818 | Snord37l | R_norvegicus | Animals | mono_intronic | C/D | ENSRNOG00000020266 | Eef2 | protein_coding |
| ENSRNOG00000060835 | LOC120097286 | R_norvegicus | Animals | mono_intergenic | C/D | | | intergenic |
| ENSRNOG00000060841 | LOC120094506 | R_norvegicus | Animals | mono_intronic | H/ACA | ENSRNOG00000011521 | Filip1 | protein_coding |
| ENSRNOG00000060842 | Snord12b | R_norvegicus | Animals | intergenic_cluster | C/D | | | intergenic |
| ENSRNOG00000060850 | LOC120095754 | R_norvegicus | Animals | mono_intergenic | H/ACA | | | intergenic |
| ENSRNOG00000060856 | LOC120099500 | R_norvegicus | Animals | mono_intergenic | H/ACA | | | intergenic |
| ENSRNOG00000060861 | LOC120102680 | R_norvegicus | Animals | mono_intronic | H/ACA | ENSRNOG00000007918 | Tbxas1 | protein_coding |
| ENSRNOG00000060877 | LOC120094940 | R_norvegicus | Animals | mono_intergenic | H/ACA | | | intergenic |
| ENSRNOG00000060890 | LOC120096822 | R_norvegicus | Animals | mono_intergenic | H/ACA | | | intergenic |
| ENSRNOG00000060897 | LOC120102665 | R_norvegicus | Animals | mono_intergenic | H/ACA | | | intergenic |
| ENSRNOG00000060911 | Snord111 | R_norvegicus | Animals | mono_intronic | C/D | ENSRNOG00000017724 | Sf3b3 | protein_coding |
| ENSRNOG00000060919 | Snord72 | R_norvegicus | Animals | mono_intronic | C/D | ENSRNOG00000064545 | Rpl37 | protein_coding |
| ENSRNOG00000060922 | LOC120094491 | R_norvegicus | Animals | mono_intergenic | H/ACA | | | intergenic |
| ENSRNOG00000060932 | LOC120094473 | R_norvegicus | Animals | mono_intergenic | H/ACA | | | intergenic |
| ENSRNOG00000060933 | LOC120100285 | R_norvegicus | Animals | mono_intergenic | C/D | | | intergenic |
| ENSRNOG00000060935 | LOC120097243 | R_norvegicus | Animals | mono_intergenic | H/ACA | | | intergenic |
| ENSRNOG00000060940 | LOC120097675 | R_norvegicus | Animals | mono_intronic | C/D | ENSRNOG00000016645 | Sfmbt1 | protein_coding |
| ENSRNOG00000060942 | LOC120097591 | R_norvegicus | Animals | mono_intergenic | H/ACA | | | intergenic |
| ENSRNOG00000060943 | LOC120094505 | R_norvegicus | Animals | mono_intergenic | H/ACA | | | intergenic |
| ENSRNOG00000060944 | LOC120095473 | R_norvegicus | Animals | mono_intergenic | H/ACA | | | intergenic |
| ENSRNOG00000060957 | LOC120103334 | R_norvegicus | Animals | mono_intergenic | H/ACA | | | intergenic |
| ENSRNOG00000060965 | LOC120100422 | R_norvegicus | Animals | mono_intronic | H/ACA | ENSRNOG00000063290 | Hs3st4 | protein_coding |
| ENSRNOG00000060982 | LOC120099454 | R_norvegicus | Animals | mono_intergenic | H/ACA | | | intergenic |
| ENSRNOG00000060985 | LOC120097990 | R_norvegicus | Animals | mono_intergenic | H/ACA | | | intergenic |
| ENSRNOG00000060993 | LOC120093980 | R_norvegicus | Animals | mono_intergenic | H/ACA | | | intergenic |
| ENSRNOG00000061010 | LOC120102739 | R_norvegicus | Animals | mono_intronic | C/D | ENSRNOG00000043035 | Fbxl13 | protein_coding |
| ENSRNOG00000061016 | LOC120093451 | R_norvegicus | Animals | mono_intergenic | H/ACA | | | intergenic |
| ENSRNOG00000061017 | LOC120098362 | R_norvegicus | Animals | mono_intergenic | H/ACA | | | intergenic |
| ENSRNOG00000061024 | Snord121b | R_norvegicus | Animals | mono_intronic | C/D | ENSRNOG00000052087 | Ubap2 | protein_coding |
| ENSRNOG00000061030 | LOC120098379 | R_norvegicus | Animals | mono_intergenic | H/ACA | | | intergenic |
| ENSRNOG00000061044 | LOC120103296 | R_norvegicus | Animals | mono_intergenic | H/ACA | | | intergenic |
| ENSRNOG00000061079 | LOC120093986 | R_norvegicus | Animals | mono_intronic | H/ACA | ENSRNOG00000043390 | Samd12 | protein_coding |
| ENSRNOG00000061087 | Snora26l1 | R_norvegicus | Animals | mono_intronic | H/ACA | ENSRNOG00000005101 | Mia2 | protein_coding |
| ENSRNOG00000061090 | LOC120097609 | R_norvegicus | Animals | mono_intergenic | H/ACA | | | intergenic |
| ENSRNOG00000061098 | LOC120103298 | R_norvegicus | Animals | mono_intergenic | H/ACA | | | intergenic |
| ENSRNOG00000061103 | LOC120102709 | R_norvegicus | Animals | mono_intergenic | H/ACA | | | intergenic |
| ENSRNOG00000061115 | LOC120098385 | R_norvegicus | Animals | mono_intergenic | H/ACA | | | intergenic |

| | | | | | | | | |
|---|---|---|---|---|---|---|---|---|
| ENSRNOG00000061119 | LOC120093047 | R_norvegicus | Animals | mono_intronic | H/ACA | ENSRNOG00000054331 | Wdpcp | protein_coding |
| ENSRNOG00000061123 | LOC120101328 | R_norvegicus | Animals | mono_intergenic | H/ACA | | | intergenic |
| ENSRNOG00000061138 | Snord30 | R_norvegicus | Animals | mono_intronic | C/D | ENSRNOG00000067892 | LOC100912041 | non_coding |
| ENSRNOG00000061145 | LOC120097231 | R_norvegicus | Animals | mono_intergenic | H/ACA | | | intergenic |
| ENSRNOG00000061149 | LOC120103353 | R_norvegicus | Animals | mono_intergenic | C/D | | | intergenic |
| ENSRNOG00000061150 | LOC120094923 | R_norvegicus | Animals | mono_intergenic | H/ACA | | | intergenic |
| ENSRNOG00000061186 | Snord123 | R_norvegicus | Animals | mono_intergenic | C/D | | | intergenic |
| ENSRNOG00000061190 | LOC120101319 | R_norvegicus | Animals | mono_intronic | H/ACA | ENSRNOG00000015226 | Man1a2 | protein_coding |
| ENSRNOG00000061195 | LOC120099449 | R_norvegicus | Animals | mono_intronic | H/ACA | ENSRNOG00000043348 | Rpl39-ps13 | protein_coding |
| ENSRNOG00000061206 | LOC120093448 | R_norvegicus | Animals | mono_intergenic | H/ACA | | | intergenic |
| ENSRNOG00000061208 | LOC120094920 | R_norvegicus | Animals | mono_intergenic | H/ACA | | | intergenic |
| ENSRNOG00000061234 | LOC120102731 | R_norvegicus | Animals | mono_intergenic | H/ACA | | | intergenic |
| ENSRNOG00000061257 | LOC120099521 | R_norvegicus | Animals | mono_intergenic | H/ACA | | | intergenic |
| ENSRNOG00000061287 | LOC120097236 | R_norvegicus | Animals | mono_intergenic | H/ACA | | | intergenic |
| ENSRNOG00000061296 | LOC120098386 | R_norvegicus | Animals | mono_intergenic | H/ACA | | | intergenic |
| ENSRNOG00000061323 | LOC120094912 | R_norvegicus | Animals | mono_intergenic | H/ACA | | | intergenic |
| ENSRNOG00000061325 | LOC120098689 | R_norvegicus | Animals | mono_intergenic | H/ACA | | | intergenic |
| ENSRNOG00000061330 | LOC120102659 | R_norvegicus | Animals | mono_intronic | H/ACA | ENSRNOG00000043035 | Fbxl13 | protein_coding |
| ENSRNOG00000061338 | LOC120100276 | R_norvegicus | Animals | mono_intergenic | C/D | | | intergenic |
| ENSRNOG00000061349 | Snora24 | R_norvegicus | Animals | mono_intergenic | H/ACA | | | intergenic |
| ENSRNOG00000061360 | LOC120099037 | R_norvegicus | Animals | mono_intergenic | C/D | | | intergenic |
| ENSRNOG00000061361 | Snord46 | R_norvegicus | Animals | mono_intronic | C/D | ENSRNOG00000054626 | Rps8 | protein_coding |
| ENSRNOG00000061362 | LOC120101247 | R_norvegicus | Animals | mono_intergenic | H/ACA | | | intergenic |
| ENSRNOG00000061393 | LOC120095734 | R_norvegicus | Animals | mono_intergenic | H/ACA | | | intergenic |
| ENSRNOG00000061396 | LOC120099025 | R_norvegicus | Animals | mono_intergenic | H/ACA | | | intergenic |
| ENSRNOG00000061412 | LOC120100290 | R_norvegicus | Animals | mono_intergenic | C/D | | | intergenic |
| ENSRNOG00000061427 | LOC120101232 | R_norvegicus | Animals | mono_intergenic | C/D | | | intergenic |
| ENSRNOG00000061433 | Snord113l2 | R_norvegicus | Animals | mono_intergenic | C/D | | | intergenic |
| ENSRNOG00000061440 | LOC120097670 | R_norvegicus | Animals | mono_intronic | H/ACA | ENSRNOG00000019811 | Timm23 | protein_coding |
| ENSRNOG00000061471 | Snord48 | R_norvegicus | Animals | mono_intergenic | C/D | | | intergenic |
| ENSRNOG00000061477 | LOC120096833 | R_norvegicus | Animals | mono_intergenic | H/ACA | | | intergenic |
| ENSRNOG00000061486 | LOC120095402 | R_norvegicus | Animals | mono_intergenic | H/ACA | | | intergenic |
| ENSRNOG00000061512 | LOC120094929 | R_norvegicus | Animals | mono_intergenic | H/ACA | | | intergenic |
| ENSRNOG00000061517 | LOC120102051 | R_norvegicus | Animals | mono_intergenic | H/ACA | | | intergenic |
| ENSRNOG00000061534 | LOC120101352 | R_norvegicus | Animals | mono_intergenic | H/ACA | | | intergenic |
| ENSRNOG00000061536 | LOC120103240 | R_norvegicus | Animals | intergenic_cluster | H/ACA | | | intergenic |
| ENSRNOG00000061550 | LOC120099016 | R_norvegicus | Animals | mono_intergenic | H/ACA | | | intergenic |
| ENSRNOG00000061551 | LOC120096413 | R_norvegicus | Animals | intergenic_cluster | H/ACA | | | intergenic |
| ENSRNOG00000061554 | LOC120103260 | R_norvegicus | Animals | mono_intronic | C/D | ENSRNOG00000011709 | Pum1 | protein_coding |
| ENSRNOG00000061556 | LOC120100477 | R_norvegicus | Animals | mono_intronic | H/ACA | ENSRNOG00000023296 | Wtip | protein_coding |
| ENSRNOG00000061565 | LOC120101361 | R_norvegicus | Animals | mono_intergenic | H/ACA | | | intergenic |
| ENSRNOG00000061566 | LOC120096819 | R_norvegicus | Animals | mono_intergenic | H/ACA | | | intergenic |
| ENSRNOG00000061585 | LOC120102676 | R_norvegicus | Animals | mono_intergenic | H/ACA | | | intergenic |

| | | | | | | | | |
|---|---|---|---|---|---|---|---|---|
| ENSRNOG00000061586 | LOC120099462 | R_norvegicus | Animals | mono_intronic | H/ACA | ENSRNOG00000060869 | Irak1 | protein_coding |
| ENSRNOG00000061589 | LOC120103318 | R_norvegicus | Animals | mono_intergenic | H/ACA | | | intergenic |
| ENSRNOG00000061593 | LOC120100349 | R_norvegicus | Animals | mono_intergenic | H/ACA | | | intergenic |
| ENSRNOG00000061617 | LOC120102661 | R_norvegicus | Animals | intronic_cluster | H/ACA | ENSRNOG00000012337 | Pde1c | protein_coding |
| ENSRNOG00000061627 | LOC120098383 | R_norvegicus | Animals | mono_intergenic | H/ACA | | | intergenic |
| ENSRNOG00000061634 | LOC120097611 | R_norvegicus | Animals | mono_intergenic | H/ACA | | | intergenic |
| ENSRNOG00000061641 | LOC120097209 | R_norvegicus | Animals | mono_intronic | H/ACA | ENSRNOG00000071105 | Gpc5 | protein_coding |
| ENSRNOG00000061642 | LOC120099070 | R_norvegicus | Animals | mono_intergenic | H/ACA | | | intergenic |
| ENSRNOG00000061657 | LOC120093054 | R_norvegicus | Animals | mono_intronic | H/ACA | ENSRNOG00000010117 | Eif3a | protein_coding |
| ENSRNOG00000061662 | LOC120103239 | R_norvegicus | Animals | intergenic_cluster | H/ACA | | | intergenic |
| ENSRNOG00000061671 | LOC120095458 | R_norvegicus | Animals | mono_intronic | H/ACA | ENSRNOG00000003276 | Myo1d | protein_coding |
| ENSRNOG00000061678 | Snord100 | R_norvegicus | Animals | mono_intronic | C/D | ENSRNOG00000016411 | Rps12 | protein_coding |
| ENSRNOG00000061680 | LOC120093043 | R_norvegicus | Animals | mono_intronic | H/ACA | ENSRNOG00000016269 | Rpl7l1 | protein_coding |
| ENSRNOG00000061686 | LOC120099443 | R_norvegicus | Animals | mono_intergenic | H/ACA | | | intergenic |
| ENSRNOG00000061688 | LOC120094472 | R_norvegicus | Animals | mono_exonic | H/ACA | ENSRNOG00000010921 | Taf1d | protein_coding |
| ENSRNOG00000061689 | LOC120102061 | R_norvegicus | Animals | mono_intergenic | H/ACA | | | intergenic |
| ENSRNOG00000061736 | LOC120095448 | R_norvegicus | Animals | mono_intergenic | H/ACA | | | intergenic |
| ENSRNOG00000061755 | LOC120100355 | R_norvegicus | Animals | mono_intronic | H/ACA | ENSRNOG00000013351 | Stxbp5 | protein_coding |
| ENSRNOG00000061758 | LOC120098004 | R_norvegicus | Animals | mono_intergenic | H/ACA | | | intergenic |
| ENSRNOG00000061759 | LOC120097246 | R_norvegicus | Animals | mono_intergenic | H/ACA | | | intergenic |
| ENSRNOG00000061762 | LOC120094445 | R_norvegicus | Animals | mono_intronic | H/ACA | ENSRNOG00000048919 | Iho1 | protein_coding |
| ENSRNOG00000061767 | LOC120099056 | R_norvegicus | Animals | mono_intronic | C/D | ENSRNOG00000000841 | Ddx39b | protein_coding |
| ENSRNOG00000061787 | LOC120099501 | R_norvegicus | Animals | mono_intergenic | H/ACA | | | intergenic |
| ENSRNOG00000061788 | Scarna18 | R_norvegicus | Animals | mono_intronic | H/ACA | ENSRNOG00000016686 | Tmem167a | protein_coding |
| ENSRNOG00000061790 | LOC120097993 | R_norvegicus | Animals | mono_intergenic | H/ACA | | | intergenic |
| ENSRNOG00000061806 | LOC120100385 | R_norvegicus | Animals | mono_intergenic | H/ACA | | | intergenic |
| ENSRNOG00000061810 | LOC120102684 | R_norvegicus | Animals | mono_intergenic | H/ACA | | | intergenic |
| ENSRNOG00000061817 | LOC120096415 | R_norvegicus | Animals | mono_intergenic | H/ACA | | | intergenic |
| ENSRNOG00000061820 | LOC120094926 | R_norvegicus | Animals | mono_intergenic | H/ACA | | | intergenic |
| ENSRNOG00000061830 | LOC120093457 | R_norvegicus | Animals | mono_intergenic | H/ACA | | | intergenic |
| ENSRNOG00000061838 | LOC120097235 | R_norvegicus | Animals | mono_intergenic | H/ACA | | | intergenic |
| ENSRNOG00000061849 | LOC120099030 | R_norvegicus | Animals | mono_intronic | C/D | ENSRNOG00000053288 | Ank3 | protein_coding |
| ENSRNOG00000061858 | LOC120094500 | R_norvegicus | Animals | mono_intergenic | H/ACA | | | intergenic |
| ENSRNOG00000061864 | LOC120102710 | R_norvegicus | Animals | mono_intergenic | H/ACA | | | intergenic |
| ENSRNOG00000061866 | LOC120101327 | R_norvegicus | Animals | mono_intergenic | H/ACA | | | intergenic |
| ENSRNOG00000061867 | LOC120094882 | R_norvegicus | Animals | mono_intergenic | H/ACA | | | intergenic |
| ENSRNOG00000061894 | LOC120099508 | R_norvegicus | Animals | mono_intergenic | H/ACA | | | intergenic |
| ENSRNOG00000061903 | LOC120093425 | R_norvegicus | Animals | mono_intronic | H/ACA | ENSRNOG00000052418 | Cpsf3 | protein_coding |
| ENSRNOG00000061907 | LOC120097671 | R_norvegicus | Animals | mono_intergenic | H/ACA | | | intergenic |
| ENSRNOG00000062322 | LOC120098733 | R_norvegicus | Animals | mono_intergenic | C/D | | | intergenic |
| ENSRNOG00000062330 | Snord62a | R_norvegicus | Animals | mono_intronic | C/D | ENSRNOG00000010217 | Prrc2b | protein_coding |
| ENSRNOG00000062332 | LOC120094517 | R_norvegicus | Animals | mono_intergenic | H/ACA | | | intergenic |
| ENSRNOG00000062338 | LOC120100301 | R_norvegicus | Animals | mono_intergenic | C/D | | | intergenic |

| | | | | | | | | |
|---|---|---|---|---|---|---|---|---|
| ENSRNOG00000062341 | LOC120101286 | R_norvegicus | Animals | mono_intergenic | H/ACA | | | intergenic |
| ENSRNOG00000062355 | LOC120098735 | R_norvegicus | Animals | mono_intergenic | C/D | | | intergenic |
| ENSRNOG00000062380 | LOC120102771 | R_norvegicus | Animals | mono_intronic | C/D | ENSRNOG00000009184 | Foxp1 | protein_coding |
| ENSRNOG00000062382 | LOC120098688 | R_norvegicus | Animals | mono_intergenic | H/ACA | | | intergenic |
| ENSRNOG00000062402 | LOC120093344 | R_norvegicus | Animals | mono_intergenic | C/D | | | intergenic |
| ENSRNOG00000062417 | LOC120102691 | R_norvegicus | Animals | mono_intergenic | H/ACA | | | intergenic |
| ENSRNOG00000062488 | LOC120101272 | R_norvegicus | Animals | mono_intergenic | H/ACA | | | intergenic |
| ENSRNOG00000062499 | LOC120100326 | R_norvegicus | Animals | mono_intergenic | C/D | | | intergenic |
| ENSRNOG00000062515 | LOC120102653 | R_norvegicus | Animals | mono_intergenic | H/ACA | | | intergenic |
| ENSRNOG00000062520 | LOC120093476 | R_norvegicus | Animals | mono_intronic | H/ACA | ENSRNOG00000034190 | Ighm | protein_coding |
| ENSRNOG00000062549 | LOC120095447 | R_norvegicus | Animals | mono_intergenic | H/ACA | | | intergenic |
| ENSRNOG00000062566 | LOC120095779 | R_norvegicus | Animals | mono_intergenic | C/D | | | intergenic |
| ENSRNOG00000062570 | LOC120098011 | R_norvegicus | Animals | mono_intergenic | C/D | | | intergenic |
| ENSRNOG00000062574 | LOC120093321 | R_norvegicus | Animals | mono_intergenic | C/D | | | intergenic |
| ENSRNOG00000062579 | LOC120100243 | R_norvegicus | Animals | mono_intergenic | C/D | | | intergenic |
| ENSRNOG00000062601 | LOC120096393 | R_norvegicus | Animals | mono_intergenic | H/ACA | | | intergenic |
| ENSRNOG00000062614 | LOC120093355 | R_norvegicus | Animals | mono_intergenic | C/D | | | intergenic |
| ENSRNOG00000062732 | LOC120093340 | R_norvegicus | Animals | mono_intergenic | C/D | | | intergenic |
| ENSRNOG00000062757 | LOC120095740 | R_norvegicus | Animals | mono_intergenic | H/ACA | | | intergenic |
| ENSRNOG00000062767 | LOC120093437 | R_norvegicus | Animals | mono_intergenic | H/ACA | | | intergenic |
| ENSRNOG00000062770 | LOC120096426 | R_norvegicus | Animals | mono_intergenic | C/D | | | intergenic |
| ENSRNOG00000062771 | LOC120100262 | R_norvegicus | Animals | intergenic_cluster | C/D | | | intergenic |
| ENSRNOG00000062773 | LOC120096106 | R_norvegicus | Animals | mono_intronic | H/ACA | ENSRNOG00000008673 | Arpc3 | protein_coding |
| ENSRNOG00000062774 | LOC120097244 | R_norvegicus | Animals | mono_intergenic | H/ACA | | | intergenic |
| ENSRNOG00000062785 | LOC120100482 | R_norvegicus | Animals | mono_intergenic | C/D | | | intergenic |
| ENSRNOG00000062788 | LOC120093366 | R_norvegicus | Animals | mono_intergenic | C/D | | | intergenic |
| ENSRNOG00000062791 | LOC120100287 | R_norvegicus | Animals | mono_intergenic | C/D | | | intergenic |
| ENSRNOG00000062832 | LOC120097229 | R_norvegicus | Animals | mono_intergenic | H/ACA | | | intergenic |
| ENSRNOG00000062847 | LOC120100246 | R_norvegicus | Animals | mono_intergenic | C/D | | | intergenic |
| ENSRNOG00000062850 | LOC120098737 | R_norvegicus | Animals | mono_intergenic | C/D | | | intergenic |
| ENSRNOG00000062857 | LOC120100299 | R_norvegicus | Animals | mono_intergenic | C/D | | | intergenic |
| ENSRNOG00000062886 | LOC120100313 | R_norvegicus | Animals | mono_intronic | C/D | ENSRNOG00000065457 | | non_coding |
| ENSRNOG00000062891 | LOC120103276 | R_norvegicus | Animals | mono_intergenic | H/ACA | | | intergenic |
| ENSRNOG00000062905 | LOC120100269 | R_norvegicus | Animals | mono_intergenic | C/D | | | intergenic |
| ENSRNOG00000062908 | LOC120097238 | R_norvegicus | Animals | mono_intergenic | H/ACA | | | intergenic |
| ENSRNOG00000062913 | LOC120096797 | R_norvegicus | Animals | mono_intergenic | H/ACA | | | intergenic |
| ENSRNOG00000062921 | LOC120102081 | R_norvegicus | Animals | mono_intergenic | H/ACA | | | intergenic |
| ENSRNOG00000062940 | LOC120097978 | R_norvegicus | Animals | mono_exonic | H/ACA | ENSRNOG00000067254 | | non_coding |
| ENSRNOG00000062950 | LOC120095778 | R_norvegicus | Animals | mono_intergenic | C/D | | | intergenic |
| ENSRNOG00000062991 | LOC120093329 | R_norvegicus | Animals | mono_intergenic | C/D | | | intergenic |
| ENSRNOG00000063078 | LOC120097985 | R_norvegicus | Animals | mono_intergenic | H/ACA | | | intergenic |
| ENSRNOG00000063090 | LOC120096436 | R_norvegicus | Animals | mono_intergenic | C/D | | | intergenic |
| ENSRNOG00000063107 | LOC120099482 | R_norvegicus | Animals | mono_intergenic | H/ACA | | | intergenic |

| | | | | | | | | |
|---|---|---|---|---|---|---|---|---|
| ENSRNOG00000063116 | Snora2l1 | R_norvegicus | Animals | mono_intronic | H/ACA | ENSRNOG00000060185 | Kansl2 | protein_coding |
| ENSRNOG00000063140 | LOC120100260 | R_norvegicus | Animals | mono_intergenic | C/D | | | intergenic |
| ENSRNOG00000063143 | LOC120102028 | R_norvegicus | Animals | mono_intergenic | H/ACA | | | intergenic |
| ENSRNOG00000063147 | LOC120094903 | R_norvegicus | Animals | mono_intergenic | H/ACA | | | intergenic |
| ENSRNOG00000063162 | LOC120094547 | R_norvegicus | Animals | mono_intronic | C/D | ENSRNOG00000034066 | Hspa8 | protein_coding |
| ENSRNOG00000063240 | LOC120097606 | R_norvegicus | Animals | mono_intronic | H/ACA | ENSRNOG00000012802 | Tenm3 | protein_coding |
| ENSRNOG00000063267 | LOC120098350 | R_norvegicus | Animals | mono_intronic | H/ACA | ENSRNOG00000028105 | Hars1 | protein_coding |
| ENSRNOG00000063269 | LOC120095490 | R_norvegicus | Animals | intronic_cluster | C/D | ENSRNOG00000023698 | Tex14 | protein_coding |
| ENSRNOG00000063270 | LOC120094477 | R_norvegicus | Animals | mono_intergenic | H/ACA | | | intergenic |
| ENSRNOG00000063274 | LOC120102025 | R_norvegicus | Animals | mono_intergenic | H/ACA | | | intergenic |
| ENSRNOG00000063296 | LOC120099524 | R_norvegicus | Animals | mono_intergenic | H/ACA | | | intergenic |
| ENSRNOG00000063303 | LOC120100332 | R_norvegicus | Animals | mono_intergenic | C/D | | | intergenic |
| ENSRNOG00000063321 | LOC120100445 | R_norvegicus | Animals | mono_intergenic | H/ACA | | | intergenic |
| ENSRNOG00000063324 | Snora20 | R_norvegicus | Animals | mono_intronic | H/ACA | ENSRNOG00000014160 | Tcp1 | protein_coding |
| ENSRNOG00000063343 | LOC120099039 | R_norvegicus | Animals | mono_intronic | C/D | ENSRNOG00000000368 | Grik2 | protein_coding |
| ENSRNOG00000063369 | LOC120097586 | R_norvegicus | Animals | mono_intergenic | H/ACA | | | intergenic |
| ENSRNOG00000063396 | LOC120100238 | R_norvegicus | Animals | mono_intergenic | C/D | | | intergenic |
| ENSRNOG00000063410 | LOC120100483 | R_norvegicus | Animals | mono_intergenic | C/D | | | intergenic |
| ENSRNOG00000063420 | LOC120097265 | R_norvegicus | Animals | mono_intergenic | H/ACA | | | intergenic |
| ENSRNOG00000063424 | LOC120098684 | R_norvegicus | Animals | mono_intergenic | H/ACA | | | intergenic |
| ENSRNOG00000063473 | LOC120102008 | R_norvegicus | Animals | mono_intergenic | H/ACA | | | intergenic |
| ENSRNOG00000063504 | LOC120103259 | R_norvegicus | Animals | mono_intronic | C/D | ENSRNOG00000011709 | Pum1 | protein_coding |
| ENSRNOG00000063530 | LOC120100295 | R_norvegicus | Animals | mono_intergenic | C/D | | | intergenic |
| ENSRNOG00000063542 | LOC120093363 | R_norvegicus | Animals | mono_intergenic | C/D | | | intergenic |
| ENSRNOG00000063558 | LOC120093386 | R_norvegicus | Animals | mono_intergenic | C/D | | | intergenic |
| ENSRNOG00000063561 | LOC120097250 | R_norvegicus | Animals | mono_intergenic | H/ACA | | | intergenic |
| ENSRNOG00000063624 | LOC120100448 | R_norvegicus | Animals | mono_intergenic | H/ACA | | | intergenic |
| ENSRNOG00000063628 | LOC120093998 | R_norvegicus | Animals | mono_intergenic | H/ACA | | | intergenic |
| ENSRNOG00000063641 | LOC120100292 | R_norvegicus | Animals | mono_intergenic | C/D | | | intergenic |
| ENSRNOG00000063652 | LOC120097251 | R_norvegicus | Animals | mono_intergenic | H/ACA | | | intergenic |
| ENSRNOG00000063661 | LOC120093332 | R_norvegicus | Animals | mono_intergenic | C/D | | | intergenic |
| ENSRNOG00000063666 | LOC120102664 | R_norvegicus | Animals | mono_intergenic | H/ACA | | | intergenic |
| ENSRNOG00000063743 | LOC120100253 | R_norvegicus | Animals | mono_intergenic | C/D | | | intergenic |
| ENSRNOG00000063750 | LOC120093407 | R_norvegicus | Animals | intergenic_cluster | C/D | | | intergenic |
| ENSRNOG00000063770 | LOC120096107 | R_norvegicus | Animals | mono_intronic | H/ACA | ENSRNOG00000061346 | B3glct | protein_coding |
| ENSRNOG00000063774 | LOC120097220 | R_norvegicus | Animals | mono_intergenic | H/ACA | | | intergenic |
| ENSRNOG00000063782 | LOC120101983 | R_norvegicus | Animals | mono_intronic | C/D | ENSRNOG00000070974 | Rpl7a | protein_coding |
| ENSRNOG00000063789 | LOC120093382 | R_norvegicus | Animals | mono_intergenic | C/D | | | intergenic |
| ENSRNOG00000063802 | LOC120100330 | R_norvegicus | Animals | mono_intergenic | C/D | | | intergenic |
| ENSRNOG00000063809 | LOC120101228 | R_norvegicus | Animals | mono_intergenic | C/D | | | intergenic |
| ENSRNOG00000063853 | LOC120102120 | R_norvegicus | Animals | mono_exonic | C/D | ENSRNOG00000008868 | Dusp19 | protein_coding |
| ENSRNOG00000063886 | LOC120102713 | R_norvegicus | Animals | mono_intergenic | H/ACA | | | intergenic |
| ENSRNOG00000063896 | LOC120102060 | R_norvegicus | Animals | mono_intergenic | H/ACA | | | intergenic |

| | | | | | | | | |
|---|---|---|---|---|---|---|---|---|
| ENSRNOG00000063926 | LOC120099483 | R_norvegicus | Animals | mono_intergenic | H/ACA | | | intergenic |
| ENSRNOG00000063928 | LOC120093389 | R_norvegicus | Animals | mono_intergenic | C/D | | | intergenic |
| ENSRNOG00000063955 | LOC120100271 | R_norvegicus | Animals | mono_intergenic | C/D | | | intergenic |
| ENSRNOG00000063962 | LOC120093384 | R_norvegicus | Animals | mono_intergenic | C/D | | | intergenic |
| ENSRNOG00000063998 | LOC120093322 | R_norvegicus | Animals | mono_intergenic | C/D | | | intergenic |
| ENSRNOG00000064016 | LOC120100305 | R_norvegicus | Animals | mono_intergenic | C/D | | | intergenic |
| ENSRNOG00000064029 | LOC120096801 | R_norvegicus | Animals | mono_intergenic | H/ACA | | | intergenic |
| ENSRNOG00000064056 | LOC120103290 | R_norvegicus | Animals | mono_intergenic | H/ACA | | | intergenic |
| ENSRNOG00000064098 | LOC120100236 | R_norvegicus | Animals | mono_intergenic | C/D | | | intergenic |
| ENSRNOG00000064115 | LOC120100382 | R_norvegicus | Animals | mono_intergenic | H/ACA | | | intergenic |
| ENSRNOG00000064221 | LOC120093402 | R_norvegicus | Animals | mono_intergenic | C/D | | | intergenic |
| ENSRNOG00000064250 | LOC120103277 | R_norvegicus | Animals | mono_intergenic | H/ACA | | | intergenic |
| ENSRNOG00000064253 | LOC120093378 | R_norvegicus | Animals | mono_intergenic | C/D | | | intergenic |
| ENSRNOG00000064261 | LOC120101294 | R_norvegicus | Animals | mono_intergenic | H/ACA | | | intergenic |
| ENSRNOG00000064263 | LOC120093395 | R_norvegicus | Animals | mono_intergenic | C/D | | | intergenic |
| ENSRNOG00000064270 | LOC120102733 | R_norvegicus | Animals | mono_intergenic | H/ACA | | | intergenic |
| ENSRNOG00000064295 | LOC120095431 | R_norvegicus | Animals | mono_intergenic | H/ACA | | | intergenic |
| ENSRNOG00000064297 | LOC120100426 | R_norvegicus | Animals | mono_intergenic | H/ACA | | | intergenic |
| ENSRNOG00000064305 | LOC120097212 | R_norvegicus | Animals | mono_intronic | H/ACA | ENSRNOG00000009871 | Piwil2 | protein_coding |
| ENSRNOG00000064306 | LOC120100242 | R_norvegicus | Animals | mono_intergenic | C/D | | | intergenic |
| ENSRNOG00000064313 | LOC120100310 | R_norvegicus | Animals | mono_intergenic | C/D | | | intergenic |
| ENSRNOG00000064326 | LOC120102032 | R_norvegicus | Animals | mono_intergenic | H/ACA | | | intergenic |
| ENSRNOG00000064356 | LOC120093326 | R_norvegicus | Animals | mono_intergenic | C/D | | | intergenic |
| ENSRNOG00000064367 | LOC120100237 | R_norvegicus | Animals | mono_intergenic | C/D | | | intergenic |
| ENSRNOG00000064386 | LOC120102038 | R_norvegicus | Animals | mono_intergenic | H/ACA | | | intergenic |
| ENSRNOG00000064388 | LOC120099475 | R_norvegicus | Animals | mono_intergenic | H/ACA | | | intergenic |
| ENSRNOG00000064394 | LOC120100309 | R_norvegicus | Animals | mono_intergenic | C/D | | | intergenic |
| ENSRNOG00000064403 | LOC120103299 | R_norvegicus | Animals | mono_intergenic | H/ACA | | | intergenic |
| ENSRNOG00000064435 | LOC120100317 | R_norvegicus | Animals | intronic_cluster | C/D | ENSRNOG00000065457 | | non_coding |
| ENSRNOG00000064439 | LOC120094484 | R_norvegicus | Animals | mono_intergenic | H/ACA | | | intergenic |
| ENSRNOG00000064450 | LOC120093388 | R_norvegicus | Animals | mono_intergenic | C/D | | | intergenic |
| ENSRNOG00000064458 | LOC120101371 | R_norvegicus | Animals | mono_intergenic | C/D | | | intergenic |
| ENSRNOG00000064465 | LOC120094466 | R_norvegicus | Animals | mono_intronic | H/ACA | ENSRNOG00000055251 | Prtg | protein_coding |
| ENSRNOG00000064468 | LOC120102029 | R_norvegicus | Animals | mono_intergenic | H/ACA | | | intergenic |
| ENSRNOG00000064470 | LOC120100261 | R_norvegicus | Animals | mono_intergenic | C/D | | | intergenic |
| ENSRNOG00000064476 | LOC120097228 | R_norvegicus | Animals | mono_intergenic | H/ACA | | | intergenic |
| ENSRNOG00000064494 | LOC120094003 | R_norvegicus | Animals | mono_intergenic | H/ACA | | | intergenic |
| ENSRNOG00000064509 | Snord64 | R_norvegicus | Animals | mono_exonic | C/D | ENSRNOG00000070519 | | non_coding |
| ENSRNOG00000064518 | LOC120093970 | R_norvegicus | Animals | mono_intergenic | H/ACA | | | intergenic |
| ENSRNOG00000064522 | LOC120100251 | R_norvegicus | Animals | mono_intergenic | C/D | | | intergenic |
| ENSRNOG00000064543 | LOC120099479 | R_norvegicus | Animals | mono_intergenic | H/ACA | | | intergenic |
| ENSRNOG00000064560 | LOC120097599 | R_norvegicus | Animals | mono_intergenic | H/ACA | | | intergenic |
| ENSRNOG00000064566 | LOC120102682 | R_norvegicus | Animals | mono_intergenic | H/ACA | | | intergenic |

| | | | | | | | | |
|---|---|---|---|---|---|---|---|---|
| ENSRNOG00000064599 | LOC120096796 | R_norvegicus | Animals | mono_intergenic | H/ACA | | | intergenic |
| ENSRNOG00000064600 | LOC120102101 | R_norvegicus | Animals | mono_intergenic | C/D | | | intergenic |
| ENSRNOG00000064631 | LOC120094483 | R_norvegicus | Animals | mono_intergenic | H/ACA | | | intergenic |
| ENSRNOG00000064632 | LOC120102663 | R_norvegicus | Animals | mono_intergenic | H/ACA | | | intergenic |
| ENSRNOG00000064681 | LOC120100249 | R_norvegicus | Animals | mono_intergenic | C/D | | | intergenic |
| ENSRNOG00000064686 | LOC120102650 | R_norvegicus | Animals | mono_intergenic | H/ACA | | | intergenic |
| ENSRNOG00000064725 | LOC120095741 | R_norvegicus | Animals | mono_intergenic | H/ACA | | | intergenic |
| ENSRNOG00000064736 | LOC120098389 | R_norvegicus | Animals | mono_intergenic | H/ACA | | | intergenic |
| ENSRNOG00000064740 | LOC120097594 | R_norvegicus | Animals | mono_intergenic | H/ACA | | | intergenic |
| ENSRNOG00000064758 | LOC120098417 | R_norvegicus | Animals | mono_intergenic | C/D | | | intergenic |
| ENSRNOG00000064761 | LOC120102667 | R_norvegicus | Animals | mono_intergenic | H/ACA | | | intergenic |
| ENSRNOG00000064763 | LOC120093972 | R_norvegicus | Animals | mono_intergenic | H/ACA | | | intergenic |
| ENSRNOG00000064765 | Snora30 | R_norvegicus | Animals | mono_intronic | H/ACA | ENSRNOG00000050282 | Srcap | protein_coding |
| ENSRNOG00000064768 | LOC120098363 | R_norvegicus | Animals | mono_intergenic | H/ACA | | | intergenic |
| ENSRNOG00000064802 | LOC120096838 | R_norvegicus | Animals | mono_intergenic | C/D | | | intergenic |
| ENSRNOG00000064817 | LOC120100265 | R_norvegicus | Animals | mono_intergenic | C/D | | | intergenic |
| ENSRNOG00000064837 | LOC120094478 | R_norvegicus | Animals | mono_intergenic | H/ACA | | | intergenic |
| ENSRNOG00000064859 | LOC120100323 | R_norvegicus | Animals | mono_intergenic | C/D | | | intergenic |
| ENSRNOG00000064924 | LOC120101263 | R_norvegicus | Animals | mono_intergenic | H/ACA | | | intergenic |
| ENSRNOG00000064927 | LOC120093365 | R_norvegicus | Animals | mono_intergenic | C/D | | | intergenic |
| ENSRNOG00000064928 | LOC120098375 | R_norvegicus | Animals | mono_intronic | H/ACA | ENSRNOG00000030321 | LOC502176 | protein_coding |
| ENSRNOG00000064960 | LOC120100254 | R_norvegicus | Animals | mono_intergenic | C/D | | | intergenic |
| ENSRNOG00000064967 | LOC120103295 | R_norvegicus | Animals | mono_intergenic | H/ACA | | | intergenic |
| ENSRNOG00000064979 | LOC120103291 | R_norvegicus | Animals | mono_intergenic | H/ACA | | | intergenic |
| ENSRNOG00000065002 | LOC120103342 | R_norvegicus | Animals | mono_intergenic | H/ACA | | | intergenic |
| ENSRNOG00000065008 | LOC120102721 | R_norvegicus | Animals | mono_intergenic | H/ACA | | | intergenic |
| ENSRNOG00000065018 | LOC120093494 | R_norvegicus | Animals | mono_intergenic | C/D | | | intergenic |
| ENSRNOG00000065039 | LOC120093351 | R_norvegicus | Animals | mono_intergenic | C/D | | | intergenic |
| ENSRNOG00000065052 | LOC120100259 | R_norvegicus | Animals | mono_intergenic | C/D | | | intergenic |
| ENSRNOG00000065069 | LOC120096837 | R_norvegicus | Animals | mono_intergenic | C/D | | | intergenic |
| ENSRNOG00000065074 | LOC120097986 | R_norvegicus | Animals | mono_intronic | H/ACA | ENSRNOG00000024832 | Gpr158 | protein_coding |
| ENSRNOG00000065134 | LOC120101258 | R_norvegicus | Animals | mono_intergenic | H/ACA | | | intergenic |
| ENSRNOG00000065136 | LOC120100303 | R_norvegicus | Animals | mono_intergenic | C/D | | | intergenic |
| ENSRNOG00000065172 | LOC120100314 | R_norvegicus | Animals | intronic_cluster | C/D | ENSRNOG00000065457 | | non_coding |
| ENSRNOG00000065224 | LOC120099546 | R_norvegicus | Animals | mono_intergenic | C/D | | | intergenic |
| ENSRNOG00000065233 | LOC120097266 | R_norvegicus | Animals | mono_intergenic | H/ACA | | | intergenic |
| ENSRNOG00000065309 | LOC120098702 | R_norvegicus | Animals | mono_intergenic | H/ACA | | | intergenic |
| ENSRNOG00000065315 | LOC120093348 | R_norvegicus | Animals | mono_intergenic | C/D | | | intergenic |
| ENSRNOG00000065337 | LOC120101304 | R_norvegicus | Animals | mono_intergenic | H/ACA | | | intergenic |
| ENSRNOG00000065344 | LOC120102099 | R_norvegicus | Animals | mono_intergenic | C/D | | | intergenic |
| ENSRNOG00000065349 | LOC120102700 | R_norvegicus | Animals | mono_intergenic | H/ACA | | | intergenic |
| ENSRNOG00000065361 | Snora52 | R_norvegicus | Animals | mono_intronic | H/ACA | ENSRNOG00000068445 | Rplp2 | protein_coding |
| ENSRNOG00000065384 | LOC120096798 | R_norvegicus | Animals | mono_intergenic | H/ACA | | | intergenic |

| | | | | | | | | |
|---|---|---|---|---|---|---|---|---|
| ENSRNOG00000065436 | LOC120094904 | R_norvegicus | Animals | mono_intergenic | H/ACA | | | intergenic |
| ENSRNOG00000065437 | LOC120100247 | R_norvegicus | Animals | mono_intergenic | C/D | | | intergenic |
| ENSRNOG00000065445 | LOC120093442 | R_norvegicus | Animals | mono_intergenic | H/ACA | | | intergenic |
| ENSRNOG00000065466 | LOC120097982 | R_norvegicus | Animals | mono_intergenic | H/ACA | | | intergenic |
| ENSRNOG00000065473 | LOC120100294 | R_norvegicus | Animals | mono_intergenic | C/D | | | intergenic |
| ENSRNOG00000065477 | LOC120100470 | R_norvegicus | Animals | mono_intronic | C/D | ENSRNOG00000019132 | Fv1 | protein_coding |
| ENSRNOG00000065481 | LOC120100272 | R_norvegicus | Animals | mono_intergenic | C/D | | | intergenic |
| ENSRNOG00000065483 | LOC120093383 | R_norvegicus | Animals | mono_intergenic | C/D | | | intergenic |
| ENSRNOG00000065490 | LOC120093436 | R_norvegicus | Animals | mono_intergenic | H/ACA | | | intergenic |
| ENSRNOG00000065498 | LOC120097247 | R_norvegicus | Animals | mono_intergenic | H/ACA | | | intergenic |
| ENSRNOG00000065548 | LOC120102655 | R_norvegicus | Animals | mono_intergenic | H/ACA | | | intergenic |
| ENSRNOG00000065558 | LOC120101265 | R_norvegicus | Animals | mono_intergenic | H/ACA | | | intergenic |
| ENSRNOG00000065570 | LOC120100463 | R_norvegicus | Animals | mono_intronic | H/ACA | ENSRNOG00000015956 | Usp29 | protein_coding |
| ENSRNOG00000065575 | LOC120099474 | R_norvegicus | Animals | mono_intergenic | H/ACA | | | intergenic |
| ENSRNOG00000065576 | Snord1c | R_norvegicus | Animals | mono_intronic | C/D | ENSRNOG00000063207 | | non_coding |
| ENSRNOG00000065598 | LOC120100375 | R_norvegicus | Animals | mono_intergenic | H/ACA | | | intergenic |
| ENSRNOG00000065607 | Snora61 | R_norvegicus | Animals | mono_intronic | H/ACA | ENSRNOG00000071131 | LOC120102992 | non_coding |
| ENSRNOG00000065632 | LOC120100263 | R_norvegicus | Animals | mono_intergenic | C/D | | | intergenic |
| ENSRNOG00000065651 | LOC120099514 | R_norvegicus | Animals | mono_intergenic | H/ACA | | | intergenic |
| ENSRNOG00000065657 | LOC120093360 | R_norvegicus | Animals | mono_intergenic | C/D | | | intergenic |
| ENSRNOG00000065660 | LOC120102722 | R_norvegicus | Animals | mono_intergenic | H/ACA | | | intergenic |
| ENSRNOG00000065702 | LOC120102098 | R_norvegicus | Animals | mono_intergenic | C/D | | | intergenic |
| ENSRNOG00000065708 | LOC120102037 | R_norvegicus | Animals | mono_intergenic | H/ACA | | | intergenic |
| ENSRNOG00000065714 | LOC120094451 | R_norvegicus | Animals | mono_intergenic | C/D | | | intergenic |
| ENSRNOG00000065715 | LOC120097239 | R_norvegicus | Animals | mono_intergenic | H/ACA | | | intergenic |
| ENSRNOG00000065719 | Snord113l5 | R_norvegicus | Animals | mono_intergenic | C/D | | | intergenic |
| ENSRNOG00000065738 | LOC120100315 | R_norvegicus | Animals | mono_intergenic | C/D | | | intergenic |
| ENSRNOG00000065760 | LOC120103274 | R_norvegicus | Animals | mono_intronic | H/ACA | ENSRNOG00000071131 | LOC120102992 | non_coding |
| ENSRNOG00000065764 | LOC120093354 | R_norvegicus | Animals | mono_intergenic | C/D | | | intergenic |
| ENSRNOG00000065785 | LOC120103347 | R_norvegicus | Animals | mono_intergenic | H/ACA | | | intergenic |
| ENSRNOG00000065819 | LOC120100376 | R_norvegicus | Animals | mono_intergenic | H/ACA | | | intergenic |
| ENSRNOG00000065834 | LOC120093330 | R_norvegicus | Animals | mono_intergenic | C/D | | | intergenic |
| ENSRNOG00000065841 | LOC120099499 | R_norvegicus | Animals | mono_intronic | H/ACA | ENSRNOG00000062454 | Tenm1 | protein_coding |
| ENSRNOG00000065849 | LOC120099522 | R_norvegicus | Animals | mono_intergenic | H/ACA | | | intergenic |
| ENSRNOG00000065859 | LOC120098355 | R_norvegicus | Animals | mono_intronic | C/D | ENSRNOG00000019525 | Hspa9 | protein_coding |
| ENSRNOG00000065894 | LOC120097271 | R_norvegicus | Animals | mono_intergenic | H/ACA | | | intergenic |
| ENSRNOG00000065900 | LOC120100306 | R_norvegicus | Animals | mono_intergenic | C/D | | | intergenic |
| ENSRNOG00000065930 | LOC120094038 | R_norvegicus | Animals | mono_intergenic | C/D | | | intergenic |
| ENSRNOG00000065933 | LOC120100493 | R_norvegicus | Animals | mono_intergenic | C/D | | | intergenic |
| ENSRNOG00000065959 | LOC120093396 | R_norvegicus | Animals | mono_intergenic | C/D | | | intergenic |
| ENSRNOG00000065965 | Snord13l1 | R_norvegicus | Animals | mono_intergenic | C/D | | | intergenic |
| ENSRNOG00000065966 | LOC120098370 | R_norvegicus | Animals | mono_intergenic | H/ACA | | | intergenic |
| ENSRNOG00000065973 | LOC120102737 | R_norvegicus | Animals | mono_intergenic | H/ACA | | | intergenic |

| | | | | | | | | |
|---|---|---|---|---|---|---|---|---|
| ENSRNOG00000065976 | LOC120094021 | R_norvegicus | Animals | mono_intronic | C/D | ENSRNOG00000016896 | Rpl3 | protein_coding |
| ENSRNOG00000065994 | LOC120100257 | R_norvegicus | Animals | mono_intergenic | C/D | | | intergenic |
| ENSRNOG00000065996 | LOC120093393 | R_norvegicus | Animals | mono_intergenic | C/D | | | intergenic |
| ENSRNOG00000065999 | LOC120102746 | R_norvegicus | Animals | mono_intergenic | C/D | | | intergenic |
| ENSRNOG00000066014 | LOC120103367 | R_norvegicus | Animals | mono_intergenic | C/D | | | intergenic |
| ENSRNOG00000066017 | LOC120099477 | R_norvegicus | Animals | mono_intergenic | H/ACA | | | intergenic |
| ENSRNOG00000066034 | LOC120099470 | R_norvegicus | Animals | mono_intergenic | H/ACA | | | intergenic |
| ENSRNOG00000066043 | LOC120094039 | R_norvegicus | Animals | mono_intergenic | C/D | | | intergenic |
| ENSRNOG00000066081 | LOC120096803 | R_norvegicus | Animals | mono_intergenic | H/ACA | | | intergenic |
| ENSRNOG00000066105 | LOC120100401 | R_norvegicus | Animals | mono_intergenic | H/ACA | | | intergenic |
| ENSRNOG00000066107 | LOC120093367 | R_norvegicus | Animals | mono_intergenic | C/D | | | intergenic |
| ENSRNOG00000066112 | LOC120103293 | R_norvegicus | Animals | mono_intergenic | H/ACA | | | intergenic |
| ENSRNOG00000066121 | Snord93 | R_norvegicus | Animals | mono_intergenic | C/D | | | intergenic |
| ENSRNOG00000066150 | LOC120093983 | R_norvegicus | Animals | mono_intergenic | H/ACA | | | intergenic |
| ENSRNOG00000066170 | LOC120100402 | R_norvegicus | Animals | mono_intronic | H/ACA | ENSRNOG00000023496 | Entrep2 | protein_coding |
| ENSRNOG00000066206 | LOC120099509 | R_norvegicus | Animals | mono_intergenic | H/ACA | | | intergenic |
| ENSRNOG00000066210 | LOC120094465 | R_norvegicus | Animals | mono_intergenic | H/ACA | | | intergenic |
| ENSRNOG00000066251 | ENSRNOG00000066251 | R_norvegicus | Animals | mono_intronic | C/D | ENSRNOG00000025244 | Trrap | protein_coding |
| ENSRNOG00000066259 | LOC120101340 | R_norvegicus | Animals | mono_intergenic | H/ACA | | | intergenic |
| ENSRNOG00000066273 | LOC120100241 | R_norvegicus | Animals | mono_intergenic | C/D | | | intergenic |
| ENSRNOG00000066337 | LOC120093976 | R_norvegicus | Animals | mono_intronic | H/ACA | ENSRNOG00000005190 | Nipal2 | protein_coding |
| ENSRNOG00000066338 | LOC120099473 | R_norvegicus | Animals | mono_intergenic | H/ACA | | | intergenic |
| ENSRNOG00000066347 | LOC120094906 | R_norvegicus | Animals | mono_intergenic | H/ACA | | | intergenic |
| ENSRNOG00000066350 | LOC120101270 | R_norvegicus | Animals | mono_intergenic | H/ACA | | | intergenic |
| ENSRNOG00000066357 | LOC120093369 | R_norvegicus | Animals | mono_intergenic | C/D | | | intergenic |
| ENSRNOG00000066362 | LOC120103258 | R_norvegicus | Animals | mono_intronic | C/D | ENSRNOG00000011709 | Pum1 | protein_coding |
| ENSRNOG00000066363 | LOC120093975 | R_norvegicus | Animals | mono_intergenic | H/ACA | | | intergenic |
| ENSRNOG00000066382 | LOC120093377 | R_norvegicus | Animals | mono_intergenic | C/D | | | intergenic |
| ENSRNOG00000066388 | LOC120096391 | R_norvegicus | Animals | mono_intergenic | H/ACA | | | intergenic |
| ENSRNOG00000066403 | LOC120101273 | R_norvegicus | Animals | mono_intergenic | H/ACA | | | intergenic |
| ENSRNOG00000066419 | LOC120102031 | R_norvegicus | Animals | mono_intergenic | H/ACA | | | intergenic |
| ENSRNOG00000066430 | LOC120100302 | R_norvegicus | Animals | mono_intergenic | C/D | | | intergenic |
| ENSRNOG00000066457 | LOC120093381 | R_norvegicus | Animals | mono_intergenic | C/D | | | intergenic |
| ENSRNOG00000066459 | LOC120093336 | R_norvegicus | Animals | mono_intergenic | C/D | | | intergenic |
| ENSRNOG00000066468 | LOC120101261 | R_norvegicus | Animals | mono_intergenic | H/ACA | | | intergenic |
| ENSRNOG00000066476 | LOC120093385 | R_norvegicus | Animals | mono_intergenic | C/D | | | intergenic |
| ENSRNOG00000066483 | LOC120100319 | R_norvegicus | Animals | mono_intronic | C/D | ENSRNOG00000065457 | | non_coding |
| ENSRNOG00000066494 | LOC120100331 | R_norvegicus | Animals | mono_intronic | C/D | ENSRNOG00000065457 | | non_coding |
| ENSRNOG00000066510 | LOC120094907 | R_norvegicus | Animals | mono_intronic | H/ACA | ENSRNOG00000011871 | Kat2b | protein_coding |
| ENSRNOG00000066529 | LOC120100485 | R_norvegicus | Animals | mono_intergenic | C/D | | | intergenic |
| ENSRNOG00000066533 | LOC120102717 | R_norvegicus | Animals | mono_intergenic | H/ACA | | | intergenic |
| ENSRNOG00000066535 | LOC120100459 | R_norvegicus | Animals | mono_intergenic | H/ACA | | | intergenic |
| ENSRNOG00000066593 | LOC120094479 | R_norvegicus | Animals | mono_intergenic | H/ACA | | | intergenic |

| | | | | | | | | |
|---|---|---|---|---|---|---|---|---|
| ENSRNOG00000066607 | LOC120093431 | R_norvegicus | Animals | mono_intergenic | H/ACA | | | intergenic |
| ENSRNOG00000066610 | LOC120101302 | R_norvegicus | Animals | mono_intronic | H/ACA | ENSRNOG00000010535 | Cdh18 | protein_coding |
| ENSRNOG00000066631 | LOC120094522 | R_norvegicus | Animals | mono_intergenic | H/ACA | | | intergenic |
| ENSRNOG00000066656 | LOC120097602 | R_norvegicus | Animals | mono_intergenic | H/ACA | | | intergenic |
| ENSRNOG00000066664 | LOC120101274 | R_norvegicus | Animals | mono_intronic | H/ACA | ENSRNOG00000033064 | Edil3 | protein_coding |
| ENSRNOG00000066680 | LOC120094480 | R_norvegicus | Animals | mono_intergenic | H/ACA | | | intergenic |
| ENSRNOG00000066690 | LOC120096802 | R_norvegicus | Animals | mono_intergenic | H/ACA | | | intergenic |
| ENSRNOG00000066699 | LOC120101250 | R_norvegicus | Animals | mono_intergenic | H/ACA | | | intergenic |
| ENSRNOG00000066717 | LOC120099472 | R_norvegicus | Animals | intronic_cluster | H/ACA | ENSRNOG00000003674 | Pir | protein_coding |
| ENSRNOG00000066730 | LOC120094450 | R_norvegicus | Animals | mono_intergenic | C/D | | | intergenic |
| ENSRNOG00000066742 | LOC120093376 | R_norvegicus | Animals | mono_intergenic | C/D | | | intergenic |
| ENSRNOG00000066756 | LOC120098026 | R_norvegicus | Animals | mono_intergenic | C/D | | | intergenic |
| ENSRNOG00000066792 | LOC120102715 | R_norvegicus | Animals | mono_intergenic | H/ACA | | | intergenic |
| ENSRNOG00000066801 | LOC120102658 | R_norvegicus | Animals | mono_intergenic | H/ACA | | | intergenic |
| ENSRNOG00000066821 | LOC120100304 | R_norvegicus | Animals | mono_intergenic | C/D | | | intergenic |
| ENSRNOG00000066825 | LOC120102652 | R_norvegicus | Animals | mono_intronic | H/ACA | ENSRNOG00000049829 | AABR07060872.1 | protein_coding |
| ENSRNOG00000066860 | LOC120100366 | R_norvegicus | Animals | mono_intergenic | H/ACA | | | intergenic |
| ENSRNOG00000066861 | LOC120100329 | R_norvegicus | Animals | mono_intergenic | C/D | | | intergenic |
| ENSRNOG00000066898 | LOC120100471 | R_norvegicus | Animals | mono_intronic | C/D | ENSRNOG00000019132 | Fv1 | protein_coding |
| ENSRNOG00000066907 | LOC120100296 | R_norvegicus | Animals | mono_intergenic | C/D | | | intergenic |
| ENSRNOG00000066944 | LOC120098374 | R_norvegicus | Animals | mono_intergenic | H/ACA | | | intergenic |
| ENSRNOG00000066947 | LOC120097593 | R_norvegicus | Animals | mono_intergenic | H/ACA | | | intergenic |
| ENSRNOG00000066957 | LOC120098007 | R_norvegicus | Animals | mono_intergenic | H/ACA | | | intergenic |
| ENSRNOG00000066984 | LOC120093327 | R_norvegicus | Animals | mono_intergenic | C/D | | | intergenic |
| ENSRNOG00000066990 | LOC120101123 | R_norvegicus | Animals | mono_intergenic | C/D | | | intergenic |
| ENSRNOG00000066997 | LOC120097595 | R_norvegicus | Animals | mono_intergenic | H/ACA | | | intergenic |
| ENSRNOG00000067009 | LOC120098010 | R_norvegicus | Animals | mono_intergenic | C/D | | | intergenic |
| ENSRNOG00000067019 | LOC120101266 | R_norvegicus | Animals | mono_intergenic | H/ACA | | | intergenic |
| ENSRNOG00000067057 | LOC120093997 | R_norvegicus | Animals | mono_intergenic | H/ACA | | | intergenic |
| ENSRNOG00000067116 | LOC120093333 | R_norvegicus | Animals | mono_intergenic | C/D | | | intergenic |
| ENSRNOG00000067133 | LOC120098734 | R_norvegicus | Animals | mono_intergenic | C/D | | | intergenic |
| ENSRNOG00000067136 | LOC120100284 | R_norvegicus | Animals | mono_intergenic | C/D | | | intergenic |
| ENSRNOG00000067158 | LOC120100492 | R_norvegicus | Animals | mono_intergenic | C/D | | | intergenic |
| ENSRNOG00000067166 | LOC120103292 | R_norvegicus | Animals | mono_intergenic | H/ACA | | | intergenic |
| ENSRNOG00000067258 | LOC120093373 | R_norvegicus | Animals | mono_intergenic | C/D | | | intergenic |
| ENSRNOG00000067294 | LOC120093331 | R_norvegicus | Animals | mono_intergenic | C/D | | | intergenic |
| ENSRNOG00000067319 | LOC120099495 | R_norvegicus | Animals | mono_intergenic | H/ACA | | | intergenic |
| ENSRNOG00000067324 | LOC120093977 | R_norvegicus | Animals | mono_intronic | H/ACA | ENSRNOG00000005917 | Pawr | protein_coding |
| ENSRNOG00000067345 | LOC120093979 | R_norvegicus | Animals | mono_intergenic | H/ACA | | | intergenic |
| ENSRNOG00000067351 | LOC120093397 | R_norvegicus | Animals | mono_intergenic | C/D | | | intergenic |
| ENSRNOG00000067353 | LOC120100406 | R_norvegicus | Animals | mono_intergenic | H/ACA | | | intergenic |
| ENSRNOG00000067359 | LOC120100407 | R_norvegicus | Animals | mono_intronic | H/ACA | ENSRNOG00000013057 | Prc1 | protein_coding |
| ENSRNOG00000067371 | LOC120100244 | R_norvegicus | Animals | mono_intergenic | C/D | | | intergenic |

| | | | | | | | | |
|---|---|---|---|---|---|---|---|---|
| ENSRNOG00000067376 | Snord62b | R_norvegicus | Animals | mono_intronic | C/D | ENSRNOG00000010217 | Prrc2b | protein_coding |
| ENSRNOG00000067383 | LOC120100481 | R_norvegicus | Animals | mono_intronic | C/D | ENSRNOG00000014590 | Stk33 | protein_coding |
| ENSRNOG00000067398 | LOC120101262 | R_norvegicus | Animals | mono_intergenic | H/ACA | | | intergenic |
| ENSRNOG00000067405 | LOC120101982 | R_norvegicus | Animals | mono_intronic | C/D | ENSRNOG00000070974 | Rpl7a | protein_coding |
| ENSRNOG00000067453 | LOC120096799 | R_norvegicus | Animals | mono_intergenic | H/ACA | | | intergenic |
| ENSRNOG00000067485 | LOC120098012 | R_norvegicus | Animals | mono_intronic | H/ACA | ENSRNOG00000014616 | Iars1 | protein_coding |
| ENSRNOG00000067492 | LOC120094529 | R_norvegicus | Animals | mono_intergenic | C/D | | | intergenic |
| ENSRNOG00000067497 | LOC120094548 | R_norvegicus | Animals | mono_intronic | C/D | ENSRNOG00000034066 | Hspa8 | protein_coding |
| ENSRNOG00000067498 | LOC120102770 | R_norvegicus | Animals | mono_intergenic | C/D | | | intergenic |
| ENSRNOG00000067508 | ENSRNOG00000067508 | R_norvegicus | Animals | mono_intronic | H/ACA | ENSRNOG00000056128 | AABR07064719.2 | protein_coding |
| ENSRNOG00000067518 | LOC120103294 | R_norvegicus | Animals | mono_intergenic | H/ACA | | | intergenic |
| ENSRNOG00000067533 | LOC120097587 | R_norvegicus | Animals | mono_intergenic | H/ACA | | | intergenic |
| ENSRNOG00000067550 | LOC120095742 | R_norvegicus | Animals | mono_intergenic | H/ACA | | | intergenic |
| ENSRNOG00000067566 | LOC120101341 | R_norvegicus | Animals | mono_intergenic | H/ACA | | | intergenic |
| ENSRNOG00000067616 | LOC120095440 | R_norvegicus | Animals | mono_intronic | H/ACA | ENSRNOG00000005360 | Dhrs7b | protein_coding |
| ENSRNOG00000067621 | LOC120097984 | R_norvegicus | Animals | mono_intergenic | H/ACA | | | intergenic |
| ENSRNOG00000067627 | LOC120102648 | R_norvegicus | Animals | mono_intergenic | H/ACA | | | intergenic |
| ENSRNOG00000067629 | LOC120094521 | R_norvegicus | Animals | mono_intronic | H/ACA | ENSRNOG00000010921 | Taf1d | protein_coding |
| ENSRNOG00000067635 | LOC120095446 | R_norvegicus | Animals | mono_intergenic | H/ACA | | | intergenic |
| ENSRNOG00000067641 | LOC120102727 | R_norvegicus | Animals | mono_intergenic | H/ACA | | | intergenic |
| ENSRNOG00000067649 | LOC120099058 | R_norvegicus | Animals | mono_intergenic | H/ACA | | | intergenic |
| ENSRNOG00000067688 | LOC120093380 | R_norvegicus | Animals | mono_intergenic | C/D | | | intergenic |
| ENSRNOG00000067698 | LOC120097248 | R_norvegicus | Animals | mono_intergenic | H/ACA | | | intergenic |
| ENSRNOG00000067707 | LOC120093400 | R_norvegicus | Animals | mono_intergenic | C/D | | | intergenic |
| ENSRNOG00000067746 | LOC120093339 | R_norvegicus | Animals | mono_intergenic | C/D | | | intergenic |
| ENSRNOG00000067748 | LOC120093343 | R_norvegicus | Animals | mono_intergenic | C/D | | | intergenic |
| ENSRNOG00000067751 | LOC120097585 | R_norvegicus | Animals | mono_intronic | H/ACA | ENSRNOG00000059057 | Shld2 | protein_coding |
| ENSRNOG00000067784 | LOC120093328 | R_norvegicus | Animals | mono_intergenic | C/D | | | intergenic |
| ENSRNOG00000067806 | LOC120100273 | R_norvegicus | Animals | mono_intergenic | C/D | | | intergenic |
| ENSRNOG00000067808 | LOC120098710 | R_norvegicus | Animals | mono_intergenic | C/D | | | intergenic |
| ENSRNOG00000067813 | LOC120095732 | R_norvegicus | Animals | mono_intronic | H/ACA | ENSRNOG00000001789 | Zfp148 | protein_coding |
| ENSRNOG00000067815 | LOC120093325 | R_norvegicus | Animals | mono_intergenic | C/D | | | intergenic |
| ENSRNOG00000067818 | LOC120093352 | R_norvegicus | Animals | mono_intergenic | C/D | | | intergenic |
| ENSRNOG00000067839 | LOC120102027 | R_norvegicus | Animals | mono_intergenic | H/ACA | | | intergenic |
| ENSRNOG00000067864 | LOC120094470 | R_norvegicus | Animals | mono_intronic | H/ACA | ENSRNOG00000010921 | Taf1d | protein_coding |
| ENSRNOG00000067871 | LOC120096854 | R_norvegicus | Animals | mono_intergenic | C/D | | | intergenic |
| ENSRNOG00000067874 | LOC120093392 | R_norvegicus | Animals | mono_intergenic | C/D | | | intergenic |
| ENSRNOG00000067875 | LOC120100274 | R_norvegicus | Animals | mono_intergenic | C/D | | | intergenic |
| ENSRNOG00000067876 | LOC120102649 | R_norvegicus | Animals | mono_intergenic | H/ACA | | | intergenic |
| ENSRNOG00000067888 | LOC120093982 | R_norvegicus | Animals | mono_intergenic | H/ACA | | | intergenic |
| ENSRNOG00000067904 | LOC120100281 | R_norvegicus | Animals | mono_intergenic | C/D | | | intergenic |
| ENSRNOG00000067918 | LOC120102702 | R_norvegicus | Animals | mono_intergenic | H/ACA | | | intergenic |
| ENSRNOG00000067922 | LOC120093950 | R_norvegicus | Animals | mono_intergenic | C/D | | | intergenic |

| | | | | | | | | |
|---|---|---|---|---|---|---|---|---|
| ENSRNOG00000067928 | LOC120098407 | R_norvegicus | Animals | mono_intergenic | C/D | | | intergenic |
| ENSRNOG00000067944 | LOC120100383 | R_norvegicus | Animals | mono_intronic | H/ACA | ENSRNOG00000021052 | Patl1 | protein_coding |
| ENSRNOG00000067981 | LOC120102083 | R_norvegicus | Animals | mono_intergenic | H/ACA | | | intergenic |
| ENSRNOG00000068049 | LOC120099485 | R_norvegicus | Animals | intronic_cluster | H/ACA | ENSRNOG00000003674 | Pir | protein_coding |
| ENSRNOG00000068102 | LOC120100280 | R_norvegicus | Animals | mono_intergenic | C/D | | | intergenic |
| ENSRNOG00000068128 | LOC120102036 | R_norvegicus | Animals | mono_intronic | H/ACA | ENSRNOG00000010659 | Gabpb1 | protein_coding |
| ENSRNOG00000068141 | LOC120093362 | R_norvegicus | Animals | mono_intergenic | C/D | | | intergenic |
| ENSRNOG00000068157 | LOC120100324 | R_norvegicus | Animals | mono_intergenic | C/D | | | intergenic |
| ENSRNOG00000068172 | LOC120093342 | R_norvegicus | Animals | mono_intergenic | C/D | | | intergenic |
| ENSRNOG00000068176 | LOC120102043 | R_norvegicus | Animals | mono_intergenic | H/ACA | | | intergenic |
| ENSRNOG00000068178 | LOC120098373 | R_norvegicus | Animals | mono_intergenic | H/ACA | | | intergenic |
| ENSRNOG00000068196 | LOC120102656 | R_norvegicus | Animals | mono_intronic | H/ACA | ENSRNOG00000049829 | AABR07060872.1 | protein_coding |
| ENSRNOG00000068208 | LOC120102118 | R_norvegicus | Animals | mono_intergenic | C/D | | | intergenic |
| ENSRNOG00000068214 | LOC120096777 | R_norvegicus | Animals | mono_intronic | H/ACA | ENSRNOG00000023529 | Rpl5 | protein_coding |
| ENSRNOG00000068219 | LOC120094537 | R_norvegicus | Animals | mono_intronic | C/D | ENSRNOG00000008356 | Myo5c | protein_coding |
| ENSRNOG00000068223 | LOC120101260 | R_norvegicus | Animals | mono_intronic | H/ACA | ENSRNOG00000018063 | Rad1 | protein_coding |
| ENSRNOG00000068228 | LOC120100268 | R_norvegicus | Animals | mono_intergenic | C/D | | | intergenic |
| ENSRNOG00000068240 | LOC120095491 | R_norvegicus | Animals | intronic_cluster | C/D | ENSRNOG00000023698 | Tex14 | protein_coding |
| ENSRNOG00000068242 | LOC120095770 | R_norvegicus | Animals | mono_intergenic | H/ACA | | | intergenic |
| ENSRNOG00000068244 | LOC120093444 | R_norvegicus | Animals | mono_intergenic | H/ACA | | | intergenic |
| ENSRNOG00000068275 | LOC120100277 | R_norvegicus | Animals | mono_intergenic | C/D | | | intergenic |
| ENSRNOG00000068295 | LOC120102769 | R_norvegicus | Animals | mono_intergenic | C/D | | | intergenic |
| ENSRNOG00000068302 | LOC120099498 | R_norvegicus | Animals | mono_intergenic | H/ACA | | | intergenic |
| ENSRNOG00000068304 | LOC120097272 | R_norvegicus | Animals | mono_intergenic | H/ACA | | | intergenic |
| ENSRNOG00000068321 | LOC120095442 | R_norvegicus | Animals | mono_intergenic | H/ACA | | | intergenic |
| ENSRNOG00000068328 | LOC120101245 | R_norvegicus | Animals | mono_intergenic | H/ACA | | | intergenic |
| ENSRNOG00000068368 | LOC120100464 | R_norvegicus | Animals | mono_intergenic | H/ACA | | | intergenic |
| ENSRNOG00000068372 | LOC120094481 | R_norvegicus | Animals | mono_intergenic | H/ACA | | | intergenic |
| ENSRNOG00000068384 | LOC120101278 | R_norvegicus | Animals | mono_intergenic | H/ACA | | | intergenic |
| ENSRNOG00000068390 | LOC120099476 | R_norvegicus | Animals | mono_intergenic | H/ACA | | | intergenic |
| ENSRNOG00000068398 | LOC120100286 | R_norvegicus | Animals | mono_intergenic | C/D | | | intergenic |
| ENSRNOG00000068409 | LOC120093987 | R_norvegicus | Animals | mono_intergenic | H/ACA | | | intergenic |
| ENSRNOG00000068435 | Snora66 | R_norvegicus | Animals | mono_intronic | H/ACA | ENSRNOG00000023529 | Rpl5 | protein_coding |
| ENSRNOG00000068470 | U3 | R_norvegicus | Animals | mono_intergenic | C/D | | | intergenic |
| ENSRNOG00000068482 | LOC120093971 | R_norvegicus | Animals | mono_intergenic | H/ACA | | | intergenic |
| ENSRNOG00000068531 | LOC120093374 | R_norvegicus | Animals | mono_intergenic | C/D | | | intergenic |
| ENSRNOG00000068546 | LOC120093370 | R_norvegicus | Animals | mono_intergenic | C/D | | | intergenic |
| ENSRNOG00000068553 | LOC120101124 | R_norvegicus | Animals | mono_intergenic | C/D | | | intergenic |
| ENSRNOG00000068562 | LOC120102729 | R_norvegicus | Animals | mono_intergenic | H/ACA | | | intergenic |
| ENSRNOG00000068573 | LOC120097230 | R_norvegicus | Animals | mono_intergenic | H/ACA | | | intergenic |
| ENSRNOG00000068590 | LOC120096788 | R_norvegicus | Animals | mono_intronic | H/ACA | ENSRNOG00000027801 | Zpbp | protein_coding |
| ENSRNOG00000068593 | LOC120102119 | R_norvegicus | Animals | mono_intergenic | C/D | | | intergenic |
| ENSRNOG00000068604 | LOC120093372 | R_norvegicus | Animals | mono_intergenic | C/D | | | intergenic |

| | | | | | | | | |
|---|---|---|---|---|---|---|---|---|
| ENSRNOG00000068627 | LOC120100312 | R_norvegicus | Animals | mono_intergenic | C/D | | | intergenic |
| ENSRNOG00000068629 | LOC120094908 | R_norvegicus | Animals | mono_intergenic | H/ACA | | | intergenic |
| ENSRNOG00000068630 | LOC120093349 | R_norvegicus | Animals | mono_intergenic | C/D | | | intergenic |
| ENSRNOG00000068641 | LOC120101348 | R_norvegicus | Animals | mono_intergenic | H/ACA | | | intergenic |
| ENSRNOG00000068664 | LOC120100410 | R_norvegicus | Animals | mono_intergenic | H/ACA | | | intergenic |
| ENSRNOG00000068668 | LOC120100405 | R_norvegicus | Animals | mono_intergenic | H/ACA | | | intergenic |
| ENSRNOG00000068671 | LOC120099077 | R_norvegicus | Animals | mono_intergenic | H/ACA | | | intergenic |
| ENSRNOG00000068674 | LOC120095439 | R_norvegicus | Animals | mono_intergenic | H/ACA | | | intergenic |
| ENSRNOG00000068693 | ENSRNOG00000068693 | R_norvegicus | Animals | mono_intronic | C/D | ENSRNOG00000003970 | Tnpo2 | protein_coding |
| ENSRNOG00000068737 | LOC120100421 | R_norvegicus | Animals | mono_intergenic | H/ACA | | | intergenic |
| ENSRNOG00000068742 | LOC120098371 | R_norvegicus | Animals | mono_intergenic | H/ACA | | | intergenic |
| ENSRNOG00000068746 | LOC120096830 | R_norvegicus | Animals | mono_intergenic | H/ACA | | | intergenic |
| ENSRNOG00000068769 | LOC120100275 | R_norvegicus | Animals | mono_intergenic | C/D | | | intergenic |
| ENSRNOG00000068791 | LOC120101275 | R_norvegicus | Animals | mono_intergenic | H/ACA | | | intergenic |
| ENSRNOG00000068798 | LOC120099038 | R_norvegicus | Animals | mono_intergenic | C/D | | | intergenic |
| ENSRNOG00000068799 | LOC120093364 | R_norvegicus | Animals | mono_intergenic | C/D | | | intergenic |
| ENSRNOG00000068804 | LOC120093403 | R_norvegicus | Animals | mono_intergenic | C/D | | | intergenic |
| ENSRNOG00000068808 | LOC120100428 | R_norvegicus | Animals | mono_intergenic | H/ACA | | | intergenic |
| ENSRNOG00000068812 | LOC120093334 | R_norvegicus | Animals | mono_intergenic | C/D | | | intergenic |
| ENSRNOG00000068817 | LOC120093359 | R_norvegicus | Animals | mono_intergenic | C/D | | | intergenic |
| ENSRNOG00000068818 | LOC120093345 | R_norvegicus | Animals | mono_intergenic | C/D | | | intergenic |
| ENSRNOG00000068877 | LOC120093433 | R_norvegicus | Animals | mono_intergenic | H/ACA | | | intergenic |
| ENSRNOG00000068891 | LOC120095730 | R_norvegicus | Animals | mono_intergenic | H/ACA | | | intergenic |
| ENSRNOG00000068901 | LOC120100279 | R_norvegicus | Animals | mono_intergenic | C/D | | | intergenic |
| ENSRNOG00000068927 | LOC120099492 | R_norvegicus | Animals | mono_intronic | H/ACA | ENSRNOG00000023440 | Phex | protein_coding |
| ENSRNOG00000068948 | LOC120100282 | R_norvegicus | Animals | mono_intergenic | C/D | | | intergenic |
| ENSRNOG00000068952 | LOC120102033 | R_norvegicus | Animals | mono_intergenic | H/ACA | | | intergenic |
| ENSRNOG00000068979 | LOC120093337 | R_norvegicus | Animals | mono_intergenic | C/D | | | intergenic |
| ENSRNOG00000068998 | LOC120100321 | R_norvegicus | Animals | mono_intergenic | C/D | | | intergenic |
| ENSRNOG00000069002 | LOC120093394 | R_norvegicus | Animals | mono_intergenic | C/D | | | intergenic |
| ENSRNOG00000069018 | LOC120093435 | R_norvegicus | Animals | mono_intergenic | H/ACA | | | intergenic |
| ENSRNOG00000069028 | LOC120102654 | R_norvegicus | Animals | mono_intergenic | H/ACA | | | intergenic |
| ENSRNOG00000069038 | LOC120100240 | R_norvegicus | Animals | mono_intergenic | C/D | | | intergenic |
| ENSRNOG00000069040 | LOC120100403 | R_norvegicus | Animals | mono_intergenic | H/ACA | | | intergenic |
| ENSRNOG00000069042 | LOC120101264 | R_norvegicus | Animals | mono_intergenic | H/ACA | | | intergenic |
| ENSRNOG00000069135 | Snord97 | R_norvegicus | Animals | mono_intronic | C/D | ENSRNOG00000017158 | Eif4g2 | protein_coding |
| ENSRNOG00000069139 | LOC120100250 | R_norvegicus | Animals | mono_intergenic | C/D | | | intergenic |
| ENSRNOG00000069141 | LOC120101269 | R_norvegicus | Animals | mono_intronic | H/ACA | ENSRNOG00000063312 | LOC120100783 | non_coding |
| ENSRNOG00000069147 | LOC120100255 | R_norvegicus | Animals | mono_intergenic | C/D | | | intergenic |
| ENSRNOG00000069158 | LOC120093399 | R_norvegicus | Animals | mono_intergenic | C/D | | | intergenic |
| ENSRNOG00000069190 | LOC120100239 | R_norvegicus | Animals | mono_intergenic | C/D | | | intergenic |
| ENSRNOG00000069192 | LOC120100248 | R_norvegicus | Animals | mono_intergenic | C/D | | | intergenic |
| ENSRNOG00000069199 | LOC120096425 | R_norvegicus | Animals | mono_intronic | C/D | ENSRNOG00000028627 | Hmcn1 | protein_coding |

| | | | | | | | | |
|---|---|---|---|---|---|---|---|---|
| ENSRNOG00000069223 | LOC120094442 | R_norvegicus | Animals | mono_intronic | C/D | ENSRNOG00000009378 | Rpl4 | protein_coding |
| ENSRNOG00000069273 | LOC120093341 | R_norvegicus | Animals | mono_intergenic | C/D | | | intergenic |
| ENSRNOG00000069284 | LOC120100318 | R_norvegicus | Animals | mono_intergenic | C/D | | | intergenic |
| ENSRNOG00000069316 | LOC120102030 | R_norvegicus | Animals | mono_intergenic | H/ACA | | | intergenic |
| ENSRNOG00000069327 | LOC120098354 | R_norvegicus | Animals | mono_intronic | C/D | ENSRNOG00000019525 | Hspa9 | protein_coding |
| ENSRNOG00000069343 | LOC120100316 | R_norvegicus | Animals | mono_intergenic | C/D | | | intergenic |
| ENSRNOG00000069354 | LOC120099478 | R_norvegicus | Animals | mono_intergenic | H/ACA | | | intergenic |
| ENSRNOG00000069360 | LOC120097983 | R_norvegicus | Animals | mono_intronic | H/ACA | ENSRNOG00000063546 | | non_coding |
| ENSRNOG00000069371 | LOC120095436 | R_norvegicus | Animals | mono_intergenic | H/ACA | | | intergenic |
| ENSRNOG00000069404 | LOC120095397 | R_norvegicus | Animals | mono_intronic | C/D | ENSRNOG00000063207 | | non_coding |
| ENSRNOG00000069415 | LOC120100307 | R_norvegicus | Animals | mono_intergenic | C/D | | | intergenic |
| ENSRNOG00000069465 | LOC120099076 | R_norvegicus | Animals | mono_intergenic | H/ACA | | | intergenic |
| ENSRNOG00000069470 | LOC120093966 | R_norvegicus | Animals | mono_intergenic | H/ACA | | | intergenic |
| ENSRNOG00000069474 | LOC120102705 | R_norvegicus | Animals | mono_intergenic | H/ACA | | | intergenic |
| ENSRNOG00000069513 | LOC120102651 | R_norvegicus | Animals | mono_intergenic | H/ACA | | | intergenic |
| ENSRNOG00000069514 | LOC120100270 | R_norvegicus | Animals | mono_intergenic | C/D | | | intergenic |
| ENSRNOG00000069597 | LOC120102728 | R_norvegicus | Animals | mono_intergenic | H/ACA | | | intergenic |
| ENSRNOG00000069602 | LOC120098403 | R_norvegicus | Animals | mono_intronic | H/ACA | ENSRNOG00000063070 | Pcdhga5 | protein_coding |
| ENSRNOG00000069614 | LOC120100327 | R_norvegicus | Animals | mono_intergenic | C/D | | | intergenic |
| ENSRNOG00000069626 | LOC120100308 | R_norvegicus | Animals | mono_intergenic | C/D | | | intergenic |
| ENSRNOG00000069639 | LOC120095441 | R_norvegicus | Animals | mono_intergenic | H/ACA | | | intergenic |
| ENSRNOG00000069641 | LOC120097232 | R_norvegicus | Animals | mono_intergenic | H/ACA | | | intergenic |
| ENSRNOG00000069668 | LOC120100429 | R_norvegicus | Animals | mono_intergenic | H/ACA | | | intergenic |
| ENSRNOG00000069675 | LOC120101289 | R_norvegicus | Animals | mono_intergenic | H/ACA | | | intergenic |
| ENSRNOG00000069691 | LOC120101279 | R_norvegicus | Animals | mono_intergenic | H/ACA | | | intergenic |
| ENSRNOG00000069717 | Snora71e | R_norvegicus | Animals | intronic_cluster | H/ACA | ENSRNOG00000069588 | | non_coding |
| ENSRNOG00000069742 | LOC120101267 | R_norvegicus | Animals | mono_intergenic | H/ACA | | | intergenic |
| ENSRNOG00000069747 | LOC120093973 | R_norvegicus | Animals | mono_intergenic | H/ACA | | | intergenic |
| ENSRNOG00000069748 | LOC120094447 | R_norvegicus | Animals | mono_intergenic | C/D | | | intergenic |
| ENSRNOG00000069753 | LOC120099488 | R_norvegicus | Animals | mono_intergenic | H/ACA | | | intergenic |
| ENSRNOG00000069804 | LOC120100325 | R_norvegicus | Animals | mono_intergenic | C/D | | | intergenic |
| ENSRNOG00000069810 | LOC120103251 | R_norvegicus | Animals | mono_intergenic | C/D | | | intergenic |
| ENSRNOG00000069842 | LOC120102666 | R_norvegicus | Animals | mono_intergenic | H/ACA | | | intergenic |
| ENSRNOG00000069846 | LOC120099489 | R_norvegicus | Animals | mono_intergenic | H/ACA | | | intergenic |
| ENSRNOG00000069866 | LOC120100411 | R_norvegicus | Animals | mono_intergenic | H/ACA | | | intergenic |
| ENSRNOG00000069889 | LOC120093434 | R_norvegicus | Animals | mono_intergenic | H/ACA | | | intergenic |
| ENSRNOG00000069892 | LOC120094469 | R_norvegicus | Animals | mono_intergenic | H/ACA | | | intergenic |
| ENSRNOG00000069917 | LOC120101984 | R_norvegicus | Animals | mono_intronic | C/D | ENSRNOG00000070974 | Rpl7a | protein_coding |
| ENSRNOG00000069943 | LOC120102026 | R_norvegicus | Animals | mono_intergenic | H/ACA | | | intergenic |
| ENSRNOG00000069951 | LOC120101268 | R_norvegicus | Animals | mono_intergenic | H/ACA | | | intergenic |
| ENSRNOG00000069997 | LOC120101276 | R_norvegicus | Animals | mono_intergenic | H/ACA | | | intergenic |
| ENSRNOG00000070000 | LOC120093483 | R_norvegicus | Animals | mono_intronic | C/D | ENSRNOG00000027317 | Ylpm1 | protein_coding |
| ENSRNOG00000070006 | LOC120093984 | R_norvegicus | Animals | mono_intergenic | H/ACA | | | intergenic |

| | | | | | | | | |
|---|---|---|---|---|---|---|---|---|
| ENSRNOG00000070016 | LOC120101271 | R_norvegicus | Animals | mono_intergenic | H/ACA | | | intergenic |
| ENSRNOG00000070019 | LOC120095437 | R_norvegicus | Animals | mono_intronic | H/ACA | ENSRNOG00000024535 | Zzef1 | protein_coding |
| ENSRNOG00000070138 | LOC120093358 | R_norvegicus | Animals | mono_intergenic | C/D | | | intergenic |
| ENSRNOG00000070142 | LOC120093999 | R_norvegicus | Animals | mono_intergenic | H/ACA | | | intergenic |
| ENSRNOG00000070143 | LOC120098682 | R_norvegicus | Animals | mono_intronic | H/ACA | ENSRNOG00000012271 | Cnot1 | protein_coding |
| ENSRNOG00000070147 | LOC120094887 | R_norvegicus | Animals | mono_intergenic | H/ACA | | | intergenic |
| ENSRNOG00000070175 | LOC120093353 | R_norvegicus | Animals | mono_intergenic | C/D | | | intergenic |
| ENSRNOG00000070179 | LOC120093493 | R_norvegicus | Animals | mono_intergenic | C/D | | | intergenic |
| ENSRNOG00000070200 | LOC120100384 | R_norvegicus | Animals | mono_intergenic | H/ACA | | | intergenic |
| ENSRNOG00000070203 | LOC120100293 | R_norvegicus | Animals | mono_intergenic | C/D | | | intergenic |
| ENSRNOG00000070213 | LOC120101293 | R_norvegicus | Animals | mono_intergenic | H/ACA | | | intergenic |
| ENSRNOG00000070214 | LOC120097258 | R_norvegicus | Animals | mono_intergenic | H/ACA | | | intergenic |
| ENSRNOG00000070216 | LOC120100252 | R_norvegicus | Animals | mono_intergenic | C/D | | | intergenic |
| ENSRNOG00000070224 | LOC120101246 | R_norvegicus | Animals | mono_intronic | H/ACA | ENSRNOG00000062777 | Rsrc1 | protein_coding |
| ENSRNOG00000070240 | LOC120096808 | R_norvegicus | Animals | mono_intergenic | H/ACA | | | intergenic |
| ENSRNOG00000070253 | LOC120097979 | R_norvegicus | Animals | mono_intergenic | H/ACA | | | intergenic |
| ENSRNOG00000070258 | LOC120101227 | R_norvegicus | Animals | mono_intergenic | C/D | | | intergenic |
| ENSRNOG00000070272 | LOC120093361 | R_norvegicus | Animals | mono_intergenic | C/D | | | intergenic |
| ENSRNOG00000070286 | Snord99 | R_norvegicus | Animals | mono_exonic | C/D | ENSRNOG00000071131 | LOC120102992 | non_coding |
| ENSRNOG00000070315 | LOC120100291 | R_norvegicus | Animals | mono_intergenic | C/D | | | intergenic |
| ENSRNOG00000070327 | LOC120103366 | R_norvegicus | Animals | mono_intergenic | C/D | | | intergenic |
| ENSRNOG00000070339 | LOC120093323 | R_norvegicus | Animals | mono_intergenic | C/D | | | intergenic |
| ENSRNOG00000070344 | LOC120097589 | R_norvegicus | Animals | mono_intergenic | H/ACA | | | intergenic |
| ENSRNOG00000070348 | LOC120099444 | R_norvegicus | Animals | mono_intronic | H/ACA | ENSRNOG00000004160 | Prps2 | protein_coding |
| ENSRNOG00000070350 | ENSRNOG00000070350 | R_norvegicus | Animals | intronic_cluster | C/D | ENSRNOG00000037418 | Inca1 | protein_coding |
| ENSRNOG00000070358 | LOC120101280 | R_norvegicus | Animals | mono_intergenic | H/ACA | | | intergenic |
| ENSRNOG00000070373 | LOC120100333 | R_norvegicus | Animals | mono_intergenic | C/D | | | intergenic |
| ENSRNOG00000070403 | LOC120098372 | R_norvegicus | Animals | mono_intergenic | H/ACA | | | intergenic |
| ENSRNOG00000070439 | LOC120102647 | R_norvegicus | Animals | mono_intergenic | H/ACA | | | intergenic |
| ENSRNOG00000070464 | LOC120097249 | R_norvegicus | Animals | mono_intergenic | H/ACA | | | intergenic |
| ENSRNOG00000070473 | LOC120093387 | R_norvegicus | Animals | mono_intergenic | C/D | | | intergenic |
| ENSRNOG00000070487 | LOC120100408 | R_norvegicus | Animals | mono_intergenic | H/ACA | | | intergenic |
| ENSRNOG00000070492 | LOC120100484 | R_norvegicus | Animals | mono_intergenic | C/D | | | intergenic |
| ENSRNOG00000070515 | LOC120094455 | R_norvegicus | Animals | mono_intronic | H/ACA | ENSRNOG00000018009 | Rab8b | protein_coding |
| ENSRNOG00000070525 | ENSRNOG00000070525 | R_norvegicus | Animals | intronic_cluster | C/D | ENSRNOG00000028461 | Gnl3 | protein_coding |
| ENSRNOG00000070528 | LOC120103269 | R_norvegicus | Animals | mono_intergenic | H/ACA | | | intergenic |
| ENSRNOG00000070538 | LOC120093371 | R_norvegicus | Animals | mono_intergenic | C/D | | | intergenic |
| ENSRNOG00000070557 | LOC120095468 | R_norvegicus | Animals | mono_intergenic | C/D | | | intergenic |
| ENSRNOG00000070599 | LOC120100400 | R_norvegicus | Animals | mono_intergenic | H/ACA | | | intergenic |
| ENSRNOG00000070610 | LOC120098418 | R_norvegicus | Animals | mono_intergenic | C/D | | | intergenic |
| ENSRNOG00000070636 | Snora2l2 | R_norvegicus | Animals | mono_intronic | H/ACA | ENSRNOG00000060185 | Kansl2 | protein_coding |
| ENSRNOG00000070663 | LOC120102035 | R_norvegicus | Animals | mono_intergenic | H/ACA | | | intergenic |
| ENSRNOG00000070680 | LOC120103304 | R_norvegicus | Animals | mono_intergenic | H/ACA | | | intergenic |

| | | | | | | | | |
|---|---|---|---|---|---|---|---|---|
| ENSRNOG00000070708 | LOC120103273 | R_norvegicus | Animals | mono_intergenic | H/ACA | | | intergenic |
| ENSRNOG00000070713 | LOC120093338 | R_norvegicus | Animals | mono_intergenic | C/D | | | intergenic |
| ENSRNOG00000070743 | LOC120095469 | R_norvegicus | Animals | mono_intergenic | C/D | | | intergenic |
| ENSRNOG00000070756 | LOC120101330 | R_norvegicus | Animals | mono_intergenic | H/ACA | | | intergenic |
| ENSRNOG00000070798 | LOC120102696 | R_norvegicus | Animals | mono_intergenic | H/ACA | | | intergenic |
| ENSRNOG00000070806 | LOC120093347 | R_norvegicus | Animals | mono_intergenic | C/D | | | intergenic |
| ENSRNOG00000070820 | LOC120100404 | R_norvegicus | Animals | mono_intergenic | H/ACA | | | intergenic |
| ENSRNOG00000070821 | LOC120094004 | R_norvegicus | Animals | mono_intergenic | H/ACA | | | intergenic |
| ENSRNOG00000070841 | Snord24 | R_norvegicus | Animals | mono_intronic | C/D | ENSRNOG00000070974 | Rpl7a | protein_coding |
| ENSRNOG00000070873 | LOC120101285 | R_norvegicus | Animals | mono_intergenic | H/ACA | | | intergenic |
| ENSRNOG00000070879 | LOC120102743 | R_norvegicus | Animals | mono_intronic | C/D | ENSRNOG00000069504 | | protein_coding |
| ENSRNOG00000070884 | LOC120100358 | R_norvegicus | Animals | mono_intronic | H/ACA | ENSRNOG00000048138 | Zfp566 | protein_coding |
| ENSRNOG00000070908 | LOC120093356 | R_norvegicus | Animals | mono_intergenic | C/D | | | intergenic |
| ENSRNOG00000070942 | LOC120094949 | R_norvegicus | Animals | mono_intergenic | C/D | | | intergenic |
| ENSRNOG00000070969 | LOC120101317 | R_norvegicus | Animals | mono_intergenic | H/ACA | | | intergenic |
| ENSRNOG00000070975 | LOC120100298 | R_norvegicus | Animals | mono_intergenic | C/D | | | intergenic |
| ENSRNOG00000070981 | Snord13 | R_norvegicus | Animals | mono_intergenic | C/D | | | intergenic |
| ENSRNOG00000070984 | LOC120100297 | R_norvegicus | Animals | mono_intergenic | C/D | | | intergenic |
| ENSRNOG00000071006 | LOC120100235 | R_norvegicus | Animals | mono_intergenic | C/D | | | intergenic |
| ENSRNOG00000071043 | LOC120101283 | R_norvegicus | Animals | mono_intergenic | H/ACA | | | intergenic |
| ENSRNOG00000071055 | LOC120099481 | R_norvegicus | Animals | mono_intergenic | H/ACA | | | intergenic |
| ENSRNOG00000071058 | LOC120095443 | R_norvegicus | Animals | mono_intergenic | H/ACA | | | intergenic |
| ENSRNOG00000071061 | LOC120097981 | R_norvegicus | Animals | mono_intergenic | H/ACA | | | intergenic |
| ENSRNOG00000071063 | LOC120098687 | R_norvegicus | Animals | mono_intergenic | H/ACA | | | intergenic |
| ENSRNOG00000071126 | LOC120097241 | R_norvegicus | Animals | mono_intergenic | H/ACA | | | intergenic |
| ENSRNOG00000071149 | LOC120096840 | R_norvegicus | Animals | mono_intergenic | H/ACA | | | intergenic |
| ENSRNOG00000071174 | LOC120100258 | R_norvegicus | Animals | mono_intergenic | C/D | | | intergenic |
| ENSRNOG00000071199 | LOC120094441 | R_norvegicus | Animals | mono_intronic | C/D | ENSRNOG00000009378 | Rpl4 | protein_coding |
| ENSRNOG00000071211 | LOC120102623 | R_norvegicus | Animals | mono_intergenic | H/ACA | | | intergenic |
| ENSRNOG00000071213 | LOC120093350 | R_norvegicus | Animals | mono_intergenic | C/D | | | intergenic |
| ENSXETG00000028111 | SNORD36 | X_tropicalis | Animals | mono_intronic | C/D | ENSXETG00000042208 | rpl7a | protein_coding |
| ENSXETG00000028167 | SNORD18 | X_tropicalis | Animals | mono_intronic | C/D | ENSXETG00000015898 | rpl4 | protein_coding |
| ENSXETG00000028168 | SNORA71 | X_tropicalis | Animals | mono_intronic | H/ACA | ENSXETG00000010656 | rps17 | protein_coding |
| ENSXETG00000028188 | SNORD15 | X_tropicalis | Animals | mono_intronic | C/D | ENSXETG00000010729 | rps3 | protein_coding |
| ENSXETG00000028210 | SNORA62 | X_tropicalis | Animals | mono_intronic | H/ACA | ENSXETG00000027344 | rpsa | protein_coding |
| ENSXETG00000028216 | SNORD18 | X_tropicalis | Animals | mono_intronic | C/D | ENSXETG00000015898 | rpl4 | protein_coding |
| ENSXETG00000028217 | ENSXETG00000028217 | X_tropicalis | Animals | intergenic_cluster | C/D | | | intergenic |
| ENSXETG00000028251 | SNORA63 | X_tropicalis | Animals | mono_intronic | H/ACA | ENSXETG00000019390 | eif4a2 | protein_coding |
| ENSXETG00000028266 | SNORD27 | X_tropicalis | Animals | intergenic_cluster | C/D | | | intergenic |
| ENSXETG00000028273 | SNORD15 | X_tropicalis | Animals | mono_intronic | C/D | ENSXETG00000010729 | rps3 | protein_coding |
| ENSXETG00000028289 | SNORD31 | X_tropicalis | Animals | intergenic_cluster | C/D | | | intergenic |
| ENSXETG00000028563 | SNORD18 | X_tropicalis | Animals | mono_intronic | C/D | ENSXETG00000015898 | rpl4 | protein_coding |
| ENSXETG00000028593 | SNORA63 | X_tropicalis | Animals | mono_intronic | H/ACA | ENSXETG00000019390 | eif4a2 | protein_coding |

| | | | | | | | | |
|---|---|---|---|---|---|---|---|---|
| ENSXETG00000028667 | SNORD29 | X_tropicalis | Animals | intergenic_cluster | C/D | | | intergenic |
| ENSXETG00000028853 | SNORD36 | X_tropicalis | Animals | mono_intronic | C/D | ENSXETG00000042208 | rpl7a | protein_coding |
| ENSXETG00000028882 | ENSXETG00000028882 | X_tropicalis | Animals | mono_intronic | H/ACA | ENSXETG00000009782 | kansl2 | protein_coding |
| ENSXETG00000028886 | SNORD18 | X_tropicalis | Animals | mono_intronic | C/D | ENSXETG00000015898 | rpl4 | protein_coding |
| ENSXETG00000029030 | SNORA62 | X_tropicalis | Animals | mono_intronic | H/ACA | ENSXETG00000027344 | rpsa | protein_coding |
| ENSXETG00000029042 | SNORA71 | X_tropicalis | Animals | mono_intronic | H/ACA | ENSXETG00000010656 | rps17 | protein_coding |
| ENSXETG00000029233 | ENSXETG00000029233 | X_tropicalis | Animals | mono_intronic | C/D | ENSXETG00000022073 | nop58 | protein_coding |
| ENSXETG00000029242 | ENSXETG00000029242 | X_tropicalis | Animals | mono_intronic | H/ACA | ENSXETG00000010077 | cct6a | protein_coding |
| ENSXETG00000029243 | ENSXETG00000029243 | X_tropicalis | Animals | mono_intergenic | C/D | | | intergenic |
| ENSXETG00000029246 | ENSXETG00000029246 | X_tropicalis | Animals | intergenic_cluster | C/D | | | intergenic |
| ENSXETG00000029250 | ENSXETG00000029250 | X_tropicalis | Animals | mono_intergenic | C/D | | | intergenic |
| ENSXETG00000029259 | ENSXETG00000029259 | X_tropicalis | Animals | mono_intronic | H/ACA | ENSXETG00000010077 | cct6a | protein_coding |
| ENSXETG00000029268 | ENSXETG00000029268 | X_tropicalis | Animals | mono_intronic | H/ACA | ENSXETG00000007440 | upf3b | protein_coding |
| ENSXETG00000029269 | SNORD26 | X_tropicalis | Animals | intergenic_cluster | C/D | | | intergenic |
| ENSXETG00000029270 | ENSXETG00000029270 | X_tropicalis | Animals | intergenic_cluster | C/D | | | intergenic |
| ENSXETG00000029272 | SNORD22 | X_tropicalis | Animals | mono_intergenic | C/D | | | intergenic |
| ENSXETG00000029279 | ENSXETG00000029279 | X_tropicalis | Animals | intergenic_cluster | C/D | | | intergenic |
| ENSXETG00000029283 | SNORD30 | X_tropicalis | Animals | intergenic_cluster | C/D | | | intergenic |
| ENSXETG00000029286 | ENSXETG00000029286 | X_tropicalis | Animals | mono_intronic | H/ACA | ENSXETG00000024201 | ipo7 | protein_coding |
| ENSXETG00000029287 | ENSXETG00000029287 | X_tropicalis | Animals | mono_intronic | C/D | ENSXETG00000003427 | rhbdd2 | protein_coding |
| ENSXETG00000029289 | ENSXETG00000029289 | X_tropicalis | Animals | mono_intronic | H/ACA | ENSXETG00000024201 | ipo7 | protein_coding |
| ENSXETG00000029293 | ENSXETG00000029293 | X_tropicalis | Animals | mono_intronic | H/ACA | ENSXETG00000049411 | rpl23 | protein_coding |
| ENSXETG00000029309 | ENSXETG00000029309 | X_tropicalis | Animals | intergenic_cluster | C/D | | | intergenic |
| ENSXETG00000029311 | ENSXETG00000029311 | X_tropicalis | Animals | intergenic_cluster | C/D | | | intergenic |
| ENSXETG00000029314 | ENSXETG00000029314 | X_tropicalis | Animals | mono_intronic | H/ACA | ENSXETG00000018767 | slc25a3 | protein_coding |
| ENSXETG00000029364 | ENSXETG00000029364 | X_tropicalis | Animals | mono_intergenic | C/D | | | intergenic |
| ENSXETG00000029382 | ENSXETG00000029382 | X_tropicalis | Animals | mono_intronic | C/D | ENSXETG00000017970 | ptcd3 | protein_coding |
| ENSXETG00000029389 | ENSXETG00000029389 | X_tropicalis | Animals | mono_intronic | H/ACA | ENSXETG00000045758 | | protein_coding |
| ENSXETG00000029399 | ENSXETG00000029399 | X_tropicalis | Animals | mono_intronic | C/D | ENSXETG00000017970 | ptcd3 | protein_coding |
| ENSXETG00000029400 | SNORD100 | X_tropicalis | Animals | mono_intronic | C/D | ENSXETG00000000109 | rps12 | protein_coding |
| ENSXETG00000029415 | ENSXETG00000029415 | X_tropicalis | Animals | mono_intronic | H/ACA | ENSXETG00000021667 | rpl18a | protein_coding |
| ENSXETG00000029417 | ENSXETG00000029417 | X_tropicalis | Animals | mono_intronic | C/D | ENSXETG00000000109 | rps12 | protein_coding |
| ENSXETG00000029433 | ENSXETG00000029433 | X_tropicalis | Animals | mono_intronic | H/ACA | ENSXETG00000045758 | | protein_coding |
| ENSXETG00000029465 | ENSXETG00000029465 | X_tropicalis | Animals | mono_intronic | H/ACA | ENSXETG00000021667 | rpl18a | protein_coding |
| ENSXETG00000029520 | ENSXETG00000029520 | X_tropicalis | Animals | mono_intronic | C/D | ENSXETG00000027024 | rpl37 | protein_coding |
| ENSXETG00000029527 | SNORA66 | X_tropicalis | Animals | mono_intronic | H/ACA | ENSXETG00000015598 | zfr | protein_coding |
| ENSXETG00000029539 | SNORA74 | X_tropicalis | Animals | mono_intronic | H/ACA | ENSXETG00000026175 | timm23 | protein_coding |
| ENSXETG00000029541 | SNORD72 | X_tropicalis | Animals | mono_intronic | C/D | ENSXETG00000027024 | rpl37 | protein_coding |
| ENSXETG00000029544 | SNORD83B | X_tropicalis | Animals | mono_intronic | C/D | ENSXETG00000006914 | rpl3 | protein_coding |
| ENSXETG00000029554 | ENSXETG00000029554 | X_tropicalis | Animals | mono_intronic | H/ACA | ENSXETG00000009782 | kansl2 | protein_coding |
| ENSXETG00000029555 | ENSXETG00000029555 | X_tropicalis | Animals | mono_intronic | H/ACA | ENSXETG00000049197 | rpl12 | protein_coding |
| ENSXETG00000029570 | ENSXETG00000029570 | X_tropicalis | Animals | mono_intronic | H/ACA | ENSXETG00000010241 | eif3a | protein_coding |
| ENSXETG00000029578 | SNORD10 | X_tropicalis | Animals | mono_intronic | C/D | ENSXETG00000020123 | eif4a1 | protein_coding |

| | | | | | | | | |
|---|---|---|---|---|---|---|---|---|
| ENSXETG00000029588 | ENSXETG00000029588 | X_tropicalis | Animals | mono_intronic | H/ACA | ENSXETG00000010241 | eif3a | protein_coding |
| ENSXETG00000029596 | ENSXETG00000029596 | X_tropicalis | Animals | intergenic_cluster | H/ACA | | | intergenic |
| ENSXETG00000029599 | SNORA74 | X_tropicalis | Animals | mono_intronic | H/ACA | ENSXETG00000026175 | timm23 | protein_coding |
| ENSXETG00000029605 | ENSXETG00000029605 | X_tropicalis | Animals | intergenic_cluster | H/ACA | | | intergenic |
| ENSXETG00000029617 | ENSXETG00000029617 | X_tropicalis | Animals | mono_intronic | C/D | ENSXETG00000019390 | eif4a2 | protein_coding |
| ENSXETG00000029625 | ENSXETG00000029625 | X_tropicalis | Animals | mono_intergenic | H/ACA | | | intergenic |
| ENSXETG00000029651 | ENSXETG00000029651 | X_tropicalis | Animals | mono_exonic | H/ACA | ENSXETG00000038604 | tcp1 | protein_coding |
| ENSXETG00000029653 | ENSXETG00000029653 | X_tropicalis | Animals | mono_intronic | H/ACA | ENSXETG00000023866 | tbrg4 | protein_coding |
| ENSXETG00000029655 | ENSXETG00000029655 | X_tropicalis | Animals | intergenic_cluster | H/ACA | | | intergenic |
| ENSXETG00000029658 | ENSXETG00000029658 | X_tropicalis | Animals | mono_intergenic | C/D | | | intergenic |
| ENSXETG00000029667 | ENSXETG00000029667 | X_tropicalis | Animals | mono_intronic | C/D | ENSXETG00000002157 | ubap2 | protein_coding |
| ENSXETG00000029669 | ENSXETG00000029669 | X_tropicalis | Animals | mono_intronic | C/D | ENSXETG00000001986 | ckap5 | protein_coding |
| ENSXETG00000029670 | ENSXETG00000029670 | X_tropicalis | Animals | mono_intronic | C/D | ENSXETG00000013674 | eif4g2 | protein_coding |
| ENSXETG00000029677 | SNORD127 | X_tropicalis | Animals | mono_intronic | C/D | ENSXETG00000003066 | prpf39.1 | protein_coding |
| ENSXETG00000029680 | ENSXETG00000029680 | X_tropicalis | Animals | mono_intronic | H/ACA | ENSXETG00000019390 | eif4a2 | protein_coding |
| ENSXETG00000029684 | U3 | X_tropicalis | Animals | mono_intergenic | C/D | | | intergenic |
| ENSXETG00000029700 | ENSXETG00000029700 | X_tropicalis | Animals | mono_intronic | C/D | ENSXETG00000011528 | ncapd2 | protein_coding |
| ENSXETG00000029705 | ENSXETG00000029705 | X_tropicalis | Animals | mono_intronic | H/ACA | ENSXETG00000020903 | iars1 | protein_coding |
| ENSXETG00000029719 | SNORA74 | X_tropicalis | Animals | mono_intronic | H/ACA | ENSXETG00000026175 | timm23 | protein_coding |
| ENSXETG00000029721 | ENSXETG00000029721 | X_tropicalis | Animals | mono_intronic | H/ACA | ENSXETG00000047318 | | protein_coding |
| ENSXETG00000029725 | ENSXETG00000029725 | X_tropicalis | Animals | mono_intronic | H/ACA | ENSXETG00000014962 | nop56 | protein_coding |
| ENSXETG00000029727 | ENSXETG00000029727 | X_tropicalis | Animals | mono_intronic | H/ACA | ENSXETG00000007221 | rpl3l | protein_coding |
| ENSXETG00000029728 | ENSXETG00000029728 | X_tropicalis | Animals | intergenic_cluster | C/D | | | intergenic |
| ENSXETG00000029738 | ENSXETG00000029738 | X_tropicalis | Animals | mono_intronic | H/ACA | ENSXETG00000049197 | rpl12 | protein_coding |
| ENSXETG00000029745 | ENSXETG00000029745 | X_tropicalis | Animals | mono_intergenic | C/D | | | intergenic |
| ENSXETG00000029750 | SNORA28 | X_tropicalis | Animals | mono_intronic | H/ACA | ENSXETG00000012766 | eif5 | protein_coding |
| ENSXETG00000029757 | ENSXETG00000029757 | X_tropicalis | Animals | mono_intronic | H/ACA | ENSXETG00000023866 | tbrg4 | protein_coding |
| ENSXETG00000029766 | SNORD79 | X_tropicalis | Animals | intergenic_cluster | C/D | | | intergenic |
| ENSXETG00000029775 | SNORA70 | X_tropicalis | Animals | mono_intronic | H/ACA | ENSXETG00000010878 | rpl10 | protein_coding |
| ENSXETG00000029776 | SNORA71 | X_tropicalis | Animals | mono_intronic | H/ACA | ENSXETG00000010656 | rps17 | protein_coding |
| ENSXETG00000029793 | ENSXETG00000029793 | X_tropicalis | Animals | mono_intronic | C/D | ENSXETG00000019390 | eif4a2 | protein_coding |
| ENSXETG00000029800 | ENSXETG00000029800 | X_tropicalis | Animals | mono_intronic | H/ACA | ENSXETG00000010946 | pabpc4 | protein_coding |
| ENSXETG00000029805 | ENSXETG00000029805 | X_tropicalis | Animals | mono_intronic | H/ACA | ENSXETG00000009802 | rpl21 | protein_coding |
| ENSXETG00000029813 | SNORA79B | X_tropicalis | Animals | mono_intergenic | H/ACA | | | intergenic |
| ENSXETG00000029841 | ENSXETG00000029841 | X_tropicalis | Animals | mono_intronic | H/ACA | ENSXETG00000023866 | tbrg4 | protein_coding |
| ENSXETG00000029849 | ENSXETG00000029849 | X_tropicalis | Animals | mono_intergenic | C/D | | | intergenic |
| ENSXETG00000029854 | ENSXETG00000029854 | X_tropicalis | Animals | mono_intronic | C/D | ENSXETG00000011528 | ncapd2 | protein_coding |
| ENSXETG00000029860 | SNORA29 | X_tropicalis | Animals | mono_intronic | H/ACA | ENSXETG00000038604 | tcp1 | protein_coding |
| ENSXETG00000029866 | ENSXETG00000029866 | X_tropicalis | Animals | mono_intergenic | H/ACA | | | intergenic |
| ENSXETG00000029880 | ENSXETG00000029880 | X_tropicalis | Animals | mono_intronic | H/ACA | ENSXETG00000039364 | | protein_coding |
| ENSXETG00000029891 | SNORD28 | X_tropicalis | Animals | intergenic_cluster | C/D | | | intergenic |
| ENSXETG00000029906 | ENSXETG00000029906 | X_tropicalis | Animals | mono_intronic | C/D | ENSXETG00000013674 | eif4g2 | protein_coding |
| ENSXETG00000029908 | ENSXETG00000029908 | X_tropicalis | Animals | mono_intronic | H/ACA | ENSXETG00000014661 | tpt1 | protein_coding |

| | | | | | | | | |
|---|---|---|---|---|---|---|---|---|
| ENSXETG00000030914 | ENSXETG00000030914 | X_tropicalis | Animals | mono_intronic | H/ACA | ENSXETG00000018767 | slc25a3 | protein_coding |
| ENSXETG00000034432 | ENSXETG00000034432 | X_tropicalis | Animals | mono_intronic | C/D | ENSXETG00000022073 | nop58 | protein_coding |
| ENSXETG00000034569 | ENSXETG00000034569 | X_tropicalis | Animals | mono_intergenic | C/D | | | intergenic |
| ENSXETG00000034644 | SNORD58 | X_tropicalis | Animals | mono_intronic | C/D | ENSXETG00000020308 | rpl17 | protein_coding |
| ENSXETG00000034829 | ENSXETG00000034829 | X_tropicalis | Animals | mono_intronic | C/D | ENSXETG00000004771 | tnpo2 | protein_coding |
| ENSXETG00000034834 | ENSXETG00000034834 | X_tropicalis | Animals | mono_intronic | C/D | ENSXETG00000002157 | ubap2 | protein_coding |
| ENSXETG00000034853 | SNORA73 | X_tropicalis | Animals | mono_intronic | H/ACA | ENSXETG00000001209 | rps7 | protein_coding |
| ENSXETG00000034959 | SNORD31 | X_tropicalis | Animals | mono_intronic | C/D | ENSXETG00000005580 | ints5 | protein_coding |
| ENSXETG00000035085 | ENSXETG00000035085 | X_tropicalis | Animals | mono_intronic | C/D | ENSXETG00000004771 | tnpo2 | protein_coding |
| ENSXETG00000035238 | SNORD42 | X_tropicalis | Animals | mono_intronic | C/D | ENSXETG00000026189 | rpl23a | protein_coding |
| ENSXETG00000035415 | ENSXETG00000035415 | X_tropicalis | Animals | mono_intergenic | C/D | | | intergenic |
| ENSXETG00000035484 | ENSXETG00000035484 | X_tropicalis | Animals | mono_intronic | C/D | ENSXETG00000025081 | cox7c | protein_coding |
| ENSXETG00000035539 | ENSXETG00000035539 | X_tropicalis | Animals | mono_intronic | H/ACA | ENSXETG00000034836 | ranbp1 | protein_coding |
| ENSXETG00000035554 | SNORD42 | X_tropicalis | Animals | mono_intronic | C/D | ENSXETG00000026189 | rpl23a | protein_coding |
| ENSXETG00000035667 | SNORD61 | X_tropicalis | Animals | mono_intronic | C/D | ENSXETG00000003481 | rbmx | protein_coding |
| ENSXETG00000035715 | ENSXETG00000035715 | X_tropicalis | Animals | mono_intronic | C/D | ENSXETG00000022073 | nop58 | protein_coding |
| ENSXETG00000035752 | ENSXETG00000035752 | X_tropicalis | Animals | mono_intronic | H/ACA | ENSXETG00000037966 | pabir2 | protein_coding |
| ENSXETG00000035777 | ENSXETG00000035777 | X_tropicalis | Animals | mono_intronic | C/D | ENSXETG00000019835 | rpl13 | protein_coding |
| ENSXETG00000035812 | SNORA73 | X_tropicalis | Animals | intronic_cluster | H/ACA | ENSXETG00000001209 | rps7 | protein_coding |
| ENSXETG00000035814 | ENSXETG00000035814 | X_tropicalis | Animals | mono_intronic | C/D | ENSXETG00000024665 | atp5f1b | protein_coding |
| ENSXETG00000035891 | ENSXETG00000035891 | X_tropicalis | Animals | mono_intergenic | C/D | | | intergenic |
| ENSXETG00000035900 | ENSXETG00000035900 | X_tropicalis | Animals | mono_intronic | C/D | ENSXETG00000005652 | rbm6 | protein_coding |
| ENSXETG00000035967 | SNORD36 | X_tropicalis | Animals | mono_intronic | C/D | ENSXETG00000042208 | rpl7a | protein_coding |
| ENSXETG00000035982 | SNORD62 | X_tropicalis | Animals | mono_intronic | C/D | ENSXETG00000001480 | prrc2b | protein_coding |
| ENSXETG00000036212 | ENSXETG00000036212 | X_tropicalis | Animals | mono_intronic | C/D | ENSXETG00000002157 | ubap2 | protein_coding |
| ENSXETG00000036225 | ENSXETG00000036225 | X_tropicalis | Animals | intergenic_cluster | C/D | | | intergenic |
| ENSXETG00000036325 | ENSXETG00000036325 | X_tropicalis | Animals | intergenic_cluster | C/D | | | intergenic |
| ENSXETG00000036326 | ENSXETG00000036326 | X_tropicalis | Animals | mono_intronic | C/D | ENSXETG00000000109 | rps12 | protein_coding |
| ENSXETG00000036360 | SNORD34 | X_tropicalis | Animals | mono_intergenic | C/D | | | intergenic |
| ENSXETG00000036476 | ENSXETG00000036476 | X_tropicalis | Animals | mono_intergenic | H/ACA | | | intergenic |
| ENSXETG00000036592 | ENSXETG00000036592 | X_tropicalis | Animals | mono_intronic | C/D | ENSXETG00000035654 | wdr43 | protein_coding |
| ENSXETG00000036632 | ENSXETG00000036632 | X_tropicalis | Animals | mono_intronic | H/ACA | ENSXETG00000035639 | | protein_coding |
| ENSXETG00000036744 | ENSXETG00000036744 | X_tropicalis | Animals | mono_intronic | H/ACA | ENSXETG00000010077 | cct6a | protein_coding |
| ENSXETG00000036971 | U3 | X_tropicalis | Animals | mono_intergenic | C/D | | | intergenic |
| ENSXETG00000037005 | ENSXETG00000037005 | X_tropicalis | Animals | mono_intronic | C/D | ENSXETG00000035654 | wdr43 | protein_coding |
| ENSXETG00000037133 | SNORD11B | X_tropicalis | Animals | mono_intronic | C/D | ENSXETG00000022073 | nop58 | protein_coding |
| ENSXETG00000037139 | SNORD73 | X_tropicalis | Animals | mono_intronic | C/D | ENSXETG00000009396 | rps3a | protein_coding |
| ENSXETG00000037259 | SNORA74 | X_tropicalis | Animals | mono_intronic | H/ACA | ENSXETG00000026175 | timm23 | protein_coding |
| ENSXETG00000037284 | ENSXETG00000037284 | X_tropicalis | Animals | mono_intergenic | C/D | | | intergenic |
| ENSXETG00000037315 | SNORD39 | X_tropicalis | Animals | intergenic_cluster | C/D | | | intergenic |
| ENSXETG00000037376 | SNORD34 | X_tropicalis | Animals | mono_intergenic | C/D | | | intergenic |
| ENSXETG00000037405 | ENSXETG00000037405 | X_tropicalis | Animals | intronic_cluster | C/D | ENSXETG00000031841 | MGC145244 | protein_coding |
| ENSXETG00000037423 | ENSXETG00000037423 | X_tropicalis | Animals | intronic_cluster | C/D | ENSXETG00000031841 | MGC145244 | protein_coding |

| | | | | | | | | |
|---|---|---|---|---|---|---|---|---|
| ENSXETG00000037449 | ENSXETG00000037449 | X_tropicalis | Animals | mono_intronic | C/D | ENSXETG00000025081 | cox7c | protein_coding |
| ENSXETG00000038020 | ENSXETG00000038020 | X_tropicalis | Animals | mono_intergenic | H/ACA | | | intergenic |
| ENSXETG00000038023 | ENSXETG00000038023 | X_tropicalis | Animals | mono_intronic | C/D | ENSXETG00000035654 | wdr43 | protein_coding |
| ENSXETG00000038033 | SNORD33 | X_tropicalis | Animals | mono_intronic | C/D | ENSXETG00000014144 | rpl13a | protein_coding |
| ENSXETG00000038037 | ENSXETG00000038037 | X_tropicalis | Animals | mono_intronic | C/D | ENSXETG00000036581 | cep295 | protein_coding |
| ENSXETG00000038078 | ENSXETG00000038078 | X_tropicalis | Animals | mono_intronic | C/D | ENSXETG00000026189 | rpl23a | protein_coding |
| ENSXETG00000038289 | ENSXETG00000038289 | X_tropicalis | Animals | mono_intergenic | C/D | | | intergenic |
| ENSXETG00000038349 | ENSXETG00000038349 | X_tropicalis | Animals | mono_intronic | C/D | ENSXETG00000014962 | nop56 | protein_coding |
| ENSXETG00000038394 | ENSXETG00000038394 | X_tropicalis | Animals | intergenic_cluster | C/D | | | intergenic |
| ENSXETG00000038606 | ENSXETG00000038606 | X_tropicalis | Animals | mono_intergenic | H/ACA | | | intergenic |
| ENSXETG00000038718 | ENSXETG00000038718 | X_tropicalis | Animals | mono_intronic | C/D | ENSXETG00000009517 | ncl | protein_coding |
| ENSXETG00000038780 | SNORA72 | X_tropicalis | Animals | mono_intronic | H/ACA | ENSXETG00000036866 | rpl30 | protein_coding |
| ENSXETG00000038784 | ENSXETG00000038784 | X_tropicalis | Animals | mono_intronic | C/D | ENSXETG00000013669 | gnl3 | protein_coding |
| ENSXETG00000038902 | ENSXETG00000038902 | X_tropicalis | Animals | mono_intergenic | H/ACA | | | intergenic |
| ENSXETG00000039013 | ENSXETG00000039013 | X_tropicalis | Animals | mono_intronic | C/D | ENSXETG00000031841 | MGC145244 | protein_coding |
| ENSXETG00000039056 | ENSXETG00000039056 | X_tropicalis | Animals | mono_intronic | H/ACA | ENSXETG00000010946 | pabpc4 | protein_coding |
| ENSXETG00000039143 | ENSXETG00000039143 | X_tropicalis | Animals | mono_intronic | C/D | ENSXETG00000001986 | ckap5 | protein_coding |
| ENSXETG00000039197 | SNORD83 | X_tropicalis | Animals | mono_intronic | C/D | ENSXETG00000032517 | dkc1 | protein_coding |
| ENSXETG00000039219 | ENSXETG00000039219 | X_tropicalis | Animals | mono_intronic | C/D | ENSXETG00000024665 | atp5f1b | protein_coding |
| ENSXETG00000039385 | ENSXETG00000039385 | X_tropicalis | Animals | mono_intergenic | C/D | | | intergenic |
| ENSXETG00000039395 | ENSXETG00000039395 | X_tropicalis | Animals | mono_intronic | C/D | ENSXETG00000024665 | atp5f1b | protein_coding |
| ENSXETG00000039432 | SNORD58 | X_tropicalis | Animals | mono_intronic | C/D | ENSXETG00000020308 | rpl17 | protein_coding |
| ENSXETG00000039462 | ENSXETG00000039462 | X_tropicalis | Animals | mono_intronic | C/D | ENSXETG00000019835 | rpl13 | protein_coding |
| ENSXETG00000039531 | U3 | X_tropicalis | Animals | mono_intergenic | C/D | | | intergenic |
| ENSXETG00000039588 | SNORD39 | X_tropicalis | Animals | intergenic_cluster | C/D | | | intergenic |
| ENSXETG00000039675 | SNORA62 | X_tropicalis | Animals | mono_intronic | H/ACA | ENSXETG00000027344 | rpsa | protein_coding |
| ENSXETG00000039777 | SNORA73 | X_tropicalis | Animals | mono_intronic | H/ACA | ENSXETG00000001209 | rps7 | protein_coding |
| ENSXETG00000039921 | ENSXETG00000039921 | X_tropicalis | Animals | mono_intronic | H/ACA | ENSXETG00000037966 | pabir2 | protein_coding |
| ENSXETG00000040016 | ENSXETG00000040016 | X_tropicalis | Animals | mono_intronic | C/D | ENSXETG00000026189 | rpl23a | protein_coding |
| ENSXETG00000040098 | ENSXETG00000040098 | X_tropicalis | Animals | mono_intergenic | C/D | | | intergenic |
| ENSXETG00000040175 | ENSXETG00000040175 | X_tropicalis | Animals | mono_intronic | C/D | ENSXETG00000035654 | wdr43 | protein_coding |
| ENSXETG00000040217 | ENSXETG00000040217 | X_tropicalis | Animals | mono_intronic | C/D | ENSXETG00000013596 | rps8 | protein_coding |
| ENSXETG00000040378 | ENSXETG00000040378 | X_tropicalis | Animals | intergenic_cluster | C/D | | | intergenic |
| ENSXETG00000040423 | SNORD33 | X_tropicalis | Animals | mono_intronic | C/D | ENSXETG00000014144 | rpl13a | protein_coding |
| ENSXETG00000040439 | ENSXETG00000040439 | X_tropicalis | Animals | mono_intronic | H/ACA | ENSXETG00000010077 | cct6a | protein_coding |
| ENSXETG00000040446 | SNORD58 | X_tropicalis | Animals | mono_intronic | C/D | ENSXETG00000020308 | rpl17 | protein_coding |
| ENSXETG00000040545 | ENSXETG00000040545 | X_tropicalis | Animals | intergenic_cluster | C/D | | | intergenic |
| ENSXETG00000040549 | ENSXETG00000040549 | X_tropicalis | Animals | intergenic_cluster | C/D | | | intergenic |
| ENSXETG00000040573 | ENSXETG00000040573 | X_tropicalis | Animals | intergenic_cluster | C/D | | | intergenic |
| ENSXETG00000040669 | ENSXETG00000040669 | X_tropicalis | Animals | intergenic_cluster | C/D | | | intergenic |
| ENSXETG00000040859 | SNORD31 | X_tropicalis | Animals | mono_intergenic | C/D | | | intergenic |
| ENSXETG00000040909 | SNORD73 | X_tropicalis | Animals | mono_intronic | C/D | ENSXETG00000009396 | rps3a | protein_coding |
| ENSXETG00000040939 | ENSXETG00000040939 | X_tropicalis | Animals | mono_intronic | C/D | ENSXETG00000019835 | rpl13 | protein_coding |

| | | | | | | | | |
|---|---|---|---|---|---|---|---|---|
| ENSXETG00000041014 | ENSXETG00000041014 | X_tropicalis | Animals | mono_intergenic | C/D | | | intergenic |
| ENSXETG00000041276 | ENSXETG00000041276 | X_tropicalis | Animals | mono_intronic | C/D | ENSXETG00000026004 | chd7 | protein_coding |
| ENSXETG00000041282 | SNORD73 | X_tropicalis | Animals | mono_intronic | C/D | ENSXETG00000009396 | rps3a | protein_coding |
| ENSXETG00000041371 | SNORD82 | X_tropicalis | Animals | mono_intronic | C/D | ENSXETG00000009517 | ncl | protein_coding |
| ENSXETG00000041459 | ENSXETG00000041459 | X_tropicalis | Animals | mono_intergenic | H/ACA | | | intergenic |
| ENSXETG00000041518 | U3 | X_tropicalis | Animals | mono_intergenic | C/D | | | intergenic |
| ENSXETG00000041641 | SNORD16 | X_tropicalis | Animals | mono_intronic | C/D | ENSXETG00000015898 | rpl4 | protein_coding |
| ENSXETG00000041685 | ENSXETG00000041685 | X_tropicalis | Animals | intergenic_cluster | C/D | | | intergenic |
| ENSXETG00000041707 | ENSXETG00000041707 | X_tropicalis | Animals | mono_intronic | H/ACA | ENSXETG00000003912 | rpl27a | protein_coding |
| ENSXETG00000041734 | SNORA73 | X_tropicalis | Animals | mono_intronic | H/ACA | ENSXETG00000001209 | rps7 | protein_coding |
| ENSXETG00000041801 | ENSXETG00000041801 | X_tropicalis | Animals | mono_intergenic | H/ACA | | | intergenic |
| ENSXETG00000041811 | ENSXETG00000041811 | X_tropicalis | Animals | mono_intergenic | H/ACA | | | intergenic |
| ENSXETG00000041817 | U3 | X_tropicalis | Animals | mono_intergenic | C/D | | | intergenic |
| ENSXETG00000042012 | ENSXETG00000042012 | X_tropicalis | Animals | mono_intronic | H/ACA | ENSXETG00000010241 | eif3a | protein_coding |
| ENSXETG00000042248 | ENSXETG00000042248 | X_tropicalis | Animals | intergenic_cluster | C/D | | | intergenic |
| ENSXETG00000042304 | U3 | X_tropicalis | Animals | mono_intergenic | C/D | | | intergenic |
| ENSXETG00000042311 | ENSXETG00000042311 | X_tropicalis | Animals | mono_intronic | H/ACA | ENSXETG00000023866 | tbrg4 | protein_coding |
| ENSXETG00000042443 | ENSXETG00000042443 | X_tropicalis | Animals | mono_intronic | H/ACA | ENSXETG00000031917 | | protein_coding |
| ENSXETG00000042463 | ENSXETG00000042463 | X_tropicalis | Animals | intergenic_cluster | H/ACA | | | intergenic |
| ENSXETG00000042553 | SNORD24 | X_tropicalis | Animals | mono_intronic | C/D | ENSXETG00000042208 | rpl7a | protein_coding |
| ENSXETG00000042610 | U3 | X_tropicalis | Animals | mono_intergenic | C/D | | | intergenic |
| ENSXETG00000042630 | ENSXETG00000042630 | X_tropicalis | Animals | mono_intronic | H/ACA | ENSXETG00000010241 | eif3a | protein_coding |
| ENSXETG00000042754 | U3 | X_tropicalis | Animals | intronic_cluster | C/D | ENSXETG00000047181 | large1 | protein_coding |
| ENSXETG00000042842 | ENSXETG00000042842 | X_tropicalis | Animals | intergenic_cluster | H/ACA | | | intergenic |
| ENSXETG00000042872 | ENSXETG00000042872 | X_tropicalis | Animals | intergenic_cluster | H/ACA | | | intergenic |
| ENSXETG00000042888 | ENSXETG00000042888 | X_tropicalis | Animals | mono_intronic | H/ACA | ENSXETG00000023866 | tbrg4 | protein_coding |
| ENSXETG00000042918 | SNORA73 | X_tropicalis | Animals | mono_intronic | H/ACA | ENSXETG00000001209 | rps7 | protein_coding |
| ENSXETG00000042945 | SNORD15 | X_tropicalis | Animals | mono_intronic | C/D | ENSXETG00000010729 | rps3 | protein_coding |
| ENSXETG00000042964 | ENSXETG00000042964 | X_tropicalis | Animals | mono_intergenic | H/ACA | | | intergenic |
| ENSXETG00000042999 | ENSXETG00000042999 | X_tropicalis | Animals | intergenic_cluster | C/D | | | intergenic |
| ENSXETG00000043110 | U3 | X_tropicalis | Animals | intronic_cluster | C/D | ENSXETG00000042282 | | protein_coding |
| ENSXETG00000043189 | U3 | X_tropicalis | Animals | mono_intergenic | C/D | | | intergenic |
| ENSXETG00000043195 | ENSXETG00000043195 | X_tropicalis | Animals | mono_intergenic | C/D | | | intergenic |
| ENSXETG00000043287 | U3 | X_tropicalis | Animals | mono_intergenic | C/D | | | intergenic |
| ENSXETG00000043348 | U3 | X_tropicalis | Animals | mono_intergenic | C/D | | | intergenic |
| ENSXETG00000043377 | U3 | X_tropicalis | Animals | mono_intergenic | C/D | | | intergenic |
| ENSXETG00000043388 | U8 | X_tropicalis | Animals | mono_intergenic | C/D | | | intergenic |
| ENSXETG00000043476 | U3 | X_tropicalis | Animals | mono_intergenic | C/D | | | intergenic |
| ENSXETG00000043565 | ENSXETG00000043565 | X_tropicalis | Animals | mono_intronic | H/ACA | ENSXETG00000010241 | eif3a | protein_coding |
| ENSXETG00000043587 | U3 | X_tropicalis | Animals | intergenic_cluster | C/D | | | intergenic |
| ENSXETG00000043733 | U3 | X_tropicalis | Animals | mono_intergenic | C/D | | | intergenic |
| ENSXETG00000043831 | U3 | X_tropicalis | Animals | mono_intergenic | C/D | | | intergenic |
| ENSXETG00000043846 | U3 | X_tropicalis | Animals | intronic_cluster | C/D | ENSXETG00000047181 | large1 | protein_coding |

| | | | | | | | | |
|---|---|---|---|---|---|---|---|---|
| ENSXETG00000043905 | U3 | X_tropicalis | Animals | intronic_cluster | C/D | ENSXETG00000008923 | | protein_coding |
| ENSXETG00000043910 | U3 | X_tropicalis | Animals | mono_intergenic | C/D | | | intergenic |
| ENSXETG00000043994 | SNORA73 | X_tropicalis | Animals | intronic_cluster | H/ACA | ENSXETG00000001209 | rps7 | protein_coding |
| ENSXETG00000044009 | SNORD14 | X_tropicalis | Animals | mono_intronic | C/D | ENSXETG00000013038 | hspa8 | protein_coding |
| ENSXETG00000044059 | SNORA66 | X_tropicalis | Animals | mono_intronic | H/ACA | ENSXETG00000015598 | zfr | protein_coding |
| ENSXETG00000044091 | U3 | X_tropicalis | Animals | mono_intergenic | C/D | | | intergenic |
| ENSXETG00000044122 | ENSXETG00000044122 | X_tropicalis | Animals | intergenic_cluster | C/D | | | intergenic |
| ENSXETG00000044264 | ENSXETG00000044264 | X_tropicalis | Animals | mono_intergenic | C/D | | | intergenic |
| ENSXETG00000044281 | ENSXETG00000044281 | X_tropicalis | Animals | mono_intergenic | C/D | | | intergenic |
| ENSXETG00000044312 | ENSXETG00000044312 | X_tropicalis | Animals | intergenic_cluster | C/D | | | intergenic |
| ENSXETG00000044416 | ENSXETG00000044416 | X_tropicalis | Animals | mono_intronic | C/D | ENSXETG00000031841 | MGC145244 | protein_coding |
| ENSXETG00000044433 | U3 | X_tropicalis | Animals | mono_intergenic | C/D | | | intergenic |
| ENSXETG00000044458 | ENSXETG00000044458 | X_tropicalis | Animals | intergenic_cluster | H/ACA | | | intergenic |
| ENSXETG00000044569 | ENSXETG00000044569 | X_tropicalis | Animals | intergenic_cluster | C/D | | | intergenic |
| ENSXETG00000044621 | ENSXETG00000044621 | X_tropicalis | Animals | mono_intergenic | H/ACA | | | intergenic |
| ENSXETG00000044655 | ENSXETG00000044655 | X_tropicalis | Animals | intergenic_cluster | C/D | | | intergenic |
| ENSXETG00000044656 | U3 | X_tropicalis | Animals | mono_intergenic | C/D | | | intergenic |
| ENSXETG00000044672 | SNORA73 | X_tropicalis | Animals | mono_intronic | H/ACA | ENSXETG00000001209 | rps7 | protein_coding |
| ENSXETG00000044767 | ENSXETG00000044767 | X_tropicalis | Animals | intergenic_cluster | H/ACA | | | intergenic |
| ENSXETG00000044785 | ENSXETG00000044785 | X_tropicalis | Animals | intergenic_cluster | C/D | | | intergenic |
| ENSXETG00000044797 | U3 | X_tropicalis | Animals | mono_intergenic | C/D | | | intergenic |
| ENSXETG00000044827 | U8 | X_tropicalis | Animals | mono_intergenic | C/D | | | intergenic |
| ENSXETG00000044949 | U3 | X_tropicalis | Animals | intergenic_cluster | C/D | | | intergenic |
| ENSXETG00000044976 | U3 | X_tropicalis | Animals | mono_intergenic | C/D | | | intergenic |
| ENSXETG00000045144 | U3 | X_tropicalis | Animals | intergenic_cluster | C/D | | | intergenic |
| ENSXETG00000045154 | U3 | X_tropicalis | Animals | mono_intergenic | C/D | | | intergenic |
| ENSXETG00000045158 | ENSXETG00000045158 | X_tropicalis | Animals | intergenic_cluster | H/ACA | | | intergenic |
| ENSXETG00000045208 | ENSXETG00000045208 | X_tropicalis | Animals | intronic_cluster | C/D | ENSXETG00000042282 | | protein_coding |
| ENSXETG00000045216 | ENSXETG00000045216 | X_tropicalis | Animals | mono_intergenic | H/ACA | | | intergenic |
| ENSXETG00000045240 | ENSXETG00000045240 | X_tropicalis | Animals | mono_intergenic | H/ACA | | | intergenic |
| ENSXETG00000045287 | U3 | X_tropicalis | Animals | intronic_cluster | C/D | ENSXETG00000047181 | large1 | protein_coding |
| ENSXETG00000045368 | U3 | X_tropicalis | Animals | mono_intergenic | C/D | | | intergenic |
| ENSXETG00000045395 | U3 | X_tropicalis | Animals | mono_intergenic | C/D | | | intergenic |
| ENSXETG00000045428 | U3 | X_tropicalis | Animals | mono_intergenic | C/D | | | intergenic |
| ENSXETG00000045497 | U3 | X_tropicalis | Animals | mono_intergenic | C/D | | | intergenic |
| ENSXETG00000045517 | SNORD58 | X_tropicalis | Animals | mono_intronic | C/D | ENSXETG00000020308 | rpl17 | protein_coding |
| ENSXETG00000045563 | U3 | X_tropicalis | Animals | mono_intergenic | C/D | | | intergenic |
| ENSXETG00000045565 | U3 | X_tropicalis | Animals | mono_intergenic | C/D | | | intergenic |
| ENSXETG00000045597 | U3 | X_tropicalis | Animals | mono_intergenic | C/D | | | intergenic |
| ENSXETG00000045642 | U3 | X_tropicalis | Animals | mono_intergenic | C/D | | | intergenic |
| ENSXETG00000045886 | ENSXETG00000045886 | X_tropicalis | Animals | mono_intronic | H/ACA | ENSXETG00000003912 | rpl27a | protein_coding |
| ENSXETG00000045893 | ENSXETG00000045893 | X_tropicalis | Animals | intergenic_cluster | C/D | | | intergenic |
| ENSXETG00000045987 | U3 | X_tropicalis | Animals | mono_intergenic | C/D | | | intergenic |

| | | | | | | | | |
|---|---|---|---|---|---|---|---|---|
| ENSXETG00000045989 | U3 | X_tropicalis | Animals | mono_intergenic | C/D | | | intergenic |
| ENSXETG00000046025 | ENSXETG00000046025 | X_tropicalis | Animals | intergenic_cluster | H/ACA | | | intergenic |
| ENSXETG00000046066 | U3 | X_tropicalis | Animals | mono_intergenic | C/D | | | intergenic |
| ENSXETG00000046094 | U8 | X_tropicalis | Animals | mono_intergenic | C/D | | | intergenic |
| ENSXETG00000046109 | U3 | X_tropicalis | Animals | mono_intergenic | C/D | | | intergenic |
| ENSXETG00000046258 | ENSXETG00000046258 | X_tropicalis | Animals | mono_intronic | C/D | ENSXETG00000014962 | nop56 | protein_coding |
| ENSXETG00000046358 | SNORA66 | X_tropicalis | Animals | mono_intronic | H/ACA | ENSXETG00000015598 | zfr | protein_coding |
| ENSXETG00000046387 | SNORD15 | X_tropicalis | Animals | mono_intronic | C/D | ENSXETG00000010729 | rps3 | protein_coding |
| ENSXETG00000046424 | ENSXETG00000046424 | X_tropicalis | Animals | intergenic_cluster | H/ACA | | | intergenic |
| ENSXETG00000046472 | SNORD14 | X_tropicalis | Animals | mono_intronic | C/D | ENSXETG00000013038 | hspa8 | protein_coding |
| ENSXETG00000046524 | U3 | X_tropicalis | Animals | intergenic_cluster | C/D | | | intergenic |
| ENSXETG00000046563 | U3 | X_tropicalis | Animals | mono_intergenic | C/D | | | intergenic |
| ENSXETG00000046609 | U3 | X_tropicalis | Animals | mono_intergenic | C/D | | | intergenic |
| ENSXETG00000046649 | ENSXETG00000046649 | X_tropicalis | Animals | mono_intronic | C/D | ENSXETG00000031841 | MGC145244 | protein_coding |
| ENSXETG00000046717 | ENSXETG00000046717 | X_tropicalis | Animals | intergenic_cluster | C/D | | | intergenic |
| ENSXETG00000046803 | ENSXETG00000046803 | X_tropicalis | Animals | intergenic_cluster | H/ACA | | | intergenic |
| ENSXETG00000046805 | ENSXETG00000046805 | X_tropicalis | Animals | mono_intronic | H/ACA | ENSXETG00000049411 | rpl23 | protein_coding |
| ENSXETG00000046816 | U3 | X_tropicalis | Animals | mono_intergenic | C/D | | | intergenic |
| ENSXETG00000046832 | ENSXETG00000046832 | X_tropicalis | Animals | mono_intergenic | C/D | | | intergenic |
| ENSXETG00000046856 | ENSXETG00000046856 | X_tropicalis | Animals | mono_intergenic | H/ACA | | | intergenic |
| ENSXETG00000046861 | ENSXETG00000046861 | X_tropicalis | Animals | intergenic_cluster | H/ACA | | | intergenic |
| ENSXETG00000046898 | ENSXETG00000046898 | X_tropicalis | Animals | mono_intergenic | H/ACA | | | intergenic |
| ENSXETG00000046907 | U3 | X_tropicalis | Animals | mono_intergenic | C/D | | | intergenic |
| ENSXETG00000046916 | ENSXETG00000046916 | X_tropicalis | Animals | mono_intronic | H/ACA | ENSXETG00000031917 | | protein_coding |
| ENSXETG00000047021 | U8 | X_tropicalis | Animals | mono_intergenic | C/D | | | intergenic |
| ENSXETG00000047065 | SNORD14 | X_tropicalis | Animals | mono_intronic | C/D | ENSXETG00000013038 | hspa8 | protein_coding |
| ENSXETG00000047069 | ENSXETG00000047069 | X_tropicalis | Animals | mono_intronic | H/ACA | ENSXETG00000049411 | rpl23 | protein_coding |
| ENSXETG00000047115 | ENSXETG00000047115 | X_tropicalis | Animals | mono_intronic | C/D | ENSXETG00000035654 | wdr43 | protein_coding |
| ENSXETG00000047178 | U3 | X_tropicalis | Animals | mono_intergenic | C/D | | | intergenic |
| ENSXETG00000047251 | U3 | X_tropicalis | Animals | mono_intergenic | C/D | | | intergenic |
| ENSXETG00000047297 | ENSXETG00000047297 | X_tropicalis | Animals | mono_intronic | H/ACA | ENSXETG00000049197 | rpl12 | protein_coding |
| ENSXETG00000047320 | U3 | X_tropicalis | Animals | mono_intergenic | C/D | | | intergenic |
| ENSXETG00000047535 | SNORD62 | X_tropicalis | Animals | mono_intronic | C/D | ENSXETG00000001480 | prrc2b | protein_coding |
| ENSXETG00000047562 | ENSXETG00000047562 | X_tropicalis | Animals | mono_intergenic | C/D | | | intergenic |
| ENSXETG00000047579 | U3 | X_tropicalis | Animals | mono_intergenic | C/D | | | intergenic |
| ENSXETG00000047582 | U3 | X_tropicalis | Animals | mono_intergenic | C/D | | | intergenic |
| ENSXETG00000047602 | SNORD24 | X_tropicalis | Animals | mono_intronic | C/D | ENSXETG00000042208 | rpl7a | protein_coding |
| ENSXETG00000047631 | U3 | X_tropicalis | Animals | mono_intergenic | C/D | | | intergenic |
| ENSXETG00000047636 | ENSXETG00000047636 | X_tropicalis | Animals | intergenic_cluster | H/ACA | | | intergenic |
| ENSXETG00000047819 | ENSXETG00000047819 | X_tropicalis | Animals | intergenic_cluster | C/D | | | intergenic |
| ENSXETG00000047918 | ENSXETG00000047918 | X_tropicalis | Animals | mono_intergenic | H/ACA | | | intergenic |
| ENSXETG00000047962 | ENSXETG00000047962 | X_tropicalis | Animals | mono_intronic | C/D | ENSXETG00000035654 | wdr43 | protein_coding |
| ENSXETG00000047990 | ENSXETG00000047990 | X_tropicalis | Animals | mono_intergenic | C/D | | | intergenic |

| | | | | | | | | |
|---|---|---|---|---|---|---|---|---|
| ENSXETG00000048017 | U3 | X_tropicalis | Animals | mono_intergenic | C/D | | | intergenic |
| ENSXETG00000048144 | U8 | X_tropicalis | Animals | mono_intergenic | C/D | | | intergenic |
| ENSXETG00000048245 | ENSXETG00000048245 | X_tropicalis | Animals | mono_intergenic | H/ACA | | | intergenic |
| ENSXETG00000048248 | ENSXETG00000048248 | X_tropicalis | Animals | intergenic_cluster | C/D | | | intergenic |
| ENSXETG00000048318 | U3 | X_tropicalis | Animals | mono_intergenic | C/D | | | intergenic |
| ENSXETG00000048450 | ENSXETG00000048450 | X_tropicalis | Animals | mono_intergenic | C/D | | | intergenic |
| ENSXETG00000048505 | U3 | X_tropicalis | Animals | mono_intergenic | C/D | | | intergenic |
| ENSXETG00000048521 | SNORD58 | X_tropicalis | Animals | mono_intronic | C/D | ENSXETG00000020308 | rpl17 | protein_coding |
| ENSXETG00000048553 | SNORD14 | X_tropicalis | Animals | mono_intronic | C/D | ENSXETG00000026454 | rps13 | protein_coding |
| ENSXETG00000048568 | U3 | X_tropicalis | Animals | mono_intergenic | C/D | | | intergenic |
| ENSXETG00000048609 | ENSXETG00000048609 | X_tropicalis | Animals | intergenic_cluster | C/D | | | intergenic |
| ENSXETG00000048613 | ENSXETG00000048613 | X_tropicalis | Animals | intergenic_cluster | C/D | | | intergenic |
| ENSXETG00000048619 | U3 | X_tropicalis | Animals | mono_intergenic | C/D | | | intergenic |
| ENSXETG00000048685 | U3 | X_tropicalis | Animals | mono_intergenic | C/D | | | intergenic |
| ENSXETG00000048780 | ENSXETG00000048780 | X_tropicalis | Animals | intergenic_cluster | H/ACA | | | intergenic |
| ENSXETG00000048812 | U3 | X_tropicalis | Animals | mono_intergenic | C/D | | | intergenic |
| ENSXETG00000048815 | SNORD62 | X_tropicalis | Animals | mono_intronic | C/D | ENSXETG00000001480 | prrc2b | protein_coding |
| ENSXETG00000048879 | ENSXETG00000048879 | X_tropicalis | Animals | intergenic_cluster | H/ACA | | | intergenic |
| ENSXETG00000048880 | ENSXETG00000048880 | X_tropicalis | Animals | mono_intergenic | C/D | | | intergenic |
| ENSXETG00000048964 | ENSXETG00000048964 | X_tropicalis | Animals | intronic_cluster | C/D | ENSXETG00000008923 | | protein_coding |
| ENSXETG00000048991 | ENSXETG00000048991 | X_tropicalis | Animals | intergenic_cluster | C/D | | | intergenic |
| ENSXETG00000049003 | ENSXETG00000049003 | X_tropicalis | Animals | mono_intergenic | C/D | | | intergenic |
| ENSXETG00000049014 | ENSXETG00000049014 | X_tropicalis | Animals | mono_intergenic | C/D | | | intergenic |
| ENSXETG00000049015 | U3 | X_tropicalis | Animals | mono_intergenic | C/D | | | intergenic |
| ENSXETG00000049054 | U3 | X_tropicalis | Animals | mono_intergenic | C/D | | | intergenic |
| ENSXETG00000049124 | ENSXETG00000049124 | X_tropicalis | Animals | mono_intergenic | H/ACA | | | intergenic |
| ENSXETG00000049151 | ENSXETG00000049151 | X_tropicalis | Animals | mono_intergenic | H/ACA | | | intergenic |
| ENSXETG00000049250 | SNORD16 | X_tropicalis | Animals | mono_intronic | C/D | ENSXETG00000015898 | rpl4 | protein_coding |
| ENSXETG00000049278 | ENSXETG00000049278 | X_tropicalis | Animals | mono_intronic | H/ACA | ENSXETG00000010241 | eif3a | protein_coding |
| ENSXETG00000049333 | SNORD14 | X_tropicalis | Animals | mono_intronic | C/D | ENSXETG00000026454 | rps13 | protein_coding |
| ENSXETG00000049425 | U3 | X_tropicalis | Animals | mono_intergenic | C/D | | | intergenic |
| ENSXETG00000049434 | U3 | X_tropicalis | Animals | mono_intergenic | C/D | | | intergenic |
| ENSXETG00000049451 | U3 | X_tropicalis | Animals | mono_intergenic | C/D | | | intergenic |
| C1_01720C_A | C1_01720C_A | C_albicans | Fungi | mono_intergenic | Unknown | | | intergenic |
| C1_05550C_A | C1_05550C_A | C_albicans | Fungi | mono_intergenic | Unknown | | | intergenic |
| C1_05730W_A | C1_05730W_A | C_albicans | Fungi | mono_intronic | Unknown | C1_05720W_A | | protein_coding |
| C1_06300W_A | C1_06300W_A | C_albicans | Fungi | intergenic_cluster | Unknown | | | intergenic |
| C1_06310W_A | C1_06310W_A | C_albicans | Fungi | intergenic_cluster | Unknown | | | intergenic |
| C1_06490C_A | C1_06490C_A | C_albicans | Fungi | mono_intergenic | Unknown | | | intergenic |
| C1_06830W_A | C1_06830W_A | C_albicans | Fungi | mono_intergenic | Unknown | | | intergenic |
| C1_07320W_A | C1_07320W_A | C_albicans | Fungi | mono_intergenic | Unknown | | | intergenic |
| C1_07450W_A | C1_07450W_A | C_albicans | Fungi | mono_intergenic | Unknown | | | intergenic |
| C1_07940W_A | C1_07940W_A | C_albicans | Fungi | mono_intergenic | C/D | | | intergenic |

| | | | | | | | | |
|---|---|---|---|---|---|---|---|---|
| C1_08960W_A | C1_08960W_A | C_albicans | Fungi | intergenic_cluster | Unknown | | | intergenic |
| C1_08970W_A | C1_08970W_A | C_albicans | Fungi | intergenic_cluster | Unknown | | | intergenic |
| C1_09560C_A | C1_09560C_A | C_albicans | Fungi | mono_intergenic | H/ACA | | | intergenic |
| C1_10130W_A | C1_10130W_A | C_albicans | Fungi | mono_intergenic | Unknown | | | intergenic |
| C1_10590W_A | C1_10590W_A | C_albicans | Fungi | mono_intergenic | Unknown | | | intergenic |
| C1_11180C_A | C1_11180C_A | C_albicans | Fungi | mono_intergenic | Unknown | | | intergenic |
| C1_11780W_A | C1_11780W_A | C_albicans | Fungi | mono_intergenic | Unknown | | | intergenic |
| C1_12980W_A | C1_12980W_A | C_albicans | Fungi | intergenic_cluster | C/D | | | intergenic |
| C1_12990W_A | C1_12990W_A | C_albicans | Fungi | intergenic_cluster | Unknown | | | intergenic |
| C1_13000W_A | C1_13000W_A | C_albicans | Fungi | intergenic_cluster | Unknown | | | intergenic |
| C1_13040C_A | C1_13040C_A | C_albicans | Fungi | mono_intergenic | Unknown | | | intergenic |
| C1_13500C_A | C1_13500C_A | C_albicans | Fungi | mono_intergenic | H/ACA | | | intergenic |
| C2_02080W_A | C2_02080W_A | C_albicans | Fungi | mono_intergenic | C/D | | | intergenic |
| C2_03580W_A | C2_03580W_A | C_albicans | Fungi | mono_intergenic | Unknown | | | intergenic |
| C2_04610C_A | C2_04610C_A | C_albicans | Fungi | mono_intergenic | Unknown | | | intergenic |
| C2_05310C_A | C2_05310C_A | C_albicans | Fungi | mono_intronic | Unknown | C2_05300C_A | | protein_coding |
| C2_05420C_A | C2_05420C_A | C_albicans | Fungi | mono_intergenic | Unknown | | | intergenic |
| C2_05600W_A | C2_05600W_A | C_albicans | Fungi | mono_intergenic | Unknown | | | intergenic |
| C2_05880W_A | C2_05880W_A | C_albicans | Fungi | mono_exonic | Unknown | C2_05870W_A | | protein_coding |
| C2_06400C_A | C2_06400C_A | C_albicans | Fungi | mono_intronic | C/D | C2_06390C_A | IMH3 | protein_coding |
| C2_06410W_A | C2_06410W_A | C_albicans | Fungi | mono_intergenic | Unknown | | | intergenic |
| C2_06560W_A | C2_06560W_A | C_albicans | Fungi | mono_intergenic | Unknown | | | intergenic |
| C2_09540W_A | C2_09540W_A | C_albicans | Fungi | intergenic_cluster | Unknown | | | intergenic |
| C2_09550C_A | C2_09550C_A | C_albicans | Fungi | intergenic_cluster | Unknown | | | intergenic |
| C2_10800W_A | C2_10800W_A | C_albicans | Fungi | mono_intergenic | C/D | | | intergenic |
| C3_04150W_A | C3_04150W_A | C_albicans | Fungi | mono_intergenic | C/D | | | intergenic |
| C3_05820W_A | C3_05820W_A | C_albicans | Fungi | mono_intergenic | Unknown | | | intergenic |
| C3_07510C_A | SNR52 | C_albicans | Fungi | mono_intergenic | Unknown | | | intergenic |
| C4_01570C_A | C4_01570C_A | C_albicans | Fungi | mono_intergenic | Unknown | | | intergenic |
| C4_01580C_A | C4_01580C_A | C_albicans | Fungi | intergenic_cluster | C/D | | | intergenic |
| C4_01590C_A | C4_01590C_A | C_albicans | Fungi | intergenic_cluster | Unknown | | | intergenic |
| C4_01600C_A | C4_01600C_A | C_albicans | Fungi | intergenic_cluster | C/D | | | intergenic |
| C4_01610C_A | C4_01610C_A | C_albicans | Fungi | intergenic_cluster | Unknown | | | intergenic |
| C4_01620C_A | C4_01620C_A | C_albicans | Fungi | intergenic_cluster | C/D | | | intergenic |
| C4_01630C_A | C4_01630C_A | C_albicans | Fungi | intergenic_cluster | C/D | | | intergenic |
| C4_01640C_A | C4_01640C_A | C_albicans | Fungi | intergenic_cluster | Unknown | | | intergenic |
| C4_02650C_A | C4_02650C_A | C_albicans | Fungi | mono_intergenic | C/D | | | intergenic |
| C4_04490C_A | C4_04490C_A | C_albicans | Fungi | mono_intronic | C/D | C4_04480C_A | EFB1 | protein_coding |
| C4_06520W_A | C4_06520W_A | C_albicans | Fungi | mono_intergenic | Unknown | | | intergenic |
| C5_01030W_A | C5_01030W_A | C_albicans | Fungi | mono_intergenic | H/ACA | | | intergenic |
| C5_01150C_A | C5_01150C_A | C_albicans | Fungi | intergenic_cluster | Unknown | | | intergenic |
| C5_01160C_A | C5_01160C_A | C_albicans | Fungi | intergenic_cluster | Unknown | | | intergenic |
| C5_02850W_A | C5_02850W_A | C_albicans | Fungi | mono_intergenic | Unknown | | | intergenic |

| | | | | | | | | |
|---|---|---|---|---|---|---|---|---|
| C5_03450W_A | C5_03450W_A | C_albicans | Fungi | mono_intergenic | C/D | | | intergenic |
| C6_00220C_A | C6_00220C_A | C_albicans | Fungi | mono_intergenic | Unknown | | | intergenic |
| C6_01540C_A | C6_01540C_A | C_albicans | Fungi | mono_intergenic | Unknown | | | intergenic |
| C6_02390W_A | C6_02390W_A | C_albicans | Fungi | intergenic_cluster | Unknown | | | intergenic |
| C6_02400W_A | C6_02400W_A | C_albicans | Fungi | intergenic_cluster | Unknown | | | intergenic |
| C7_00540W_A | C7_00540W_A | C_albicans | Fungi | mono_intergenic | Unknown | | | intergenic |
| C7_01260W_A | C7_01260W_A | C_albicans | Fungi | mono_intronic | Unknown | C7_01250W_A | ASC1 | protein_coding |
| C7_02190W_A | C7_02190W_A | C_albicans | Fungi | mono_intergenic | Unknown | | | intergenic |
| CR_00450C_A | CR_00450C_A | C_albicans | Fungi | mono_intergenic | Unknown | | | intergenic |
| CR_02320W_A | CR_02320W_A | C_albicans | Fungi | mono_intergenic | C/D | | | intergenic |
| CR_02470W_A | CR_02470W_A | C_albicans | Fungi | mono_intergenic | Unknown | | | intergenic |
| CR_02660C_A | CR_02660C_A | C_albicans | Fungi | mono_intergenic | Unknown | | | intergenic |
| CR_05350W_A | CR_05350W_A | C_albicans | Fungi | mono_intergenic | Unknown | | | intergenic |
| CR_05470C_A | CR_05470C_A | C_albicans | Fungi | mono_intergenic | Unknown | | | intergenic |
| CR_06190C_A | CR_06190C_A | C_albicans | Fungi | intergenic_cluster | C/D | | | intergenic |
| CR_06200C_A | CR_06200C_A | C_albicans | Fungi | intergenic_cluster | C/D | | | intergenic |
| CR_06210C_A | CR_06210C_A | C_albicans | Fungi | intergenic_cluster | Unknown | | | intergenic |
| CR_06220C_A | CR_06220C_A | C_albicans | Fungi | intergenic_cluster | C/D | | | intergenic |
| CR_06600C_A | CR_06600C_A | C_albicans | Fungi | mono_intergenic | Unknown | | | intergenic |
| CR_06630W_A | CR_06630W_A | C_albicans | Fungi | mono_intergenic | Unknown | | | intergenic |
| CR_08140W_A | CR_08140W_A | C_albicans | Fungi | mono_intergenic | Unknown | | | intergenic |
| CR_10460W_A | CR_10460W_A | C_albicans | Fungi | mono_intergenic | Unknown | | | intergenic |
| ENSRNA049513387 | Fungi_U3 | N_crassa | Fungi | mono_intergenic | C/D | | | intergenic |
| ENSRNA049513523 | snoZ13_snr52 | N_crassa | Fungi | mono_intronic | C/D | NCU03366 | | protein_coding |
| ENSRNA049514225 | SNORD14 | N_crassa | Fungi | mono_intronic | C/D | NCU14140 | | non_coding |
| ENSRNA049514776 | snosnR60_Z15 | N_crassa | Fungi | mono_intronic | C/D | NCU04647 | | protein_coding |
| ENSRNA049515271 | Fungi_U3 | N_crassa | Fungi | mono_intergenic | C/D | | | intergenic |
| ENSRNA049515290 | Fungi_U3 | N_crassa | Fungi | mono_intergenic | C/D | | | intergenic |
| ENSRNA049515376 | Afu_335 | N_crassa | Fungi | mono_intronic | C/D | NCU02611 | | protein_coding |
| ENSRNA049515544 | Afu_455 | N_crassa | Fungi | mono_intergenic | C/D | | | intergenic |
| ENSRNA049515692 | snR191 | N_crassa | Fungi | mono_intronic | H/ACA | NCU02546 | | protein_coding |
| ENSRNA049515785 | snosnR69 | N_crassa | Fungi | mono_intronic | C/D | NCU02044 | | protein_coding |
| ENSRNA049515857 | Afu_264 | N_crassa | Fungi | mono_intergenic | C/D | | | intergenic |
| ENSRNA049515901 | Afu_514 | N_crassa | Fungi | mono_exonic | C/D | NCU16323 | | protein_coding |
| snR10 | snR10 | S_cerevisiae | Fungi | mono_intergenic | H/ACA | | | intergenic |
| snR11 | snR11 | S_cerevisiae | Fungi | mono_intergenic | H/ACA | | | intergenic |
| snR128 | snR128 | S_cerevisiae | Fungi | intergenic_cluster | C/D | | | intergenic |
| snR13 | snR13 | S_cerevisiae | Fungi | mono_intergenic | C/D | | | intergenic |
| snR161 | snR161 | S_cerevisiae | Fungi | mono_intergenic | H/ACA | | | intergenic |
| snR17a | snR17a | S_cerevisiae | Fungi | mono_intergenic | C/D | | | intergenic |
| snR17b | snR17b | S_cerevisiae | Fungi | mono_intergenic | C/D | | | intergenic |
| snR18 | snR18 | S_cerevisiae | Fungi | mono_intronic | C/D | YAL003W | EFB1 | protein_coding |
| snR189 | snR189 | S_cerevisiae | Fungi | mono_intergenic | H/ACA | | | intergenic |

| | | | | | | | | |
|---|---|---|---|---|---|---|---|---|
| snR190 | snR190 | S_cerevisiae | Fungi | intergenic_cluster | C/D | | | intergenic |
| snR191 | snR191 | S_cerevisiae | Fungi | mono_intronic | H/ACA | YNR053C | NOG2 | protein_coding |
| snR24 | snR24 | S_cerevisiae | Fungi | mono_intronic | C/D | YMR116C | ASC1 | protein_coding |
| snR3 | snR3 | S_cerevisiae | Fungi | mono_intergenic | H/ACA | | | intergenic |
| snR30 | snR30 | S_cerevisiae | Fungi | mono_intergenic | H/ACA | | | intergenic |
| snR31 | snR31 | S_cerevisiae | Fungi | intergenic_cluster | H/ACA | | | intergenic |
| snR32 | snR32 | S_cerevisiae | Fungi | mono_intergenic | H/ACA | | | intergenic |
| snR33 | snR33 | S_cerevisiae | Fungi | mono_intergenic | H/ACA | | | intergenic |
| snR34 | snR34 | S_cerevisiae | Fungi | mono_intergenic | H/ACA | | | intergenic |
| snR35 | snR35 | S_cerevisiae | Fungi | mono_intergenic | H/ACA | | | intergenic |
| snR36 | snR36 | S_cerevisiae | Fungi | mono_intergenic | H/ACA | | | intergenic |
| snR37 | snR37 | S_cerevisiae | Fungi | mono_intergenic | H/ACA | | | intergenic |
| snR38 | snR38 | S_cerevisiae | Fungi | mono_intronic | C/D | YKL081W | TEF4 | protein_coding |
| snR39 | snR39 | S_cerevisiae | Fungi | mono_intronic | C/D | YGL076C | RPL7A | protein_coding |
| snR39B | snR39B | S_cerevisiae | Fungi | mono_intergenic | C/D | | | intergenic |
| snR4 | snR4 | S_cerevisiae | Fungi | mono_intergenic | C/D | | | intergenic |
| snR40 | snR40 | S_cerevisiae | Fungi | mono_intergenic | C/D | | | intergenic |
| snR41 | snR41 | S_cerevisiae | Fungi | intergenic_cluster | C/D | | | intergenic |
| snR42 | snR42 | S_cerevisiae | Fungi | mono_intergenic | H/ACA | | | intergenic |
| snR43 | snR43 | S_cerevisiae | Fungi | mono_intergenic | H/ACA | | | intergenic |
| snR44 | snR44 | S_cerevisiae | Fungi | mono_intronic | H/ACA | YLR367W | RPS22B | protein_coding |
| snR45 | snR45 | S_cerevisiae | Fungi | mono_intergenic | C/D | | | intergenic |
| snR46 | snR46 | S_cerevisiae | Fungi | mono_intergenic | H/ACA | | | intergenic |
| snR47 | snR47 | S_cerevisiae | Fungi | mono_intergenic | C/D | | | intergenic |
| snR48 | snR48 | S_cerevisiae | Fungi | mono_intergenic | C/D | | | intergenic |
| snR49 | snR49 | S_cerevisiae | Fungi | mono_intergenic | H/ACA | | | intergenic |
| snR5 | snR5 | S_cerevisiae | Fungi | intergenic_cluster | H/ACA | | | intergenic |
| snR50 | snR50 | S_cerevisiae | Fungi | mono_intergenic | C/D | | | intergenic |
| snR51 | snR51 | S_cerevisiae | Fungi | intergenic_cluster | C/D | | | intergenic |
| snR52 | snR52 | S_cerevisiae | Fungi | mono_intergenic | C/D | | | intergenic |
| snR53 | snR53 | S_cerevisiae | Fungi | intergenic_cluster | C/D | | | intergenic |
| snR54 | snR54 | S_cerevisiae | Fungi | mono_intronic | C/D | YML056C | IMD4 | protein_coding |
| snR55 | snR55 | S_cerevisiae | Fungi | intergenic_cluster | C/D | | | intergenic |
| snR56 | snR56 | S_cerevisiae | Fungi | mono_intergenic | C/D | | | intergenic |
| snR57 | snR57 | S_cerevisiae | Fungi | intergenic_cluster | C/D | | | intergenic |
| snR58 | snR58 | S_cerevisiae | Fungi | mono_intergenic | C/D | | | intergenic |
| snR59 | snR59 | S_cerevisiae | Fungi | mono_intronic | C/D | YPL198W | RPL7B | protein_coding |
| snR60 | snR60 | S_cerevisiae | Fungi | mono_intergenic | C/D | | | intergenic |
| snR61 | snR61 | S_cerevisiae | Fungi | intergenic_cluster | C/D | | | intergenic |
| snR62 | snR62 | S_cerevisiae | Fungi | mono_intergenic | C/D | | | intergenic |
| snR63 | snR63 | S_cerevisiae | Fungi | mono_intergenic | C/D | | | intergenic |
| snR64 | snR64 | S_cerevisiae | Fungi | mono_intergenic | C/D | | | intergenic |
| snR65 | snR65 | S_cerevisiae | Fungi | mono_intergenic | C/D | | | intergenic |

| | | | | | | | | |
|---|---|---|---|---|---|---|---|---|
| snR66 | snR66 | S_cerevisiae | Fungi | intergenic_cluster | C/D | | | intergenic |
| snR67 | snR67 | S_cerevisiae | Fungi | intergenic_cluster | C/D | | | intergenic |
| snR68 | snR68 | S_cerevisiae | Fungi | mono_intergenic | C/D | | | intergenic |
| snR69 | snR69 | S_cerevisiae | Fungi | mono_intergenic | C/D | | | intergenic |
| snR70 | snR70 | S_cerevisiae | Fungi | intergenic_cluster | C/D | | | intergenic |
| snR71 | snR71 | S_cerevisiae | Fungi | mono_intergenic | C/D | | | intergenic |
| snR72 | snR72 | S_cerevisiae | Fungi | intergenic_cluster | C/D | | | intergenic |
| snR73 | snR73 | S_cerevisiae | Fungi | intergenic_cluster | C/D | | | intergenic |
| snR74 | snR74 | S_cerevisiae | Fungi | intergenic_cluster | C/D | | | intergenic |
| snR75 | snR75 | S_cerevisiae | Fungi | intergenic_cluster | C/D | | | intergenic |
| snR76 | snR76 | S_cerevisiae | Fungi | intergenic_cluster | C/D | | | intergenic |
| snR77 | snR77 | S_cerevisiae | Fungi | intergenic_cluster | C/D | | | intergenic |
| snR78 | snR78 | S_cerevisiae | Fungi | intergenic_cluster | C/D | | | intergenic |
| snR79 | snR79 | S_cerevisiae | Fungi | mono_intergenic | C/D | | | intergenic |
| snR8 | snR8 | S_cerevisiae | Fungi | mono_intergenic | H/ACA | | | intergenic |
| snR80 | snR80 | S_cerevisiae | Fungi | mono_intergenic | H/ACA | | | intergenic |
| snR81 | snR81 | S_cerevisiae | Fungi | mono_intergenic | H/ACA | | | intergenic |
| snR82 | snR82 | S_cerevisiae | Fungi | mono_intergenic | H/ACA | | | intergenic |
| snR83 | snR83 | S_cerevisiae | Fungi | mono_intergenic | H/ACA | | | intergenic |
| snR84 | snR84 | S_cerevisiae | Fungi | mono_intergenic | H/ACA | | | intergenic |
| snR85 | snR85 | S_cerevisiae | Fungi | mono_intergenic | H/ACA | | | intergenic |
| snR86 | snR86 | S_cerevisiae | Fungi | mono_intergenic | H/ACA | | | intergenic |
| snR87 | snR87 | S_cerevisiae | Fungi | mono_intergenic | C/D | | | intergenic |
| snR9 | snR9 | S_cerevisiae | Fungi | mono_intergenic | H/ACA | | | intergenic |
| ENSRNA049676222 | S_pombe_snR98 | S_pombe | Fungi | exonic_cluster | H/ACA | SPNCRNA.814 | | non_coding |
| ENSRNA049676282 | Fungi_U3 | S_pombe | Fungi | intergenic_cluster | C/D | | | intergenic |
| ENSRNA049676312 | S_pombe_snR90 | S_pombe | Fungi | exonic_cluster | H/ACA | SPNCRNA.1038 | | non_coding |
| ENSRNA049676761 | snR79 | S_pombe | Fungi | intergenic_cluster | C/D | | | intergenic |
| ENSRNA049676807 | snR75 | S_pombe | Fungi | exonic_cluster | C/D | SPNCRNA.659 | psf3-antisense-1 | non_coding |
| ENSRNA049676829 | snR56 | S_pombe | Fungi | intergenic_cluster | Unknown | | | intergenic |
| ENSRNA049676922 | snoZ196 | S_pombe | Fungi | intronic_cluster | Unknown | SPAC664.06 | rlp7 | protein_coding |
| ENSRNA049676952 | SNORD24 | S_pombe | Fungi | intronic_cluster | C/D | SPAC4D7.10c | spt20 | protein_coding |
| ENSRNA049677710 | Fungi_U3 | S_pombe | Fungi | intergenic_cluster | C/D | | | intergenic |
| ENSRNA049677755 | S_pombe_snR5 | S_pombe | Fungi | intergenic_cluster | H/ACA | | | intergenic |
| ENSRNA049678217 | SNORD14 | S_pombe | Fungi | intergenic_cluster | Unknown | | | intergenic |
| ENSRNA049678266 | snosnR61 | S_pombe | Fungi | intergenic_cluster | C/D | | | intergenic |
| SPNCRNA.445 | snoR61 | S_pombe | Fungi | intergenic_cluster | C/D | | | intergenic |
| SPNCRNA.499 | snR191 | S_pombe | Fungi | mono_intronic | Unknown | SPAC6F6.03c | | protein_coding |
| SPSNORNA.01 | snR40 | S_pombe | Fungi | mono_intergenic | C/D | | | intergenic |
| SPSNORNA.02 | snoZ8 | S_pombe | Fungi | exonic_cluster | C/D | SPNCRNA.659 | psf3-antisense-1 | non_coding |
| SPSNORNA.03 | snoZ7 | S_pombe | Fungi | exonic_cluster | C/D | SPNCRNA.659 | psf3-antisense-1 | non_coding |
| SPSNORNA.04 | snoZ5 | S_pombe | Fungi | exonic_cluster | C/D | SPNCRNA.659 | psf3-antisense-1 | non_coding |
| SPSNORNA.05 | snoZ4 | S_pombe | Fungi | exonic_cluster | C/D | SPNCRNA.659 | psf3-antisense-1 | non_coding |

| | | | | | | | | |
|---|---|---|---|---|---|---|---|---|
| SPSNORNA.06 | snoZ3 | S_pombe | Fungi | exonic_cluster | C/D | SPNCRNA.659 | psf3-antisense-1 | non_coding |
| SPSNORNA.07 | snR41 | S_pombe | Fungi | exonic_cluster | C/D | SPNCRNA.723 | | non_coding |
| SPSNORNA.08 | snR70 | S_pombe | Fungi | exonic_cluster | C/D | SPNCRNA.723 | | non_coding |
| SPSNORNA.09 | snR51b | S_pombe | Fungi | exonic_cluster | C/D | SPNCRNA.723 | | non_coding |
| SPSNORNA.10 | snoZ16 | S_pombe | Fungi | mono_exonic | C/D | SPNCRNA.727 | | non_coding |
| SPSNORNA.11 | snoU24b | S_pombe | Fungi | mono_intronic | C/D | SPAC6B12.15 | cpc2 | protein_coding |
| SPSNORNA.12 | snoR02 | S_pombe | Fungi | mono_intergenic | C/D | | | intergenic |
| SPSNORNA.13 | snoR69b | S_pombe | Fungi | mono_intergenic | C/D | | | intergenic |
| SPSNORNA.14 | snoR54b | S_pombe | Fungi | mono_intergenic | C/D | | | intergenic |
| SPSNORNA.15 | snoR38 | S_pombe | Fungi | mono_intronic | C/D | SPAC29A4.02c | | protein_coding |
| SPSNORNA.16 | snoR56 | S_pombe | Fungi | intergenic_cluster | C/D | | | intergenic |
| SPSNORNA.17 | snoR58 | S_pombe | Fungi | mono_intronic | C/D | SPAC4D7.08c | ade4 | protein_coding |
| SPSNORNA.18 | snoU24 | S_pombe | Fungi | intronic_cluster | C/D | SPAC4D7.10c | spt20 | protein_coding |
| SPSNORNA.19 | snoR69 | S_pombe | Fungi | mono_intergenic | C/D | | | intergenic |
| SPSNORNA.20 | snR30 | S_pombe | Fungi | mono_intergenic | H/ACA | | | intergenic |
| SPSNORNA.21 | snoU14 | S_pombe | Fungi | intergenic_cluster | C/D | | | intergenic |
| SPSNORNA.22 | snoR54 | S_pombe | Fungi | mono_intronic | C/D | SPBC2F12.14c | gua1 | protein_coding |
| SPSNORNA.23 | snoZ15 | S_pombe | Fungi | mono_intronic | C/D | SPBC1D7.01 | gim6 | protein_coding |
| SPSNORNA.24 | snoR39b | S_pombe | Fungi | mono_intronic | C/D | SPBC18H10.12c | rpl701 | protein_coding |
| SPSNORNA.25 | snoZ30 | S_pombe | Fungi | mono_exonic | C/D | SPNCRNA.1483 | | non_coding |
| SPSNORNA.26 | snoU18 | S_pombe | Fungi | mono_intronic | C/D | SPBC4F6.16c | ero11 | protein_coding |
| SPSNORNA.27 | snoR47 | S_pombe | Fungi | mono_intronic | C/D | SPBP8B7.12c | fta3 | protein_coding |
| SPSNORNA.28 | snoR68 | S_pombe | Fungi | mono_exonic | C/D | SPNCRNA.1650 | | non_coding |
| SPSNORNA.29 | sno52 | S_pombe | Fungi | mono_intergenic | C/D | | | intergenic |
| SPSNORNA.30 | snr62 | S_pombe | Fungi | mono_exonic | C/D | SPAC4H3.12c | | protein_coding |
| SPSNORNA.31 | snoR39a | S_pombe | Fungi | intronic_cluster | C/D | SPAC664.06 | rlp7 | protein_coding |
| SPSNORNA.32 | sno12 | S_pombe | Fungi | mono_intergenic | H/ACA | | | intergenic |
| SPSNORNA.33 | sno16 | S_pombe | Fungi | mono_exonic | H/ACA | SPNCRNA.1597 | SPBC3B8.03-antisense-1 | non_coding |
| SPSNORNA.34 | sno20 | S_pombe | Fungi | mono_intergenic | H/ACA | | | intergenic |
| SPSNORNA.35 | snR3 | S_pombe | Fungi | mono_exonic | H/ACA | SPNCRNA.1394 | | non_coding |
| SPSNORNA.36 | snR5 | S_pombe | Fungi | intergenic_cluster | H/ACA | | | intergenic |
| SPSNORNA.37 | snR10 | S_pombe | Fungi | mono_intergenic | H/ACA | | | intergenic |
| SPSNORNA.38 | snR33 | S_pombe | Fungi | mono_intergenic | H/ACA | | | intergenic |
| SPSNORNA.39 | snR36 | S_pombe | Fungi | mono_intergenic | H/ACA | | | intergenic |
| SPSNORNA.40 | snR42 | S_pombe | Fungi | mono_intergenic | H/ACA | | | intergenic |
| SPSNORNA.41 | snR46 | S_pombe | Fungi | mono_intergenic | H/ACA | | | intergenic |
| SPSNORNA.42 | snR90 | S_pombe | Fungi | exonic_cluster | H/ACA | SPNCRNA.1038 | | non_coding |
| SPSNORNA.43 | snR91 | S_pombe | Fungi | mono_intergenic | H/ACA | | | intergenic |
| SPSNORNA.44 | snR92 | S_pombe | Fungi | mono_intergenic | H/ACA | | | intergenic |
| SPSNORNA.45 | snR93 | S_pombe | Fungi | mono_intergenic | H/ACA | | | intergenic |
| SPSNORNA.46 | snR94 | S_pombe | Fungi | mono_intergenic | H/ACA | | | intergenic |
| SPSNORNA.47 | snR95 | S_pombe | Fungi | mono_intergenic | H/ACA | | | intergenic |
| SPSNORNA.48 | snR96 | S_pombe | Fungi | mono_intergenic | H/ACA | | | intergenic |

| | | | | | | | | |
|---|---|---|---|---|---|---|---|---|
| SPSNORNA.49 | snR97 | S_pombe | Fungi | mono_exonic | H/ACA | SPNCRNA.617 | rad8-antisense-1 | non_coding |
| SPSNORNA.50 | snR98 | S_pombe | Fungi | exonic_cluster | H/ACA | SPNCRNA.814 | | non_coding |
| SPSNORNA.52 | snR101 | S_pombe | Fungi | mono_intergenic | H/ACA | | | intergenic |
| SPSNORNA.53 | snR88 | S_pombe | Fungi | mono_intergenic | C/D | | | intergenic |
| SPSNORNA.54 | snr79 | S_pombe | Fungi | intergenic_cluster | C/D | | | intergenic |
| SPSNRNA.03 | snu3 | S_pombe | Fungi | intergenic_cluster | C/D | | | intergenic |
| SPSNRNA.07 | snu32 | S_pombe | Fungi | intergenic_cluster | C/D | | | intergenic |
| AT1G03502 | AT1G03502 | A_thaliana | Plants | intergenic_cluster | C/D | | | intergenic |
| AT1G03506 | AT1G03506 | A_thaliana | Plants | intergenic_cluster | C/D | | | intergenic |
| AT1G03743 | AT1G03743 | A_thaliana | Plants | intergenic_cluster | C/D | | | intergenic |
| AT1G03746 | AT1G03746 | A_thaliana | Plants | intergenic_cluster | C/D | | | intergenic |
| AT1G03935 | AT1G03935 | A_thaliana | Plants | intergenic_cluster | C/D | | | intergenic |
| AT1G04187 | AT1G04187 | A_thaliana | Plants | intergenic_cluster | H/ACA | | | intergenic |
| AT1G04193 | AT1G04193 | A_thaliana | Plants | intergenic_cluster | C/D | | | intergenic |
| AT1G04197 | AT1G04197 | A_thaliana | Plants | intergenic_cluster | H/ACA | | | intergenic |
| AT1G04213 | AT1G04213 | A_thaliana | Plants | intergenic_cluster | Unknown | | | intergenic |
| AT1G04217 | AT1G04217 | A_thaliana | Plants | intergenic_cluster | H/ACA | | | intergenic |
| AT1G04223 | AT1G04223 | A_thaliana | Plants | intergenic_cluster | C/D | | | intergenic |
| AT1G04227 | AT1G04227 | A_thaliana | Plants | intergenic_cluster | H/ACA | | | intergenic |
| AT1G04267 | AT1G04267 | A_thaliana | Plants | intronic_cluster | Unknown | AT1G04480 | AT1G04480 | protein_coding |
| AT1G04273 | AT1G04273 | A_thaliana | Plants | intronic_cluster | Unknown | AT1G04480 | AT1G04480 | protein_coding |
| AT1G04277 | AT1G04277 | A_thaliana | Plants | intronic_cluster | Unknown | AT1G04480 | AT1G04480 | protein_coding |
| AT1G04517 | AT1G04517 | A_thaliana | Plants | mono_intergenic | Unknown | | | intergenic |
| AT1G04527 | AT1G04527 | A_thaliana | Plants | mono_intergenic | Unknown | | | intergenic |
| AT1G04547 | AT1G04547 | A_thaliana | Plants | mono_intergenic | Unknown | | | intergenic |
| AT1G05137 | AT1G05137 | A_thaliana | Plants | mono_exonic | Unknown | AT1G15002 | AT1G15002 | non_coding |
| AT1G05247 | AT1G05247 | A_thaliana | Plants | mono_exonic | Unknown | AT1G16635 | AT1G16635 | non_coding |
| AT1G05417 | AT1G05417 | A_thaliana | Plants | exonic_cluster | C/D | AT1G18745 | AT1G18745 | non_coding |
| AT1G05423 | AT1G05423 | A_thaliana | Plants | exonic_cluster | C/D | AT1G18745 | AT1G18745 | non_coding |
| AT1G05427 | AT1G05427 | A_thaliana | Plants | exonic_cluster | C/D | AT1G18745 | AT1G18745 | non_coding |
| AT1G05433 | AT1G05433 | A_thaliana | Plants | exonic_cluster | C/D | AT1G18745 | AT1G18745 | non_coding |
| AT1G05563 | AT1G05563 | A_thaliana | Plants | mono_exonic | C/D | AT1G20691 | AT1G20691 | non_coding |
| AT1G05907 | AT1G05907 | A_thaliana | Plants | mono_intergenic | C/D | | | intergenic |
| AT1G05917 | AT1G05917 | A_thaliana | Plants | mono_exonic | C/D | AT1G05913 | AT1G05913 | non_coding |
| AT1G05993 | AT1G05993 | A_thaliana | Plants | intergenic_cluster | C/D | | | intergenic |
| AT1G05997 | AT1G05997 | A_thaliana | Plants | intergenic_cluster | Unknown | | | intergenic |
| AT1G06033 | AT1G06033 | A_thaliana | Plants | mono_intronic | Unknown | AT1G26880 | AT1G26880 | protein_coding |
| AT1G06087 | AT1G06087 | A_thaliana | Plants | mono_intronic | H/ACA | AT1G27400 | AT1G27400 | protein_coding |
| AT1G06233 | AT1G06233 | A_thaliana | Plants | intergenic_cluster | C/D | | | intergenic |
| AT1G06237 | AT1G06237 | A_thaliana | Plants | intergenic_cluster | C/D | | | intergenic |
| AT1G06243 | AT1G06243 | A_thaliana | Plants | intergenic_cluster | C/D | | | intergenic |
| AT1G06247 | AT1G06247 | A_thaliana | Plants | exonic_cluster | C/D | AT1G29418 | | protein_coding |
| AT1G06253 | AT1G06253 | A_thaliana | Plants | exonic_cluster | Unknown | AT1G29418 | | protein_coding |

| | | | | | | | | |
|---|---|---|---|---|---|---|---|---|
| AT1G06263 | AT1G06263 | A_thaliana | Plants | mono_exonic | C/D | AT1G29465 | AT1G29465 | protein_coding |
| AT1G06267 | AT1G06267 | A_thaliana | Plants | intronic_cluster | C/D | AT1G29465 | AT1G29465 | protein_coding |
| AT1G06273 | AT1G06273 | A_thaliana | Plants | intronic_cluster | C/D | AT1G29465 | AT1G29465 | protein_coding |
| AT1G06277 | AT1G06277 | A_thaliana | Plants | intronic_cluster | C/D | AT1G29465 | AT1G29465 | protein_coding |
| AT1G06283 | AT1G06283 | A_thaliana | Plants | intronic_cluster | Unknown | AT1G29465 | AT1G29465 | protein_coding |
| AT1G06397 | AT1G06397 | A_thaliana | Plants | mono_exonic | Unknown | AT1G30970 | SUF4 | protein_coding |
| AT1G06453 | AT1G06453 | A_thaliana | Plants | mono_exonic | H/ACA | AT1G31835 | AT1G31835 | protein_coding |
| AT1G06457 | AT1G06457 | A_thaliana | Plants | intronic_cluster | Unknown | AT1G31860 | AT-IE | protein_coding |
| AT1G06463 | AT1G06463 | A_thaliana | Plants | intronic_cluster | Unknown | AT1G31860 | AT-IE | protein_coding |
| AT1G06467 | AT1G06467 | A_thaliana | Plants | intronic_cluster | Unknown | AT1G31860 | AT-IE | protein_coding |
| AT1G06547 | AT1G06547 | A_thaliana | Plants | intergenic_cluster | C/D | | | intergenic |
| AT1G06553 | AT1G06553 | A_thaliana | Plants | intergenic_cluster | H/ACA | | | intergenic |
| AT1G06667 | AT1G06667 | A_thaliana | Plants | mono_intergenic | C/D | | | intergenic |
| AT1G06673 | AT1G06673 | A_thaliana | Plants | mono_intergenic | C/D | | | intergenic |
| AT1G07393 | AT1G07393 | A_thaliana | Plants | mono_intergenic | Unknown | | | intergenic |
| AT1G07487 | AT1G07487 | A_thaliana | Plants | mono_exonic | C/D | AT1G48460 | AT1G48460 | protein_coding |
| AT1G07593 | AT1G07593 | A_thaliana | Plants | mono_intergenic | C/D | | | intergenic |
| AT1G07702 | AT1G07702 | A_thaliana | Plants | mono_intergenic | C/D | | | intergenic |
| AT1G07897 | AT1G07897 | A_thaliana | Plants | mono_intergenic | Unknown | | | intergenic |
| AT1G07903 | AT1G07903 | A_thaliana | Plants | mono_exonic | Unknown | AT1G53542 | AT1G53542 | protein_coding |
| AT1G07957 | AT1G07957 | A_thaliana | Plants | mono_intronic | H/ACA | AT1G54270 | EIF4A-2 | protein_coding |
| AT1G08107 | AT1G08107 | A_thaliana | Plants | mono_exonic | C/D | AT1G08103 | AT1G08103 | non_coding |
| AT1G08113 | AT1G08113 | A_thaliana | Plants | intronic_cluster | C/D | AT1G08103 | AT1G08103 | non_coding |
| AT1G08117 | AT1G08117 | A_thaliana | Plants | intronic_cluster | C/D | AT1G08103 | AT1G08103 | non_coding |
| AT1G08123 | AT1G08123 | A_thaliana | Plants | mono_exonic | C/D | AT1G08103 | AT1G08103 | non_coding |
| AT1G08327 | AT1G08327 | A_thaliana | Plants | mono_intergenic | Unknown | | | intergenic |
| AT1G08347 | AT1G08347 | A_thaliana | Plants | mono_intergenic | C/D | | | intergenic |
| AT1G08353 | AT1G08353 | A_thaliana | Plants | mono_intergenic | Unknown | | | intergenic |
| AT1G08643 | AT1G08643 | A_thaliana | Plants | mono_intergenic | C/D | | | intergenic |
| AT1G08937 | AT1G08937 | A_thaliana | Plants | mono_intronic | H/ACA | AT1G67430 | AT1G67430 | protein_coding |
| AT1G09027 | AT1G09027 | A_thaliana | Plants | mono_intergenic | Unknown | | | intergenic |
| AT1G09083 | AT1G09083 | A_thaliana | Plants | intronic_cluster | H/ACA | AT1G69620 | RPL34 | protein_coding |
| AT1G09087 | AT1G09087 | A_thaliana | Plants | intronic_cluster | H/ACA | AT1G69620 | RPL34 | protein_coding |
| AT1G09333 | AT1G09333 | A_thaliana | Plants | mono_exonic | Unknown | AT1G72645 | AT1G72645 | protein_coding |
| AT1G09467 | AT1G09467 | A_thaliana | Plants | intergenic_cluster | C/D | | | intergenic |
| AT1G09533 | AT1G09533 | A_thaliana | Plants | intergenic_cluster | Unknown | | | intergenic |
| AT1G09787 | AT1G09787 | A_thaliana | Plants | mono_intergenic | Unknown | | | intergenic |
| AT1G09867 | AT1G09867 | A_thaliana | Plants | mono_intergenic | Unknown | | | intergenic |
| AT1G09873 | AT1G09873 | A_thaliana | Plants | mono_exonic | Unknown | AT1G79520 | AT1G79520 | protein_coding |
| AT1G09877 | AT1G09877 | A_thaliana | Plants | mono_exonic | C/D | AT1G79520 | AT1G79520 | protein_coding |
| AT1G09883 | AT1G09883 | A_thaliana | Plants | mono_exonic | C/D | AT1G79520 | AT1G79520 | protein_coding |
| AT1G12013 | SNOR111 | A_thaliana | Plants | intergenic_cluster | H/ACA | | | intergenic |
| AT1G12015 | AT1G12015 | A_thaliana | Plants | intergenic_cluster | C/D | | | intergenic |

| | | | | | | | | |
|---|---|---|---|---|---|---|---|---|
| AT1G19373 | AT1G19373 | A_thaliana | Plants | intergenic_cluster | C/D | | | intergenic |
| AT1G19376 | AT1G19376 | A_thaliana | Plants | intergenic_cluster | Unknown | | | intergenic |
| AT1G20015 | AT1G20015 | A_thaliana | Plants | mono_intergenic | C/D | | | intergenic |
| AT1G26233 | SNOR95 | A_thaliana | Plants | intergenic_cluster | Unknown | | | intergenic |
| AT1G29071 | SNOR105 | A_thaliana | Plants | mono_intergenic | C/D | | | intergenic |
| AT1G32385 | AT1G32385 | A_thaliana | Plants | intergenic_cluster | C/D | | | intergenic |
| AT1G74453 | AT1G74453 | A_thaliana | Plants | intergenic_cluster | C/D | | | intergenic |
| AT1G74456 | AT1G74456 | A_thaliana | Plants | intergenic_cluster | Unknown | | | intergenic |
| AT1G75163 | AT1G75163 | A_thaliana | Plants | intergenic_cluster | C/D | | | intergenic |
| AT1G75166 | AT1G75166 | A_thaliana | Plants | intergenic_cluster | Unknown | | | intergenic |
| AT2G04955 | AT2G04955 | A_thaliana | Plants | intergenic_cluster | H/ACA | | | intergenic |
| AT2G04965 | AT2G04965 | A_thaliana | Plants | intergenic_cluster | C/D | | | intergenic |
| AT2G05525 | AT2G05525 | A_thaliana | Plants | mono_intergenic | Unknown | | | intergenic |
| AT2G05565 | AT2G05565 | A_thaliana | Plants | mono_intergenic | Unknown | | | intergenic |
| AT2G05765 | AT2G05765 | A_thaliana | Plants | intergenic_cluster | C/D | | | intergenic |
| AT2G06855 | AT2G06855 | A_thaliana | Plants | intergenic_cluster | C/D | | | intergenic |
| AT2G06865 | AT2G06865 | A_thaliana | Plants | intergenic_cluster | H/ACA | | | intergenic |
| AT2G07605 | AT2G07605 | A_thaliana | Plants | intergenic_cluster | C/D | | | intergenic |
| AT2G07615 | AT2G07615 | A_thaliana | Plants | intergenic_cluster | C/D | | | intergenic |
| AT2G08370 | AT2G08370 | A_thaliana | Plants | mono_intergenic | Unknown | | | intergenic |
| AT2G08375 | AT2G08375 | A_thaliana | Plants | mono_intergenic | Unknown | | | intergenic |
| AT2G08390 | AT2G08390 | A_thaliana | Plants | mono_intergenic | Unknown | | | intergenic |
| AT2G08580 | AT2G08580 | A_thaliana | Plants | intergenic_cluster | Unknown | | | intergenic |
| AT2G08585 | AT2G08585 | A_thaliana | Plants | intergenic_cluster | Unknown | | | intergenic |
| AT2G08725 | AT2G08725 | A_thaliana | Plants | intergenic_cluster | C/D | | | intergenic |
| AT2G08755 | AT2G08755 | A_thaliana | Plants | intergenic_cluster | C/D | | | intergenic |
| AT2G08760 | AT2G08760 | A_thaliana | Plants | intergenic_cluster | Unknown | | | intergenic |
| AT2G08765 | AT2G08765 | A_thaliana | Plants | intergenic_cluster | Unknown | | | intergenic |
| AT2G08770 | AT2G08770 | A_thaliana | Plants | exonic_cluster | Unknown | AT2G35750 | | protein_coding |
| AT2G08860 | AT2G08860 | A_thaliana | Plants | mono_exonic | Unknown | AT2G37250 | ADK | protein_coding |
| AT2G08900 | AT2G08900 | A_thaliana | Plants | intergenic_cluster | C/D | | | intergenic |
| AT2G08905 | AT2G08905 | A_thaliana | Plants | intergenic_cluster | C/D | | | intergenic |
| AT2G08910 | AT2G08910 | A_thaliana | Plants | intergenic_cluster | C/D | | | intergenic |
| AT2G09460 | AT2G09460 | A_thaliana | Plants | exonic_cluster | Unknown | AT2G43375 | AT2G43375 | non_coding |
| AT2G09465 | AT2G09465 | A_thaliana | Plants | exonic_cluster | Unknown | AT2G43375 | AT2G43375 | non_coding |
| AT2G09680 | AT2G09680 | A_thaliana | Plants | mono_intergenic | Unknown | | | intergenic |
| AT2G09685 | AT2G09685 | A_thaliana | Plants | exonic_cluster | Unknown | AT2G46192 | AT2G46192 | non_coding |
| AT2G09690 | AT2G09690 | A_thaliana | Plants | exonic_cluster | Unknown | AT2G46192 | AT2G46192 | non_coding |
| AT2G09695 | AT2G09695 | A_thaliana | Plants | exonic_cluster | H/ACA | AT2G46192 | AT2G46192 | non_coding |
| AT2G09700 | AT2G09700 | A_thaliana | Plants | exonic_cluster | H/ACA | AT2G46192 | AT2G46192 | non_coding |
| AT2G09705 | AT2G09705 | A_thaliana | Plants | mono_intergenic | H/ACA | | | intergenic |
| AT2G09815 | AT2G09815 | A_thaliana | Plants | mono_intronic | Unknown | AT2G47170 | ARF1A1C | protein_coding |
| AT2G17295 | AT2G17295 | A_thaliana | Plants | intergenic_cluster | C/D | | | intergenic |

| | | | | | | | | |
|---|---|---|---|---|---|---|---|---|
| AT2G20721 | AT2G20721 | A_thaliana | Plants | intergenic_cluster | C/D | | | intergenic |
| AT2G20722 | AT2G20722 | A_thaliana | Plants | intergenic_cluster | C/D | | | intergenic |
| AT2G20723 | AT2G20723 | A_thaliana | Plants | intergenic_cluster | C/D | | | intergenic |
| AT2G35382 | AT2G35382 | A_thaliana | Plants | intergenic_cluster | Unknown | | | intergenic |
| AT2G35384 | AT2G35384 | A_thaliana | Plants | intergenic_cluster | C/D | | | intergenic |
| AT2G35387 | AT2G35387 | A_thaliana | Plants | intergenic_cluster | C/D | | | intergenic |
| AT2G35742 | AT2G35742 | A_thaliana | Plants | intergenic_cluster | C/D | | | intergenic |
| AT2G35744 | AT2G35744 | A_thaliana | Plants | intergenic_cluster | C/D | | | intergenic |
| AT2G35747 | AT2G35747 | A_thaliana | Plants | exonic_cluster | C/D | AT2G35750 | | protein_coding |
| AT2G43137 | AT2G43137 | A_thaliana | Plants | intronic_cluster | C/D | AT2G43110 | AT2G43110 | protein_coding |
| AT2G43138 | AT2G43138 | A_thaliana | Plants | intronic_cluster | C/D | AT2G43110 | AT2G43110 | protein_coding |
| AT2G43139 | AT2G43139 | A_thaliana | Plants | intronic_cluster | C/D | AT2G43110 | AT2G43110 | protein_coding |
| AT2G43141 | AT2G43141 | A_thaliana | Plants | intronic_cluster | C/D | AT2G43110 | AT2G43110 | protein_coding |
| AT3G00985 | AT3G00985 | A_thaliana | Plants | mono_exonic | C/D | AT3G00980 | AT3G00980 | non_coding |
| AT3G01145 | AT3G01145 | A_thaliana | Plants | exonic_cluster | Unknown | AT3G02832 | AT3G02832 | non_coding |
| AT3G01155 | AT3G01155 | A_thaliana | Plants | exonic_cluster | Unknown | AT3G02832 | AT3G02832 | non_coding |
| AT3G01313 | AT3G01313 | A_thaliana | Plants | intergenic_cluster | C/D | | | intergenic |
| AT3G01316 | AT3G01316 | A_thaliana | Plants | intergenic_cluster | Unknown | | | intergenic |
| AT3G02045 | AT3G02045 | A_thaliana | Plants | mono_intronic | C/D | AT3G07860 | AT3G07860 | protein_coding |
| AT3G02205 | AT3G02205 | A_thaliana | Plants | mono_intronic | Unknown | AT3G09440 | AT3G09440 | protein_coding |
| AT3G02385 | AT3G02385 | A_thaliana | Plants | mono_intronic | Unknown | AT3G11400 | EIF3G1 | protein_coding |
| AT3G02435 | AT3G02435 | A_thaliana | Plants | intergenic_cluster | C/D | | | intergenic |
| AT3G02445 | AT3G02445 | A_thaliana | Plants | intergenic_cluster | C/D | | | intergenic |
| AT3G02725 | AT3G02725 | A_thaliana | Plants | intergenic_cluster | Unknown | | | intergenic |
| AT3G02795 | AT3G02795 | A_thaliana | Plants | mono_intronic | H/ACA | AT3G13920 | EIF4A1 | protein_coding |
| AT3G03175 | AT3G03175 | A_thaliana | Plants | mono_intergenic | C/D | | | intergenic |
| AT3G03415 | AT3G03415 | A_thaliana | Plants | intergenic_cluster | C/D | | | intergenic |
| AT3G03425 | AT3G03425 | A_thaliana | Plants | intergenic_cluster | Unknown | | | intergenic |
| AT3G03445 | AT3G03445 | A_thaliana | Plants | mono_exonic | Unknown | AT3G03435 | AT3G03435 | non_coding |
| AT3G03585 | AT3G03585 | A_thaliana | Plants | mono_intergenic | Unknown | | | intergenic |
| AT3G04155 | AT3G04155 | A_thaliana | Plants | intergenic_cluster | C/D | | | intergenic |
| AT3G04165 | AT3G04165 | A_thaliana | Plants | intergenic_cluster | C/D | | | intergenic |
| AT3G04175 | AT3G04175 | A_thaliana | Plants | intergenic_cluster | H/ACA | | | intergenic |
| AT3G04985 | AT3G04985 | A_thaliana | Plants | mono_exonic | H/ACA | AT3G04975 | AT3G04975 | non_coding |
| AT3G05325 | AT3G05325 | A_thaliana | Plants | intergenic_cluster | C/D | | | intergenic |
| AT3G05335 | AT3G05335 | A_thaliana | Plants | intergenic_cluster | C/D | | | intergenic |
| AT3G05355 | AT3G05355 | A_thaliana | Plants | intergenic_cluster | C/D | | | intergenic |
| AT3G05715 | AT3G05715 | A_thaliana | Plants | mono_intergenic | Unknown | | | intergenic |
| AT3G07715 | AT3G07715 | A_thaliana | Plants | intergenic_cluster | Unknown | | | intergenic |
| AT3G07725 | AT3G07725 | A_thaliana | Plants | intergenic_cluster | H/ACA | | | intergenic |
| AT3G07735 | AT3G07735 | A_thaliana | Plants | intergenic_cluster | C/D | | | intergenic |
| AT3G08165 | AT3G08165 | A_thaliana | Plants | intergenic_cluster | C/D | | | intergenic |
| AT3G08210 | AT3G08210 | A_thaliana | Plants | mono_intronic | Unknown | AT3G51800 | ATG2 | protein_coding |

| | | | | | | | | |
|---|---|---|---|---|---|---|---|---|
| AT3G08275 | AT3G08275 | A_thaliana | Plants | mono_intronic | Unknown | AT3G52590 | UBQ1 | protein_coding |
| AT3G08485 | AT3G08485 | A_thaliana | Plants | mono_exonic | C/D | AT3G55485 | AT3G55485 | non_coding |
| AT3G09225 | AT3G09225 | A_thaliana | Plants | mono_exonic | H/ACA | AT3G59710 | AT3G59710 | protein_coding |
| AT3G09865 | AT3G09865 | A_thaliana | Plants | mono_exonic | H/ACA | AT3G09855 | AT3G09855 | non_coding |
| AT3G13525 | AT3G13525 | A_thaliana | Plants | intergenic_cluster | C/D | | | intergenic |
| AT3G14475 | AT3G14475 | A_thaliana | Plants | mono_intergenic | Unknown | | | intergenic |
| AT3G21805 | AT3G21805 | A_thaliana | Plants | intergenic_cluster | C/D | | | intergenic |
| AT3G24612 | AT3G24612 | A_thaliana | Plants | mono_intergenic | C/D | | | intergenic |
| AT3G24614 | AT3G24614 | A_thaliana | Plants | exonic_cluster | Unknown | AT3G04775 | AT3G04775 | non_coding |
| AT3G24615 | AT3G24615 | A_thaliana | Plants | exonic_cluster | C/D | AT3G04775 | AT3G04775 | non_coding |
| AT3G27865 | AT3G27865 | A_thaliana | Plants | intergenic_cluster | C/D | | | intergenic |
| AT3G47342 | AT3G47342 | A_thaliana | Plants | intergenic_cluster | C/D | | | intergenic |
| AT3G47347 | AT3G47347 | A_thaliana | Plants | intergenic_cluster | Unknown | | | intergenic |
| AT3G47348 | AT3G47348 | A_thaliana | Plants | intergenic_cluster | C/D | | | intergenic |
| AT3G50825 | AT3G50825 | A_thaliana | Plants | intergenic_cluster | C/D | | | intergenic |
| AT3G58193 | AT3G58193 | A_thaliana | Plants | mono_intronic | C/D | AT3G09105 | AT3G09105 | non_coding |
| AT3G58196 | AT3G58196 | A_thaliana | Plants | mono_exonic | C/D | AT3G09105 | AT3G09105 | non_coding |
| AT4G02555 | AT4G02555 | A_thaliana | Plants | intergenic_cluster | C/D | | | intergenic |
| AT4G03295 | AT4G03295 | A_thaliana | Plants | intergenic_cluster | C/D | | | intergenic |
| AT4G03915 | AT4G03915 | A_thaliana | Plants | mono_exonic | H/ACA | AT4G03905 | AT4G03905 | non_coding |
| AT4G03925 | AT4G03925 | A_thaliana | Plants | mono_intergenic | H/ACA | | | intergenic |
| AT4G04205 | AT4G04205 | A_thaliana | Plants | intergenic_cluster | C/D | | | intergenic |
| AT4G04465 | AT4G04465 | A_thaliana | Plants | intergenic_cluster | Unknown | | | intergenic |
| AT4G04475 | AT4G04475 | A_thaliana | Plants | intergenic_cluster | C/D | | | intergenic |
| AT4G04485 | AT4G04485 | A_thaliana | Plants | intergenic_cluster | H/ACA | | | intergenic |
| AT4G05045 | AT4G05045 | A_thaliana | Plants | intergenic_cluster | Unknown | | | intergenic |
| AT4G05048 | U49.1 | A_thaliana | Plants | intergenic_cluster | C/D | | | intergenic |
| AT4G05055 | AT4G05055 | A_thaliana | Plants | intergenic_cluster | H/ACA | | | intergenic |
| AT4G06010 | AT4G06010 | A_thaliana | Plants | intergenic_cluster | C/D | | | intergenic |
| AT4G06015 | AT4G06015 | A_thaliana | Plants | intergenic_cluster | Unknown | | | intergenic |
| AT4G06240 | AT4G06240 | A_thaliana | Plants | mono_exonic | C/D | AT4G06225 | AT4G06225 | non_coding |
| AT4G06245 | AT4G06245 | A_thaliana | Plants | intergenic_cluster | C/D | | | intergenic |
| AT4G06250 | AT4G06250 | A_thaliana | Plants | intergenic_cluster | C/D | | | intergenic |
| AT4G06255 | AT4G06255 | A_thaliana | Plants | intergenic_cluster | H/ACA | | | intergenic |
| AT4G06305 | AT4G06305 | A_thaliana | Plants | intergenic_cluster | H/ACA | | | intergenic |
| AT4G06310 | AT4G06310 | A_thaliana | Plants | intergenic_cluster | C/D | | | intergenic |
| AT4G06315 | AT4G06315 | A_thaliana | Plants | intergenic_cluster | C/D | | | intergenic |
| AT4G06320 | AT4G06320 | A_thaliana | Plants | intergenic_cluster | C/D | | | intergenic |
| AT4G06705 | AT4G06705 | A_thaliana | Plants | mono_intronic | Unknown | AT4G17390 | AT4G17390 | protein_coding |
| AT4G07075 | AT4G07075 | A_thaliana | Plants | mono_exonic | H/ACA | AT4G07070 | AT4G07070 | non_coding |
| AT4G07625 | AT4G07625 | A_thaliana | Plants | intergenic_cluster | C/D | | | intergenic |
| AT4G07635 | AT4G07635 | A_thaliana | Plants | intergenic_cluster | C/D | | | intergenic |
| AT4G08125 | AT4G08125 | A_thaliana | Plants | intronic_cluster | C/D | AT4G29410 | AT4G29410 | protein_coding |

| | | | | | | | | |
|---|---|---|---|---|---|---|---|---|
| AT4G08155 | AT4G08155 | A_thaliana | Plants | intronic_cluster | C/D | AT4G29410 | AT4G29410 | protein_coding |
| AT4G08165 | AT4G08165 | A_thaliana | Plants | intronic_cluster | C/D | AT4G29410 | AT4G29410 | protein_coding |
| AT4G08175 | AT4G08175 | A_thaliana | Plants | intronic_cluster | C/D | AT4G29410 | AT4G29410 | protein_coding |
| AT4G08235 | AT4G08235 | A_thaliana | Plants | mono_exonic | Unknown | AT4G29860 | EMB2757 | protein_coding |
| AT4G08325 | AT4G08325 | A_thaliana | Plants | mono_intronic | H/ACA | AT4G30680 | AT4G30680 | protein_coding |
| AT4G08425 | AT4G08425 | A_thaliana | Plants | mono_exonic | Unknown | AT4G30993 | AT4G30993 | protein_coding |
| AT4G08615 | AT4G08615 | A_thaliana | Plants | intronic_cluster | C/D | AT4G31980 | AT4G31980 | protein_coding |
| AT4G08625 | AT4G08625 | A_thaliana | Plants | intronic_cluster | C/D | AT4G31980 | AT4G31980 | protein_coding |
| AT4G09475 | AT4G09475 | A_thaliana | Plants | mono_intronic | Unknown | AT4G36960 | AT4G36960 | protein_coding |
| AT4G09845 | AT4G09845 | A_thaliana | Plants | intergenic_cluster | H/ACA | | | intergenic |
| AT4G13245 | AT4G13245 | A_thaliana | Plants | intergenic_cluster | C/D | | | intergenic |
| AT4G15258 | SNOR37-2 | A_thaliana | Plants | intergenic_cluster | C/D | | | intergenic |
| AT4G25631 | U60.2F | A_thaliana | Plants | mono_intronic | C/D | AT4G25630 | FIB2 | protein_coding |
| AT4G26365 | AT4G26365 | A_thaliana | Plants | intergenic_cluster | Unknown | | | intergenic |
| AT4G39361 | AT4G39361 | A_thaliana | Plants | intergenic_cluster | C/D | | | intergenic |
| AT4G39363 | AT4G39363 | A_thaliana | Plants | intergenic_cluster | C/D | | | intergenic |
| AT4G39364 | AT4G39364 | A_thaliana | Plants | intergenic_cluster | C/D | | | intergenic |
| AT4G39366 | AT4G39366 | A_thaliana | Plants | intergenic_cluster | C/D | | | intergenic |
| AT5G00750 | AT5G00750 | A_thaliana | Plants | intergenic_cluster | H/ACA | | | intergenic |
| AT5G00755 | AT5G00755 | A_thaliana | Plants | intergenic_cluster | Unknown | | | intergenic |
| AT5G00760 | AT5G00760 | A_thaliana | Plants | intronic_cluster | Unknown | AT5G01350 | AT5G01350 | protein_coding |
| AT5G00765 | AT5G00765 | A_thaliana | Plants | intronic_cluster | Unknown | AT5G01350 | AT5G01350 | protein_coding |
| AT5G01135 | AT5G01135 | A_thaliana | Plants | intronic_cluster | Unknown | AT5G06000 | EIF3G2 | protein_coding |
| AT5G01145 | AT5G01145 | A_thaliana | Plants | intronic_cluster | Unknown | AT5G06000 | EIF3G2 | protein_coding |
| AT5G01165 | AT5G01165 | A_thaliana | Plants | mono_exonic | C/D | AT5G06165 | AT5G06165 | non_coding |
| AT5G01195 | AT5G01195 | A_thaliana | Plants | mono_exonic | C/D | AT5G06165 | AT5G06165 | non_coding |
| AT5G01495 | AT5G01495 | A_thaliana | Plants | intronic_cluster | H/ACA | AT5G08180 | AT5G08180 | protein_coding |
| AT5G01505 | AT5G01505 | A_thaliana | Plants | intronic_cluster | H/ACA | AT5G08180 | AT5G08180 | protein_coding |
| AT5G01515 | AT5G01515 | A_thaliana | Plants | intronic_cluster | H/ACA | AT5G08180 | AT5G08180 | protein_coding |
| AT5G01785 | AT5G01785 | A_thaliana | Plants | mono_exonic | Unknown | AT5G01775 | AT5G01775 | non_coding |
| AT5G02295 | AT5G02295 | A_thaliana | Plants | exonic_cluster | C/D | AT5G15022 | AT5G15022 | non_coding |
| AT5G02305 | AT5G02305 | A_thaliana | Plants | exonic_cluster | C/D | AT5G15022 | AT5G15022 | non_coding |
| AT5G02315 | AT5G02315 | A_thaliana | Plants | exonic_cluster | C/D | AT5G15022 | AT5G15022 | non_coding |
| AT5G02325 | AT5G02325 | A_thaliana | Plants | exonic_cluster | C/D | AT5G15022 | AT5G15022 | non_coding |
| AT5G02565 | AT5G02565 | A_thaliana | Plants | intergenic_cluster | Unknown | | | intergenic |
| AT5G02575 | AT5G02575 | A_thaliana | Plants | intergenic_cluster | Unknown | | | intergenic |
| AT5G02655 | AT5G02655 | A_thaliana | Plants | mono_exonic | C/D | AT5G02645 | AT5G02645 | non_coding |
| AT5G02665 | AT5G02665 | A_thaliana | Plants | mono_exonic | C/D | AT5G02645 | AT5G02645 | non_coding |
| AT5G02675 | AT5G02675 | A_thaliana | Plants | mono_intronic | C/D | AT5G02645 | AT5G02645 | non_coding |
| AT5G02915 | AT5G02915 | A_thaliana | Plants | mono_intronic | H/ACA | AT5G18580 | FASS | protein_coding |
| AT5G04425 | AT5G04425 | A_thaliana | Plants | mono_intronic | C/D | AT5G27720 | emb1644 | protein_coding |
| AT5G04545 | AT5G04545 | A_thaliana | Plants | mono_exonic | Unknown | AT5G27860 | AT5G27860 | protein_coding |
| AT5G04945 | AT5G04945 | A_thaliana | Plants | mono_intergenic | Unknown | | | intergenic |

| | | | | | | | | |
|---|---|---|---|---|---|---|---|---|
| AT5G05375 | AT5G05375 | A_thaliana | Plants | intergenic_cluster | C/D | | | intergenic |
| AT5G05385 | AT5G05385 | A_thaliana | Plants | intergenic_cluster | C/D | | | intergenic |
| AT5G06115 | AT5G06115 | A_thaliana | Plants | mono_intronic | H/ACA | AT5G41010 | NRPB12 | protein_coding |
| AT5G06305 | AT5G06305 | A_thaliana | Plants | mono_intergenic | Unknown | | | intergenic |
| AT5G06565 | AT5G06565 | A_thaliana | Plants | intergenic_cluster | C/D | | | intergenic |
| AT5G06825 | AT5G06825 | A_thaliana | Plants | mono_intronic | Unknown | AT5G46430 | AT5G46430 | protein_coding |
| AT5G06835 | AT5G06835 | A_thaliana | Plants | mono_intronic | Unknown | AT5G46510 | AT5G46510 | protein_coding |
| AT5G07095 | AT5G07095 | A_thaliana | Plants | intergenic_cluster | Unknown | | | intergenic |
| AT5G07105 | AT5G07105 | A_thaliana | Plants | intergenic_cluster | Unknown | | | intergenic |
| AT5G07725 | AT5G07725 | A_thaliana | Plants | intergenic_cluster | C/D | | | intergenic |
| AT5G07735 | AT5G07735 | A_thaliana | Plants | intergenic_cluster | C/D | | | intergenic |
| AT5G08495 | AT5G08495 | A_thaliana | Plants | intergenic_cluster | Unknown | | | intergenic |
| AT5G08515 | AT5G08515 | A_thaliana | Plants | intergenic_cluster | C/D | | | intergenic |
| AT5G08525 | AT5G08525 | A_thaliana | Plants | intergenic_cluster | C/D | | | intergenic |
| AT5G08805 | AT5G08805 | A_thaliana | Plants | mono_intronic | H/ACA | AT5G57870 | eIFiso4G1 | protein_coding |
| AT5G08850 | AT5G08850 | A_thaliana | Plants | intergenic_cluster | Unknown | | | intergenic |
| AT5G08860 | AT5G08860 | A_thaliana | Plants | intergenic_cluster | C/D | | | intergenic |
| AT5G08865 | AT5G08865 | A_thaliana | Plants | intergenic_cluster | Unknown | | | intergenic |
| AT5G09000 | AT5G09000 | A_thaliana | Plants | intergenic_cluster | C/D | | | intergenic |
| AT5G09005 | AT5G09005 | A_thaliana | Plants | intergenic_cluster | C/D | | | intergenic |
| AT5G09020 | AT5G09020 | A_thaliana | Plants | mono_intergenic | C/D | | | intergenic |
| AT5G09130 | AT5G09130 | A_thaliana | Plants | mono_exonic | Unknown | AT5G09125 | AT5G09125 | non_coding |
| AT5G09495 | AT5G09495 | A_thaliana | Plants | mono_exonic | C/D | AT5G65860 | AT5G65860 | protein_coding |
| AT5G09515 | AT5G09515 | A_thaliana | Plants | mono_exonic | Unknown | AT5G09505 | AT5G09505 | non_coding |
| AT5G10572 | SNOR77 | A_thaliana | Plants | mono_exonic | H/ACA | AT5G01775 | AT5G01775 | non_coding |
| AT5G13225 | AT5G13225 | A_thaliana | Plants | mono_exonic | C/D | AT5G02055 | AT5G02055 | non_coding |
| AT5G41471 | SNOR108 | A_thaliana | Plants | mono_intergenic | C/D | | | intergenic |
| AT5G44286 | AT5G44286 | A_thaliana | Plants | intergenic_cluster | C/D | | | intergenic |
| AT5G51174 | SNOR30 | A_thaliana | Plants | intergenic_cluster | C/D | | | intergenic |
| AT5G52471 | U60.1F | A_thaliana | Plants | mono_intronic | C/D | AT5G52470 | FIB1 | protein_coding |
| AT5G53902 | U3B | A_thaliana | Plants | mono_intergenic | C/D | | | intergenic |
| AT5G54075 | U3D | A_thaliana | Plants | mono_intergenic | C/D | | | intergenic |
| AT5G58595 | AT5G58595 | A_thaliana | Plants | intergenic_cluster | C/D | | | intergenic |
| AT5G66562 | AT5G66562 | A_thaliana | Plants | intergenic_cluster | C/D | | | intergenic |
| AT5G66564 | AT5G66564 | A_thaliana | Plants | intergenic_cluster | C/D | | | intergenic |
| AT5G66567 | AT5G66567 | A_thaliana | Plants | intergenic_cluster | C/D | | | intergenic |
| ENSRNA049464515 | snoZ159 | O_sativa | Plants | mono_exonic | C/D | Os12g0554300 | | non_coding |
| ENSRNA049464535 | snoZ159 | O_sativa | Plants | mono_intergenic | C/D | | | intergenic |
| ENSRNA049464568 | snoZ194 | O_sativa | Plants | mono_intronic | C/D | Os12g0569700 | OsHsp23.7 | protein_coding |
| ENSRNA049464586 | snoZ194 | O_sativa | Plants | mono_exonic | C/D | Os12g0569700 | OsHsp23.7 | protein_coding |
| ENSRNA049464941 | snoR86 | O_sativa | Plants | mono_intergenic | H/ACA | | | intergenic |
| ENSRNA049464961 | snoR86 | O_sativa | Plants | mono_intergenic | H/ACA | | | intergenic |
| ENSRNA049464972 | snoR86 | O_sativa | Plants | mono_intergenic | H/ACA | | | intergenic |

| | | | | | | | | |
|---|---|---|---|---|---|---|---|---|
| ENSRNA049464985 | snoR86 | O_sativa | Plants | mono_exonic | H/ACA | Os12g0228032 | | protein_coding |
| ENSRNA049465003 | snoZ279_R105_R108 | O_sativa | Plants | mono_intergenic | C/D | | | intergenic |
| ENSRNA049465052 | snoZ248 | O_sativa | Plants | mono_exonic | C/D | Os12g0117500 | | protein_coding |
| ENSRNA049465332 | U54 | O_sativa | Plants | intronic_cluster | C/D | Os05g0256000 | OsWD40-116 | protein_coding |
| ENSRNA049465347 | U54 | O_sativa | Plants | intronic_cluster | C/D | Os05g0256000 | OsWD40-116 | protein_coding |
| ENSRNA049465362 | U54 | O_sativa | Plants | intronic_cluster | C/D | Os05g0256000 | OsWD40-116 | protein_coding |
| ENSRNA049465381 | U54 | O_sativa | Plants | intronic_cluster | C/D | Os05g0256000 | OsWD40-116 | protein_coding |
| ENSRNA049465397 | snoZ159 | O_sativa | Plants | mono_intergenic | C/D | | | intergenic |
| ENSRNA049465412 | snoR60 | O_sativa | Plants | intronic_cluster | C/D | Os05g0176000 | OsMed36_2 | protein_coding |
| ENSRNA049465423 | snoR60 | O_sativa | Plants | intronic_cluster | C/D | Os05g0176000 | OsMed36_2 | protein_coding |
| ENSRNA049465477 | Plant_U3 | O_sativa | Plants | mono_intergenic | C/D | | | intergenic |
| ENSRNA049465677 | snoZ163 | O_sativa | Plants | intergenic_cluster | C/D | | | intergenic |
| ENSRNA049465797 | snoZ256 | O_sativa | Plants | mono_intergenic | C/D | | | intergenic |
| ENSRNA049465857 | snoZ185 | O_sativa | Plants | intergenic_cluster | C/D | | | intergenic |
| ENSRNA049465882 | snoZ188 | O_sativa | Plants | intergenic_cluster | C/D | | | intergenic |
| ENSRNA049465909 | snoZ169 | O_sativa | Plants | mono_intergenic | C/D | | | intergenic |
| ENSRNA049465951 | snoZ169 | O_sativa | Plants | intergenic_cluster | C/D | | | intergenic |
| ENSRNA049465986 | snoR77 | O_sativa | Plants | mono_exonic | H/ACA | Os10g0509700 | | protein_coding |
| ENSRNA049466001 | snoZ175 | O_sativa | Plants | intergenic_cluster | C/D | | | intergenic |
| ENSRNA049466017 | snoZ168 | O_sativa | Plants | intergenic_cluster | C/D | | | intergenic |
| ENSRNA049466028 | snoZ163 | O_sativa | Plants | intergenic_cluster | C/D | | | intergenic |
| ENSRNA049466052 | snoZ163 | O_sativa | Plants | intergenic_cluster | C/D | | | intergenic |
| ENSRNA049466069 | snoZ168 | O_sativa | Plants | intergenic_cluster | C/D | | | intergenic |
| ENSRNA049466088 | snoZ182 | O_sativa | Plants | intergenic_cluster | C/D | | | intergenic |
| ENSRNA049466099 | snoZ168 | O_sativa | Plants | intergenic_cluster | C/D | | | intergenic |
| ENSRNA049466121 | snoZ168 | O_sativa | Plants | mono_intergenic | C/D | | | intergenic |
| ENSRNA049466137 | snoZ163 | O_sativa | Plants | intergenic_cluster | C/D | | | intergenic |
| ENSRNA049466151 | snoZ185 | O_sativa | Plants | intergenic_cluster | C/D | | | intergenic |
| ENSRNA049466168 | snoZ188 | O_sativa | Plants | mono_intronic | C/D | Os10g0389150 | | non_coding |
| ENSRNA049466182 | snoZ165 | O_sativa | Plants | mono_intergenic | C/D | | | intergenic |
| ENSRNA049466194 | snoZ165 | O_sativa | Plants | mono_intergenic | C/D | | | intergenic |
| ENSRNA049466206 | snoZ188 | O_sativa | Plants | intronic_cluster | C/D | Os10g0389150 | | non_coding |
| ENSRNA049466221 | snoZ182 | O_sativa | Plants | mono_intergenic | C/D | | | intergenic |
| ENSRNA049466235 | snoZ188 | O_sativa | Plants | intronic_cluster | C/D | Os10g0389150 | | non_coding |
| ENSRNA049466248 | snoZ185 | O_sativa | Plants | intergenic_cluster | C/D | | | intergenic |
| ENSRNA049466264 | snoZ188 | O_sativa | Plants | intergenic_cluster | C/D | | | intergenic |
| ENSRNA049466282 | snoZ173 | O_sativa | Plants | mono_intergenic | C/D | | | intergenic |
| ENSRNA049466729 | snoR103 | O_sativa | Plants | intronic_cluster | H/ACA | Os04g0117200 | | protein_coding |
| ENSRNA049466778 | snoR103 | O_sativa | Plants | intronic_cluster | H/ACA | Os04g0117200 | | protein_coding |
| ENSRNA049466796 | snoR103 | O_sativa | Plants | intronic_cluster | H/ACA | Os04g0117200 | | protein_coding |
| ENSRNA049466805 | snoZ256 | O_sativa | Plants | mono_intergenic | C/D | | | intergenic |
| ENSRNA049467312 | snoR135 | O_sativa | Plants | intergenic_cluster | H/ACA | | | intergenic |
| ENSRNA049467328 | Plant_U3 | O_sativa | Plants | mono_intergenic | C/D | | | intergenic |

| | | | | | | | | |
|---|---|---|---|---|---|---|---|---|
| ENSRNA049467392 | snoR135 | O_sativa | Plants | intergenic_cluster | H/ACA | | | intergenic |
| ENSRNA049467406 | snoR135 | O_sativa | Plants | intergenic_cluster | H/ACA | | | intergenic |
| ENSRNA049467579 | SNORD24 | O_sativa | Plants | exonic_cluster | C/D | Os07g0168700 | | non_coding |
| ENSRNA049467589 | SNORD24 | O_sativa | Plants | exonic_cluster | C/D | Os07g0168700 | | non_coding |
| ENSRNA049467602 | snoZ105 | O_sativa | Plants | mono_intronic | C/D | Os07g0139600 | | non_coding |
| ENSRNA049467638 | snoR8a | O_sativa | Plants | mono_exonic | C/D | Os07g0124400 | | protein_coding |
| ENSRNA049467656 | snoU36a | O_sativa | Plants | intergenic_cluster | C/D | | | intergenic |
| ENSRNA049467663 | snoU36a | O_sativa | Plants | intergenic_cluster | C/D | | | intergenic |
| ENSRNA049467692 | snoZ223 | O_sativa | Plants | intergenic_cluster | C/D | | | intergenic |
| ENSRNA049467705 | snoZ223 | O_sativa | Plants | intergenic_cluster | C/D | | | intergenic |
| ENSRNA049467714 | snoR134 | O_sativa | Plants | intergenic_cluster | H/ACA | | | intergenic |
| ENSRNA049467734 | snoZ278 | O_sativa | Plants | intergenic_cluster | C/D | | | intergenic |
| ENSRNA049467750 | snoZ278 | O_sativa | Plants | intergenic_cluster | C/D | | | intergenic |
| ENSRNA049467759 | snoJ26 | O_sativa | Plants | mono_intergenic | C/D | | | intergenic |
| ENSRNA049468027 | SNORD36 | O_sativa | Plants | intronic_cluster | C/D | Os06g0134000 | RPS20 | protein_coding |
| ENSRNA049468042 | snoR44_J54 | O_sativa | Plants | intronic_cluster | C/D | Os06g0230801 | | protein_coding |
| ENSRNA049468053 | SNORD36 | O_sativa | Plants | intronic_cluster | C/D | Os06g0134000 | RPS20 | protein_coding |
| ENSRNA049468095 | snoR44_J54 | O_sativa | Plants | intronic_cluster | C/D | Os06g0230801 | | protein_coding |
| ENSRNA049468126 | snoR44_J54 | O_sativa | Plants | intronic_cluster | C/D | Os06g0230801 | | protein_coding |
| ENSRNA049468141 | snoR28 | O_sativa | Plants | mono_exonic | C/D | Os06g0187000 | OsORC1 | protein_coding |
| ENSRNA049468709 | snoR134 | O_sativa | Plants | intergenic_cluster | H/ACA | | | intergenic |
| ENSRNA049468722 | snoZ223 | O_sativa | Plants | intergenic_cluster | C/D | | | intergenic |
| ENSRNA049468908 | snoU31b | O_sativa | Plants | mono_intergenic | C/D | | | intergenic |
| ENSRNA049469003 | snoR44_J54 | O_sativa | Plants | intergenic_cluster | C/D | | | intergenic |
| ENSRNA049469014 | snoR44_J54 | O_sativa | Plants | intergenic_cluster | C/D | | | intergenic |
| ENSRNA049469476 | snoZ256 | O_sativa | Plants | mono_intergenic | C/D | | | intergenic |
| ENSRNA049469734 | snoZ279_R105_R108 | O_sativa | Plants | mono_intergenic | C/D | | | intergenic |
| ENSRNA049469839 | snoZ248 | O_sativa | Plants | mono_exonic | C/D | Os11g0118350 | | protein_coding |
| ENSRNA049470054 | Plant_U3 | O_sativa | Plants | mono_intergenic | C/D | | | intergenic |
| ENSRNA049470113 | snoR117 | O_sativa | Plants | mono_intronic | C/D | Os06g0550000 | | protein_coding |
| ENSRNA049470229 | snoR80 | O_sativa | Plants | mono_exonic | H/ACA | Os06g0682633 | | protein_coding |
| ENSRNA049470255 | snoZ101 | O_sativa | Plants | intergenic_cluster | C/D | | | intergenic |
| ENSRNA049470291 | snoZ102_R77 | O_sativa | Plants | intergenic_cluster | C/D | | | intergenic |
| ENSRNA049470299 | snoU36a | O_sativa | Plants | exonic_cluster | C/D | Os06g0679200 | | non_coding |
| ENSRNA049470317 | snoZ223 | O_sativa | Plants | mono_intergenic | C/D | | | intergenic |
| ENSRNA049470335 | snoZ223 | O_sativa | Plants | exonic_cluster | C/D | Os06g0679200 | | non_coding |
| ENSRNA049470370 | snoR134 | O_sativa | Plants | exonic_cluster | H/ACA | Os06g0679200 | | non_coding |
| ENSRNA049470377 | snoZ278 | O_sativa | Plants | mono_intergenic | C/D | | | intergenic |
| ENSRNA049470386 | snoZ278 | O_sativa | Plants | exonic_cluster | C/D | Os06g0679200 | | non_coding |
| ENSRNA049470406 | U54 | O_sativa | Plants | intronic_cluster | C/D | Os08g0308100 | OsWD40-159 | protein_coding |
| ENSRNA049470425 | U54 | O_sativa | Plants | intronic_cluster | C/D | Os08g0308100 | OsWD40-159 | protein_coding |
| ENSRNA049470508 | snoJ26 | O_sativa | Plants | mono_intergenic | C/D | | | intergenic |
| ENSRNA049470757 | snoR60 | O_sativa | Plants | mono_intronic | C/D | Os02g0821800 | OsMed18_1 | protein_coding |

| | | | | | | | | |
|---|---|---|---|---|---|---|---|---|
| ENSRNA049470784 | snoR60 | O_sativa | Plants | mono_intronic | C/D | Os02g0821800 | OsMed18_1 | protein_coding |
| ENSRNA049470941 | SNORD14 | O_sativa | Plants | exonic_cluster | C/D | Os02g0651500 | | protein_coding |
| ENSRNA049470950 | snoR31_Z110_Z27 | O_sativa | Plants | exonic_cluster | C/D | Os02g0651500 | | protein_coding |
| ENSRNA049470962 | snoR77Y | O_sativa | Plants | exonic_cluster | C/D | Os02g0651500 | | protein_coding |
| ENSRNA049470982 | SNORD25 | O_sativa | Plants | mono_exonic | C/D | Os02g0618000 | | non_coding |
| ENSRNA049470992 | snoZ221_snoR21b | O_sativa | Plants | mono_intergenic | C/D | | | intergenic |
| ENSRNA049471020 | SNORD14 | O_sativa | Plants | exonic_cluster | C/D | Os02g0651500 | | protein_coding |
| ENSRNA049471032 | SNORD14 | O_sativa | Plants | exonic_cluster | C/D | Os02g0651500 | | protein_coding |
| ENSRNA049471225 | snoZ103 | O_sativa | Plants | intronic_cluster | C/D | Os01g0276000 | | protein_coding |
| ENSRNA049471251 | snoZ103 | O_sativa | Plants | intergenic_cluster | C/D | | | intergenic |
| ENSRNA049471263 | snoR83 | O_sativa | Plants | mono_exonic | H/ACA | Os01g0192550 | | protein_coding |
| ENSRNA049471273 | snoZ101 | O_sativa | Plants | intergenic_cluster | C/D | | | intergenic |
| ENSRNA049471283 | snoZ103 | O_sativa | Plants | intronic_cluster | C/D | Os01g0276000 | | protein_coding |
| ENSRNA049471325 | snoZ102_R77 | O_sativa | Plants | intergenic_cluster | C/D | | | intergenic |
| ENSRNA049471342 | snoZ103 | O_sativa | Plants | intronic_cluster | C/D | Os01g0276000 | | protein_coding |
| ENSRNA049471527 | snoR101 | O_sativa | Plants | mono_intronic | C/D | Os01g0698100 | | protein_coding |
| ENSRNA049471555 | snoJ33 | O_sativa | Plants | mono_intergenic | C/D | | | intergenic |
| ENSRNA049471632 | snoZ155 | O_sativa | Plants | exonic_cluster | C/D | Os01g0934901 | | non_coding |
| ENSRNA049471650 | snoR41 | O_sativa | Plants | exonic_cluster | C/D | Os01g0934901 | | non_coding |
| ENSRNA049471802 | snoJ33 | O_sativa | Plants | mono_exonic | C/D | Os01g0754200 | OsGSL3 | protein_coding |
| ENSRNA049471985 | snoZ118 | O_sativa | Plants | intronic_cluster | C/D | Os02g0114600 | OsFbox060 | protein_coding |
| ENSRNA049472003 | snoZ118 | O_sativa | Plants | intronic_cluster | C/D | Os02g0114600 | OsFbox060 | protein_coding |
| ENSRNA049472018 | snoR11 | O_sativa | Plants | mono_intergenic | C/D | | | intergenic |
| ENSRNA049472036 | snoR1 | O_sativa | Plants | exonic_cluster | C/D | Os02g0134100 | | protein_coding |
| ENSRNA049472044 | snoZ118 | O_sativa | Plants | intronic_cluster | C/D | Os02g0114600 | OsFbox060 | protein_coding |
| ENSRNA049472058 | snoR11 | O_sativa | Plants | exonic_cluster | C/D | Os02g0198366 | | protein_coding |
| ENSRNA049472073 | snoZ118 | O_sativa | Plants | intronic_cluster | C/D | Os02g0114600 | OsFbox060 | protein_coding |
| ENSRNA049472089 | snoZ152 | O_sativa | Plants | exonic_cluster | C/D | Os02g0198366 | | protein_coding |
| ENSRNA049472108 | snoR64 | O_sativa | Plants | intronic_cluster | C/D | Os02g0111700 | | protein_coding |
| ENSRNA049472120 | snoZ199 | O_sativa | Plants | intronic_cluster | C/D | Os02g0111700 | | protein_coding |
| ENSRNA049472132 | snoZ199 | O_sativa | Plants | intronic_cluster | C/D | Os02g0111700 | | protein_coding |
| ENSRNA049472154 | snoR1 | O_sativa | Plants | exonic_cluster | C/D | Os02g0134100 | | protein_coding |
| ENSRNA049472167 | snoR1 | O_sativa | Plants | exonic_cluster | C/D | Os02g0134100 | | protein_coding |
| ENSRNA049472178 | snoZ118 | O_sativa | Plants | intronic_cluster | C/D | Os02g0114600 | OsFbox060 | protein_coding |
| ENSRNA049472195 | snoZ118 | O_sativa | Plants | intronic_cluster | C/D | Os02g0114600 | OsFbox060 | protein_coding |
| ENSRNA049472208 | snoZ118 | O_sativa | Plants | intronic_cluster | C/D | Os02g0114600 | OsFbox060 | protein_coding |
| ENSRNA049472220 | snoZ118 | O_sativa | Plants | intronic_cluster | C/D | Os02g0114600 | OsFbox060 | protein_coding |
| ENSRNA049472231 | snoZ118 | O_sativa | Plants | intronic_cluster | C/D | Os02g0114600 | OsFbox060 | protein_coding |
| ENSRNA049472455 | snoR41 | O_sativa | Plants | exonic_cluster | C/D | Os05g0371133 | | non_coding |
| ENSRNA049472500 | snoZ155 | O_sativa | Plants | exonic_cluster | C/D | Os05g0371133 | | non_coding |
| ENSRNA049472532 | snoZ159 | O_sativa | Plants | mono_exonic | C/D | Os05g0316100 | | protein_coding |
| ENSRNA049472690 | snoZ105 | O_sativa | Plants | intronic_cluster | C/D | Os01g0348700 | OsRPL23A | protein_coding |
| ENSRNA049472737 | snoZ122 | O_sativa | Plants | exonic_cluster | C/D | Os01g0332900 | | protein_coding |

| | | | | | | | | |
|---|---|---|---|---|---|---|---|---|
| ENSRNA049472745 | snoZ221_snoR21b | O_sativa | Plants | mono_intergenic | C/D | | | intergenic |
| ENSRNA049472760 | SNOR75 | O_sativa | Plants | intronic_cluster | C/D | Os01g0348700 | OsRPL23A | protein_coding |
| ENSRNA049472768 | snoZ119 | O_sativa | Plants | exonic_cluster | C/D | Os01g0332900 | | protein_coding |
| ENSRNA049472772 | snoZ119 | O_sativa | Plants | exonic_cluster | C/D | Os01g0332900 | | protein_coding |
| ENSRNA049472894 | SNORD14 | O_sativa | Plants | intergenic_cluster | C/D | | | intergenic |
| ENSRNA049472901 | SNORD14 | O_sativa | Plants | intergenic_cluster | C/D | | | intergenic |
| ENSRNA049472907 | SNORD14 | O_sativa | Plants | intergenic_cluster | C/D | | | intergenic |
| ENSRNA049472915 | SNORD18 | O_sativa | Plants | intronic_cluster | C/D | Os03g0781501 | | non_coding |
| ENSRNA049472923 | snoZ105 | O_sativa | Plants | intronic_cluster | C/D | Os03g0781501 | | non_coding |
| ENSRNA049472947 | SNORD24 | O_sativa | Plants | intronic_cluster | C/D | Os03g0755700 | | protein_coding |
| ENSRNA049472954 | SNORD24 | O_sativa | Plants | mono_intronic | C/D | Os03g0756000 | | protein_coding |
| ENSRNA049472976 | snoU31b | O_sativa | Plants | intergenic_cluster | C/D | | | intergenic |
| ENSRNA049472987 | snoR31_Z110_Z27 | O_sativa | Plants | intergenic_cluster | C/D | | | intergenic |
| ENSRNA049472998 | snoR31_Z110_Z27 | O_sativa | Plants | intergenic_cluster | C/D | | | intergenic |
| ENSRNA049473016 | snoR77Y | O_sativa | Plants | intronic_cluster | C/D | Os03g0755700 | | protein_coding |
| ENSRNA049473030 | snoR77Y | O_sativa | Plants | exonic_cluster | C/D | Os03g0755625 | | protein_coding |
| ENSRNA049473038 | snoR31_Z110_Z27 | O_sativa | Plants | intronic_cluster | C/D | Os03g0755700 | | protein_coding |
| ENSRNA049473047 | snoR31_Z110_Z27 | O_sativa | Plants | intergenic_cluster | C/D | | | intergenic |
| ENSRNA049473057 | snoR31_Z110_Z27 | O_sativa | Plants | exonic_cluster | C/D | Os03g0755625 | | protein_coding |
| ENSRNA049473071 | snoR77Y | O_sativa | Plants | intergenic_cluster | C/D | | | intergenic |
| ENSRNA049473086 | snoR77Y | O_sativa | Plants | intergenic_cluster | C/D | | | intergenic |
| ENSRNA049473108 | snoR77Y | O_sativa | Plants | intergenic_cluster | C/D | | | intergenic |
| ENSRNA049473120 | snoU31b | O_sativa | Plants | intergenic_cluster | C/D | | | intergenic |
| ENSRNA049473128 | SNORD14 | O_sativa | Plants | mono_intergenic | C/D | | | intergenic |
| ENSRNA049473150 | SNORD14 | O_sativa | Plants | intronic_cluster | C/D | Os03g0755700 | | protein_coding |
| ENSRNA049473164 | SNORD14 | O_sativa | Plants | intergenic_cluster | C/D | | | intergenic |
| ENSRNA049473172 | SNORD14 | O_sativa | Plants | intergenic_cluster | C/D | | | intergenic |
| ENSRNA049473180 | SNORD14 | O_sativa | Plants | intergenic_cluster | C/D | | | intergenic |
| ENSRNA049473196 | SNOR75 | O_sativa | Plants | intronic_cluster | C/D | Os03g0781501 | | non_coding |
| ENSRNA049473206 | SNOR75 | O_sativa | Plants | intronic_cluster | C/D | Os03g0781501 | | non_coding |
| ENSRNA049473214 | SNORD14 | O_sativa | Plants | intergenic_cluster | C/D | | | intergenic |
| ENSRNA049473223 | SNORD14 | O_sativa | Plants | intronic_cluster | C/D | Os03g0755700 | | protein_coding |
| ENSRNA049473229 | SNORD14 | O_sativa | Plants | intergenic_cluster | C/D | | | intergenic |
| ENSRNA049473235 | SNORD14 | O_sativa | Plants | intergenic_cluster | C/D | | | intergenic |
| ENSRNA049473325 | snoZ278 | O_sativa | Plants | mono_intronic | C/D | Os04g0635800 | | protein_coding |
| ENSRNA049473437 | Plant_U3 | O_sativa | Plants | mono_intergenic | C/D | | | intergenic |
| ENSRNA049473446 | Plant_U3 | O_sativa | Plants | mono_intergenic | C/D | | | intergenic |
| ENSRNA049473455 | Plant_U3 | O_sativa | Plants | mono_intergenic | C/D | | | intergenic |
| ENSRNA049473568 | snoZ199 | O_sativa | Plants | intergenic_cluster | C/D | | | intergenic |
| ENSRNA049473582 | snoR64 | O_sativa | Plants | intergenic_cluster | C/D | | | intergenic |
| ENSRNA049473596 | SNORD25 | O_sativa | Plants | intronic_cluster | C/D | Os04g0508100 | | protein_coding |
| ENSRNA049473610 | SNORD25 | O_sativa | Plants | intronic_cluster | C/D | Os04g0508100 | | protein_coding |
| ENSRNA049473620 | SNORD25 | O_sativa | Plants | intronic_cluster | C/D | Os04g0508100 | | protein_coding |

| | | | | | | | | |
|---|---|---|---|---|---|---|---|---|
| ENSRNA049473627 | SNORD25 | O_sativa | Plants | intronic_cluster | C/D | Os04g0508100 | | protein_coding |
| ENSRNA049473641 | SNORD14 | O_sativa | Plants | intergenic_cluster | C/D | | | intergenic |
| ENSRNA049473653 | SNORD14 | O_sativa | Plants | intergenic_cluster | C/D | | | intergenic |
| ENSRNA049473661 | SNORD14 | O_sativa | Plants | intergenic_cluster | C/D | | | intergenic |
| ENSRNA049473842 | snoZ266 | O_sativa | Plants | intronic_cluster | C/D | Os03g0234200 | ZIURP1 | protein_coding |
| ENSRNA049473866 | snoZ266 | O_sativa | Plants | intronic_cluster | C/D | Os03g0234200 | ZIURP1 | protein_coding |
| ENSRNA049473876 | snoZ103 | O_sativa | Plants | intergenic_cluster | C/D | | | intergenic |
| ENSRNA049473879 | snoZ103 | O_sativa | Plants | intergenic_cluster | C/D | | | intergenic |
| ENSRNA049473887 | snoZ103 | O_sativa | Plants | intergenic_cluster | C/D | | | intergenic |
| ENSRNA049473893 | snoZ103 | O_sativa | Plants | intergenic_cluster | C/D | | | intergenic |
| ENSRNA049473899 | snoZ223 | O_sativa | Plants | mono_intergenic | C/D | | | intergenic |
| ENSRNA049473908 | snoR134 | O_sativa | Plants | mono_exonic | H/ACA | Os03g0222401 | | non_coding |
| ENSRNA049474111 | snoJ26 | O_sativa | Plants | mono_intergenic | C/D | | | intergenic |
| ENSRNA049474136 | snoJ26 | O_sativa | Plants | mono_intergenic | C/D | | | intergenic |
| ENSRNA049475575 | snoZ43 | O_sativa | Plants | intergenic_cluster | C/D | | | intergenic |
| ENSRNA049475602 | snosnR60_Z15 | O_sativa | Plants | intergenic_cluster | C/D | | | intergenic |
| ENSRNA049475715 | U54 | O_sativa | Plants | intergenic_cluster | C/D | | | intergenic |
| ENSRNA049475720 | U54 | O_sativa | Plants | intergenic_cluster | C/D | | | intergenic |
| ENSRNA049475726 | U54 | O_sativa | Plants | mono_exonic | C/D | Os11g0606300 | | protein_coding |
| ENSRNA049475731 | Plant_U3 | O_sativa | Plants | mono_intergenic | C/D | | | intergenic |
| ENSRNA049475930 | snoZ159 | O_sativa | Plants | mono_intergenic | C/D | | | intergenic |
| ENSRNA049475950 | snoR135 | O_sativa | Plants | mono_intergenic | H/ACA | | | intergenic |
| ENSRNA049475955 | snoR80 | O_sativa | Plants | mono_exonic | H/ACA | Os03g0408401 | | non_coding |
| ENSRNA049476083 | snoR44_J54 | O_sativa | Plants | mono_exonic | C/D | Os09g0411975 | | non_coding |
| ENSRNA049476183 | snoZ242 | O_sativa | Plants | mono_intergenic | C/D | | | intergenic |
| ENSRNA049476188 | snoZ242 | O_sativa | Plants | mono_intergenic | C/D | | | intergenic |
| ENSRNA049476201 | Plant_U3 | O_sativa | Plants | mono_intergenic | C/D | | | intergenic |
| ENSRNA049476209 | Plant_U3 | O_sativa | Plants | mono_intergenic | C/D | | | intergenic |
| ENSRNA049476289 | snoR134 | O_sativa | Plants | mono_intronic | H/ACA | Os08g0154700 | | protein_coding |
| ENSRNA049476293 | snoZ242 | O_sativa | Plants | mono_intergenic | C/D | | | intergenic |
| ENSRNA049476307 | snoZ199 | O_sativa | Plants | intronic_cluster | C/D | Os08g0128500 | | protein_coding |
| ENSRNA049476316 | snoR64 | O_sativa | Plants | intronic_cluster | C/D | Os08g0128500 | | protein_coding |
| ENSRNA049476319 | snoZ199 | O_sativa | Plants | intronic_cluster | C/D | Os08g0128500 | | protein_coding |
| ENSRNA049476335 | snosnR60_Z15 | O_sativa | Plants | mono_intronic | C/D | Os08g0156800 | | protein_coding |
| ENSRNA049476388 | snoJ26 | O_sativa | Plants | mono_intergenic | C/D | | | intergenic |
| ENSRNA049476404 | snoZ266 | O_sativa | Plants | intergenic_cluster | C/D | | | intergenic |
| ENSRNA049476411 | snoZ266 | O_sativa | Plants | intergenic_cluster | C/D | | | intergenic |
| ENSRNA050013733 | snoR60 | T_aestivum | Plants | mono_intergenic | C/D | | | intergenic |
| ENSRNA050013737 | snoU36a | T_aestivum | Plants | mono_intergenic | C/D | | | intergenic |
| ENSRNA050013739 | snoR11 | T_aestivum | Plants | intergenic_cluster | C/D | | | intergenic |
| ENSRNA050013740 | snoZ152 | T_aestivum | Plants | intergenic_cluster | C/D | | | intergenic |
| ENSRNA050013746 | snoZ279_R105_R108 | T_aestivum | Plants | mono_intergenic | C/D | | | intergenic |
| ENSRNA050013749 | snoR64 | T_aestivum | Plants | intergenic_cluster | C/D | | | intergenic |

| | | | | | | |
|---|---|---|---|---|---|---|
| ENSRNA050013750 | snoZ199 | T_aestivum | Plants | intergenic_cluster | C/D | intergenic |
| ENSRNA050013751 | snoZ199 | T_aestivum | Plants | intergenic_cluster | C/D | intergenic |
| ENSRNA050013752 | snoZ43 | T_aestivum | Plants | intergenic_cluster | C/D | intergenic |
| ENSRNA050013753 | snoU36a | T_aestivum | Plants | intergenic_cluster | C/D | intergenic |
| ENSRNA050013754 | snoZ223 | T_aestivum | Plants | intergenic_cluster | C/D | intergenic |
| ENSRNA050013755 | snoZ223 | T_aestivum | Plants | intergenic_cluster | C/D | intergenic |
| ENSRNA050013756 | SNORD25 | T_aestivum | Plants | intergenic_cluster | C/D | intergenic |
| ENSRNA050013757 | SNORD25 | T_aestivum | Plants | mono_intergenic | C/D | intergenic |
| ENSRNA050013758 | snoR64 | T_aestivum | Plants | intergenic_cluster | C/D | intergenic |
| ENSRNA050013759 | SNORD25 | T_aestivum | Plants | mono_intergenic | C/D | intergenic |
| ENSRNA050013760 | snoR64 | T_aestivum | Plants | intergenic_cluster | C/D | intergenic |
| ENSRNA050013761 | snoU36a | T_aestivum | Plants | mono_intergenic | C/D | intergenic |
| ENSRNA050013762 | snoZ278 | T_aestivum | Plants | intergenic_cluster | C/D | intergenic |
| ENSRNA050013763 | snoZ101 | T_aestivum | Plants | intergenic_cluster | C/D | intergenic |
| ENSRNA050013764 | snoZ278 | T_aestivum | Plants | intergenic_cluster | C/D | intergenic |
| ENSRNA050013765 | snoU31b | T_aestivum | Plants | intergenic_cluster | C/D | intergenic |
| ENSRNA050013766 | snoZ199 | T_aestivum | Plants | intergenic_cluster | C/D | intergenic |
| ENSRNA050013767 | snosnR60_Z15 | T_aestivum | Plants | intergenic_cluster | C/D | intergenic |
| ENSRNA050013768 | snoZ278 | T_aestivum | Plants | intergenic_cluster | C/D | intergenic |
| ENSRNA050013769 | snoZ199 | T_aestivum | Plants | intergenic_cluster | C/D | intergenic |
| ENSRNA050013770 | snoR8a | T_aestivum | Plants | intergenic_cluster | C/D | intergenic |
| ENSRNA050013771 | snoZ102_R77 | T_aestivum | Plants | intergenic_cluster | C/D | intergenic |
| ENSRNA050013772 | snoZ101 | T_aestivum | Plants | intergenic_cluster | C/D | intergenic |
| ENSRNA050013773 | snoZ102_R77 | T_aestivum | Plants | intergenic_cluster | C/D | intergenic |
| ENSRNA050013775 | snoR77 | T_aestivum | Plants | intergenic_cluster | H/ACA | intergenic |
| ENSRNA050013776 | snoR77 | T_aestivum | Plants | intergenic_cluster | H/ACA | intergenic |
| ENSRNA050013777 | snoR77 | T_aestivum | Plants | intergenic_cluster | H/ACA | intergenic |
| ENSRNA050013778 | snoR77 | T_aestivum | Plants | intergenic_cluster | H/ACA | intergenic |
| ENSRNA050013779 | snoR44_J54 | T_aestivum | Plants | intergenic_cluster | C/D | intergenic |
| ENSRNA050013780 | snoR44_J54 | T_aestivum | Plants | intergenic_cluster | C/D | intergenic |
| ENSRNA050013781 | snoR77 | T_aestivum | Plants | mono_intergenic | H/ACA | intergenic |
| ENSRNA050013782 | snoR77 | T_aestivum | Plants | mono_intergenic | H/ACA | intergenic |
| ENSRNA050013784 | snoZ159 | T_aestivum | Plants | mono_intergenic | C/D | intergenic |
| ENSRNA050013785 | snoZ159 | T_aestivum | Plants | mono_intergenic | C/D | intergenic |
| ENSRNA050013786 | snoZ199 | T_aestivum | Plants | intergenic_cluster | C/D | intergenic |
| ENSRNA050013787 | snoR64 | T_aestivum | Plants | intergenic_cluster | C/D | intergenic |
| ENSRNA050013788 | snoZ199 | T_aestivum | Plants | intergenic_cluster | C/D | intergenic |
| ENSRNA050013790 | snoR64 | T_aestivum | Plants | intergenic_cluster | C/D | intergenic |
| ENSRNA050013791 | snoZ199 | T_aestivum | Plants | intergenic_cluster | C/D | intergenic |
| ENSRNA050013792 | snoZ199 | T_aestivum | Plants | intergenic_cluster | C/D | intergenic |
| ENSRNA050013795 | snoU31b | T_aestivum | Plants | intergenic_cluster | C/D | intergenic |
| ENSRNA050013796 | snoR117 | T_aestivum | Plants | intergenic_cluster | C/D | intergenic |
| ENSRNA050013797 | snoR44_J54 | T_aestivum | Plants | mono_intergenic | C/D | intergenic |

| | | | | | | | |
|---|---|---|---|---|---|---|---|
| ENSRNA050013799 | snoR101 | T_aestivum | Plants | mono_intergenic | C/D | | intergenic |
| ENSRNA050013805 | snoR80 | T_aestivum | Plants | mono_intergenic | H/ACA | | intergenic |
| ENSRNA050013821 | U54 | T_aestivum | Plants | intronic_cluster | C/D | TraesCS4D02G149800 | protein_coding |
| ENSRNA050013822 | U54 | T_aestivum | Plants | intronic_cluster | C/D | TraesCS4D02G149800 | protein_coding |
| ENSRNA050013823 | U54 | T_aestivum | Plants | intronic_cluster | C/D | TraesCS4D02G149800 | protein_coding |
| ENSRNA050013824 | U54 | T_aestivum | Plants | intronic_cluster | C/D | TraesCS4D02G149800 | protein_coding |
| ENSRNA050013944 | snoR80 | T_aestivum | Plants | mono_intergenic | H/ACA | | intergenic |
| ENSRNA050013955 | snoZ223 | T_aestivum | Plants | mono_intergenic | C/D | | intergenic |
| ENSRNA050014025 | snoU36a | T_aestivum | Plants | intergenic_cluster | C/D | | intergenic |
| ENSRNA050014026 | snoZ223 | T_aestivum | Plants | intergenic_cluster | C/D | | intergenic |
| ENSRNA050014040 | snoR77 | T_aestivum | Plants | mono_intergenic | H/ACA | | intergenic |
| ENSRNA050014048 | snoR86 | T_aestivum | Plants | intronic_cluster | H/ACA | TraesCS4D02G006900 | protein_coding |
| ENSRNA050014049 | snoR86 | T_aestivum | Plants | intronic_cluster | H/ACA | TraesCS4D02G006900 | protein_coding |
| ENSRNA050014050 | snoR86 | T_aestivum | Plants | intronic_cluster | H/ACA | TraesCS4D02G006900 | protein_coding |
| ENSRNA050014051 | snoR86 | T_aestivum | Plants | intronic_cluster | H/ACA | TraesCS4D02G006900 | protein_coding |
| ENSRNA050014052 | snoZ266 | T_aestivum | Plants | intronic_cluster | C/D | TraesCS4D02G006900 | protein_coding |
| ENSRNA050014053 | snoR86 | T_aestivum | Plants | intronic_cluster | H/ACA | TraesCS4D02G006900 | protein_coding |
| ENSRNA050014054 | snoR86 | T_aestivum | Plants | intronic_cluster | H/ACA | TraesCS4D02G006900 | protein_coding |
| ENSRNA050014055 | snoR80 | T_aestivum | Plants | mono_intergenic | H/ACA | | intergenic |
| ENSRNA050014056 | snoR80 | T_aestivum | Plants | mono_intergenic | H/ACA | | intergenic |
| ENSRNA050014057 | SNORD24 | T_aestivum | Plants | mono_intergenic | C/D | | intergenic |
| ENSRNA050014058 | snoU36a | T_aestivum | Plants | intergenic_cluster | C/D | | intergenic |
| ENSRNA050014059 | snoZ223 | T_aestivum | Plants | intergenic_cluster | C/D | | intergenic |
| ENSRNA050014060 | snoR134 | T_aestivum | Plants | intergenic_cluster | H/ACA | | intergenic |
| ENSRNA050014061 | SNORD24 | T_aestivum | Plants | mono_intergenic | C/D | | intergenic |
| ENSRNA050014064 | snoZ279_R105_R108 | T_aestivum | Plants | mono_intergenic | C/D | | intergenic |
| ENSRNA050014065 | snoR80 | T_aestivum | Plants | mono_intergenic | H/ACA | | intergenic |
| ENSRNA050014379 | snoR101 | T_aestivum | Plants | mono_intergenic | C/D | | intergenic |
| ENSRNA050014385 | snoR103 | T_aestivum | Plants | intronic_cluster | H/ACA | TraesCS2D02G008300 | protein_coding |
| ENSRNA050014386 | snoR103 | T_aestivum | Plants | intronic_cluster | H/ACA | TraesCS2D02G008300 | protein_coding |
| ENSRNA050014387 | snoR103 | T_aestivum | Plants | intronic_cluster | H/ACA | TraesCS2D02G008300 | protein_coding |
| ENSRNA050014388 | snoR103 | T_aestivum | Plants | intronic_cluster | H/ACA | TraesCS2D02G008300 | protein_coding |
| ENSRNA050014389 | snoR103 | T_aestivum | Plants | intronic_cluster | H/ACA | TraesCS2D02G008300 | protein_coding |
| ENSRNA050014498 | snoZ118 | T_aestivum | Plants | mono_intergenic | C/D | | intergenic |
| ENSRNA050014501 | snoR28 | T_aestivum | Plants | mono_exonic | C/D | TraesCS7A02G151700 | protein_coding |
| ENSRNA050014924 | snoU36a | T_aestivum | Plants | intergenic_cluster | C/D | | intergenic |
| ENSRNA050014925 | snoZ223 | T_aestivum | Plants | intergenic_cluster | C/D | | intergenic |
| ENSRNA050015054 | snoR41 | T_aestivum | Plants | intergenic_cluster | C/D | | intergenic |
| ENSRNA050015056 | snoZ155 | T_aestivum | Plants | intergenic_cluster | C/D | | intergenic |
| ENSRNA050015090 | snoZ118 | T_aestivum | Plants | mono_intergenic | C/D | | intergenic |
| ENSRNA050015095 | snoR135 | T_aestivum | Plants | intergenic_cluster | H/ACA | | intergenic |
| ENSRNA050015096 | snoR135 | T_aestivum | Plants | intergenic_cluster | H/ACA | | intergenic |
| ENSRNA050015097 | snoZ43 | T_aestivum | Plants | intergenic_cluster | C/D | | intergenic |

| | | | | | | | |
|---|---|---|---|---|---|---|---|
| ENSRNA050015098 | snoR135 | T_aestivum | Plants | intergenic_cluster | H/ACA | | intergenic |
| ENSRNA050015100 | snosnR60_Z15 | T_aestivum | Plants | intergenic_cluster | C/D | | intergenic |
| ENSRNA050015104 | SNORD24 | T_aestivum | Plants | mono_intergenic | C/D | | intergenic |
| ENSRNA050015106 | snoZ279_R105_R108 | T_aestivum | Plants | mono_intergenic | C/D | | intergenic |
| ENSRNA050015175 | snoZ223 | T_aestivum | Plants | intergenic_cluster | C/D | | intergenic |
| ENSRNA050015177 | snoZ223 | T_aestivum | Plants | intergenic_cluster | C/D | | intergenic |
| ENSRNA050015178 | snoZ223 | T_aestivum | Plants | intergenic_cluster | C/D | | intergenic |
| ENSRNA050015180 | snoR134 | T_aestivum | Plants | intergenic_cluster | H/ACA | | intergenic |
| ENSRNA050015182 | snoR134 | T_aestivum | Plants | intergenic_cluster | H/ACA | | intergenic |
| ENSRNA050015184 | snoR134 | T_aestivum | Plants | intergenic_cluster | H/ACA | | intergenic |
| ENSRNA050015188 | snoZ223 | T_aestivum | Plants | mono_intergenic | C/D | | intergenic |
| ENSRNA050015197 | SNORD25 | T_aestivum | Plants | intronic_cluster | C/D | TraesCS2D02G360500 | protein_coding |
| ENSRNA050015198 | SNORD25 | T_aestivum | Plants | intronic_cluster | C/D | TraesCS2D02G360500 | protein_coding |
| ENSRNA050015199 | SNORD25 | T_aestivum | Plants | intronic_cluster | C/D | TraesCS2D02G360800 | protein_coding |
| ENSRNA050015200 | SNORD25 | T_aestivum | Plants | intronic_cluster | C/D | TraesCS2D02G360800 | protein_coding |
| ENSRNA050015202 | SNORD25 | T_aestivum | Plants | intronic_cluster | C/D | TraesCS2D02G360800 | protein_coding |
| ENSRNA050015203 | snoU31b | T_aestivum | Plants | intronic_cluster | C/D | TraesCS2D02G360500 | protein_coding |
| ENSRNA050015204 | snoU31b | T_aestivum | Plants | intronic_cluster | C/D | TraesCS2D02G360500 | protein_coding |
| ENSRNA050015205 | snoU31b | T_aestivum | Plants | intronic_cluster | C/D | TraesCS2D02G360800 | protein_coding |
| ENSRNA050015208 | snoU31b | T_aestivum | Plants | intronic_cluster | C/D | TraesCS2D02G360800 | protein_coding |
| ENSRNA050015209 | snosnR60_Z15 | T_aestivum | Plants | intronic_cluster | C/D | TraesCS2D02G360800 | protein_coding |
| ENSRNA050015210 | snosnR60_Z15 | T_aestivum | Plants | intronic_cluster | C/D | TraesCS2D02G360500 | protein_coding |
| ENSRNA050015586 | snoZ223 | T_aestivum | Plants | mono_intergenic | C/D | | intergenic |
| ENSRNA050015588 | snoZ223 | T_aestivum | Plants | intergenic_cluster | C/D | | intergenic |
| ENSRNA050015589 | snoZ223 | T_aestivum | Plants | mono_intergenic | C/D | | intergenic |
| ENSRNA050015592 | snoR134 | T_aestivum | Plants | intergenic_cluster | H/ACA | | intergenic |
| ENSRNA050015598 | SNOR75 | T_aestivum | Plants | mono_intergenic | C/D | | intergenic |
| ENSRNA050015599 | snoZ188 | T_aestivum | Plants | mono_intergenic | C/D | | intergenic |
| ENSRNA050015600 | snoZ118 | T_aestivum | Plants | mono_intergenic | C/D | | intergenic |
| ENSRNA050015602 | SNORD24 | T_aestivum | Plants | mono_intergenic | C/D | | intergenic |
| ENSRNA050015633 | snoZ188 | T_aestivum | Plants | intergenic_cluster | C/D | | intergenic |
| ENSRNA050015634 | snoZ188 | T_aestivum | Plants | intergenic_cluster | C/D | | intergenic |
| ENSRNA050015636 | snoZ188 | T_aestivum | Plants | mono_intergenic | C/D | | intergenic |
| ENSRNA050015645 | snoR135 | T_aestivum | Plants | mono_intergenic | H/ACA | | intergenic |
| ENSRNA050015646 | snoR64 | T_aestivum | Plants | intergenic_cluster | C/D | | intergenic |
| ENSRNA050015647 | snoZ199 | T_aestivum | Plants | intergenic_cluster | C/D | | intergenic |
| ENSRNA050015654 | snoZ43 | T_aestivum | Plants | intergenic_cluster | C/D | | intergenic |
| ENSRNA050015655 | snoZ43 | T_aestivum | Plants | intergenic_cluster | C/D | | intergenic |
| ENSRNA050015656 | snoZ279_R105_R108 | T_aestivum | Plants | mono_intergenic | C/D | | intergenic |
| ENSRNA050015658 | snoR77Y | T_aestivum | Plants | intergenic_cluster | C/D | | intergenic |
| ENSRNA050015659 | snoZ43 | T_aestivum | Plants | mono_intergenic | C/D | | intergenic |
| ENSRNA050015660 | snoU31b | T_aestivum | Plants | intergenic_cluster | C/D | | intergenic |
| ENSRNA050015661 | snosnR60_Z15 | T_aestivum | Plants | intergenic_cluster | C/D | | intergenic |

| | | | | | | | |
|---|---|---|---|---|---|---|---|
| ENSRNA050015662 | snoR8a | T_aestivum | Plants | mono_intergenic | C/D | | intergenic |
| ENSRNA050015664 | snoR31_Z110_Z27 | T_aestivum | Plants | intergenic_cluster | C/D | | intergenic |
| ENSRNA050015665 | snoR77Y | T_aestivum | Plants | intergenic_cluster | C/D | | intergenic |
| ENSRNA050015666 | snoU31b | T_aestivum | Plants | mono_intergenic | C/D | | intergenic |
| ENSRNA050015667 | snoR77Y | T_aestivum | Plants | intergenic_cluster | C/D | | intergenic |
| ENSRNA050015668 | snoU31b | T_aestivum | Plants | mono_intergenic | C/D | | intergenic |
| ENSRNA050015669 | snoR31_Z110_Z27 | T_aestivum | Plants | intergenic_cluster | C/D | | intergenic |
| ENSRNA050015670 | snoR31_Z110_Z27 | T_aestivum | Plants | intergenic_cluster | C/D | | intergenic |
| ENSRNA050015917 | SNOR75 | T_aestivum | Plants | intronic_cluster | C/D | TraesCS7A02G469100 | protein_coding |
| ENSRNA050015919 | SNOR75 | T_aestivum | Plants | intronic_cluster | C/D | TraesCS7A02G469100 | protein_coding |
| ENSRNA050015959 | snoR77Y | T_aestivum | Plants | mono_intergenic | C/D | | intergenic |
| ENSRNA050015969 | snoZ279_R105_R108 | T_aestivum | Plants | mono_intergenic | C/D | | intergenic |
| ENSRNA050016258 | snoR83 | T_aestivum | Plants | intergenic_cluster | H/ACA | | intergenic |
| ENSRNA050016260 | snoU36a | T_aestivum | Plants | intergenic_cluster | C/D | | intergenic |
| ENSRNA050016264 | snoZ223 | T_aestivum | Plants | intergenic_cluster | C/D | | intergenic |
| ENSRNA050016266 | snoR77Y | T_aestivum | Plants | intergenic_cluster | C/D | | intergenic |
| ENSRNA050016282 | snoZ278 | T_aestivum | Plants | intergenic_cluster | C/D | | intergenic |
| ENSRNA050016290 | snoR8a | T_aestivum | Plants | intergenic_cluster | C/D | | intergenic |
| ENSRNA050016292 | snoR31_Z110_Z27 | T_aestivum | Plants | intergenic_cluster | C/D | | intergenic |
| ENSRNA050016302 | snoZ43 | T_aestivum | Plants | mono_intergenic | C/D | | intergenic |
| ENSRNA050016303 | snoU31b | T_aestivum | Plants | mono_intergenic | C/D | | intergenic |
| ENSRNA050016668 | snoZ223 | T_aestivum | Plants | intergenic_cluster | C/D | | intergenic |
| ENSRNA050016670 | snoR134 | T_aestivum | Plants | intergenic_cluster | H/ACA | | intergenic |
| ENSRNA050016671 | SNORD24 | T_aestivum | Plants | mono_intergenic | C/D | | intergenic |
| ENSRNA050016672 | SNORD24 | T_aestivum | Plants | mono_intergenic | C/D | | intergenic |
| ENSRNA050016674 | snoR77Y | T_aestivum | Plants | mono_intergenic | C/D | | intergenic |
| ENSRNA050016675 | snoU31b | T_aestivum | Plants | mono_intergenic | C/D | | intergenic |
| ENSRNA050016676 | SNOR75 | T_aestivum | Plants | intronic_cluster | C/D | TraesCS7B02G370800 | protein_coding |
| ENSRNA050016677 | SNOR75 | T_aestivum | Plants | intronic_cluster | C/D | TraesCS7B02G370800 | protein_coding |
| ENSRNA050016682 | snoZ223 | T_aestivum | Plants | mono_intergenic | C/D | | intergenic |
| ENSRNA050016739 | snoZ43 | T_aestivum | Plants | mono_intergenic | C/D | | intergenic |
| ENSRNA050016741 | snoU31b | T_aestivum | Plants | mono_intergenic | C/D | | intergenic |
| ENSRNA050016780 | snoZ223 | T_aestivum | Plants | mono_intergenic | C/D | | intergenic |
| ENSRNA050016781 | snoZ223 | T_aestivum | Plants | mono_intergenic | C/D | | intergenic |
| ENSRNA050017153 | snoR44_J54 | T_aestivum | Plants | intergenic_cluster | C/D | | intergenic |
| ENSRNA050017154 | snoR44_J54 | T_aestivum | Plants | intergenic_cluster | C/D | | intergenic |
| ENSRNA050017163 | snoR44_J54 | T_aestivum | Plants | intergenic_cluster | C/D | | intergenic |
| ENSRNA050017164 | snoR44_J54 | T_aestivum | Plants | intergenic_cluster | C/D | | intergenic |
| ENSRNA050017165 | snoR44_J54 | T_aestivum | Plants | intergenic_cluster | C/D | | intergenic |
| ENSRNA050017166 | snoR44_J54 | T_aestivum | Plants | intergenic_cluster | C/D | | intergenic |
| ENSRNA050017167 | snoR44_J54 | T_aestivum | Plants | intergenic_cluster | C/D | | intergenic |
| ENSRNA050017174 | snoR11 | T_aestivum | Plants | intronic_cluster | C/D | TraesCS1D02G219000 | protein_coding |
| ENSRNA050017175 | snoZ152 | T_aestivum | Plants | intronic_cluster | C/D | TraesCS1D02G219000 | protein_coding |

| | | | | | | |
|---|---|---|---|---|---|---|
| ENSRNA050017247 | snoU36a | T_aestivum | Plants | intergenic_cluster | C/D | intergenic |
| ENSRNA050017252 | snoZ223 | T_aestivum | Plants | intergenic_cluster | C/D | intergenic |
| ENSRNA050017253 | snoZ159 | T_aestivum | Plants | intergenic_cluster | C/D | intergenic |
| ENSRNA050017254 | snoR44_J54 | T_aestivum | Plants | intergenic_cluster | C/D | intergenic |
| ENSRNA050017255 | snoR44_J54 | T_aestivum | Plants | intergenic_cluster | C/D | intergenic |
| ENSRNA050017258 | SNOR75 | T_aestivum | Plants | intergenic_cluster | C/D | intergenic |
| ENSRNA050017261 | SNOR75 | T_aestivum | Plants | intergenic_cluster | C/D | intergenic |
| ENSRNA050017262 | snoZ105 | T_aestivum | Plants | intergenic_cluster | C/D | intergenic |
| ENSRNA050017306 | snoZ159 | T_aestivum | Plants | intergenic_cluster | C/D | intergenic |
| ENSRNA050017307 | snoZ159 | T_aestivum | Plants | intergenic_cluster | C/D | intergenic |
| ENSRNA050017574 | snoZ118 | T_aestivum | Plants | intergenic_cluster | C/D | intergenic |
| ENSRNA050017575 | snoR60 | T_aestivum | Plants | mono_intergenic | C/D | intergenic |
| ENSRNA050017577 | snoZ118 | T_aestivum | Plants | intergenic_cluster | C/D | intergenic |
| ENSRNA050017580 | snoZ118 | T_aestivum | Plants | intergenic_cluster | C/D | intergenic |
| ENSRNA050017582 | snoZ118 | T_aestivum | Plants | intergenic_cluster | C/D | intergenic |
| ENSRNA050017583 | snoR101 | T_aestivum | Plants | mono_intergenic | C/D | intergenic |
| ENSRNA050017584 | snoZ223 | T_aestivum | Plants | mono_intergenic | C/D | intergenic |
| ENSRNA050017593 | snoR64 | T_aestivum | Plants | intergenic_cluster | C/D | intergenic |
| ENSRNA050017594 | snoZ199 | T_aestivum | Plants | intergenic_cluster | C/D | intergenic |
| ENSRNA050017596 | snoZ199 | T_aestivum | Plants | intergenic_cluster | C/D | intergenic |
| ENSRNA050017600 | snoR80 | T_aestivum | Plants | mono_intergenic | H/ACA | intergenic |
| ENSRNA050017602 | snoR77 | T_aestivum | Plants | mono_intergenic | H/ACA | intergenic |
| ENSRNA050017608 | snoR64 | T_aestivum | Plants | intergenic_cluster | C/D | intergenic |
| ENSRNA050017609 | snoZ199 | T_aestivum | Plants | intergenic_cluster | C/D | intergenic |
| ENSRNA050017611 | snoZ43 | T_aestivum | Plants | intergenic_cluster | C/D | intergenic |
| ENSRNA050017612 | snoZ43 | T_aestivum | Plants | intergenic_cluster | C/D | intergenic |
| ENSRNA050017613 | snoZ279_R105_R108 | T_aestivum | Plants | mono_intergenic | C/D | intergenic |
| ENSRNA050017614 | snoZ43 | T_aestivum | Plants | mono_intergenic | C/D | intergenic |
| ENSRNA050017615 | snoR77Y | T_aestivum | Plants | intergenic_cluster | C/D | intergenic |
| ENSRNA050017616 | snoZ43 | T_aestivum | Plants | mono_intergenic | C/D | intergenic |
| ENSRNA050017617 | snosnR60_Z15 | T_aestivum | Plants | intergenic_cluster | C/D | intergenic |
| ENSRNA050017618 | snoU31b | T_aestivum | Plants | intergenic_cluster | C/D | intergenic |
| ENSRNA050017619 | snoR8a | T_aestivum | Plants | mono_intergenic | C/D | intergenic |
| ENSRNA050017620 | snoR77Y | T_aestivum | Plants | intergenic_cluster | C/D | intergenic |
| ENSRNA050017621 | snoR77Y | T_aestivum | Plants | intergenic_cluster | C/D | intergenic |
| ENSRNA050017622 | snoU31b | T_aestivum | Plants | mono_intergenic | C/D | intergenic |
| ENSRNA050017623 | snoU31b | T_aestivum | Plants | mono_intergenic | C/D | intergenic |
| ENSRNA050017624 | snoR31_Z110_Z27 | T_aestivum | Plants | intergenic_cluster | C/D | intergenic |
| ENSRNA050017625 | snoR31_Z110_Z27 | T_aestivum | Plants | intergenic_cluster | C/D | intergenic |
| ENSRNA050017626 | snoU31b | T_aestivum | Plants | mono_intergenic | C/D | intergenic |
| ENSRNA050017627 | snoR31_Z110_Z27 | T_aestivum | Plants | intergenic_cluster | C/D | intergenic |
| ENSRNA050017628 | snoU31b | T_aestivum | Plants | mono_intergenic | C/D | intergenic |
| ENSRNA050017630 | snoU31b | T_aestivum | Plants | intergenic_cluster | C/D | intergenic |

| | | | | | | | |
|---|---|---|---|---|---|---|---|
| ENSRNA050017631 | snoR117 | T_aestivum | Plants | intergenic_cluster | C/D | | intergenic |
| ENSRNA050017644 | snoR64 | T_aestivum | Plants | intergenic_cluster | C/D | | intergenic |
| ENSRNA050017645 | snoZ199 | T_aestivum | Plants | intergenic_cluster | C/D | | intergenic |
| ENSRNA050017646 | snoZ199 | T_aestivum | Plants | intergenic_cluster | C/D | | intergenic |
| ENSRNA050017650 | snoR101 | T_aestivum | Plants | mono_intergenic | C/D | | intergenic |
| ENSRNA050017671 | snoU36a | T_aestivum | Plants | intergenic_cluster | C/D | | intergenic |
| ENSRNA050017673 | snoR83 | T_aestivum | Plants | intergenic_cluster | H/ACA | | intergenic |
| ENSRNA050017674 | snoR77Y | T_aestivum | Plants | mono_intergenic | C/D | | intergenic |
| ENSRNA050017675 | snoZ223 | T_aestivum | Plants | intergenic_cluster | C/D | | intergenic |
| ENSRNA050017676 | snoR77Y | T_aestivum | Plants | intergenic_cluster | C/D | | intergenic |
| ENSRNA050017679 | snoR31_Z110_Z27 | T_aestivum | Plants | intergenic_cluster | C/D | | intergenic |
| ENSRNA050017680 | snoZ278 | T_aestivum | Plants | intergenic_cluster | C/D | | intergenic |
| ENSRNA050017683 | snoR8a | T_aestivum | Plants | intergenic_cluster | C/D | | intergenic |
| ENSRNA050017684 | snoR80 | T_aestivum | Plants | mono_intergenic | H/ACA | | intergenic |
| ENSRNA050017689 | snoR64 | T_aestivum | Plants | intergenic_cluster | C/D | | intergenic |
| ENSRNA050017690 | snoZ199 | T_aestivum | Plants | intergenic_cluster | C/D | | intergenic |
| ENSRNA050017692 | snoZ199 | T_aestivum | Plants | intergenic_cluster | C/D | | intergenic |
| ENSRNA050017695 | snoZ101 | T_aestivum | Plants | intergenic_cluster | C/D | | intergenic |
| ENSRNA050017696 | snoR80 | T_aestivum | Plants | mono_intergenic | H/ACA | | intergenic |
| ENSRNA050017697 | snoR64 | T_aestivum | Plants | intergenic_cluster | C/D | | intergenic |
| ENSRNA050017698 | snoZ278 | T_aestivum | Plants | mono_intergenic | C/D | | intergenic |
| ENSRNA050017700 | snoZ101 | T_aestivum | Plants | intergenic_cluster | C/D | | intergenic |
| ENSRNA050017702 | snoZ199 | T_aestivum | Plants | intergenic_cluster | C/D | | intergenic |
| ENSRNA050017704 | snoZ199 | T_aestivum | Plants | intergenic_cluster | C/D | | intergenic |
| ENSRNA050017705 | snoR64 | T_aestivum | Plants | intergenic_cluster | C/D | | intergenic |
| ENSRNA050017730 | snoR11 | T_aestivum | Plants | intronic_cluster | C/D | TraesCS1A02G217200 | protein_coding |
| ENSRNA050017731 | snoZ152 | T_aestivum | Plants | intronic_cluster | C/D | TraesCS1A02G217200 | protein_coding |
| ENSRNA050018050 | snoR60 | T_aestivum | Plants | mono_intergenic | C/D | | intergenic |
| ENSRNA050018558 | snoZ101 | T_aestivum | Plants | intergenic_cluster | C/D | | intergenic |
| ENSRNA050018562 | snoZ101 | T_aestivum | Plants | intergenic_cluster | C/D | | intergenic |
| ENSRNA050018564 | snoZ101 | T_aestivum | Plants | intergenic_cluster | C/D | | intergenic |
| ENSRNA050018566 | snoZ101 | T_aestivum | Plants | intergenic_cluster | C/D | | intergenic |
| ENSRNA050018568 | snoZ101 | T_aestivum | Plants | intergenic_cluster | C/D | | intergenic |
| ENSRNA050018570 | snoZ101 | T_aestivum | Plants | intergenic_cluster | C/D | | intergenic |
| ENSRNA050018572 | snoZ101 | T_aestivum | Plants | intergenic_cluster | C/D | | intergenic |
| ENSRNA050018574 | snoZ101 | T_aestivum | Plants | intergenic_cluster | C/D | | intergenic |
| ENSRNA050018575 | snoZ101 | T_aestivum | Plants | intergenic_cluster | C/D | | intergenic |
| ENSRNA050018576 | snoZ101 | T_aestivum | Plants | intergenic_cluster | C/D | | intergenic |
| ENSRNA050018577 | snoZ101 | T_aestivum | Plants | intergenic_cluster | C/D | | intergenic |
| ENSRNA050018578 | snoZ101 | T_aestivum | Plants | mono_intergenic | C/D | | intergenic |
| ENSRNA050018579 | snoR64 | T_aestivum | Plants | mono_exonic | C/D | TraesCS1B02G039300 | protein_coding |
| ENSRNA050018580 | snoZ101 | T_aestivum | Plants | intergenic_cluster | C/D | | intergenic |
| ENSRNA050018581 | snoZ101 | T_aestivum | Plants | mono_intergenic | C/D | | intergenic |

| | | | | | | | |
|---|---|---|---|---|---|---|---|
| ENSRNA050018582 | snoZ101 | T_aestivum | Plants | intergenic_cluster | C/D | | intergenic |
| ENSRNA050018583 | snoZ101 | T_aestivum | Plants | mono_intergenic | C/D | | intergenic |
| ENSRNA050018584 | snoR8a | T_aestivum | Plants | intergenic_cluster | C/D | | intergenic |
| ENSRNA050018585 | snoR8a | T_aestivum | Plants | intergenic_cluster | C/D | | intergenic |
| ENSRNA050018603 | snoR8a | T_aestivum | Plants | intergenic_cluster | C/D | | intergenic |
| ENSRNA050018606 | snoR8a | T_aestivum | Plants | intergenic_cluster | C/D | | intergenic |
| ENSRNA050018611 | snoZ199 | T_aestivum | Plants | intronic_cluster | C/D | TraesCS1B02G039300 | protein_coding |
| ENSRNA050018614 | snoZ102_R77 | T_aestivum | Plants | intergenic_cluster | C/D | | intergenic |
| ENSRNA050018617 | snoZ199 | T_aestivum | Plants | intronic_cluster | C/D | TraesCS1B02G039300 | protein_coding |
| ENSRNA050018638 | snoZ155 | T_aestivum | Plants | mono_intergenic | C/D | | intergenic |
| ENSRNA050018855 | snoZ223 | T_aestivum | Plants | mono_intergenic | C/D | | intergenic |
| ENSRNA050018875 | snoZ43 | T_aestivum | Plants | intergenic_cluster | C/D | | intergenic |
| ENSRNA050018876 | snoR77 | T_aestivum | Plants | intergenic_cluster | H/ACA | | intergenic |
| ENSRNA050018877 | snoR77 | T_aestivum | Plants | intergenic_cluster | H/ACA | | intergenic |
| ENSRNA050018878 | snoR77 | T_aestivum | Plants | intergenic_cluster | H/ACA | | intergenic |
| ENSRNA050018879 | snoR77 | T_aestivum | Plants | intergenic_cluster | H/ACA | | intergenic |
| ENSRNA050018880 | snoR77 | T_aestivum | Plants | intergenic_cluster | H/ACA | | intergenic |
| ENSRNA050018881 | snoR77 | T_aestivum | Plants | intergenic_cluster | H/ACA | | intergenic |
| ENSRNA050018882 | snoR44_J54 | T_aestivum | Plants | intergenic_cluster | C/D | | intergenic |
| ENSRNA050018883 | snoZ159 | T_aestivum | Plants | intergenic_cluster | C/D | | intergenic |
| ENSRNA050018884 | snoZ159 | T_aestivum | Plants | mono_intergenic | C/D | | intergenic |
| ENSRNA050018885 | snoR44_J54 | T_aestivum | Plants | intergenic_cluster | C/D | | intergenic |
| ENSRNA050018886 | snoR44_J54 | T_aestivum | Plants | intergenic_cluster | C/D | | intergenic |
| ENSRNA050018887 | snosnR60_Z15 | T_aestivum | Plants | mono_intergenic | C/D | | intergenic |
| ENSRNA050018888 | snosnR60_Z15 | T_aestivum | Plants | intergenic_cluster | C/D | | intergenic |
| ENSRNA050018889 | snoZ159 | T_aestivum | Plants | mono_intergenic | C/D | | intergenic |
| ENSRNA050018890 | snoR44_J54 | T_aestivum | Plants | intergenic_cluster | C/D | | intergenic |
| ENSRNA050018891 | snoZ152 | T_aestivum | Plants | intergenic_cluster | C/D | | intergenic |
| ENSRNA050018892 | snoR77Y | T_aestivum | Plants | mono_intergenic | C/D | | intergenic |
| ENSRNA050018893 | snoU31b | T_aestivum | Plants | mono_intergenic | C/D | | intergenic |
| ENSRNA050018894 | snoU31b | T_aestivum | Plants | mono_intergenic | C/D | | intergenic |
| ENSRNA050018895 | snoU31b | T_aestivum | Plants | mono_intergenic | C/D | | intergenic |
| ENSRNA050018896 | snoR77Y | T_aestivum | Plants | mono_intergenic | C/D | | intergenic |
| ENSRNA050018897 | snoR11 | T_aestivum | Plants | intergenic_cluster | C/D | | intergenic |
| ENSRNA050018898 | snoR8a | T_aestivum | Plants | mono_intergenic | C/D | | intergenic |
| ENSRNA050018899 | snoR77Y | T_aestivum | Plants | mono_intergenic | C/D | | intergenic |
| ENSRNA050018900 | snosnR60_Z15 | T_aestivum | Plants | intergenic_cluster | C/D | | intergenic |
| ENSRNA050018901 | snoR77 | T_aestivum | Plants | intergenic_cluster | H/ACA | | intergenic |
| ENSRNA050019257 | snoZ279_R105_R108 | T_aestivum | Plants | mono_intergenic | C/D | | intergenic |
| ENSRNA050019258 | snoZ279_R105_R108 | T_aestivum | Plants | mono_intergenic | C/D | | intergenic |
| ENSRNA050019277 | SNOR75 | T_aestivum | Plants | mono_intergenic | C/D | | intergenic |
| ENSRNA050019278 | snoR77 | T_aestivum | Plants | intergenic_cluster | H/ACA | | intergenic |
| ENSRNA050019279 | snoR77 | T_aestivum | Plants | intergenic_cluster | H/ACA | | intergenic |

| | | | | | | | |
|---|---|---|---|---|---|---|---|
| ENSRNA050019280 | snoR77 | T_aestivum | Plants | intergenic_cluster | H/ACA | | intergenic |
| ENSRNA050019281 | snoR77 | T_aestivum | Plants | intergenic_cluster | H/ACA | | intergenic |
| ENSRNA050019282 | snoR77 | T_aestivum | Plants | intergenic_cluster | H/ACA | | intergenic |
| ENSRNA050019283 | snoZ159 | T_aestivum | Plants | mono_intergenic | C/D | | intergenic |
| ENSRNA050019284 | snoR44_J54 | T_aestivum | Plants | intergenic_cluster | C/D | | intergenic |
| ENSRNA050019285 | snoR44_J54 | T_aestivum | Plants | intergenic_cluster | C/D | | intergenic |
| ENSRNA050019286 | snoR44_J54 | T_aestivum | Plants | intergenic_cluster | C/D | | intergenic |
| ENSRNA050019287 | snoZ155 | T_aestivum | Plants | mono_intergenic | C/D | | intergenic |
| ENSRNA050019288 | snoZ159 | T_aestivum | Plants | mono_intergenic | C/D | | intergenic |
| ENSRNA050019289 | snoR11 | T_aestivum | Plants | mono_intergenic | C/D | | intergenic |
| ENSRNA050019290 | snoZ152 | T_aestivum | Plants | intergenic_cluster | C/D | | intergenic |
| ENSRNA050019291 | snoR11 | T_aestivum | Plants | intergenic_cluster | C/D | | intergenic |
| ENSRNA050019292 | snoZ159 | T_aestivum | Plants | mono_intergenic | C/D | | intergenic |
| ENSRNA050019293 | snoZ155 | T_aestivum | Plants | mono_intergenic | C/D | | intergenic |
| ENSRNA050019304 | snoZ223 | T_aestivum | Plants | mono_intergenic | C/D | | intergenic |
| ENSRNA050019306 | snoR77Y | T_aestivum | Plants | mono_intergenic | C/D | | intergenic |
| ENSRNA050019307 | snoU31b | T_aestivum | Plants | mono_intergenic | C/D | | intergenic |
| ENSRNA050019313 | snoR83 | T_aestivum | Plants | intergenic_cluster | H/ACA | | intergenic |
| ENSRNA050019314 | snoU36a | T_aestivum | Plants | intergenic_cluster | C/D | | intergenic |
| ENSRNA050019315 | snoR77Y | T_aestivum | Plants | intergenic_cluster | C/D | | intergenic |
| ENSRNA050019316 | snoZ223 | T_aestivum | Plants | intergenic_cluster | C/D | | intergenic |
| ENSRNA050019318 | snoZ223 | T_aestivum | Plants | mono_intergenic | C/D | | intergenic |
| ENSRNA050019319 | snoR31_Z110_Z27 | T_aestivum | Plants | intergenic_cluster | C/D | | intergenic |
| ENSRNA050019320 | snoZ278 | T_aestivum | Plants | intergenic_cluster | C/D | | intergenic |
| ENSRNA050019321 | snoR8a | T_aestivum | Plants | intergenic_cluster | C/D | | intergenic |
| ENSRNA050019401 | snoZ152 | T_aestivum | Plants | intergenic_cluster | C/D | | intergenic |
| ENSRNA050019402 | snoR11 | T_aestivum | Plants | intergenic_cluster | C/D | | intergenic |
| ENSRNA050019406 | SNORD24 | T_aestivum | Plants | intronic_cluster | C/D | TraesCS2D02G263000 | protein_coding |
| ENSRNA050019407 | SNORD24 | T_aestivum | Plants | intronic_cluster | C/D | TraesCS2D02G263000 | protein_coding |
| ENSRNA050019409 | snoR86 | T_aestivum | Plants | intronic_cluster | H/ACA | TraesCS4A02G306500 | protein_coding |
| ENSRNA050019410 | snoR86 | T_aestivum | Plants | intronic_cluster | H/ACA | TraesCS4A02G306500 | protein_coding |
| ENSRNA050019411 | snoR86 | T_aestivum | Plants | intronic_cluster | H/ACA | TraesCS4A02G306500 | protein_coding |
| ENSRNA050019412 | snoR86 | T_aestivum | Plants | intronic_cluster | H/ACA | TraesCS4A02G306500 | protein_coding |
| ENSRNA050019413 | snoR86 | T_aestivum | Plants | intronic_cluster | H/ACA | TraesCS4A02G306500 | protein_coding |
| ENSRNA050019414 | snoZ266 | T_aestivum | Plants | intronic_cluster | C/D | TraesCS4A02G306500 | protein_coding |
| ENSRNA050019415 | snoR86 | T_aestivum | Plants | intronic_cluster | H/ACA | TraesCS4A02G306500 | protein_coding |
| ENSRNA050019416 | snoR86 | T_aestivum | Plants | intronic_cluster | H/ACA | TraesCS4A02G306500 | protein_coding |
| ENSRNA050019449 | snoZ43 | T_aestivum | Plants | mono_intergenic | C/D | | intergenic |
| ENSRNA050019451 | snoR11 | T_aestivum | Plants | intergenic_cluster | C/D | | intergenic |
| ENSRNA050019452 | snoZ152 | T_aestivum | Plants | intergenic_cluster | C/D | | intergenic |
| ENSRNA050019460 | snoR77Y | T_aestivum | Plants | mono_intergenic | C/D | | intergenic |
| ENSRNA050019462 | snoZ43 | T_aestivum | Plants | mono_intergenic | C/D | | intergenic |
| ENSRNA050019468 | snoR44_J54 | T_aestivum | Plants | intergenic_cluster | C/D | | intergenic |

| | | | | | | | |
|---|---|---|---|---|---|---|---|
| ENSRNA050019469 | snoR44_J54 | T_aestivum | Plants | intergenic_cluster | C/D | | intergenic |
| ENSRNA050019470 | snoR77Y | T_aestivum | Plants | intergenic_cluster | C/D | | intergenic |
| ENSRNA050019471 | snoR31_Z110_Z27 | T_aestivum | Plants | intergenic_cluster | C/D | | intergenic |
| ENSRNA050019472 | snoR60 | T_aestivum | Plants | intronic_cluster | C/D | TraesCS7A02G479900 | protein_coding |
| ENSRNA050019473 | snoR60 | T_aestivum | Plants | intronic_cluster | C/D | TraesCS7A02G479900 | protein_coding |
| ENSRNA050019474 | snoR60 | T_aestivum | Plants | intronic_cluster | C/D | TraesCS7A02G479900 | protein_coding |
| ENSRNA050019476 | snoR60 | T_aestivum | Plants | intronic_cluster | C/D | TraesCS7A02G479900 | protein_coding |
| ENSRNA050019566 | snoR80 | T_aestivum | Plants | mono_intergenic | H/ACA | | intergenic |
| ENSRNA050019569 | snoR28 | T_aestivum | Plants | mono_exonic | C/D | TraesCS7B02G055600 | protein_coding |
| ENSRNA050019577 | snoU36a | T_aestivum | Plants | intergenic_cluster | C/D | | intergenic |
| ENSRNA050019578 | snoZ223 | T_aestivum | Plants | intergenic_cluster | C/D | | intergenic |
| ENSRNA050019579 | snoZ159 | T_aestivum | Plants | intergenic_cluster | C/D | | intergenic |
| ENSRNA050019580 | snoZ159 | T_aestivum | Plants | intergenic_cluster | C/D | | intergenic |
| ENSRNA050019582 | snoR31_Z110_Z27 | T_aestivum | Plants | mono_intergenic | C/D | | intergenic |
| ENSRNA050019583 | snoZ223 | T_aestivum | Plants | intergenic_cluster | C/D | | intergenic |
| ENSRNA050019585 | SNORD24 | T_aestivum | Plants | mono_intergenic | C/D | | intergenic |
| ENSRNA050019589 | snoZ199 | T_aestivum | Plants | intergenic_cluster | C/D | | intergenic |
| ENSRNA050019590 | snoR64 | T_aestivum | Plants | intergenic_cluster | C/D | | intergenic |
| ENSRNA050019591 | snoZ199 | T_aestivum | Plants | intergenic_cluster | C/D | | intergenic |
| ENSRNA050019593 | snoR64 | T_aestivum | Plants | intergenic_cluster | C/D | | intergenic |
| ENSRNA050019595 | snoZ199 | T_aestivum | Plants | intergenic_cluster | C/D | | intergenic |
| ENSRNA050019596 | snoZ199 | T_aestivum | Plants | intergenic_cluster | C/D | | intergenic |
| ENSRNA050019601 | snoZ279_R105_R108 | T_aestivum | Plants | mono_intergenic | C/D | | intergenic |
| ENSRNA050019602 | SNOR75 | T_aestivum | Plants | mono_exonic | C/D | TraesCS1D02G373100 | protein_coding |
| ENSRNA050019603 | SNOR75 | T_aestivum | Plants | mono_intergenic | C/D | | intergenic |
| ENSRNA050019605 | snoZ188 | T_aestivum | Plants | mono_intergenic | C/D | | intergenic |
| ENSRNA050019606 | snoZ223 | T_aestivum | Plants | mono_intronic | C/D | TraesCS4D02G169400 | protein_coding |
| ENSRNA050019609 | snoR101 | T_aestivum | Plants | mono_intergenic | C/D | | intergenic |
| ENSRNA050019613 | U54 | T_aestivum | Plants | intergenic_cluster | C/D | | intergenic |
| ENSRNA050019614 | U54 | T_aestivum | Plants | intergenic_cluster | C/D | | intergenic |
| ENSRNA050019615 | U54 | T_aestivum | Plants | intergenic_cluster | C/D | | intergenic |
| ENSRNA050019616 | U54 | T_aestivum | Plants | intergenic_cluster | C/D | | intergenic |
| ENSRNA050019618 | U54 | T_aestivum | Plants | intergenic_cluster | C/D | | intergenic |
| ENSRNA050019623 | SNORD24 | T_aestivum | Plants | mono_intergenic | C/D | | intergenic |
| ENSRNA050019632 | snoR80 | T_aestivum | Plants | mono_intergenic | H/ACA | | intergenic |
| ENSRNA050019650 | snoZ118 | T_aestivum | Plants | mono_intergenic | C/D | | intergenic |
| ENSRNA050019653 | snoZ223 | T_aestivum | Plants | mono_intergenic | C/D | | intergenic |
| ENSRNA050019654 | snoZ223 | T_aestivum | Plants | intergenic_cluster | C/D | | intergenic |
| ENSRNA050019655 | snoR134 | T_aestivum | Plants | intergenic_cluster | H/ACA | | intergenic |
| ENSRNA050019656 | SNORD24 | T_aestivum | Plants | mono_intergenic | C/D | | intergenic |
| ENSRNA050019675 | snoU36a | T_aestivum | Plants | intergenic_cluster | C/D | | intergenic |
| ENSRNA050019676 | snoZ223 | T_aestivum | Plants | intergenic_cluster | C/D | | intergenic |
| ENSRNA050019677 | snoZ278 | T_aestivum | Plants | intergenic_cluster | C/D | | intergenic |

| | | | | | | | |
|---|---|---|---|---|---|---|---|
| ENSRNA050019679 | snoR60 | T_aestivum | Plants | intronic_cluster | C/D | TraesCS6A02G400300 | protein_coding |
| ENSRNA050019680 | snoR60 | T_aestivum | Plants | intronic_cluster | C/D | TraesCS6A02G400300 | protein_coding |
| ENSRNA050019681 | SNORD25 | T_aestivum | Plants | intronic_cluster | C/D | TraesCS6A02G400300 | protein_coding |
| ENSRNA050019682 | snoR60 | T_aestivum | Plants | mono_intergenic | C/D | | intergenic |
| ENSRNA050019686 | snoR101 | T_aestivum | Plants | mono_intergenic | C/D | | intergenic |
| ENSRNA050019691 | snoR60 | T_aestivum | Plants | mono_intergenic | C/D | | intergenic |
| ENSRNA050019723 | snoZ43 | T_aestivum | Plants | intergenic_cluster | C/D | | intergenic |
| ENSRNA050019724 | snoZ101 | T_aestivum | Plants | intergenic_cluster | C/D | | intergenic |
| ENSRNA050019725 | snoZ101 | T_aestivum | Plants | intergenic_cluster | C/D | | intergenic |
| ENSRNA050019726 | snoZ101 | T_aestivum | Plants | intergenic_cluster | C/D | | intergenic |
| ENSRNA050019727 | snoZ101 | T_aestivum | Plants | intergenic_cluster | C/D | | intergenic |
| ENSRNA050019728 | snoZ101 | T_aestivum | Plants | intergenic_cluster | C/D | | intergenic |
| ENSRNA050019729 | snoZ101 | T_aestivum | Plants | intergenic_cluster | C/D | | intergenic |
| ENSRNA050019731 | snosnR60_Z15 | T_aestivum | Plants | intergenic_cluster | C/D | | intergenic |
| ENSRNA050019733 | snoU31b | T_aestivum | Plants | intergenic_cluster | C/D | | intergenic |
| ENSRNA050019734 | snoR77Y | T_aestivum | Plants | mono_intergenic | C/D | | intergenic |
| ENSRNA050019736 | snoZ199 | T_aestivum | Plants | intergenic_cluster | C/D | | intergenic |
| ENSRNA050019737 | snoR8a | T_aestivum | Plants | intergenic_cluster | C/D | | intergenic |
| ENSRNA050019738 | snoZ102_R77 | T_aestivum | Plants | intergenic_cluster | C/D | | intergenic |
| ENSRNA050019740 | snoZ102_R77 | T_aestivum | Plants | intergenic_cluster | C/D | | intergenic |
| ENSRNA050019741 | snoR64 | T_aestivum | Plants | intergenic_cluster | C/D | | intergenic |
| ENSRNA050019742 | snoZ102_R77 | T_aestivum | Plants | intergenic_cluster | C/D | | intergenic |
| ENSRNA050019743 | snoZ102_R77 | T_aestivum | Plants | intergenic_cluster | C/D | | intergenic |
| ENSRNA050019744 | snoU31b | T_aestivum | Plants | intergenic_cluster | C/D | | intergenic |
| ENSRNA050019745 | snoU31b | T_aestivum | Plants | intergenic_cluster | C/D | | intergenic |
| ENSRNA050019746 | snoR77Y | T_aestivum | Plants | mono_intergenic | C/D | | intergenic |
| ENSRNA050019747 | snoR8a | T_aestivum | Plants | intergenic_cluster | C/D | | intergenic |
| ENSRNA050019750 | snoZ118 | T_aestivum | Plants | intergenic_cluster | C/D | | intergenic |
| ENSRNA050019751 | snoZ118 | T_aestivum | Plants | intergenic_cluster | C/D | | intergenic |
| ENSRNA050019752 | snoZ118 | T_aestivum | Plants | intergenic_cluster | C/D | | intergenic |
| ENSRNA050019753 | snoZ118 | T_aestivum | Plants | intergenic_cluster | C/D | | intergenic |
| ENSRNA050019754 | snoZ118 | T_aestivum | Plants | mono_exonic | C/D | TraesCS6D02G043400 | protein_coding |
| ENSRNA050019755 | snoZ199 | T_aestivum | Plants | intronic_cluster | C/D | TraesCS6D02G033200 | protein_coding |
| ENSRNA050019756 | snoZ118 | T_aestivum | Plants | intergenic_cluster | C/D | | intergenic |
| ENSRNA050019757 | snoZ118 | T_aestivum | Plants | intergenic_cluster | C/D | | intergenic |
| ENSRNA050019758 | snoZ118 | T_aestivum | Plants | mono_exonic | C/D | TraesCS6D02G043400 | protein_coding |
| ENSRNA050019759 | snoR64 | T_aestivum | Plants | intronic_cluster | C/D | TraesCS6D02G033200 | protein_coding |
| ENSRNA050019760 | snoZ199 | T_aestivum | Plants | intronic_cluster | C/D | TraesCS6D02G033200 | protein_coding |
| ENSRNA050019761 | snoZ118 | T_aestivum | Plants | intergenic_cluster | C/D | | intergenic |
| ENSRNA050019762 | snoZ118 | T_aestivum | Plants | intergenic_cluster | C/D | | intergenic |
| ENSRNA050019764 | snoR60 | T_aestivum | Plants | intronic_cluster | C/D | TraesCS6D02G384500 | protein_coding |
| ENSRNA050019765 | snoR60 | T_aestivum | Plants | intronic_cluster | C/D | TraesCS6D02G384500 | protein_coding |
| ENSRNA050019766 | SNORD25 | T_aestivum | Plants | intronic_cluster | C/D | TraesCS6D02G384500 | protein_coding |

| | | | | | | | |
|---|---|---|---|---|---|---|---|
| ENSRNA050019768 | snoR77Y | T_aestivum | Plants | mono_intergenic | C/D | | intergenic |
| ENSRNA050019769 | snosnR60_Z15 | T_aestivum | Plants | mono_intergenic | C/D | | intergenic |
| ENSRNA050019801 | snoZ199 | T_aestivum | Plants | mono_intergenic | C/D | | intergenic |
| ENSRNA050019802 | SNORD25 | T_aestivum | Plants | mono_intergenic | C/D | | intergenic |
| ENSRNA050019812 | snoR101 | T_aestivum | Plants | mono_intergenic | C/D | | intergenic |
| ENSRNA050019835 | snoR77 | T_aestivum | Plants | mono_intergenic | H/ACA | | intergenic |
| ENSRNA050019867 | snoZ43 | T_aestivum | Plants | intergenic_cluster | C/D | | intergenic |
| ENSRNA050019868 | snoZ43 | T_aestivum | Plants | intergenic_cluster | C/D | | intergenic |
| ENSRNA050019869 | snoZ279_R105_R108 | T_aestivum | Plants | mono_intergenic | C/D | | intergenic |
| ENSRNA050019870 | snoU31b | T_aestivum | Plants | intergenic_cluster | C/D | | intergenic |
| ENSRNA050019871 | snosnR60_Z15 | T_aestivum | Plants | intergenic_cluster | C/D | | intergenic |
| ENSRNA050019872 | snoR77Y | T_aestivum | Plants | intergenic_cluster | C/D | | intergenic |
| ENSRNA050019873 | snoR8a | T_aestivum | Plants | mono_intergenic | C/D | | intergenic |
| ENSRNA050019874 | snoR31_Z110_Z27 | T_aestivum | Plants | intergenic_cluster | C/D | | intergenic |
| ENSRNA050019875 | snoZ118 | T_aestivum | Plants | mono_intergenic | C/D | | intergenic |
| ENSRNA050019879 | snoR44_J54 | T_aestivum | Plants | mono_intergenic | C/D | | intergenic |
| ENSRNA050019880 | snoZ152 | T_aestivum | Plants | mono_intergenic | C/D | | intergenic |
| ENSRNA050019907 | snoR80 | T_aestivum | Plants | mono_intergenic | H/ACA | | intergenic |
| ENSRNA050019915 | snoZ188 | T_aestivum | Plants | mono_intergenic | C/D | | intergenic |
| ENSRNA050019916 | snoZ188 | T_aestivum | Plants | mono_intergenic | C/D | | intergenic |
| ENSRNA050019920 | snoZ118 | T_aestivum | Plants | mono_intergenic | C/D | | intergenic |
| ENSRNA050019924 | snoZ279_R105_R108 | T_aestivum | Plants | mono_intergenic | C/D | | intergenic |
| ENSRNA050019929 | SNORD24 | T_aestivum | Plants | mono_intergenic | C/D | | intergenic |
| ENSRNA050019930 | snoZ152 | T_aestivum | Plants | intergenic_cluster | C/D | | intergenic |
| ENSRNA050019931 | snoR11 | T_aestivum | Plants | intergenic_cluster | C/D | | intergenic |
| ENSRNA050019934 | snoR80 | T_aestivum | Plants | mono_intergenic | H/ACA | | intergenic |
| ENSRNA050019949 | snoR64 | T_aestivum | Plants | intergenic_cluster | C/D | | intergenic |
| ENSRNA050019950 | snoZ199 | T_aestivum | Plants | intergenic_cluster | C/D | | intergenic |
| ENSRNA050019951 | snoZ199 | T_aestivum | Plants | intergenic_cluster | C/D | | intergenic |
| ENSRNA050019957 | snoR86 | T_aestivum | Plants | intronic_cluster | H/ACA | TraesCS4B02G008400 | protein_coding |
| ENSRNA050019958 | snoZ266 | T_aestivum | Plants | intronic_cluster | C/D | TraesCS4B02G008400 | protein_coding |
| ENSRNA050019959 | snoR86 | T_aestivum | Plants | intronic_cluster | H/ACA | TraesCS4B02G008400 | protein_coding |
| ENSRNA050019960 | snoR86 | T_aestivum | Plants | intronic_cluster | H/ACA | TraesCS4B02G008400 | protein_coding |
| ENSRNA050019961 | snoR86 | T_aestivum | Plants | intronic_cluster | H/ACA | TraesCS4B02G008400 | protein_coding |
| ENSRNA050019962 | snoR86 | T_aestivum | Plants | intronic_cluster | H/ACA | TraesCS4B02G008400 | protein_coding |
| ENSRNA050019967 | snoR1 | T_aestivum | Plants | mono_intergenic | C/D | | intergenic |
| ENSRNA050019974 | snoZ118 | T_aestivum | Plants | intronic_cluster | C/D | TraesCS6A02G037900 | protein_coding |
| ENSRNA050019975 | snoZ118 | T_aestivum | Plants | intronic_cluster | C/D | TraesCS6A02G037900 | protein_coding |
| ENSRNA050019976 | snoZ118 | T_aestivum | Plants | intronic_cluster | C/D | TraesCS6A02G037900 | protein_coding |
| ENSRNA050019977 | snoZ118 | T_aestivum | Plants | intronic_cluster | C/D | TraesCS6A02G037900 | protein_coding |
| ENSRNA050019978 | snoZ118 | T_aestivum | Plants | intronic_cluster | C/D | TraesCS6A02G037900 | protein_coding |
| ENSRNA050019979 | snoZ199 | T_aestivum | Plants | intergenic_cluster | C/D | | intergenic |
| ENSRNA050019980 | snoZ118 | T_aestivum | Plants | intronic_cluster | C/D | TraesCS6A02G037900 | protein_coding |

| | | | | | | | |
|---|---|---|---|---|---|---|---|
| ENSRNA050019981 | snoZ118 | T_aestivum | Plants | intronic_cluster | C/D | TraesCS6A02G037900 | protein_coding |
| ENSRNA050019982 | snoZ118 | T_aestivum | Plants | intronic_cluster | C/D | TraesCS6A02G037900 | protein_coding |
| ENSRNA050019983 | snoR64 | T_aestivum | Plants | intergenic_cluster | C/D | | intergenic |
| ENSRNA050019984 | snoZ118 | T_aestivum | Plants | intronic_cluster | C/D | TraesCS6A02G037900 | protein_coding |
| ENSRNA050019985 | snoZ118 | T_aestivum | Plants | intronic_cluster | C/D | TraesCS6A02G037900 | protein_coding |
| ENSRNA050019986 | snoZ199 | T_aestivum | Plants | intergenic_cluster | C/D | | intergenic |
| ENSRNA050019987 | SNORD25 | T_aestivum | Plants | mono_intergenic | C/D | | intergenic |
| ENSRNA050019991 | snoZ118 | T_aestivum | Plants | mono_intergenic | C/D | | intergenic |
| ENSRNA050019993 | snoZ223 | T_aestivum | Plants | mono_intergenic | C/D | | intergenic |
| ENSRNA050020003 | snoZ279_R105_R108 | T_aestivum | Plants | mono_intergenic | C/D | | intergenic |
| ENSRNA050020021 | snoR1 | T_aestivum | Plants | intergenic_cluster | C/D | | intergenic |
| ENSRNA050020022 | snoR1 | T_aestivum | Plants | intergenic_cluster | C/D | | intergenic |
| ENSRNA050020023 | snoR1 | T_aestivum | Plants | intergenic_cluster | C/D | | intergenic |
| ENSRNA050020024 | snoZ223 | T_aestivum | Plants | mono_intergenic | C/D | | intergenic |
| ENSRNA050020025 | snoZ278 | T_aestivum | Plants | intergenic_cluster | C/D | | intergenic |
| ENSRNA050020026 | snoZ199 | T_aestivum | Plants | intergenic_cluster | C/D | | intergenic |
| ENSRNA050020030 | snoZ159 | T_aestivum | Plants | intergenic_cluster | C/D | | intergenic |
| ENSRNA050020031 | snoR77Y | T_aestivum | Plants | mono_intergenic | C/D | | intergenic |
| ENSRNA050020032 | snoU31b | T_aestivum | Plants | mono_intergenic | C/D | | intergenic |
| ENSRNA050020033 | snoZ159 | T_aestivum | Plants | mono_intergenic | C/D | | intergenic |
| ENSRNA050020034 | snoR80 | T_aestivum | Plants | intergenic_cluster | H/ACA | | intergenic |
| ENSRNA050020035 | snoR80 | T_aestivum | Plants | intergenic_cluster | H/ACA | | intergenic |
| ENSRNA050020036 | snoR80 | T_aestivum | Plants | intergenic_cluster | H/ACA | | intergenic |
| ENSRNA050020037 | snoZ159 | T_aestivum | Plants | intergenic_cluster | C/D | | intergenic |
| ENSRNA050020038 | snoR8a | T_aestivum | Plants | mono_intergenic | C/D | | intergenic |
| ENSRNA050020039 | SNORD24 | T_aestivum | Plants | intergenic_cluster | C/D | | intergenic |
| ENSRNA050020040 | SNORD24 | T_aestivum | Plants | intergenic_cluster | C/D | | intergenic |
| ENSRNA050020041 | snoZ159 | T_aestivum | Plants | intergenic_cluster | C/D | | intergenic |
| ENSRNA050020042 | SNORD24 | T_aestivum | Plants | intergenic_cluster | C/D | | intergenic |
| ENSRNA050020043 | snoR117 | T_aestivum | Plants | intronic_cluster | C/D | TraesCS6D02G087300 | protein_coding |
| ENSRNA050020044 | snoR117 | T_aestivum | Plants | intronic_cluster | C/D | TraesCS6D02G087300 | protein_coding |
| ENSRNA050020058 | snoR44_J54 | T_aestivum | Plants | mono_intergenic | C/D | | intergenic |
| ENSRNA050020059 | snoR44_J54 | T_aestivum | Plants | intergenic_cluster | C/D | | intergenic |
| ENSRNA050020060 | snoZ223 | T_aestivum | Plants | mono_intergenic | C/D | | intergenic |
| ENSRNA050020061 | snoR44_J54 | T_aestivum | Plants | intergenic_cluster | C/D | | intergenic |
| ENSRNA050020062 | snoR44_J54 | T_aestivum | Plants | intergenic_cluster | C/D | | intergenic |
| ENSRNA050020063 | snoU36a | T_aestivum | Plants | intergenic_cluster | C/D | | intergenic |
| ENSRNA050020064 | snoZ223 | T_aestivum | Plants | intergenic_cluster | C/D | | intergenic |
| ENSRNA050020079 | snoR101 | T_aestivum | Plants | mono_intergenic | C/D | | intergenic |
| ENSRNA050020086 | snoR60 | T_aestivum | Plants | intronic_cluster | C/D | TraesCS7B02G382200 | protein_coding |
| ENSRNA050020087 | snoR60 | T_aestivum | Plants | intronic_cluster | C/D | TraesCS7B02G382200 | protein_coding |
| ENSRNA050020091 | snoZ43 | T_aestivum | Plants | mono_intergenic | C/D | | intergenic |
| ENSRNA050020094 | snoR64 | T_aestivum | Plants | intergenic_cluster | C/D | | intergenic |

| | | | | | | | |
|---|---|---|---|---|---|---|---|
| ENSRNA050020095 | snoZ199 | T_aestivum | Plants | intergenic_cluster | C/D | | intergenic |
| ENSRNA050020098 | snoZ43 | T_aestivum | Plants | mono_intergenic | C/D | | intergenic |
| ENSRNA050020099 | snoZ152 | T_aestivum | Plants | intergenic_cluster | C/D | | intergenic |
| ENSRNA050020100 | snoR11 | T_aestivum | Plants | intergenic_cluster | C/D | | intergenic |
| ENSRNA050020102 | snoR64 | T_aestivum | Plants | intergenic_cluster | C/D | | intergenic |
| ENSRNA050020103 | snoZ199 | T_aestivum | Plants | intergenic_cluster | C/D | | intergenic |
| ENSRNA050020105 | snoZ199 | T_aestivum | Plants | intergenic_cluster | C/D | | intergenic |
| ENSRNA050020179 | snoR101 | T_aestivum | Plants | mono_intergenic | C/D | | intergenic |
| ENSRNA050020192 | snoZ43 | T_aestivum | Plants | intergenic_cluster | C/D | | intergenic |
| ENSRNA050020194 | SNORD25 | T_aestivum | Plants | mono_intergenic | C/D | | intergenic |
| ENSRNA050020195 | snoR77Y | T_aestivum | Plants | intergenic_cluster | C/D | | intergenic |
| ENSRNA050020196 | snosnR60_Z15 | T_aestivum | Plants | intergenic_cluster | C/D | | intergenic |
| ENSRNA050020197 | SNORD25 | T_aestivum | Plants | mono_intergenic | C/D | | intergenic |
| ENSRNA050020198 | snoR31_Z110_Z27 | T_aestivum | Plants | intergenic_cluster | C/D | | intergenic |
| ENSRNA050020199 | SNORD25 | T_aestivum | Plants | mono_intergenic | C/D | | intergenic |
| ENSRNA050020623 | snoU36a | T_aestivum | Plants | intergenic_cluster | C/D | | intergenic |
| ENSRNA050020624 | snoR1 | T_aestivum | Plants | intergenic_cluster | C/D | | intergenic |
| ENSRNA050020625 | snoR134 | T_aestivum | Plants | intergenic_cluster | H/ACA | | intergenic |
| ENSRNA050020626 | SNORD25 | T_aestivum | Plants | intergenic_cluster | C/D | | intergenic |
| ENSRNA050020627 | snoR1 | T_aestivum | Plants | intergenic_cluster | C/D | | intergenic |
| ENSRNA050020628 | snoZ223 | T_aestivum | Plants | intergenic_cluster | C/D | | intergenic |
| ENSRNA050020629 | snoZ223 | T_aestivum | Plants | intergenic_cluster | C/D | | intergenic |
| ENSRNA050020630 | snoR1 | T_aestivum | Plants | intergenic_cluster | C/D | | intergenic |
| ENSRNA050020632 | snoZ199 | T_aestivum | Plants | mono_intergenic | C/D | | intergenic |
| ENSRNA050020726 | snoU36a | T_aestivum | Plants | intergenic_cluster | C/D | | intergenic |
| ENSRNA050020731 | snoZ223 | T_aestivum | Plants | intergenic_cluster | C/D | | intergenic |
| ENSRNA050020735 | snoZ159 | T_aestivum | Plants | intergenic_cluster | C/D | | intergenic |
| ENSRNA050020745 | snoZ43 | T_aestivum | Plants | mono_intergenic | C/D | | intergenic |
| ENSRNA050020746 | snoR11 | T_aestivum | Plants | intergenic_cluster | C/D | | intergenic |
| ENSRNA050020747 | snoZ152 | T_aestivum | Plants | intergenic_cluster | C/D | | intergenic |
| ENSRNA050020748 | snoZ279_R105_R108 | T_aestivum | Plants | mono_intergenic | C/D | | intergenic |
| ENSRNA050020750 | snoZ159 | T_aestivum | Plants | mono_intergenic | C/D | | intergenic |
| ENSRNA050020751 | snoZ159 | T_aestivum | Plants | mono_intergenic | C/D | | intergenic |
| ENSRNA050020752 | snoR80 | T_aestivum | Plants | mono_intergenic | H/ACA | | intergenic |
| ENSRNA050020755 | snoZ118 | T_aestivum | Plants | mono_intergenic | C/D | | intergenic |
| ENSRNA050020756 | snoZ118 | T_aestivum | Plants | mono_intergenic | C/D | | intergenic |
| ENSRNA050020763 | snoZ223 | T_aestivum | Plants | intergenic_cluster | C/D | | intergenic |
| ENSRNA050020764 | snoU36a | T_aestivum | Plants | intergenic_cluster | C/D | | intergenic |
| ENSRNA050020765 | snoZ159 | T_aestivum | Plants | intergenic_cluster | C/D | | intergenic |
| ENSRNA050020766 | snoU36a | T_aestivum | Plants | intergenic_cluster | C/D | | intergenic |
| ENSRNA050020767 | snoZ223 | T_aestivum | Plants | intergenic_cluster | C/D | | intergenic |
| ENSRNA050020768 | snoR31_Z110_Z27 | T_aestivum | Plants | mono_intergenic | C/D | | intergenic |
| ENSRNA050020769 | snoZ118 | T_aestivum | Plants | intronic_cluster | C/D | TraesCS6B02G052900 | protein_coding |

| | | | | | | | |
|---|---|---|---|---|---|---|---|
| ENSRNA050020770 | snoZ118 | T_aestivum | Plants | intronic_cluster | C/D | TraesCS6B02G052900 | protein_coding |
| ENSRNA050020771 | snoZ118 | T_aestivum | Plants | intronic_cluster | C/D | TraesCS6B02G052900 | protein_coding |
| ENSRNA050020772 | snoZ118 | T_aestivum | Plants | intronic_cluster | C/D | TraesCS6B02G052900 | protein_coding |
| ENSRNA050020773 | snoZ118 | T_aestivum | Plants | intronic_cluster | C/D | TraesCS6B02G052900 | protein_coding |
| ENSRNA050020774 | snoZ118 | T_aestivum | Plants | intronic_cluster | C/D | TraesCS6B02G052900 | protein_coding |
| ENSRNA050020775 | snoZ118 | T_aestivum | Plants | intronic_cluster | C/D | TraesCS6B02G052900 | protein_coding |
| ENSRNA050020776 | snoZ118 | T_aestivum | Plants | intronic_cluster | C/D | TraesCS6B02G052900 | protein_coding |
| ENSRNA050020777 | snoZ118 | T_aestivum | Plants | intronic_cluster | C/D | TraesCS6B02G052900 | protein_coding |
| ENSRNA050020781 | snoR1 | T_aestivum | Plants | mono_intergenic | C/D | | intergenic |
| ENSRNA050020782 | snoZ43 | T_aestivum | Plants | intergenic_cluster | C/D | | intergenic |
| ENSRNA050020783 | snoR1 | T_aestivum | Plants | mono_intergenic | C/D | | intergenic |
| ENSRNA050020784 | snosnR60_Z15 | T_aestivum | Plants | intergenic_cluster | C/D | | intergenic |
| ENSRNA050020787 | snoZ223 | T_aestivum | Plants | mono_intergenic | C/D | | intergenic |
| ENSRNA050020788 | snoZ223 | T_aestivum | Plants | mono_intergenic | C/D | | intergenic |
| ENSRNA050020789 | snoZ223 | T_aestivum | Plants | mono_intergenic | C/D | | intergenic |
| ENSRNA050020790 | snoZ223 | T_aestivum | Plants | mono_intergenic | C/D | | intergenic |
| ENSRNA050020791 | snoZ223 | T_aestivum | Plants | mono_intergenic | C/D | | intergenic |
| ENSRNA050020792 | snoZ223 | T_aestivum | Plants | mono_intergenic | C/D | | intergenic |
| ENSRNA050020793 | snoZ223 | T_aestivum | Plants | mono_intergenic | C/D | | intergenic |
| ENSRNA050020794 | snoZ223 | T_aestivum | Plants | mono_intergenic | C/D | | intergenic |
| ENSRNA050020795 | snoZ223 | T_aestivum | Plants | mono_intergenic | C/D | | intergenic |
| ENSRNA050020796 | snoZ223 | T_aestivum | Plants | mono_intergenic | C/D | | intergenic |
| ENSRNA050020815 | SNORD24 | T_aestivum | Plants | mono_intergenic | C/D | | intergenic |
| ENSRNA050020818 | SNORD24 | T_aestivum | Plants | mono_intergenic | C/D | | intergenic |
| ENSRNA050020820 | snoJ33 | T_aestivum | Plants | mono_exonic | C/D | TraesCS3D02G246000 | protein_coding |
| ENSRNA050020847 | SNORD24 | T_aestivum | Plants | mono_intergenic | C/D | | intergenic |
| ENSRNA050020851 | snoU36a | T_aestivum | Plants | intergenic_cluster | C/D | | intergenic |
| ENSRNA050020852 | snoU36a | T_aestivum | Plants | intergenic_cluster | C/D | | intergenic |
| ENSRNA050020853 | snoU36a | T_aestivum | Plants | intergenic_cluster | C/D | | intergenic |
| ENSRNA050020855 | snoR134 | T_aestivum | Plants | intergenic_cluster | H/ACA | | intergenic |
| ENSRNA050020856 | snoZ223 | T_aestivum | Plants | intergenic_cluster | C/D | | intergenic |
| ENSRNA050020857 | snoR80 | T_aestivum | Plants | mono_intergenic | H/ACA | | intergenic |
| ENSRNA050020858 | snoZ223 | T_aestivum | Plants | intergenic_cluster | C/D | | intergenic |
| ENSRNA050020859 | snoZ223 | T_aestivum | Plants | intergenic_cluster | C/D | | intergenic |
| ENSRNA050020860 | snoR134 | T_aestivum | Plants | intergenic_cluster | H/ACA | | intergenic |
| ENSRNA050020863 | snoU36a | T_aestivum | Plants | intergenic_cluster | C/D | | intergenic |
| ENSRNA050020864 | snoZ223 | T_aestivum | Plants | intergenic_cluster | C/D | | intergenic |
| ENSRNA050020865 | snoU36a | T_aestivum | Plants | intergenic_cluster | C/D | | intergenic |
| ENSRNA050020866 | snoU36a | T_aestivum | Plants | intergenic_cluster | C/D | | intergenic |
| ENSRNA050020867 | snoR134 | T_aestivum | Plants | intergenic_cluster | H/ACA | | intergenic |
| ENSRNA050020868 | snoR134 | T_aestivum | Plants | intergenic_cluster | H/ACA | | intergenic |
| ENSRNA050020869 | snoZ223 | T_aestivum | Plants | intergenic_cluster | C/D | | intergenic |
| ENSRNA050020870 | snoZ223 | T_aestivum | Plants | intergenic_cluster | C/D | | intergenic |

| | | | | | | | |
|---|---|---|---|---|---|---|---|
| ENSRNA050020871 | snoR103 | T_aestivum | Plants | intronic_cluster | H/ACA | TraesCS2D02G303000 | protein_coding |
| ENSRNA050020872 | snoR103 | T_aestivum | Plants | intronic_cluster | H/ACA | TraesCS2D02G303000 | protein_coding |
| ENSRNA050020873 | snoR103 | T_aestivum | Plants | intronic_cluster | H/ACA | TraesCS2D02G303000 | protein_coding |
| ENSRNA050020874 | snoR103 | T_aestivum | Plants | intronic_cluster | H/ACA | TraesCS2D02G303000 | protein_coding |
| ENSRNA050020877 | snoZ43 | T_aestivum | Plants | mono_intergenic | C/D | | intergenic |
| ENSRNA050021085 | SNOR75 | T_aestivum | Plants | mono_intergenic | C/D | | intergenic |
| ENSRNA050021089 | snoZ155 | T_aestivum | Plants | intergenic_cluster | C/D | | intergenic |
| ENSRNA050021091 | snoZ155 | T_aestivum | Plants | intergenic_cluster | C/D | | intergenic |
| ENSRNA050021095 | snoR41 | T_aestivum | Plants | intergenic_cluster | C/D | | intergenic |
| ENSRNA050021179 | snoZ279_R105_R108 | T_aestivum | Plants | mono_intergenic | C/D | | intergenic |
| ENSRNA050021187 | SNORD24 | T_aestivum | Plants | mono_intronic | C/D | TraesCS5D02G402800 | protein_coding |
| ENSRNA050021197 | snoR60 | T_aestivum | Plants | mono_intergenic | C/D | | intergenic |
| ENSRNA050021201 | snoR60 | T_aestivum | Plants | mono_intergenic | C/D | | intergenic |
| ENSRNA050021210 | snoR64 | T_aestivum | Plants | intergenic_cluster | C/D | | intergenic |
| ENSRNA050021211 | snoZ199 | T_aestivum | Plants | intergenic_cluster | C/D | | intergenic |
| ENSRNA050021212 | snoZ199 | T_aestivum | Plants | intergenic_cluster | C/D | | intergenic |
| ENSRNA050021216 | SNOR75 | T_aestivum | Plants | intergenic_cluster | C/D | | intergenic |
| ENSRNA050021217 | SNOR75 | T_aestivum | Plants | intergenic_cluster | C/D | | intergenic |
| ENSRNA050021219 | snoZ105 | T_aestivum | Plants | intergenic_cluster | C/D | | intergenic |
| ENSRNA050021437 | snoZ223 | T_aestivum | Plants | mono_intergenic | C/D | | intergenic |
| ENSRNA050021445 | snoR44_J54 | T_aestivum | Plants | intergenic_cluster | C/D | | intergenic |
| ENSRNA050021446 | snoR44_J54 | T_aestivum | Plants | intergenic_cluster | C/D | | intergenic |
| ENSRNA050021447 | SNORD24 | T_aestivum | Plants | mono_intergenic | C/D | | intergenic |
| ENSRNA050021448 | snoZ223 | T_aestivum | Plants | mono_intergenic | C/D | | intergenic |
| ENSRNA050021450 | SNORD24 | T_aestivum | Plants | mono_intergenic | C/D | | intergenic |
| ENSRNA050021451 | snoR60 | T_aestivum | Plants | intronic_cluster | C/D | TraesCS7D02G467000 | protein_coding |
| ENSRNA050021452 | snoR60 | T_aestivum | Plants | intronic_cluster | C/D | TraesCS7D02G467000 | protein_coding |
| ENSRNA050021453 | snoZ43 | T_aestivum | Plants | intergenic_cluster | C/D | | intergenic |
| ENSRNA050021454 | snoU36a | T_aestivum | Plants | intergenic_cluster | C/D | | intergenic |
| ENSRNA050021455 | snoZ101 | T_aestivum | Plants | intergenic_cluster | C/D | | intergenic |
| ENSRNA050021456 | snoZ101 | T_aestivum | Plants | intergenic_cluster | C/D | | intergenic |
| ENSRNA050021457 | snoZ101 | T_aestivum | Plants | intergenic_cluster | C/D | | intergenic |
| ENSRNA050021458 | SNORD25 | T_aestivum | Plants | mono_intergenic | C/D | | intergenic |
| ENSRNA050021459 | SNORD25 | T_aestivum | Plants | intergenic_cluster | C/D | | intergenic |
| ENSRNA050021460 | SNORD25 | T_aestivum | Plants | mono_intergenic | C/D | | intergenic |
| ENSRNA050021461 | snoZ223 | T_aestivum | Plants | intergenic_cluster | C/D | | intergenic |
| ENSRNA050021462 | snoR64 | T_aestivum | Plants | intergenic_cluster | C/D | | intergenic |
| ENSRNA050021463 | snoZ223 | T_aestivum | Plants | intergenic_cluster | C/D | | intergenic |
| ENSRNA050021464 | snoR64 | T_aestivum | Plants | intergenic_cluster | C/D | | intergenic |
| ENSRNA050021465 | snoU36a | T_aestivum | Plants | mono_intergenic | C/D | | intergenic |
| ENSRNA050021466 | snoZ278 | T_aestivum | Plants | intergenic_cluster | C/D | | intergenic |
| ENSRNA050021467 | snosnR60_Z15 | T_aestivum | Plants | intergenic_cluster | C/D | | intergenic |
| ENSRNA050021468 | snoZ223 | T_aestivum | Plants | intergenic_cluster | C/D | | intergenic |

| | | | | | | | |
|---|---|---|---|---|---|---|---|
| ENSRNA050021469 | snoR64 | T_aestivum | Plants | intergenic_cluster | C/D | | intergenic |
| ENSRNA050021470 | snoZ199 | T_aestivum | Plants | intergenic_cluster | C/D | | intergenic |
| ENSRNA050021471 | snoZ199 | T_aestivum | Plants | intergenic_cluster | C/D | | intergenic |
| ENSRNA050021472 | snoZ278 | T_aestivum | Plants | intergenic_cluster | C/D | | intergenic |
| ENSRNA050021473 | snoU31b | T_aestivum | Plants | intergenic_cluster | C/D | | intergenic |
| ENSRNA050021474 | snoZ102_R77 | T_aestivum | Plants | intergenic_cluster | C/D | | intergenic |
| ENSRNA050021475 | snoU36a | T_aestivum | Plants | intergenic_cluster | C/D | | intergenic |
| ENSRNA050021476 | snoR8a | T_aestivum | Plants | intergenic_cluster | C/D | | intergenic |
| ENSRNA050021480 | snoR1 | T_aestivum | Plants | intergenic_cluster | C/D | | intergenic |
| ENSRNA050021481 | snoR1 | T_aestivum | Plants | intergenic_cluster | C/D | | intergenic |
| ENSRNA050021482 | snoZ223 | T_aestivum | Plants | intergenic_cluster | C/D | | intergenic |
| ENSRNA050021483 | SNORD25 | T_aestivum | Plants | intergenic_cluster | C/D | | intergenic |
| ENSRNA050021484 | snoR1 | T_aestivum | Plants | intergenic_cluster | C/D | | intergenic |
| ENSRNA050021485 | snoZ278 | T_aestivum | Plants | intergenic_cluster | C/D | | intergenic |
| ENSRNA050021486 | snoZ159 | T_aestivum | Plants | intergenic_cluster | C/D | | intergenic |
| ENSRNA050021487 | snoR80 | T_aestivum | Plants | intergenic_cluster | H/ACA | | intergenic |
| ENSRNA050021488 | snoR80 | T_aestivum | Plants | intergenic_cluster | H/ACA | | intergenic |
| ENSRNA050021489 | snoZ159 | T_aestivum | Plants | intergenic_cluster | C/D | | intergenic |
| ENSRNA050021490 | snoZ159 | T_aestivum | Plants | intergenic_cluster | C/D | | intergenic |
| ENSRNA050021491 | snoZ199 | T_aestivum | Plants | intergenic_cluster | C/D | | intergenic |
| ENSRNA050021492 | snoU36a | T_aestivum | Plants | intergenic_cluster | C/D | | intergenic |
| ENSRNA050021493 | snoR117 | T_aestivum | Plants | intronic_cluster | C/D | TraesCS6A02G096200 | protein_coding |
| ENSRNA050021494 | SNORD24 | T_aestivum | Plants | intergenic_cluster | C/D | | intergenic |
| ENSRNA050021496 | snoR117 | T_aestivum | Plants | intronic_cluster | C/D | TraesCS6A02G096200 | protein_coding |
| ENSRNA050021497 | snoZ159 | T_aestivum | Plants | intergenic_cluster | C/D | | intergenic |
| ENSRNA050021511 | snoZ223 | T_aestivum | Plants | intronic_cluster | C/D | TraesCS4B02G166800 | protein_coding |
| ENSRNA050021512 | snoR134 | T_aestivum | Plants | intronic_cluster | H/ACA | TraesCS4B02G166800 | protein_coding |
| ENSRNA050021513 | snoZ199 | T_aestivum | Plants | intergenic_cluster | C/D | | intergenic |
| ENSRNA050021514 | snoR64 | T_aestivum | Plants | intergenic_cluster | C/D | | intergenic |
| ENSRNA050021515 | snoZ199 | T_aestivum | Plants | intergenic_cluster | C/D | | intergenic |
| ENSRNA050021523 | snoR60 | T_aestivum | Plants | intronic_cluster | C/D | TraesCS6B02G440500 | protein_coding |
| ENSRNA050021524 | snoR60 | T_aestivum | Plants | intronic_cluster | C/D | TraesCS6B02G440500 | protein_coding |
| ENSRNA050021525 | SNORD25 | T_aestivum | Plants | intronic_cluster | C/D | TraesCS6B02G440500 | protein_coding |
| ENSRNA050021527 | SNOR75 | T_aestivum | Plants | intergenic_cluster | C/D | | intergenic |
| ENSRNA050021528 | SNOR75 | T_aestivum | Plants | intergenic_cluster | C/D | | intergenic |
| ENSRNA050021529 | SNOR75 | T_aestivum | Plants | intergenic_cluster | C/D | | intergenic |
| ENSRNA050021530 | SNOR75 | T_aestivum | Plants | intergenic_cluster | C/D | | intergenic |
| ENSRNA050021531 | SNOR75 | T_aestivum | Plants | intergenic_cluster | C/D | | intergenic |
| ENSRNA050021532 | SNOR75 | T_aestivum | Plants | intronic_cluster | C/D | TraesCS5D02G425600 | protein_coding |
| ENSRNA050021533 | SNOR75 | T_aestivum | Plants | intergenic_cluster | C/D | | intergenic |
| ENSRNA050021534 | SNOR75 | T_aestivum | Plants | intronic_cluster | C/D | TraesCS5D02G425600 | protein_coding |
| ENSRNA050021535 | SNOR75 | T_aestivum | Plants | intergenic_cluster | C/D | | intergenic |
| ENSRNA050021536 | SNOR75 | T_aestivum | Plants | intergenic_cluster | C/D | | intergenic |

| | | | | | | | |
|---|---|---|---|---|---|---|---|
| ENSRNA050021537 | SNOR75 | T_aestivum | Plants | intergenic_cluster | C/D | | intergenic |
| ENSRNA050021538 | SNOR75 | T_aestivum | Plants | intergenic_cluster | C/D | | intergenic |
| ENSRNA050021539 | snoZ221_snoR21b | T_aestivum | Plants | intergenic_cluster | C/D | | intergenic |
| ENSRNA050021540 | snoZ221_snoR21b | T_aestivum | Plants | intergenic_cluster | C/D | | intergenic |
| ENSRNA050021541 | snoZ105 | T_aestivum | Plants | intergenic_cluster | C/D | | intergenic |
| ENSRNA050021542 | snoZ105 | T_aestivum | Plants | intergenic_cluster | C/D | | intergenic |
| ENSRNA050021544 | snoZ105 | T_aestivum | Plants | intergenic_cluster | C/D | | intergenic |
| ENSRNA050021545 | snoZ105 | T_aestivum | Plants | intergenic_cluster | C/D | | intergenic |
| ENSRNA050021546 | snoZ221_snoR21b | T_aestivum | Plants | intergenic_cluster | C/D | | intergenic |
| ENSRNA050021552 | SNORD24 | T_aestivum | Plants | mono_intergenic | C/D | | intergenic |
| ENSRNA050021553 | snoZ118 | T_aestivum | Plants | mono_intergenic | C/D | | intergenic |
| ENSRNA050021563 | SNORD24 | T_aestivum | Plants | mono_intergenic | C/D | | intergenic |
| ENSRNA050021601 | snoZ43 | T_aestivum | Plants | intergenic_cluster | C/D | | intergenic |
| ENSRNA050021602 | snoR135 | T_aestivum | Plants | mono_intergenic | H/ACA | | intergenic |
| ENSRNA050021604 | snoU31b | T_aestivum | Plants | mono_intergenic | C/D | | intergenic |
| ENSRNA050021605 | snoR77Y | T_aestivum | Plants | mono_intergenic | C/D | | intergenic |
| ENSRNA050021606 | snoR77Y | T_aestivum | Plants | mono_intergenic | C/D | | intergenic |
| ENSRNA050021607 | snoU31b | T_aestivum | Plants | mono_intergenic | C/D | | intergenic |
| ENSRNA050021608 | snosnR60_Z15 | T_aestivum | Plants | mono_intergenic | C/D | | intergenic |
| ENSRNA050021609 | snosnR60_Z15 | T_aestivum | Plants | mono_intergenic | C/D | | intergenic |
| ENSRNA050021610 | snoR77Y | T_aestivum | Plants | mono_intergenic | C/D | | intergenic |
| ENSRNA050021611 | snoR8a | T_aestivum | Plants | mono_intergenic | C/D | | intergenic |
| ENSRNA050021612 | snosnR60_Z15 | T_aestivum | Plants | mono_intergenic | C/D | | intergenic |
| ENSRNA050021613 | snosnR60_Z15 | T_aestivum | Plants | intergenic_cluster | C/D | | intergenic |
| ENSRNA050021626 | snoR44_J54 | T_aestivum | Plants | intergenic_cluster | C/D | | intergenic |
| ENSRNA050021627 | snoR44_J54 | T_aestivum | Plants | intergenic_cluster | C/D | | intergenic |
| ENSRNA050021633 | snoR135 | T_aestivum | Plants | mono_intergenic | H/ACA | | intergenic |
| ENSRNA050021634 | snoU31b | T_aestivum | Plants | intergenic_cluster | C/D | | intergenic |
| ENSRNA050021636 | snoR117 | T_aestivum | Plants | intergenic_cluster | C/D | | intergenic |
| ENSRNA050021643 | snoZ223 | T_aestivum | Plants | mono_intergenic | C/D | | intergenic |
| ENSRNA050021647 | snoZ102_R77 | T_aestivum | Plants | mono_intronic | C/D | TraesCS5B02G448100 | protein_coding |
| ENSRNA050021658 | snoZ118 | T_aestivum | Plants | mono_intergenic | C/D | | intergenic |
| ENSRNA050021665 | snoJ33 | T_aestivum | Plants | mono_exonic | C/D | TraesCS3B02G327500 | protein_coding |
| ENSRNA050021669 | SNORD24 | T_aestivum | Plants | mono_intergenic | C/D | | intergenic |
| ENSRNA050021710 | snoZ223 | T_aestivum | Plants | intergenic_cluster | C/D | | intergenic |
| ENSRNA050021711 | snoZ278 | T_aestivum | Plants | intergenic_cluster | C/D | | intergenic |
| ENSRNA050021749 | snoZ159 | T_aestivum | Plants | intergenic_cluster | C/D | | intergenic |
| ENSRNA050021750 | snoZ159 | T_aestivum | Plants | intergenic_cluster | C/D | | intergenic |
| ENSRNA050021755 | SNOR75 | T_aestivum | Plants | intergenic_cluster | C/D | | intergenic |
| ENSRNA050021756 | SNOR75 | T_aestivum | Plants | intergenic_cluster | C/D | | intergenic |
| ENSRNA050021768 | snoZ101 | T_aestivum | Plants | mono_intergenic | C/D | | intergenic |
| ENSRNA050021769 | snoZ101 | T_aestivum | Plants | mono_intergenic | C/D | | intergenic |
| ENSRNA050021770 | snoR83 | T_aestivum | Plants | mono_intergenic | H/ACA | | intergenic |

| | | | | | | | |
|---|---|---|---|---|---|---|---|
| ENSRNA050022693 | snoR41 | T_aestivum | Plants | intergenic_cluster | C/D | | intergenic |
| ENSRNA050022694 | snoZ155 | T_aestivum | Plants | intergenic_cluster | C/D | | intergenic |
| ENSRNA050022695 | snoJ33 | T_aestivum | Plants | mono_exonic | C/D | TraesCS3D02G292500 | protein_coding |
| ENSRNA050022703 | snoR135 | T_aestivum | Plants | mono_intergenic | H/ACA | | intergenic |
| ENSRNA050022704 | snoZ43 | T_aestivum | Plants | intergenic_cluster | C/D | | intergenic |
| ENSRNA050022705 | snoR77 | T_aestivum | Plants | intergenic_cluster | H/ACA | | intergenic |
| ENSRNA050022706 | snoR77 | T_aestivum | Plants | intergenic_cluster | H/ACA | | intergenic |
| ENSRNA050022707 | snoR77 | T_aestivum | Plants | intergenic_cluster | H/ACA | | intergenic |
| ENSRNA050022709 | snoR44_J54 | T_aestivum | Plants | intergenic_cluster | C/D | | intergenic |
| ENSRNA050022710 | snoU31b | T_aestivum | Plants | mono_intergenic | C/D | | intergenic |
| ENSRNA050022711 | snosnR60_Z15 | T_aestivum | Plants | mono_intergenic | C/D | | intergenic |
| ENSRNA050022712 | snoZ43 | T_aestivum | Plants | intergenic_cluster | C/D | | intergenic |
| ENSRNA050022713 | snoZ159 | T_aestivum | Plants | mono_intergenic | C/D | | intergenic |
| ENSRNA050022714 | snoR44_J54 | T_aestivum | Plants | intergenic_cluster | C/D | | intergenic |
| ENSRNA050022715 | SNOR75 | T_aestivum | Plants | mono_intergenic | C/D | | intergenic |
| ENSRNA050022716 | snoU36a | T_aestivum | Plants | intergenic_cluster | C/D | | intergenic |
| ENSRNA050022717 | snoU36a | T_aestivum | Plants | intergenic_cluster | C/D | | intergenic |
| ENSRNA050022718 | snoZ223 | T_aestivum | Plants | intergenic_cluster | C/D | | intergenic |
| ENSRNA050022719 | snoZ223 | T_aestivum | Plants | intergenic_cluster | C/D | | intergenic |
| ENSRNA050022721 | snoZ199 | T_aestivum | Plants | intronic_cluster | C/D | TraesCS6B02G041500 | protein_coding |
| ENSRNA050022722 | snoR64 | T_aestivum | Plants | intronic_cluster | C/D | TraesCS6B02G041500 | protein_coding |
| ENSRNA050022723 | snoZ199 | T_aestivum | Plants | intronic_cluster | C/D | TraesCS6B02G041500 | protein_coding |
| ENSRNA050022727 | snoZ188 | T_aestivum | Plants | intergenic_cluster | C/D | | intergenic |
| ENSRNA050022728 | snoZ188 | T_aestivum | Plants | intergenic_cluster | C/D | | intergenic |
| ENSRNA050022733 | snoZ43 | T_aestivum | Plants | mono_intergenic | C/D | | intergenic |
| ENSRNA050022734 | snoR135 | T_aestivum | Plants | intergenic_cluster | H/ACA | | intergenic |
| ENSRNA050022735 | snoR135 | T_aestivum | Plants | intergenic_cluster | H/ACA | | intergenic |
| ENSRNA050022737 | snoR135 | T_aestivum | Plants | intergenic_cluster | H/ACA | | intergenic |
| ENSRNA050022739 | U54 | T_aestivum | Plants | mono_intronic | C/D | TraesCS4B02G164300 | protein_coding |
| ENSRNA050022740 | U54 | T_aestivum | Plants | mono_intronic | C/D | TraesCS4B02G164300 | protein_coding |
| ENSRNA050022753 | snoZ221_snoR21b | T_aestivum | Plants | intronic_cluster | C/D | TraesCS2D02G531800 | protein_coding |
| ENSRNA050022754 | snoZ221_snoR21b | T_aestivum | Plants | intronic_cluster | C/D | TraesCS2D02G531800 | protein_coding |
| ENSRNA050022755 | snoZ221_snoR21b | T_aestivum | Plants | intronic_cluster | C/D | TraesCS2D02G531800 | protein_coding |
| ENSRNA050022773 | snoR80 | T_aestivum | Plants | mono_intergenic | H/ACA | | intergenic |
| ENSRNA050022777 | snoZ43 | T_aestivum | Plants | intergenic_cluster | C/D | | intergenic |
| ENSRNA050022778 | snoU31b | T_aestivum | Plants | intergenic_cluster | C/D | | intergenic |
| ENSRNA050022779 | snoR77Y | T_aestivum | Plants | mono_intergenic | C/D | | intergenic |
| ENSRNA050022781 | snoZ43 | T_aestivum | Plants | mono_intergenic | C/D | | intergenic |
| ENSRNA050022782 | SNORD24 | T_aestivum | Plants | mono_intergenic | C/D | | intergenic |
| ENSRNA050022788 | snoZ118 | T_aestivum | Plants | intergenic_cluster | C/D | | intergenic |
| ENSRNA050022789 | snoZ118 | T_aestivum | Plants | intergenic_cluster | C/D | | intergenic |
| ENSRNA050022804 | snoZ159 | T_aestivum | Plants | intergenic_cluster | C/D | | intergenic |
| ENSRNA050022805 | snoZ159 | T_aestivum | Plants | intergenic_cluster | C/D | | intergenic |

| | | | | | | | |
|---|---|---|---|---|---|---|---|
| ENSRNA050022807 | snoR80 | T_aestivum | Plants | mono_intergenic | H/ACA | | intergenic |
| ENSRNA050022808 | SNORD24 | T_aestivum | Plants | mono_intergenic | C/D | | intergenic |
| ENSRNA050022813 | snoZ199 | T_aestivum | Plants | mono_intergenic | C/D | | intergenic |
| ENSRNA050022814 | snoJ33 | T_aestivum | Plants | mono_exonic | C/D | TraesCS3B02G274100 | protein_coding |
| ENSRNA050022816 | snoR28 | T_aestivum | Plants | mono_exonic | C/D | TraesCS7D02G153500 | protein_coding |
| ENSRNA050022822 | snoR64 | T_aestivum | Plants | intergenic_cluster | C/D | | intergenic |
| ENSRNA050022823 | snoZ199 | T_aestivum | Plants | intergenic_cluster | C/D | | intergenic |
| ENSRNA050022824 | snoZ199 | T_aestivum | Plants | intergenic_cluster | C/D | | intergenic |
| ENSRNA050022834 | SNOR75 | T_aestivum | Plants | intergenic_cluster | C/D | | intergenic |
| ENSRNA050022835 | SNOR75 | T_aestivum | Plants | intergenic_cluster | C/D | | intergenic |
| ENSRNA050022836 | SNOR75 | T_aestivum | Plants | intergenic_cluster | C/D | | intergenic |
| ENSRNA050022837 | SNOR75 | T_aestivum | Plants | intergenic_cluster | C/D | | intergenic |
| ENSRNA050022838 | SNOR75 | T_aestivum | Plants | intergenic_cluster | C/D | | intergenic |
| ENSRNA050022839 | SNOR75 | T_aestivum | Plants | intergenic_cluster | C/D | | intergenic |
| ENSRNA050022841 | SNOR75 | T_aestivum | Plants | intergenic_cluster | C/D | | intergenic |
| ENSRNA050022842 | SNOR75 | T_aestivum | Plants | intergenic_cluster | C/D | | intergenic |
| ENSRNA050022843 | SNOR75 | T_aestivum | Plants | intergenic_cluster | C/D | | intergenic |
| ENSRNA050022844 | SNOR75 | T_aestivum | Plants | intergenic_cluster | C/D | | intergenic |
| ENSRNA050022845 | SNOR75 | T_aestivum | Plants | intergenic_cluster | C/D | | intergenic |
| ENSRNA050022846 | SNOR75 | T_aestivum | Plants | intergenic_cluster | C/D | | intergenic |
| ENSRNA050022847 | SNOR75 | T_aestivum | Plants | intergenic_cluster | C/D | | intergenic |
| ENSRNA050022848 | snoZ221_snoR21b | T_aestivum | Plants | mono_intergenic | C/D | | intergenic |
| ENSRNA050022849 | SNOR75 | T_aestivum | Plants | intergenic_cluster | C/D | | intergenic |
| ENSRNA050022850 | SNOR75 | T_aestivum | Plants | intergenic_cluster | C/D | | intergenic |
| ENSRNA050022851 | SNOR75 | T_aestivum | Plants | intergenic_cluster | C/D | | intergenic |
| ENSRNA050022853 | snoZ105 | T_aestivum | Plants | intergenic_cluster | C/D | | intergenic |
| ENSRNA050022854 | snoZ105 | T_aestivum | Plants | intergenic_cluster | C/D | | intergenic |
| ENSRNA050022855 | SNOR75 | T_aestivum | Plants | intergenic_cluster | C/D | | intergenic |
| ENSRNA050022856 | snoZ105 | T_aestivum | Plants | intergenic_cluster | C/D | | intergenic |
| ENSRNA050022857 | snoZ105 | T_aestivum | Plants | intergenic_cluster | C/D | | intergenic |
| ENSRNA050022858 | SNOR75 | T_aestivum | Plants | mono_intergenic | C/D | | intergenic |
| ENSRNA050022863 | SNOR75 | T_aestivum | Plants | mono_intergenic | C/D | | intergenic |
| ENSRNA050022865 | SNOR75 | T_aestivum | Plants | mono_intergenic | C/D | | intergenic |
| ENSRNA050022866 | snoZ155 | T_aestivum | Plants | intergenic_cluster | C/D | | intergenic |
| ENSRNA050022867 | snoZ155 | T_aestivum | Plants | intergenic_cluster | C/D | | intergenic |
| ENSRNA050022873 | snoR1 | T_aestivum | Plants | mono_intergenic | C/D | | intergenic |
| ENSRNA050022874 | snoR101 | T_aestivum | Plants | intronic_cluster | C/D | TraesCS3A02G256900 | protein_coding |
| ENSRNA050022875 | snoR101 | T_aestivum | Plants | intronic_cluster | C/D | TraesCS3A02G256900 | protein_coding |
| ENSRNA050022876 | snoR101 | T_aestivum | Plants | intergenic_cluster | C/D | | intergenic |
| ENSRNA050022877 | snoR101 | T_aestivum | Plants | intergenic_cluster | C/D | | intergenic |
| ENSRNA050022879 | snoZ102_R77 | T_aestivum | Plants | mono_intronic | C/D | TraesCS5A02G443700 | protein_coding |
| ENSRNA050022900 | snoR80 | T_aestivum | Plants | mono_intergenic | H/ACA | | intergenic |
| ENSRNA050022903 | snoR103 | T_aestivum | Plants | intronic_cluster | H/ACA | TraesCS2A02G304400 | protein_coding |

| | | | | | | | |
|---|---|---|---|---|---|---|---|
| ENSRNA050022904 | snoR103 | T_aestivum | Plants | intronic_cluster | H/ACA | TraesCS2A02G304400 | protein_coding |
| ENSRNA050022905 | snoR103 | T_aestivum | Plants | intronic_cluster | H/ACA | TraesCS2A02G304400 | protein_coding |
| ENSRNA050022911 | snoR83 | T_aestivum | Plants | mono_intergenic | H/ACA | | intergenic |
| ENSRNA050022912 | snoR11 | T_aestivum | Plants | intergenic_cluster | C/D | | intergenic |
| ENSRNA050022913 | snoZ152 | T_aestivum | Plants | intergenic_cluster | C/D | | intergenic |
| ENSRNA050022914 | SNORD24 | T_aestivum | Plants | mono_intergenic | C/D | | intergenic |
| ENSRNA050022915 | SNORD24 | T_aestivum | Plants | mono_intergenic | C/D | | intergenic |
| ENSRNA050022919 | SNORD25 | T_aestivum | Plants | mono_intergenic | C/D | | intergenic |
| ENSRNA050022924 | snoZ101 | T_aestivum | Plants | mono_intergenic | C/D | | intergenic |
| ENSRNA050022925 | SNORD24 | T_aestivum | Plants | mono_intergenic | C/D | | intergenic |
| ENSRNA050022931 | snoR101 | T_aestivum | Plants | mono_intergenic | C/D | | intergenic |
| ENSRNA050022932 | snoZ223 | T_aestivum | Plants | mono_intergenic | C/D | | intergenic |
| ENSRNA050022933 | snoZ223 | T_aestivum | Plants | mono_intergenic | C/D | | intergenic |
| ENSRNA050022934 | snoZ223 | T_aestivum | Plants | mono_intergenic | C/D | | intergenic |
| ENSRNA050022935 | snoZ223 | T_aestivum | Plants | mono_intergenic | C/D | | intergenic |
| ENSRNA050022946 | snoZ159 | T_aestivum | Plants | intergenic_cluster | C/D | | intergenic |
| ENSRNA050022947 | snoR80 | T_aestivum | Plants | intergenic_cluster | H/ACA | | intergenic |
| ENSRNA050022948 | snoZ159 | T_aestivum | Plants | intergenic_cluster | C/D | | intergenic |
| ENSRNA050022950 | snoZ159 | T_aestivum | Plants | intergenic_cluster | C/D | | intergenic |
| ENSRNA050022951 | snoR77Y | T_aestivum | Plants | mono_intergenic | C/D | | intergenic |
| ENSRNA050022952 | snoZ159 | T_aestivum | Plants | intergenic_cluster | C/D | | intergenic |
| ENSRNA050022953 | snoZ102_R77 | T_aestivum | Plants | mono_intronic | C/D | TraesCS5D02G451100 | protein_coding |
| ENSRNA050022954 | snoR101 | T_aestivum | Plants | intronic_cluster | C/D | TraesCS3D02G257000 | protein_coding |
| ENSRNA050022955 | snoR101 | T_aestivum | Plants | intronic_cluster | C/D | TraesCS3D02G257000 | protein_coding |
| ENSRNA050022966 | snoZ43 | T_aestivum | Plants | intronic_cluster | C/D | TraesCS2B02G198400 | protein_coding |
| ENSRNA050022967 | snoR135 | T_aestivum | Plants | intronic_cluster | H/ACA | TraesCS2B02G198400 | protein_coding |
| ENSRNA050022968 | snosnR60_Z15 | T_aestivum | Plants | intronic_cluster | C/D | TraesCS2B02G198400 | protein_coding |
| ENSRNA050022978 | SNORD24 | T_aestivum | Plants | mono_intergenic | C/D | | intergenic |
| ENSRNA050022982 | snoR11 | T_aestivum | Plants | intronic_cluster | C/D | TraesCS1B02G230300 | protein_coding |
| ENSRNA050022983 | snoZ152 | T_aestivum | Plants | intronic_cluster | C/D | TraesCS1B02G230300 | protein_coding |
| ENSRNA050022993 | SNORD24 | T_aestivum | Plants | mono_intergenic | C/D | | intergenic |
| ENSRNA050023013 | snoZ101 | T_aestivum | Plants | intergenic_cluster | C/D | | intergenic |
| ENSRNA050023014 | snoZ101 | T_aestivum | Plants | intergenic_cluster | C/D | | intergenic |
| ENSRNA050023015 | snoZ101 | T_aestivum | Plants | intergenic_cluster | C/D | | intergenic |
| ENSRNA050023016 | snoZ199 | T_aestivum | Plants | intergenic_cluster | C/D | | intergenic |
| ENSRNA050023019 | snoR64 | T_aestivum | Plants | intergenic_cluster | C/D | | intergenic |
| ENSRNA050023021 | snoR80 | T_aestivum | Plants | mono_intergenic | H/ACA | | intergenic |
| ENSRNA050023022 | snoZ101 | T_aestivum | Plants | intergenic_cluster | C/D | | intergenic |
| ENSRNA050023023 | snoR8a | T_aestivum | Plants | intergenic_cluster | C/D | | intergenic |
| ENSRNA050023025 | snoR80 | T_aestivum | Plants | mono_intergenic | H/ACA | | intergenic |
| ENSRNA050023408 | SNORD25 | T_aestivum | Plants | intronic_cluster | C/D | TraesCS2B02G381100 | protein_coding |
| ENSRNA050023410 | SNORD25 | T_aestivum | Plants | intronic_cluster | C/D | TraesCS2B02G381200 | protein_coding |
| ENSRNA050023412 | SNORD25 | T_aestivum | Plants | intronic_cluster | C/D | TraesCS2B02G381100 | protein_coding |

| | | | | | | | |
|---|---|---|---|---|---|---|---|
| ENSRNA050023415 | SNORD25 | T_aestivum | Plants | intronic_cluster | C/D | TraesCS2B02G381200 | protein_coding |
| ENSRNA050023417 | snoU31b | T_aestivum | Plants | intronic_cluster | C/D | TraesCS2B02G381200 | protein_coding |
| ENSRNA050023419 | snoU31b | T_aestivum | Plants | intronic_cluster | C/D | TraesCS2B02G381200 | protein_coding |
| ENSRNA050023421 | snoU31b | T_aestivum | Plants | intronic_cluster | C/D | TraesCS2B02G381100 | protein_coding |
| ENSRNA050023423 | snosnR60_Z15 | T_aestivum | Plants | intronic_cluster | C/D | TraesCS2B02G381100 | protein_coding |
| ENSRNA050023425 | snosnR60_Z15 | T_aestivum | Plants | intronic_cluster | C/D | TraesCS2B02G381200 | protein_coding |
| ENSRNA050023430 | SNORD25 | T_aestivum | Plants | intronic_cluster | C/D | TraesCS2B02G381200 | protein_coding |
| ENSRNA050023547 | snoR31_Z110_Z27 | T_aestivum | Plants | mono_intergenic | C/D | | intergenic |
| ENSRNA050023548 | snoR31_Z110_Z27 | T_aestivum | Plants | mono_intergenic | C/D | | intergenic |
| ENSRNA050023549 | snoR31_Z110_Z27 | T_aestivum | Plants | mono_intergenic | C/D | | intergenic |
| ENSRNA050023552 | snoZ119 | T_aestivum | Plants | intronic_cluster | C/D | TraesCS3D02G337200 | protein_coding |
| ENSRNA050023553 | snoZ119 | T_aestivum | Plants | intronic_cluster | C/D | TraesCS3D02G337200 | protein_coding |
| ENSRNA050023554 | snoZ119 | T_aestivum | Plants | intronic_cluster | C/D | TraesCS3D02G337200 | protein_coding |
| ENSRNA050023555 | snoZ119 | T_aestivum | Plants | intronic_cluster | C/D | TraesCS3D02G337200 | protein_coding |
| ENSRNA050023556 | snoZ122 | T_aestivum | Plants | intronic_cluster | C/D | TraesCS3D02G337200 | protein_coding |
| ENSRNA050023557 | snoZ119 | T_aestivum | Plants | intronic_cluster | Unknown | TraesCS3D02G337200 | protein_coding |
| ENSRNA050023566 | snoR135 | T_aestivum | Plants | mono_intergenic | H/ACA | | intergenic |
| ENSRNA050023567 | snoR64 | T_aestivum | Plants | intergenic_cluster | C/D | | intergenic |
| ENSRNA050023568 | snoZ199 | T_aestivum | Plants | intergenic_cluster | C/D | | intergenic |
| ENSRNA050023574 | snoZ152 | T_aestivum | Plants | intergenic_cluster | C/D | | intergenic |
| ENSRNA050023575 | snoR11 | T_aestivum | Plants | intergenic_cluster | C/D | | intergenic |
| ENSRNA050023576 | snoR11 | T_aestivum | Plants | intergenic_cluster | C/D | | intergenic |
| ENSRNA050023577 | snoZ152 | T_aestivum | Plants | intergenic_cluster | C/D | | intergenic |
| ENSRNA050023579 | SNORD25 | T_aestivum | Plants | mono_intergenic | C/D | | intergenic |
| ENSRNA050023581 | U54 | T_aestivum | Plants | intronic_cluster | C/D | TraesCS4A02G166500 | protein_coding |
| ENSRNA050023582 | U54 | T_aestivum | Plants | intronic_cluster | C/D | TraesCS4A02G166500 | protein_coding |
| ENSRNA050023583 | U54 | T_aestivum | Plants | intronic_cluster | C/D | TraesCS4A02G166500 | protein_coding |
| ENSRNA050023584 | U54 | T_aestivum | Plants | intronic_cluster | C/D | TraesCS4A02G166500 | protein_coding |
| ENSRNA050023592 | snoZ119 | T_aestivum | Plants | intronic_cluster | C/D | TraesCS3B02G375500 | protein_coding |
| ENSRNA050023593 | snoZ119 | T_aestivum | Plants | intronic_cluster | C/D | TraesCS3B02G375500 | protein_coding |
| ENSRNA050023594 | snoZ119 | T_aestivum | Plants | intronic_cluster | C/D | TraesCS3B02G375500 | protein_coding |
| ENSRNA050023595 | snoZ119 | T_aestivum | Plants | intronic_cluster | C/D | TraesCS3B02G375500 | protein_coding |
| ENSRNA050023596 | snoZ119 | T_aestivum | Plants | intronic_cluster | C/D | TraesCS3B02G375500 | protein_coding |
| ENSRNA050023597 | snoZ122 | T_aestivum | Plants | intronic_cluster | C/D | TraesCS3B02G375500 | protein_coding |
| ENSRNA050023604 | SNORD24 | T_aestivum | Plants | mono_intronic | C/D | TraesCS5B02G397800 | protein_coding |
| ENSRNA050023610 | SNOR75 | T_aestivum | Plants | intergenic_cluster | C/D | | intergenic |
| ENSRNA050023611 | snoR77 | T_aestivum | Plants | intergenic_cluster | H/ACA | | intergenic |
| ENSRNA050023612 | snoR77 | T_aestivum | Plants | intergenic_cluster | H/ACA | | intergenic |
| ENSRNA050023613 | snoR44_J54 | T_aestivum | Plants | intergenic_cluster | C/D | | intergenic |
| ENSRNA050023614 | snoZ159 | T_aestivum | Plants | mono_intergenic | C/D | | intergenic |
| ENSRNA050023615 | snoR44_J54 | T_aestivum | Plants | mono_intergenic | C/D | | intergenic |
| ENSRNA050023617 | SNOR75 | T_aestivum | Plants | intergenic_cluster | C/D | | intergenic |
| ENSRNA050023618 | snoZ155 | T_aestivum | Plants | mono_intergenic | C/D | | intergenic |

| | | | | | | | |
|---|---|---|---|---|---|---|---|
| ENSRNA050023620 | snoZ152 | T_aestivum | Plants | intergenic_cluster | C/D | | intergenic |
| ENSRNA050023621 | snoR11 | T_aestivum | Plants | intergenic_cluster | C/D | | intergenic |
| ENSRNA050023623 | snoZ155 | T_aestivum | Plants | mono_intergenic | C/D | | intergenic |
| ENSRNA050023624 | snoZ155 | T_aestivum | Plants | mono_intergenic | C/D | | intergenic |
| ENSRNA050023632 | snoR44_J54 | T_aestivum | Plants | intergenic_cluster | C/D | | intergenic |
| ENSRNA050023633 | snoR44_J54 | T_aestivum | Plants | intergenic_cluster | C/D | | intergenic |
| ENSRNA050023638 | SNORD24 | T_aestivum | Plants | mono_intergenic | C/D | | intergenic |
| ENSRNA050023643 | snoR80 | T_aestivum | Plants | mono_intergenic | H/ACA | | intergenic |
| ENSRNA050023645 | snoZ199 | T_aestivum | Plants | intergenic_cluster | C/D | | intergenic |
| ENSRNA050023646 | snoR64 | T_aestivum | Plants | intergenic_cluster | C/D | | intergenic |
| ENSRNA050023648 | snoR64 | T_aestivum | Plants | intergenic_cluster | C/D | | intergenic |
| ENSRNA050023649 | snoZ199 | T_aestivum | Plants | intergenic_cluster | C/D | | intergenic |
| ENSRNA050023650 | snoR135 | T_aestivum | Plants | mono_intergenic | H/ACA | | intergenic |
| ENSRNA050023651 | snoR64 | T_aestivum | Plants | intergenic_cluster | C/D | | intergenic |
| ENSRNA050023652 | snoZ199 | T_aestivum | Plants | intergenic_cluster | C/D | | intergenic |
| ENSRNA050023662 | snoR1 | T_aestivum | Plants | mono_intergenic | C/D | | intergenic |
| ENSRNA050023695 | SNORD24 | T_aestivum | Plants | mono_intergenic | C/D | | intergenic |
| ENSRNA050023698 | snoU36a | T_aestivum | Plants | intergenic_cluster | C/D | | intergenic |
| ENSRNA050023699 | snoZ223 | T_aestivum | Plants | intergenic_cluster | C/D | | intergenic |
| ENSRNA050023714 | SNOR75 | T_aestivum | Plants | intergenic_cluster | C/D | | intergenic |
| ENSRNA050023716 | SNOR75 | T_aestivum | Plants | intergenic_cluster | C/D | | intergenic |
| ENSRNA050023718 | snoZ105 | T_aestivum | Plants | intergenic_cluster | C/D | | intergenic |
| ENSRNA050023740 | SNORD24 | T_aestivum | Plants | intronic_cluster | C/D | TraesCS2A02G260700 | protein_coding |
| ENSRNA050023741 | SNORD24 | T_aestivum | Plants | intronic_cluster | C/D | TraesCS2A02G260700 | protein_coding |
| ENSRNA050023742 | SNORD24 | T_aestivum | Plants | intronic_cluster | C/D | TraesCS2A02G260700 | protein_coding |
| ENSRNA050023774 | snoJ33 | T_aestivum | Plants | mono_exonic | C/D | TraesCS3A02G245500 | protein_coding |
| ENSRNA050023776 | snoR31_Z110_Z27 | T_aestivum | Plants | mono_intergenic | C/D | | intergenic |
| ENSRNA050023778 | SNOR75 | T_aestivum | Plants | mono_intergenic | C/D | | intergenic |
| ENSRNA050023779 | snoZ188 | T_aestivum | Plants | mono_intergenic | C/D | | intergenic |
| ENSRNA050023780 | snoR77Y | T_aestivum | Plants | intergenic_cluster | C/D | | intergenic |
| ENSRNA050023782 | snoR31_Z110_Z27 | T_aestivum | Plants | intergenic_cluster | C/D | | intergenic |
| ENSRNA050023799 | SNOR75 | T_aestivum | Plants | mono_intergenic | C/D | | intergenic |
| ENSRNA050023805 | snoR80 | T_aestivum | Plants | mono_intergenic | H/ACA | | intergenic |
| ENSRNA050023808 | snoR80 | T_aestivum | Plants | mono_intergenic | H/ACA | | intergenic |
| ENSRNA050023810 | SNORD24 | T_aestivum | Plants | intronic_cluster | C/D | TraesCS2B02G281300 | protein_coding |
| ENSRNA050023811 | SNORD24 | T_aestivum | Plants | intronic_cluster | C/D | TraesCS2B02G281300 | protein_coding |
| ENSRNA050023812 | SNORD24 | T_aestivum | Plants | intronic_cluster | C/D | TraesCS2B02G281300 | protein_coding |
| ENSRNA050023813 | SNORD24 | T_aestivum | Plants | intronic_cluster | C/D | TraesCS2B02G281300 | protein_coding |
| ENSRNA050023835 | snoZ188 | T_aestivum | Plants | intergenic_cluster | C/D | | intergenic |
| ENSRNA050023836 | snoZ188 | T_aestivum | Plants | intergenic_cluster | C/D | | intergenic |
| ENSRNA050023841 | SNORD24 | T_aestivum | Plants | mono_intergenic | C/D | | intergenic |
| ENSRNA050023846 | snoZ101 | T_aestivum | Plants | mono_intergenic | C/D | | intergenic |
| ENSRNA050023852 | snoR83 | T_aestivum | Plants | mono_intergenic | H/ACA | | intergenic |

| | | | | | | | |
|---|---|---|---|---|---|---|---|
| ENSRNA050023854 | snoR11 | T_aestivum | Plants | intergenic_cluster | C/D | | intergenic |
| ENSRNA050023855 | snoZ152 | T_aestivum | Plants | intergenic_cluster | C/D | | intergenic |
| ENSRNA050023871 | snoU36a | T_aestivum | Plants | intergenic_cluster | C/D | | intergenic |
| ENSRNA050023872 | snoZ223 | T_aestivum | Plants | intergenic_cluster | C/D | | intergenic |
| ENSRNA050023873 | snoZ159 | T_aestivum | Plants | intergenic_cluster | C/D | | intergenic |
| ENSRNA050023874 | snoR31_Z110_Z27 | T_aestivum | Plants | mono_intergenic | C/D | | intergenic |
| ENSRNA050023889 | snoR64 | T_aestivum | Plants | intergenic_cluster | C/D | | intergenic |
| ENSRNA050023890 | snoZ199 | T_aestivum | Plants | intergenic_cluster | C/D | | intergenic |
| ENSRNA050023892 | snoZ279_R105_R108 | T_aestivum | Plants | mono_intergenic | C/D | | intergenic |
| ENSRNA050023895 | SNOR75 | T_aestivum | Plants | intergenic_cluster | C/D | | intergenic |
| ENSRNA050023896 | SNOR75 | T_aestivum | Plants | intergenic_cluster | C/D | | intergenic |
| ENSRNA050023897 | SNOR75 | T_aestivum | Plants | intergenic_cluster | C/D | | intergenic |
| ENSRNA050023899 | SNOR75 | T_aestivum | Plants | intronic_cluster | C/D | TraesCS5A02G417600 | protein_coding |
| ENSRNA050023900 | snoZ118 | T_aestivum | Plants | mono_intergenic | C/D | | intergenic |
| ENSRNA050023901 | SNOR75 | T_aestivum | Plants | intergenic_cluster | C/D | | intergenic |
| ENSRNA050023902 | SNOR75 | T_aestivum | Plants | intronic_cluster | C/D | TraesCS5A02G417600 | protein_coding |
| ENSRNA050023903 | snoU36a | T_aestivum | Plants | intergenic_cluster | C/D | | intergenic |
| ENSRNA050023904 | SNOR75 | T_aestivum | Plants | intergenic_cluster | C/D | | intergenic |
| ENSRNA050023905 | SNOR75 | T_aestivum | Plants | intergenic_cluster | C/D | | intergenic |
| ENSRNA050023906 | snoZ221_snoR21b | T_aestivum | Plants | intergenic_cluster | C/D | | intergenic |
| ENSRNA050023907 | snoZ221_snoR21b | T_aestivum | Plants | intergenic_cluster | C/D | | intergenic |
| ENSRNA050023908 | snoZ221_snoR21b | T_aestivum | Plants | intergenic_cluster | C/D | | intergenic |
| ENSRNA050023909 | snoZ223 | T_aestivum | Plants | intergenic_cluster | C/D | | intergenic |
| ENSRNA050023910 | SNOR75 | T_aestivum | Plants | intergenic_cluster | C/D | | intergenic |
| ENSRNA050023911 | snoZ105 | T_aestivum | Plants | intronic_cluster | C/D | TraesCS5A02G417600 | protein_coding |
| ENSRNA050023912 | snoZ105 | T_aestivum | Plants | intergenic_cluster | C/D | | intergenic |
| ENSRNA050023913 | snoZ105 | T_aestivum | Plants | intergenic_cluster | C/D | | intergenic |
| ENSRNA050023914 | snoZ105 | T_aestivum | Plants | intergenic_cluster | C/D | | intergenic |
| ENSRNA050023916 | snoZ118 | T_aestivum | Plants | mono_intergenic | C/D | | intergenic |
| ENSRNA050023917 | snoZ105 | T_aestivum | Plants | intergenic_cluster | C/D | | intergenic |
| ENSRNA050023919 | snoR77 | T_aestivum | Plants | intergenic_cluster | H/ACA | | intergenic |
| ENSRNA050023921 | snoR44_J54 | T_aestivum | Plants | intergenic_cluster | C/D | | intergenic |
| ENSRNA050023922 | snoU36a | T_aestivum | Plants | intergenic_cluster | C/D | | intergenic |
| ENSRNA050023923 | snoZ159 | T_aestivum | Plants | mono_intergenic | C/D | | intergenic |
| ENSRNA050023924 | snoZ223 | T_aestivum | Plants | intergenic_cluster | C/D | | intergenic |
| ENSRNA050023929 | SNORD24 | T_aestivum | Plants | mono_intronic | C/D | TraesCS5A02G392900 | protein_coding |
| ENSRNA050023933 | snoR103 | T_aestivum | Plants | intronic_cluster | H/ACA | TraesCS2A02G007200 | protein_coding |
| ENSRNA050023934 | snoR103 | T_aestivum | Plants | intronic_cluster | H/ACA | TraesCS2A02G007200 | protein_coding |
| ENSRNA050023935 | snoR103 | T_aestivum | Plants | intronic_cluster | H/ACA | TraesCS2A02G007200 | protein_coding |
| ENSRNA050023936 | snoR103 | T_aestivum | Plants | intronic_cluster | H/ACA | TraesCS2A02G007200 | protein_coding |
| ENSRNA050023937 | snoR103 | T_aestivum | Plants | intronic_cluster | H/ACA | TraesCS2A02G007200 | protein_coding |
| ENSRNA050023938 | snoR103 | T_aestivum | Plants | intronic_cluster | H/ACA | TraesCS2A02G007200 | protein_coding |
| ENSRNA050023939 | snoR103 | T_aestivum | Plants | intronic_cluster | H/ACA | TraesCS2A02G007200 | protein_coding |

| | | | | | | | |
|---|---|---|---|---|---|---|---|
| ENSRNA050023940 | snoR101 | T_aestivum | Plants | intronic_cluster | C/D | TraesCS3B02G289900 | protein_coding |
| ENSRNA050023941 | snoR101 | T_aestivum | Plants | intergenic_cluster | C/D | | intergenic |
| ENSRNA050023942 | snoR101 | T_aestivum | Plants | intergenic_cluster | C/D | | intergenic |
| ENSRNA050023943 | snoR101 | T_aestivum | Plants | intronic_cluster | C/D | TraesCS3B02G289900 | protein_coding |
| ENSRNA050023944 | snoR101 | T_aestivum | Plants | intergenic_cluster | C/D | | intergenic |
| ENSRNA050023945 | snoR101 | T_aestivum | Plants | intergenic_cluster | C/D | | intergenic |
| ENSRNA050023947 | snoU36a | T_aestivum | Plants | intergenic_cluster | C/D | | intergenic |
| ENSRNA050023948 | SNORD25 | T_aestivum | Plants | mono_intergenic | C/D | | intergenic |
| ENSRNA050023949 | snoZ223 | T_aestivum | Plants | intergenic_cluster | C/D | | intergenic |
| ENSRNA050023950 | snoZ159 | T_aestivum | Plants | mono_intergenic | C/D | | intergenic |
| ENSRNA050023951 | snoZ159 | T_aestivum | Plants | intergenic_cluster | C/D | | intergenic |
| ENSRNA050023952 | snoZ159 | T_aestivum | Plants | mono_intergenic | C/D | | intergenic |
| ENSRNA050023953 | snoZ159 | T_aestivum | Plants | mono_intergenic | C/D | | intergenic |
| ENSRNA050023954 | snoR80 | T_aestivum | Plants | mono_intergenic | H/ACA | | intergenic |
| ENSRNA050023955 | snoZ43 | T_aestivum | Plants | intergenic_cluster | C/D | | intergenic |
| ENSRNA050023956 | snoZ279_R105_R108 | T_aestivum | Plants | mono_intergenic | C/D | | intergenic |
| ENSRNA050023958 | snoU36a | T_aestivum | Plants | intergenic_cluster | C/D | | intergenic |
| ENSRNA050023959 | snoU36a | T_aestivum | Plants | intergenic_cluster | C/D | | intergenic |
| ENSRNA050023960 | snoZ101 | T_aestivum | Plants | intergenic_cluster | C/D | | intergenic |
| ENSRNA050023961 | snoZ101 | T_aestivum | Plants | intergenic_cluster | C/D | | intergenic |
| ENSRNA050023962 | snoZ101 | T_aestivum | Plants | intergenic_cluster | C/D | | intergenic |
| ENSRNA050023963 | snoU36a | T_aestivum | Plants | intergenic_cluster | C/D | | intergenic |
| ENSRNA050023964 | snoZ101 | T_aestivum | Plants | intergenic_cluster | C/D | | intergenic |
| ENSRNA050023965 | snoZ101 | T_aestivum | Plants | intergenic_cluster | C/D | | intergenic |
| ENSRNA050023966 | snoZ223 | T_aestivum | Plants | intergenic_cluster | C/D | | intergenic |
| ENSRNA050023967 | snoZ223 | T_aestivum | Plants | intergenic_cluster | C/D | | intergenic |
| ENSRNA050023968 | snoZ223 | T_aestivum | Plants | intergenic_cluster | C/D | | intergenic |
| ENSRNA050023969 | snoZ159 | T_aestivum | Plants | mono_intergenic | C/D | | intergenic |
| ENSRNA050023970 | snoZ159 | T_aestivum | Plants | intergenic_cluster | C/D | | intergenic |
| ENSRNA050023971 | snoZ159 | T_aestivum | Plants | intergenic_cluster | C/D | | intergenic |
| ENSRNA050023972 | snosnR60_Z15 | T_aestivum | Plants | intergenic_cluster | C/D | | intergenic |
| ENSRNA050023973 | snoU31b | T_aestivum | Plants | intergenic_cluster | C/D | | intergenic |
| ENSRNA050023974 | snoR77Y | T_aestivum | Plants | mono_intergenic | C/D | | intergenic |
| ENSRNA050023975 | snoZ102_R77 | T_aestivum | Plants | intergenic_cluster | C/D | | intergenic |
| ENSRNA050023976 | snoR8a | T_aestivum | Plants | intergenic_cluster | C/D | | intergenic |
| ENSRNA050023978 | snoZ102_R77 | T_aestivum | Plants | intergenic_cluster | C/D | | intergenic |
| ENSRNA050023979 | snoR77Y | T_aestivum | Plants | mono_intergenic | C/D | | intergenic |
| ENSRNA050023980 | snoZ159 | T_aestivum | Plants | intergenic_cluster | C/D | | intergenic |
| ENSRNA050023983 | snoU36a | T_aestivum | Plants | intergenic_cluster | C/D | | intergenic |
| ENSRNA050023984 | snoZ223 | T_aestivum | Plants | intergenic_cluster | C/D | | intergenic |
| ENSRNA050023988 | snoZ278 | T_aestivum | Plants | mono_intergenic | C/D | | intergenic |
| ENSRNA050023992 | snoR80 | T_aestivum | Plants | mono_intergenic | H/ACA | | intergenic |
| ENSRNA050023999 | snoZ43 | T_aestivum | Plants | intergenic_cluster | C/D | | intergenic |

| | | | | | | | |
|---|---|---|---|---|---|---|---|
| ENSRNA050024000 | snoZ43 | T_aestivum | Plants | intergenic_cluster | C/D | | intergenic |
| ENSRNA050024001 | snoZ101 | T_aestivum | Plants | intergenic_cluster | C/D | | intergenic |
| ENSRNA050024002 | snoZ101 | T_aestivum | Plants | intergenic_cluster | C/D | | intergenic |
| ENSRNA050024003 | snoZ101 | T_aestivum | Plants | intergenic_cluster | C/D | | intergenic |
| ENSRNA050024004 | snoZ101 | T_aestivum | Plants | intergenic_cluster | C/D | | intergenic |
| ENSRNA050024005 | snoZ101 | T_aestivum | Plants | intergenic_cluster | C/D | | intergenic |
| ENSRNA050024006 | snoZ101 | T_aestivum | Plants | intergenic_cluster | C/D | | intergenic |
| ENSRNA050024009 | snosnR60_Z15 | T_aestivum | Plants | intergenic_cluster | C/D | | intergenic |
| ENSRNA050024010 | snosnR60_Z15 | T_aestivum | Plants | intergenic_cluster | C/D | | intergenic |
| ENSRNA050024011 | snoU31b | T_aestivum | Plants | intergenic_cluster | C/D | | intergenic |
| ENSRNA050024012 | snoR8a | T_aestivum | Plants | intergenic_cluster | C/D | | intergenic |
| ENSRNA050024013 | snoR8a | T_aestivum | Plants | intergenic_cluster | C/D | | intergenic |
| ENSRNA050024014 | snoR8a | T_aestivum | Plants | intergenic_cluster | C/D | | intergenic |
| ENSRNA050024015 | snoZ102_R77 | T_aestivum | Plants | intergenic_cluster | C/D | | intergenic |
| ENSRNA050024016 | snoZ102_R77 | T_aestivum | Plants | intergenic_cluster | C/D | | intergenic |
| ENSRNA050024017 | snoR77Y | T_aestivum | Plants | mono_intergenic | C/D | | intergenic |
| ENSRNA050024018 | snoR8a | T_aestivum | Plants | intergenic_cluster | C/D | | intergenic |
| ENSRNA050024019 | snoZ101 | T_aestivum | Plants | intergenic_cluster | C/D | | intergenic |
| ENSRNA050024020 | snoZ102_R77 | T_aestivum | Plants | intergenic_cluster | C/D | | intergenic |
| ENSRNA050024025 | SNOR75 | T_aestivum | Plants | mono_intergenic | C/D | | intergenic |
| ENSRNA050024028 | snoR103 | T_aestivum | Plants | intronic_cluster | H/ACA | TraesCS2B02G013500 | protein_coding |
| ENSRNA050024029 | snoR103 | T_aestivum | Plants | intronic_cluster | H/ACA | TraesCS2B02G013500 | protein_coding |
| ENSRNA050024030 | snoR103 | T_aestivum | Plants | intronic_cluster | H/ACA | TraesCS2B02G013500 | protein_coding |
| ENSRNA050024031 | snoR103 | T_aestivum | Plants | intronic_cluster | H/ACA | TraesCS2B02G013500 | protein_coding |
| ENSRNA050024032 | snoR103 | T_aestivum | Plants | intronic_cluster | H/ACA | TraesCS2B02G013500 | protein_coding |
| ENSRNA050024033 | snoR103 | T_aestivum | Plants | intronic_cluster | H/ACA | TraesCS2B02G013500 | protein_coding |
| ENSRNA050024036 | snoR103 | T_aestivum | Plants | intronic_cluster | H/ACA | TraesCS2B02G013500 | protein_coding |
| ENSRNA050024040 | snoJ33 | T_aestivum | Plants | mono_exonic | C/D | TraesCS3A02G292700 | protein_coding |
| ENSRNA050024042 | SNOR75 | T_aestivum | Plants | intronic_cluster | C/D | TraesCS2B02G539700 | protein_coding |
| ENSRNA050024043 | SNOR75 | T_aestivum | Plants | intronic_cluster | C/D | TraesCS2B02G539700 | protein_coding |
| ENSRNA050024044 | U54 | T_aestivum | Plants | intronic_cluster | C/D | TraesCS2B02G539500 | protein_coding |
| ENSRNA050024045 | U54 | T_aestivum | Plants | intronic_cluster | C/D | TraesCS2B02G539500 | protein_coding |
| ENSRNA050024046 | U54 | T_aestivum | Plants | intronic_cluster | C/D | TraesCS2B02G539500 | protein_coding |
| ENSRNA050024047 | U54 | T_aestivum | Plants | intronic_cluster | C/D | TraesCS2B02G539500 | protein_coding |
| ENSRNA050024048 | U54 | T_aestivum | Plants | intronic_cluster | C/D | TraesCS2B02G539500 | protein_coding |
| ENSRNA050024049 | U54 | T_aestivum | Plants | intronic_cluster | C/D | TraesCS2B02G539500 | protein_coding |
| ENSRNA050024051 | U54 | T_aestivum | Plants | intronic_cluster | C/D | TraesCS2B02G539500 | protein_coding |
| ENSRNA050024053 | SNORD24 | T_aestivum | Plants | mono_intergenic | C/D | | intergenic |
| ENSRNA050024054 | snoR44_J54 | T_aestivum | Plants | mono_intergenic | C/D | | intergenic |
| ENSRNA050024061 | snoR77Y | T_aestivum | Plants | mono_intergenic | C/D | | intergenic |
| ENSRNA050024062 | snoR8a | T_aestivum | Plants | mono_intergenic | C/D | | intergenic |
| ENSRNA050024063 | snoU31b | T_aestivum | Plants | mono_intergenic | C/D | | intergenic |
| ENSRNA050024076 | snoZ199 | T_aestivum | Plants | intergenic_cluster | C/D | | intergenic |

| | | | | | | | |
|---|---|---|---|---|---|---|---|
| ENSRNA050024077 | SNORD24 | T_aestivum | Plants | mono_intergenic | C/D | | intergenic |
| ENSRNA050024078 | snoZ199 | T_aestivum | Plants | intergenic_cluster | C/D | | intergenic |
| ENSRNA050024084 | SNOR75 | T_aestivum | Plants | intergenic_cluster | C/D | | intergenic |
| ENSRNA050024085 | SNOR75 | T_aestivum | Plants | intergenic_cluster | C/D | | intergenic |
| ENSRNA050024086 | snoR77Y | T_aestivum | Plants | mono_intergenic | C/D | | intergenic |
| ENSRNA050024091 | snoZ199 | T_aestivum | Plants | intergenic_cluster | C/D | | intergenic |
| ENSRNA050024092 | snoR64 | T_aestivum | Plants | intergenic_cluster | C/D | | intergenic |
| ENSRNA050024093 | snoZ199 | T_aestivum | Plants | intergenic_cluster | C/D | | intergenic |
| ENSRNA050024101 | SNORD24 | T_aestivum | Plants | mono_intergenic | C/D | | intergenic |
| ENSRNA050024102 | snoR103 | T_aestivum | Plants | mono_intergenic | H/ACA | | intergenic |
| ENSRNA050024103 | snoR103 | T_aestivum | Plants | intergenic_cluster | H/ACA | | intergenic |
| ENSRNA050024104 | snoR103 | T_aestivum | Plants | intergenic_cluster | H/ACA | | intergenic |
| ENSRNA050024106 | SNORD24 | T_aestivum | Plants | mono_intergenic | C/D | | intergenic |
| ENSRNA050024108 | snoZ221_snoR21b | T_aestivum | Plants | intronic_cluster | C/D | TraesCS2B02G559300 | protein_coding |
| ENSRNA050024109 | snoZ221_snoR21b | T_aestivum | Plants | intronic_cluster | C/D | TraesCS2B02G559300 | protein_coding |
| ENSRNA050024110 | snoZ221_snoR21b | T_aestivum | Plants | intronic_cluster | C/D | TraesCS2B02G559300 | protein_coding |
| ENSRNA050024119 | snoZ279_R105_R108 | T_aestivum | Plants | mono_intergenic | C/D | | intergenic |
| ENSRNA050024124 | snoZ43 | T_aestivum | Plants | intergenic_cluster | C/D | | intergenic |
| ENSRNA050024125 | snoZ101 | T_aestivum | Plants | intergenic_cluster | C/D | | intergenic |
| ENSRNA050024126 | snoZ101 | T_aestivum | Plants | intergenic_cluster | C/D | | intergenic |
| ENSRNA050024127 | snoR64 | T_aestivum | Plants | intergenic_cluster | C/D | | intergenic |
| ENSRNA050024128 | snoZ101 | T_aestivum | Plants | intergenic_cluster | C/D | | intergenic |
| ENSRNA050024129 | snoR64 | T_aestivum | Plants | intergenic_cluster | C/D | | intergenic |
| ENSRNA050024130 | snoZ223 | T_aestivum | Plants | intergenic_cluster | C/D | | intergenic |
| ENSRNA050024131 | snoZ223 | T_aestivum | Plants | intergenic_cluster | C/D | | intergenic |
| ENSRNA050024132 | SNORD25 | T_aestivum | Plants | intergenic_cluster | C/D | | intergenic |
| ENSRNA050024133 | SNORD25 | T_aestivum | Plants | mono_intergenic | C/D | | intergenic |
| ENSRNA050024134 | SNORD25 | T_aestivum | Plants | mono_intergenic | C/D | | intergenic |
| ENSRNA050024135 | snoZ278 | T_aestivum | Plants | intergenic_cluster | C/D | | intergenic |
| ENSRNA050024136 | snoZ199 | T_aestivum | Plants | intergenic_cluster | C/D | | intergenic |
| ENSRNA050024137 | snoZ199 | T_aestivum | Plants | intergenic_cluster | C/D | | intergenic |
| ENSRNA050024138 | snoZ278 | T_aestivum | Plants | intergenic_cluster | C/D | | intergenic |
| ENSRNA050024139 | snoZ278 | T_aestivum | Plants | intergenic_cluster | C/D | | intergenic |
| ENSRNA050024140 | snoZ199 | T_aestivum | Plants | intergenic_cluster | C/D | | intergenic |
| ENSRNA050024141 | snoU31b | T_aestivum | Plants | intergenic_cluster | C/D | | intergenic |
| ENSRNA050024142 | snoZ102_R77 | T_aestivum | Plants | intergenic_cluster | C/D | | intergenic |
| ENSRNA050024143 | snosnR60_Z15 | T_aestivum | Plants | intergenic_cluster | C/D | | intergenic |
| ENSRNA050024160 | snoZ223 | T_aestivum | Plants | intergenic_cluster | C/D | | intergenic |
| ENSRNA050024161 | snoU36a | T_aestivum | Plants | intergenic_cluster | C/D | | intergenic |
| ENSRNA050024164 | snoZ119 | T_aestivum | Plants | intronic_cluster | C/D | TraesCS3A02G343300 | protein_coding |
| ENSRNA050024165 | snoZ119 | T_aestivum | Plants | intronic_cluster | C/D | TraesCS3A02G343300 | protein_coding |
| ENSRNA050024166 | snoZ119 | T_aestivum | Plants | intronic_cluster | C/D | TraesCS3A02G343300 | protein_coding |
| ENSRNA050024167 | snoZ119 | T_aestivum | Plants | intronic_cluster | C/D | TraesCS3A02G343300 | protein_coding |

| | | | | | | | |
|---|---|---|---|---|---|---|---|
| ENSRNA050024168 | snoZ119 | T_aestivum | Plants | intronic_cluster | C/D | TraesCS3A02G343300 | protein_coding |
| ENSRNA050024169 | snoZ119 | T_aestivum | Plants | intronic_cluster | Unknown | TraesCS3A02G343300 | protein_coding |
| ENSRNA050024171 | snoZ122 | T_aestivum | Plants | intronic_cluster | C/D | TraesCS3A02G343300 | protein_coding |
| ENSRNA050024179 | snoZ101 | T_aestivum | Plants | mono_intergenic | C/D | | intergenic |
| ENSRNA050024181 | snoZ101 | T_aestivum | Plants | mono_intergenic | C/D | | intergenic |
| ENSRNA050024183 | SNOR75 | T_aestivum | Plants | intergenic_cluster | C/D | | intergenic |
| ENSRNA050024184 | SNOR75 | T_aestivum | Plants | intergenic_cluster | C/D | | intergenic |
| ENSRNA050024186 | snoZ105 | T_aestivum | Plants | intergenic_cluster | C/D | | intergenic |
| ENSRNA050024187 | SNORD25 | T_aestivum | Plants | intronic_cluster | C/D | TraesCS2A02G363200 | protein_coding |
| ENSRNA050024188 | SNORD25 | T_aestivum | Plants | intronic_cluster | C/D | TraesCS2A02G363200 | protein_coding |
| ENSRNA050024189 | SNORD25 | T_aestivum | Plants | intronic_cluster | C/D | TraesCS2A02G363300 | protein_coding |
| ENSRNA050024190 | SNORD25 | T_aestivum | Plants | intronic_cluster | C/D | TraesCS2A02G363300 | protein_coding |
| ENSRNA050024191 | snoU31b | T_aestivum | Plants | intronic_cluster | C/D | TraesCS2A02G363200 | protein_coding |
| ENSRNA050024192 | snoU31b | T_aestivum | Plants | intronic_cluster | C/D | TraesCS2A02G363200 | protein_coding |
| ENSRNA050024193 | SNORD25 | T_aestivum | Plants | intronic_cluster | C/D | TraesCS2A02G363300 | protein_coding |
| ENSRNA050024194 | snoU31b | T_aestivum | Plants | intronic_cluster | C/D | TraesCS2A02G363300 | protein_coding |
| ENSRNA050024196 | snosnR60_Z15 | T_aestivum | Plants | intronic_cluster | C/D | TraesCS2A02G363200 | protein_coding |
| ENSRNA050024200 | snoZ43 | T_aestivum | Plants | intronic_cluster | C/D | TraesCS2A02G363300 | protein_coding |
| ENSRNA050024204 | SNORD24 | T_aestivum | Plants | mono_intergenic | C/D | | intergenic |
| ENSRNA050024205 | SNORD24 | T_aestivum | Plants | mono_intergenic | C/D | | intergenic |
| ENSRNA050024209 | snoJ33 | T_aestivum | Plants | mono_exonic | C/D | TraesCS2B02G521100 | protein_coding |
| ENSRNA050024223 | SNORD25 | T_aestivum | Plants | intergenic_cluster | C/D | | intergenic |
| ENSRNA050024224 | SNORD25 | T_aestivum | Plants | intergenic_cluster | C/D | | intergenic |
| ENSRNA050024225 | snoU31b | T_aestivum | Plants | mono_intergenic | C/D | | intergenic |
| ENSRNA050024227 | snoU31b | T_aestivum | Plants | mono_intergenic | C/D | | intergenic |
| ENSRNA050024229 | snoR41 | T_aestivum | Plants | intergenic_cluster | C/D | | intergenic |
| ENSRNA050024230 | snoZ155 | T_aestivum | Plants | intergenic_cluster | C/D | | intergenic |
| ENSRNA050024237 | SNOR75 | T_aestivum | Plants | mono_intergenic | C/D | | intergenic |
| ENSRNA050024239 | SNOR75 | T_aestivum | Plants | intergenic_cluster | C/D | | intergenic |
| ENSRNA050024240 | SNOR75 | T_aestivum | Plants | intergenic_cluster | C/D | | intergenic |
| ENSRNA050024241 | snoZ155 | T_aestivum | Plants | intergenic_cluster | C/D | | intergenic |
| ENSRNA050024242 | snoZ155 | T_aestivum | Plants | intergenic_cluster | C/D | | intergenic |
| ENSRNA050024243 | snoR41 | T_aestivum | Plants | intergenic_cluster | C/D | | intergenic |
| ENSRNA050024254 | snoZ221_snoR21b | T_aestivum | Plants | intronic_cluster | C/D | TraesCS2A02G528900 | protein_coding |
| ENSRNA050024255 | snoZ221_snoR21b | T_aestivum | Plants | intronic_cluster | C/D | TraesCS2A02G528900 | protein_coding |
| ENSRNA050024256 | snoZ221_snoR21b | T_aestivum | Plants | intronic_cluster | C/D | TraesCS2A02G528900 | protein_coding |
| ENSRNA050024259 | snoZ43 | T_aestivum | Plants | intergenic_cluster | C/D | | intergenic |
| ENSRNA050024260 | snoZ43 | T_aestivum | Plants | intergenic_cluster | C/D | | intergenic |
| ENSRNA050024261 | snoZ101 | T_aestivum | Plants | intergenic_cluster | C/D | | intergenic |
| ENSRNA050024262 | snoZ101 | T_aestivum | Plants | intergenic_cluster | C/D | | intergenic |
| ENSRNA050024263 | snoZ101 | T_aestivum | Plants | intergenic_cluster | C/D | | intergenic |
| ENSRNA050024264 | snoZ101 | T_aestivum | Plants | intergenic_cluster | C/D | | intergenic |
| ENSRNA050024265 | snoZ101 | T_aestivum | Plants | intergenic_cluster | C/D | | intergenic |

| | | | | | | | |
|---|---|---|---|---|---|---|---|
| ENSRNA050024266 | snoZ101 | T_aestivum | Plants | intergenic_cluster | C/D | | intergenic |
| ENSRNA050024267 | snoZ101 | T_aestivum | Plants | intergenic_cluster | C/D | | intergenic |
| ENSRNA050024269 | snosnR60_Z15 | T_aestivum | Plants | intergenic_cluster | C/D | | intergenic |
| ENSRNA050024270 | snosnR60_Z15 | T_aestivum | Plants | intergenic_cluster | C/D | | intergenic |
| ENSRNA050024271 | snoR8a | T_aestivum | Plants | intergenic_cluster | C/D | | intergenic |
| ENSRNA050024272 | snoR8a | T_aestivum | Plants | intergenic_cluster | C/D | | intergenic |
| ENSRNA050024273 | snoU31b | T_aestivum | Plants | intergenic_cluster | C/D | | intergenic |
| ENSRNA050024274 | snoR77Y | T_aestivum | Plants | mono_intergenic | C/D | | intergenic |
| ENSRNA050024275 | snoR8a | T_aestivum | Plants | intergenic_cluster | C/D | | intergenic |
| ENSRNA050024276 | snoZ102_R77 | T_aestivum | Plants | intergenic_cluster | C/D | | intergenic |
| ENSRNA050024277 | snoR8a | T_aestivum | Plants | intergenic_cluster | C/D | | intergenic |
| ENSRNA050024278 | snoZ102_R77 | T_aestivum | Plants | intergenic_cluster | C/D | | intergenic |
| ENSRNA050024279 | snoZ102_R77 | T_aestivum | Plants | intergenic_cluster | C/D | | intergenic |
| ENSRNA050024280 | snoZ102_R77 | T_aestivum | Plants | intergenic_cluster | C/D | | intergenic |
| ENSRNA050024281 | snoR77Y | T_aestivum | Plants | mono_intergenic | C/D | | intergenic |
| ENSRNA050024282 | snoU31b | T_aestivum | Plants | intergenic_cluster | C/D | | intergenic |
| ENSRNA050024288 | snoR77Y | T_aestivum | Plants | intergenic_cluster | C/D | | intergenic |
| ENSRNA050024290 | snoR31_Z110_Z27 | T_aestivum | Plants | intergenic_cluster | C/D | | intergenic |
| ENSRNA050024294 | snoR80 | T_aestivum | Plants | mono_intergenic | H/ACA | | intergenic |
| ENSRNA050024339 | snoZ101 | T_aestivum | Plants | intergenic_cluster | C/D | | intergenic |
| ENSRNA050024340 | snoZ101 | T_aestivum | Plants | intergenic_cluster | C/D | | intergenic |
| ENSRNA050024341 | snoZ101 | T_aestivum | Plants | intergenic_cluster | C/D | | intergenic |
| ENSRNA050024342 | snoZ102_R77 | T_aestivum | Plants | intergenic_cluster | C/D | | intergenic |
| ENSRNA050024343 | snoZ101 | T_aestivum | Plants | intergenic_cluster | C/D | | intergenic |
| ENSRNA050024344 | snoR8a | T_aestivum | Plants | mono_intergenic | C/D | | intergenic |
| ENSRNA050024345 | snoZ102_R77 | T_aestivum | Plants | intergenic_cluster | C/D | | intergenic |
| ENSRNA050024351 | U54 | T_aestivum | Plants | intronic_cluster | C/D | TraesCS2A02G511400 | protein_coding |
| ENSRNA050024352 | U54 | T_aestivum | Plants | intronic_cluster | C/D | TraesCS2A02G511400 | protein_coding |
| ENSRNA050024353 | U54 | T_aestivum | Plants | intronic_cluster | C/D | TraesCS2A02G511400 | protein_coding |
| ENSRNA050024354 | U54 | T_aestivum | Plants | intronic_cluster | C/D | TraesCS2A02G511400 | protein_coding |
| ENSRNA050024355 | U54 | T_aestivum | Plants | intronic_cluster | C/D | TraesCS2A02G511400 | protein_coding |
| ENSRNA050024511 | snoZ223 | T_aestivum | Plants | mono_intergenic | C/D | | intergenic |
| ENSRNA050024554 | snoR80 | T_aestivum | Plants | mono_intergenic | H/ACA | | intergenic |
| ENSRNA050024555 | snoR80 | T_aestivum | Plants | intergenic_cluster | H/ACA | | intergenic |
| ENSRNA050024556 | snoR80 | T_aestivum | Plants | mono_intergenic | H/ACA | | intergenic |
| ENSRNA050024557 | SNORD24 | T_aestivum | Plants | intergenic_cluster | C/D | | intergenic |
| ENSRNA050024558 | snoR8a | T_aestivum | Plants | mono_intergenic | C/D | | intergenic |
| ENSRNA050024559 | snoR77Y | T_aestivum | Plants | intergenic_cluster | C/D | | intergenic |
| ENSRNA050024560 | SNORD24 | T_aestivum | Plants | mono_intergenic | C/D | | intergenic |
| ENSRNA050024561 | snoR117 | T_aestivum | Plants | mono_intergenic | C/D | | intergenic |
| ENSRNA050024562 | snoR117 | T_aestivum | Plants | mono_intergenic | C/D | | intergenic |
| ENSRNA050024563 | snoR117 | T_aestivum | Plants | mono_intergenic | C/D | | intergenic |
| ENSRNA050024564 | snoU31b | T_aestivum | Plants | intergenic_cluster | C/D | | intergenic |

| | | | | | | |
|---|---|---|---|---|---|---|
| ENSRNA050024742 | snoJ33 | T_aestivum | Plants | mono_intergenic | C/D | intergenic |
| ENSRNA050024788 | snoR64 | T_aestivum | Plants | intergenic_cluster | C/D | intergenic |
| ENSRNA050024789 | snoZ199 | T_aestivum | Plants | intergenic_cluster | C/D | intergenic |
| ENSRNA050024790 | SNORD24 | T_aestivum | Plants | mono_intergenic | C/D | intergenic |
| DDB_G0294409 | sno1 | D_discoideum | Protists | intergenic_cluster | C/D | intergenic |
| DDB_G0295499 | sno3 | D_discoideum | Protists | mono_intergenic | C/D | intergenic |
| DDB_G0295511 | sno10 | D_discoideum | Protists | mono_intergenic | C/D | intergenic |
| DDB_G0295517 | sno17 | D_discoideum | Protists | mono_intergenic | C/D | intergenic |
| DDB_G0295525 | sno11b | D_discoideum | Protists | intergenic_cluster | C/D | intergenic |
| DDB_G0295527 | sno11a | D_discoideum | Protists | intergenic_cluster | C/D | intergenic |
| DDB_G0295531 | sno6 | D_discoideum | Protists | mono_intergenic | C/D | intergenic |
| DDB_G0295543 | sno18 | D_discoideum | Protists | intergenic_cluster | H/ACA | intergenic |
| DDB_G0295545 | sno4 | D_discoideum | Protists | intergenic_cluster | C/D | intergenic |
| DDB_G0295551 | sno14 | D_discoideum | Protists | mono_intergenic | C/D | intergenic |
| DDB_G0295555 | sno9 | D_discoideum | Protists | intergenic_cluster | C/D | intergenic |
| DDB_G0295561 | sno15 | D_discoideum | Protists | mono_intergenic | C/D | intergenic |
| DDB_G0295603 | sno8 | D_discoideum | Protists | mono_intergenic | C/D | intergenic |
| DDB_G0295605 | sno12 | D_discoideum | Protists | mono_intergenic | C/D | intergenic |
| DDB_G0295615 | sno7 | D_discoideum | Protists | mono_intergenic | C/D | intergenic |
| DDB_G0295617 | sno5 | D_discoideum | Protists | mono_intergenic | C/D | intergenic |
| DDB_G0295619 | sno2 | D_discoideum | Protists | intergenic_cluster | C/D | intergenic |
| DDB_G0295621 | sno13 | D_discoideum | Protists | intergenic_cluster | C/D | intergenic |
| DDB_G0295631 | sno16 | D_discoideum | Protists | mono_intergenic | C/D | intergenic |
| EPrTTHG00000000101 | EPrTTHG00000000101 | T_thermophila | Protists | mono_intergenic | C/D | intergenic |
| EPrTTHG00000000153 | EPrTTHG00000000153 | T_thermophila | Protists | mono_intergenic | C/D | intergenic |
| EPrTTHG00000000320 | EPrTTHG00000000320 | T_thermophila | Protists | mono_intergenic | C/D | intergenic |
| EPrTTHG00000000675 | EPrTTHG00000000675 | T_thermophila | Protists | mono_intergenic | C/D | intergenic |
| EPrTTHG00000000679 | EPrTTHG00000000679 | T_thermophila | Protists | mono_intergenic | C/D | intergenic |

**Supplementary Table S2**: The origin, localization, mode of action and fate of the snoRNA hybrids and their host gene transcript

| SnoRNA hybrid | Origin | Localization | Mode of action | SnoRNA hybrid fate | Host gene transcript fate |
|---|---|---|---|---|---|
| cSPA | Alternative splice site usage which retains *SNORD86* in the host gene transcript | Cytoplasm | Sequesters C/D box snoRNP core proteins in the cytoplasm to regulate NOP56 production | Stabilized in the cytoplasm when there is an excess of NOP56 protein | When NOP56 levels are in excess, cSPA is the major host transcript and is stabilized in the cytoplasm; when NOP56 levels are low, cSPA is not produced and the host gene transcript coding for NOP56 is produced and translated into NOP56 protein |
| snoRT | Retention of a snoRNA-containing intron in a host gene transcript | Cytoplasm | Sequesters H/ACA box snoRNP core proteins in the cytoplasm and potentially guides the pseudouridylation of some mRNAs | Stabilized in the cytoplasm | Both mature host transcript and snoRT are produced from the same host genes, with the mature host transcripts being translated into proteins |
| slb-snoRNA | Spliced lariat of a host gene that is not linearized due to an atypical branch point in vertebrate species | Nucleus and cytoplasm | Hinders the processing of snoRNAs into their mature form by being exported as slb-snoRNAs to the cytoplasm and potentially sequesters snoRNP core proteins to the cytoplasm | Stabilized in the nucleus and sometimes exported to the cytoplasm | The host gene transcript is presumably processed as other non-host genes (spliced, capped and polyadenylated) |

| | | | | | |
|---|---|---|---|---|---|
| hmsnoRNA | Depletion of splicing factors which causes the retention of a snoRNA in its host gene transcript followed by exonucleolytic trimming up to the snoRNA end | Cytoplasm | By-product of host genes due to splicing defects | Degraded or sometimes stabilized in the cytoplasm | The hmsnoRNA usually represents the majority of host gene transcripts (when splicing factors are depleted) and are mostly degraded in the cytoplasm |
| sno-lncRNA | Splicing, debranching and exonucleolytic trimming of an intron containing two snoRNAs | Nucleus | Binds to splicing factors to control their availability and thereby regulates splicing events in neuronal-specific genes | Stabilized in the nucleus | The host gene transcript is processed as other non-host genes (spliced, capped and polyadenylated) |
| *SLERT* (sno-lncRNA) | Skipping of two exons in a host transcript, one of which is flanked by two introns containing each a H/ACA box snoRNA, resulting in a lariat that is debranched and trimmed up to the snoRNA ends | Nucleus | Interacts with the RNA helicase DDX21 to promote the transcription of rDNA genes and cell proliferation | Stabilized in the nucleus | When *SLERT* is produced, a host gene transcript with skipped exons is also produced, but displays rapid degradation rate |

| | | | | | |
|---|---|---|---|---|---|
| *LNC-SNO49AB* | Retention of an intron containing *SNORD49B* and *SNORD49A* in a host gene transcript followed by exonucleolytic trimming up to *SNORD49A* 's 3' end | Nucleolus | Acts as a scaffold to promote the dimerization and thus the activity of the A-to-I editing enzyme ADAR1 | Stabilized in the nucleolus | Both mature host transcript and *LNC-SNO49AB* are produced from the same host gene (*SNHG29* ), with the host transcript, a lncRNA, being capped and polyadenylated |
| SPA | Transcript read-through of a gene that is flanked at its 3' end by snoRNAs, resulting in a 5' snoRNA-capped and 3' poyladenylated transcript | Nucleus | Binds to splicing factors to control their availability and thereby regulates splicing events in neuronal-specific genes | Stabilized in the nucleus | The *SNURF-SNRPN* gene is transcribed and processed as other protein-coding genes (spliced, capped and polyadenylated), while its following read-through produces the SPAs |
